# Investigating the association between gut microbiome and aortic aneurysm diseases: a bidirectional two-sample Mendelian randomization analysis

**DOI:** 10.3389/fcimb.2024.1406845

**Published:** 2024-07-30

**Authors:** Yaodong Sun, Haoju Dong, Chao Sun, Dongdong Du, Ruirong Gao, Mikhail Voevoda, Roman Knyazev, Naishi Wu

**Affiliations:** ^1^ Department of Cardiovascular Surgery, Tianjin Medical University General Hospital, Tianjin, China; ^2^ Pediatric Cardiac Surgery, Fuwai Central China Cardiovascular Hospital, Zhengzhou, China; ^3^ Department of Cardiovascular Surgery, Henan Provincial People’s Hospital, Zhengzhou, China; ^4^ Department of Orthopedic Surgery, Tianjin Medical University General Hospital, Tianjin, China; ^5^ Department of Cardiovascular Surgery, Shandong Provincial Hospital Affiliated to Shandong First Medical University, Jinan, Shandong, China; ^6^ Federal Research Center of Fundamental and Translational Medicine (FRC FTM), Novosibirsk, Russia

**Keywords:** abdominal aortic aneurysm, thoracic aortic aneurysm, aortic dissection, gut microbiome, Mendelian randomization

## Abstract

**Objective:**

This study aims to investigate the associations between specific bacterial taxa of the gut microbiome and the development of aortic aneurysm diseases, utilizing Mendelian Randomization (MR) to explore these associations and overcome the confounding factors commonly present in observational studies.

**Methods:**

Employing the largest available gut microbiome and aortic aneurysm Genome-Wide Association Study databases, including MiBioGen, Dutch Microbiome Project, FinnGen, UK Biobank, and Michigan Genomics Initiative, this study performs two-sample bidirectional MR analyses. Instrumental variables, linked to microbiome taxa at significant levels, were selected for identifying relationships with abdominal aortic aneurysms (AAA), thoracic aortic aneurysms (TAA), and aortic dissection (AD). Methods like inverse variance weighted, MR-PRESSO, MR-Egger, weighted median, simple mode, and mode-based estimate were used for MR analysis. Heterogeneity was assessed with the Cochran Q test. MR-Egger regression and MR-PRESSO addressed potential unbalanced horizontal pleiotropy.

**Results:**

The analysis did not find any evidence of statistically significant associations between the gut microbiome and aortic aneurysm diseases after adjusting for the false discovery rate (FDR). Specifically, while initial results suggested correlations between 19 taxa and AAA, 25 taxa and TAA, and 13 taxa with AD, these suggested associations did not hold statistical significance post-FDR correction. Therefore, the role of individual gut microbial taxa as independent factors in the development and progression of aortic aneurysm diseases remains inconclusive. This finding underscores the necessity for larger sample sizes and more comprehensive studies to further investigate these potential links.

**Conclusion:**

The study emphasizes the complex relationship between the gut microbiome and aortic aneurysm diseases. Although no statistically significant associations were found after FDR correction, the findings provide valuable insights and highlight the importance of considering gut microbiota in aortic aneurysm diseases research. Understanding these interactions may eventually contribute to identifying new therapeutic and preventive strategies for aortic aneurysm diseases.

## 1 Introduction

Aortic aneurysms and dissections are life-threatening conditions representing serious diseases of the aorta. Aortic aneurysms, including thoracic and abdominal variants, mainly occur due to chronic dilative changes caused by weakening of the aortic wall, with rupture of the aneurysms being a leading cause of death (Johnston et al., 1991). Aortic dissection (AD) occurs when there is an intimal tear that allows blood to flow between the layers of the aortic wall, splitting the intima longitudinally and creating a dissection flap that divides the true lumen from a newly formed false lumen (Isselbacher et al., 2022). Aortic aneurysm diseases are the 15th most common cause of death in individuals over 55 years of age and also impose significant economic burdens on patients’ families and society (Mcclure et al., 2020; Bossone and Eagle, 2021). Currently, the etiology of aortic aneurysm diseases is not fully understood, making it essential to find effective ways to prevent and reduce the incidence of these conditions.

Historically, it was presumed that dietary components were solely absorbed and metabolized by human cells within the intestine. However, recent insights reveal that the gut microbiome (GM), comprising trillions of commensal organisms, acts as a metabolically active organ akin to an endocrine system. This complex microbial community significantly impacts host physiological functions, predominantly through the production of biologically active metabolites. It plays a crucial role in vitamin synthesis and the modulation of key mechanisms such as inflammation, immune response, and oxidative stress mitigation (Tang et al., 2019; Witkowski et al., 2020). Imbalances in the GM can influence the occurrence and development of various diseases, including aortic aneurysms and dissections.

Multicentric clinical studies have identified an association between imbalances in the GM and the development of aortic aneurysms and dissections (Nakayama et al., 2022; Ito et al., 2023; Jiang et al., 2023; Manabe et al., 2023). Experimental studies have shown that imbalances in the GM can affect the occurrence of aneurysms (Shinohara et al., 2022), and gut microbial metabolites such as Trimethylamine N-oxide (TMAO) can promote the occurrence of aneurysms (Benson et al., 2023), while metabolites like butyrate and Indole-3-aldehyde can inhibit the development of aortic aneurysms and dissections (Tian et al., 2022; Huang et al., 2023). These studies objectively demonstrate the significant role of the GM and its metabolic products in the disease progression of aortic aneurysms and dissections, with the pathological mechanisms primarily based on the direct damage to the aortic wall by gut microbes and the indirect effects of inflammation mediated by microbial imbalances. However, observational studies struggle to establish clear causal and reverse causal relationships, and although randomized controlled trials (RCTs) are considered the gold standard for establishing causality, their implementation can sometimes be hindered by ethical, practical, or financial obstacles.

Mendelian randomization (MR) analysis is an epidemiological design that can strengthen causal inference by using genetic variants as instrumental variables (IVs) for an exposure (Davies et al., 2018). The MR design, characterized by its immunity to confounding biases and reverse causality, owes its robustness to the random assortment of genetic alleles at conception and their insusceptibility to modification by diseases (Pingault et al., 2018). Consequently, this study seeks to elucidate the potential causal associations between individual bacterial taxa and aortic aneurysm diseases through bidirectional MR analyses. This methodology has the potential to lay down a theoretical foundation and open up new avenues for the prevention and treatment of aortic aneurysm diseases.

## 2 Methods

### 2.1 Study design

**Figure 1:**
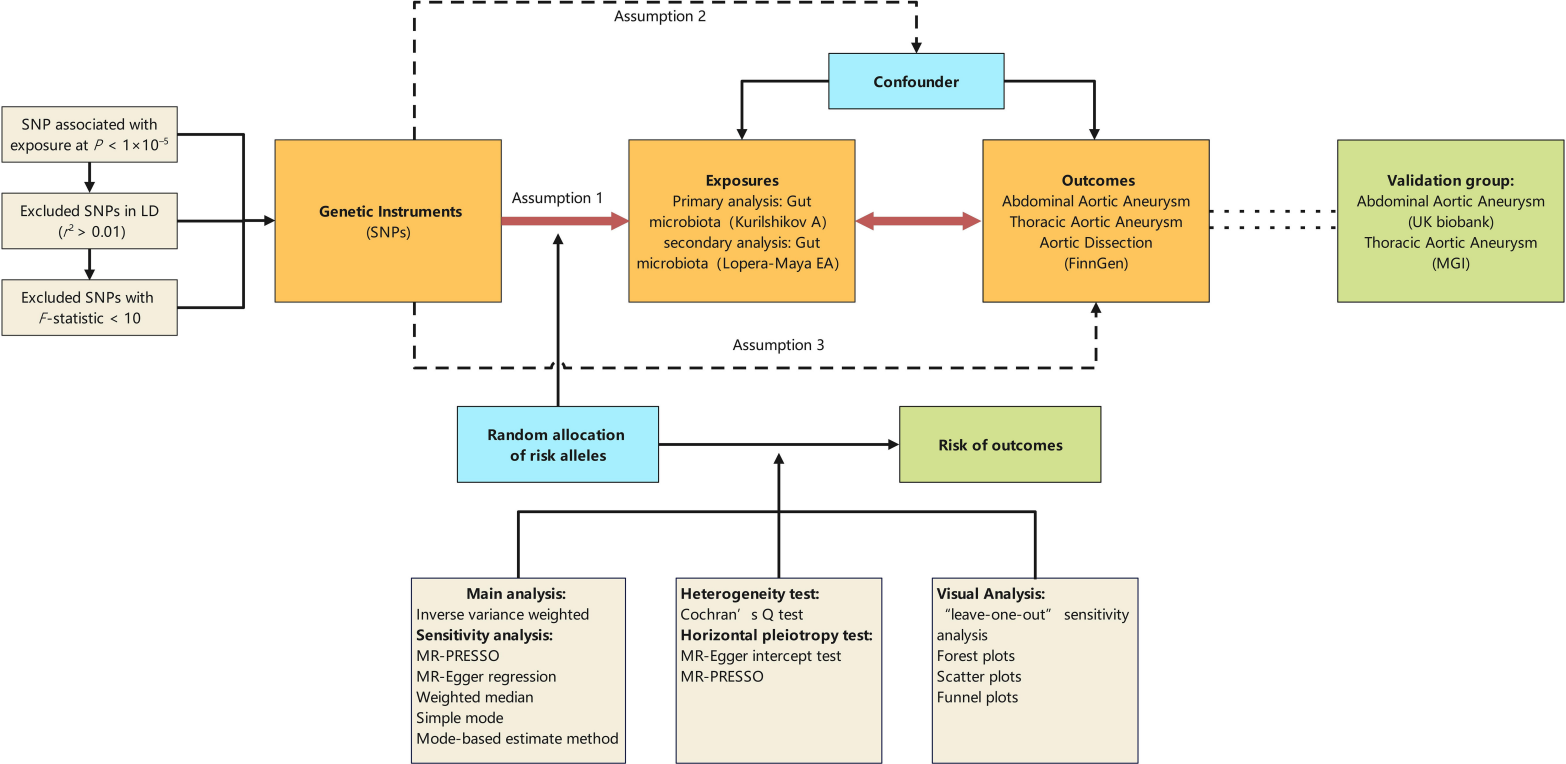
Schematic representation of the Mendelian randomization design. The traditional assumptions of Mendelian randomization are that the genetic instrumental variable is associated with the risk factor (assumption 1); the variants should not be associated with confounders (assumption 2); and that the variants should influence the outcome only through effects on the risk factor under investigation (assumption 3). SNP, single nucleotide polymorphism; LD, linkage disequilibrium; *P*, probability value; r^2^, coefficient of determination; MGI, the Michigan genomics initiative.

**Figure 1** illustrates the diagram of our study design, highlighting that the causal interpretation of MR estimates relies on three critical assumptions: (1) the IVs, which are single nucleotide polymorphisms (SNPs), must be associated with the exposures of interest; (2) the IVs must not be related to any confounders of the exposure-outcome relationship; (3) the association between the IVs and the outcome must be exclusively through the exposure of interest (Lawlor, 2016). This study conducts bidirectional Mendelian randomization analyses between the GM and aortic aneurysm diseases, using GM Genome-Wide Association Study (GWAS) data based on 16S rRNA sequencing methods for primary analysis and GM GWAS data based on Metagenomic Sequencing (MGS) methods for secondary analysis. Additionally, other GWAS datasets for abdominal aortic aneurysms (AAA) and thoracic aortic aneurysms (TAA) serve as validation sets for the positive findings identified.

### 2.2 Data sources

The characteristics of corresponding GWAS data sources are detailed in **Table 1**. Summary data on genes from the MiBioGen consortium, comprising participants of diverse ancestries from 24 cohorts totaling 18,340 individuals, represent the largest whole-genome meta-analysis to date. The genetic sequencing method was 16S rRNA, with 78% of participants being of European descent, comprising 211 taxonomic units across various classifications (Kurilshikov et al., 2021) (**Supplementary Additional File 1**; Order 1). We selected summary data from 7,738 individuals of European ancestry from the Dutch Microbiome Project (DMP) for the largest species-level GM dataset, sequenced using MGS, featuring 207 taxonomic units across different classifications (Lopera-Maya et al., 2022) (**Supplementary Additional File 1**; Order 2).

**Table 1:** Characteristics of summary genome-wide association studies.

	Trait	Sample size	Ancestry	Dataset source	Year	Web source/PMID
Exposure	Gut microbiota	18 340 individuals	Mixed (78% European)	MiBioGen	2021	PMID: 33462485
	Gut microbiota	7 738 individuals	European	Dutch Microbiome Project	2022	PMID: 35115690
Outcome	Abdominal Aortic Aneurysm	3 869 cases and 381 977 controls	European	FinnGen	2023	https://www.finngen.fi/fi
	Thoracic Aortic Aneurysm	3 880 cases and 381 977 controls	European	FinnGen	2023	https://www.finngen.fi/fi
	Aortic Dissection	967 cases and 381 977 controls	European	FinnGen	2023	https://www.finngen.fi/fi
	Abdominal Aortic Aneurysm	1 306 cases and 408 565 controls	European	UK biobank	2023	https://pan.ukbb.broadinstitute.org/
	Thoracic Aortic Aneurysm	1 351 cases and 18 295 controls	European	CHIP,MGI	2021	PMID: 34265237

CHIP, The Cardiovascular Health Improvement Project.

MGI, The Michigan Genomics Initiative.

GWAS data for AAA, TAA, and AD were obtained from FinnGen Release 10, which maps genotype-phenotype correlations using Finnish biobank data. This includes genome and health data from 3,869 AAA, 3,880 TAA, 967 AD individuals and 381,977 controls. Diagnoses for the following conditions were based on hospital records and classified according to the respective International Classification of Diseases (ICD) codes: AAA: ICD-10: I71.3, I71.4, ICD-9: 4413A, 4414A, ICD-8: 44120; TAA: ICD-10: I71.01, I71.1, I71.2, Q25.43; AD: ICD-10: I71.00, I71.01, I71.09, ICD-9: 4410. Endpoint and control definitions are detailed on the FinnGen website (https://www.finngen.fi/en/researchers/clinical-endpoints). Validation datasets for AAA were sourced from the UK Biobank database, including 1,306 cases and 408,565 controls, and TAA datasets were obtained from The Cardiovascular Health Improvement Project (CHIP) and The Michigan Genomics Initiative (MGI), including 1,351 cases and 18,295 controls (Roychowdhury et al., 2021).

### 2.3 Instrumental variable

For MR analysis, it’s crucial that the genetic variants used are representative of microbiome features. The selection criteria for IVs included: (1) SNPs associated with each genus at the locus-wide significance threshold (*P* < 1.0×10^-5^) were chosen as potential IVs (Sanna et al., 2019); (2) 1000 Genomes Project European samples data were referenced to calculate the linkage disequilibrium (LD) between SNPs, retaining only those with the lowest *P*-values and an R^2^ < 0.001 (Sudmant et al., 2015); (3) SNPs with an F-statistic < 10 were excluded to avoid weak instrumental bias (Burgess and Thompson, 2011).

### 2.4 Genetic analyses to elucidate causality

Bidirectional MR analyses were initially conducted to explore the causal relationship between the GM and aortic aneurysm diseases. The main method for causal estimation was the Wald method when only one SNP tool was available. When multiple valid instrumental variables were available, the inverse-variance weighted method (IVW) was employed for maximum efficiency. The IVW method meta-analyzed SNP-specific Wald estimates using random effects to obtain the final estimate of the causal effect (Burgess et al., 2019), which was reported in beta (β) value with standard error for continuous outcomes and odds ratio (OR) with a 95% confidence interval (CI) for binary outcomes; *P*-values < 0.05 were considered nominally significant. Results were adjusted for the False Discovery Rate (FDR) to account for multiple testing.

### 2.5 Sensitivity analyses

Methods such as MR-Presso, MR-Egger, weighted median, simple mode, and Mode-based estimate method were utilized for sensitivity analysis (Bowden et al., 2015; Hemani et al., 2018). Heterogeneity was assessed using the Cochran Q test, with a *P*-value less than 0.05 indicating significant heterogeneity. A Q value substantially exceeding its degrees of freedom indicates evidence of heterogeneity and suggests the presence of invalid instruments (Greco et al., 2015). MR-Egger regression and MR-Presso were utilized to analyze potential unbalanced horizontal pleiotropy, combining the Wald ratio into a meta-regression with an intercept and slope parameter. This approach estimates the causal effect while adjusting for any directional pleiotropy (Bowden et al., 2015). All statistical analyses were performed using R platform (version 4.3.1). MR analyses were performed using the packages including TwoSampleMR (version 0.5.7) (Hemani et al., 2018), MRPRESSO (version 1.0) (Verbanck et al., 2018), vroom (version 1.6.5), dplyr (version 1.1.4), and others.

### 2.6 Ethical approval and consent to participate

This study was based on publicly available data. Each GWAS within the study received approval from the relevant Institutional Review Board, and informed consent was secured from all participants or their respective legal representatives.

## 3 Results

For the 211 GM taxa from the MiBioGen consortium, 2,774 SNPs were used as IVs. For the 207 GM taxa from the DMP, 1,961 SNPs served as IVs. From the FinnGen database, 65, 56, and 24 SNPs were used as IVs for AAA, TAA, and AD, respectively. In the case of AAA data from the UK Biobank, 33 SNPs were utilized as IVs, and for TAA data from the MGI, 26 SNPs served as IVs. All SNPs included in our analysis had an F-statistic greater than 16, indicating that all are robust instruments (**Supplementary Additional File 2**).

**Figure 2:**
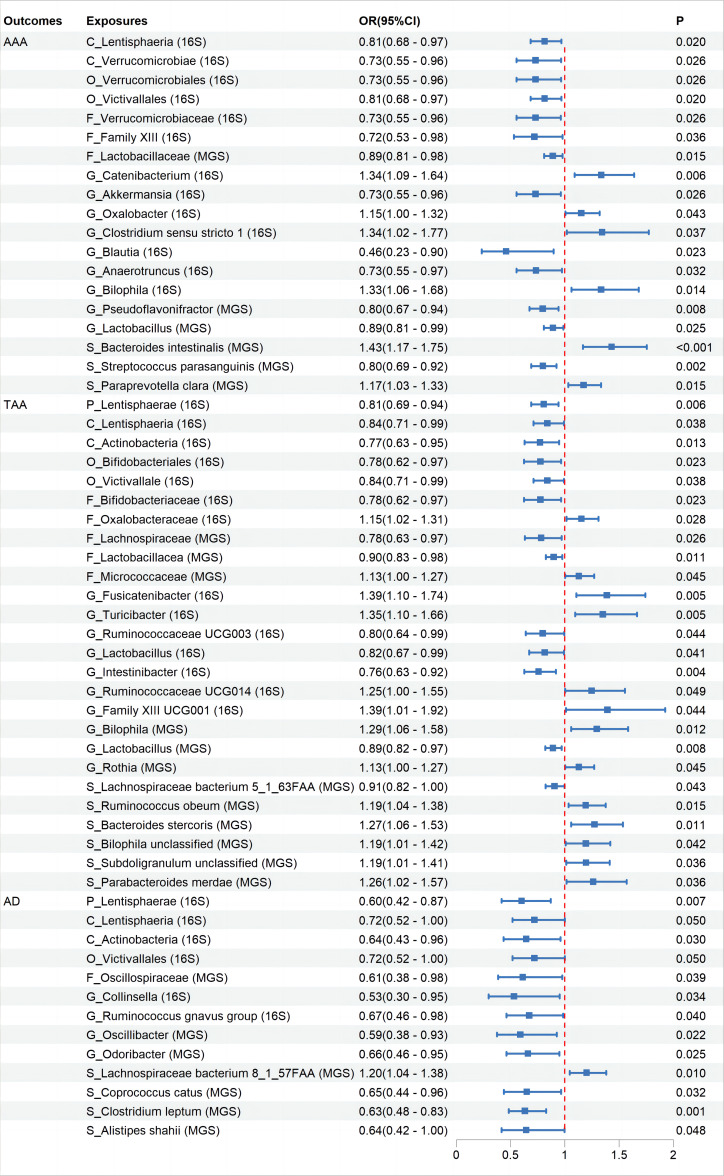
The association between Gut Microbiota and Aortic Aneurysm Disease using the inverse variance weighted method. OR, odds ratio; CI, confidence interval; *P*, probability value; AAA, Abdominal Aortic Aneurysm; TAA, Thoracic Aortic Aneurysm; AD, Aortic Dissection; 16S rRNA, 16S ribosomal RNA; MGS, Metagenomic Species.

None of the associations showed statistical significance after FDR correction (**Supplementary Additional File 3**). Specifically, while initial results suggested correlations between 19 taxa and AAA, 25 taxa and TAA, and 13 taxa with AD, these suggested associations did not hold statistical significance post-FDR correction (**Figure 2**, **Supplementary Figures 1**–**3**).All suggestive positive results are presented with visual information through “leave-one-out” sensitivity analysis, forest plots, scatter plots, and funnel plots (**Supplementary Additional File 5**).

Further MR analysis on the AAA and TAA validation groups revealed that replicating suggestive associations with microbiota categories in the validation sets was challenging. We evaluated whether the direction of effect for relevant microbiota in the validation sets was consistent with that in the initial groups, based on their OR levels. Results demonstrated that nine microbiota categories maintained consistency with AAA and ten microbiota categories maintained consistency with TAA (**Supplementary Figures 1**–**3**).

**Figure 3:**
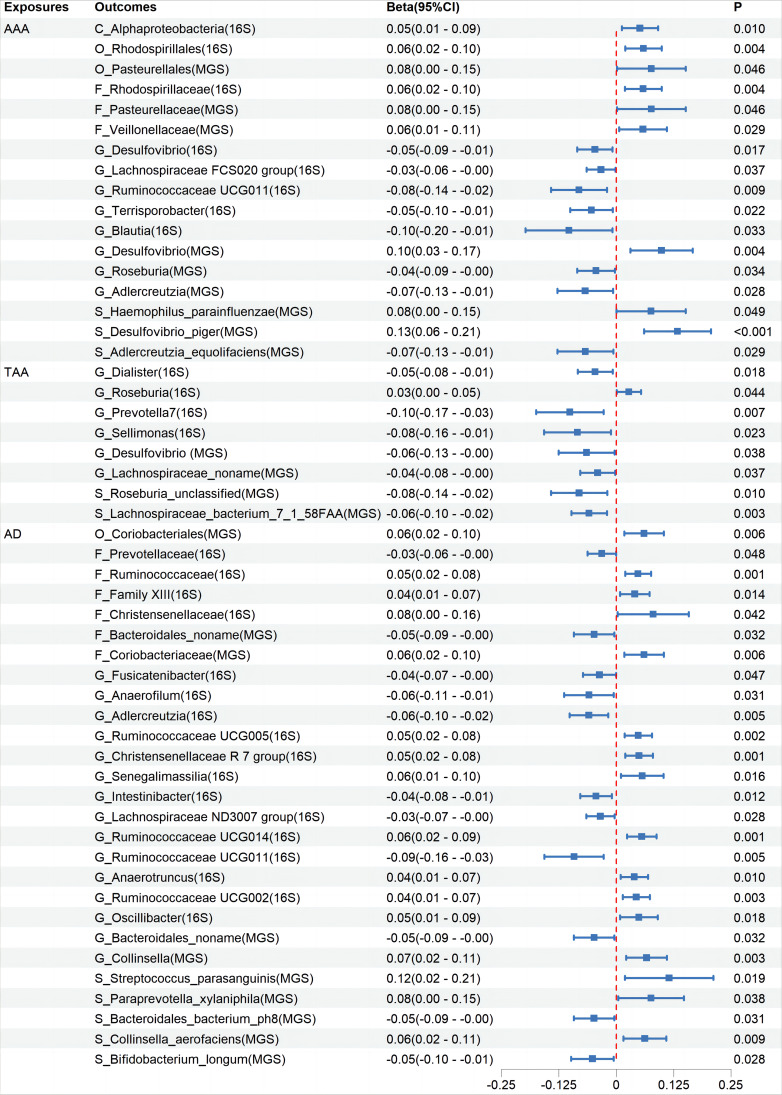
The association between Aortic Aneurysm Disease and Gut Microbiota using the inverse variance weighted method. CI, confidence interval; *P*, probability value; AAA, Abdominal Aortic Aneurysm; TAA, Thoracic Aortic Aneurysm; AD, Aortic Dissection; 16S rRNA, 16S ribosomal RNA; MGS, Metagenomic Species.

Cochran’s IVW Q test results showed no significant heterogeneity among these IVs (**Supplementary Additional File 4**: Order 1). Additionally, MR-PRESSO analysis indicated that there was pleiotropy in the relationship between Ruminococcaceae UCG003 and TAA (**Supplementary Additional File 4**: Order 3), but further analysis found no significant directional horizontal pleiotropy according to the MR-Egger regression intercept analysis (**Supplementary Additional File 4**: Order 2). Reverse MR analyses revealed bidirectional causal relationships between 17, 8, and 27 microbiota categories with AAA, TAA, and AD, respectively (**Figure 3**, **Supplementary Additional File 3**: Order 11-20), among which, Blautia showed a bidirectional causal relationship with AAA (**Supplementary Additional File 3**: Order 11).

## 4 Discussion

In this large-scale MR study, none of the identified associations between gut microbiota and aortic aneurysm diseases showed statistical significance after FDR correction. Despite this, the initial analysis combining information from two GM GWAS databases suggested potential associations between 19 taxa and AAA, 25 taxa and TAA, and 13 taxa and AD. Furthermore, the abundances of 16, 8, and 26 taxa were respectively affected by AAA, TAA, and AD.

In this MR study, we did not find significant associations between the GM and aortic aneurysm diseases after FDR correction, and several factors may contribute to this negative result. Aortic aneurysm diseases are relatively rare, leading to an insufficient sample size and thus inadequate statistical power to detect potential significant associations. As the sample size in GWAS databases related to aortic aneurysms increases in the future, the likelihood of identifying significant associations may also increase. The GM generally exerts its effects through alterations in microbial communities and microbially derived metabolites. These factors are influenced by the complex dynamics of microbial interactions and are particularly susceptible to modulation by diet, lifestyle, and environmental factors. For example, carnitine from red meat and phosphatidylcholine from egg yolks are metabolized by gut bacteria into trimethylamine, which is then converted into TMAO in the liver, a known contributor to various vascular diseases (Wang et al., 2011; Koeth et al., 2013). These confounding factors, along with the impact of host health on microbial function, may affect the pathogenic or protective roles of individual gut microbes as independent factors, resulting in low statistical significance in the analyses.

Despite the negative results after FDR correction, exploring the mechanisms behind the positive trends observed in the MR analysis of GM and aortic aneurysm diseases remains valuable. The pathogenesis of aortic aneurysmal diseases remains a subject of debate. Typical pathological changes include the degradation of the extracellular matrix, endothelial damage, inflammatory responses, and oxidative stress (Sakalihasan et al., 2005; Gao et al., 2023). GM and their secretions can influence the occurrence of aortic aneurysmal diseases through various mechanisms. As mentioned earlier, microbes can exert effects through their derived metabolites. Firstly, the TMAO pathway: TMAO increases PERK-mediated unfolded protein response, endoplasmic reticulum stress, and the expression of cleaved caspase-3, leading to increased apoptosis of vascular smooth muscle cells (VSMCs) (Benson et al., 2023). TMAO can also upregulate the expression of matrix metalloproteinase-2 (MMP-2) in aortic VSMCs, mediating extracellular matrix (ECM) degradation (Benson et al., 2023). Additionally, TMAO can induce vascular inflammation through various mechanisms, such as inducing mitochondrial reactive oxygen species production, activating the NLRP3 inflammasome, stimulating the NF-κB pathway, and releasing downstream pro-inflammatory cytokines (Liu and Dai, 2020). In this study, Akkermansia from the phylum Verrucomicrobia and Lactobacillus from the phylum Firmicutes were showed suggestive negatively associations with the disease, while Paraprevotella clara under the phylum Bacteroidetes, Bilophila from the phylum Proteobacteria, Ruminococcaceae UCG014 and Subdoligranulum from the family Ruminococcaceae, and Fusicatenibacter from the family Lachnospiraceae were showed suggestive positively associations with the disease. Research has shown corresponding abundance changes in these taxa in aortic aneurysms (Xie et al., 2020; Liu et al., 2022; Xiao et al., 2023) and they can influence disease development through the TMAO pathway (Liu et al., 2019; Fu et al., 2020; Liu et al., 2020; Franck et al., 2022; Luo et al., 2022; Gan et al., 2023; Jiang et al., 2023). Secondly, the butyrate pathway: butyrate can exert protective effects by maintaining the integrity of the gut barrier, inhibiting neutrophil infiltration and the formation of neutrophil extracellular traps, and downregulating the expression of MMP-2 and MMP-9 in the aortic wall, thereby reducing ECM elastin fiber degradation (Peng et al., 2009; Tian et al., 2022). Thirdly, the tryptophan pathway: Tryptophan, primarily secreted by Lactobacillus, and its metabolic product 3-IAId exert beneficial effects by inhibiting the phenotype transition of VSMCs from a contractile to a synthetic state. It also mitigates extracellular matrix degradation, attenuates macrophage infiltration, and suppresses the expression of inflammatory cytokines, collectively contributing to the attenuation of AD development (Huang et al., 2023). In this study, Lactobacillus from the phylum Firmicutes was showed suggestive negatively associations with AAA and TAA. Clinical studies have found that, compared to patients with atherosclerosis, AAA patients have a lower relative abundance of Firmicutes (Ji et al., 2021). Clinical trials have shown that taking Lactobacillus improved vascular endothelial function and decreased systemic inflammation in men (Malik et al., 2018). Additionally, GM can exert effects through direct colonization or by influencing the distribution of other microbiota. Bacteroides can directly invade and colonize in humans, promoting the occurrence of AAA diseases (Matsuoka et al., 2021). Akkermansia inhibited the formation of AAA by restoring gut microbiota diversity, altering the expression of peripheral immune factors, and the functions of E. coli, Clostridium, and Lactobacillus (He et al., 2022). Some taxa showing positive trends in this study have unclear mechanisms. For example, Family XIII shows a suggestive negative associations with AAA, while Victivallales from Verrucomicrobia and Lentisphaeria from Lentisphaerae are suggestive protective factors for TAA and AD. Oxalobacter from the phylum Proteobacteria has been identified as having a suggestive association as a potential risk factor for aortic aneurysmal diseases; however, studies on the species characteristics of the aforementioned microbial taxa are scarce. Further research is needed to validate these results and elucidate the potential mechanisms involved. Additionally, there is a contradiction with previous studies; this study found that Parabacteroides merdae from the phylum Bacteroidetes has a suggestive association as a potential risk factor for TAA. In contrast, past research suggests this species may be beneficial for aneurysmal diseases, as it can reduce inflammation and cardiovascular damage, with no evidence proving it as a risk factor for aortic aneurysmal diseases (Qiao et al., 2022; Chen et al., 2023; He et al., 2023).

Through this study, several points warrant further consideration. We found reverse causal relationships between aortic aneurysm diseases and certain GM. The development of aortic aneurysms is typically accompanied by systemic inflammation and metabolic disturbances. These changes may alter the gut environment and barrier function, affecting the distribution of GM and potentially leading to perioperative gastrointestinal complications associated with aortic aneurysm diseases. Moreover, it is important to note that many diseases, including aortic aneurysms, often undergo a prolonged progression before diagnosis or onset. During this period, changes in the levels of some bacteria might not necessarily be harmful. On the contrary, these changes might represent a compensatory response by the microbiome to mitigate adverse reactions in the host. This possibility should be understood within a broader context. This study has analyzed and discussed the impact of gut bacteria on aortic aneurysm diseases. In fact, opportunistic pathogens in the oral microbiome also play a significant role in the progression of cardiovascular diseases. Clinically, various pathological oral bacteria have been detected in aortic aneurysm specimens (Kurihara et al., 2004; Marques et al., 2006; Nakano et al., 2008; Ding et al., 2014; Salhi et al., 2021). Infection with Porphyromonas gingivalis can promote the phenotypic transition of human aortic smooth muscle cells from a contractile to a synthetic state (Inaba et al., 2009). Periodontitis-induced inflammation and oxidative stress can lead to arterial stiffness, vascular dysfunction, and hypertension (Czesnikiewicz-Guzik et al., 2019; Munoz et al., 2020), and can activate the renin-angiotensin system, causing the release of Angiotensin II (Viafara-Garcia et al., 2019). These factors might contribute to the development of aortic aneurysms. Additionally, various non-bacterial microorganisms in the gut may also influence disease development, such as fungi and viruses in the gut. For instance, cytomegalovirus infection can promote aortic inflammation (Jablonska et al., 2021). Therefore, when exploring the relationship between human microbiota and aortic aneurysm diseases, it is essential to consider this broader, comprehensive perspective.

The strengths of our study include leveraging the largest available GM GWAS and metagenomic sequencing databases, enabling species-level microbiota classification. We utilized the FinnGen database, the most comprehensive for aortic aneurysm diseases, and confirmed our findings with data from the UK Biobank and MGI. Our MR analysis, which uses genetic variants as instrumental variables, reduces the influence of confounding factors. However, this study also has some limitations. Initially, the accuracy and reliability of MR analysis largely depend on the sample size and representativeness. Both the GM and aortic aneurysm disease GWAS data are relatively small in volume, which may reduce the statistical power of the analysis and the generalizability of the results, necessitating further expansion with updated data to strengthen the findings. Furthermore, despite employing several methods to assess and adjust for potential heterogeneity or pleiotropic effects, we cannot entirely exclude the impact of unknown heterogeneity or pleiotropy. This could confound the causal inference and necessitates further validation through different analytical approaches or additional datasets. Additionally, the study primarily includes data from individuals of European descent, which may not fully capture the genetic diversity and microbiome variations present in other populations. This limitation calls for caution when generalizing the findings to other ethnic groups. Lastly, to further confirm the mechanistic relationship between the gut microbiome and aortic aneurysm diseases, further experimental research is necessary to elucidate the biological pathways involved.

In conclusion, this study provides valuable insights into the potential role of the GM in cardiovascular health, particularly concerning aortic aneurysm diseases. It paves the way for new research directions and highlights the potential for microbiome-based interventions in mitigating the risk of these severe conditions.

## Data availability statement

The datasets presented in this study can be found in online repositories. The names of the repository/repositories and accession number(s) can be found in the article/**Supplementary Material**.

## Ethics statement

Ethical approval was not required for the study involving humans in accordance with the local legislation and institutional requirements. Written informed consent to participate in this study was not required from the participants or the participants’ legal guardians/next of kin in accordance with the national legislation and the institutional requirements.

## Author contributions

YS: Conceptualization, Data curation, Investigation, Software, Visualization, Writing – original draft, Writing – review & editing, Formal analysis, Supervision. HD: Formal analysis, Writing – original draft, Data curation, Resources, Visualization. CS: Writing – original draft, Software. DD: Data curation, Formal analysis, Writing – review & editing. RG: Writing – review & editing, Resources. MV: Writing – review & editing, Validation. RK: Writing – review & editing, Conceptualization, Methodology, Supervision. NW: Writing – original draft.

## Supplementary material

Supplementary Additional File 1 Information on GWAS samples of gut microbiota used in this study

Supplementary Additional File 2 Detailed Information of Instrumental Variables used in this study

Supplementary Additional File 3 Mendelian randomization analysis results of gut microbiota and aortic aneurysm diseases

Supplementary Additional File 4 Heterogeneity, level pleiotropy test, and the power of Mendelian randomization analysis

Supplementary Additional File 5 The scatter plot effect size, leave-one-out analyses, and funnel plot for gut microbiota on gastrointestinal diseases in MR analysis.

Supplementary Figure 1 An overview of the Mendelian randomization study results on Gut Microbiota in relation to Abdominal Aortic Aneurysm. The analysis methods include inverse variance weighted, weighted median, and MR-PRESSO analysis. 16S rRNA = 16S ribosomal RNA; MGS = Metagenomic Species; SNP = single nucleotide polymorphism; IVW = inverse variance weighted; WM = weighted median; *P* = probability value

Supplementary Figure 2 An overview of the Mendelian randomization study results on Gut Microbiota in relation to Thoracic Aortic Aneurysm. The analysis methods include inverse variance weighted, weighted median, and MR-PRESSO analysis. 16S rRNA = 16S ribosomal RNA; MGS = Metagenomic Species; SNP = single nucleotide polymorphism; IVW = inverse variance weighted; WM = weighted median; *P* = probability value.

Supplementary Figure 3 An overview of the Mendelian randomization study results on Gut Microbiota in relation to Aortic Dissection. The analysis methods include inverse variance weighted, weighted median, and MR-PRESSO analysis. 16S rRNA = 16S ribosomal RNA; MGS = Metagenomic Species; SNP = single nucleotide polymorphism; IVW = inverse variance weighted; WM = weighted median; *P* = probability value.

## References

[B1] Benson T. W. Conrad K. A. Li X. S. Wang Z. Helsley R. N. Schugar R. C.. (2023). Gut microbiota-derived trimethylamine n-oxide contributes to abdominal aortic aneurysm through inflammatory and apoptotic mechanisms. Circulation 147, 1079–1096. doi: 10.1161/CIRCULATIONAHA.122.060573 37011073 PMC10071415

[B2] Bossone E. Eagle K. A. (2021). Epidemiology and management of aortic disease: aortic aneurysms and acute aortic syndromes. Nat. Rev. Cardiol. 18, 331–348. doi: 10.1038/s41569-020-00472-6 33353985

[B3] Bowden J. Davey S. G. Burgess S. (2015). Mendelian randomization with invalid instruments: effect estimation and bias detection through egger regression. Int. J. Epidemiol. 44, 512–525. doi: 10.1093/ije/dyv080 26050253 PMC4469799

[B4] Burgess S. Davey S. G. Davies N. M. Dudbridge F. Gill D. Glymour M. M.. (2019). Guidelines for performing mendelian randomization investigations: update for summer 2023. Wellcome Open Res. 4, 186. doi: 10.12688/wellcomeopenres.15555.3 32760811 PMC7384151

[B5] Burgess S. Thompson S. G. (2011). Avoiding bias from weak instruments in mendelian randomization studies. Int. J. Epidemiol. 40, 755–764. doi: 10.1093/ije/dyr036 21414999

[B6] Chen X. Wu R. Li L. Zeng Y. Chen J. Wei M.. (2023). Pregnancy-induced changes to the gut microbiota drive macrophage pyroptosis and exacerbate septic inflammation. Immunity 56, 336–352. doi: 10.1016/j.immuni.2023.01.015 36792573

[B7] Czesnikiewicz-Guzik M. Osmenda G. Siedlinski M. Nosalski R. Pelka P. Nowakowski D.. (2019). Causal association between periodontitis and hypertension: evidence from mendelian randomization and a randomized controlled trial of non-surgical periodontal therapy. Eur. Heart J. 40, 3459–3470. doi: 10.1093/eurheartj/ehz646 31504461 PMC6837161

[B8] Davies N. M. Holmes M. V. Davey S. G. (2018). Reading mendelian randomisation studies: a guide, glossary, and checklist for clinicians. BMJ. 362, k601. doi: 10.1136/bmj.k601 30002074 PMC6041728

[B9] Ding F. Lyu Y. Han X. Zhang H. Liu D. Hei W.. (2014). Detection of periodontal pathogens in the patients with aortic aneurysm. Chin. Med. J. (Engl). 127, 4114–4118. doi: 10.3760/cma.j.issn.0366-6999.20141208 25430459

[B10] Franck M. de Toro-Martin J. Varin T. V. Garneau V. Pilon G. Roy D.. (2022). Gut microbial signatures of distinct trimethylamine n-oxide response to raspberry consumption. Nutrients 14 (8), 1656. doi: 10.3390/nu14081656 35458219 PMC9027468

[B11] Fu B. C. Hullar M. Randolph T. W. Franke A. A. Monroe K. R. Cheng I.. (2020). Associations of plasma trimethylamine n-oxide, choline, carnitine, and betaine with inflammatory and cardiometabolic risk biomarkers and the fecal microbiome in the multiethnic cohort adiposity phenotype study. Am. J. Clin. Nutr. 111, 1226–1234. doi: 10.1093/ajcn/nqaa015 32055828 PMC7266689

[B12] Gan G. Zhang R. Lu B. Luo Y. Chen S. Lei H.. (2023). Gut microbiota may mediate the impact of chronic apical periodontitis on atherosclerosis in apolipoprotein e-deficient mice. Int. Endod. J. 56, 53–68. doi: 10.1111/iej.13845 36208054

[B13] Gao J. Cao H. Hu G. Wu Y. Xu Y. Cui H.. (2023). The mechanism and therapy of aortic aneurysms. Signal Transduction Targeting Ther. 8, 55. doi: 10.1038/s41392-023-01325-7 PMC9898314 36737432

[B14] Greco M. F. Minelli C. Sheehan N. A. Thompson J. R. (2015). Detecting pleiotropy in mendelian randomisation studies with summary data and a continuous outcome. Stat. Med. 34, 2926–2940. doi: 10.1002/sim.6522 25950993

[B15] He X. Bai Y. Zhou H. Wu K. (2022). Akkermansia muciniphila alters gut microbiota and immune system to improve cardiovascular diseases in murine model. Front. Microbiol. 13. doi: 10.3389/fmicb.2022.906920 PMC9237526 35774450

[B16] He Z. Zhu H. Liu J. Kwek E. Ma K. Y. Chen Z. Y. (2023). Mangiferin alleviates trimethylamine-n-oxide (tmao)-induced atherogenesis and modulates gut microbiota in mice. Food Funct. 14, 9212–9225. doi: 10.1039/D3FO02791K 37781894

[B17] Hemani G. Zheng J. Elsworth B. Wade K. H. Haberland V. Baird D.. (2018). The mr-base platform supports systematic causal inference across the human phenome. Elife 7, e34408. doi: 10.7554/eLife.34408 29846171 PMC5976434

[B18] Huang S. S. Liu R. Chang S. Li X. Weng X. Ge J. (2023). Gut microbiota-derived tryptophan metabolite indole-3-aldehyde ameliorates aortic dissection. Nutrients 15 (19), 4150. doi: 10.3390/nu15194150 37836434 PMC10574575

[B19] Inaba H. Hokamura K. Nakano K. Nomura R. Katayama K. Nakajima A.. (2009). Upregulation of s100 calcium-binding protein a9 is required for induction of smooth muscle cell proliferation by a periodontal pathogen. FEBS Lett. 583, 128–134. doi: 10.1016/j.febslet.2008.11.036 19059406

[B20] Isselbacher E. M. Preventza O. Hamilton B. J. R. Augoustides J. G. Beck A. W. Bolen M. A.. (2022). 2022 acc/aha guideline for the diagnosis and management of aortic disease: a report of the american heart association/american college of cardiology joint committee on clinical practice guidelines. Circulation 146, e334–e482. doi: 10.1161/CIR.0000000000001106 36322642 PMC9876736

[B21] Ito E. Ohki T. Toya N. Nakagawa H. Horigome A. Odamaki T.. (2023). Impact of bifidobacterium adolescentis in patients with abdominal aortic aneurysm: a cross-sectional study. Biosci. Microbiota Food Health 42, 81–86. doi: 10.12938/bmfh.2022-055 36660598 PMC9816055

[B22] Jablonska A. Zagrapan B. Paradowska E. Neumayer C. Eilenberg W. Brostjan C.. (2021). Abdominal aortic aneurysm and virus infection: a potential causative role for cytomegalovirus infection? J. Med. Virol. 93, 5017–5024. doi: 10.1002/jmv.26901 33629381

[B23] Ji L. Gu G. C. Chen S. L. Wang W. Ren J. R. Li F. D.. (2021). [Differences of gut microbiota diversity between patients with abdominal aortic aneurysm and atherosclerosis]. Zhongguo Yi Xue Ke Xue Yuan Xue Bao 43, 677–684. doi: 10.3881/j.issn.1000-503X.13443 34728028

[B24] Jiang F. Cai M. Peng Y. Li S. Liang B. Ni H.. (2023). Changes in the gut microbiome of patients with type a aortic dissection. Front. Microbiol. 14. doi: 10.3389/fmicb.2023.1092360 PMC9992204 36910178

[B25] Johnston K. W. Rutherford R. B. Tilson M. D. Shah D. M. Hollier L. Stanley J. C. (1991). Suggested standards for reporting on arterial aneurysms. Subcommittee on reporting standards for arterial aneurysms, *ad hoc* committee on reporting standards, society for vascular surgery and north american chapter, international society for cardiovascular surgery. J. Vasc. Surg. 13, 452–458. doi: 10.1067/mva.1991.26737 1999868

[B26] Koeth R. A. Wang Z. Levison B. S. Buffa J. A. Org E. Sheehy B. T.. (2013). Intestinal microbiota metabolism of l-carnitine, a nutrient in red meat, promotes atherosclerosis. Nat. Med. 19, 576–585. doi: 10.1038/nm.3145 23563705 PMC3650111

[B27] Kurihara N. Inoue Y. Iwai T. Umeda M. Huang Y. Ishikawa I. (2004). Detection and localization of periodontopathic bacteria in abdominal aortic aneurysms. Eur. J. Vasc. Endovasc. Surg. 28, 553–558. doi: 10.1016/j.ejvs.2004.08.010 15465379

[B28] Kurilshikov A. Medina-Gomez C. Bacigalupe R. Radjabzadeh D. Wang J. Demirkan A.. (2021). Large-scale association analyses identify host factors influencing human gut microbiome composition. Nat. Genet. 53, 156–165. doi: 10.1038/s41588-020-00763-1 33462485 PMC8515199

[B29] Lawlor D. A. (2016). Commentary: two-sample mendelian randomization: opportunities and challenges. Int. J. Epidemiol. 45, 908–915. doi: 10.1093/ije/dyw127 27427429 PMC5005949

[B30] Liu Y. Dai M. (2020). Trimethylamine n-oxide generated by the gut microbiota is associated with vascular inflammation: new insights into atherosclerosis. Mediat. Inflamm. 2020, 4634172. doi: 10.1155/2020/4634172 PMC7048942 32148438

[B31] Liu J. Lai L. Lin J. Zheng J. Nie X. Zhu X.. (2020). Ranitidine and finasteride inhibit the synthesis and release of trimethylamine n-oxide and mitigates its cardiovascular and renal damage through modulating gut microbiota. Int. J. Biol. Sci. 16, 790–802. doi: 10.7150/ijbs.40934 32071549 PMC7019130

[B32] Liu J. Li T. Wu H. Shi H. Bai J. Zhao W.. (2019). Lactobacillus rhamnosus gg strain mitigated the development of obstructive sleep apnea-induced hypertension in a high salt diet *via* regulating tmao level and cd4(+) t cell induced-type i inflammation. Biomed. Pharmacother. 112, 108580. doi: 10.1016/j.biopha.2019.01.041 30784906

[B33] Liu S. Liu Y. Zhao J. Yang P. Wang W. Liao M. (2022). Effects of spermidine on gut microbiota modulation in experimental abdominal aortic aneurysm mice. Nutrients 14 (116), 3349. doi: 10.3390/nu14163349 36014855 PMC9415871

[B34] Lopera-Maya E. A. Kurilshikov A. van der Graaf A. Hu S. Andreu-Sanchez S. Chen L.. (2022). Effect of host genetics on the gut microbiome in 7,738 participants of the dutch microbiome project. Nat. Genet. 54, 143–151. doi: 10.1038/s41588-021-00992-y 35115690

[B35] Luo Y. Zhang Y. Han X. Yuan Y. Zhou Y. Gao Y.. (2022). Akkermansia muciniphila prevents cold-related atrial fibrillation in rats by modulation of tmao induced cardiac pyroptosis. EBioMedicine 82, 104087. doi: 10.1016/j.ebiom.2022.104087 35797768 PMC9270211

[B36] Malik M. Suboc T. M. Tyagi S. Salzman N. Wang J. Ying R.. (2018). Lactobacillus plantarum 299v supplementation improves vascular endothelial function and reduces inflammatory biomarkers in men with stable coronary artery disease. Circ. Res. 123, 1091–1102. doi: 10.1161/CIRCRESAHA.118.313565 30355158 PMC6205737

[B37] Manabe Y. Ishibashi T. Asano R. Tonomura S. Maeda Y. Motooka D.. (2023). Gut dysbiosis is associated with aortic aneurysm formation and progression in takayasu arteritis. Arthritis Res. Ther. 25, 46. doi: 10.1186/s13075-023-03031-9 36964623 PMC10037851

[B38] Marques D. S. R. Caugant D. A. Eribe E. R. Aas J. A. Lingaas P. S. Geiran O.. (2006). Bacterial diversity in aortic aneurysms determined by 16s ribosomal rna gene analysis. J. Vasc. Surg. 44, 1055–1060. doi: 10.1016/j.jvs.2006.07.021 17098542

[B39] Matsuoka T. Shimizu T. Minagawa T. Hiranuma W. Takeda M. Kakuta R.. (2021). First case of an invasive bacteroides dorei infection detected in a patient with a mycotic aortic aneurysm-raising a rebellion of major indigenous bacteria in humans: a case report and review. BMC Infect. Dis. 21, 625. doi: 10.1186/s12879-021-06345-8 34193073 PMC8247135

[B40] Mcclure R. S. Brogly S. B. Lajkosz K. Mcclintock C. Payne D. Smith H. N.. (2020). Economic burden and healthcare resource use for thoracic aortic dissections and thoracic aortic aneurysms-a population-based cost-of-illness analysis. J. Am. Heart Assoc. 9, e14981. doi: 10.1161/JAHA.119.014981 PMC7428990 32458716

[B41] Munoz A. E. Suvan J. Buti J. Czesnikiewicz-Guzik M. Barbosa R. A. Orlandi M.. (2020). Periodontitis is associated with hypertension: a systematic review and meta-analysis. Cardiovasc. Res. 116, 28–39. doi: 10.1093/cvr/cvz201 31549149

[B42] Nakano K. Inaba H. Nomura R. Nemoto H. Takeuchi H. Yoshioka H.. (2008). Distribution of porphyromonas gingivalis fima genotypes in cardiovascular specimens from Japanese patients. Oral. Microbiol. Immunol. 23, 170–172. doi: 10.1111/j.1399-302X.2007.00406.x 18279186

[B43] Nakayama K. Furuyama T. Matsubara Y. Morisaki K. Onohara T. Ikeda T.. (2022). Gut dysbiosis and bacterial translocation in the aneurysmal wall and blood in patients with abdominal aortic aneurysm. PLoS One 17, e278995. doi: 10.1371/journal.pone.0278995 PMC9749999 36516156

[B44] Peng L. Li Z. R. Green R. S. Holzman I. R. Lin J. (2009). Butyrate enhances the intestinal barrier by facilitating tight junction assembly *via* activation of amp-activated protein kinase in caco-2 cell monolayers. J. Nutr. 139, 1619–1625. doi: 10.3945/jn.109.104638 19625695 PMC2728689

[B45] Pingault J. B. O'Reilly P. F. Schoeler T. Ploubidis G. B. Rijsdijk F. Dudbridge F. (2018). Using genetic data to strengthen causal inference in observational research. Nat. Rev. Genet. 19, 566–580. doi: 10.1038/s41576-018-0020-3 29872216

[B46] Qiao S. Liu C. Sun L. Wang T. Dai H. Wang K.. (2022). Gut parabacteroides merdae protects against cardiovascular damage by enhancing branched-chain amino acid catabolism. Nat. Metab. 4, 1271–1286. doi: 10.1038/s42255-022-00649-y 36253620

[B47] Roychowdhury T. Lu H. Hornsby W. E. Crone B. Wang G. T. Guo D. C.. (2021). Regulatory variants in tcf7l2 are associated with thoracic aortic aneurysm. Am. J. Hum. Genet. 108, 1578–1589. doi: 10.1016/j.ajhg.2021.06.016 34265237 PMC8456156

[B48] Sakalihasan N. Limet R. Defawe O. D. (2005). Abdominal aortic aneurysm. Lancet 365, 1577–1589. doi: 10.1016/S0140-6736(05)66459-8 15866312

[B49] Salhi L. Rijkschroeff P. Van Hede D. Laine M. L. Teughels W. Sakalihasan N.. (2021). Blood biomarkers and serologic immunological profiles related to periodontitis in abdominal aortic aneurysm patients. Front. Cell. Infect. Microbiol. 11. doi: 10.3389/fcimb.2021.766462 PMC8798408 35096635

[B50] Sanna S. van Zuydam N. R. Mahajan A. Kurilshikov A. Vich V. A. Vosa U.. (2019). Causal relationships among the gut microbiome, short-chain fatty acids and metabolic diseases. Nat. Genet. 51, 600–605. doi: 10.1038/s41588-019-0350-x 30778224 PMC6441384

[B51] Shinohara R. Nakashima H. Emoto T. Yamashita T. Saito Y. Yoshida N.. (2022). Gut microbiota influence the development of abdominal aortic aneurysm by suppressing macrophage accumulation in mice. Hypertension 79, 2821–2829. doi: 10.1161/HYPERTENSIONAHA.122.19422 36252141

[B52] Sudmant P. H. Rausch T. Gardner E. J. Handsaker R. E. Abyzov A. Huddleston J.. (2015). An integrated map of structural variation in 2,504 human genomes. Nature 526, 75–81. doi: 10.1038/nature15394 26432246 PMC4617611

[B53] Tang W. Li D. Y. Hazen S. L. (2019). Dietary metabolism, the gut microbiome, and heart failure. Nat. Rev. Cardiol. 16, 137–154. doi: 10.1038/s41569-018-0108-7 30410105 PMC6377322

[B54] Tian Z. Zhang Y. Zheng Z. Zhang M. Zhang T. Jin J.. (2022). Gut microbiome dysbiosis contributes to abdominal aortic aneurysm by promoting neutrophil extracellular trap formation. Cell Host Microbe 30, 1450–1463. doi: 10.1016/j.chom.2022.09.004 36228585

[B55] Verbanck M. Chen C. Y. Neale B. Do R. (2018). Detection of widespread horizontal pleiotropy in causal relationships inferred from mendelian randomization between complex traits and diseases. Nat. Genet. 50, 693–698. doi: 10.1038/s41588-018-0099-7 29686387 PMC6083837

[B56] Viafara-Garcia S. M. Morantes S. J. Chacon-Quintero Y. Castillo D. M. Lafaurie G. I. Buitrago D. M. (2019). Repeated porphyromonas gingivalis w83 exposure leads to release pro-inflammatory cytokynes and angiotensin ii in coronary artery endothelial cells. Sci. Rep. 9, 19379. doi: 10.1038/s41598-019-54259-y 31852912 PMC6920421

[B57] Wang Z. Klipfell E. Bennett B. J. Koeth R. Levison B. S. Dugar B.. (2011). Gut flora metabolism of phosphatidylcholine promotes cardiovascular disease. Nature 472, 57–63. doi: 10.1038/nature09922 21475195 PMC3086762

[B58] Witkowski M. Weeks T. L. Hazen S. L. (2020). Gut microbiota and cardiovascular disease. Circ. Res. 127, 553–570. doi: 10.1161/CIRCRESAHA.120.316242 32762536 PMC7416843

[B59] Xiao J. Wei Z. Yang C. Dai S. Wang X. Shang Y. (2023). The gut microbiota in experimental abdominal aortic aneurysm. Front. Cardiovasc. Med. 10. doi: 10.3389/fcvm.2023.1051648 PMC9992639 36910527

[B60] Xie J. Lu W. Zhong L. Hu Y. Li Q. Ding R.. (2020). Alterations in gut microbiota of abdominal aortic aneurysm mice. BMC Cardiovasc. Disord. 20, 32. doi: 10.1186/s12872-020-01334-2 31992206 PMC6988222

