# Supplementary material for: Investigating the association between gut microbiome and aortic aneurysm diseases: a bidirectional two-sample Mendelian randomization analysis
Source: Front Cell Infect Microbiol. 2024 Jul 30;14:1406845. doi: 10.3389/fcimb.2024.1406845 (PMC11319299; doi:10.3389/fcimb.2024.1406845)
Supplement: Supplementary Additional File 5 — The scatter plot effect size, leave-one-out analyses, and funnel plot for gut microbiota on gastrointestinal diseases in MR analysis. [file DataSheet_1.pdf]

page	Exposure (ID)	Outcome (ID)	Trait
1	ebi-a-GCST90016918	Abdominal Aortic Aneurysm (FinnGen)	leave-one-out analyses
2	ebi-a-GCST90016918	Abdominal Aortic Aneurysm (FinnGen)	funnel plot
3	ebi-a-GCST90016918	Abdominal Aortic Aneurysm (FinnGen)	MR effect size
4	ebi-a-GCST90016918	Abdominal Aortic Aneurysm (FinnGen)	scatter plot
5	ebi-a-GCST90016923	Abdominal Aortic Aneurysm (FinnGen)	leave-one-out analyses
6	ebi-a-GCST90016923	Abdominal Aortic Aneurysm (FinnGen)	funnel plot
7	ebi-a-GCST90016923	Abdominal Aortic Aneurysm (FinnGen)	MR effect size
8	ebi-a-GCST90016923	Abdominal Aortic Aneurysm (FinnGen)	scatter plot
9	ebi-a-GCST90016939	Abdominal Aortic Aneurysm (FinnGen)	leave-one-out analyses
10	ebi-a-GCST90016939	Abdominal Aortic Aneurysm (FinnGen)	funnel plot
11	ebi-a-GCST90016939	Abdominal Aortic Aneurysm (FinnGen)	MR effect size
12	ebi-a-GCST90016939	Abdominal Aortic Aneurysm (FinnGen)	scatter plot
13	ebi-a-GCST90016957	Abdominal Aortic Aneurysm (FinnGen)	leave-one-out analyses
14	ebi-a-GCST90016957	Abdominal Aortic Aneurysm (FinnGen)	funnel plot
15	ebi-a-GCST90016957	Abdominal Aortic Aneurysm (FinnGen)	MR effect size
16	ebi-a-GCST90016957	Abdominal Aortic Aneurysm (FinnGen)	scatter plot
17	ebi-a-GCST90016961	Abdominal Aortic Aneurysm (FinnGen)	leave-one-out analyses
18	ebi-a-GCST90016961	Abdominal Aortic Aneurysm (FinnGen)	funnel plot
19	ebi-a-GCST90016961	Abdominal Aortic Aneurysm (FinnGen)	MR effect size
20	ebi-a-GCST90016961	Abdominal Aortic Aneurysm (FinnGen)	scatter plot
21	ebi-a-GCST90016967	Abdominal Aortic Aneurysm (FinnGen)	leave-one-out analyses
22	ebi-a-GCST90016967	Abdominal Aortic Aneurysm (FinnGen)	funnel plot
23	ebi-a-GCST90016967	Abdominal Aortic Aneurysm (FinnGen)	MR effect size
24	ebi-a-GCST90016967	Abdominal Aortic Aneurysm (FinnGen)	scatter plot
25	ebi-a-GCST90016971	Abdominal Aortic Aneurysm (FinnGen)	leave-one-out analyses
26	ebi-a-GCST90016971	Abdominal Aortic Aneurysm (FinnGen)	funnel plot
27	ebi-a-GCST90016971	Abdominal Aortic Aneurysm (FinnGen)	MR effect size
28	ebi-a-GCST90016971	Abdominal Aortic Aneurysm (FinnGen)	scatter plot
29	ebi-a-GCST90016972	Abdominal Aortic Aneurysm (FinnGen)	funnel plot
30	ebi-a-GCST90016972	Abdominal Aortic Aneurysm (FinnGen)	MR effect size
31	ebi-a-GCST90016972	Abdominal Aortic Aneurysm (FinnGen)	scatter plot
32	ebi-a-GCST90016977	Abdominal Aortic Aneurysm (FinnGen)	leave-one-out analyses
33	ebi-a-GCST90016977	Abdominal Aortic Aneurysm (FinnGen)	funnel plot
34	ebi-a-GCST90016977	Abdominal Aortic Aneurysm (FinnGen)	MR effect size
35	ebi-a-GCST90016977	Abdominal Aortic Aneurysm (FinnGen)	scatter plot
36	ebi-a-GCST90016980	Abdominal Aortic Aneurysm (FinnGen)	leave-one-out analyses
37	ebi-a-GCST90016980	Abdominal Aortic Aneurysm (FinnGen)	funnel plot
38	ebi-a-GCST90016980	Abdominal Aortic Aneurysm (FinnGen)	MR effect size

39	ebi-a-GCST90016980	Abdominal Aortic Aneurysm (FinnGen)	scatter plot
40	ebi-a-GCST90017038	Abdominal Aortic Aneurysm (FinnGen)	leave-one-out analyses
41	ebi-a-GCST90017038	Abdominal Aortic Aneurysm (FinnGen)	funnel plot
42	ebi-a-GCST90017038	Abdominal Aortic Aneurysm (FinnGen)	MR effect size
43	ebi-a-GCST90017038	Abdominal Aortic Aneurysm (FinnGen)	scatter plot
44	ebi-a-GCST90017108	Abdominal Aortic Aneurysm (FinnGen)	leave-one-out analyses
45	ebi-a-GCST90017108	Abdominal Aortic Aneurysm (FinnGen)	funnel plot
46	ebi-a-GCST90017108	Abdominal Aortic Aneurysm (FinnGen)	MR effect size
47	ebi-a-GCST90017108	Abdominal Aortic Aneurysm (FinnGen)	scatter plot
48	ebi-a-GCST90017109	Abdominal Aortic Aneurysm (FinnGen)	leave-one-out analyses
49	ebi-a-GCST90017109	Abdominal Aortic Aneurysm (FinnGen)	funnel plot
50	ebi-a-GCST90017109	Abdominal Aortic Aneurysm (FinnGen)	MR effect size
51	ebi-a-GCST90017109	Abdominal Aortic Aneurysm (FinnGen)	scatter plot
52	ebi-a-GCST90027669	Abdominal Aortic Aneurysm (FinnGen)	leave-one-out analyses
53	ebi-a-GCST90027669	Abdominal Aortic Aneurysm (FinnGen)	funnel plot
54	ebi-a-GCST90027669	Abdominal Aortic Aneurysm (FinnGen)	MR effect size
55	ebi-a-GCST90027669	Abdominal Aortic Aneurysm (FinnGen)	scatter plot
56	ebi-a-GCST90027702	Abdominal Aortic Aneurysm (FinnGen)	leave-one-out analyses
57	ebi-a-GCST90027702	Abdominal Aortic Aneurysm (FinnGen)	funnel plot
58	ebi-a-GCST90027702	Abdominal Aortic Aneurysm (FinnGen)	MR effect size
59	ebi-a-GCST90027702	Abdominal Aortic Aneurysm (FinnGen)	scatter plot
60	ebi-a-GCST90027706	Abdominal Aortic Aneurysm (FinnGen)	leave-one-out analyses
61	ebi-a-GCST90027706	Abdominal Aortic Aneurysm (FinnGen)	funnel plot
62	ebi-a-GCST90027706	Abdominal Aortic Aneurysm (FinnGen)	MR effect size
63	ebi-a-GCST90027706	Abdominal Aortic Aneurysm (FinnGen)	scatter plot
64	ebi-a-GCST90027771	Abdominal Aortic Aneurysm (FinnGen)	leave-one-out analyses
65	ebi-a-GCST90027771	Abdominal Aortic Aneurysm (FinnGen)	funnel plot
66	ebi-a-GCST90027771	Abdominal Aortic Aneurysm (FinnGen)	MR effect size
67	ebi-a-GCST90027771	Abdominal Aortic Aneurysm (FinnGen)	scatter plot
68	ebi-a-GCST90027783	Abdominal Aortic Aneurysm (FinnGen)	leave-one-out analyses
69	ebi-a-GCST90027783	Abdominal Aortic Aneurysm (FinnGen)	funnel plot
70	ebi-a-GCST90027783	Abdominal Aortic Aneurysm (FinnGen)	MR effect size
71	ebi-a-GCST90027783	Abdominal Aortic Aneurysm (FinnGen)	scatter plot
72	ebi-a-GCST90027829	Abdominal Aortic Aneurysm (FinnGen)	leave-one-out analyses
73	ebi-a-GCST90027829	Abdominal Aortic Aneurysm (FinnGen)	funnel plot
74	ebi-a-GCST90027829	Abdominal Aortic Aneurysm (FinnGen)	MR effect size
75	ebi-a-GCST90027829	Abdominal Aortic Aneurysm (FinnGen)	scatter plot
76	ebi-a-GCST90016908	Thoracic Aortic Aneurysm (FinnGen)	leave-one-out analyses
77	ebi-a-GCST90016908	Thoracic Aortic Aneurysm (FinnGen)	funnel plot

78	ebi-a-GCST90016908	Thoracic Aortic Aneurysm (FinnGen)	MR effect size
79	ebi-a-GCST90016908	Thoracic Aortic Aneurysm (FinnGen)	scatter plot
80	ebi-a-GCST90016918	Thoracic Aortic Aneurysm (FinnGen)	leave-one-out analyses
81	ebi-a-GCST90016918	Thoracic Aortic Aneurysm (FinnGen)	funnel plot
82	ebi-a-GCST90016918	Thoracic Aortic Aneurysm (FinnGen)	MR effect size
83	ebi-a-GCST90016918	Thoracic Aortic Aneurysm (FinnGen)	scatter plot
84	ebi-a-GCST90016929	Thoracic Aortic Aneurysm (FinnGen)	leave-one-out analyses
85	ebi-a-GCST90016929	Thoracic Aortic Aneurysm (FinnGen)	funnel plot
86	ebi-a-GCST90016929	Thoracic Aortic Aneurysm (FinnGen)	MR effect size
87	ebi-a-GCST90016929	Thoracic Aortic Aneurysm (FinnGen)	scatter plot
88	ebi-a-GCST90016943	Thoracic Aortic Aneurysm (FinnGen)	leave-one-out analyses
89	ebi-a-GCST90016943	Thoracic Aortic Aneurysm (FinnGen)	funnel plot
90	ebi-a-GCST90016943	Thoracic Aortic Aneurysm (FinnGen)	MR effect size
91	ebi-a-GCST90016943	Thoracic Aortic Aneurysm (FinnGen)	scatter plot
92	ebi-a-GCST90017009	Thoracic Aortic Aneurysm (FinnGen)	leave-one-out analyses
93	ebi-a-GCST90017009	Thoracic Aortic Aneurysm (FinnGen)	funnel plot
94	ebi-a-GCST90017009	Thoracic Aortic Aneurysm (FinnGen)	MR effect size
95	ebi-a-GCST90017009	Thoracic Aortic Aneurysm (FinnGen)	scatter plot
96	ebi-a-GCST90017011	Thoracic Aortic Aneurysm (FinnGen)	leave-one-out analyses
97	ebi-a-GCST90017011	Thoracic Aortic Aneurysm (FinnGen)	funnel plot
98	ebi-a-GCST90017011	Thoracic Aortic Aneurysm (FinnGen)	MR effect size
99	ebi-a-GCST90017011	Thoracic Aortic Aneurysm (FinnGen)	scatter plot
100	ebi-a-GCST90017018	Thoracic Aortic Aneurysm (FinnGen)	leave-one-out analyses
101	ebi-a-GCST90017018	Thoracic Aortic Aneurysm (FinnGen)	funnel plot
102	ebi-a-GCST90017018	Thoracic Aortic Aneurysm (FinnGen)	MR effect size
103	ebi-a-GCST90017018	Thoracic Aortic Aneurysm (FinnGen)	scatter plot
104	ebi-a-GCST90017030	Thoracic Aortic Aneurysm (FinnGen)	leave-one-out analyses
105	ebi-a-GCST90017030	Thoracic Aortic Aneurysm (FinnGen)	funnel plot
106	ebi-a-GCST90017030	Thoracic Aortic Aneurysm (FinnGen)	MR effect size
107	ebi-a-GCST90017030	Thoracic Aortic Aneurysm (FinnGen)	scatter plot
108	ebi-a-GCST90017054	Thoracic Aortic Aneurysm (FinnGen)	leave-one-out analyses
109	ebi-a-GCST90017054	Thoracic Aortic Aneurysm (FinnGen)	funnel plot
110	ebi-a-GCST90017054	Thoracic Aortic Aneurysm (FinnGen)	MR effect size
111	ebi-a-GCST90017054	Thoracic Aortic Aneurysm (FinnGen)	scatter plot
112	ebi-a-GCST90017061	Thoracic Aortic Aneurysm (FinnGen)	leave-one-out analyses
113	ebi-a-GCST90017061	Thoracic Aortic Aneurysm (FinnGen)	funnel plot
114	ebi-a-GCST90017061	Thoracic Aortic Aneurysm (FinnGen)	MR effect size
115	ebi-a-GCST90017061	Thoracic Aortic Aneurysm (FinnGen)	scatter plot
116	ebi-a-GCST90017074	Thoracic Aortic Aneurysm (FinnGen)	leave-one-out analyses

117	ebi-a-GCST90017074	Thoracic Aortic Aneurysm (FinnGen)	funnel plot
118	ebi-a-GCST90017074	Thoracic Aortic Aneurysm (FinnGen)	MR effect size
119	ebi-a-GCST90017074	Thoracic Aortic Aneurysm (FinnGen)	scatter plot
120	ebi-a-GCST90017093	Thoracic Aortic Aneurysm (FinnGen)	leave-one-out analyses
121	ebi-a-GCST90017093	Thoracic Aortic Aneurysm (FinnGen)	funnel plot
122	ebi-a-GCST90017093	Thoracic Aortic Aneurysm (FinnGen)	MR effect size
123	ebi-a-GCST90017093	Thoracic Aortic Aneurysm (FinnGen)	scatter plot
124	ebi-a-GCST90017109	Thoracic Aortic Aneurysm (FinnGen)	leave-one-out analyses
125	ebi-a-GCST90017109	Thoracic Aortic Aneurysm (FinnGen)	funnel plot
126	ebi-a-GCST90017109	Thoracic Aortic Aneurysm (FinnGen)	MR effect size
127	ebi-a-GCST90017109	Thoracic Aortic Aneurysm (FinnGen)	scatter plot
128	ebi-a-GCST90017115	Thoracic Aortic Aneurysm (FinnGen)	leave-one-out analyses
129	ebi-a-GCST90017115	Thoracic Aortic Aneurysm (FinnGen)	funnel plot
130	ebi-a-GCST90017115	Thoracic Aortic Aneurysm (FinnGen)	MR effect size
131	ebi-a-GCST90017115	Thoracic Aortic Aneurysm (FinnGen)	scatter plot
132	ebi-a-GCST90027661	Thoracic Aortic Aneurysm (FinnGen)	leave-one-out analyses
133	ebi-a-GCST90027661	Thoracic Aortic Aneurysm (FinnGen)	funnel plot
134	ebi-a-GCST90027661	Thoracic Aortic Aneurysm (FinnGen)	MR effect size
135	ebi-a-GCST90027661	Thoracic Aortic Aneurysm (FinnGen)	scatter plot
136	ebi-a-GCST90027669	Thoracic Aortic Aneurysm (FinnGen)	leave-one-out analyses
137	ebi-a-GCST90027669	Thoracic Aortic Aneurysm (FinnGen)	funnel plot
138	ebi-a-GCST90027669	Thoracic Aortic Aneurysm (FinnGen)	MR effect size
139	ebi-a-GCST90027669	Thoracic Aortic Aneurysm (FinnGen)	scatter plot
140	ebi-a-GCST90027674	Thoracic Aortic Aneurysm (FinnGen)	leave-one-out analyses
141	ebi-a-GCST90027674	Thoracic Aortic Aneurysm (FinnGen)	funnel plot
142	ebi-a-GCST90027674	Thoracic Aortic Aneurysm (FinnGen)	MR effect size
143	ebi-a-GCST90027674	Thoracic Aortic Aneurysm (FinnGen)	scatter plot
144	ebi-a-GCST90027687	Thoracic Aortic Aneurysm (FinnGen)	leave-one-out analyses
145	ebi-a-GCST90027687	Thoracic Aortic Aneurysm (FinnGen)	funnel plot
146	ebi-a-GCST90027687	Thoracic Aortic Aneurysm (FinnGen)	MR effect size
147	ebi-a-GCST90027687	Thoracic Aortic Aneurysm (FinnGen)	scatter plot
148	ebi-a-GCST90027702	Thoracic Aortic Aneurysm (FinnGen)	leave-one-out analyses
149	ebi-a-GCST90027702	Thoracic Aortic Aneurysm (FinnGen)	funnel plot
150	ebi-a-GCST90027702	Thoracic Aortic Aneurysm (FinnGen)	MR effect size
151	ebi-a-GCST90027702	Thoracic Aortic Aneurysm (FinnGen)	scatter plot
152	ebi-a-GCST90027730	Thoracic Aortic Aneurysm (FinnGen)	leave-one-out analyses
153	ebi-a-GCST90027730	Thoracic Aortic Aneurysm (FinnGen)	funnel plot
154	ebi-a-GCST90027730	Thoracic Aortic Aneurysm (FinnGen)	MR effect size
155	ebi-a-GCST90027730	Thoracic Aortic Aneurysm (FinnGen)	scatter plot

156	ebi-a-GCST90027769	Thoracic Aortic Aneurysm (FinnGen)	leave-one-out analyses
157	ebi-a-GCST90027769	Thoracic Aortic Aneurysm (FinnGen)	funnel plot
158	ebi-a-GCST90027769	Thoracic Aortic Aneurysm (FinnGen)	MR effect size
159	ebi-a-GCST90027769	Thoracic Aortic Aneurysm (FinnGen)	scatter plot
160	ebi-a-GCST90027802	Thoracic Aortic Aneurysm (FinnGen)	leave-one-out analyses
161	ebi-a-GCST90027802	Thoracic Aortic Aneurysm (FinnGen)	funnel plot
162	ebi-a-GCST90027802	Thoracic Aortic Aneurysm (FinnGen)	MR effect size
163	ebi-a-GCST90027802	Thoracic Aortic Aneurysm (FinnGen)	scatter plot
164	ebi-a-GCST90027813	Thoracic Aortic Aneurysm (FinnGen)	leave-one-out analyses
165	ebi-a-GCST90027813	Thoracic Aortic Aneurysm (FinnGen)	funnel plot
166	ebi-a-GCST90027813	Thoracic Aortic Aneurysm (FinnGen)	MR effect size
167	ebi-a-GCST90027813	Thoracic Aortic Aneurysm (FinnGen)	scatter plot
168	ebi-a-GCST90027835	Thoracic Aortic Aneurysm (FinnGen)	leave-one-out analyses
169	ebi-a-GCST90027835	Thoracic Aortic Aneurysm (FinnGen)	funnel plot
170	ebi-a-GCST90027835	Thoracic Aortic Aneurysm (FinnGen)	MR effect size
171	ebi-a-GCST90027835	Thoracic Aortic Aneurysm (FinnGen)	scatter plot
172	ebi-a-GCST90027840	Thoracic Aortic Aneurysm (FinnGen)	leave-one-out analyses
173	ebi-a-GCST90027840	Thoracic Aortic Aneurysm (FinnGen)	funnel plot
174	ebi-a-GCST90027840	Thoracic Aortic Aneurysm (FinnGen)	MR effect size
175	ebi-a-GCST90027840	Thoracic Aortic Aneurysm (FinnGen)	scatter plot
176	ebi-a-GCST90027851	Thoracic Aortic Aneurysm (FinnGen)	leave-one-out analyses
177	ebi-a-GCST90027851	Thoracic Aortic Aneurysm (FinnGen)	funnel plot
178	ebi-a-GCST90027851	Thoracic Aortic Aneurysm (FinnGen)	MR effect size
179	ebi-a-GCST90027851	Thoracic Aortic Aneurysm (FinnGen)	scatter plot
180	ebi-a-GCST90016908	Aortic Dissection (FinnGen)	leave-one-out analyses
181	ebi-a-GCST90016908	Aortic Dissection (FinnGen)	funnel plot
182	ebi-a-GCST90016908	Aortic Dissection (FinnGen)	MR effect size
183	ebi-a-GCST90016908	Aortic Dissection (FinnGen)	scatter plot
184	ebi-a-GCST90016918	Aortic Dissection (FinnGen)	leave-one-out analyses
185	ebi-a-GCST90016918	Aortic Dissection (FinnGen)	funnel plot
186	ebi-a-GCST90016918	Aortic Dissection (FinnGen)	MR effect size
187	ebi-a-GCST90016918	Aortic Dissection (FinnGen)	scatter plot
188	ebi-a-GCST90016981	Aortic Dissection (FinnGen)	leave-one-out analyses
189	ebi-a-GCST90016981	Aortic Dissection (FinnGen)	funnel plot
190	ebi-a-GCST90016981	Aortic Dissection (FinnGen)	MR effect size
191	ebi-a-GCST90016981	Aortic Dissection (FinnGen)	scatter plot
192	ebi-a-GCST90017065	Aortic Dissection (FinnGen)	leave-one-out analyses
193	ebi-a-GCST90017065	Aortic Dissection (FinnGen)	funnel plot
194	ebi-a-GCST90017065	Aortic Dissection (FinnGen)	MR effect size

195	ebi-a-GCST90017065	Aortic Dissection (FinnGen)	scatter plot
196	ebi-a-GCST90017109	Aortic Dissection (FinnGen)	leave-one-out analyses
197	ebi-a-GCST90017109	Aortic Dissection (FinnGen)	funnel plot
198	ebi-a-GCST90017109	Aortic Dissection (FinnGen)	MR effect size
199	ebi-a-GCST90017109	Aortic Dissection (FinnGen)	scatter plot
200	ebi-a-GCST90017115	Aortic Dissection (FinnGen)	leave-one-out analyses
201	ebi-a-GCST90017115	Aortic Dissection (FinnGen)	funnel plot
202	ebi-a-GCST90017115	Aortic Dissection (FinnGen)	MR effect size
203	ebi-a-GCST90017115	Aortic Dissection (FinnGen)	scatter plot
204	ebi-a-GCST90027675	Aortic Dissection (FinnGen)	leave-one-out analyses
205	ebi-a-GCST90027675	Aortic Dissection (FinnGen)	funnel plot
206	ebi-a-GCST90027675	Aortic Dissection (FinnGen)	MR effect size
207	ebi-a-GCST90027675	Aortic Dissection (FinnGen)	scatter plot
208	ebi-a-GCST90027697	Aortic Dissection (FinnGen)	leave-one-out analyses
209	ebi-a-GCST90027697	Aortic Dissection (FinnGen)	funnel plot
210	ebi-a-GCST90027697	Aortic Dissection (FinnGen)	MR effect size
211	ebi-a-GCST90027697	Aortic Dissection (FinnGen)	scatter plot
212	ebi-a-GCST90027714	Aortic Dissection (FinnGen)	leave-one-out analyses
213	ebi-a-GCST90027714	Aortic Dissection (FinnGen)	funnel plot
214	ebi-a-GCST90027714	Aortic Dissection (FinnGen)	MR effect size
215	ebi-a-GCST90027714	Aortic Dissection (FinnGen)	scatter plot
216	ebi-a-GCST90027780	Aortic Dissection (FinnGen)	leave-one-out analyses
217	ebi-a-GCST90027780	Aortic Dissection (FinnGen)	funnel plot
218	ebi-a-GCST90027780	Aortic Dissection (FinnGen)	MR effect size
219	ebi-a-GCST90027780	Aortic Dissection (FinnGen)	scatter plot
220	ebi-a-GCST90027787	Aortic Dissection (FinnGen)	leave-one-out analyses
221	ebi-a-GCST90027787	Aortic Dissection (FinnGen)	funnel plot
222	ebi-a-GCST90027787	Aortic Dissection (FinnGen)	MR effect size
223	ebi-a-GCST90027787	Aortic Dissection (FinnGen)	scatter plot
224	ebi-a-GCST90027843	Aortic Dissection (FinnGen)	leave-one-out analyses
225	ebi-a-GCST90027843	Aortic Dissection (FinnGen)	funnel plot
226	ebi-a-GCST90027843	Aortic Dissection (FinnGen)	MR effect size
227	ebi-a-GCST90027843	Aortic Dissection (FinnGen)	scatter plot
228	ebi-a-GCST90027853	Aortic Dissection (FinnGen)	leave-one-out analyses
229	ebi-a-GCST90027853	Aortic Dissection (FinnGen)	funnel plot
230	ebi-a-GCST90027853	Aortic Dissection (FinnGen)	MR effect size
231	ebi-a-GCST90027853	Aortic Dissection (FinnGen)	scatter plot
232	Abdominal Aortic Aneurysm (FinnGen)	ebi-a-GCST90016909	leave-one-out analyses
233	Abdominal Aortic Aneurysm (FinnGen)	ebi-a-GCST90016909	funnel plot

234	Abdominal Aortic Aneurysm (FinnGen)	ebi-a-GCST90016909	MR effect size
235	Abdominal Aortic Aneurysm (FinnGen)	ebi-a-GCST90016909	scatter plot
236	Abdominal Aortic Aneurysm (FinnGen)	ebi-a-GCST90016949	leave-one-out analyses
237	Abdominal Aortic Aneurysm (FinnGen)	ebi-a-GCST90016949	funnel plot
238	Abdominal Aortic Aneurysm (FinnGen)	ebi-a-GCST90016949	MR effect size
239	Abdominal Aortic Aneurysm (FinnGen)	ebi-a-GCST90016949	scatter plot
240	Abdominal Aortic Aneurysm (FinnGen)	ebi-a-GCST90016972	leave-one-out analyses
241	Abdominal Aortic Aneurysm (FinnGen)	ebi-a-GCST90016972	funnel plot
242	Abdominal Aortic Aneurysm (FinnGen)	ebi-a-GCST90016972	MR effect size
243	Abdominal Aortic Aneurysm (FinnGen)	ebi-a-GCST90016972	scatter plot
244	Abdominal Aortic Aneurysm (FinnGen)	ebi-a-GCST90016987	leave-one-out analyses
245	Abdominal Aortic Aneurysm (FinnGen)	ebi-a-GCST90016987	funnel plot
246	Abdominal Aortic Aneurysm (FinnGen)	ebi-a-GCST90016987	MR effect size
247	Abdominal Aortic Aneurysm (FinnGen)	ebi-a-GCST90016987	scatter plot
248	Abdominal Aortic Aneurysm (FinnGen)	ebi-a-GCST90017021	leave-one-out analyses
249	Abdominal Aortic Aneurysm (FinnGen)	ebi-a-GCST90017021	funnel plot
250	Abdominal Aortic Aneurysm (FinnGen)	ebi-a-GCST90017021	MR effect size
251	Abdominal Aortic Aneurysm (FinnGen)	ebi-a-GCST90017021	scatter plot
252	Abdominal Aortic Aneurysm (FinnGen)	ebi-a-GCST90017059	leave-one-out analyses
253	Abdominal Aortic Aneurysm (FinnGen)	ebi-a-GCST90017059	funnel plot
254	Abdominal Aortic Aneurysm (FinnGen)	ebi-a-GCST90017059	MR effect size
255	Abdominal Aortic Aneurysm (FinnGen)	ebi-a-GCST90017059	scatter plot
256	Abdominal Aortic Aneurysm (FinnGen)	ebi-a-GCST90017073	leave-one-out analyses
257	Abdominal Aortic Aneurysm (FinnGen)	ebi-a-GCST90017073	funnel plot
258	Abdominal Aortic Aneurysm (FinnGen)	ebi-a-GCST90017073	MR effect size
259	Abdominal Aortic Aneurysm (FinnGen)	ebi-a-GCST90017073	scatter plot
260	Abdominal Aortic Aneurysm (FinnGen)	ebi-a-GCST90017106	leave-one-out analyses
261	Abdominal Aortic Aneurysm (FinnGen)	ebi-a-GCST90017106	funnel plot
262	Abdominal Aortic Aneurysm (FinnGen)	ebi-a-GCST90017106	MR effect size
263	Abdominal Aortic Aneurysm (FinnGen)	ebi-a-GCST90017106	scatter plot
264	Abdominal Aortic Aneurysm (FinnGen)	ebi-a-GCST90027679	leave-one-out analyses
265	Abdominal Aortic Aneurysm (FinnGen)	ebi-a-GCST90027679	funnel plot
266	Abdominal Aortic Aneurysm (FinnGen)	ebi-a-GCST90027679	MR effect size
267	Abdominal Aortic Aneurysm (FinnGen)	ebi-a-GCST90027679	scatter plot
268	Abdominal Aortic Aneurysm (FinnGen)	ebi-a-GCST90027685	leave-one-out analyses
269	Abdominal Aortic Aneurysm (FinnGen)	ebi-a-GCST90027685	funnel plot
270	Abdominal Aortic Aneurysm (FinnGen)	ebi-a-GCST90027685	MR effect size
271	Abdominal Aortic Aneurysm (FinnGen)	ebi-a-GCST90027685	scatter plot
272	Abdominal Aortic Aneurysm (FinnGen)	ebi-a-GCST90027689	leave-one-out analyses

273	Abdominal Aortic Aneurysm (FinnGen)	ebi-a-GCST90027689	funnel plot
274	Abdominal Aortic Aneurysm (FinnGen)	ebi-a-GCST90027689	MR effect size
275	Abdominal Aortic Aneurysm (FinnGen)	ebi-a-GCST90027689	scatter plot
276	Abdominal Aortic Aneurysm (FinnGen)	ebi-a-GCST90027713	leave-one-out analyses
277	Abdominal Aortic Aneurysm (FinnGen)	ebi-a-GCST90027713	funnel plot
278	Abdominal Aortic Aneurysm (FinnGen)	ebi-a-GCST90027713	MR effect size
279	Abdominal Aortic Aneurysm (FinnGen)	ebi-a-GCST90027713	scatter plot
280	Abdominal Aortic Aneurysm (FinnGen)	ebi-a-GCST90027731	leave-one-out analyses
281	Abdominal Aortic Aneurysm (FinnGen)	ebi-a-GCST90027731	funnel plot
282	Abdominal Aortic Aneurysm (FinnGen)	ebi-a-GCST90027731	MR effect size
283	Abdominal Aortic Aneurysm (FinnGen)	ebi-a-GCST90027731	scatter plot
284	Abdominal Aortic Aneurysm (FinnGen)	ebi-a-GCST90027746	leave-one-out analyses
285	Abdominal Aortic Aneurysm (FinnGen)	ebi-a-GCST90027746	funnel plot
286	Abdominal Aortic Aneurysm (FinnGen)	ebi-a-GCST90027746	MR effect size
287	Abdominal Aortic Aneurysm (FinnGen)	ebi-a-GCST90027746	scatter plot
288	Abdominal Aortic Aneurysm (FinnGen)	ebi-a-GCST90027758	leave-one-out analyses
289	Abdominal Aortic Aneurysm (FinnGen)	ebi-a-GCST90027758	funnel plot
290	Abdominal Aortic Aneurysm (FinnGen)	ebi-a-GCST90027758	MR effect size
291	Abdominal Aortic Aneurysm (FinnGen)	ebi-a-GCST90027758	scatter plot
292	Abdominal Aortic Aneurysm (FinnGen)	ebi-a-GCST90027815	leave-one-out analyses
293	Abdominal Aortic Aneurysm (FinnGen)	ebi-a-GCST90027815	funnel plot
294	Abdominal Aortic Aneurysm (FinnGen)	ebi-a-GCST90027815	MR effect size
295	Abdominal Aortic Aneurysm (FinnGen)	ebi-a-GCST90027815	scatter plot
296	Abdominal Aortic Aneurysm (FinnGen)	ebi-a-GCST90027818	leave-one-out analyses
297	Abdominal Aortic Aneurysm (FinnGen)	ebi-a-GCST90027818	funnel plot
298	Abdominal Aortic Aneurysm (FinnGen)	ebi-a-GCST90027818	MR effect size
299	Abdominal Aortic Aneurysm (FinnGen)	ebi-a-GCST90027818	scatter plot
300	Thoracic Aortic Aneurysm (FinnGen)	ebi-a-GCST90016988	leave-one-out analyses
301	Thoracic Aortic Aneurysm (FinnGen)	ebi-a-GCST90017048	funnel plot
302	Thoracic Aortic Aneurysm (FinnGen)	ebi-a-GCST90017044	MR effect size
303	Thoracic Aortic Aneurysm (FinnGen)	ebi-a-GCST90017067	scatter plot
304	Thoracic Aortic Aneurysm (FinnGen)	ebi-a-GCST90027857	leave-one-out analyses
305	Thoracic Aortic Aneurysm (FinnGen)	ebi-a-GCST90027852	funnel plot
306	Thoracic Aortic Aneurysm (FinnGen)	ebi-a-GCST90027731	MR effect size
307	Thoracic Aortic Aneurysm (FinnGen)	ebi-a-GCST90027712	scatter plot
308	Thoracic Aortic Aneurysm (FinnGen)	ebi-a-GCST90016988	leave-one-out analyses
309	Thoracic Aortic Aneurysm (FinnGen)	ebi-a-GCST90017048	funnel plot
310	Thoracic Aortic Aneurysm (FinnGen)	ebi-a-GCST90017044	MR effect size
311	Thoracic Aortic Aneurysm (FinnGen)	ebi-a-GCST90017067	scatter plot

312	Thoracic Aortic Aneurysm (FinnGen)	ebi-a-GCST90027857	leave-one-out analyses
313	Thoracic Aortic Aneurysm (FinnGen)	ebi-a-GCST90027852	funnel plot
314	Thoracic Aortic Aneurysm (FinnGen)	ebi-a-GCST90027731	MR effect size
315	Thoracic Aortic Aneurysm (FinnGen)	ebi-a-GCST90027712	scatter plot
316	Thoracic Aortic Aneurysm (FinnGen)	ebi-a-GCST90016988	leave-one-out analyses
317	Thoracic Aortic Aneurysm (FinnGen)	ebi-a-GCST90017048	funnel plot
318	Thoracic Aortic Aneurysm (FinnGen)	ebi-a-GCST90017044	MR effect size
319	Thoracic Aortic Aneurysm (FinnGen)	ebi-a-GCST90017067	scatter plot
320	Thoracic Aortic Aneurysm (FinnGen)	ebi-a-GCST90027857	leave-one-out analyses
321	Thoracic Aortic Aneurysm (FinnGen)	ebi-a-GCST90027852	funnel plot
322	Thoracic Aortic Aneurysm (FinnGen)	ebi-a-GCST90027731	MR effect size
323	Thoracic Aortic Aneurysm (FinnGen)	ebi-a-GCST90027712	scatter plot
324	Thoracic Aortic Aneurysm (FinnGen)	ebi-a-GCST90016988	leave-one-out analyses
325	Thoracic Aortic Aneurysm (FinnGen)	ebi-a-GCST90017048	funnel plot
326	Thoracic Aortic Aneurysm (FinnGen)	ebi-a-GCST90017044	MR effect size
327	Thoracic Aortic Aneurysm (FinnGen)	ebi-a-GCST90017067	scatter plot
328	Thoracic Aortic Aneurysm (FinnGen)	ebi-a-GCST90027857	leave-one-out analyses
329	Thoracic Aortic Aneurysm (FinnGen)	ebi-a-GCST90027852	funnel plot
330	Thoracic Aortic Aneurysm (FinnGen)	ebi-a-GCST90027731	MR effect size
331	Thoracic Aortic Aneurysm (FinnGen)	ebi-a-GCST90027712	scatter plot
332	Aortic Dissection (FinnGen)	ebi-a-GCST90017011	leave-one-out analyses
333	Aortic Dissection (FinnGen)	ebi-a-GCST90016965	funnel plot
334	Aortic Dissection (FinnGen)	ebi-a-GCST90016960	MR effect size
335	Aortic Dissection (FinnGen)	ebi-a-GCST90017056	scatter plot
336	Aortic Dissection (FinnGen)	ebi-a-GCST90016978	leave-one-out analyses
337	Aortic Dissection (FinnGen)	ebi-a-GCST90017068	funnel plot
338	Aortic Dissection (FinnGen)	ebi-a-GCST90017018	MR effect size
339	Aortic Dissection (FinnGen)	ebi-a-GCST90017023	scatter plot
340	Aortic Dissection (FinnGen)	ebi-a-GCST90016948	leave-one-out analyses
341	Aortic Dissection (FinnGen)	ebi-a-GCST90017061	funnel plot
342	Aortic Dissection (FinnGen)	ebi-a-GCST90017059	MR effect size
343	Aortic Dissection (FinnGen)	ebi-a-GCST90016951	scatter plot
344	Aortic Dissection (FinnGen)	ebi-a-GCST90016939	leave-one-out analyses
345	Aortic Dissection (FinnGen)	ebi-a-GCST90016967	funnel plot
346	Aortic Dissection (FinnGen)	ebi-a-GCST90017053	MR effect size
347	Aortic Dissection (FinnGen)	ebi-a-GCST90017036	scatter plot
348	Aortic Dissection (FinnGen)	ebi-a-GCST90016930	leave-one-out analyses
349	Aortic Dissection (FinnGen)	ebi-a-GCST90027783	funnel plot
350	Aortic Dissection (FinnGen)	ebi-a-GCST90027773	MR effect size

351	Aortic Dissection (FinnGen)	ebi-a-GCST90027762	scatter plot
352	Aortic Dissection (FinnGen)	ebi-a-GCST90027759	leave-one-out analyses
353	Aortic Dissection (FinnGen)	ebi-a-GCST90027757	funnel plot
354	Aortic Dissection (FinnGen)	ebi-a-GCST90027737	MR effect size
355	Aortic Dissection (FinnGen)	ebi-a-GCST90027694	scatter plot
356	Aortic Dissection (FinnGen)	ebi-a-GCST90027690	leave-one-out analyses
357	Aortic Dissection (FinnGen)	ebi-a-GCST90027665	funnel plot
358	Aortic Dissection (FinnGen)	ebi-a-GCST90027663	MR effect size
359	Aortic Dissection (FinnGen)	ebi-a-GCST90017011	scatter plot
360	Aortic Dissection (FinnGen)	ebi-a-GCST90016965	leave-one-out analyses
361	Aortic Dissection (FinnGen)	ebi-a-GCST90016960	funnel plot
362	Aortic Dissection (FinnGen)	ebi-a-GCST90017056	MR effect size
363	Aortic Dissection (FinnGen)	ebi-a-GCST90016978	scatter plot
364	Aortic Dissection (FinnGen)	ebi-a-GCST90017068	leave-one-out analyses
365	Aortic Dissection (FinnGen)	ebi-a-GCST90017018	funnel plot
366	Aortic Dissection (FinnGen)	ebi-a-GCST90017023	MR effect size
367	Aortic Dissection (FinnGen)	ebi-a-GCST90016948	scatter plot
368	Aortic Dissection (FinnGen)	ebi-a-GCST90017061	leave-one-out analyses
369	Aortic Dissection (FinnGen)	ebi-a-GCST90017059	funnel plot
370	Aortic Dissection (FinnGen)	ebi-a-GCST90016951	MR effect size
371	Aortic Dissection (FinnGen)	ebi-a-GCST90016939	scatter plot
372	Aortic Dissection (FinnGen)	ebi-a-GCST90016967	leave-one-out analyses
373	Aortic Dissection (FinnGen)	ebi-a-GCST90017053	funnel plot
374	Aortic Dissection (FinnGen)	ebi-a-GCST90017036	MR effect size
375	Aortic Dissection (FinnGen)	ebi-a-GCST90016930	scatter plot
376	Aortic Dissection (FinnGen)	ebi-a-GCST90027783	leave-one-out analyses
377	Aortic Dissection (FinnGen)	ebi-a-GCST90027773	funnel plot
378	Aortic Dissection (FinnGen)	ebi-a-GCST90027762	MR effect size
379	Aortic Dissection (FinnGen)	ebi-a-GCST90027759	scatter plot
380	Aortic Dissection (FinnGen)	ebi-a-GCST90027757	leave-one-out analyses
381	Aortic Dissection (FinnGen)	ebi-a-GCST90027737	funnel plot
382	Aortic Dissection (FinnGen)	ebi-a-GCST90027694	MR effect size
383	Aortic Dissection (FinnGen)	ebi-a-GCST90027690	scatter plot
384	Aortic Dissection (FinnGen)	ebi-a-GCST90027665	leave-one-out analyses
385	Aortic Dissection (FinnGen)	ebi-a-GCST90027663	funnel plot
386	Aortic Dissection (FinnGen)	ebi-a-GCST90017011	MR effect size
387	Aortic Dissection (FinnGen)	ebi-a-GCST90016965	scatter plot
388	Aortic Dissection (FinnGen)	ebi-a-GCST90016960	leave-one-out analyses
389	Aortic Dissection (FinnGen)	ebi-a-GCST90017056	funnel plot

390	Aortic Dissection (FinnGen)	ebi-a-GCST90016978	MR effect size
391	Aortic Dissection (FinnGen)	ebi-a-GCST90017068	scatter plot
392	Aortic Dissection (FinnGen)	ebi-a-GCST90017018	leave-one-out analyses
393	Aortic Dissection (FinnGen)	ebi-a-GCST90017023	funnel plot
394	Aortic Dissection (FinnGen)	ebi-a-GCST90016948	MR effect size
395	Aortic Dissection (FinnGen)	ebi-a-GCST90017061	scatter plot
396	Aortic Dissection (FinnGen)	ebi-a-GCST90017059	leave-one-out analyses
397	Aortic Dissection (FinnGen)	ebi-a-GCST90016951	funnel plot
398	Aortic Dissection (FinnGen)	ebi-a-GCST90016939	MR effect size
399	Aortic Dissection (FinnGen)	ebi-a-GCST90016967	scatter plot
400	Aortic Dissection (FinnGen)	ebi-a-GCST90017053	leave-one-out analyses
401	Aortic Dissection (FinnGen)	ebi-a-GCST90017036	funnel plot
402	Aortic Dissection (FinnGen)	ebi-a-GCST90016930	MR effect size
403	Aortic Dissection (FinnGen)	ebi-a-GCST90027783	scatter plot
404	Aortic Dissection (FinnGen)	ebi-a-GCST90027773	leave-one-out analyses
405	Aortic Dissection (FinnGen)	ebi-a-GCST90027762	funnel plot
406	Aortic Dissection (FinnGen)	ebi-a-GCST90027759	MR effect size
407	Aortic Dissection (FinnGen)	ebi-a-GCST90027757	scatter plot
408	Aortic Dissection (FinnGen)	ebi-a-GCST90027737	leave-one-out analyses
409	Aortic Dissection (FinnGen)	ebi-a-GCST90027694	funnel plot
410	Aortic Dissection (FinnGen)	ebi-a-GCST90027690	MR effect size
411	Aortic Dissection (FinnGen)	ebi-a-GCST90027665	scatter plot
412	Aortic Dissection (FinnGen)	ebi-a-GCST90027663	leave-one-out analyses
413	Aortic Dissection (FinnGen)	ebi-a-GCST90017011	funnel plot
414	Aortic Dissection (FinnGen)	ebi-a-GCST90016965	MR effect size
415	Aortic Dissection (FinnGen)	ebi-a-GCST90016960	scatter plot
416	Aortic Dissection (FinnGen)	ebi-a-GCST90017056	leave-one-out analyses
417	Aortic Dissection (FinnGen)	ebi-a-GCST90016978	funnel plot
418	Aortic Dissection (FinnGen)	ebi-a-GCST90017068	MR effect size
419	Aortic Dissection (FinnGen)	ebi-a-GCST90017018	scatter plot
420	Aortic Dissection (FinnGen)	ebi-a-GCST90017023	leave-one-out analyses
421	Aortic Dissection (FinnGen)	ebi-a-GCST90016948	funnel plot
422	Aortic Dissection (FinnGen)	ebi-a-GCST90017061	MR effect size
423	Aortic Dissection (FinnGen)	ebi-a-GCST90017059	scatter plot
424	Aortic Dissection (FinnGen)	ebi-a-GCST90016951	leave-one-out analyses
425	Aortic Dissection (FinnGen)	ebi-a-GCST90016939	funnel plot
426	Aortic Dissection (FinnGen)	ebi-a-GCST90016967	MR effect size
427	Aortic Dissection (FinnGen)	ebi-a-GCST90017053	scatter plot
428	Aortic Dissection (FinnGen)	ebi-a-GCST90017036	leave-one-out analyses

429	Aortic Dissection (FinnGen)	ebi-a-GCST90016930	funnel plot
430	Aortic Dissection (FinnGen)	ebi-a-GCST90027783	MR effect size
431	Aortic Dissection (FinnGen)	ebi-a-GCST90027773	scatter plot
432	Aortic Dissection (FinnGen)	ebi-a-GCST90027762	leave-one-out analyses
433	Aortic Dissection (FinnGen)	ebi-a-GCST90027759	funnel plot
434	Aortic Dissection (FinnGen)	ebi-a-GCST90027757	MR effect size
435	Aortic Dissection (FinnGen)	ebi-a-GCST90027737	scatter plot
436	Aortic Dissection (FinnGen)	ebi-a-GCST90027694	leave-one-out analyses
437	Aortic Dissection (FinnGen)	ebi-a-GCST90027690	funnel plot
438	Aortic Dissection (FinnGen)	ebi-a-GCST90027665	MR effect size
439	Aortic Dissection (FinnGen)	ebi-a-GCST90027663	scatter plot

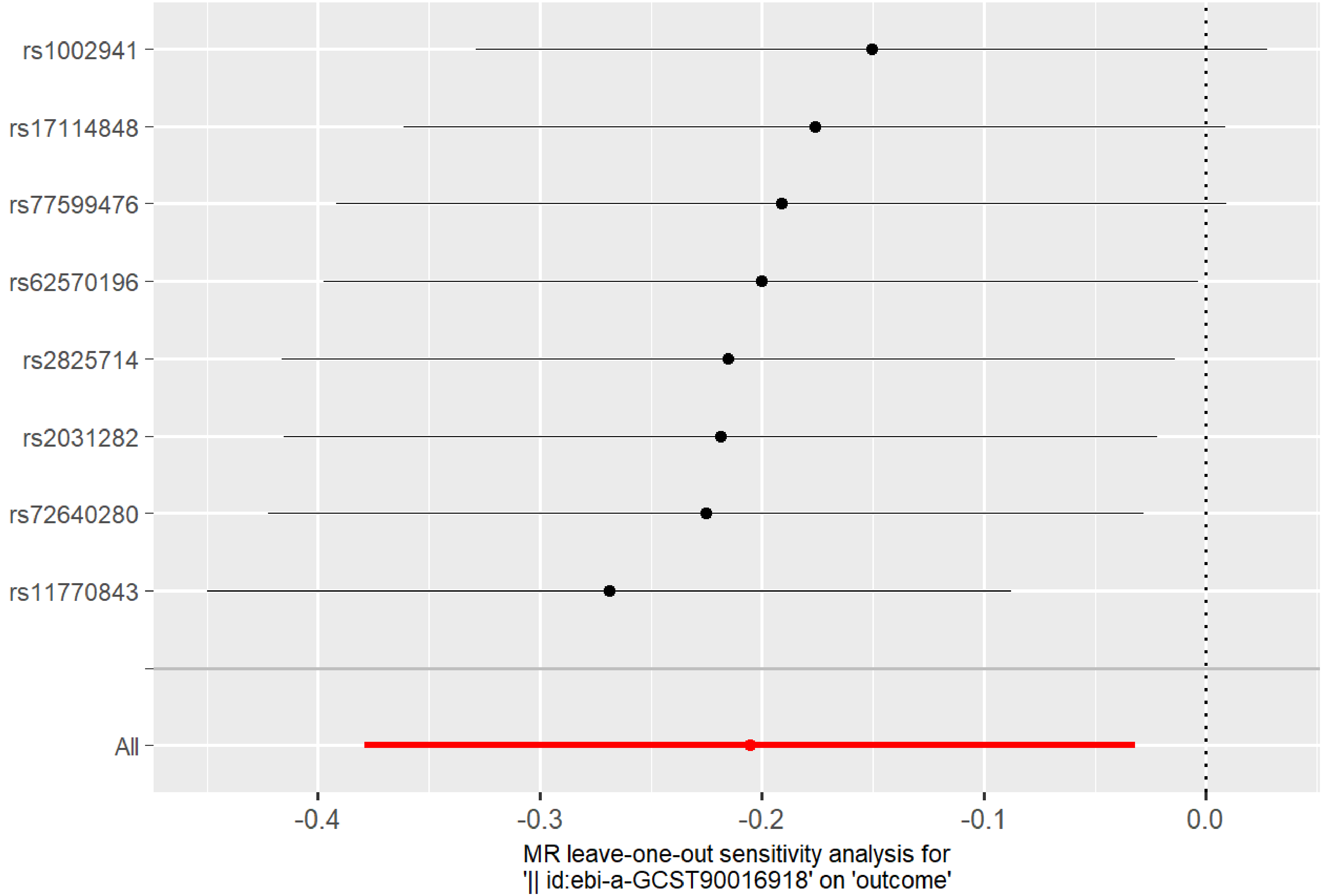
rs1002941
rs17114848
rs77599476
rs62570196
rs2825714
rs2031282
rs72640280
rs11770843
All
-0.4
-0.3
-0.2
-0.1
0.0
MR leave-one-out sensitivity analysis for
'|| id:ebi-a-GCST90016918' on 'outcome'

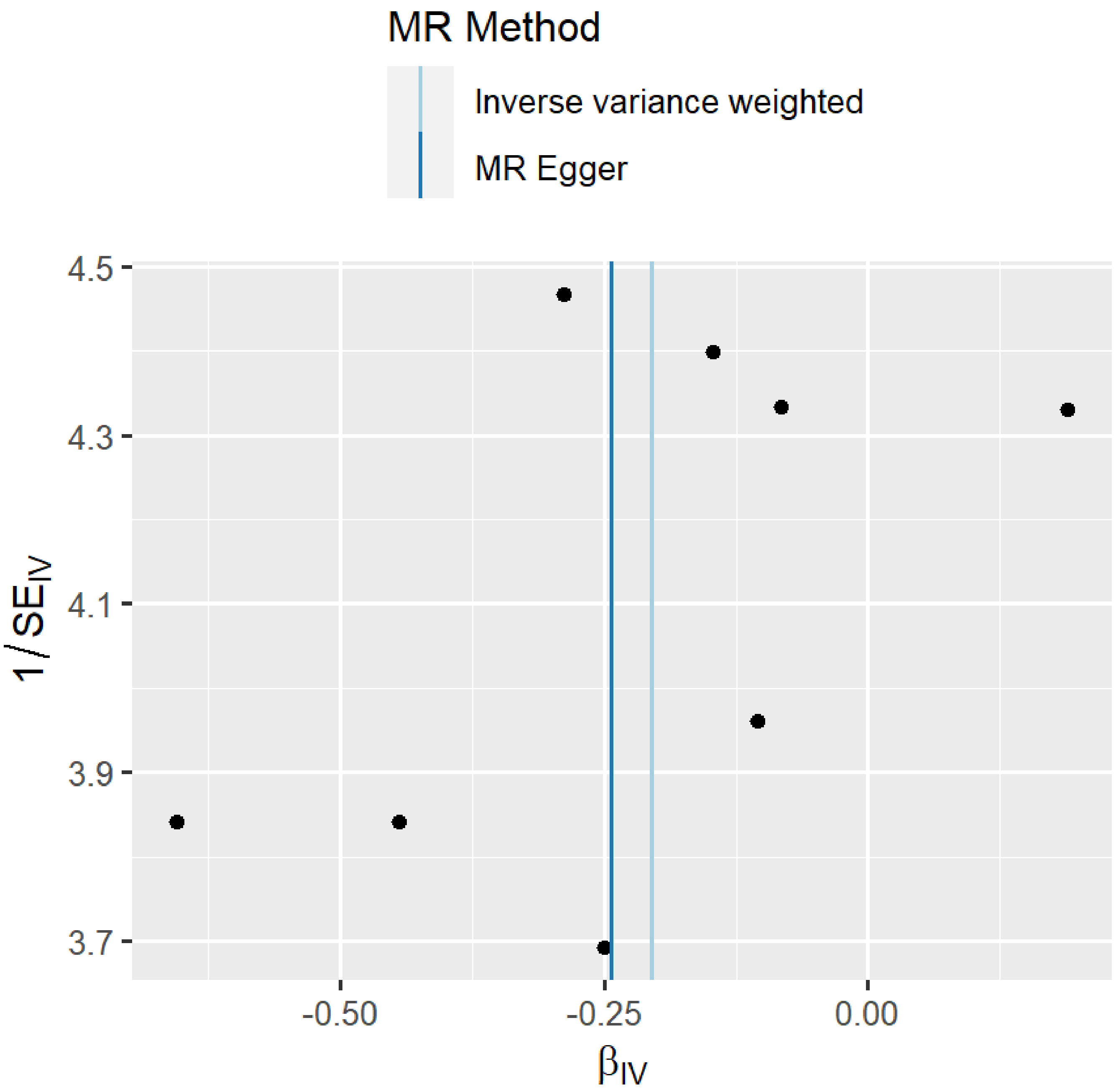

MR Method
Inverse variance weighted
MR Egger
4.5
4.3
4.1
3.9
3.7
1/SE_IV
-0.50
-0.25
0.00
β_IV

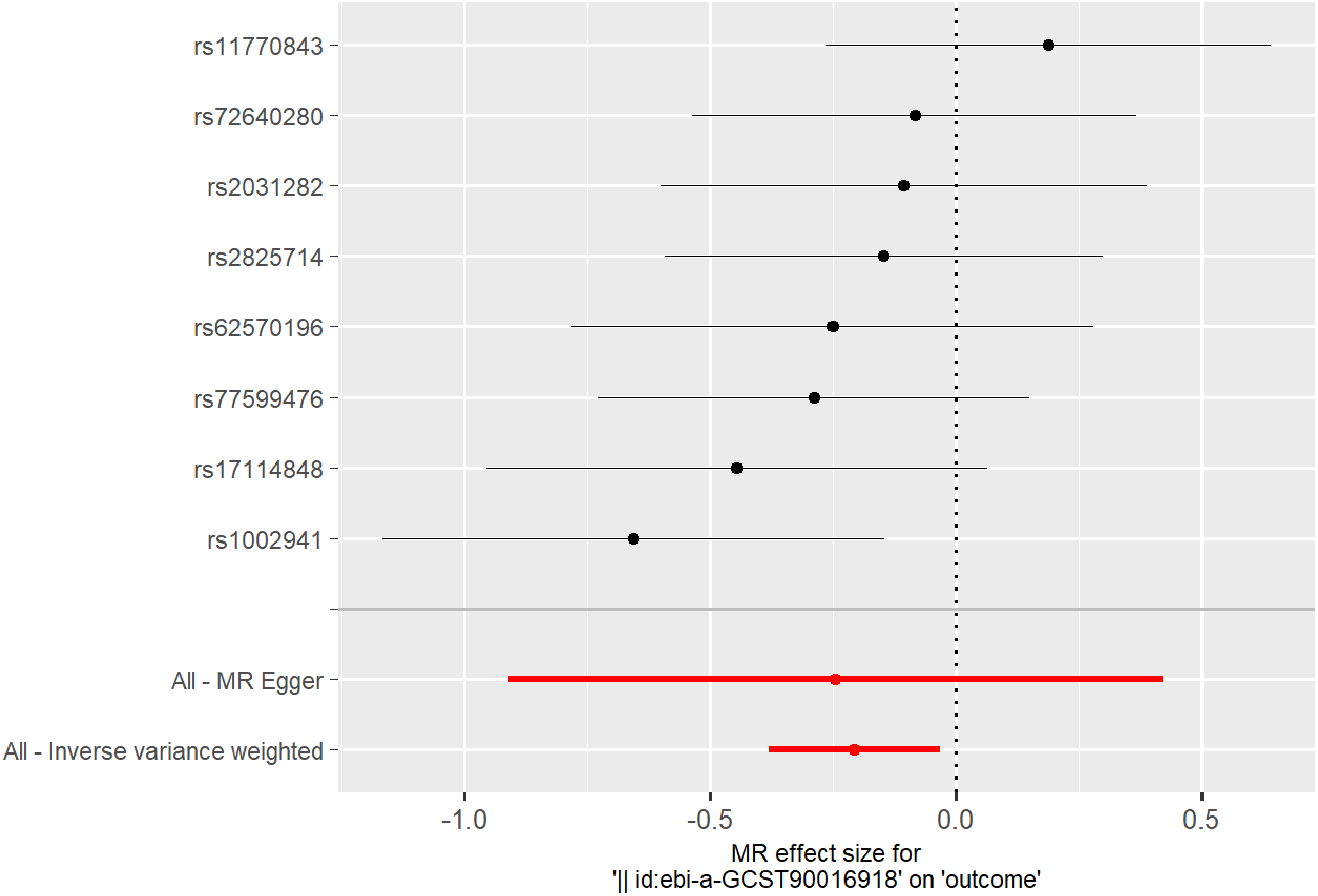

rs11770843
rs72640280
rs2031282
rs2825714
rs62570196
rs77599476
rs17114848
rs1002941
All - MR Egger
All - Inverse variance weighted
-1.0
-0.5
0.0
0.5
MR effect size for
'|| id:ebi-a-GCST90016918' on 'outcome'

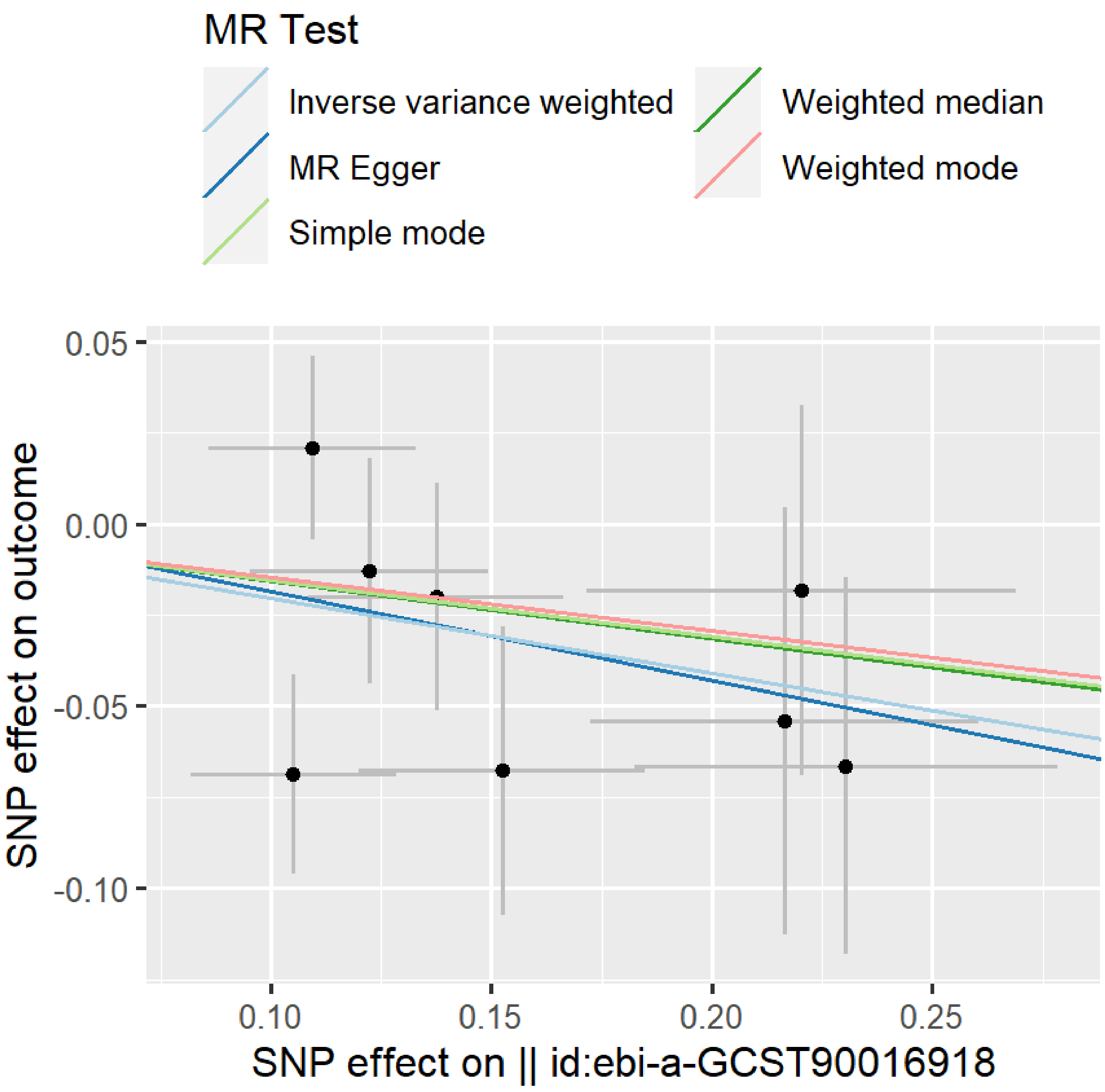

MR Test
Inverse variance weighted
MR Egger
Simple mode
Weighted median
Weighted mode
0.05
0.00
-0.05
-0.10
SNP effect on outcome
0.10
0.15
0.20
0.25
SNP effect on || id:ebi-a-GCST90016918

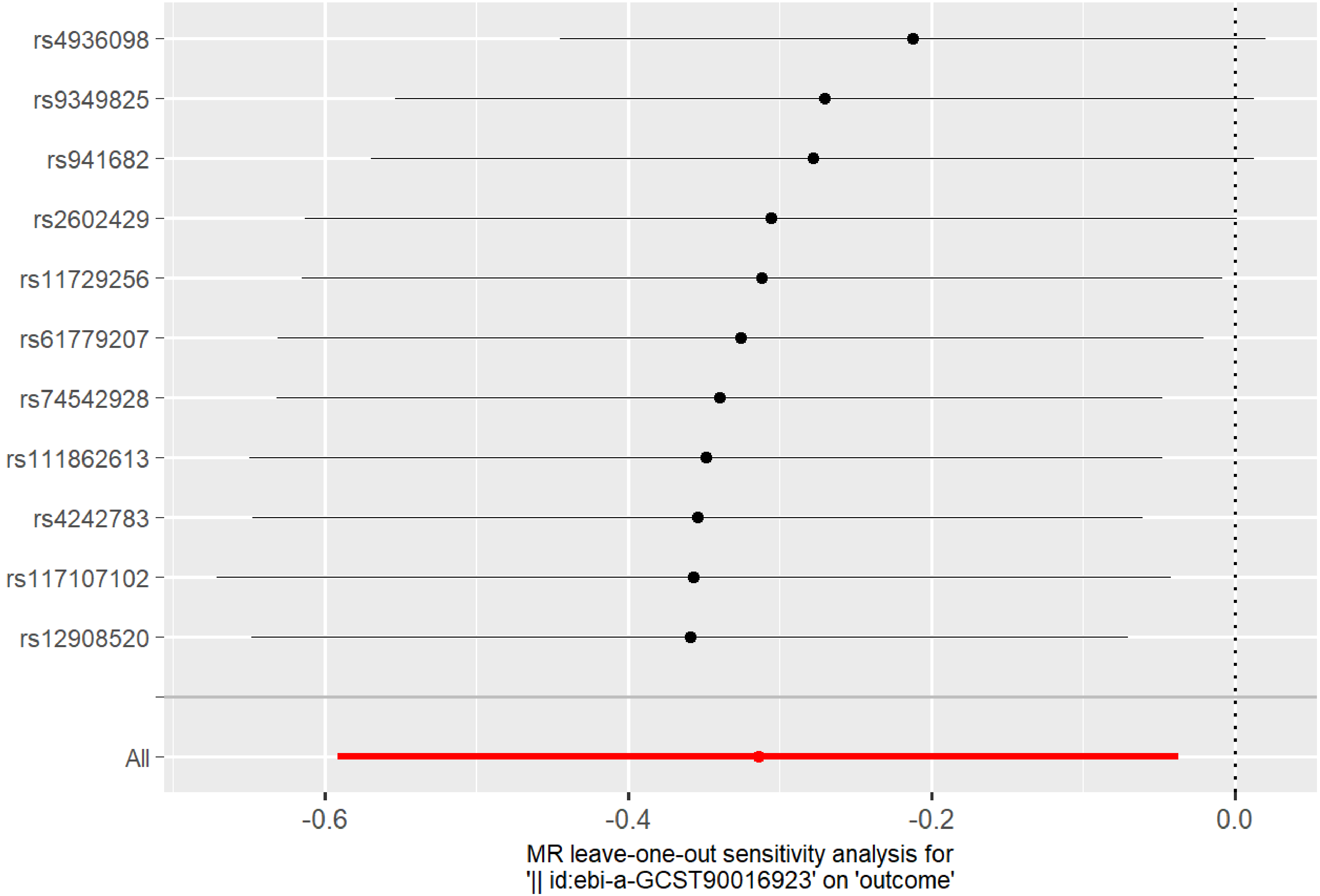
rs4936098
rs9349825
rs941682
rs2602429
rs11729256
rs61779207
rs74542928
rs111862613
rs4242783
rs117107102
rs12908520
All
-0.6
-0.4
-0.2
0.0
MR leave-one-out sensitivity analysis for
'|| id:ebi-a-GCST90016923' on 'outcome'

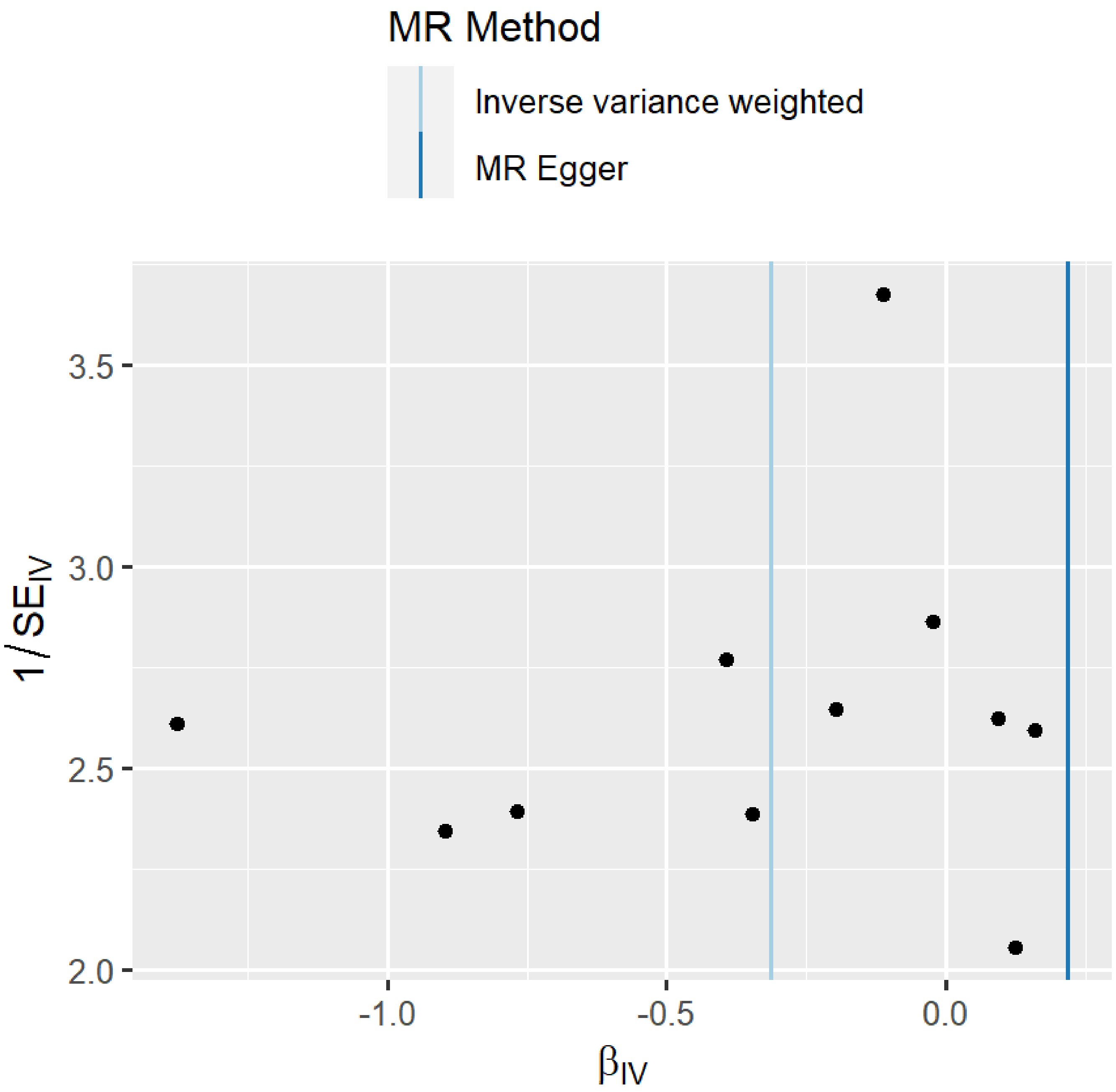

MR Method
Inverse variance weighted
MR Egger
3.5
3.0
2.5
2.0
1/SE$_{IV}$
-1.0
-0.5
0.0
β$_{IV}$

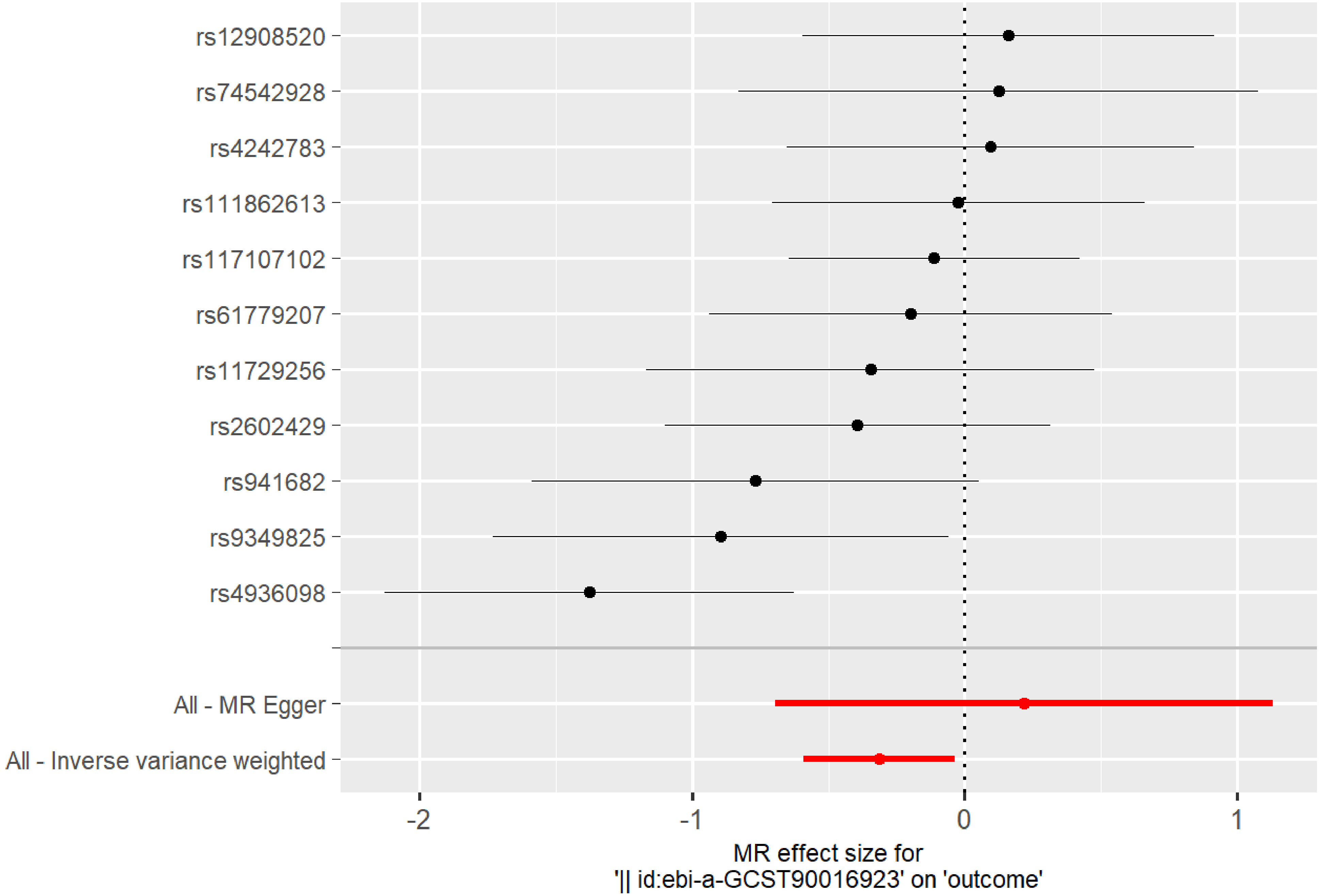
rs12908520
rs74542928
rs4242783
rs111862613
rs117107102
rs61779207
rs11729256
rs2602429
rs941682
rs9349825
rs4936098
All - MR Egger
All - Inverse variance weighted
-2
-1
0
1
MR effect size for
'|| id:ebi-a-GCST90016923' on 'outcome'

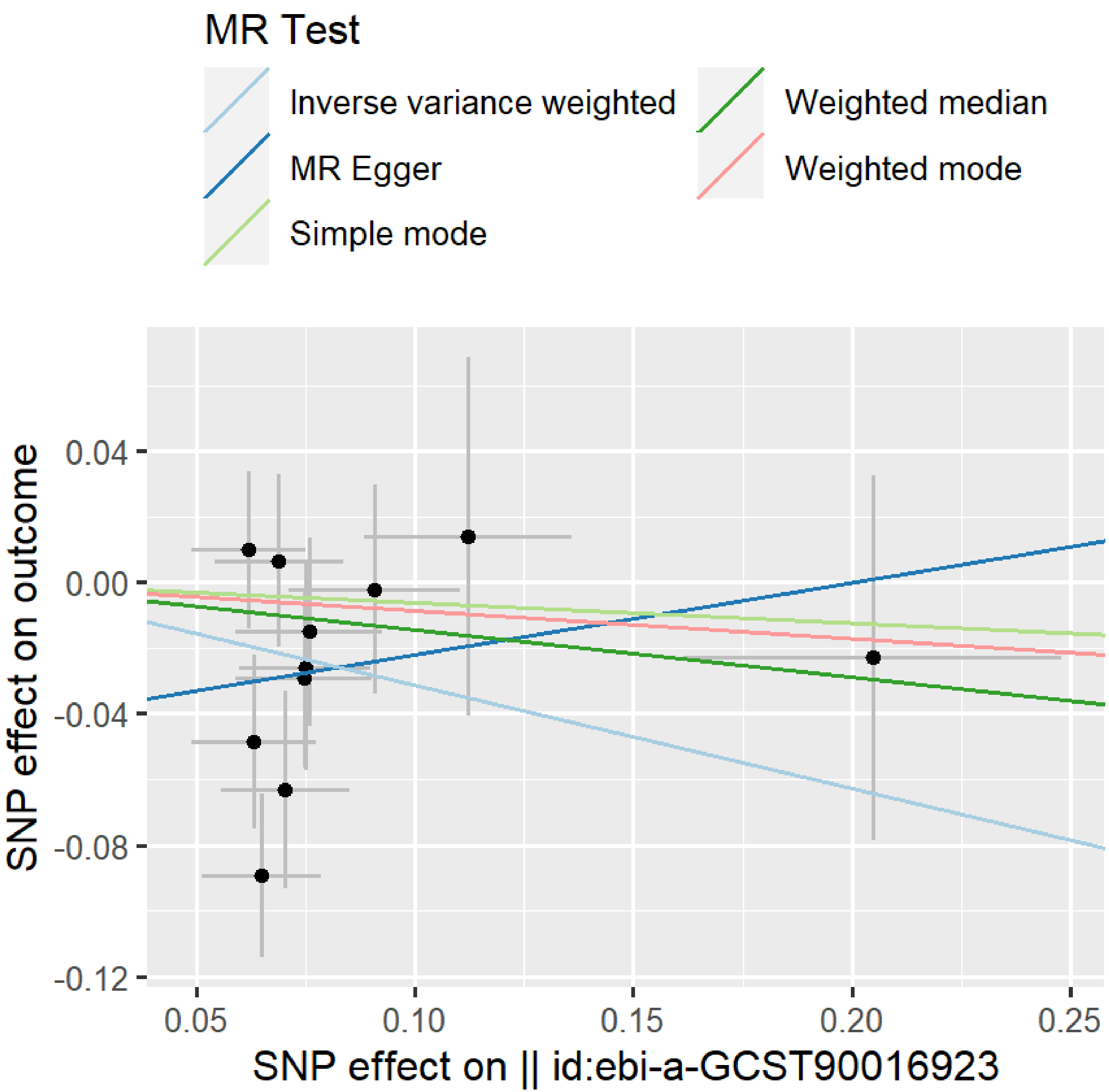
MR Test
Inverse variance weighted
MR Egger
Simple mode
Weighted median
Weighted mode
0.04
0.00
-0.04
-0.08
-0.12
SNP effect on outcome
0.05
0.10
0.15
0.20
0.25
SNP effect on || id:ebi-a-GCST90016923

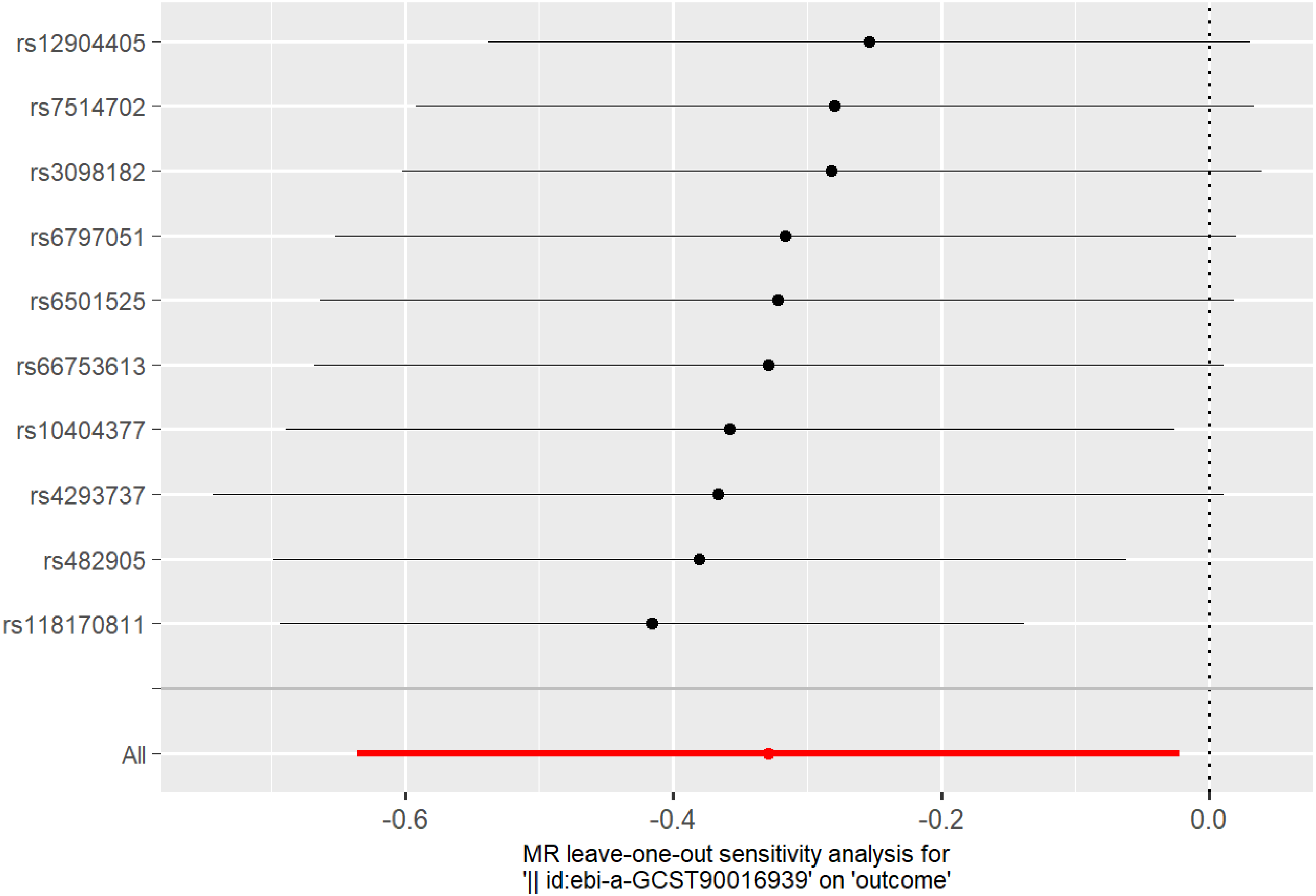
rs12904405
rs7514702
rs3098182
rs6797051
rs6501525
rs66753613
rs10404377
rs4293737
rs482905
rs118170811
All
-0.6
-0.4
-0.2
0.0
MR leave-one-out sensitivity analysis for
'|| id:ebi-a-GCST90016939' on 'outcome'

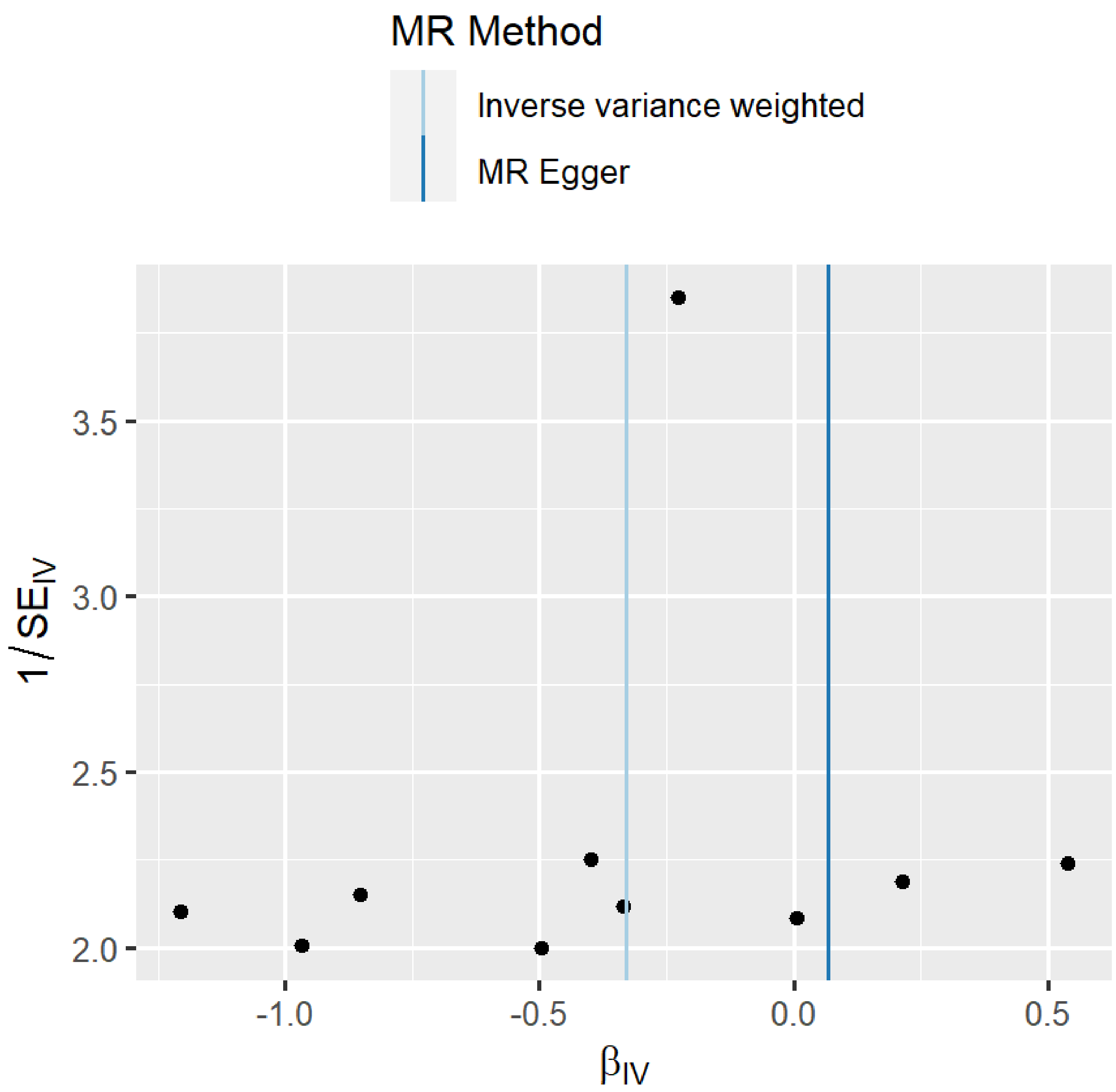
MR Method
Inverse variance weighted
MR Egger
3.5
3.0
2.5
2.0
$1/SE_{IV}$
-1.0
-0.5
0.0
0.5
β_{IV}

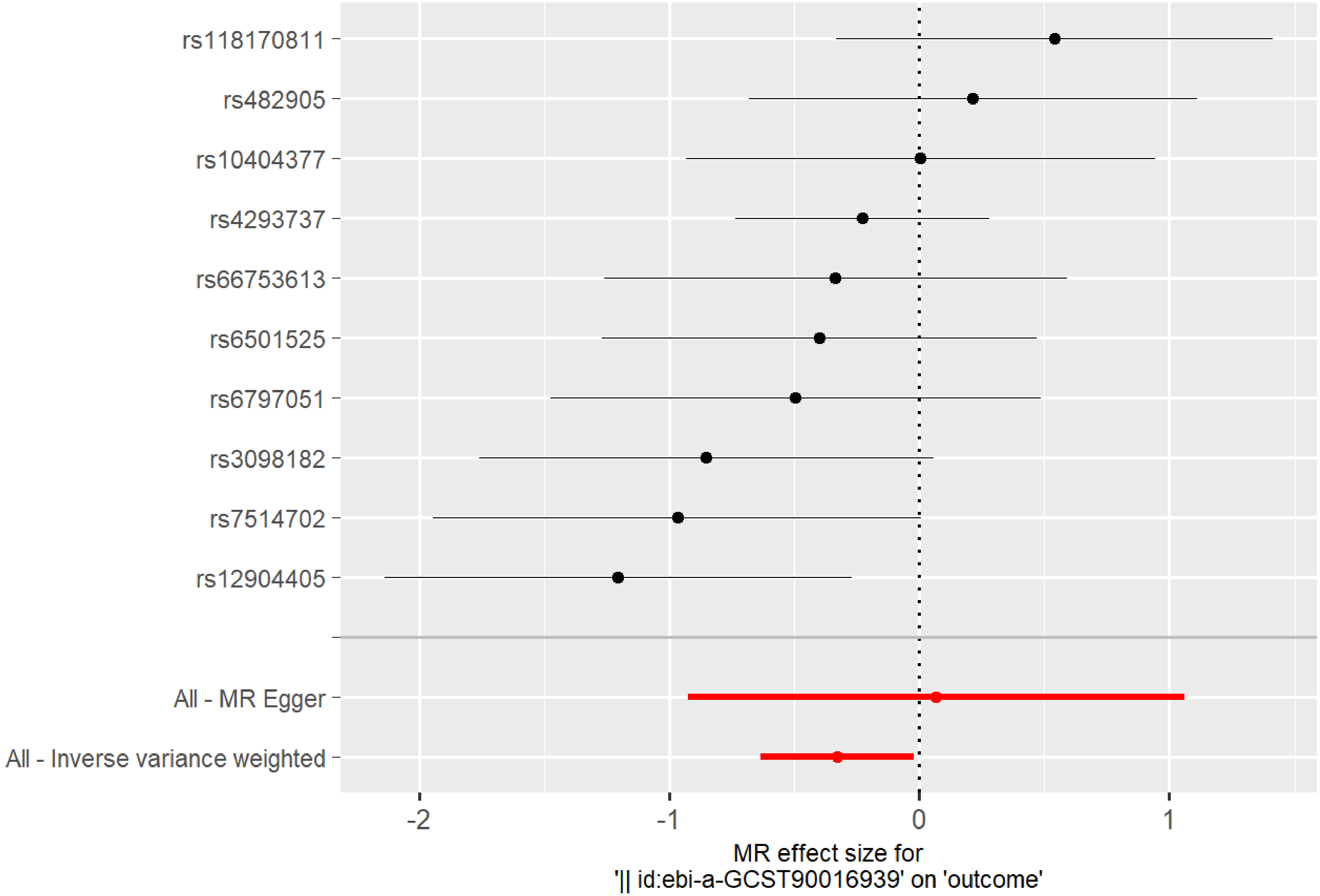
rs118170811
rs482905
rs10404377
rs4293737
rs66753613
rs6501525
rs6797051
rs3098182
rs7514702
rs12904405
All - MR Egger
All - Inverse variance weighted
-2
-1
0
1
MR effect size for
'|| id:ebi-a-GCST90016939' on 'outcome'

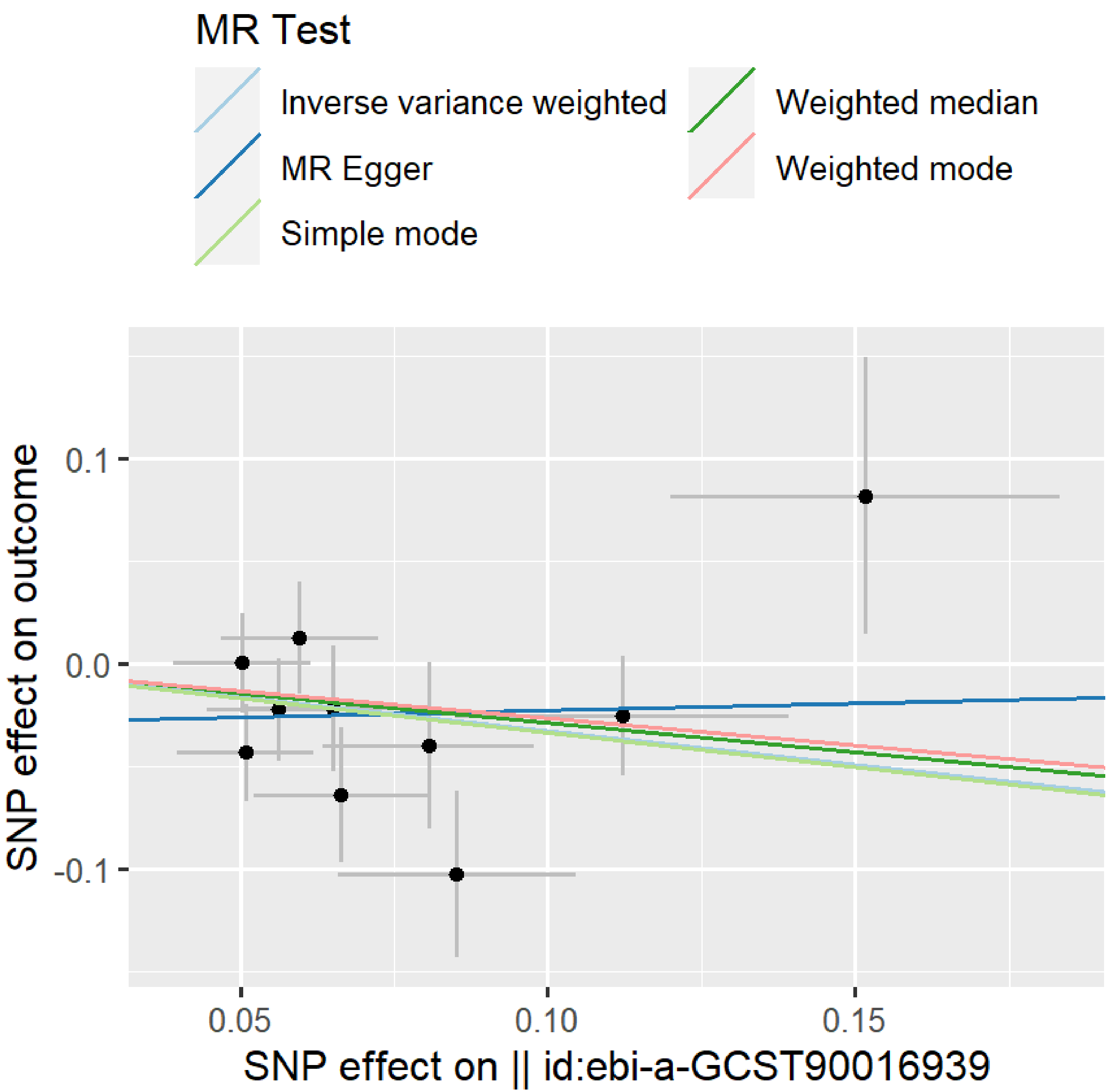
MR Test
Inverse variance weighted
MR Egger
Simple mode
Weighted median
Weighted mode
SNP effect on outcome
0.1
0.0
-0.1
0.05
0.10
0.15
SNP effect on || id:ebi-a-GCST90016939

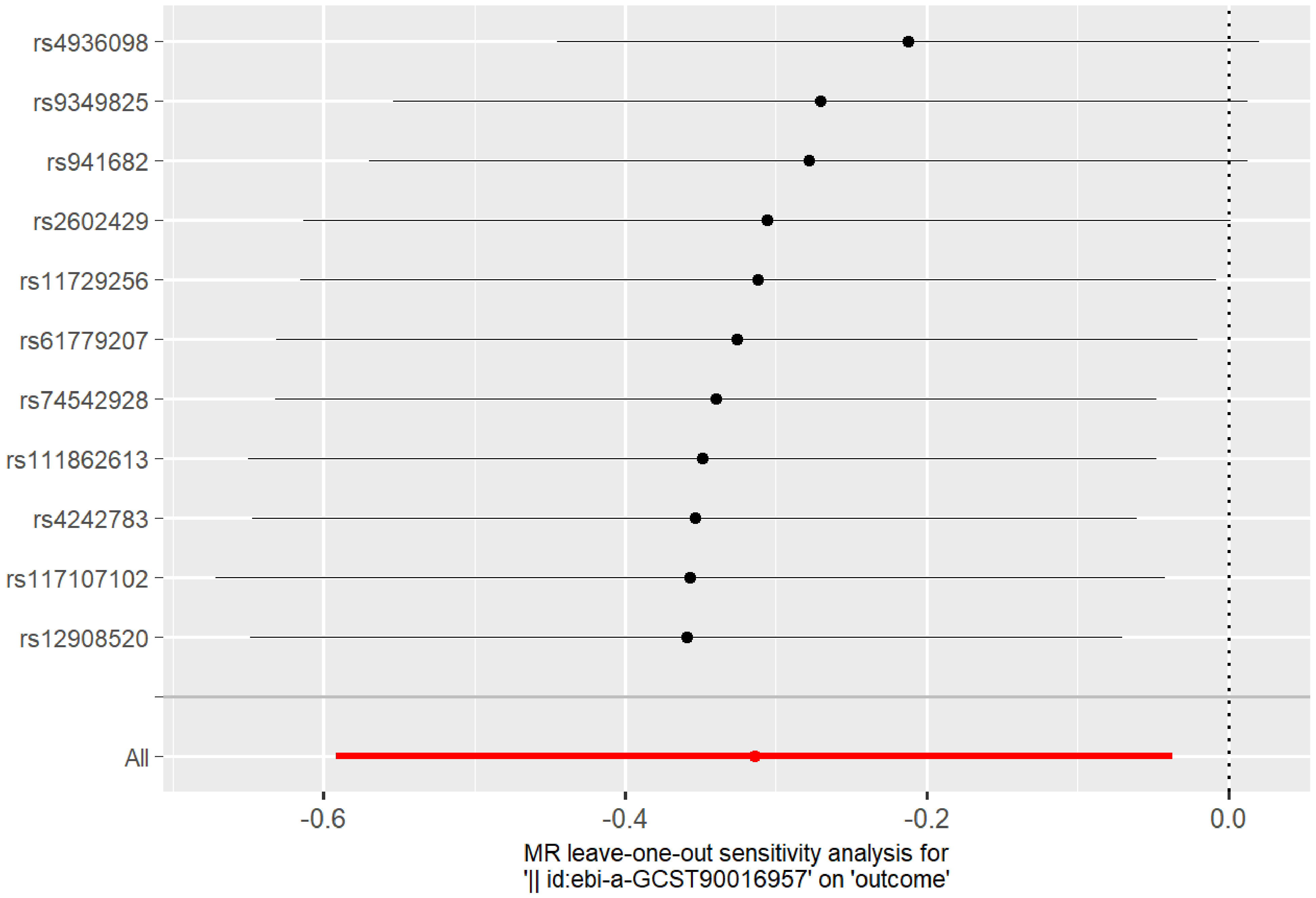
rs4936098
rs9349825
rs941682
rs2602429
rs11729256
rs61779207
rs74542928
rs111862613
rs4242783
rs117107102
rs12908520
All
-0.6
-0.4
-0.2
0.0
MR leave-one-out sensitivity analysis for
'|| id:ebi-a-GCST90016957' on 'outcome'

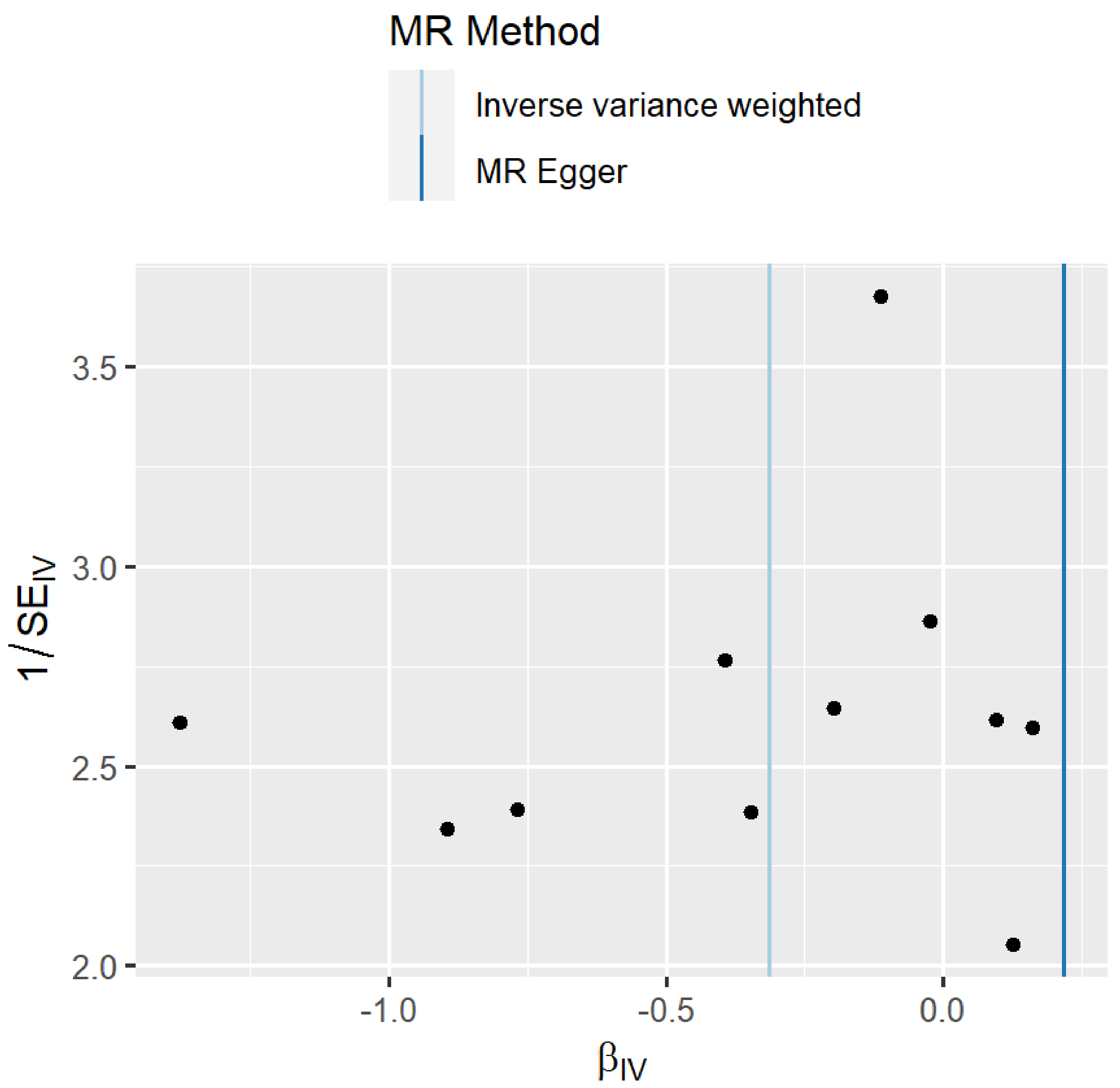
MR Method
Inverse variance weighted
MR Egger
3.5
3.0
2.5
2.0
$1/SE_{IV}$
-1.0
-0.5
0.0
β_{IV}

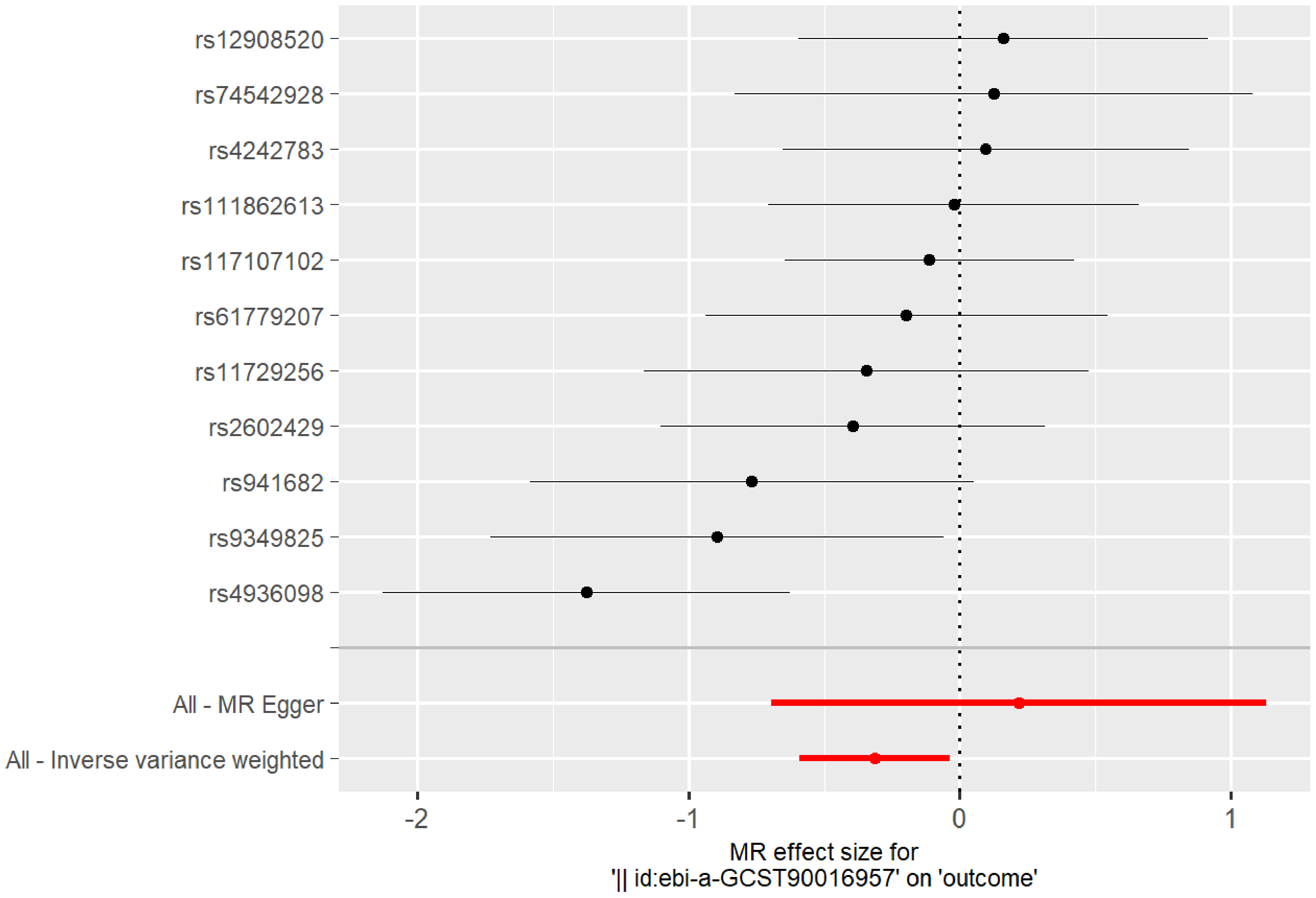
rs12908520
rs74542928
rs4242783
rs111862613
rs117107102
rs61779207
rs11729256
rs2602429
rs941682
rs9349825
rs4936098
All - MR Egger
All - Inverse variance weighted
-2
-1
0
1
MR effect size for
'|| id:ebi-a-GCST90016957' on 'outcome'

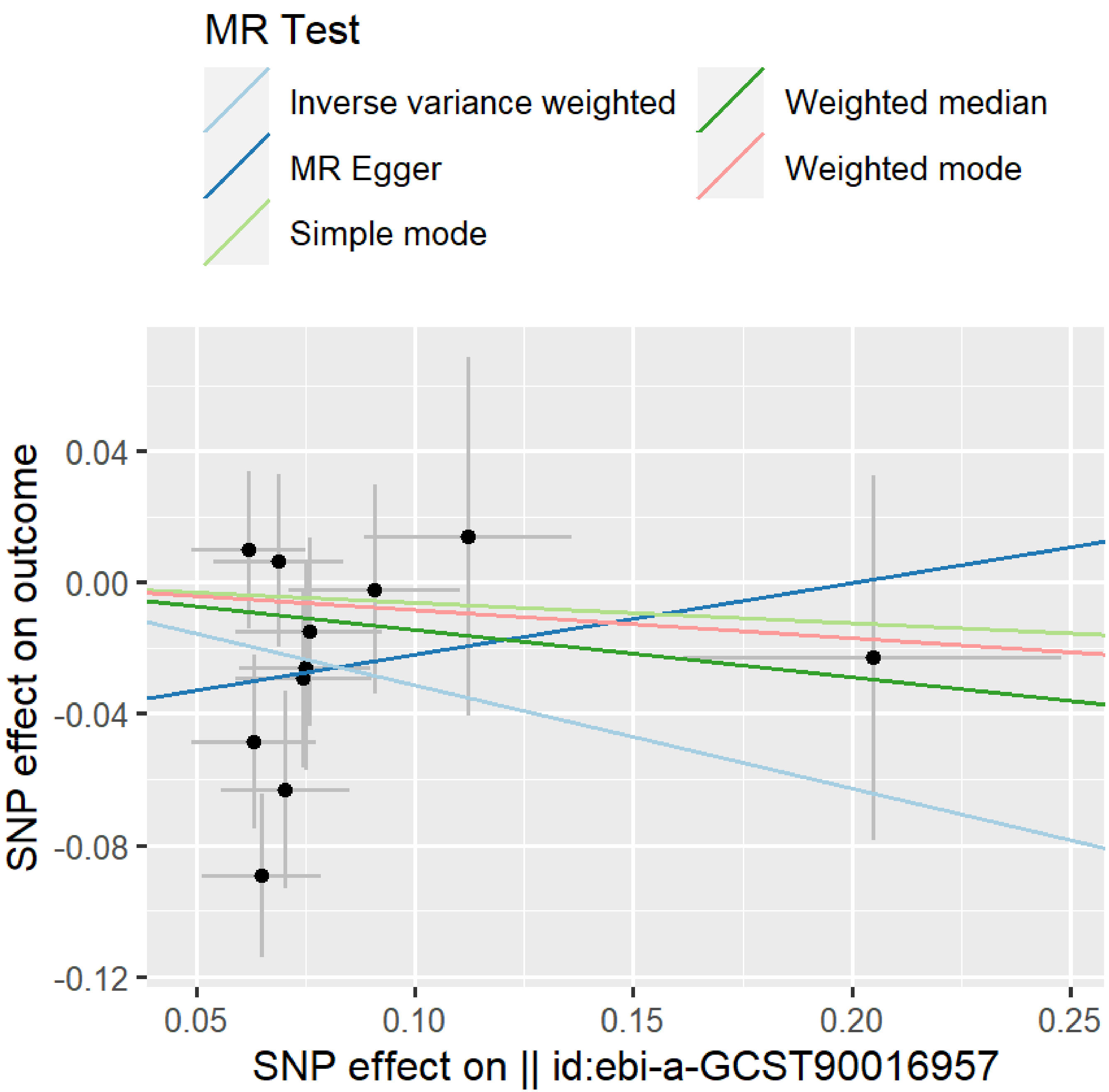
MR Test
Inverse variance weighted
MR Egger
Simple mode
Weighted median
Weighted mode
0.04
0.00
-0.04
-0.08
-0.12
SNP effect on outcome
0.05
0.10
0.15
0.20
0.25
SNP effect on || id:ebi-a-GCST90016957

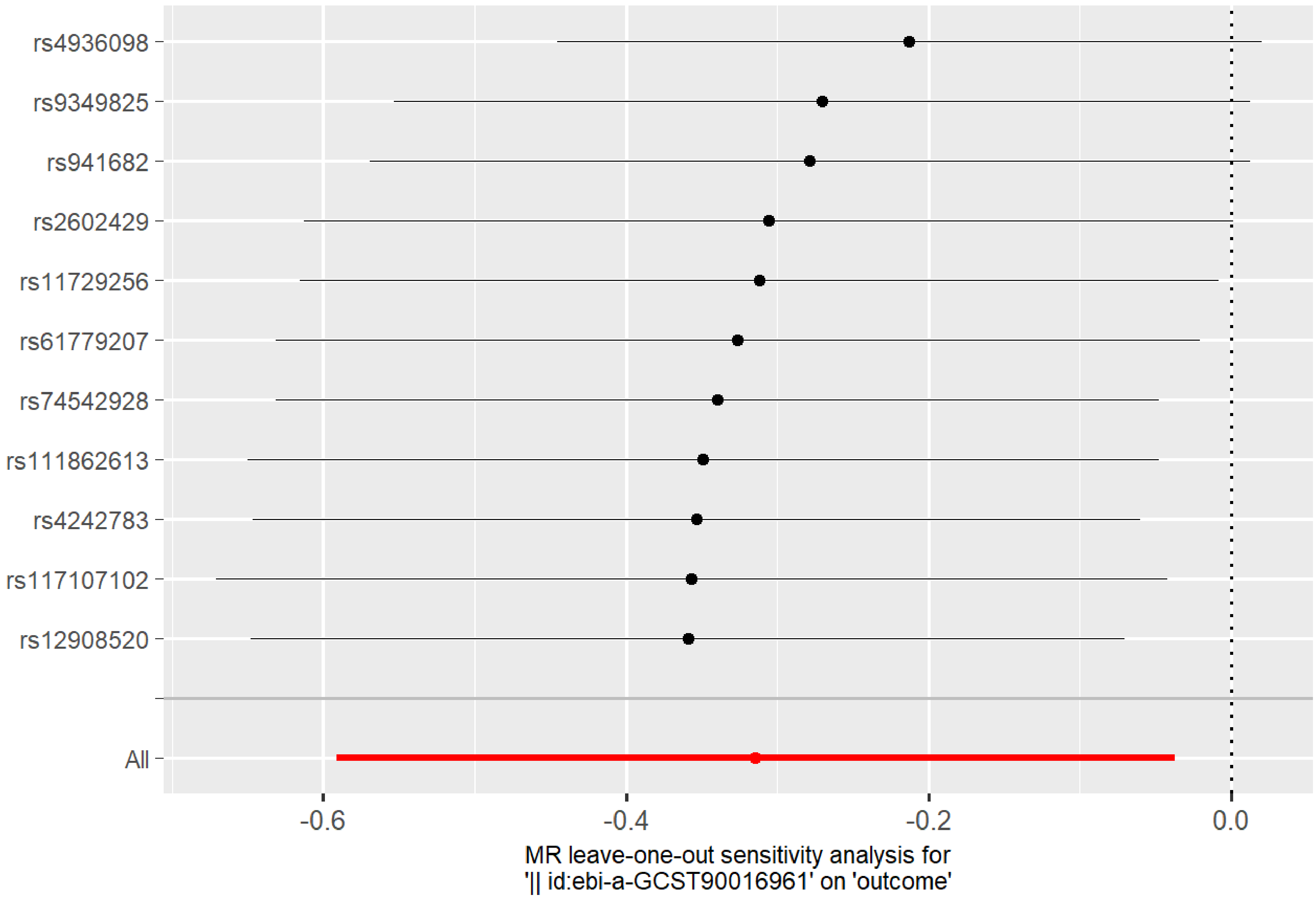
rs4936098
rs9349825
rs941682
rs2602429
rs11729256
rs61779207
rs74542928
rs111862613
rs4242783
rs117107102
rs12908520
All
-0.6
-0.4
-0.2
0.0
MR leave-one-out sensitivity analysis for
'|| id:ebi-a-GCST90016961' on 'outcome'

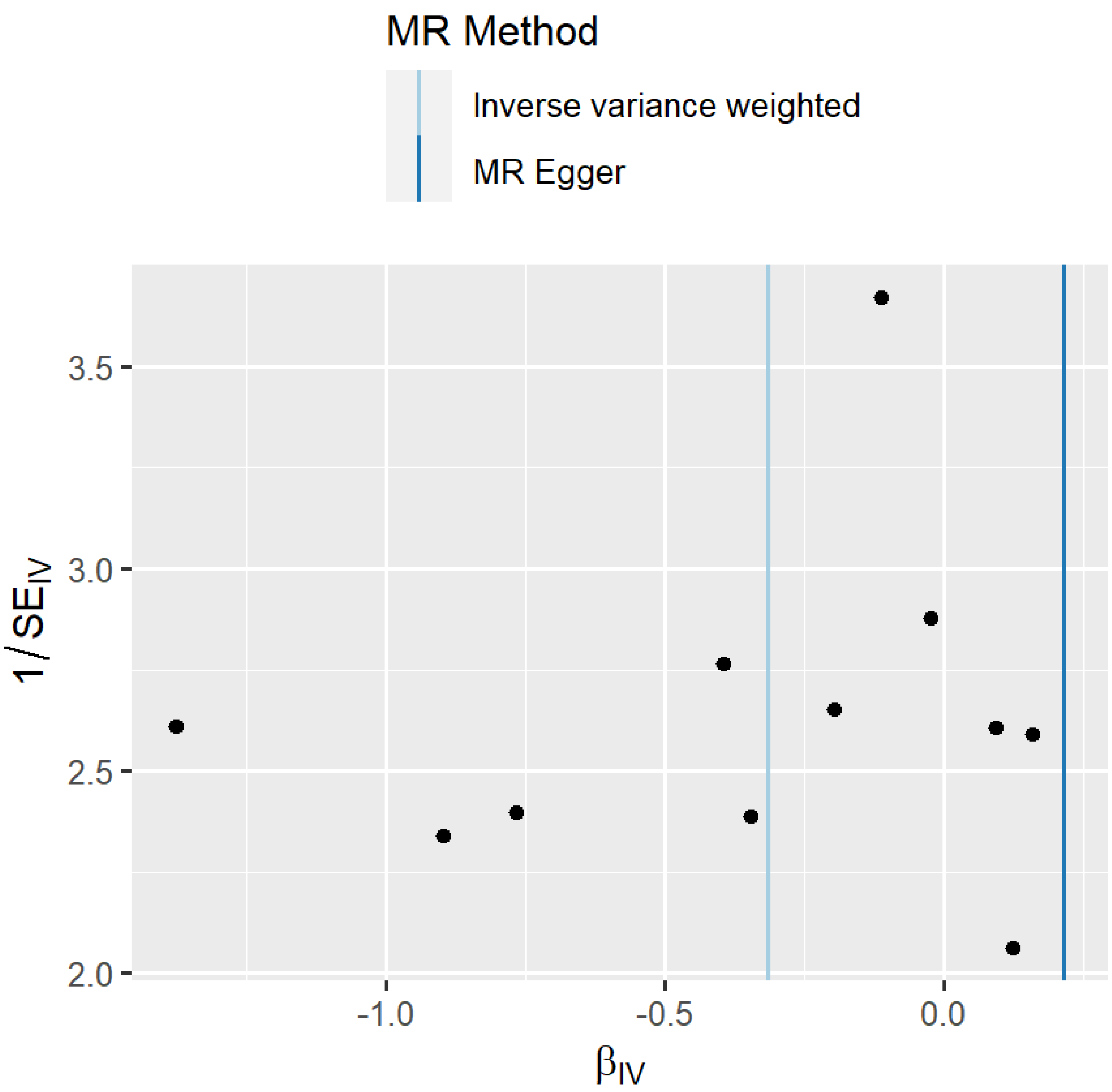
MR Method
Inverse variance weighted
MR Egger
3.5
3.0
2.5
2.0
$1/SE_{IV}$
-1.0
-0.5
0.0
β_{IV}

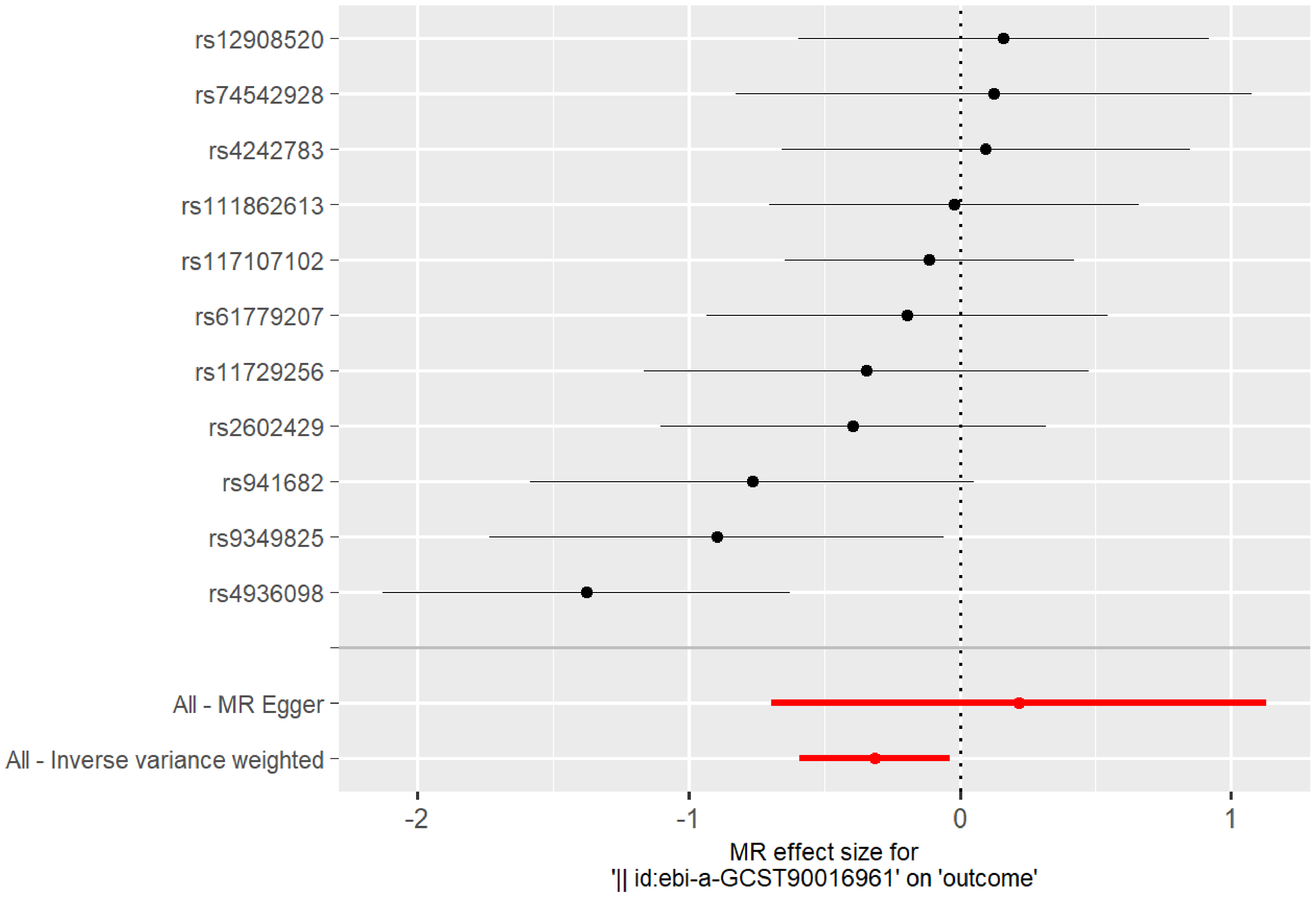
rs12908520
rs74542928
rs4242783
rs111862613
rs117107102
rs61779207
rs11729256
rs2602429
rs941682
rs9349825
rs4936098
All - MR Egger
All - Inverse variance weighted
-2
-1
0
1
MR effect size for
'|| id:ebi-a-GCST90016961' on 'outcome'

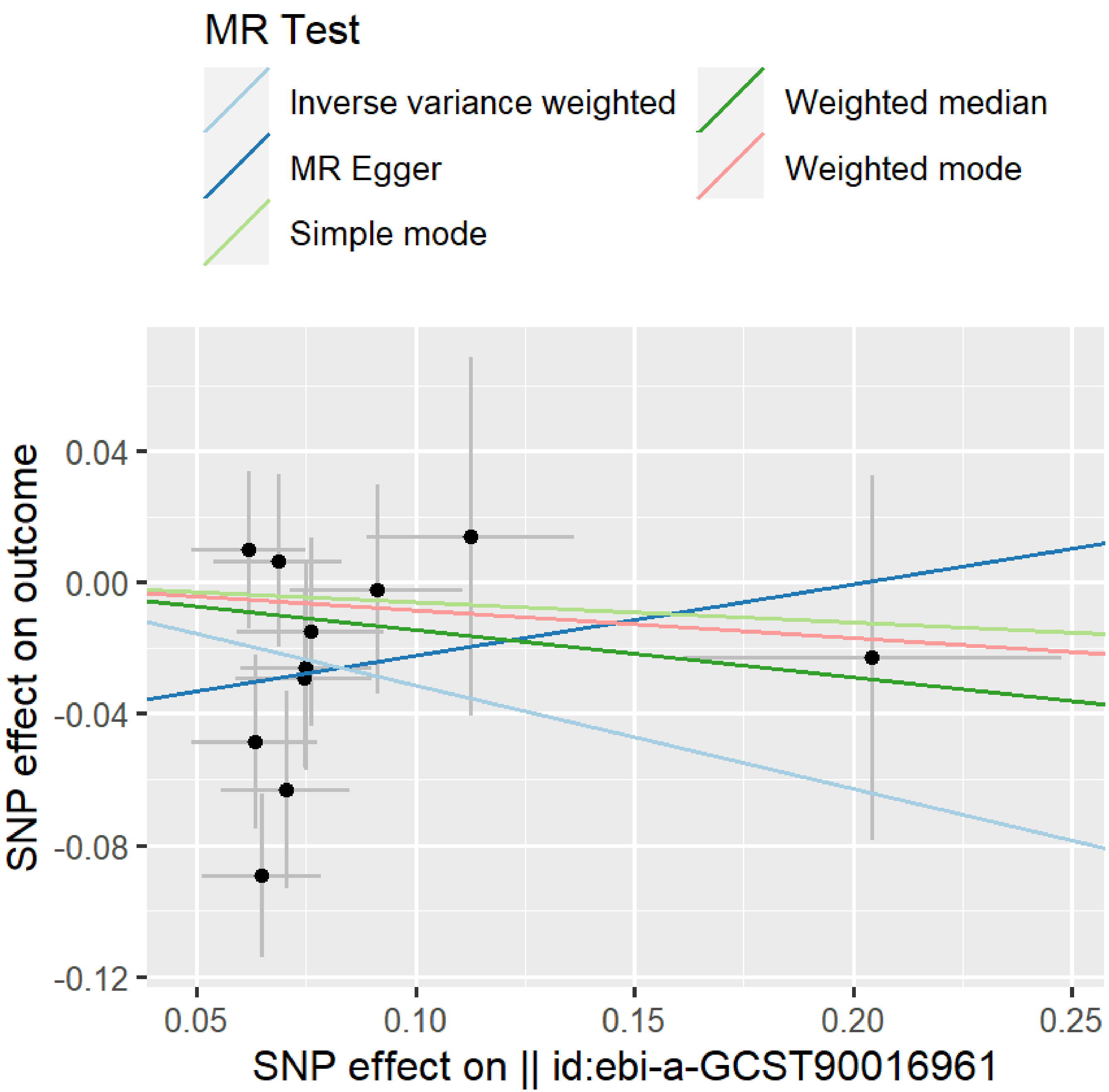
MR Test
Inverse variance weighted
MR Egger
Simple mode
Weighted median
Weighted mode
SNP effect on outcome
0.04
0.00
-0.04
-0.08
-0.12
0.05
0.10
0.15
0.20
0.25
SNP effect on || id:ebi-a-GCST90016961

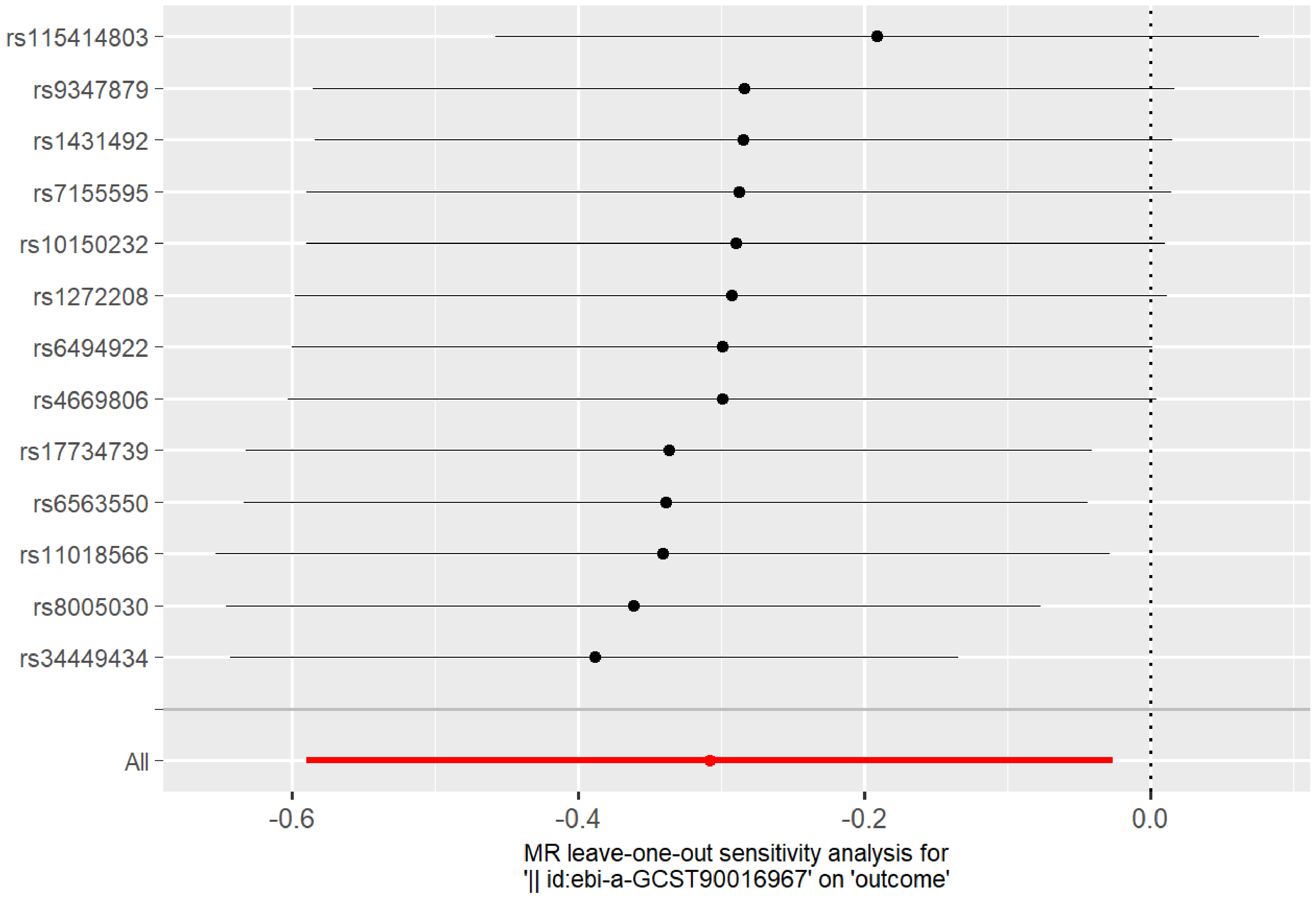
rs115414803
rs9347879
rs1431492
rs7155595
rs10150232
rs1272208
rs6494922
rs4669806
rs17734739
rs6563550
rs11018566
rs8005030
rs34449434
All
-0.6
-0.4
-0.2
0.0
MR leave-one-out sensitivity analysis for
'|| id:ebi-a-GCST90016967' on 'outcome'

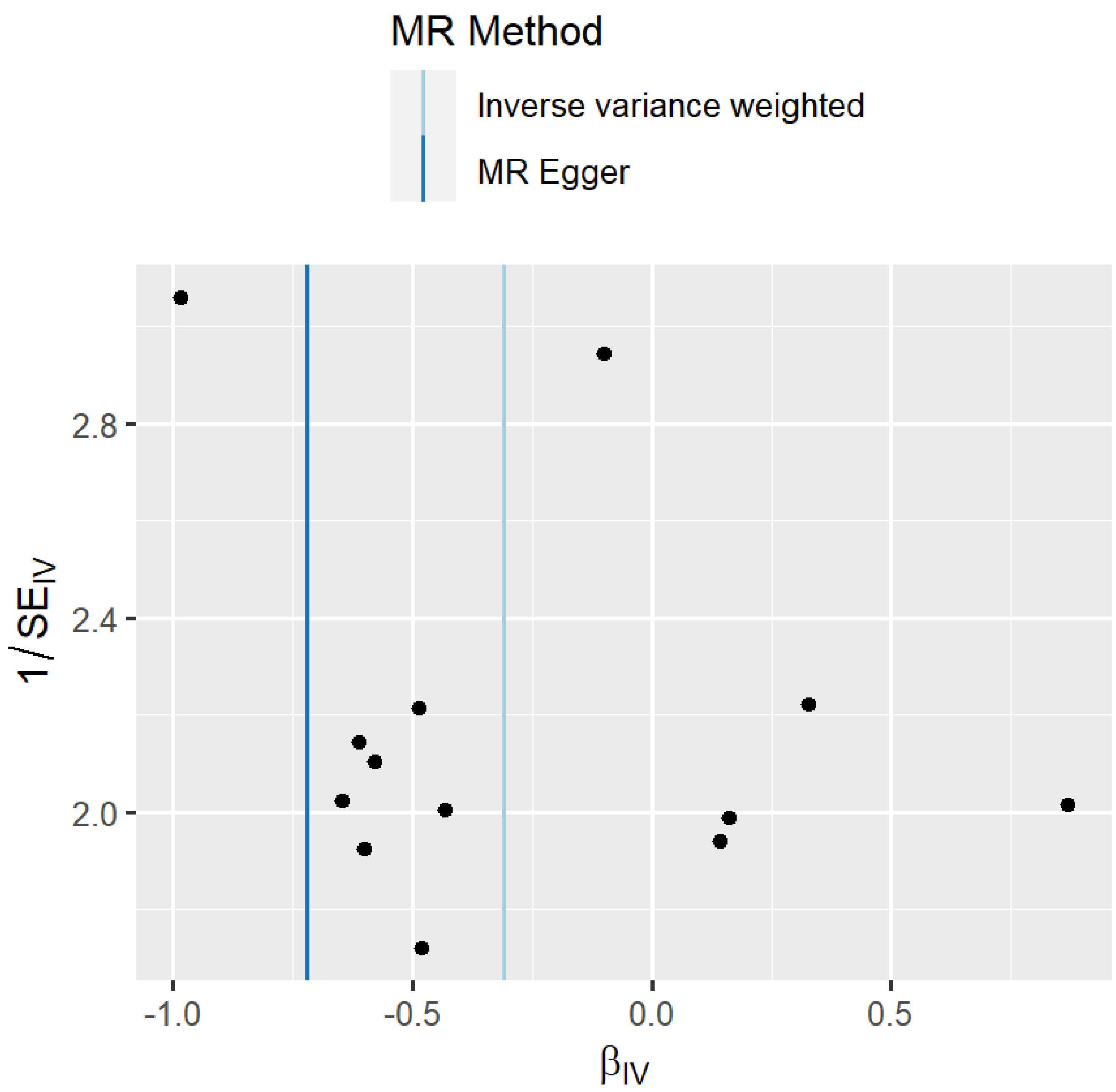

MR Method
Inverse variance weighted
MR Egger
2.8
2.4
2.0
1/SE_IV
-1.0
-0.5
0.0
0.5
β_IV

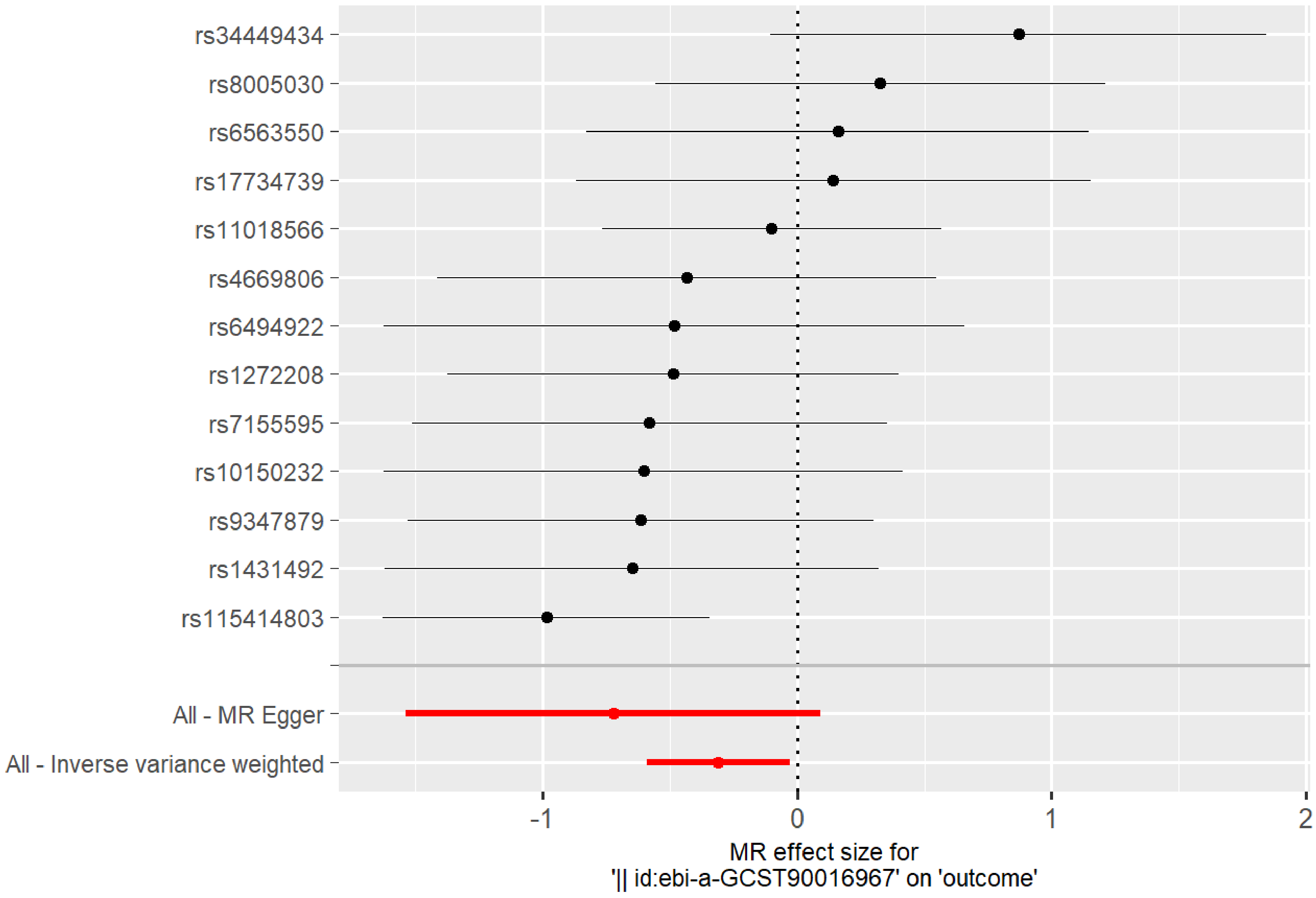
rs34449434
rs8005030
rs6563550
rs17734739
rs11018566
rs4669806
rs6494922
rs1272208
rs7155595
rs10150232
rs9347879
rs1431492
rs115414803
All - MR Egger
All - Inverse variance weighted
-1
0
1
2
MR effect size for
'|| id:ebi-a-GCST90016967' on 'outcome'

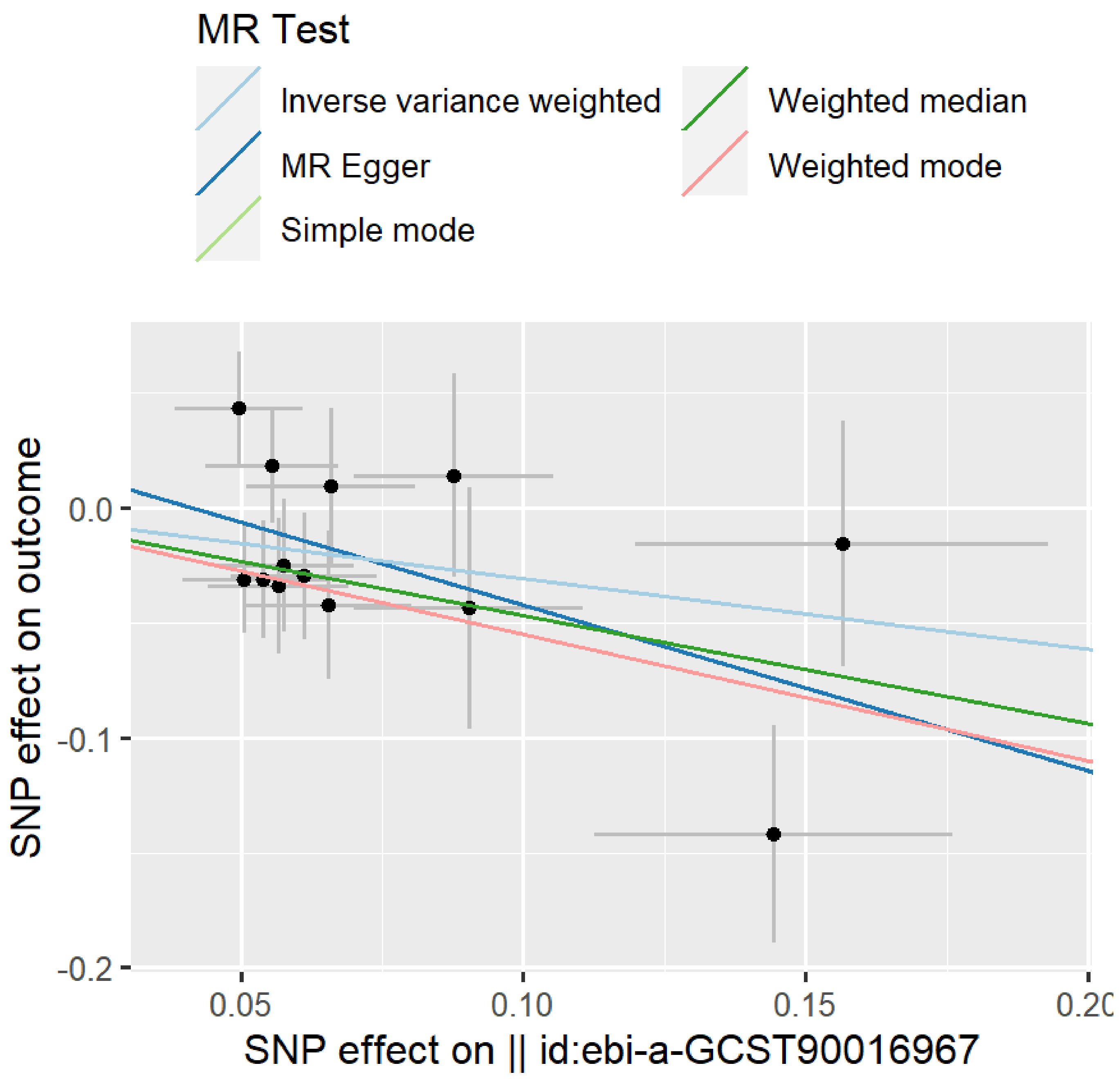
MR Test
Inverse variance weighted
MR Egger
Simple mode
Weighted median
Weighted mode
SNP effect on outcome
0.0
-0.1
-0.2
0.05
0.10
0.15
0.20
SNP effect on || id:ebi-a-GCST90016967

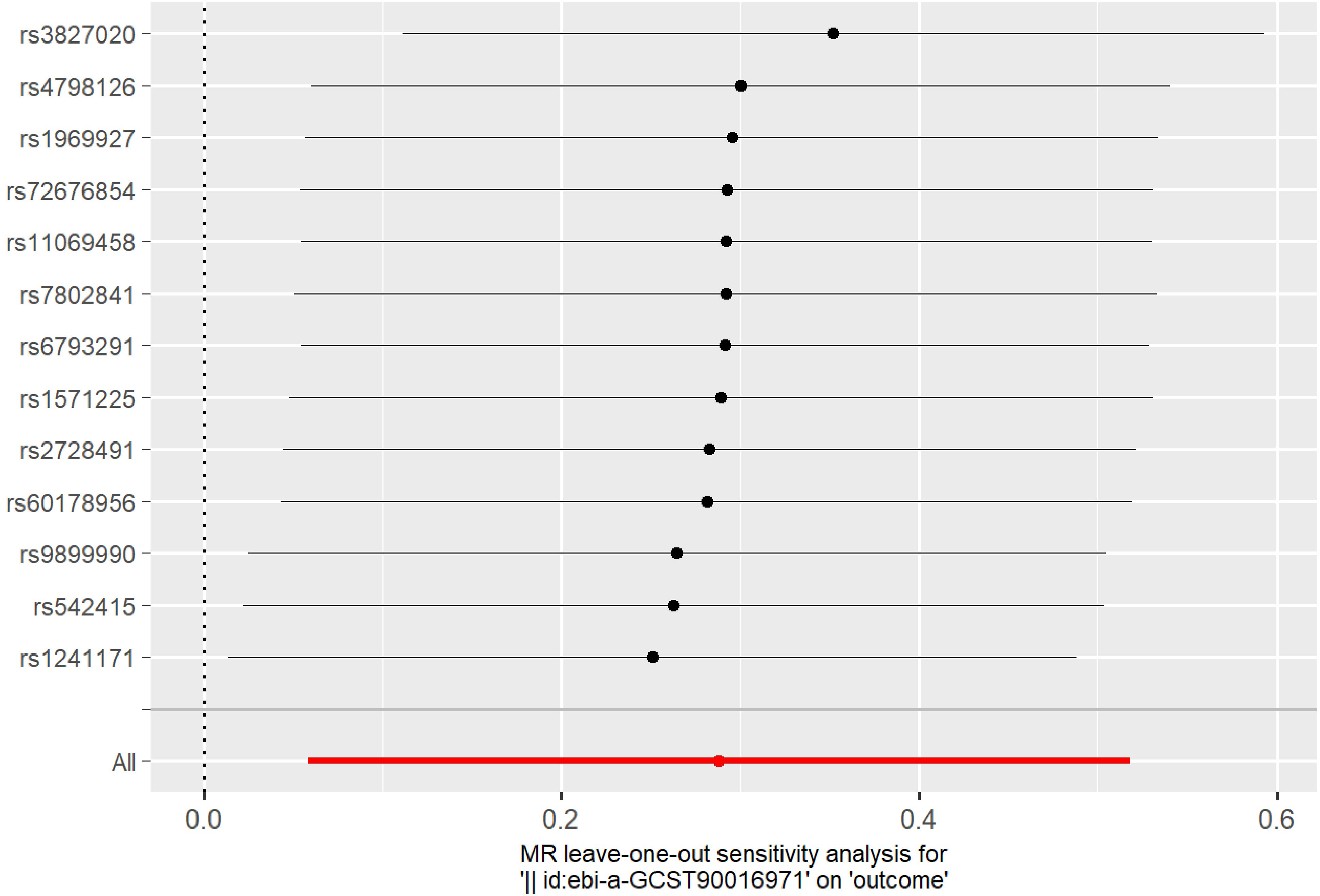

rs3827020
rs4798126
rs1969927
rs72676854
rs11069458
rs7802841
rs6793291
rs1571225
rs2728491
rs60178956
rs9899990
rs542415
rs1241171
All
0.0
0.2
0.4
0.6
MR leave-one-out sensitivity analysis for
'|| id:ebi-a-GCST90016971' on 'outcome'

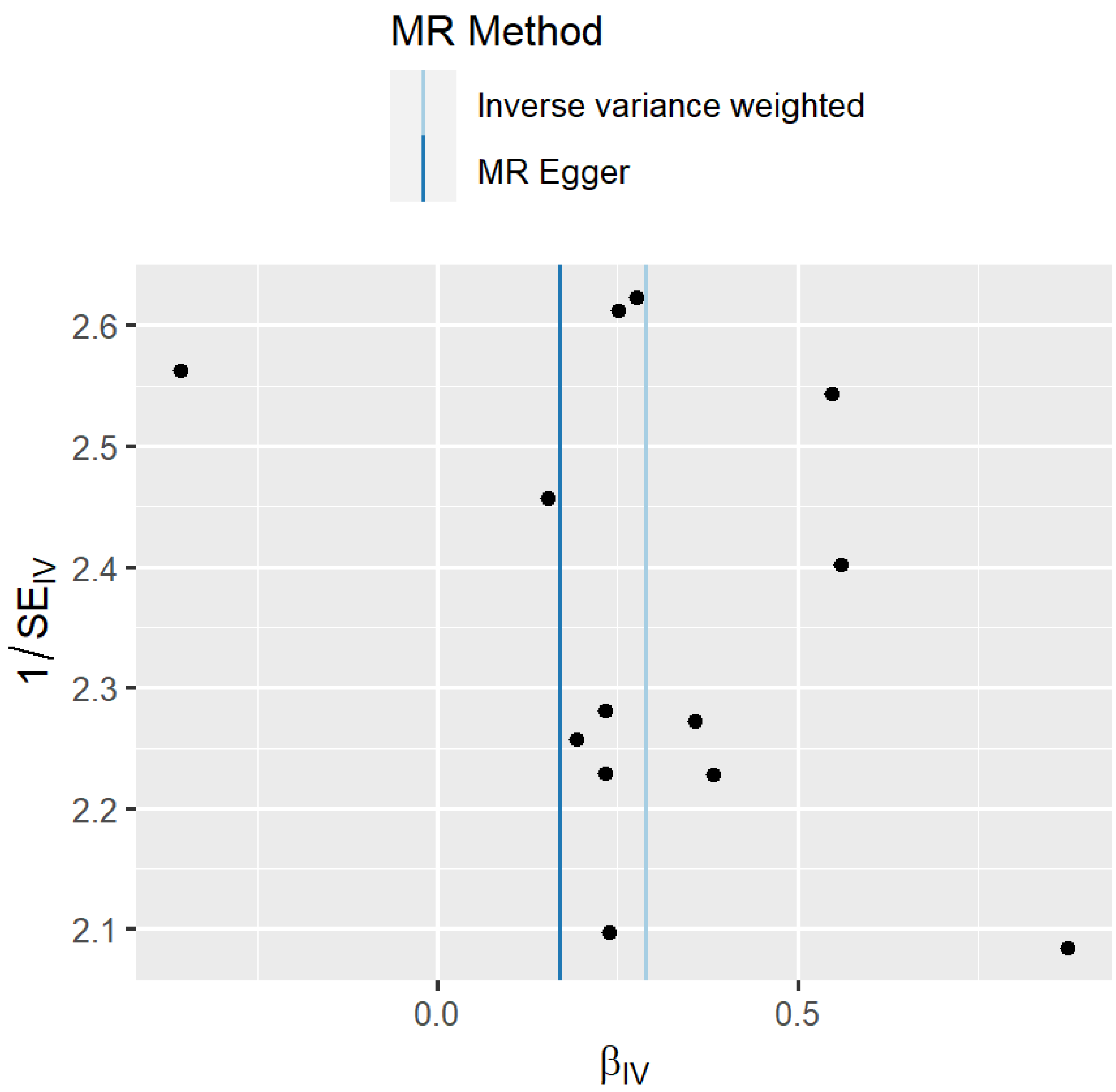

MR Method
Inverse variance weighted
MR Egger
2.6
2.5
2.4
2.3
2.2
2.1
1/SE$_{IV}$
0.0
0.5
β_{IV}

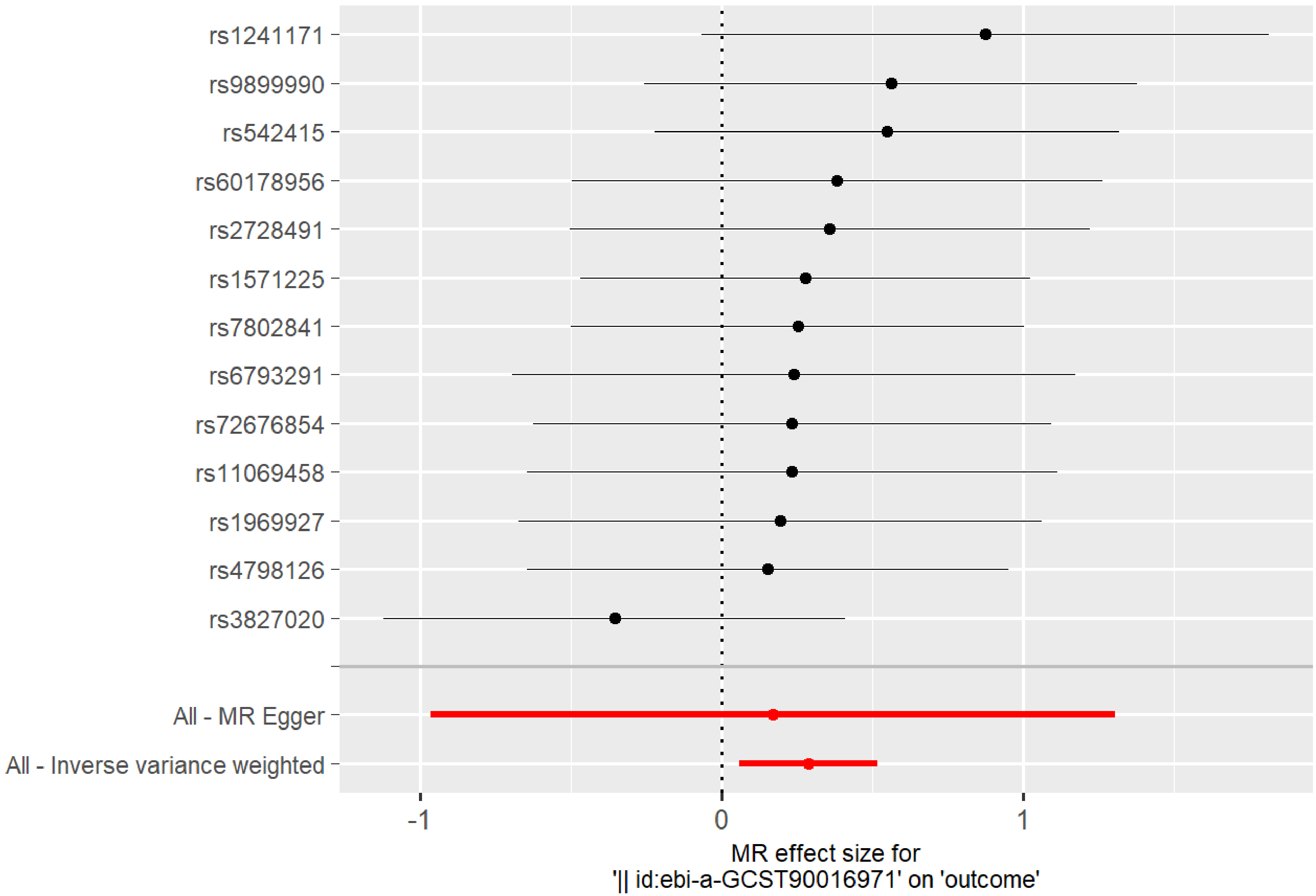
rs1241171
rs9899990
rs542415
rs60178956
rs2728491
rs1571225
rs7802841
rs6793291
rs72676854
rs11069458
rs1969927
rs4798126
rs3827020
All - MR Egger
All - Inverse variance weighted
-1
0
1
MR effect size for
'|| id:ebi-a-GCST90016971' on 'outcome'

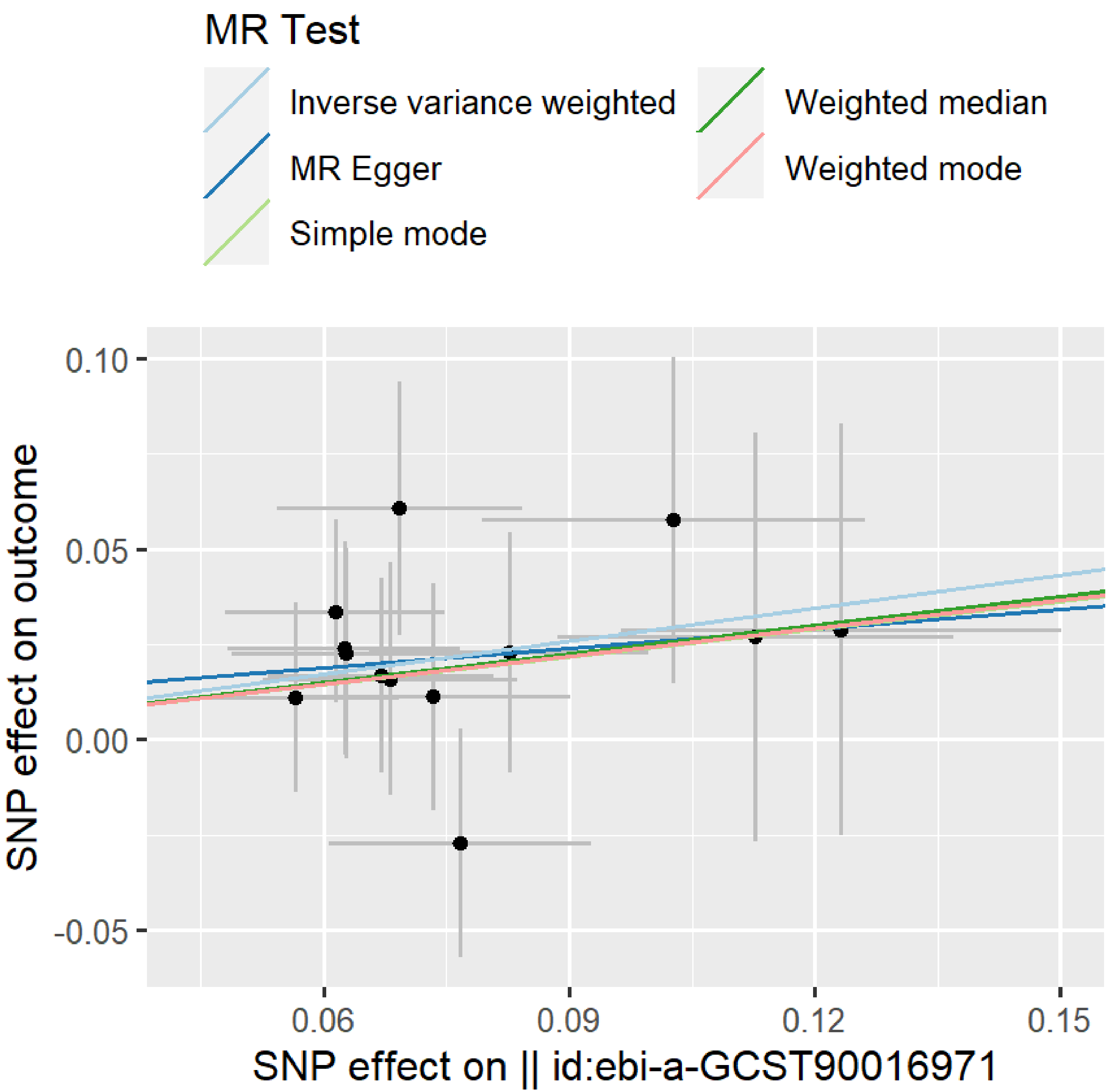

MR Test
Inverse variance weighted
MR Egger
Simple mode
Weighted median
Weighted mode
0.10
0.05
0.00
-0.05
SNP effect on outcome
0.06
0.09
0.12
0.15
SNP effect on || id:ebi-a-GCST90016971

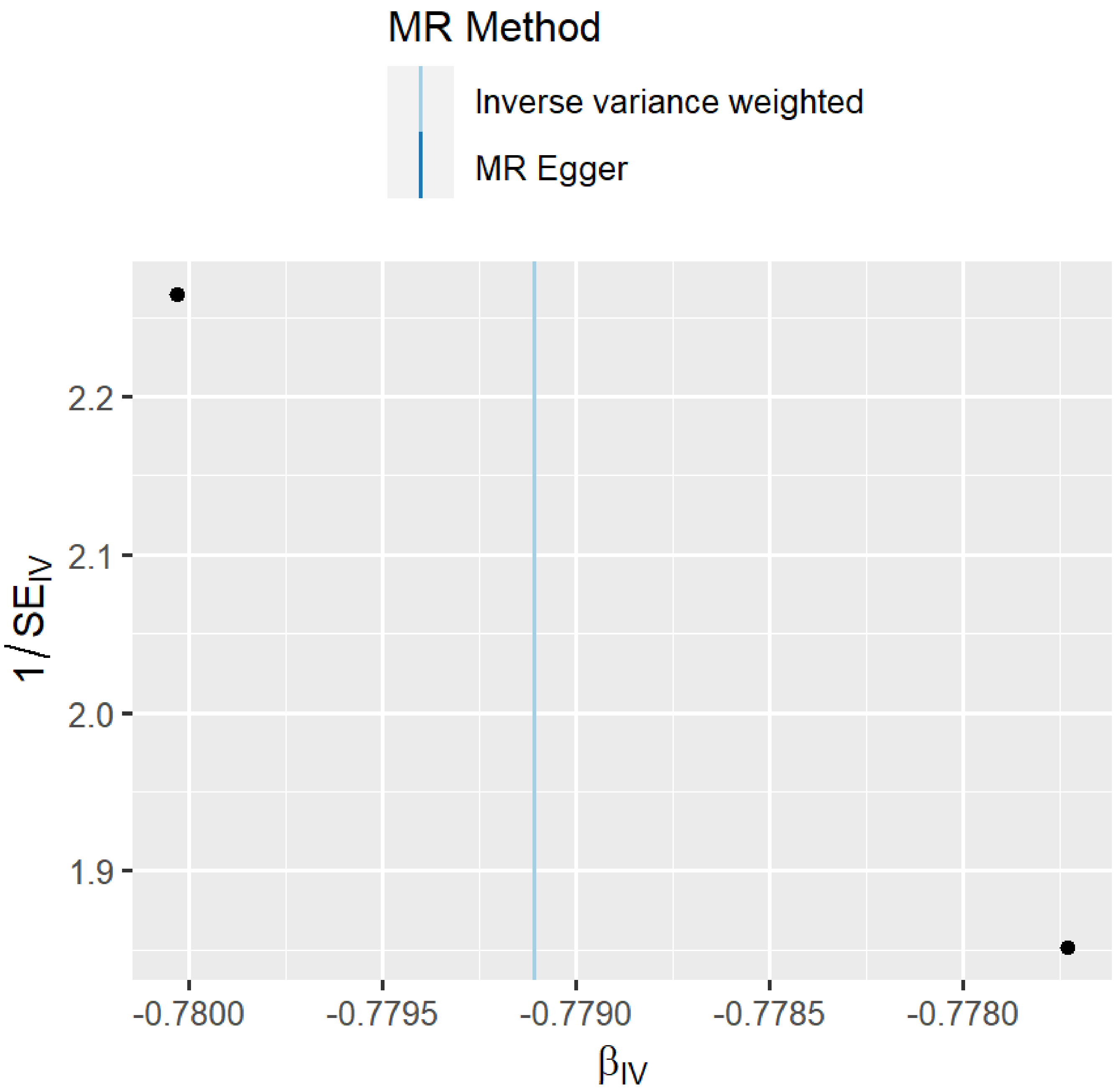
MR Method
Inverse variance weighted
MR Egger
2.2
2.1
2.0
1.9
1/SE_IV
-0.7800
-0.7795
-0.7790
-0.7785
-0.7780
β_IV

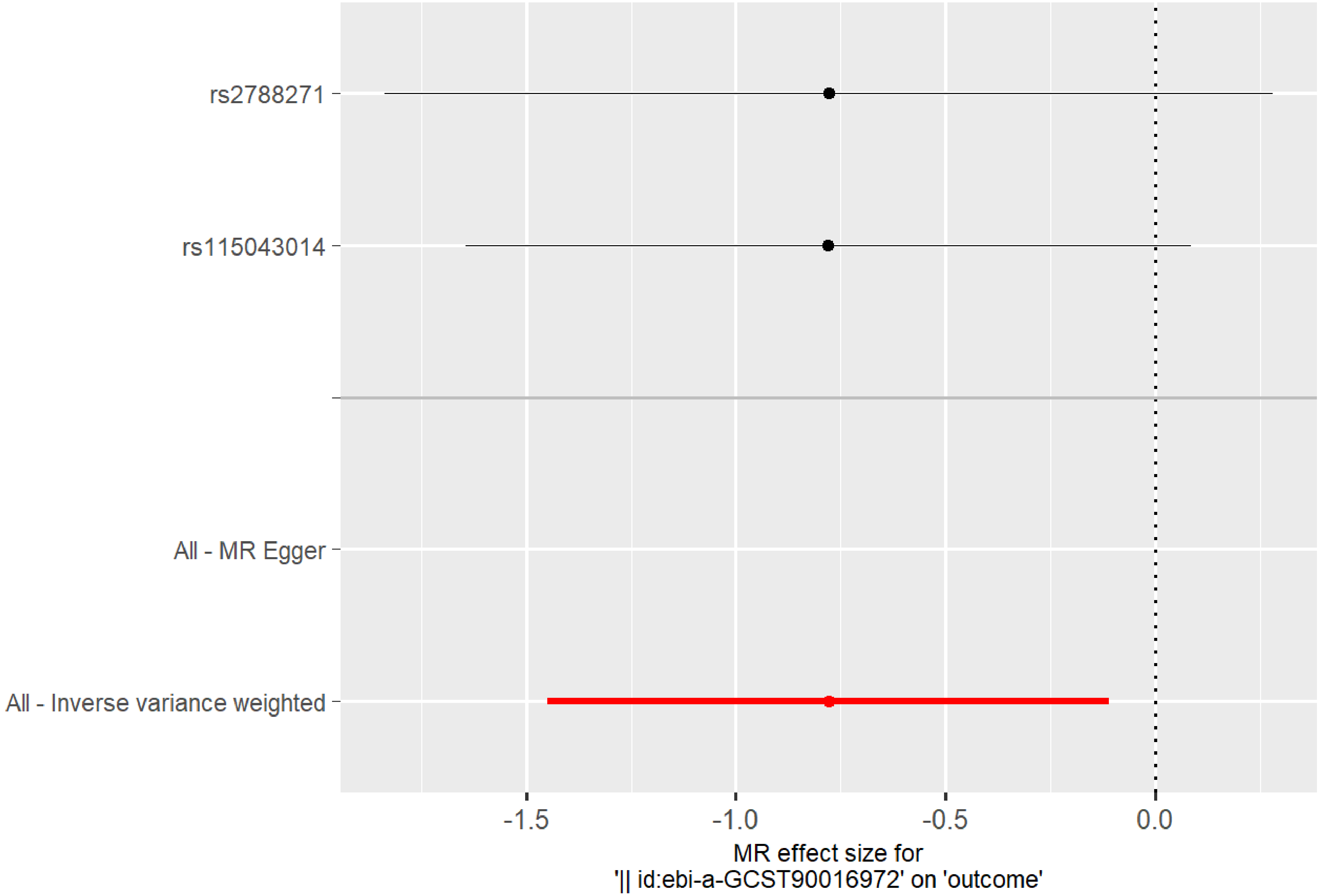

rs2788271
rs115043014
All - MR Egger
All - Inverse variance weighted
-1.5
-1.0
-0.5
0.0
MR effect size for
'|| id:ebi-a-GCST90016972' on 'outcome'

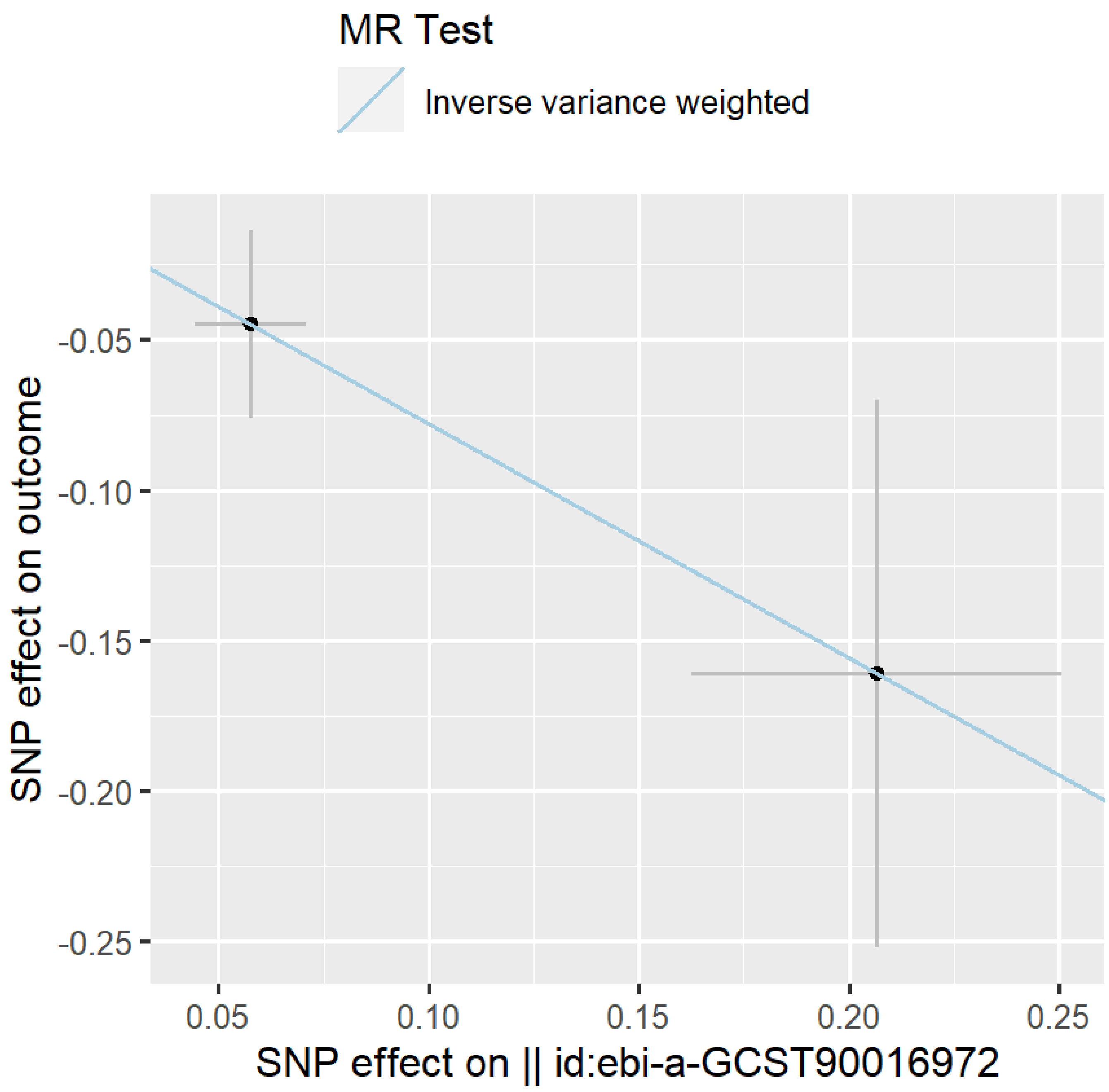
MR Test
Inverse variance weighted
-0.05
-0.10
-0.15
-0.20
-0.25
SNP effect on outcome
0.05
0.10
0.15
0.20
0.25
SNP effect on || id:ebi-a-GCST90016972

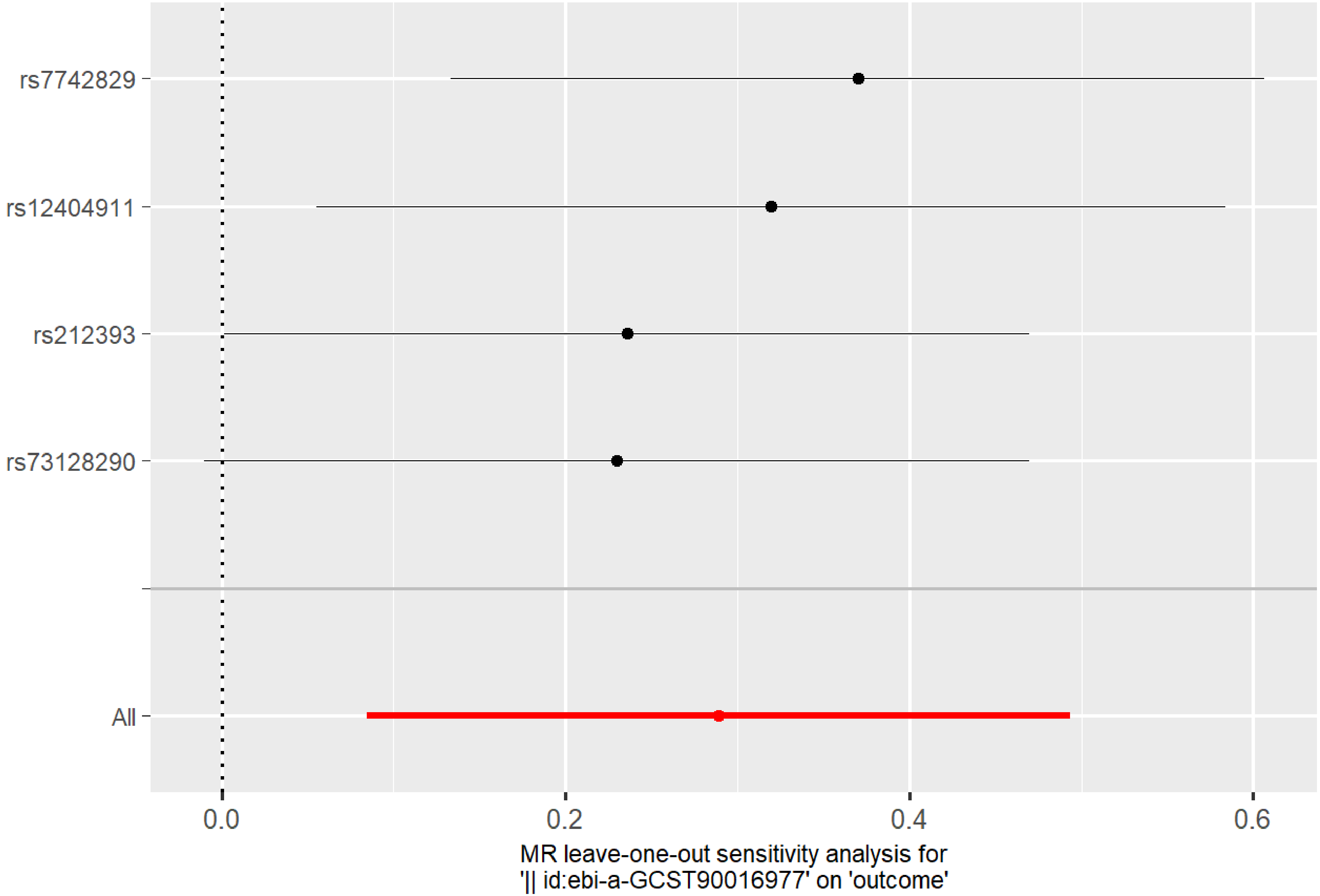
rs7742829
rs12404911
rs212393
rs73128290
All
0.0
0.2
0.4
0.6
MR leave-one-out sensitivity analysis for
'|| id:ebi-a-GCST90016977' on 'outcome'

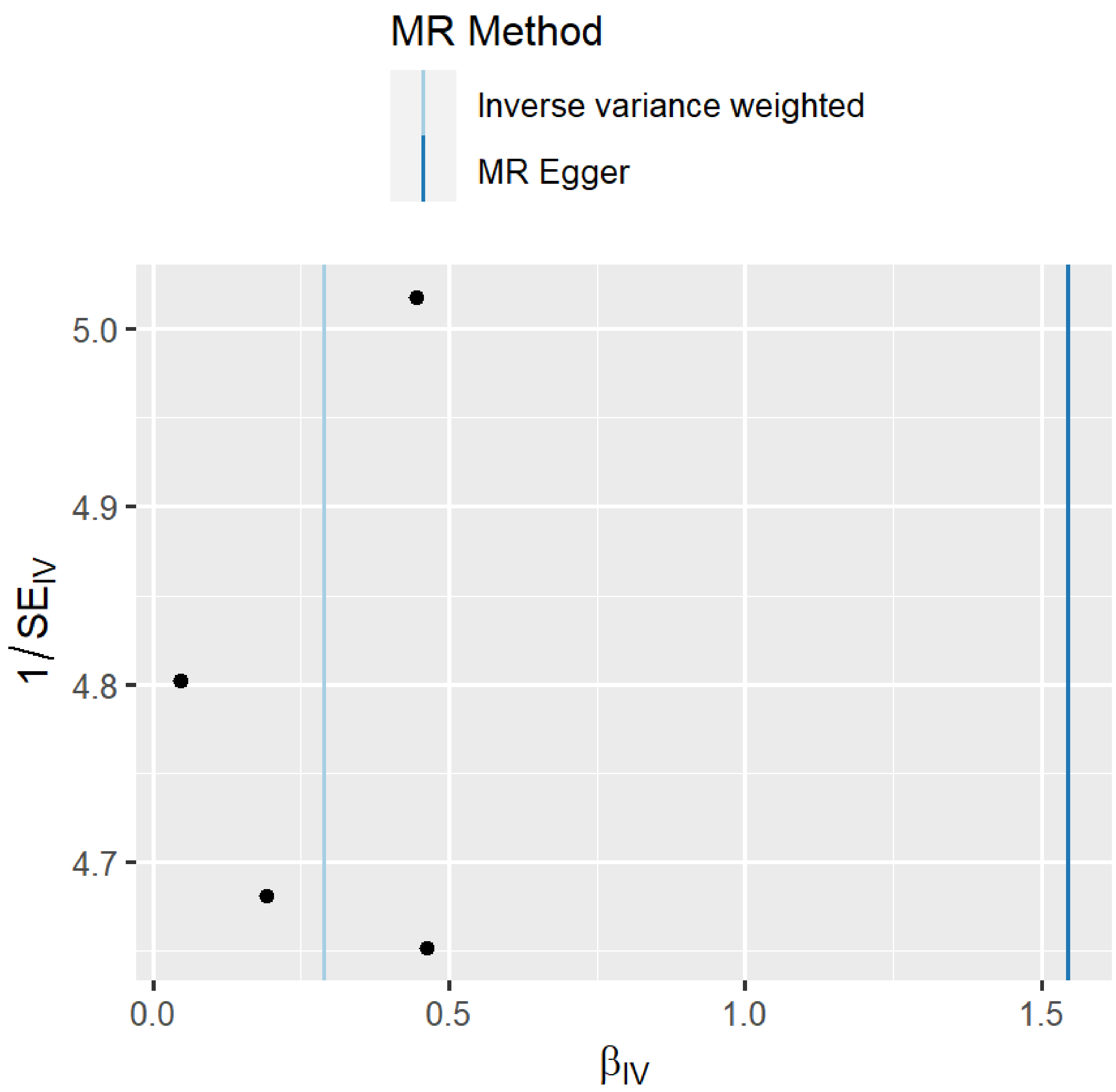
MR Method
Inverse variance weighted
MR Egger
5.0
4.9
4.8
4.7
1/SE_IV
0.0
0.5
1.0
1.5
β_IV

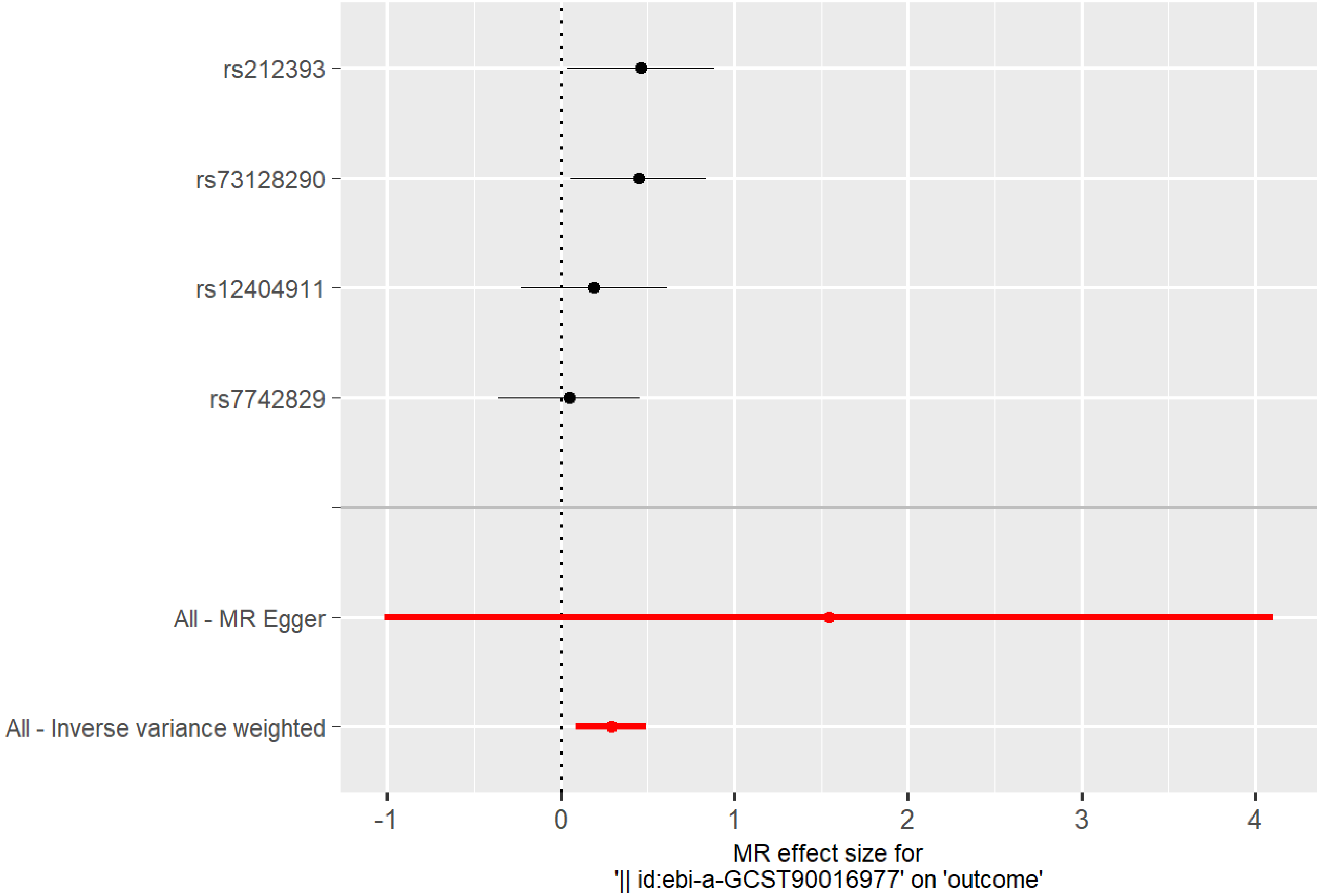
rs212393
rs73128290
rs12404911
rs7742829
All - MR Egger
All - Inverse variance weighted
-1
0
1
2
3
4
MR effect size for
'|| id:ebi-a-GCST90016977' on 'outcome'

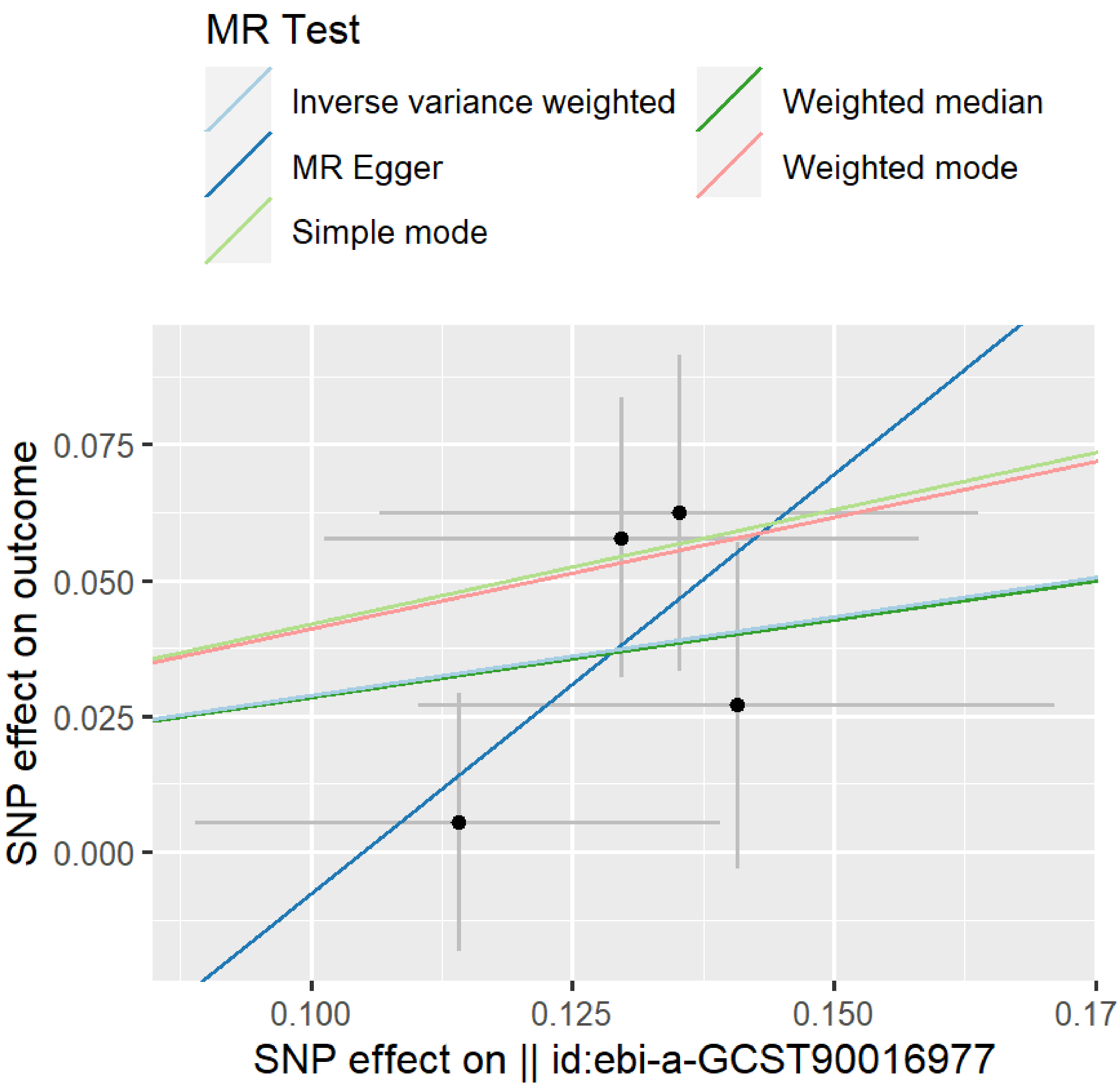
MR Test
Inverse variance weighted
MR Egger
Simple mode
Weighted median
Weighted mode
0.075
0.050
0.025
0.000
SNP effect on outcome
0.100
0.125
0.150
0.17
SNP effect on || id:ebi-a-GCST90016977

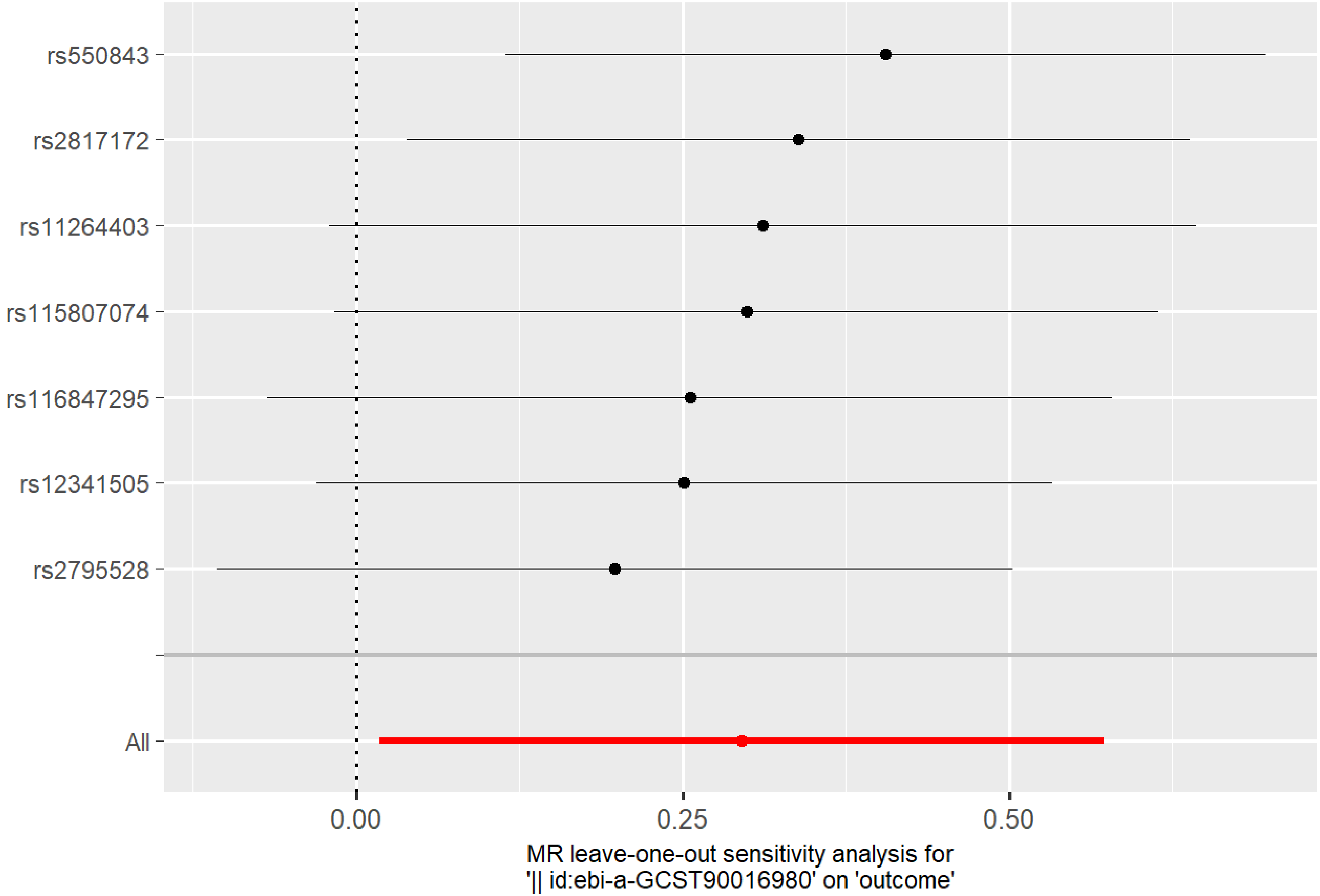

rs550843
rs2817172
rs11264403
rs115807074
rs116847295
rs12341505
rs2795528
All
0.00
0.25
0.50
MR leave-one-out sensitivity analysis for
'|| id:ebi-a-GCST90016980' on 'outcome'

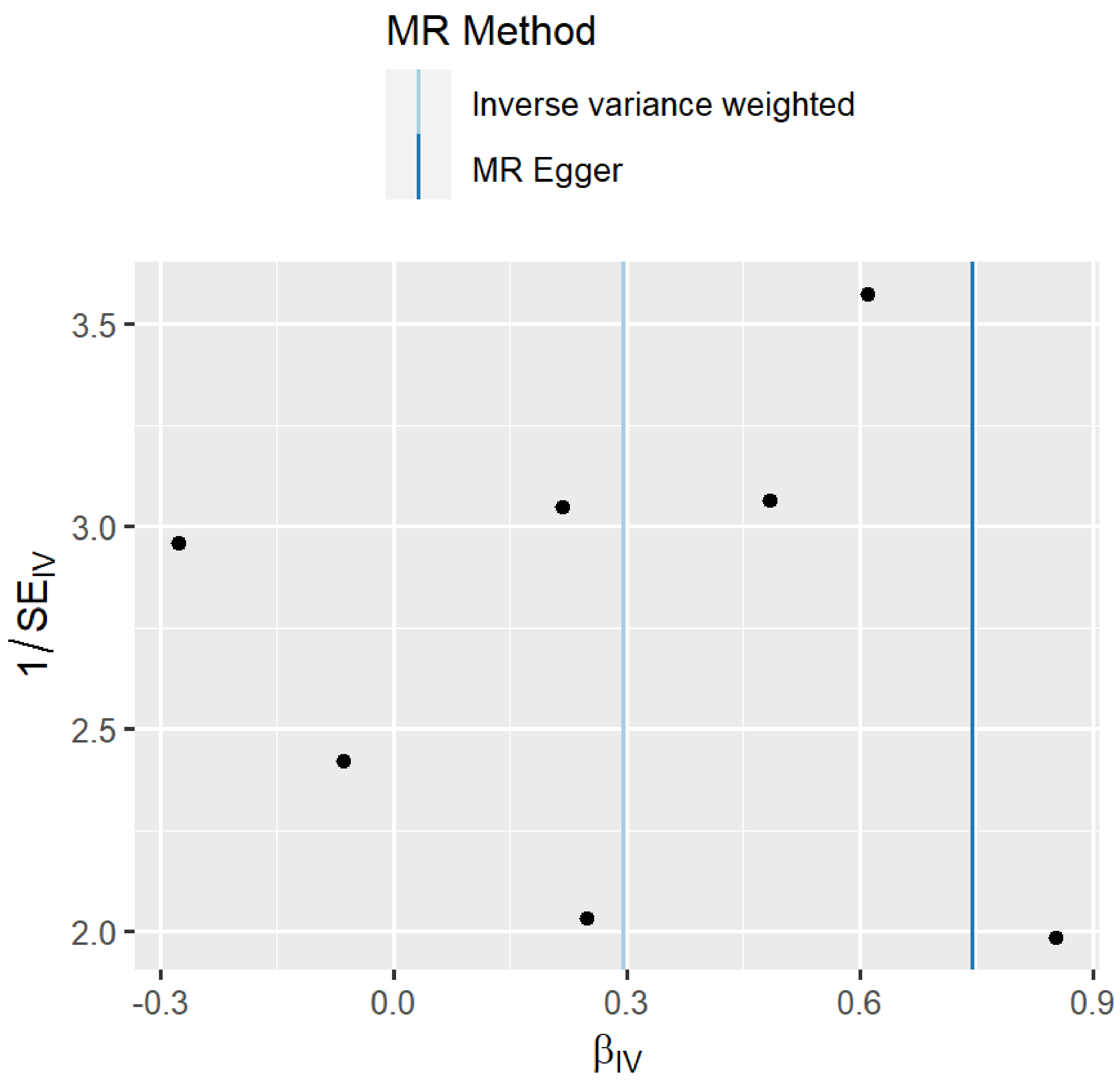

MR Method
Inverse variance weighted
MR Egger
3.5
3.0
2.5
2.0
-0.3
0.0
0.3
0.6
0.9
1/SE_IV
β_IV

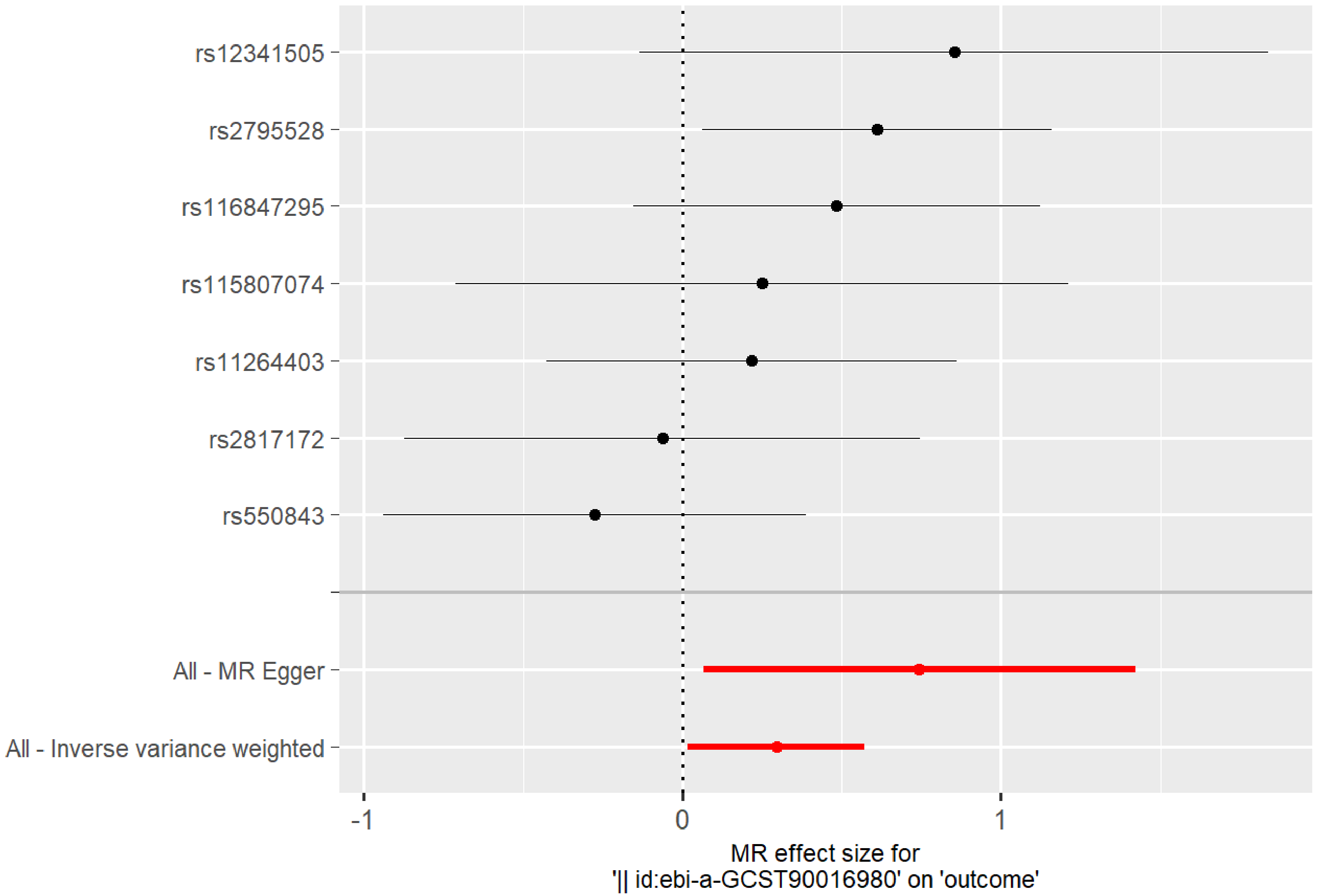

rs12341505
rs2795528
rs116847295
rs115807074
rs11264403
rs2817172
rs550843
All - MR Egger
All - Inverse variance weighted
-1
0
1
MR effect size for
'|| id:ebi-a-GCST90016980' on 'outcome'

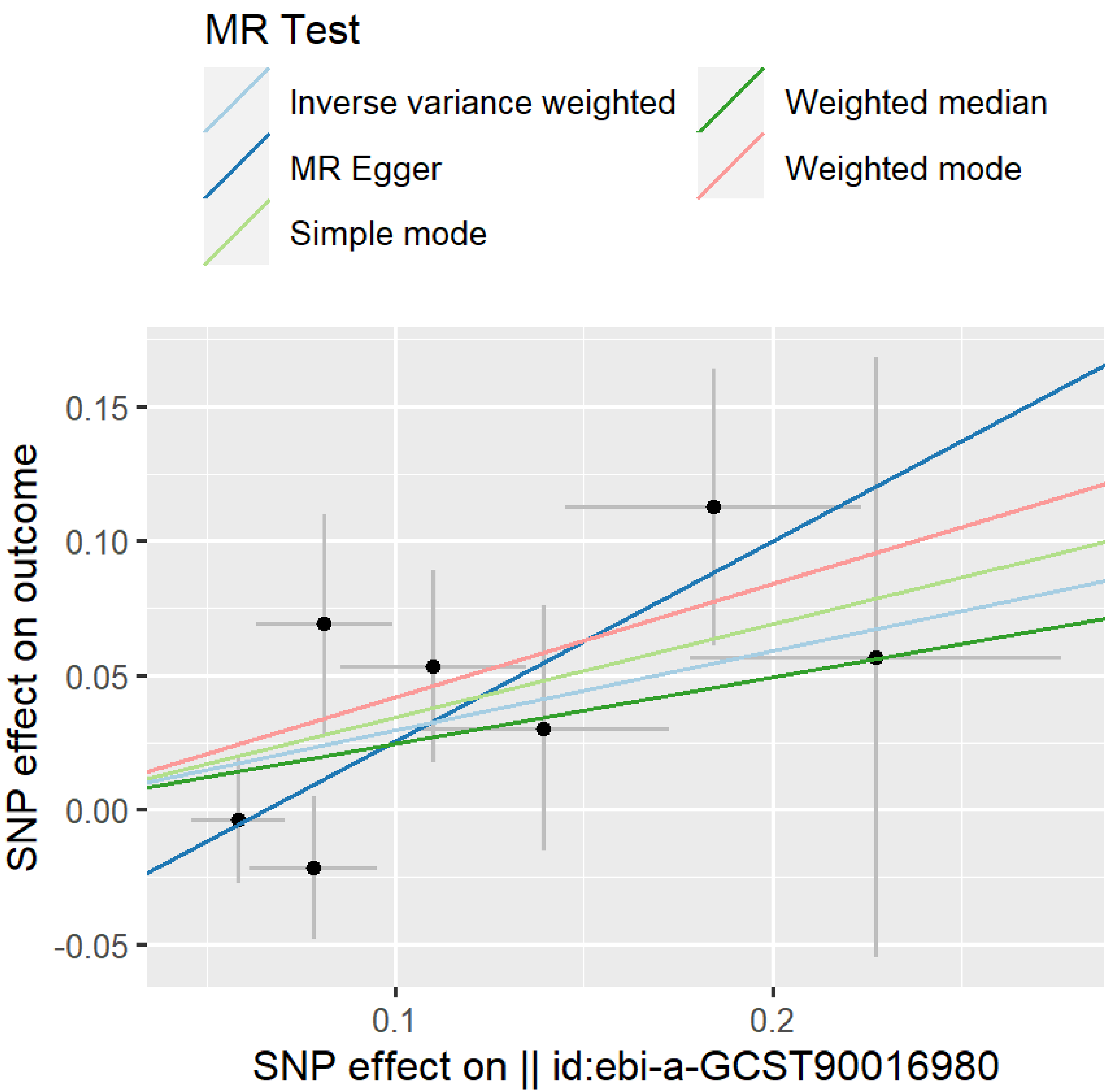
MR Test
Inverse variance weighted
MR Egger
Simple mode
Weighted median
Weighted mode
0.15
0.10
0.05
0.00
-0.05
SNP effect on outcome
0.1
0.2
SNP effect on || id:ebi-a-GCST90016980

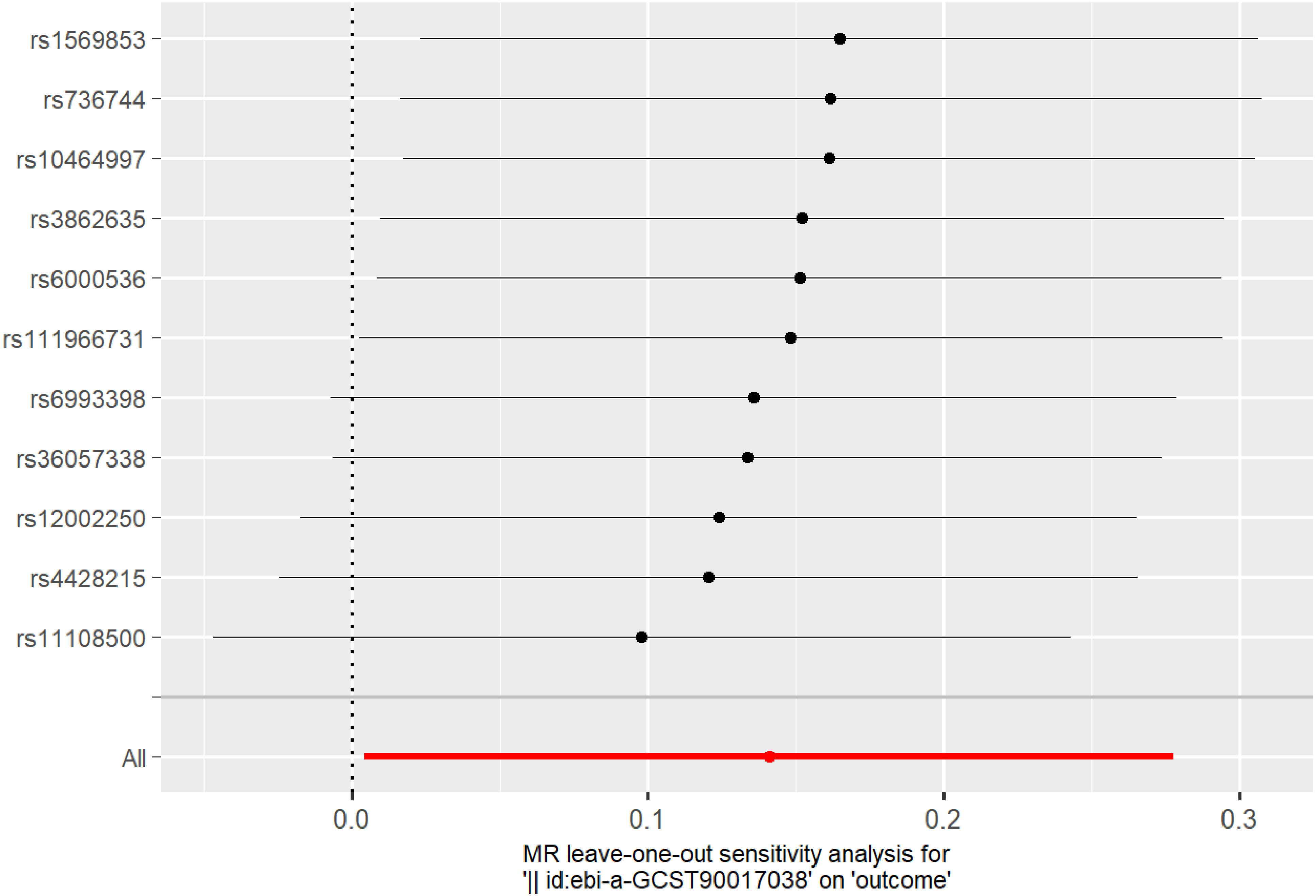
rs1569853
rs736744
rs10464997
rs3862635
rs6000536
rs111966731
rs6993398
rs36057338
rs12002250
rs4428215
rs11108500
All
0.0
0.1
0.2
0.3
MR leave-one-out sensitivity analysis for
'|| id:ebi-a-GCST90017038' on 'outcome'

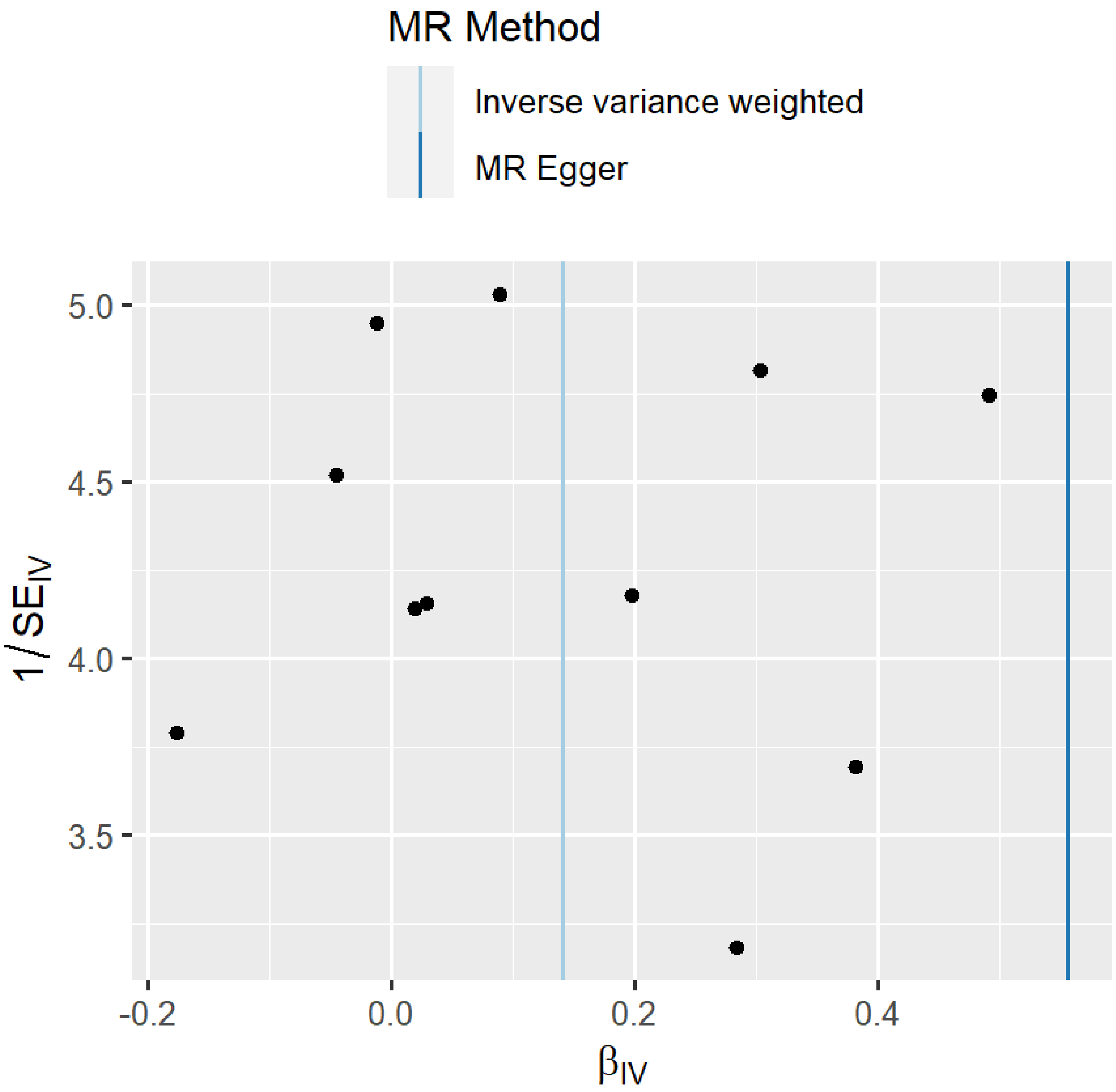

MR Method
Inverse variance weighted
MR Egger
5.0
4.5
4.0
3.5
1/SE_IV
-0.2
0.0
0.2
0.4
β_IV

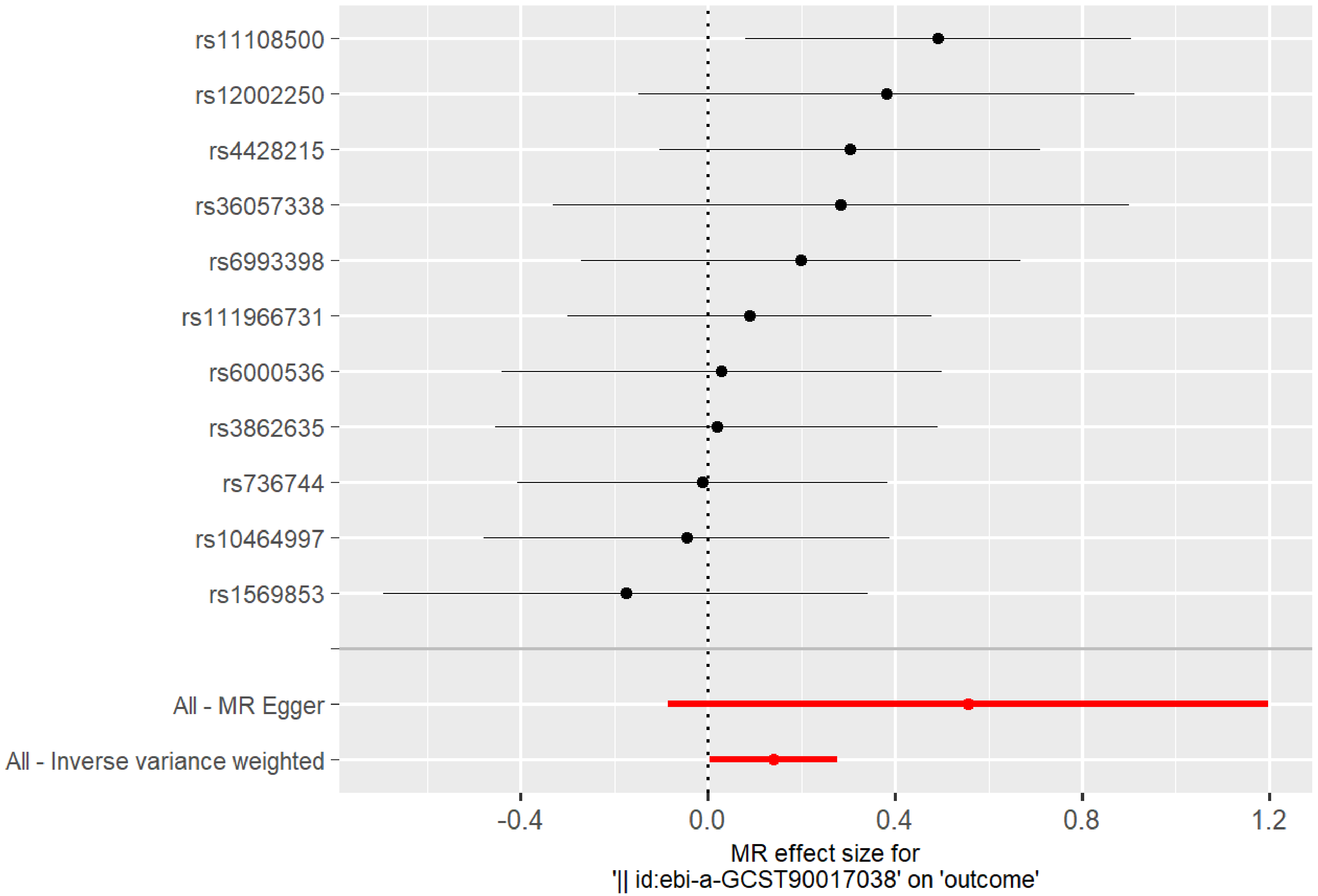
rs11108500
rs12002250
rs4428215
rs36057338
rs6993398
rs111966731
rs6000536
rs3862635
rs736744
rs10464997
rs1569853
All - MR Egger
All - Inverse variance weighted
-0.4
0.0
0.4
0.8
1.2
MR effect size for
'|| id:ebi-a-GCST90017038' on 'outcome'

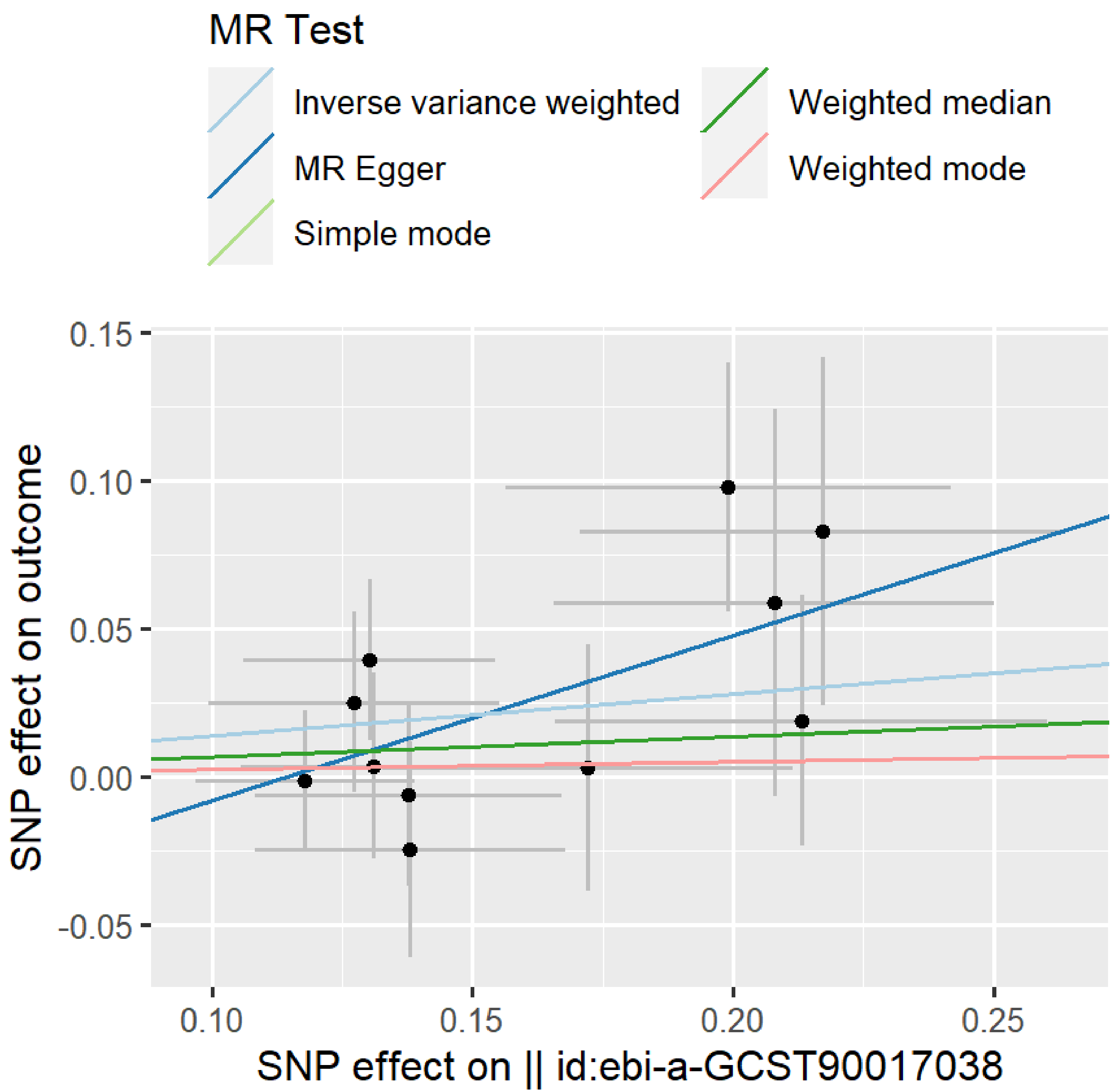
MR Test
Inverse variance weighted
MR Egger
Simple mode
Weighted median
Weighted mode
0.15
0.10
0.05
0.00
-0.05
SNP effect on outcome
0.10
0.15
0.20
0.25
SNP effect on || id:ebi-a-GCST90017038

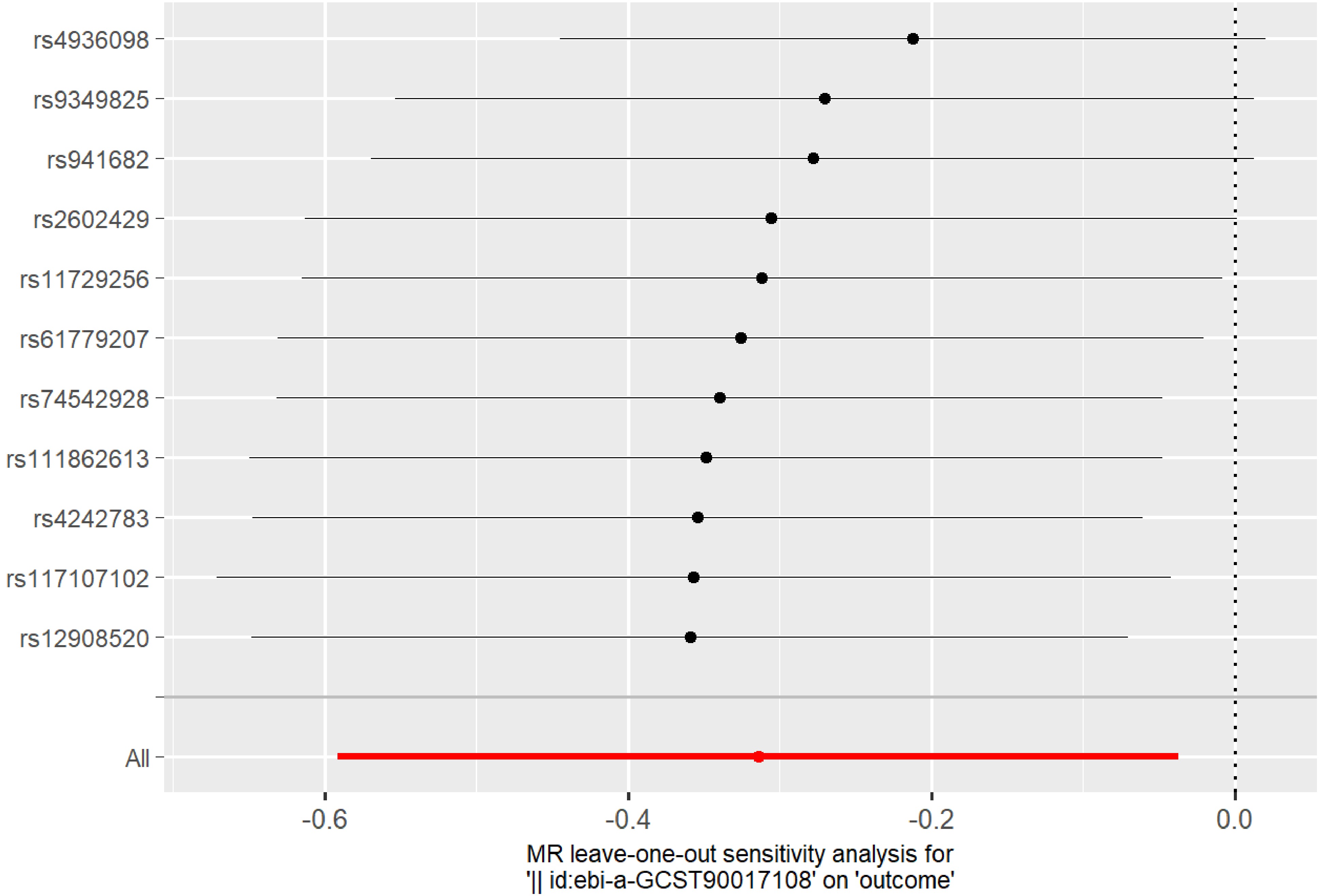
rs4936098
rs9349825
rs941682
rs2602429
rs11729256
rs61779207
rs74542928
rs111862613
rs4242783
rs117107102
rs12908520
All
-0.6
-0.4
-0.2
0.0
MR leave-one-out sensitivity analysis for
'|| id:ebi-a-GCST90017108' on 'outcome'

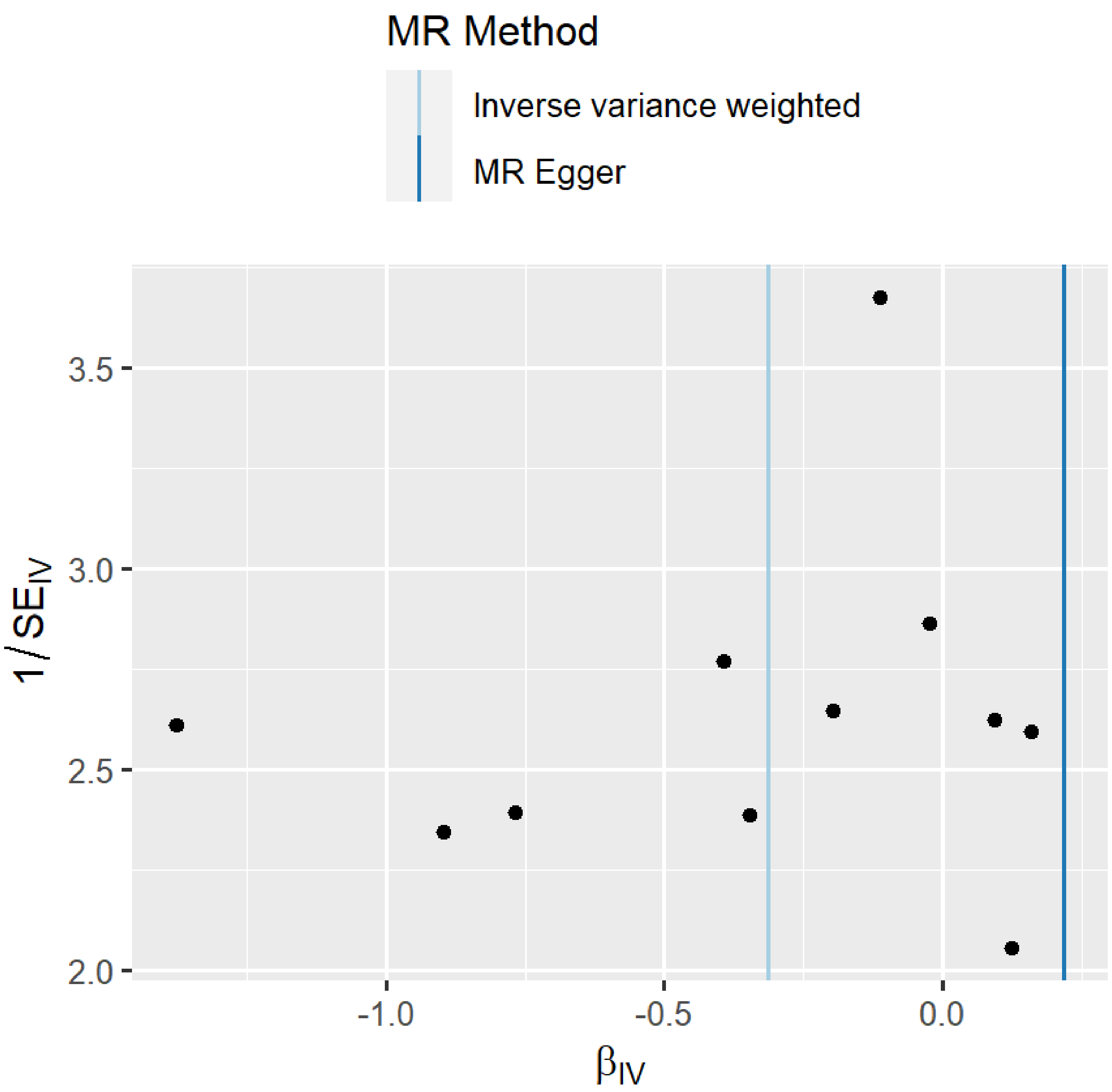
MR Method
Inverse variance weighted
MR Egger
3.5
3.0
2.5
2.0
1/SE_IV
-1.0
-0.5
0.0
β_IV

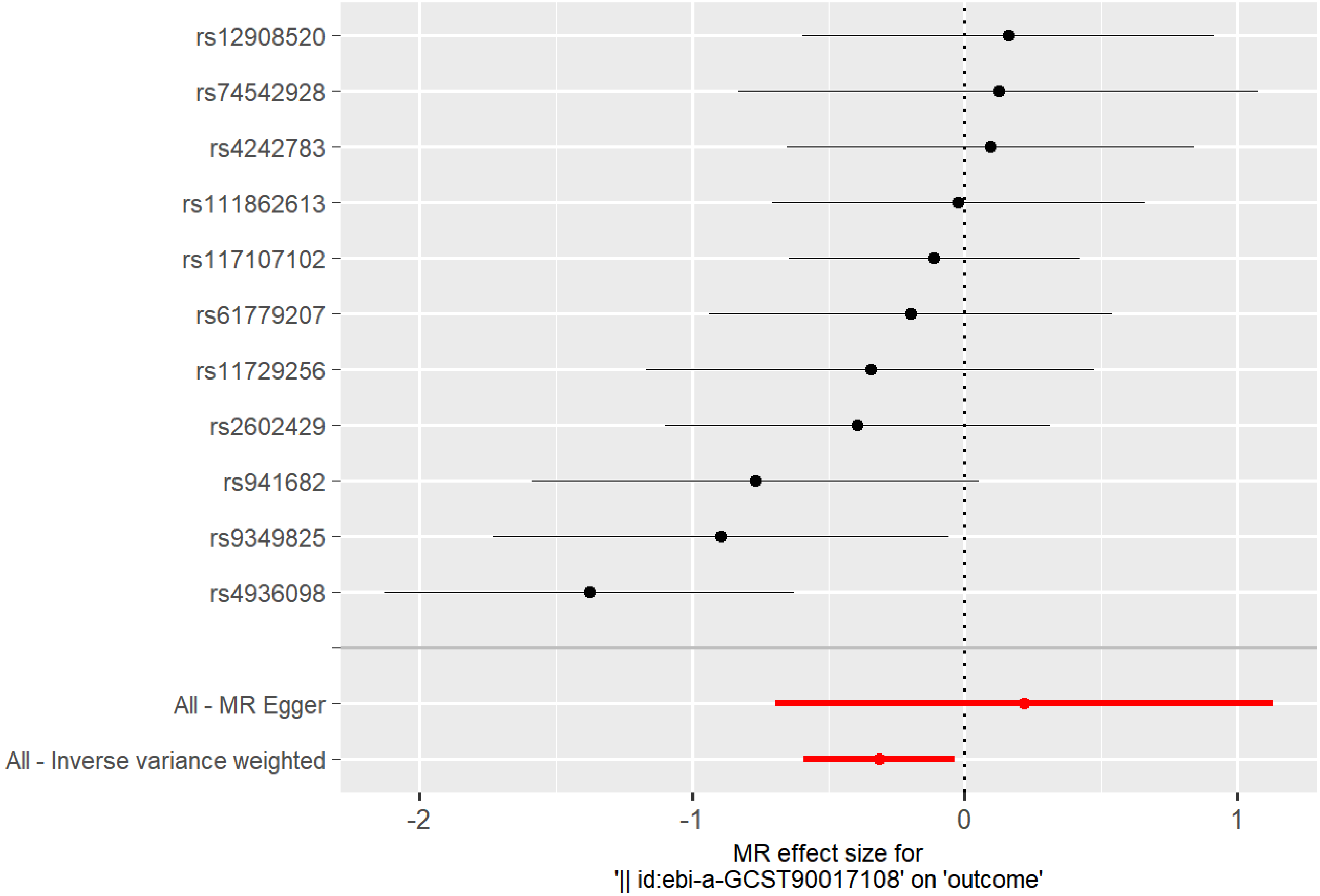

rs12908520
rs74542928
rs4242783
rs111862613
rs117107102
rs61779207
rs11729256
rs2602429
rs941682
rs9349825
rs4936098
All - MR Egger
All - Inverse variance weighted
-2
-1
0
1
MR effect size for
'|| id:ebi-a-GCST90017108' on 'outcome'

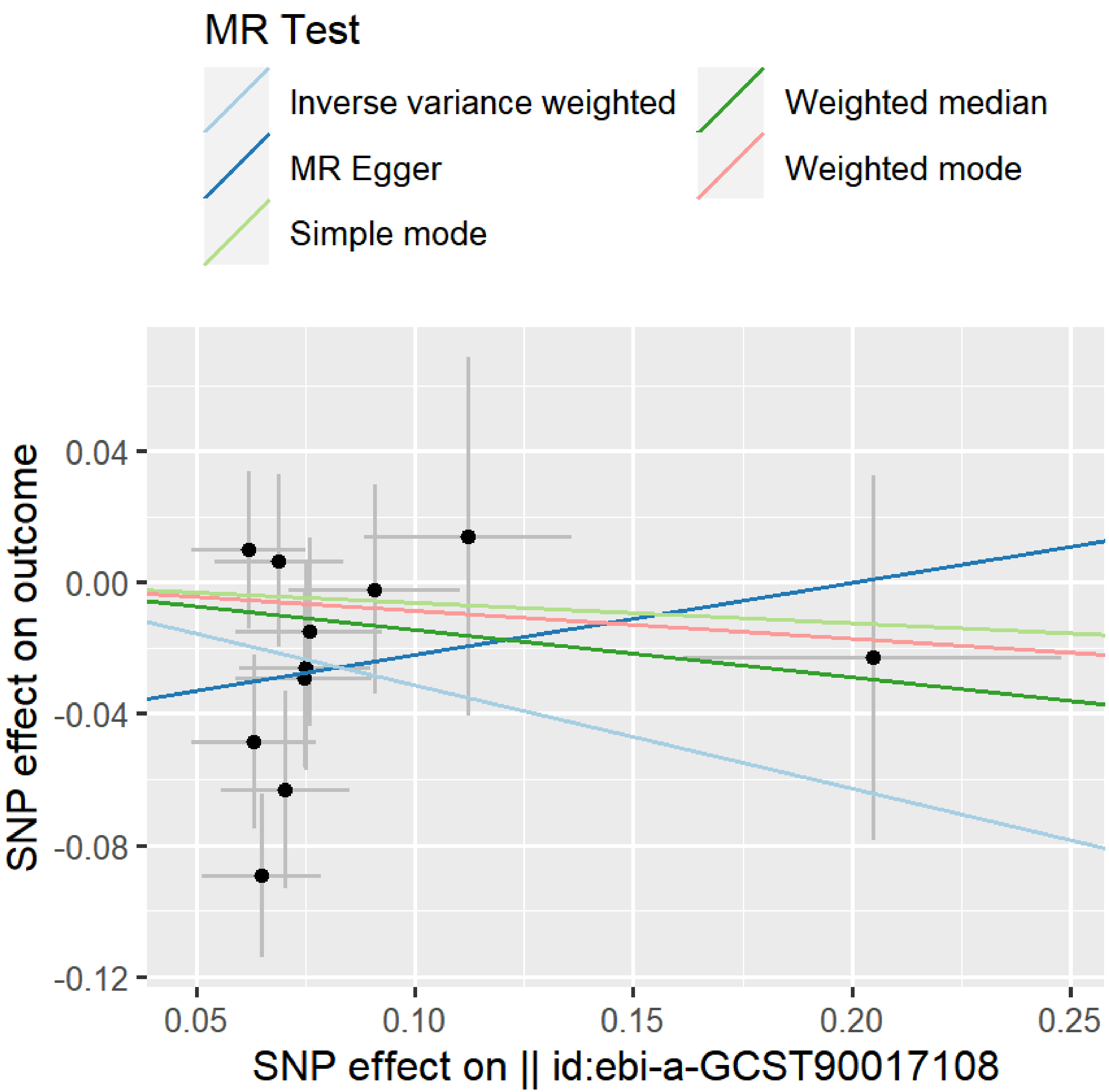
MR Test
Inverse variance weighted
MR Egger
Simple mode
Weighted median
Weighted mode
0.04
0.00
-0.04
-0.08
-0.12
SNP effect on outcome
0.05
0.10
0.15
0.20
0.25
SNP effect on || id:ebi-a-GCST90017108

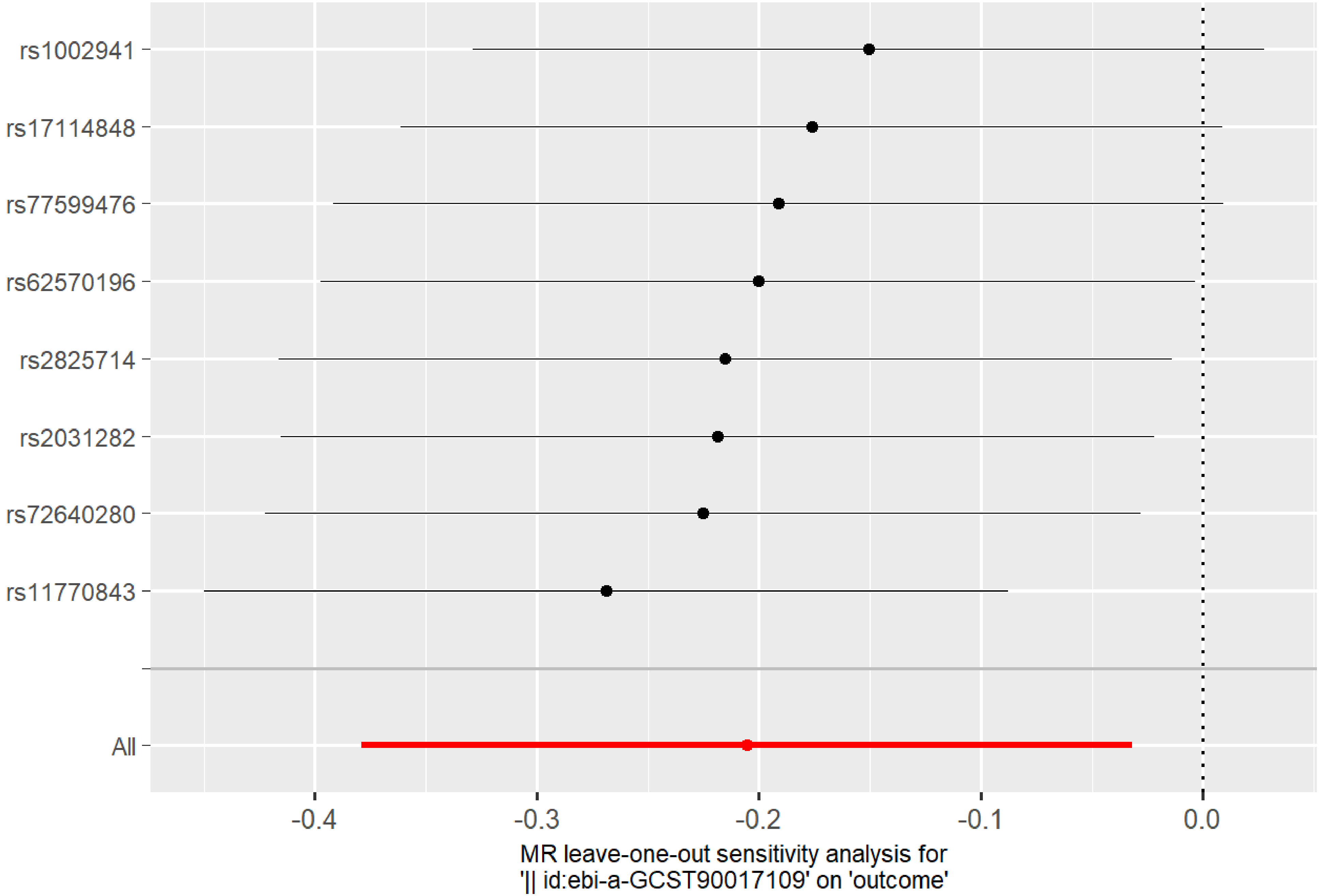
rs1002941
rs17114848
rs77599476
rs62570196
rs2825714
rs2031282
rs72640280
rs11770843
All
-0.4
-0.3
-0.2
-0.1
0.0
MR leave-one-out sensitivity analysis for
'|| id:ebi-a-GCST90017109' on 'outcome'

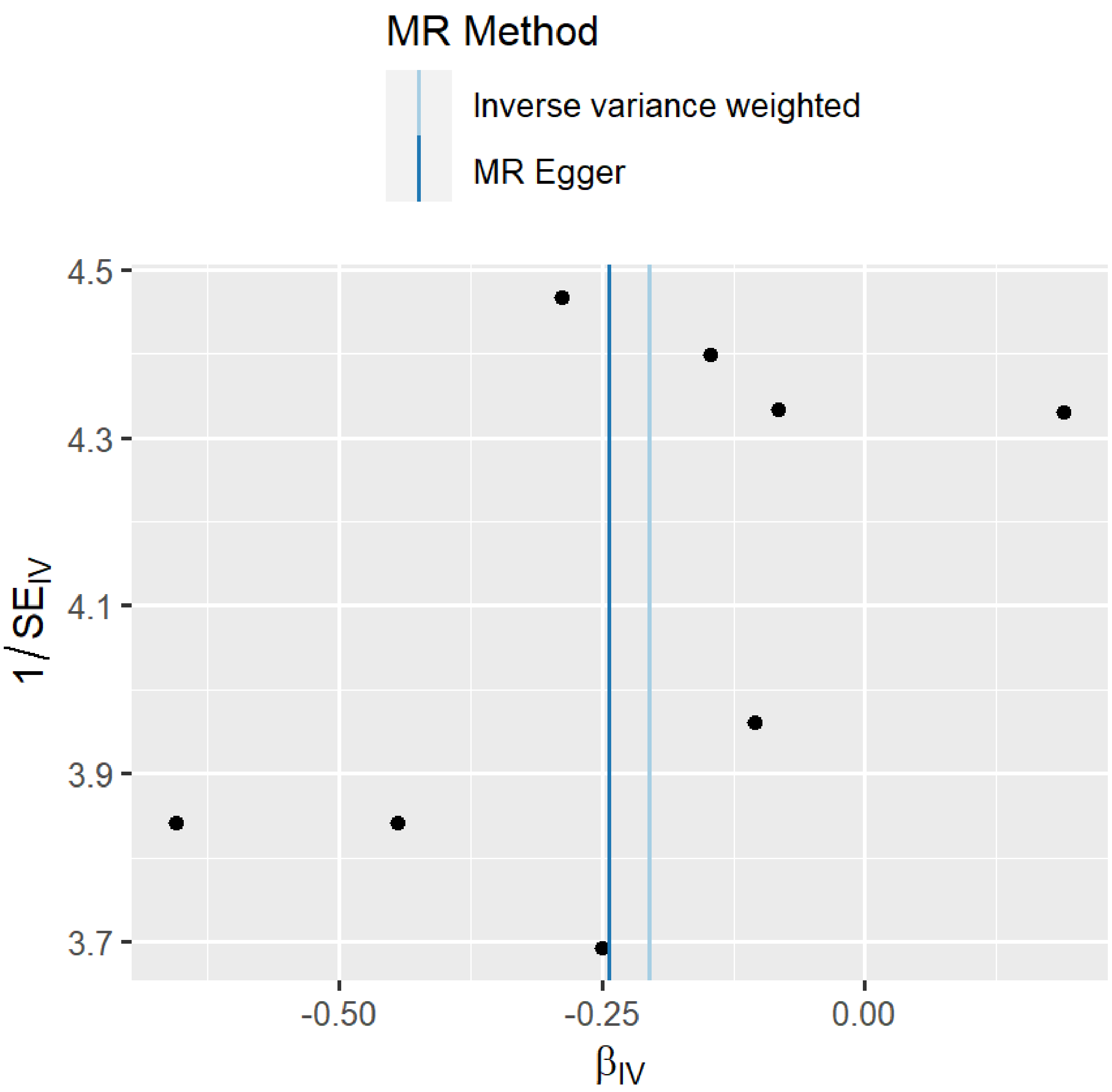

MR Method
Inverse variance weighted
MR Egger
4.5
4.3
4.1
3.9
3.7
1/SE$_{IV}$
-0.50
-0.25
0.00
β_{IV}

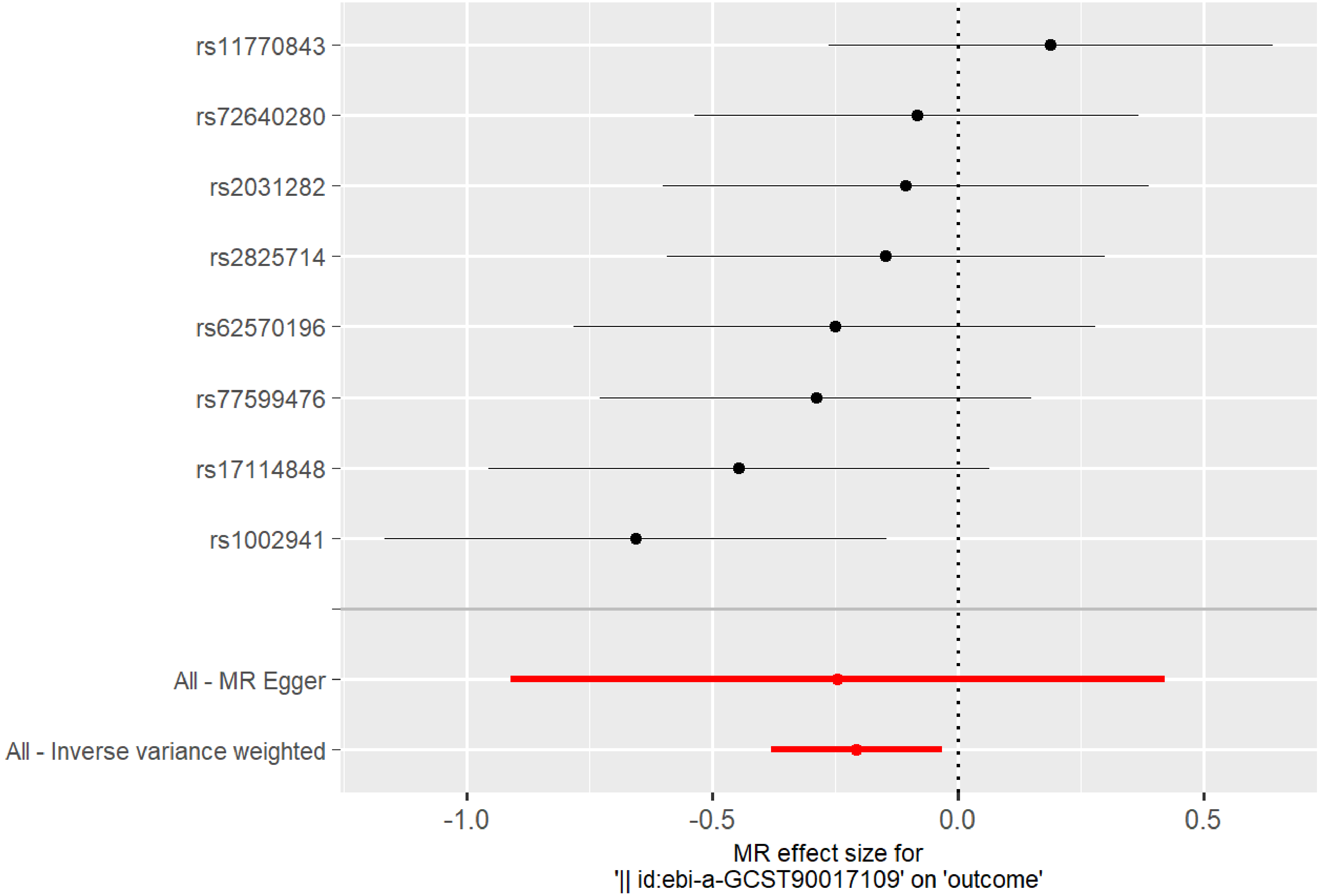
rs11770843
rs72640280
rs2031282
rs2825714
rs62570196
rs77599476
rs17114848
rs1002941
All - MR Egger
All - Inverse variance weighted
-1.0
-0.5
0.0
0.5
MR effect size for
'|| id:ebi-a-GCST90017109' on 'outcome'

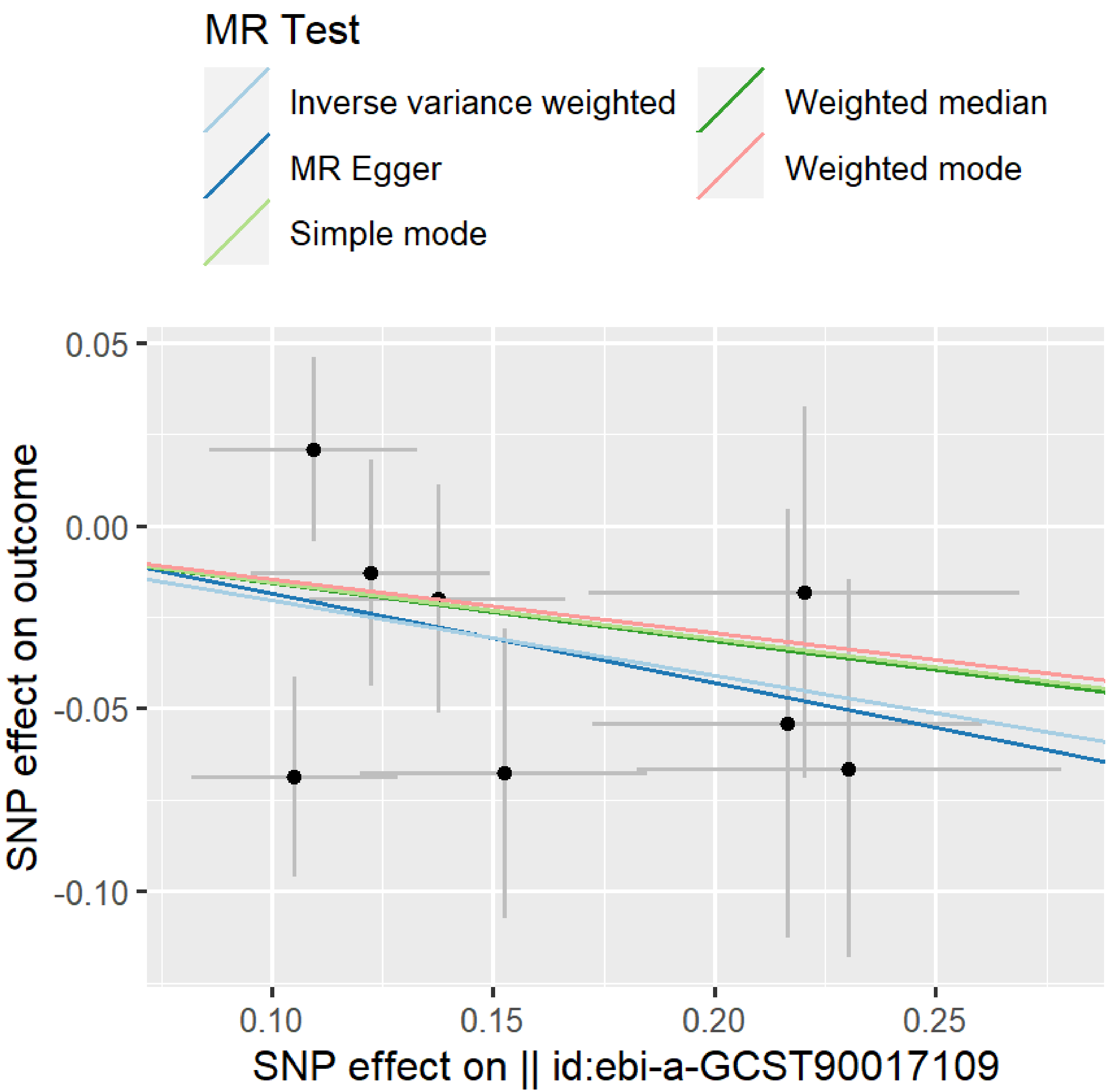
MR Test
Inverse variance weighted
MR Egger
Simple mode
Weighted median
Weighted mode
0.05
0.00
-0.05
-0.10
SNP effect on outcome
0.10
0.15
0.20
0.25
SNP effect on || id:ebi-a-GCST90017109

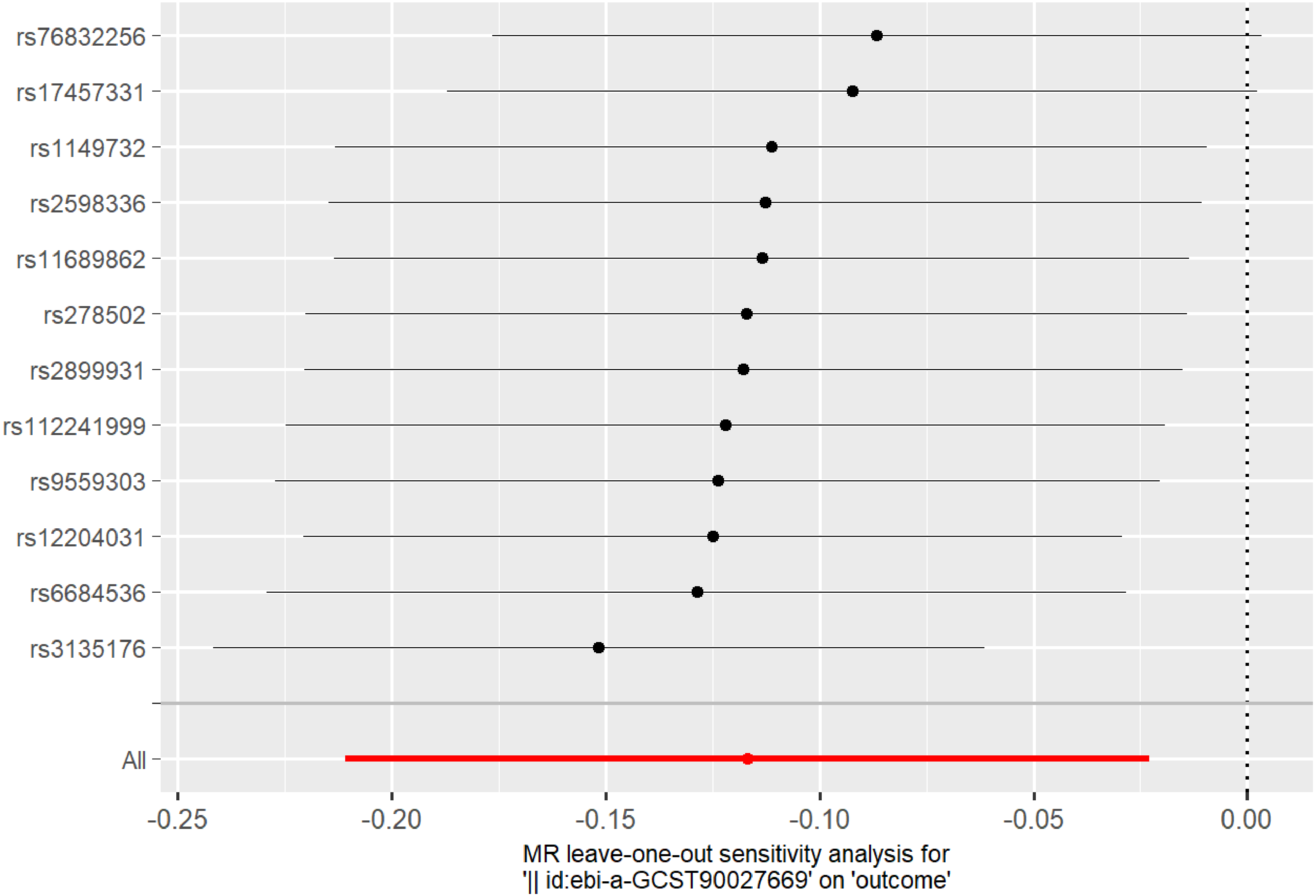
rs76832256
rs17457331
rs1149732
rs2598336
rs11689862
rs278502
rs2899931
rs112241999
rs9559303
rs12204031
rs6684536
rs3135176
All
-0.25
-0.20
-0.15
-0.10
-0.05
0.00
MR leave-one-out sensitivity analysis for
'|| id:ebi-a-GCST90027669' on 'outcome'

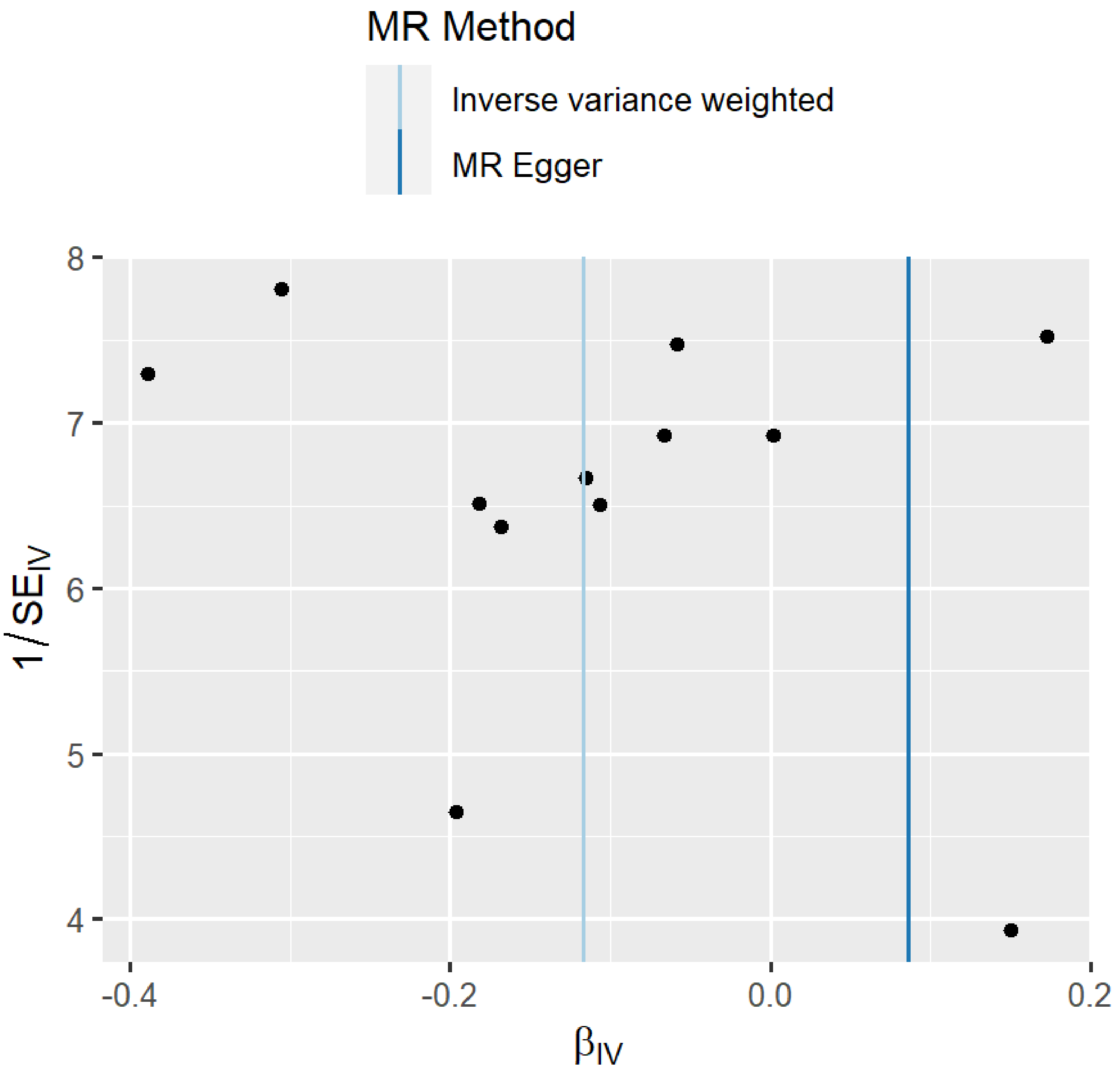

MR Method
Inverse variance weighted
MR Egger
8
7
6
5
4
1/SE_IV
-0.4
-0.2
0.0
0.2
β_IV

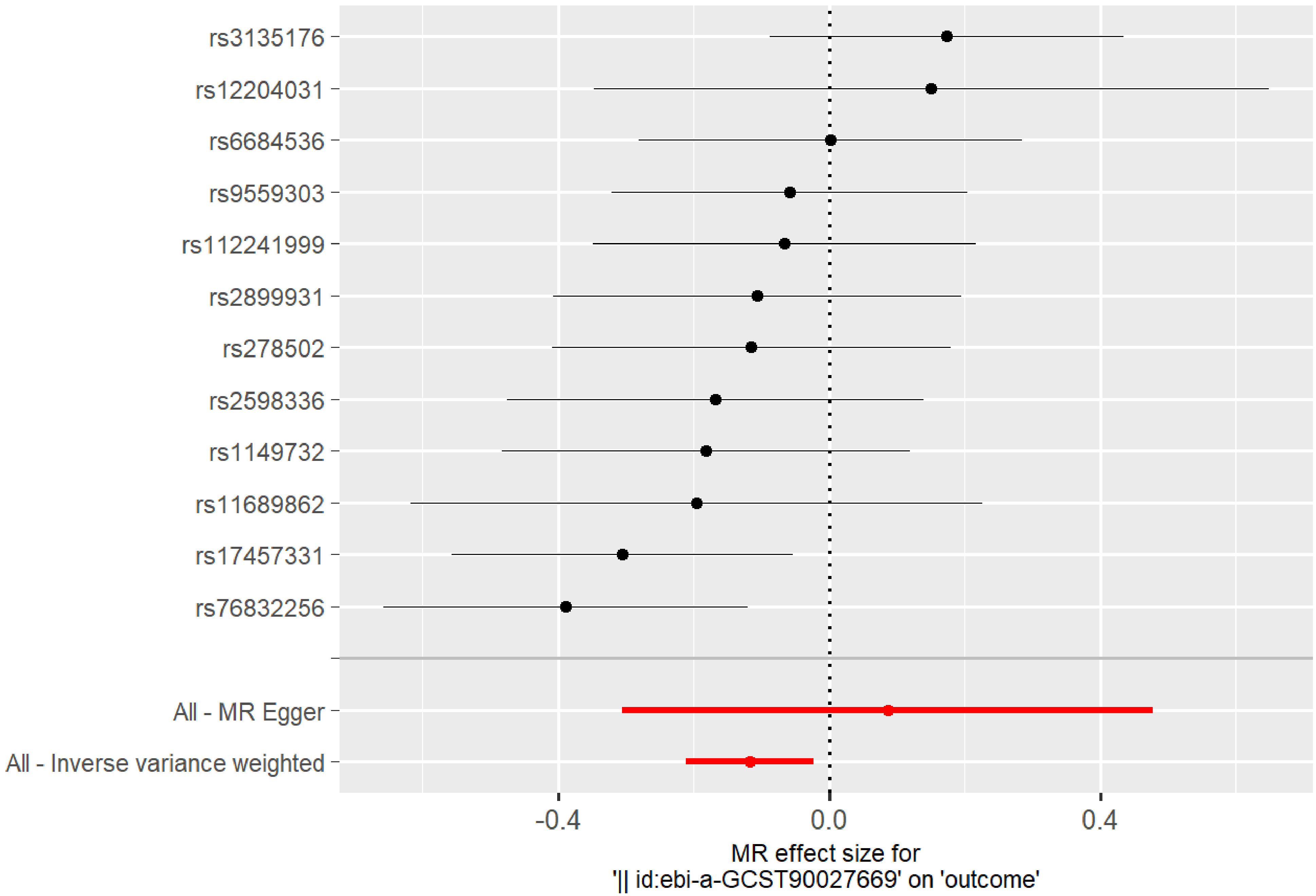
rs3135176
rs12204031
rs6684536
rs9559303
rs112241999
rs2899931
rs278502
rs2598336
rs1149732
rs11689862
rs17457331
rs76832256
All - MR Egger
All - Inverse variance weighted
-0.4
0.0
0.4
MR effect size for
'|| id:ebi-a-GCST90027669' on 'outcome'

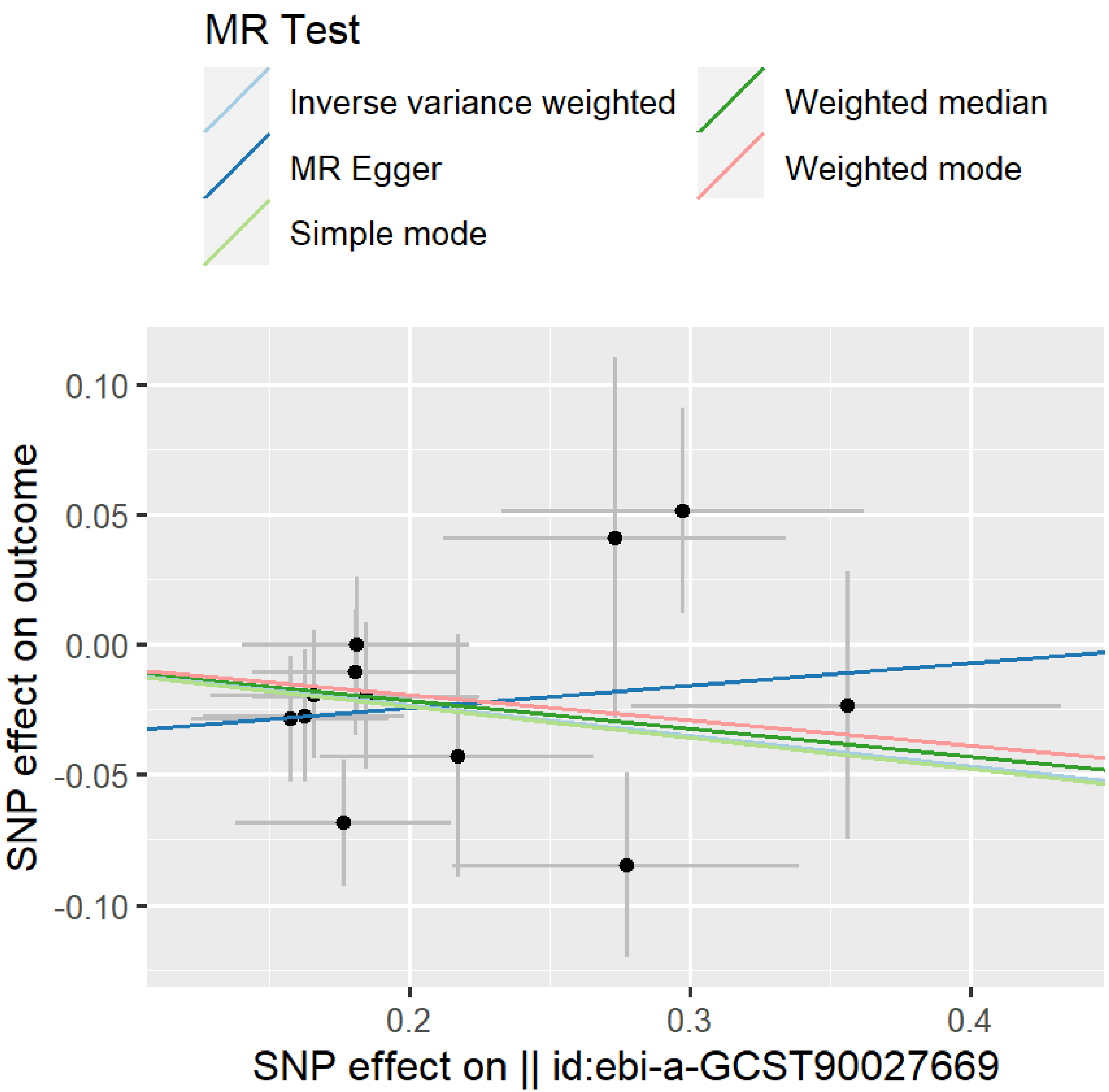
MR Test
Inverse variance weighted
MR Egger
Simple mode
Weighted median
Weighted mode
0.10
0.05
0.00
-0.05
-0.10
SNP effect on outcome
0.2
0.3
0.4
SNP effect on || id:ebi-a-GCST90027669

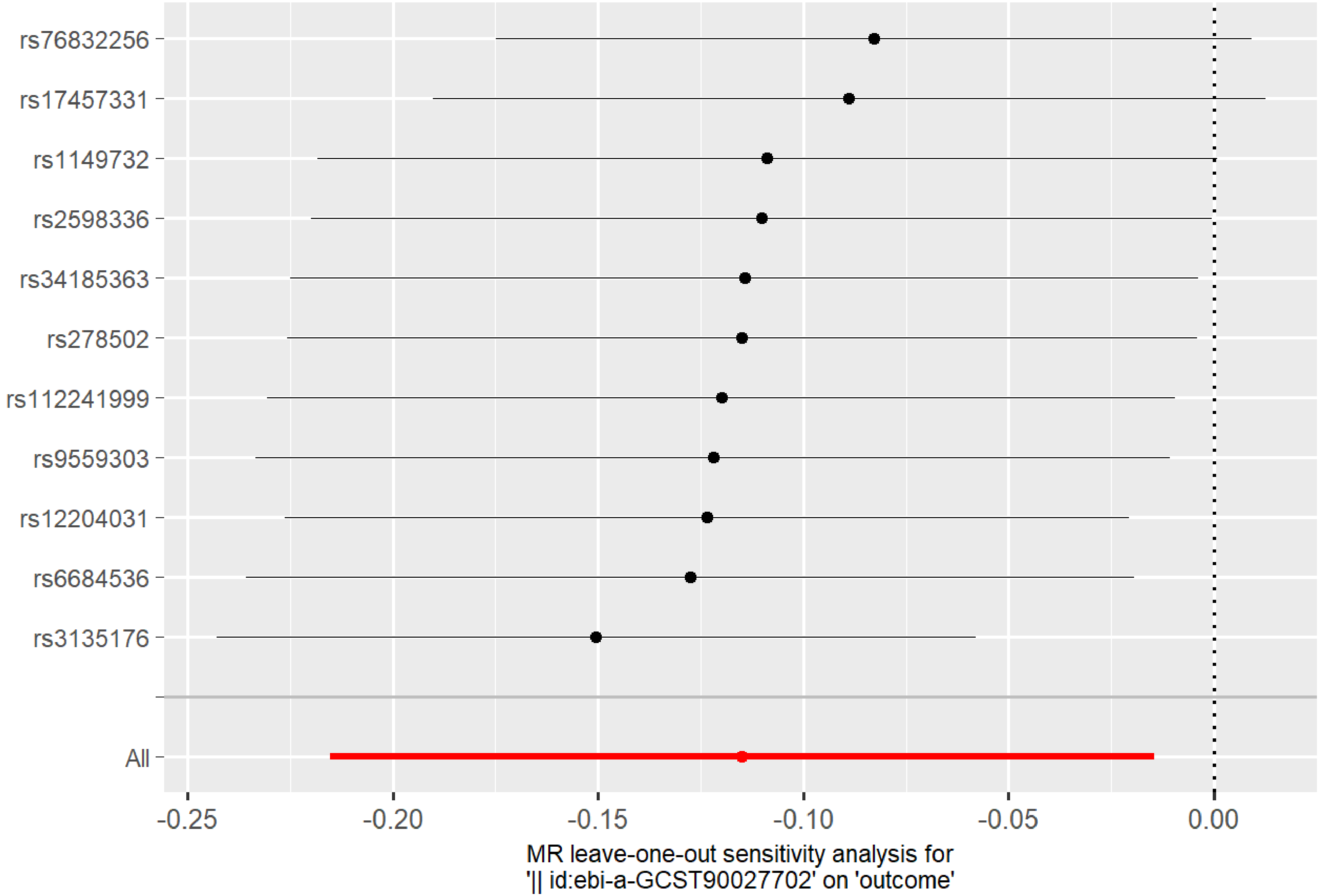
rs76832256
rs17457331
rs1149732
rs2598336
rs34185363
rs278502
rs112241999
rs9559303
rs12204031
rs6684536
rs3135176
All
-0.25
-0.20
-0.15
-0.10
-0.05
0.00
MR leave-one-out sensitivity analysis for
'|| id:ebi-a-GCST90027702' on 'outcome'

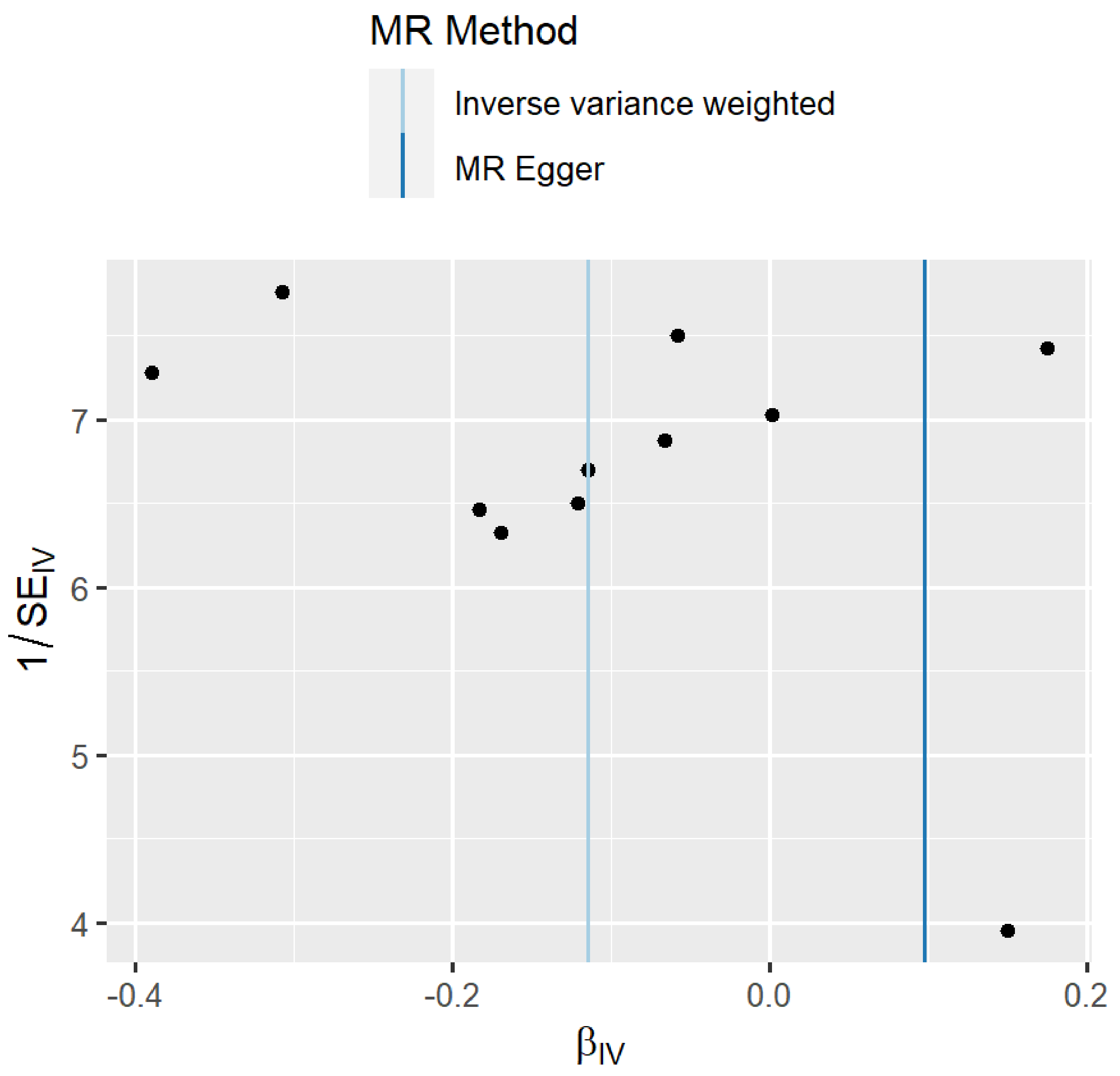
MR Method
Inverse variance weighted
MR Egger
7
6
5
4
1/SE_IV
-0.4
-0.2
0.0
0.2
β_IV

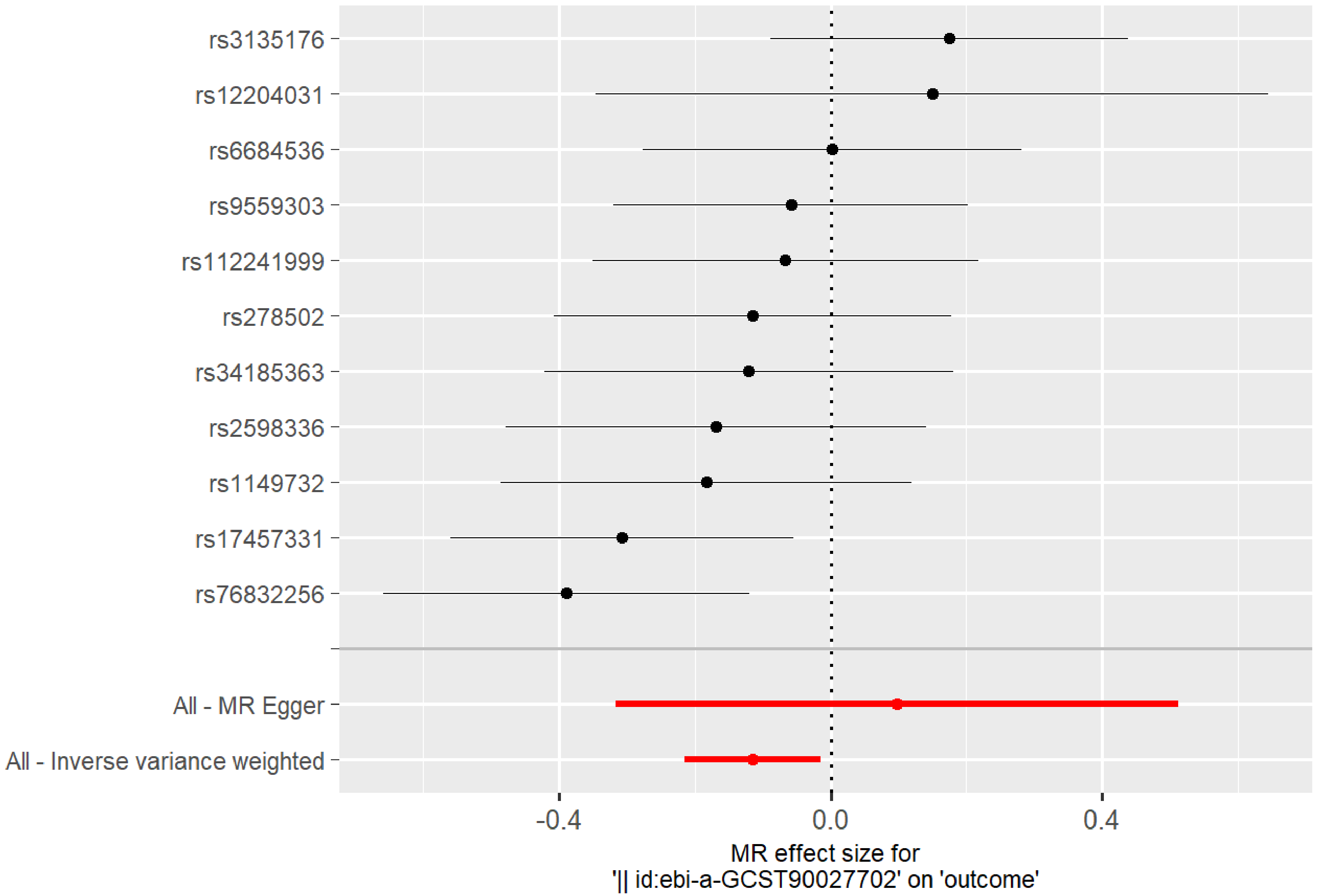
rs3135176
rs12204031
rs6684536
rs9559303
rs112241999
rs278502
rs34185363
rs2598336
rs1149732
rs17457331
rs76832256
All - MR Egger
All - Inverse variance weighted
-0.4
0.0
0.4
MR effect size for
'|| id:ebi-a-GCST90027702' on 'outcome'

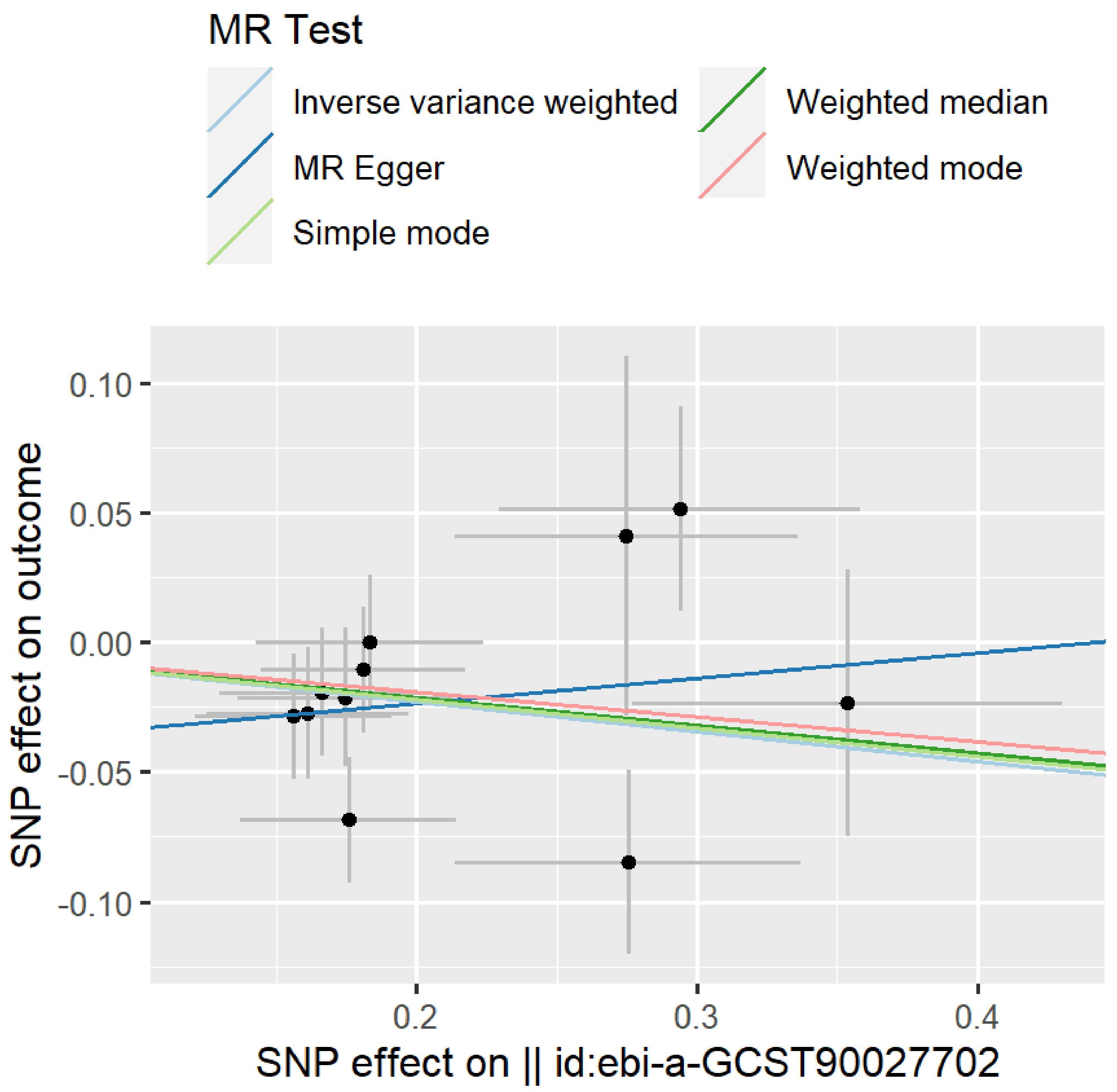
MR Test
Inverse variance weighted
MR Egger
Simple mode
Weighted median
Weighted mode
0.10
0.05
0.00
-0.05
-0.10
SNP effect on outcome
0.2
0.3
0.4
SNP effect on || id:ebi-a-GCST90027702

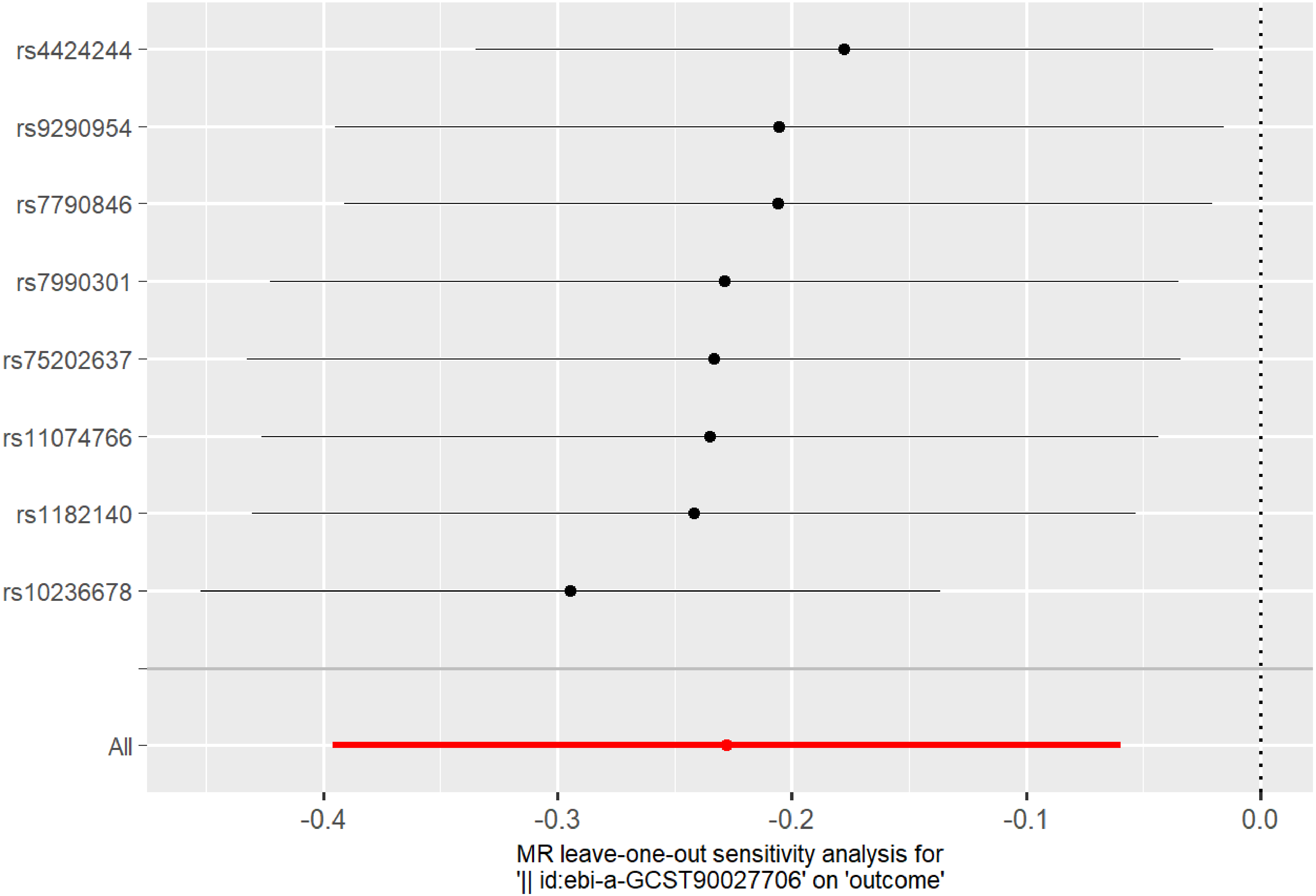
rs4424244
rs9290954
rs7790846
rs7990301
rs75202637
rs11074766
rs1182140
rs10236678
All
-0.4
-0.3
-0.2
-0.1
0.0
MR leave-one-out sensitivity analysis for
'|| id:ebi-a-GCST90027706' on 'outcome'

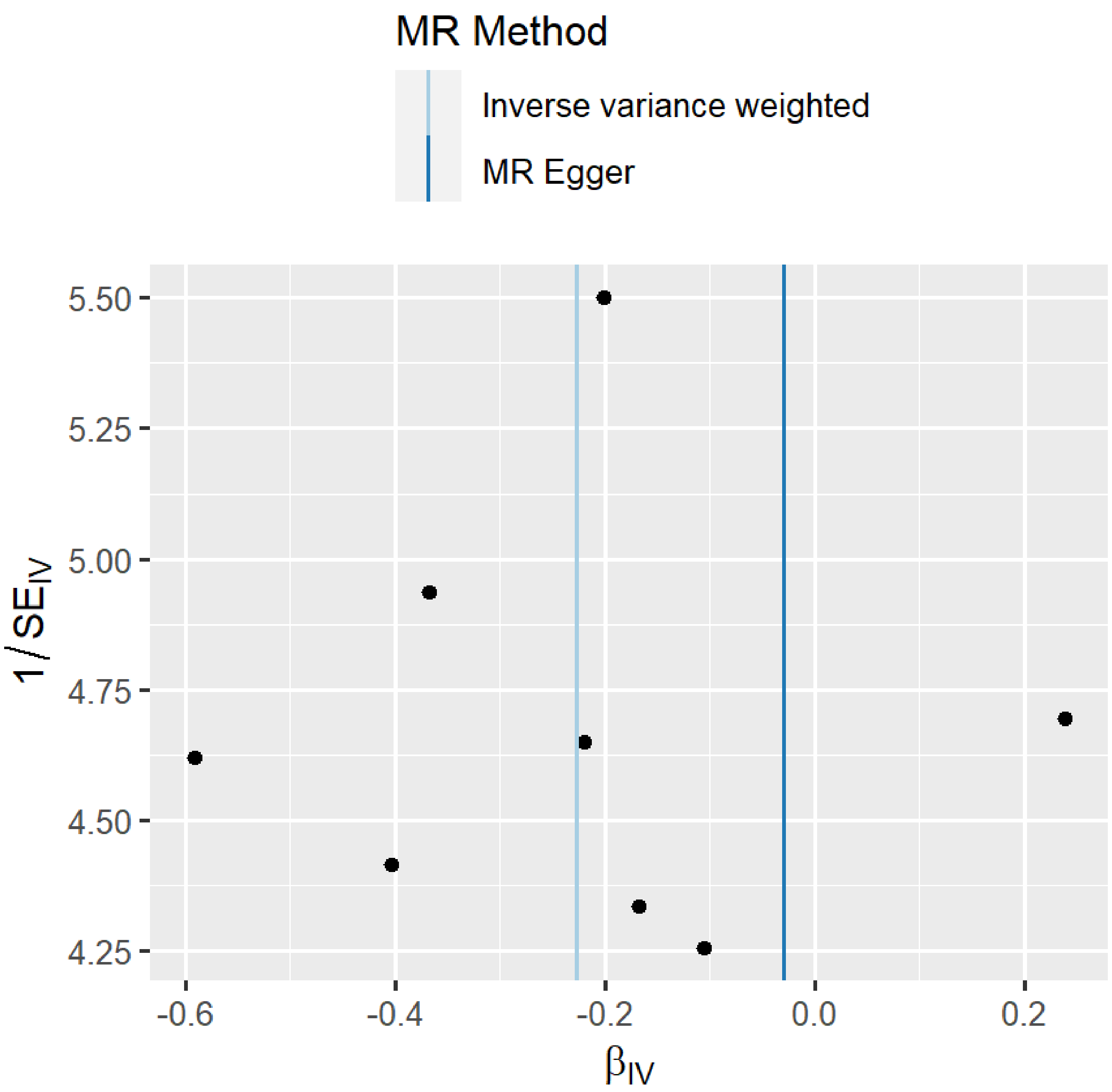
MR Method
Inverse variance weighted
MR Egger
5.50
5.25
5.00
4.75
4.50
4.25
1/SE_IV
-0.6
-0.4
-0.2
0.0
0.2
β_IV

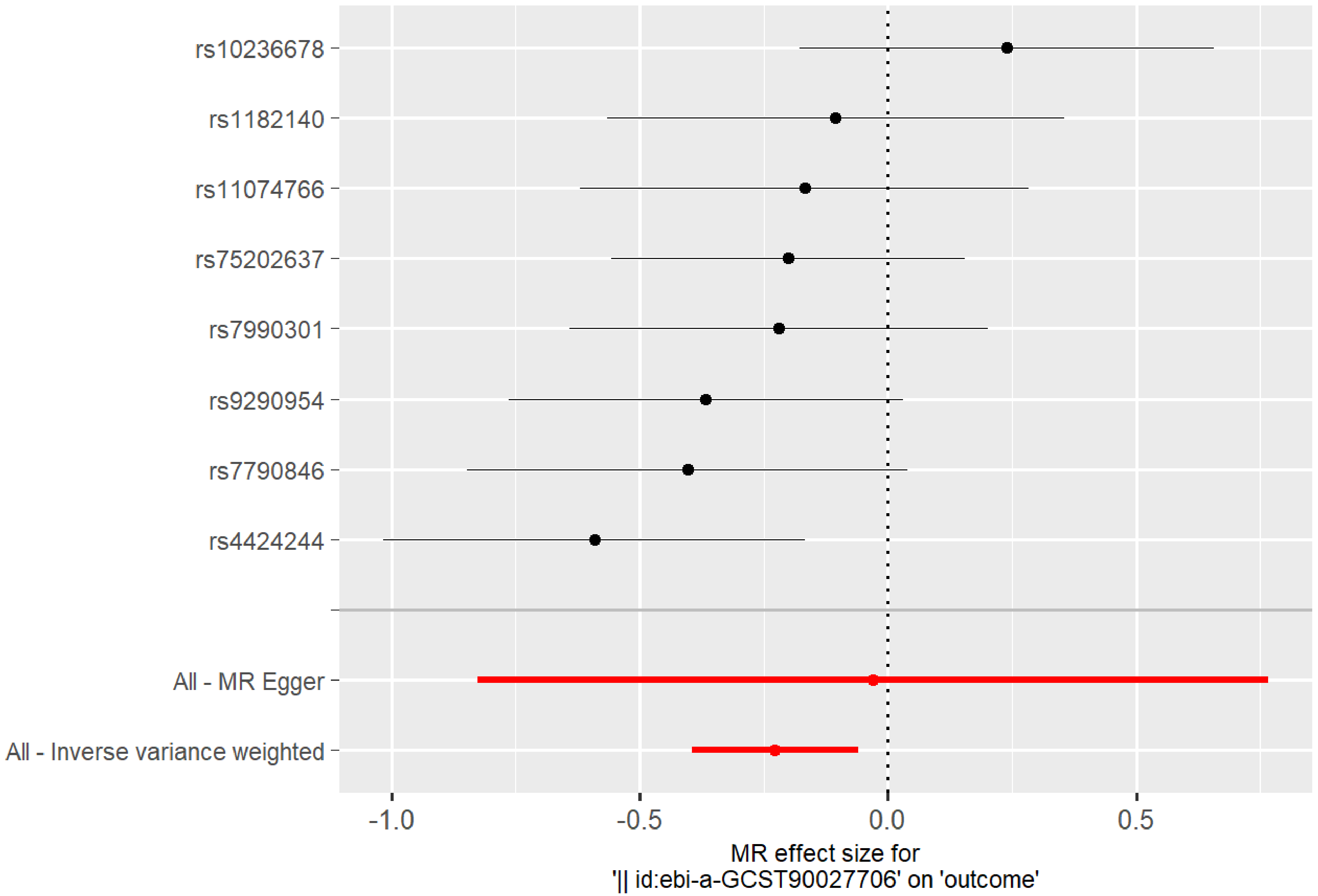

rs10236678
rs1182140
rs11074766
rs75202637
rs7990301
rs9290954
rs7790846
rs4424244
All - MR Egger
All - Inverse variance weighted
-1.0
-0.5
0.0
0.5
MR effect size for
'|| id:ebi-a-GCST90027706' on 'outcome'

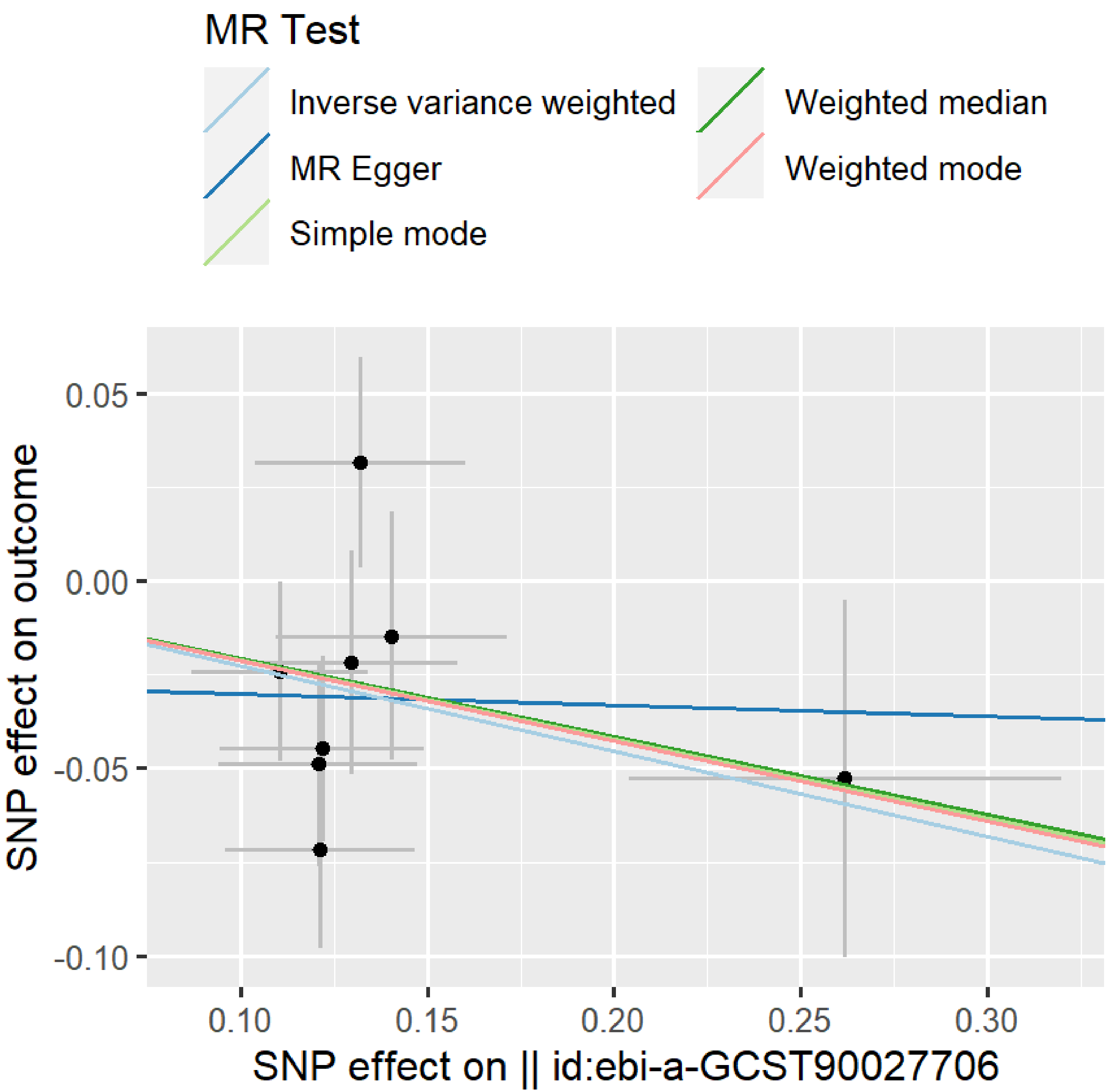
MR Test
Inverse variance weighted
MR Egger
Simple mode
Weighted median
Weighted mode
0.05
0.00
-0.05
-0.10
SNP effect on outcome
0.10
0.15
0.20
0.25
0.30
SNP effect on || id:ebi-a-GCST90027706

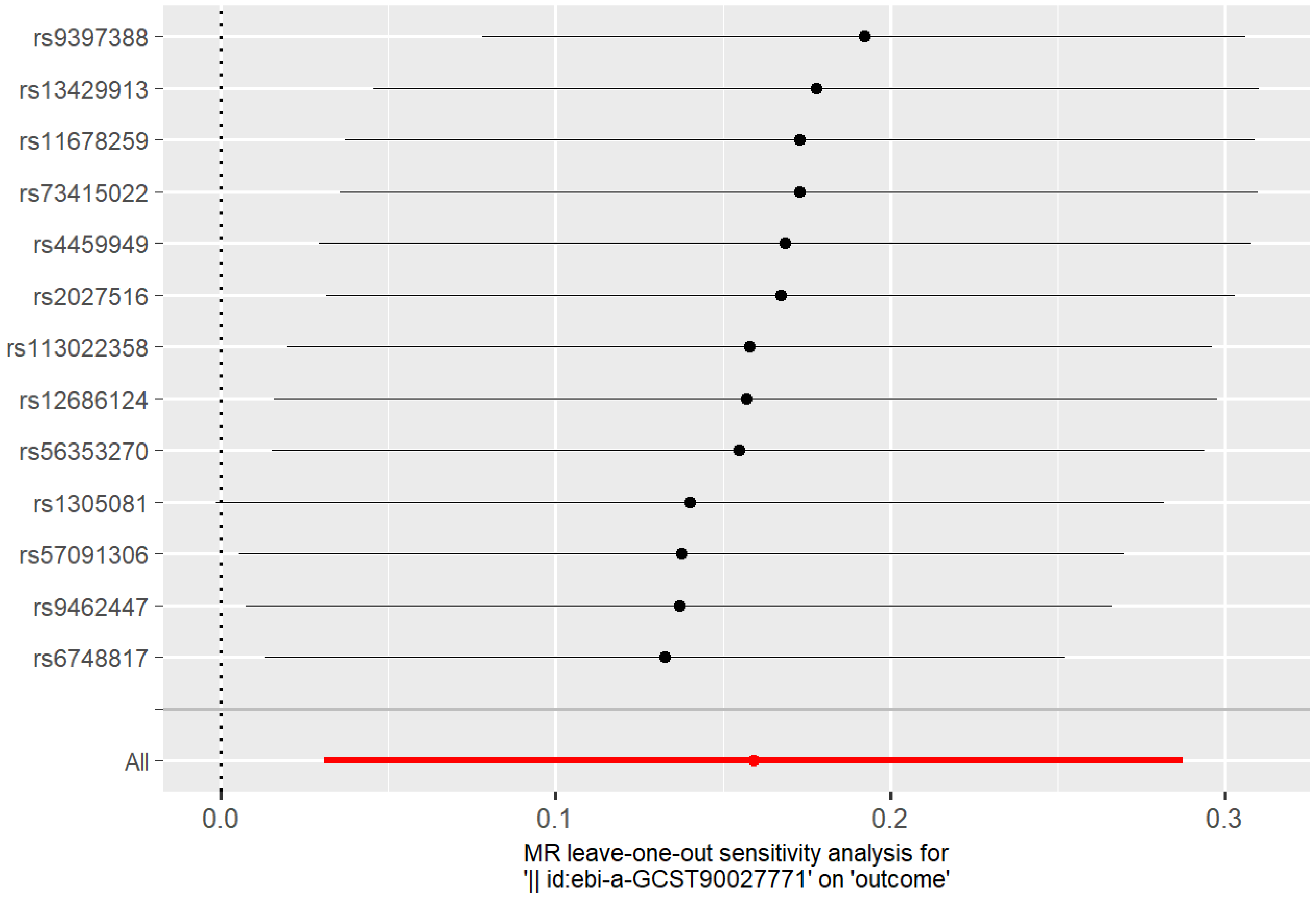

rs9397388
rs13429913
rs11678259
rs73415022
rs4459949
rs2027516
rs113022358
rs12686124
rs56353270
rs1305081
rs57091306
rs9462447
rs6748817
All
0.0
0.1
0.2
0.3
MR leave-one-out sensitivity analysis for
'|| id:ebi-a-GCST90027771' on 'outcome'

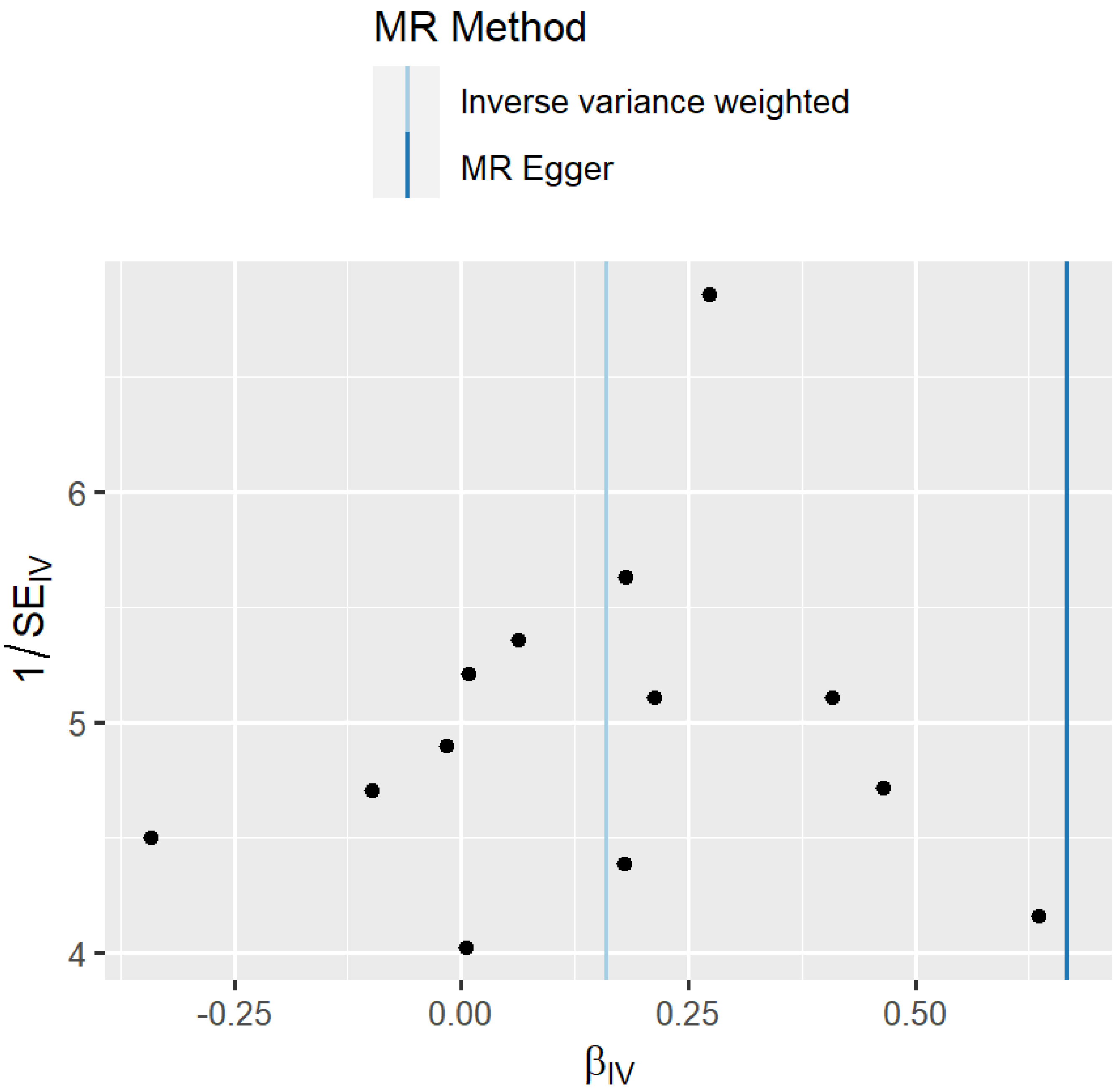

MR Method
Inverse variance weighted
MR Egger
6
5
4
1/SE_IV
-0.25
0.00
0.25
0.50
β_IV

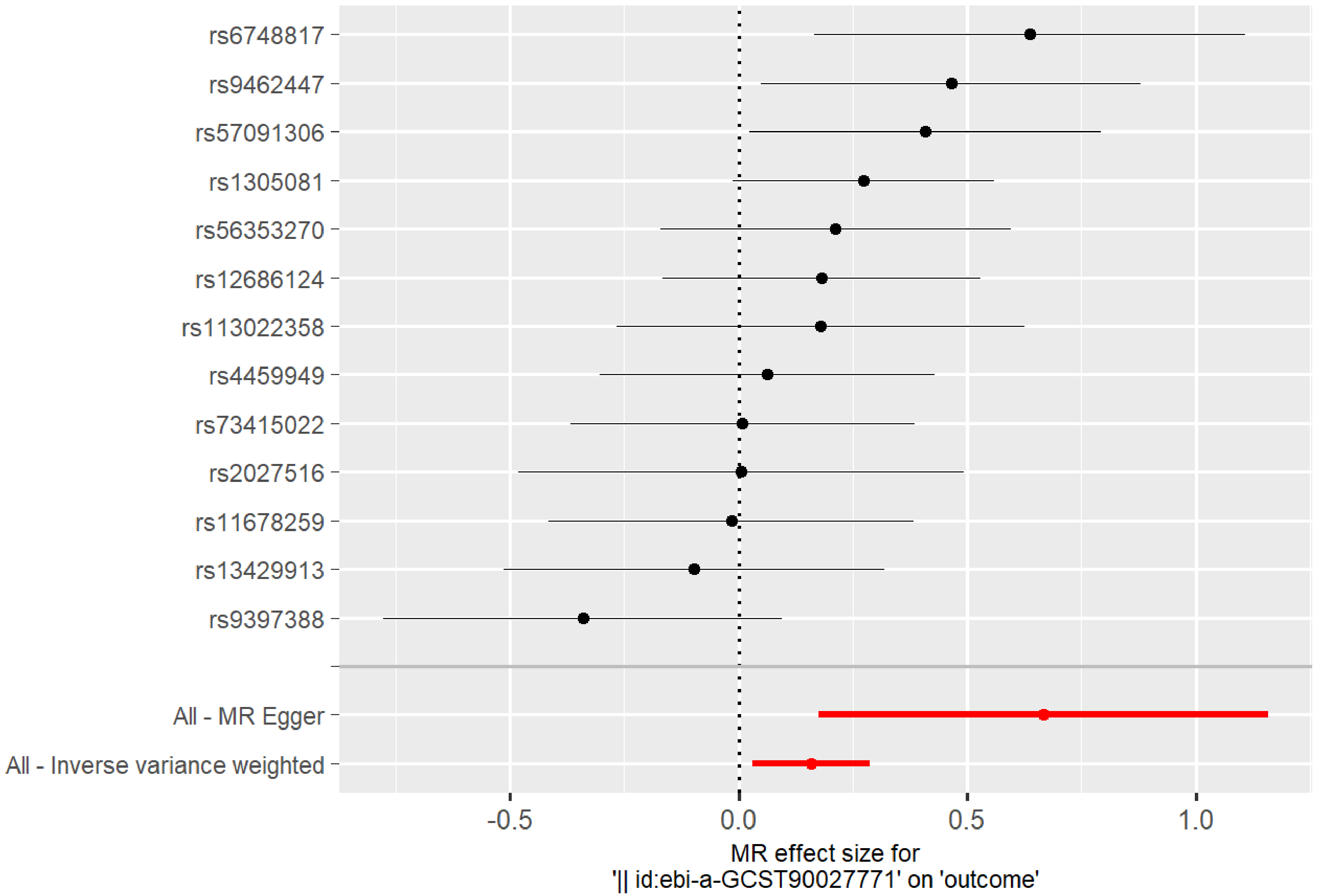
rs6748817
rs9462447
rs57091306
rs1305081
rs56353270
rs12686124
rs113022358
rs4459949
rs73415022
rs2027516
rs11678259
rs13429913
rs9397388
All - MR Egger
All - Inverse variance weighted
-0.5
0.0
0.5
1.0
MR effect size for
'|| id:ebi-a-GCST90027771' on 'outcome'

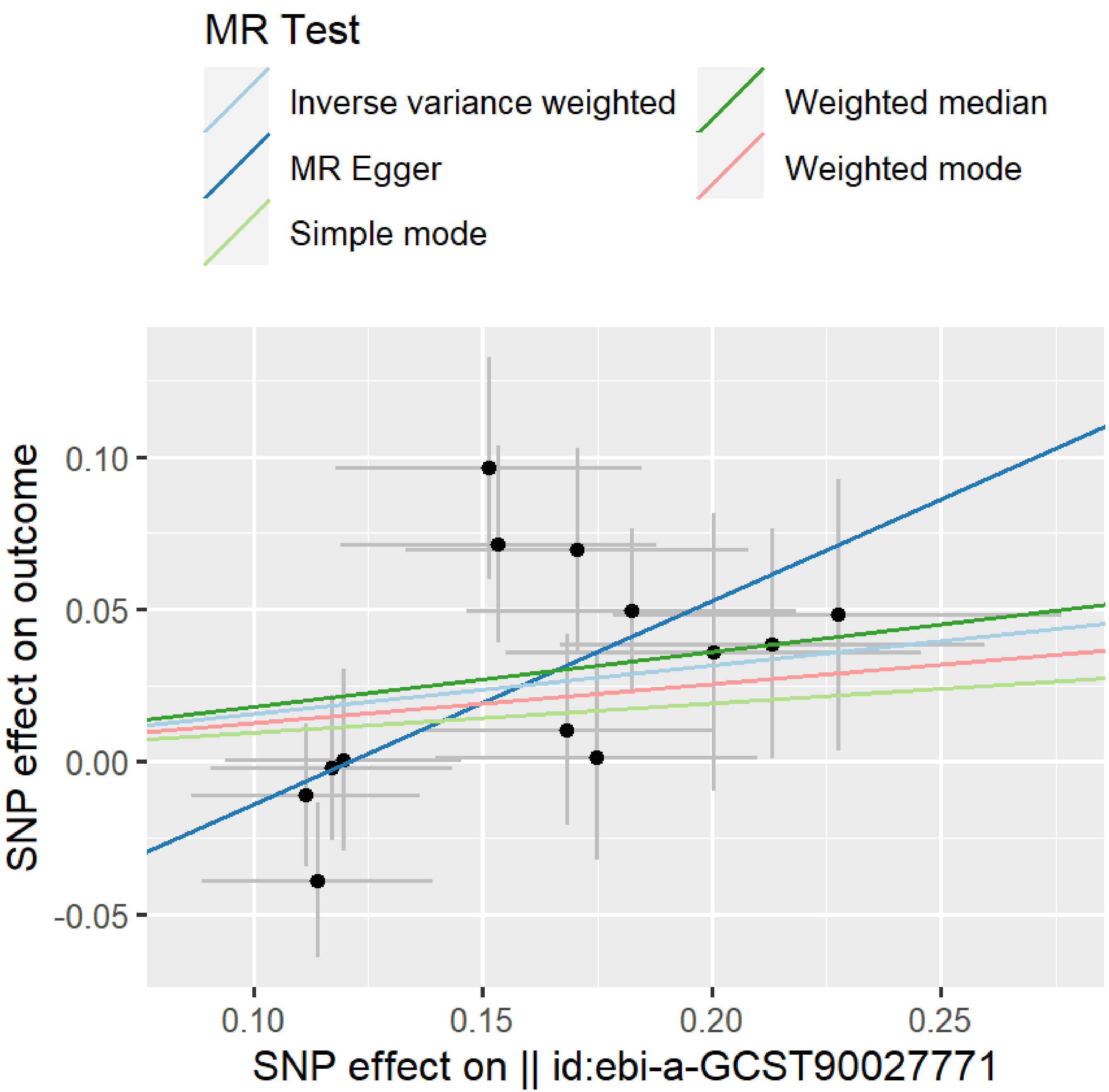
MR Test
Inverse variance weighted
MR Egger
Simple mode
Weighted median
Weighted mode
SNP effect on outcome
0.10
0.05
0.00
-0.05
0.10
0.15
0.20
0.25
SNP effect on || id:ebi-a-GCST90027771

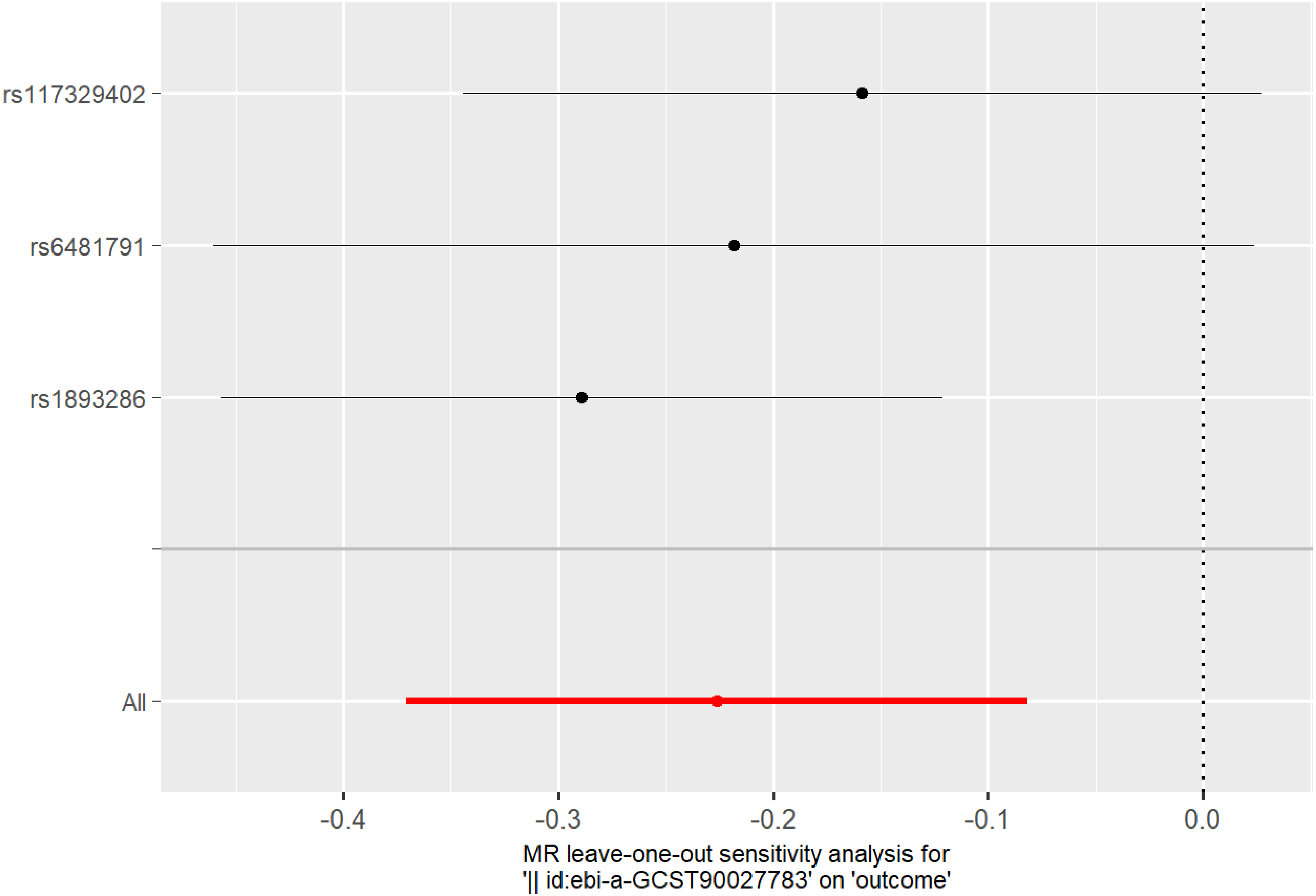
rs117329402
rs6481791
rs1893286
All
-0.4
-0.3
-0.2
-0.1
0.0
MR leave-one-out sensitivity analysis for
'|| id:ebi-a-GCST90027783' on 'outcome'

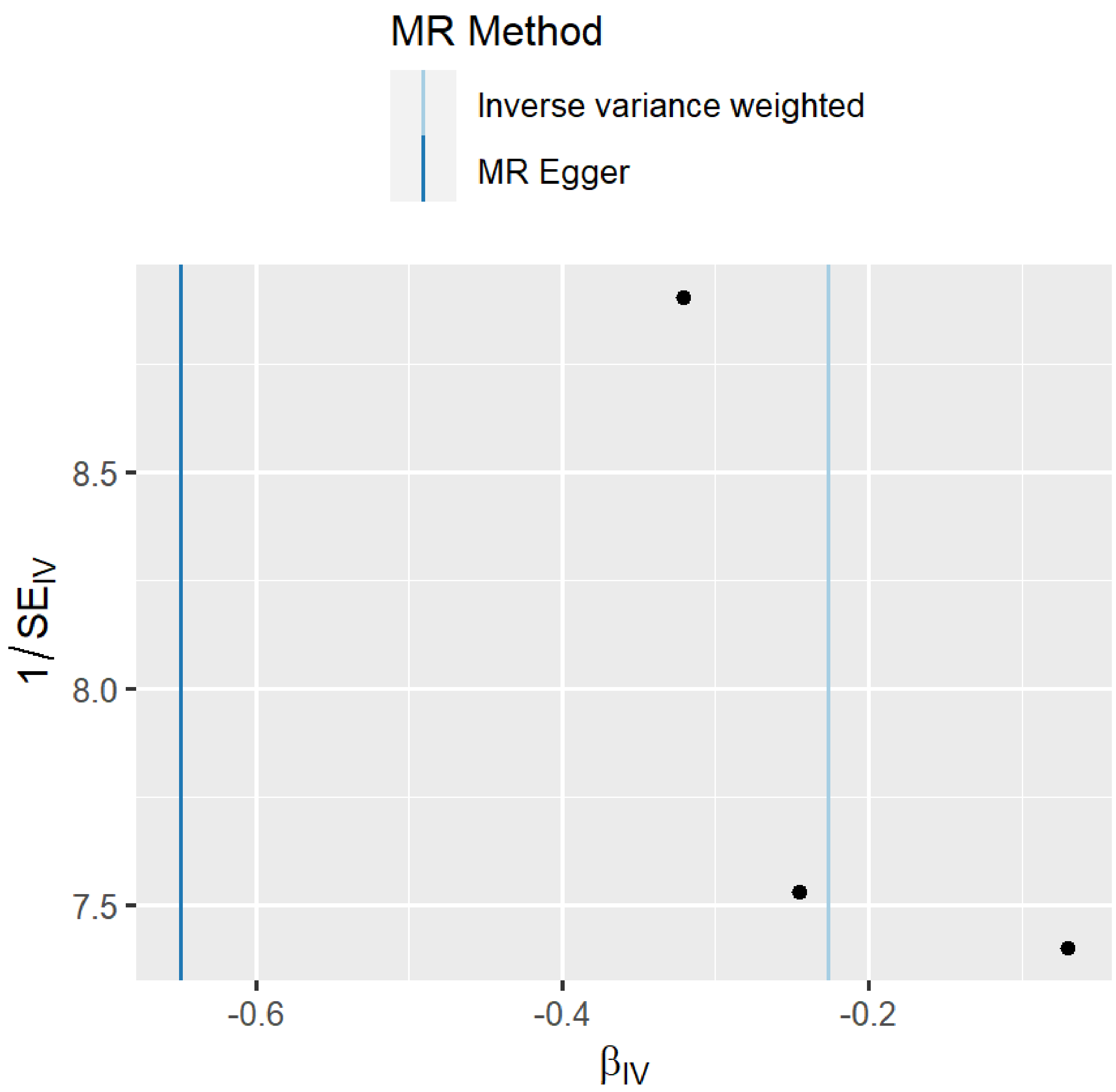

MR Method
Inverse variance weighted
MR Egger
8.5
8.0
7.5
1/SE_IV
-0.6
-0.4
-0.2
β_IV

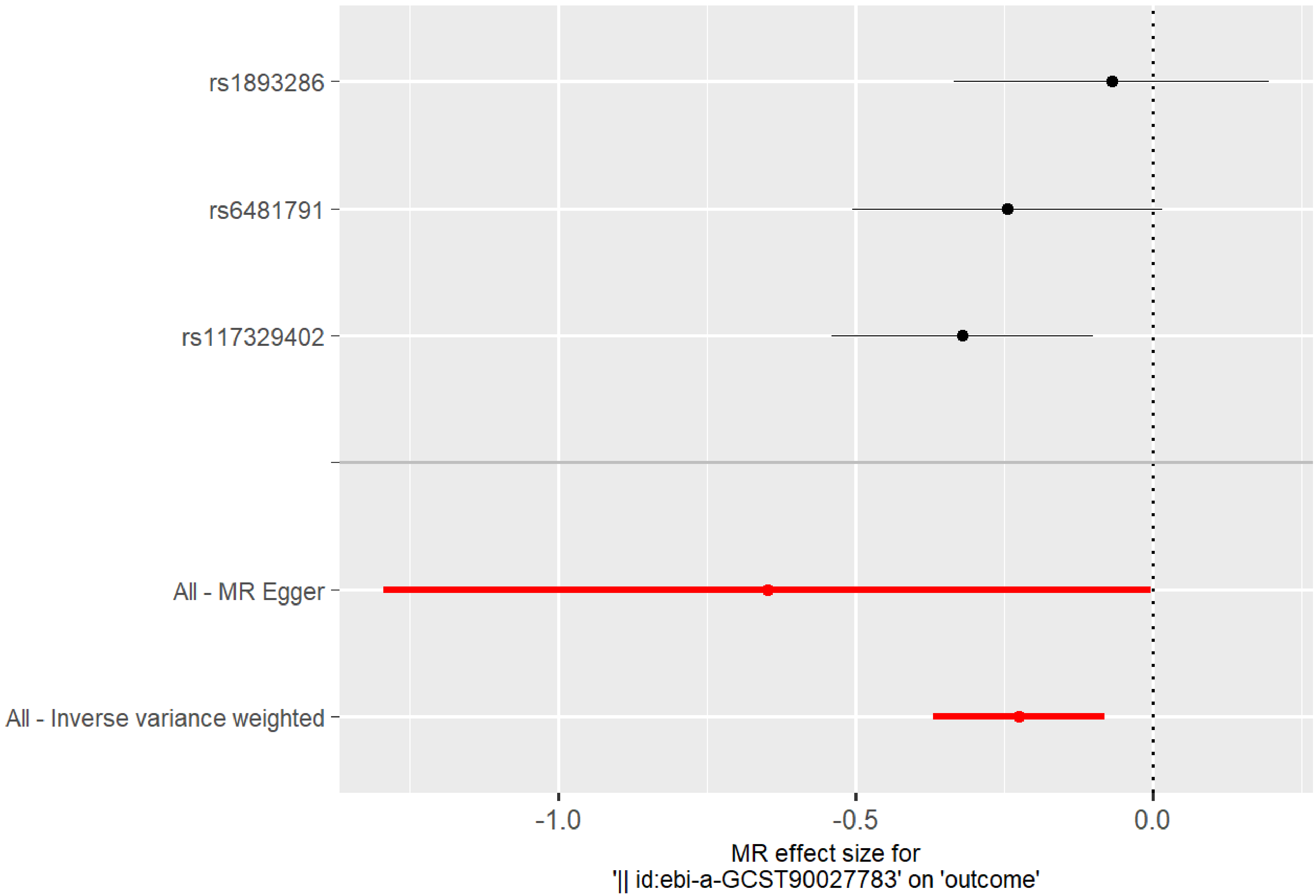
rs1893286
rs6481791
rs117329402
All - MR Egger
All - Inverse variance weighted
-1.0
-0.5
0.0
MR effect size for
'|| id:ebi-a-GCST90027783' on 'outcome'

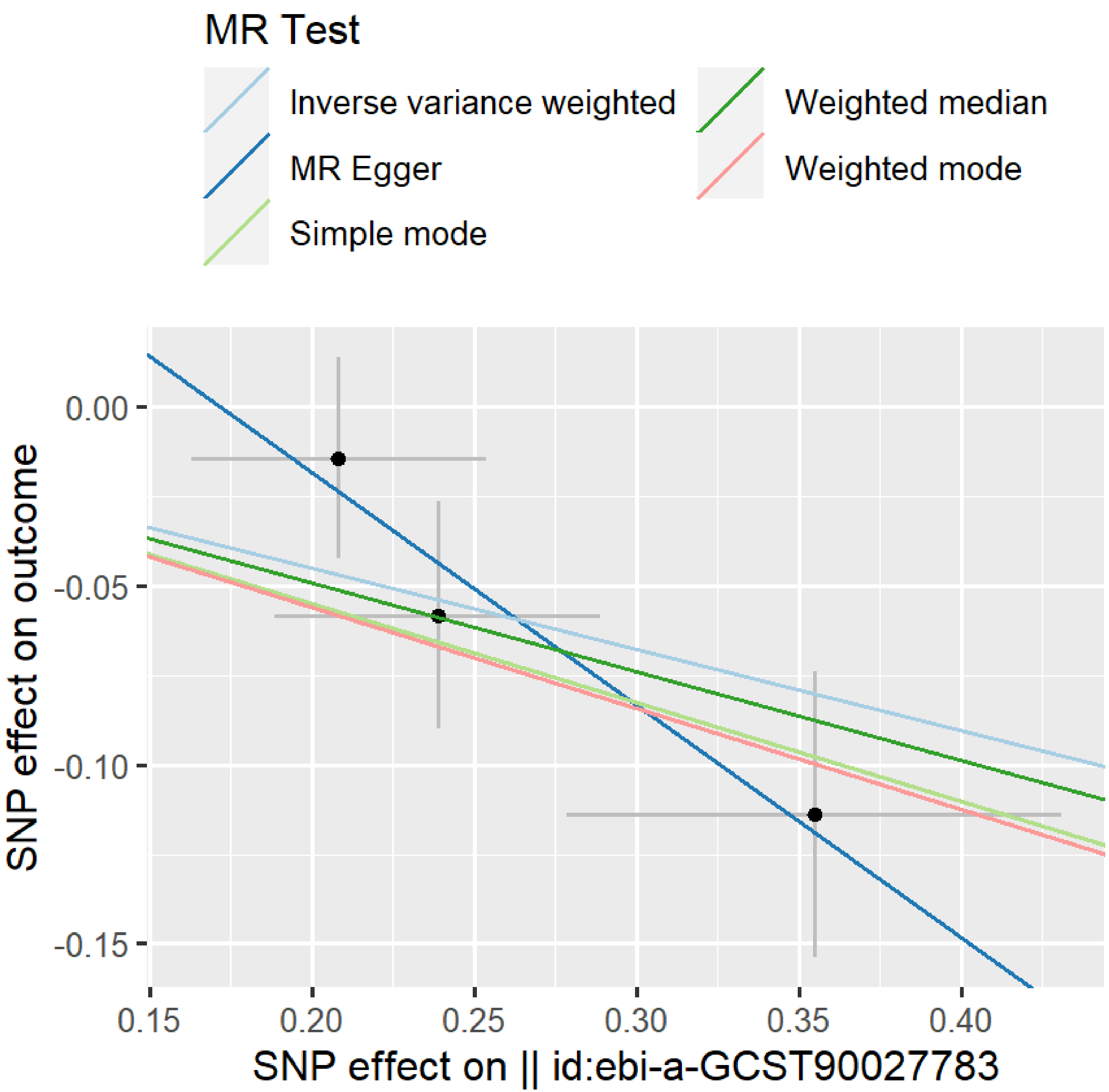
MR Test
Inverse variance weighted
MR Egger
Simple mode
Weighted median
Weighted mode
0.00
-0.05
-0.10
-0.15
SNP effect on outcome
0.15
0.20
0.25
0.30
0.35
0.40
SNP effect on || id:ebi-a-GCST90027783

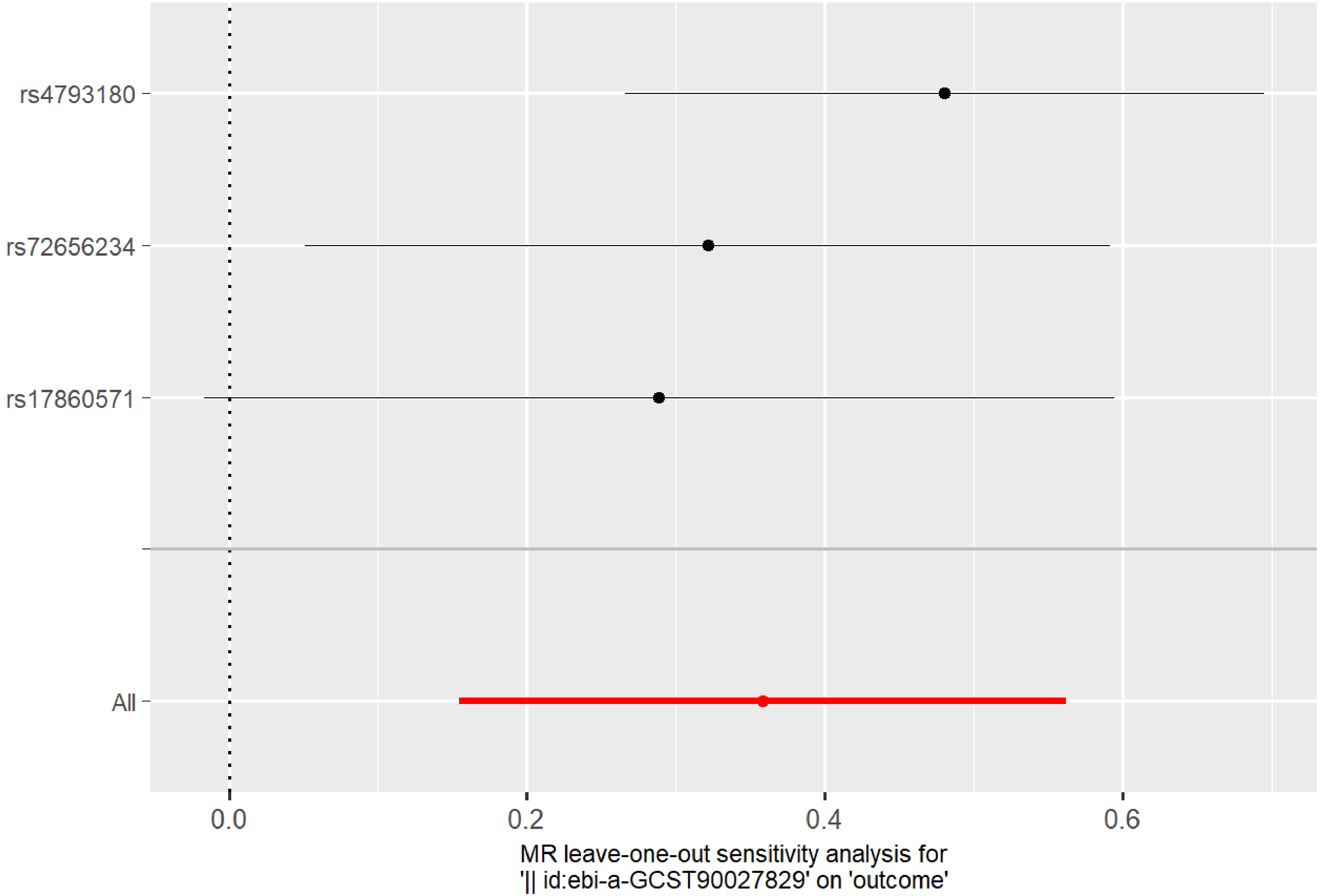
rs4793180
rs72656234
rs17860571
All
0.0
0.2
0.4
0.6
MR leave-one-out sensitivity analysis for
'|| id:ebi-a-GCST90027829' on 'outcome'

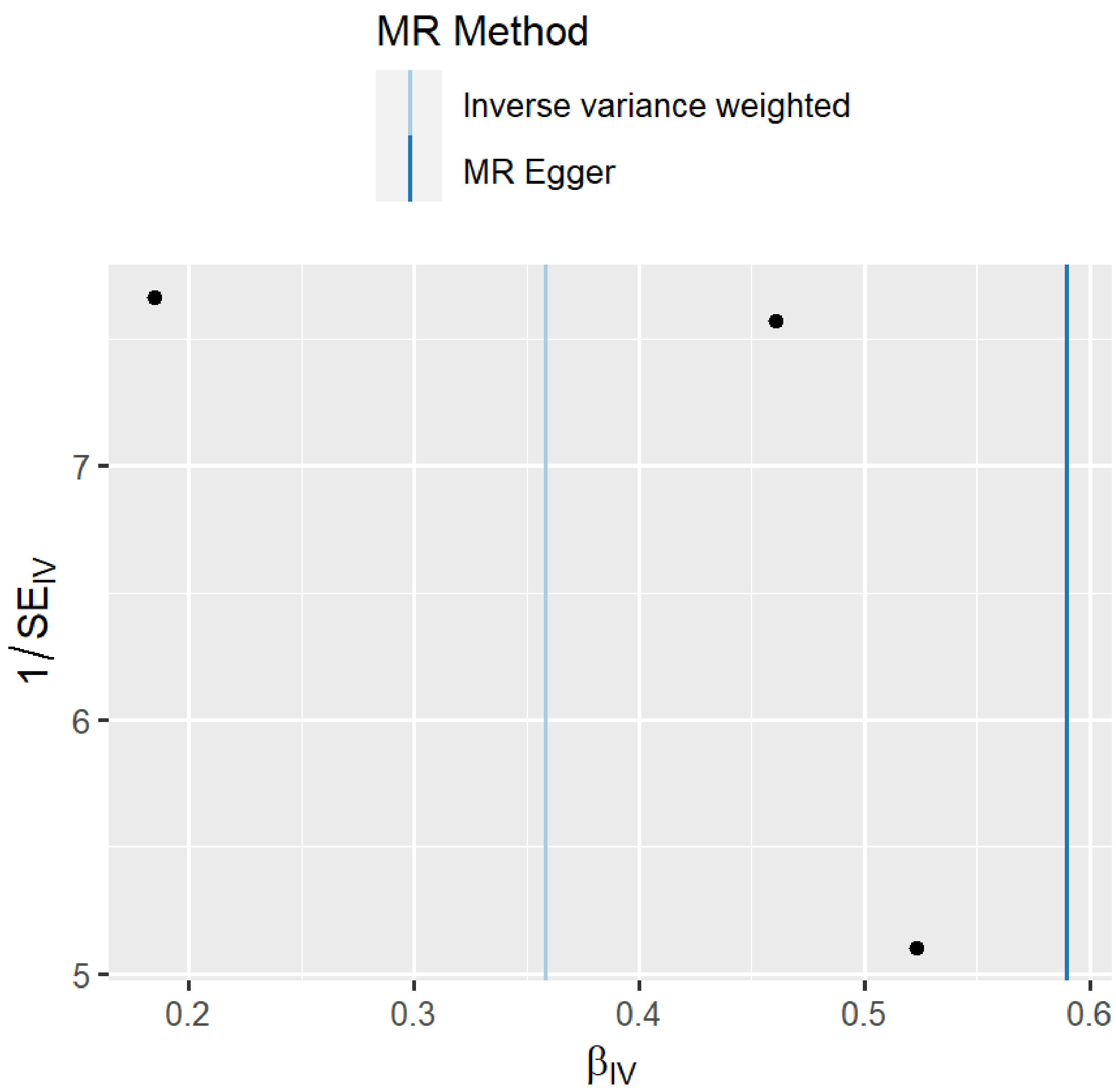
MR Method
Inverse variance weighted
MR Egger
7
6
5
0.2
0.3
0.4
0.5
0.6
$1/SE_{IV}$
β_{IV}

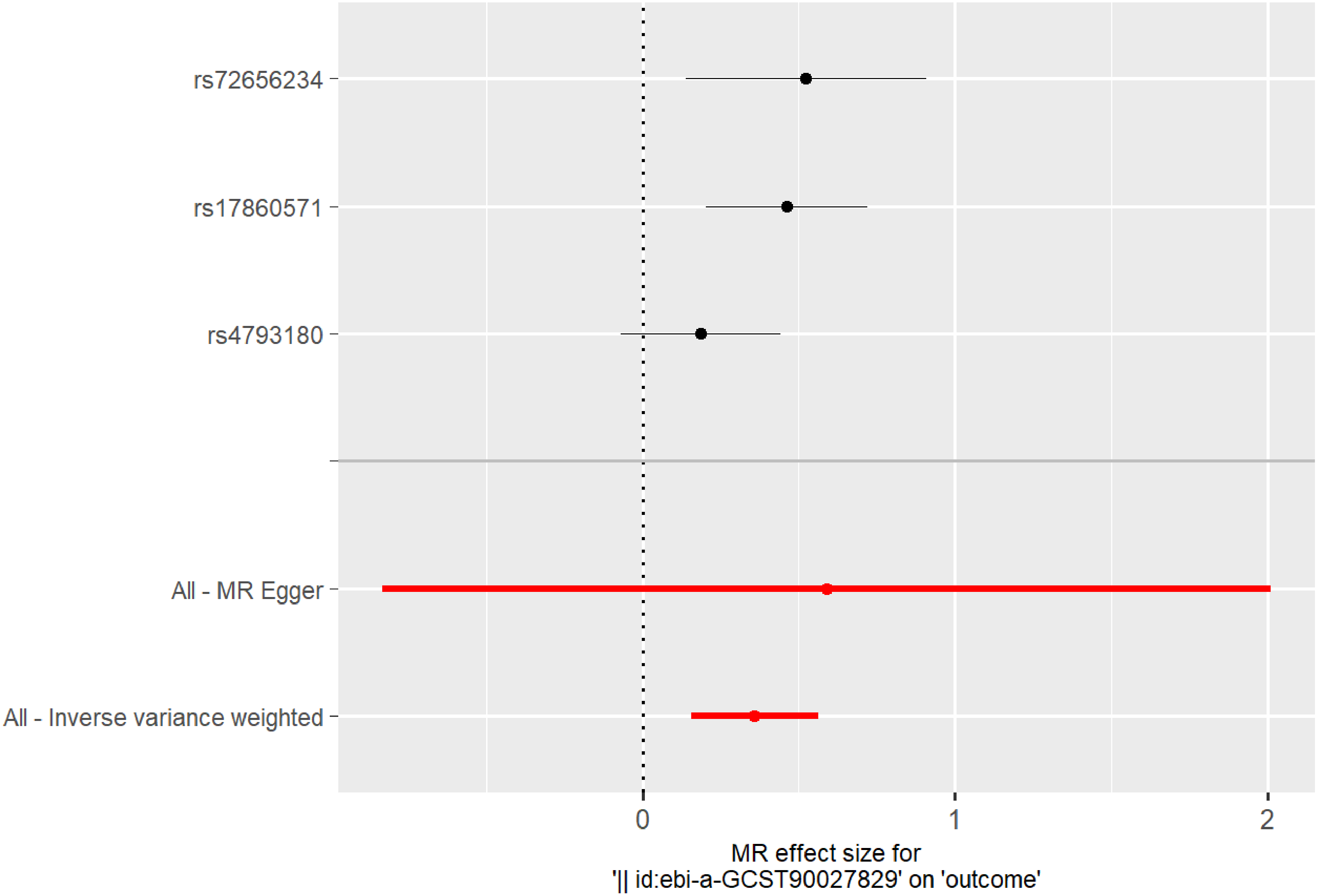

rs72656234
rs17860571
rs4793180
All - MR Egger
All - Inverse variance weighted
0
1
2
MR effect size for
'|| id:ebi-a-GCST90027829' on 'outcome'

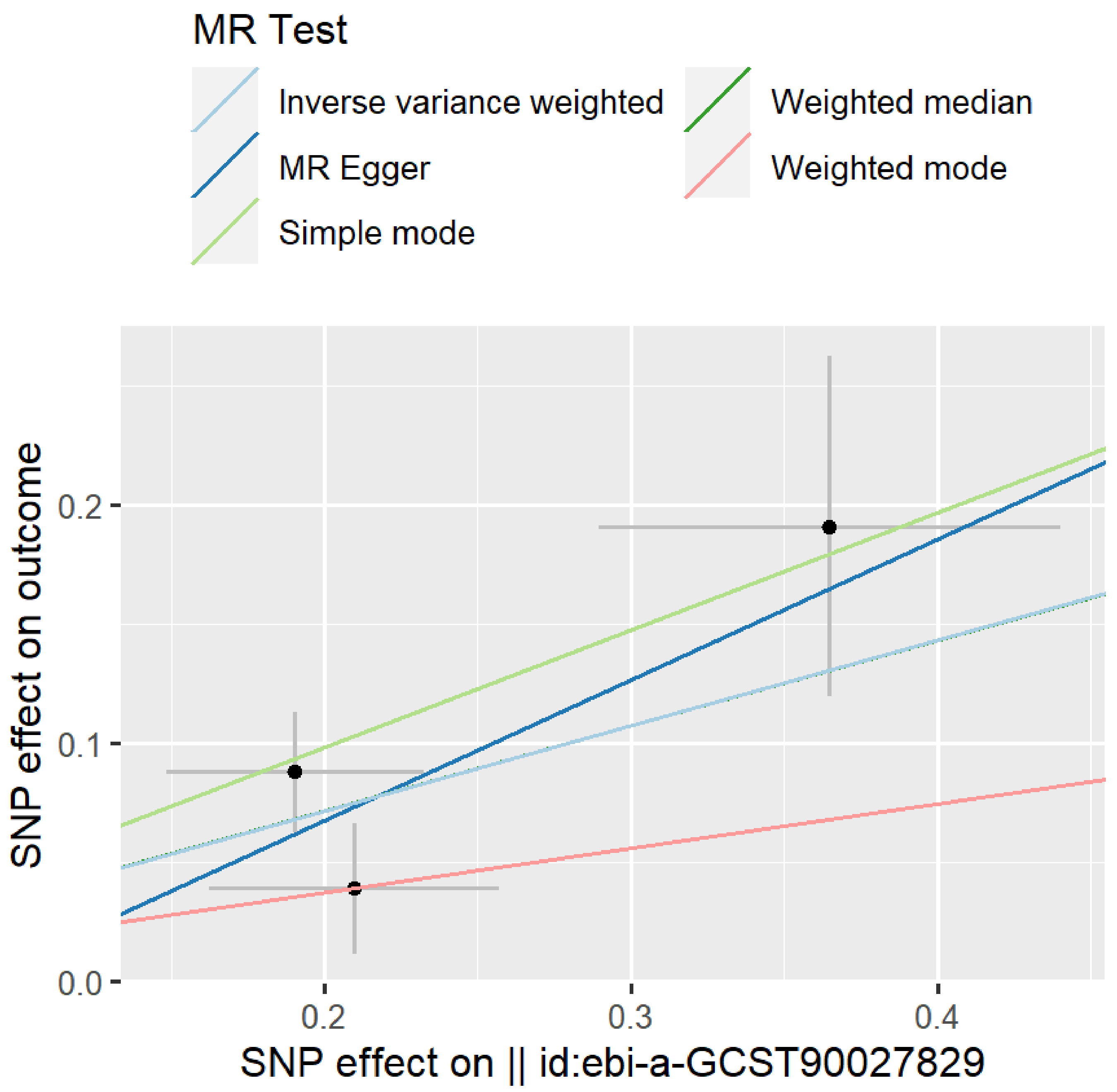
MR Test
Inverse variance weighted
MR Egger
Simple mode
Weighted median
Weighted mode
SNP effect on outcome
0.0
0.1
0.2
0.2
0.3
0.4
SNP effect on || id:ebi-a-GCST90027829

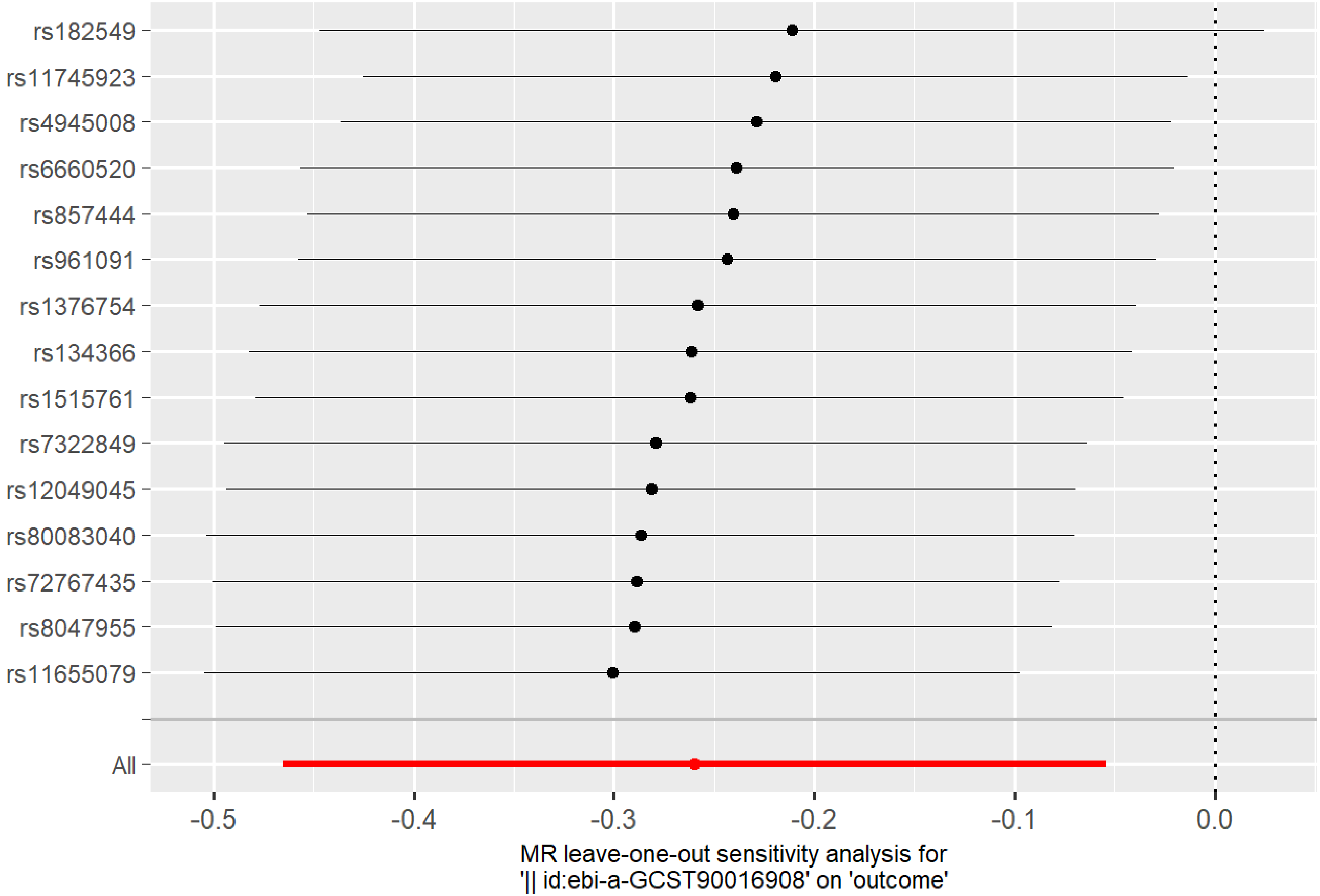
rs182549
rs11745923
rs4945008
rs6660520
rs857444
rs961091
rs1376754
rs134366
rs1515761
rs7322849
rs12049045
rs80083040
rs72767435
rs8047955
rs11655079
All
-0.5
-0.4
-0.3
-0.2
-0.1
0.0
MR leave-one-out sensitivity analysis for
'|| id:ebi-a-GCST90016908' on 'outcome'

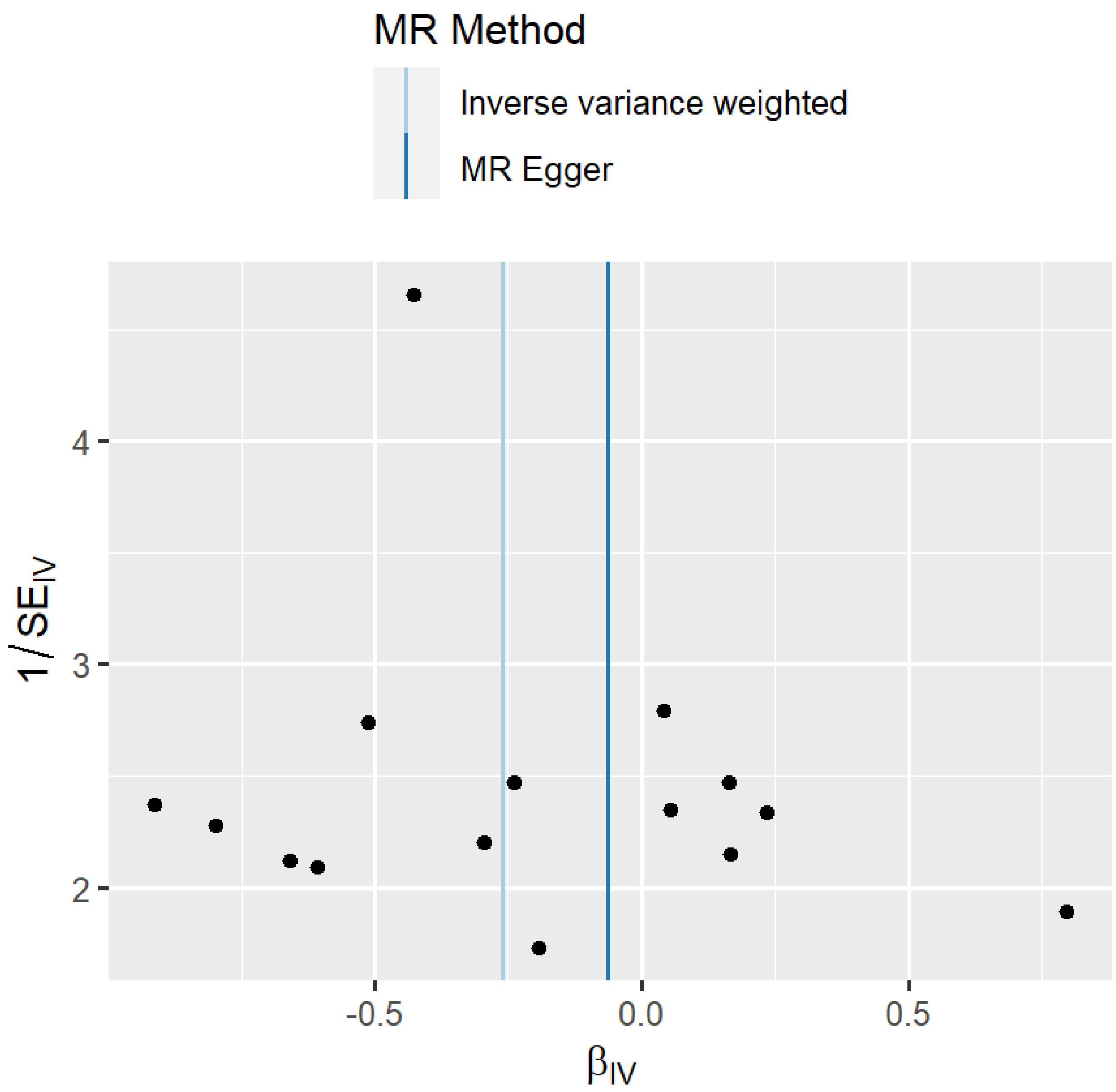
MR Method
Inverse variance weighted
MR Egger
4
3
2
1/SE_IV
-0.5
0.0
0.5
β_IV

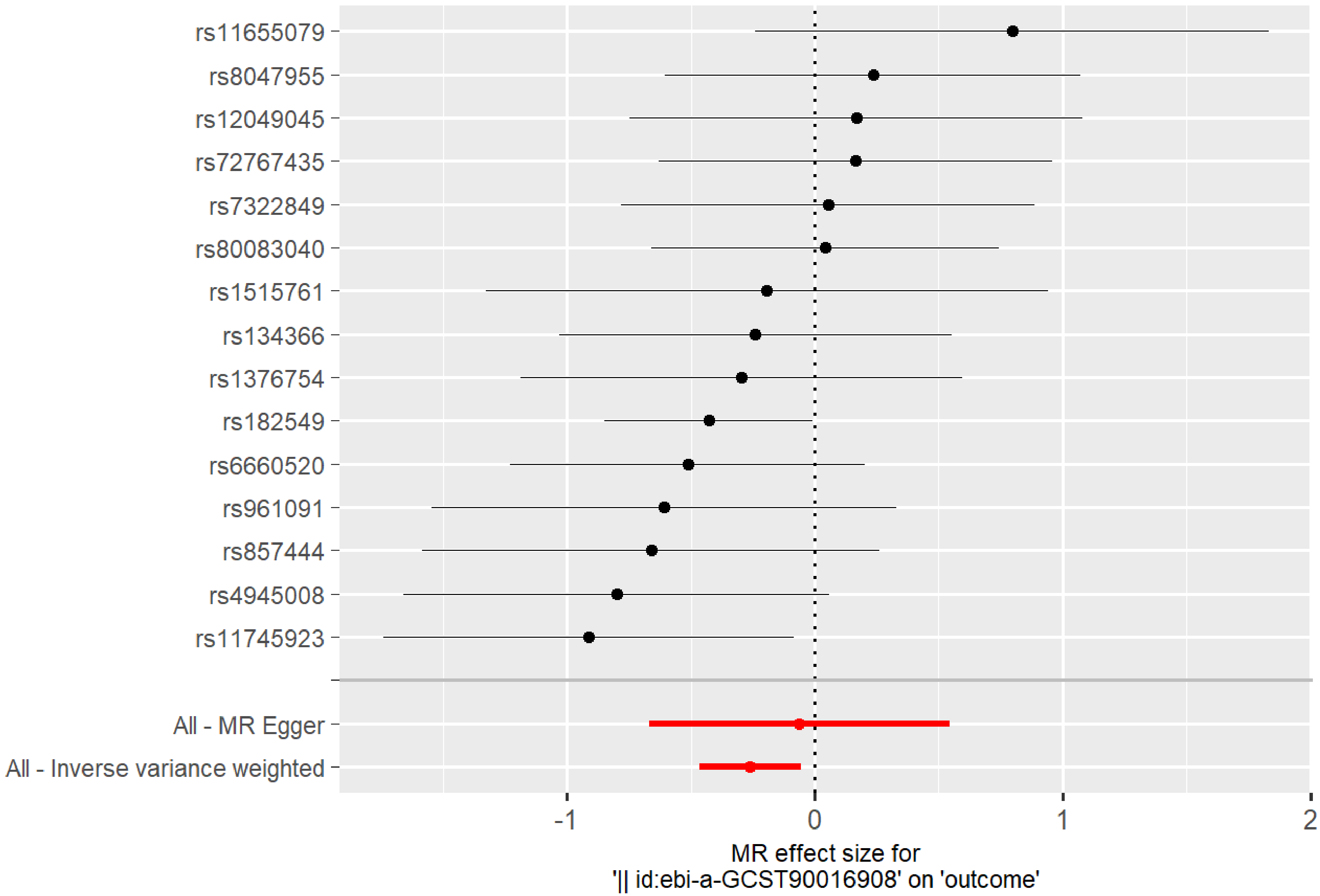

rs11655079
rs8047955
rs12049045
rs72767435
rs7322849
rs80083040
rs1515761
rs134366
rs1376754
rs182549
rs6660520
rs961091
rs857444
rs4945008
rs11745923
All - MR Egger
All - Inverse variance weighted
-1
0
1
2
MR effect size for
'|| id:ebi-a-GCST90016908' on 'outcome'

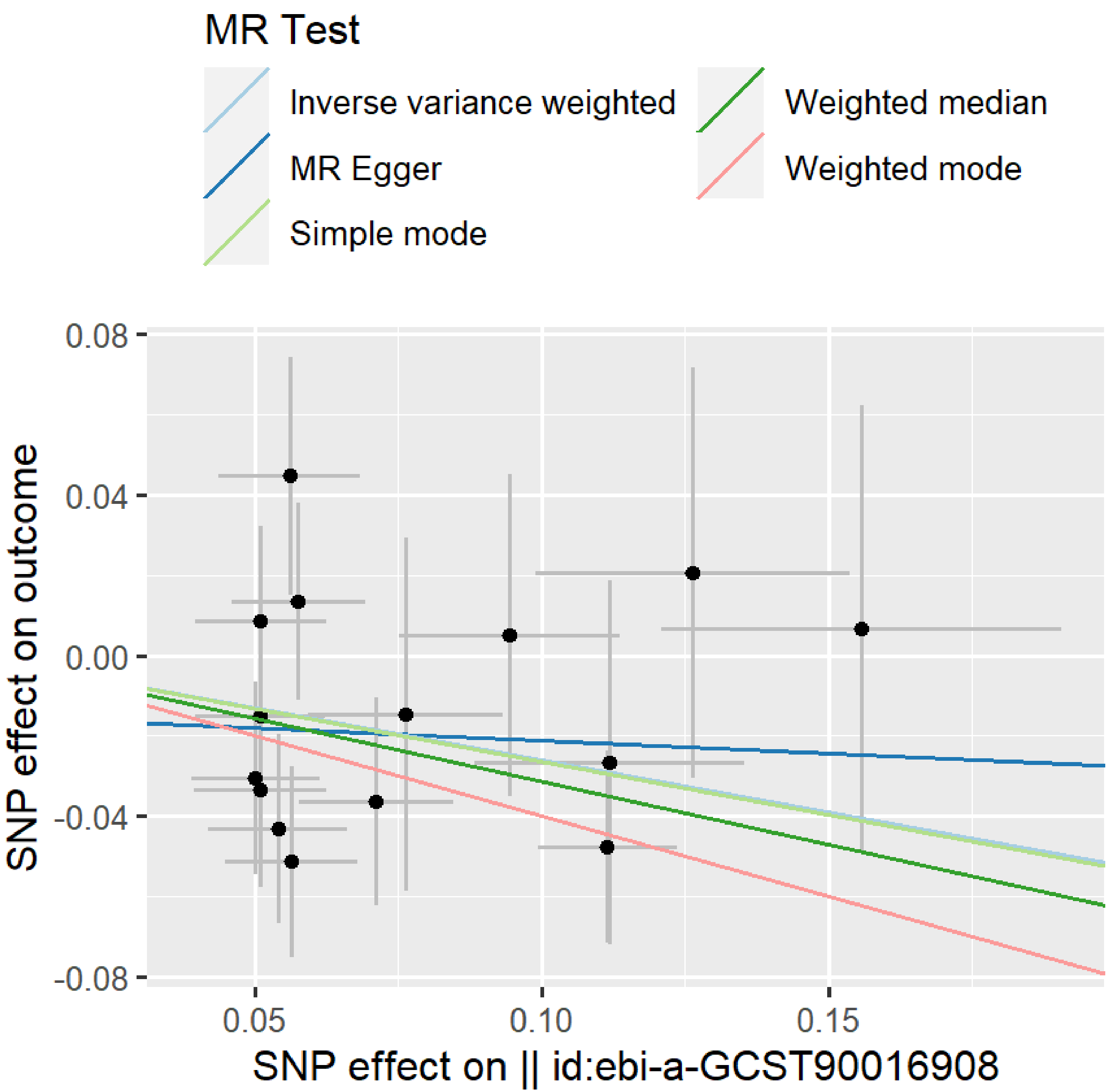
MR Test
Inverse variance weighted
MR Egger
Simple mode
Weighted median
Weighted mode
0.08
0.04
0.00
-0.04
-0.08
SNP effect on outcome
0.05
0.10
0.15
SNP effect on || id:ebi-a-GCST90016908

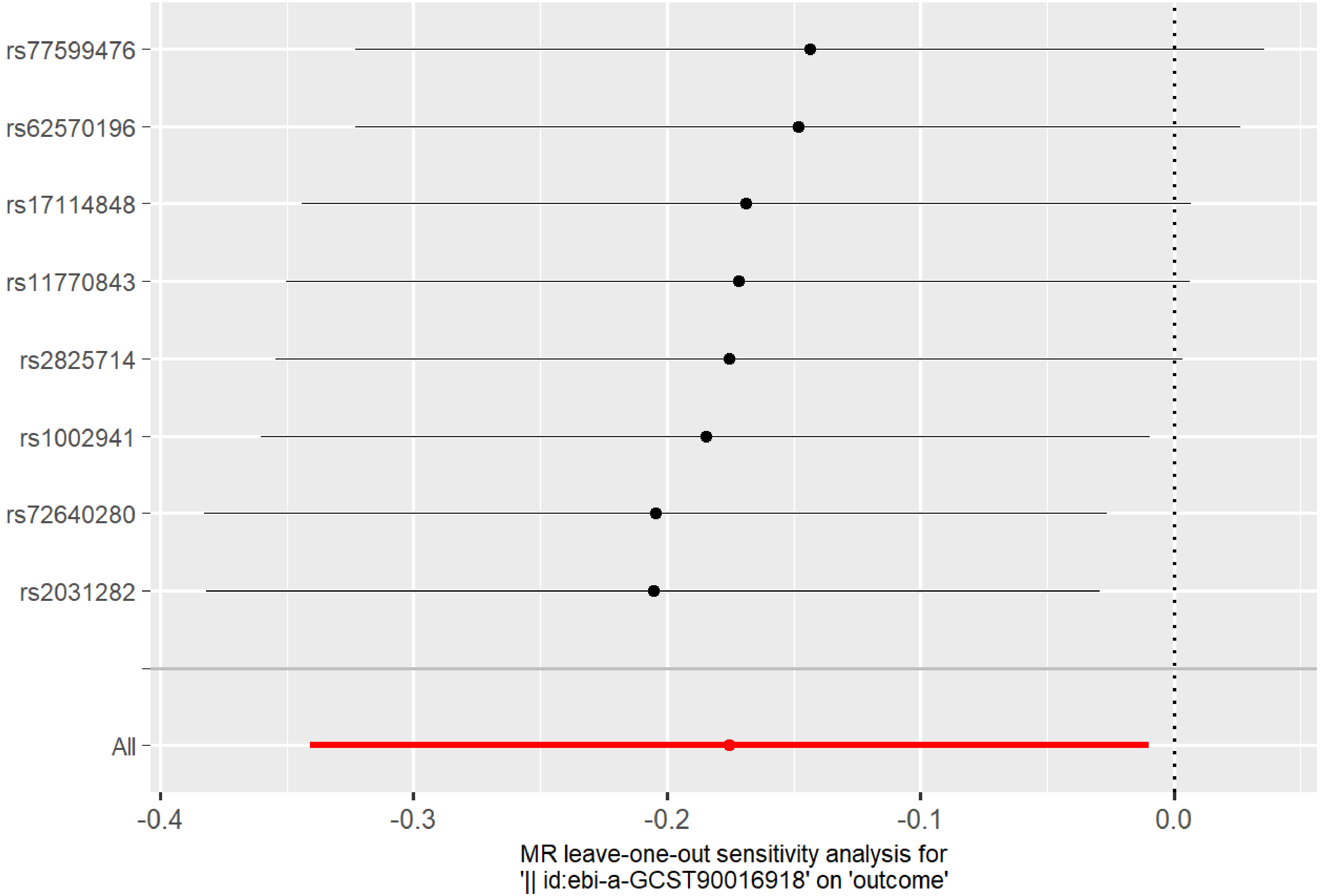
rs77599476
rs62570196
rs17114848
rs11770843
rs2825714
rs1002941
rs72640280
rs2031282
All
-0.4
-0.3
-0.2
-0.1
0.0
MR leave-one-out sensitivity analysis for
'|| id:ebi-a-GCST90016918' on 'outcome'

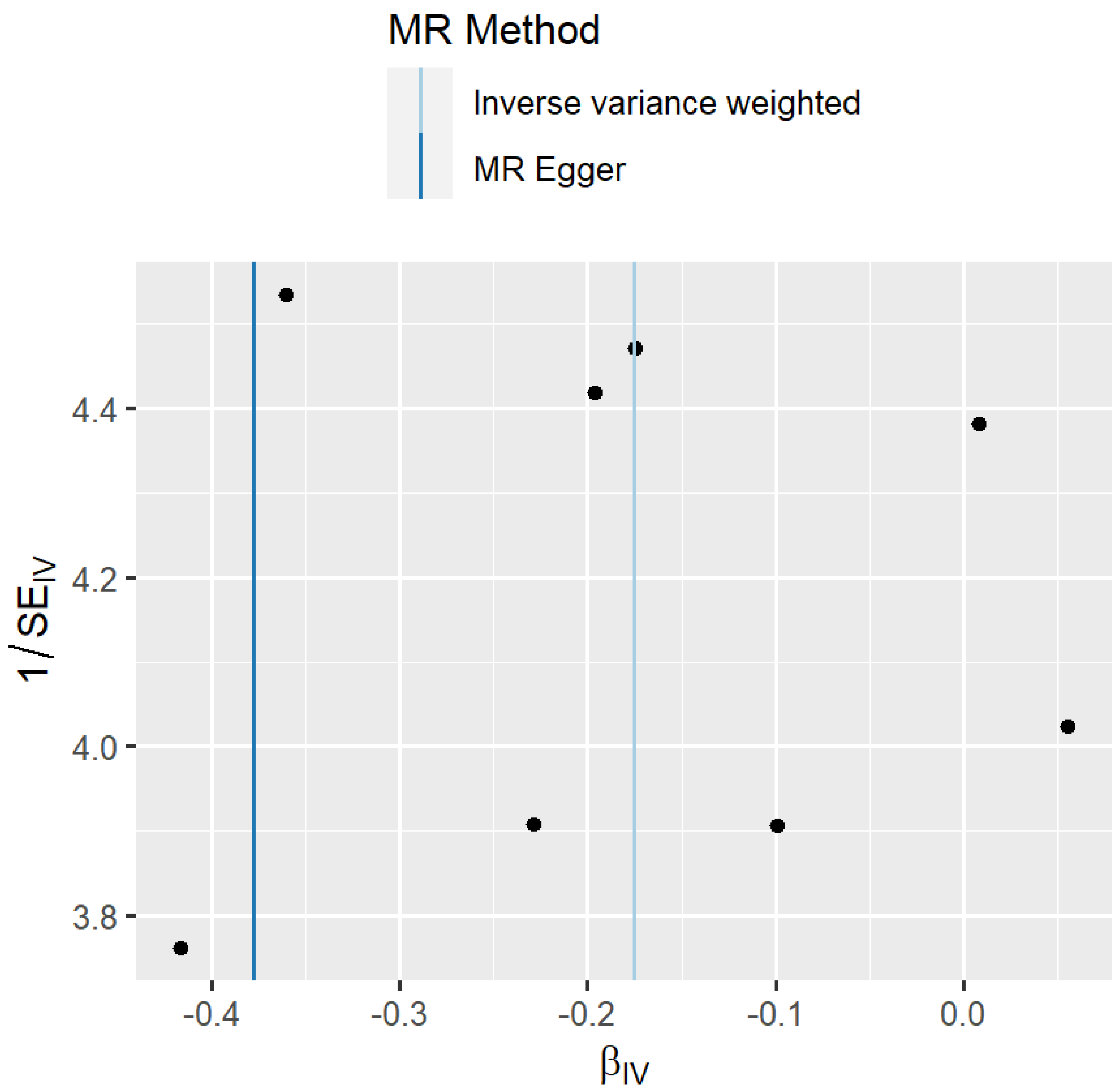
MR Method
Inverse variance weighted
MR Egger
4.4
4.2
4.0
3.8
1/SE$_{IV}$
-0.4
-0.3
-0.2
-0.1
0.0
β_{IV}

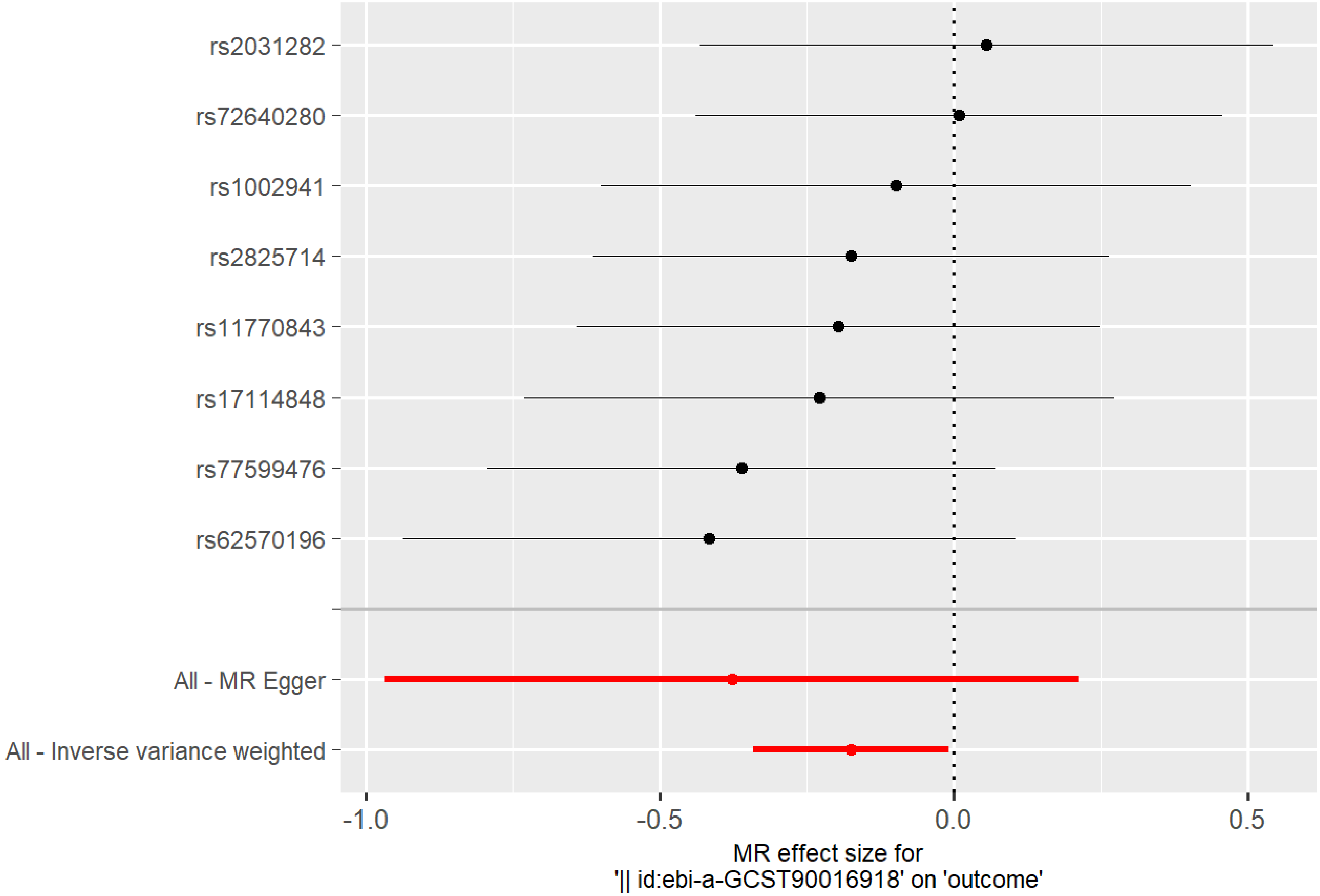
rs2031282
rs72640280
rs1002941
rs2825714
rs11770843
rs17114848
rs77599476
rs62570196
All - MR Egger
All - Inverse variance weighted
-1.0
-0.5
0.0
0.5
MR effect size for
'|| id:ebi-a-GCST90016918' on 'outcome'

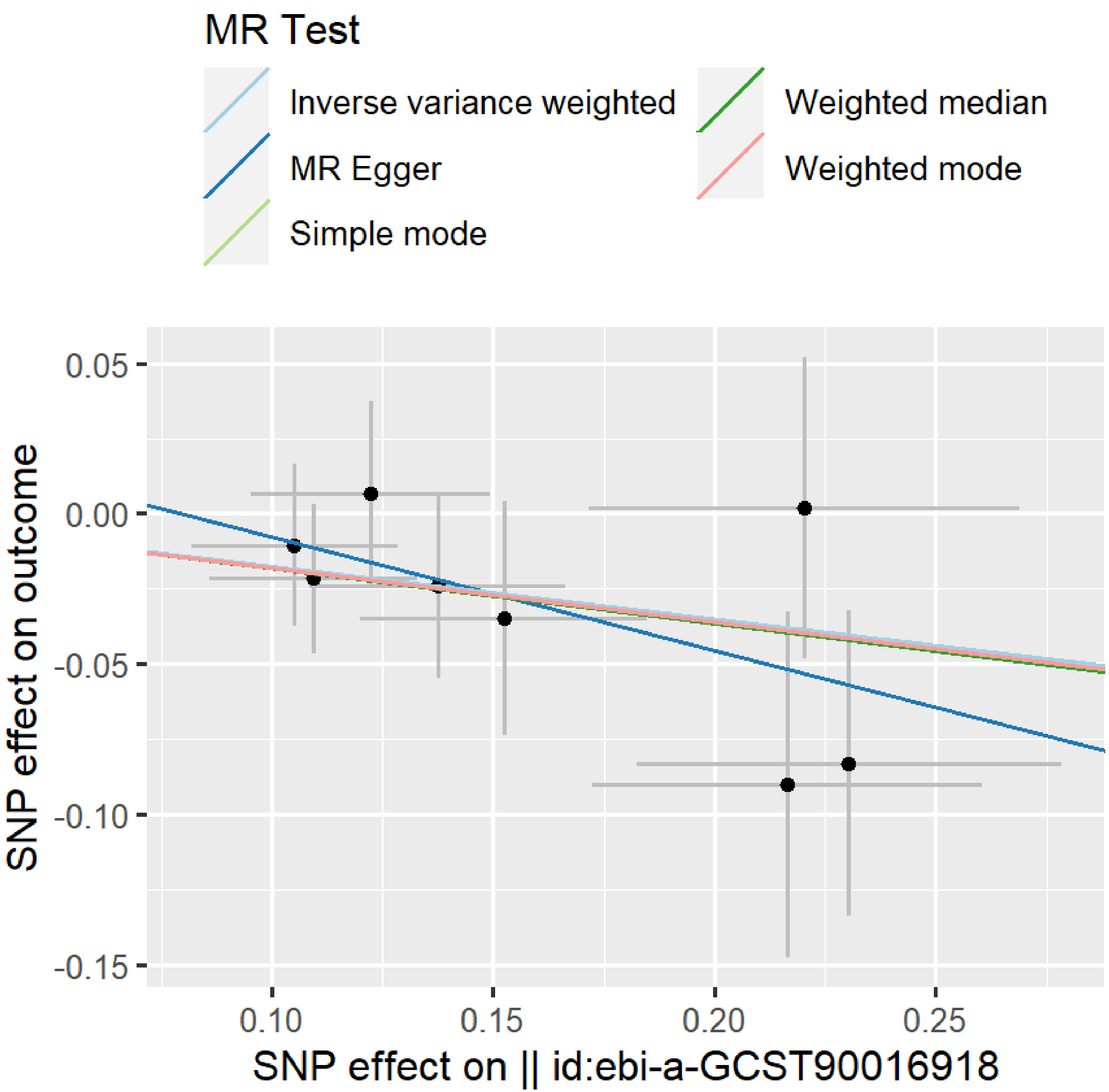

MR Test
Inverse variance weighted
MR Egger
Simple mode
Weighted median
Weighted mode
0.05
0.00
-0.05
-0.10
-0.15
0.10
0.15
0.20
0.25
SNP effect on outcome
SNP effect on || id:ebi-a-GCST90016918

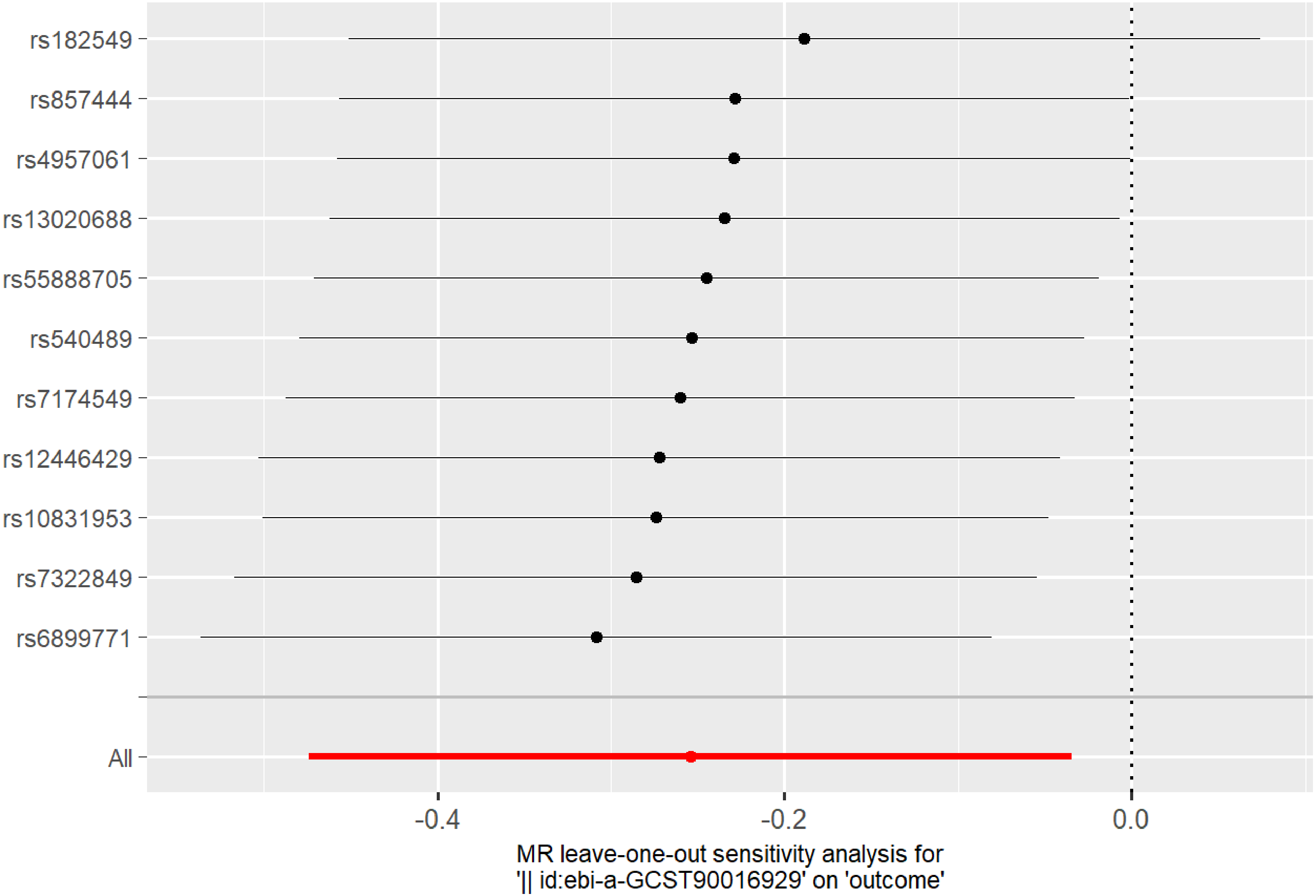
rs182549
rs857444
rs4957061
rs13020688
rs55888705
rs540489
rs7174549
rs12446429
rs10831953
rs7322849
rs6899771
All
-0.4
-0.2
0.0
MR leave-one-out sensitivity analysis for
'|| id:ebi-a-GCST90016929' on 'outcome'

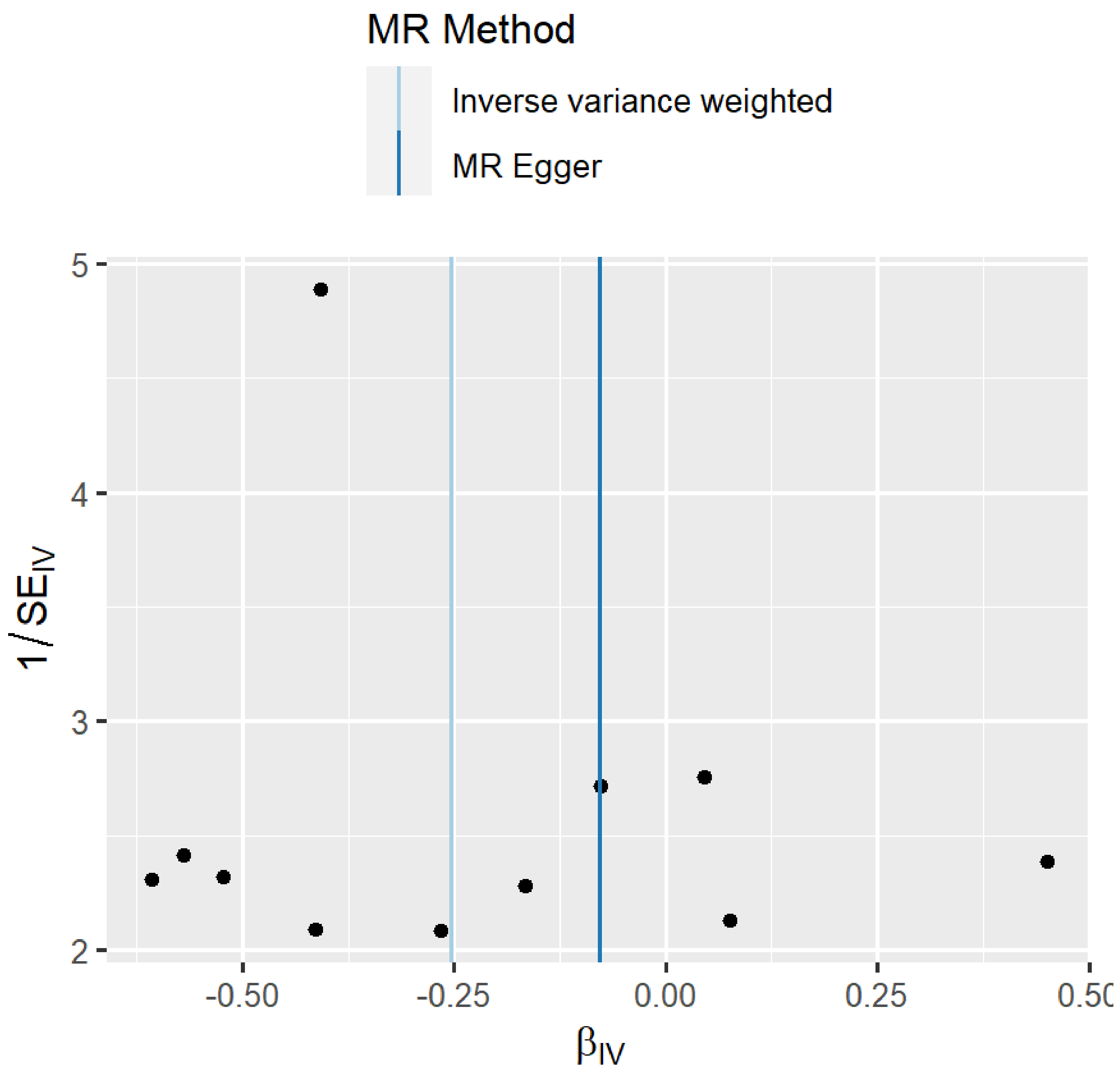
MR Method
Inverse variance weighted
MR Egger
5
4
3
2
1/SE$_{IV}$
-0.50
-0.25
0.00
0.25
0.50
β$_{IV}$

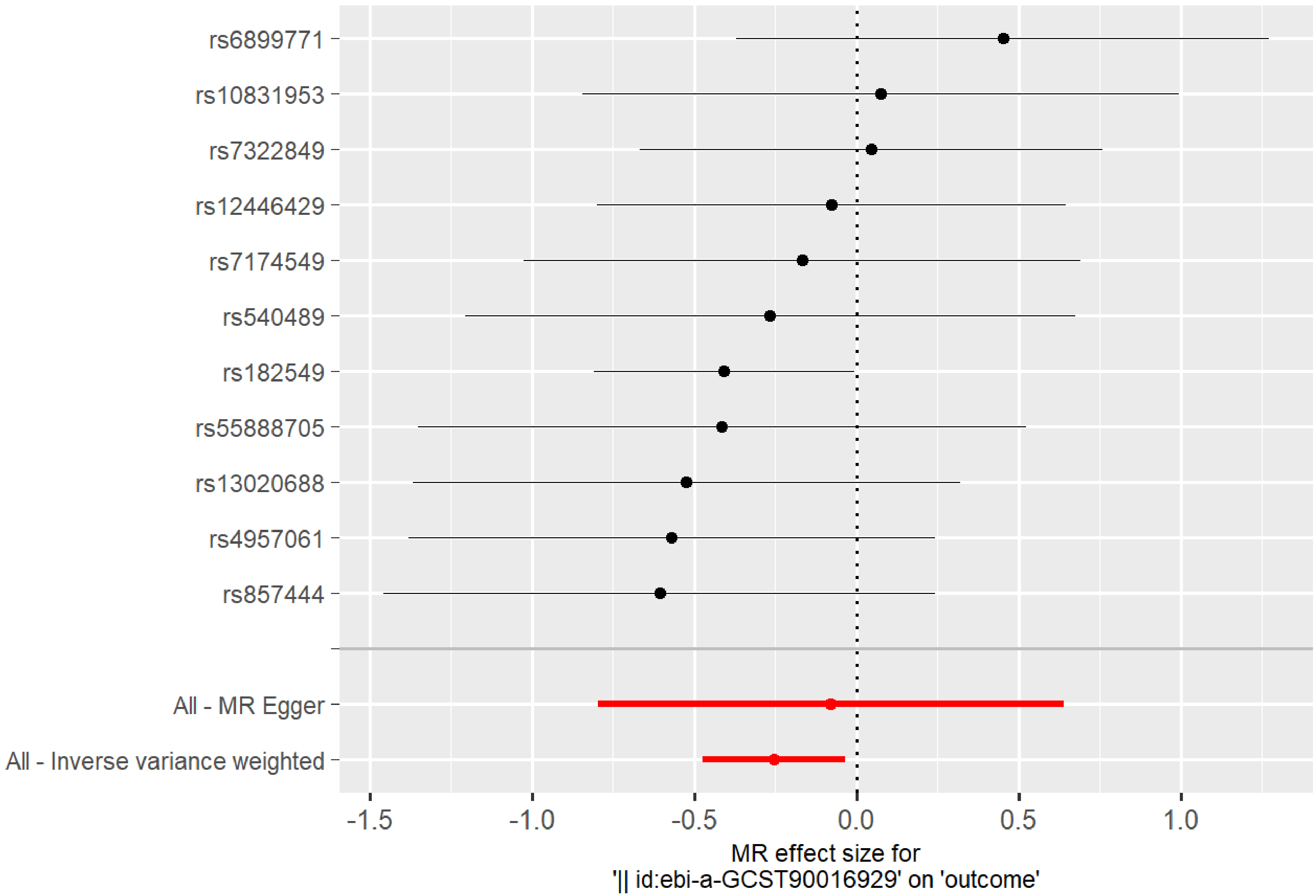
rs6899771
rs10831953
rs7322849
rs12446429
rs7174549
rs540489
rs182549
rs55888705
rs13020688
rs4957061
rs857444
All - MR Egger
All - Inverse variance weighted
-1.5
-1.0
-0.5
0.0
0.5
1.0
MR effect size for
'|| id:ebi-a-GCST90016929' on 'outcome'

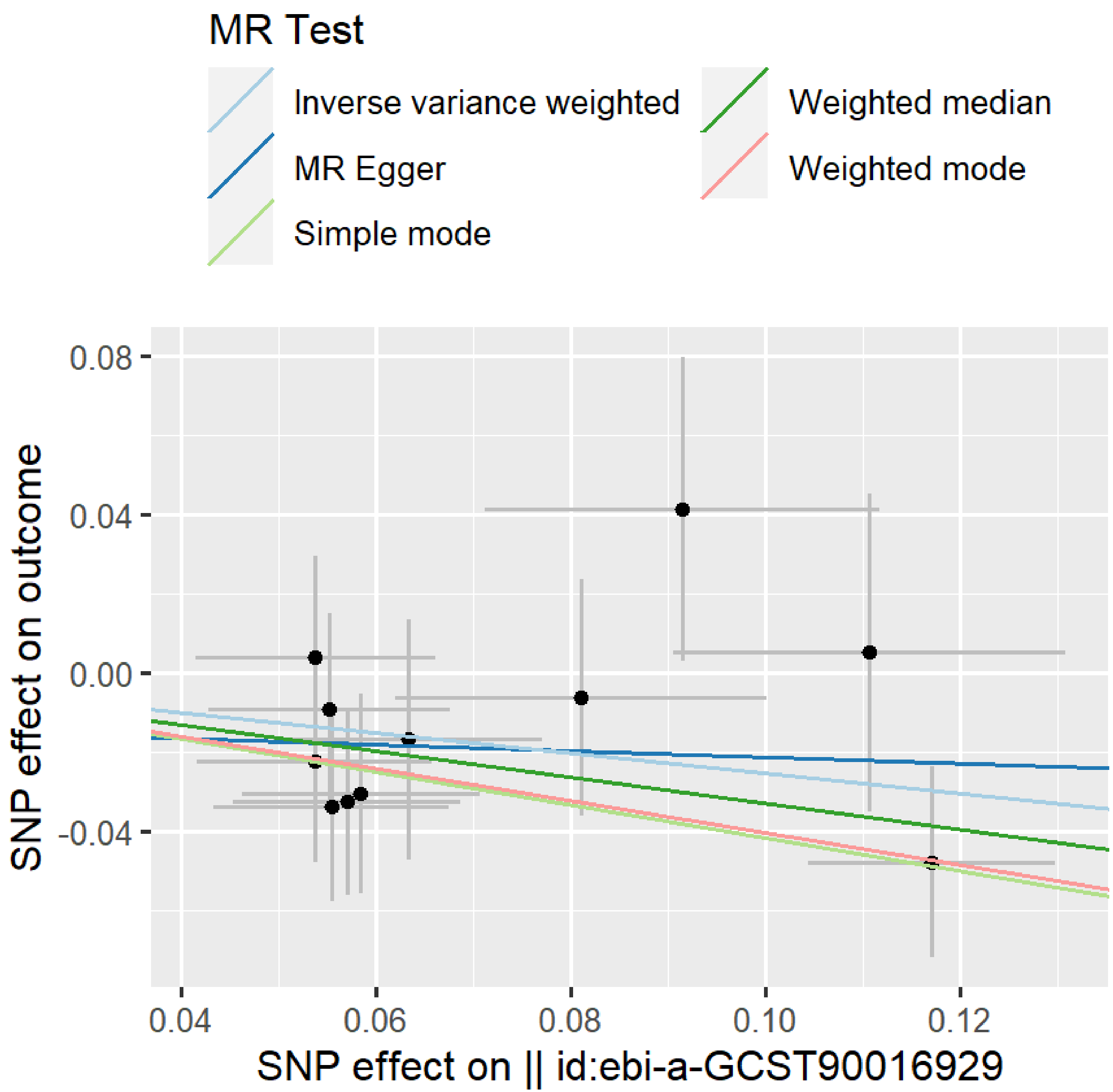
MR Test
Inverse variance weighted
MR Egger
Simple mode
Weighted median
Weighted mode
0.08
0.04
0.00
-0.04
SNP effect on outcome
0.04
0.06
0.08
0.10
0.12
SNP effect on || id:ebi-a-GCST90016929

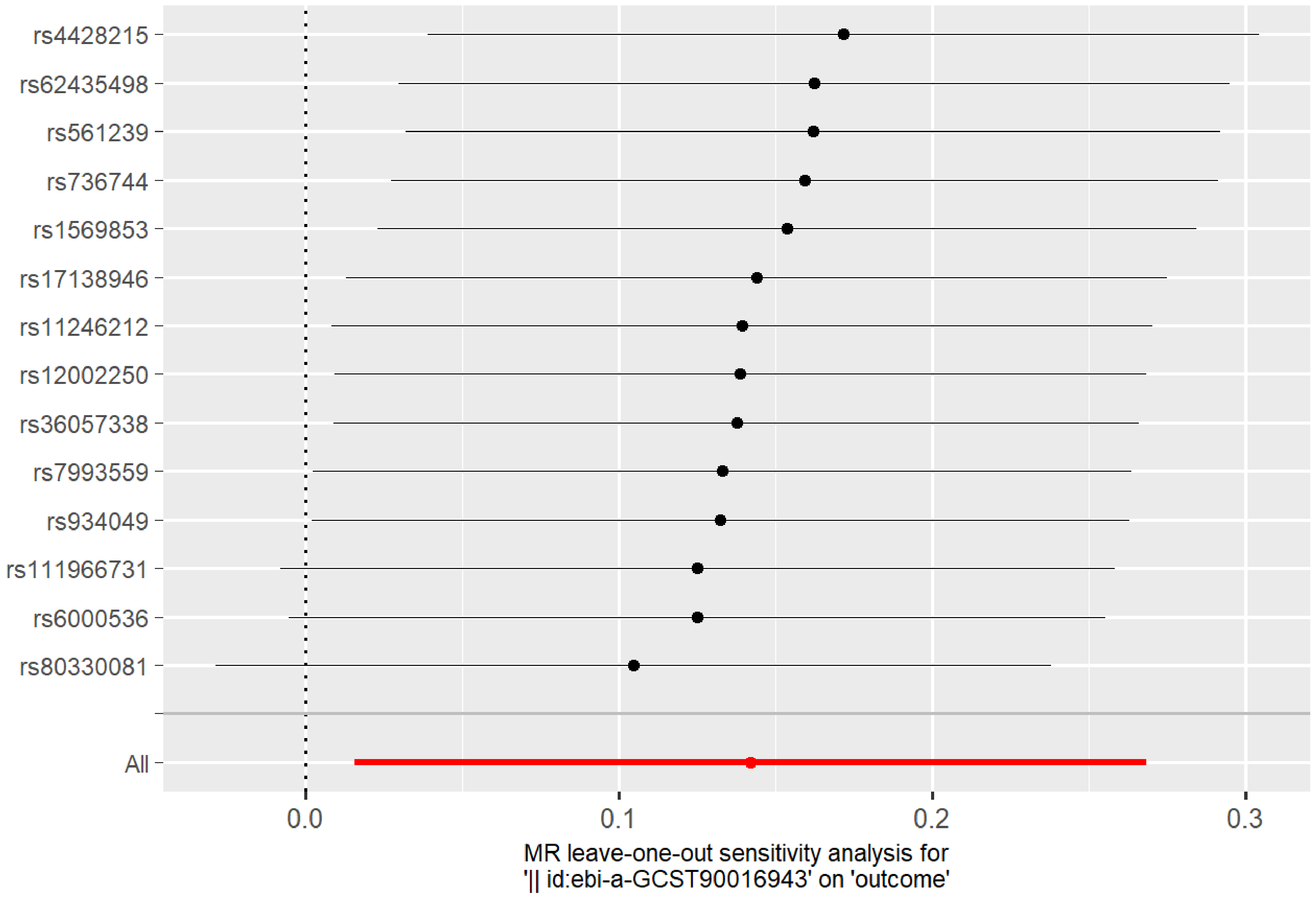

rs4428215
rs62435498
rs561239
rs736744
rs1569853
rs17138946
rs11246212
rs12002250
rs36057338
rs7993559
rs934049
rs111966731
rs6000536
rs80330081
All
0.0
0.1
0.2
0.3
MR leave-one-out sensitivity analysis for
'|| id:ebi-a-GCST90016943' on 'outcome'

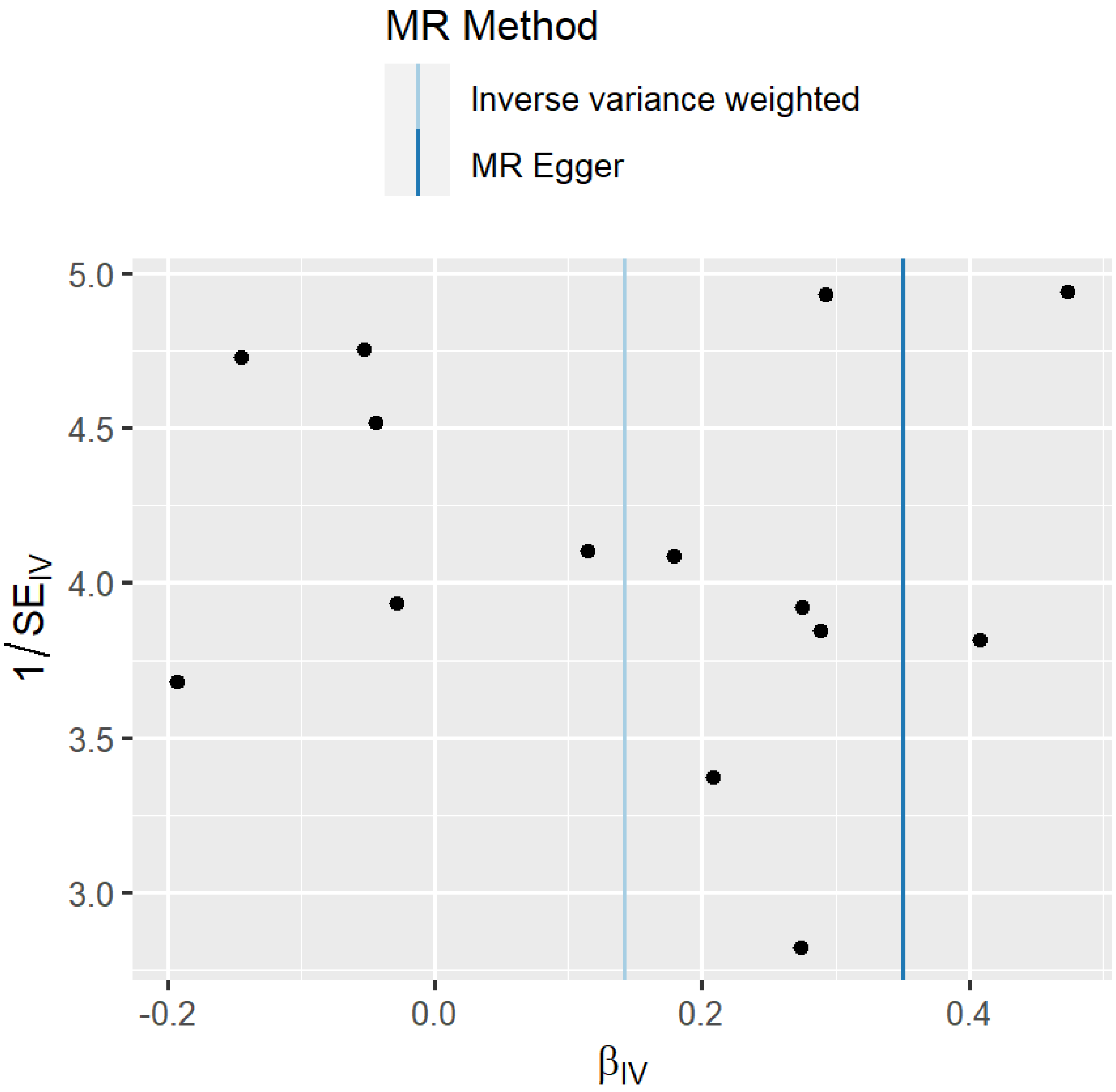
MR Method
Inverse variance weighted
MR Egger
5.0
4.5
4.0
3.5
3.0
1/SE_IV
-0.2
0.0
0.2
0.4
β_IV

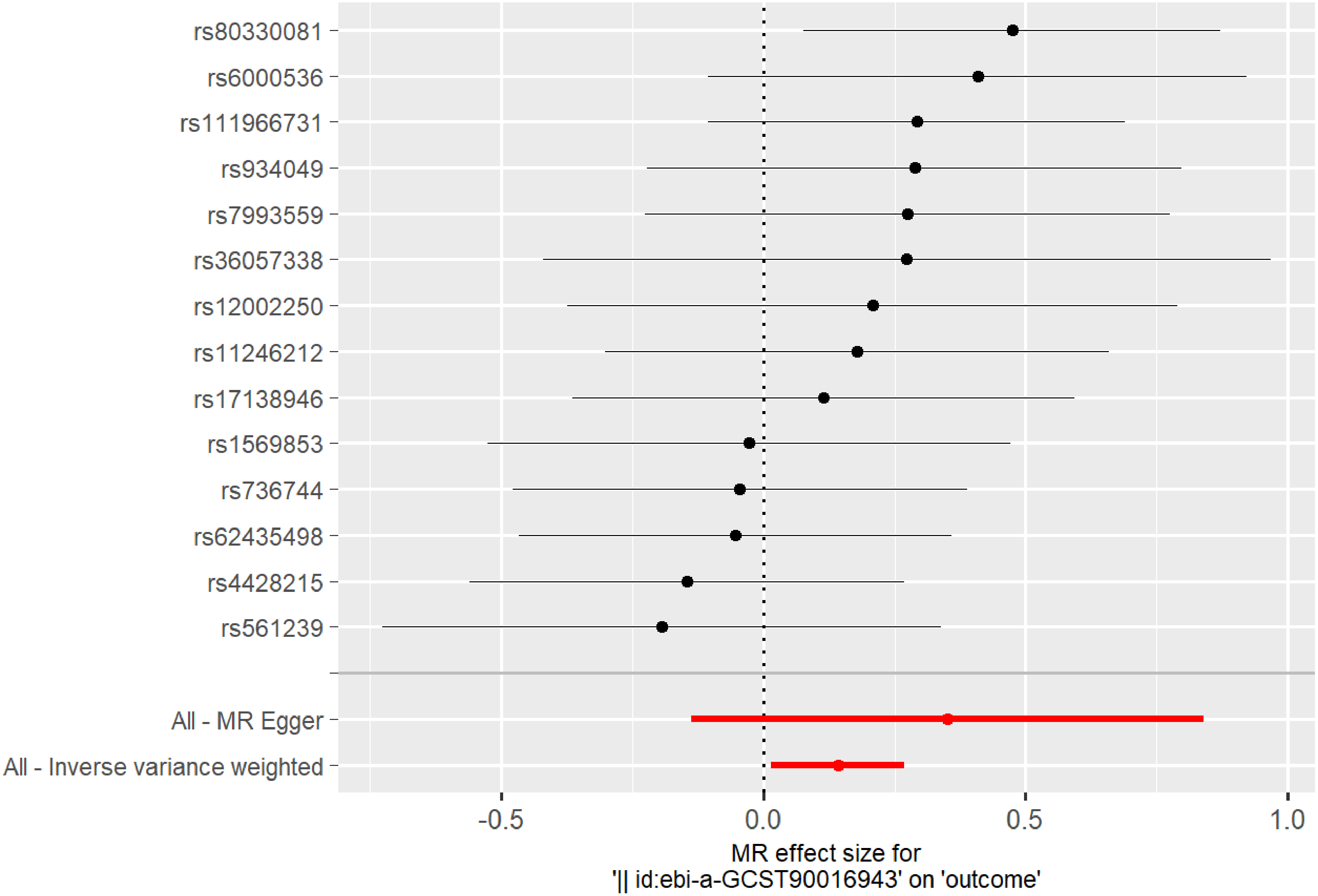
rs80330081
rs6000536
rs111966731
rs934049
rs7993559
rs36057338
rs12002250
rs11246212
rs17138946
rs1569853
rs736744
rs62435498
rs4428215
rs561239
All - MR Egger
All - Inverse variance weighted
-0.5
0.0
0.5
1.0
MR effect size for
'|| id:ebi-a-GCST90016943' on 'outcome'

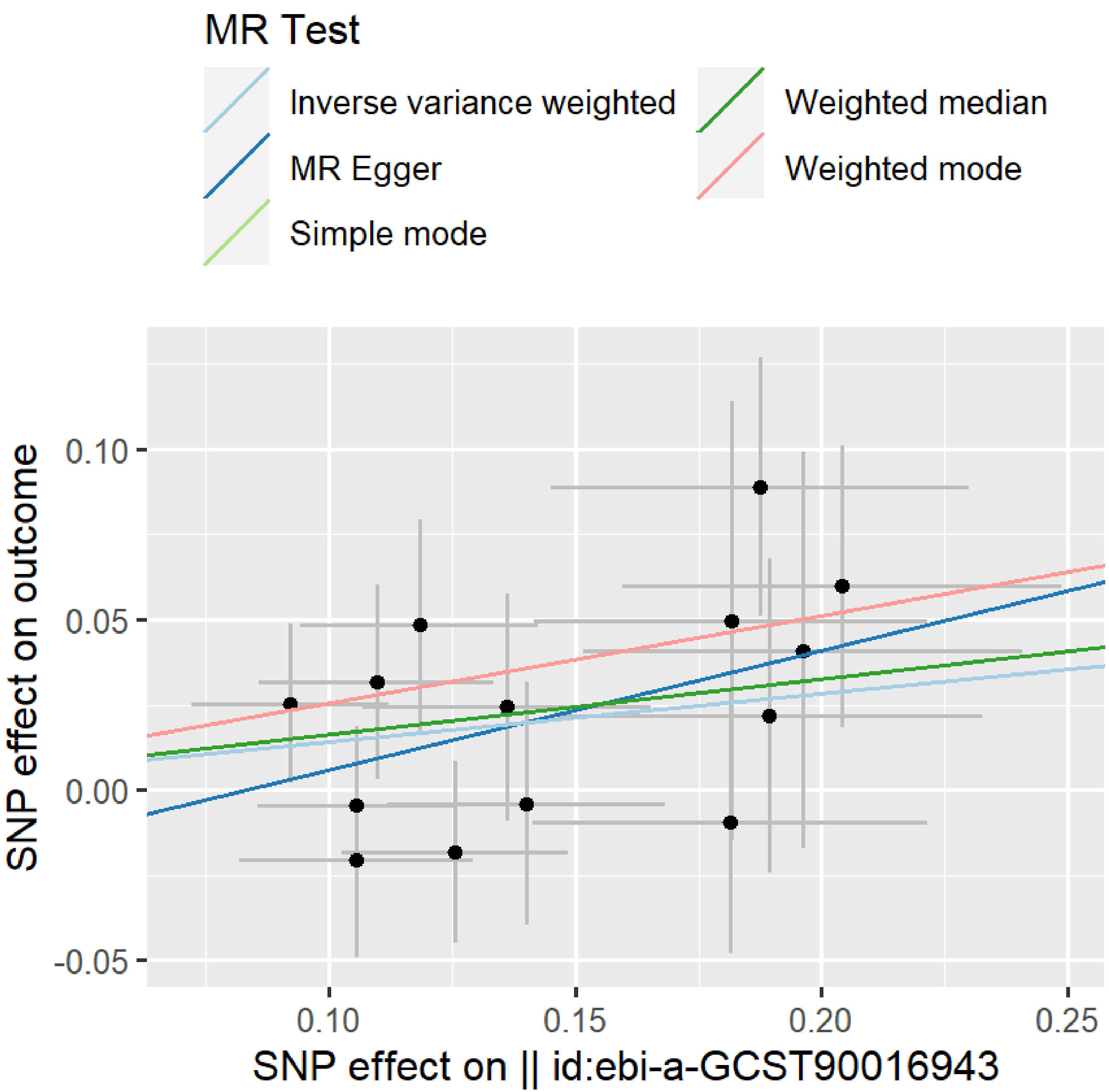

MR Test
Inverse variance weighted
MR Egger
Simple mode
Weighted median
Weighted mode
SNP effect on outcome
0.10
0.05
0.00
-0.05
0.10
0.15
0.20
0.25
SNP effect on || id:ebi-a-GCST90016943

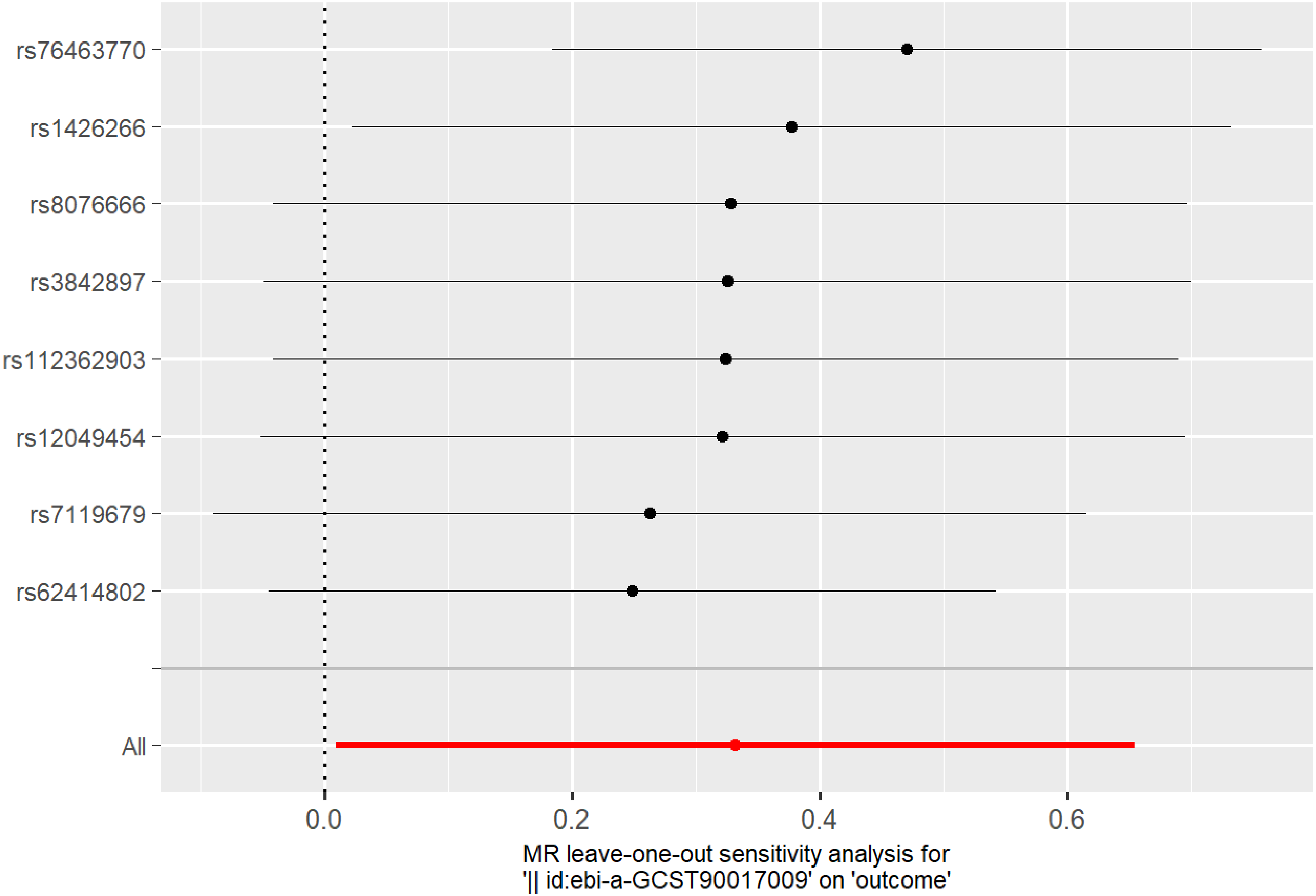
rs76463770
rs1426266
rs8076666
rs3842897
rs112362903
rs12049454
rs7119679
rs62414802
All
0.0
0.2
0.4
0.6
MR leave-one-out sensitivity analysis for
'|| id:ebi-a-GCST90017009' on 'outcome'

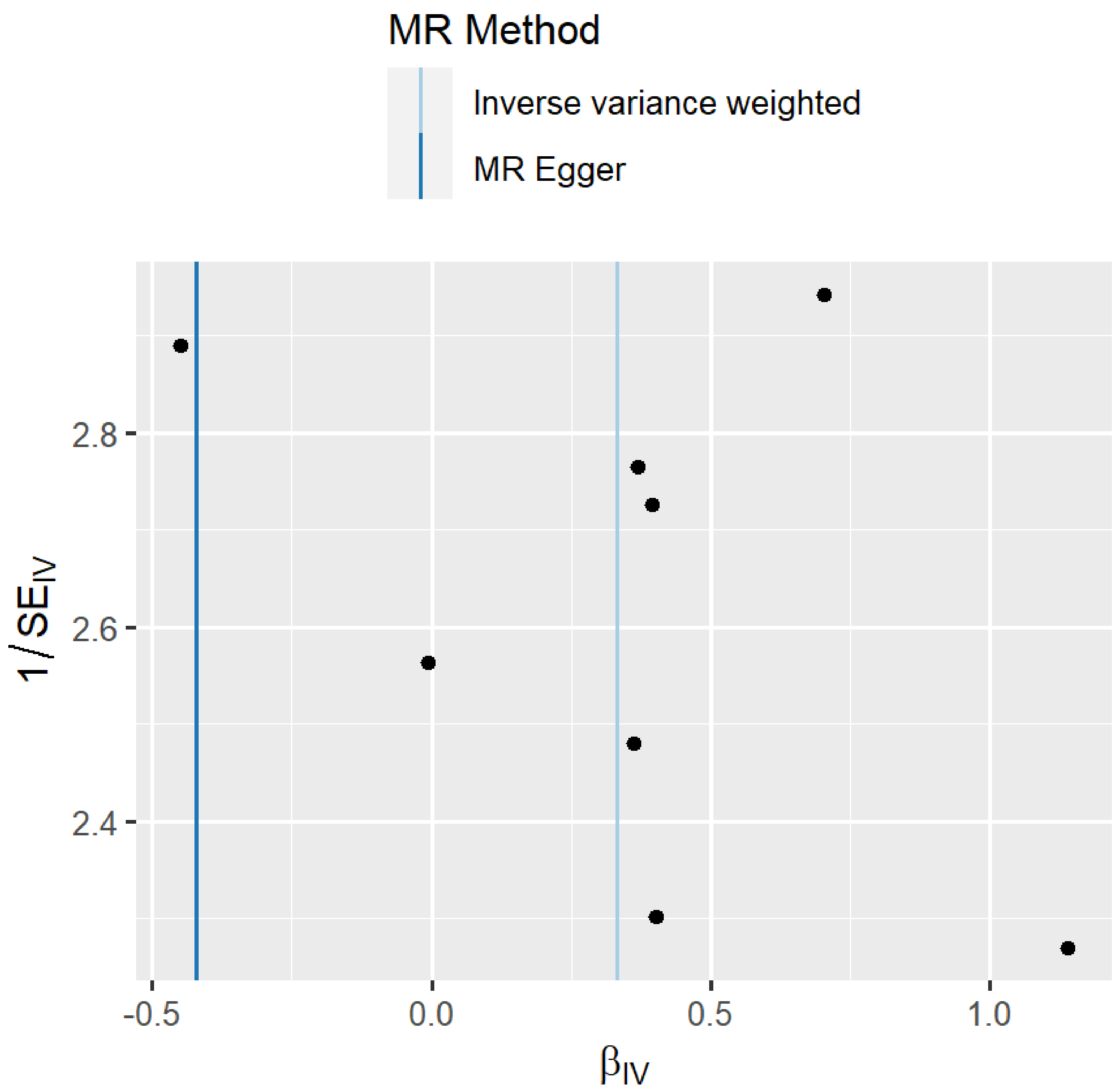
MR Method
Inverse variance weighted
MR Egger
2.8
2.6
2.4
1/SE_IV
-0.5
0.0
0.5
1.0
β_IV

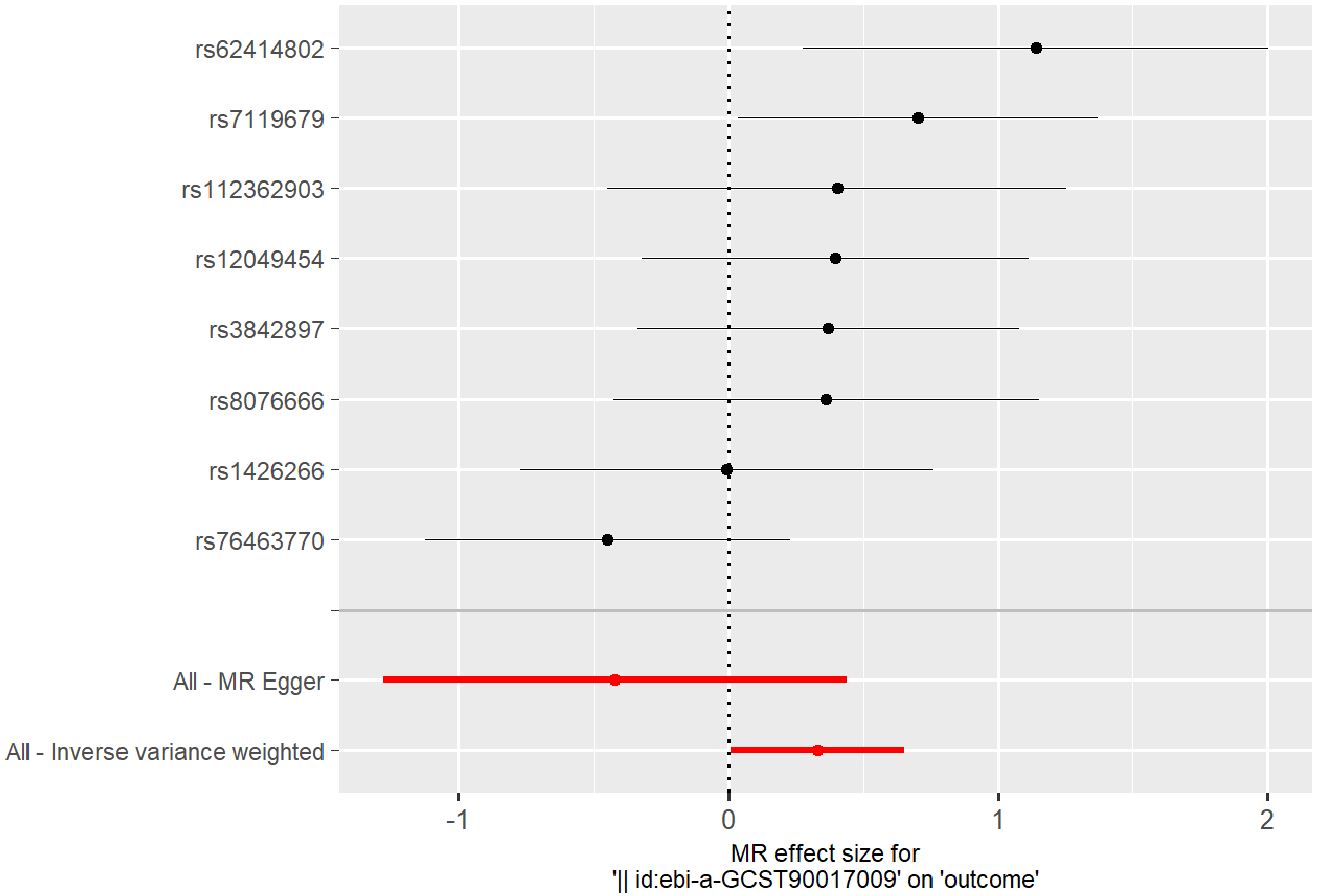
rs62414802
rs7119679
rs112362903
rs12049454
rs3842897
rs8076666
rs1426266
rs76463770
All - MR Egger
All - Inverse variance weighted
-1
0
1
2
MR effect size for
'|| id:ebi-a-GCST90017009' on 'outcome'

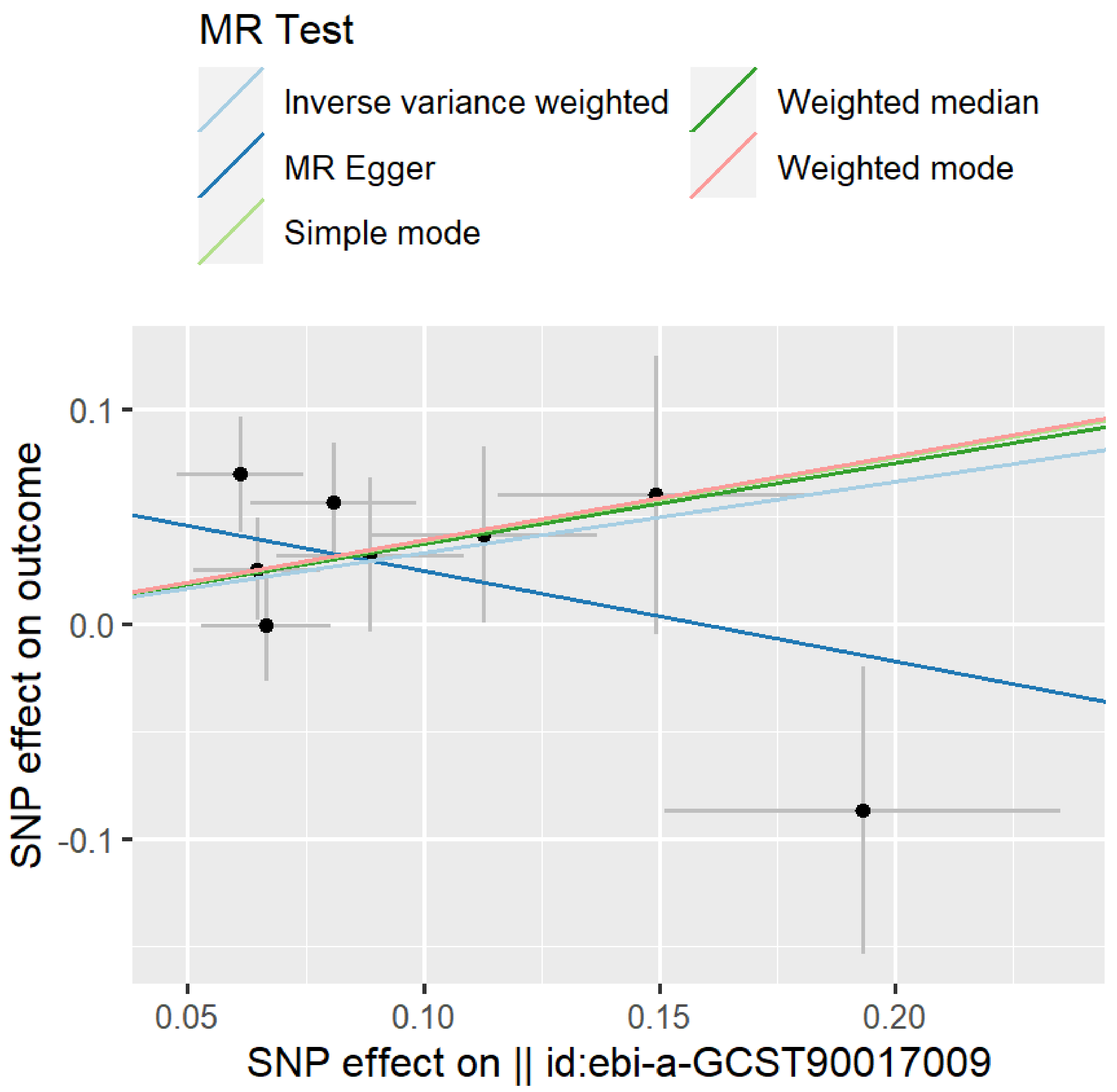
MR Test
Inverse variance weighted
MR Egger
Simple mode
Weighted median
Weighted mode
SNP effect on outcome
0.1
0.0
-0.1
0.05
0.10
0.15
0.20
SNP effect on || id:ebi-a-GCST90017009

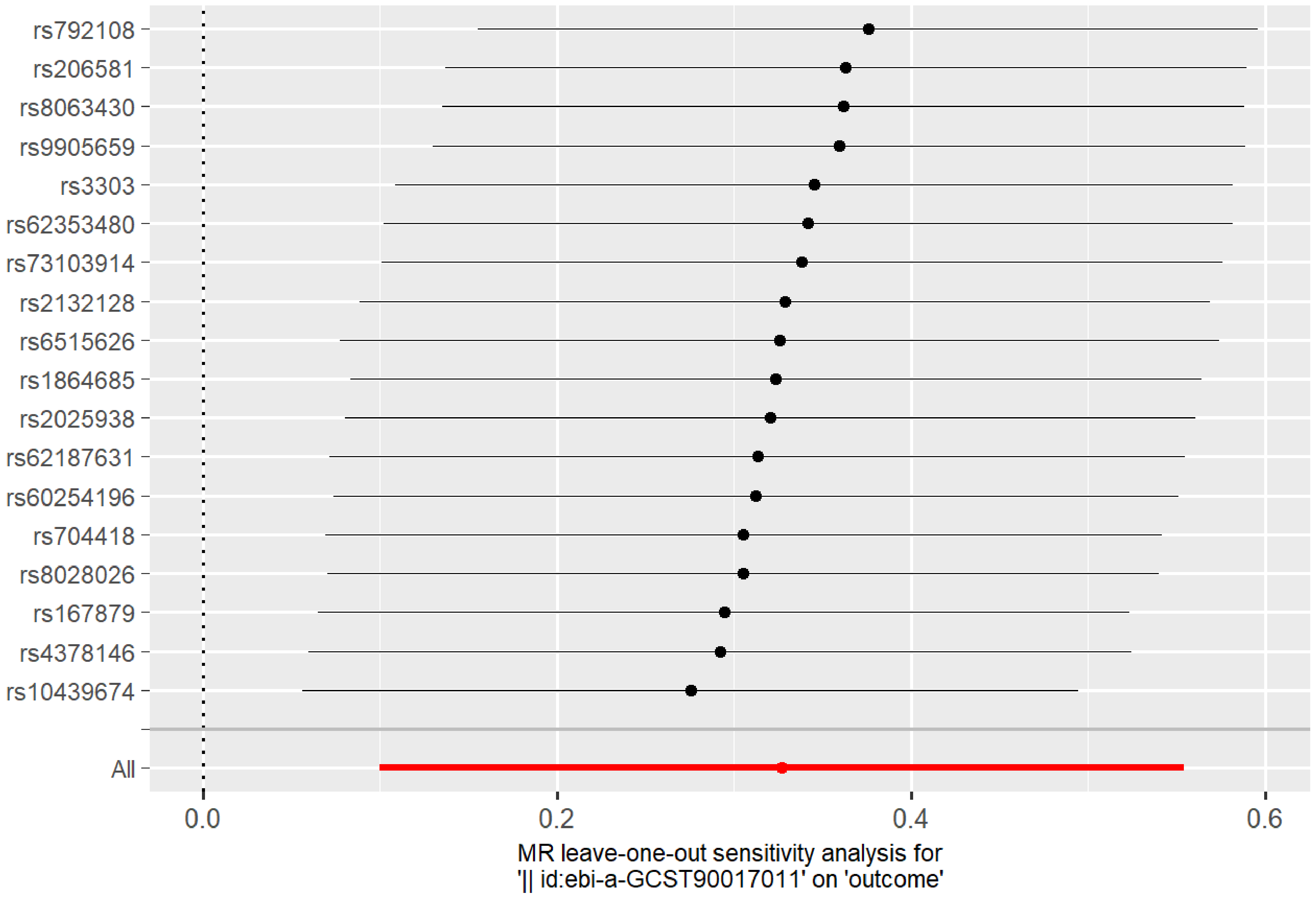
rs792108
rs206581
rs8063430
rs9905659
rs3303
rs62353480
rs73103914
rs2132128
rs6515626
rs1864685
rs2025938
rs62187631
rs60254196
rs704418
rs8028026
rs167879
rs4378146
rs10439674
All
0.0
0.2
0.4
0.6
MR leave-one-out sensitivity analysis for
'|| id:ebi-a-GCST90017011' on 'outcome'

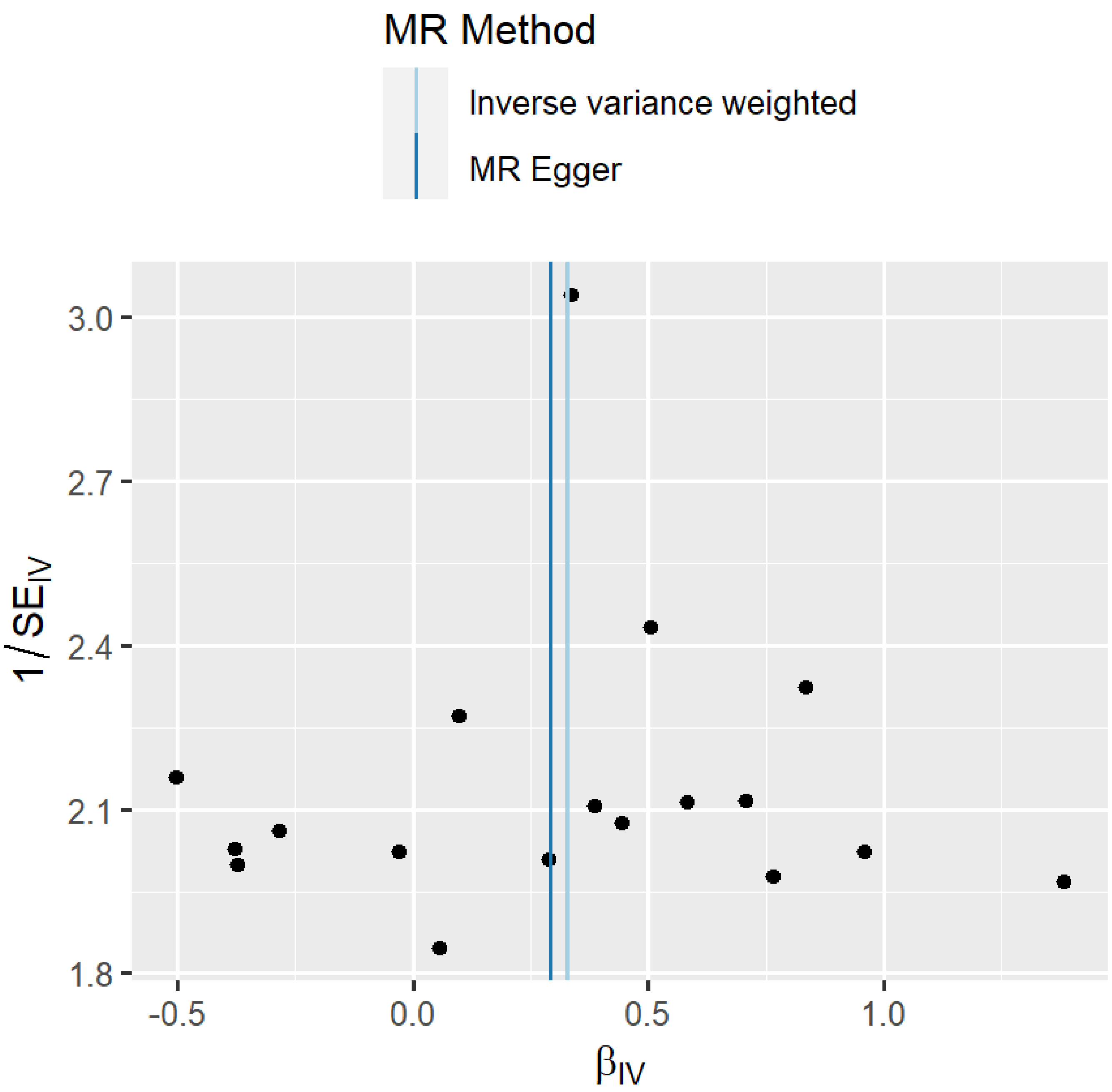
MR Method
Inverse variance weighted
MR Egger
3.0
2.7
2.4
2.1
1.8
1/SE_IV
-0.5
0.0
0.5
1.0
β_IV

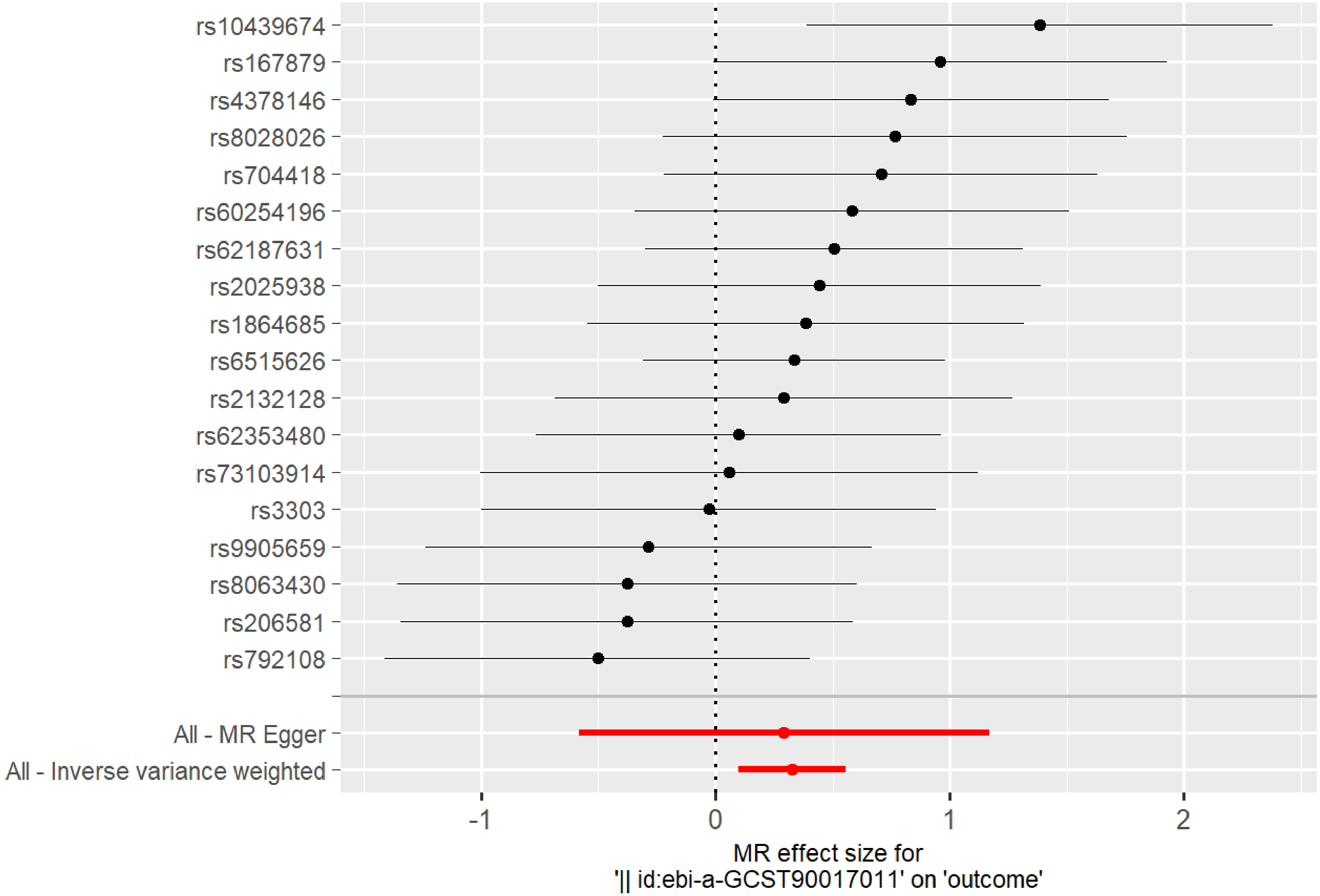
rs10439674
rs167879
rs4378146
rs8028026
rs704418
rs60254196
rs62187631
rs2025938
rs1864685
rs6515626
rs2132128
rs62353480
rs73103914
rs3303
rs9905659
rs8063430
rs206581
rs792108
All - MR Egger
All - Inverse variance weighted
-1
0
1
2
MR effect size for
'|| id:ebi-a-GCST90017011' on 'outcome'

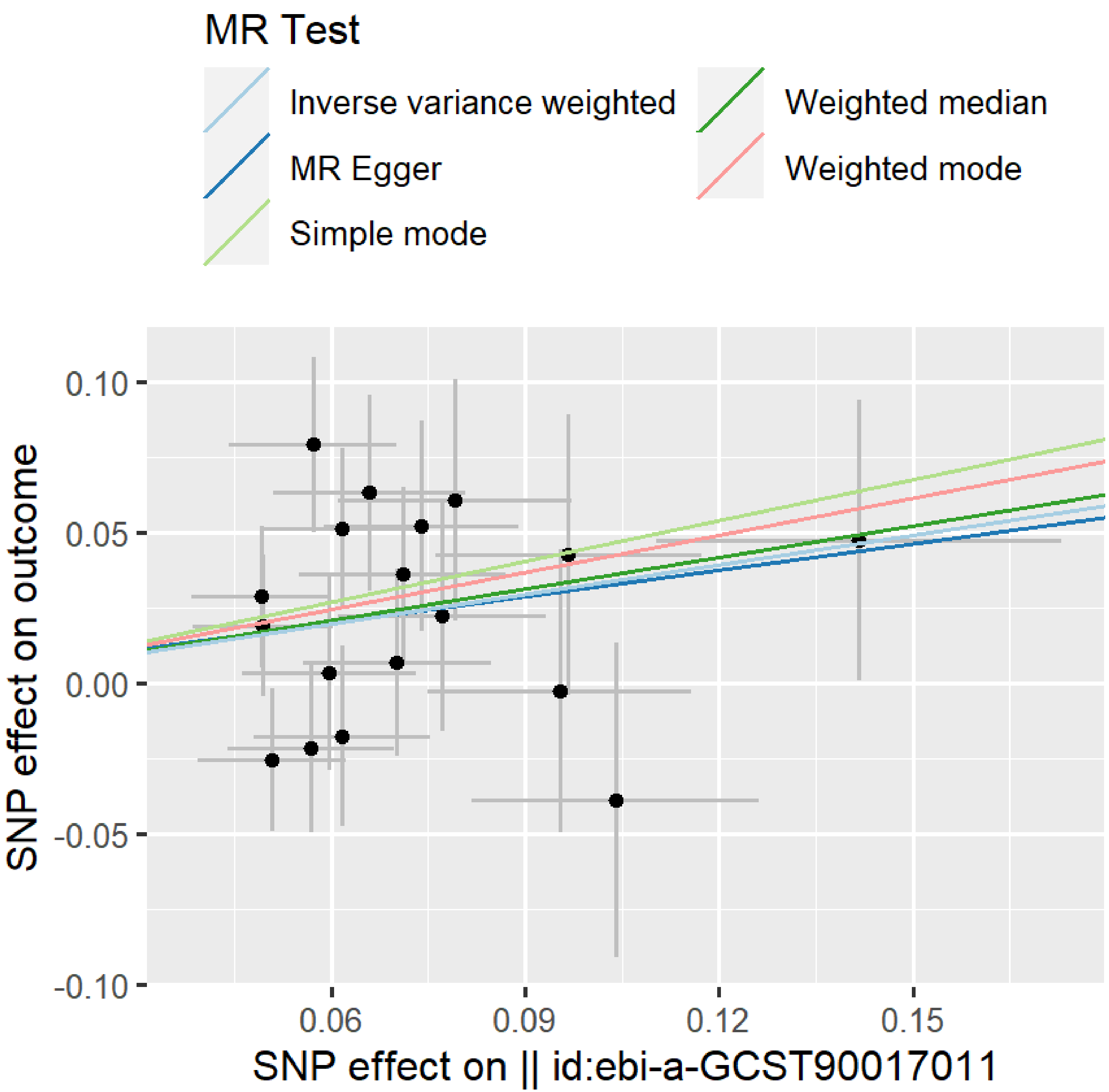
MR Test
Inverse variance weighted
MR Egger
Simple mode
Weighted median
Weighted mode
0.10
0.05
0.00
-0.05
-0.10
SNP effect on outcome
0.06
0.09
0.12
0.15
SNP effect on || id:ebi-a-GCST90017011

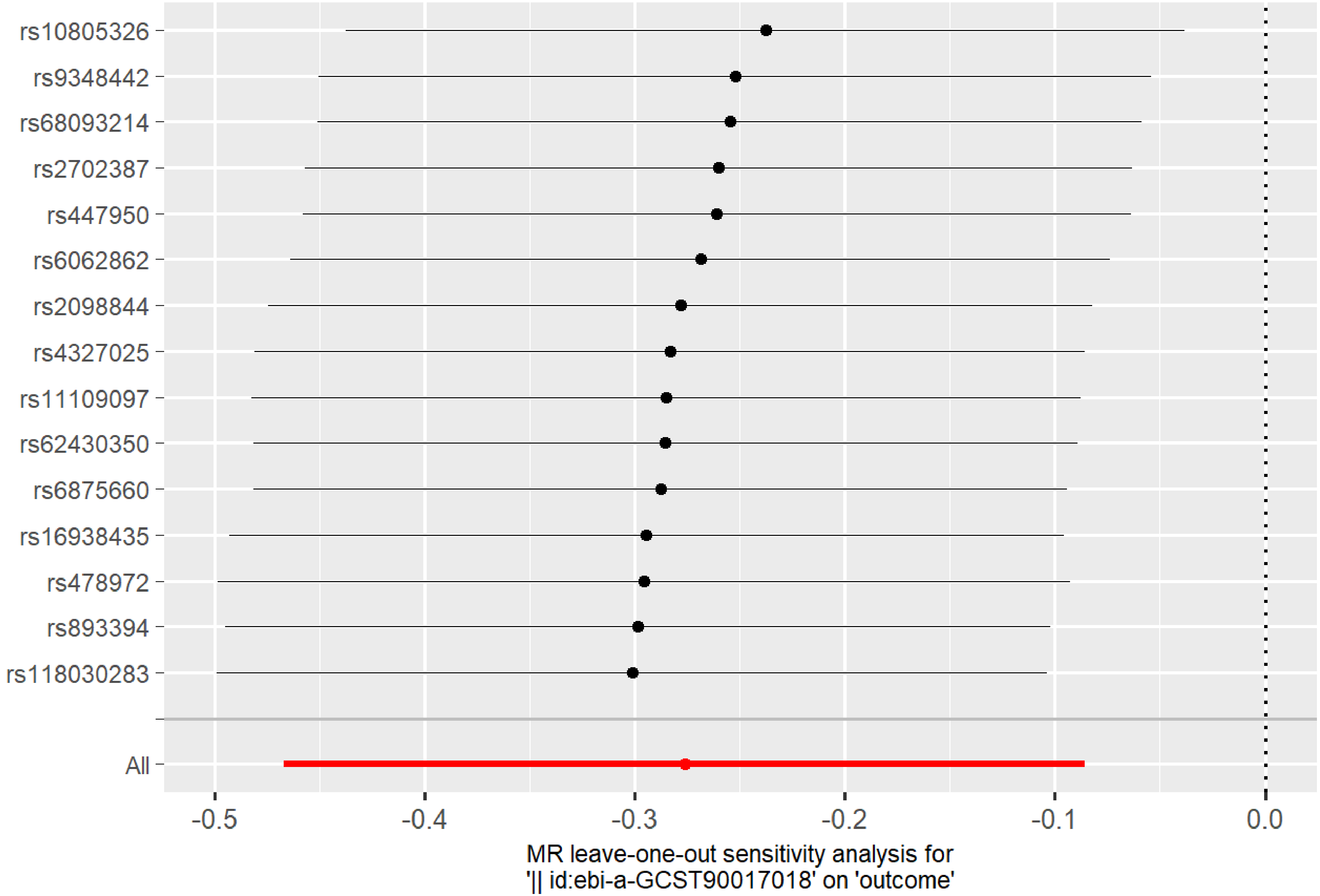

rs10805326
rs9348442
rs68093214
rs2702387
rs447950
rs6062862
rs2098844
rs4327025
rs11109097
rs62430350
rs6875660
rs16938435
rs478972
rs893394
rs118030283
All
-0.5
-0.4
-0.3
-0.2
-0.1
0.0
MR leave-one-out sensitivity analysis for
'|| id:ebi-a-GCST90017018' on 'outcome'

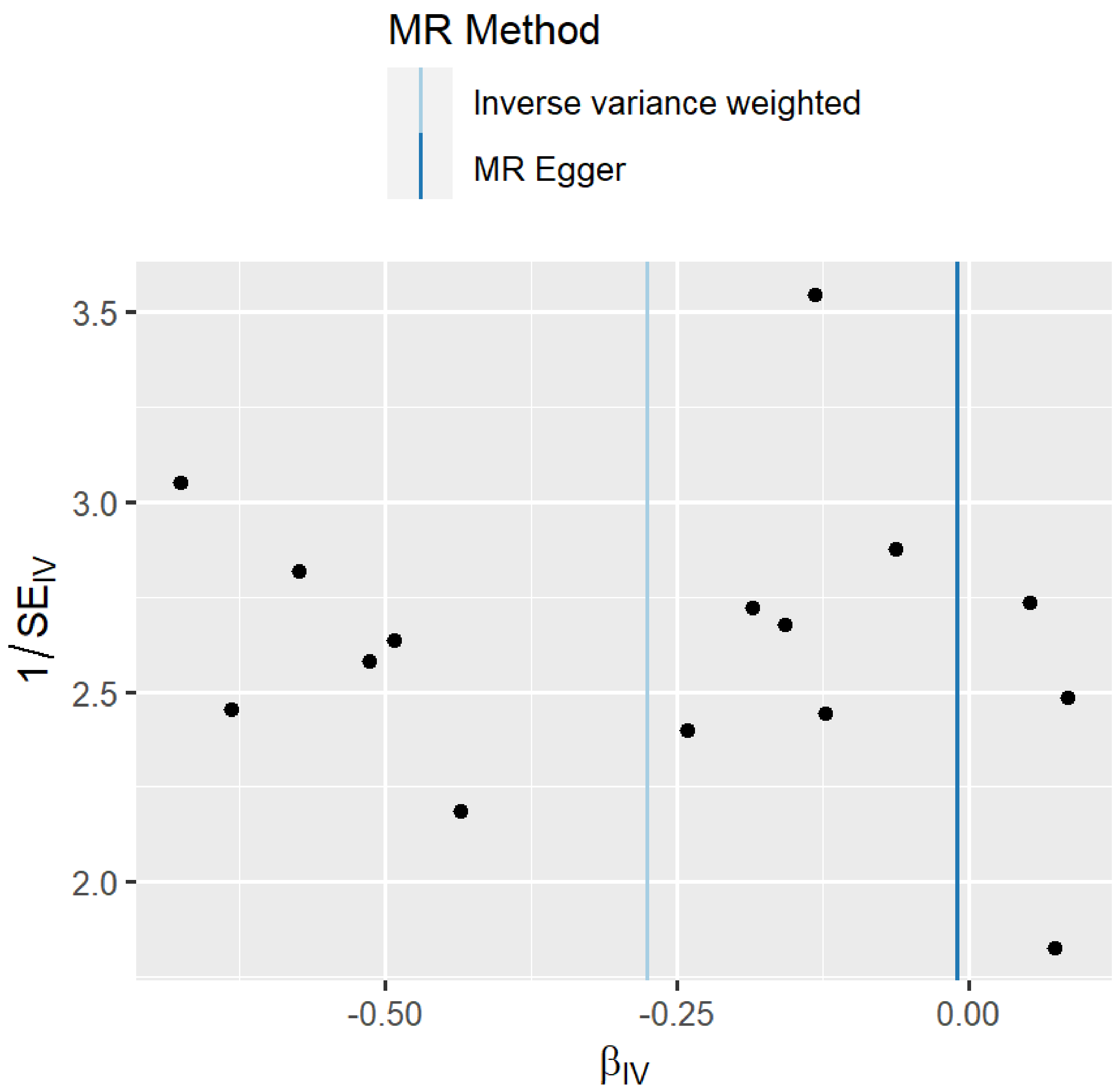
MR Method
Inverse variance weighted
MR Egger
3.5
3.0
2.5
2.0
1/SE_IV
-0.50
-0.25
0.00
β_IV

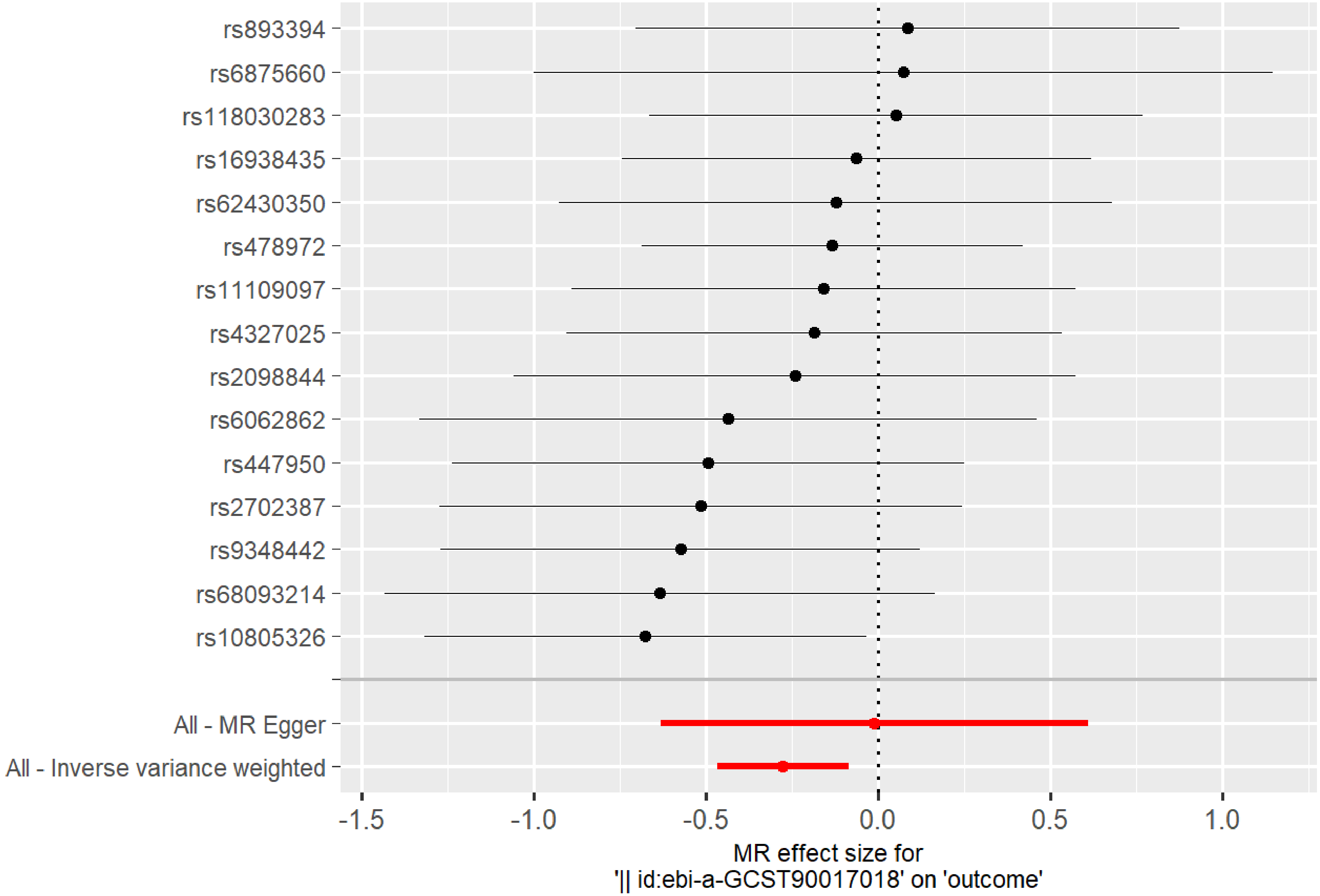
rs893394
rs6875660
rs118030283
rs16938435
rs62430350
rs478972
rs11109097
rs4327025
rs2098844
rs6062862
rs447950
rs2702387
rs9348442
rs68093214
rs10805326
All - MR Egger
All - Inverse variance weighted
-1.5
-1.0
-0.5
0.0
0.5
1.0
MR effect size for
'|| id:ebi-a-GCST90017018' on 'outcome'

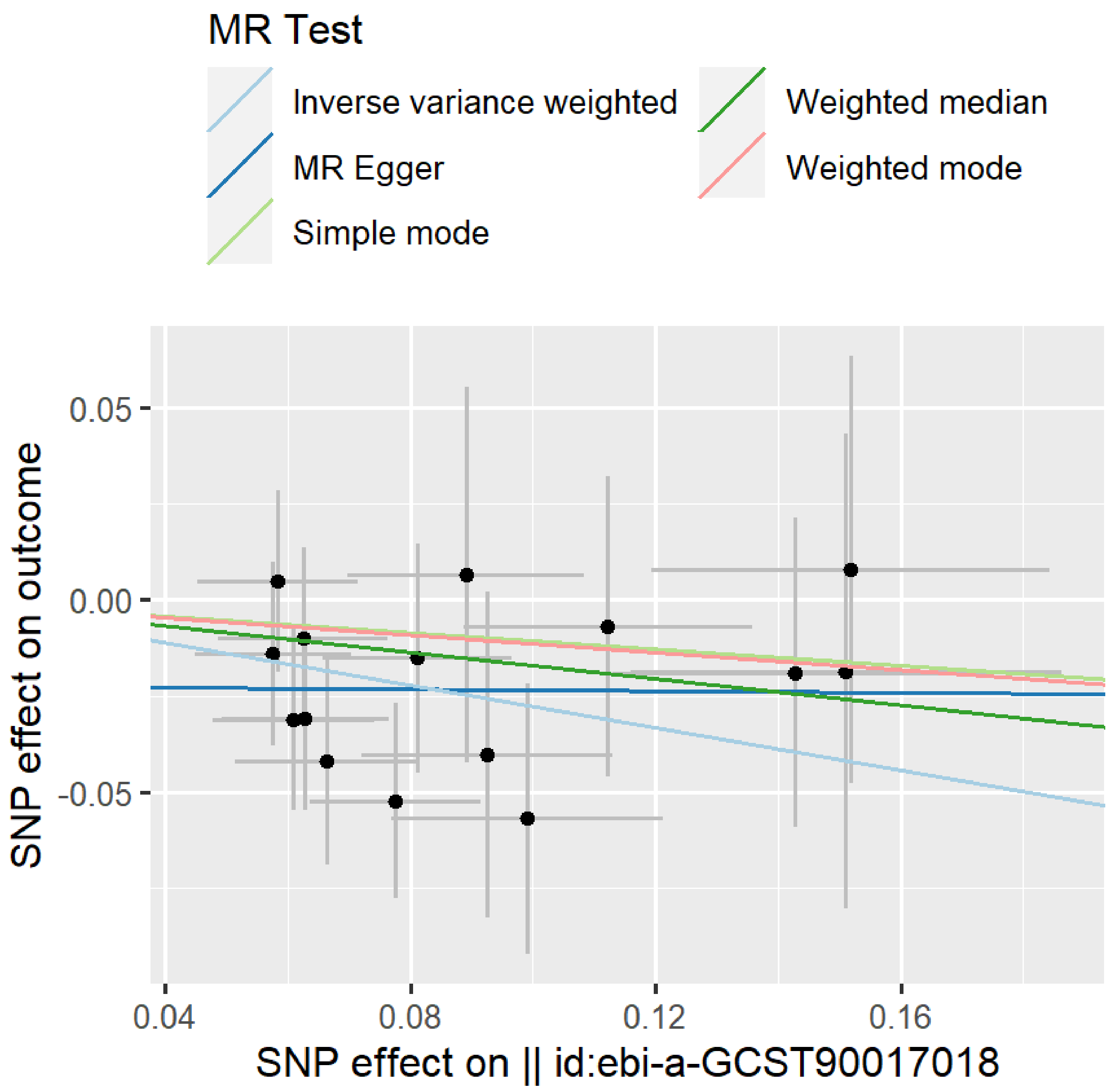
MR Test
Inverse variance weighted
MR Egger
Simple mode
Weighted median
Weighted mode
0.05
0.00
-0.05
SNP effect on outcome
0.04
0.08
0.12
0.16
SNP effect on || id:ebi-a-GCST90017018

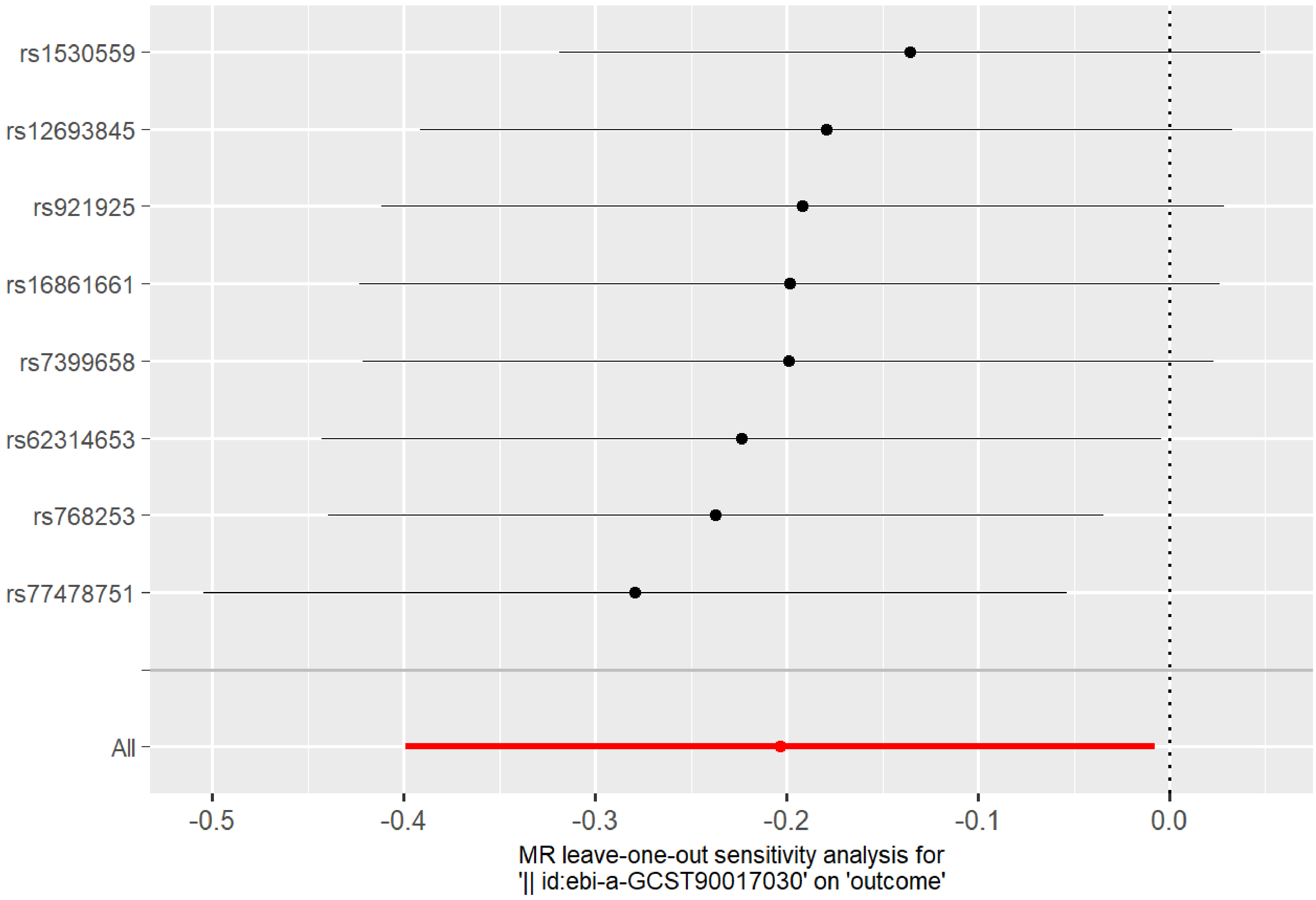
rs1530559
rs12693845
rs921925
rs16861661
rs7399658
rs62314653
rs768253
rs77478751
All
-0.5
-0.4
-0.3
-0.2
-0.1
0.0
MR leave-one-out sensitivity analysis for
'|| id:ebi-a-GCST90017030' on 'outcome'

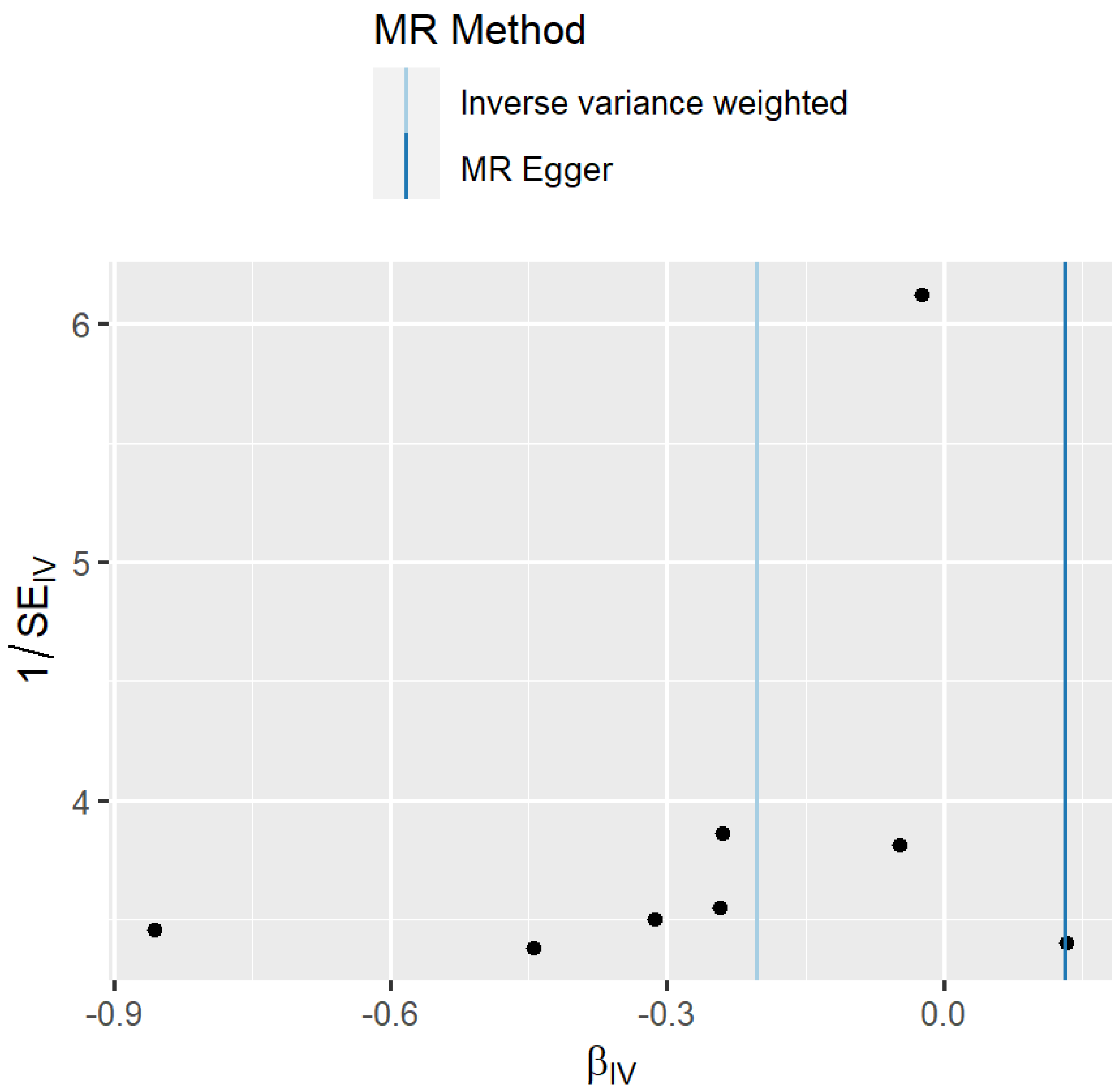

MR Method
Inverse variance weighted
MR Egger
6
5
4
-0.9
-0.6
-0.3
0.0
1/SE_IV
β_IV

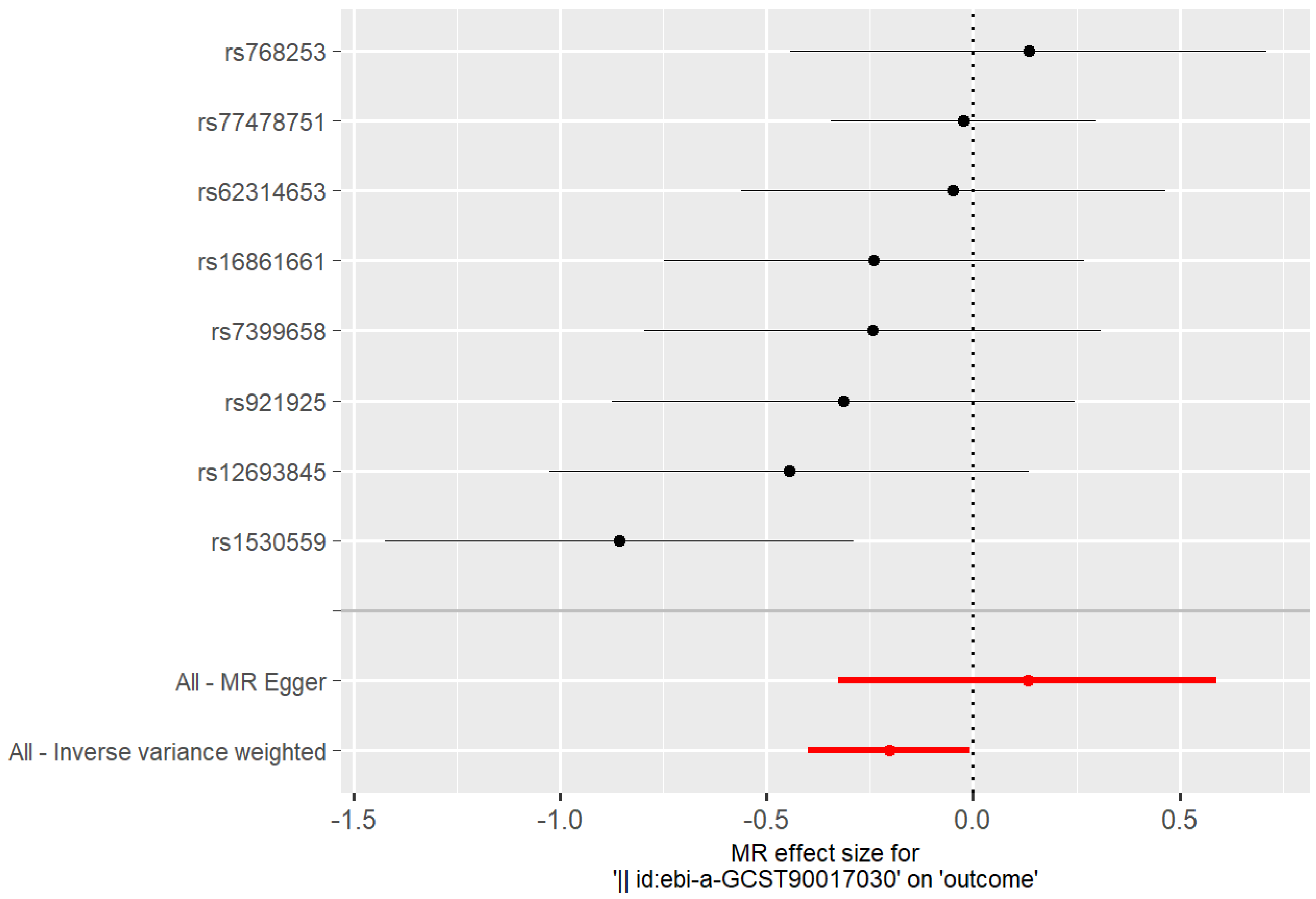
rs768253
rs77478751
rs62314653
rs16861661
rs7399658
rs921925
rs12693845
rs1530559
All - MR Egger
All - Inverse variance weighted
-1.5
-1.0
-0.5
0.0
0.5
MR effect size for
'|| id:ebi-a-GCST90017030' on 'outcome'

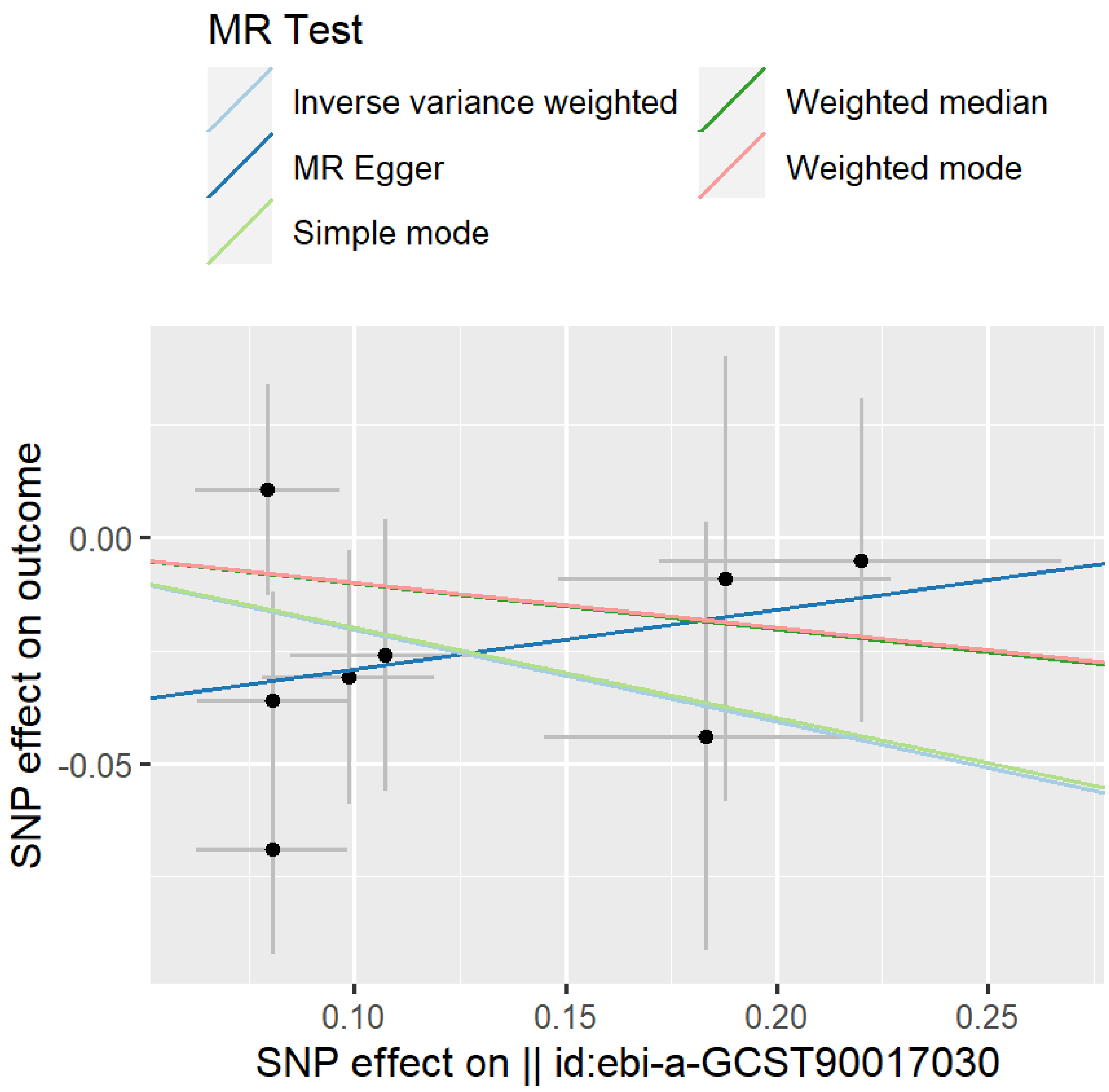
MR Test
Inverse variance weighted
MR Egger
Simple mode
Weighted median
Weighted mode
SNP effect on outcome
0.00
-0.05
0.10
0.15
0.20
0.25
SNP effect on || id:ebi-a-GCST90017030

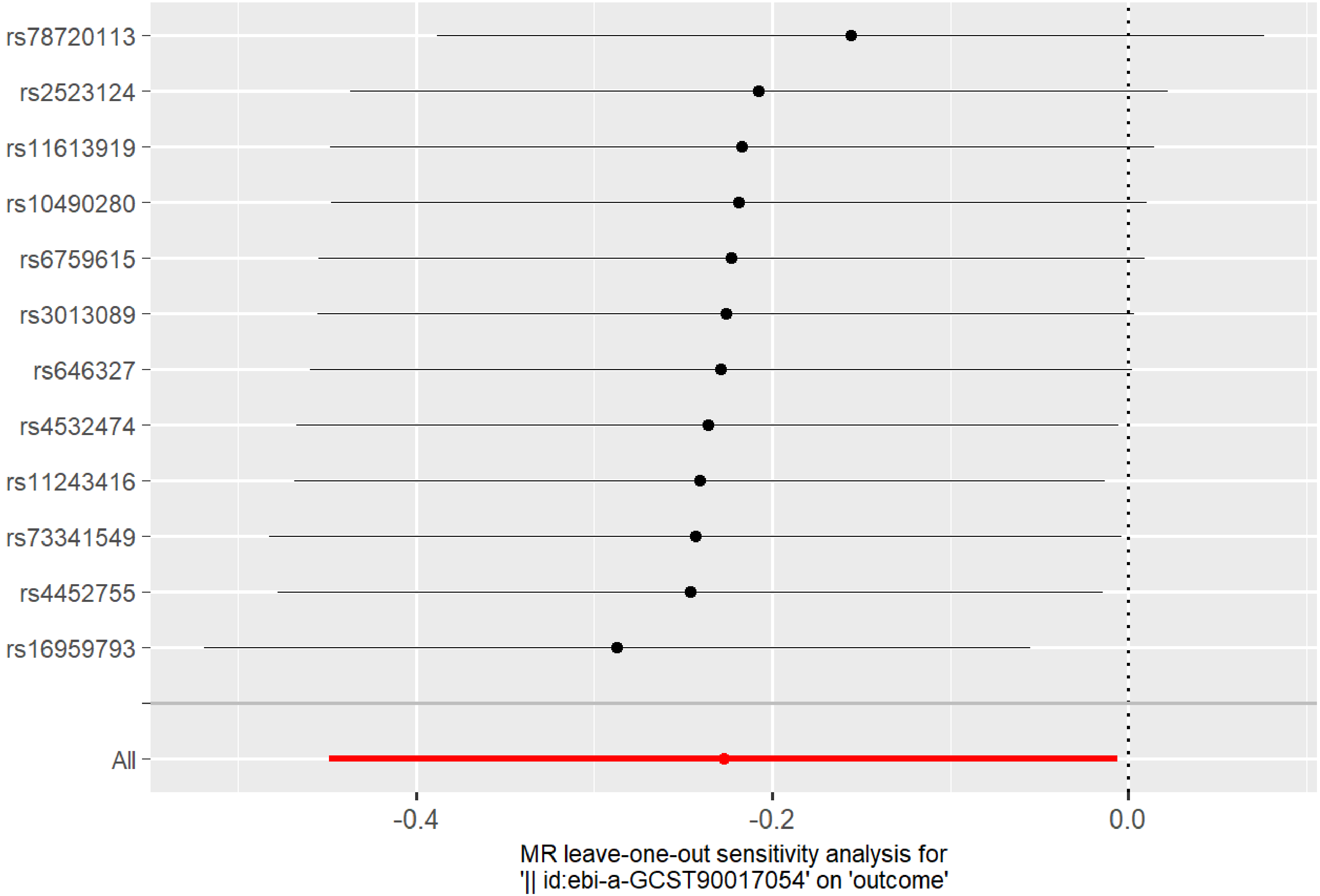

rs78720113
rs2523124
rs11613919
rs10490280
rs6759615
rs3013089
rs646327
rs4532474
rs11243416
rs73341549
rs4452755
rs16959793
All
-0.4
-0.2
0.0
MR leave-one-out sensitivity analysis for
'|| id:ebi-a-GCST90017054' on 'outcome'

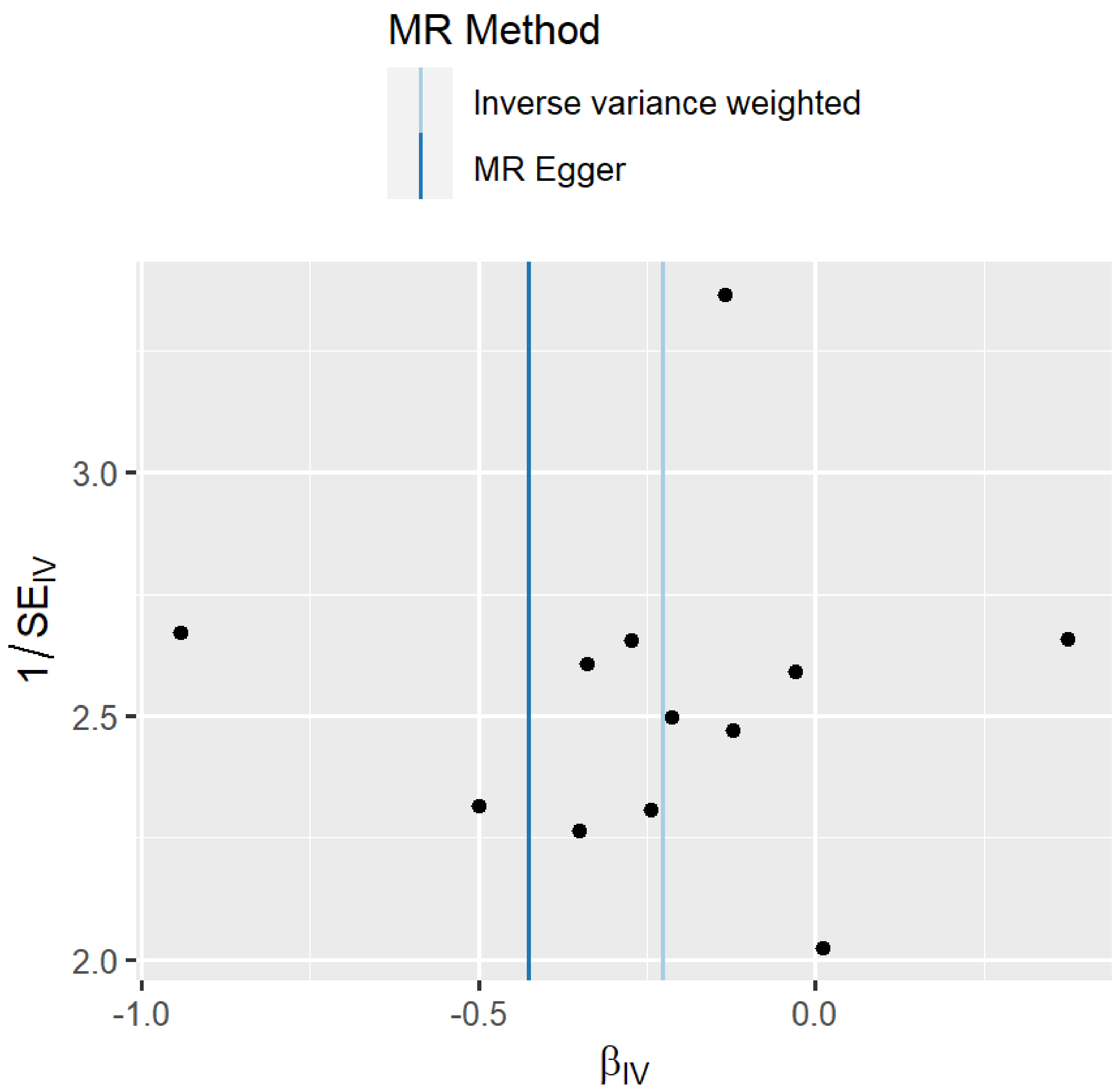
MR Method
Inverse variance weighted
MR Egger
3.0
2.5
2.0
-1.0
-0.5
0.0
1/SE_IV
β_IV

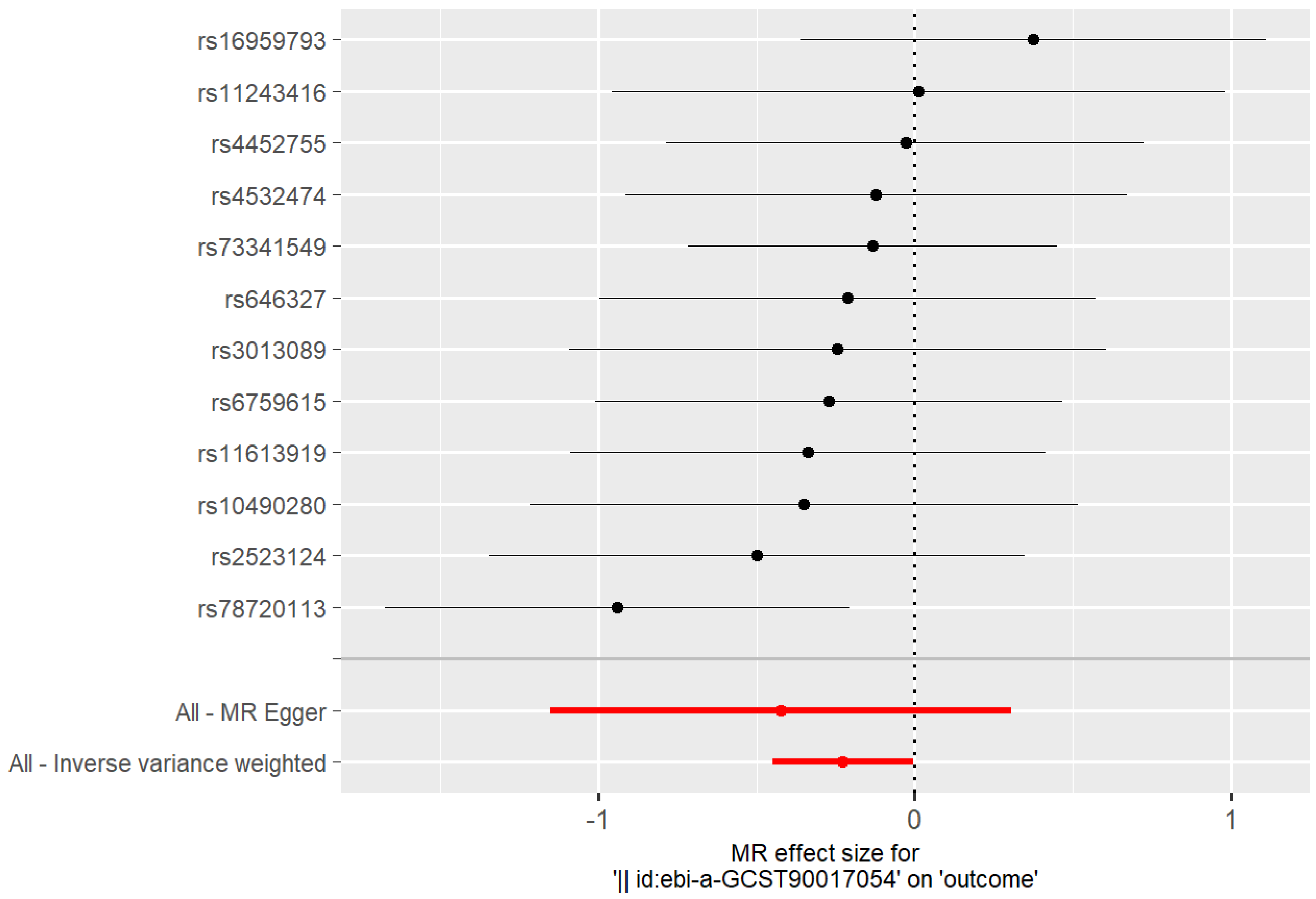
rs16959793
rs11243416
rs4452755
rs4532474
rs73341549
rs646327
rs3013089
rs6759615
rs11613919
rs10490280
rs2523124
rs78720113
All - MR Egger
All - Inverse variance weighted
-1
0
1
MR effect size for
'|| id:ebi-a-GCST90017054' on 'outcome'

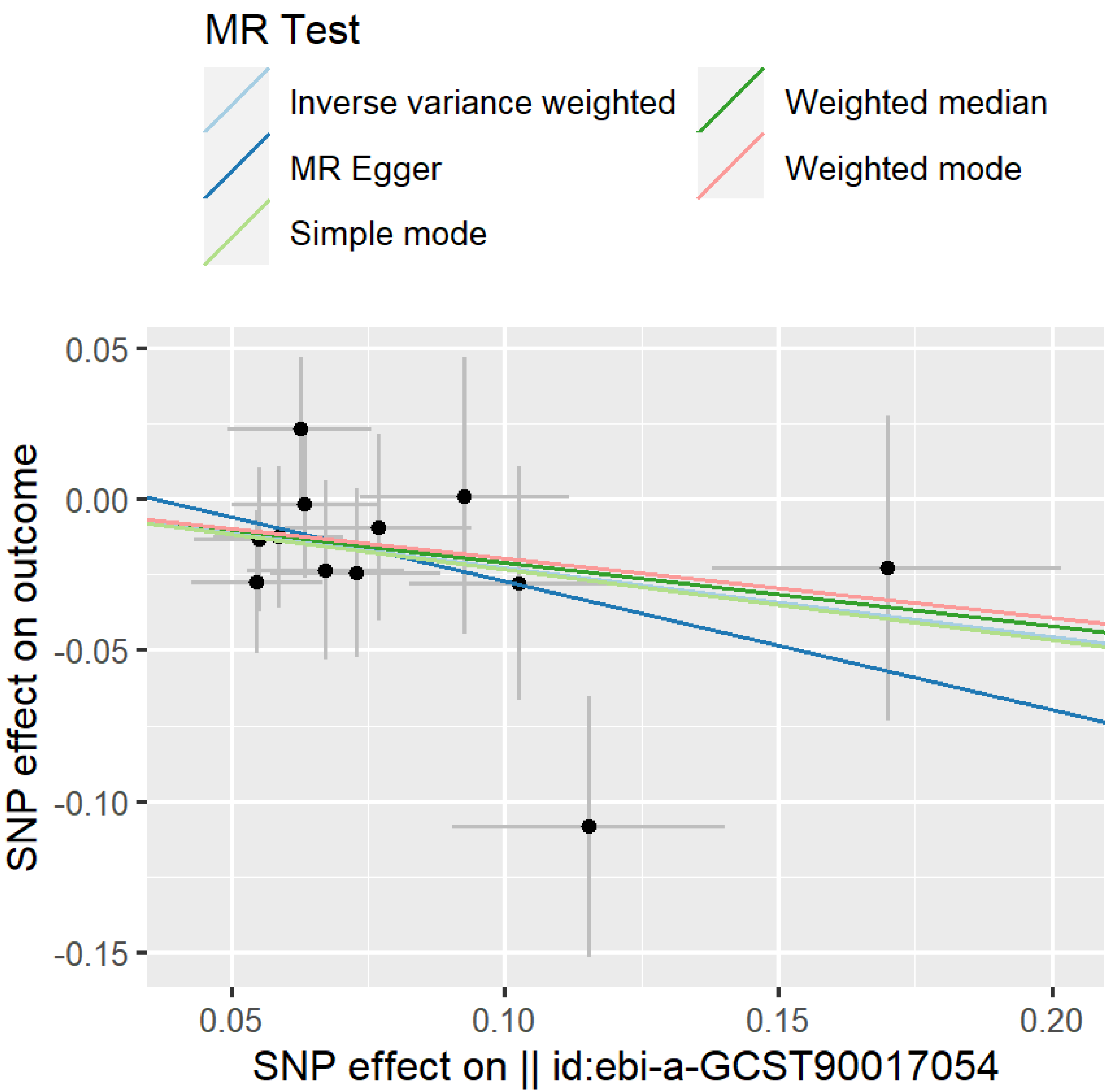
MR Test
Inverse variance weighted
MR Egger
Simple mode
Weighted median
Weighted mode
0.05
0.00
-0.05
-0.10
-0.15
SNP effect on outcome
0.05
0.10
0.15
0.20
SNP effect on || id:ebi-a-GCST90017054

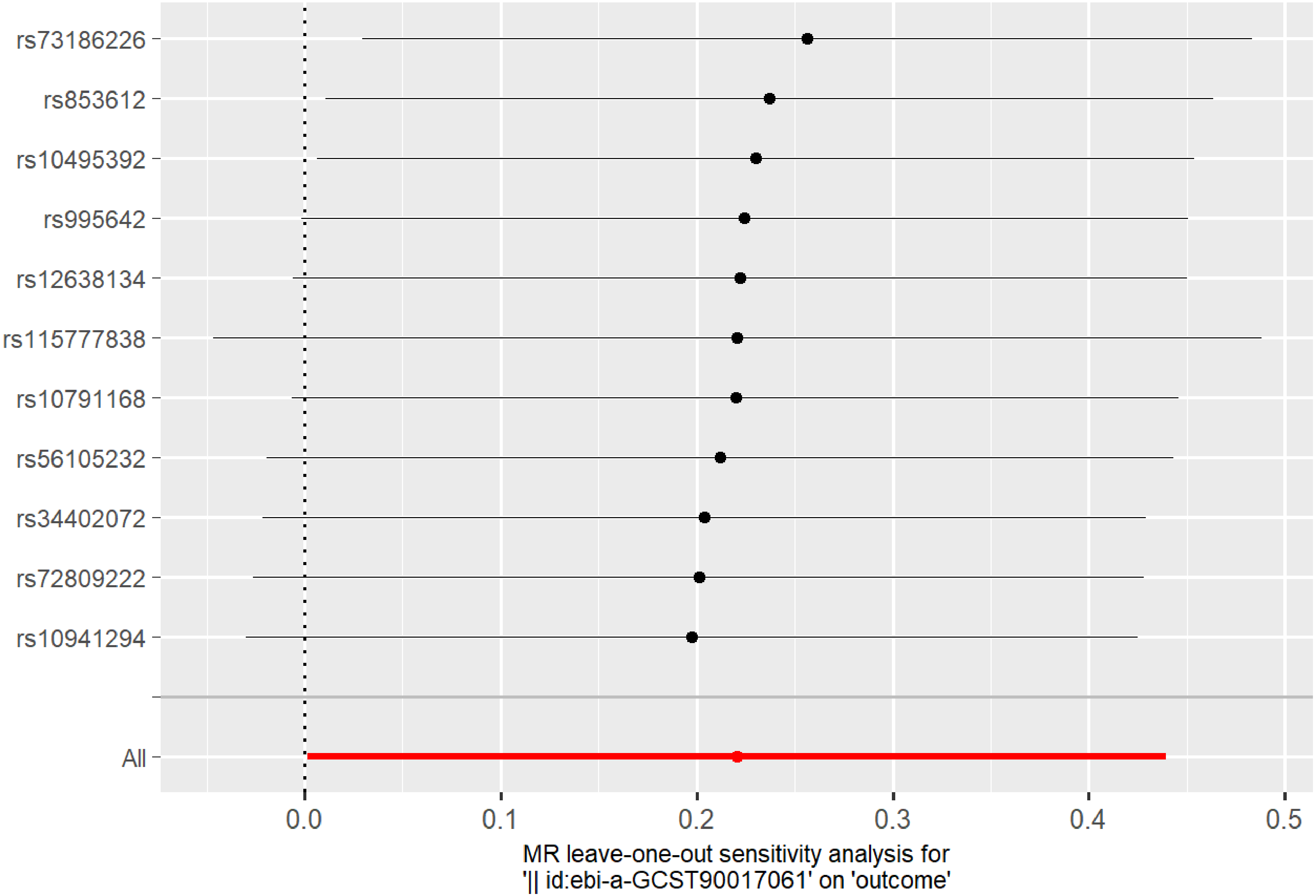
rs73186226
rs853612
rs10495392
rs995642
rs12638134
rs115777838
rs10791168
rs56105232
rs34402072
rs72809222
rs10941294
All
0.0
0.1
0.2
0.3
0.4
0.5
MR leave-one-out sensitivity analysis for
'|| id:ebi-a-GCST90017061' on 'outcome'

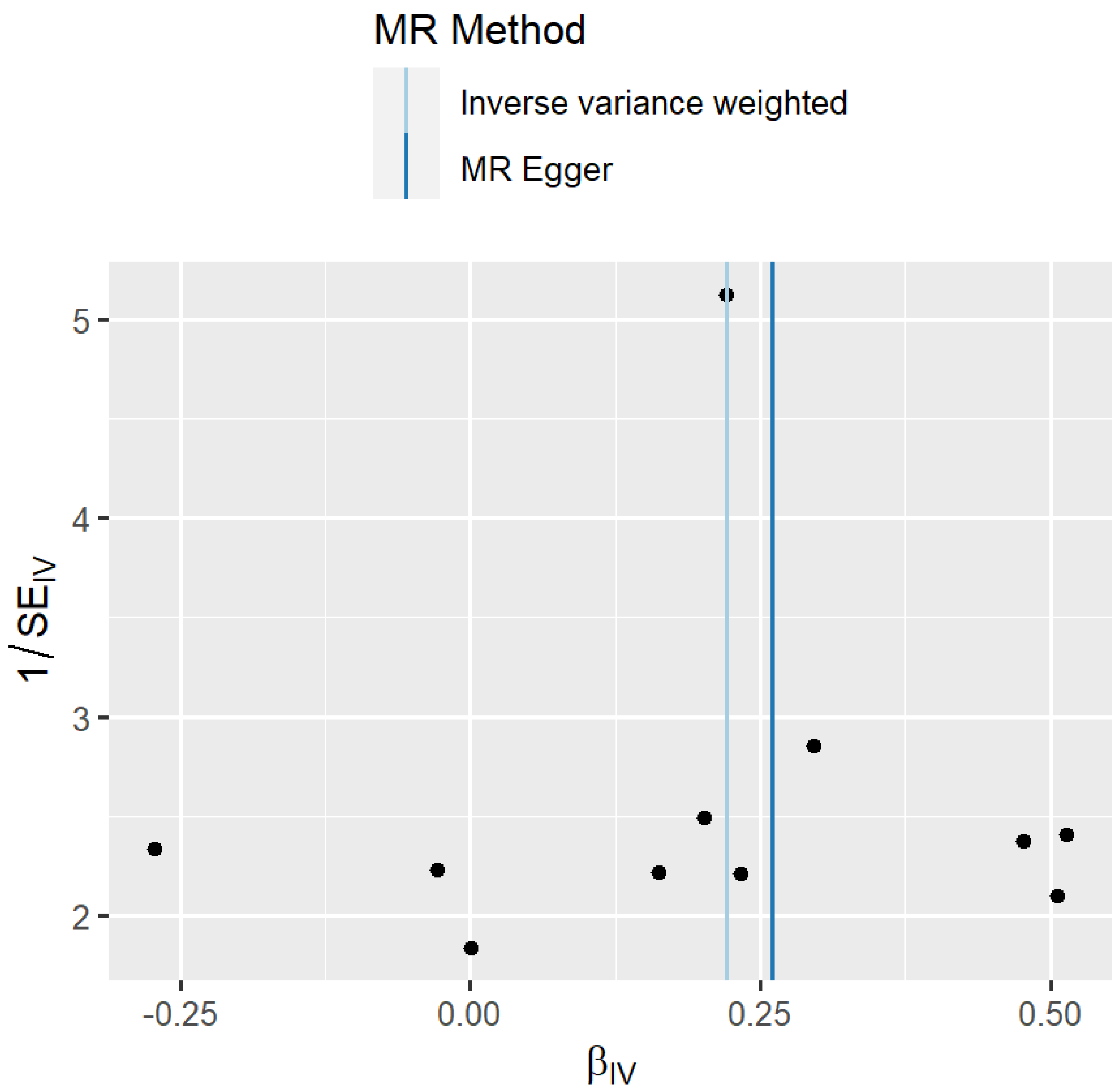
MR Method
Inverse variance weighted
MR Egger
5
4
3
2
1/SE$_{IV}$
-0.25
0.00
0.25
0.50
β$_{IV}$

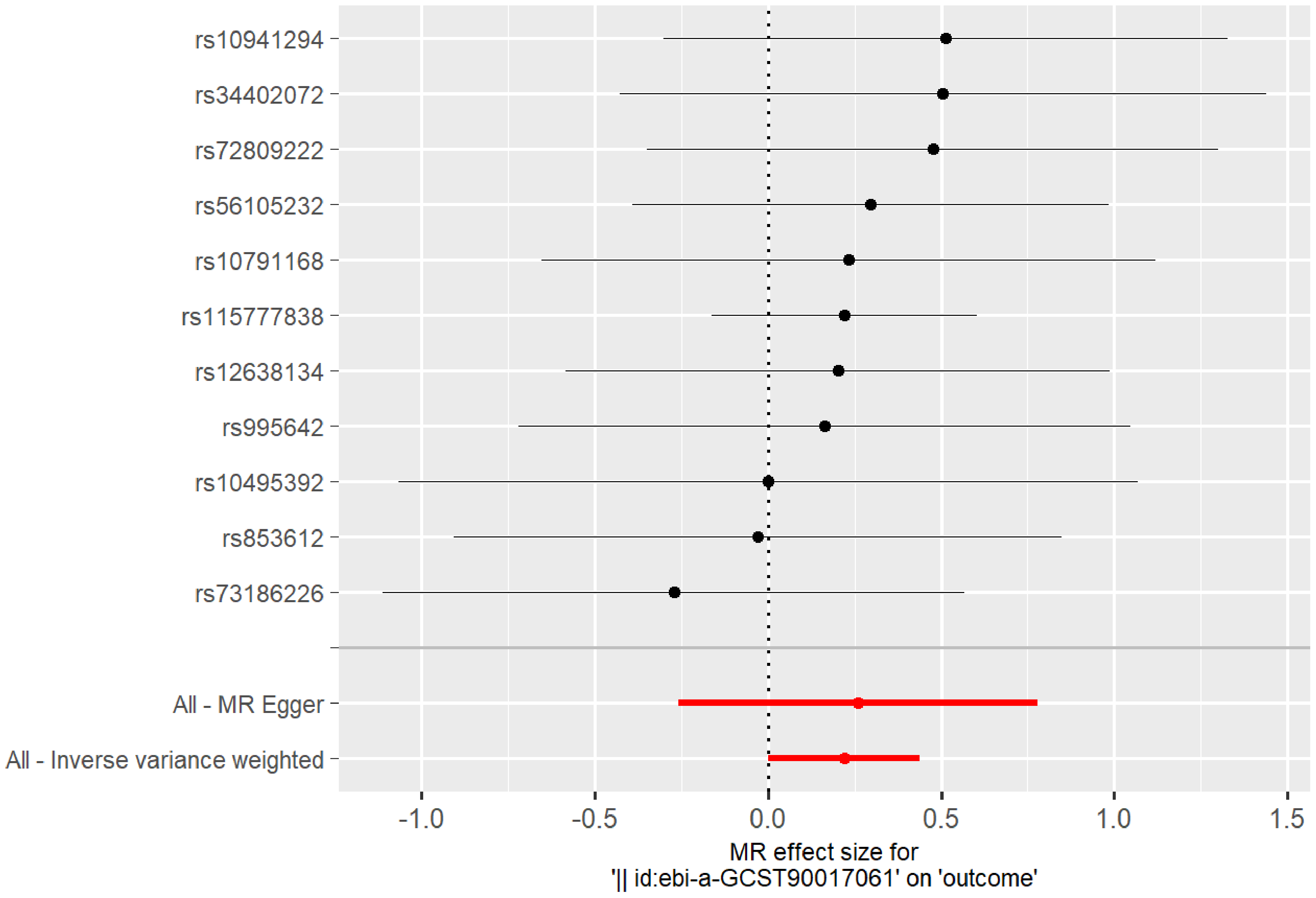
rs10941294
rs34402072
rs72809222
rs56105232
rs10791168
rs115777838
rs12638134
rs995642
rs10495392
rs853612
rs73186226
All - MR Egger
All - Inverse variance weighted
-1.0
-0.5
0.0
0.5
1.0
1.5
MR effect size for
'|| id:ebi-a-GCST90017061' on 'outcome'

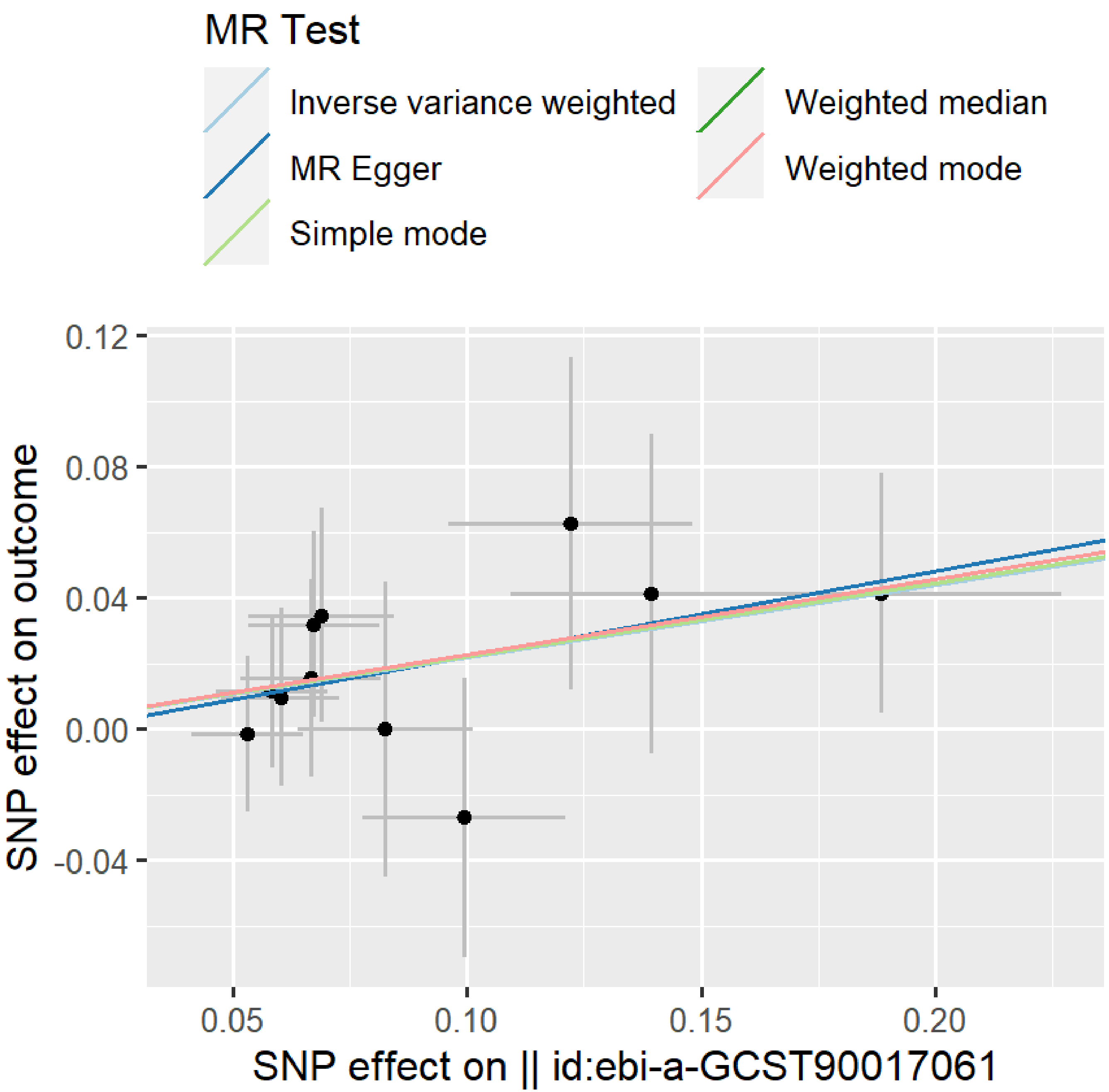
MR Test
Inverse variance weighted
MR Egger
Simple mode
Weighted median
Weighted mode
0.12
0.08
0.04
0.00
-0.04
SNP effect on outcome
0.05
0.10
0.15
0.20
SNP effect on || id:ebi-a-GCST90017061

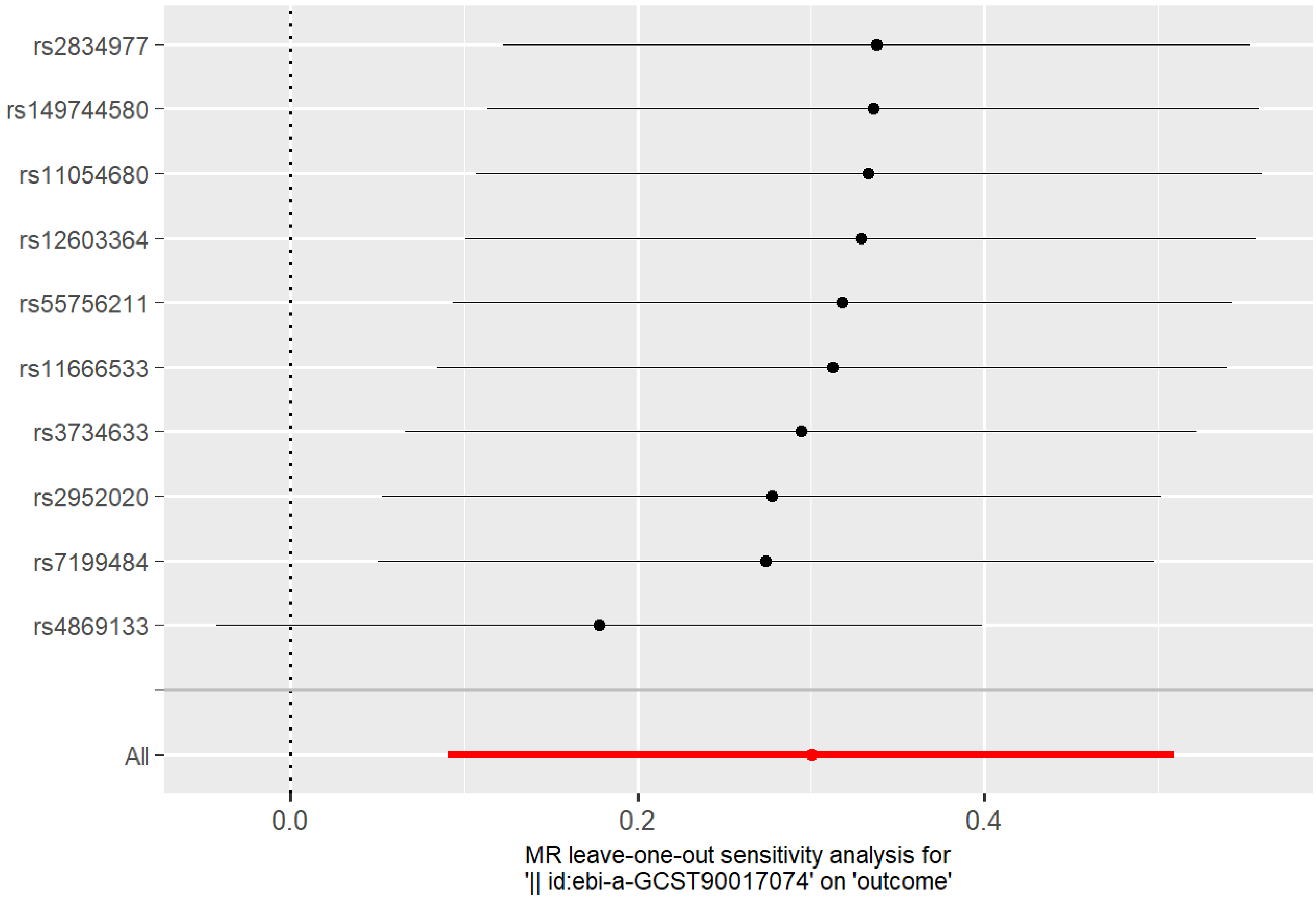
rs2834977
rs149744580
rs11054680
rs12603364
rs55756211
rs11666533
rs3734633
rs2952020
rs7199484
rs4869133
All
0.0
0.2
0.4
MR leave-one-out sensitivity analysis for
'|| id:ebi-a-GCST90017074' on 'outcome'

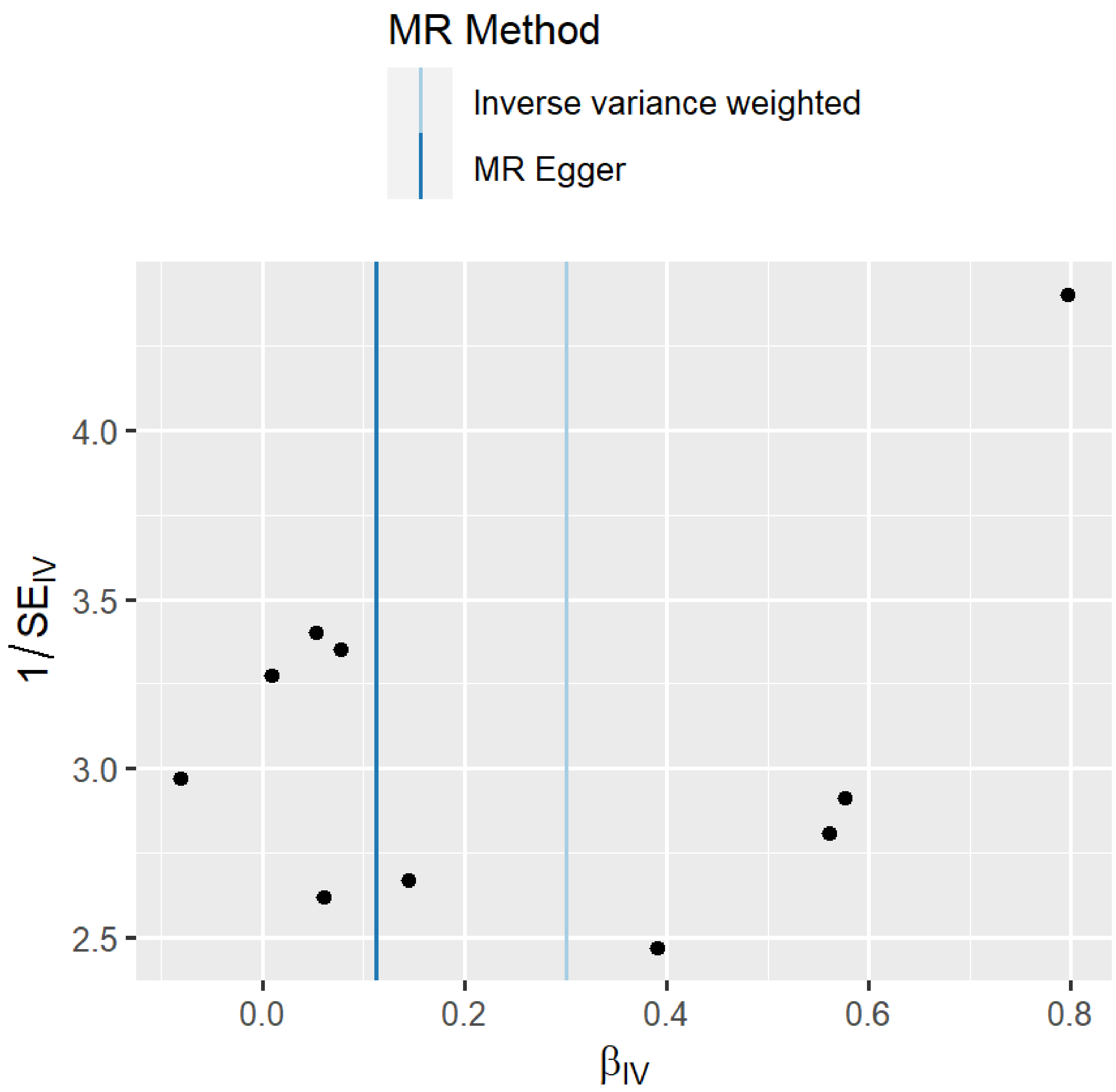
MR Method
Inverse variance weighted
MR Egger
4.0
3.5
3.0
2.5
1/SE_IV
0.0
0.2
0.4
0.6
0.8
β_IV

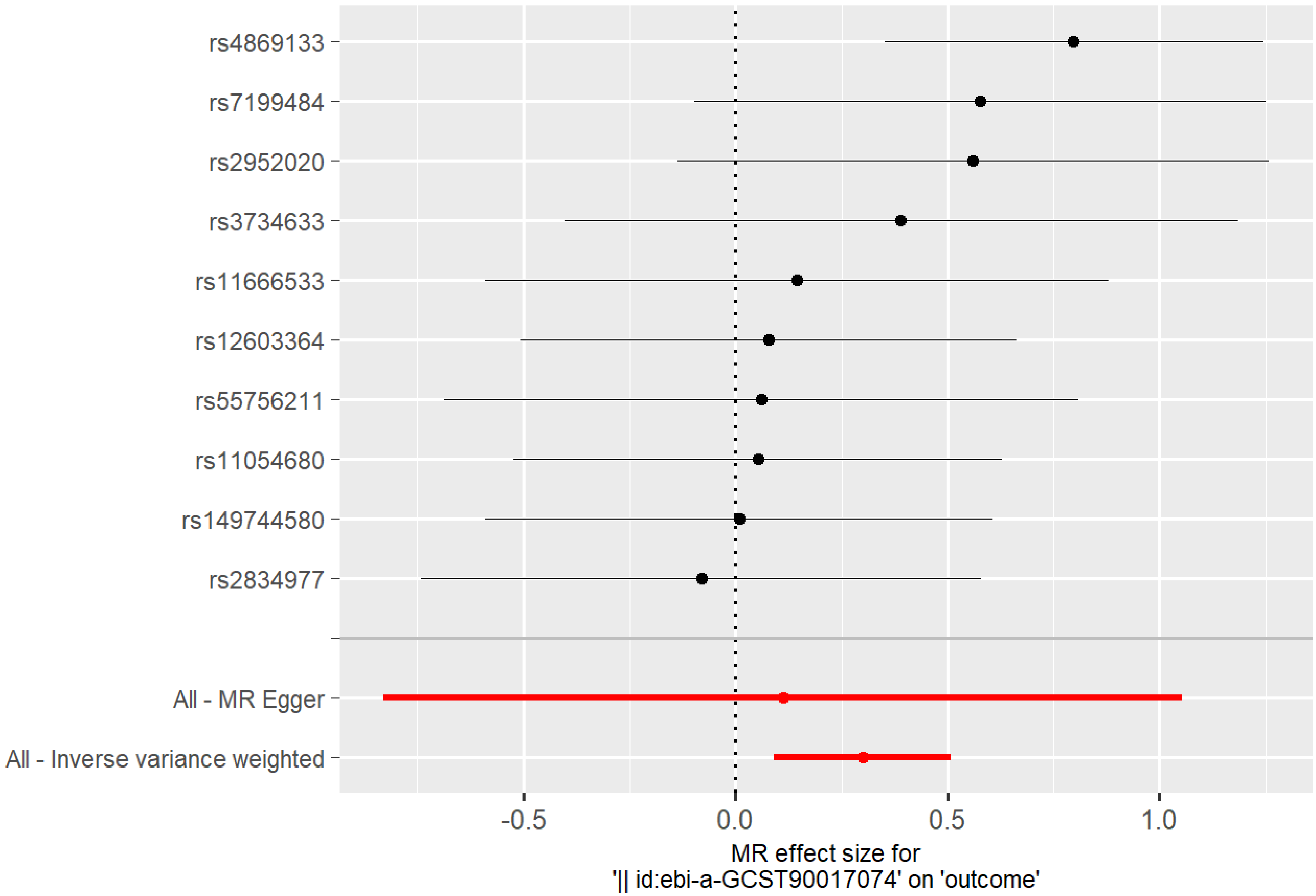
rs4869133
rs7199484
rs2952020
rs3734633
rs11666533
rs12603364
rs55756211
rs11054680
rs149744580
rs2834977
All - MR Egger
All - Inverse variance weighted
-0.5
0.0
0.5
1.0
MR effect size for
'|| id:ebi-a-GCST90017074' on 'outcome'

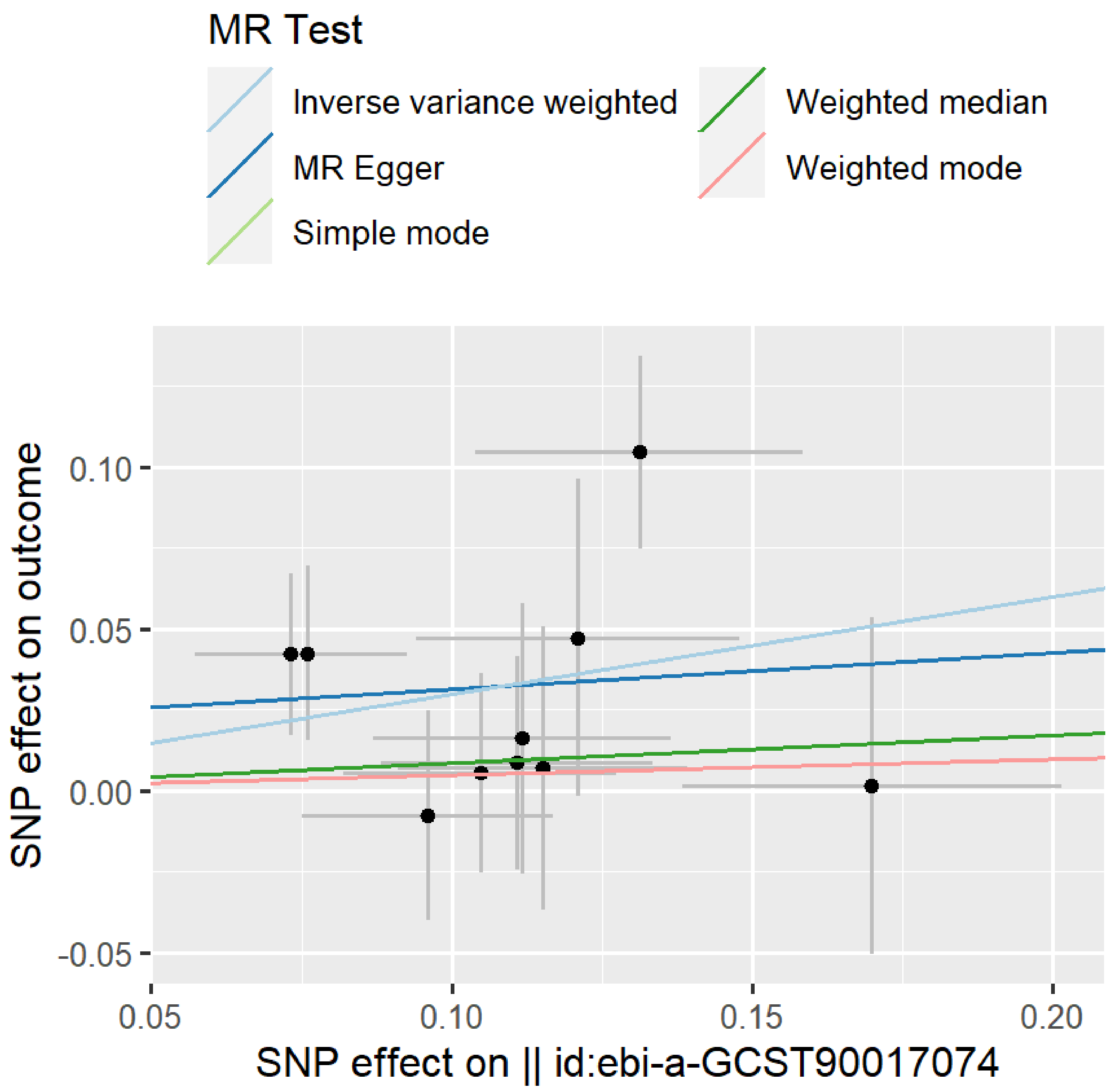
MR Test
Inverse variance weighted
MR Egger
Simple mode
Weighted median
Weighted mode
SNP effect on outcome
0.10
0.05
0.00
-0.05
0.05
0.10
0.15
0.20
SNP effect on || id:ebi-a-GCST90017074

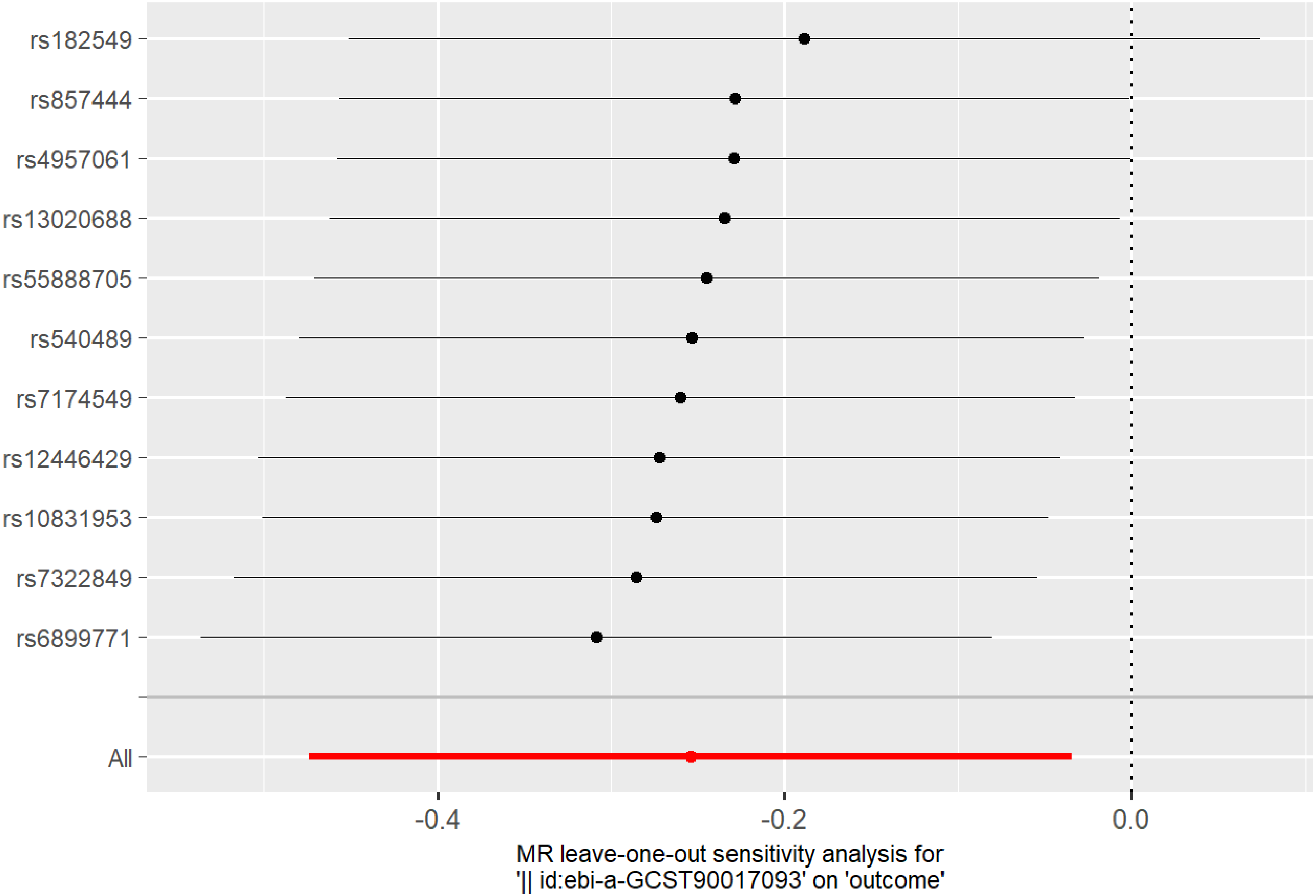
rs182549
rs857444
rs4957061
rs13020688
rs55888705
rs540489
rs7174549
rs12446429
rs10831953
rs7322849
rs6899771
All
-0.4
-0.2
0.0
MR leave-one-out sensitivity analysis for
'|| id:ebi-a-GCST90017093' on 'outcome'

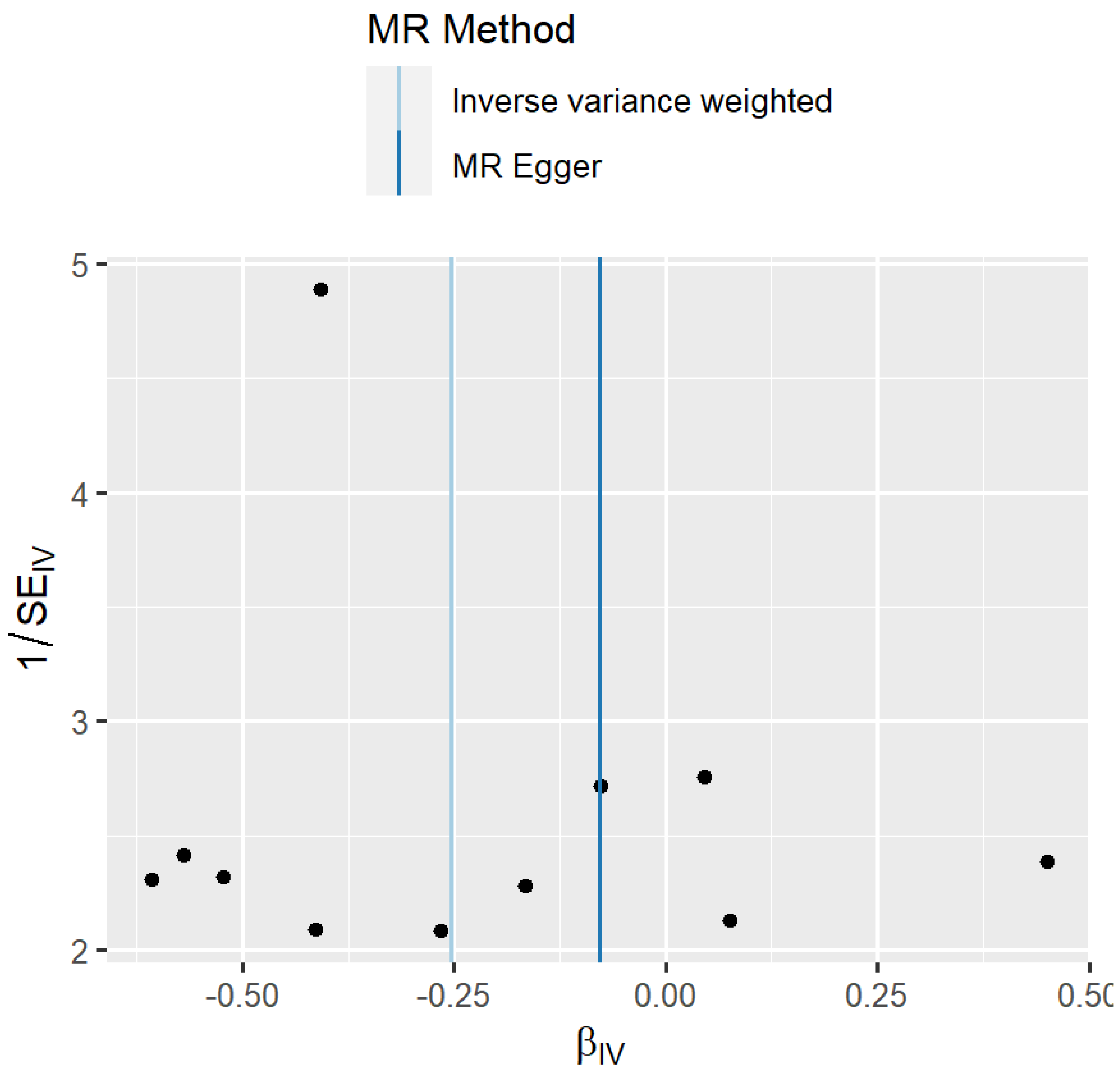
MR Method
Inverse variance weighted
MR Egger
5
4
3
2
1/SE$_{IV}$
-0.50
-0.25
0.00
0.25
0.50
β$_{IV}$

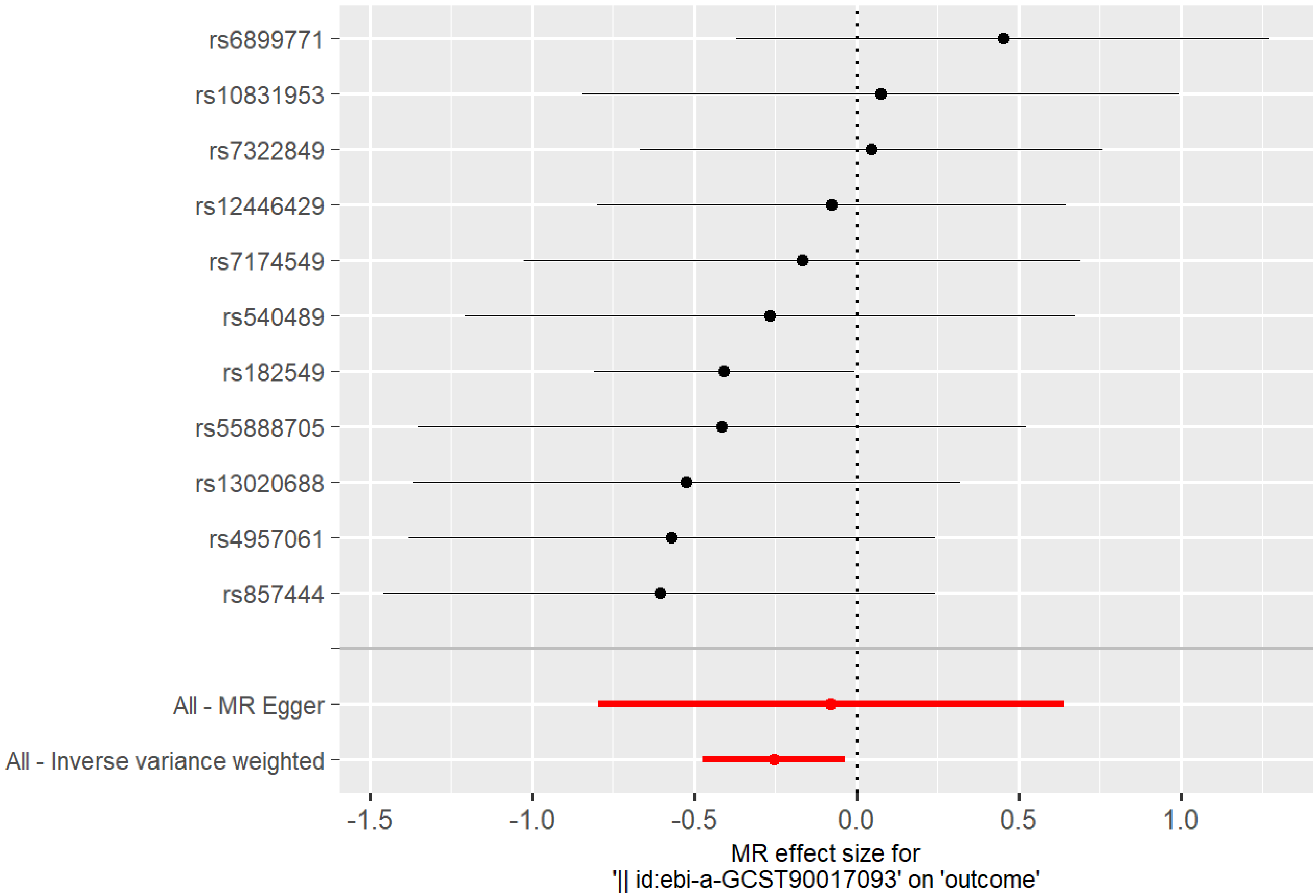
rs6899771
rs10831953
rs7322849
rs12446429
rs7174549
rs540489
rs182549
rs55888705
rs13020688
rs4957061
rs857444
All - MR Egger
All - Inverse variance weighted
-1.5
-1.0
-0.5
0.0
0.5
1.0
MR effect size for
'|| id:ebi-a-GCST90017093' on 'outcome'

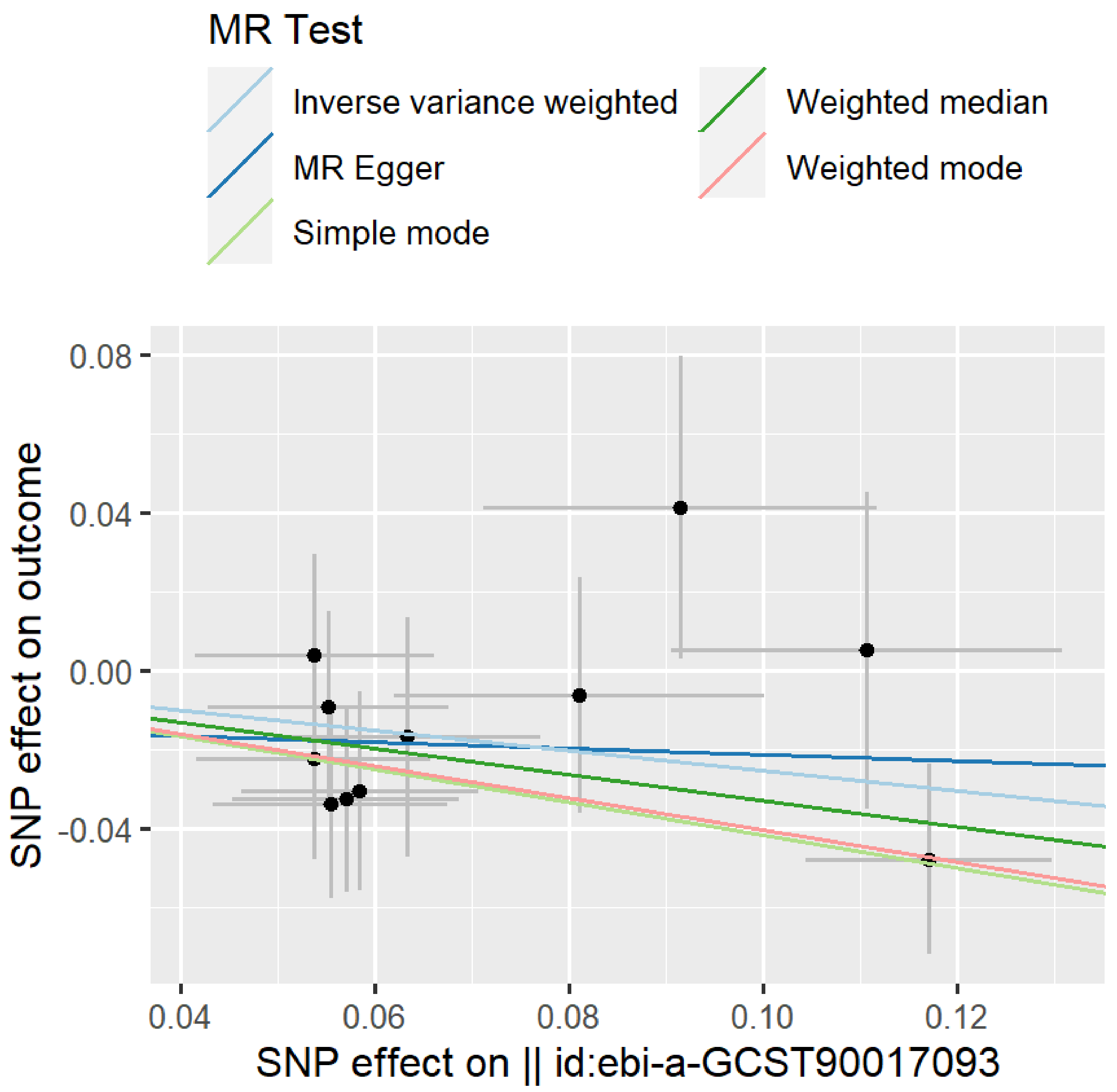
MR Test
Inverse variance weighted
MR Egger
Simple mode
Weighted median
Weighted mode
SNP effect on outcome
0.08
0.04
0.00
-0.04
0.04
0.06
0.08
0.10
0.12
SNP effect on || id:ebi-a-GCST90017093

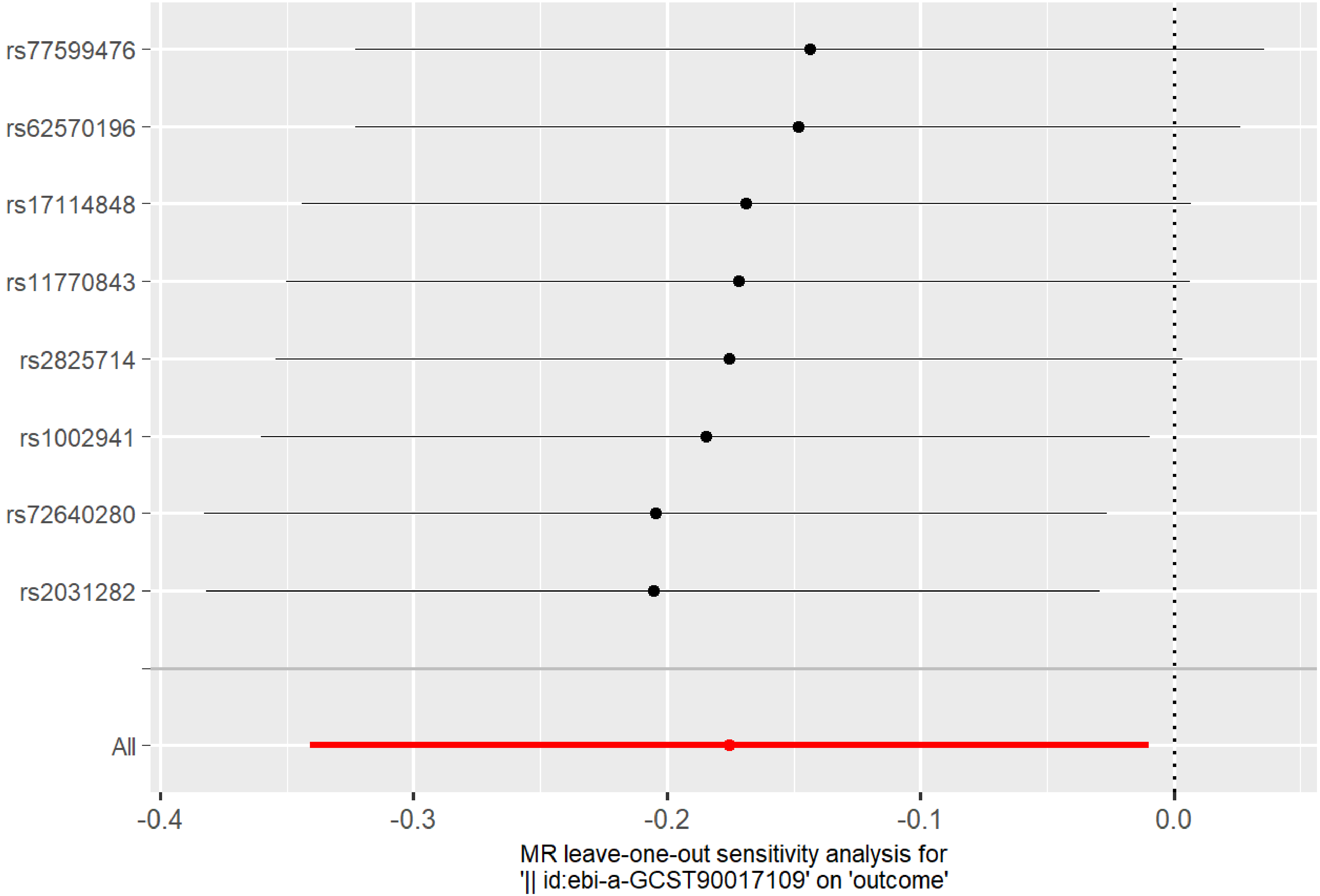
rs77599476
rs62570196
rs17114848
rs11770843
rs2825714
rs1002941
rs72640280
rs2031282
All
-0.4
-0.3
-0.2
-0.1
0.0
MR leave-one-out sensitivity analysis for
'|| id:ebi-a-GCST90017109' on 'outcome'

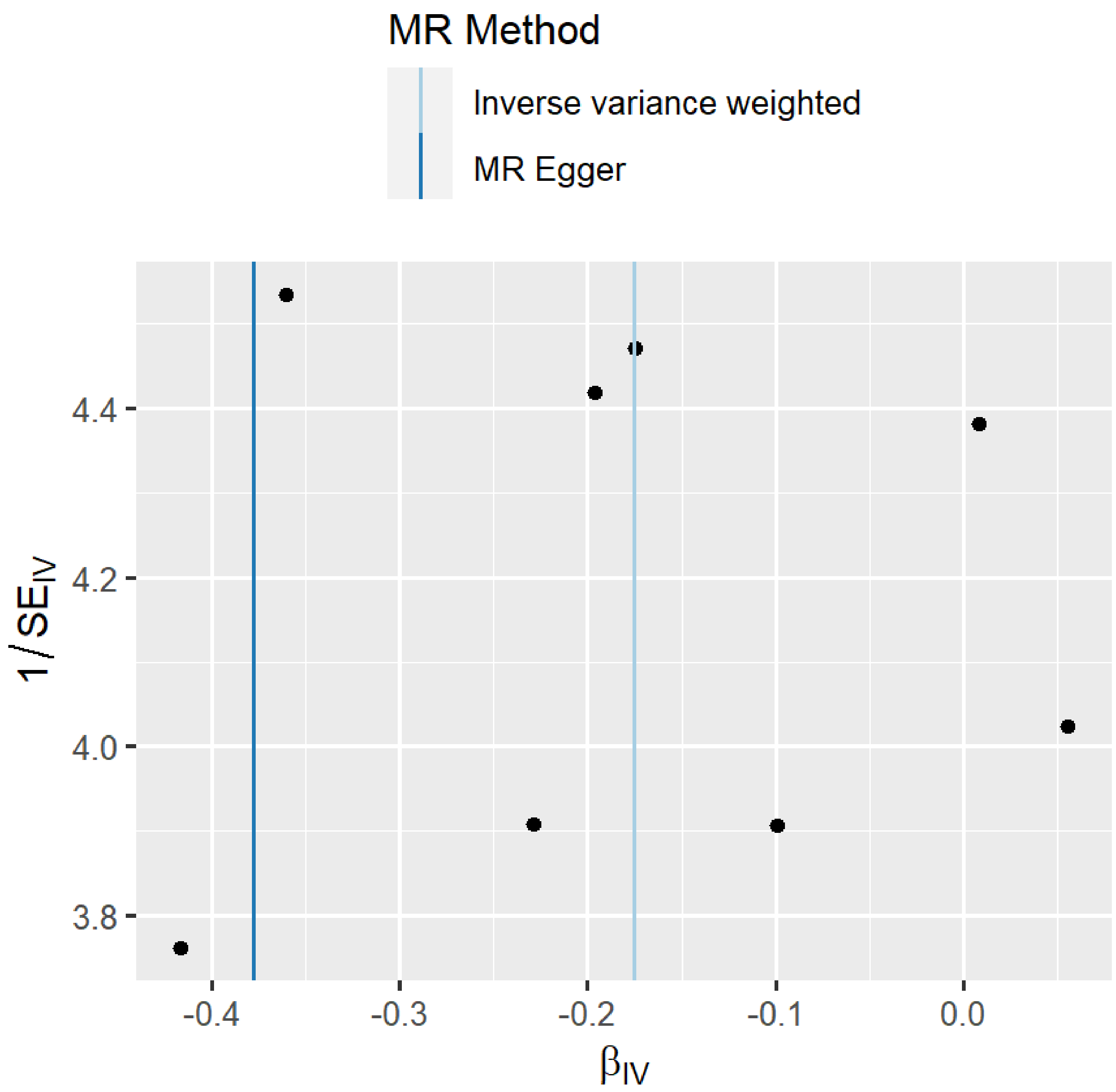
MR Method
Inverse variance weighted
MR Egger
4.4
4.2
4.0
3.8
1/SE_IV
-0.4
-0.3
-0.2
-0.1
0.0
β_IV

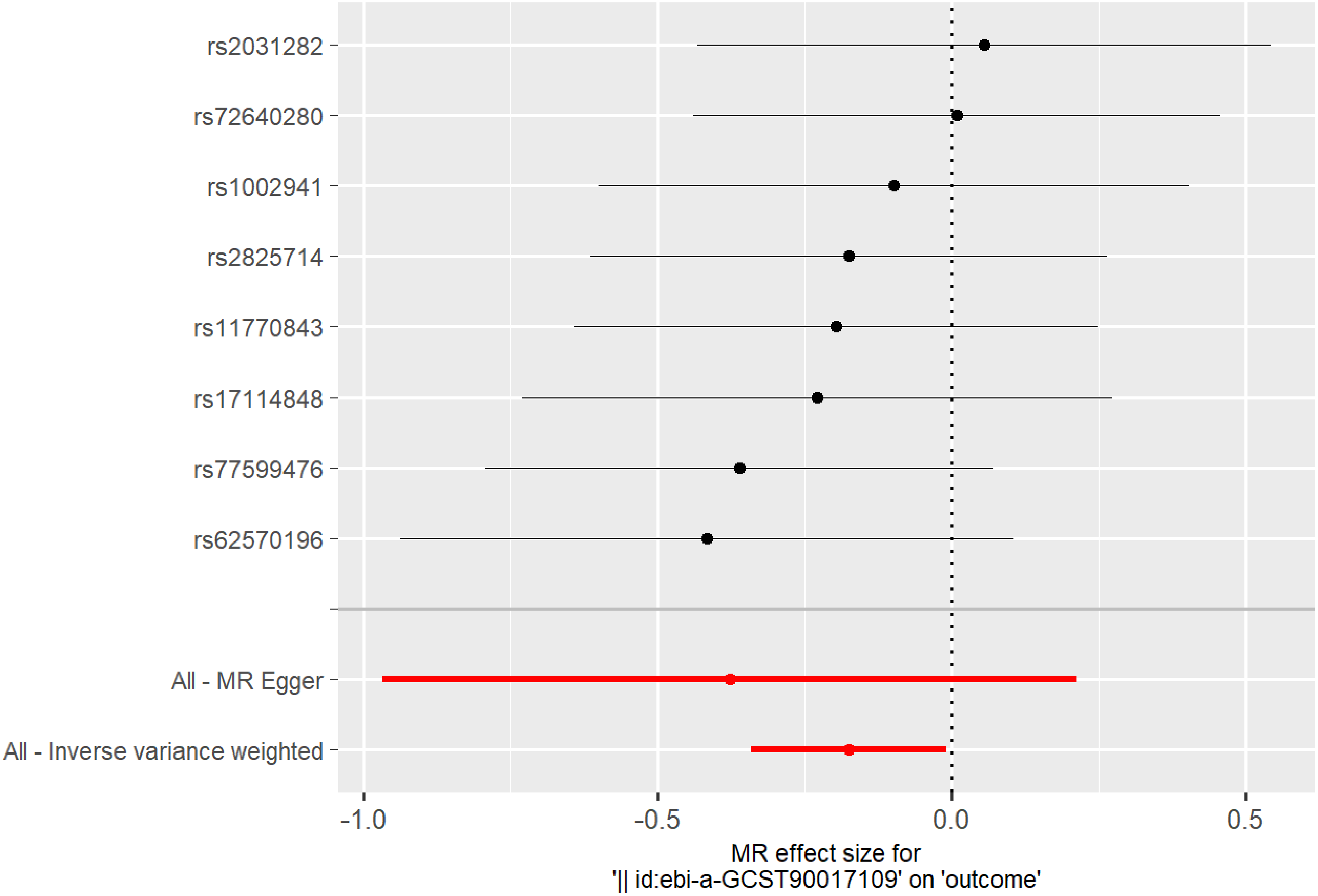
rs2031282
rs72640280
rs1002941
rs2825714
rs11770843
rs17114848
rs77599476
rs62570196
All - MR Egger
All - Inverse variance weighted
-1.0
-0.5
0.0
0.5
MR effect size for
'|| id:ebi-a-GCST90017109' on 'outcome'

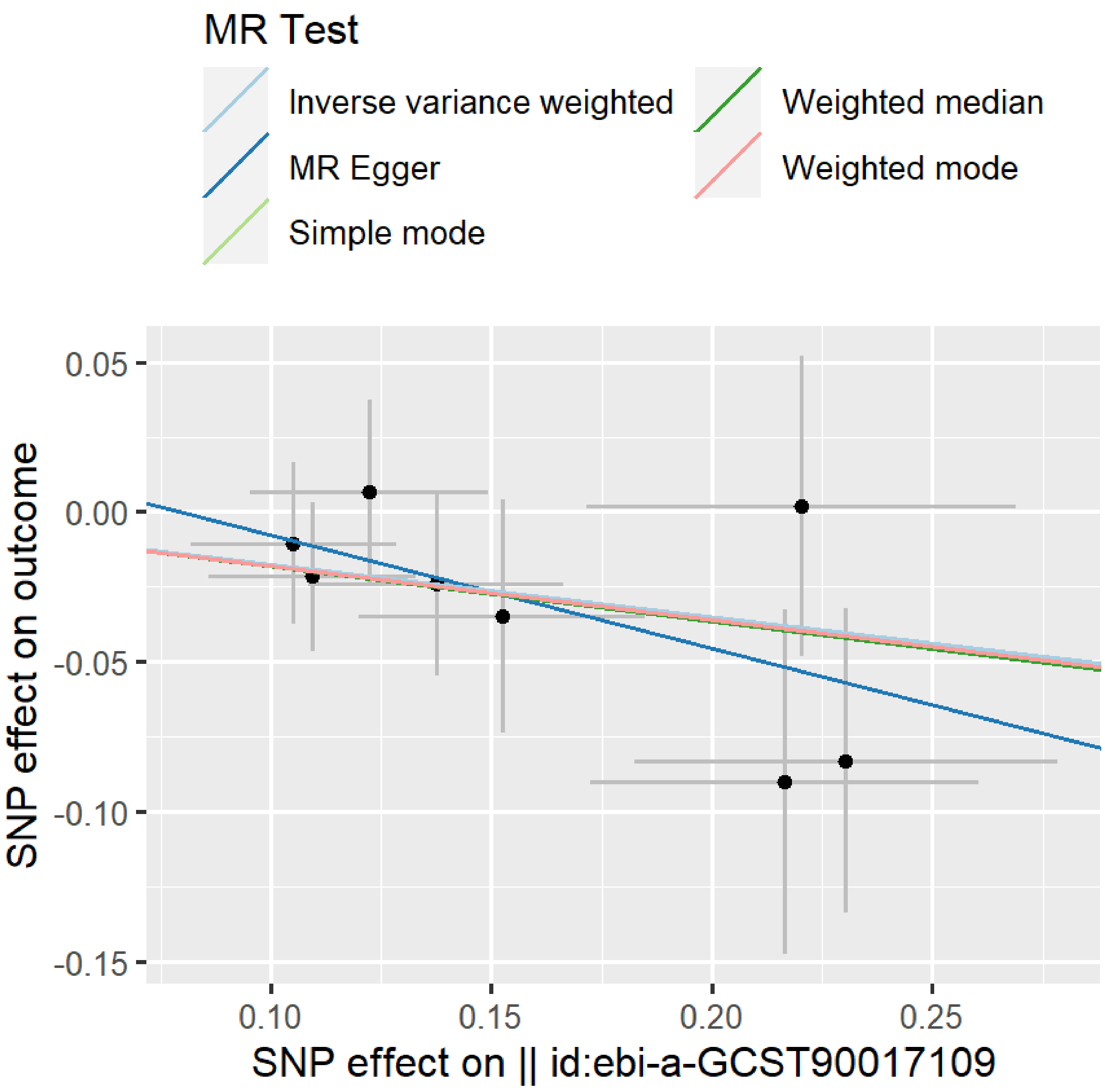
MR Test
Inverse variance weighted
MR Egger
Simple mode
Weighted median
Weighted mode
0.05
0.00
-0.05
-0.10
-0.15
SNP effect on outcome
0.10
0.15
0.20
0.25
SNP effect on || id:ebi-a-GCST90017109

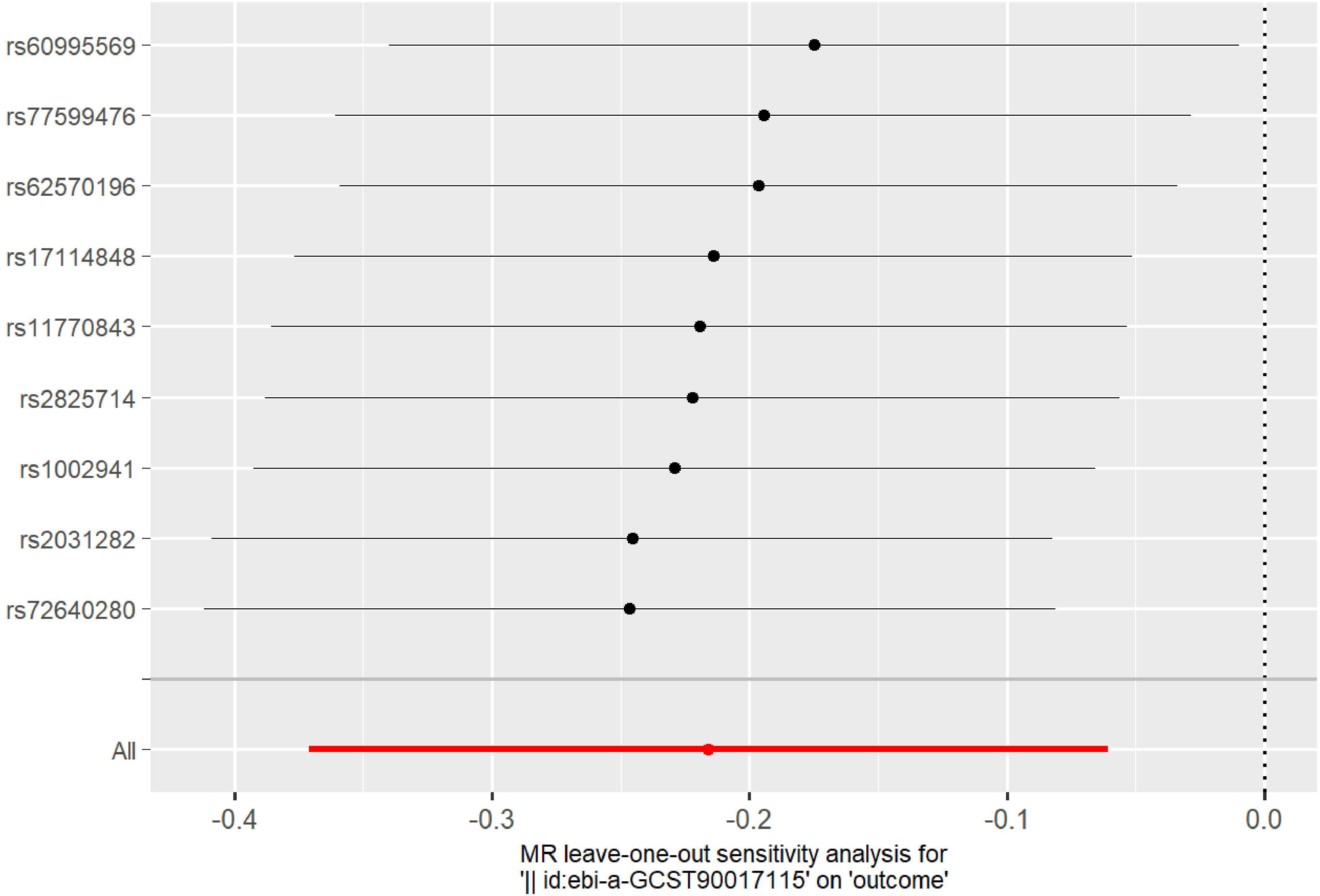
rs60995569
rs77599476
rs62570196
rs17114848
rs11770843
rs2825714
rs1002941
rs2031282
rs72640280
All
-0.4
-0.3
-0.2
-0.1
0.0
MR leave-one-out sensitivity analysis for
'|| id:ebi-a-GCST90017115' on 'outcome'

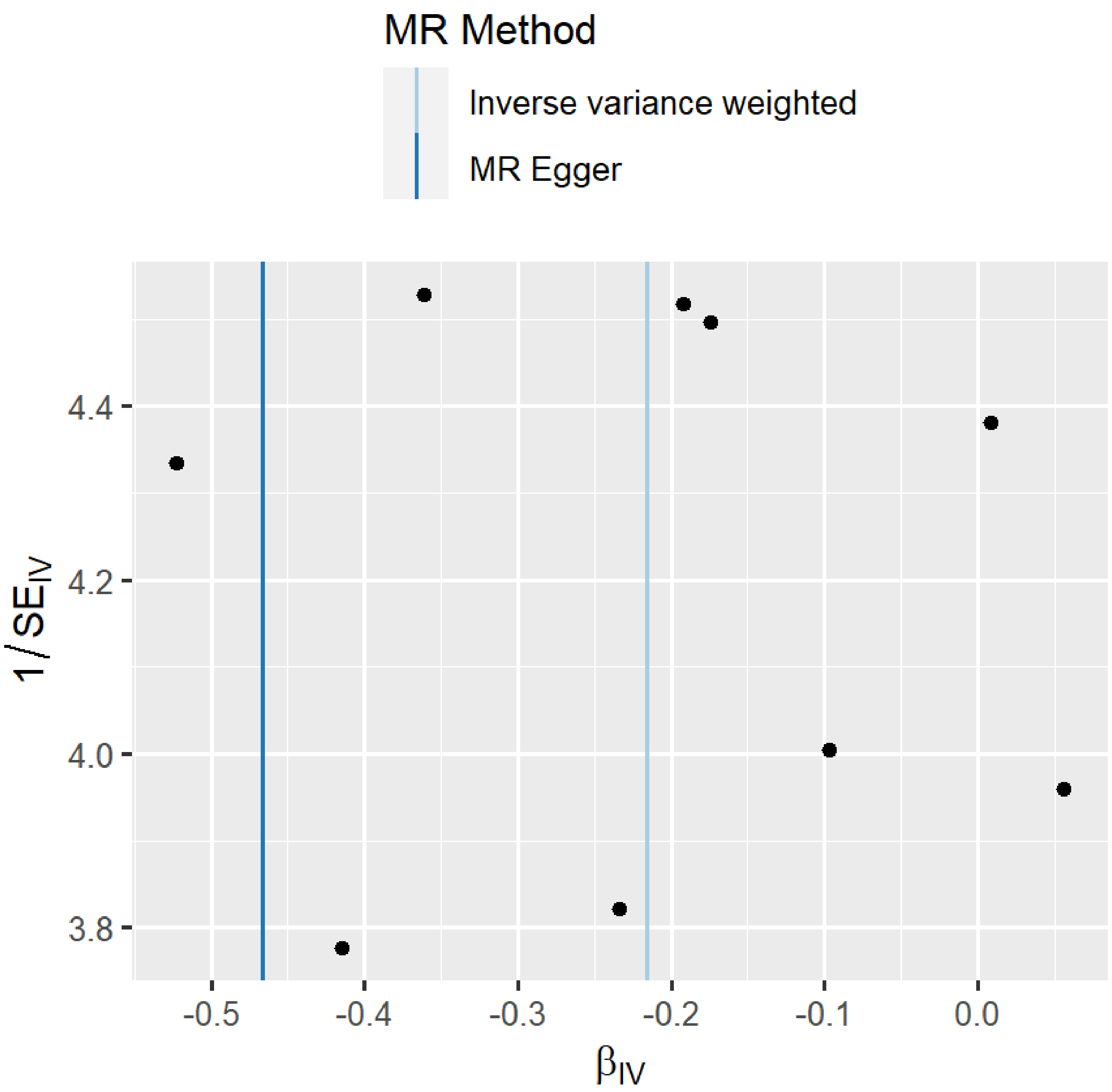
MR Method
Inverse variance weighted
MR Egger
4.4
4.2
4.0
3.8
1/SE_IV
-0.5
-0.4
-0.3
-0.2
-0.1
0.0
β_IV

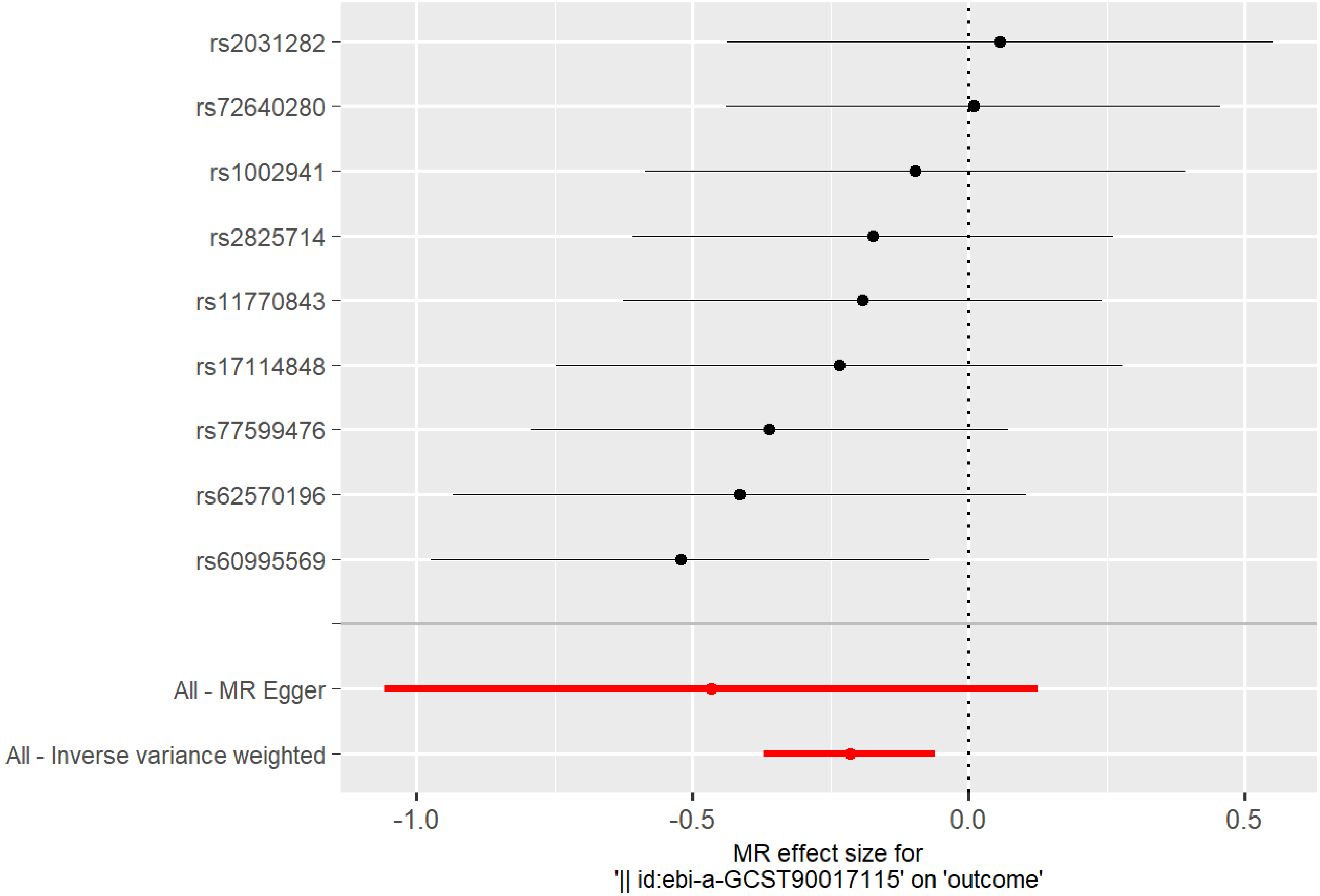

rs2031282
rs72640280
rs1002941
rs2825714
rs11770843
rs17114848
rs77599476
rs62570196
rs60995569
All - MR Egger
All - Inverse variance weighted
-1.0
-0.5
0.0
0.5
MR effect size for
'|| id:ebi-a-GCST90017115' on 'outcome'

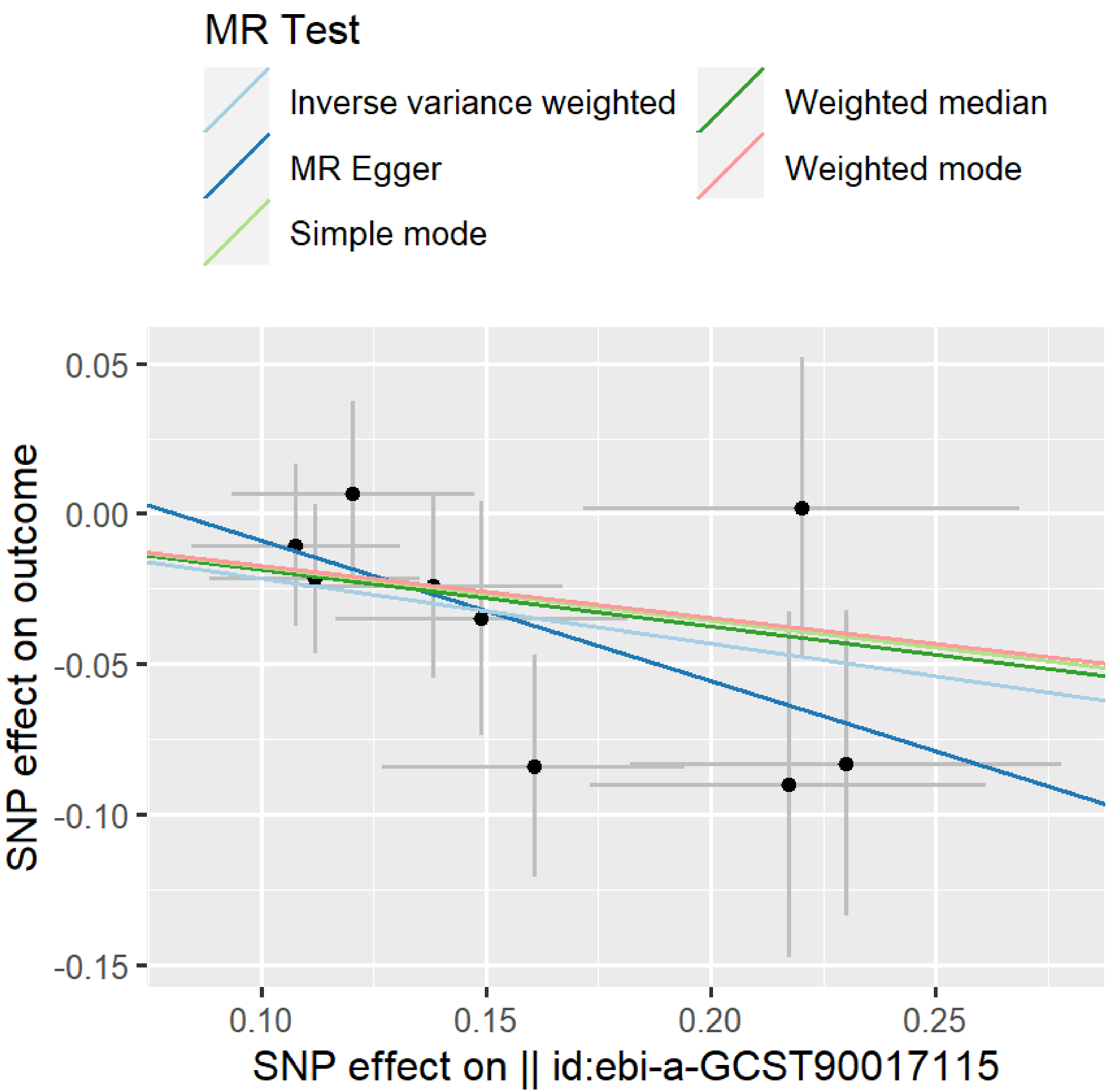
MR Test
Inverse variance weighted
MR Egger
Simple mode
Weighted median
Weighted mode
0.05
0.00
-0.05
-0.10
-0.15
0.10
0.15
0.20
0.25
SNP effect on outcome
SNP effect on || id:ebi-a-GCST90017115

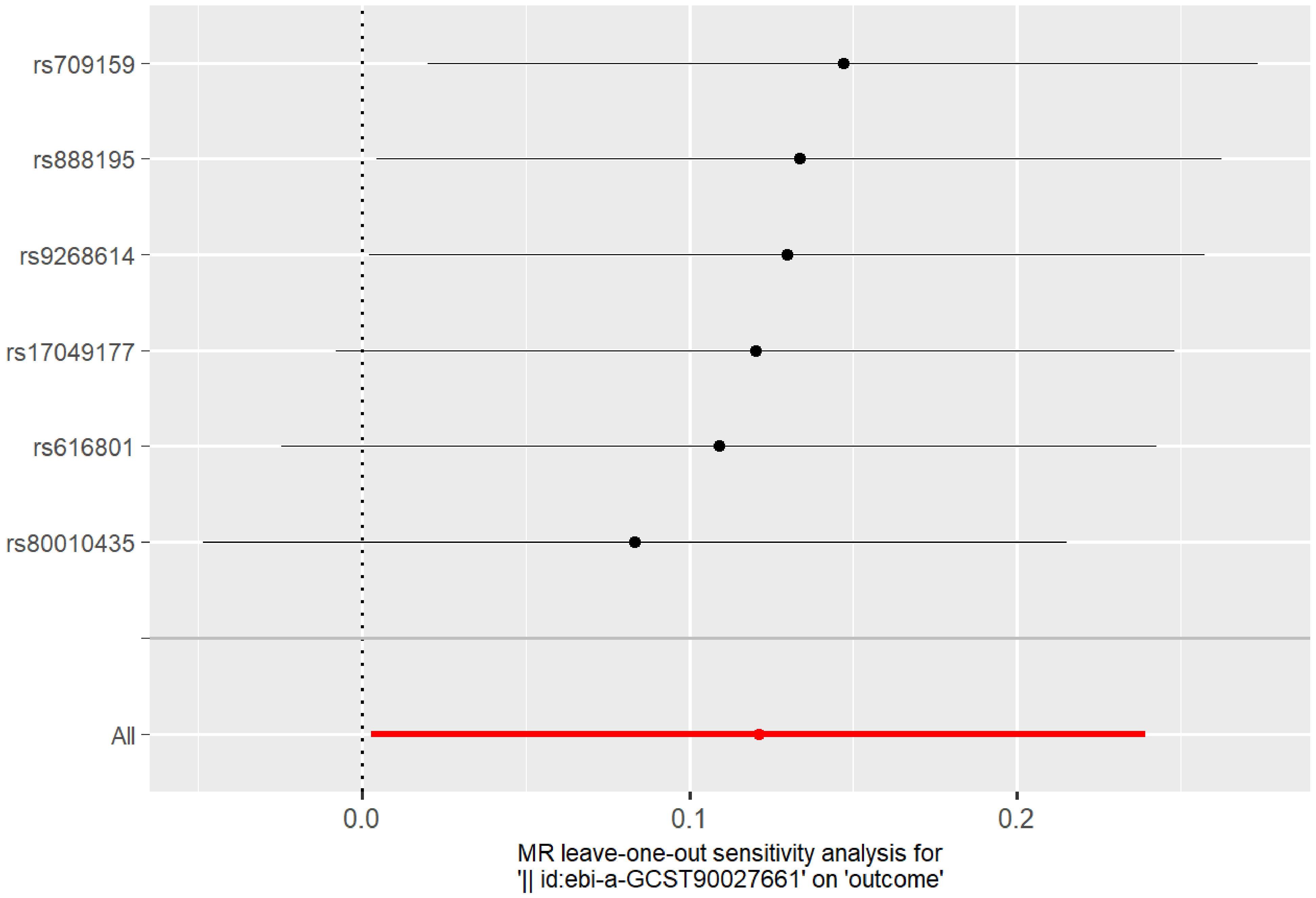
rs709159
rs888195
rs9268614
rs17049177
rs616801
rs80010435
All
0.0
0.1
0.2
MR leave-one-out sensitivity analysis for
'|| id:ebi-a-GCST90027661' on 'outcome'

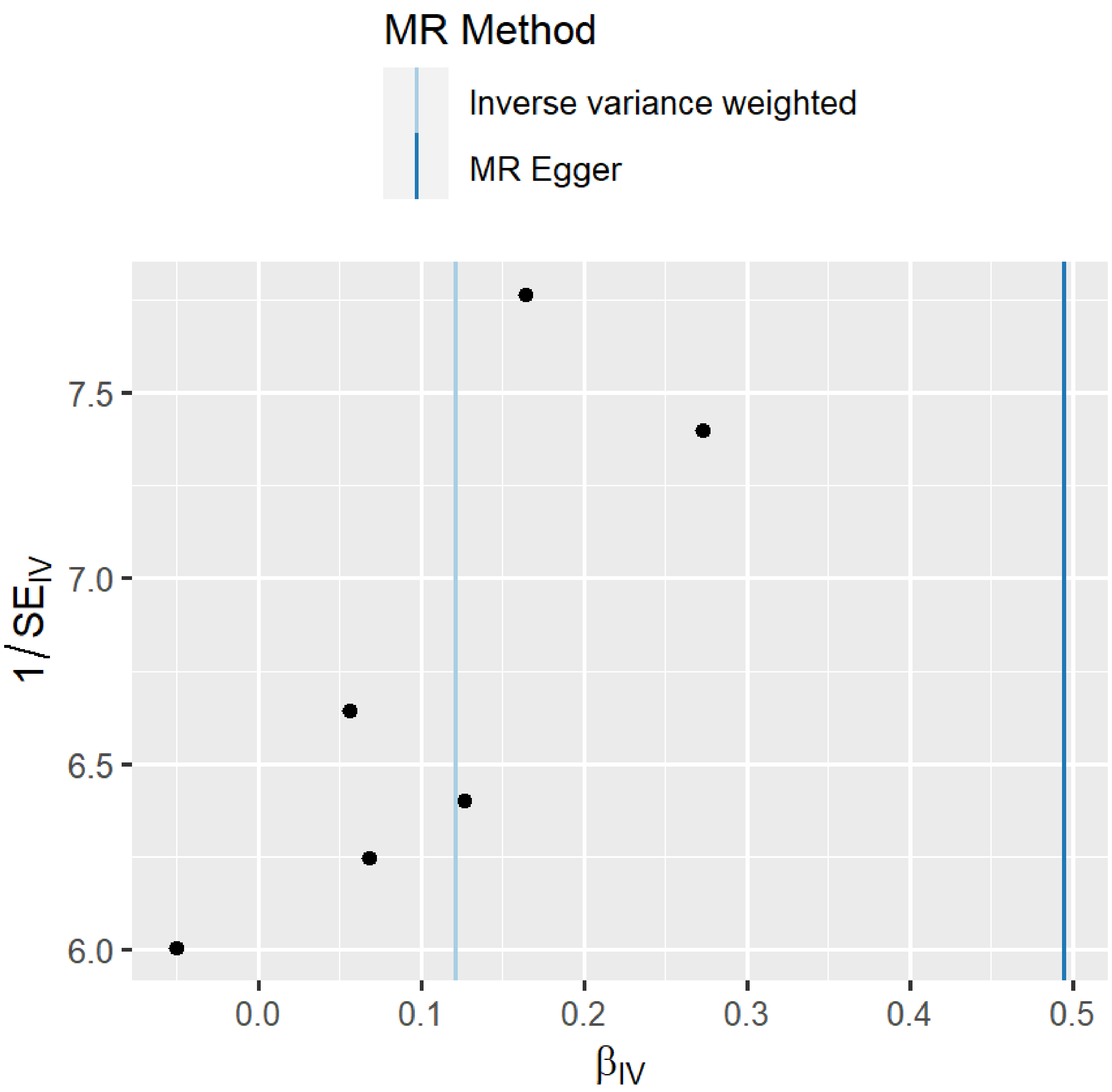
MR Method
Inverse variance weighted
MR Egger
7.5
7.0
6.5
6.0
1/SE_IV
0.0
0.1
0.2
0.3
0.4
0.5
β_IV

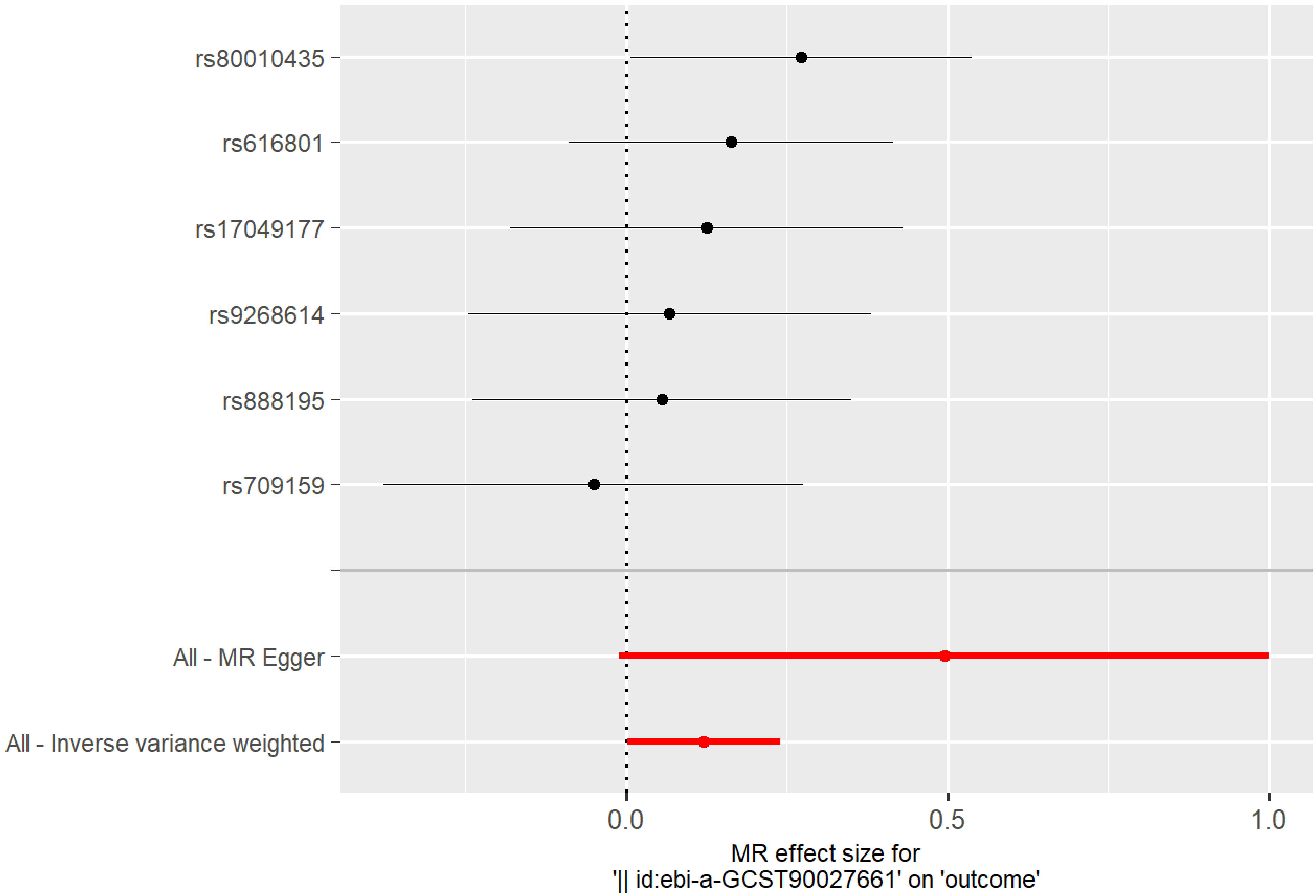

rs80010435
rs616801
rs17049177
rs9268614
rs888195
rs709159
All - MR Egger
All - Inverse variance weighted
0.0
0.5
1.0
MR effect size for
'|| id:ebi-a-GCST90027661' on 'outcome'

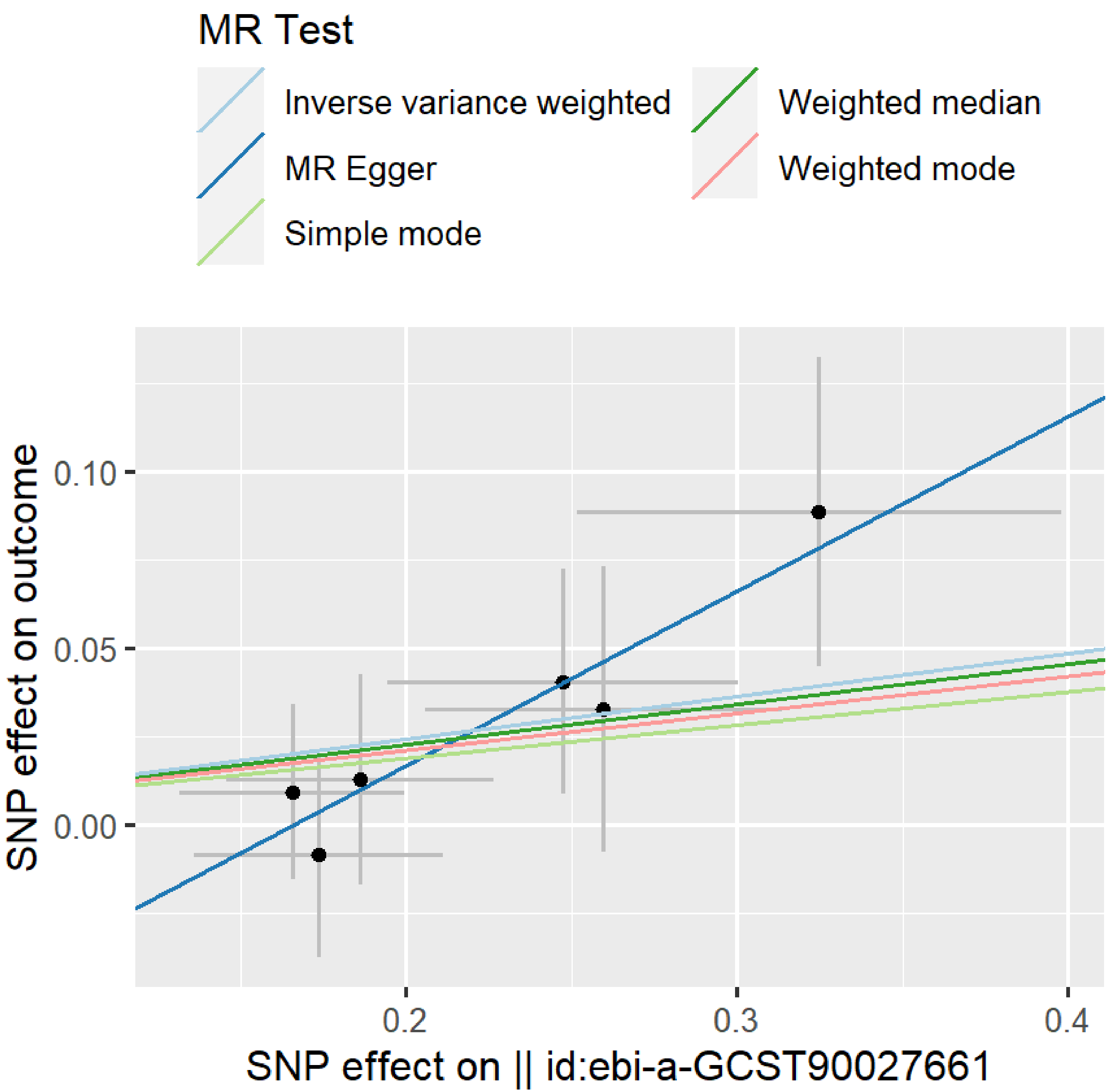

MR Test
Inverse variance weighted
MR Egger
Simple mode
Weighted median
Weighted mode
0.10
0.05
0.00
SNP effect on outcome
0.2
0.3
0.4
SNP effect on || id:ebi-a-GCST90027661

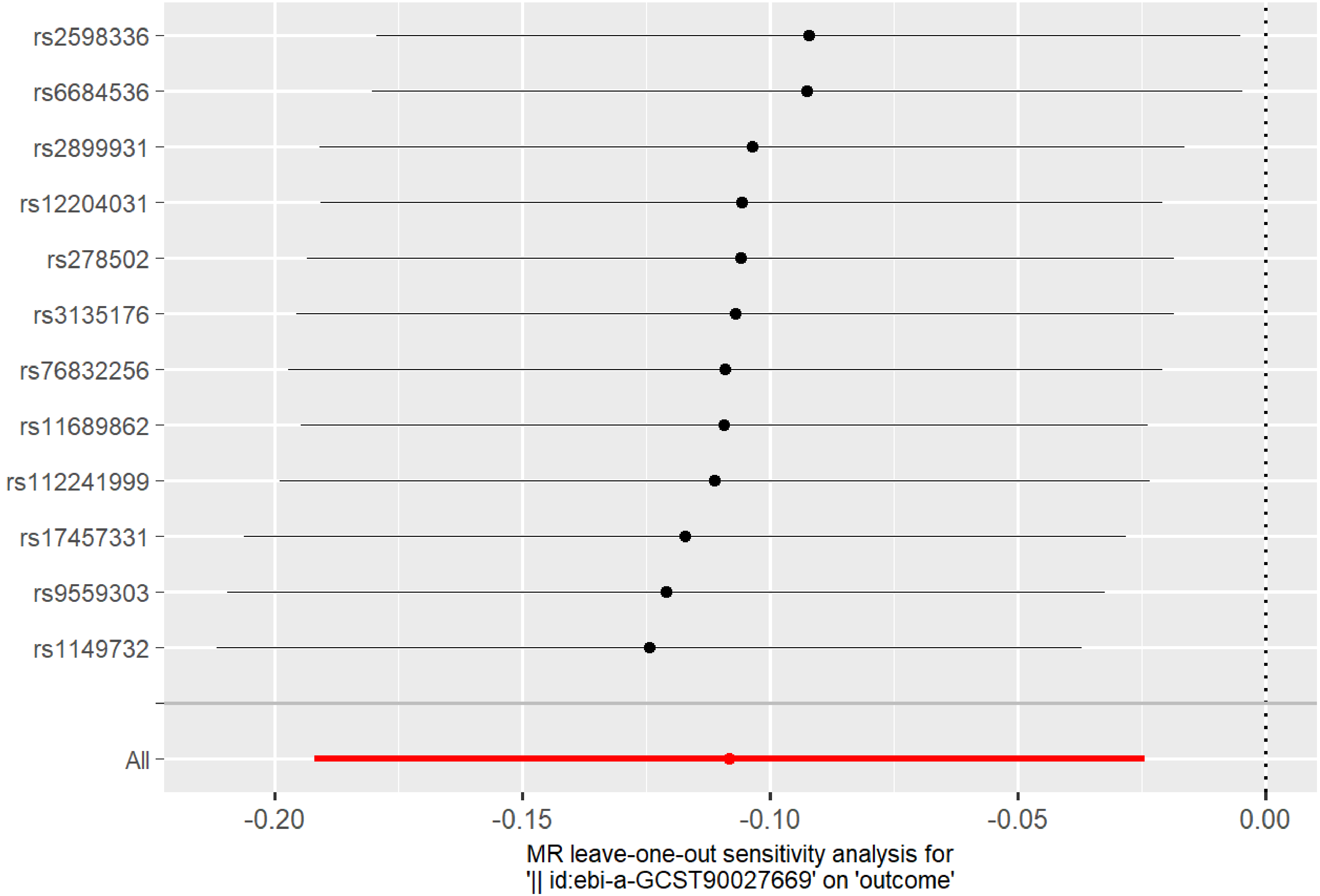
rs2598336
rs6684536
rs2899931
rs12204031
rs278502
rs3135176
rs76832256
rs11689862
rs112241999
rs17457331
rs9559303
rs1149732
All
-0.20
-0.15
-0.10
-0.05
0.00
MR leave-one-out sensitivity analysis for
'|| id:ebi-a-GCST90027669' on 'outcome'

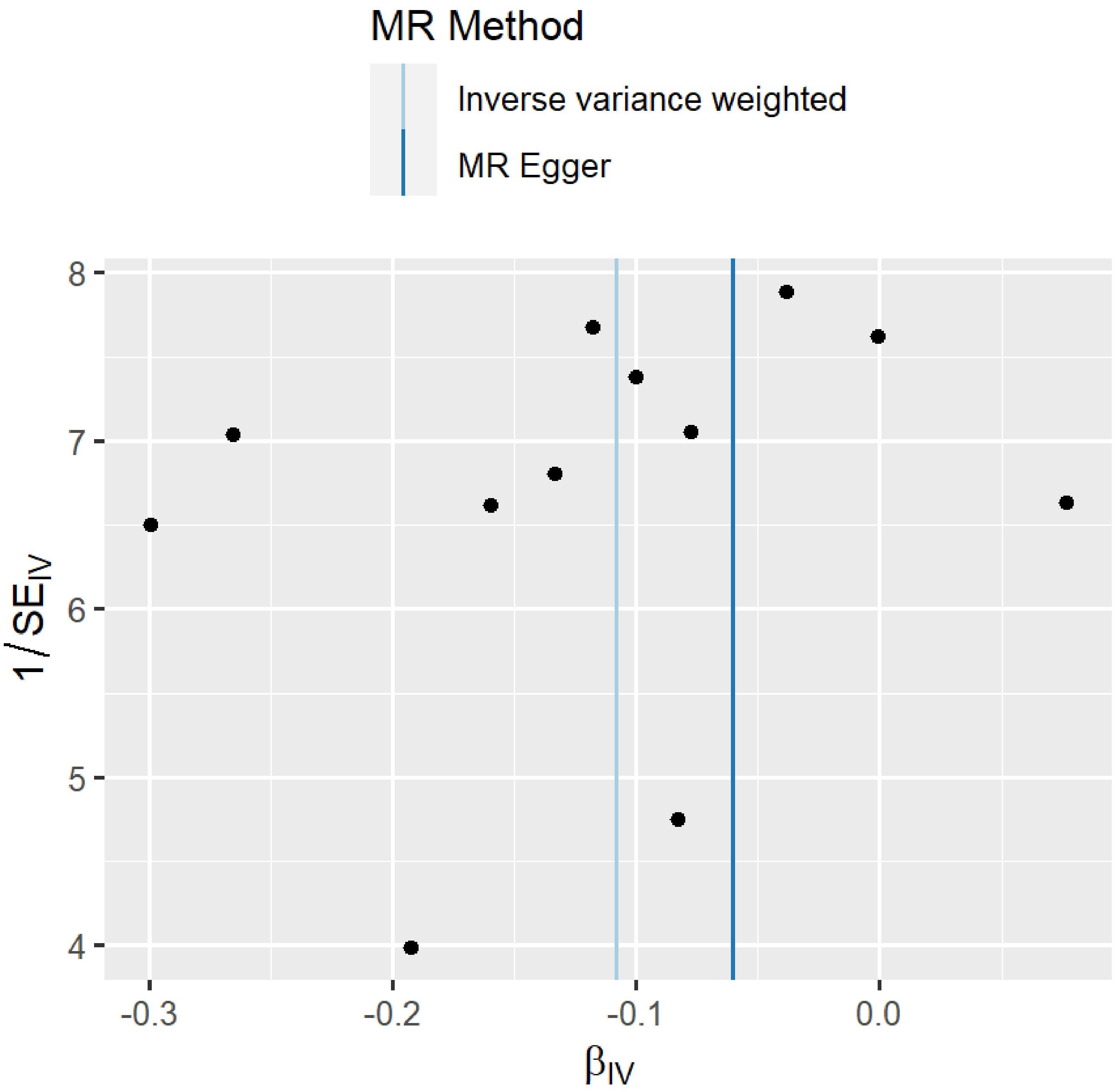
MR Method
Inverse variance weighted
MR Egger
8
7
6
5
4
$1/SE_{IV}$
-0.3
-0.2
-0.1
0.0
β_{IV}

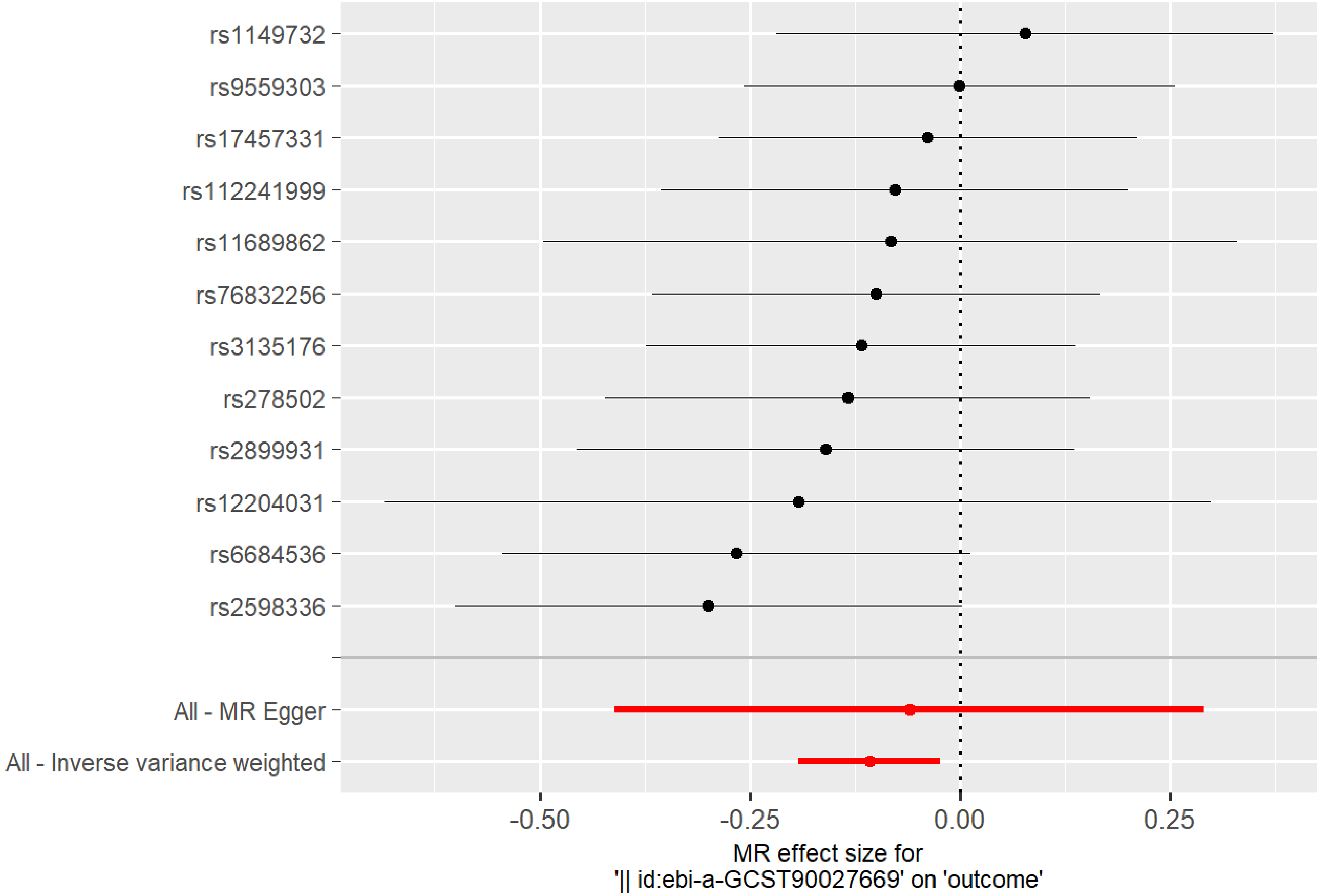
rs1149732
rs9559303
rs17457331
rs112241999
rs11689862
rs76832256
rs3135176
rs278502
rs2899931
rs12204031
rs6684536
rs2598336
All - MR Egger
All - Inverse variance weighted
-0.50
-0.25
0.00
0.25
MR effect size for
'|| id:ebi-a-GCST90027669' on 'outcome'

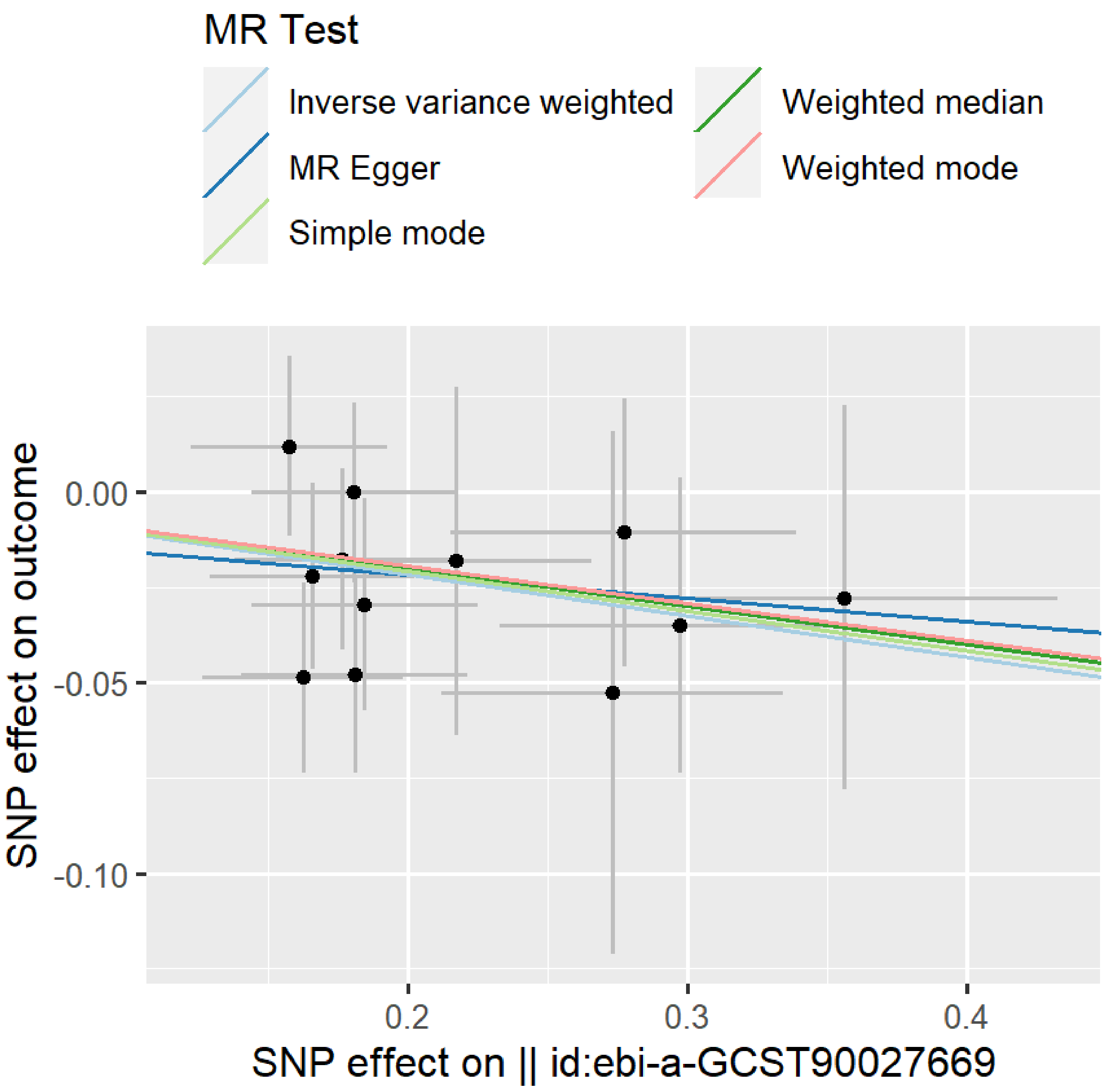
MR Test
Inverse variance weighted
MR Egger
Simple mode
Weighted median
Weighted mode
SNP effect on outcome
0.00
-0.05
-0.10
0.2
0.3
0.4
SNP effect on || id:ebi-a-GCST90027669

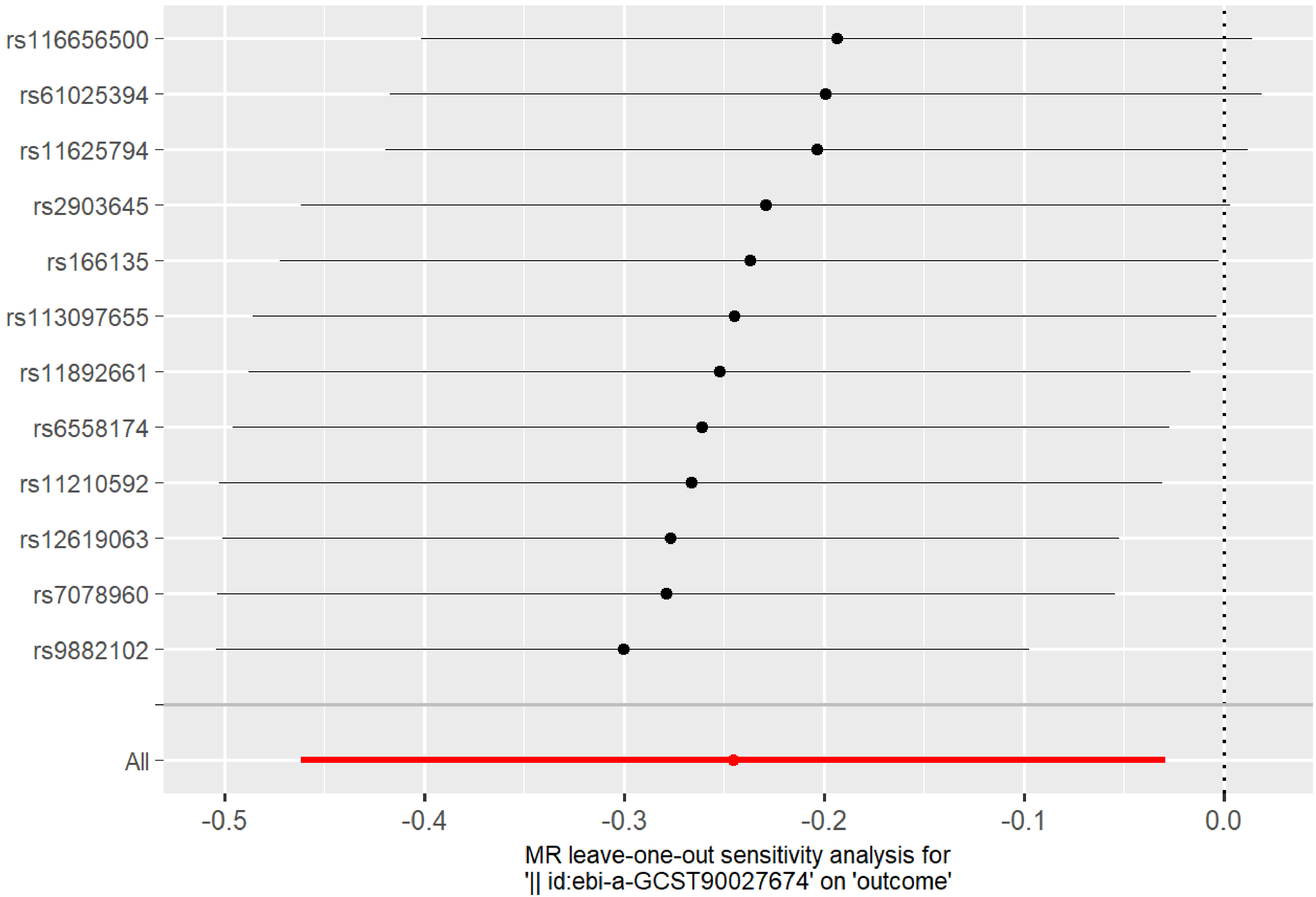
rs116656500
rs61025394
rs11625794
rs2903645
rs166135
rs113097655
rs11892661
rs6558174
rs11210592
rs12619063
rs7078960
rs9882102
All
-0.5
-0.4
-0.3
-0.2
-0.1
0.0
MR leave-one-out sensitivity analysis for
'|| id:ebi-a-GCST90027674' on 'outcome'

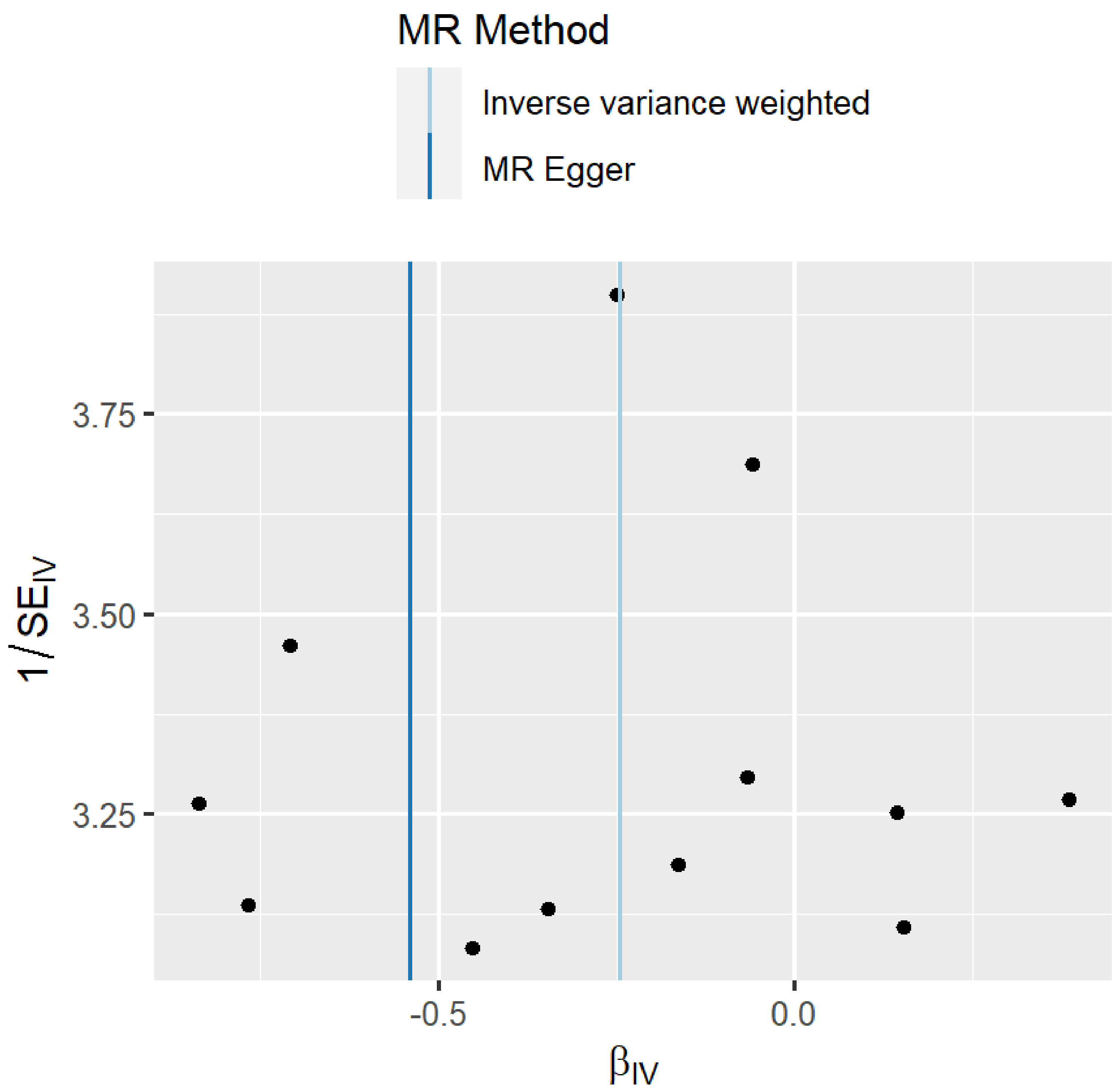
MR Method
Inverse variance weighted
MR Egger
3.75
3.50
3.25
-0.5
0.0
β_{IV}
$1/SE_{IV}$

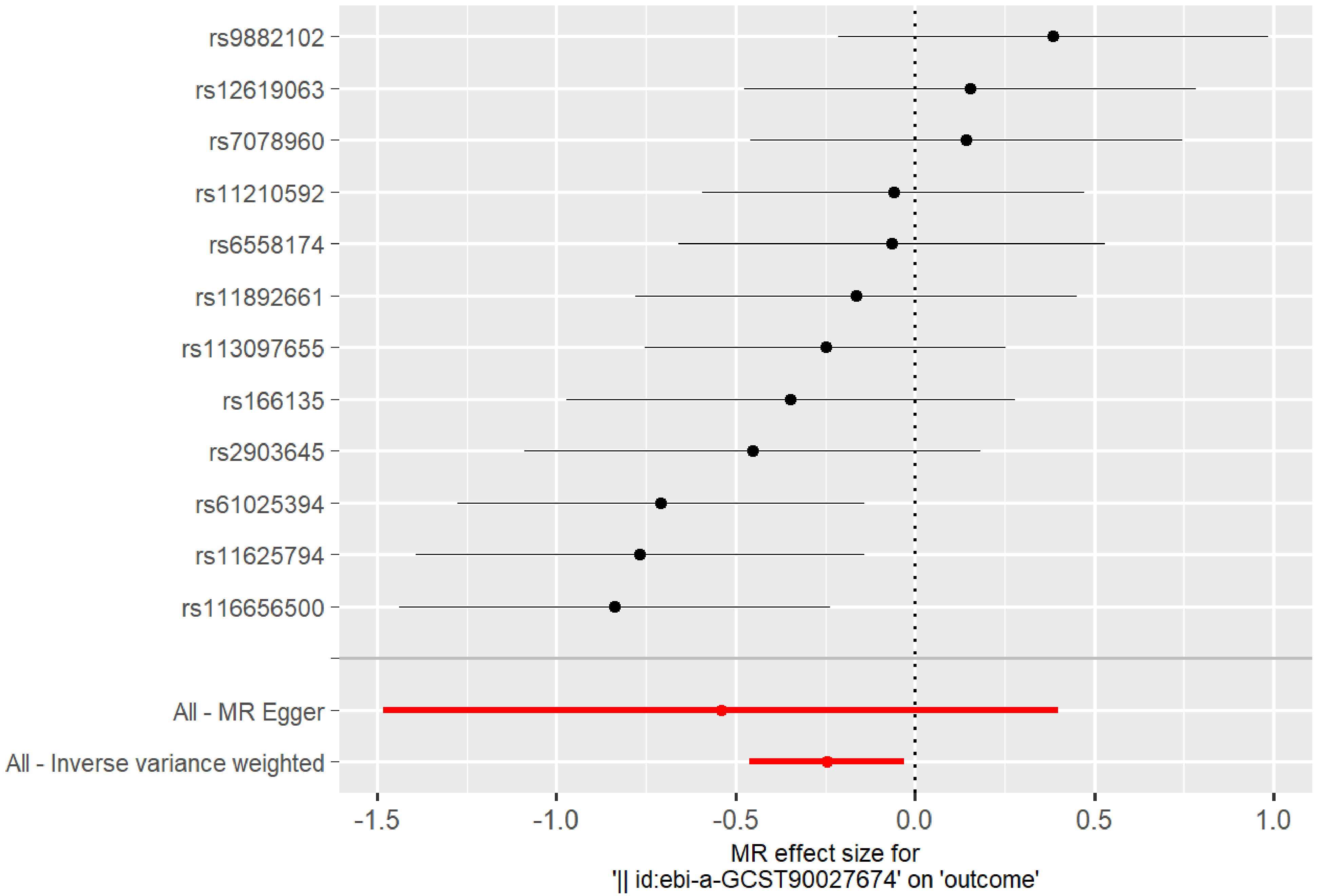
rs9882102
rs12619063
rs7078960
rs11210592
rs6558174
rs11892661
rs113097655
rs166135
rs2903645
rs61025394
rs11625794
rs116656500
All - MR Egger
All - Inverse variance weighted
-1.5
-1.0
-0.5
0.0
0.5
1.0
MR effect size for
'|| id:ebi-a-GCST90027674' on 'outcome'

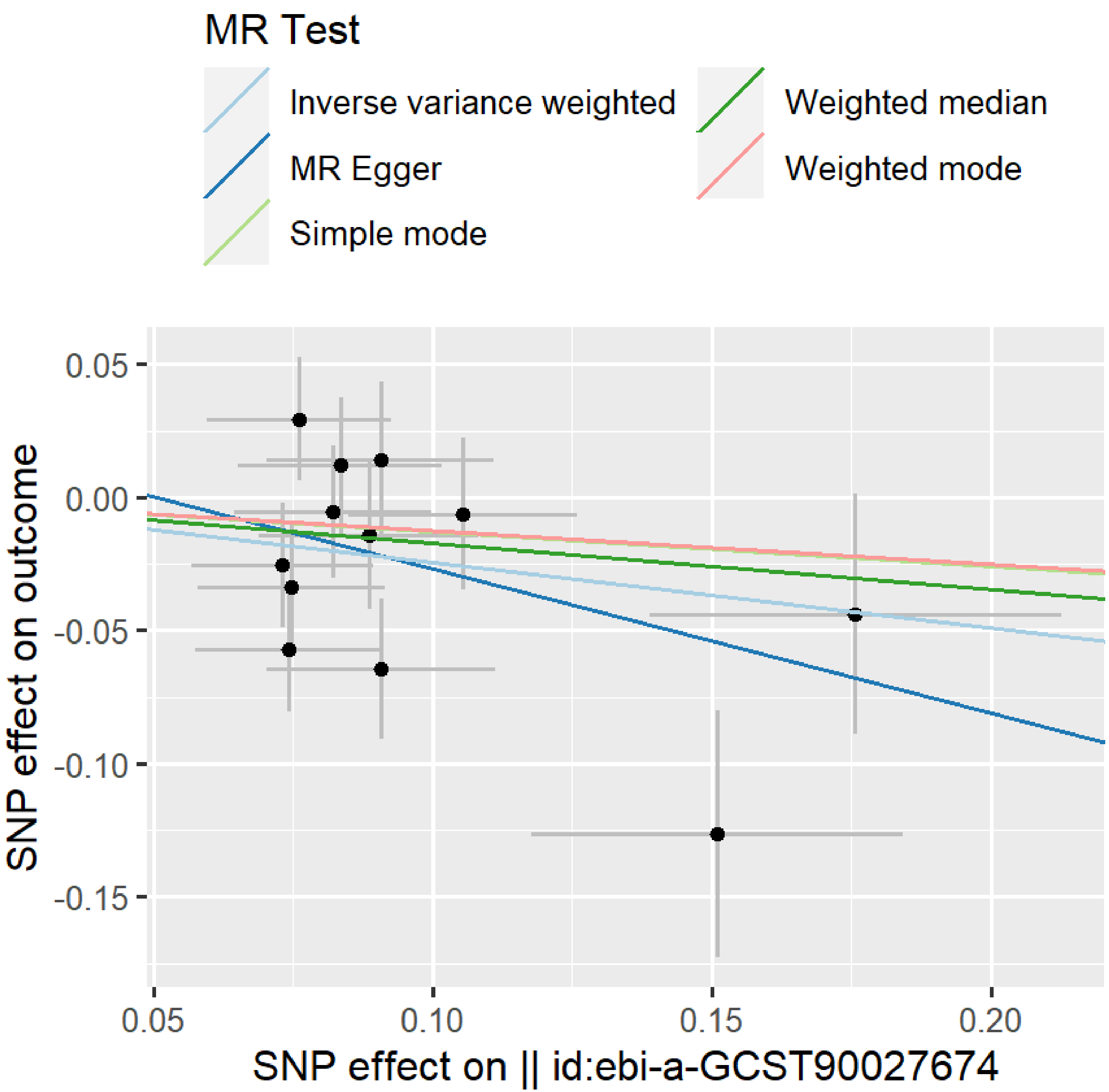
MR Test
Inverse variance weighted
MR Egger
Simple mode
Weighted median
Weighted mode
0.05
0.00
-0.05
-0.10
-0.15
SNP effect on outcome
0.05
0.10
0.15
0.20
SNP effect on || id:ebi-a-GCST90027674

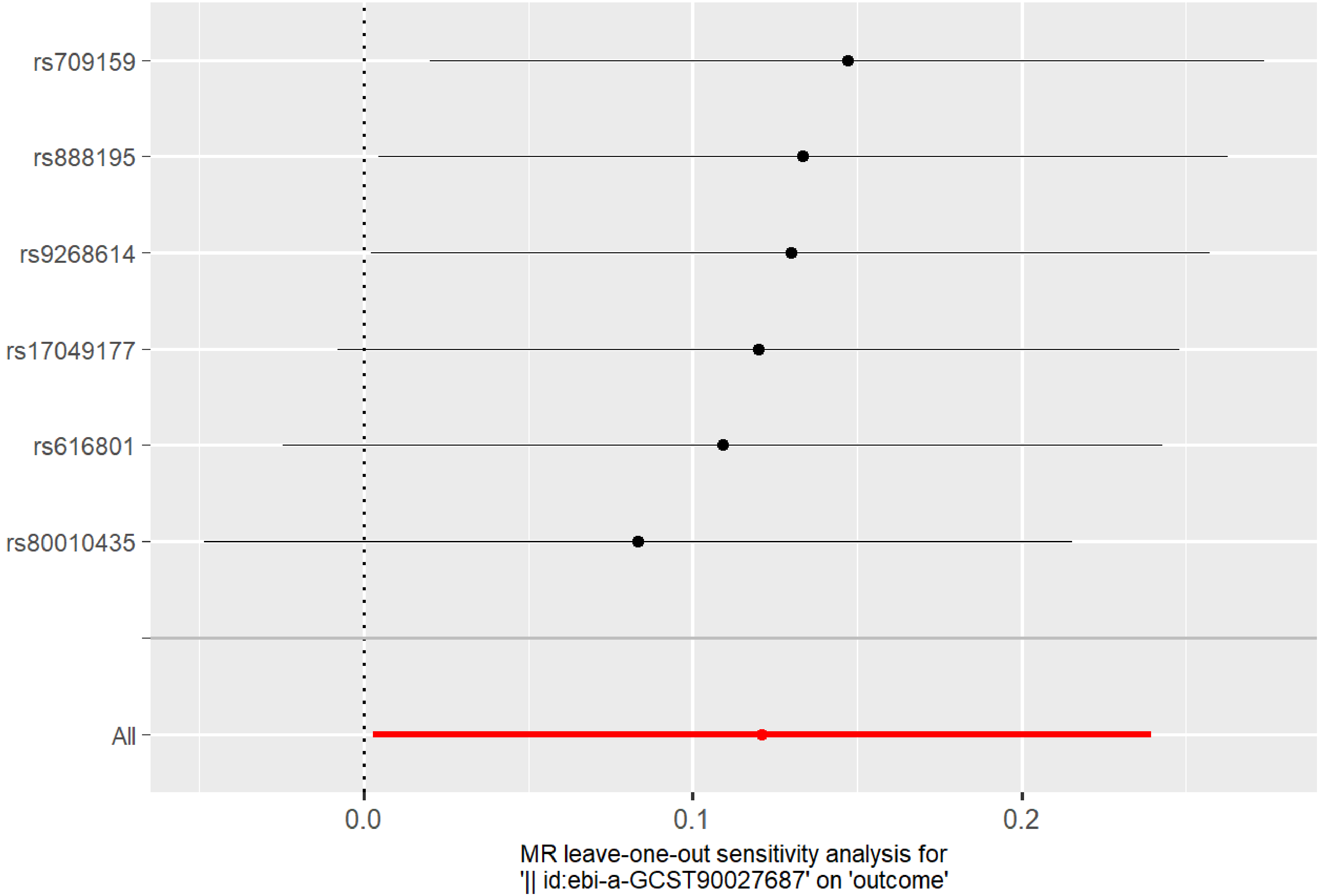
rs709159
rs888195
rs9268614
rs17049177
rs616801
rs80010435
All
0.0
0.1
0.2
MR leave-one-out sensitivity analysis for
'|| id:ebi-a-GCST90027687' on 'outcome'

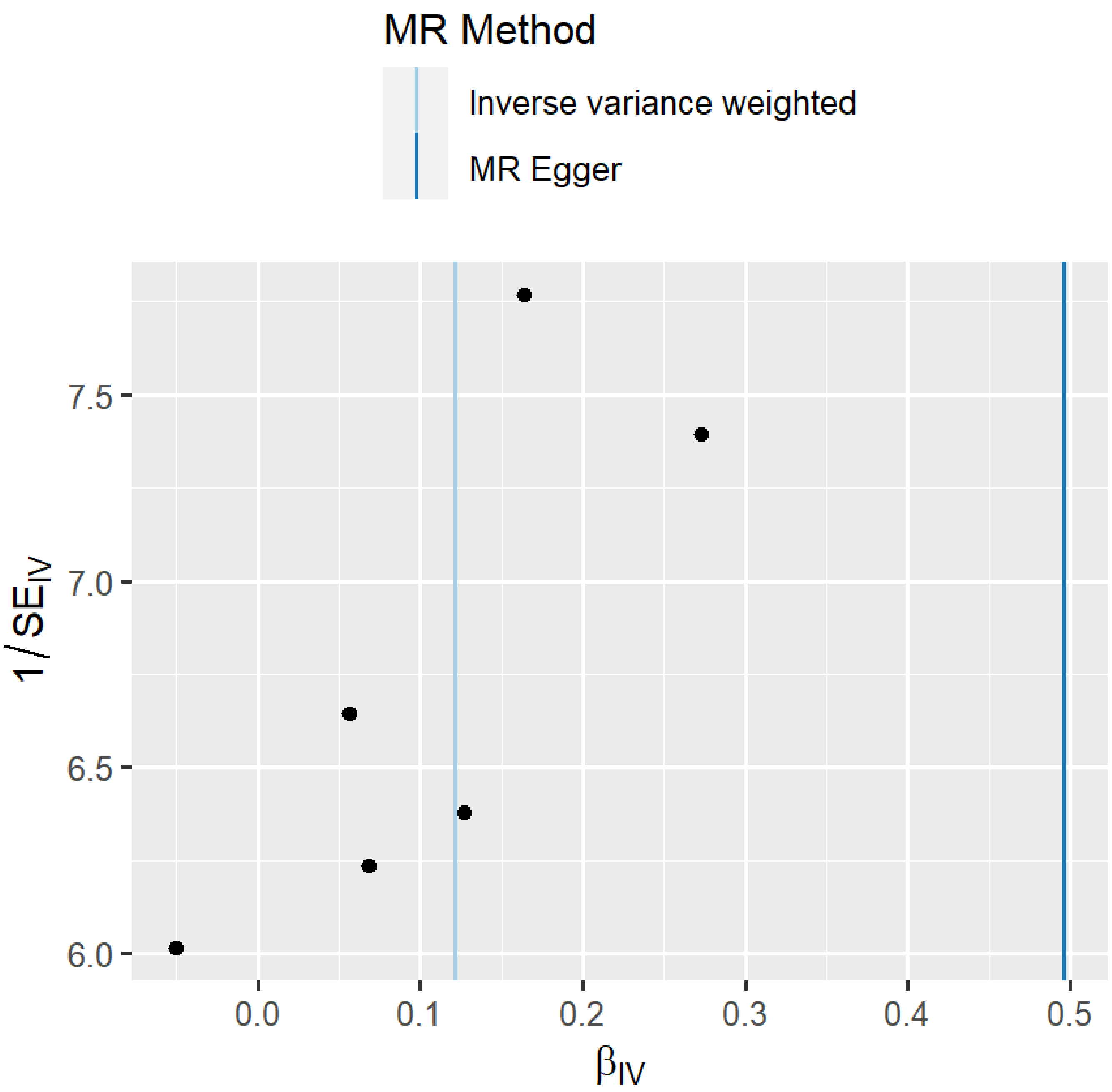

MR Method
Inverse variance weighted
MR Egger
7.5
7.0
6.5
6.0
1/SE_IV
0.0
0.1
0.2
0.3
0.4
0.5
β_IV

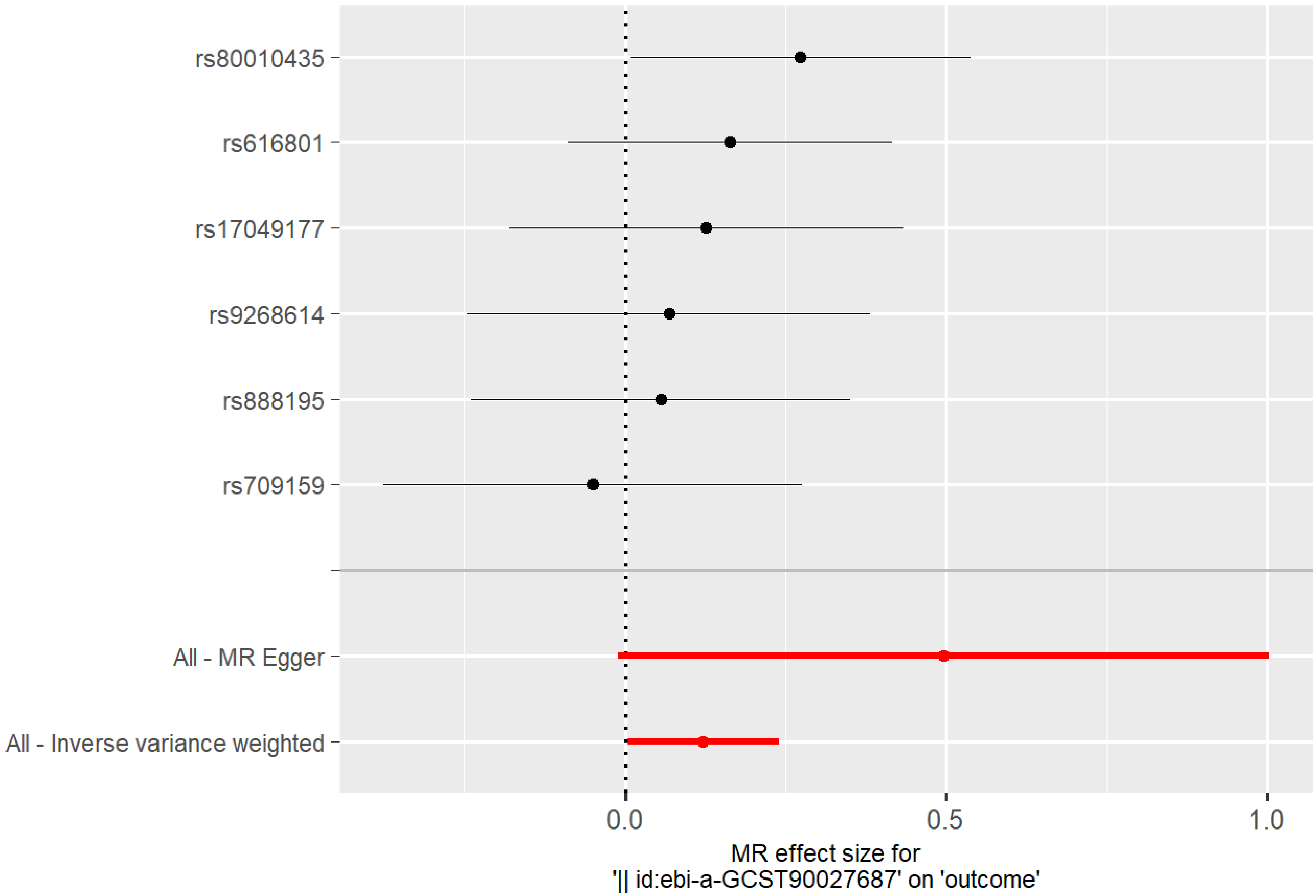
rs80010435
rs616801
rs17049177
rs9268614
rs888195
rs709159
All - MR Egger
All - Inverse variance weighted
0.0
0.5
1.0
MR effect size for
'|| id:ebi-a-GCST90027687' on 'outcome'

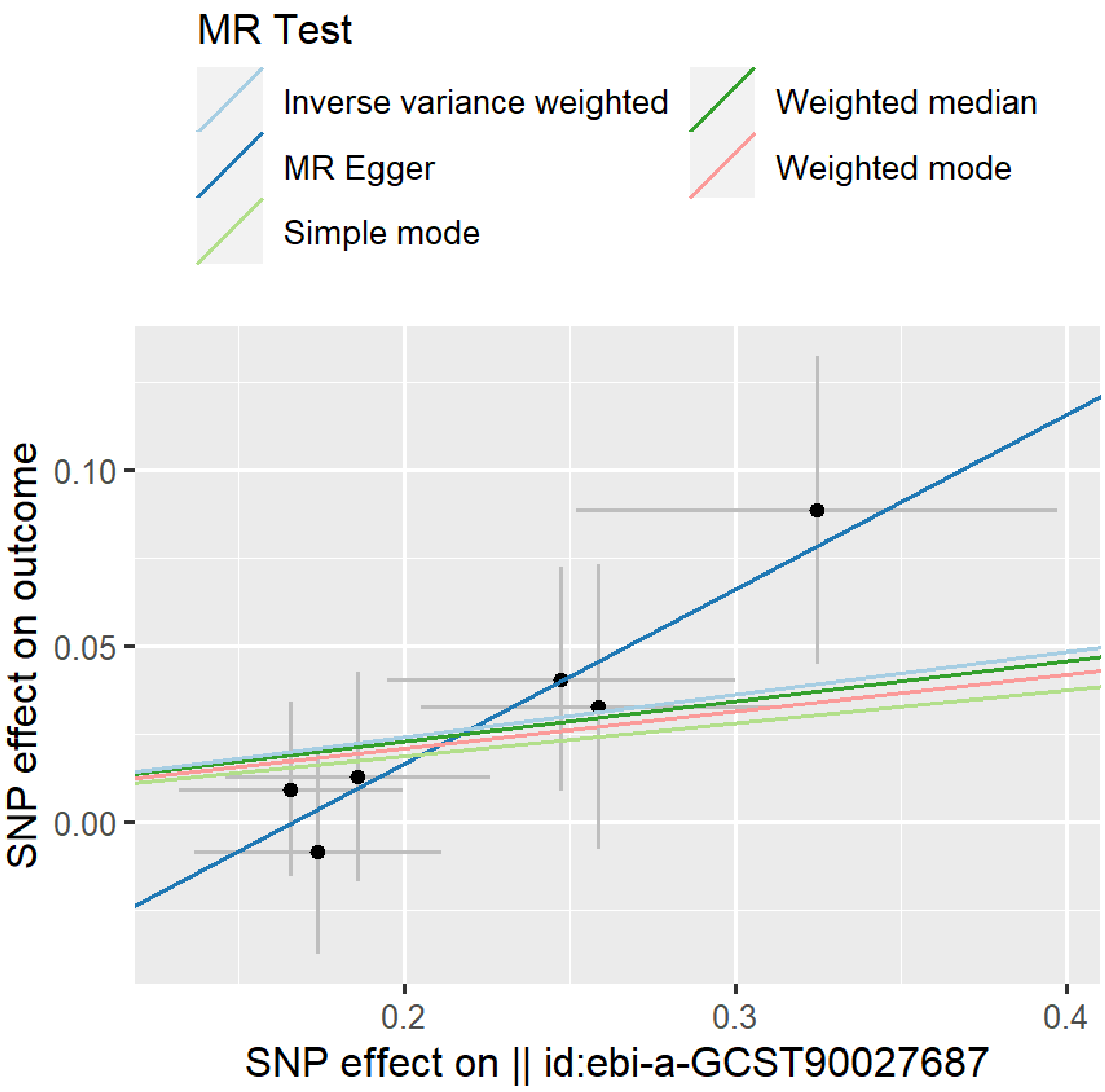
MR Test
Inverse variance weighted
MR Egger
Simple mode
Weighted median
Weighted mode
0.10
0.05
0.00
SNP effect on outcome
0.2
0.3
0.4
SNP effect on || id:ebi-a-GCST90027687

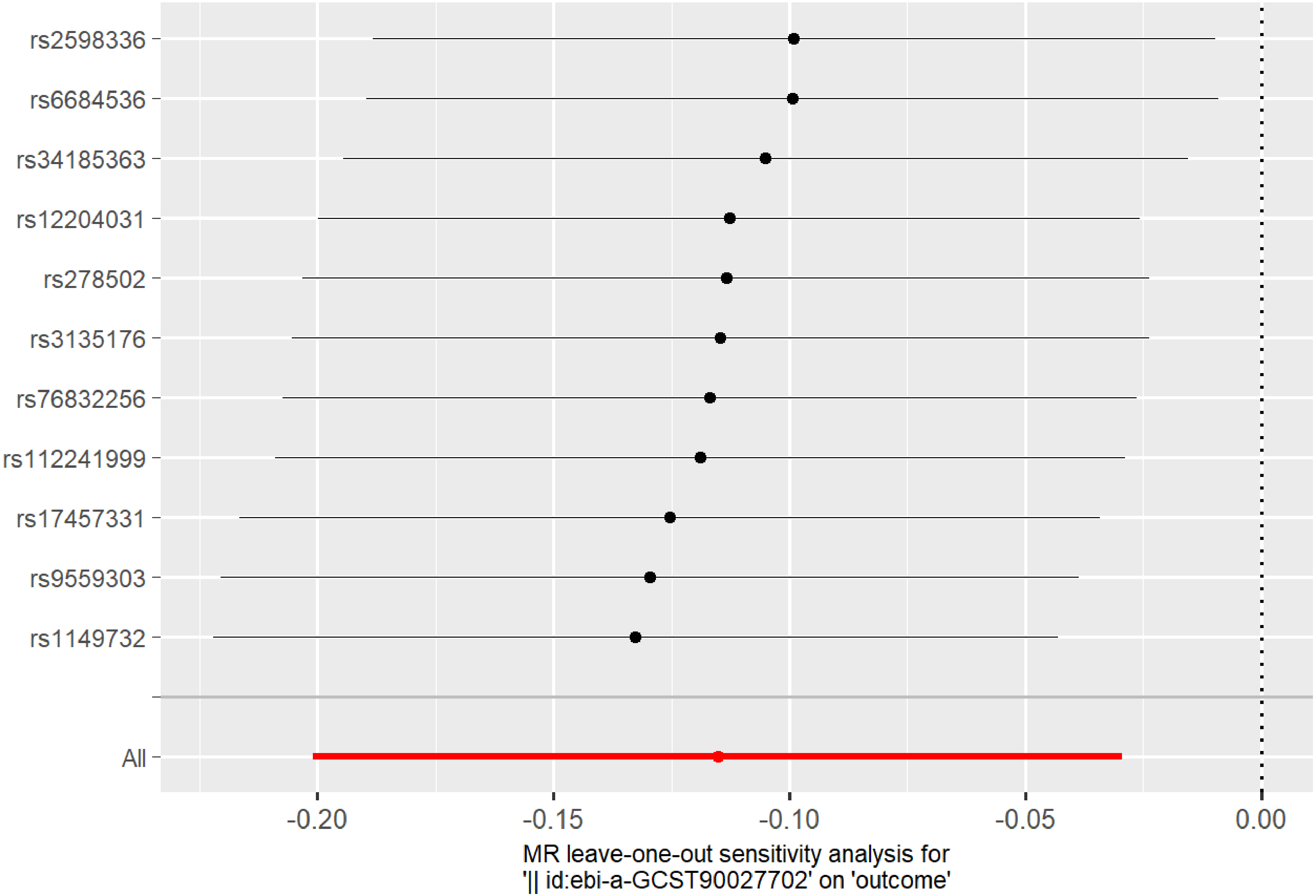

rs2598336
rs6684536
rs34185363
rs12204031
rs278502
rs3135176
rs76832256
rs112241999
rs17457331
rs9559303
rs1149732
All
-0.20
-0.15
-0.10
-0.05
0.00
MR leave-one-out sensitivity analysis for
'|| id:ebi-a-GCST90027702' on 'outcome'

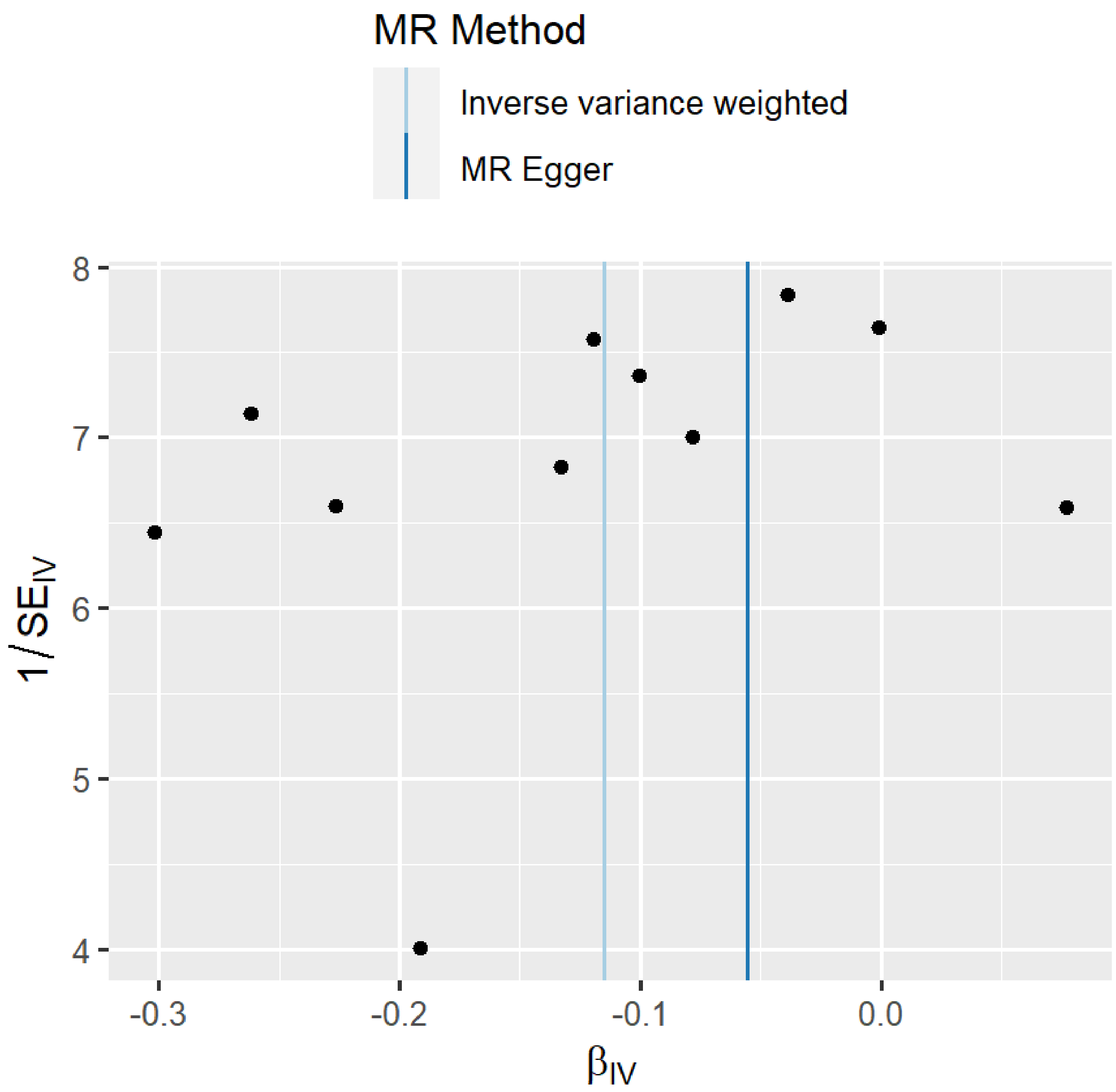
MR Method
Inverse variance weighted
MR Egger
8
7
6
5
4
-0.3
-0.2
-0.1
0.0
$1/SE_{IV}$
β_{IV}

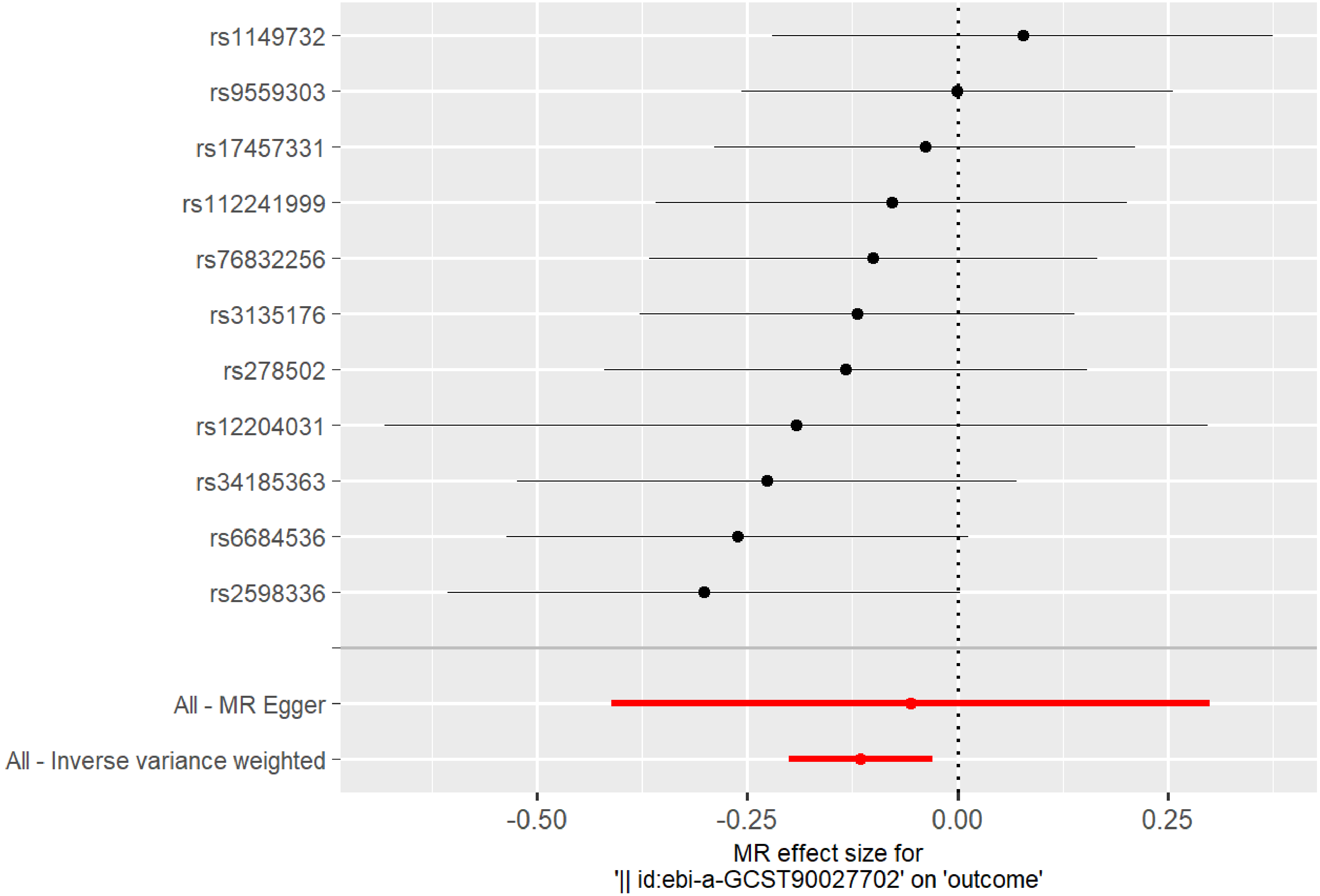
rs1149732
rs9559303
rs17457331
rs112241999
rs76832256
rs3135176
rs278502
rs12204031
rs34185363
rs6684536
rs2598336
All - MR Egger
All - Inverse variance weighted
-0.50
-0.25
0.00
0.25
MR effect size for
'|| id:ebi-a-GCST90027702' on 'outcome'

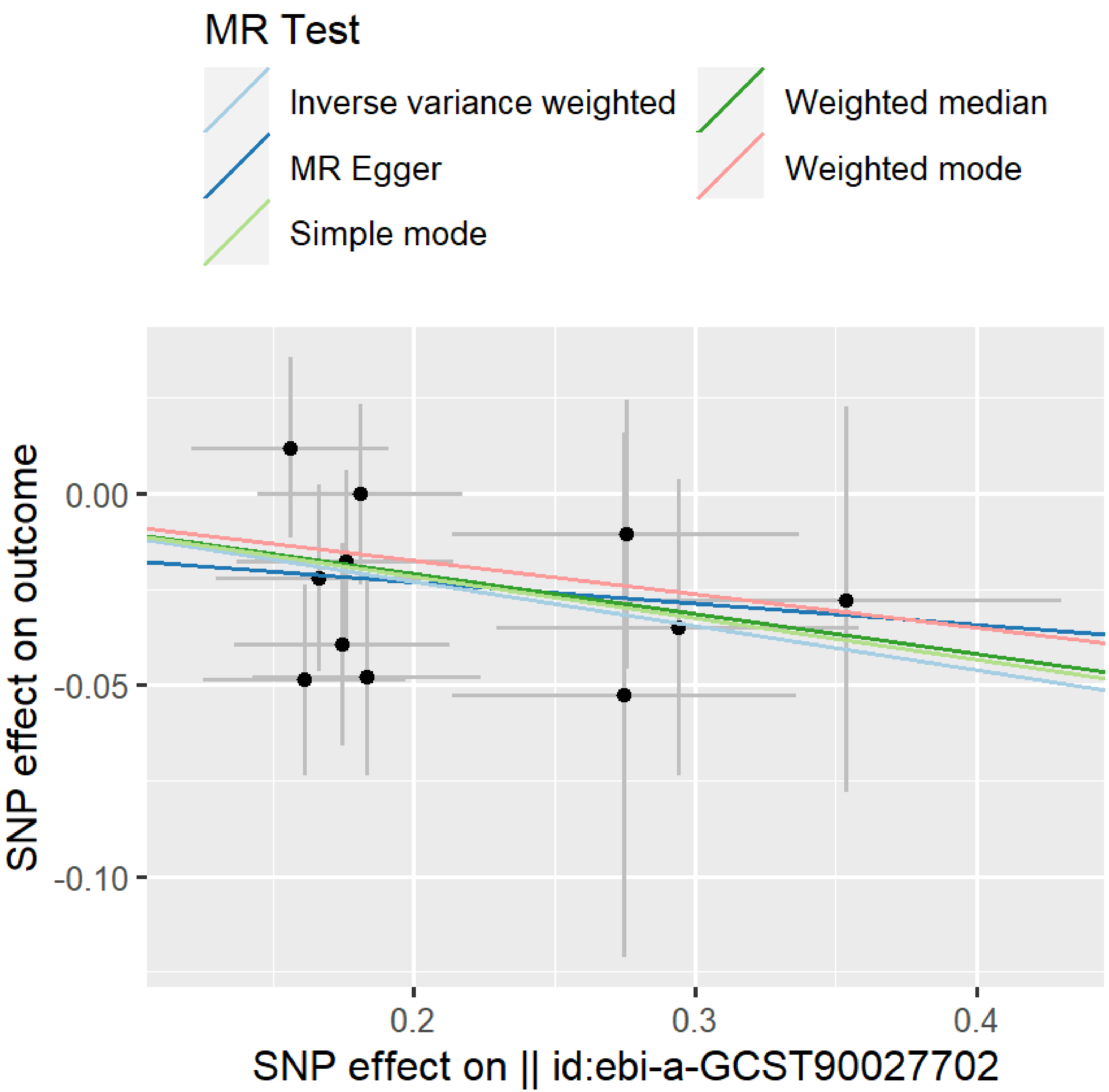

MR Test
Inverse variance weighted
MR Egger
Simple mode
Weighted median
Weighted mode
SNP effect on outcome
0.00
-0.05
-0.10
0.2
0.3
0.4
SNP effect on || id:ebi-a-GCST90027702

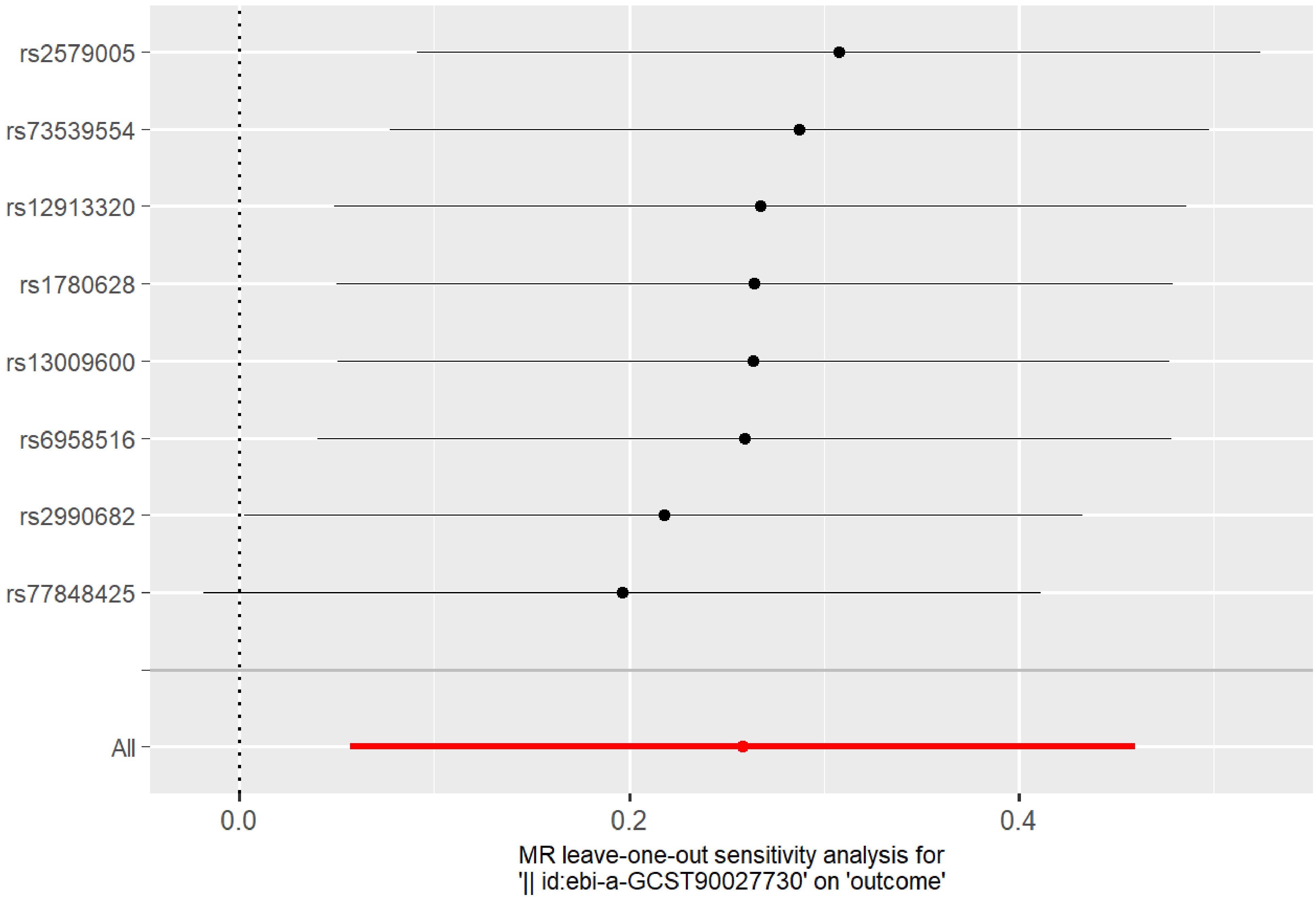
rs2579005
rs73539554
rs12913320
rs1780628
rs13009600
rs6958516
rs2990682
rs77848425
All
0.0
0.2
0.4
MR leave-one-out sensitivity analysis for
'|| id:ebi-a-GCST90027730' on 'outcome'

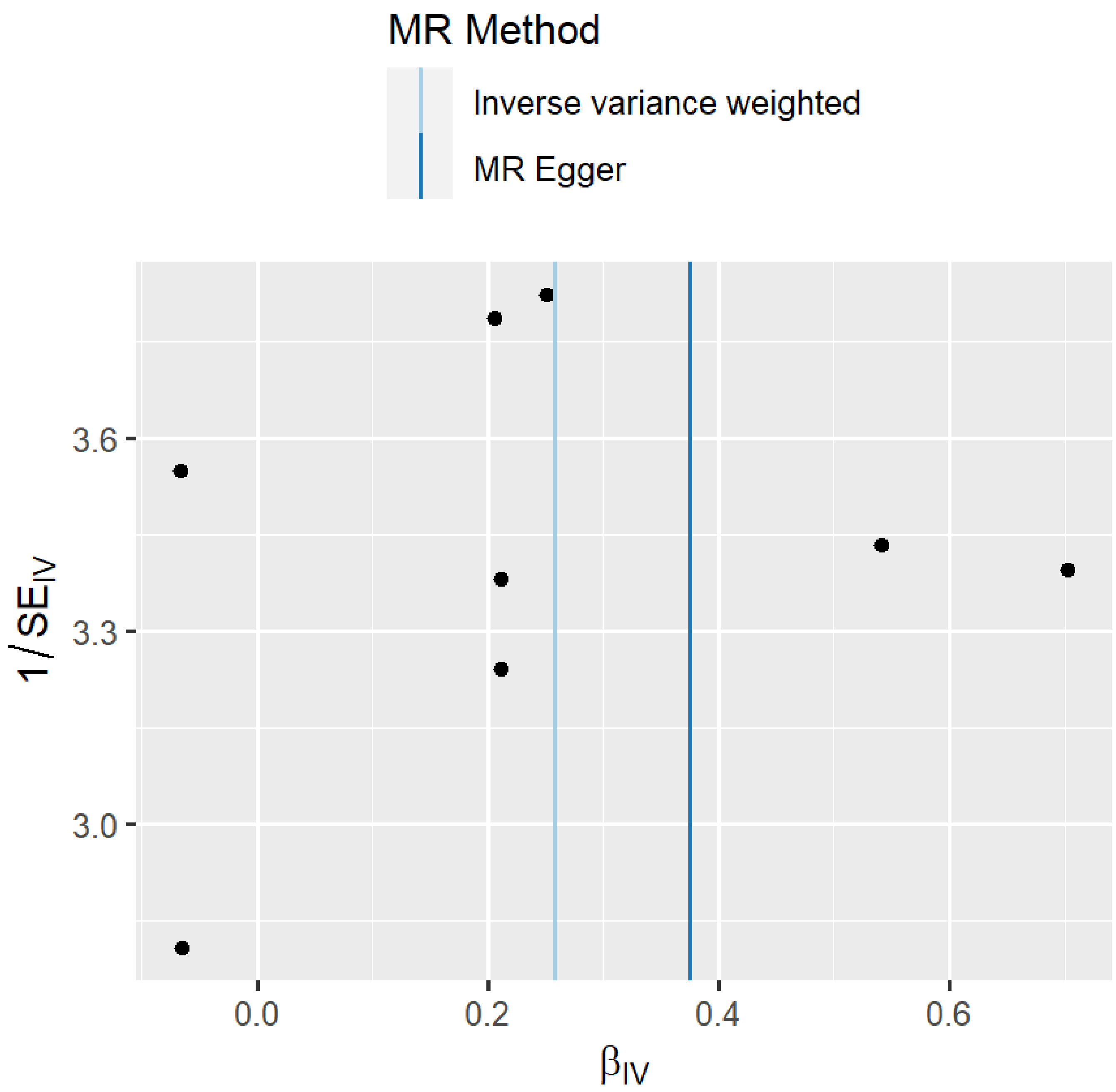
MR Method
Inverse variance weighted
MR Egger
3.6
3.3
3.0
$1/SE_{IV}$
0.0
0.2
0.4
0.6
β_{IV}

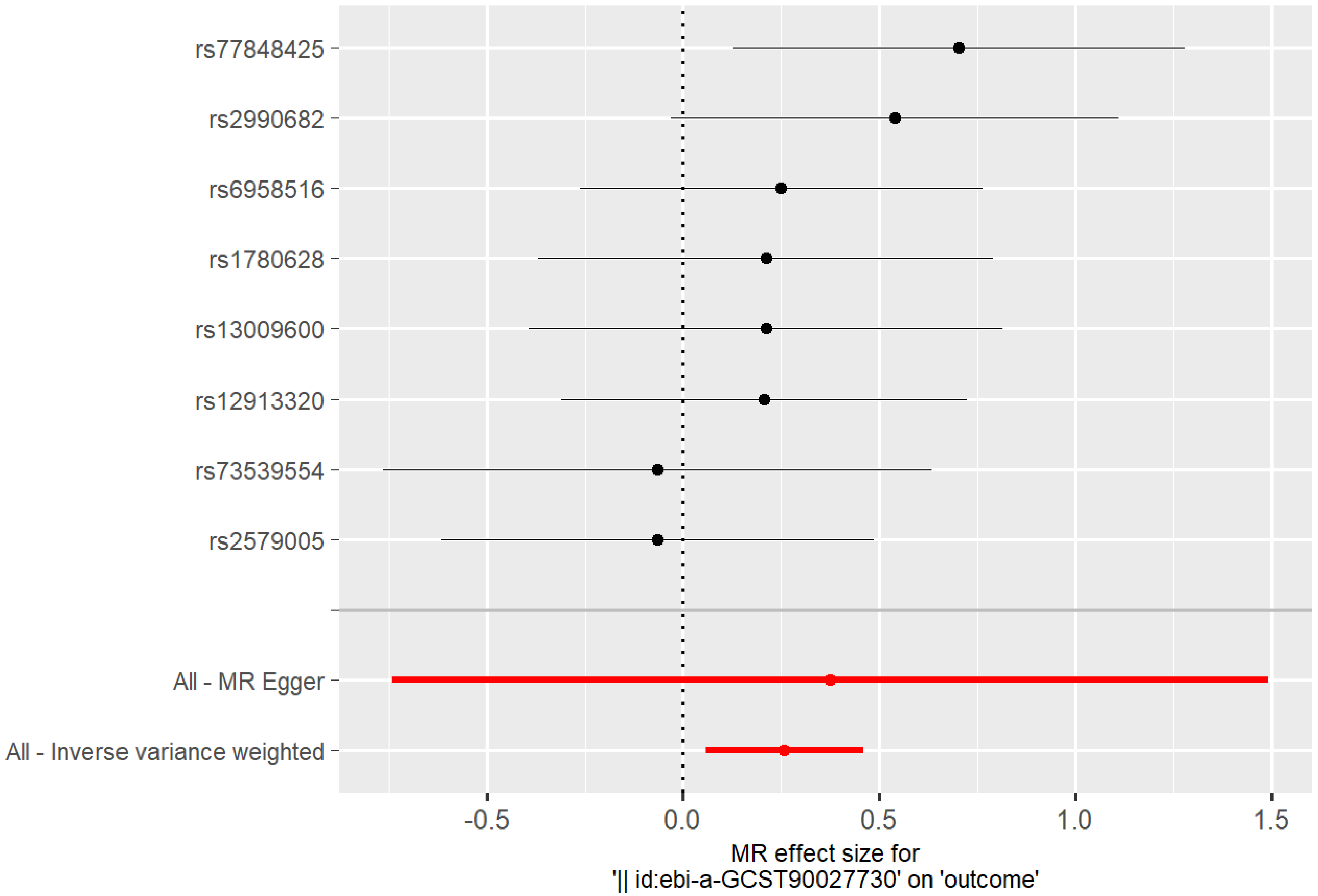
rs77848425
rs2990682
rs6958516
rs1780628
rs13009600
rs12913320
rs73539554
rs2579005
All - MR Egger
All - Inverse variance weighted
-0.5
0.0
0.5
1.0
1.5
MR effect size for
'|| id:ebi-a-GCST90027730' on 'outcome'

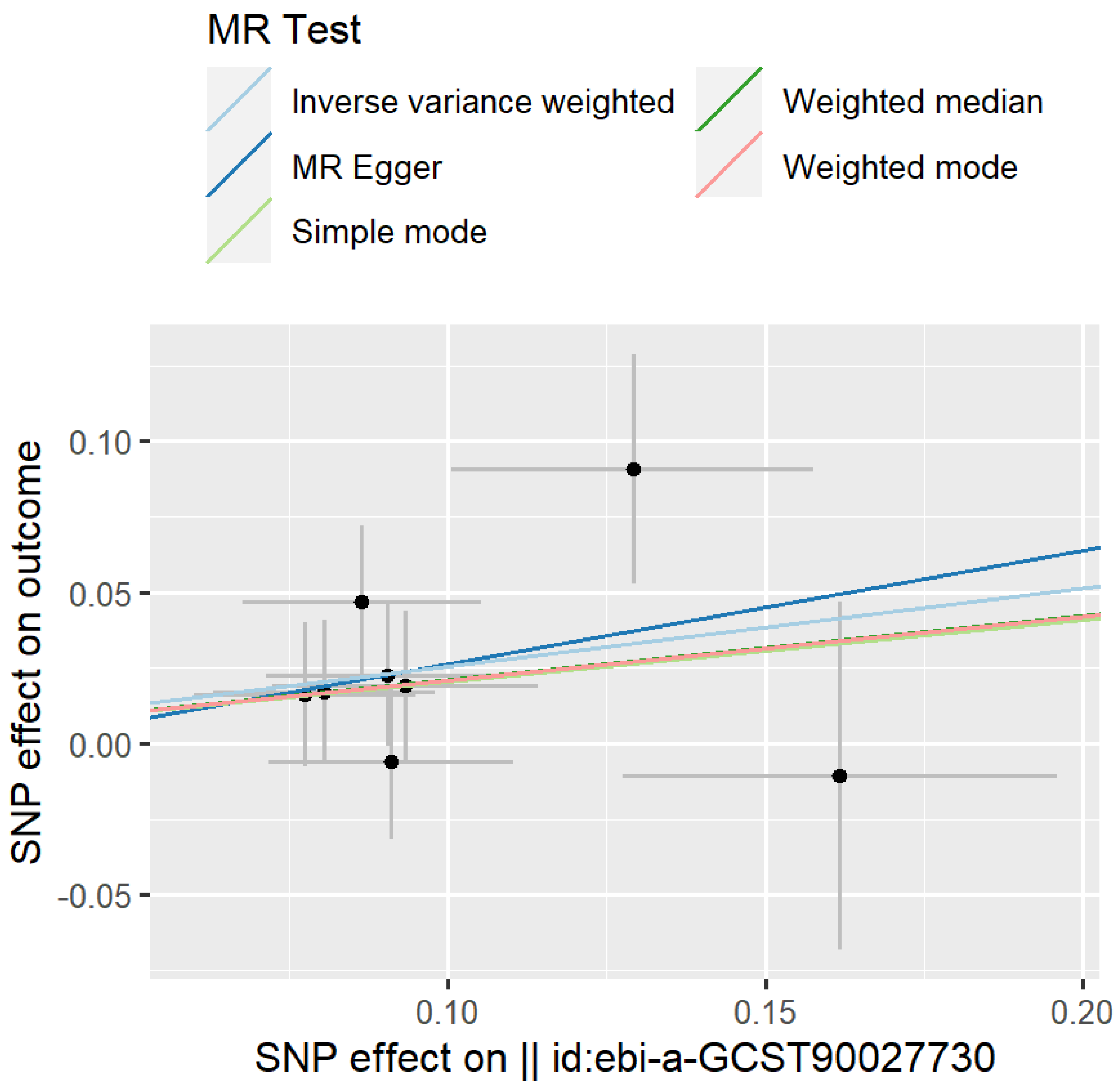
MR Test
Inverse variance weighted
MR Egger
Simple mode
Weighted median
Weighted mode
SNP effect on outcome
0.10
0.05
0.00
-0.05
0.10
0.15
0.20
SNP effect on || id:ebi-a-GCST90027730

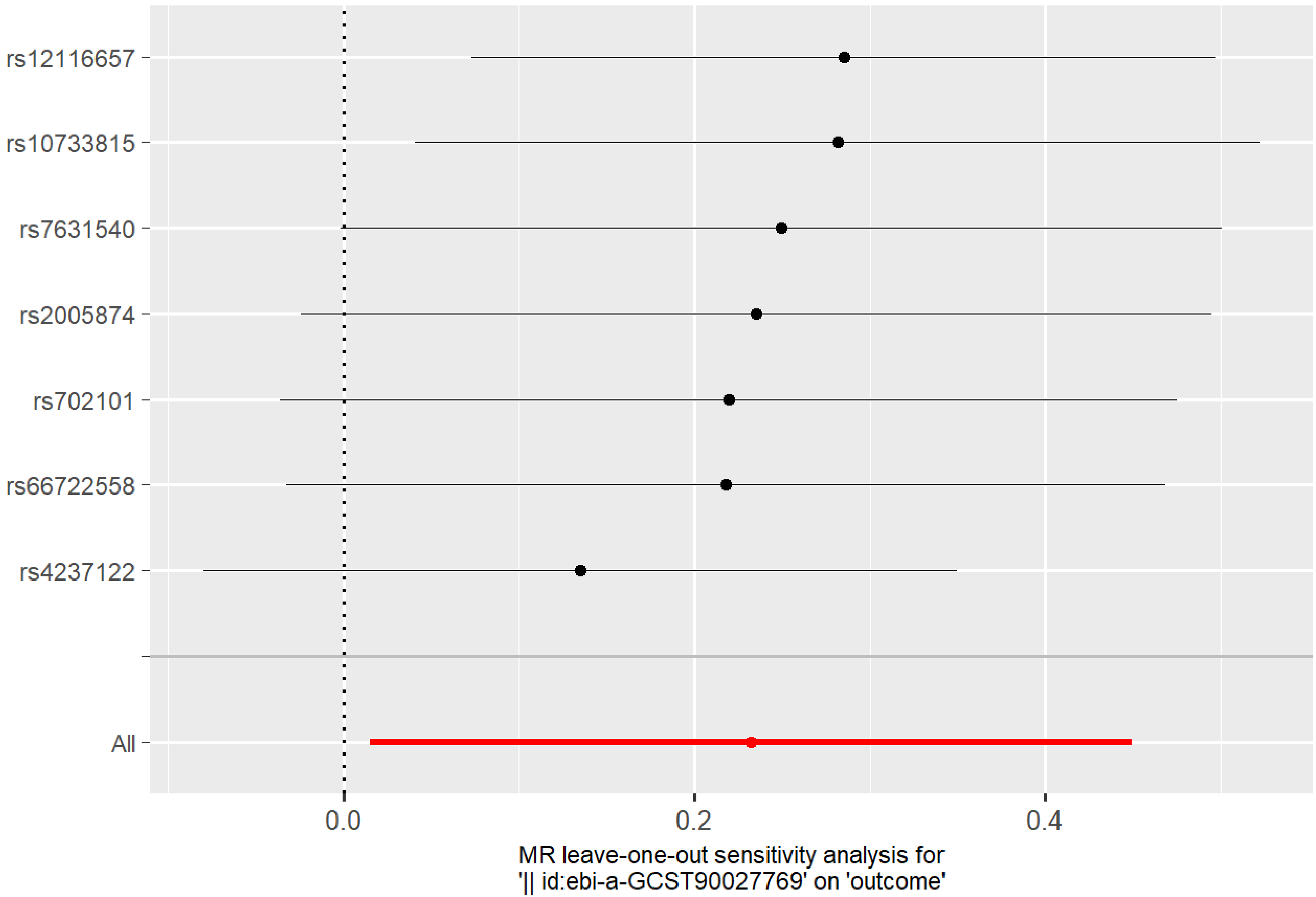
rs12116657
rs10733815
rs7631540
rs2005874
rs702101
rs66722558
rs4237122
All
0.0
0.2
0.4
MR leave-one-out sensitivity analysis for
'|| id:ebi-a-GCST90027769' on 'outcome'

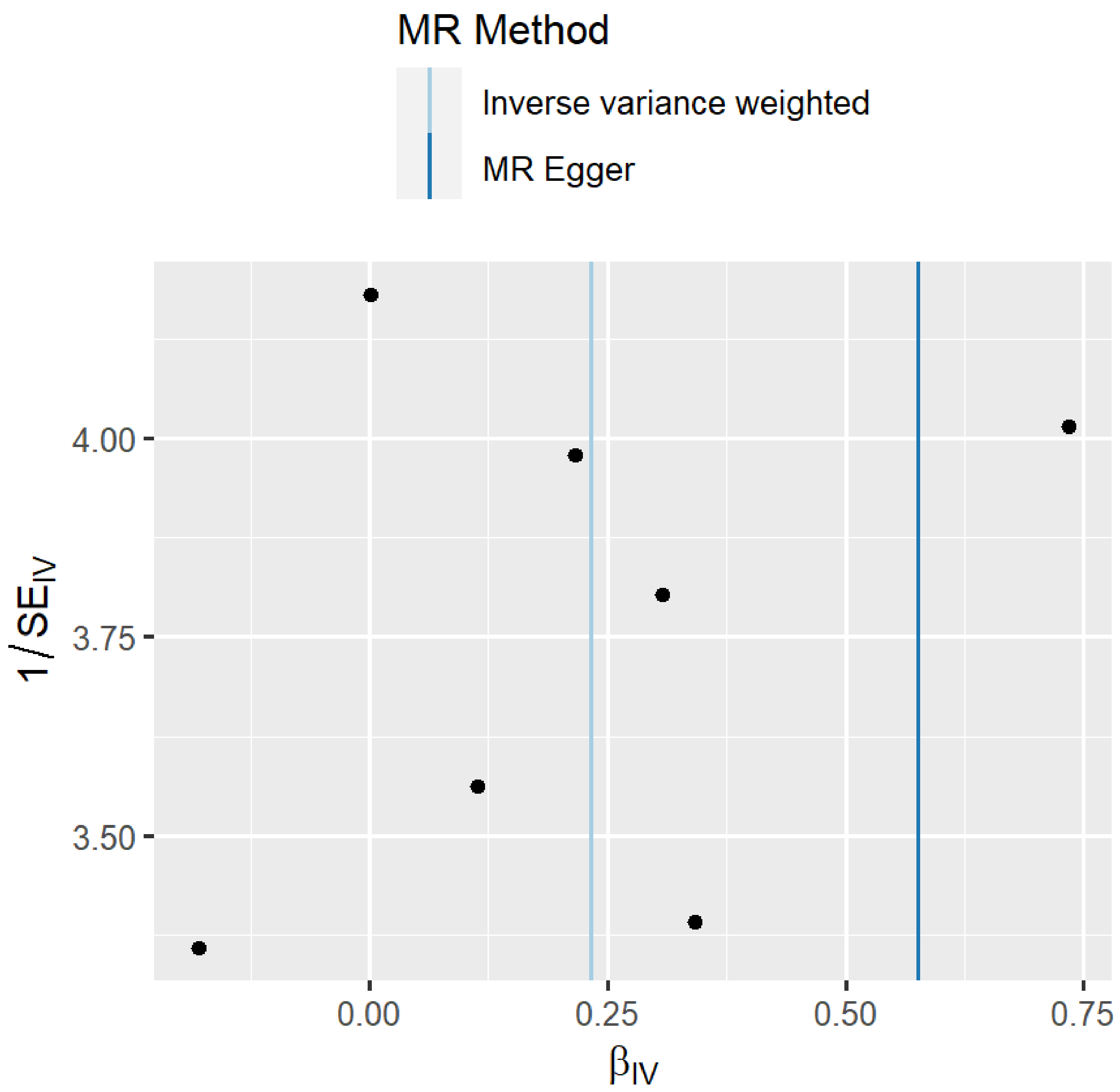
MR Method
Inverse variance weighted
MR Egger
4.00
3.75
3.50
1/SE$_{IV}$
0.00
0.25
0.50
0.75
β$_{IV}$

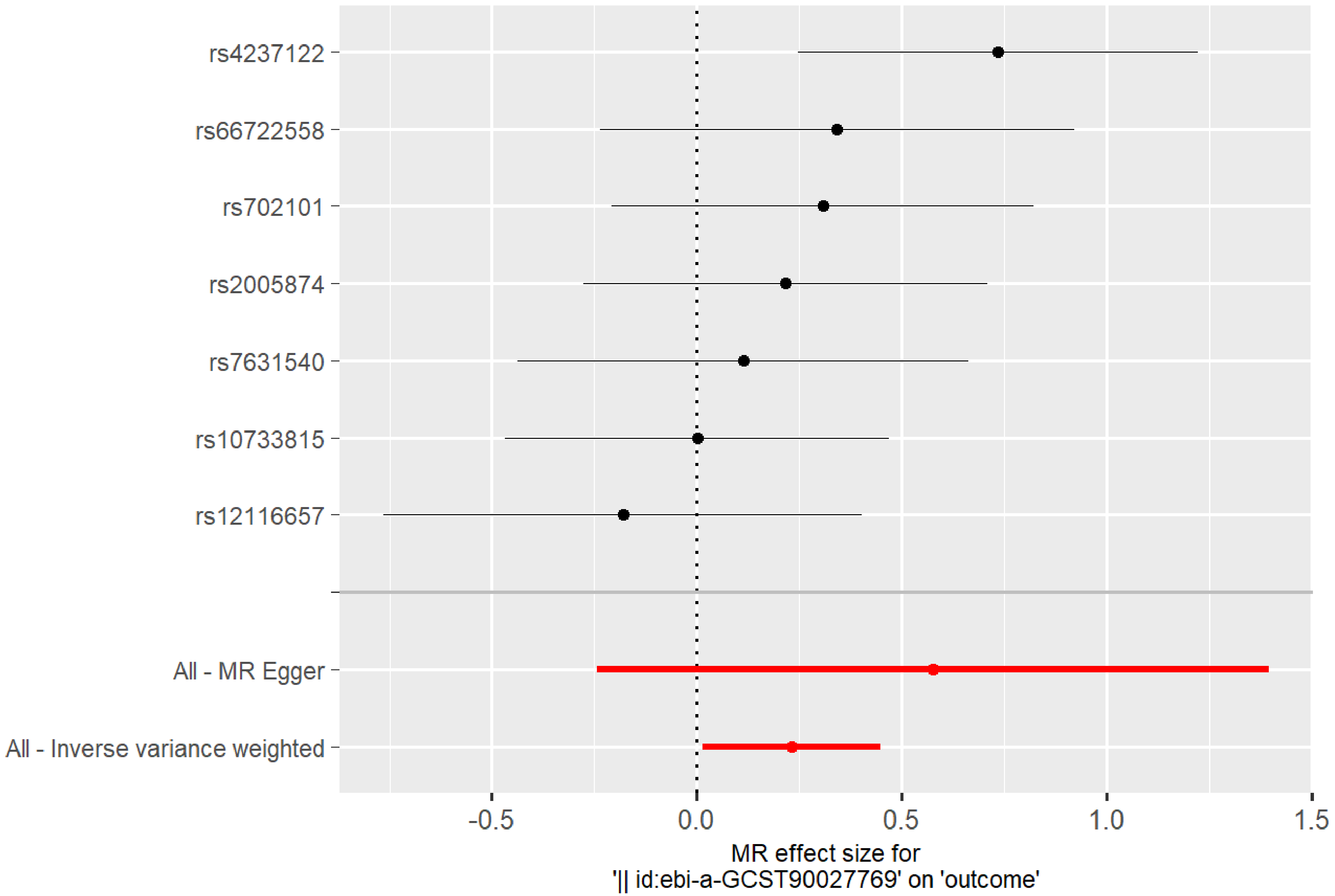
rs4237122
rs66722558
rs702101
rs2005874
rs7631540
rs10733815
rs12116657
All - MR Egger
All - Inverse variance weighted
-0.5
0.0
0.5
1.0
1.5
MR effect size for
'|| id:ebi-a-GCST90027769' on 'outcome'

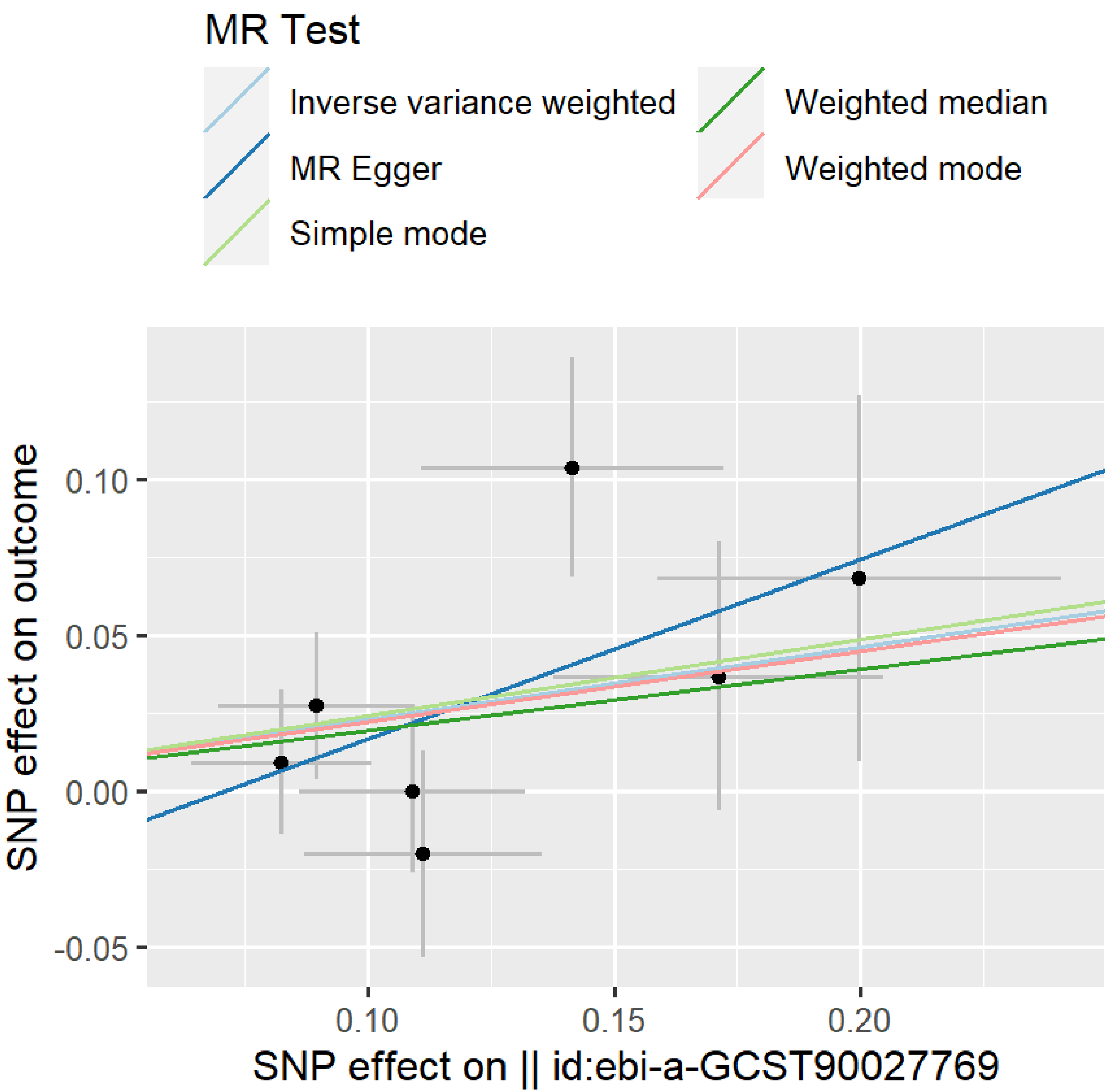
MR Test
Inverse variance weighted
MR Egger
Simple mode
Weighted median
Weighted mode
SNP effect on outcome
0.10
0.05
0.00
-0.05
0.10
0.15
0.20
SNP effect on || id:ebi-a-GCST90027769

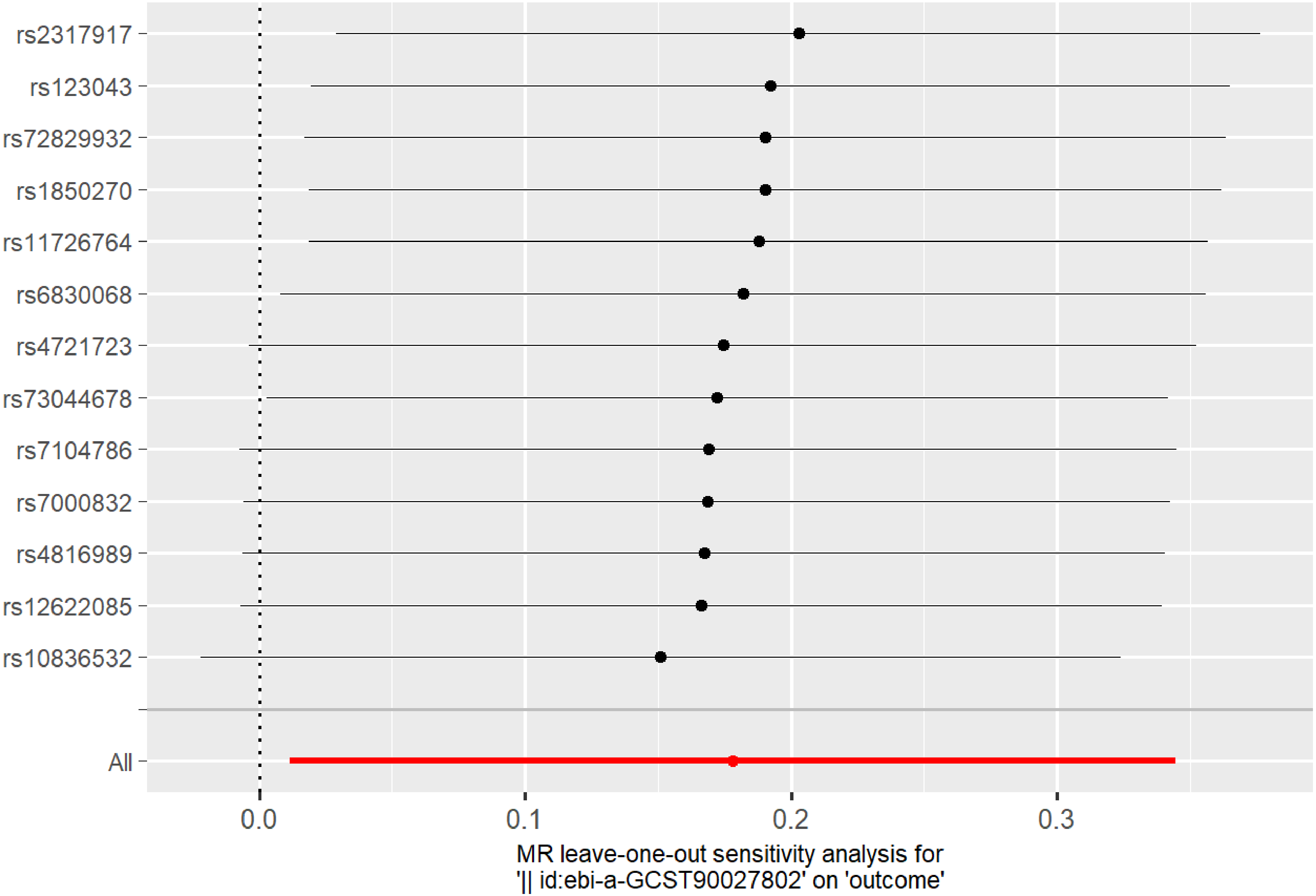
rs2317917
rs123043
rs72829932
rs1850270
rs11726764
rs6830068
rs4721723
rs73044678
rs7104786
rs7000832
rs4816989
rs12622085
rs10836532
All
0.0
0.1
0.2
0.3
MR leave-one-out sensitivity analysis for
'|| id:ebi-a-GCST90027802' on 'outcome'

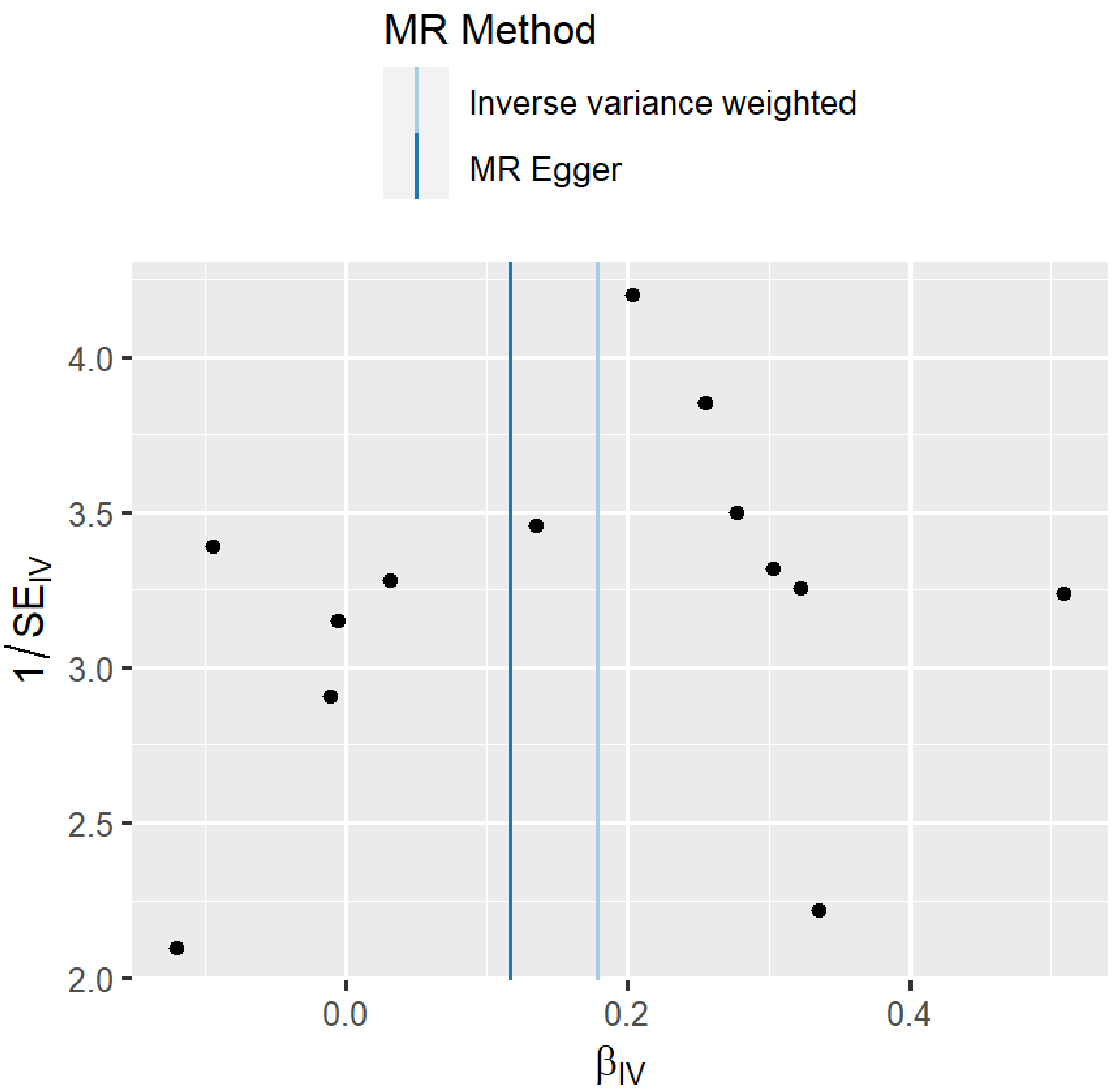

MR Method
Inverse variance weighted
MR Egger
4.0
3.5
3.0
2.5
2.0
1/SE_IV
0.0
0.2
0.4
β_IV

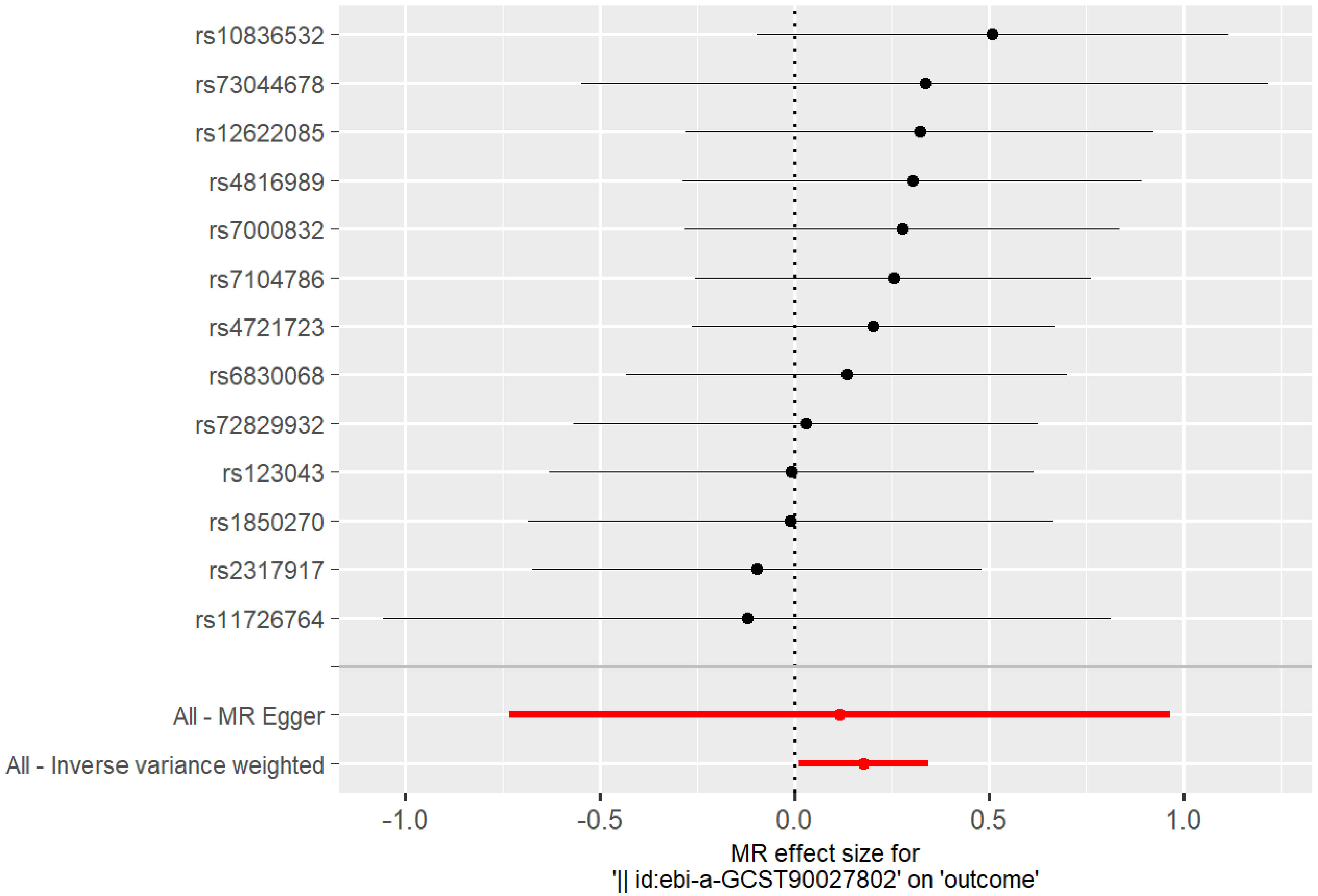
rs10836532
rs73044678
rs12622085
rs4816989
rs7000832
rs7104786
rs4721723
rs6830068
rs72829932
rs123043
rs1850270
rs2317917
rs11726764
All - MR Egger
All - Inverse variance weighted
-1.0
-0.5
0.0
0.5
1.0
MR effect size for
'|| id:ebi-a-GCST90027802' on 'outcome'

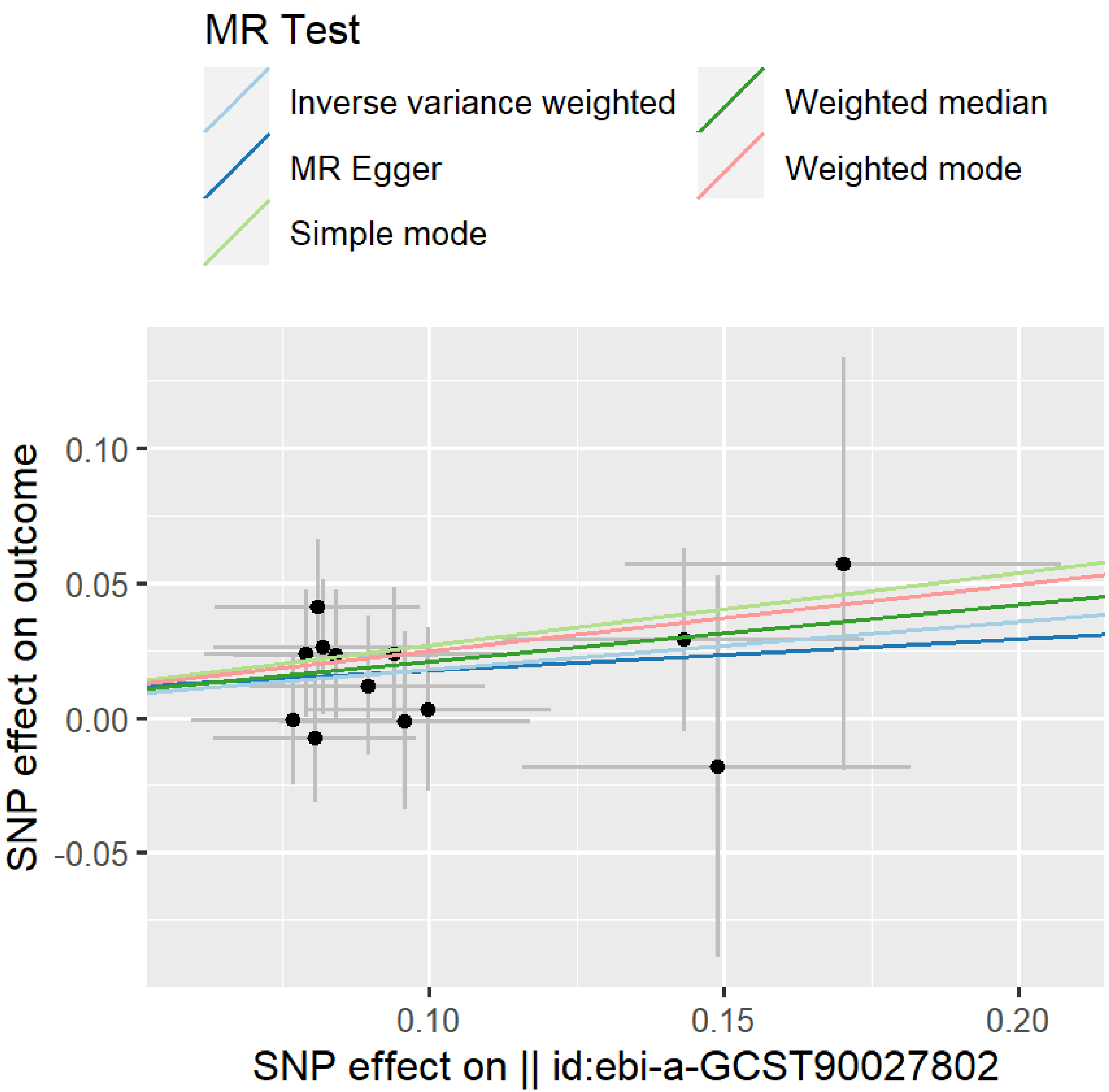
MR Test
Inverse variance weighted
MR Egger
Simple mode
Weighted median
Weighted mode
0.10
0.05
0.00
-0.05
SNP effect on outcome
0.10
0.15
0.20
SNP effect on || id:ebi-a-GCST90027802

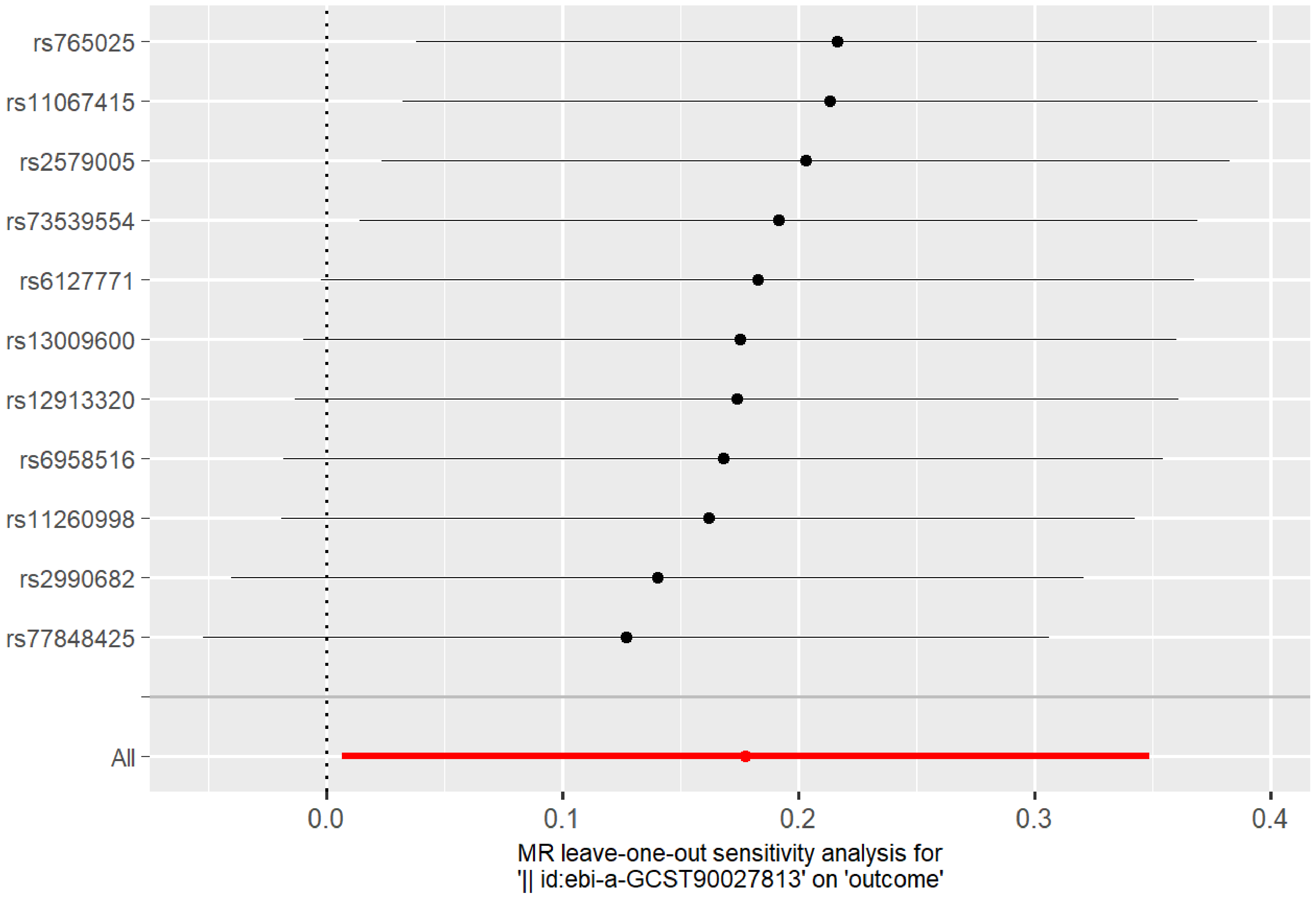
rs765025
rs11067415
rs2579005
rs73539554
rs6127771
rs13009600
rs12913320
rs6958516
rs11260998
rs2990682
rs77848425
All
0.0
0.1
0.2
0.3
0.4
MR leave-one-out sensitivity analysis for
'|| id:ebi-a-GCST90027813' on 'outcome'

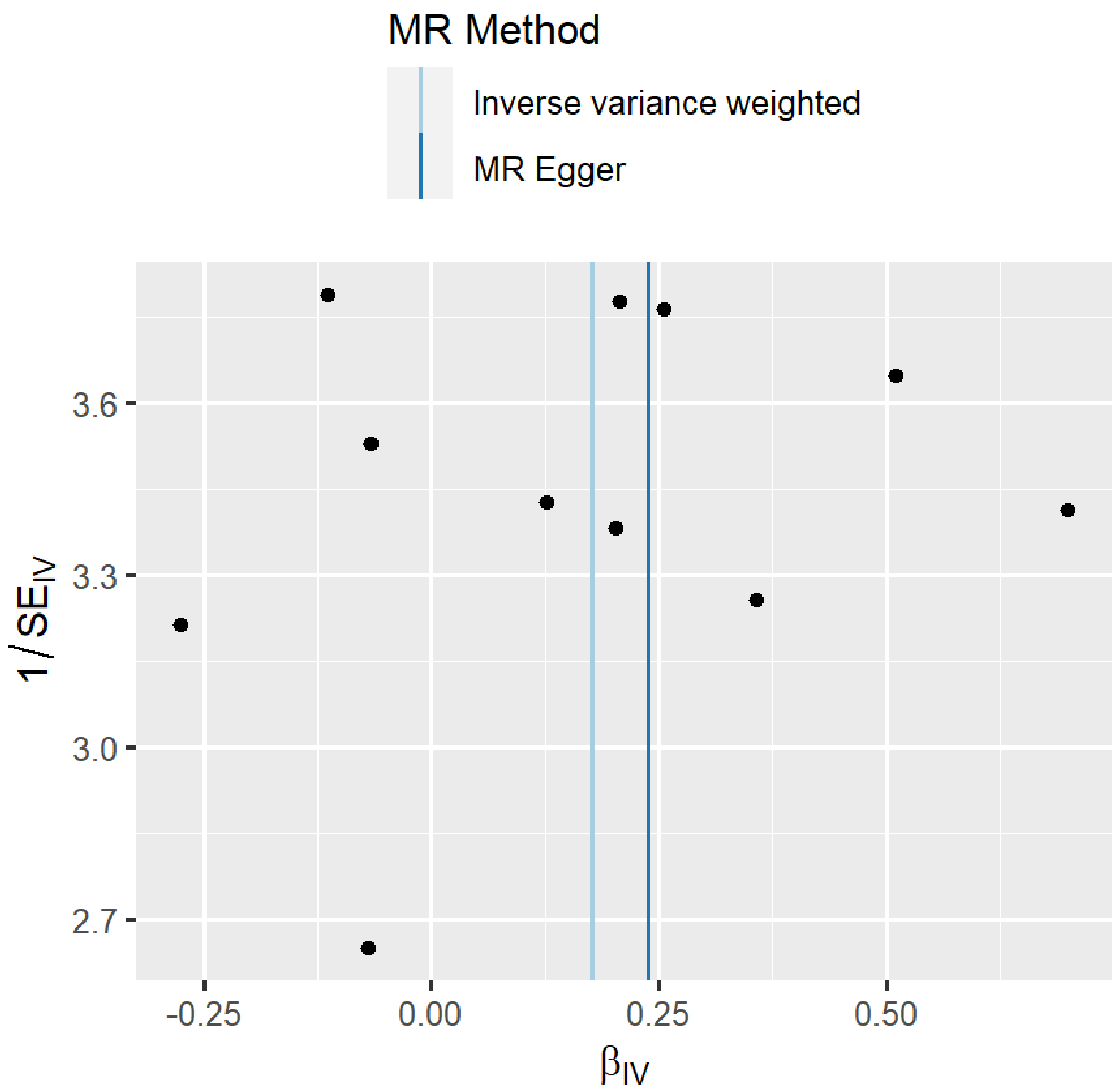
MR Method
Inverse variance weighted
MR Egger
3.6
3.3
3.0
2.7
1/SE_IV
-0.25
0.00
0.25
0.50
β_IV

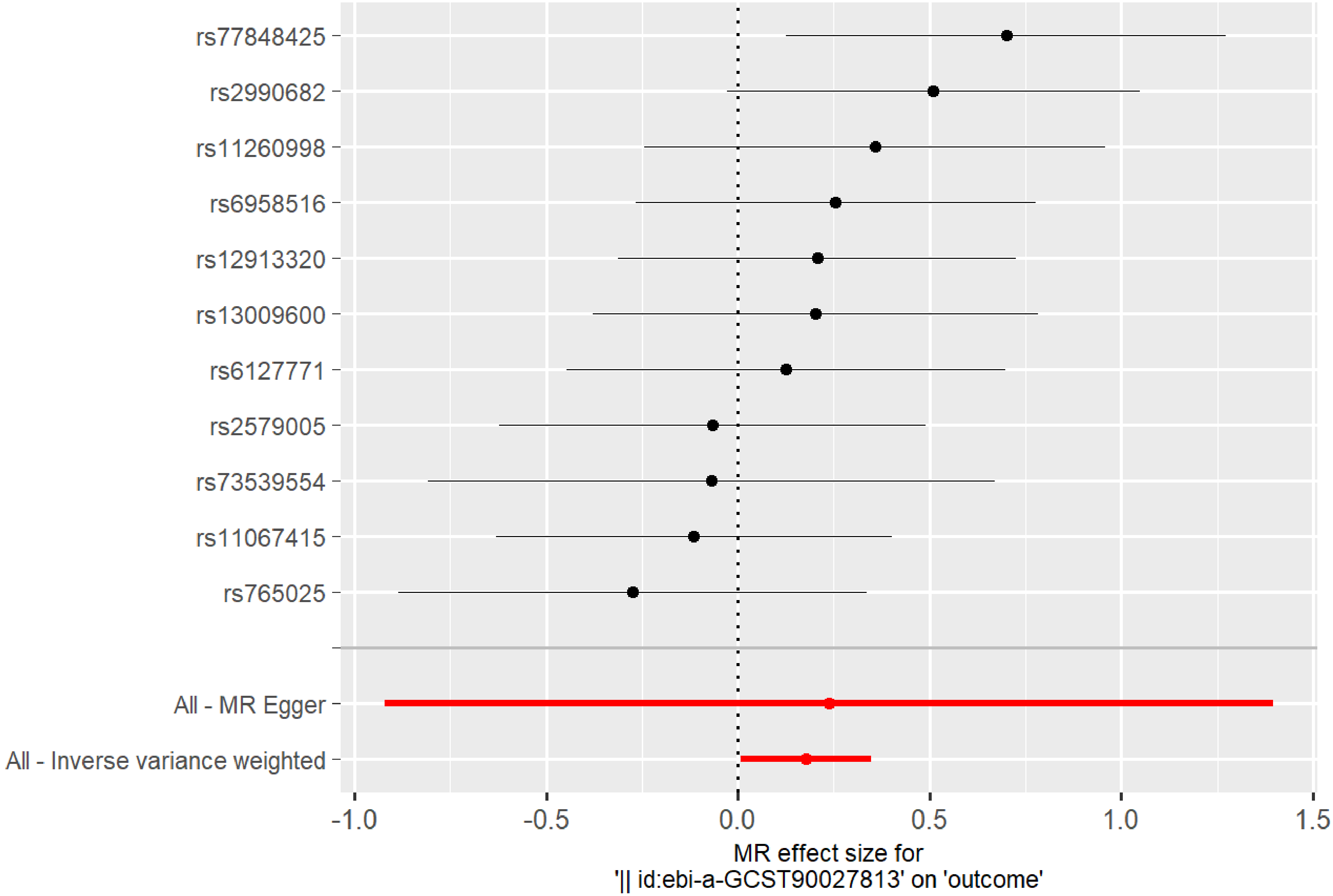
rs77848425
rs2990682
rs11260998
rs6958516
rs12913320
rs13009600
rs6127771
rs2579005
rs73539554
rs11067415
rs765025
All - MR Egger
All - Inverse variance weighted
-1.0
-0.5
0.0
0.5
1.0
1.5
MR effect size for
'|| id:ebi-a-GCST90027813' on 'outcome'

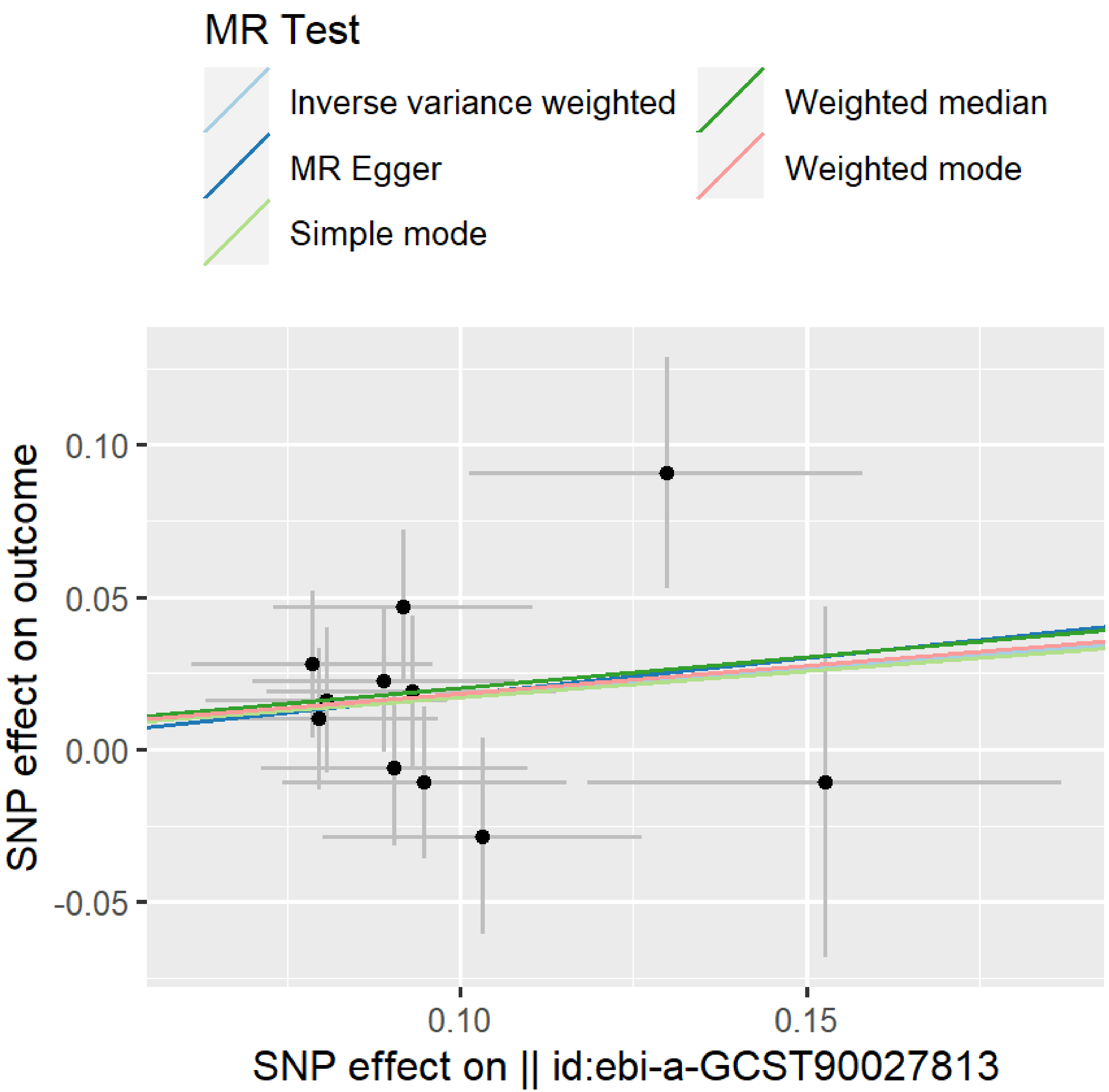

MR Test
Inverse variance weighted
MR Egger
Simple mode
Weighted median
Weighted mode
0.10
0.05
0.00
-0.05
SNP effect on outcome
0.10
0.15
SNP effect on || id:ebi-a-GCST90027813

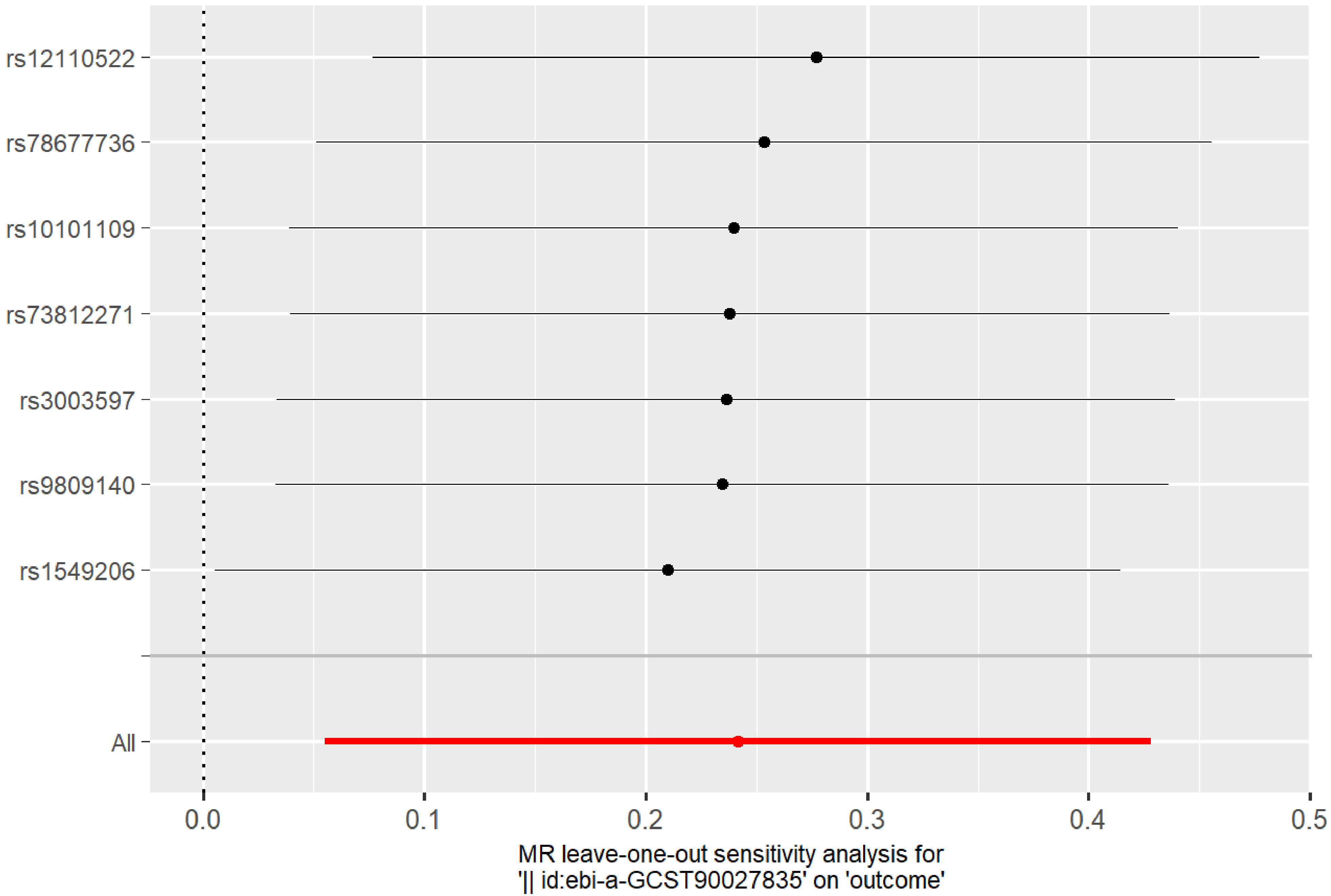
rs12110522
rs78677736
rs10101109
rs73812271
rs3003597
rs9809140
rs1549206
All
0.0
0.1
0.2
0.3
0.4
0.5
MR leave-one-out sensitivity analysis for
'|| id:ebi-a-GCST90027835' on 'outcome'

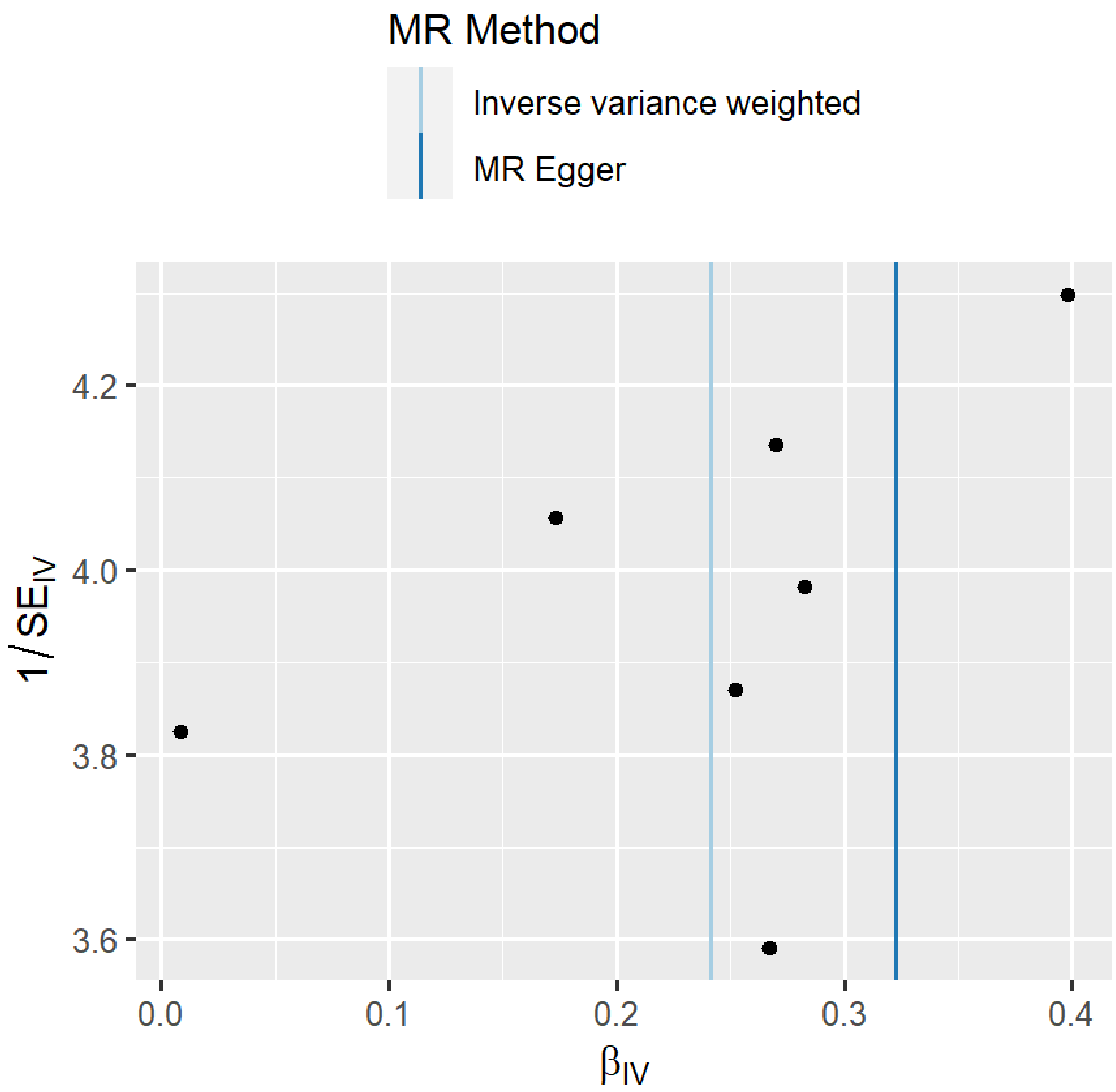
MR Method
Inverse variance weighted
MR Egger
4.2
4.0
3.8
3.6
1/SE_IV
0.0
0.1
0.2
0.3
0.4
β_IV

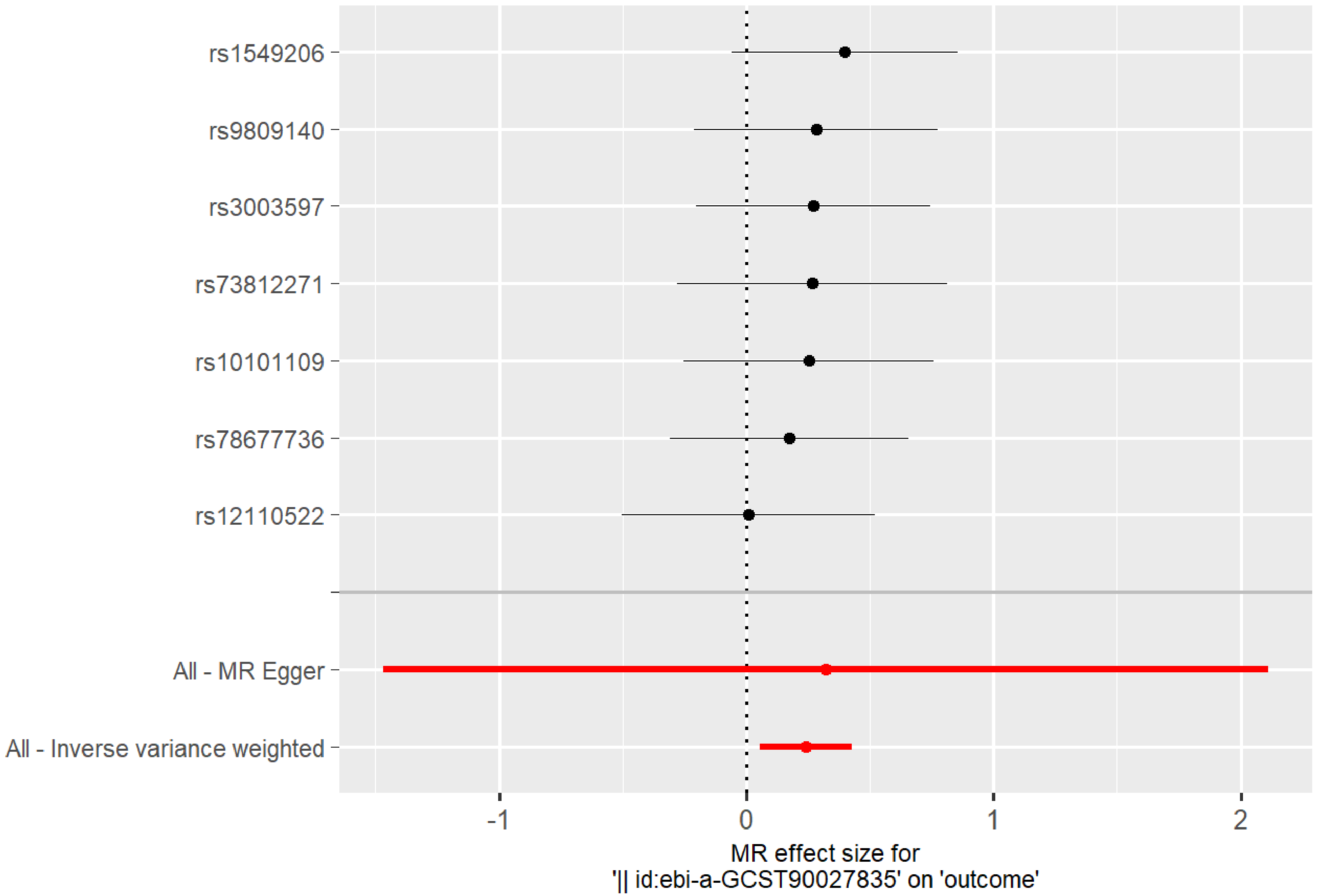
rs1549206
rs9809140
rs3003597
rs73812271
rs10101109
rs78677736
rs12110522
All - MR Egger
All - Inverse variance weighted
-1
0
1
2
MR effect size for
'|| id:ebi-a-GCST90027835' on 'outcome'

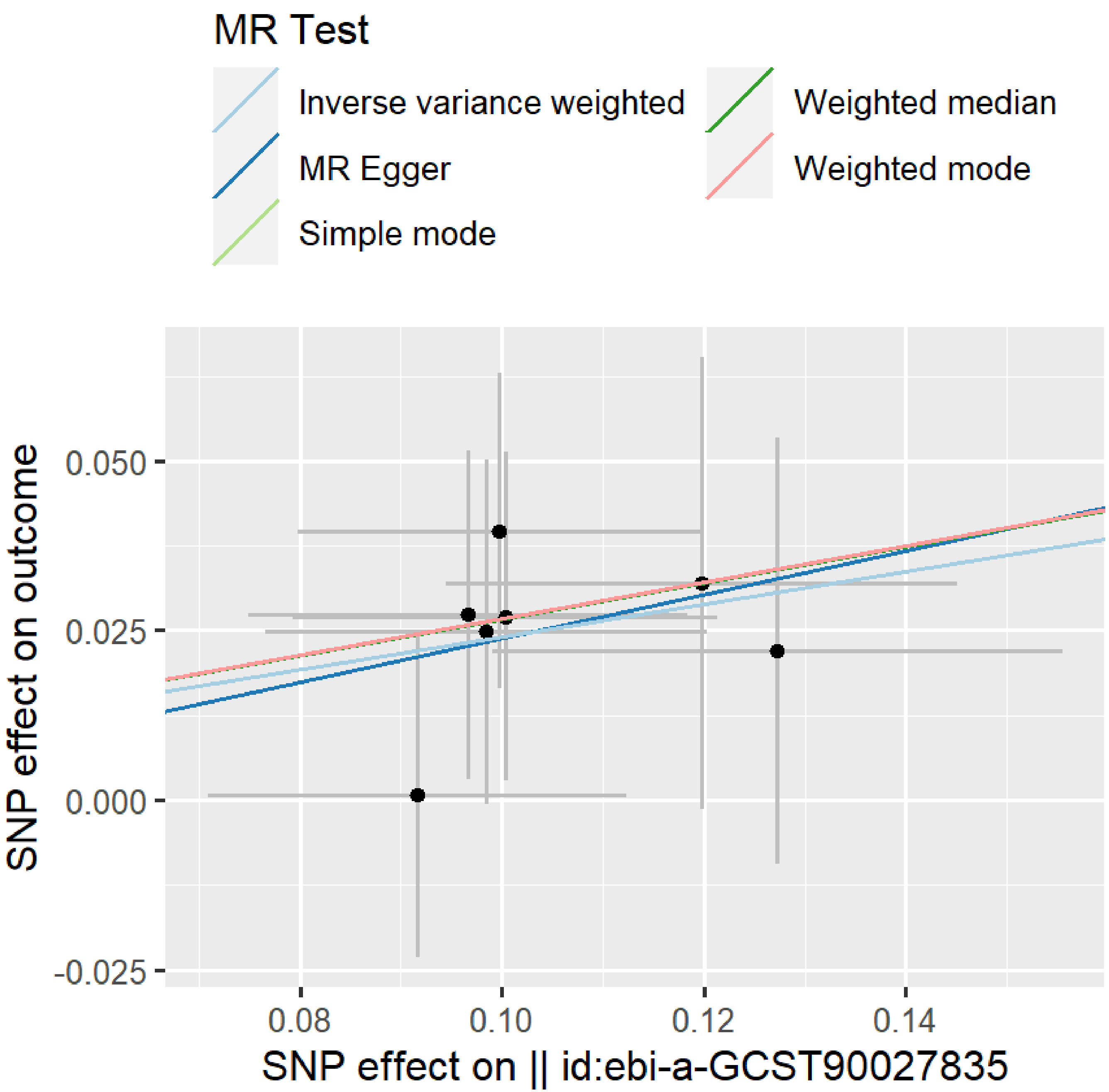

MR Test
Inverse variance weighted
MR Egger
Simple mode
Weighted median
Weighted mode
0.050
0.025
0.000
-0.025
SNP effect on outcome
0.08
0.10
0.12
0.14
SNP effect on || id:ebi-a-GCST90027835

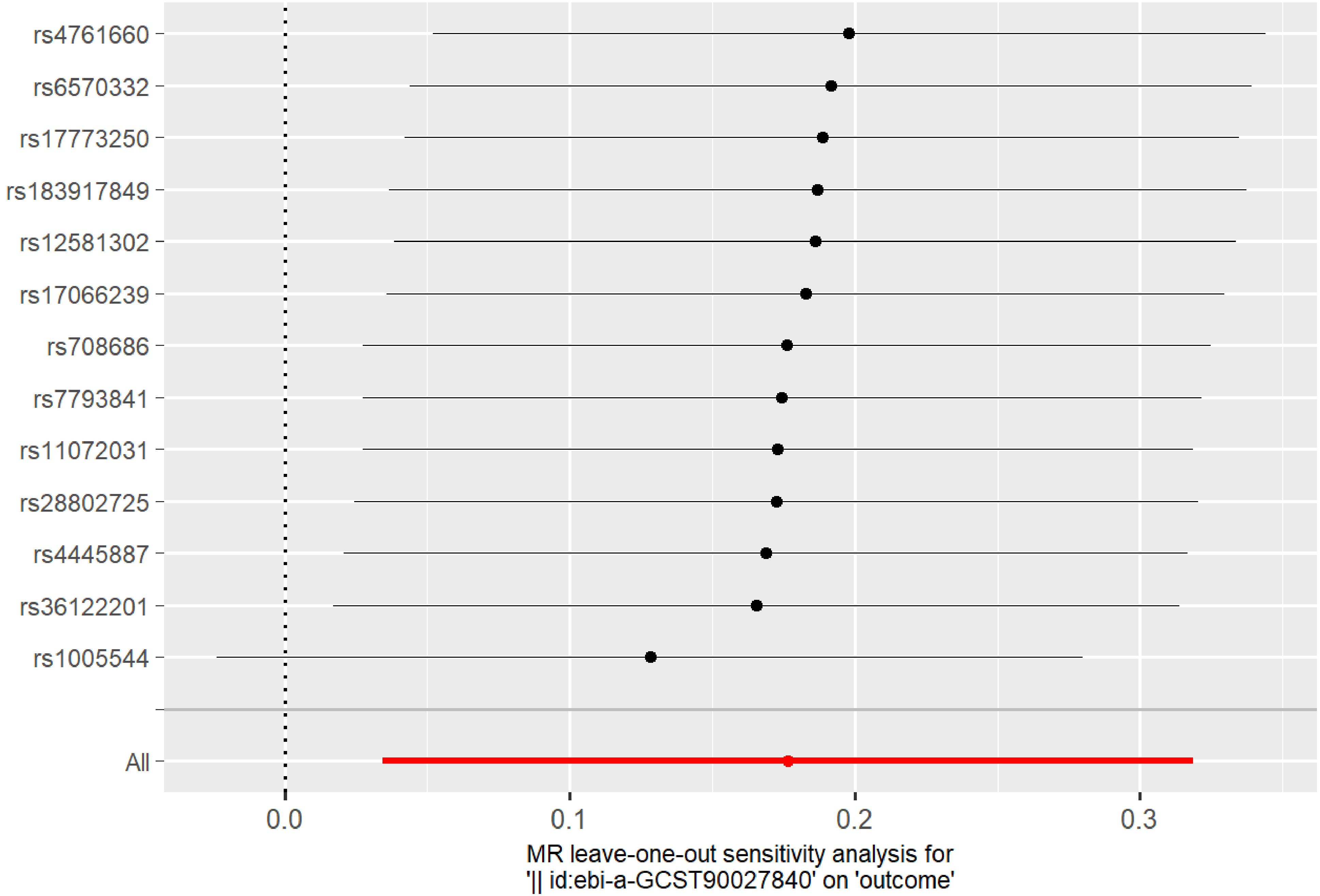
rs4761660
rs6570332
rs17773250
rs183917849
rs12581302
rs17066239
rs708686
rs7793841
rs11072031
rs28802725
rs4445887
rs36122201
rs1005544
All
0.0
0.1
0.2
0.3
MR leave-one-out sensitivity analysis for
'|| id:ebi-a-GCST90027840' on 'outcome'

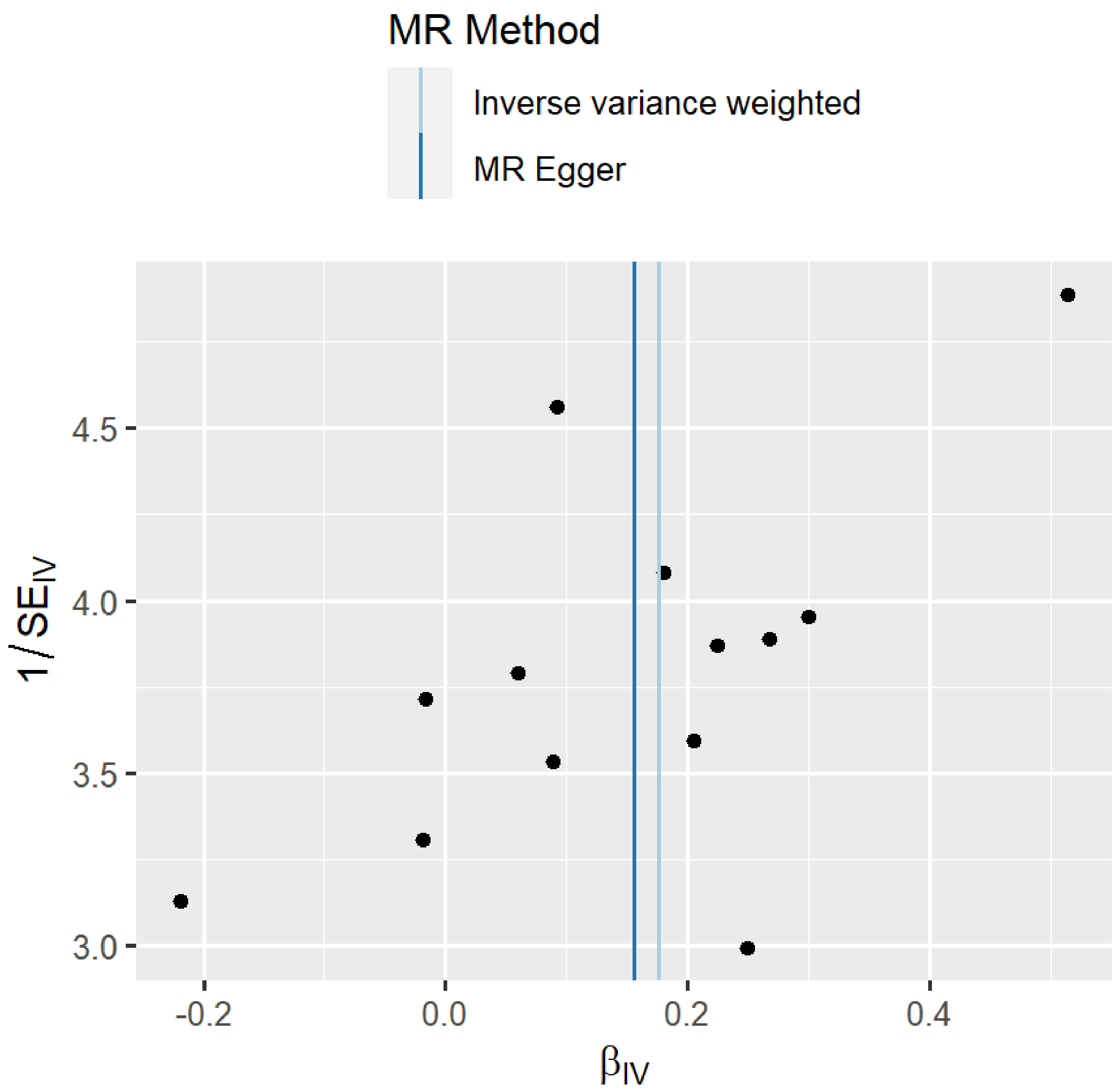
MR Method
Inverse variance weighted
MR Egger
4.5
4.0
3.5
3.0
$1/SE_{IV}$
-0.2
0.0
0.2
0.4
β_{IV}

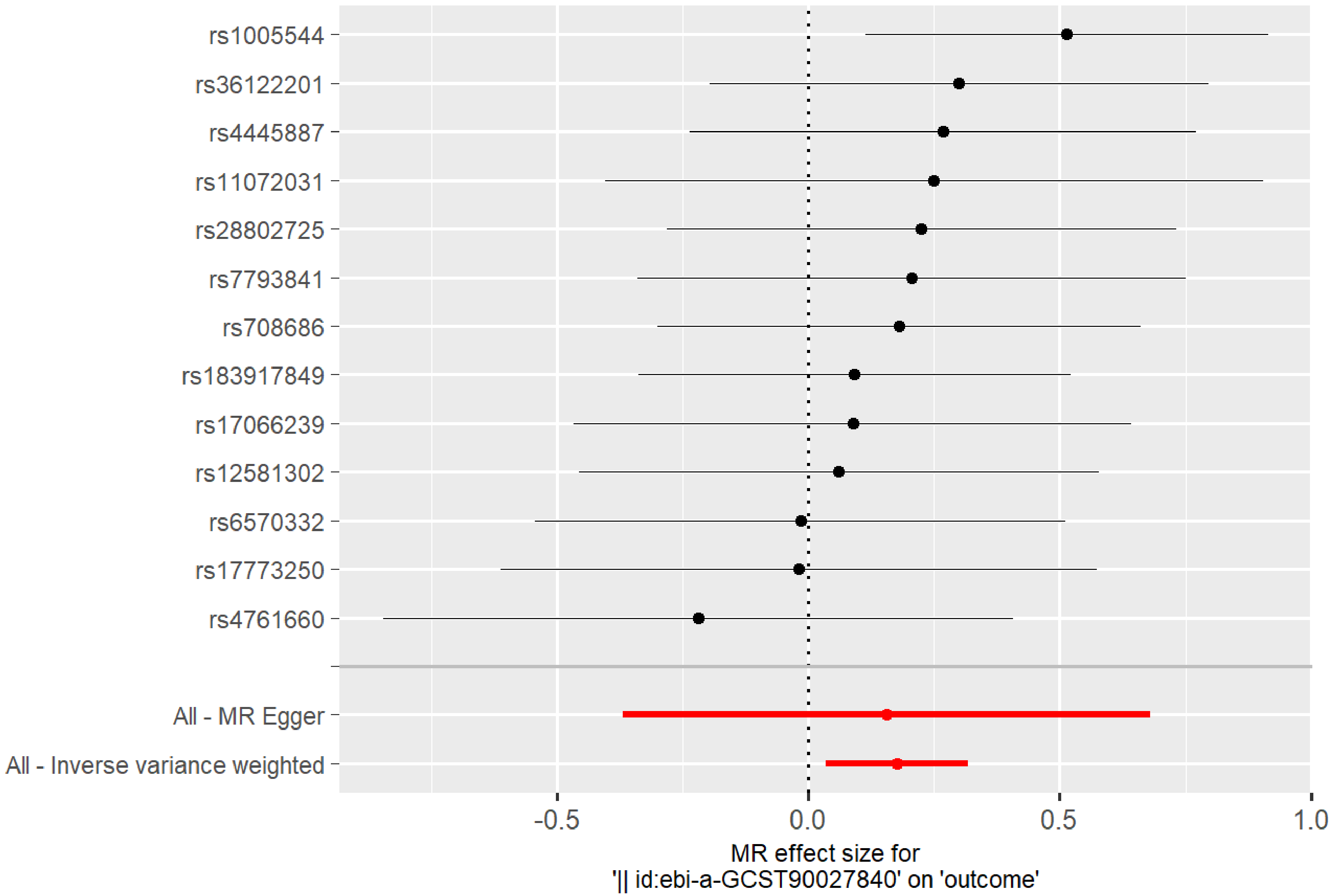
rs1005544
rs36122201
rs4445887
rs11072031
rs28802725
rs7793841
rs708686
rs183917849
rs17066239
rs12581302
rs6570332
rs17773250
rs4761660
All - MR Egger
All - Inverse variance weighted
-0.5
0.0
0.5
1.0
MR effect size for
'|| id:ebi-a-GCST90027840' on 'outcome'

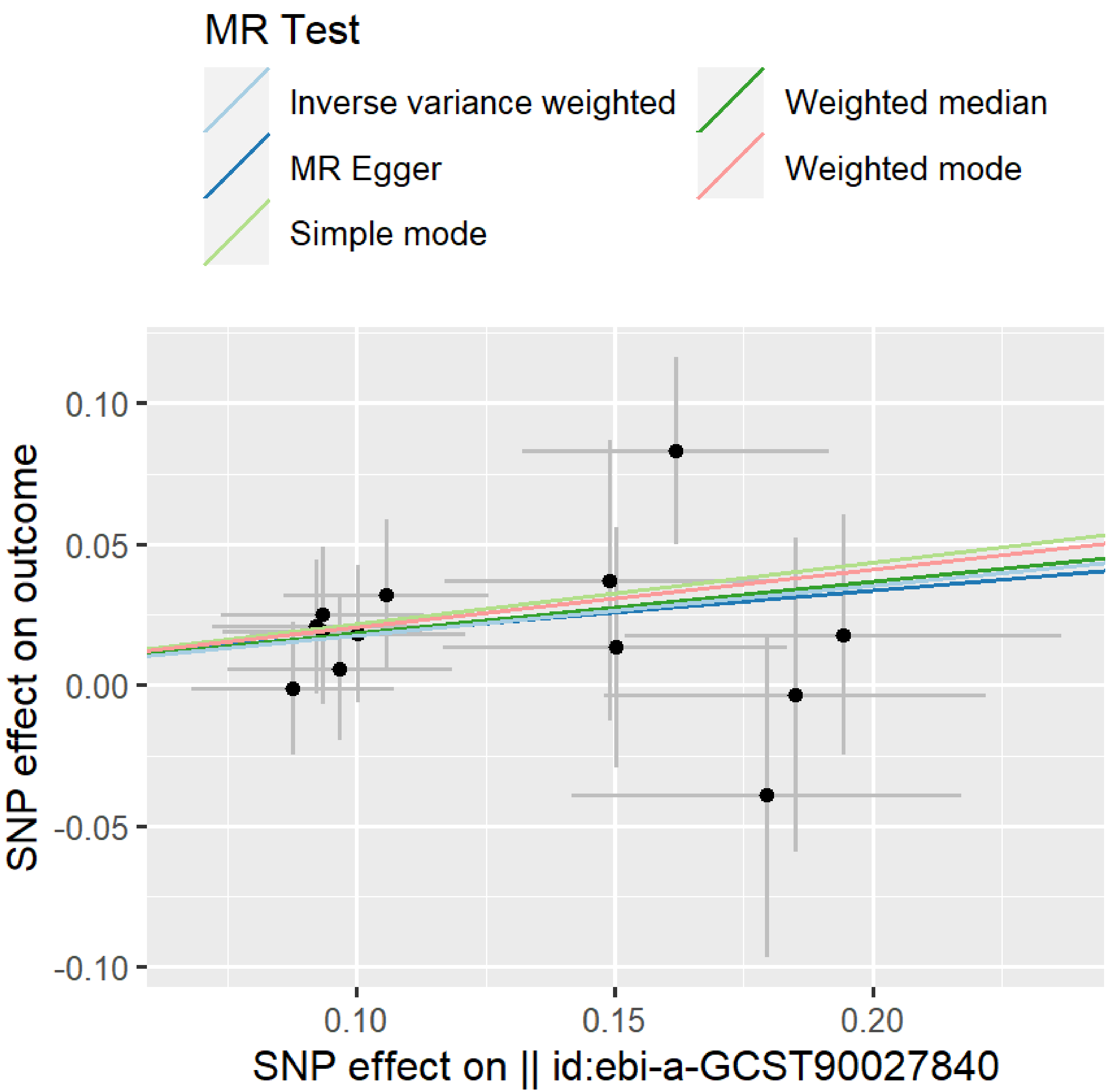
MR Test
Inverse variance weighted
MR Egger
Simple mode
Weighted median
Weighted mode
0.10
0.05
0.00
-0.05
-0.10
SNP effect on outcome
0.10
0.15
0.20
SNP effect on || id:ebi-a-GCST90027840

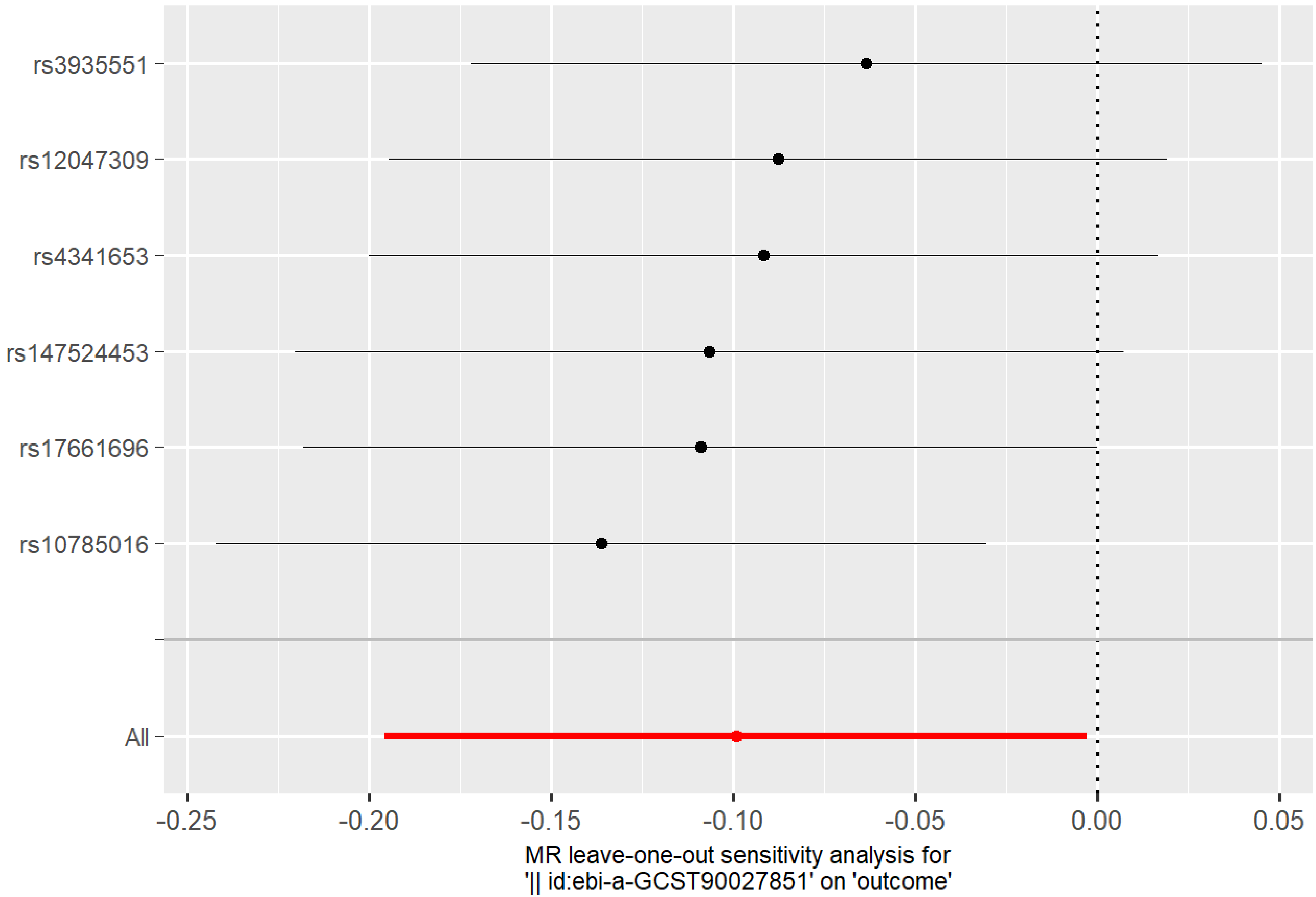
rs3935551
rs12047309
rs4341653
rs147524453
rs17661696
rs10785016
All
-0.25
-0.20
-0.15
-0.10
-0.05
0.00
0.05
MR leave-one-out sensitivity analysis for
'|| id:ebi-a-GCST90027851' on 'outcome'

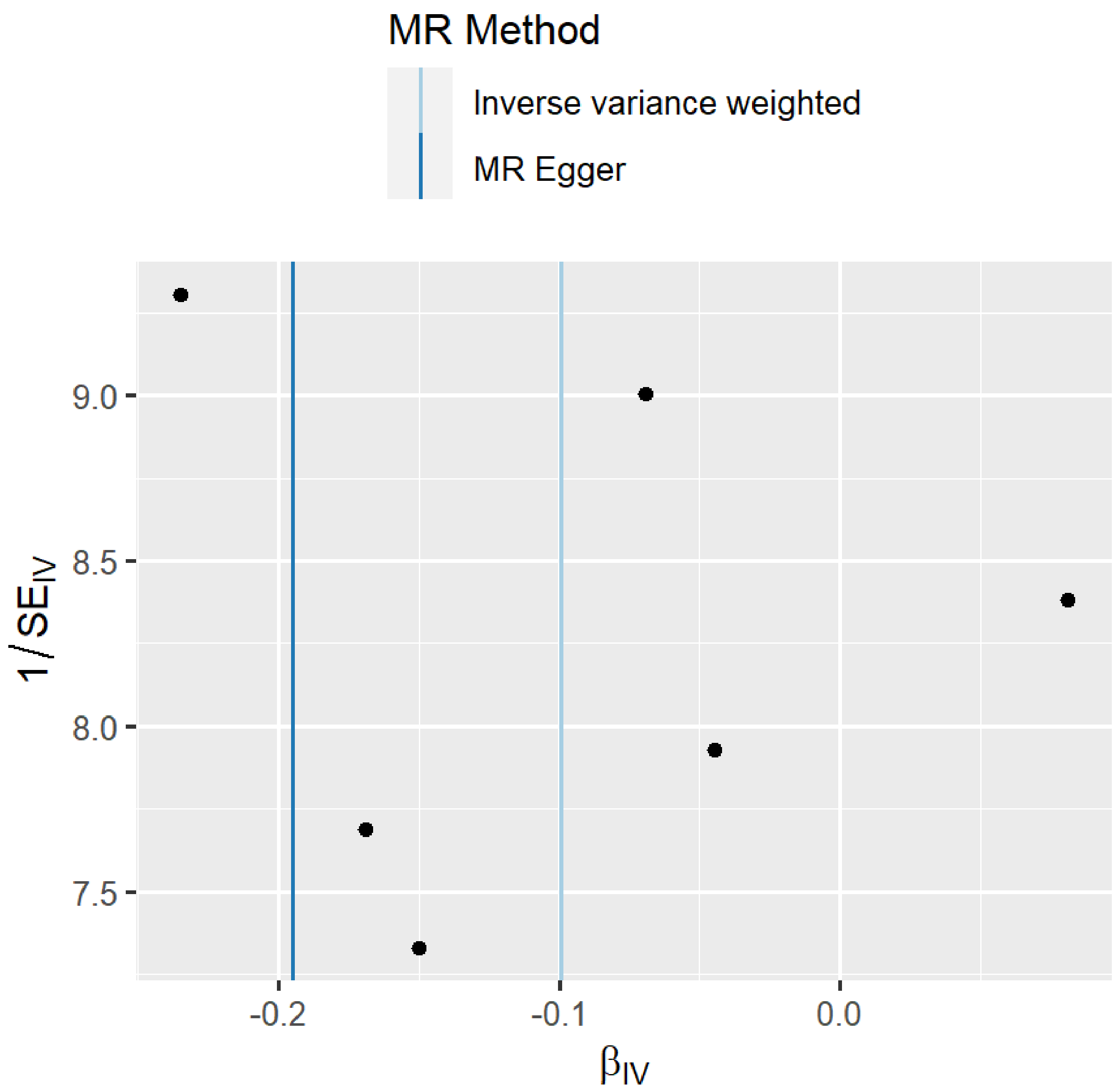
MR Method
Inverse variance weighted
MR Egger
9.0
8.5
8.0
7.5
1/SE_IV
-0.2
-0.1
0.0
β_IV

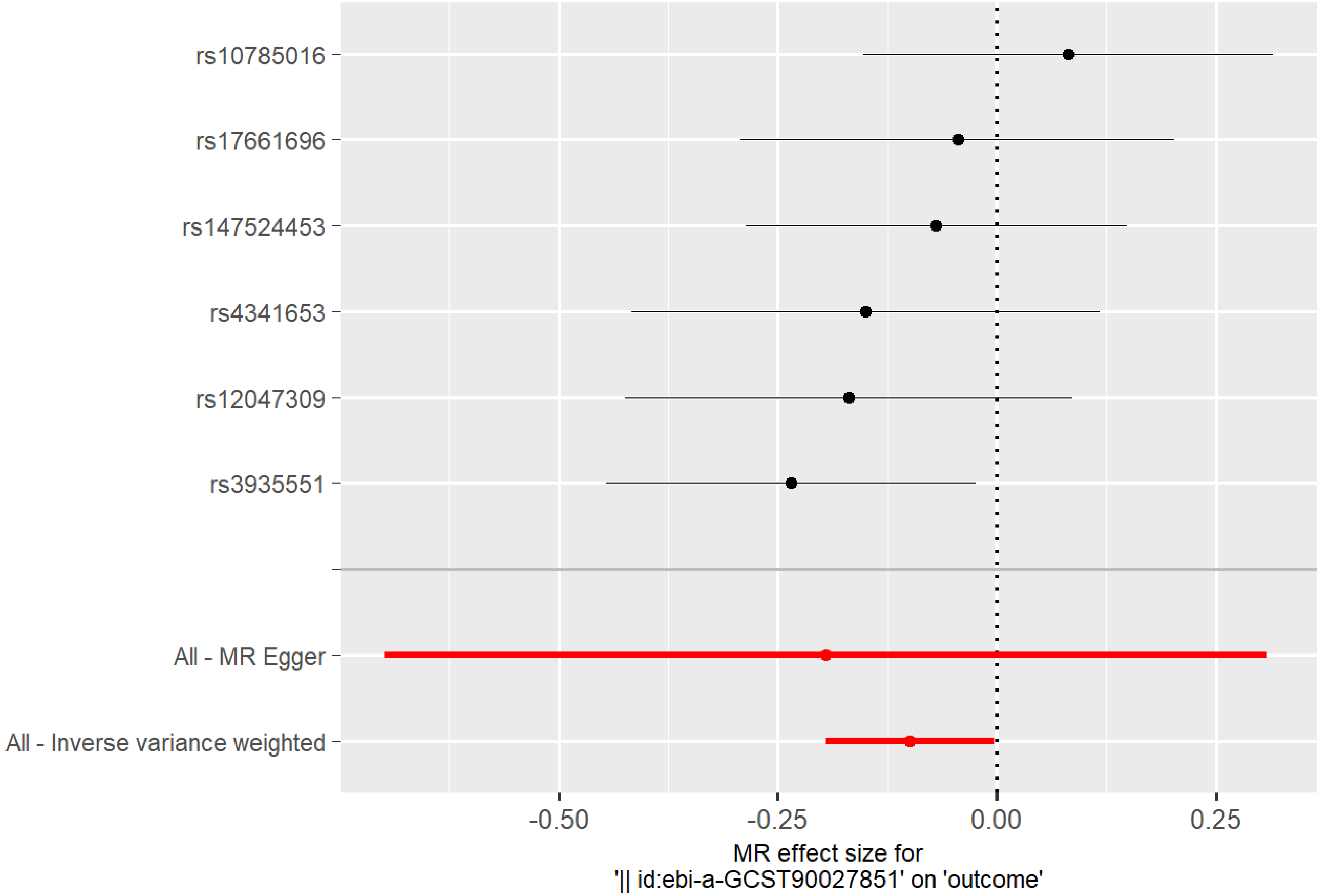
rs10785016
rs17661696
rs147524453
rs4341653
rs12047309
rs3935551
All - MR Egger
All - Inverse variance weighted
-0.50
-0.25
0.00
0.25
MR effect size for
'|| id:ebi-a-GCST90027851' on 'outcome'

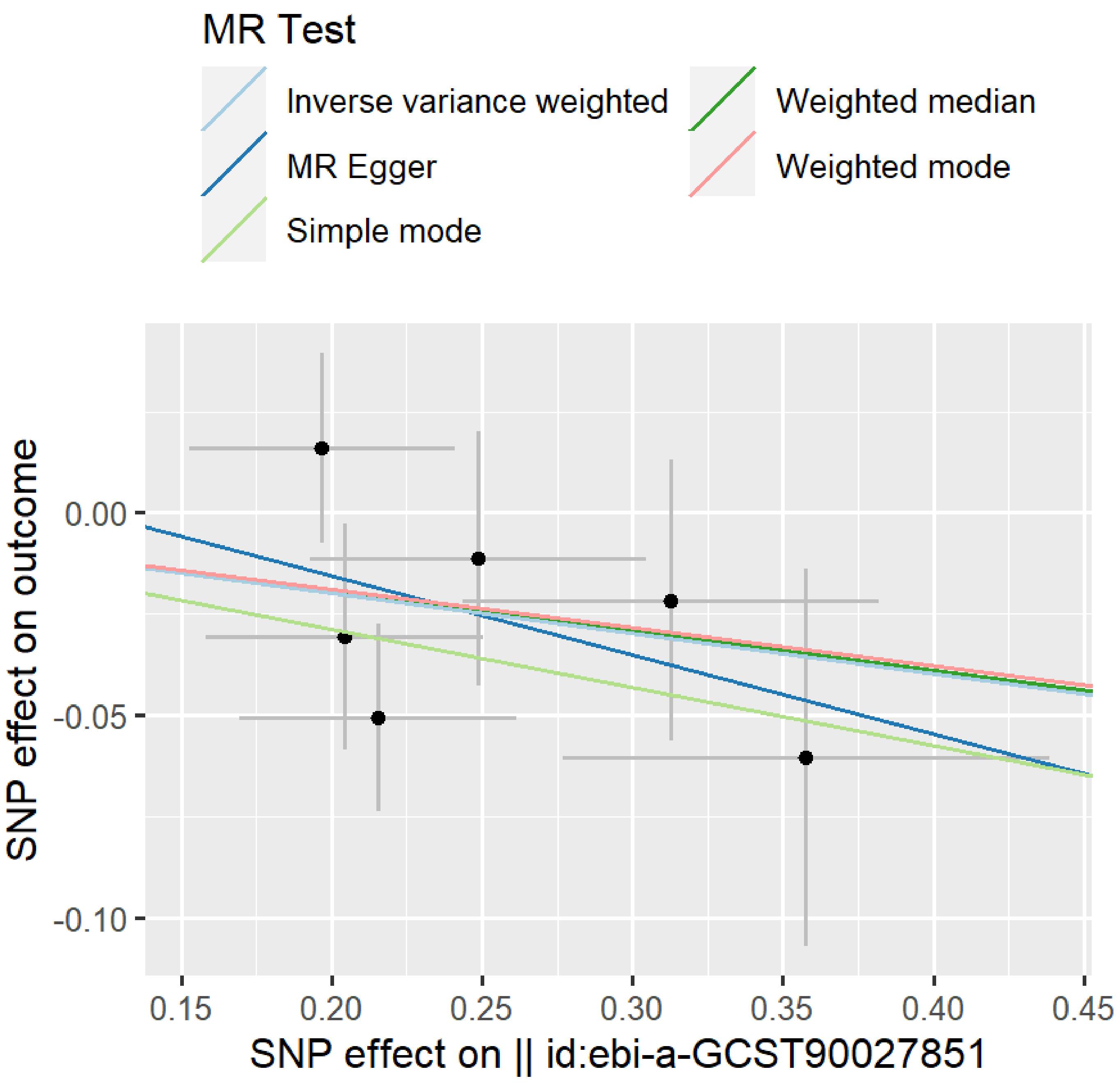
MR Test
Inverse variance weighted
MR Egger
Simple mode
Weighted median
Weighted mode
SNP effect on outcome
0.00
-0.05
-0.10
0.15
0.20
0.25
0.30
0.35
0.40
0.45
SNP effect on || id:ebi-a-GCST90027851

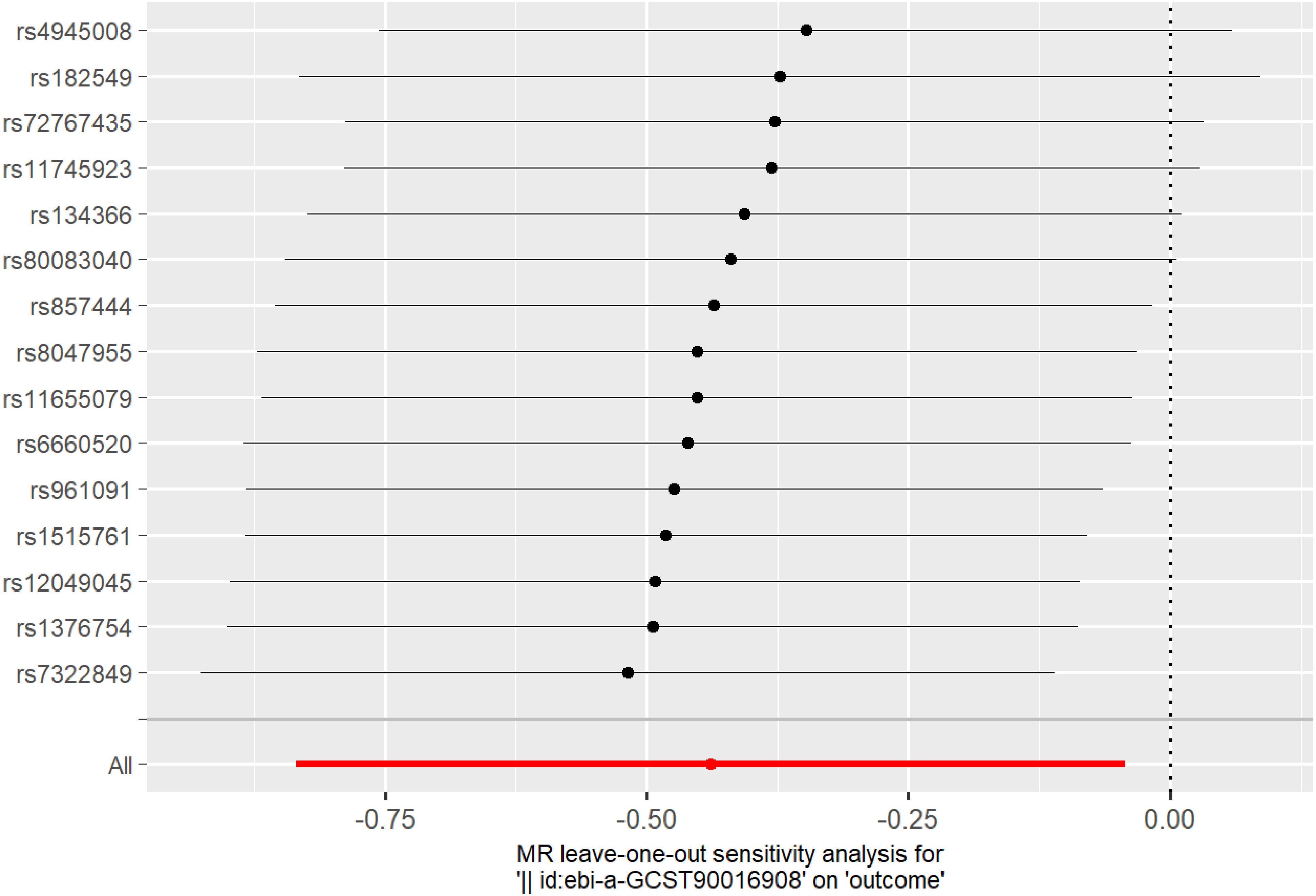
rs4945008
rs182549
rs72767435
rs11745923
rs134366
rs80083040
rs857444
rs8047955
rs11655079
rs6660520
rs961091
rs1515761
rs12049045
rs1376754
rs7322849
All
-0.75
-0.50
-0.25
0.00
MR leave-one-out sensitivity analysis for
'|| id:ebi-a-GCST90016908' on 'outcome'

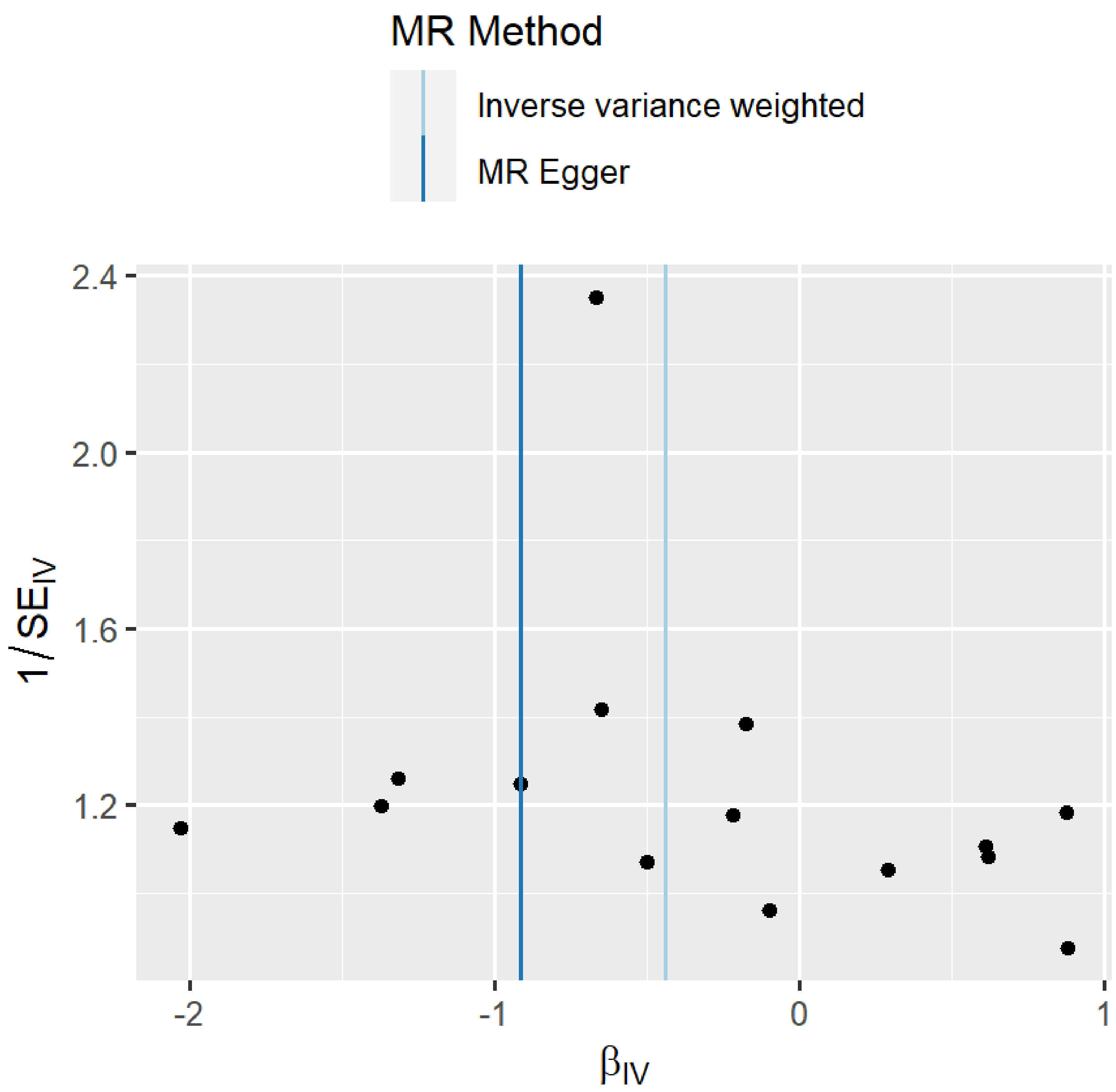
MR Method
Inverse variance weighted
MR Egger
2.4
2.0
1.6
1.2
1/SE_IV
-2
-1
0
1
β_IV

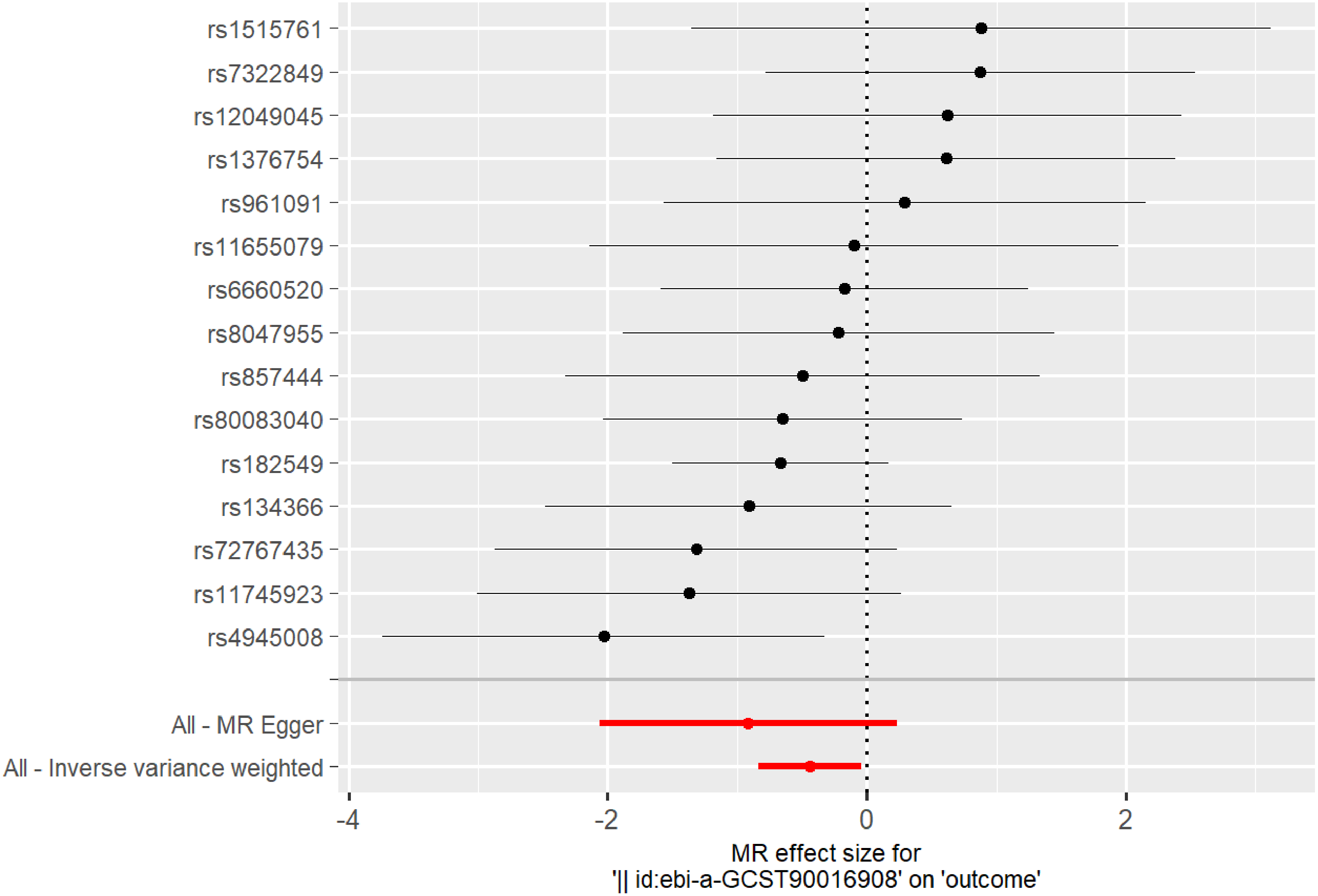
rs1515761
rs7322849
rs12049045
rs1376754
rs961091
rs11655079
rs6660520
rs8047955
rs857444
rs80083040
rs182549
rs134366
rs72767435
rs11745923
rs4945008
All - MR Egger
All - Inverse variance weighted
-4
-2
0
2
MR effect size for
'|| id:ebi-a-GCST90016908' on 'outcome'

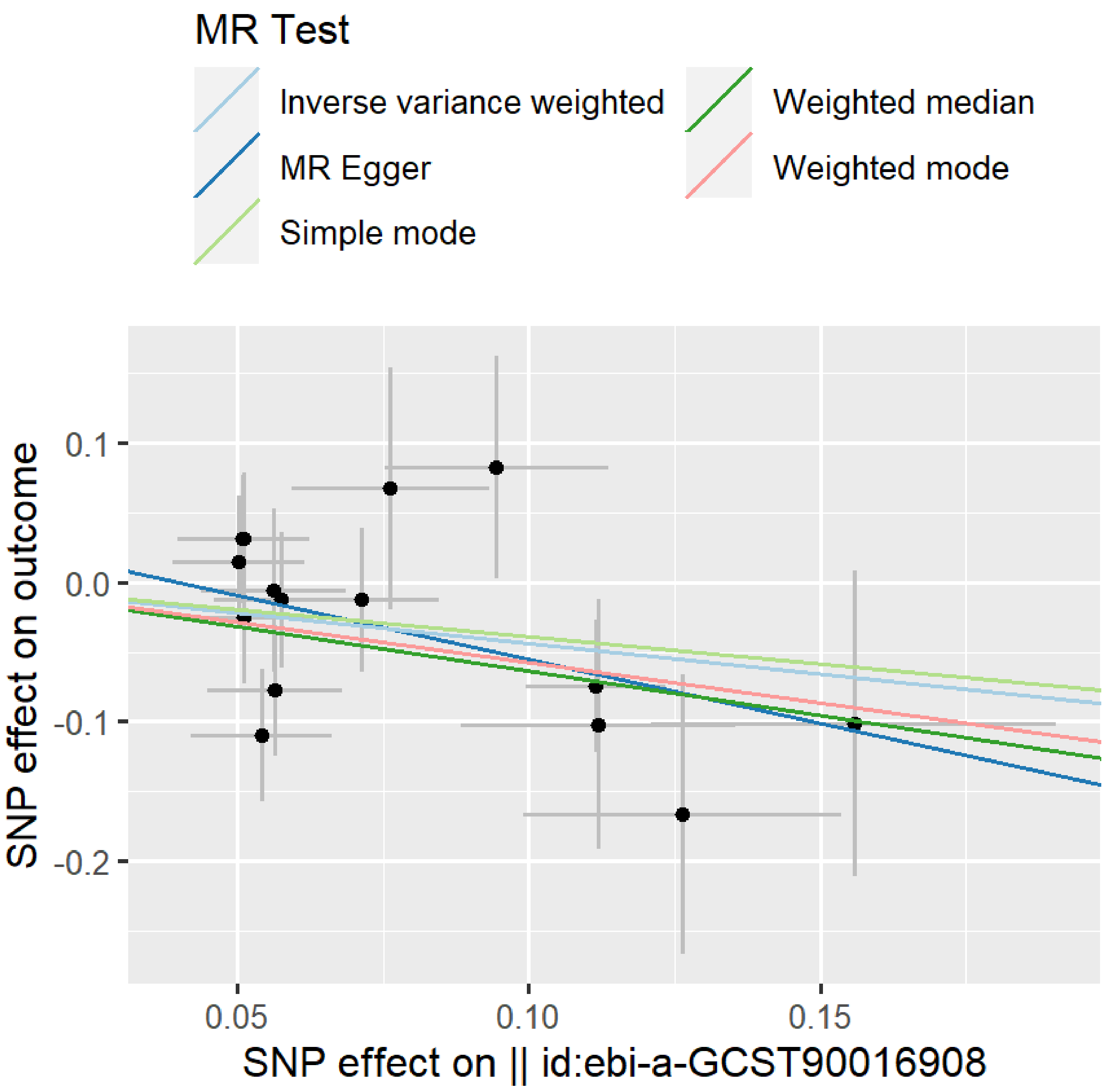

MR Test
Inverse variance weighted
MR Egger
Simple mode
Weighted median
Weighted mode
0.1
0.0
-0.1
-0.2
SNP effect on outcome
0.05
0.10
0.15
SNP effect on || id:ebi-a-GCST90016908

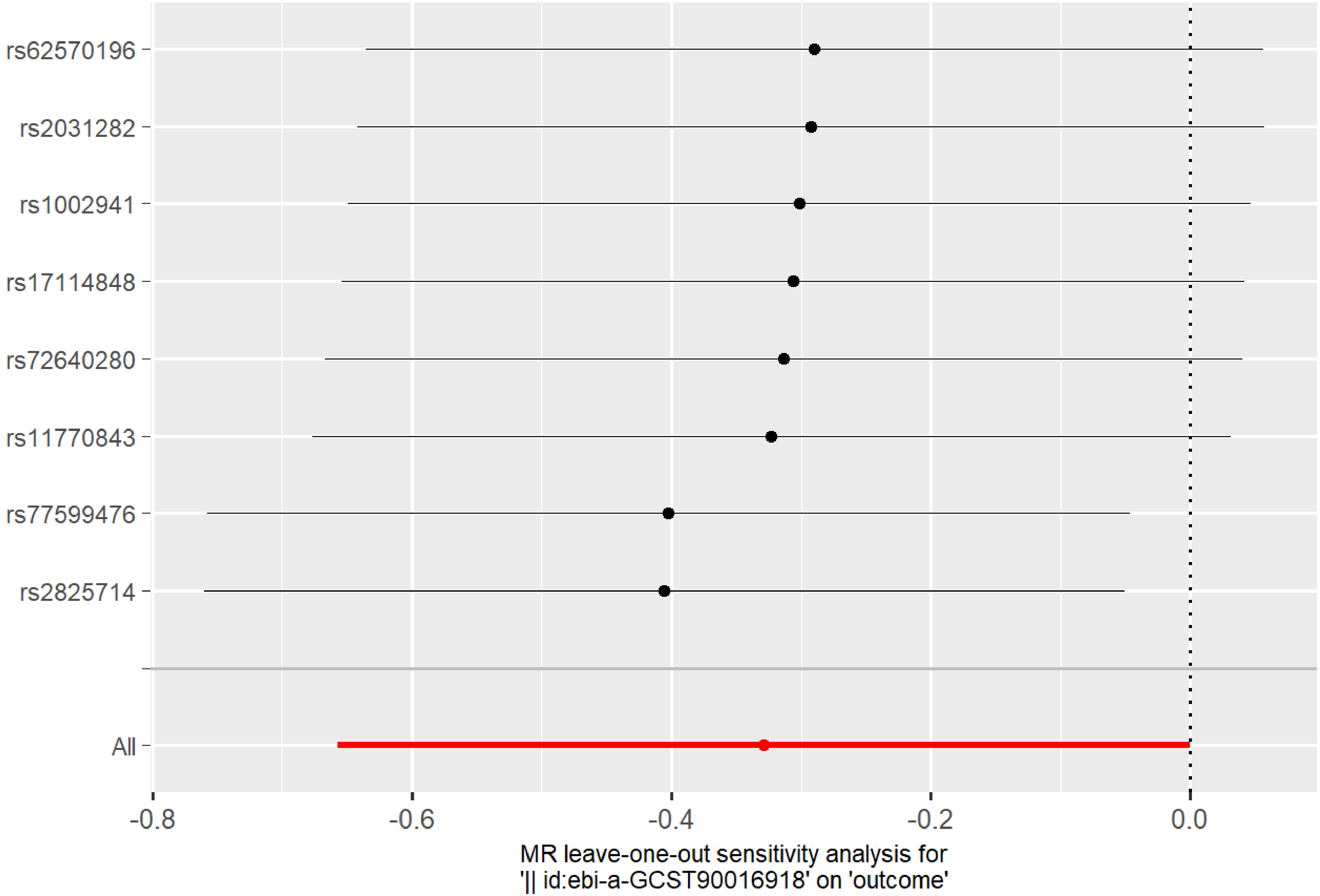
rs62570196
rs2031282
rs1002941
rs17114848
rs72640280
rs11770843
rs77599476
rs2825714
All
-0.8
-0.6
-0.4
-0.2
0.0
MR leave-one-out sensitivity analysis for
'|| id:ebi-a-GCST90016918' on 'outcome'

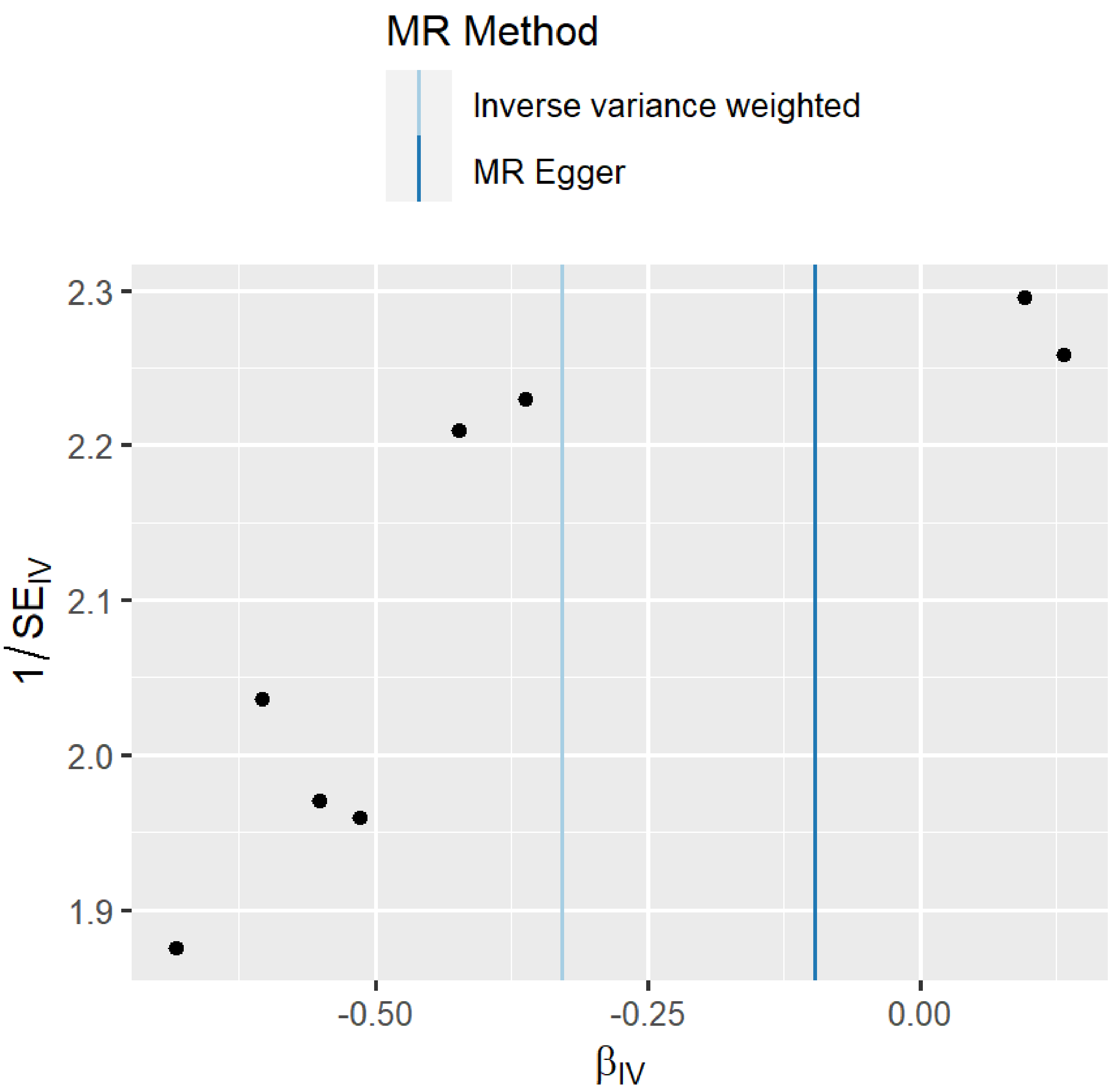

MR Method
Inverse variance weighted
MR Egger
2.3
2.2
2.1
2.0
1.9
1/SE_IV
-0.50
-0.25
0.00
β_IV

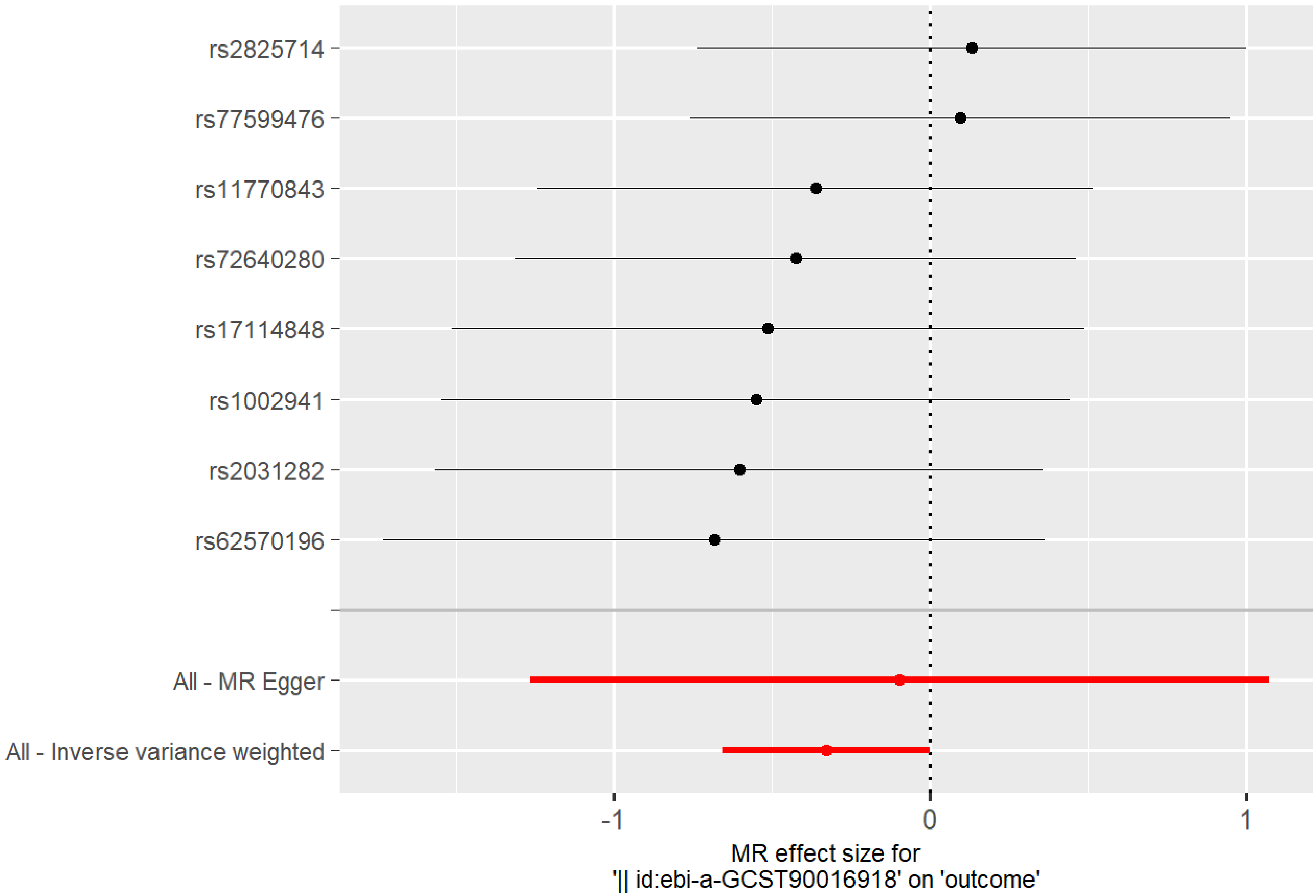
rs2825714
rs77599476
rs11770843
rs72640280
rs17114848
rs1002941
rs2031282
rs62570196
All - MR Egger
All - Inverse variance weighted
-1
0
1
MR effect size for
'|| id:ebi-a-GCST90016918' on 'outcome'

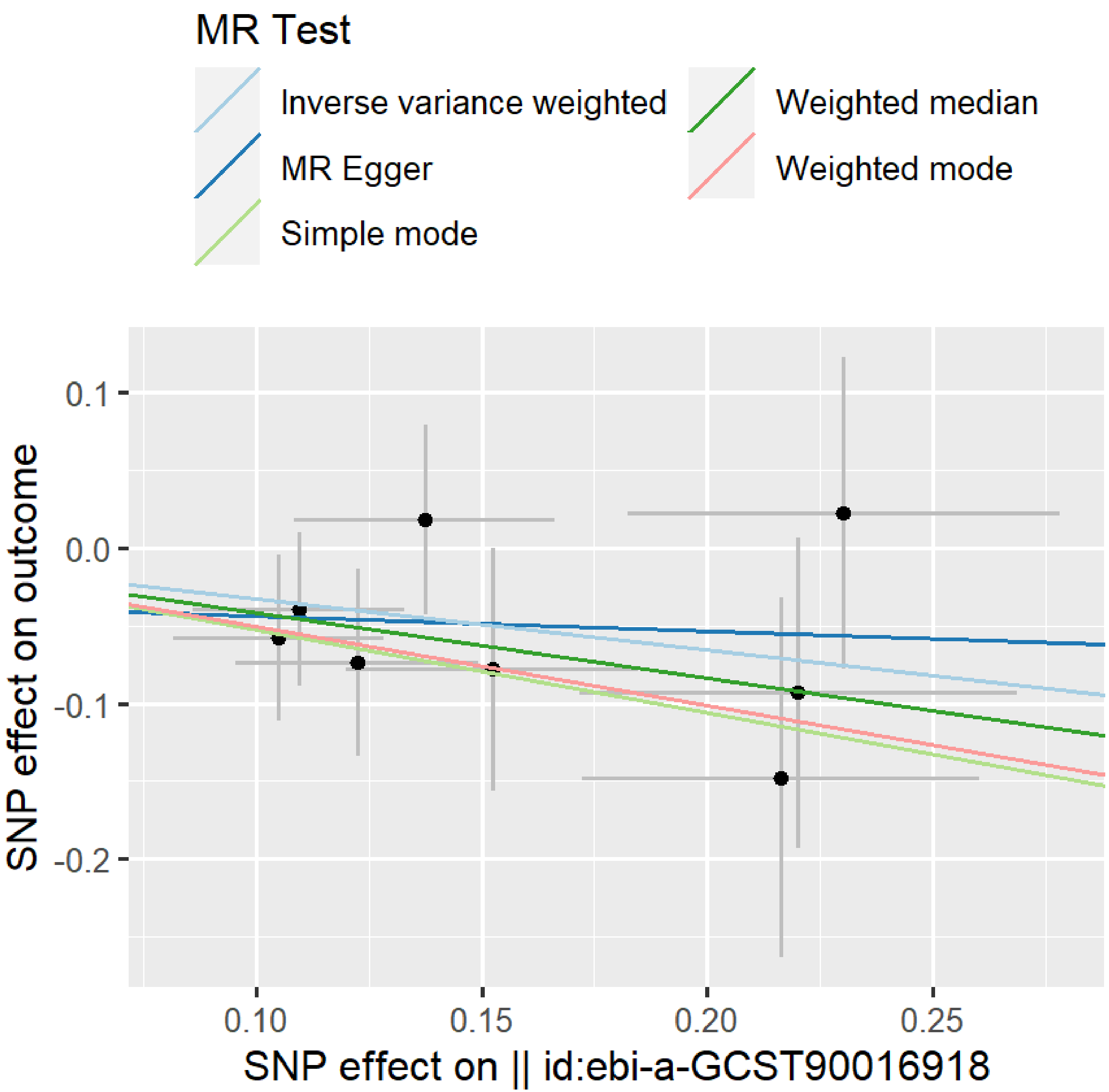

MR Test
Inverse variance weighted
MR Egger
Simple mode
Weighted median
Weighted mode
0.1
0.0
-0.1
-0.2
SNP effect on outcome
0.10
0.15
0.20
0.25
SNP effect on || id:ebi-a-GCST90016918

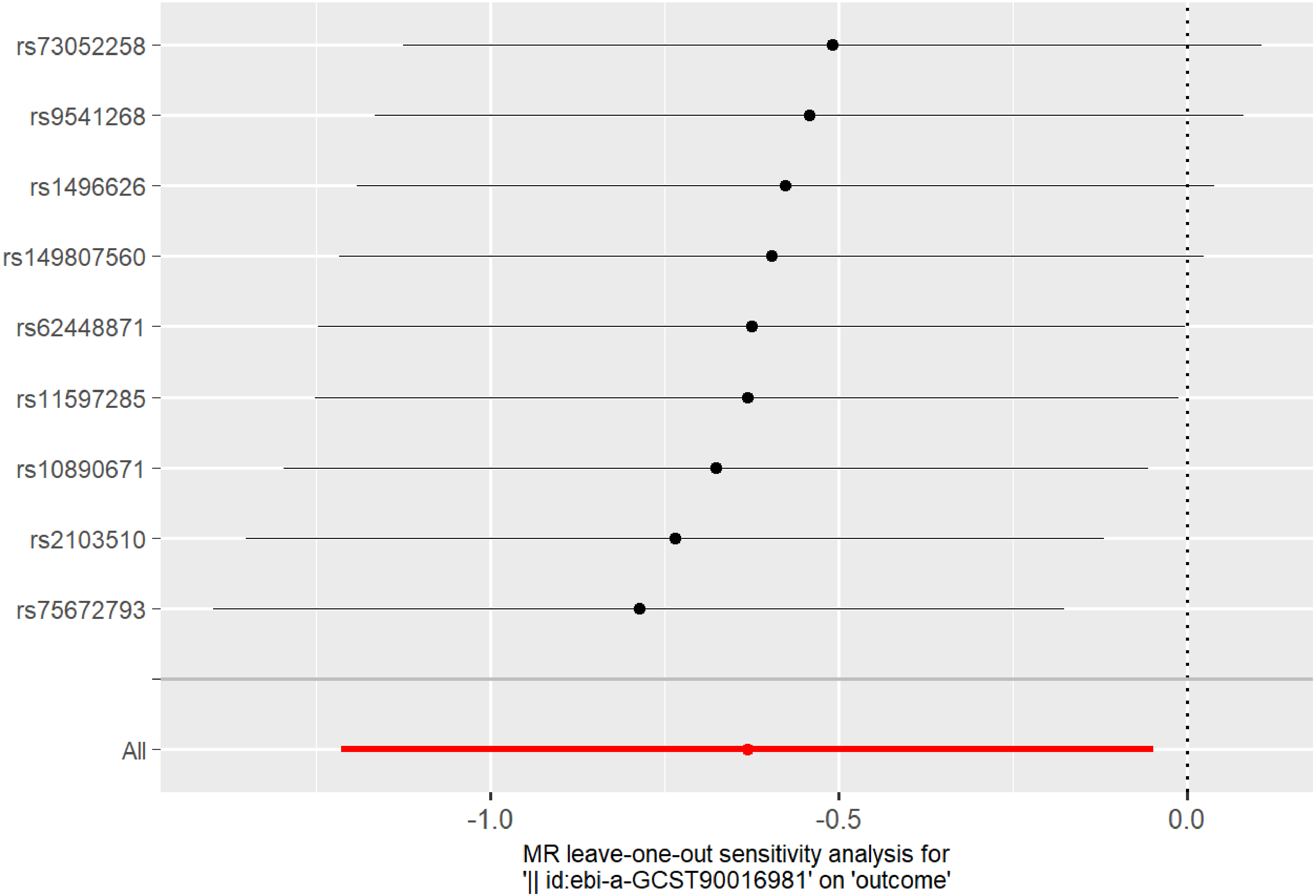
rs73052258
rs9541268
rs1496626
rs149807560
rs62448871
rs11597285
rs10890671
rs2103510
rs75672793
All
-1.0
-0.5
0.0
MR leave-one-out sensitivity analysis for
'|| id:ebi-a-GCST90016981' on 'outcome'

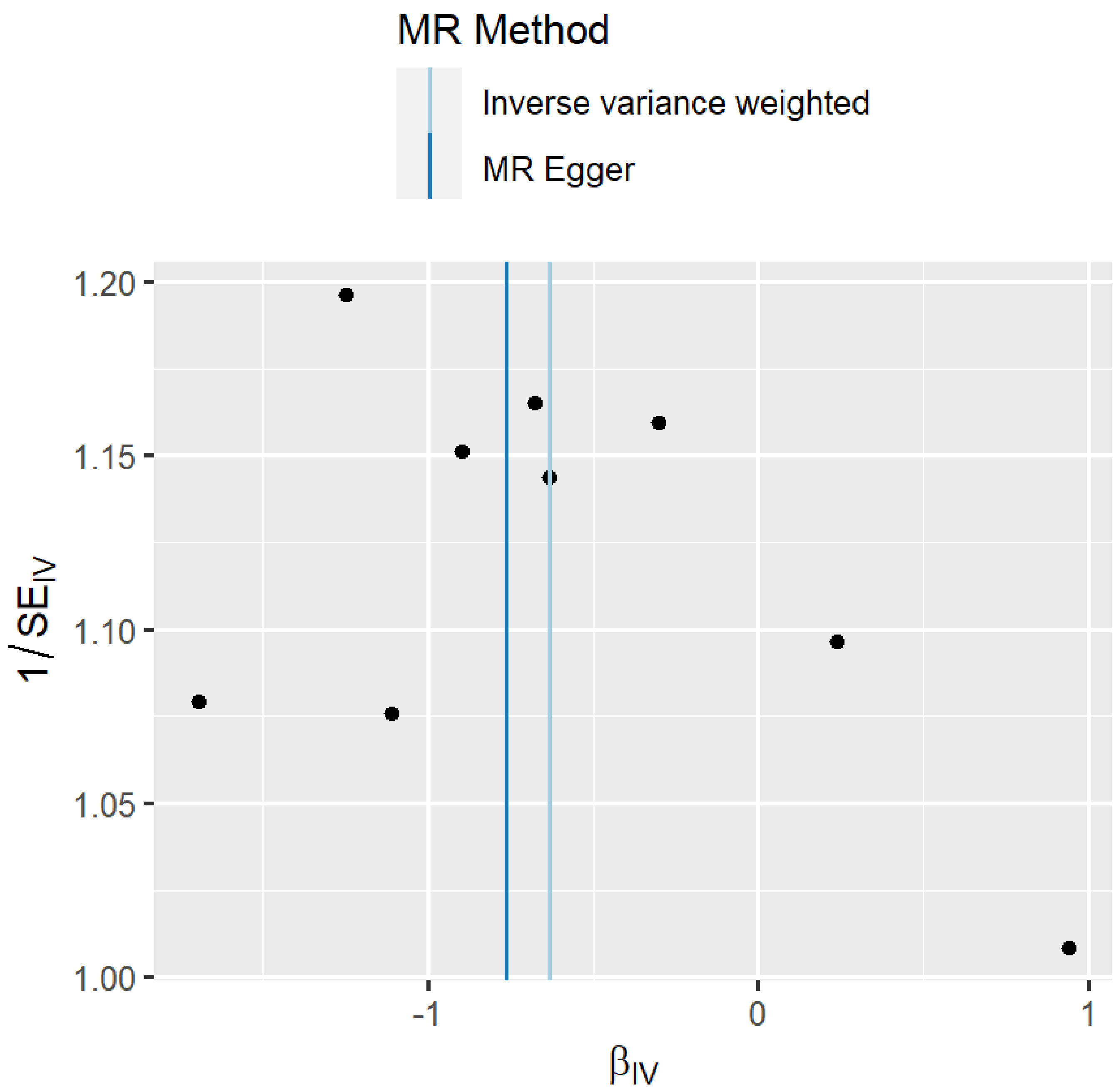
MR Method
Inverse variance weighted
MR Egger
1.20
1.15
1.10
1.05
1.00
-1
0
1
1/SE_IV
β_IV

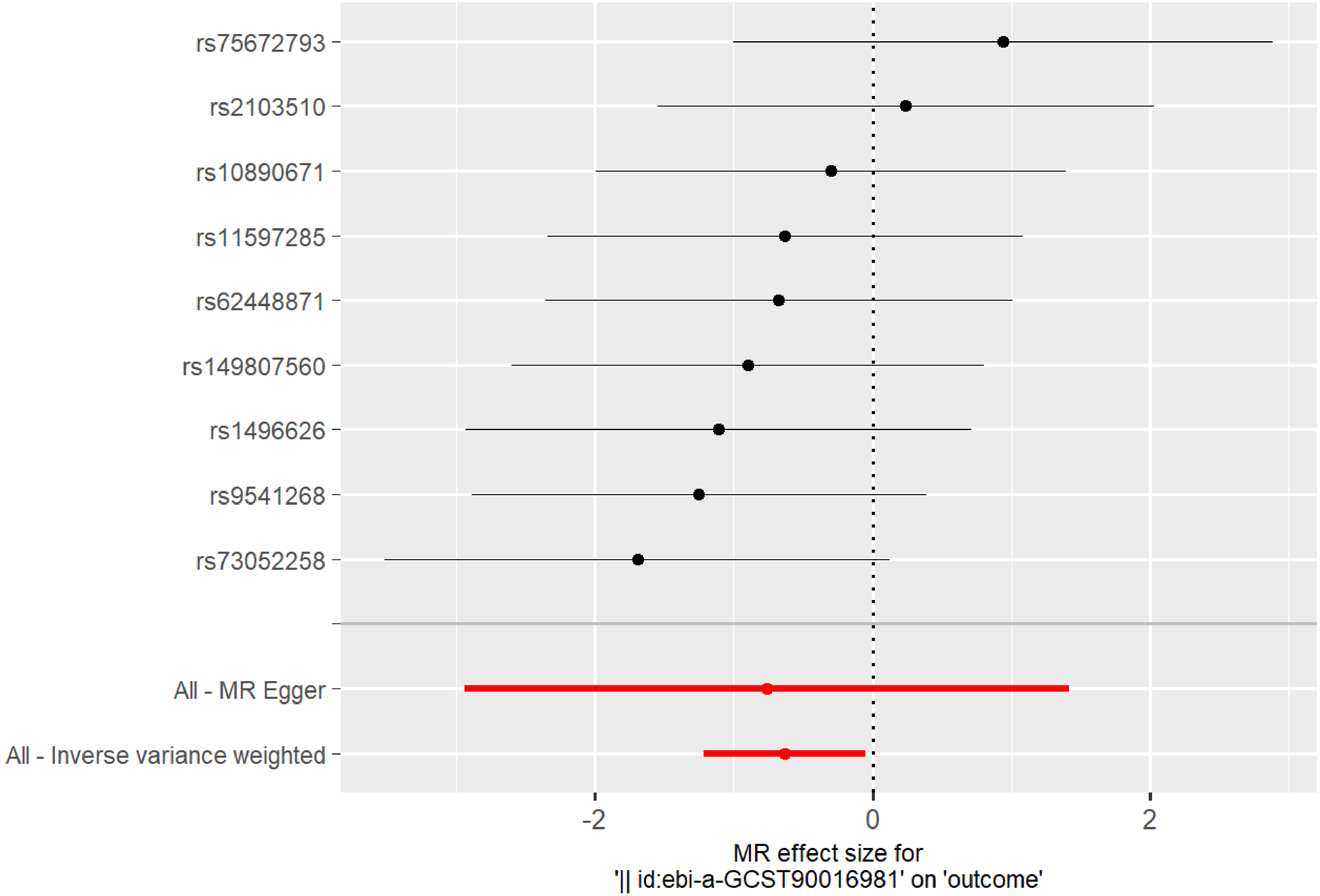

rs75672793
rs2103510
rs10890671
rs11597285
rs62448871
rs149807560
rs1496626
rs9541268
rs73052258
All - MR Egger
All - Inverse variance weighted
-2
0
2
MR effect size for
'|| id:ebi-a-GCST90016981' on 'outcome'

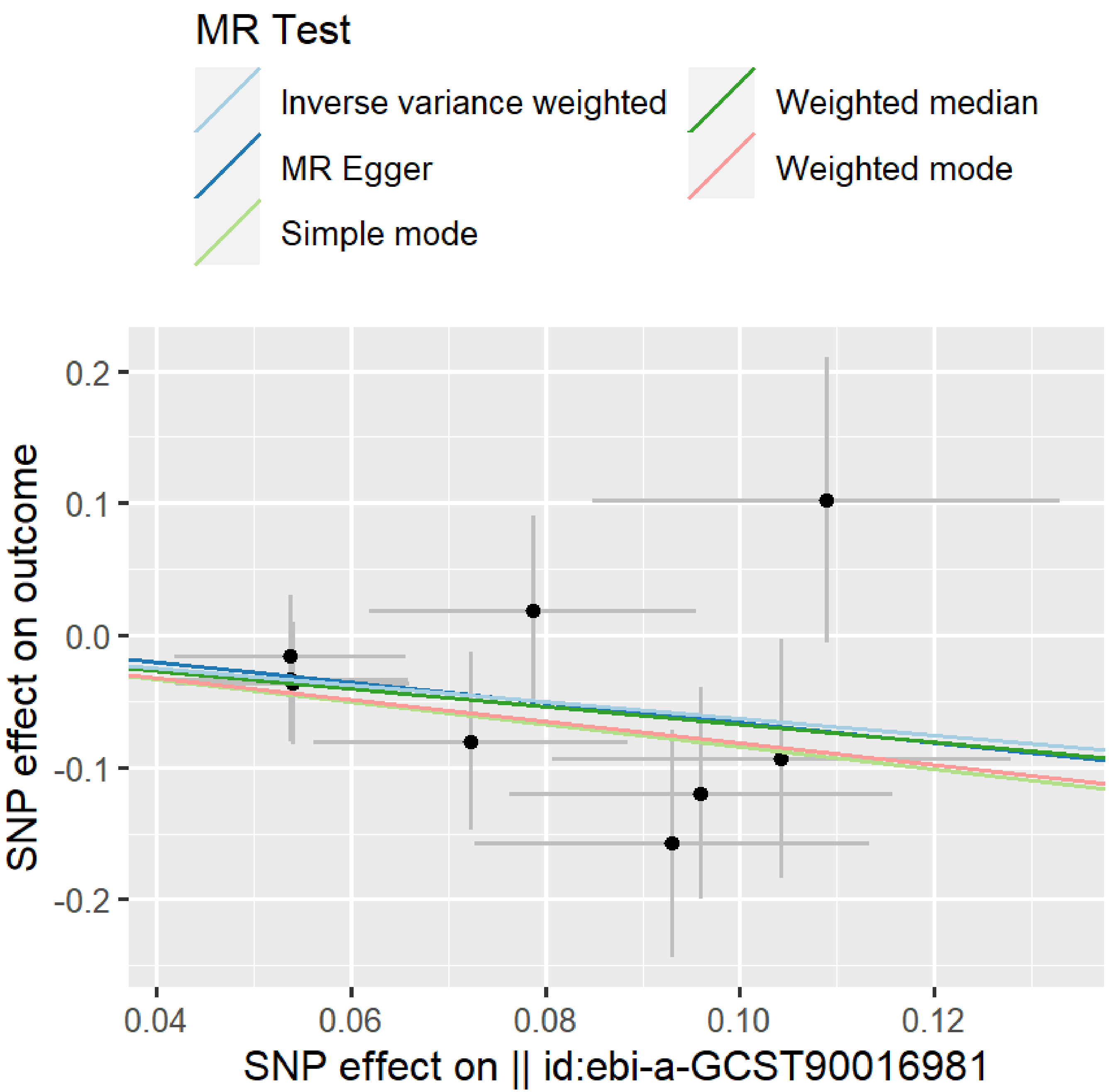

MR Test
Inverse variance weighted
MR Egger
Simple mode
Weighted median
Weighted mode
0.2
0.1
0.0
-0.1
-0.2
SNP effect on outcome
0.04
0.06
0.08
0.10
0.12
SNP effect on || id:ebi-a-GCST90016981

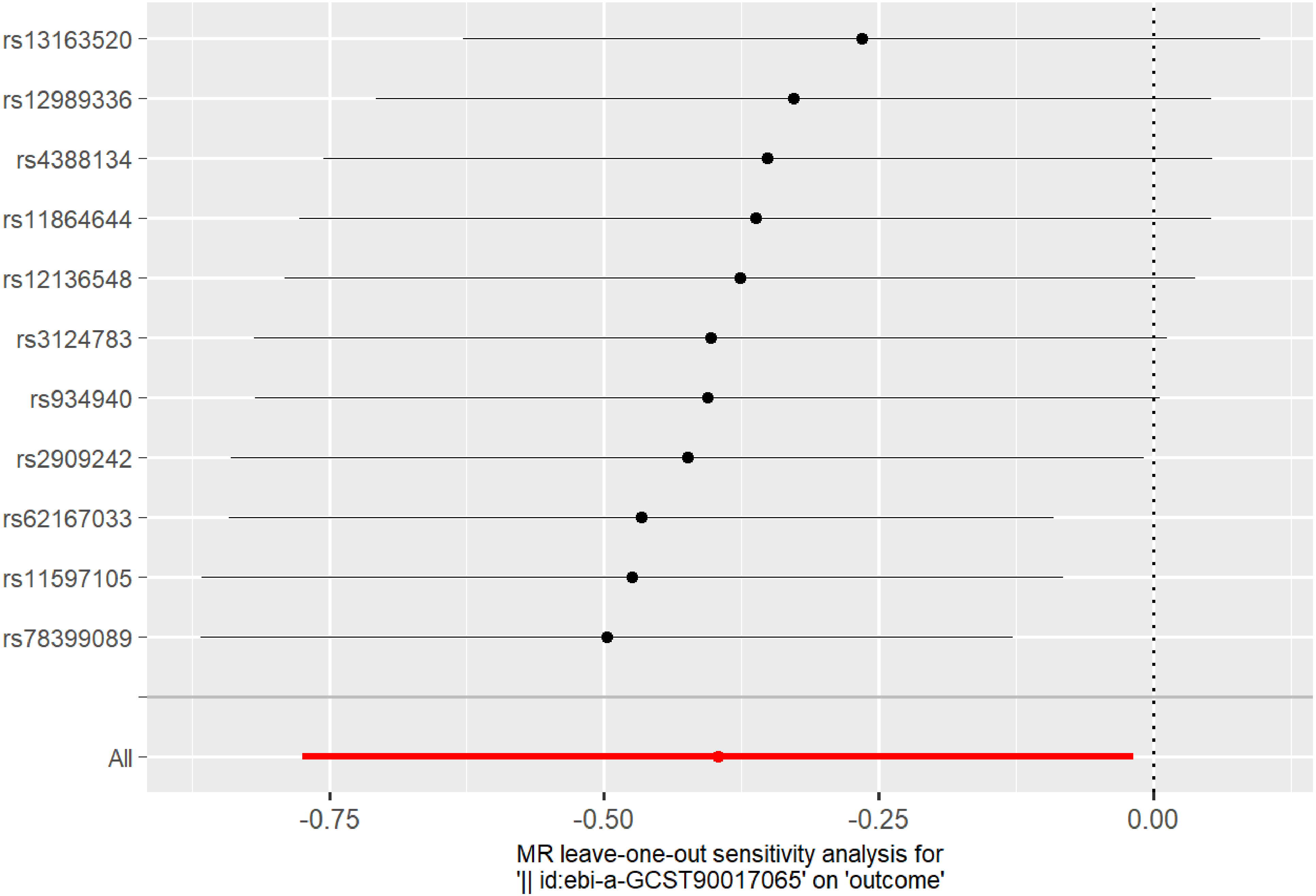
rs13163520
rs12989336
rs4388134
rs11864644
rs12136548
rs3124783
rs934940
rs2909242
rs62167033
rs11597105
rs78399089
All
-0.75
-0.50
-0.25
0.00
MR leave-one-out sensitivity analysis for
'|| id:ebi-a-GCST90017065' on 'outcome'

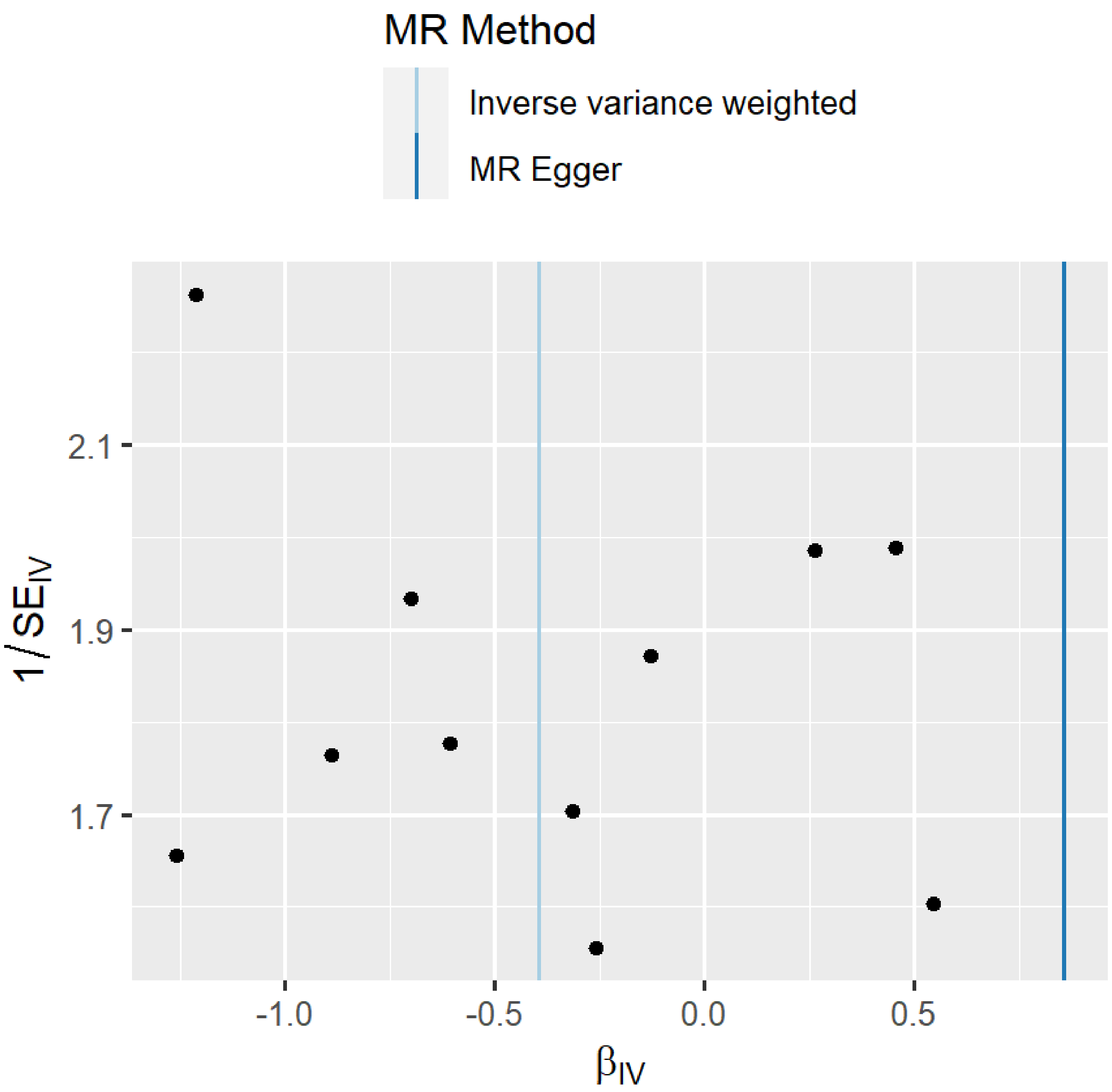
MR Method
Inverse variance weighted
MR Egger
2.1
1.9
1.7
1/SE_IV
-1.0
-0.5
0.0
0.5
β_IV

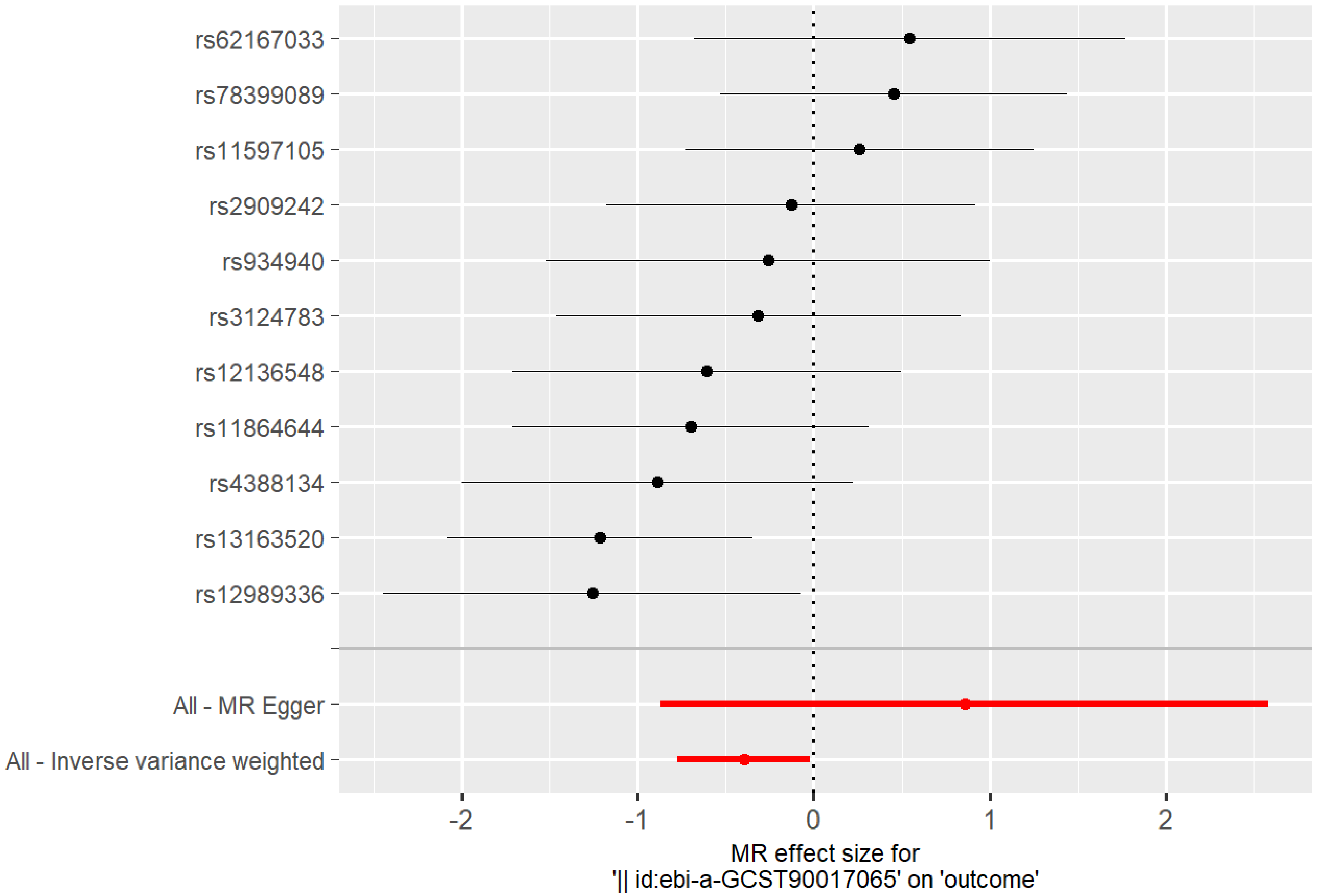
rs62167033
rs78399089
rs11597105
rs2909242
rs934940
rs3124783
rs12136548
rs11864644
rs4388134
rs13163520
rs12989336
All - MR Egger
All - Inverse variance weighted
-2
-1
0
1
2
MR effect size for
'|| id:ebi-a-GCST90017065' on 'outcome'

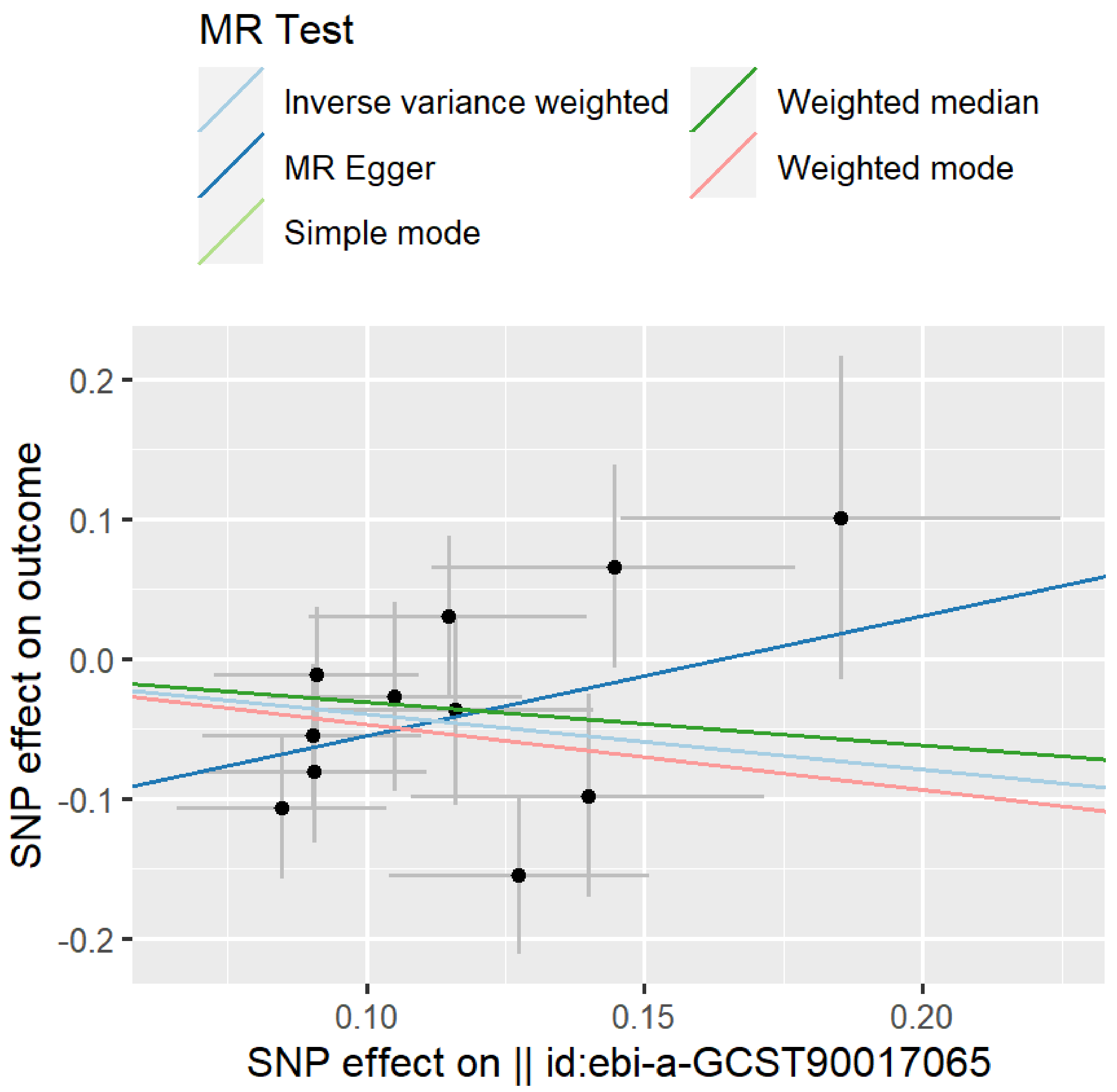
MR Test
Inverse variance weighted
MR Egger
Simple mode
Weighted median
Weighted mode
0.2
0.1
0.0
-0.1
-0.2
SNP effect on outcome
0.10
0.15
0.20
SNP effect on || id:ebi-a-GCST90017065

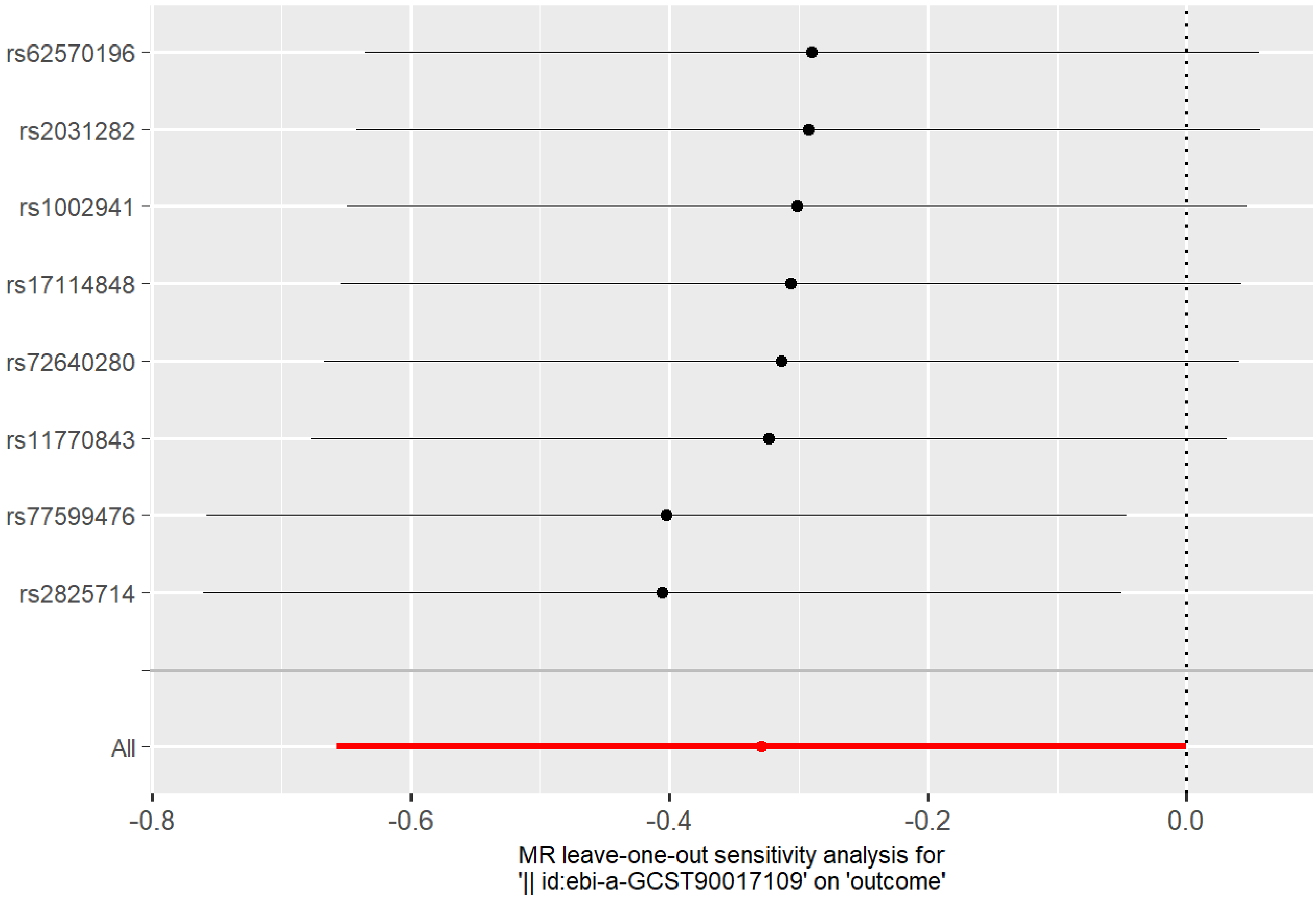
rs62570196
rs2031282
rs1002941
rs17114848
rs72640280
rs11770843
rs77599476
rs2825714
All
-0.8
-0.6
-0.4
-0.2
0.0
MR leave-one-out sensitivity analysis for
'|| id:ebi-a-GCST90017109' on 'outcome'

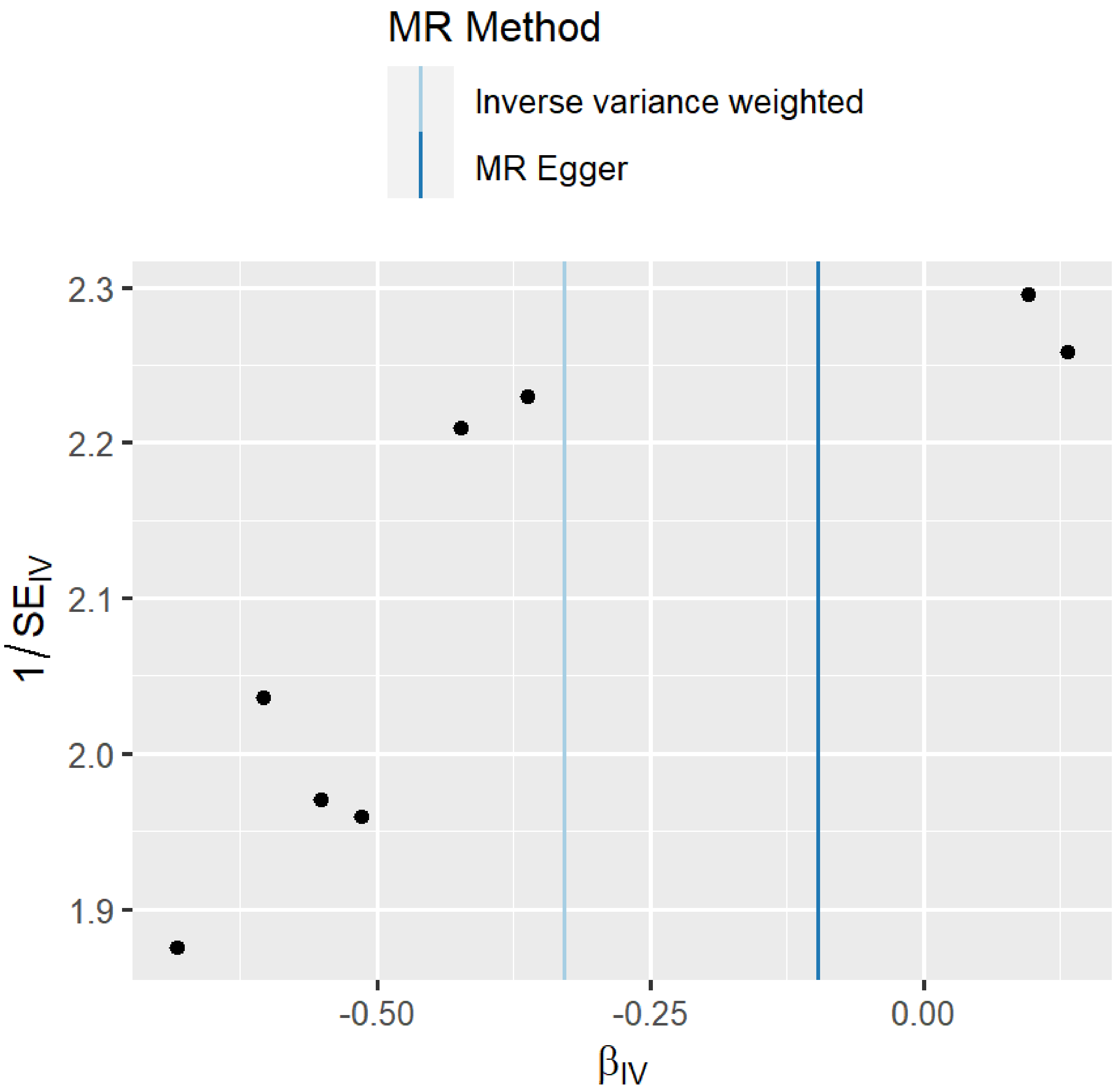
MR Method
Inverse variance weighted
MR Egger
2.3
2.2
2.1
2.0
1.9
1/SE_IV
-0.50
-0.25
0.00
β_IV

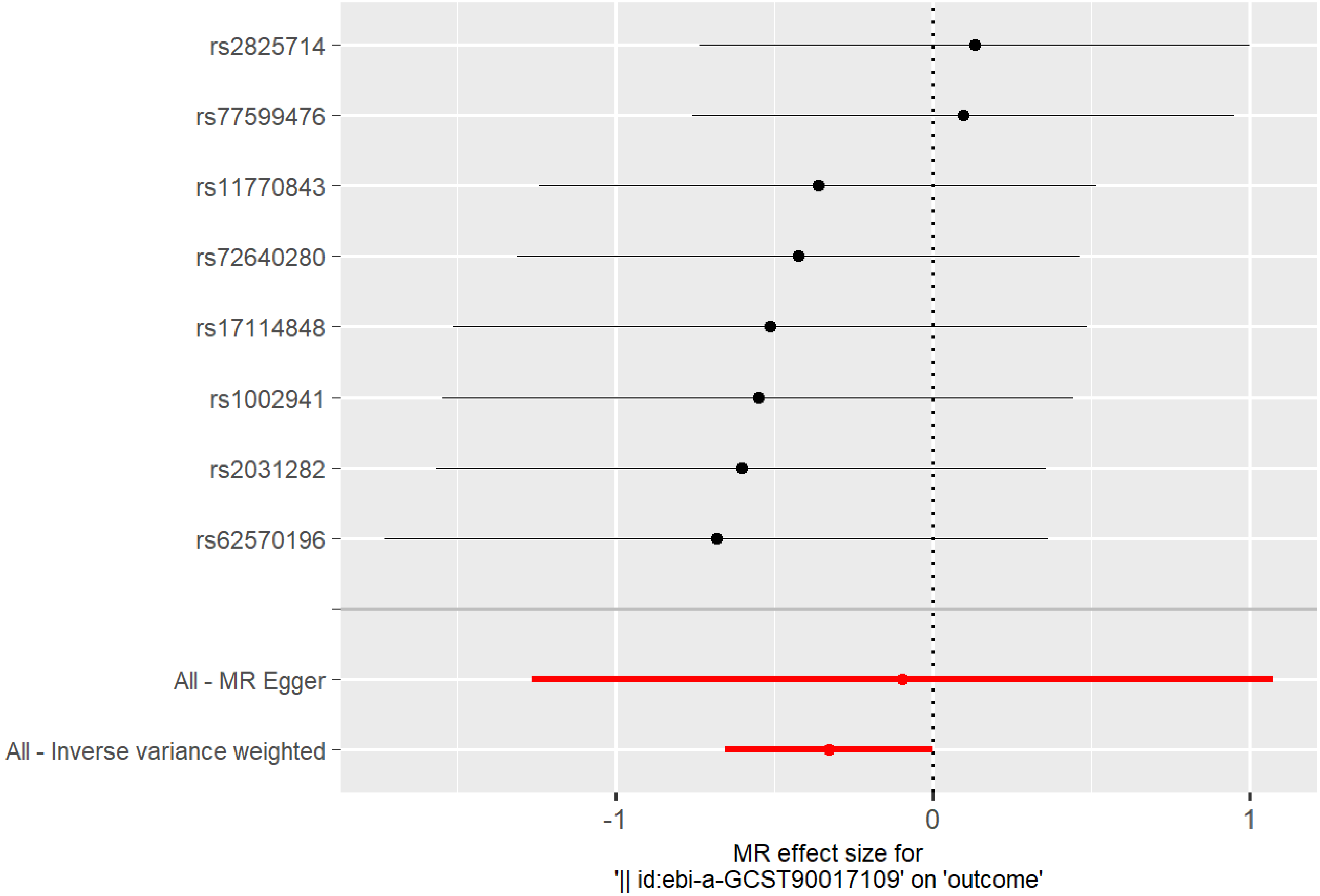
rs2825714
rs77599476
rs11770843
rs72640280
rs17114848
rs1002941
rs2031282
rs62570196
All - MR Egger
All - Inverse variance weighted
-1
0
1
MR effect size for
'|| id:ebi-a-GCST90017109' on 'outcome'

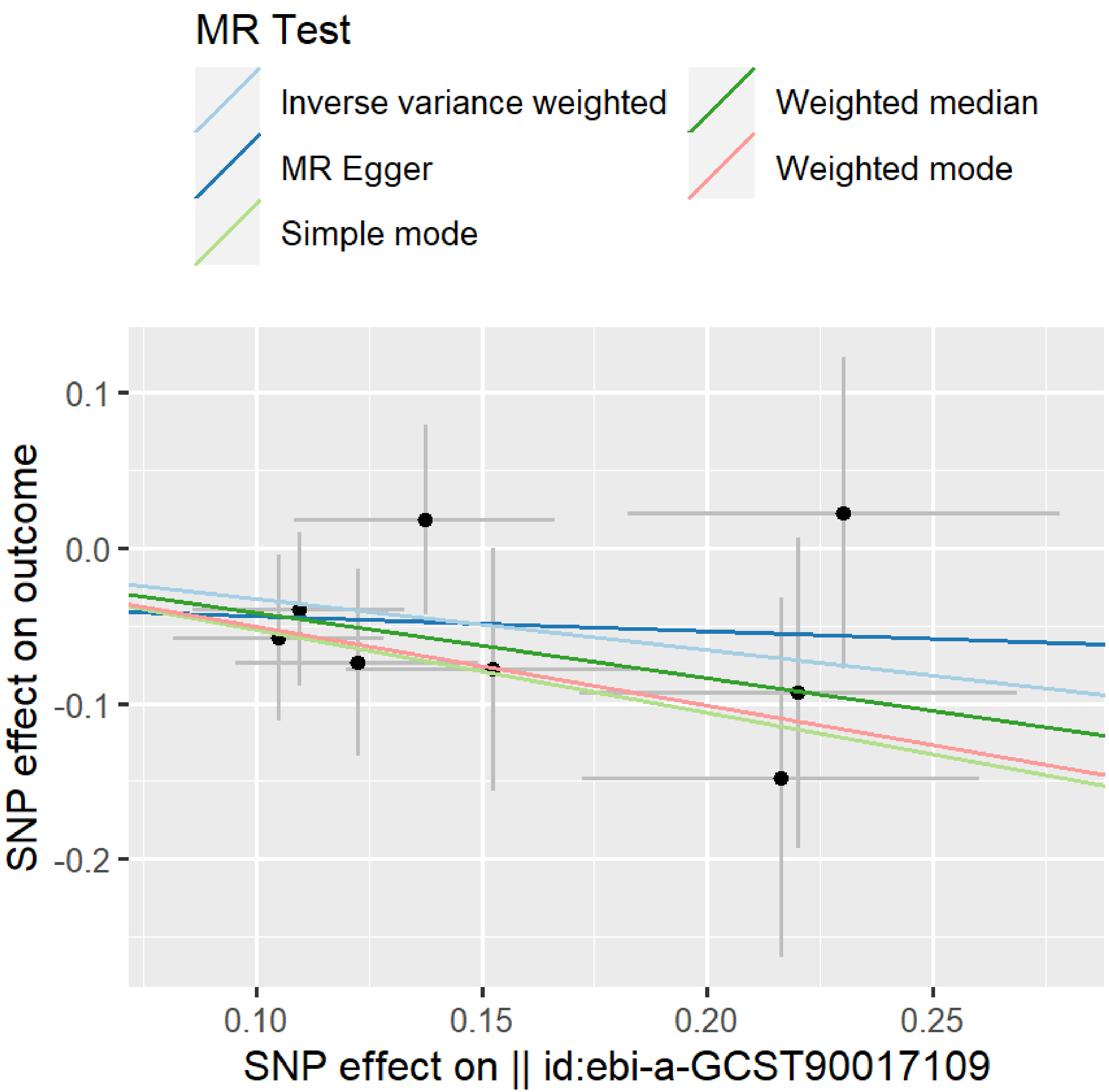

MR Test
Inverse variance weighted
MR Egger
Simple mode
Weighted median
Weighted mode
0.1
0.0
-0.1
-0.2
SNP effect on outcome
0.10
0.15
0.20
0.25
SNP effect on || id:ebi-a-GCST90017109

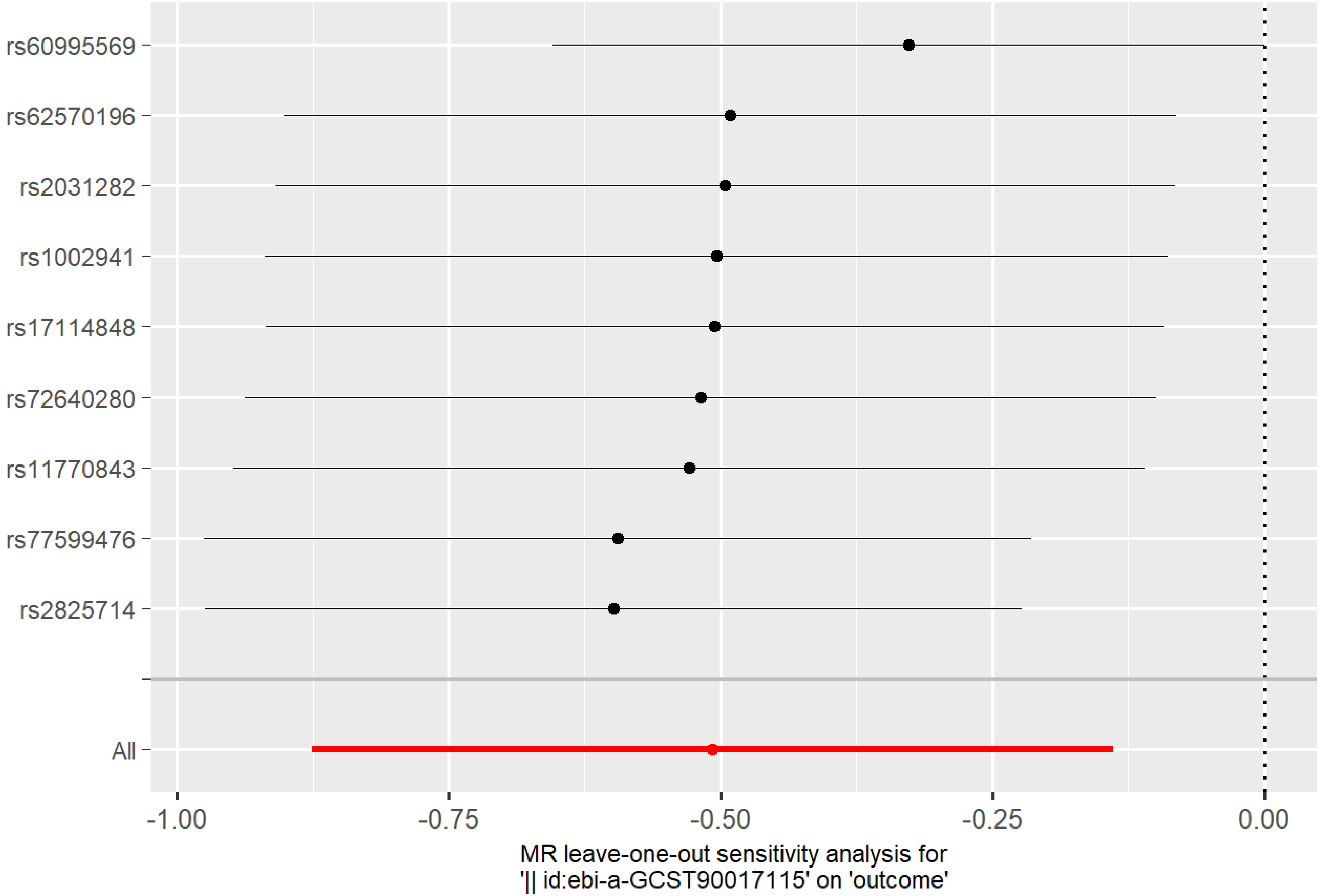
rs60995569
rs62570196
rs2031282
rs1002941
rs17114848
rs72640280
rs11770843
rs77599476
rs2825714
All
-1.00
-0.75
-0.50
-0.25
0.00
MR leave-one-out sensitivity analysis for
'|| id:ebi-a-GCST90017115' on 'outcome'

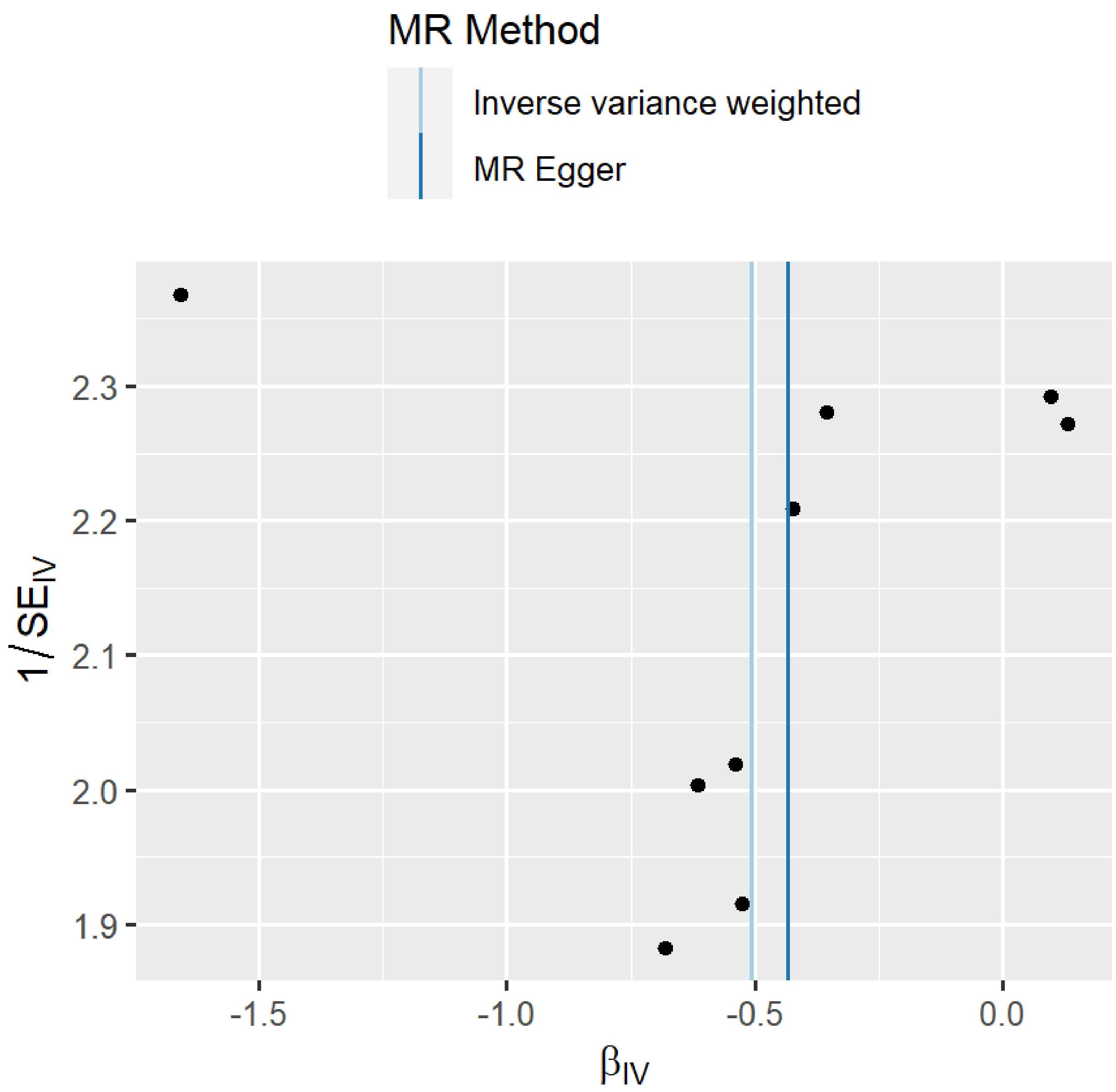
MR Method
Inverse variance weighted
MR Egger
2.3
2.2
2.1
2.0
1.9
1/SE_IV
-1.5
-1.0
-0.5
0.0
β_IV

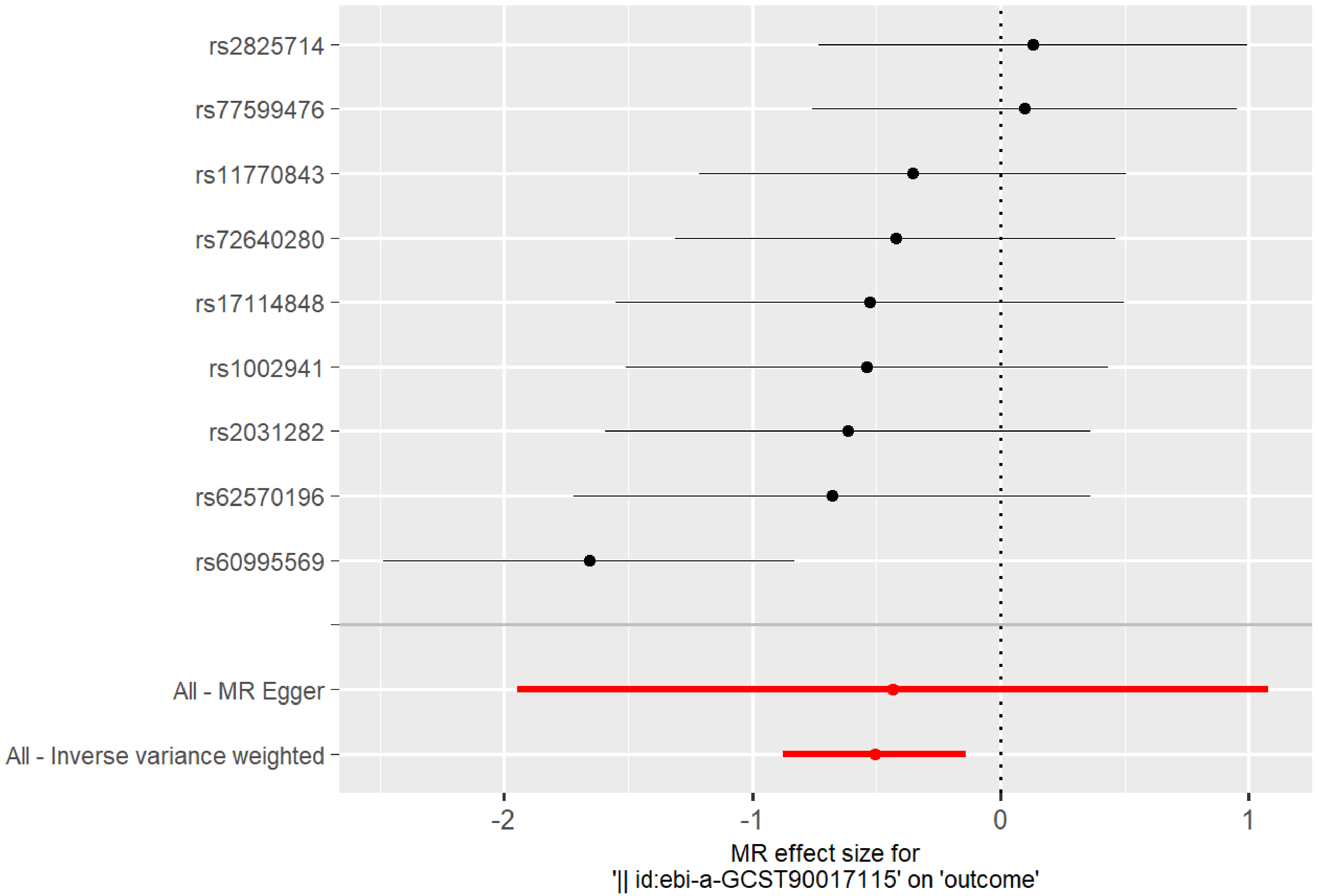
rs2825714
rs77599476
rs11770843
rs72640280
rs17114848
rs1002941
rs2031282
rs62570196
rs60995569
All - MR Egger
All - Inverse variance weighted
-2
-1
0
1
MR effect size for
'|| id:ebi-a-GCST90017115' on 'outcome'

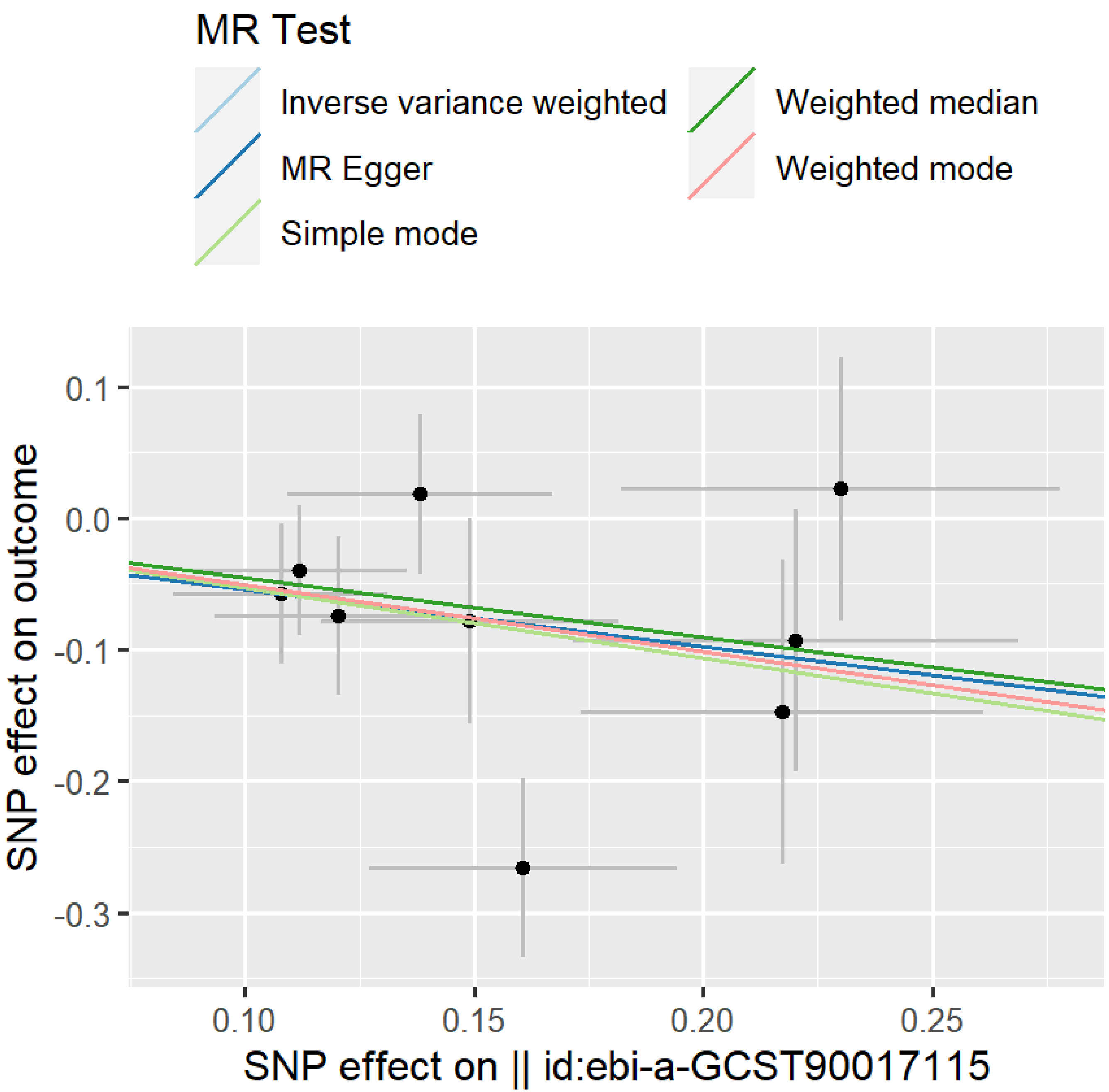
MR Test
Inverse variance weighted
MR Egger
Simple mode
Weighted median
Weighted mode
0.1
0.0
-0.1
-0.2
-0.3
SNP effect on outcome
0.10
0.15
0.20
0.25
SNP effect on || id:ebi-a-GCST90017115

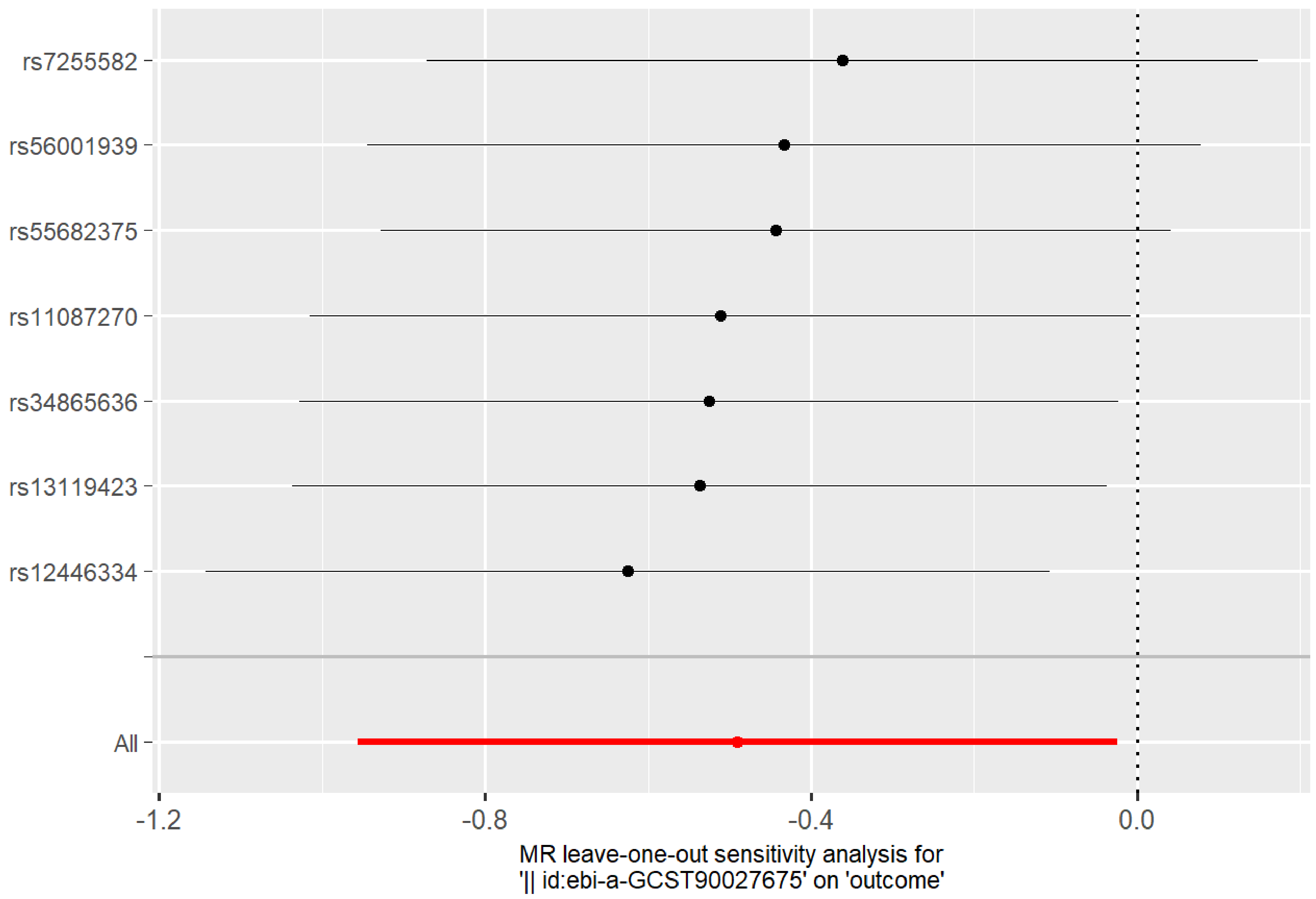
rs7255582
rs56001939
rs55682375
rs11087270
rs34865636
rs13119423
rs12446334
All
-1.2
-0.8
-0.4
0.0
MR leave-one-out sensitivity analysis for
'|| id:ebi-a-GCST90027675' on 'outcome'

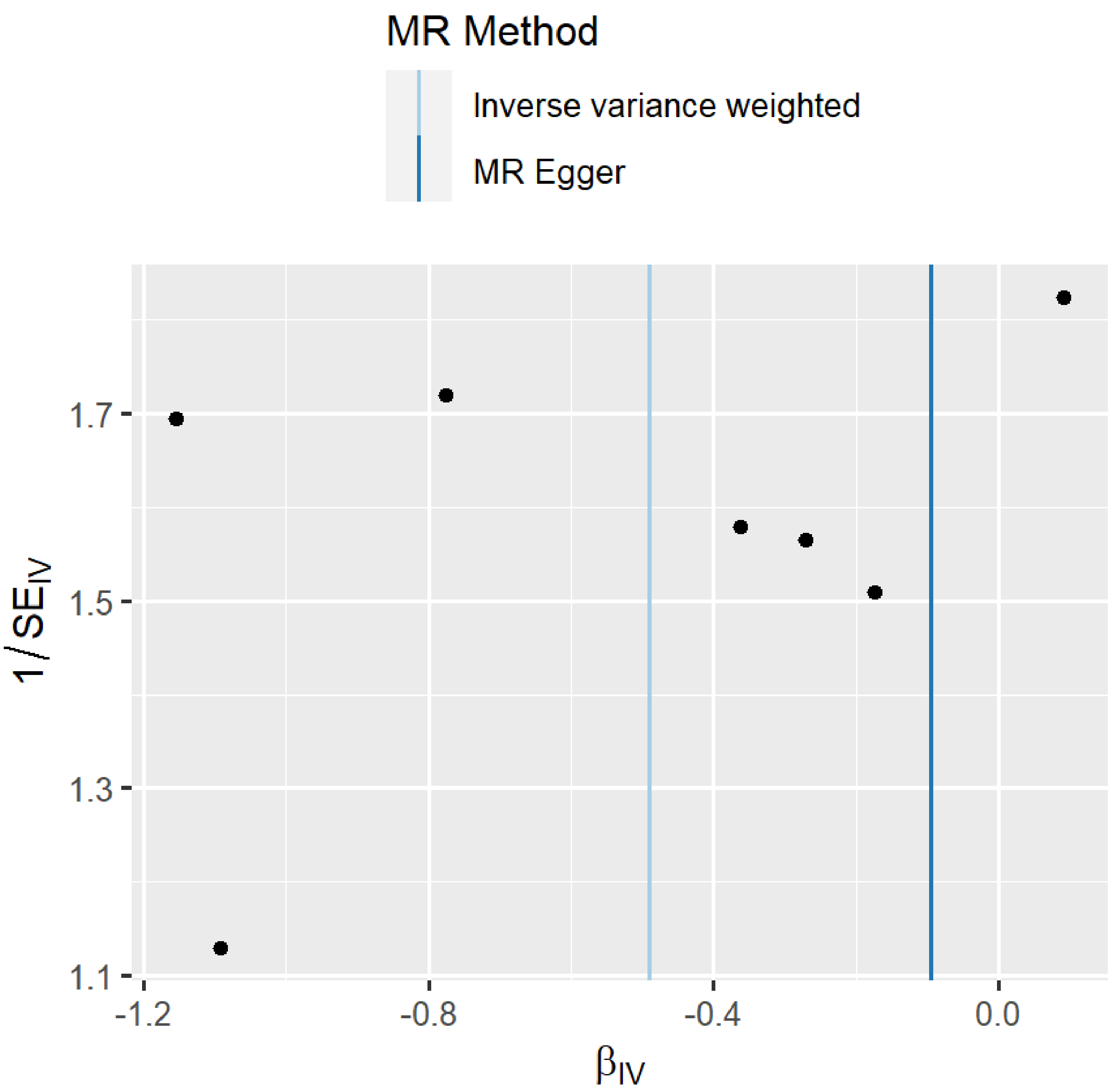
MR Method
Inverse variance weighted
MR Egger
1.7
1.5
1.3
1.1
-1.2
-0.8
-0.4
0.0
β_{IV}
$1/SE_{IV}$

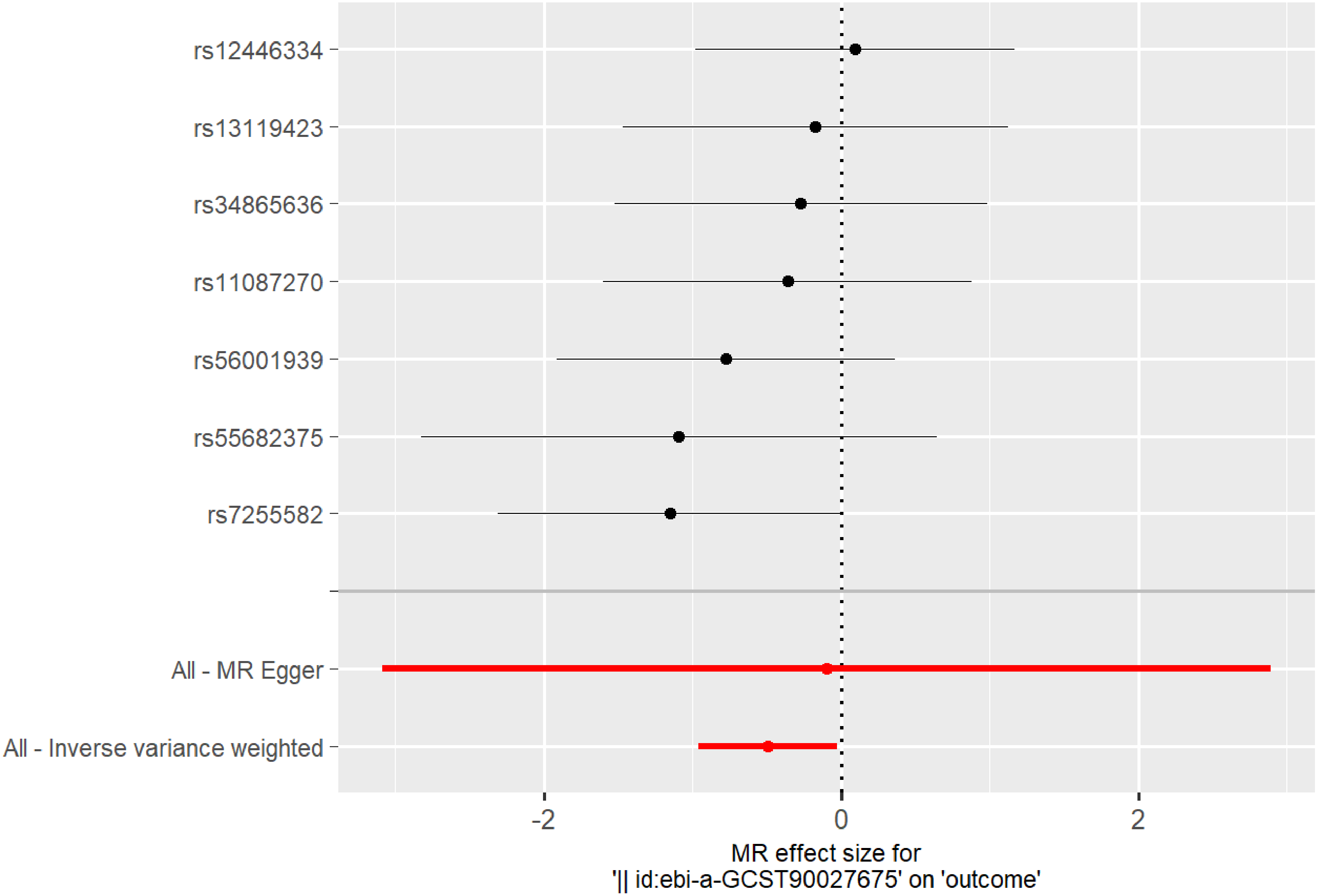
rs12446334
rs13119423
rs34865636
rs11087270
rs56001939
rs55682375
rs7255582
All - MR Egger
All - Inverse variance weighted
-2
0
2
MR effect size for
'|| id:ebi-a-GCST90027675' on 'outcome'

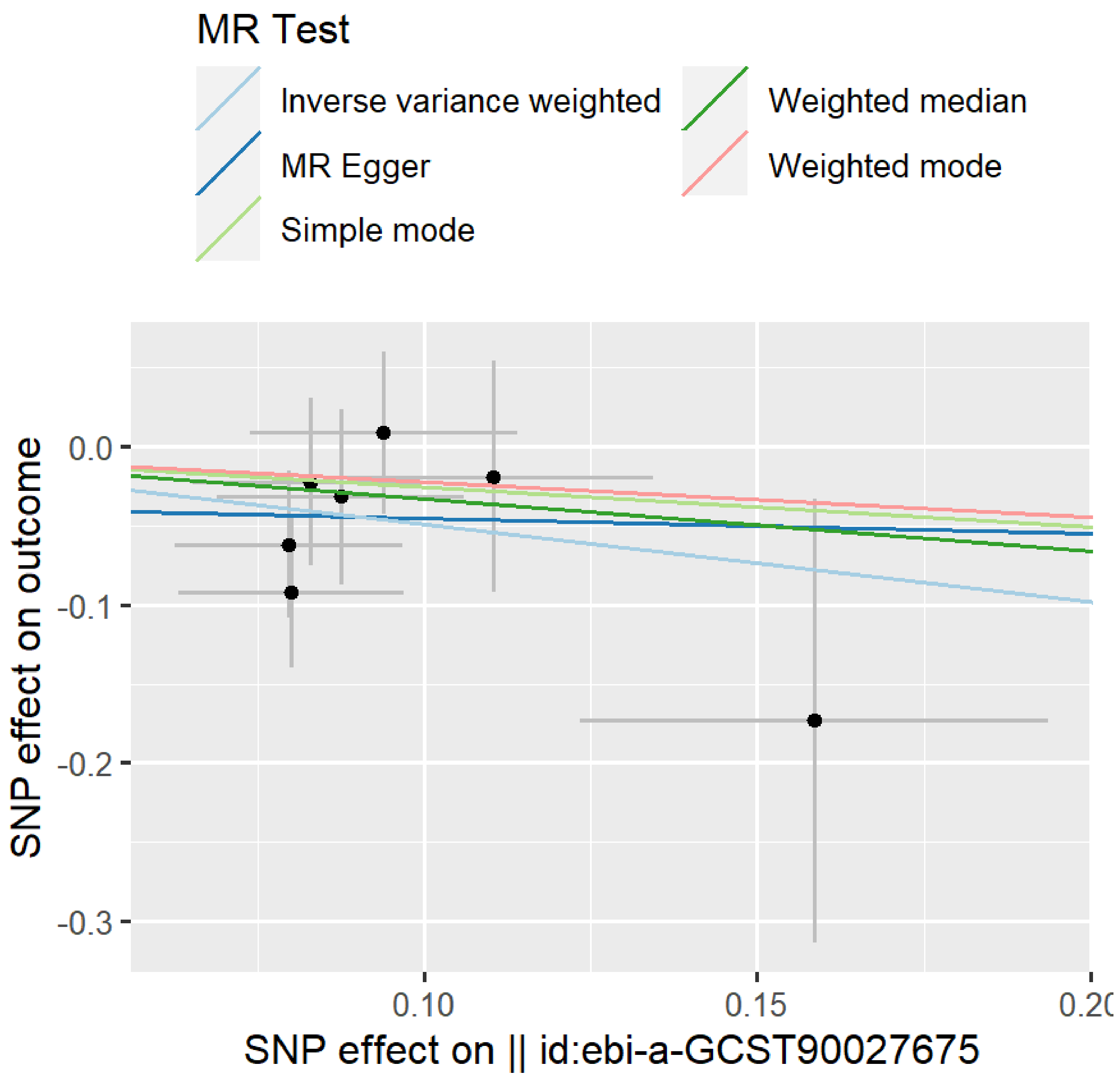

MR Test
Inverse variance weighted
MR Egger
Simple mode
Weighted median
Weighted mode
SNP effect on outcome
0.0
-0.1
-0.2
-0.3
0.10
0.15
0.20
SNP effect on || id:ebi-a-GCST90027675

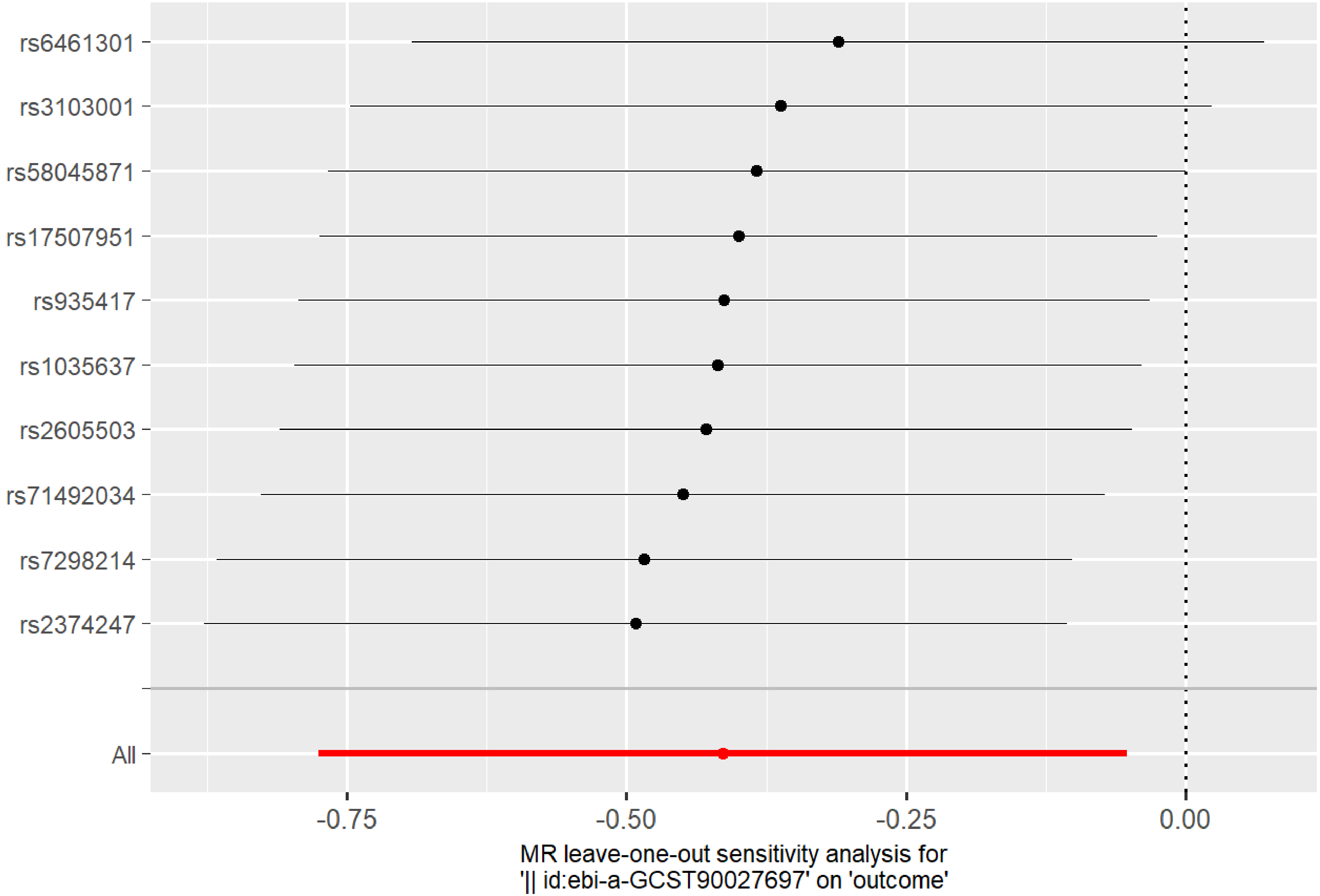
rs6461301
rs3103001
rs58045871
rs17507951
rs935417
rs1035637
rs2605503
rs71492034
rs7298214
rs2374247
All
-0.75
-0.50
-0.25
0.00
MR leave-one-out sensitivity analysis for
'|| id:ebi-a-GCST90027697' on 'outcome'

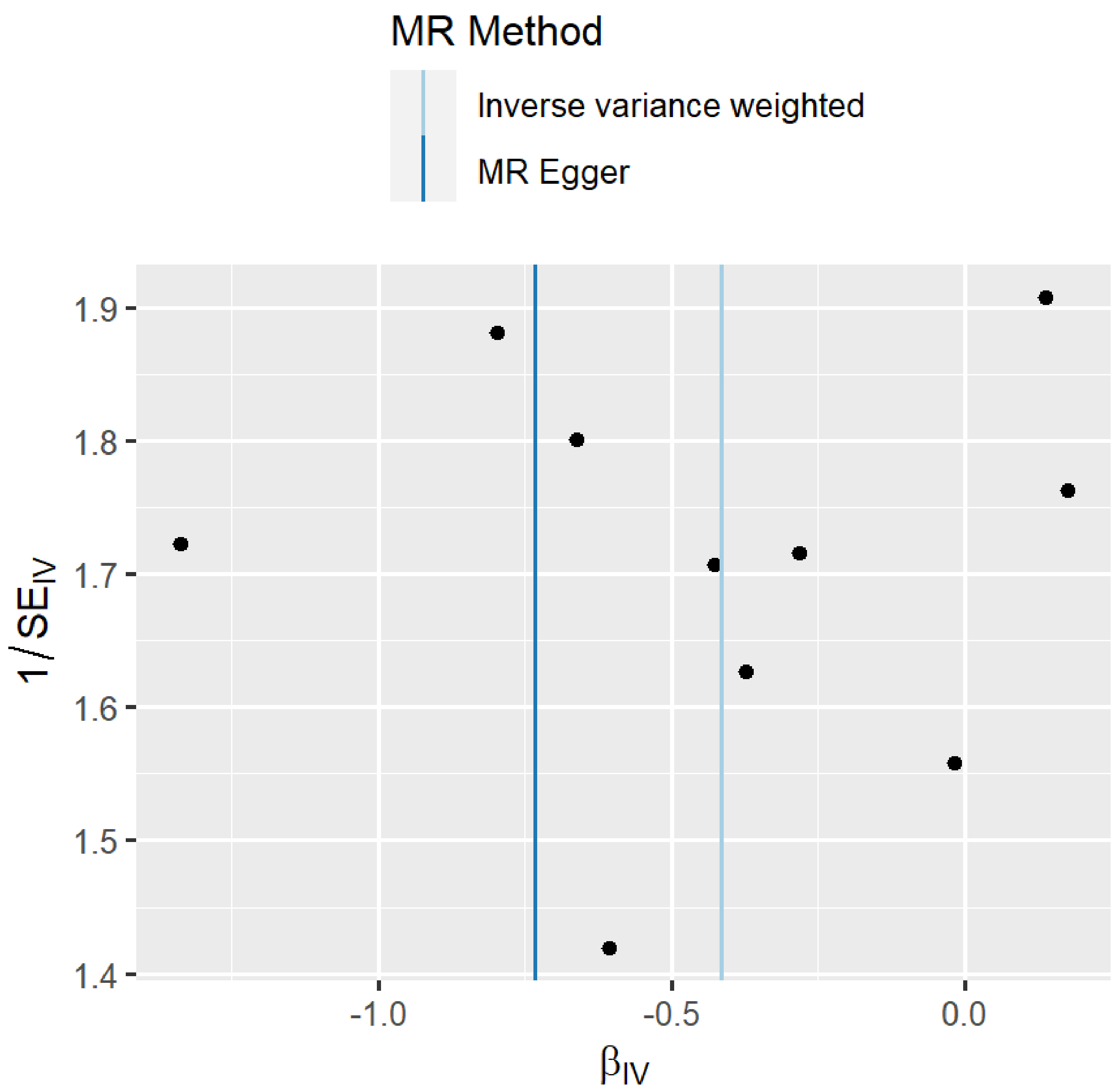
MR Method
Inverse variance weighted
MR Egger
1.9
1.8
1.7
1.6
1.5
1.4
1/SE_IV
-1.0
-0.5
0.0
β_IV

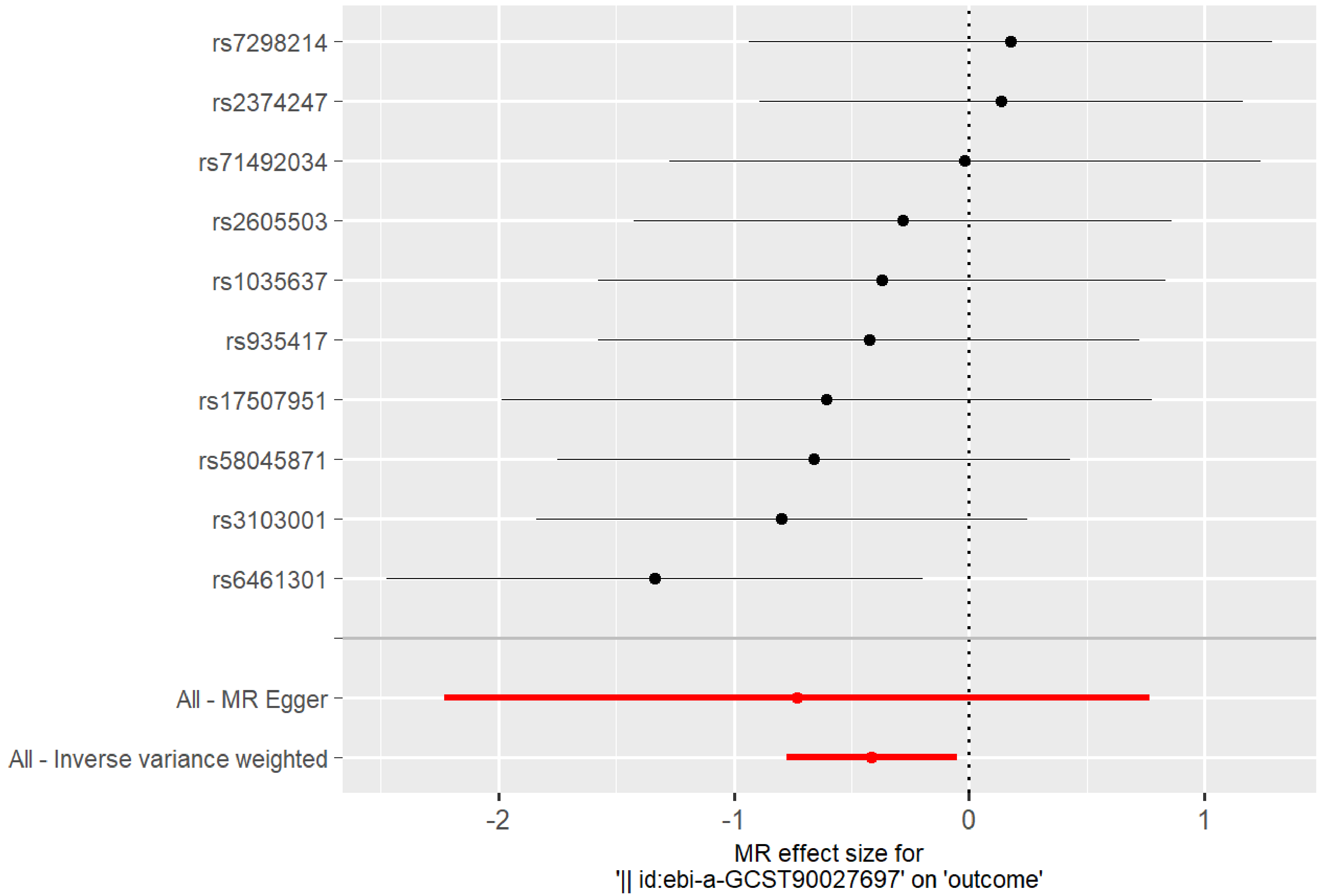
rs7298214
rs2374247
rs71492034
rs2605503
rs1035637
rs935417
rs17507951
rs58045871
rs3103001
rs6461301
All - MR Egger
All - Inverse variance weighted
-2
-1
0
1
MR effect size for
'|| id:ebi-a-GCST90027697' on 'outcome'

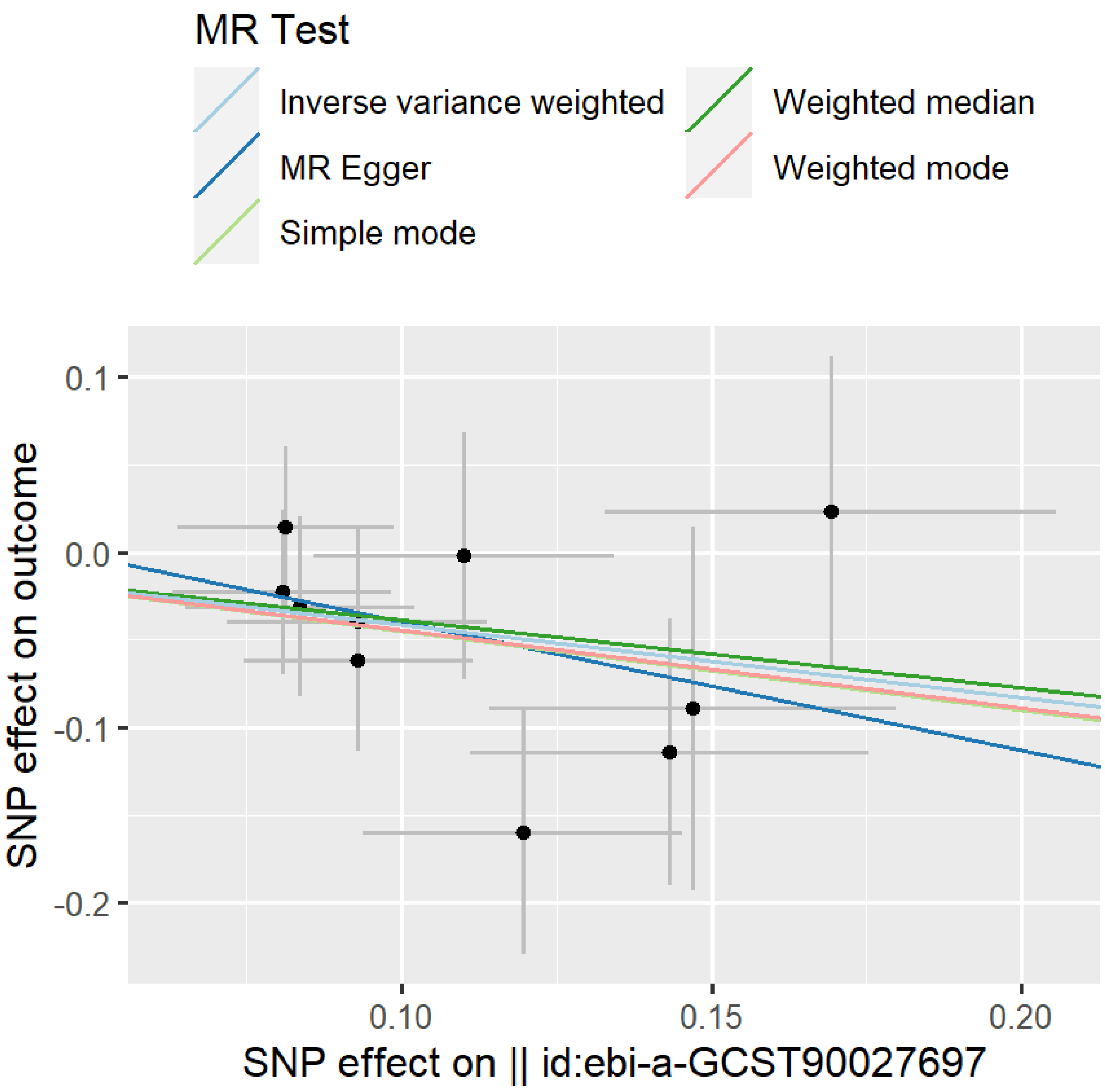
MR Test
Inverse variance weighted
MR Egger
Simple mode
Weighted median
Weighted mode
SNP effect on outcome
0.1
0.0
-0.1
-0.2
0.10
0.15
0.20
SNP effect on || id:ebi-a-GCST90027697

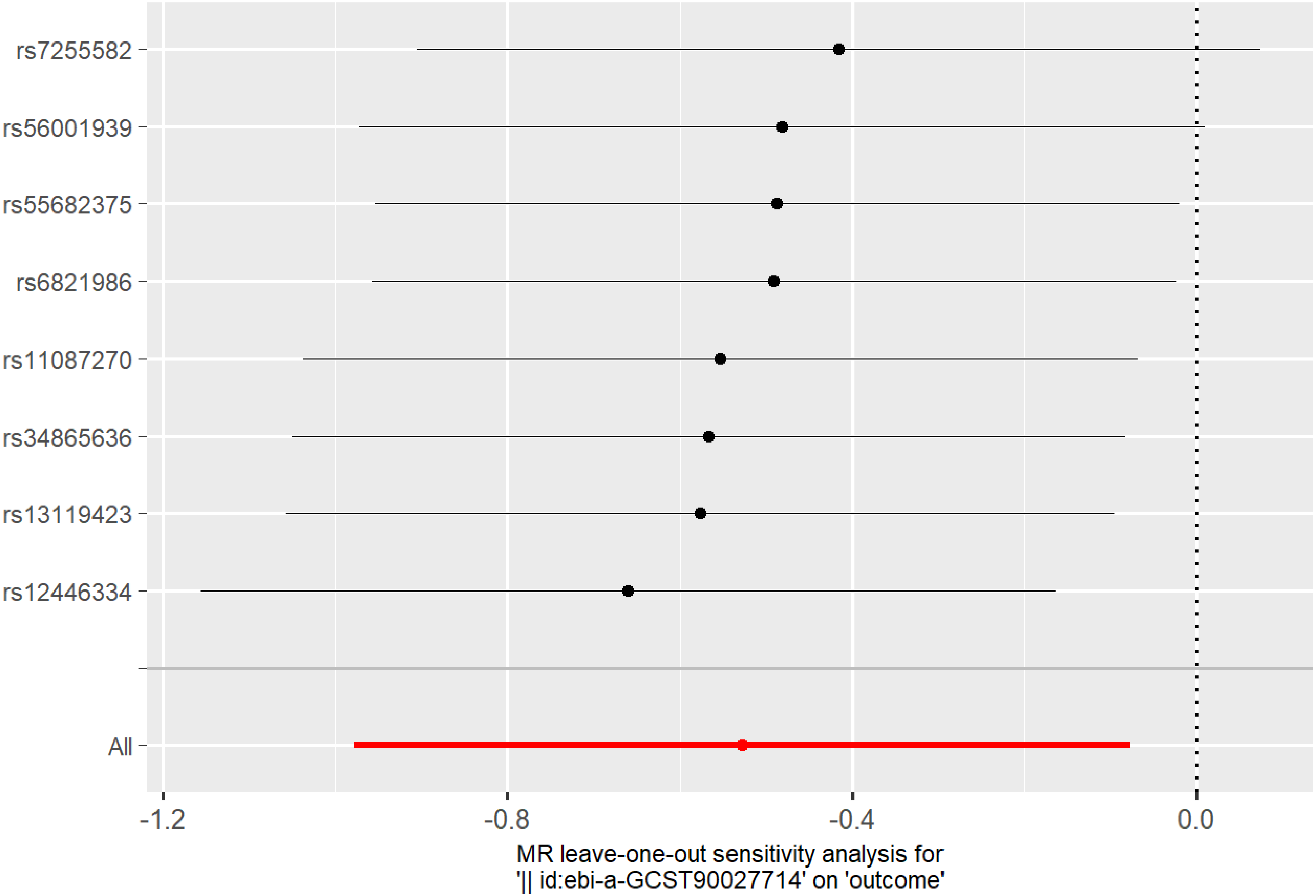
rs7255582
rs56001939
rs55682375
rs6821986
rs11087270
rs34865636
rs13119423
rs12446334
All
-1.2
-0.8
-0.4
0.0
MR leave-one-out sensitivity analysis for
'|| id:ebi-a-GCST90027714' on 'outcome'

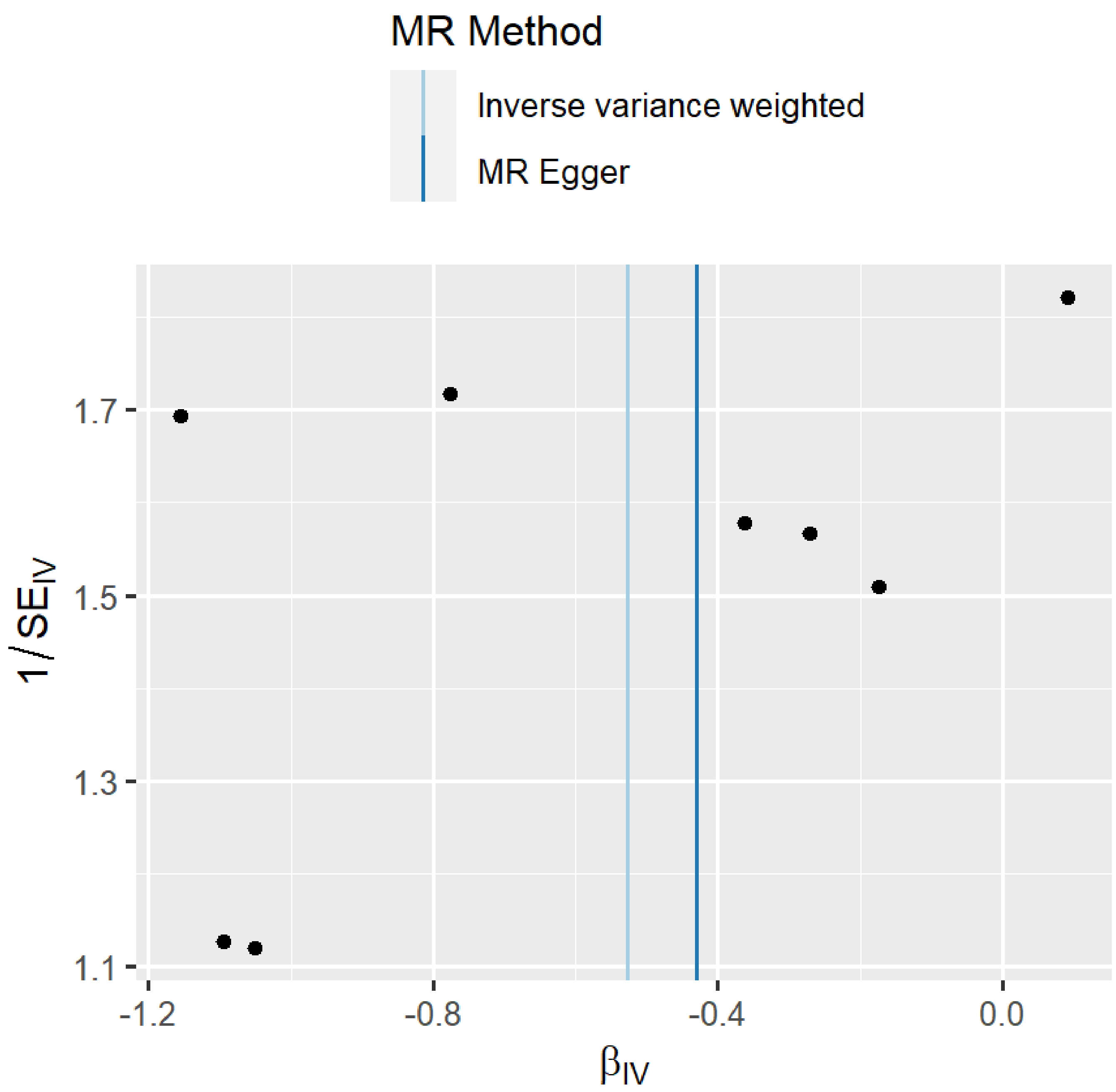
MR Method
Inverse variance weighted
MR Egger
1.7
1.5
1.3
1.1
1/SE$_{IV}$
-1.2
-0.8
-0.4
0.0
β$_{IV}$

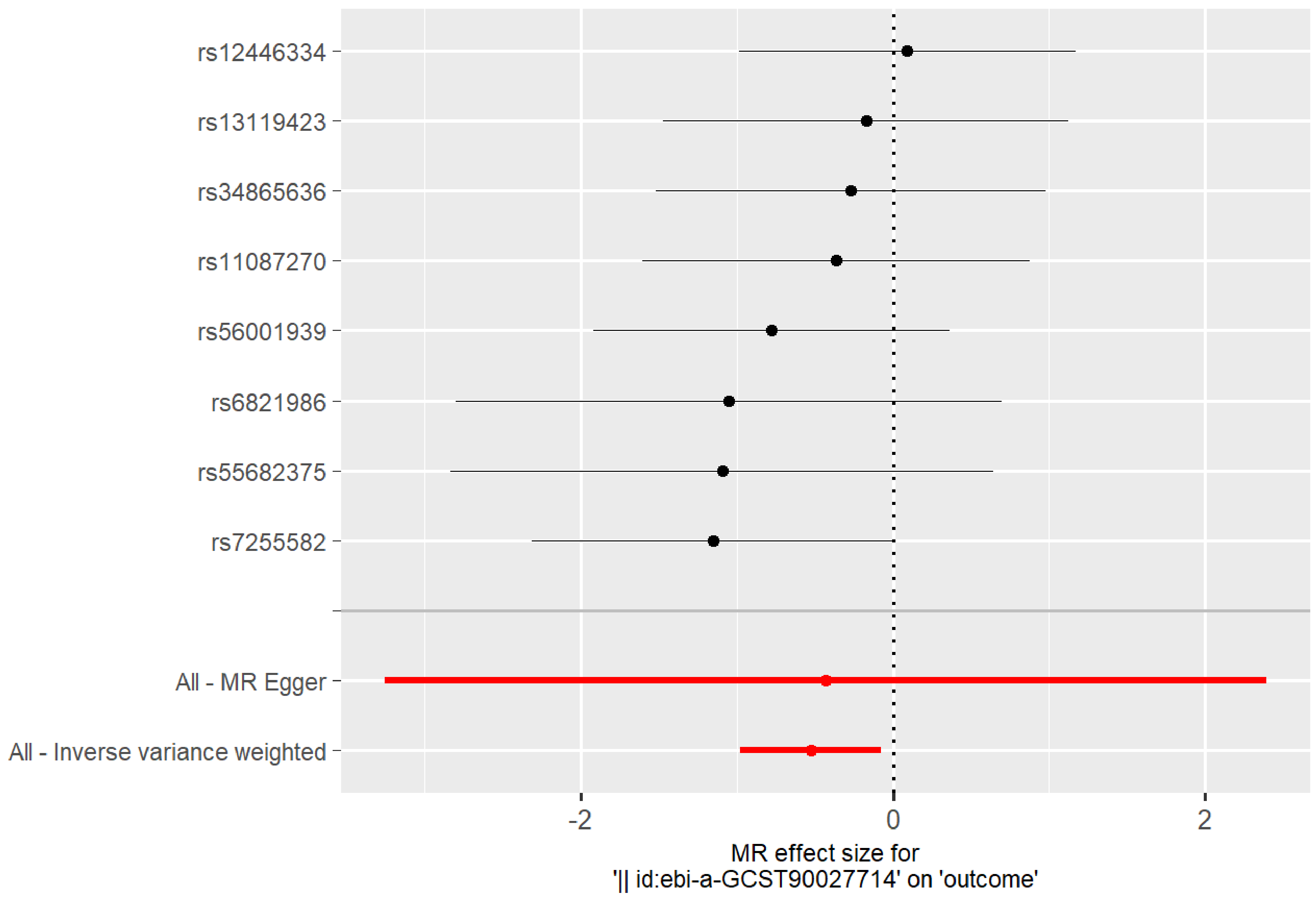
rs12446334
rs13119423
rs34865636
rs11087270
rs56001939
rs6821986
rs55682375
rs7255582
All - MR Egger
All - Inverse variance weighted
-2
0
2
MR effect size for
'|| id:ebi-a-GCST90027714' on 'outcome'

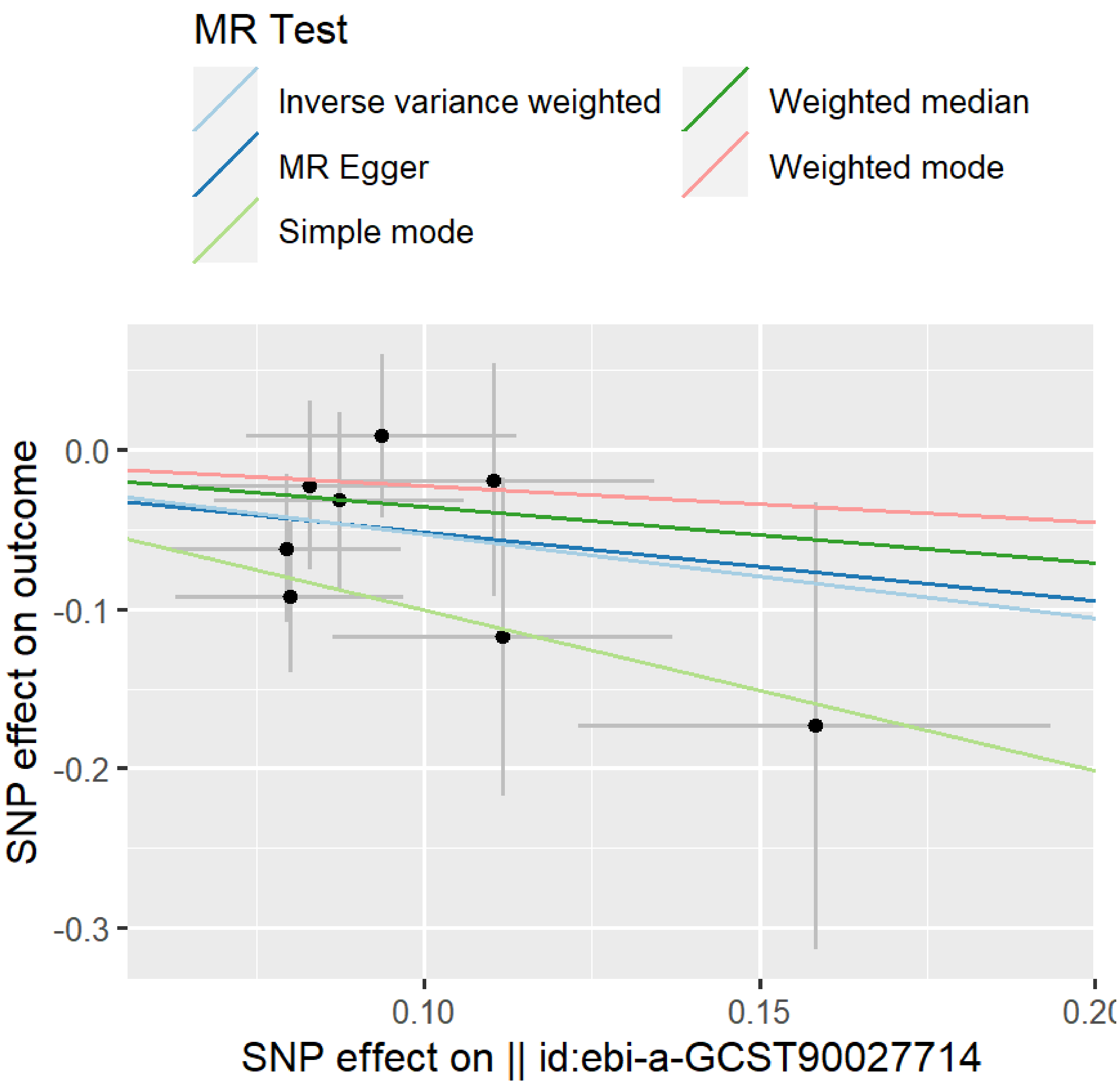

MR Test
Inverse variance weighted
MR Egger
Simple mode
Weighted median
Weighted mode
0.0
-0.1
-0.2
-0.3
SNP effect on outcome
0.10
0.15
SNP effect on || id:ebi-a-GCST90027714

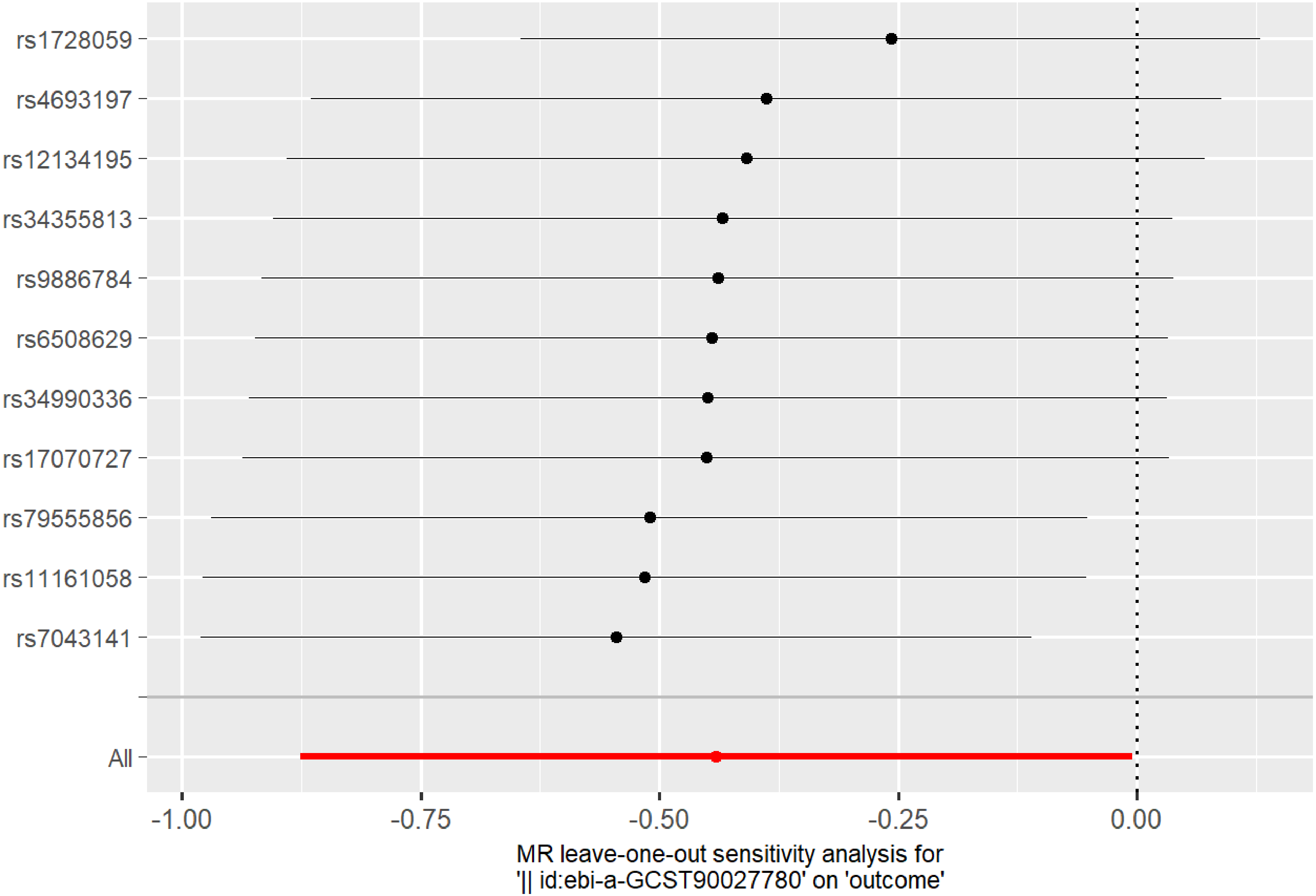
rs1728059
rs4693197
rs12134195
rs34355813
rs9886784
rs6508629
rs34990336
rs17070727
rs79555856
rs11161058
rs7043141
All
-1.00
-0.75
-0.50
-0.25
0.00
MR leave-one-out sensitivity analysis for
'|| id:ebi-a-GCST90027780' on 'outcome'

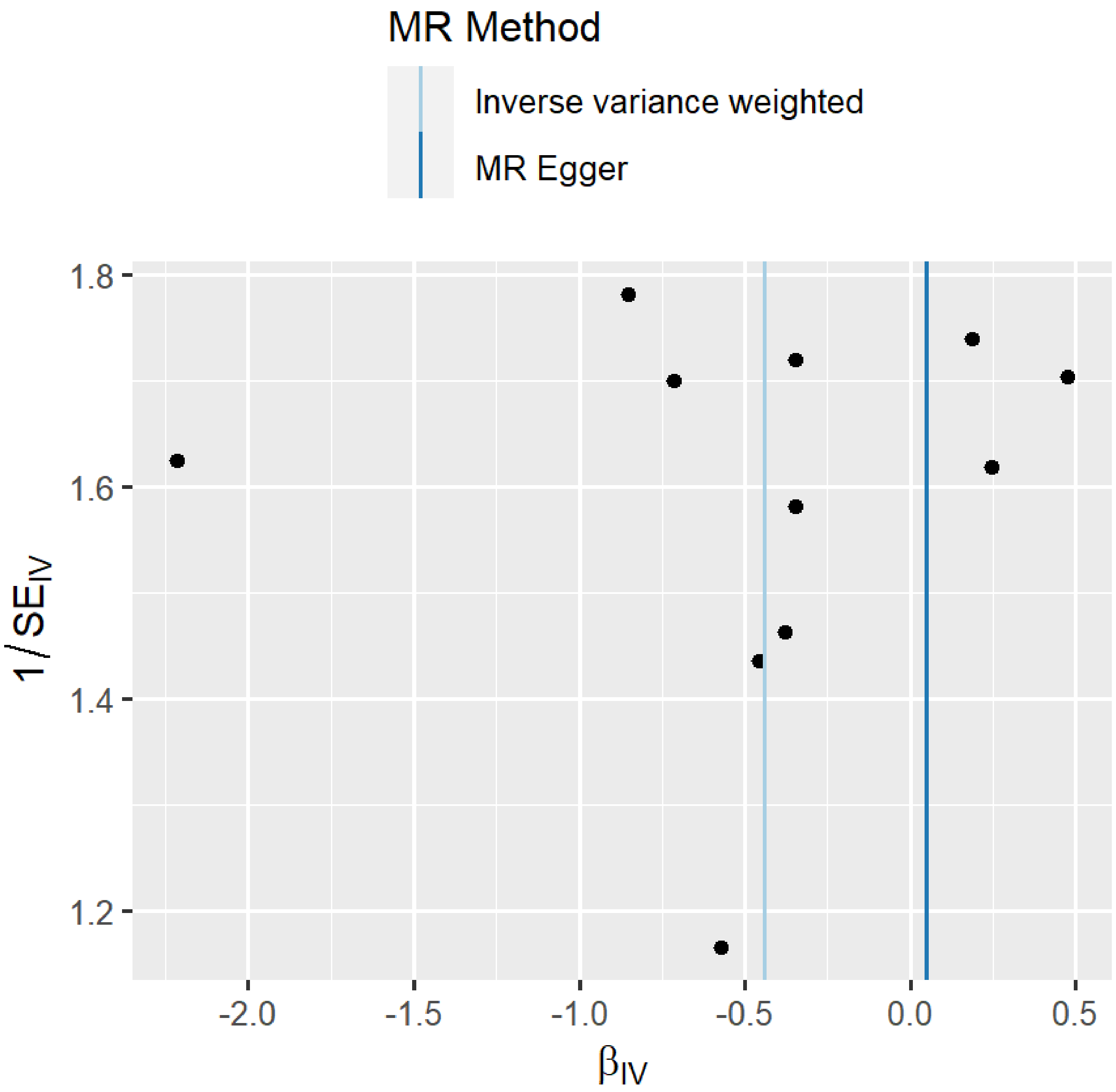
MR Method
Inverse variance weighted
MR Egger
1.8
1.6
1.4
1.2
1/SE_IV
-2.0
-1.5
-1.0
-0.5
0.0
0.5
β_IV

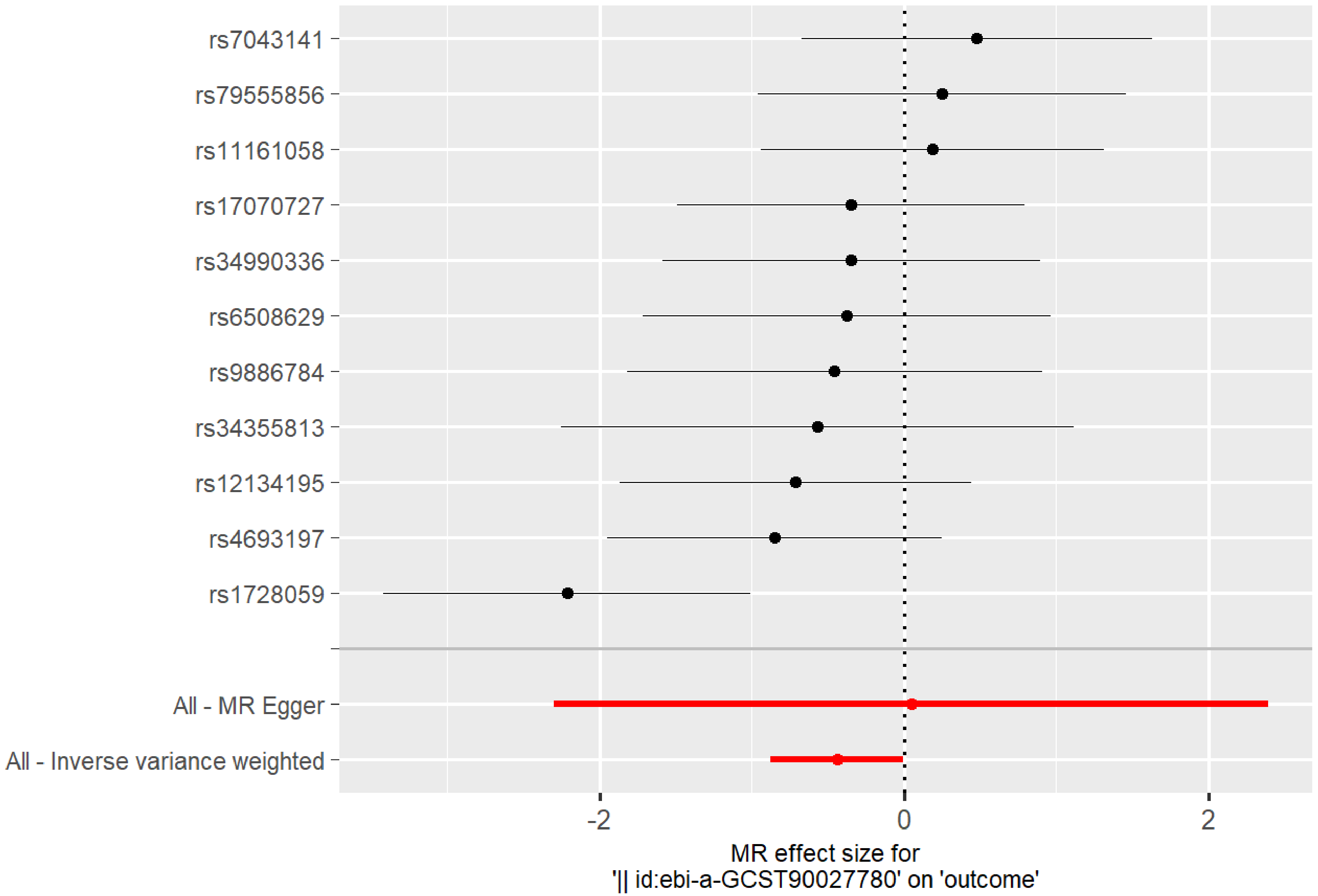
rs7043141
rs79555856
rs11161058
rs17070727
rs34990336
rs6508629
rs9886784
rs34355813
rs12134195
rs4693197
rs1728059
All - MR Egger
All - Inverse variance weighted
-2
0
2
MR effect size for
'|| id:ebi-a-GCST90027780' on 'outcome'

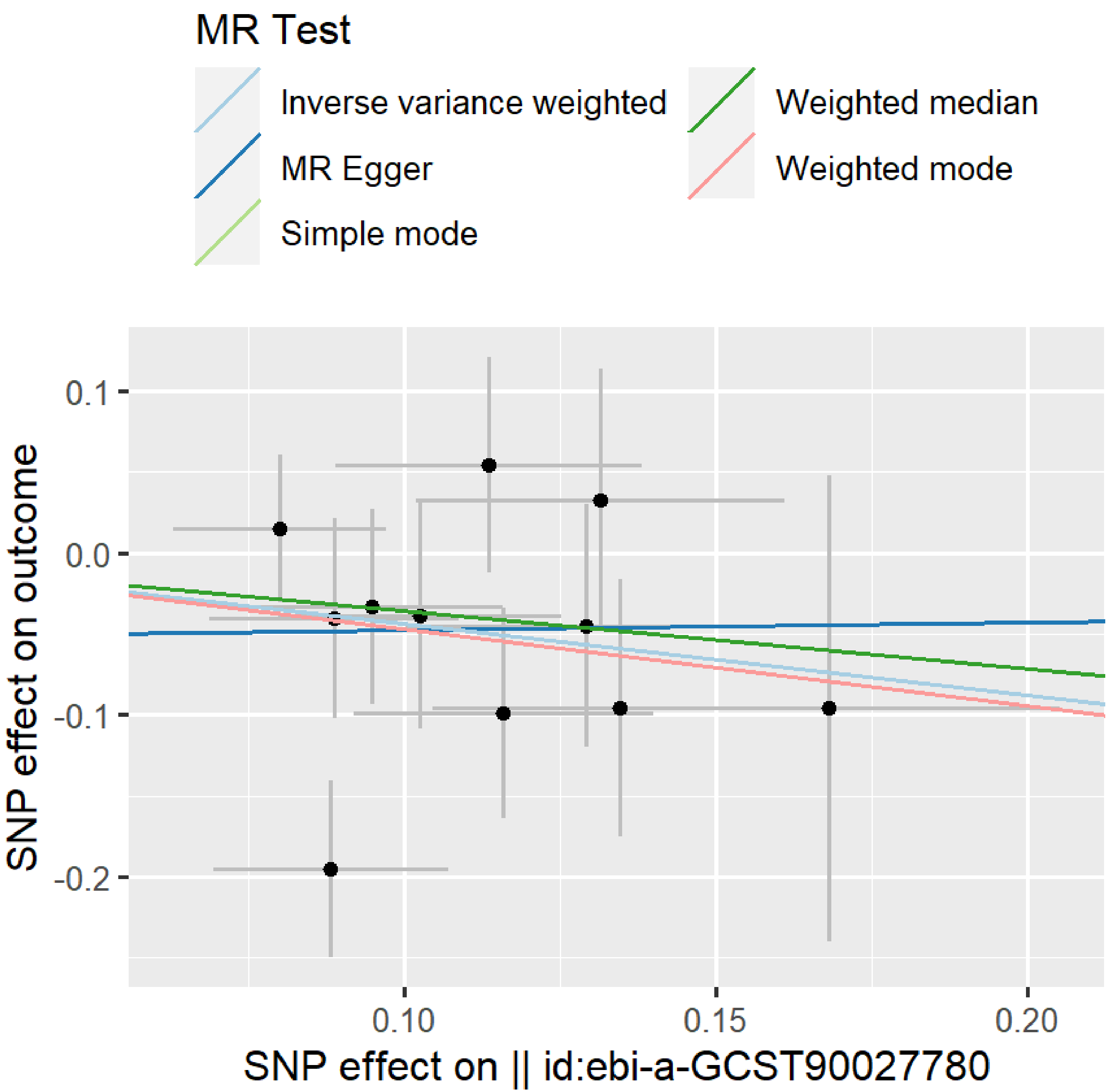
MR Test
Inverse variance weighted
MR Egger
Simple mode
Weighted median
Weighted mode
0.1
0.0
-0.1
-0.2
SNP effect on outcome
0.10
0.15
0.20
SNP effect on || id:ebi-a-GCST90027780

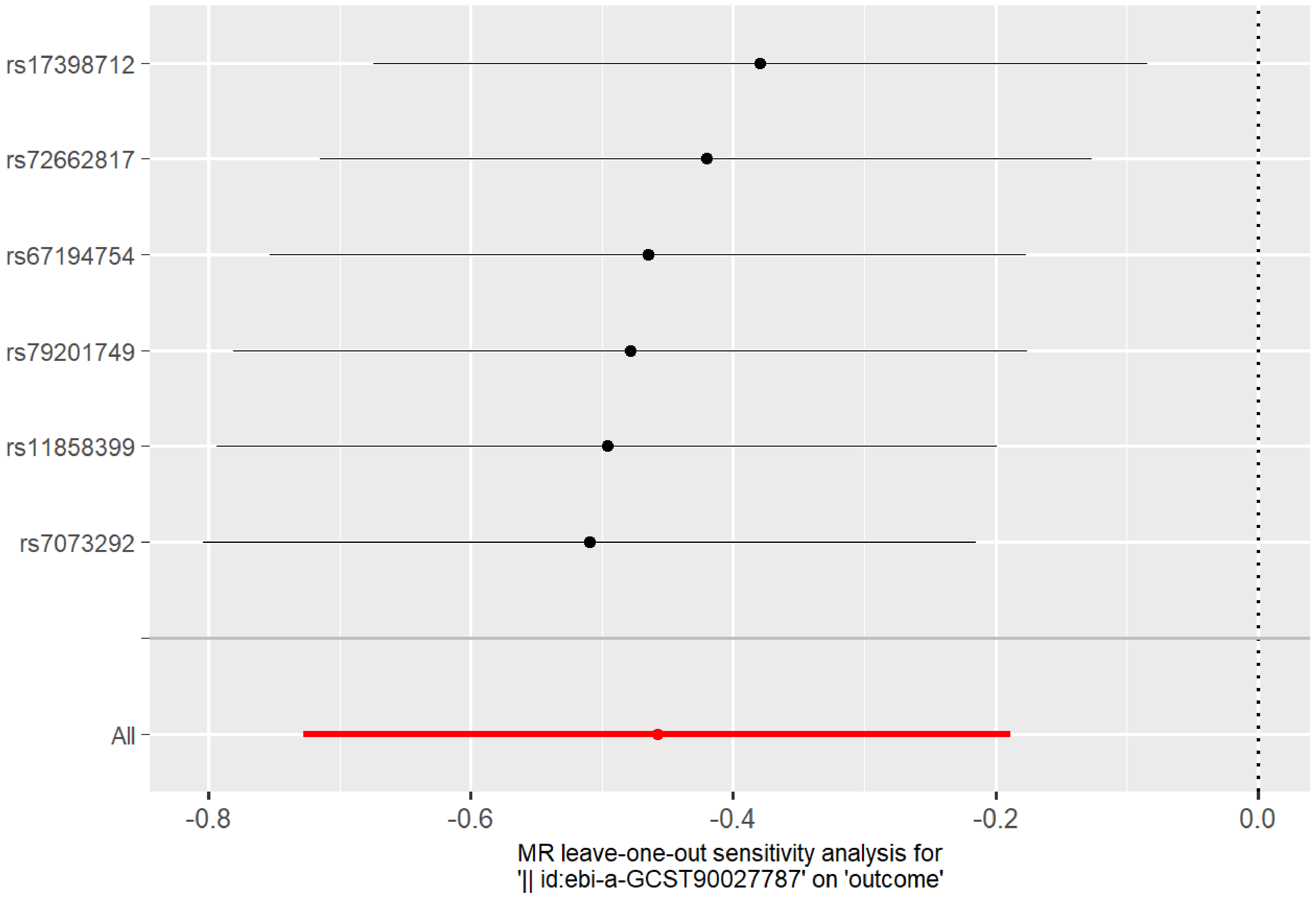

rs17398712
rs72662817
rs67194754
rs79201749
rs11858399
rs7073292
All
-0.8
-0.6
-0.4
-0.2
0.0
MR leave-one-out sensitivity analysis for
'|| id:ebi-a-GCST90027787' on 'outcome'

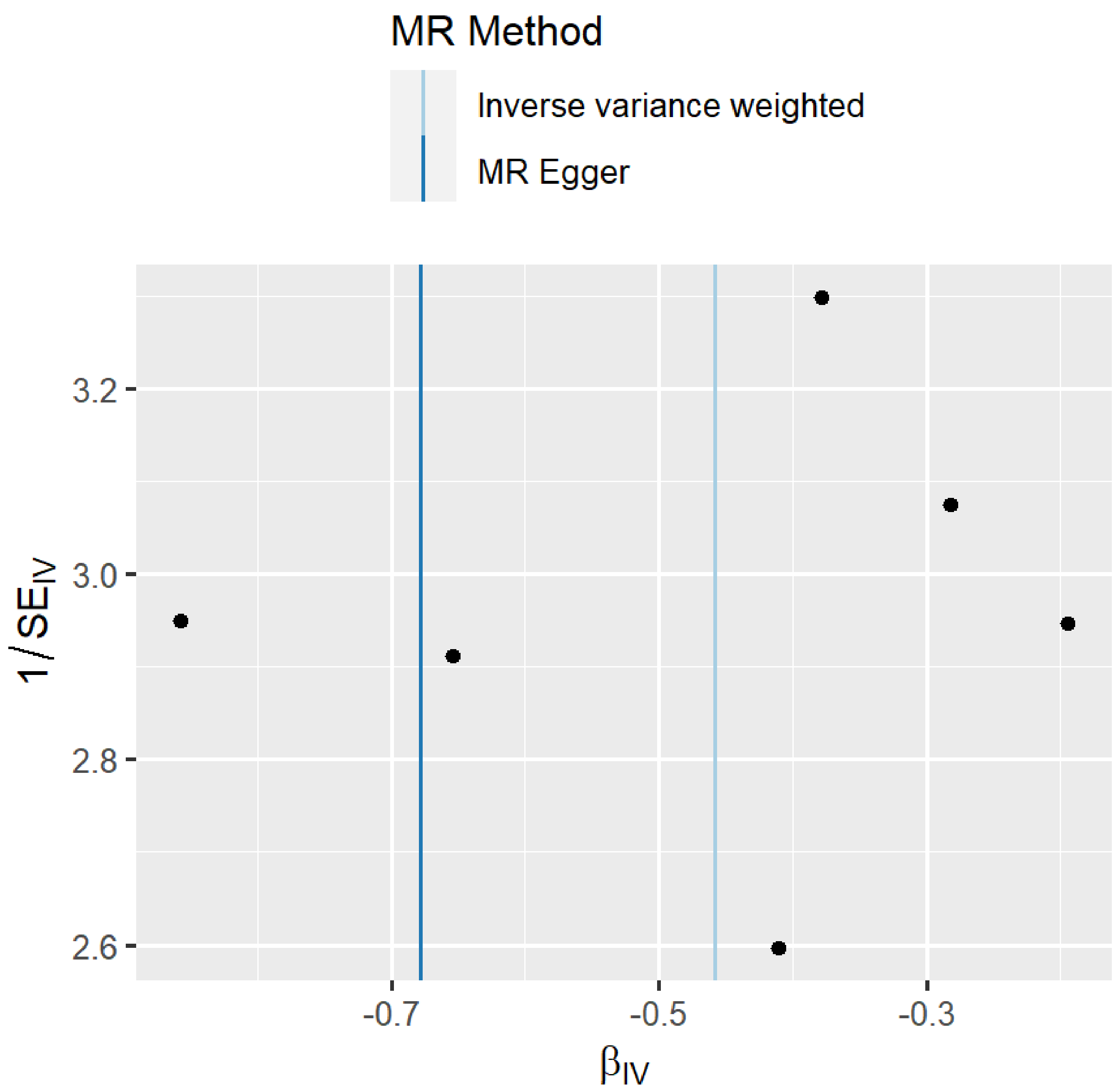
MR Method
Inverse variance weighted
MR Egger
3.2
3.0
2.8
2.6
1/SE$_{IV}$
-0.7
-0.5
-0.3
β_{IV}

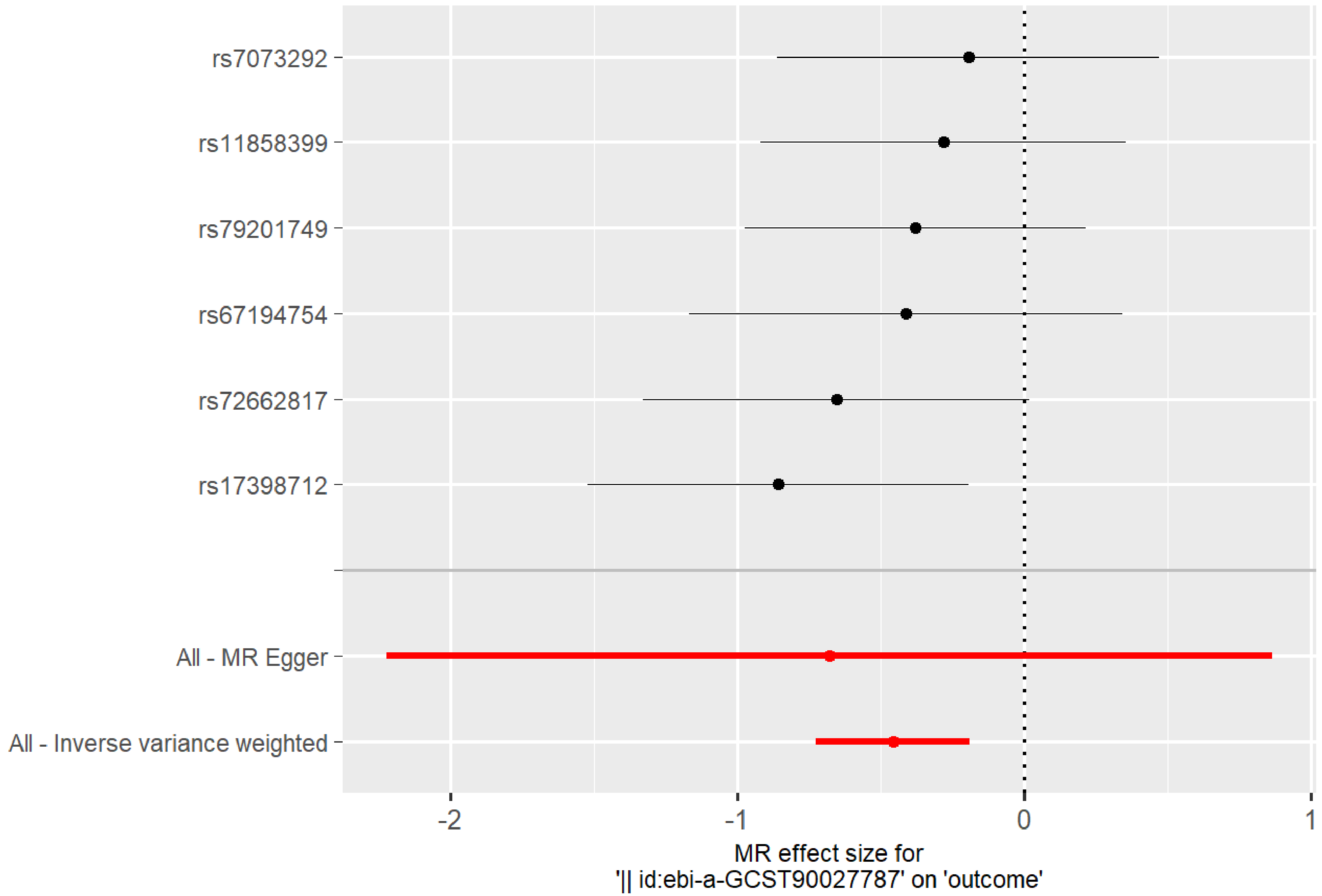
rs7073292
rs11858399
rs79201749
rs67194754
rs72662817
rs17398712
All - MR Egger
All - Inverse variance weighted
-2
-1
0
1
MR effect size for
'|| id:ebi-a-GCST90027787' on 'outcome'

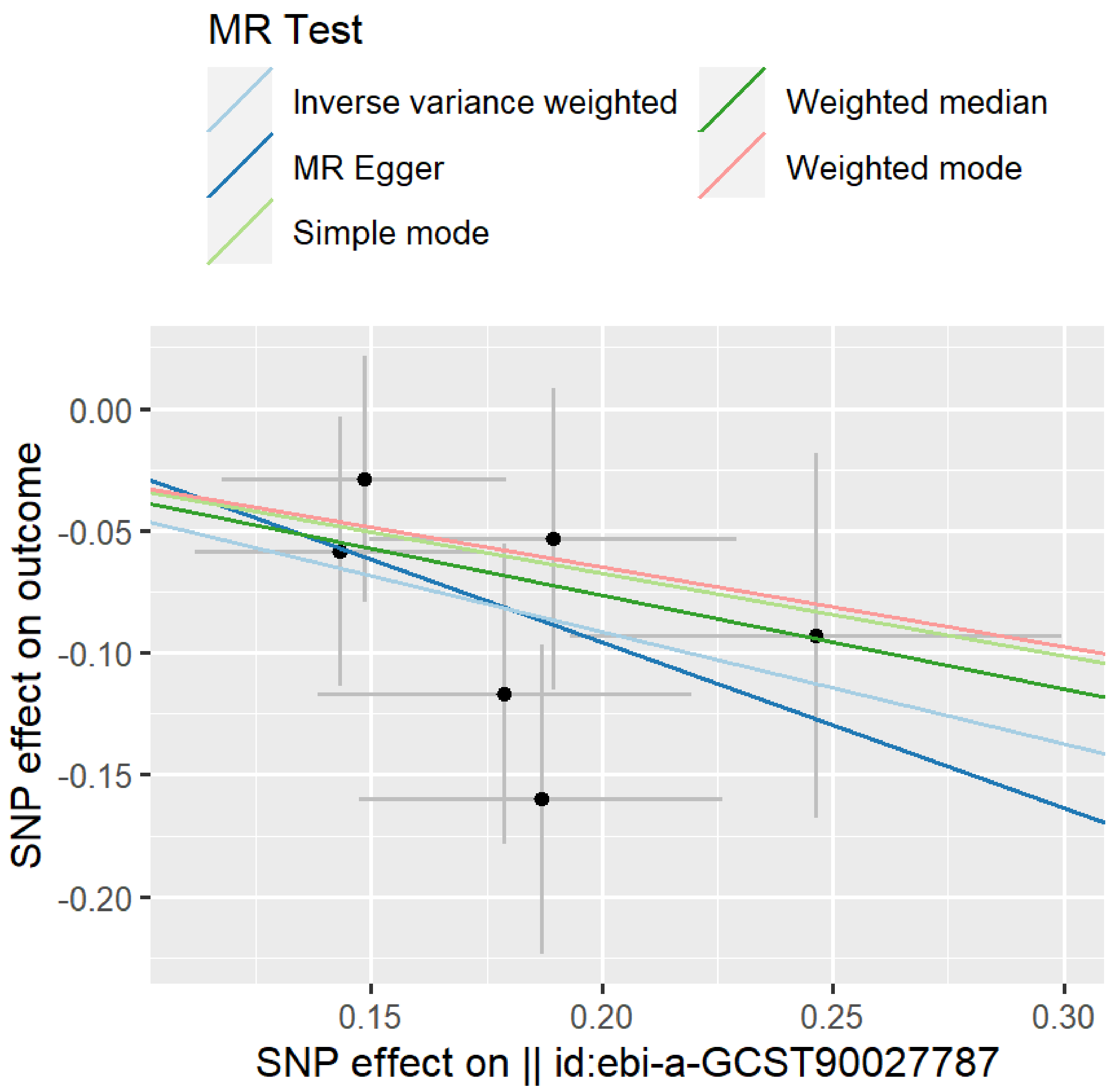
MR Test
Inverse variance weighted
MR Egger
Simple mode
Weighted median
Weighted mode
0.00
-0.05
-0.10
-0.15
-0.20
SNP effect on outcome
0.15
0.20
0.25
0.30
SNP effect on || id:ebi-a-GCST90027787

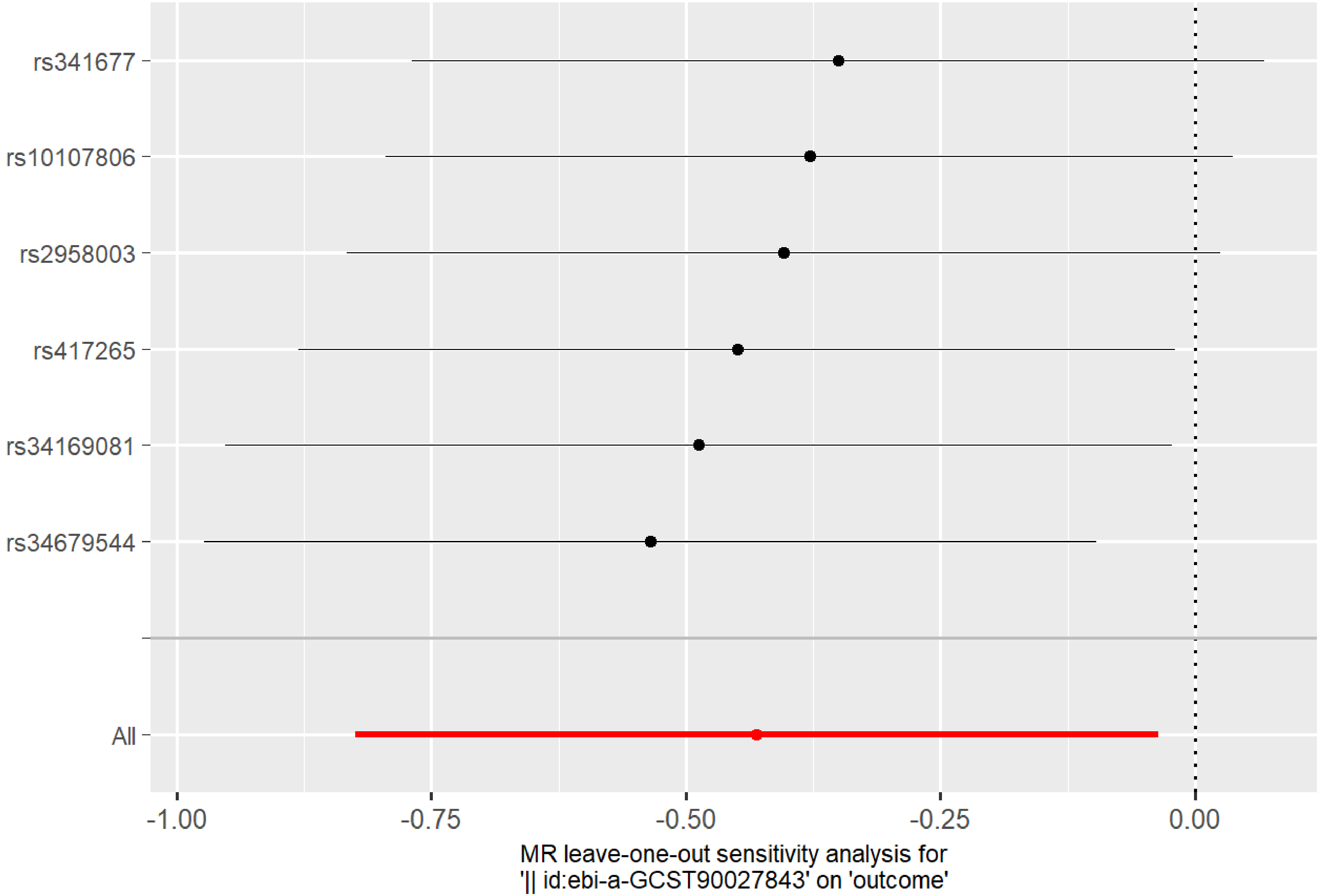
rs341677
rs10107806
rs2958003
rs417265
rs34169081
rs34679544
All
-1.00
-0.75
-0.50
-0.25
0.00
MR leave-one-out sensitivity analysis for
'|| id:ebi-a-GCST90027843' on 'outcome'

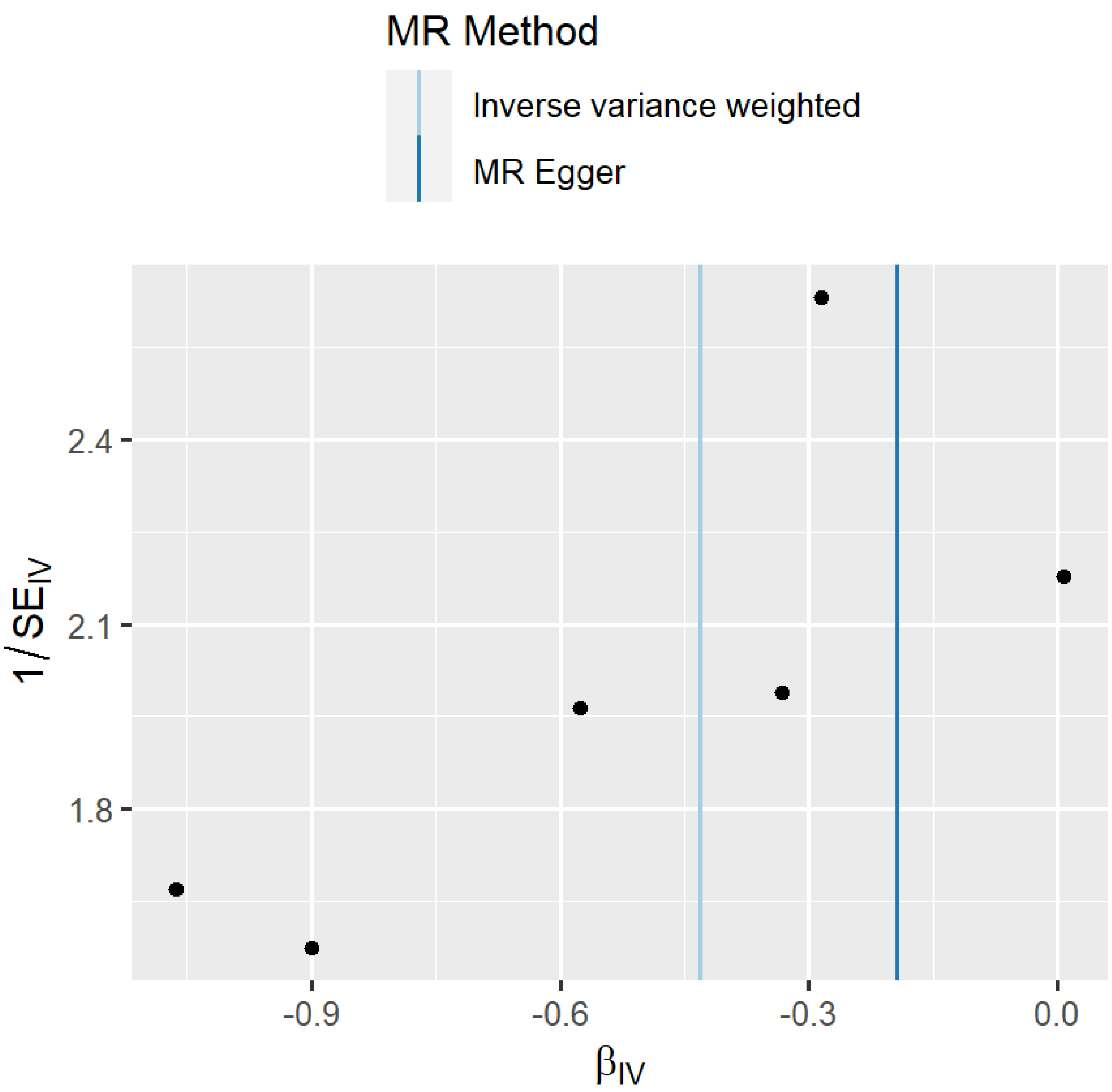
MR Method
Inverse variance weighted
MR Egger
2.4
2.1
1.8
$1/SE_{IV}$
-0.9
-0.6
-0.3
0.0
β_{IV}

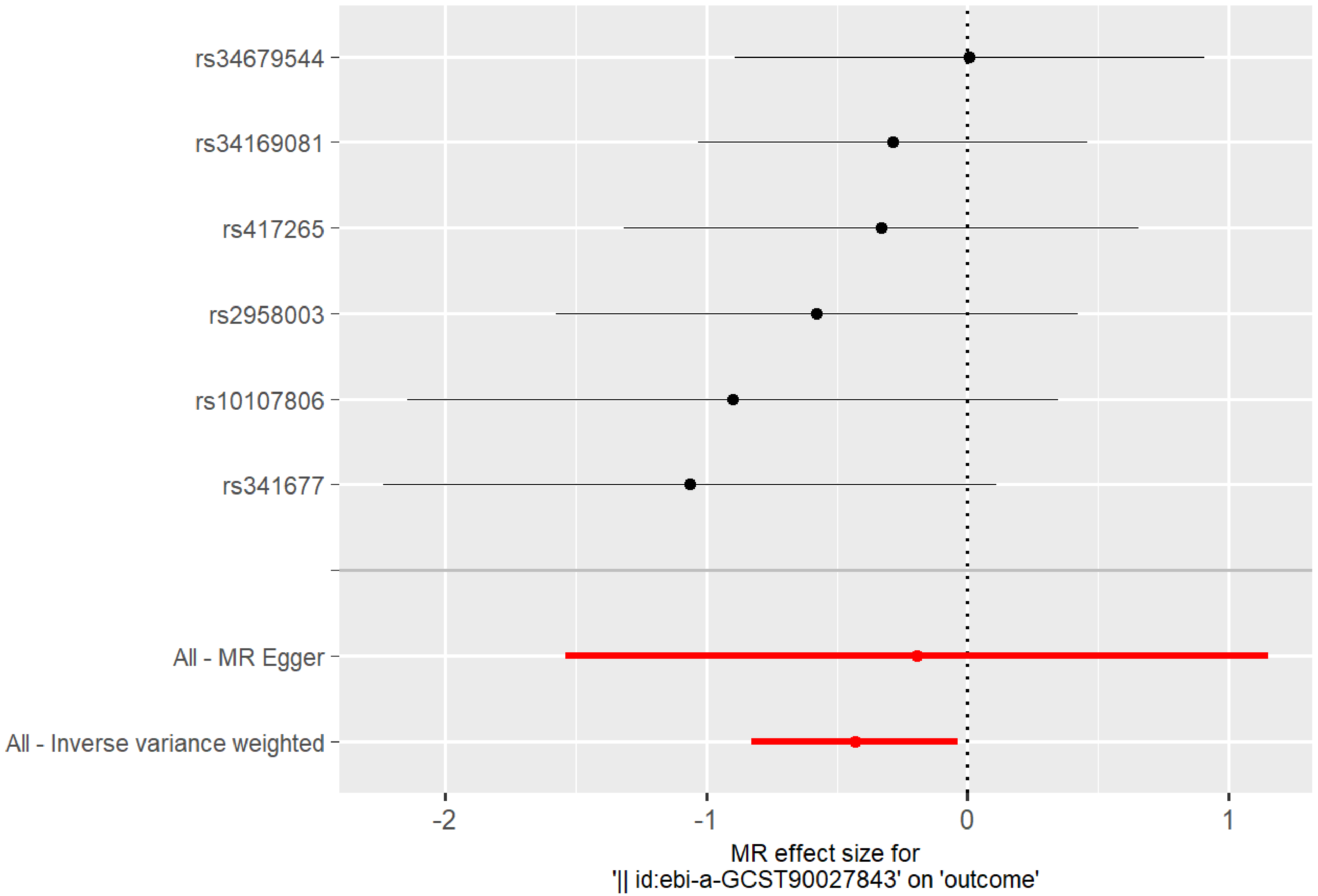
rs34679544
rs34169081
rs417265
rs2958003
rs10107806
rs341677
All - MR Egger
All - Inverse variance weighted
-2
-1
0
1
MR effect size for
'|| id:ebi-a-GCST90027843' on 'outcome'

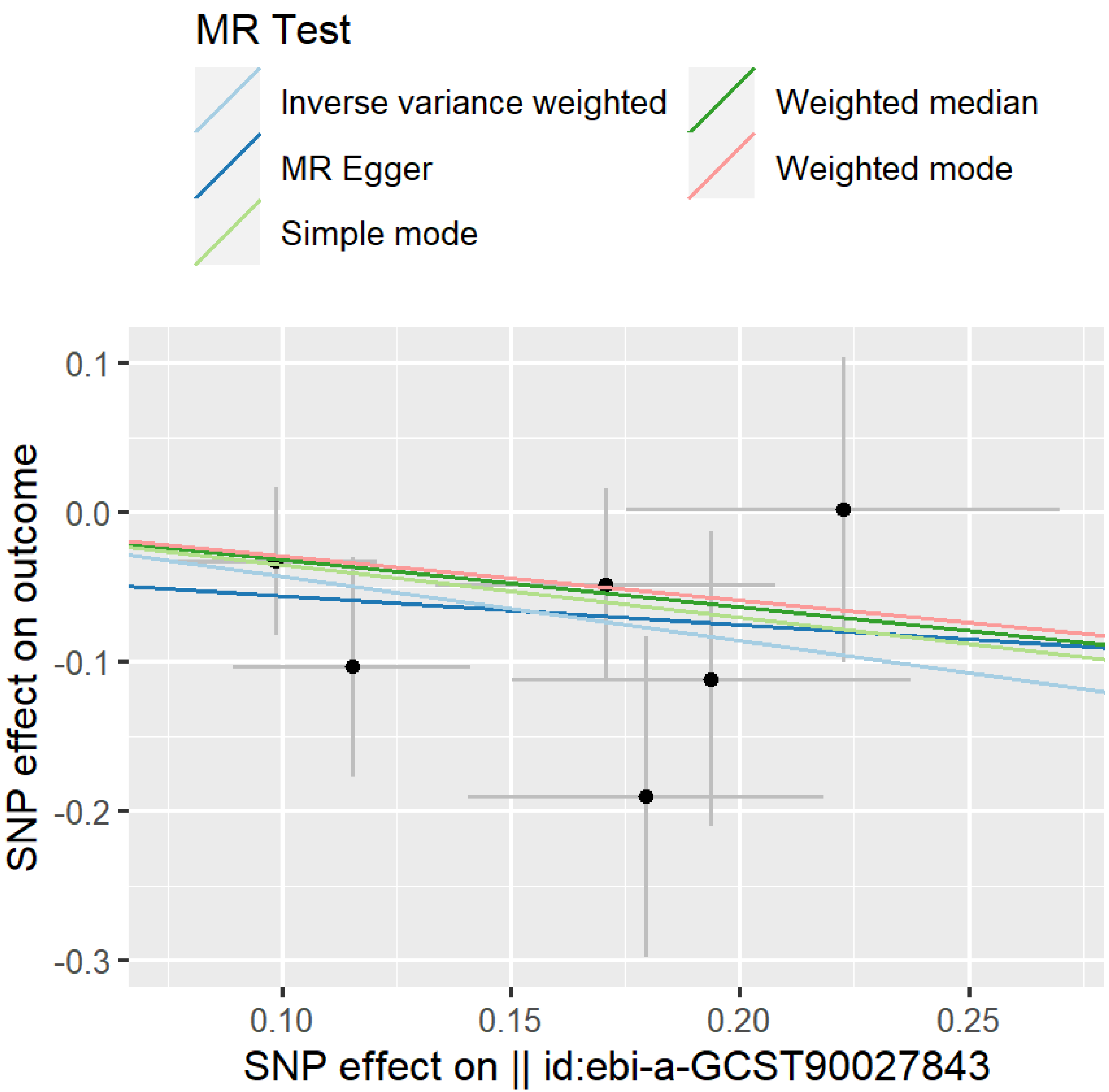
MR Test
Inverse variance weighted
MR Egger
Simple mode
Weighted median
Weighted mode
SNP effect on outcome
0.1
0.0
-0.1
-0.2
-0.3
0.10
0.15
0.20
0.25
SNP effect on || id:ebi-a-GCST90027843

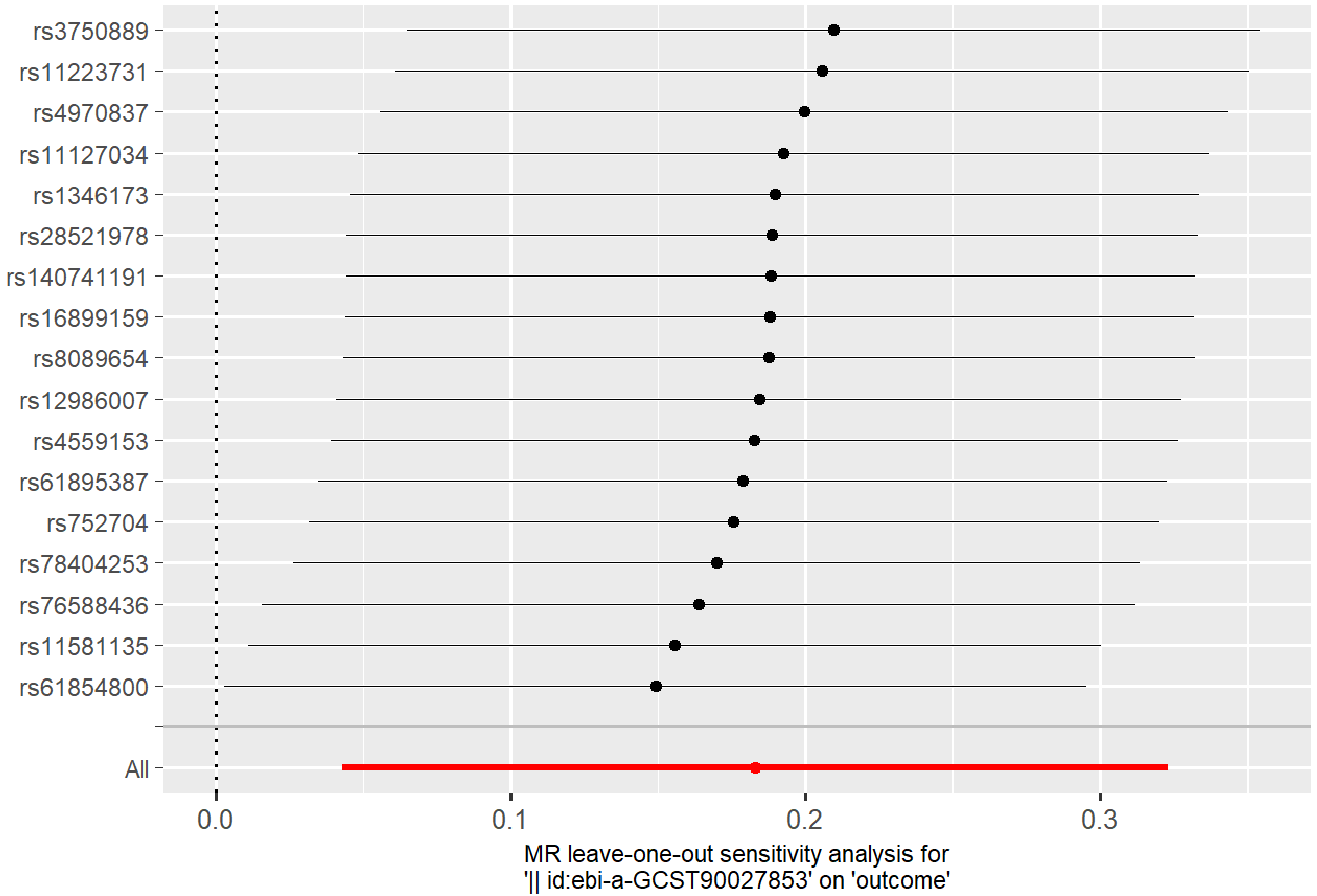
rs3750889
rs11223731
rs4970837
rs11127034
rs1346173
rs28521978
rs140741191
rs16899159
rs8089654
rs12986007
rs4559153
rs61895387
rs752704
rs78404253
rs76588436
rs11581135
rs61854800
All
0.0
0.1
0.2
0.3
MR leave-one-out sensitivity analysis for
'|| id:ebi-a-GCST90027853' on 'outcome'

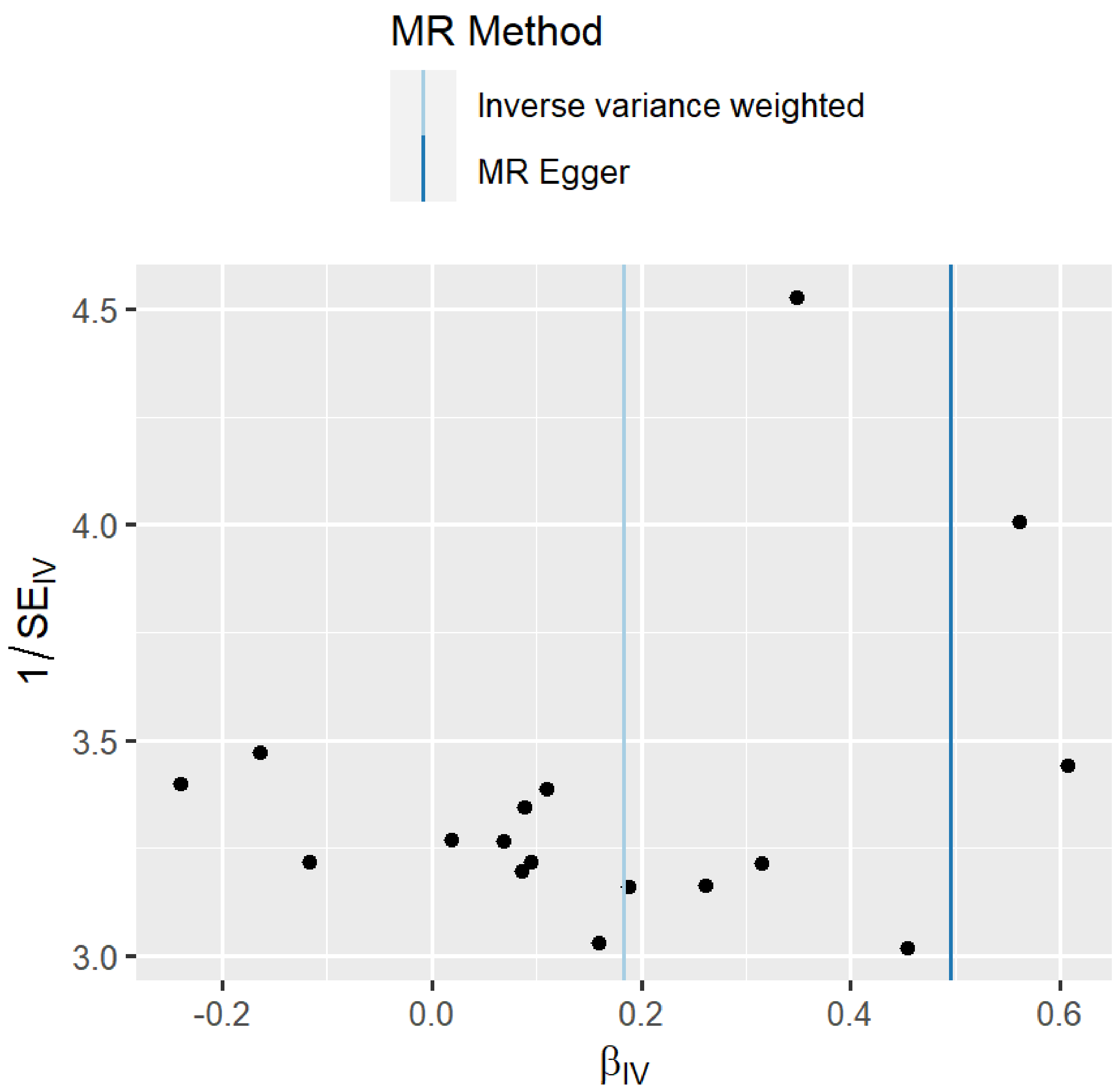

MR Method
Inverse variance weighted
MR Egger
4.5
4.0
3.5
3.0
-0.2
0.0
0.2
0.4
0.6
1/SE_IV
β_IV

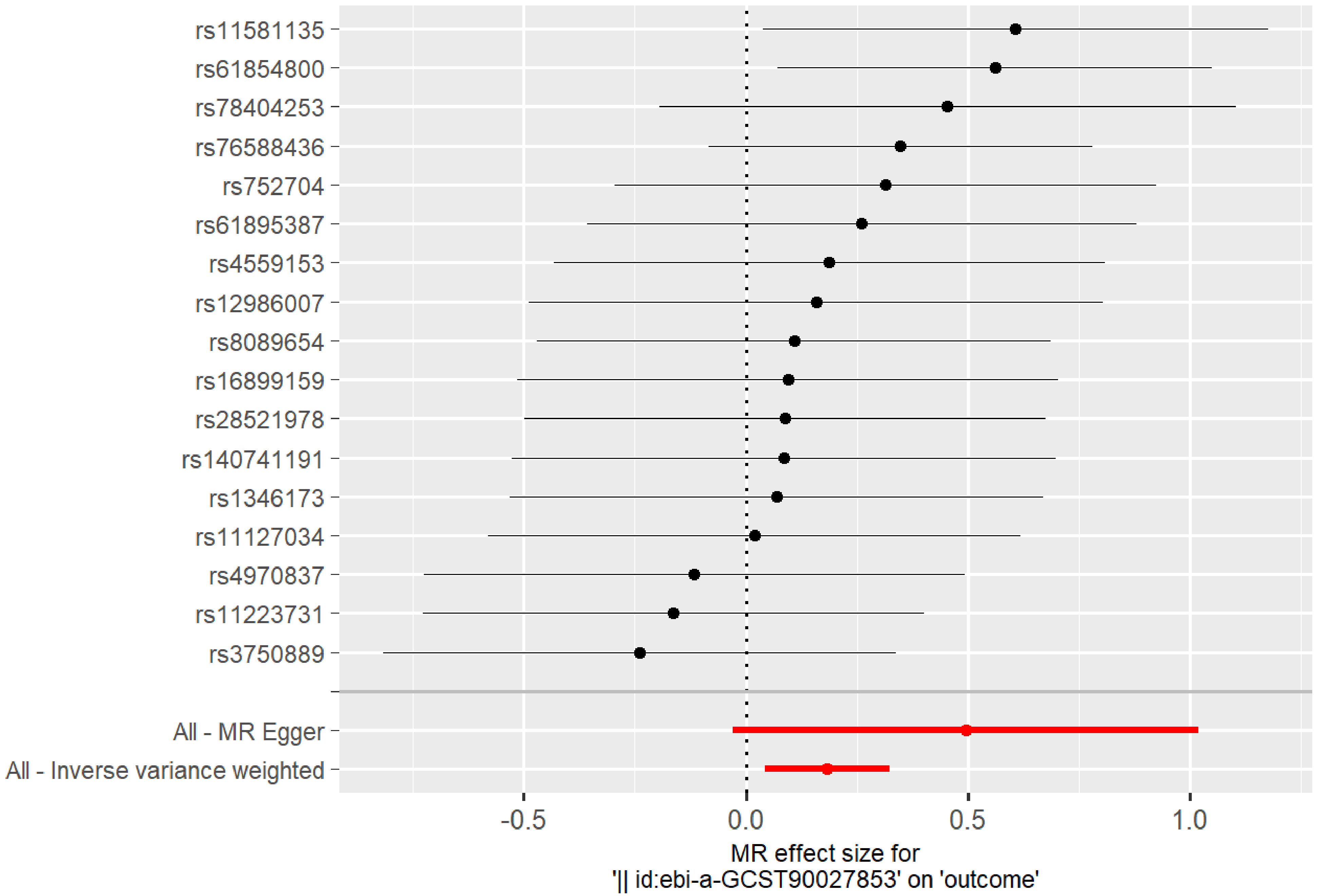
rs11581135
rs61854800
rs78404253
rs76588436
rs752704
rs61895387
rs4559153
rs12986007
rs8089654
rs16899159
rs28521978
rs140741191
rs1346173
rs11127034
rs4970837
rs11223731
rs3750889
All - MR Egger
All - Inverse variance weighted
-0.5
0.0
0.5
1.0
MR effect size for
'|| id:ebi-a-GCST90027853' on 'outcome'

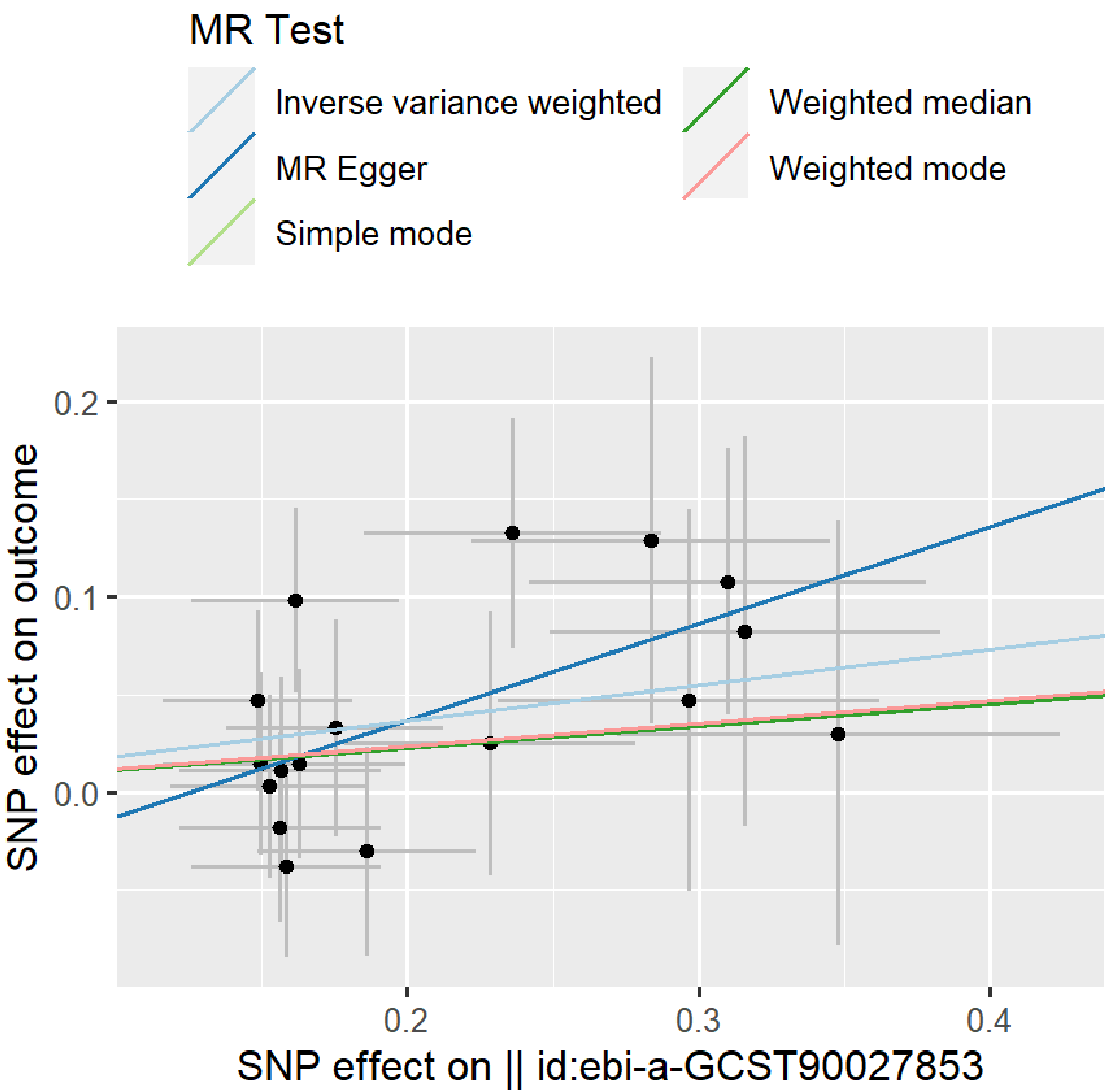
MR Test
Inverse variance weighted
MR Egger
Simple mode
Weighted median
Weighted mode
0.2
0.1
0.0
SNP effect on outcome
0.2
0.3
0.4
SNP effect on || id:ebi-a-GCST90027853

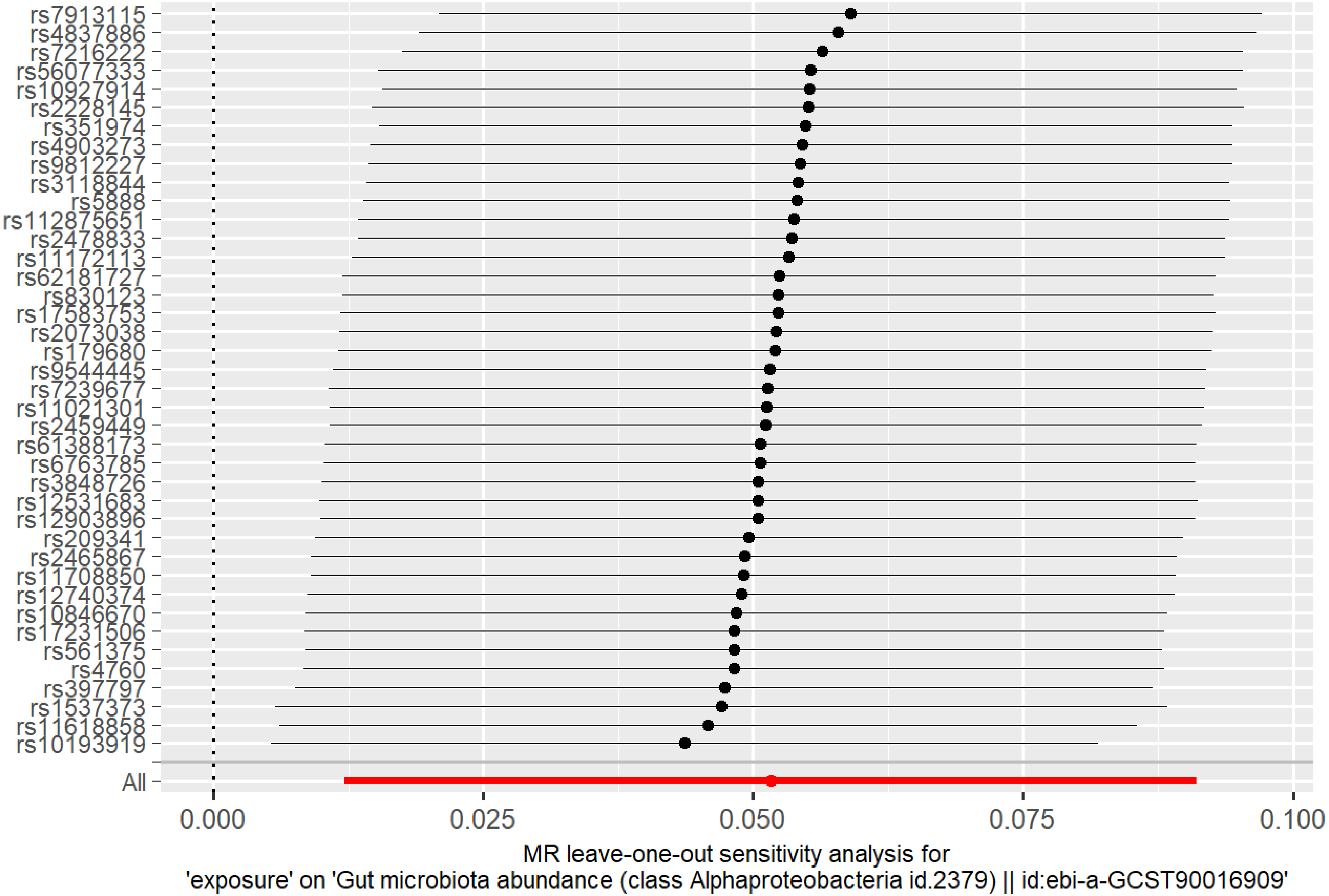
rs7913115
rs4837886
rs7216222
rs56077333
rs10927914
rs2228145
rs351974
rs4903273
rs9812227
rs3118844
rs5888
rs112875651
rs2478833
rs11172113
rs62181727
rs830123
rs17583753
rs2073038
rs179680
rs9544445
rs7239677
rs11021301
rs2459449
rs61388173
rs6763785
rs3848726
rs12531683
rs12903896
rs209341
rs2465867
rs11708850
rs12740374
rs10846670
rs17231506
rs561375
rs4760
rs397797
rs1537373
rs11618858
rs10193919
All
0.000
0.025
0.050
0.075
0.100
MR leave-one-out sensitivity analysis for
'exposure' on 'Gut microbiota abundance (class Alphaproteobacteria id.2379) || id:ebi-a-GCST90016909'

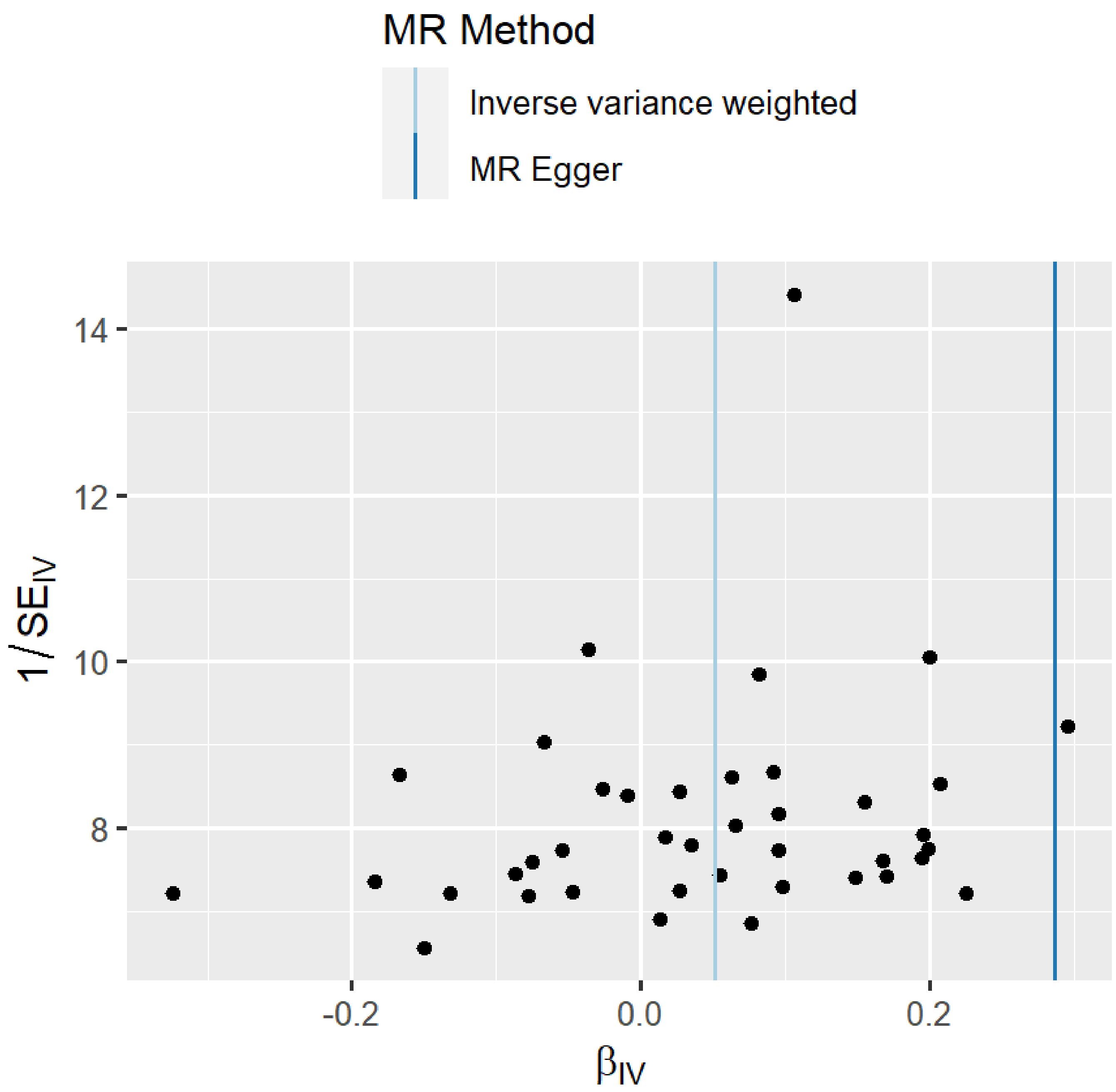
MR Method
Inverse variance weighted
MR Egger
14
12
10
8
1/SE_IV
-0.2
0.0
0.2
β_IV

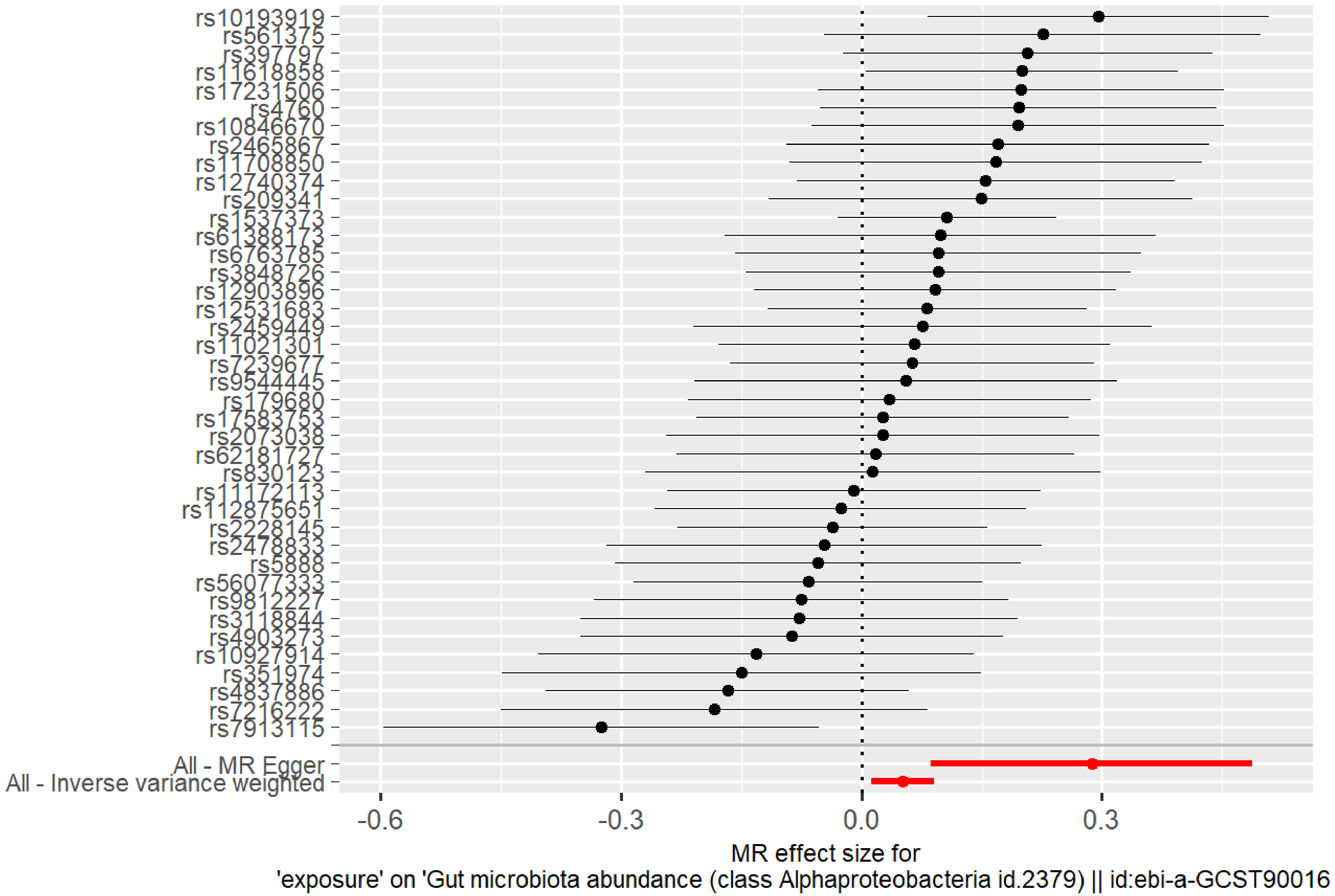
rs10193919
rs561375
rs397797
rs11618858
rs17231506
rs4760
rs10846670
rs2465867
rs11708850
rs12740374
rs209341
rs1537373
rs61388173
rs6763785
rs3848726
rs12903896
rs12531683
rs2459449
rs11021301
rs7239677
rs9544445
rs179680
rs17583753
rs2073038
rs62181727
rs830123
rs11172113
rs112875651
rs2228145
rs2478833
rs5888
rs56077333
rs9812227
rs3118844
rs4903273
rs10927914
rs351974
rs4837886
rs7216222
rs7913115
All - MR Egger
All - Inverse variance weighted
-0.6
-0.3
0.0
0.3
MR effect size for
'exposure' on 'Gut microbiota abundance (class Alphaproteobacteria id.2379) || id:ebi-a-GCST90016

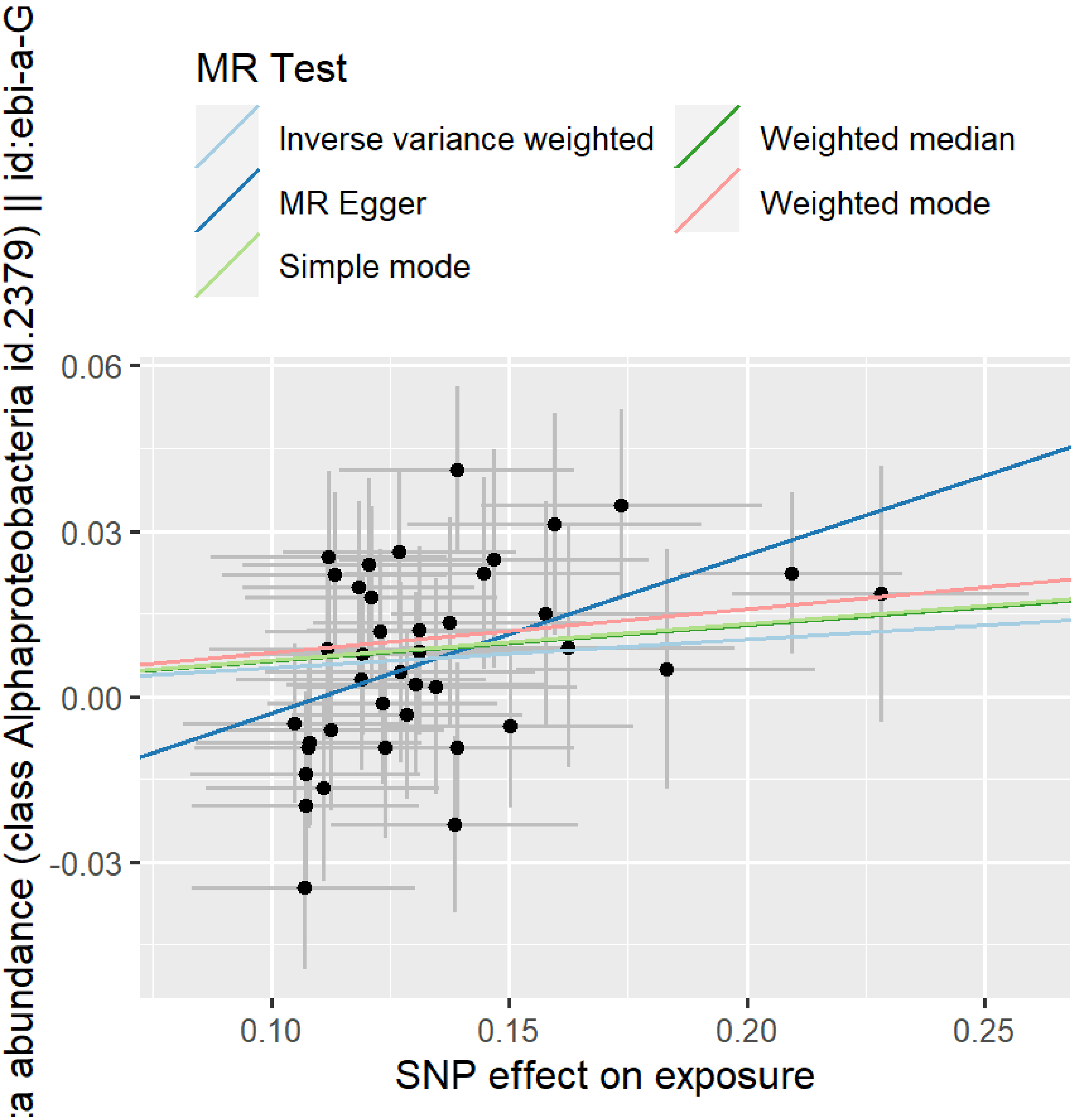
MR Test
Inverse variance weighted
MR Egger
Simple mode
Weighted median
Weighted mode
0.06
0.03
0.00
-0.03
0.10
0.15
0.20
0.25
SNP effect on exposure
:a abundance (class Alphaproteobacteria id.2379) || id:ebi-a-G

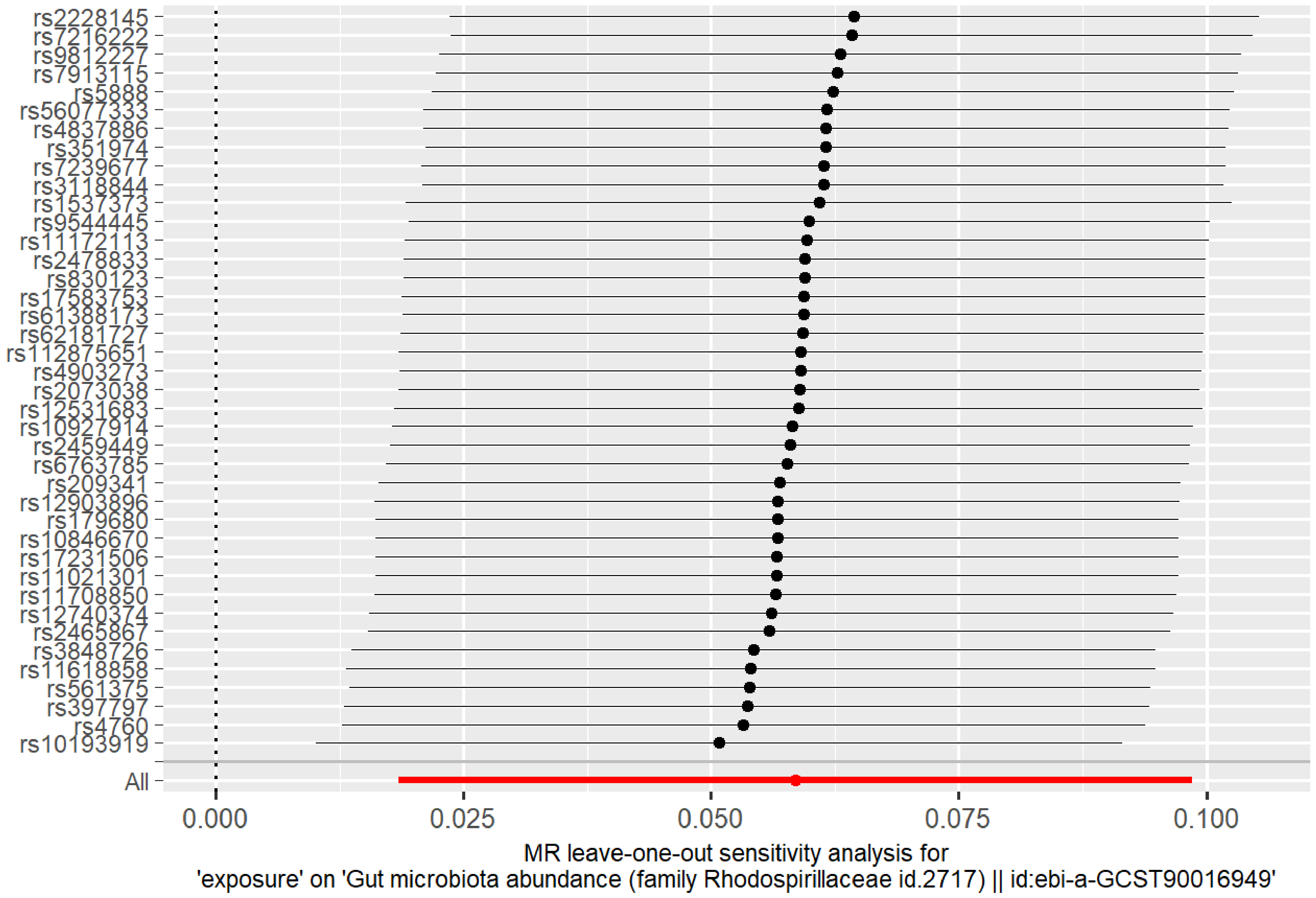
rs2228145
rs7216222
rs9812227
rs7913115
rs5888
rs56077333
rs4837886
rs351974
rs7239677
rs3118844
rs1537373
rs9544445
rs11172113
rs2478833
rs830123
rs17583753
rs61388173
rs62181727
rs112875651
rs4903273
rs2073038
rs12531683
rs10927914
rs2459449
rs6763785
rs209341
rs12903896
rs179680
rs10846670
rs17231506
rs11021301
rs11708850
rs12740374
rs2465867
rs3848726
rs11618858
rs561375
rs397797
rs4760
rs10193919
All
0.000
0.025
0.050
0.075
0.100
MR leave-one-out sensitivity analysis for
'exposure' on 'Gut microbiota abundance (family Rhodospirillaceae id.2717) || id:ebi-a-GCST90016949'

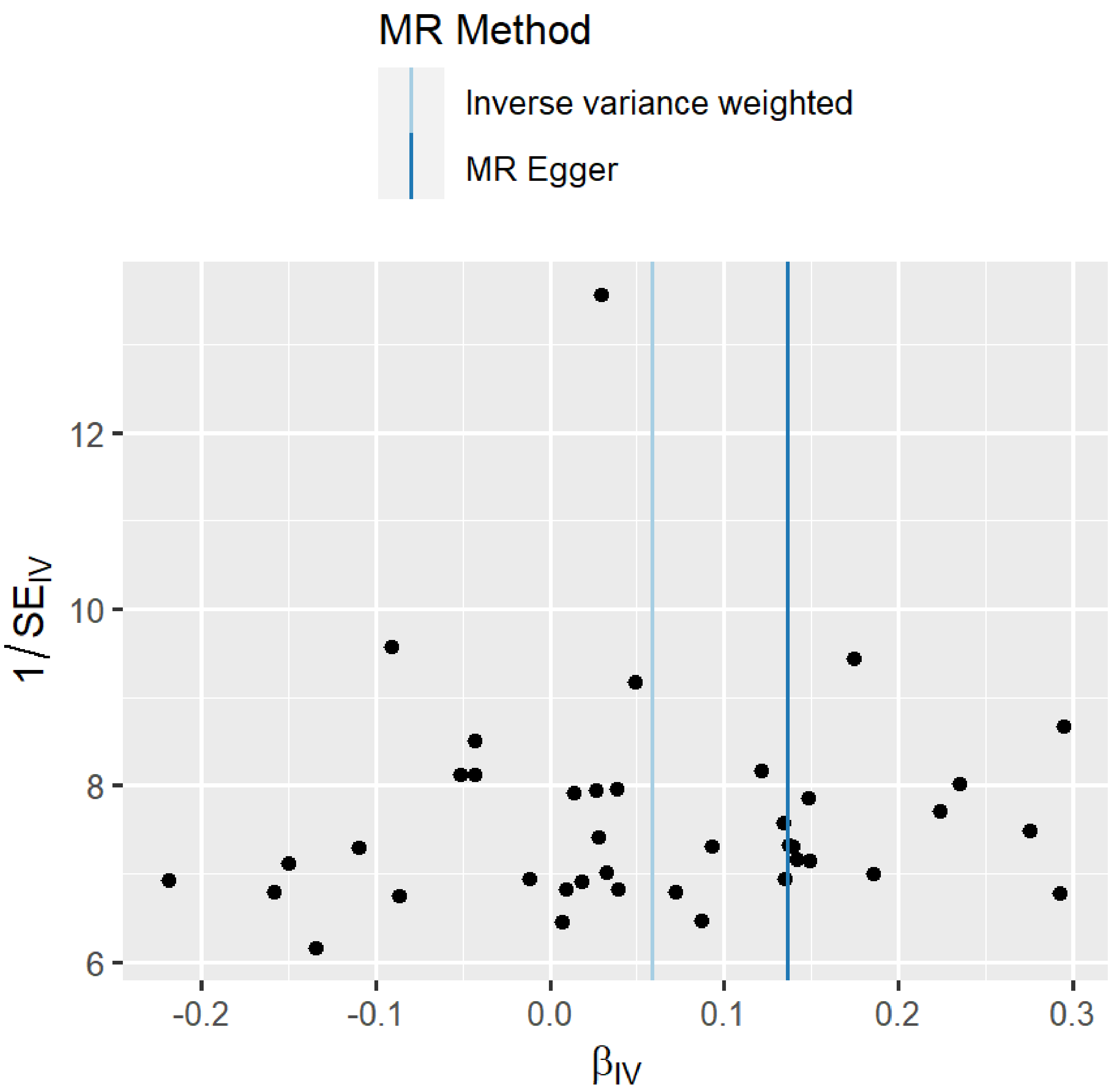
MR Method
Inverse variance weighted
MR Egger
1/SE$_{IV}$
12
10
8
6
-0.2
-0.1
0.0
0.1
0.2
0.3
β$_{IV}$

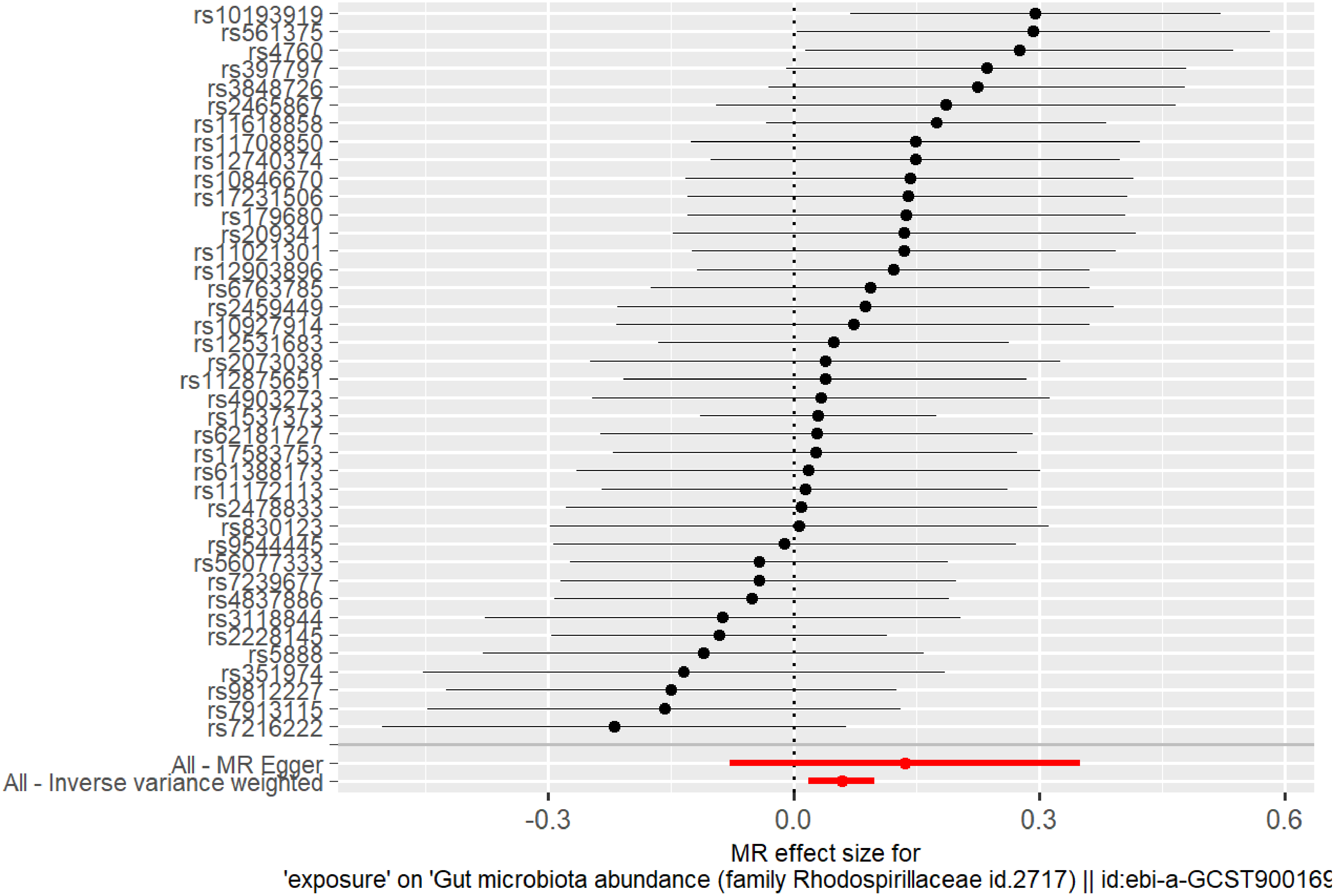
rs10193919
rs561375
rs4760
rs397797
rs3848726
rs2465867
rs11618858
rs11708850
rs12740374
rs10846670
rs17231506
rs179680
rs209341
rs11021301
rs12903896
rs6763785
rs2459449
rs10927914
rs12531683
rs2073038
rs112875651
rs4903273
rs1537373
rs62181727
rs17583753
rs61388173
rs11172113
rs2478833
rs830123
rs9544445
rs56077333
rs7239677
rs4837886
rs3118844
rs2228145
rs5888
rs351974
rs9812227
rs7913115
rs7216222
All - MR Egger
All - Inverse variance weighted
-0.3
0.0
0.3
0.6
MR effect size for
'exposure' on 'Gut microbiota abundance (family Rhodospirillaceae id.2717) || id:ebi-a-GCST90016

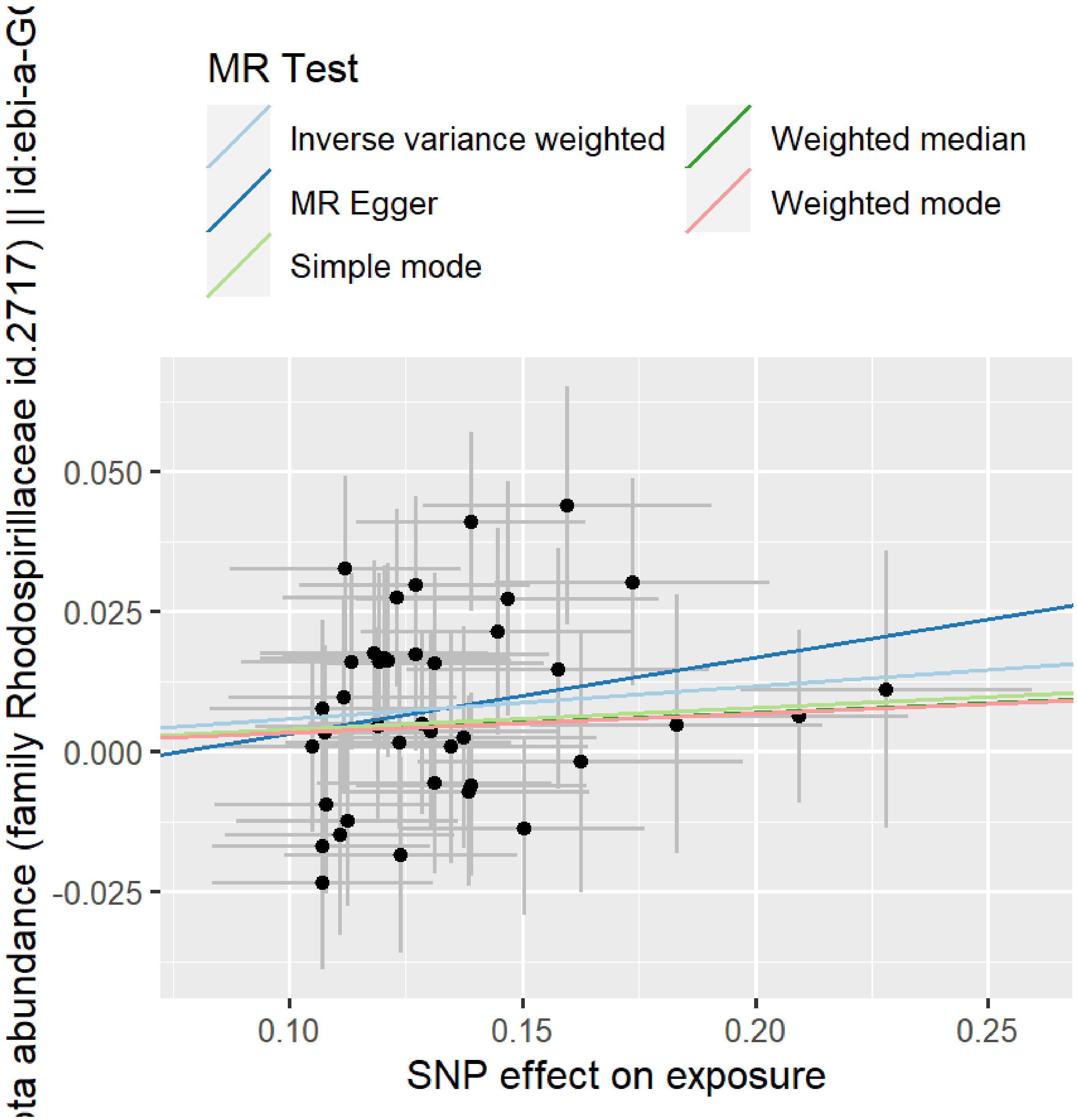
MR Test
Inverse variance weighted
MR Egger
Simple mode
Weighted median
Weighted mode
ota abundance (family Rhodospirillaceae id.2717) || id:ebi-a-GC
0.050
0.025
0.000
-0.025
0.10
0.15
0.20
0.25
SNP effect on exposure

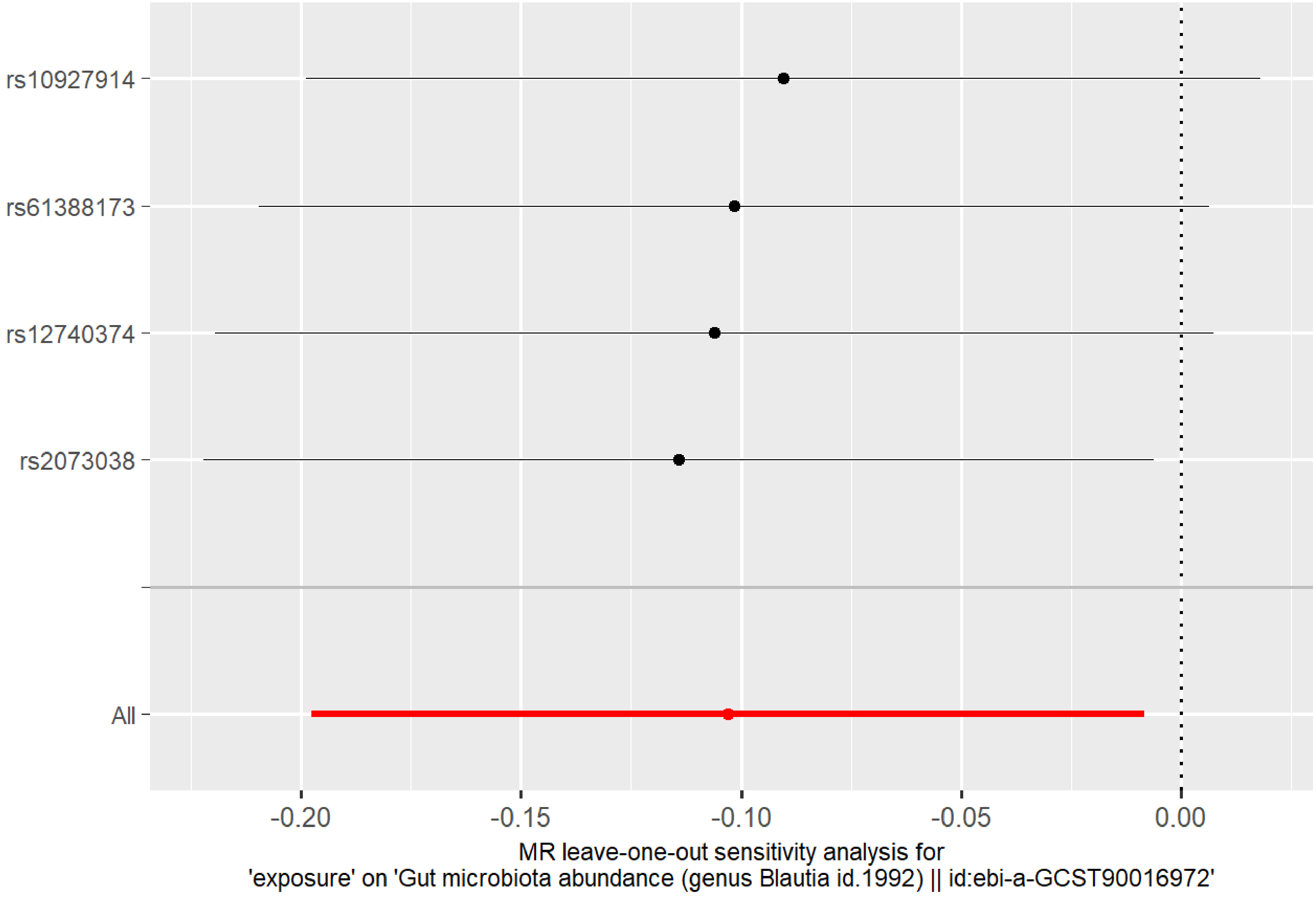
rs10927914
rs61388173
rs12740374
rs2073038
All
-0.20
-0.15
-0.10
-0.05
0.00
MR leave-one-out sensitivity analysis for
'exposure' on 'Gut microbiota abundance (genus Blautia id.1992) || id:ebi-a-GCST90016972'

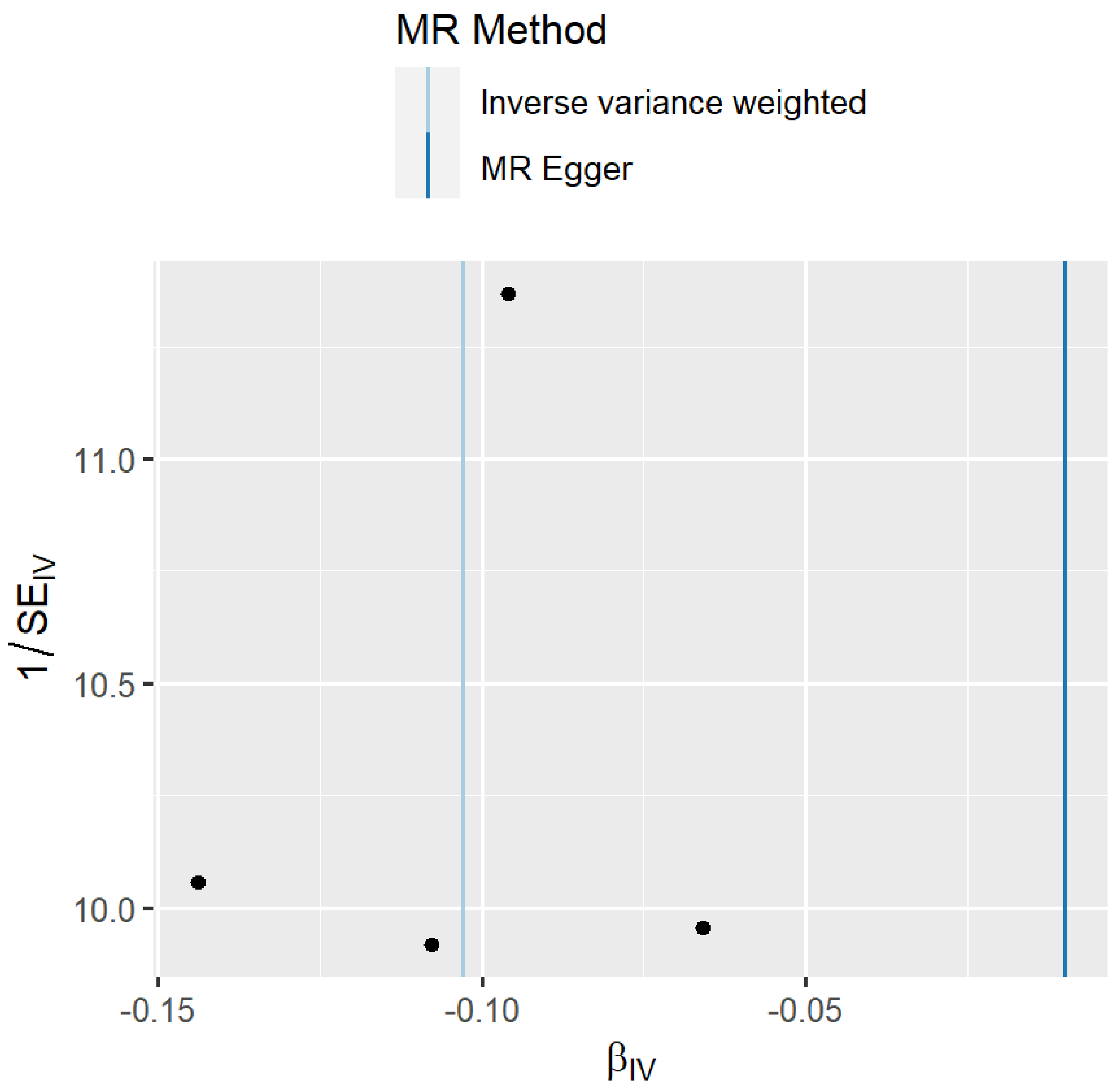

MR Method
Inverse variance weighted
MR Egger
11.0
10.5
10.0
1/SE_IV
-0.15
-0.10
-0.05
β_IV

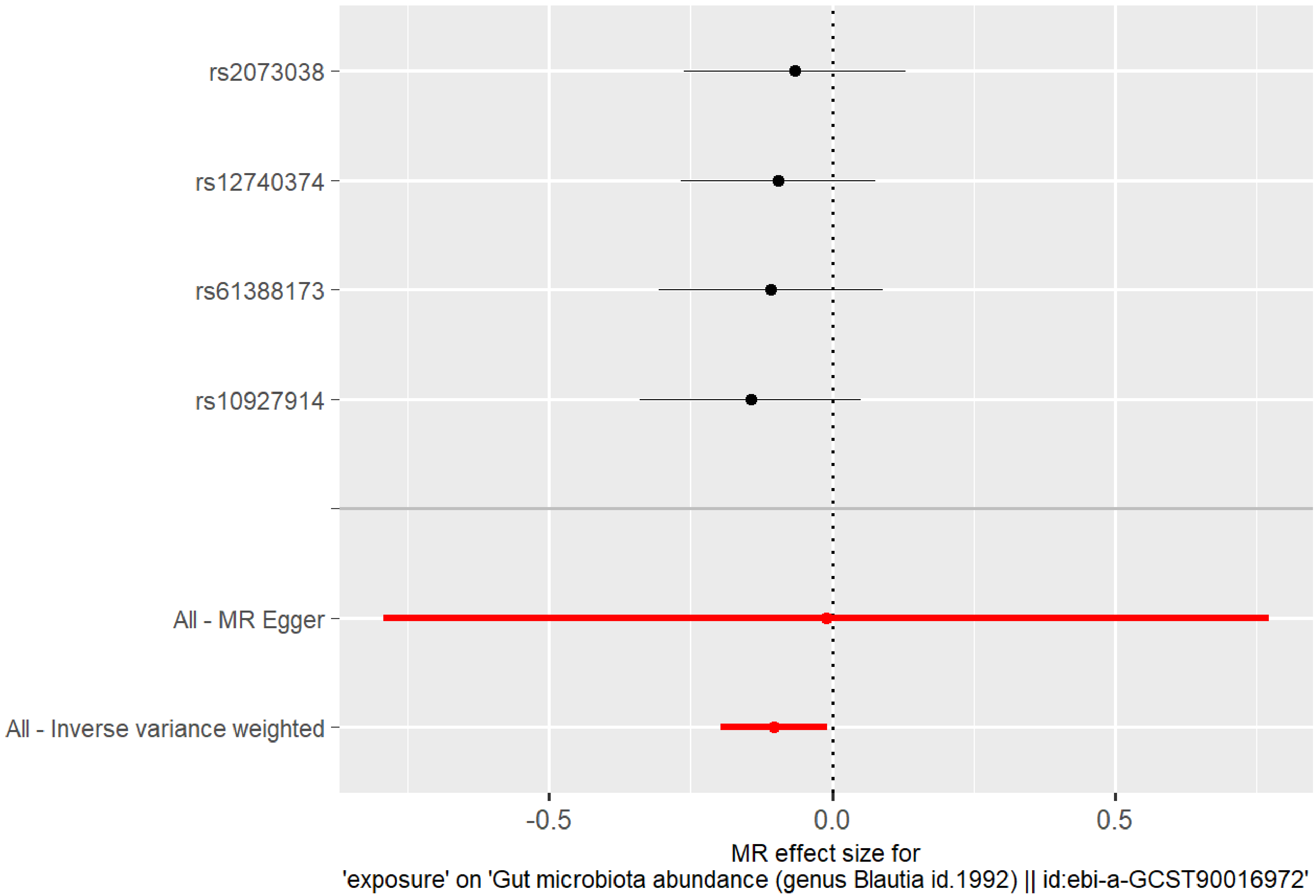

rs2073038
rs12740374
rs61388173
rs10927914
All - MR Egger
All - Inverse variance weighted
-0.5
0.0
0.5
MR effect size for
'exposure' on 'Gut microbiota abundance (genus Blautia id.1992) || id:ebi-a-GCST90016972'

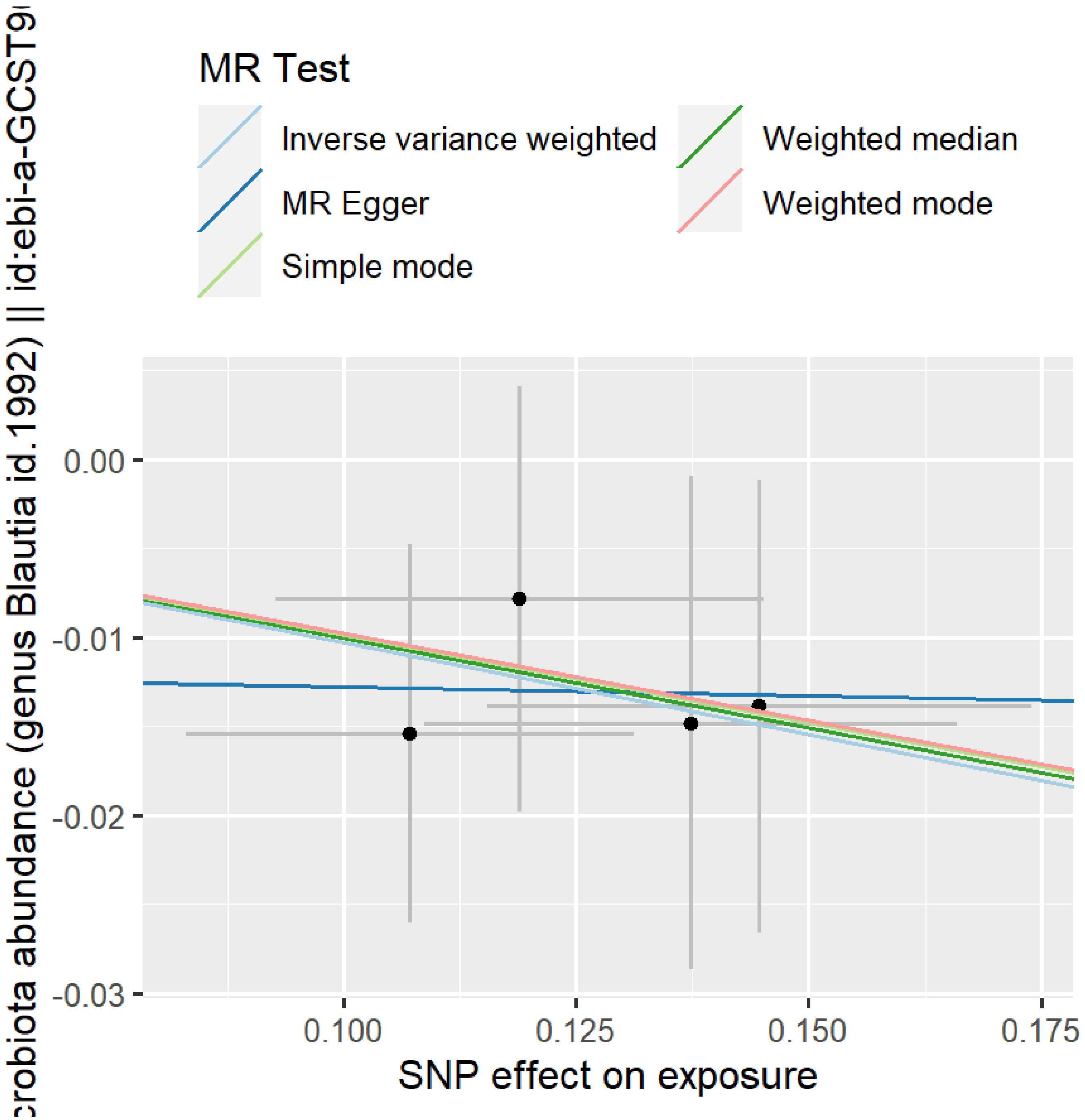
MR Test
Inverse variance weighted
MR Egger
Simple mode
Weighted median
Weighted mode
0.00
-0.01
-0.02
-0.03
0.100
0.125
0.150
0.175
SNP effect on exposure
crobiota abundance (genus Blautia id.1992) || id:ebi-a-GCST9

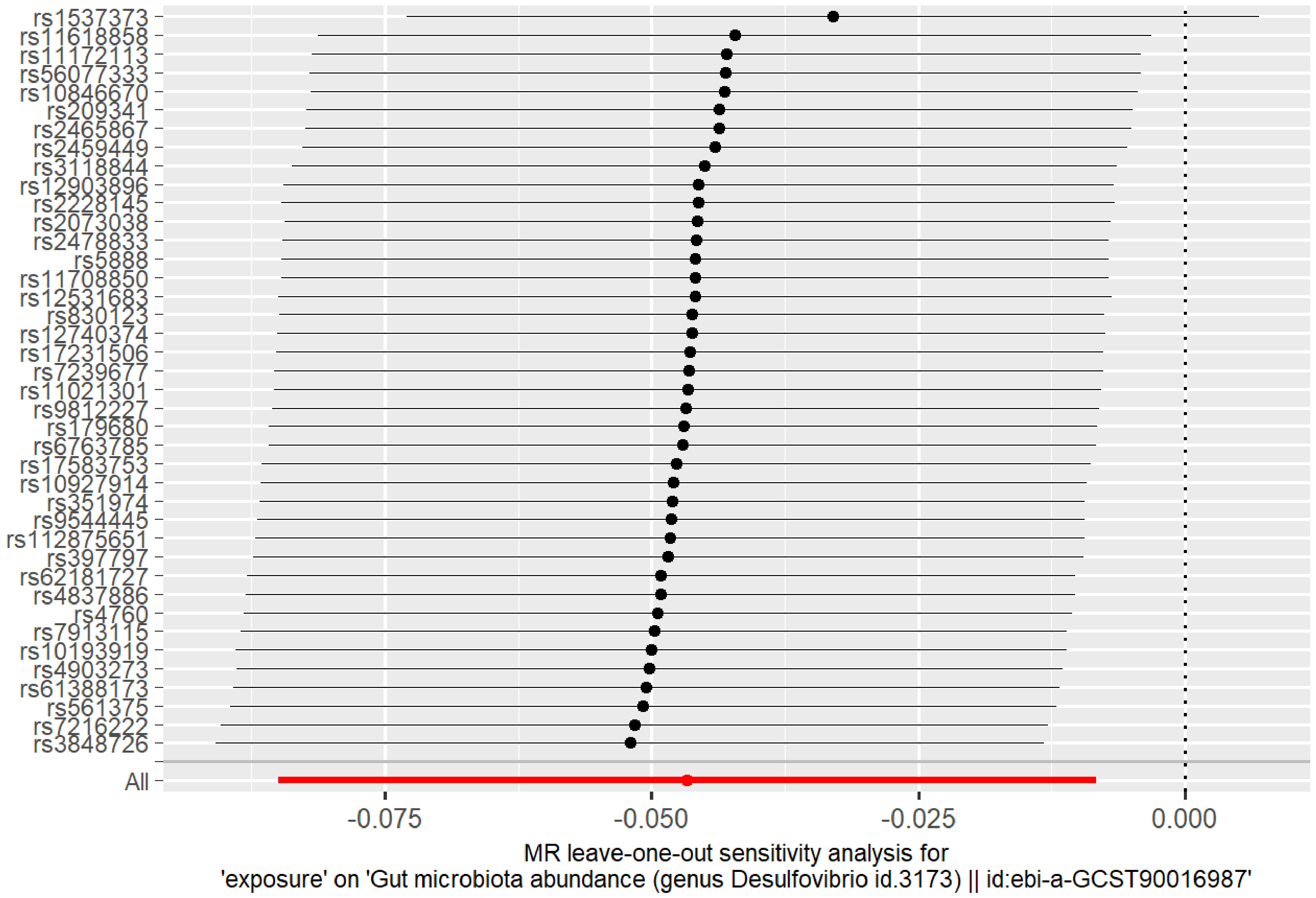
rs1537373
rs11618858
rs11172113
rs56077333
rs10846670
rs209341
rs2465867
rs2459449
rs3118844
rs12903896
rs2228145
rs2073038
rs2478833
rs5888
rs11708850
rs12531683
rs830123
rs12740374
rs17231506
rs7239677
rs11021301
rs9812227
rs179680
rs6763785
rs17583753
rs10927914
rs351974
rs9544445
rs112875651
rs397797
rs62181727
rs4837886
rs4760
rs7913115
rs10193919
rs4903273
rs61388173
rs561375
rs7216222
rs3848726
All
-0.075
-0.050
-0.025
0.000
MR leave-one-out sensitivity analysis for
'exposure' on 'Gut microbiota abundance (genus Desulfovibrio id.3173) || id:ebi-a-GCST90016987'

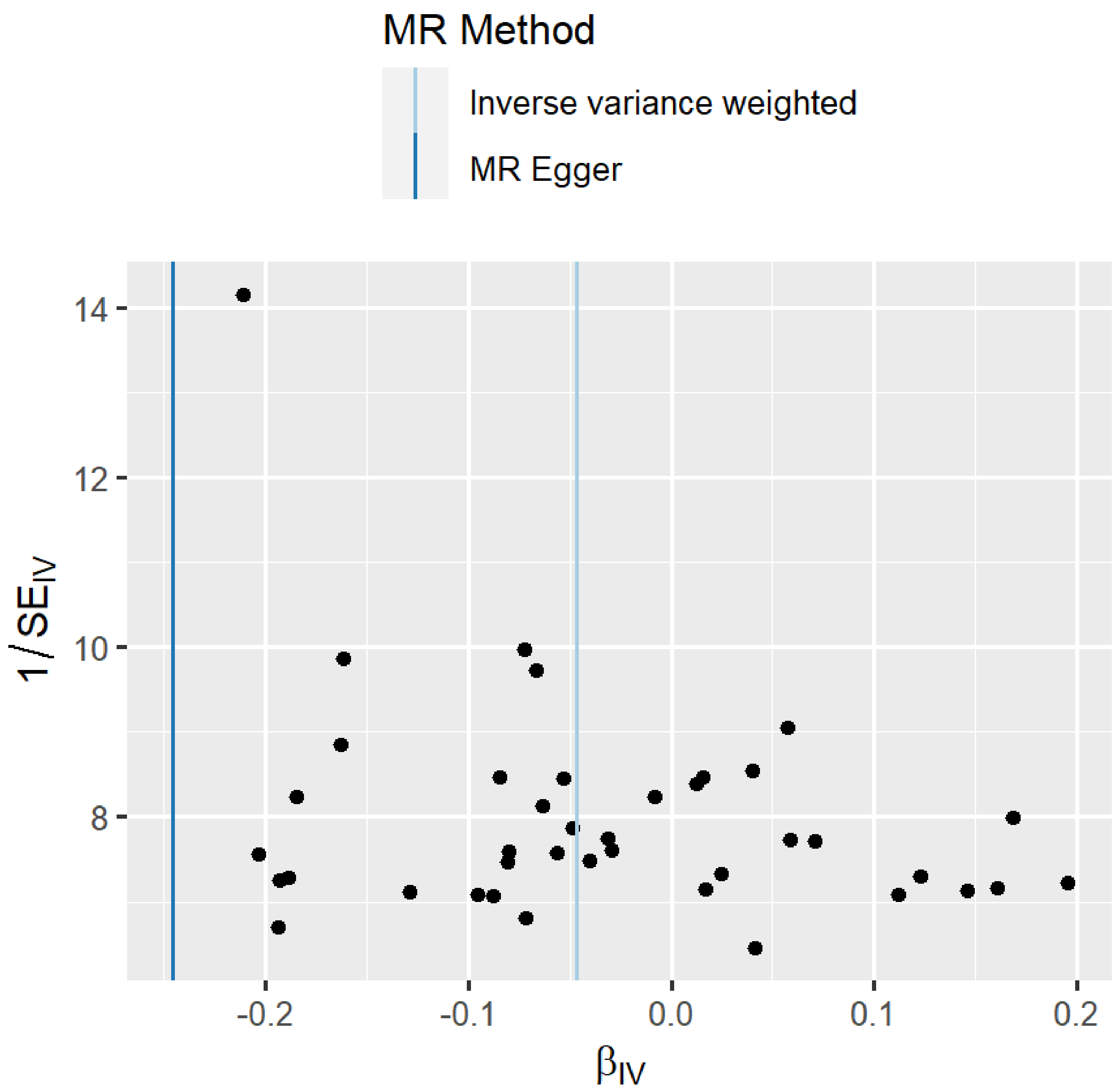
MR Method
Inverse variance weighted
MR Egger
14
12
10
8
1/SE_IV
-0.2
-0.1
0.0
0.1
0.2
β_IV

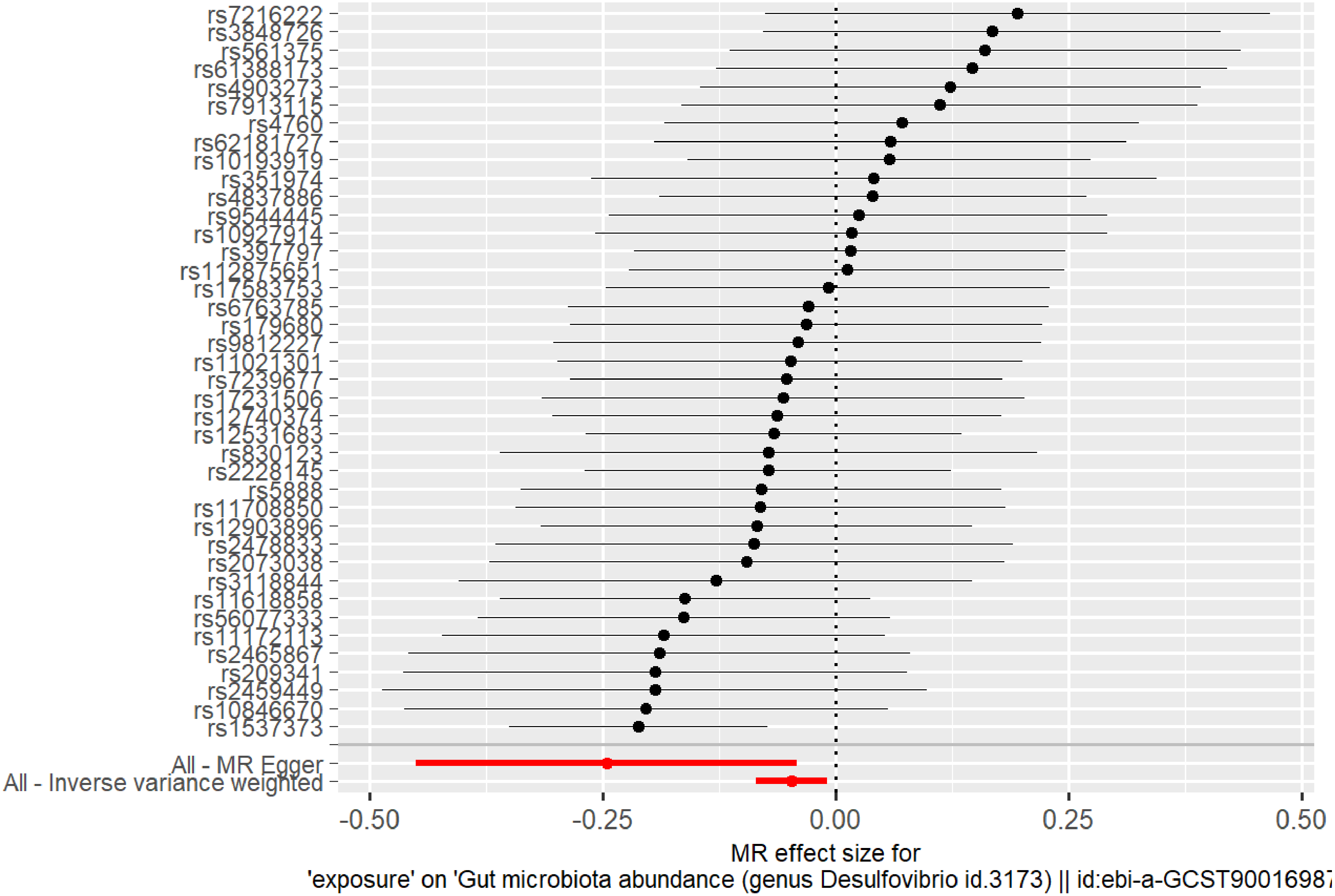
rs7216222
rs3848726
rs561375
rs61388173
rs4903273
rs7913115
rs4760
rs62181727
rs10193919
rs351974
rs4837886
rs9544445
rs10927914
rs397797
rs112875651
rs17583753
rs6763785
rs179680
rs9812227
rs11021301
rs7239677
rs17231506
rs12740374
rs12531683
rs830123
rs2228145
rs5888
rs11708850
rs12903896
rs2478833
rs2073038
rs3118844
rs11618858
rs56077333
rs11172113
rs2465867
rs209341
rs2459449
rs10846670
rs1537373
All - MR Egger
All - Inverse variance weighted
-0.50
-0.25
0.00
0.25
0.50
MR effect size for
'exposure' on 'Gut microbiota abundance (genus Desulfovibrio id.3173) || id:ebi-a-GCST9001698

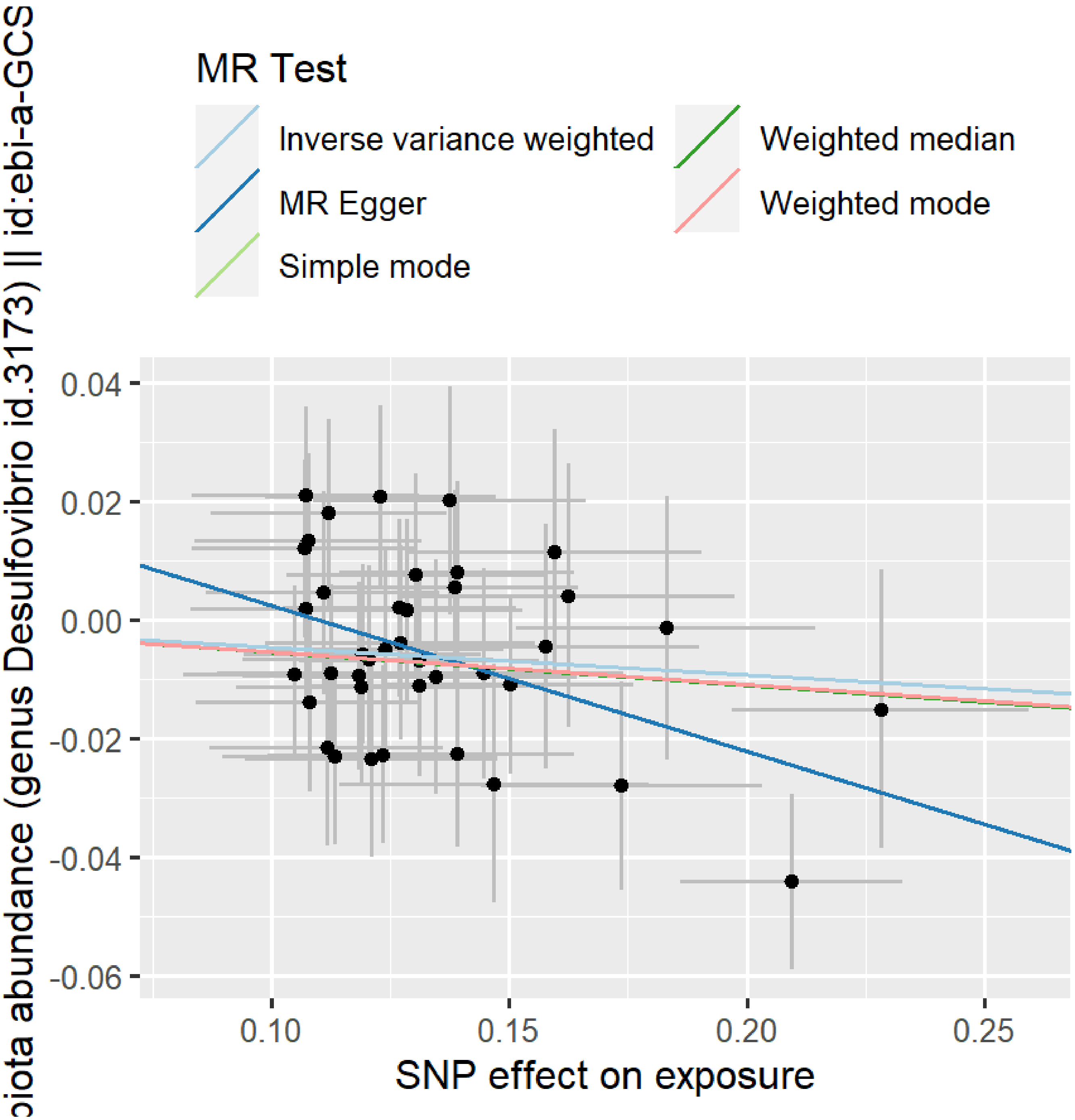

MR Test
Inverse variance weighted
MR Egger
Simple mode
Weighted median
Weighted mode
0.04
0.02
0.00
-0.02
-0.04
-0.06
0.10
0.15
0.20
0.25
SNP effect on exposure
biota abundance (genus Desulfovibrio id.3173) || id:ebi-a-GCS

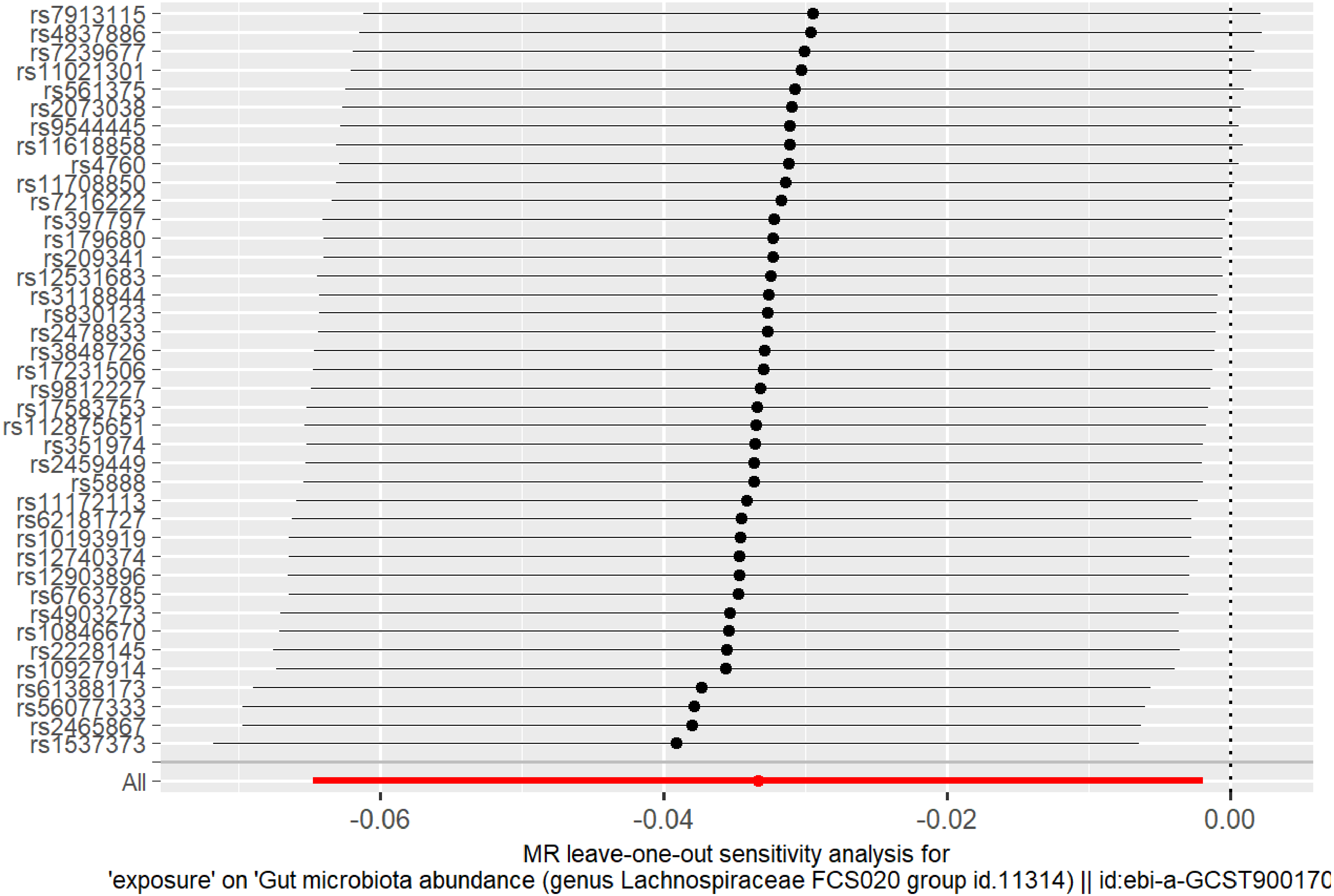
rs7913115
rs4837886
rs7239677
rs11021301
rs561375
rs2073038
rs9544445
rs11618858
rs4760
rs11708850
rs7216222
rs397797
rs179680
rs209341
rs12531683
rs3118844
rs830123
rs2478833
rs3848726
rs17231506
rs9812227
rs17583753
rs112875651
rs351974
rs2459449
rs5888
rs11172113
rs62181727
rs10193919
rs12740374
rs12903896
rs6763785
rs4903273
rs10846670
rs2228145
rs10927914
rs61388173
rs56077333
rs2465867
rs1537373
All
-0.06
-0.04
-0.02
0.00
MR leave-one-out sensitivity analysis for
'exposure' on 'Gut microbiota abundance (genus Lachnospiraceae FCS020 group id.11314) || id:ebi-a-GCST90017C

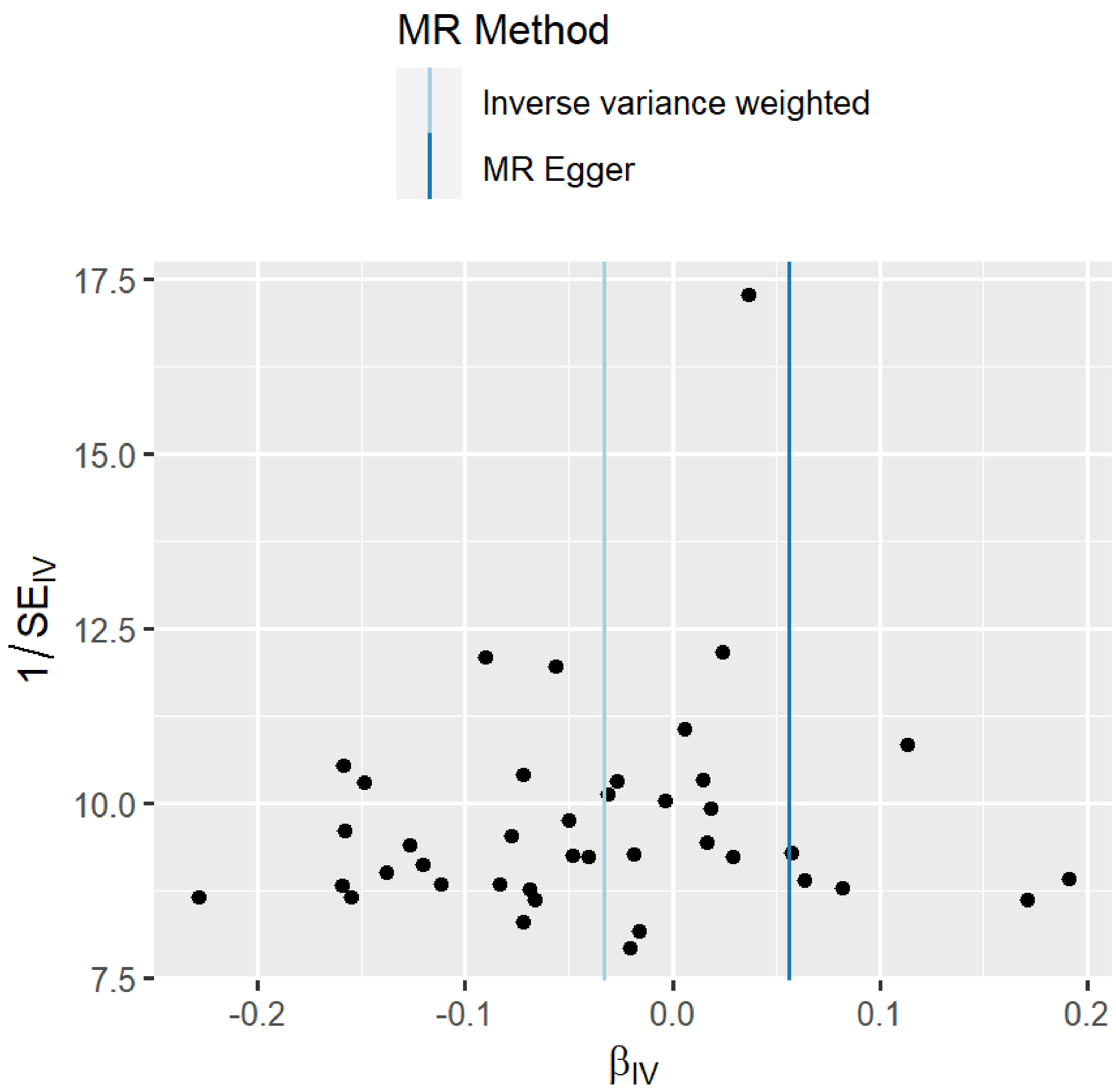
MR Method
Inverse variance weighted
MR Egger
17.5
15.0
12.5
10.0
7.5
1/SE_IV
-0.2
-0.1
0.0
0.1
0.2
β_IV

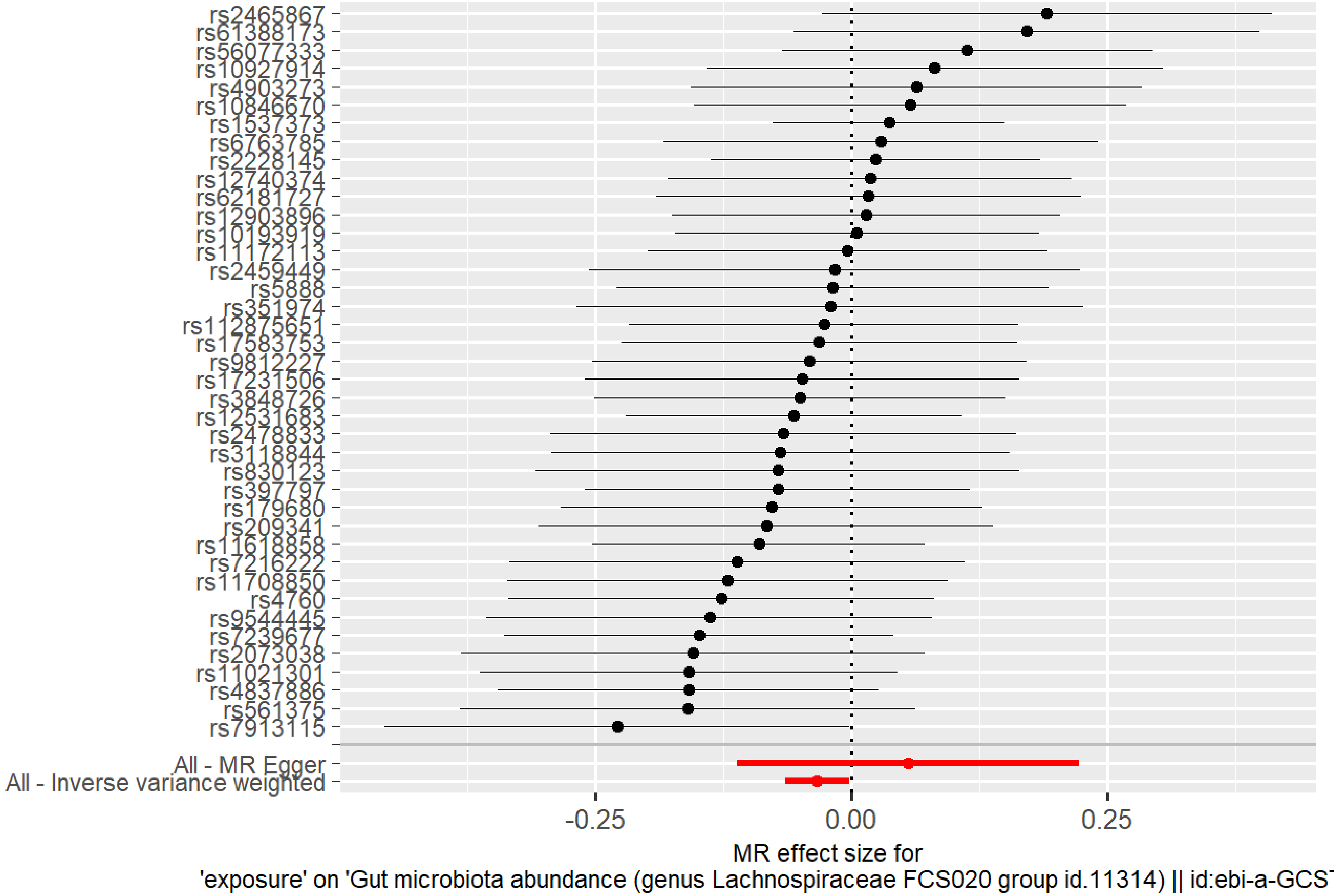
rs2465867
rs61388173
rs56077333
rs10927914
rs4903273
rs10846670
rs1537373
rs6763785
rs2228145
rs12740374
rs62181727
rs12903896
rs10193919
rs11172113
rs2459449
rs5888
rs351974
rs112875651
rs17583753
rs9812227
rs17231506
rs3848726
rs12531683
rs2478833
rs3118844
rs830123
rs397797
rs179680
rs209341
rs11618858
rs7216222
rs11708850
rs4760
rs9544445
rs7239677
rs2073038
rs11021301
rs4837886
rs561375
rs7913115
All - MR Egger
All - Inverse variance weighted
-0.25
0.00
0.25
MR effect size for
'exposure' on 'Gut microbiota abundance (genus Lachnospiraceae FCS020 group id.11314) || id:ebi-a-GCS

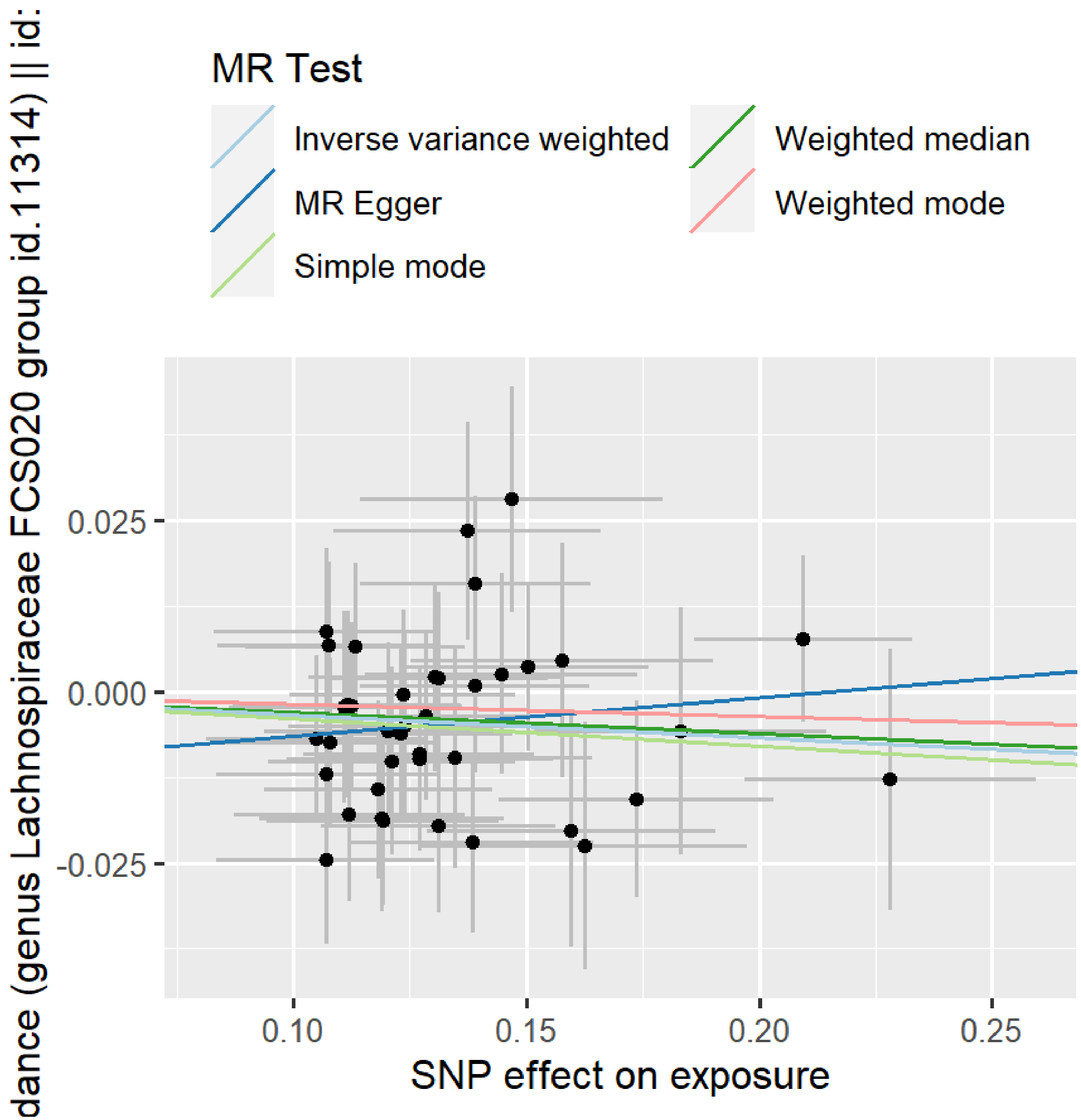
MR Test
Inverse variance weighted
MR Egger
Simple mode
Weighted median
Weighted mode
dance (genus Lachnospiraceae FCS020 group id.11314) || id:
0.025
0.000
-0.025
0.10
0.15
0.20
0.25
SNP effect on exposure

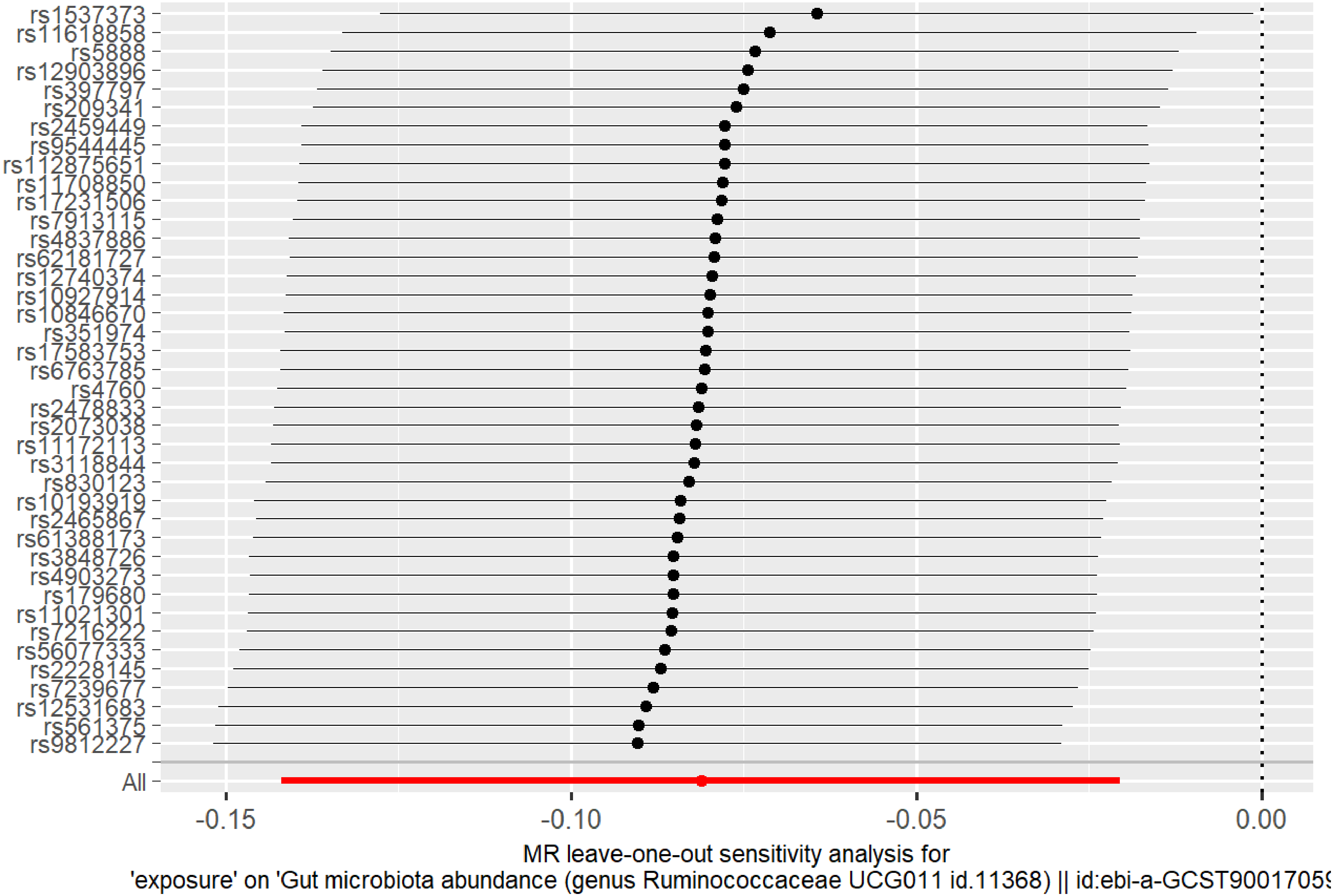

rs1537373
rs11618858
rs5888
rs12903896
rs397797
rs209341
rs2459449
rs9544445
rs112875651
rs11708850
rs17231506
rs7913115
rs4837886
rs62181727
rs12740374
rs10927914
rs10846670
rs351974
rs17583753
rs6763785
rs4760
rs2478833
rs2073038
rs11172113
rs3118844
rs830123
rs10193919
rs2465867
rs61388173
rs3848726
rs4903273
rs179680
rs11021301
rs7216222
rs56077333
rs2228145
rs7239677
rs12531683
rs561375
rs9812227
All
-0.15
-0.10
-0.05
0.00
MR leave-one-out sensitivity analysis for
'exposure' on 'Gut microbiota abundance (genus Ruminococcaceae UCG011 id.11368) || id:ebi-a-GCST9001705

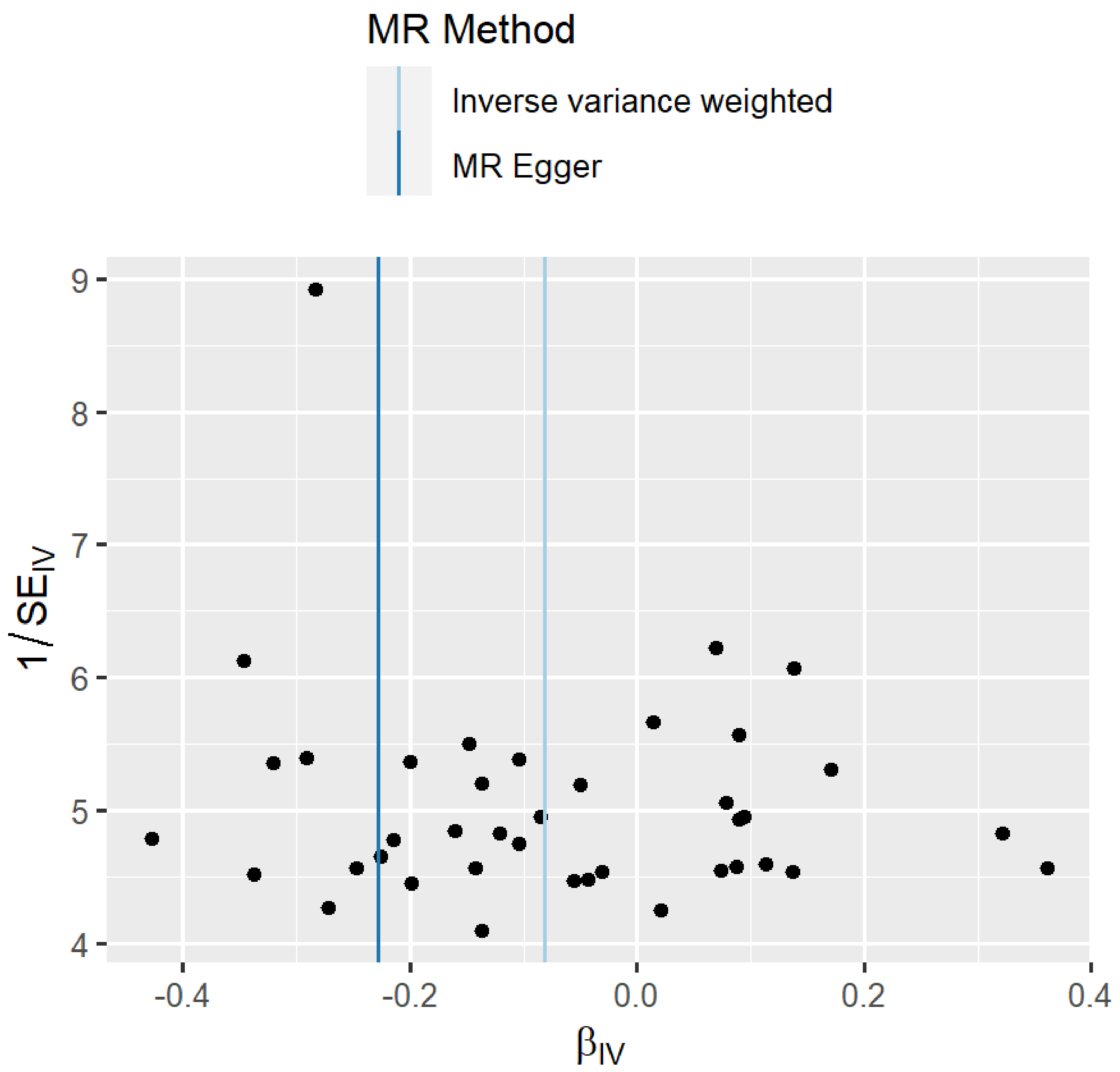
MR Method
Inverse variance weighted
MR Egger
9
8
7
6
5
4
$1/SE_{IV}$
-0.4
-0.2
0.0
0.2
0.4
β_{IV}

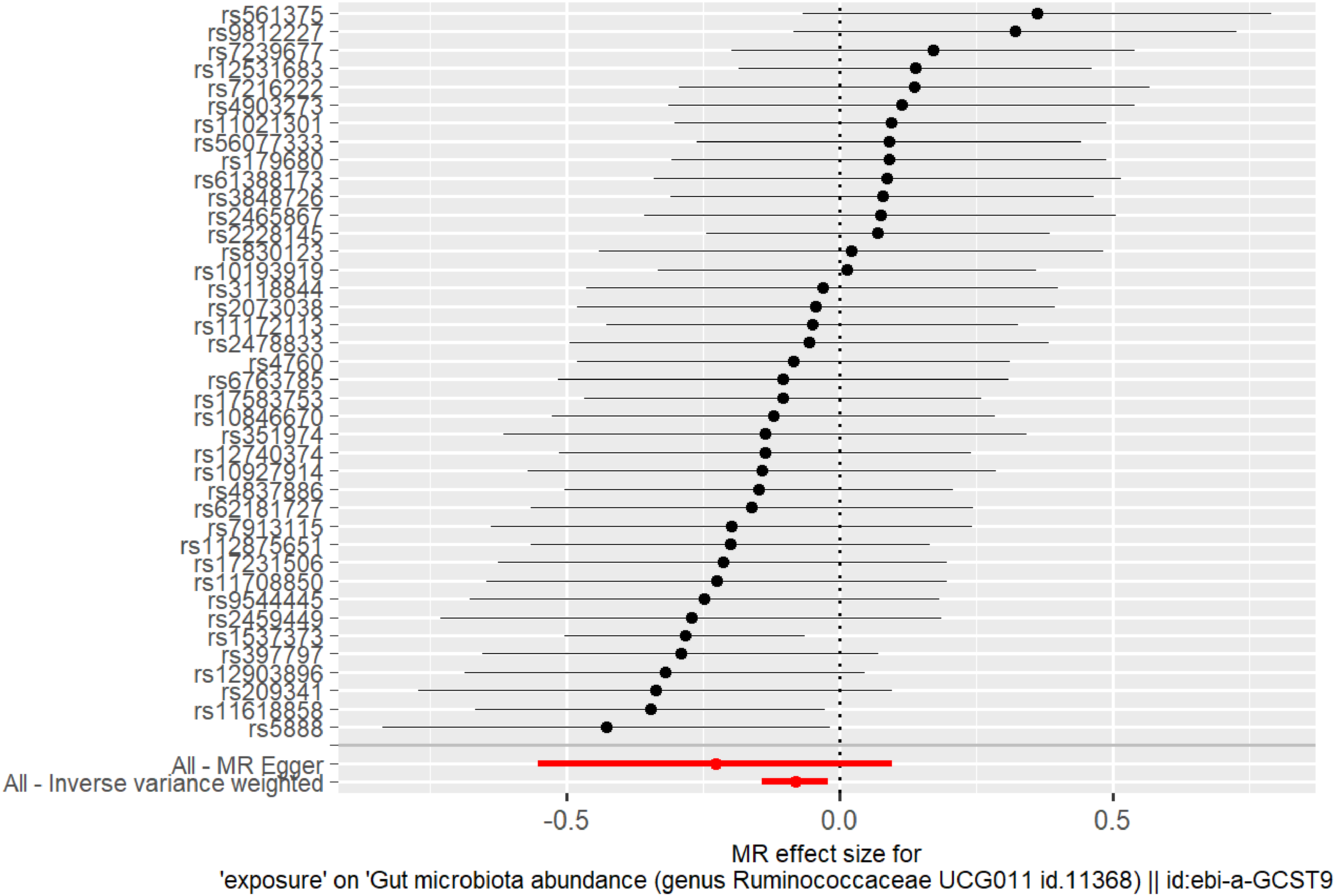
rs561375
rs9812227
rs7239677
rs12531683
rs7216222
rs4903273
rs11021301
rs56077333
rs179680
rs61388173
rs3848726
rs2465867
rs2228145
rs830123
rs10193919
rs3118844
rs2073038
rs11172113
rs2478833
rs4760
rs6763785
rs17583753
rs10846670
rs351974
rs12740374
rs10927914
rs4837886
rs62181727
rs7913115
rs112875651
rs17231506
rs11708850
rs9544445
rs2459449
rs1537373
rs397797
rs12903896
rs209341
rs11618858
rs5888
All - MR Egger
All - Inverse variance weighted
-0.5
0.0
0.5
MR effect size for
'exposure' on 'Gut microbiota abundance (genus Ruminococcaceae UCG011 id.11368) || id:ebi-a-GCST9

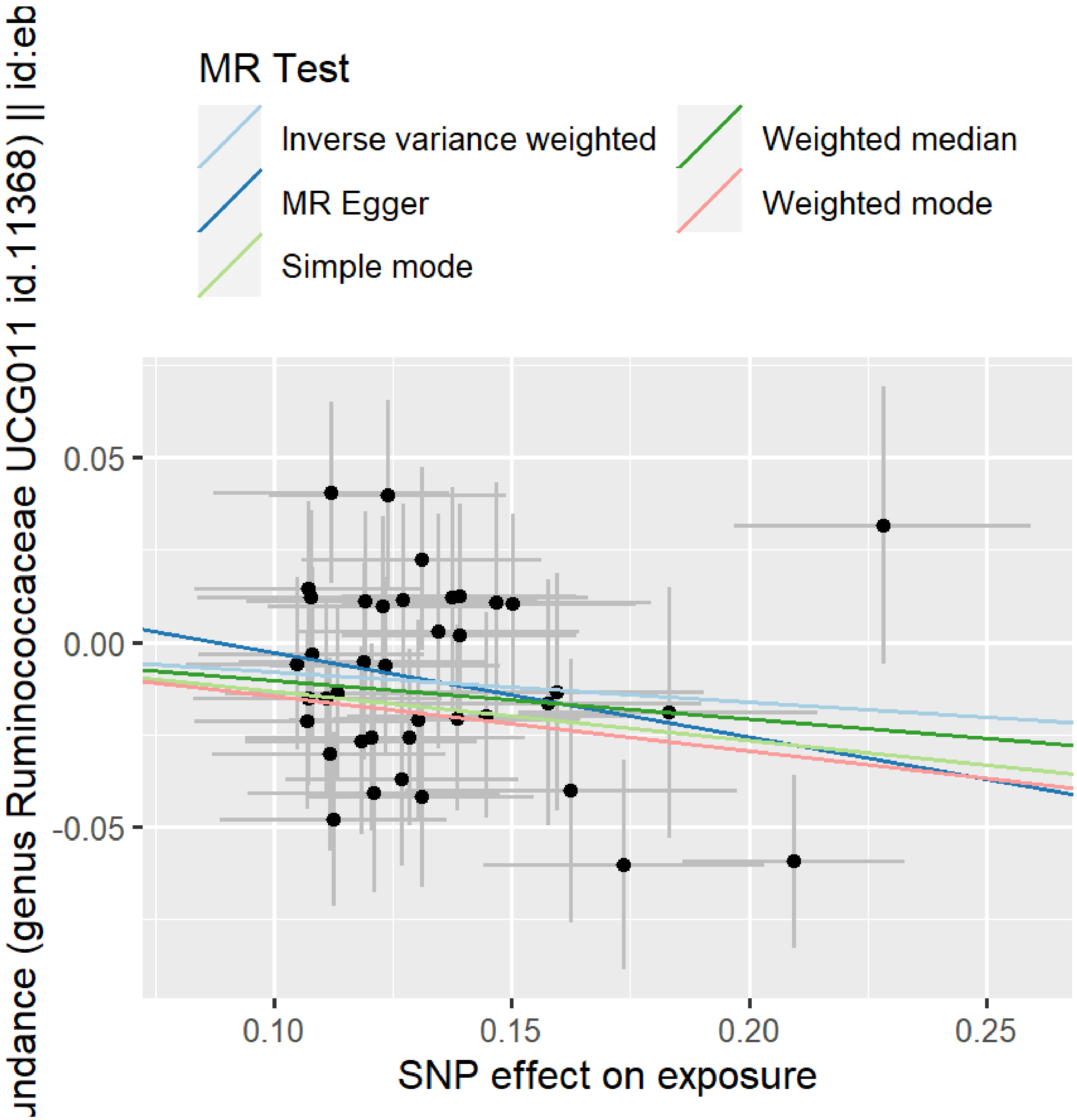
MR Test
Inverse variance weighted
MR Egger
Simple mode
Weighted median
Weighted mode
0.05
0.00
-0.05
0.10
0.15
0.20
0.25
SNP effect on exposure
undance (genus Ruminococcaceae UCG011 id.11368) || id:eb

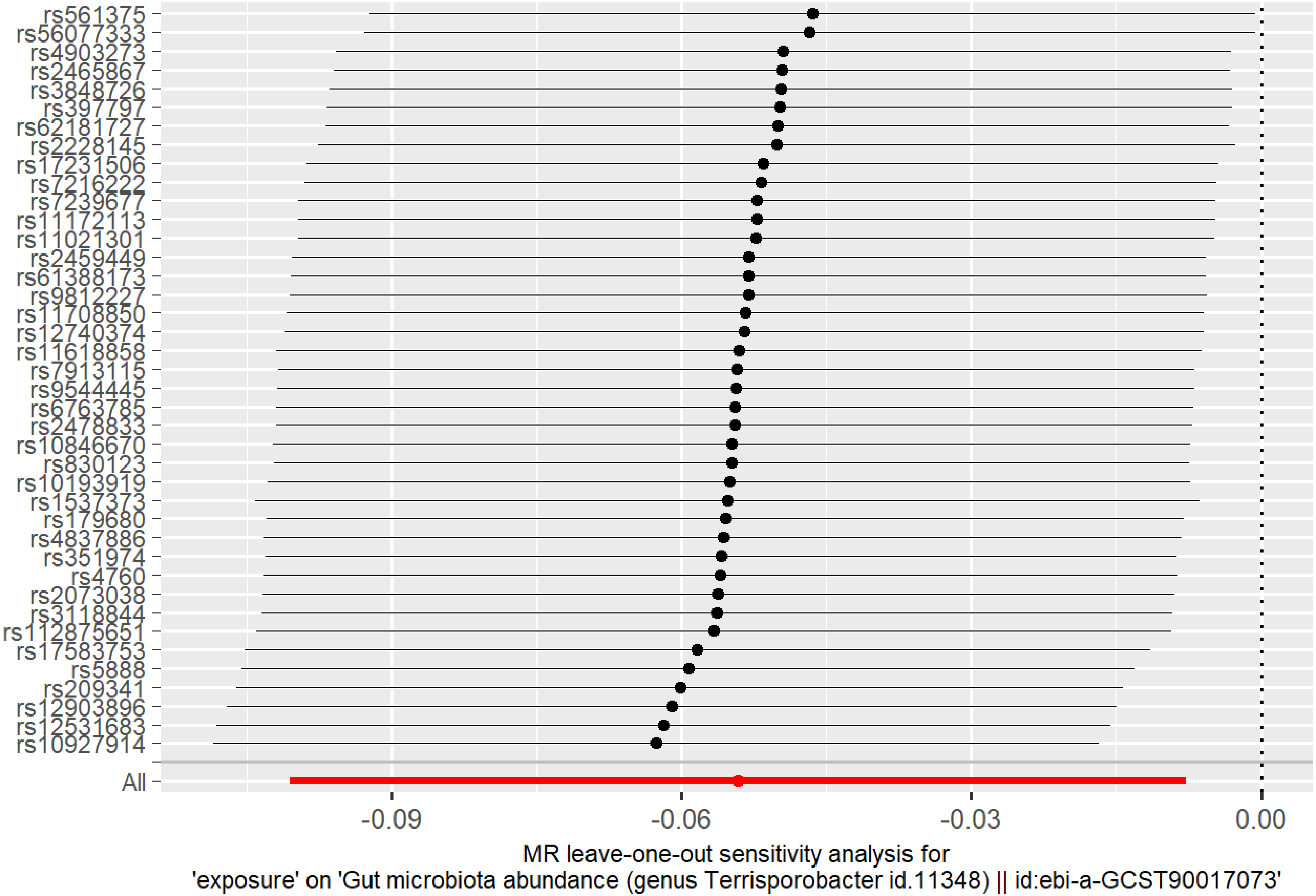
rs561375
rs56077333
rs4903273
rs2465867
rs3848726
rs397797
rs62181727
rs2228145
rs17231506
rs7216222
rs7239677
rs11172113
rs11021301
rs2459449
rs61388173
rs9812227
rs11708850
rs12740374
rs11618858
rs7913115
rs9544445
rs6763785
rs2478833
rs10846670
rs830123
rs10193919
rs1537373
rs179680
rs4837886
rs351974
rs4760
rs2073038
rs3118844
rs112875651
rs17583753
rs5888
rs209341
rs12903896
rs12531683
rs10927914
All
-0.09
-0.06
-0.03
0.00
MR leave-one-out sensitivity analysis for
'exposure' on 'Gut microbiota abundance (genus Terrisporobacter id.11348) || id:ebi-a-GCST90017073'

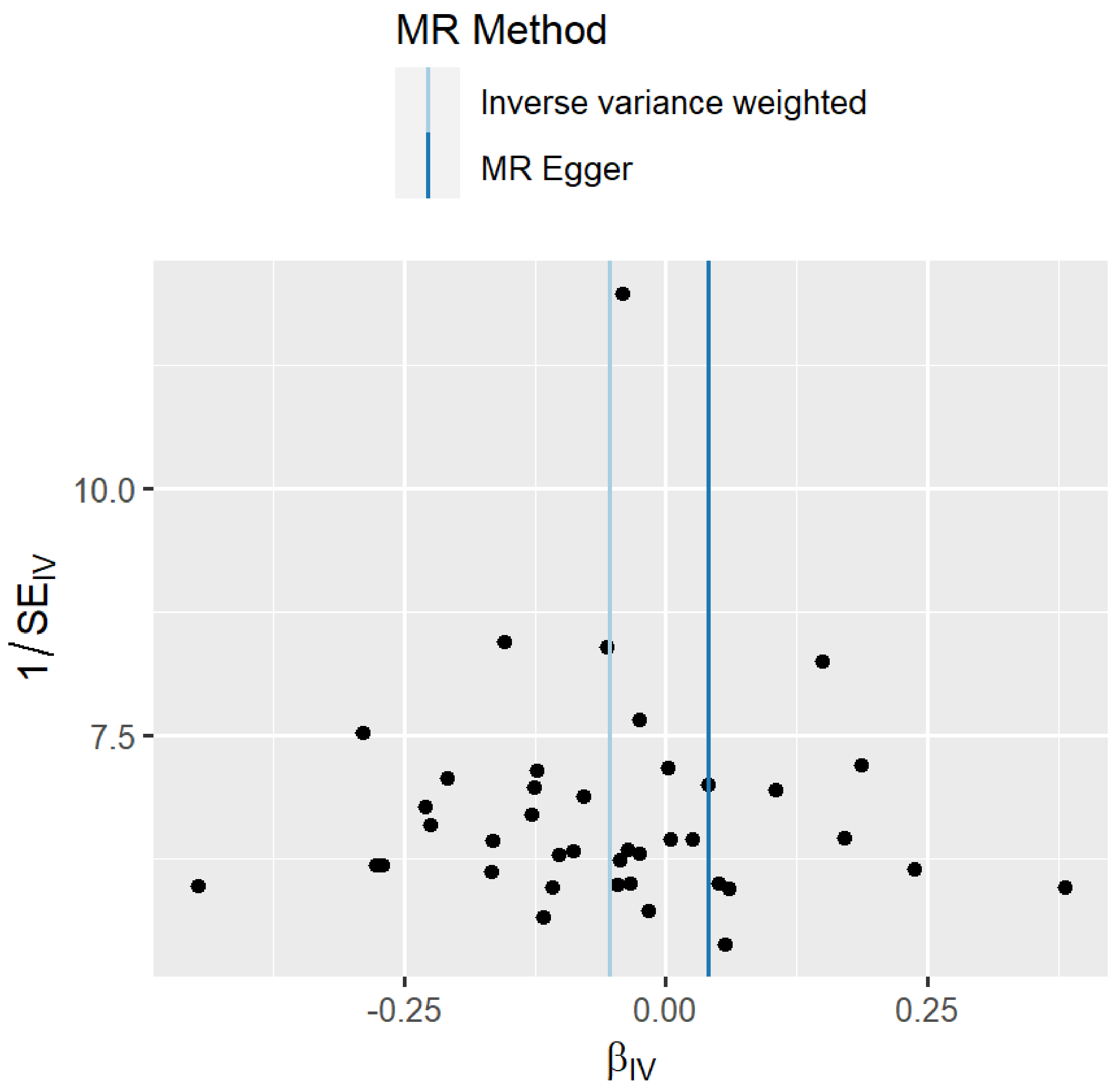
MR Method
Inverse variance weighted
MR Egger
10.0
7.5
-0.25
0.00
0.25
1/SE_IV
β_IV

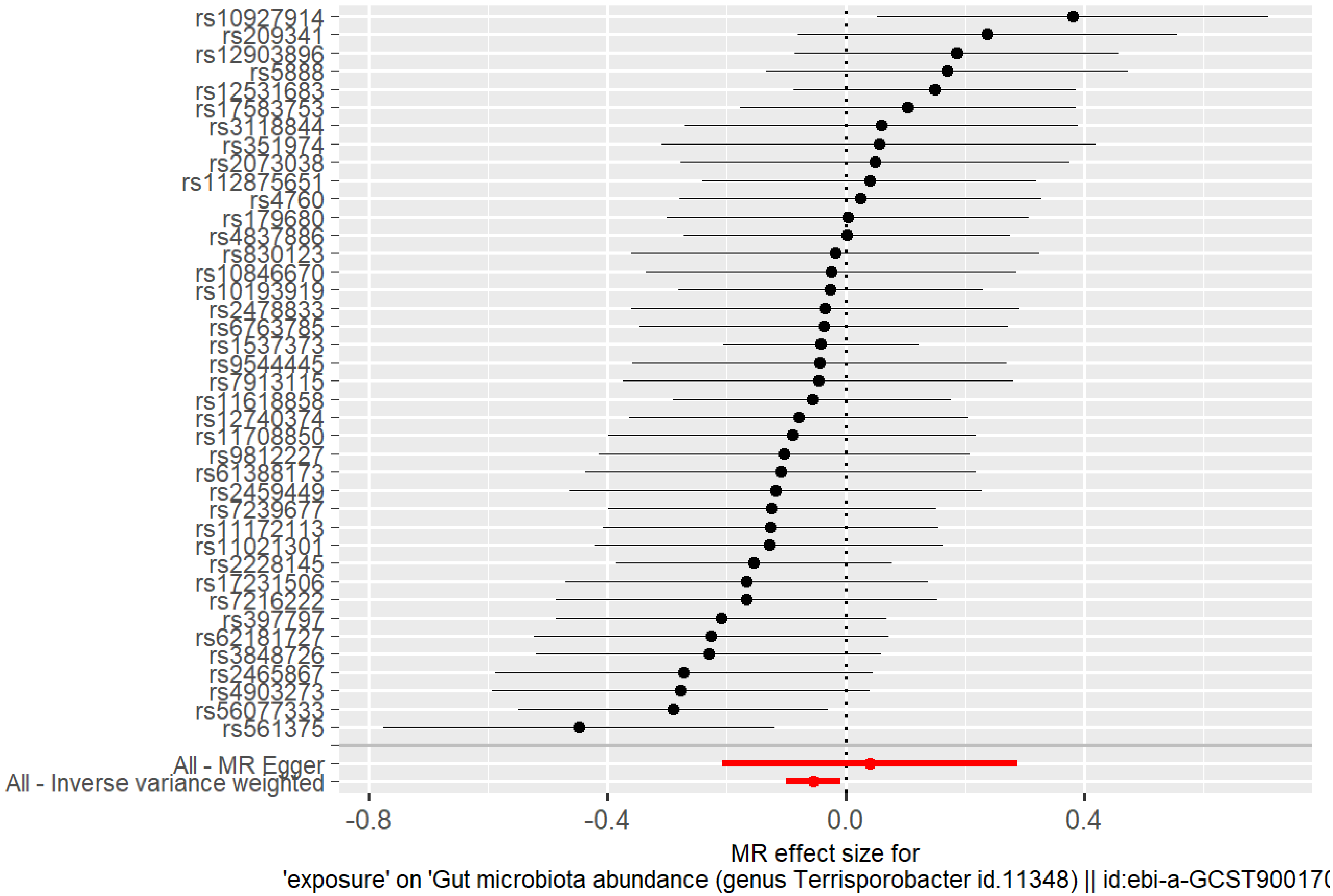
rs10927914
rs209341
rs12903896
rs5888
rs12531683
rs17583753
rs3118844
rs351974
rs2073038
rs112875651
rs4760
rs179680
rs4837886
rs830123
rs10846670
rs10193919
rs2478833
rs6763785
rs1537373
rs9544445
rs7913115
rs11618858
rs12740374
rs11708850
rs9812227
rs61388173
rs2459449
rs7239677
rs11172113
rs11021301
rs2228145
rs17231506
rs7216222
rs397797
rs62181727
rs3848726
rs2465867
rs4903273
rs56077333
rs561375
All - MR Egger
All - Inverse variance weighted
-0.8
-0.4
0.0
0.4
MR effect size for
'exposure' on 'Gut microbiota abundance (genus Terrisporobacter id.11348) || id:ebi-a-GCST90017(

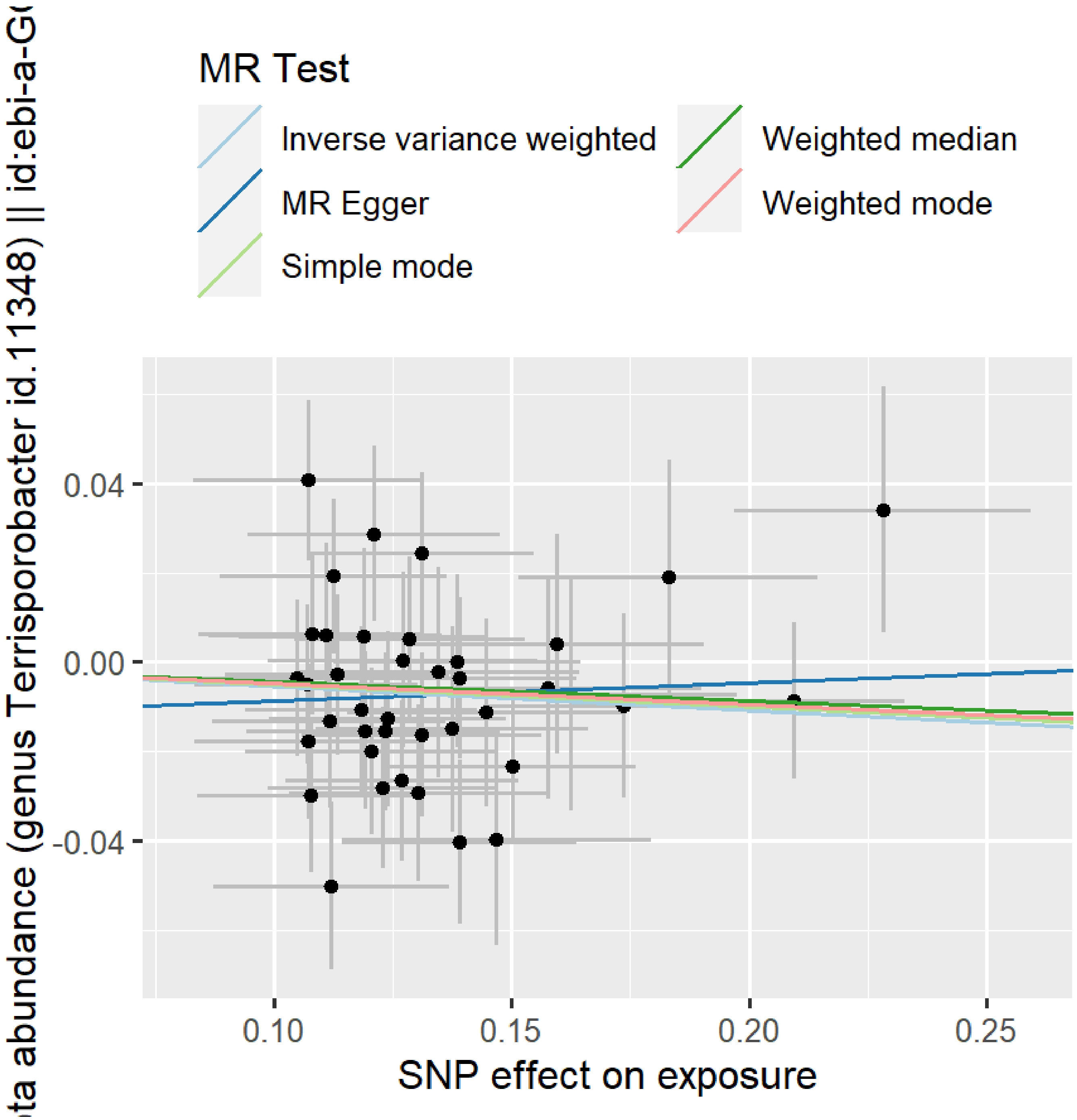

MR Test
Inverse variance weighted
MR Egger
Simple mode
Weighted median
Weighted mode
0.04
0.00
-0.04
0.10
0.15
0.20
0.25
SNP effect on exposure
ta abundance (genus Terrisporobacter id.11348) || id:ebi-a-G

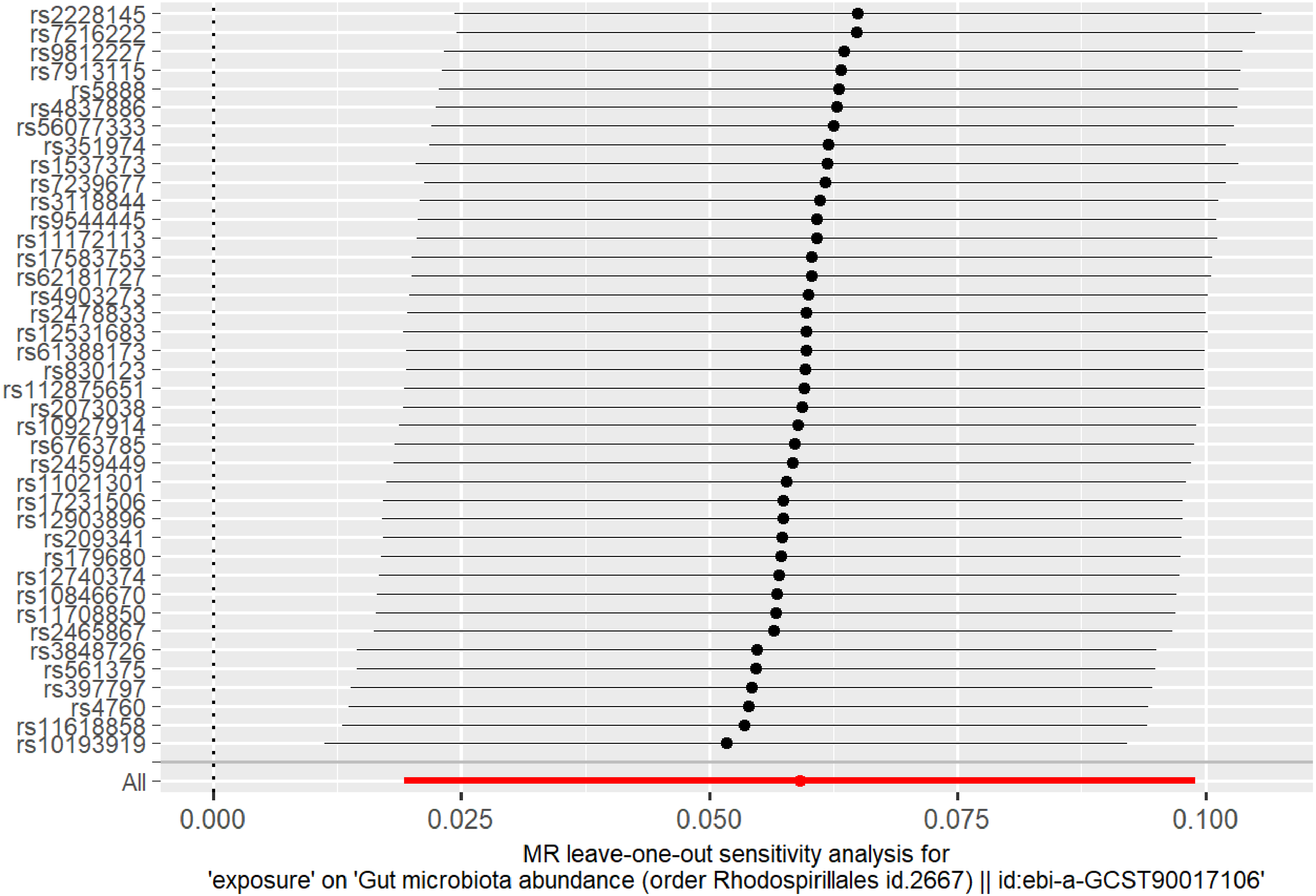
rs2228145
rs7216222
rs9812227
rs7913115
rs5888
rs4837886
rs56077333
rs351974
rs1537373
rs7239677
rs3118844
rs9544445
rs11172113
rs17583753
rs62181727
rs4903273
rs2478833
rs12531683
rs61388173
rs830123
rs112875651
rs2073038
rs10927914
rs6763785
rs2459449
rs11021301
rs17231506
rs12903896
rs209341
rs179680
rs12740374
rs10846670
rs11708850
rs2465867
rs3848726
rs561375
rs397797
rs4760
rs11618858
rs10193919
All
0.000
0.025
0.050
0.075
0.100
MR leave-one-out sensitivity analysis for
'exposure' on 'Gut microbiota abundance (order Rhodospirillales id.2667) || id:ebi-a-GCST90017106'

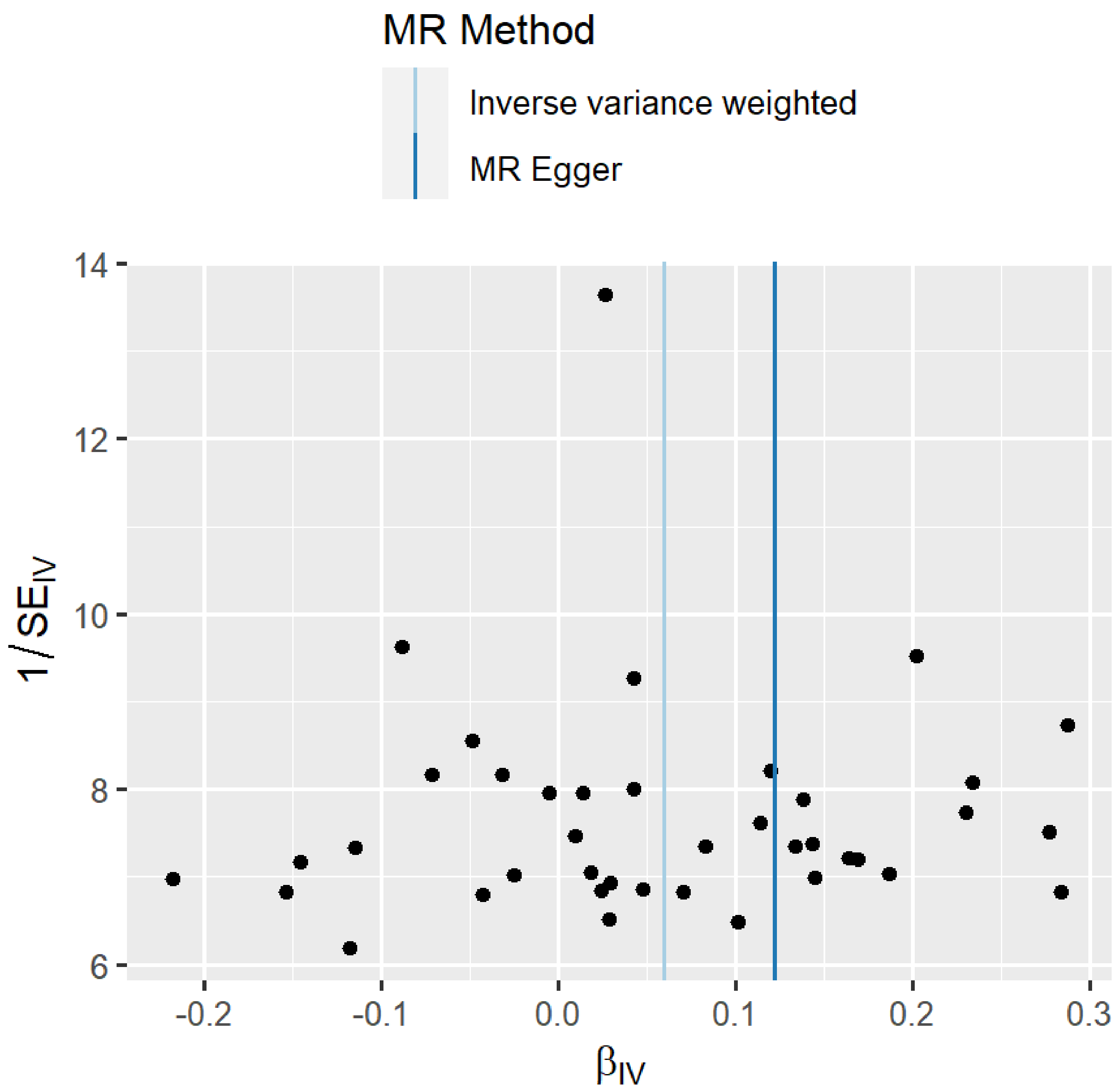
MR Method
Inverse variance weighted
MR Egger
14
12
10
8
6
1/SE_IV
-0.2
-0.1
0.0
0.1
0.2
0.3
β_IV

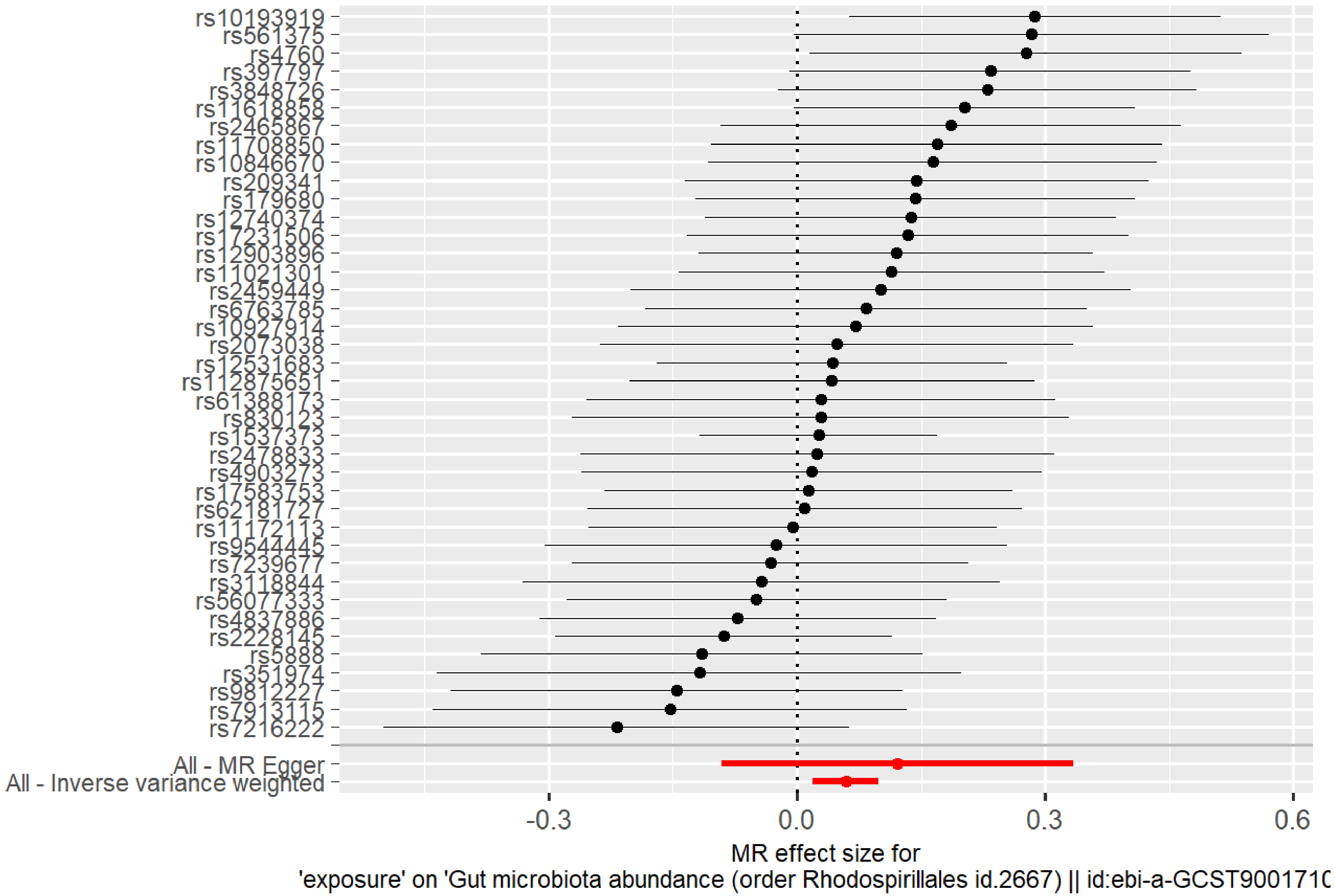

rs10193919
rs561375
rs4760
rs397797
rs3848726
rs11618858
rs2465867
rs11708850
rs10846670
rs209341
rs179680
rs12740374
rs17231506
rs12903896
rs11021301
rs2459449
rs6763785
rs10927914
rs2073038
rs12531683
rs112875651
rs61388173
rs830123
rs1537373
rs2478833
rs4903273
rs17583753
rs62181727
rs11172113
rs9544445
rs7239677
rs3118844
rs56077333
rs4837886
rs2228145
rs5888
rs351974
rs9812227
rs7913115
rs7216222
All - MR Egger
All - Inverse variance weighted
-0.3
0.0
0.3
0.6
MR effect size for
'exposure' on 'Gut microbiota abundance (order Rhodospirillales id.2667) || id:ebi-a-GCST900171(

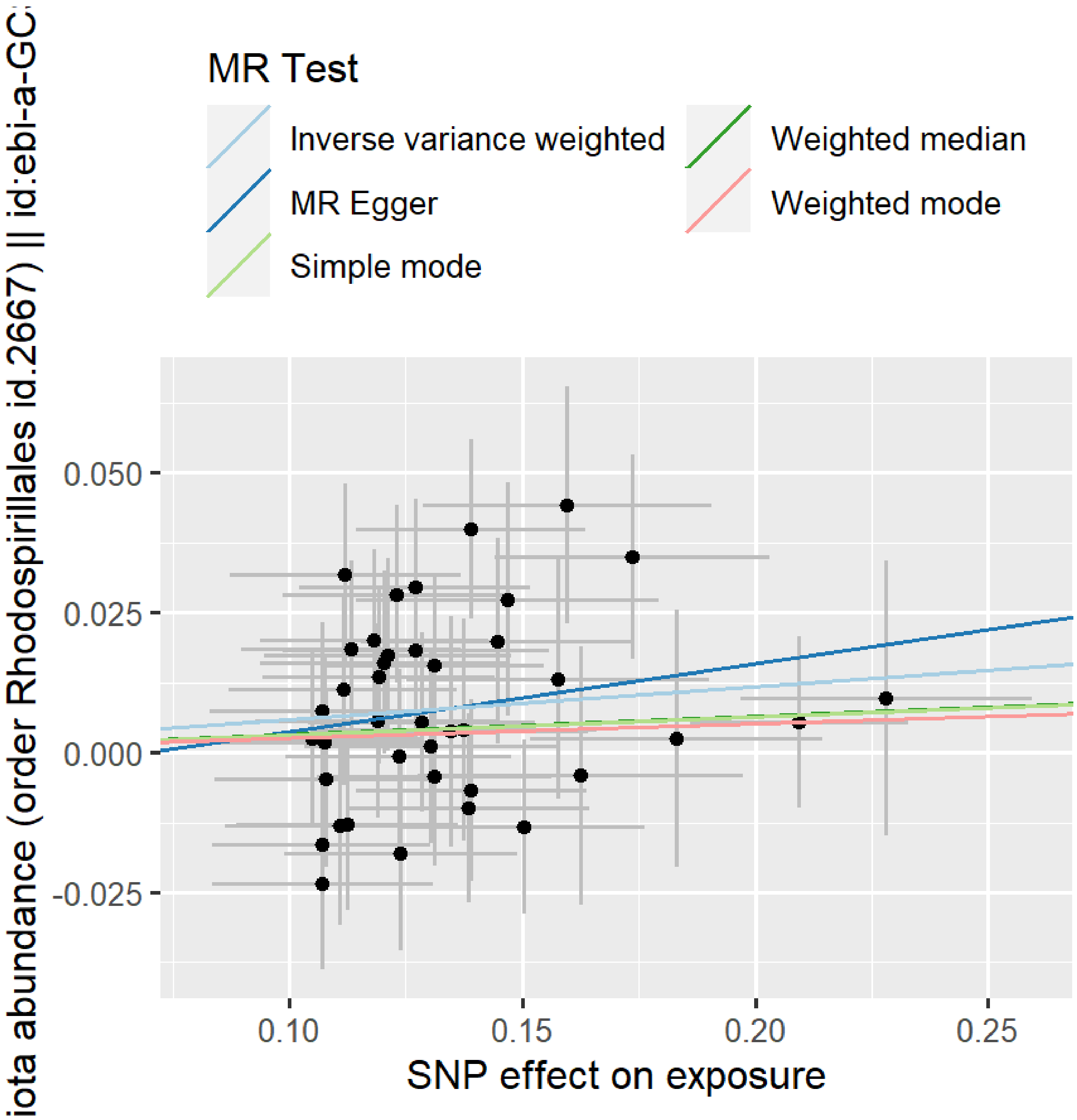
MR Test
Inverse variance weighted
MR Egger
Simple mode
Weighted median
Weighted mode
iota abundance (order Rhodospirillales id.2667) || id:ebi-a-GC
0.050
0.025
0.000
-0.025
0.10
0.15
0.20
0.25
SNP effect on exposure

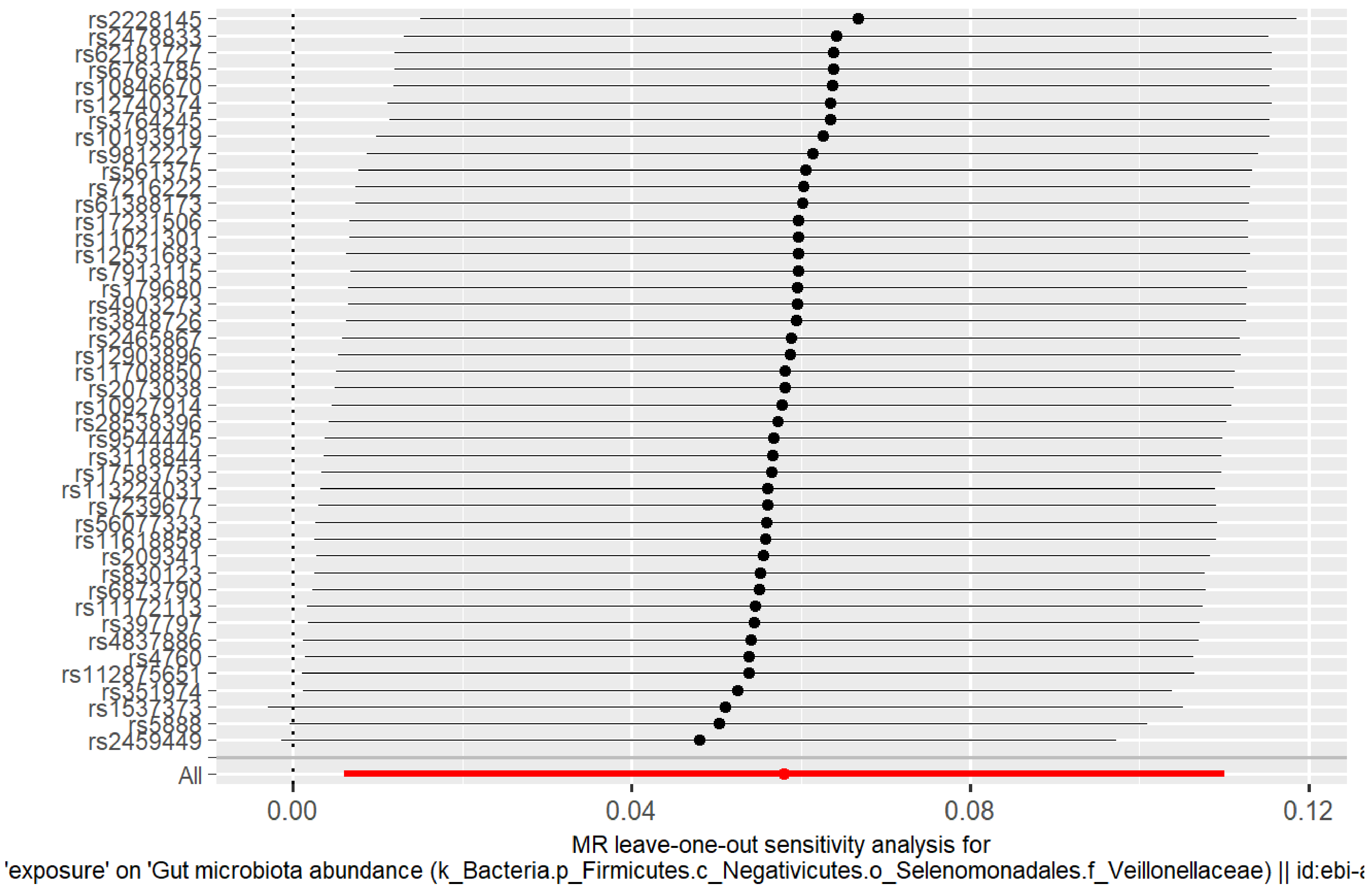
rs2228145
rs2478833
rs62181727
rs6763785
rs10846670
rs12740374
rs3764245
rs10193919
rs9812227
rs561375
rs7216222
rs61388173
rs17231506
rs11021301
rs12531683
rs7913115
rs179680
rs4903273
rs3848726
rs2465867
rs12903896
rs11708850
rs2073038
rs10927914
rs28538396
rs9544445
rs3118844
rs17583753
rs113224031
rs7239677
rs56077333
rs11618858
rs209341
rs830123
rs6873790
rs11172113
rs397797
rs4837886
rs4760
rs112875651
rs351974
rs1537373
rs5888
rs2459449
All
0.00
0.04
0.08
0.12
MR leave-one-out sensitivity analysis for
'exposure' on 'Gut microbiota abundance (k_Bacteria.p_Firmicutes.c_Negativicutes.o_Selenomonadales.f_Veillonellaceae) || id:ebi-a

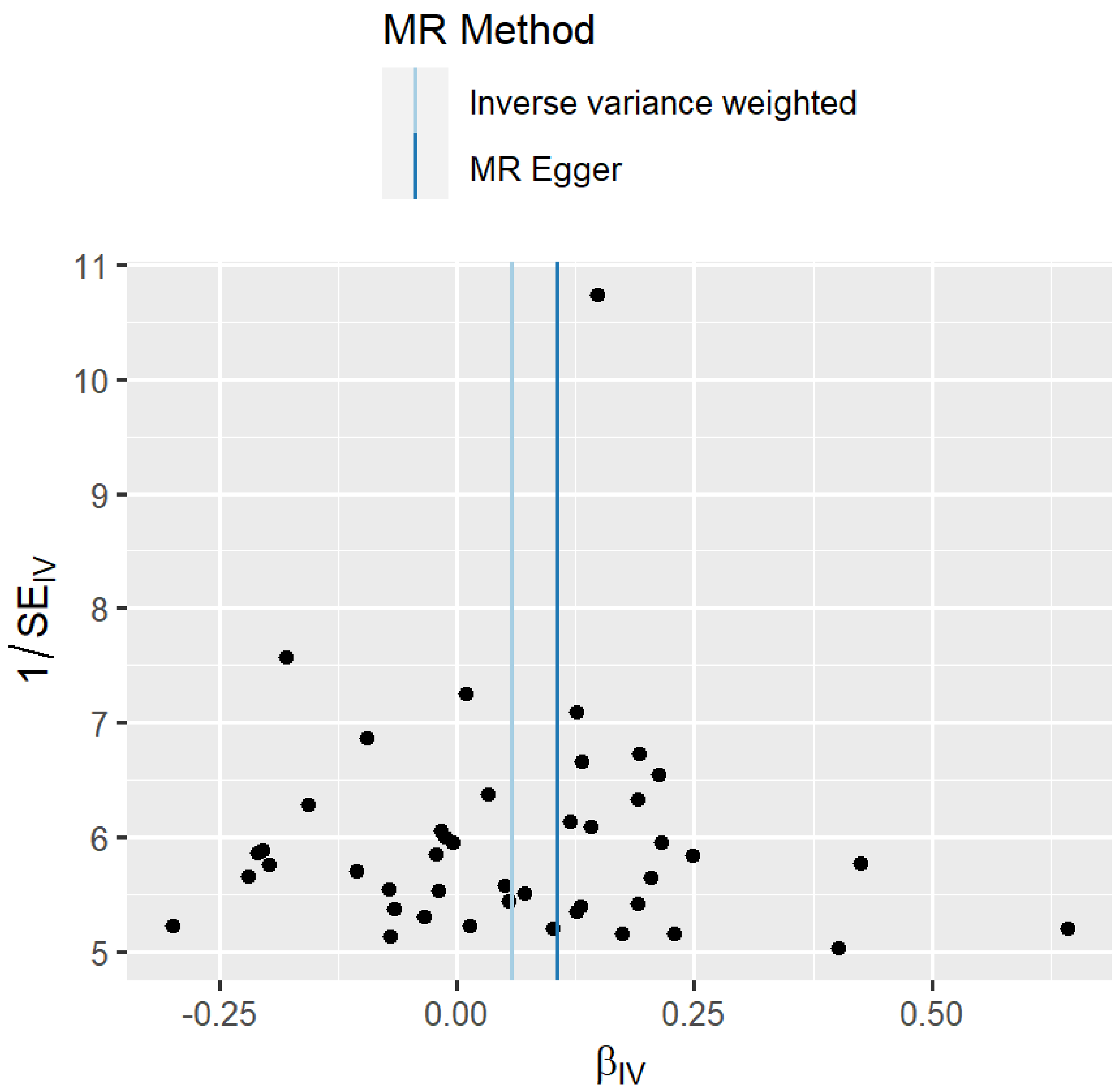

MR Method
Inverse variance weighted
MR Egger
11
10
9
8
7
6
5
1/SE_IV
-0.25
0.00
0.25
0.50
β_IV

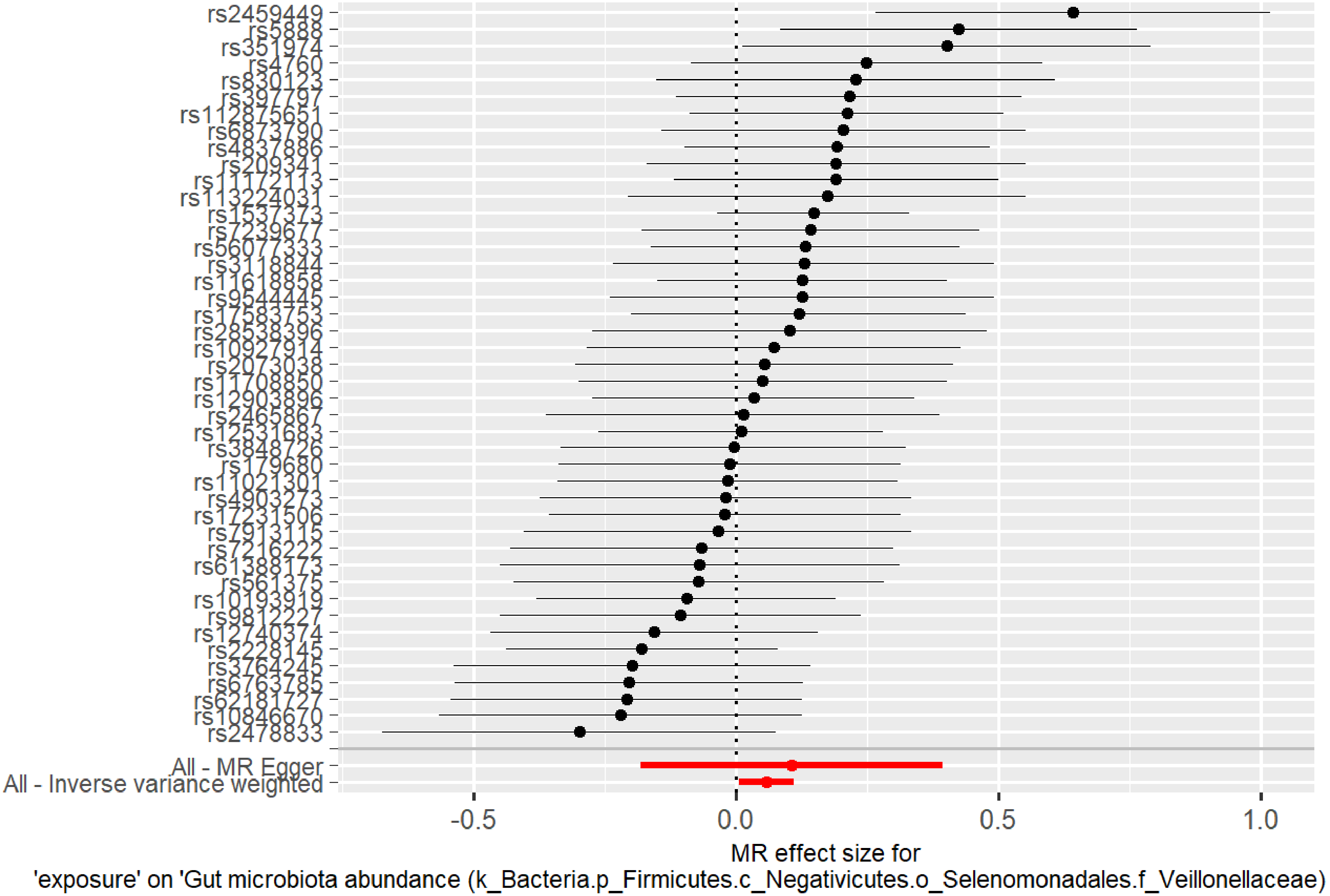
rs2459449
rs5888
rs351974
rs4760
rs830123
rs397797
rs112875651
rs6873790
rs4837886
rs209341
rs11172113
rs113224031
rs1537373
rs7239677
rs56077333
rs3118844
rs11618858
rs9544445
rs17583753
rs28538396
rs10927914
rs2073038
rs11708850
rs12903896
rs2465867
rs12531683
rs3848726
rs179680
rs11021301
rs4903273
rs17231506
rs7913115
rs7216222
rs61388173
rs561375
rs10193919
rs9812227
rs12740374
rs2228145
rs3764245
rs6763785
rs62181727
rs10846670
rs2478833
All - MR Egger
All - Inverse variance weighted
-0.5
0.0
0.5
1.0
MR effect size for
'exposure' on 'Gut microbiota abundance (k_Bacteria.p_Firmicutes.c_Negativicutes.o_Selenomonadales.f_Veillonellaceae)

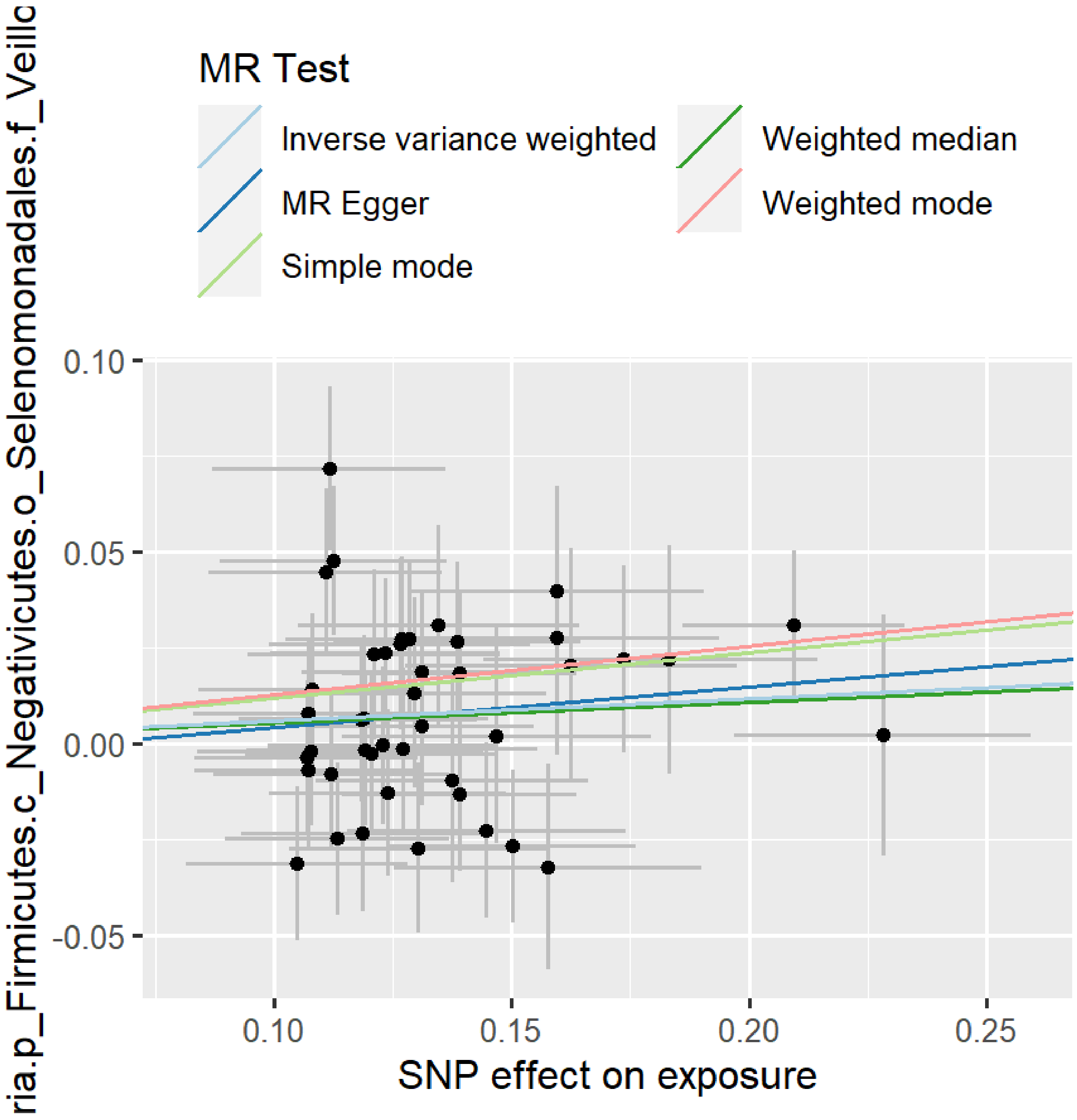
MR Test
Inverse variance weighted
MR Egger
Simple mode
Weighted median
Weighted mode
0.10
0.05
0.00
-0.05
0.10
0.15
0.20
0.25
SNP effect on exposure
ria.p_Firmicutes.c_Negativicutes.o_Selenomonadales.f_Veillc

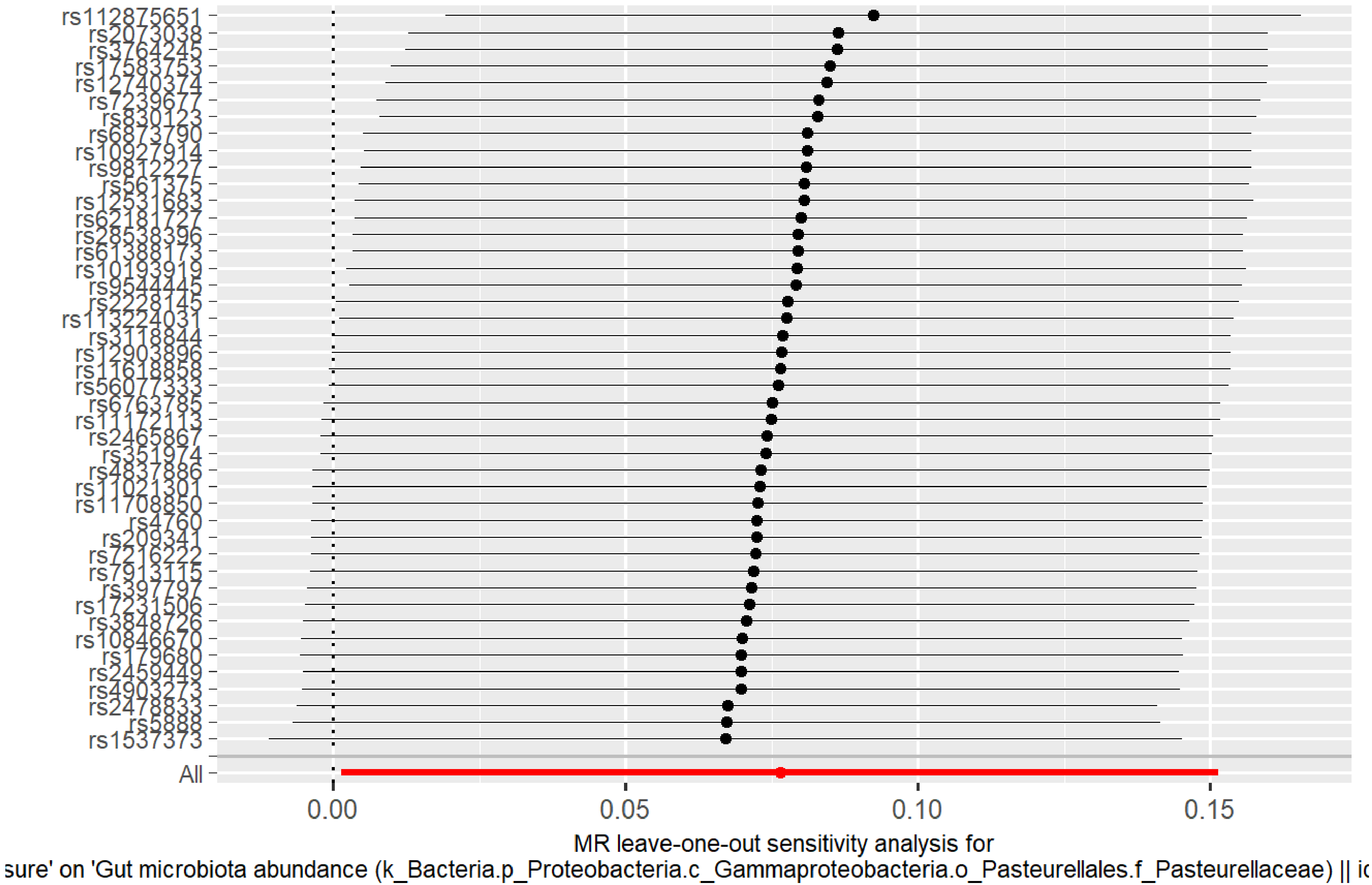
rs112875651
rs2073038
rs3764245
rs17583753
rs12740374
rs7239677
rs830123
rs6873790
rs10927914
rs9812227
rs561375
rs12531683
rs62181727
rs28538396
rs61388173
rs10193919
rs9544445
rs2228145
rs113224031
rs3118844
rs12903896
rs11618858
rs56077333
rs6763785
rs11172113
rs2465867
rs351974
rs4837886
rs11021301
rs11708850
rs4760
rs209341
rs7216222
rs7913115
rs397797
rs17231506
rs3848726
rs10846670
rs179680
rs2459449
rs4903273
rs2478833
rs5888
rs1537373
All
0.00
0.05
0.10
0.15
MR leave-one-out sensitivity analysis for
sure' on 'Gut microbiota abundance (k_Bacteria.p_Proteobacteria.c_Gammaproteobacteria.o_Pasteurellales.f_Pasteurellaceae) || ic

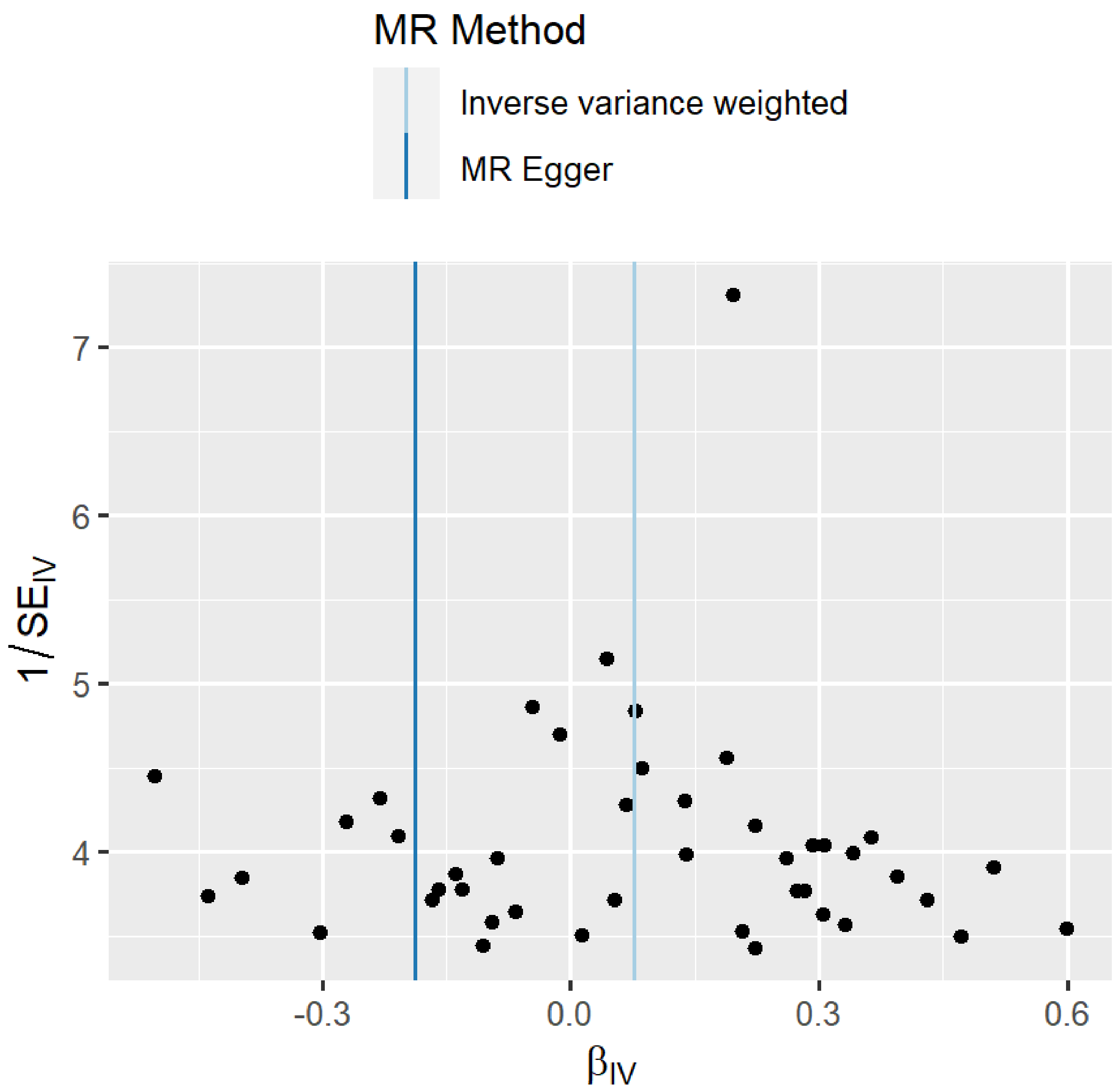
MR Method
Inverse variance weighted
MR Egger
7
6
5
4
1/SE_IV
-0.3
0.0
0.3
0.6
β_IV

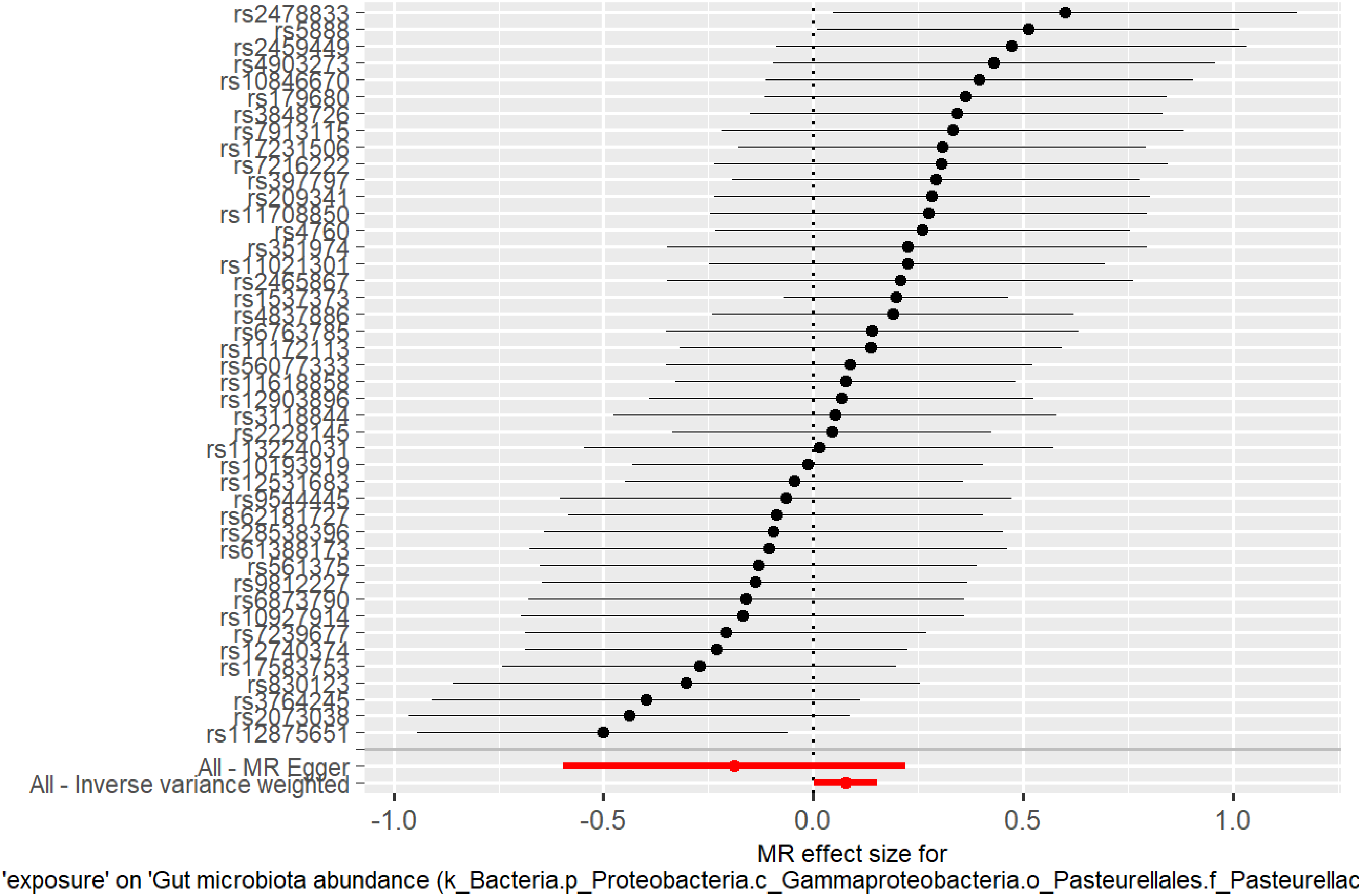

rs2478833
rs5888
rs2459449
rs4903273
rs10846670
rs179680
rs3848726
rs7913115
rs17231506
rs7216222
rs397797
rs209341
rs11708850
rs4760
rs351974
rs11021301
rs2465867
rs1537373
rs4837886
rs6763785
rs11172113
rs56077333
rs11618858
rs12903896
rs3118844
rs2228145
rs113224031
rs10193919
rs12531683
rs9544445
rs62181727
rs28538396
rs61388173
rs561375
rs9812227
rs6873790
rs10927914
rs7239677
rs12740374
rs17583753
rs830123
rs3764245
rs2073038
rs112875651
All - MR Egger
All - Inverse variance weighted
-1.0
-0.5
0.0
0.5
1.0
MR effect size for
'exposure' on 'Gut microbiota abundance (k_Bacteria.p_Proteobacteria.c_Gammaproteobacteria.o_Pasteurellales.f_Pasteurellac

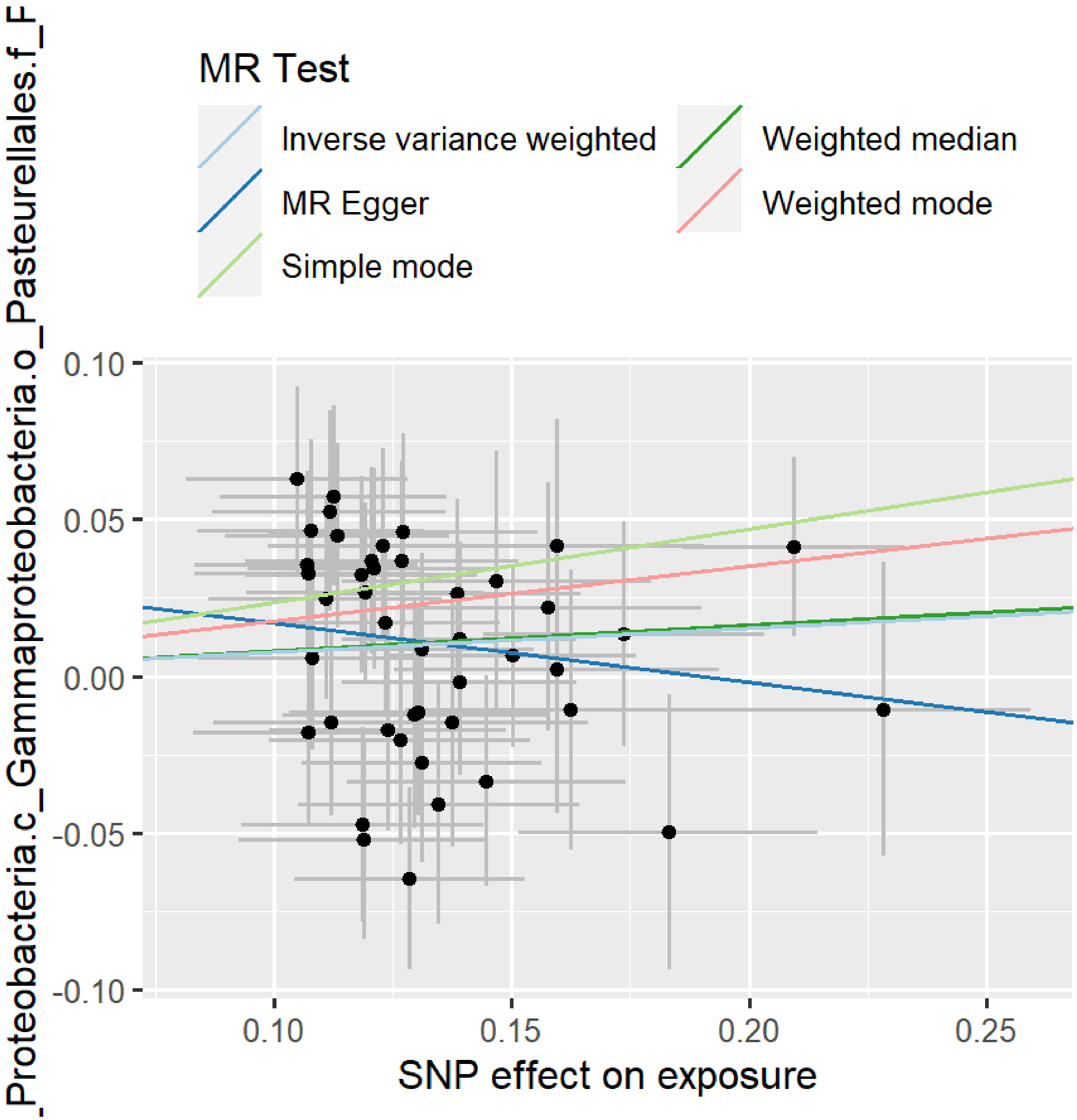

MR Test
Inverse variance weighted
MR Egger
Simple mode
Weighted median
Weighted mode
Proteobacteria.c_Gammaproteobacteria.o_Pasteurellales.f_F
SNP effect on exposure
0.10
0.05
0.00
-0.05
-0.10
0.10
0.15
0.20
0.25

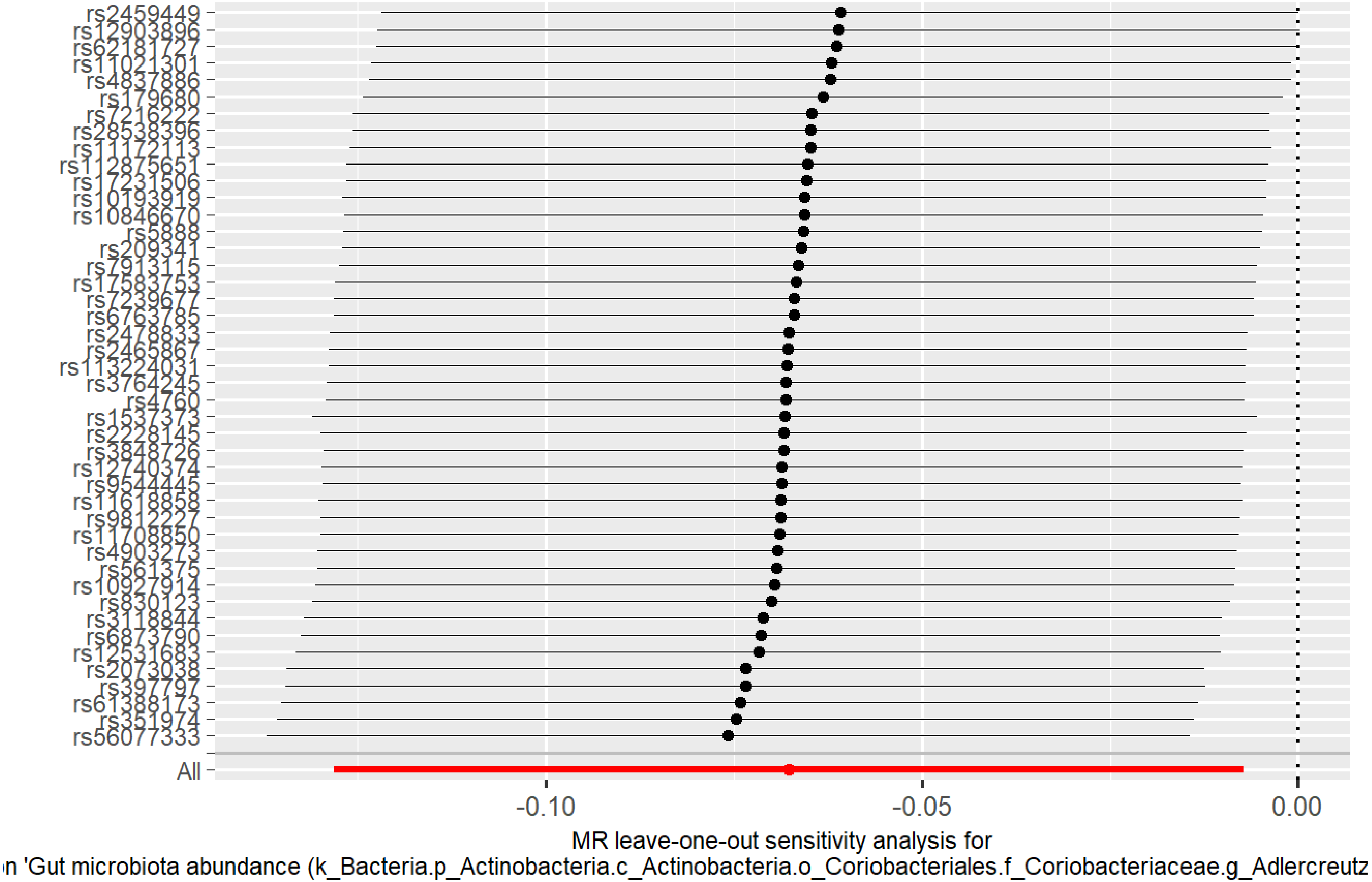
rs2459449
rs12903896
rs62181727
rs11021301
rs4837886
rs179680
rs7216222
rs28538396
rs11172113
rs112875651
rs17231506
rs10193919
rs10846670
rs5888
rs209341
rs7913115
rs1758375?
rs72396??
rs6763785
rs2478833
rs2465867
rs113224031
rs3764245
rs4760
rs1537373
rs2228145
rs3848726
rs12740374
rs9544445
rs11618858
rs9812227
rs11708850
rs4903273
rs561375
rs10927914
rs830123
rs3118844
rs6873790
rs12531683
rs2073038
rs397797
rs61388173
rs351974
rs56077333
All
-0.10
-0.05
0.00
MR leave-one-out sensitivity analysis for
n 'Gut microbiota abundance (k_Bacteria.p_Actinobacteria.c_Actinobacteria.o_Coriobacteriales.f_Coriobacteriaceae.g_Adlercreutz

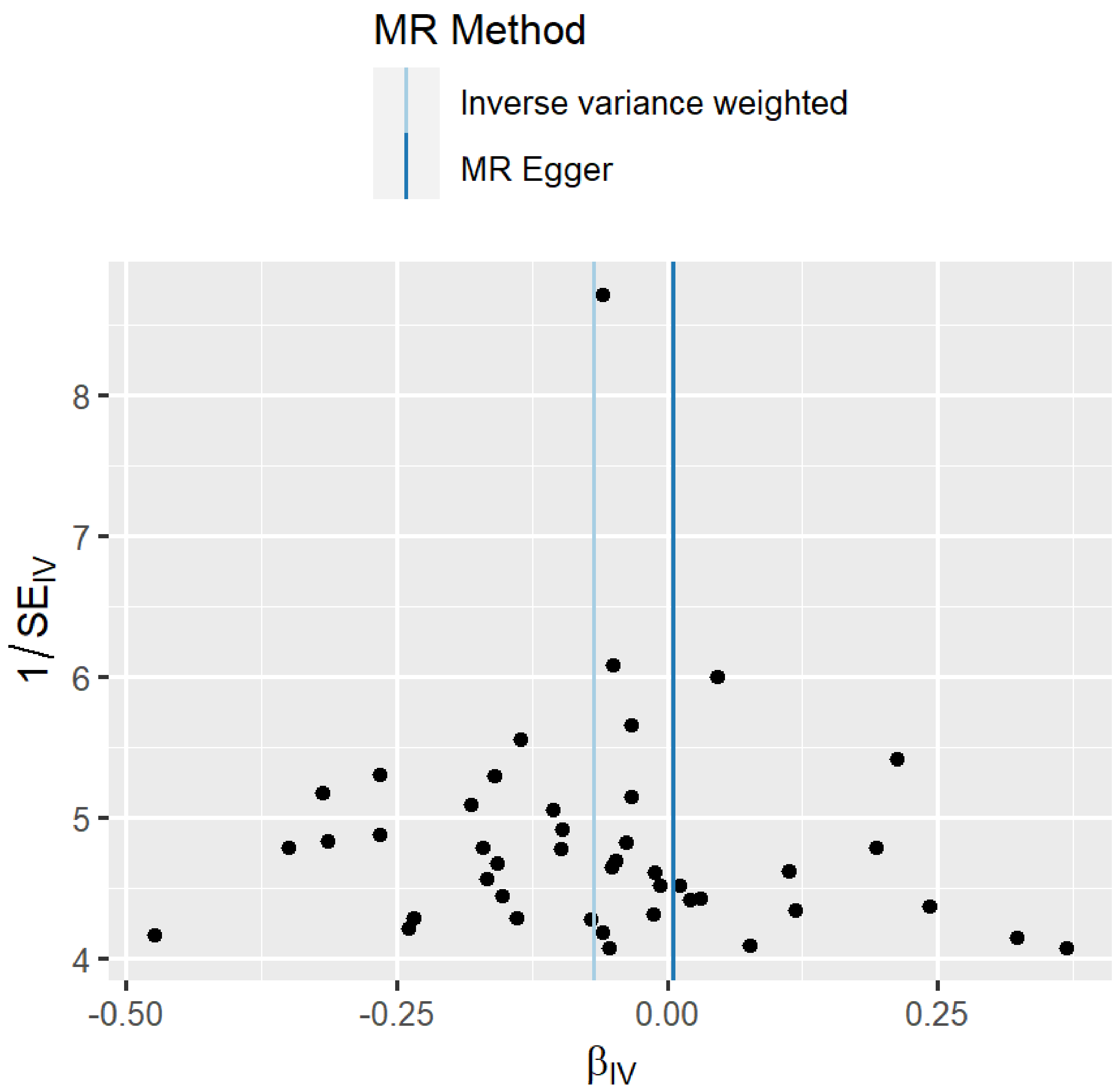
MR Method
Inverse variance weighted
MR Egger
8
7
6
5
4
$1/SE_{IV}$
-0.50
-0.25
0.00
0.25
β_{IV}

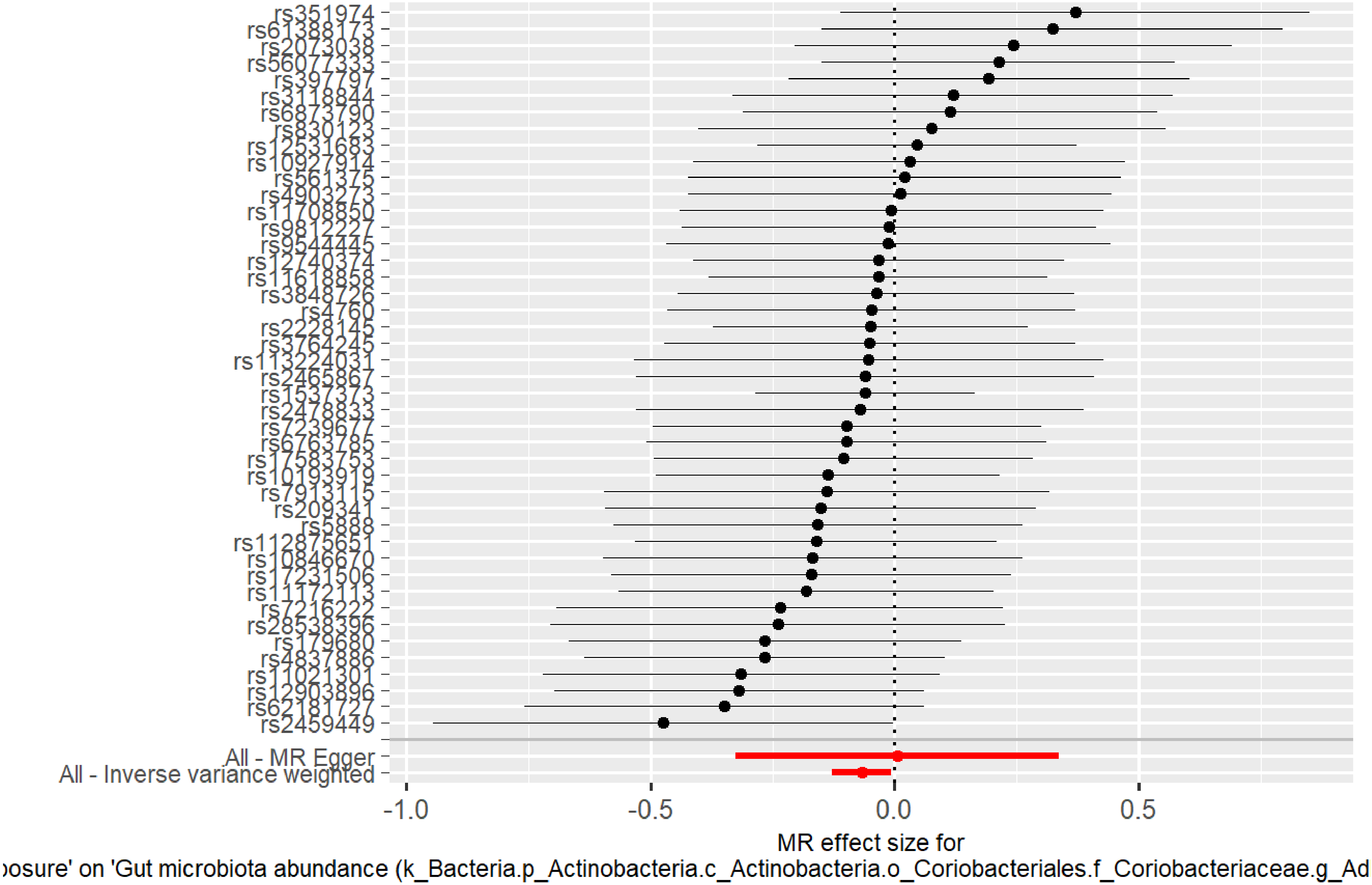
rs351974
rs61388173
rs2073038
rs56077333
rs397797
rs3118844
rs6873790
rs830123
rs12531683
rs10927914
rs561375
rs4903273
rs11708850
rs9812227
rs9544445
rs12740374
rs11618858
rs3848726
rs4760
rs2228145
rs3764245
rs113224031
rs2465867
rs1537373
rs2478833
rs7239677
rs6763785
rs17583753
rs10193919
rs7913115
rs209341
rs5888
rs112875651
rs10846670
rs17231506
rs11172113
rs7216222
rs28538396
rs179680
rs4837886
rs11021301
rs12903896
rs62181727
rs2459449
All - MR Egger
All - Inverse variance weighted
-1.0
-0.5
0.0
0.5
MR effect size for
osure' on 'Gut microbiota abundance (k_Bacteria.p_Actinobacteria.c_Actinobacteria.o_Coriobacteriales.f_Coriobacteriaceae.g_Ad

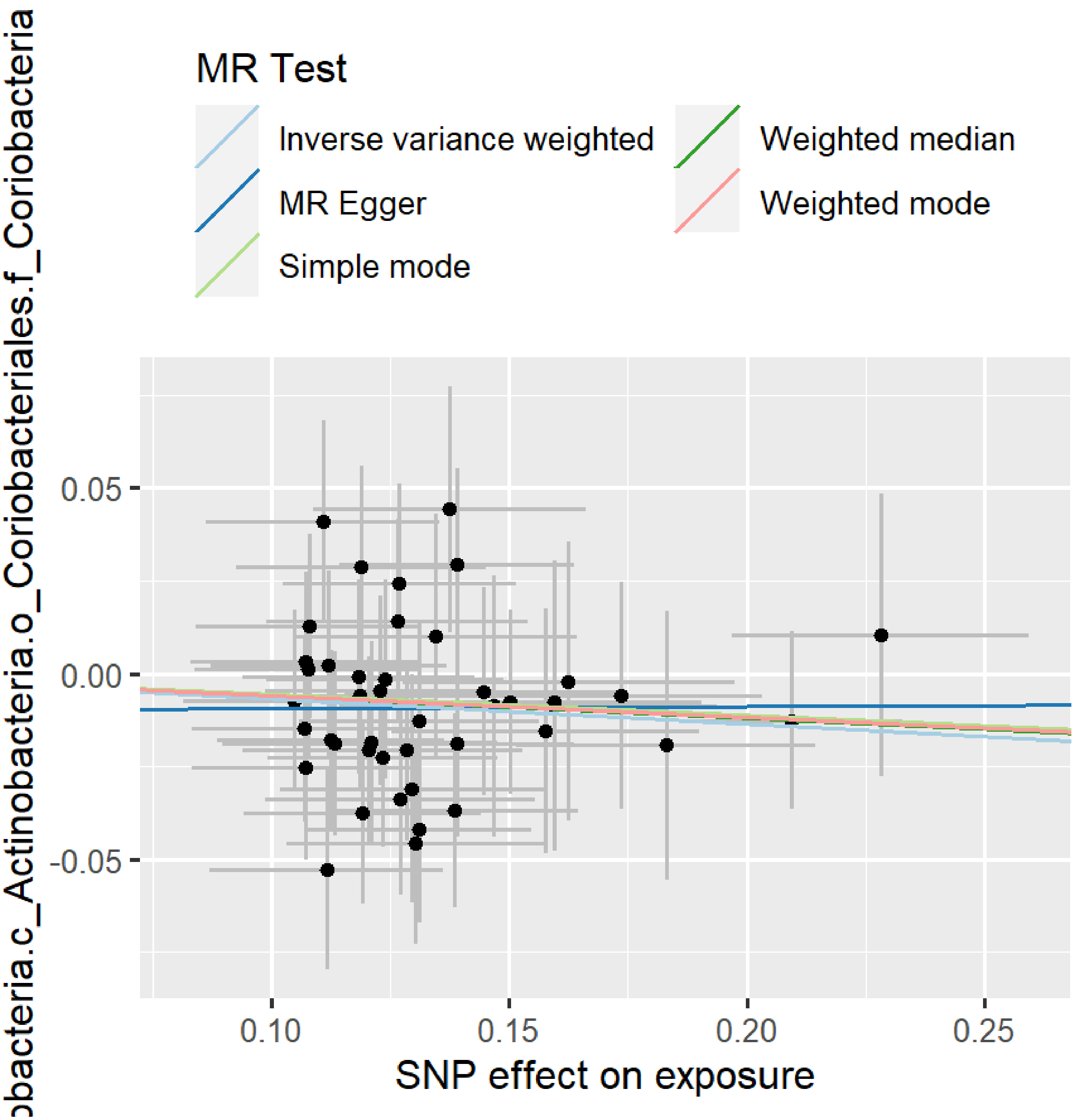
MR Test
Inverse variance weighted
MR Egger
Simple mode
Weighted median
Weighted mode
0.05
0.00
-0.05
0.10
0.15
0.20
0.25
SNP effect on exposure
obacteria.c_Actinobacteria.o_Coriobacteriales.f_Coriobacteria

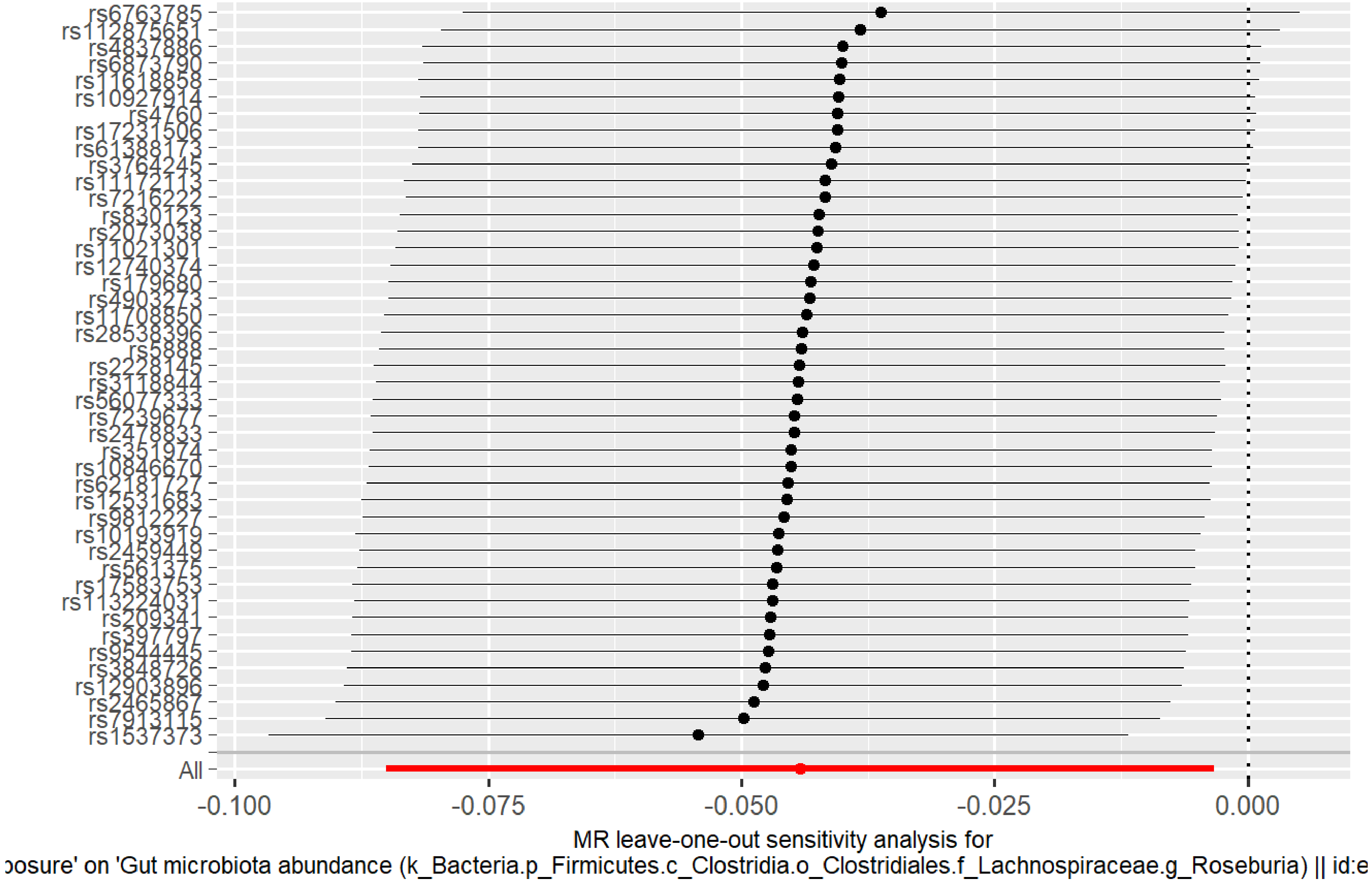
rs6763785
rs112875651
rs4837886
rs6873790
rs11618858
rs10927914
rs4760
rs17231506
rs61388173
rs3764245
rs11172113
rs7216222
rs830123
rs2073038
rs11021301
rs12740374
rs179680
rs4903273
rs11708850
rs28538396
rs5888
rs2228145
rs3118844
rs56077333
rs7239677
rs2478833
rs351974
rs10846670
rs62181727
rs12531683
rs9812227
rs10193919
rs2459449
rs561375
rs17583753
rs113224031
rs209341
rs397797
rs9544445
rs3848726
rs12903896
rs2465867
rs7913115
rs1537373
All
-0.100
-0.075
-0.050
-0.025
0.000
MR leave-one-out sensitivity analysis for
posure' on 'Gut microbiota abundance (k_Bacteria.p_Firmicutes.c_Clostridia.o_Clostridiales.f_Lachnospiraceae.g_Roseburia) || id:e

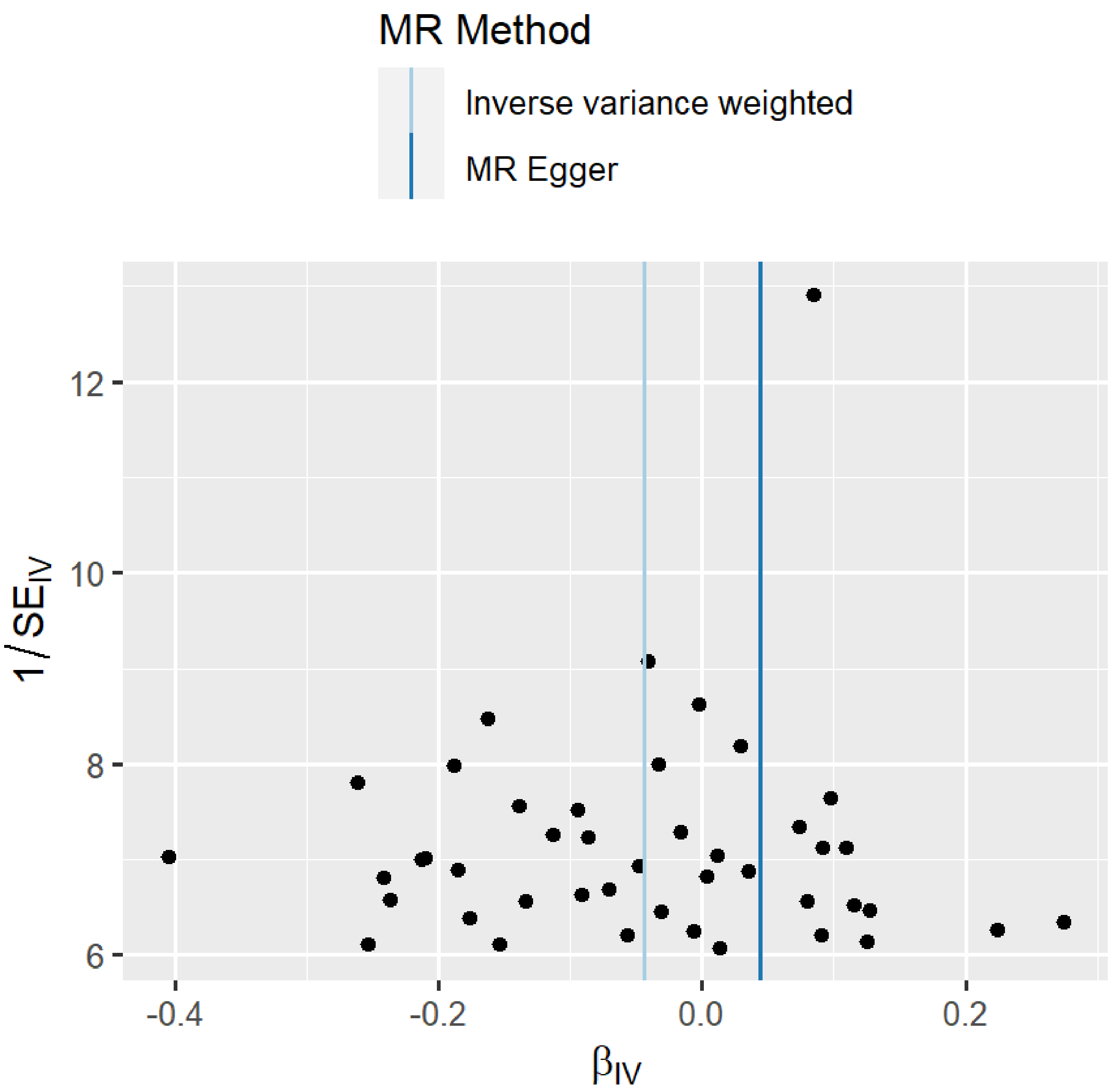
MR Method
Inverse variance weighted
MR Egger
12
10
8
6
1/SE$_{IV}$
-0.4
-0.2
0.0
0.2
β$_{IV}$

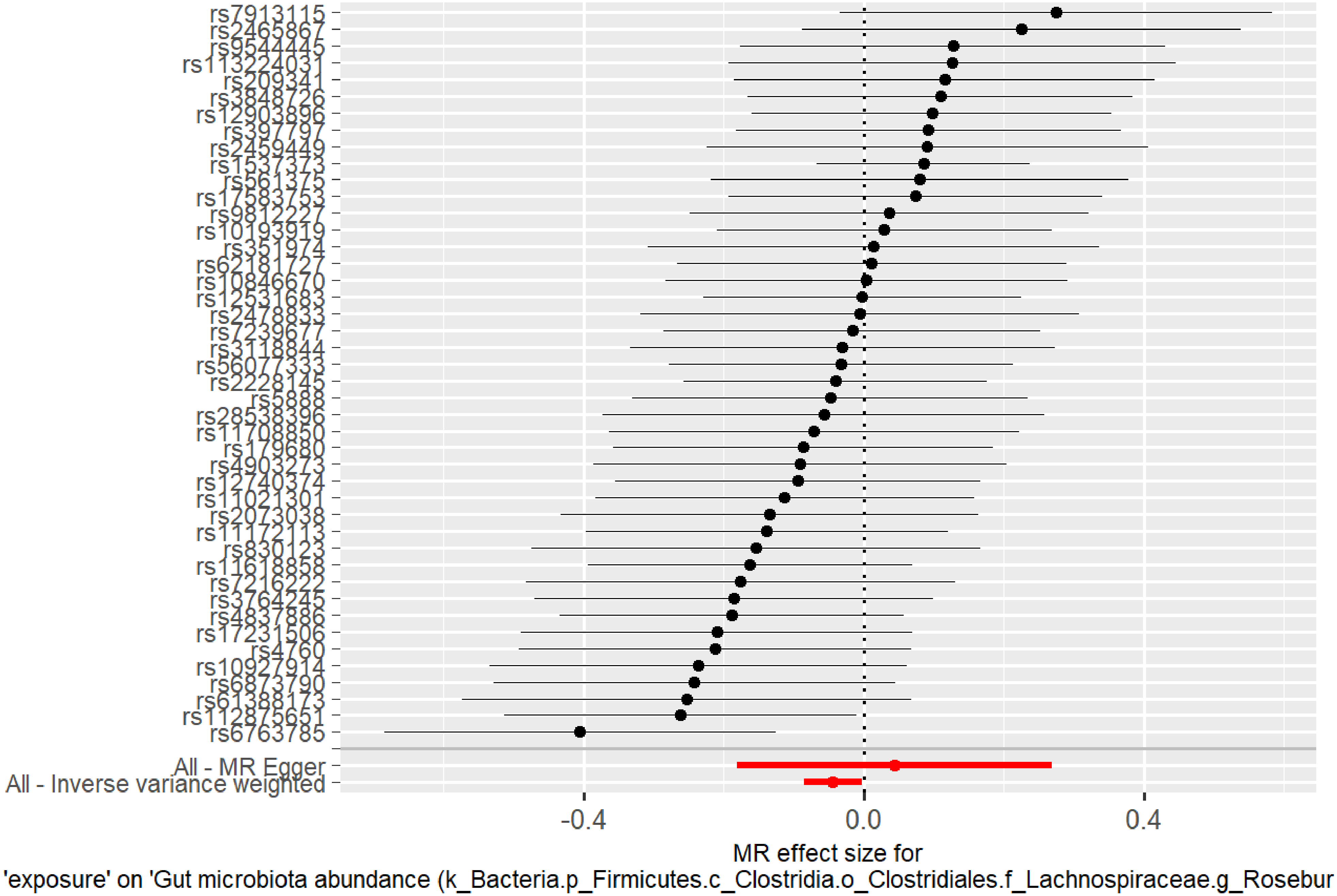
rs7913115
rs2465867
rs9544445
rs113224031
rs209341
rs3848726
rs12903896
rs397797
rs2459449
rs1537373
rs561375
rs17583753
rs9812227
rs10193919
rs351974
rs62181727
rs10846670
rs12531683
rs2478833
rs7239677
rs3118844
rs56077333
rs2228145
rs5888
rs28538396
rs11708850
rs179680
rs4903273
rs12740374
rs11021301
rs2073038
rs11172113
rs830123
rs11618858
rs7216222
rs3764245
rs4837886
rs17231506
rs4760
rs10927914
rs6873790
rs61388173
rs112875651
rs6763785
All - MR Egger
All - Inverse variance weighted
-0.4
0.0
0.4
MR effect size for
'exposure' on 'Gut microbiota abundance (k_Bacteria.p_Firmicutes.c_Clostridia.o_Clostridiales.f_Lachnospiraceae.g_Rosebur

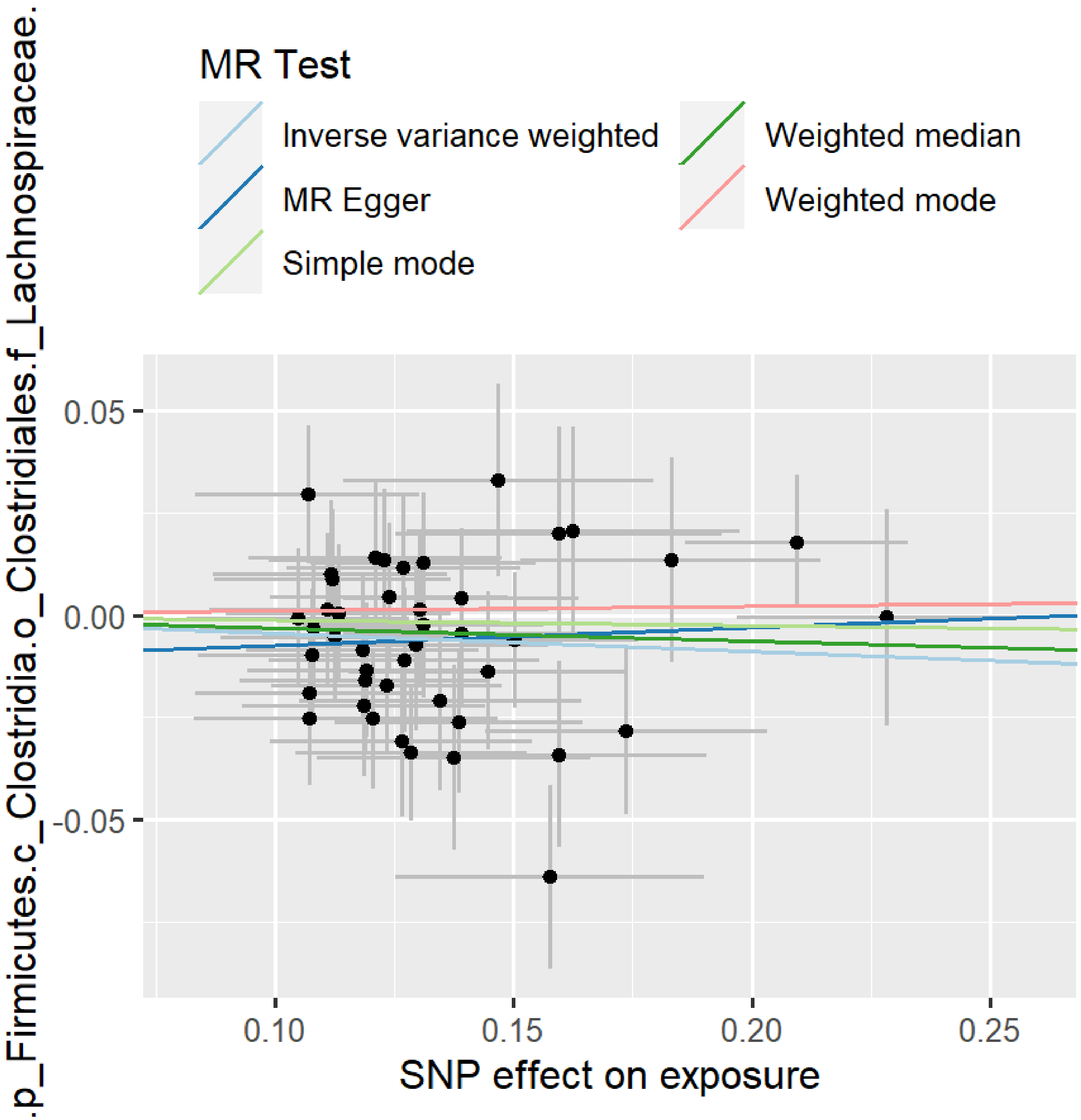
MR Test
Inverse variance weighted
MR Egger
Simple mode
Weighted median
Weighted mode
0.05
0.00
-0.05
0.10
0.15
0.20
0.25
SNP effect on exposure
p_Firmicutes.c_Clostridia.o_Clostridiales.f_Lachnospiraceae.

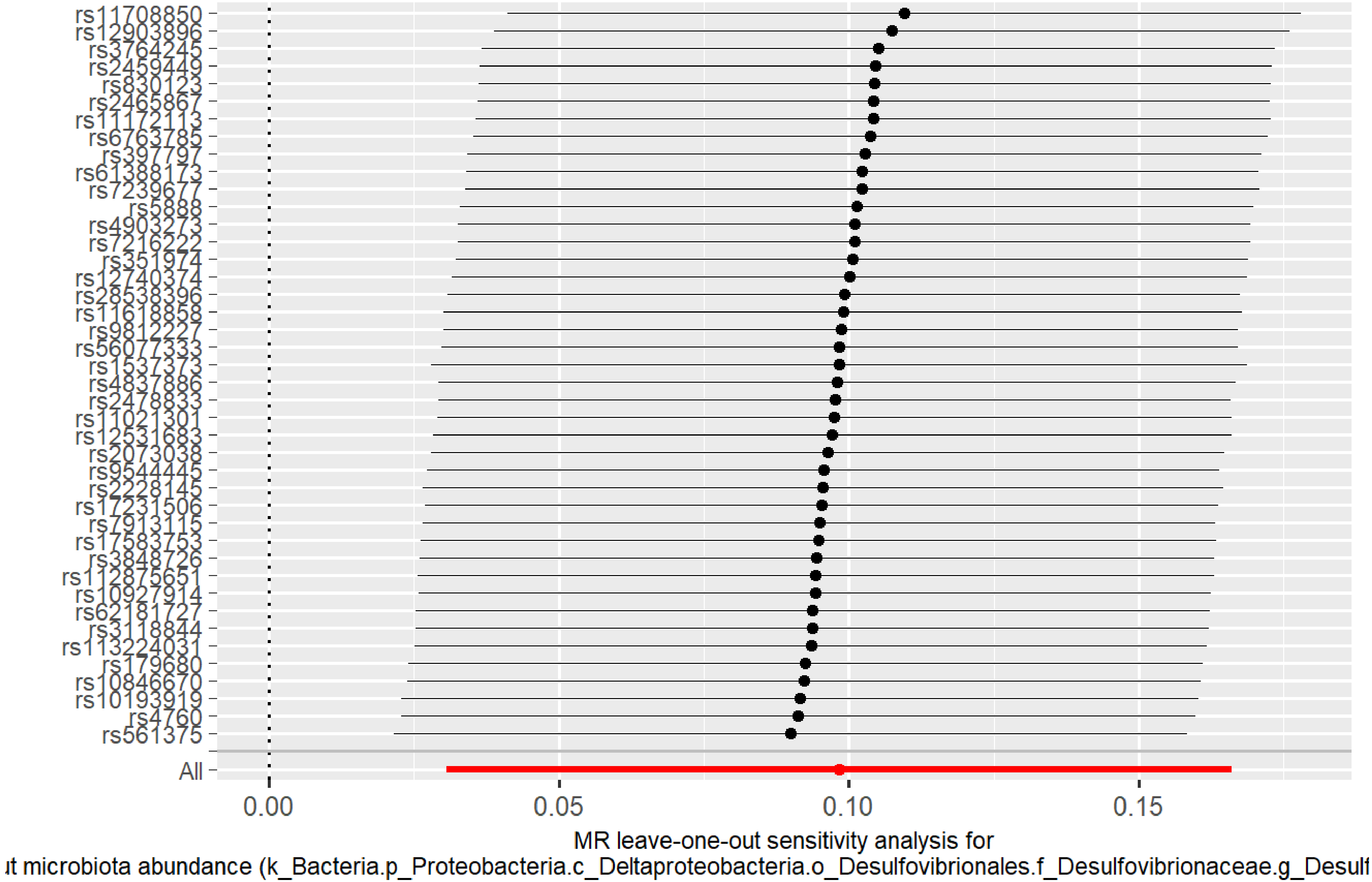
rs11708850
rs12903896
rs3764245
rs2459449
rs830123
rs2465867
rs11172113
rs6763785
rs397797
rs61388173
rs7239677
rs5888
rs4903273
rs7216222
rs351974
rs12740374
rs28538396
rs11618858
rs9812227
rs56077333
rs1537373
rs4837886
rs2478833
rs11021301
rs12531683
rs2073038
rs9544445
rs2228145
rs17231506
rs7913115
rs17583753
rs3848726
rs112875651
rs10927914
rs62181727
rs3118844
rs113224031
rs179680
rs10846670
rs10193919
rs4760
rs561375
All
0.00
0.05
0.10
0.15
MR leave-one-out sensitivity analysis for
ıt microbiota abundance (k_Bacteria.p_Proteobacteria.c_Deltaproteobacteria.o_Desulfovibrionales.f_Desulfovibrionaceae.g_Desulf

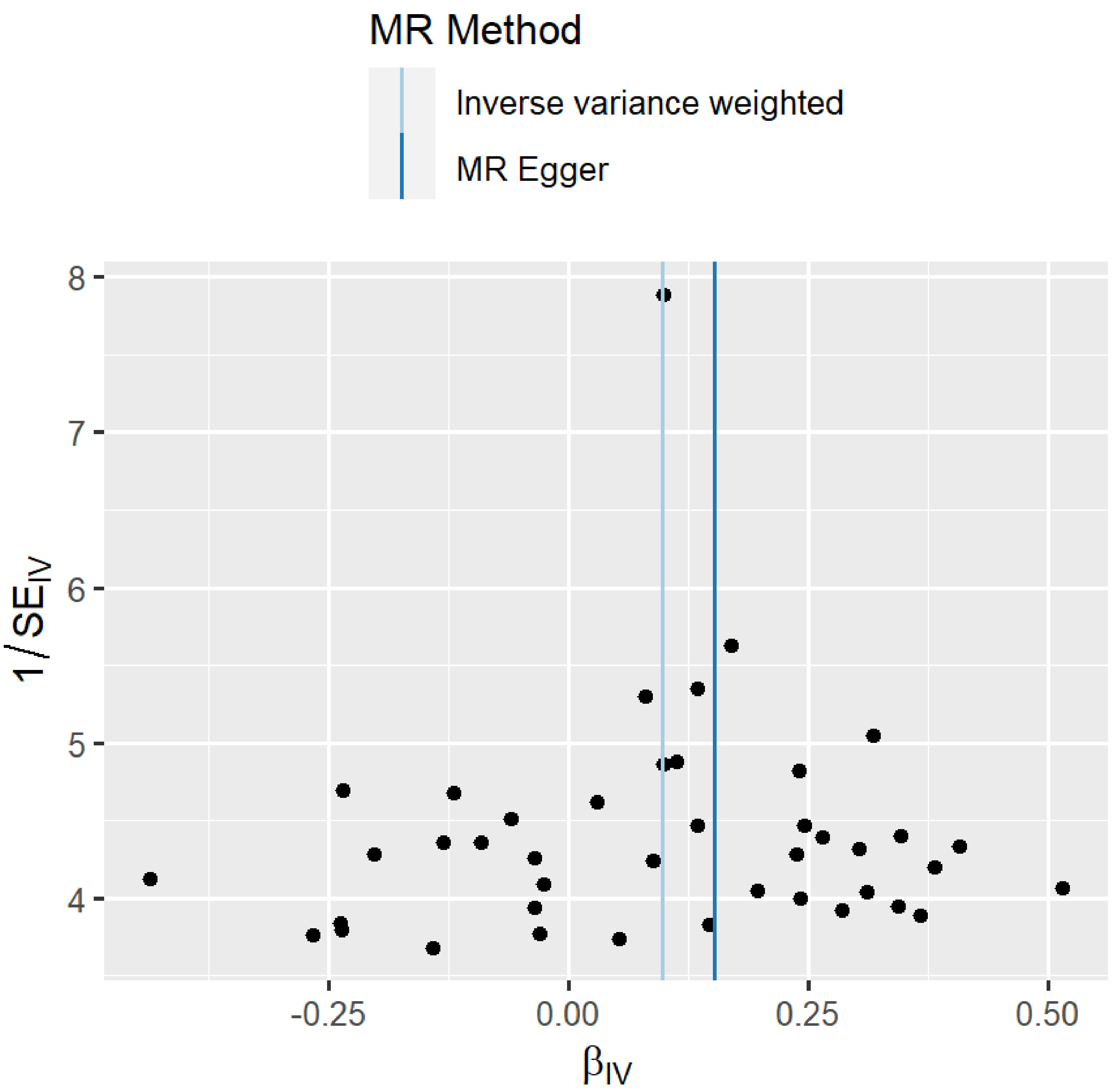
MR Method
Inverse variance weighted
MR Egger
8
7
6
5
4
1/SE_IV
-0.25
0.00
0.25
0.50
β_IV

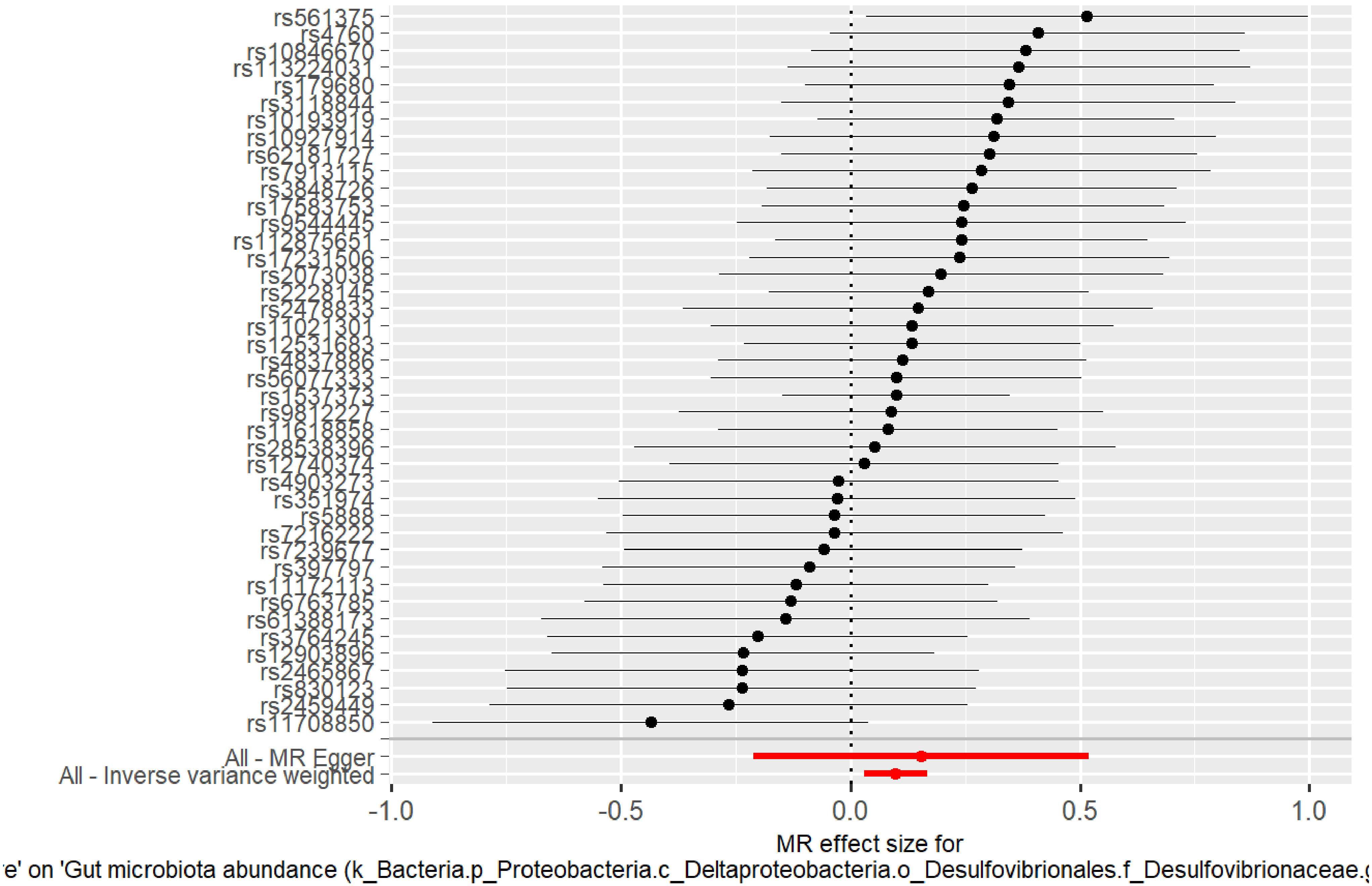
rs561375
rs4760
rs10846670
rs113224031
rs179680
rs3118844
rs10193919
rs10927914
rs62181727
rs7913115
rs3848726
rs17583753
rs9544445
rs112875651
rs17231506
rs2073038
rs2228145
rs2478833
rs11021301
rs12531683
rs4837886
rs56077333
rs1537373
rs9812227
rs11618858
rs28538396
rs12740374
rs4903273
rs351974
rs5888
rs7216222
rs7239677
rs397797
rs11172113
rs6763785
rs61388173
rs3764245
rs12903896
rs2465867
rs830123
rs2459449
rs11708850
All - MR Egger
All - Inverse variance weighted
-1.0
-0.5
0.0
0.5
1.0
MR effect size for
e' on 'Gut microbiota abundance (k_Bacteria.p_Proteobacteria.c_Deltaproteobacteria.o_Desulfovibrionales.f_Desulfovibrionaceae.

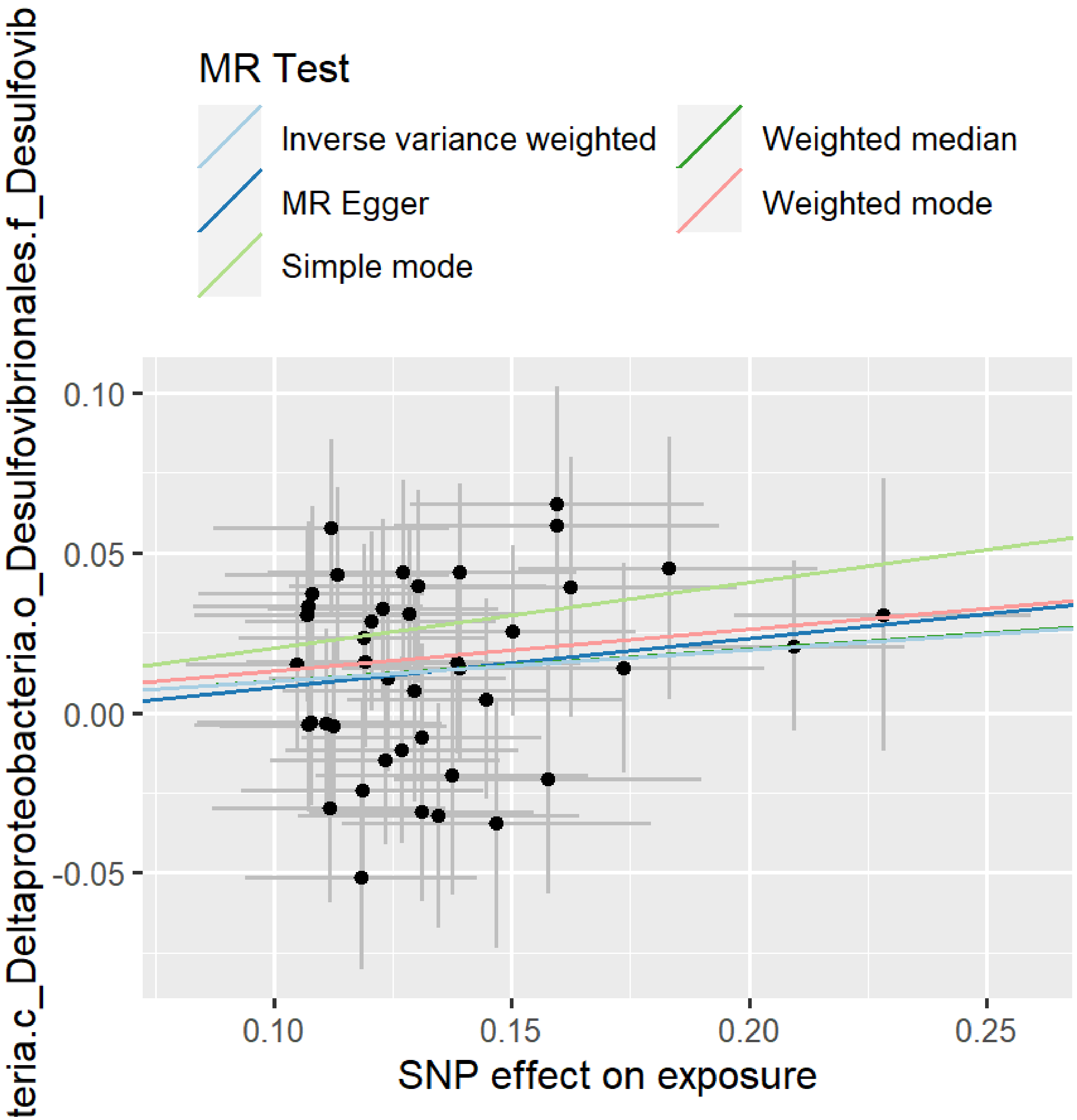
MR Test
Inverse variance weighted
MR Egger
Simple mode
Weighted median
Weighted mode
0.10
0.05
0.00
-0.05
0.10
0.15
0.20
0.25
SNP effect on exposure
teria.c_Deltaproteobacteria.o_Desulfovibrionales.f_Desulfovib

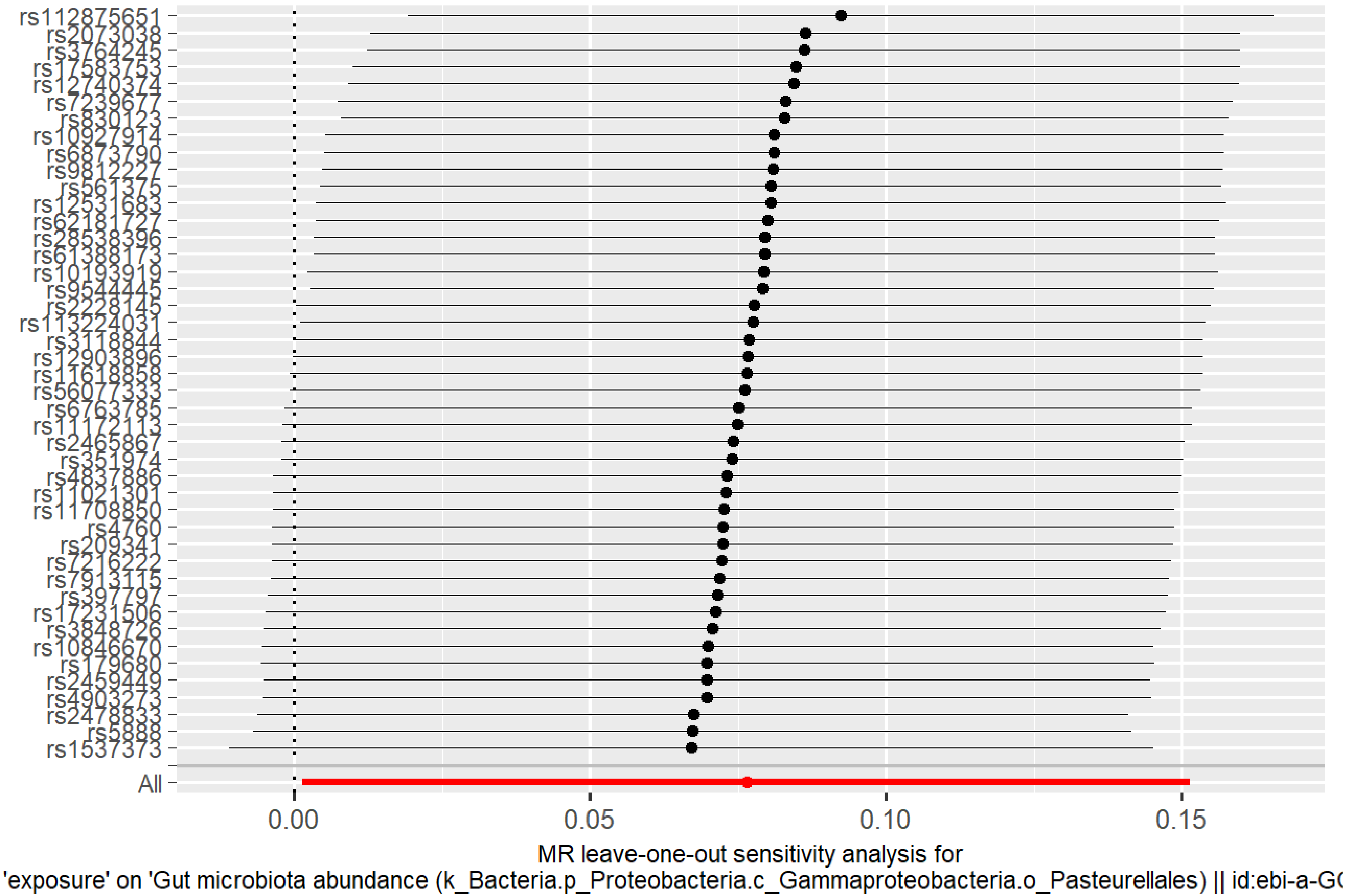
rs112875651
rs2073038
rs3764245
rs17583753
rs12740374
rs7239677
rs830123
rs10927914
rs6873790
rs9812227
rs561375
rs12531683
rs62181727
rs28538396
rs61388173
rs10193919
rs9544445
rs2228145
rs113224031
rs3118844
rs12903896
rs11618858
rs56077333
rs6763785
rs11172113
rs2465867
rs351974
rs4837886
rs11021301
rs11708850
rs4760
rs209341
rs7216222
rs7913115
rs397797
rs17231506
rs3848726
rs10846670
rs179680
rs2459449
rs4903273
rs2478833
rs5888
rs1537373
All
0.00
0.05
0.10
0.15
MR leave-one-out sensitivity analysis for
'exposure' on 'Gut microbiota abundance (k_Bacteria.p_Proteobacteria.c_Gammaproteobacteria.o_Pasteurellales) || id:ebi-a-G

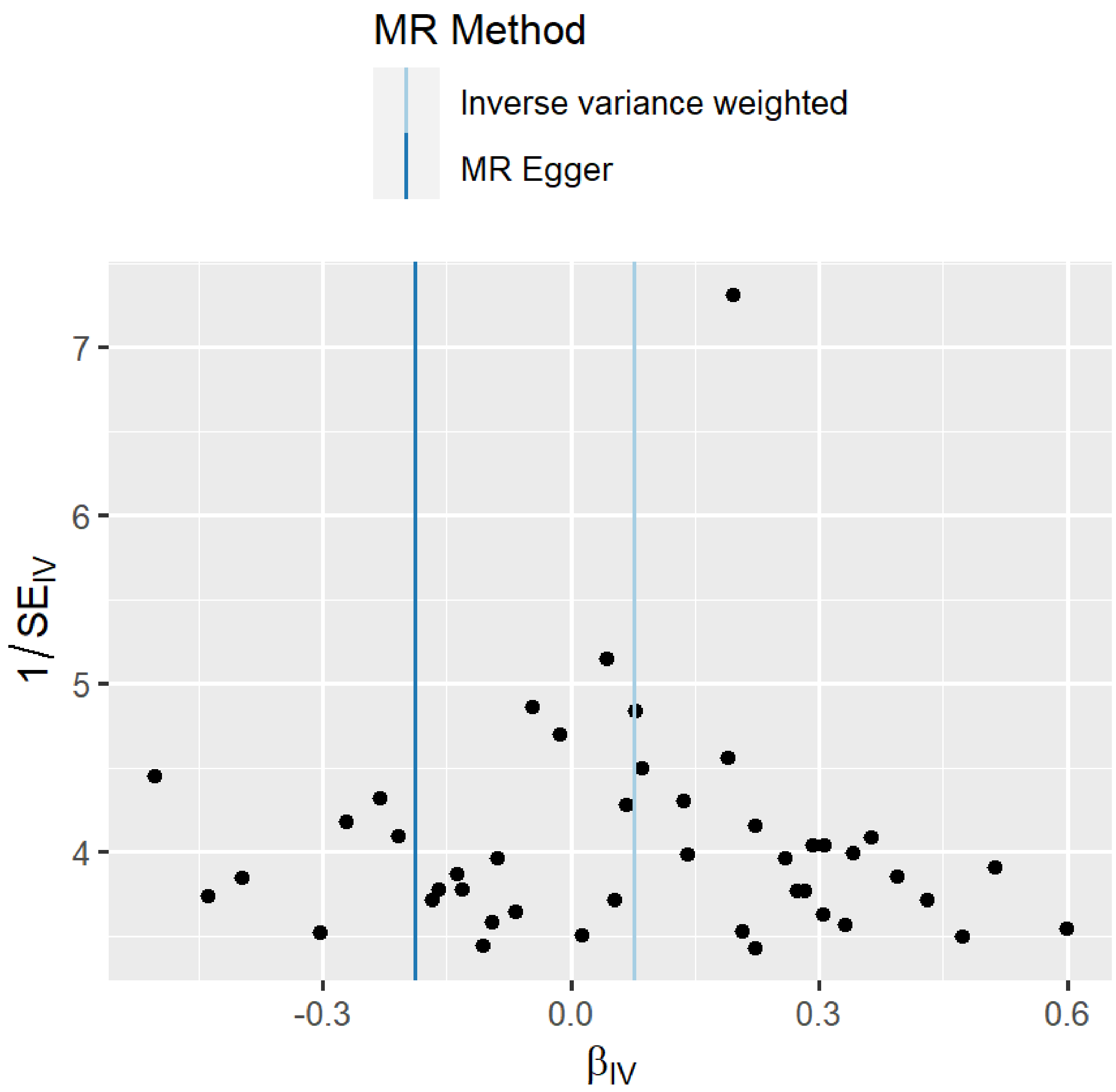
MR Method
Inverse variance weighted
MR Egger
7
6
5
4
-0.3
0.0
0.3
0.6
$1/SE_{IV}$
β_{IV}

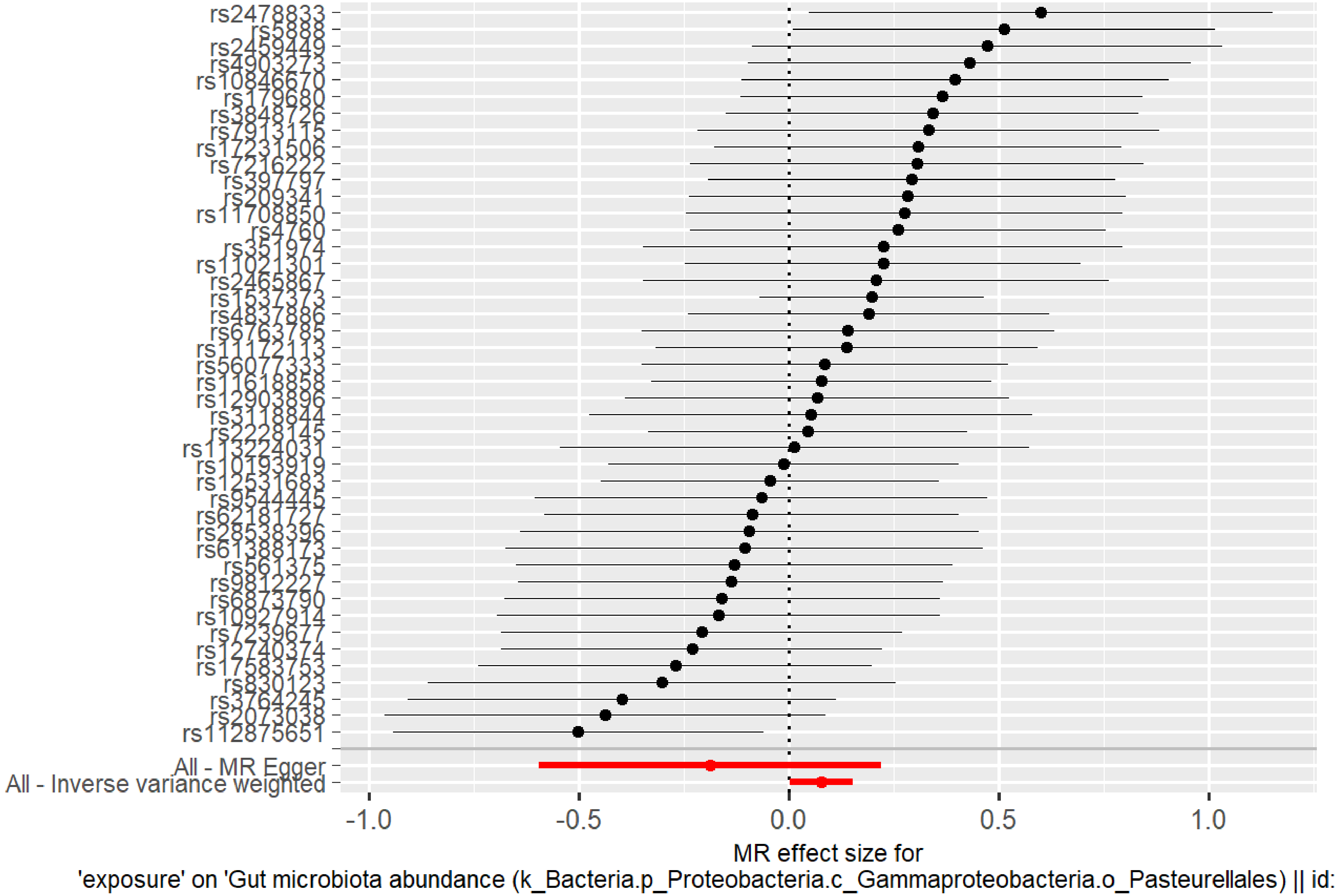
rs2478833
rs5888
rs2459449
rs4903273
rs10846670
rs179680
rs3848726
rs7913115
rs17231506
rs7216222
rs397797
rs209341
rs11708850
rs4760
rs351974
rs11021301
rs2465867
rs1537373
rs4837886
rs6763785
rs11172113
rs56077333
rs11618858
rs12903896
rs3118844
rs2228145
rs113224031
rs10193919
rs12531683
rs9544445
rs62187727
rs28538396
rs61388173
rs561375
rs9812227
rs6873790
rs10927914
rs7239677
rs12740374
rs17583753
rs830123
rs3764245
rs2073038
rs112875651
All - MR Egger
All - Inverse variance weighted
-1.0
-0.5
0.0
0.5
1.0
MR effect size for
'exposure' on 'Gut microbiota abundance (k_Bacteria.p_Proteobacteria.c_Gammaproteobacteria.o_Pasteurellales) || id:

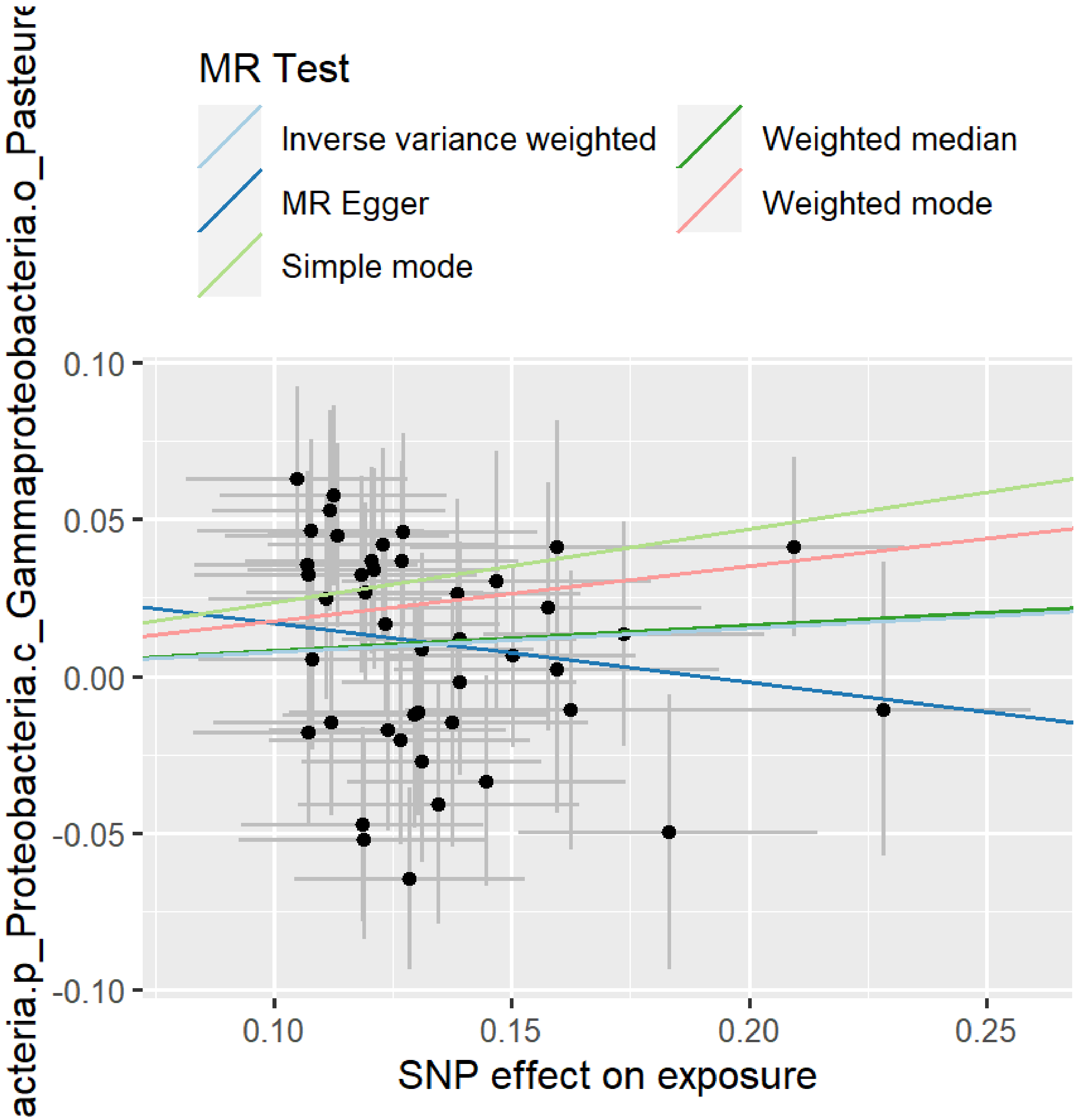

MR Test
Inverse variance weighted
MR Egger
Simple mode
Weighted median
Weighted mode
0.10
0.05
0.00
-0.05
-0.10
0.10
0.15
0.20
0.25
SNP effect on exposure
acteria.p_Proteobacteria.c_Gammaproteobacteria.o_Pasteure

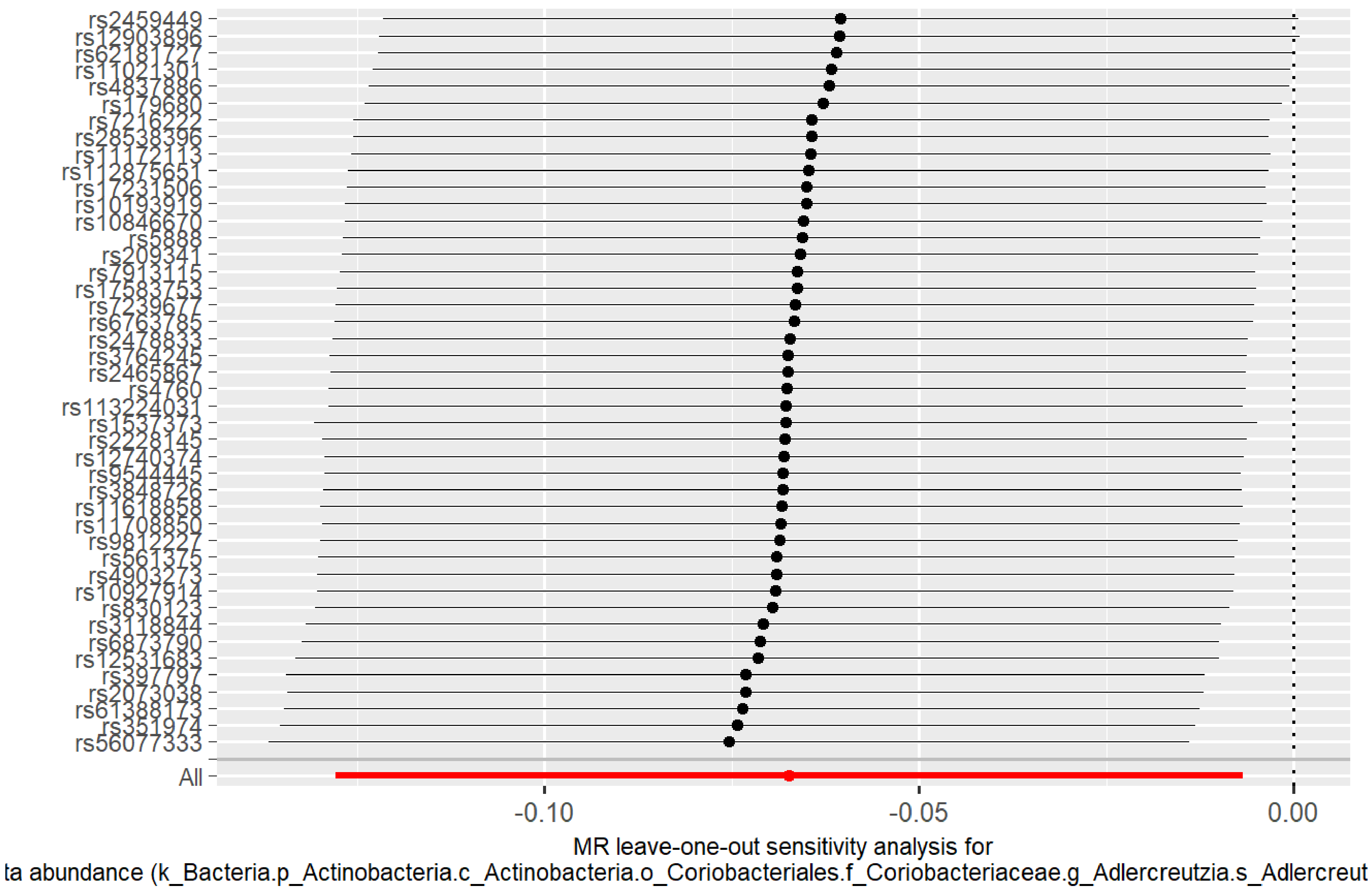

rs2459449
rs12903896
rs62181727
rs11021301
rs4837886
rs179680
rs7216222
rs28538396
rs11172113
rs112875651
rs17231506
rs10193919
rs10846670
rs5888
rs209341
rs7913115
rs17583753
rs7239677
rs6763785
rs2478833
rs3764245
rs2465867
rs4760
rs113224031
rs1537373
rs2228145
rs12740374
rs9544445
rs3848726
rs11618858
rs11708850
rs9812227
rs561375
rs4903273
rs10927914
rs830123
rs3118844
rs6873790
rs12531683
rs397797
rs2073038
rs61388173
rs351974
rs56077333
All
-0.10
-0.05
0.00
MR leave-one-out sensitivity analysis for
ta abundance (k_Bacteria.p_Actinobacteria.c_Actinobacteria.o_Coriobacteriales.f_Coriobacteriaceae.g_Adlercreutzia.s_Adlercreut

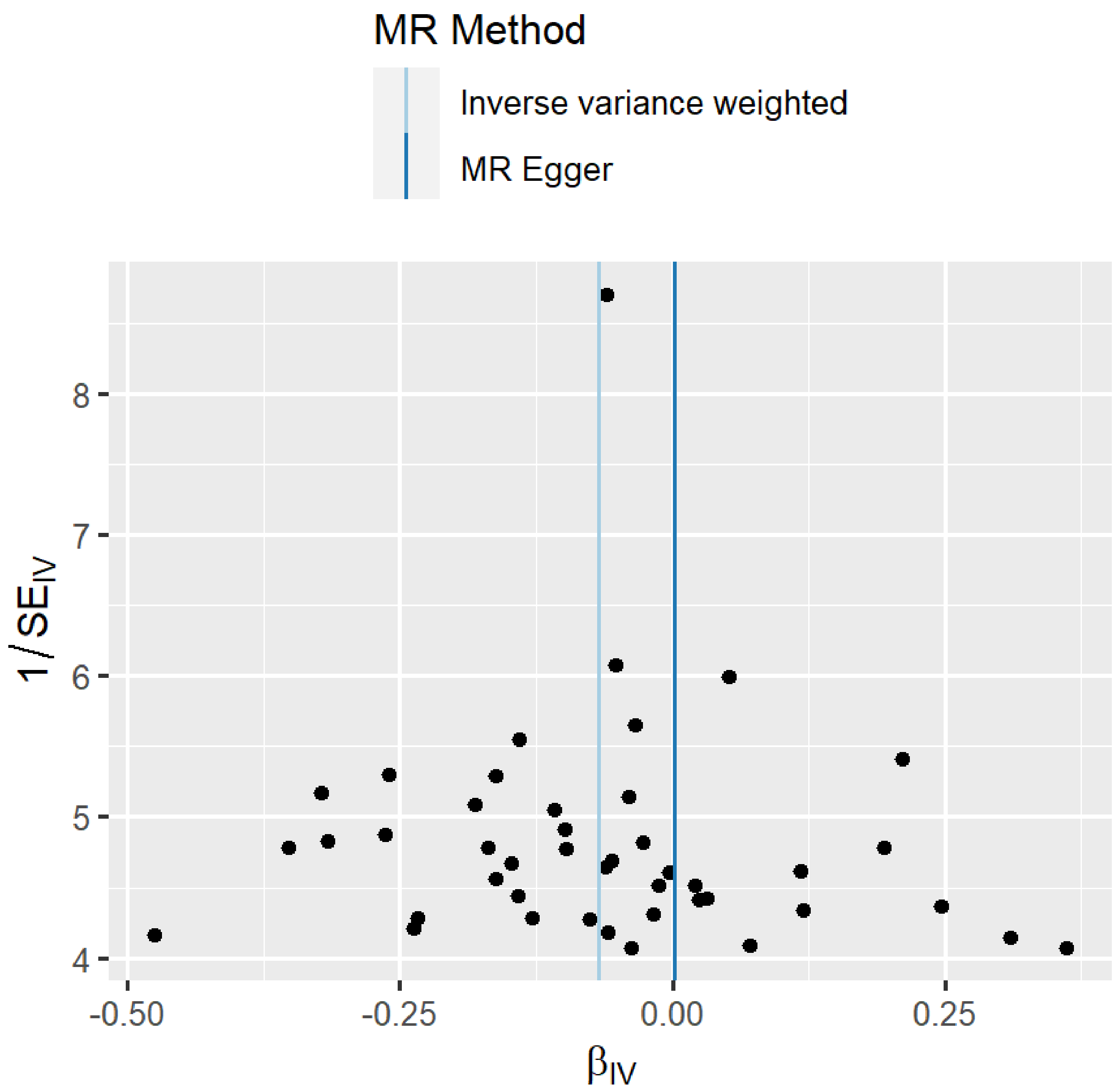

MR Method
Inverse variance weighted
MR Egger
8
7
6
5
4
1/SE_IV
-0.50
-0.25
0.00
0.25
β_IV

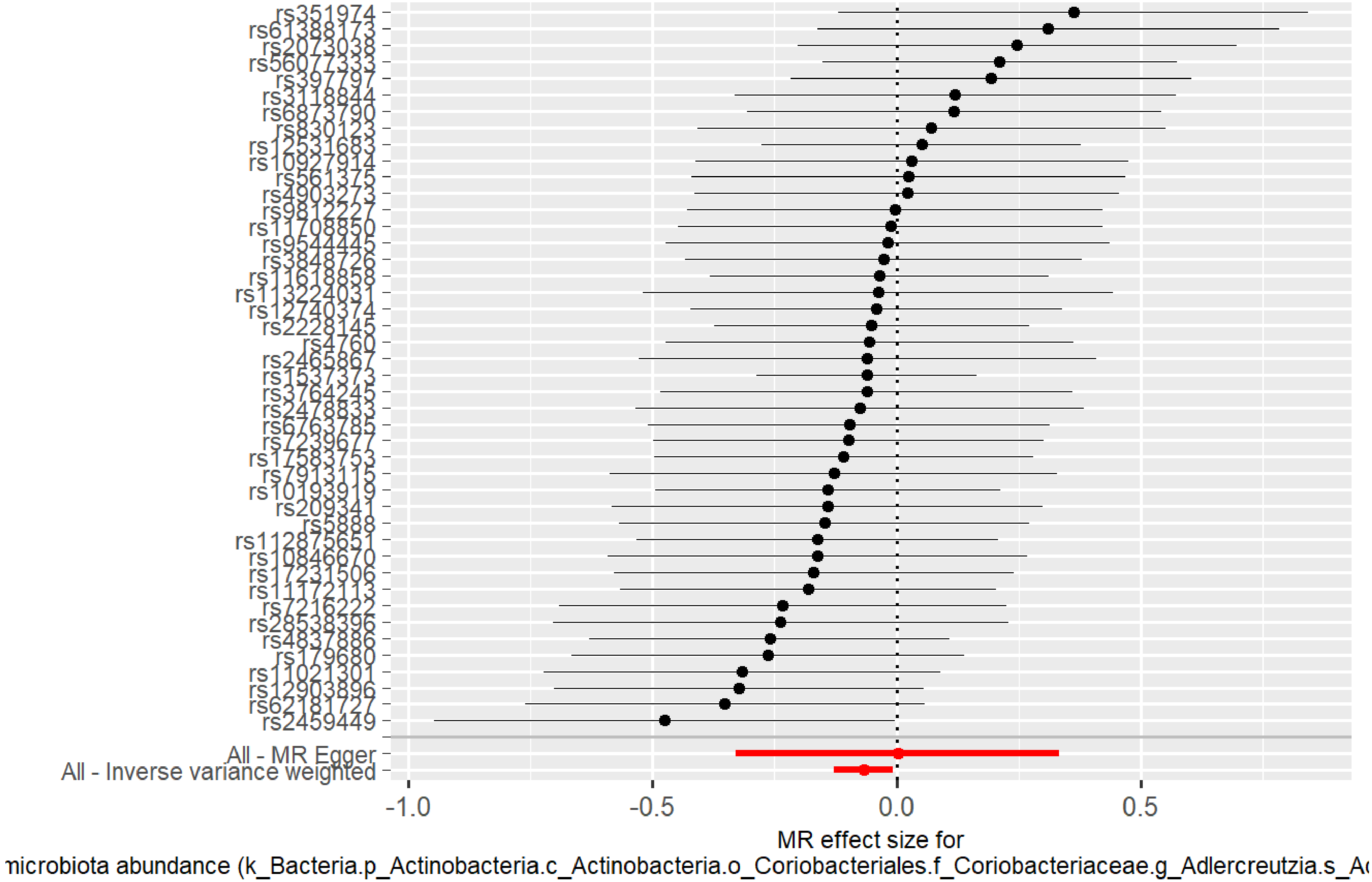
rs351974
rs61388173
rs2073038
rs56077333
rs397797
rs3118844
rs6873790
rs830123
rs12531683
rs10927914
rs561375
rs4903273
rs9812227
rs11708850
rs9544445
rs3848726
rs11618858
rs113224031
rs12740374
rs2228145
rs4760
rs2465867
rs1537373
rs3764245
rs2478833
rs6763785
rs7239677
rs17583753
rs7913115
rs10193919
rs209341
rs5888
rs112875651
rs10846670
rs17231506
rs11172113
rs7216222
rs28538396
rs4837886
rs179680
rs11021301
rs12903896
rs62181727
rs2459449
All - MR Egger
All - Inverse variance weighted
-1.0
-0.5
0.0
0.5
MR effect size for
microbiota abundance (k_Bacteria.p_Actinobacteria.c_Actinobacteria.o_Coriobacteriales.f_Coriobacteriaceae.g_Adlercreutzia.s_A

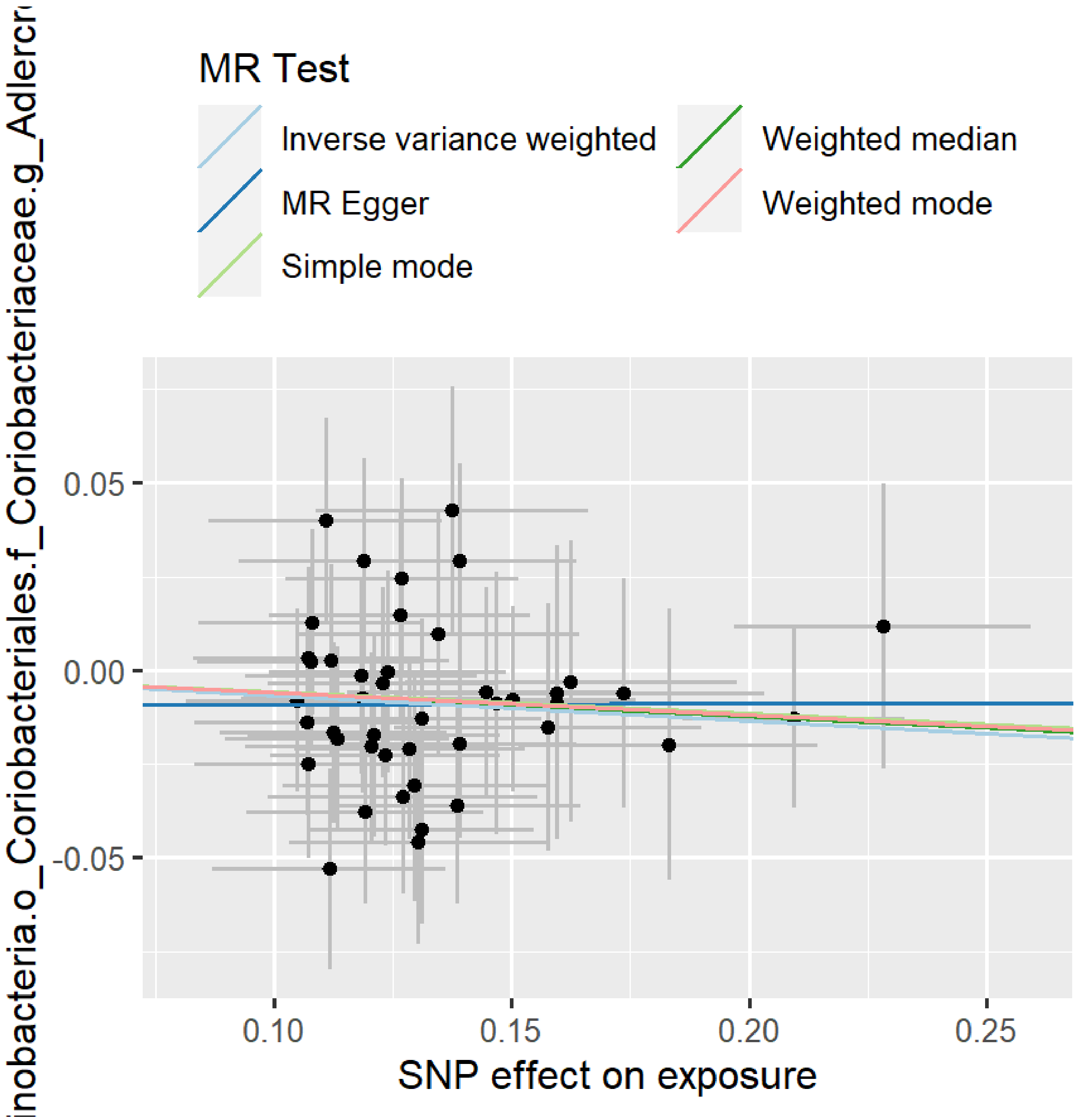

MR Test
Inverse variance weighted
MR Egger
Simple mode
Weighted median
Weighted mode
inobacteria.o_Coriobacteriales.f_Coriobacteriaceae.g_Adlercr
0.05
0.00
-0.05
0.10
0.15
0.20
0.25
SNP effect on exposure

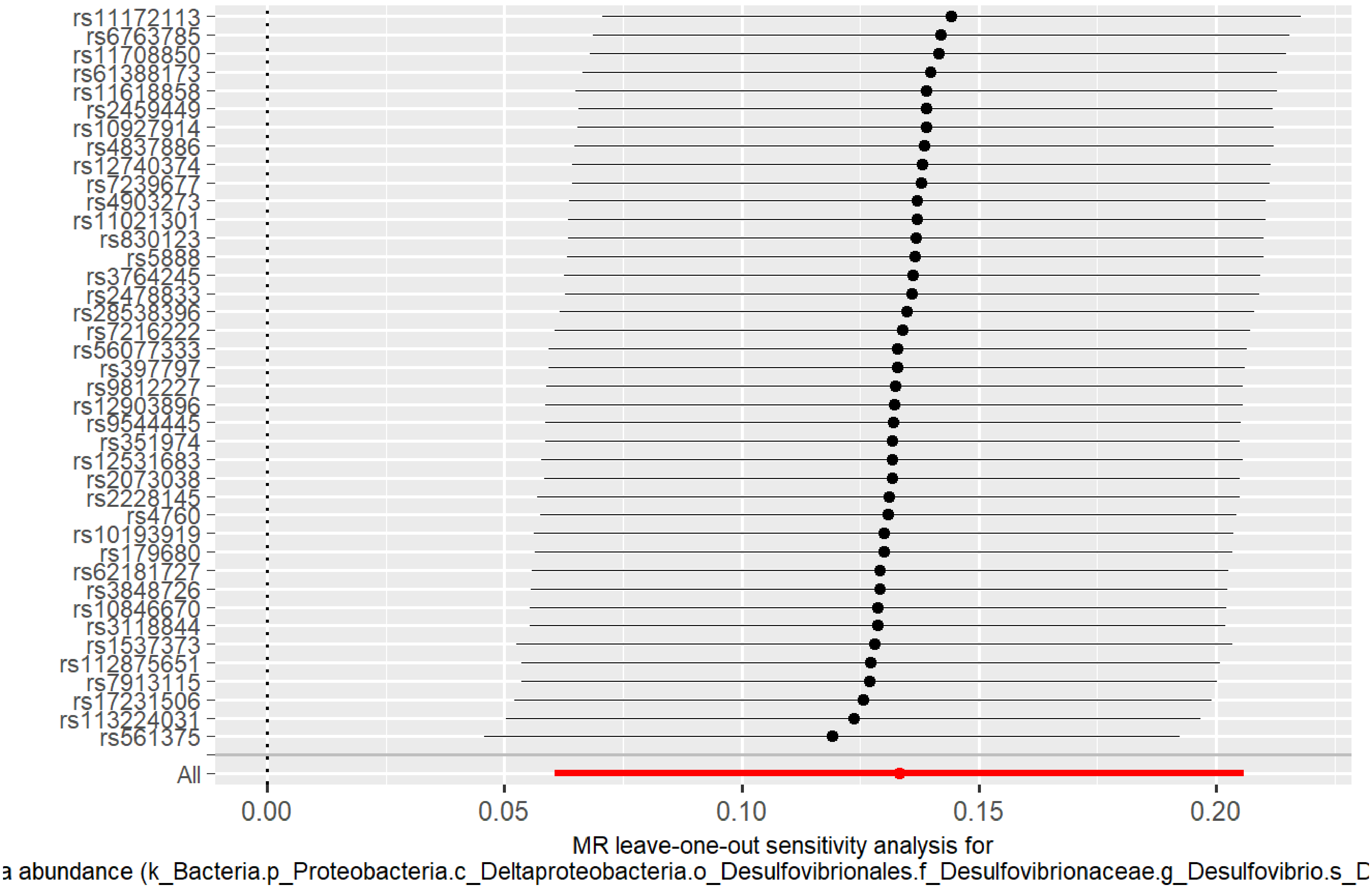

rs11172113
rs6763785
rs11708850
rs61388173
rs11618858
rs2459449
rs10927914
rs4837886
rs12740374
rs7239677
rs4903273
rs11021301
rs830123
rs5888
rs3764245
rs2478833
rs28538396
rs7216222
rs56077333
rs397797
rs9812227
rs12903896
rs9544445
rs351974
rs12531683
rs2073038
rs2228145
rs4760
rs10193919
rs179680
rs62181727
rs3848726
rs10846670
rs3118844
rs1537373
rs112875651
rs7913115
rs17231506
rs113224031
rs561375
All
0.00
0.05
0.10
0.15
0.20
MR leave-one-out sensitivity analysis for
a abundance (k_Bacteria.p_Proteobacteria.c_Deltaproteobacteria.o_Desulfovibrionales.f_Desulfovibrionaceae.g_Desulfovibrio.s_D

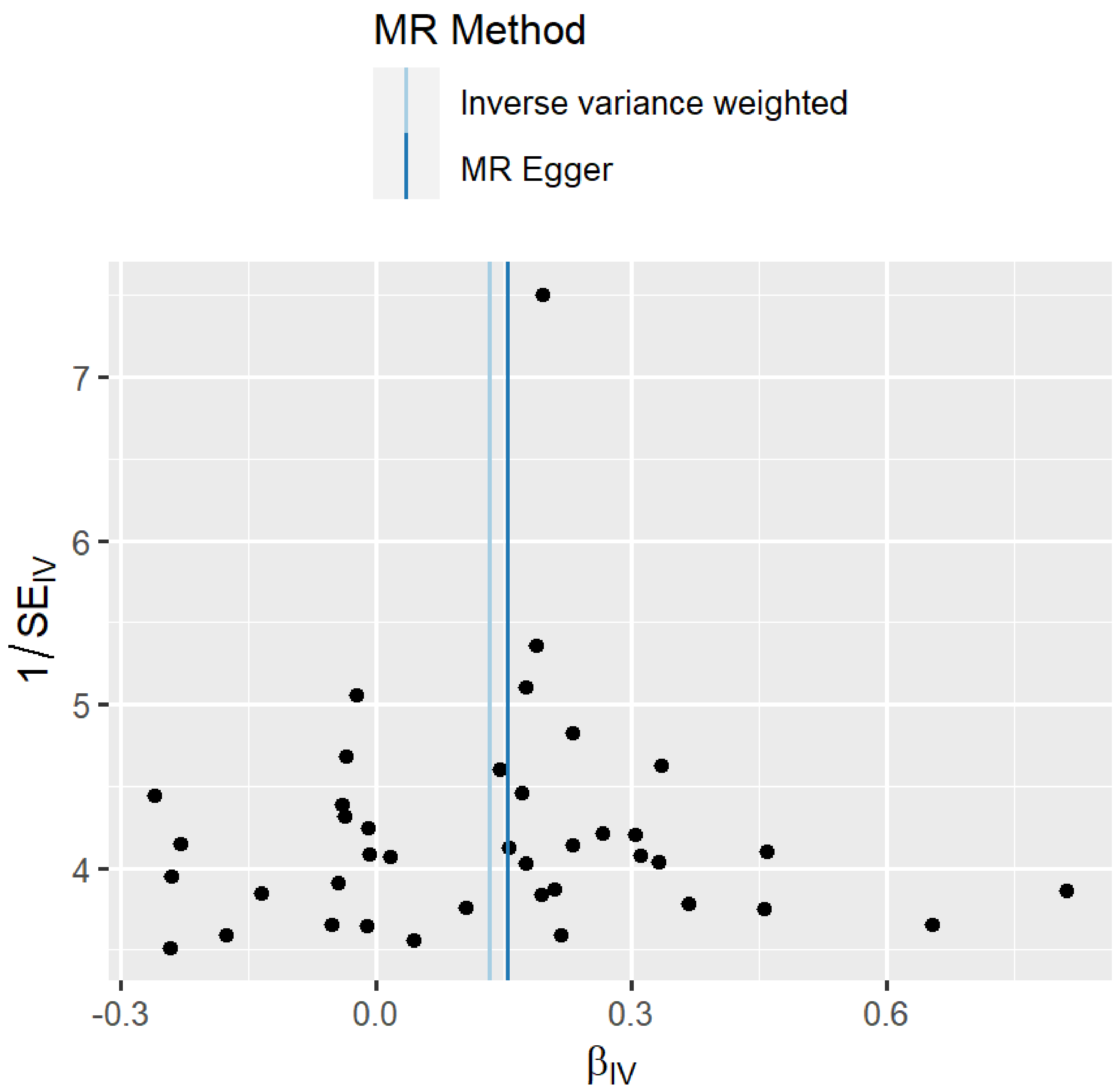
MR Method
Inverse variance weighted
MR Egger
$1/SE_{IV}$
7
6
5
4
-0.3
0.0
0.3
0.6
β_{IV}

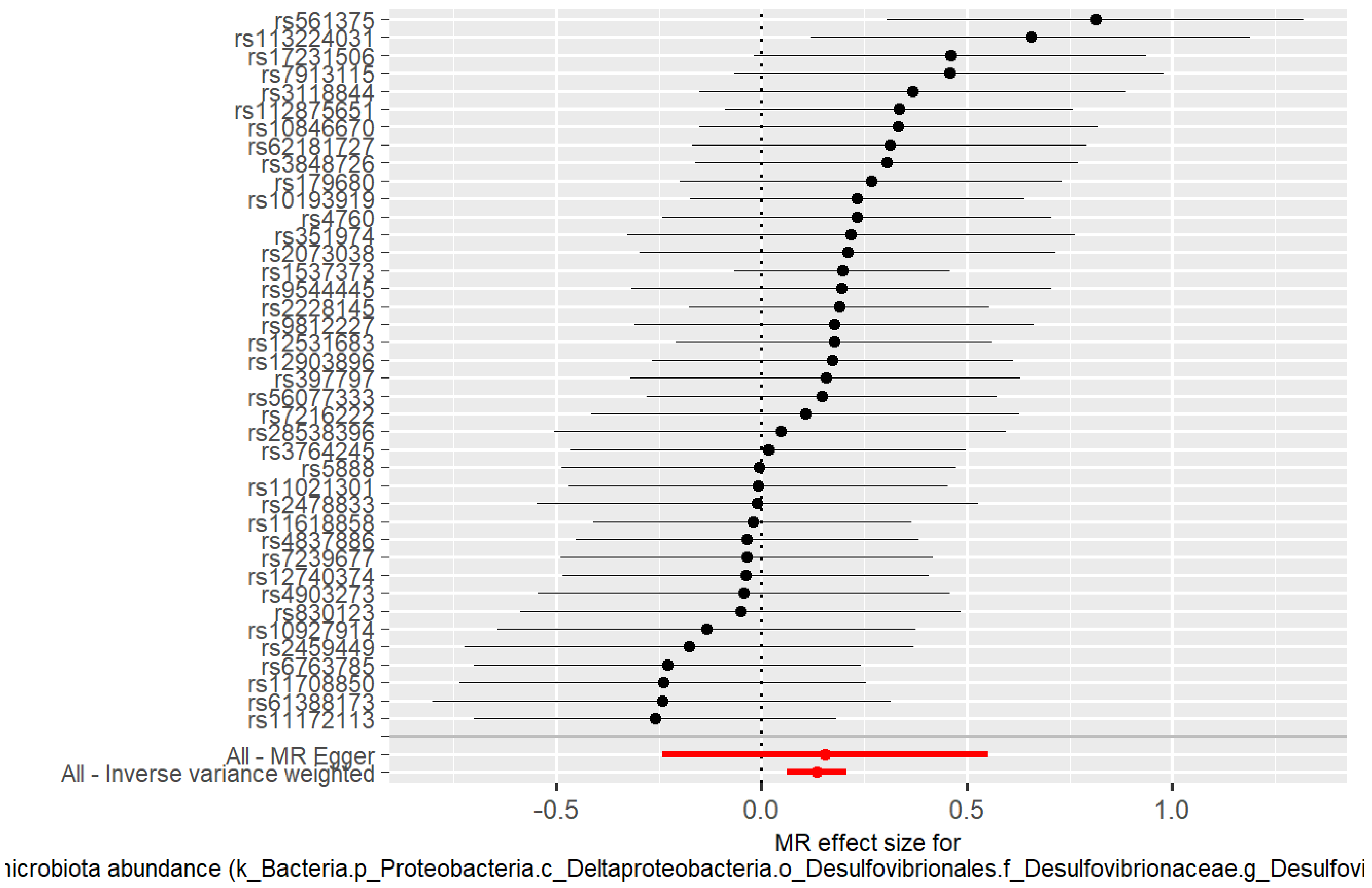
rs561375
rs113224031
rs17231506
rs7913115
rs3118844
rs112875651
rs10846670
rs62181727
rs3848726
rs179680
rs10193919
rs4760
rs351974
rs2073038
rs1537373
rs9544445
rs2228145
rs9812227
rs12531683
rs12903896
rs397797
rs56077333
rs7216222
rs28538396
rs3764245
rs5888
rs11021301
rs2478833
rs11618858
rs4837886
rs7239677
rs12740374
rs4903273
rs830123
rs10927914
rs2459449
rs6763785
rs11708850
rs61388173
rs11172113
All - MR Egger
All - Inverse variance weighted
-0.5
0.0
0.5
1.0
MR effect size for
ıicrobiota abundance (k_Bacteria.p_Proteobacteria.c_Deltaproteobacteria.o_Desulfovibrionales.f_Desulfovibrionaceae.g_Desulfovi

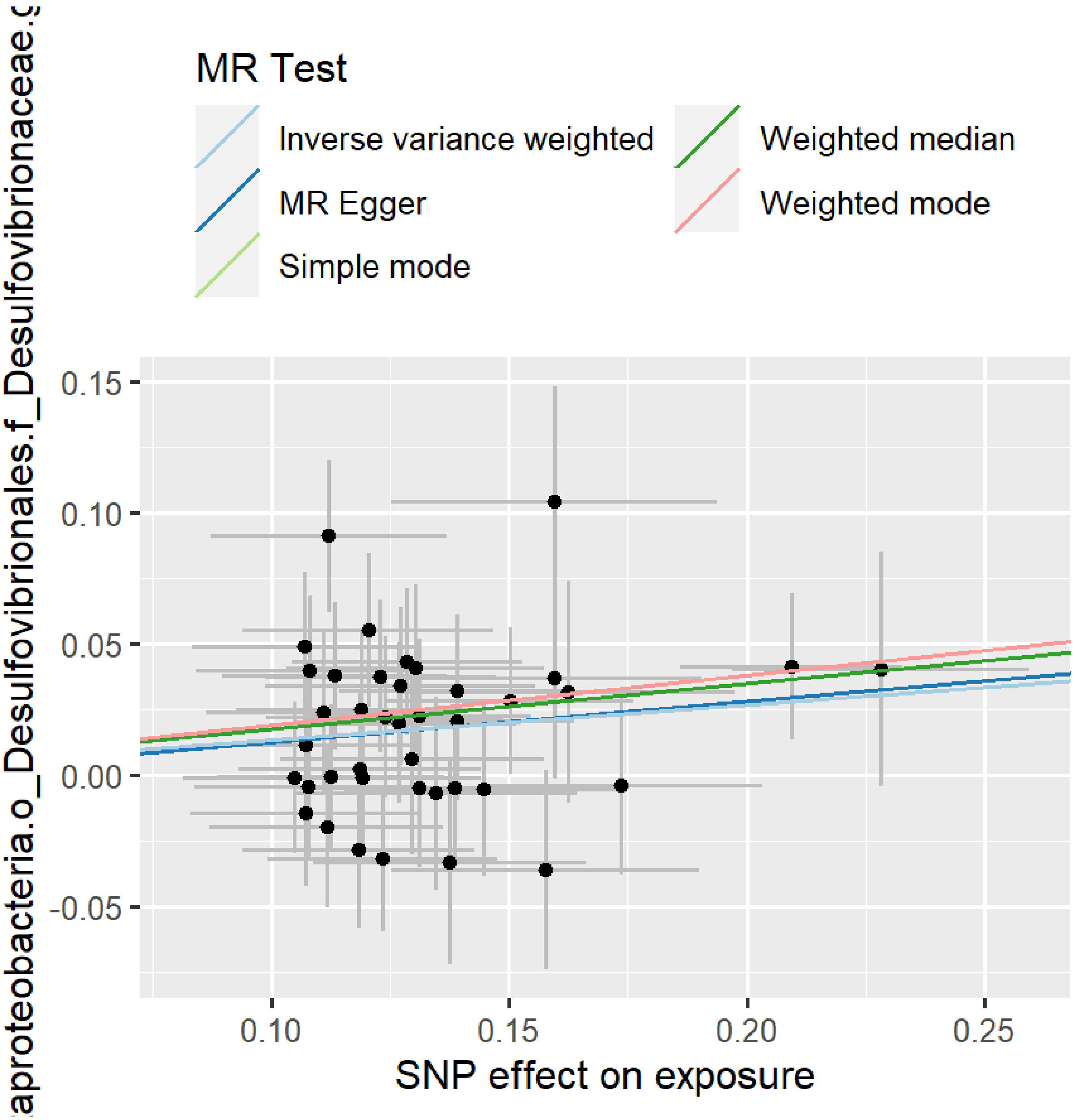
MR Test
Inverse variance weighted
MR Egger
Simple mode
Weighted median
Weighted mode
0.15
0.10
0.05
0.00
-0.05
0.10
0.15
0.20
0.25
SNP effect on exposure
aproteobacteria.o_Desulfovibrionales.f_Desulfovibrionaceae.g

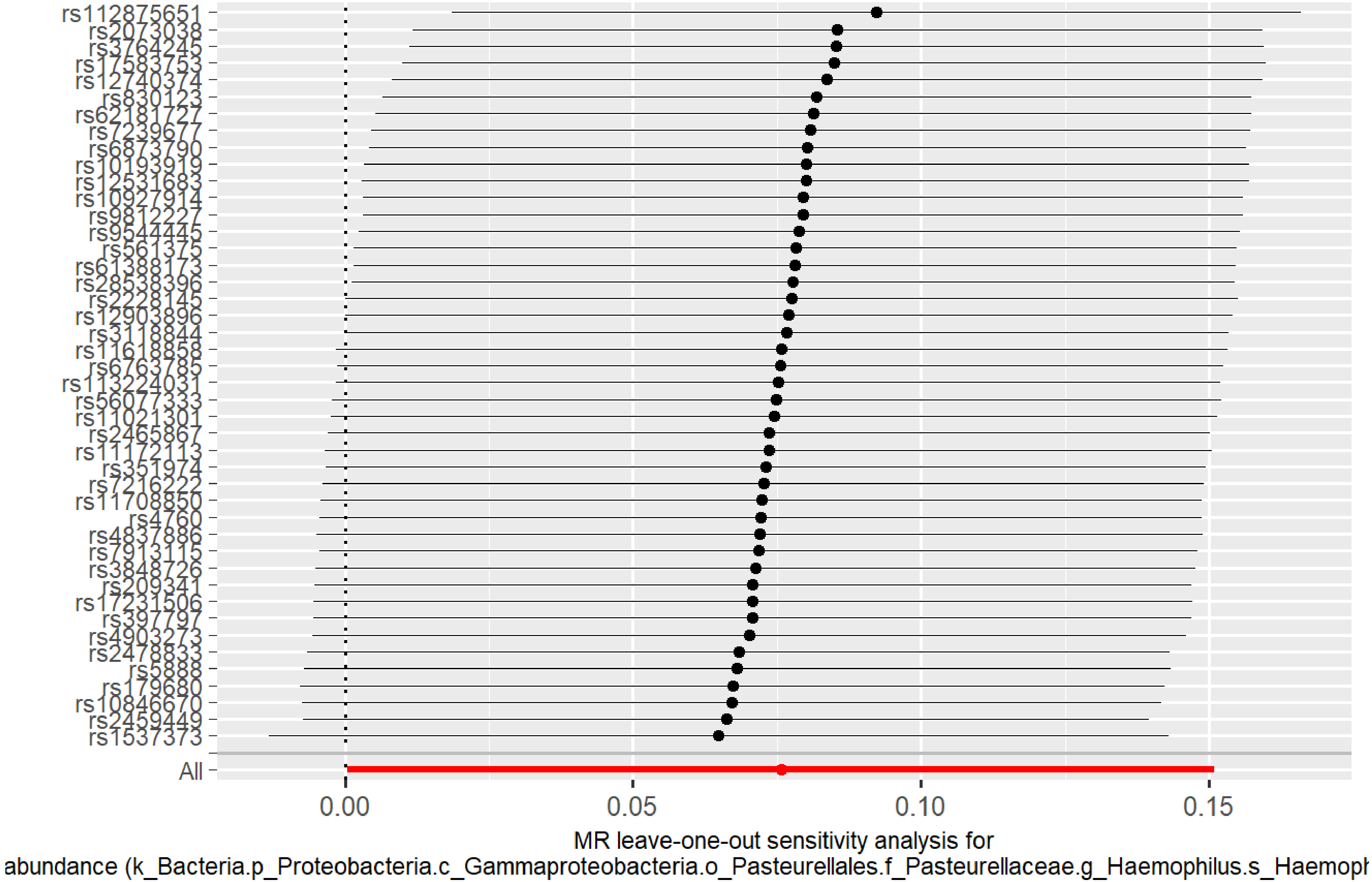
rs112875651
rs2073038
rs3764245
rs17583753
rs12740374
rs830123
rs62181727
rs7239677
rs6873790
rs10193919
rs12531683
rs10927914
rs9812227
rs9544445
rs561375
rs61388173
rs28538396
rs2228145
rs12903896
rs3118844
rs11618858
rs6763785
rs113224031
rs56077333
rs11021301
rs2465867
rs11172113
rs351974
rs7216222
rs11708850
rs4760
rs4837886
rs7913115
rs3848726
rs209341
rs17231506
rs397797
rs4903273
rs2478833
rs5888
rs179680
rs10846670
rs2459449
rs1537373
All
0.00
0.05
0.10
0.15
MR leave-one-out sensitivity analysis for
abundance (k_Bacteria.p_Proteobacteria.c_Gammaproteobacteria.o_Pasteurellales.f_Pasteurellaceae.g_Haemophilus.s_Haemopl

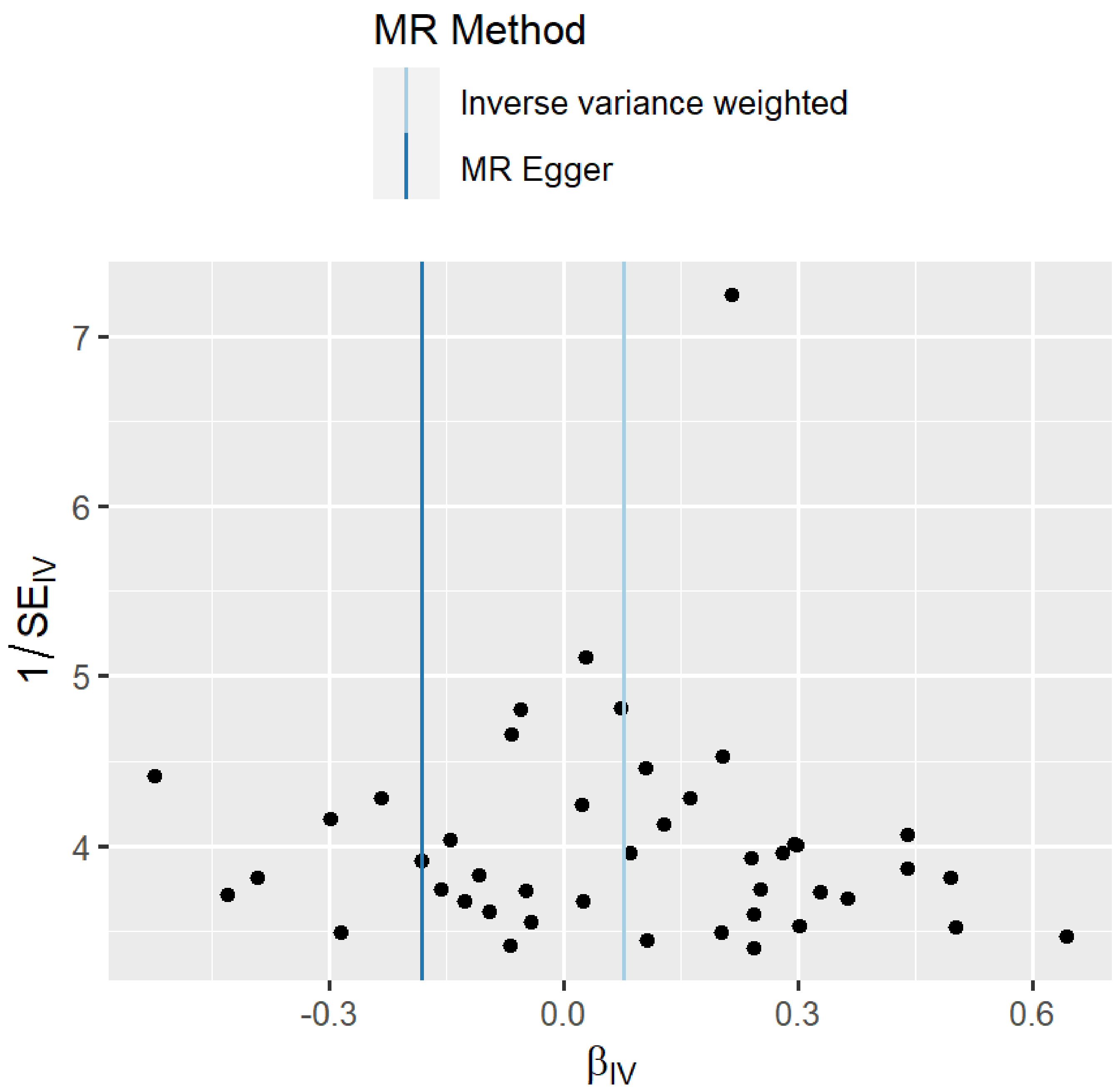
MR Method
Inverse variance weighted
MR Egger
7
6
5
4
-0.3
0.0
0.3
0.6
1/SE_IV
β_IV

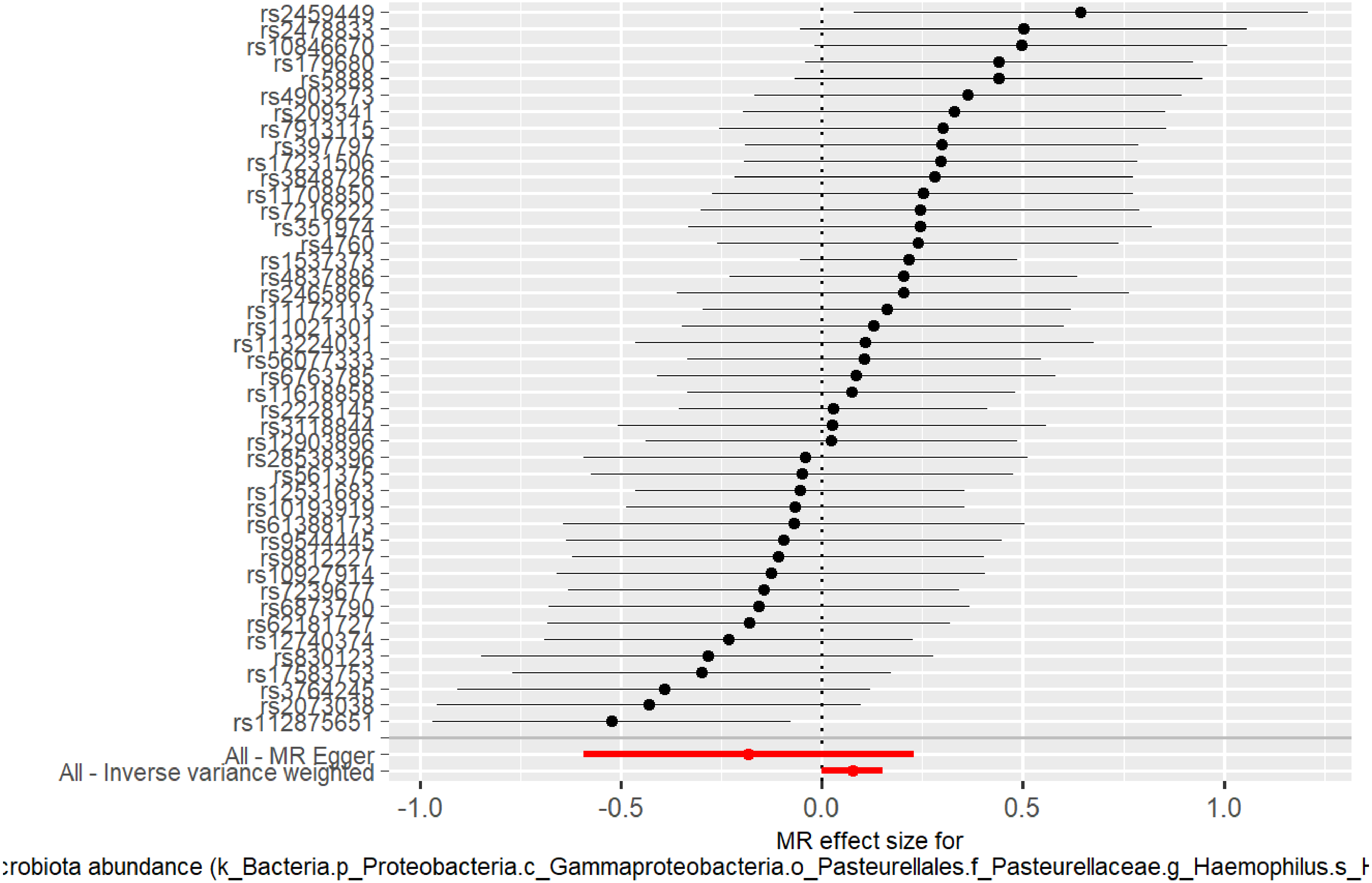
rs2459449
rs2478833
rs10846670
rs179680
rs5888
rs4903273
rs209341
rs7913115
rs397797
rs17231506
rs3848726
rs11708850
rs7216222
rs351974
rs4760
rs1537373
rs4837886
rs2465867
rs11172113
rs11021301
rs113224031
rs56077333
rs6763785
rs11618858
rs2228145
rs3118844
rs12903896
rs28538396
rs561375
rs12531683
rs10193919
rs61388173
rs9544445
rs9812227
rs10927914
rs7239677
rs6873790
rs62181727
rs12740374
rs830123
rs17583753
rs3764245
rs2073038
rs112875651
All - MR Egger
All - Inverse variance weighted
-1.0
-0.5
0.0
0.5
1.0
MR effect size for
robiota abundance (k_Bacteria.p_Proteobacteria.c_Gammaproteobacteria.o_Pasteurellales.f_Pasteurellaceae.g_Haemophilus.s_

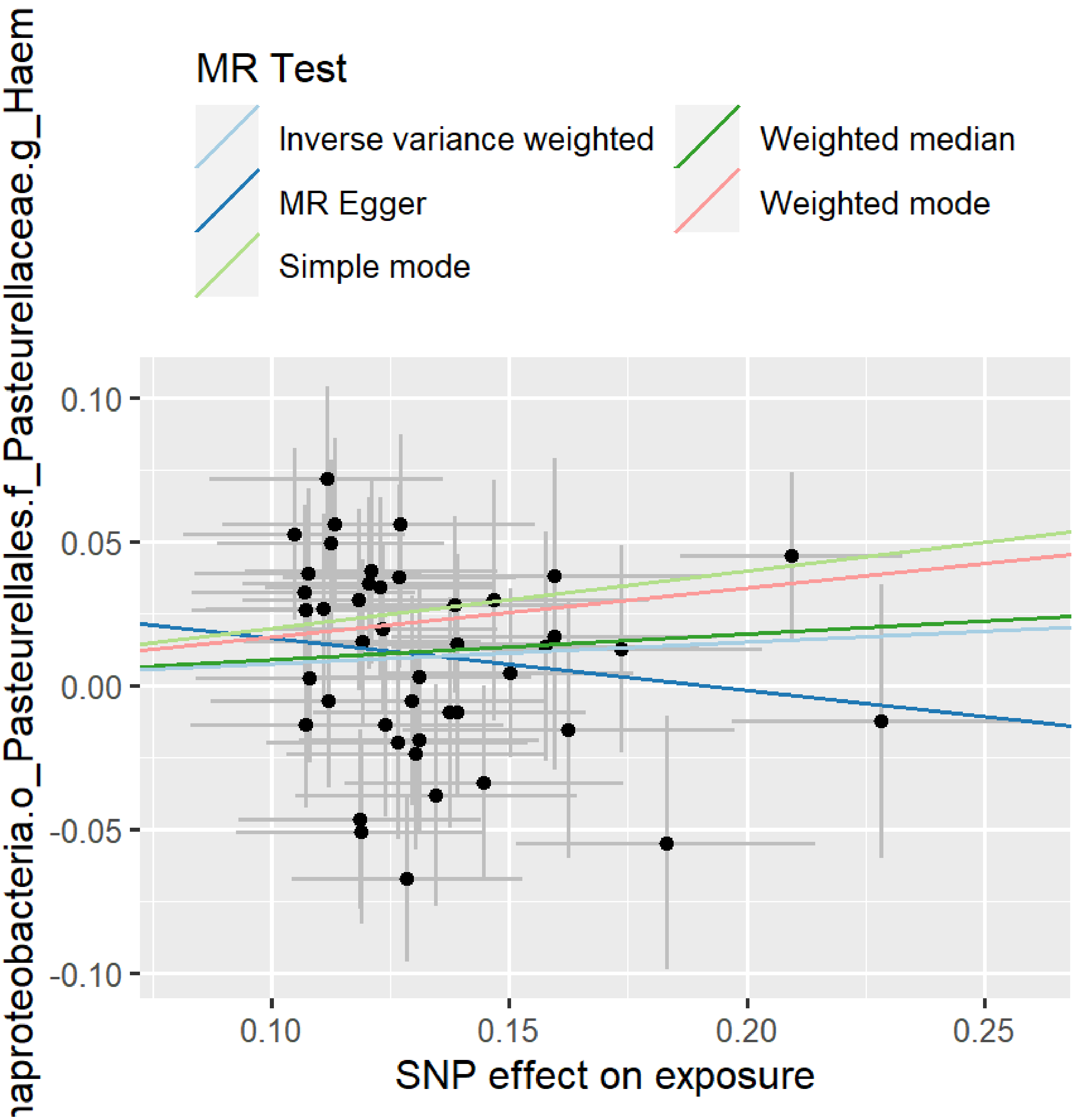
MR Test
Inverse variance weighted
MR Egger
Simple mode
Weighted median
Weighted mode
0.10
0.05
0.00
-0.05
-0.10
0.10
0.15
0.20
0.25
SNP effect on exposure
aproteobacteria.o_Pasteurellales.f_Pasteurellaceae.g_Haem

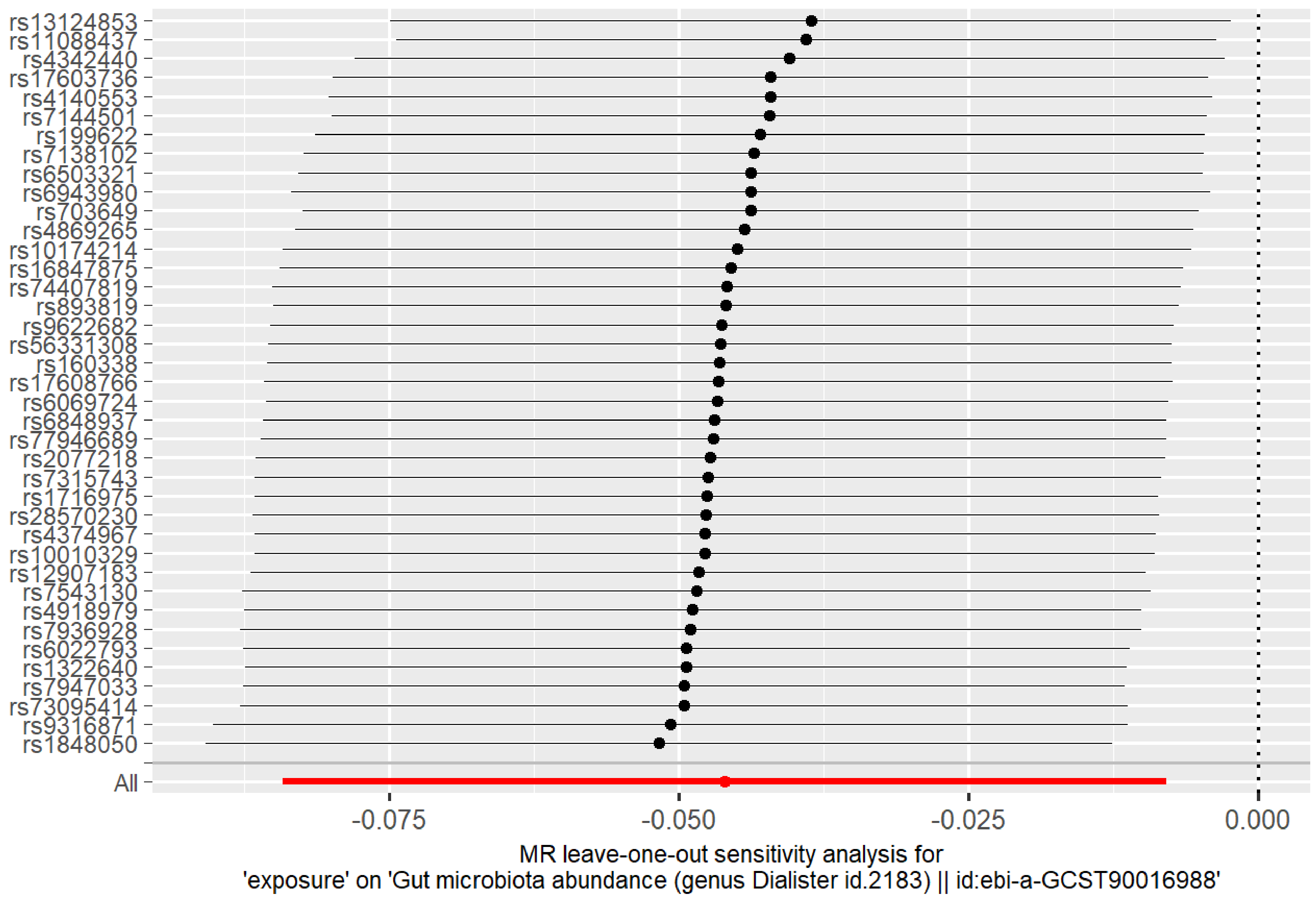
rs13124853
rs11088437
rs4342440
rs17603736
rs4140553
rs7144501
rs199622
rs7138102
rs6503321
rs6943980
rs703649
rs4869265
rs10174214
rs16847875
rs74407819
rs893819
rs9622682
rs56331308
rs160338
rs17608766
rs6069724
rs6848937
rs77946689
rs2077218
rs7315743
rs1716975
rs28570230
rs4374967
rs10010329
rs12907183
rs7543130
rs4918979
rs7936928
rs6022793
rs1322640
rs7947033
rs73095414
rs9316871
rs1848050
All
-0.075
-0.050
-0.025
0.000
MR leave-one-out sensitivity analysis for
'exposure' on 'Gut microbiota abundance (genus Dialister id.2183) || id:ebi-a-GCST90016988'

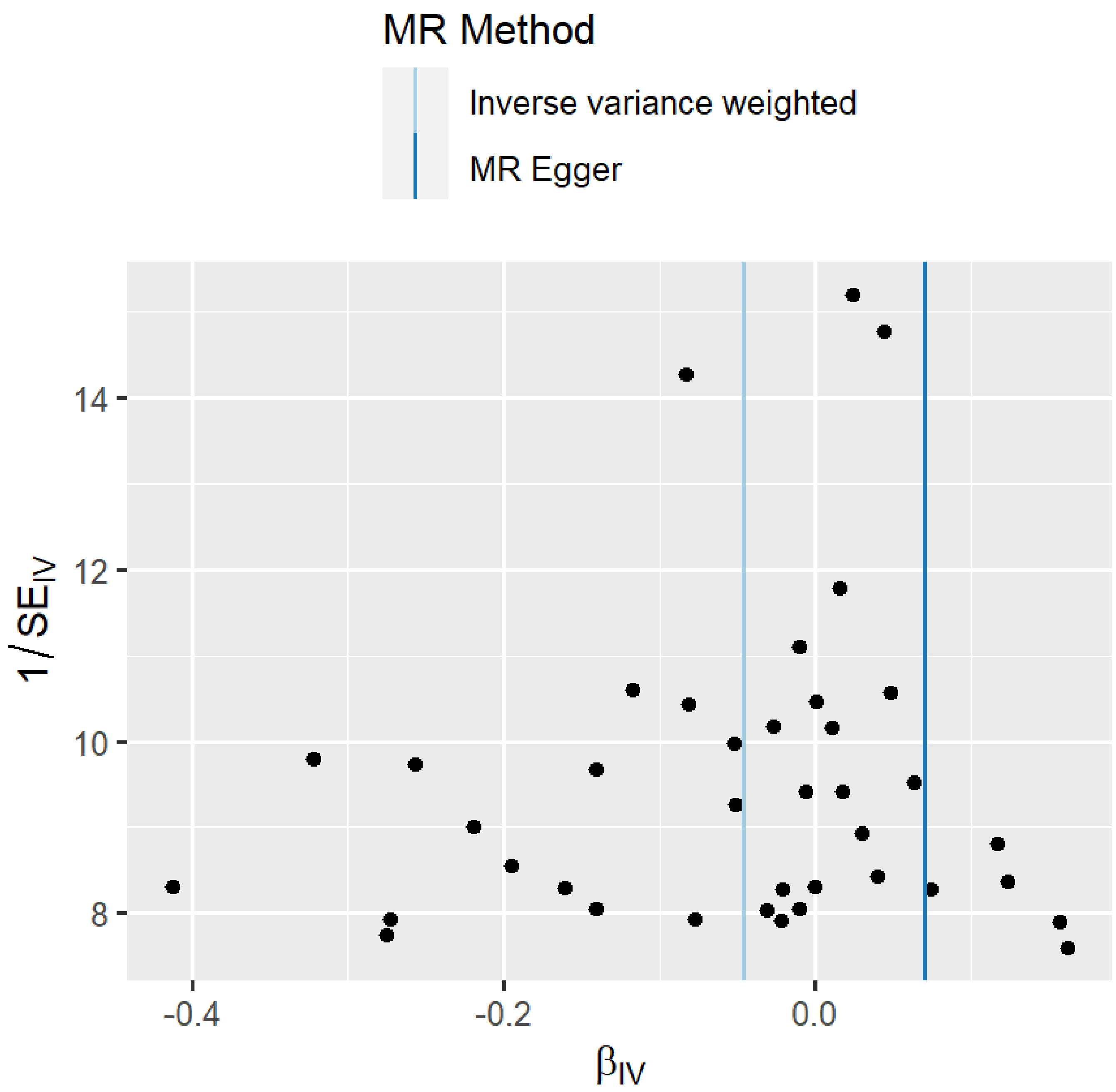
MR Method
Inverse variance weighted
MR Egger
1/SE$_{IV}$
14
12
10
8
-0.4
-0.2
0.0
β$_{IV}$

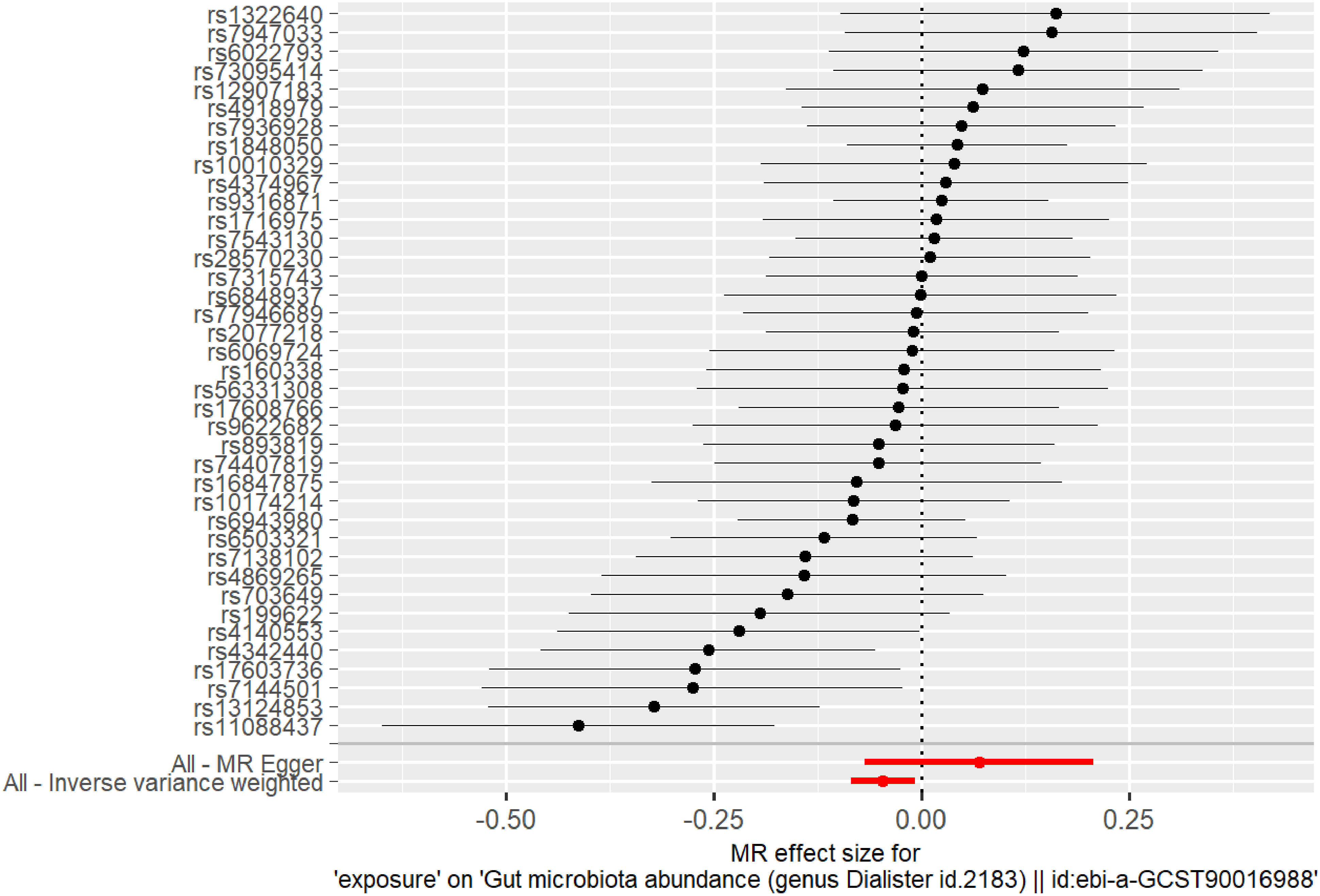
rs1322640
rs7947033
rs6022793
rs73095414
rs12907183
rs4918979
rs7936928
rs1848050
rs10010329
rs4374967
rs9316871
rs1716975
rs7543130
rs28570230
rs7315743
rs6848937
rs77946689
rs2077218
rs6069724
rs160338
rs56331308
rs17608766
rs9622682
rs893819
rs74407819
rs16847875
rs10174214
rs6943980
rs6503321
rs7138102
rs4869265
rs703649
rs199622
rs4140553
rs4342440
rs17603736
rs7144501
rs13124853
rs11088437
All - MR Egger
All - Inverse variance weighted
-0.50
-0.25
0.00
0.25
MR effect size for
'exposure' on 'Gut microbiota abundance (genus Dialister id.2183) || id:ebi-a-GCST90016988'

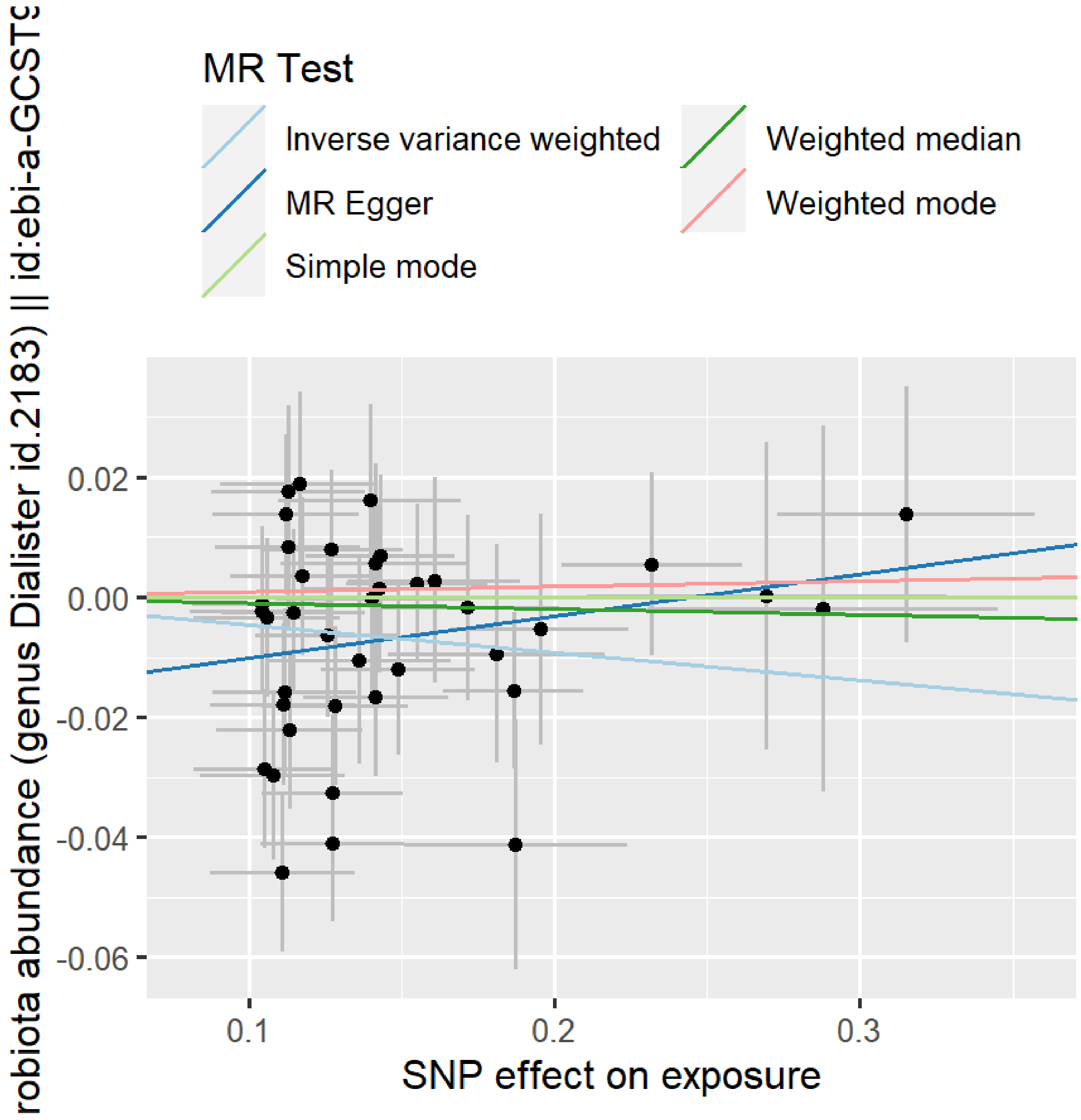
MR Test
Inverse variance weighted
MR Egger
Simple mode
Weighted median
Weighted mode
robiota abundance (genus Dialister id.2183) || id:ebi-a-GCST
0.02
0.00
-0.02
-0.04
-0.06
0.1
0.2
0.3
SNP effect on exposure

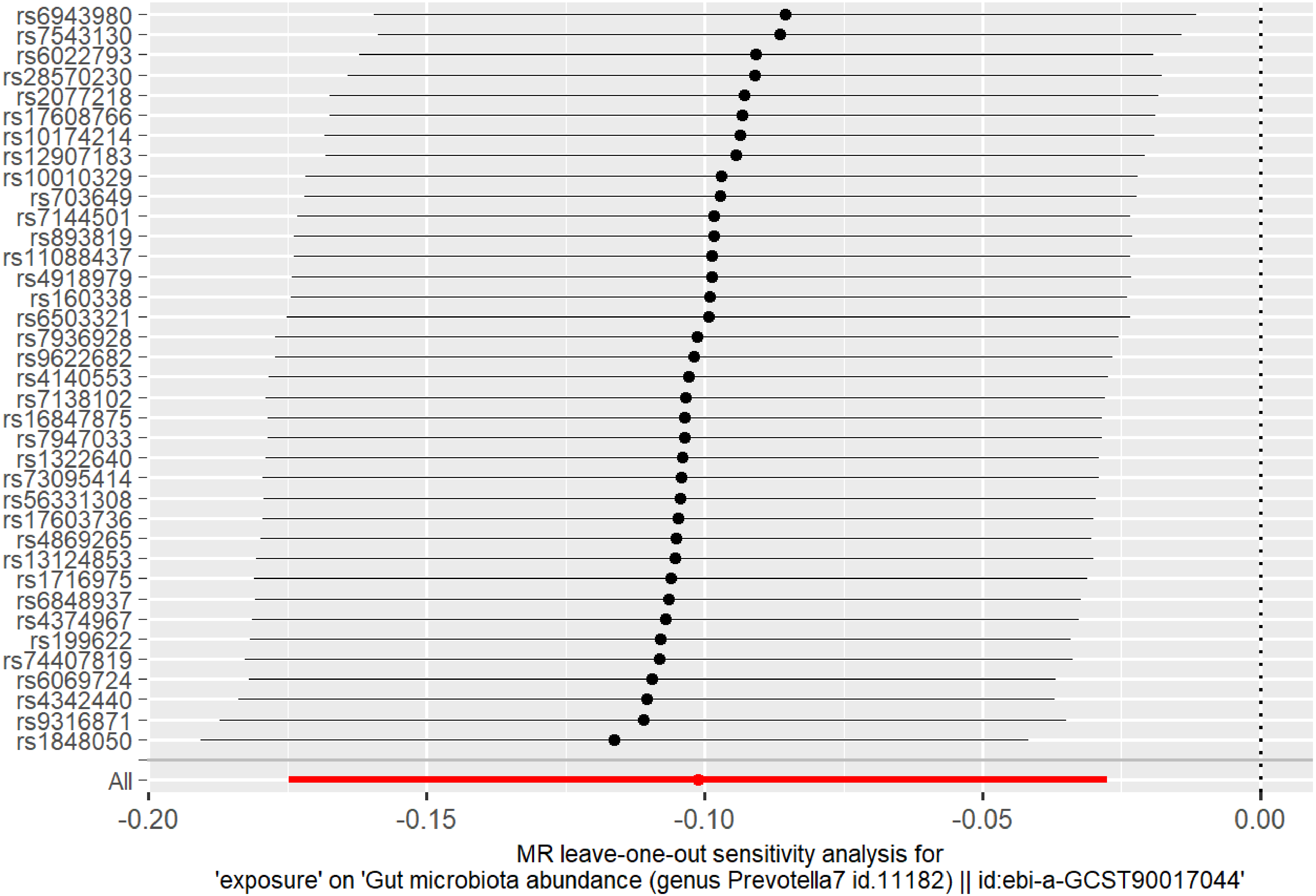
rs6943980
rs7543130
rs6022793
rs28570230
rs2077218
rs17608766
rs10174214
rs12907183
rs10010329
rs703649
rs7144501
rs893819
rs11088437
rs4918979
rs160338
rs6503321
rs7936928
rs9622682
rs4140553
rs7138102
rs16847875
rs7947033
rs1322640
rs73095414
rs56331308
rs17603736
rs4869265
rs13124853
rs1716975
rs6848937
rs4374967
rs199622
rs74407819
rs6069724
rs4342440
rs9316871
rs1848050
All
-0.20
-0.15
-0.10
-0.05
0.00
MR leave-one-out sensitivity analysis for
'exposure' on 'Gut microbiota abundance (genus Prevotella7 id.11182) || id:ebi-a-GCST90017044'

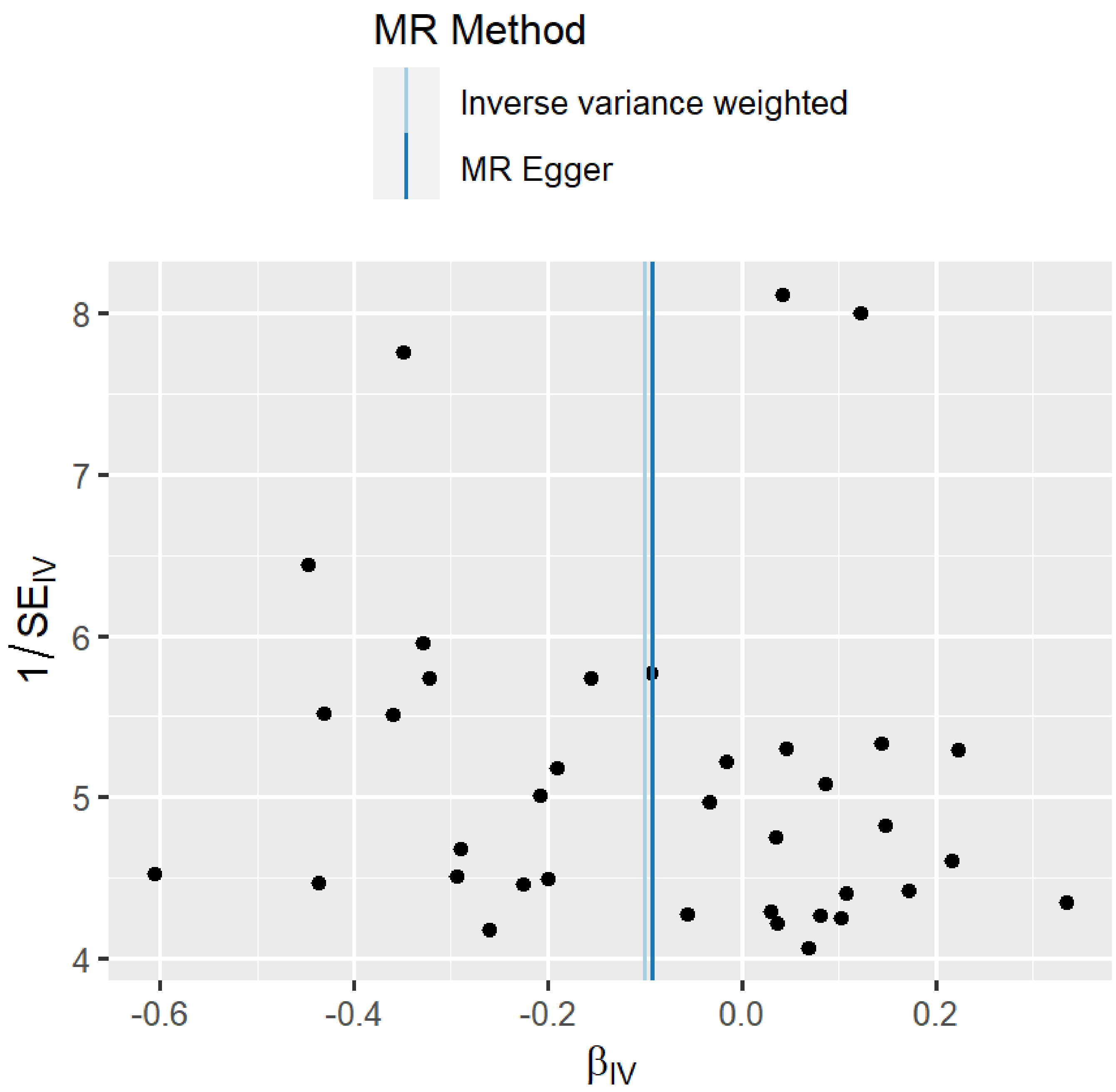
MR Method
Inverse variance weighted
MR Egger
8
7
6
5
4
1/SE_IV
-0.6
-0.4
-0.2
0.0
0.2
β_IV

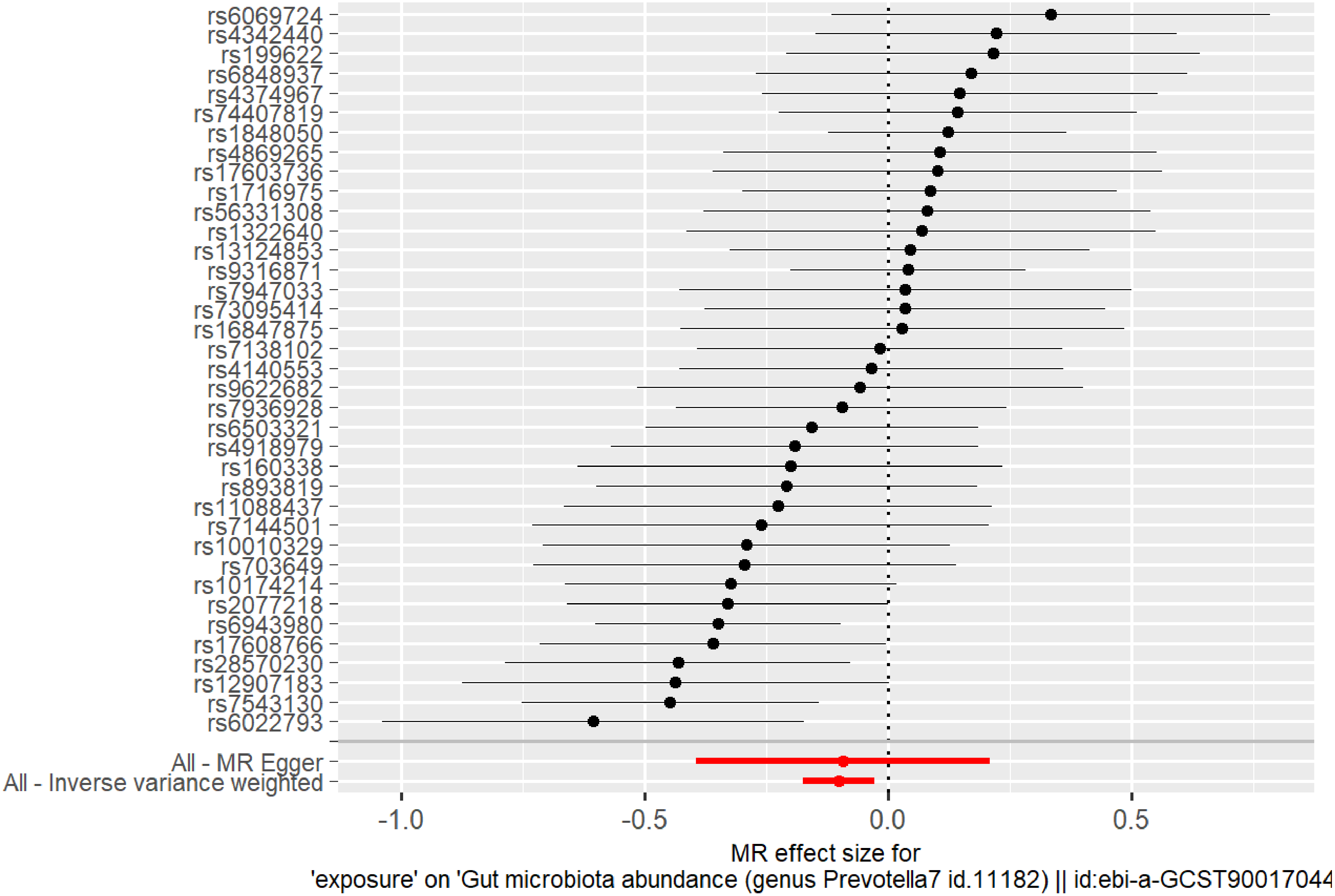
rs6069724
rs4342440
rs199622
rs6848937
rs4374967
rs74407819
rs1848050
rs4869265
rs17603736
rs1716975
rs56331308
rs1322640
rs13124853
rs9316871
rs7947033
rs73095414
rs16847875
rs7138102
rs4140553
rs9622682
rs7936928
rs6503321
rs4918979
rs160338
rs893819
rs11088437
rs7144501
rs10010329
rs703649
rs10174214
rs2077218
rs6943980
rs17608766
rs28570230
rs12907183
rs7543130
rs6022793
All - MR Egger
All - Inverse variance weighted
-1.0
-0.5
0.0
0.5
MR effect size for
'exposure' on 'Gut microbiota abundance (genus Prevotella7 id.11182) || id:ebi-a-GCST90017044

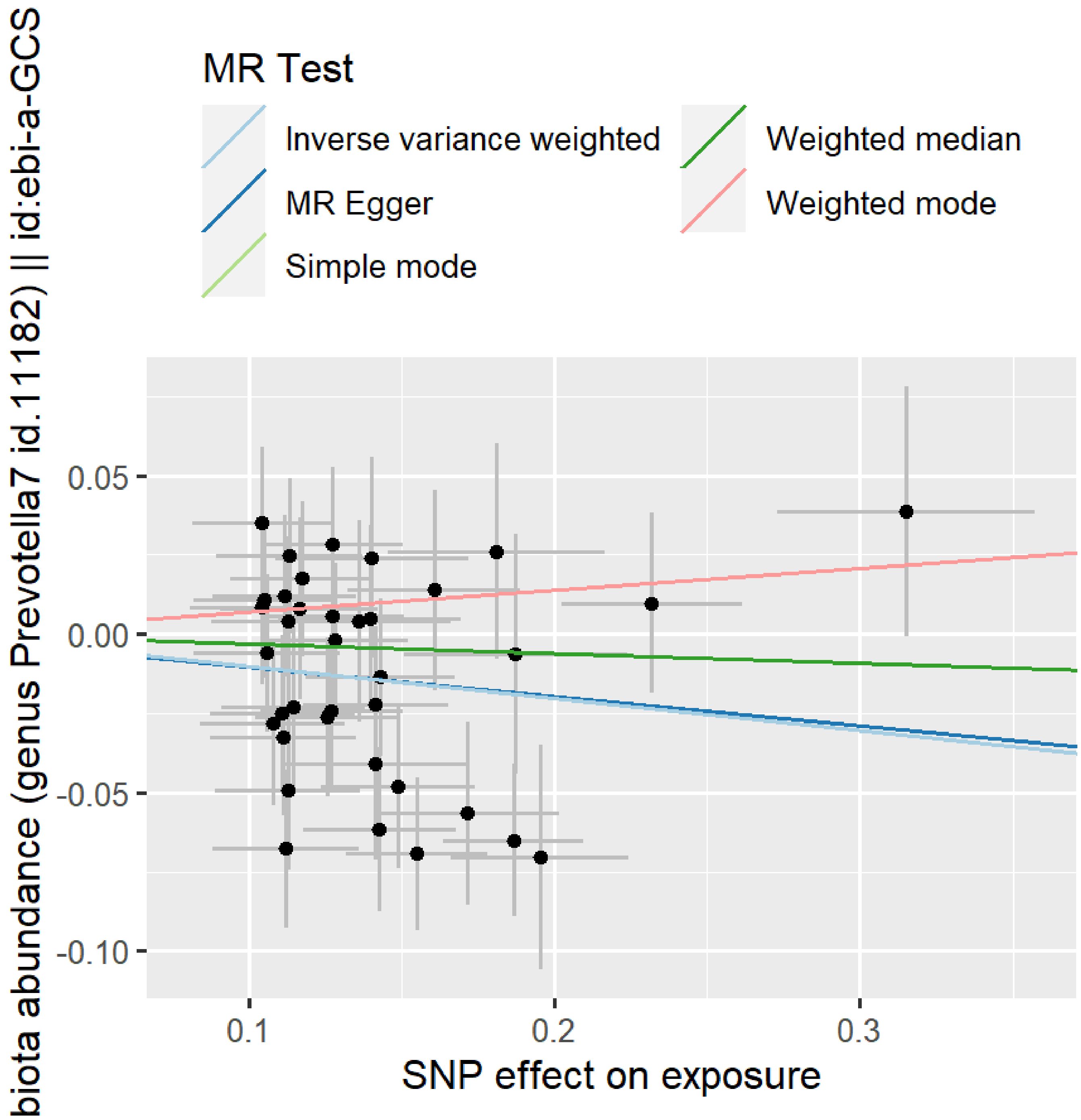

MR Test
Inverse variance weighted
MR Egger
Simple mode
Weighted median
Weighted mode
biota abundance (genus Prevotella7 id.11182) || id:ebi-a-GCS
0.05
0.00
-0.05
-0.10
0.1
0.2
0.3
SNP effect on exposure

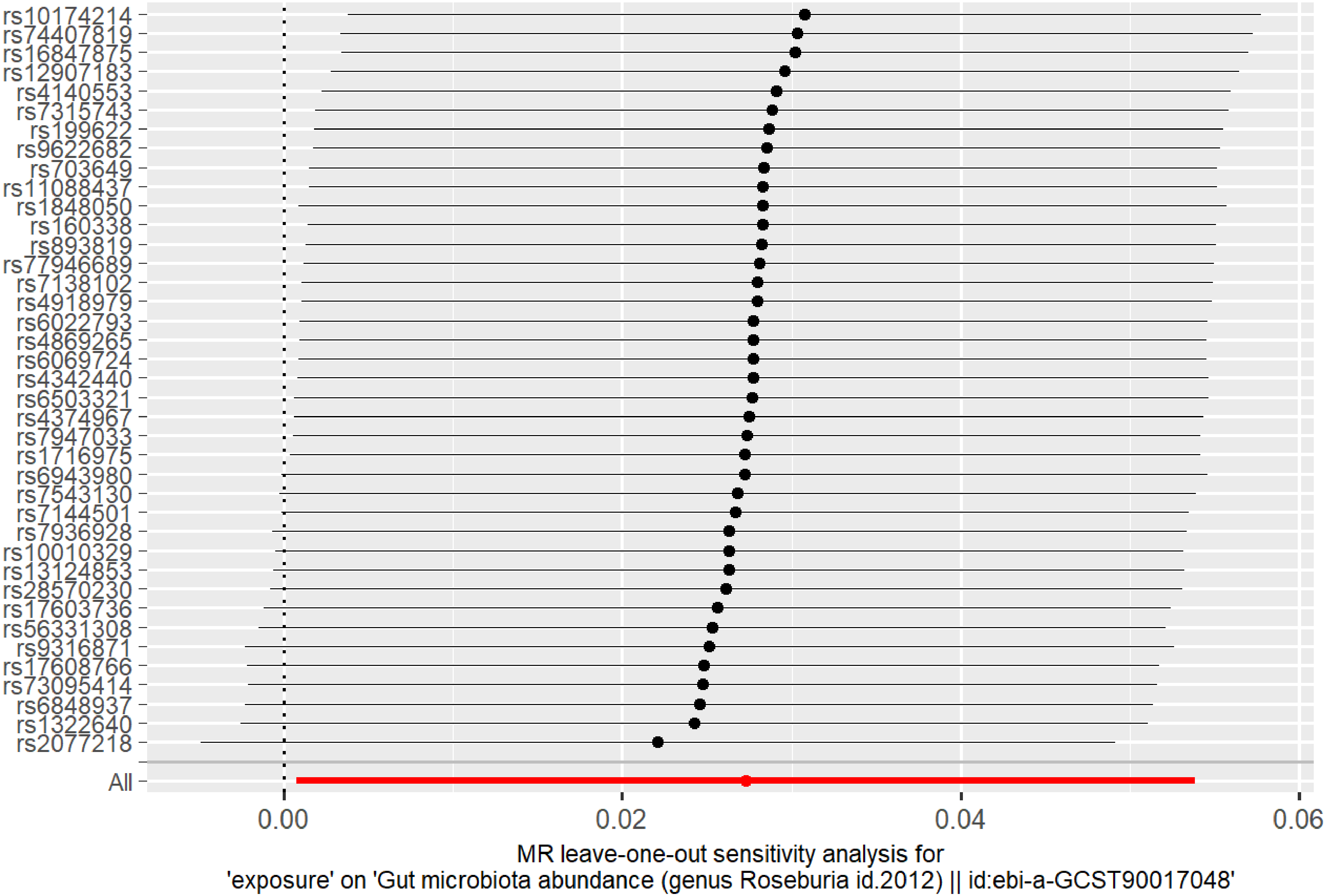
rs10174214
rs74407819
rs16847875
rs12907183
rs4140553
rs7315743
rs199622
rs9622682
rs703649
rs11088437
rs1848050
rs160338
rs893819
rs77946689
rs7138102
rs4918979
rs6022793
rs4869265
rs6069724
rs4342440
rs6503321
rs4374967
rs7947033
rs1716975
rs6943980
rs7543130
rs7144501
rs7936928
rs10010329
rs13124853
rs28570230
rs17603736
rs56331308
rs9316871
rs17608766
rs73095414
rs6848937
rs1322640
rs2077218
All
0.00
0.02
0.04
0.06
MR leave-one-out sensitivity analysis for
'exposure' on 'Gut microbiota abundance (genus Roseburia id.2012) || id:ebi-a-GCST90017048'

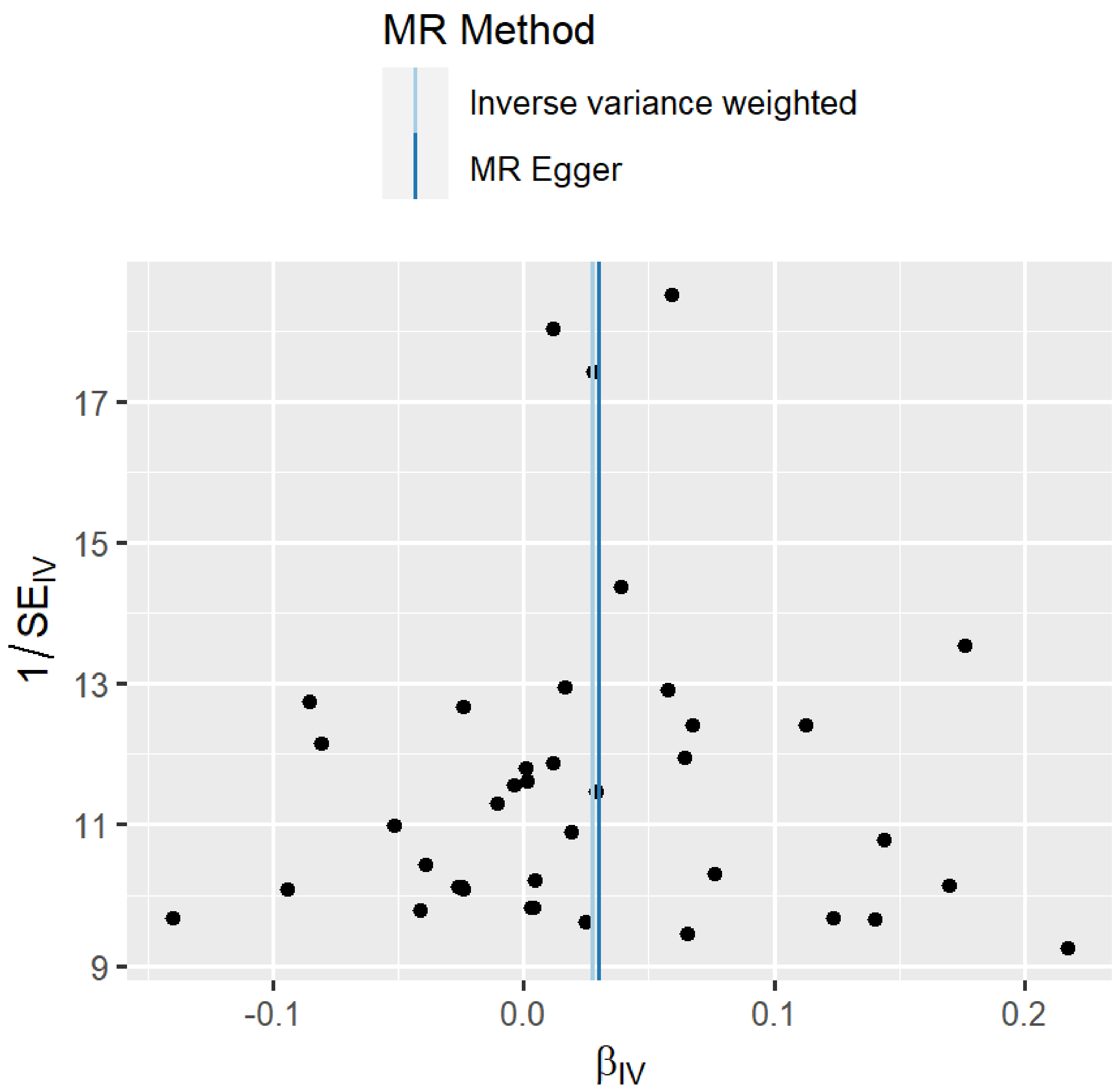
MR Method
Inverse variance weighted
MR Egger
17
15
13
11
9
1/SE_IV
-0.1
0.0
0.1
0.2
β_IV

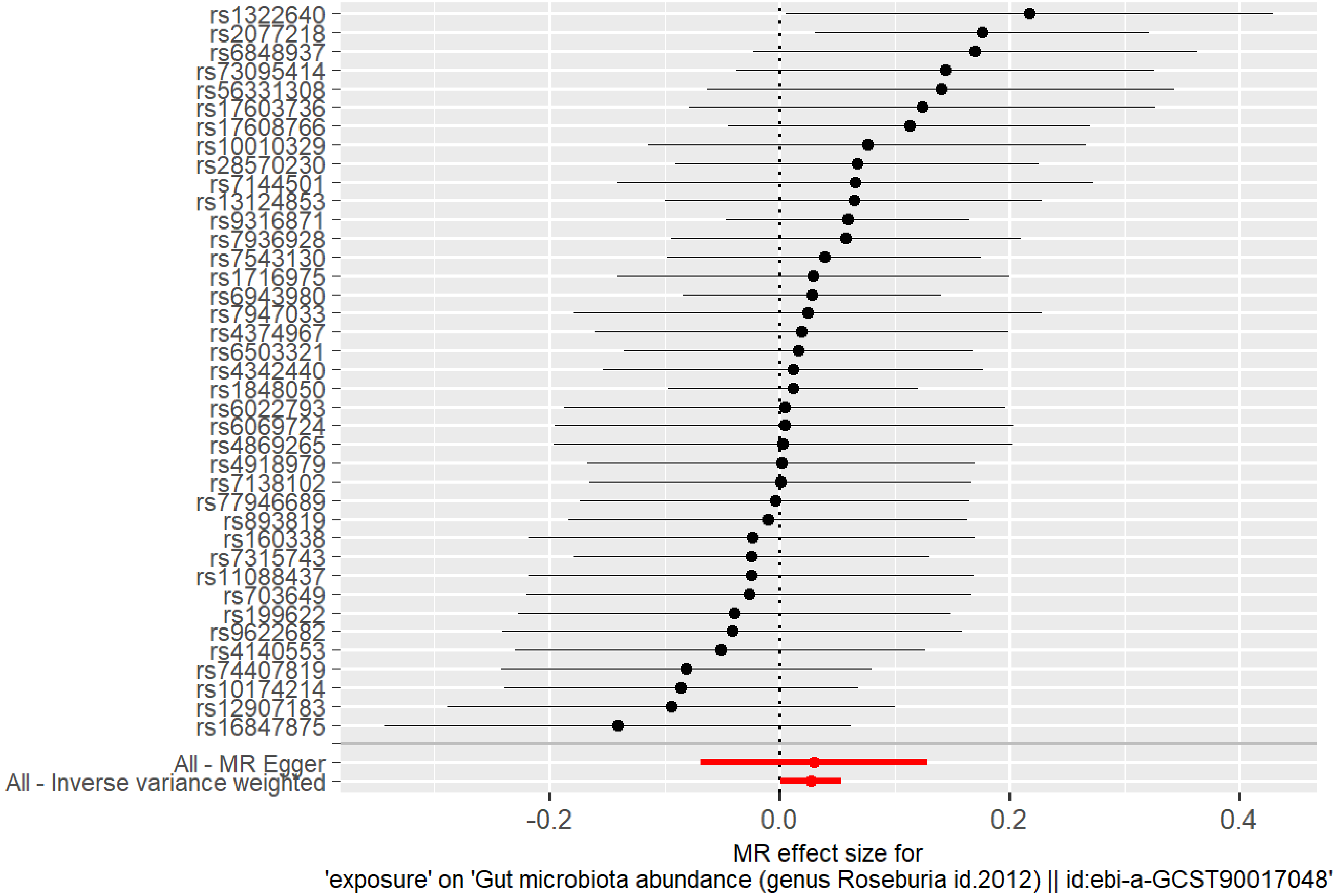
rs1322640
rs2077218
rs6848937
rs73095414
rs56331308
rs17603736
rs17608766
rs10010329
rs28570230
rs7144501
rs13124853
rs9316871
rs7936928
rs7543130
rs1716975
rs6943980
rs7947033
rs4374967
rs6503321
rs4342440
rs1848050
rs6022793
rs6069724
rs4869265
rs4918979
rs7138102
rs77946689
rs893819
rs160338
rs7315743
rs11088437
rs703649
rs199622
rs9622682
rs4140553
rs74407819
rs10174214
rs12907183
rs16847875
All - MR Egger
All - Inverse variance weighted
-0.2
0.0
0.2
0.4
MR effect size for
'exposure' on 'Gut microbiota abundance (genus Roseburia id.2012) || id:ebi-a-GCST90017048'

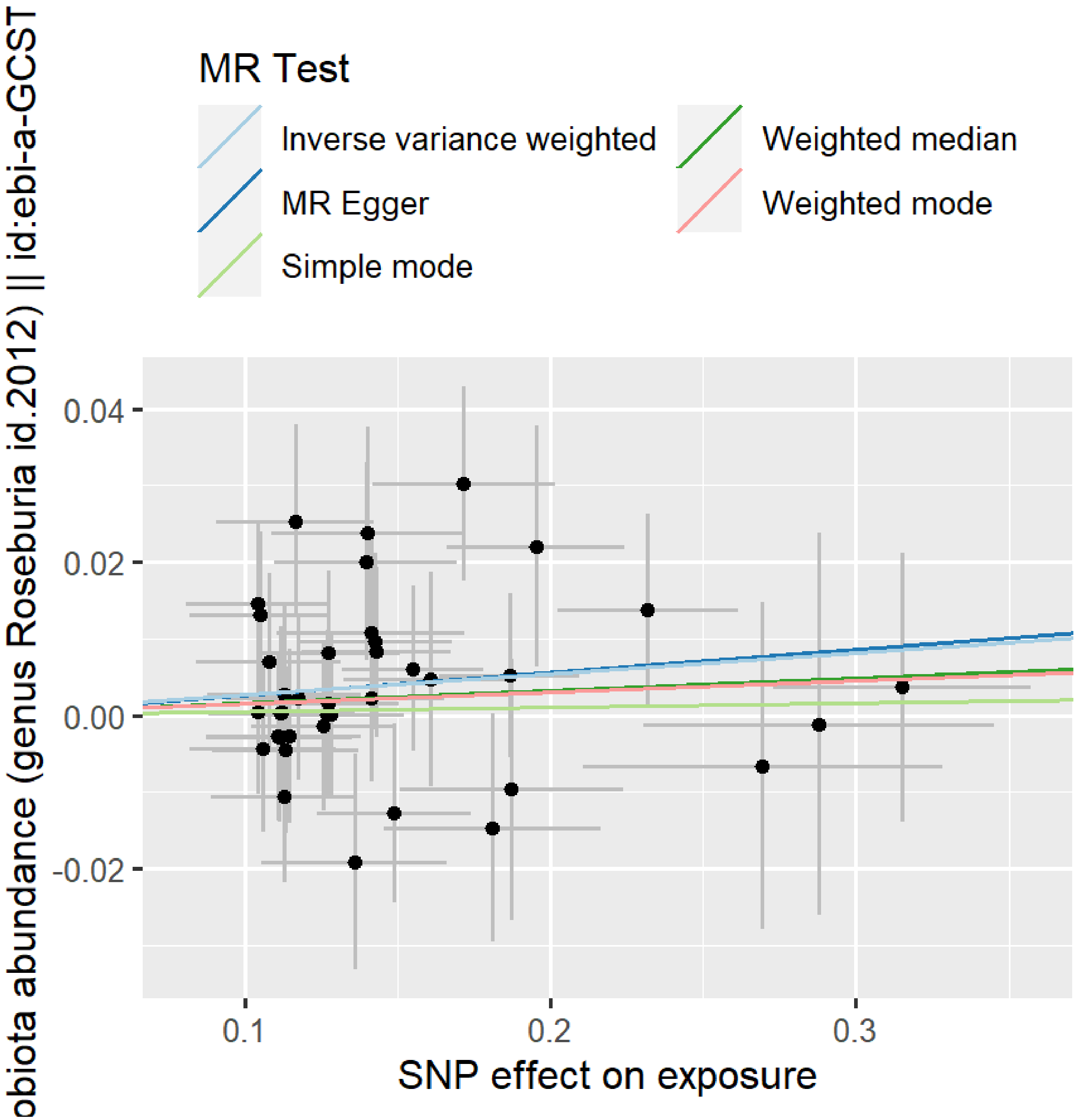
MR Test
Inverse variance weighted
MR Egger
Simple mode
Weighted median
Weighted mode
obiota abundance (genus Roseburia id.2012) || id:ebi-a-GCST
0.04
0.02
0.00
-0.02
0.1
0.2
0.3
SNP effect on exposure

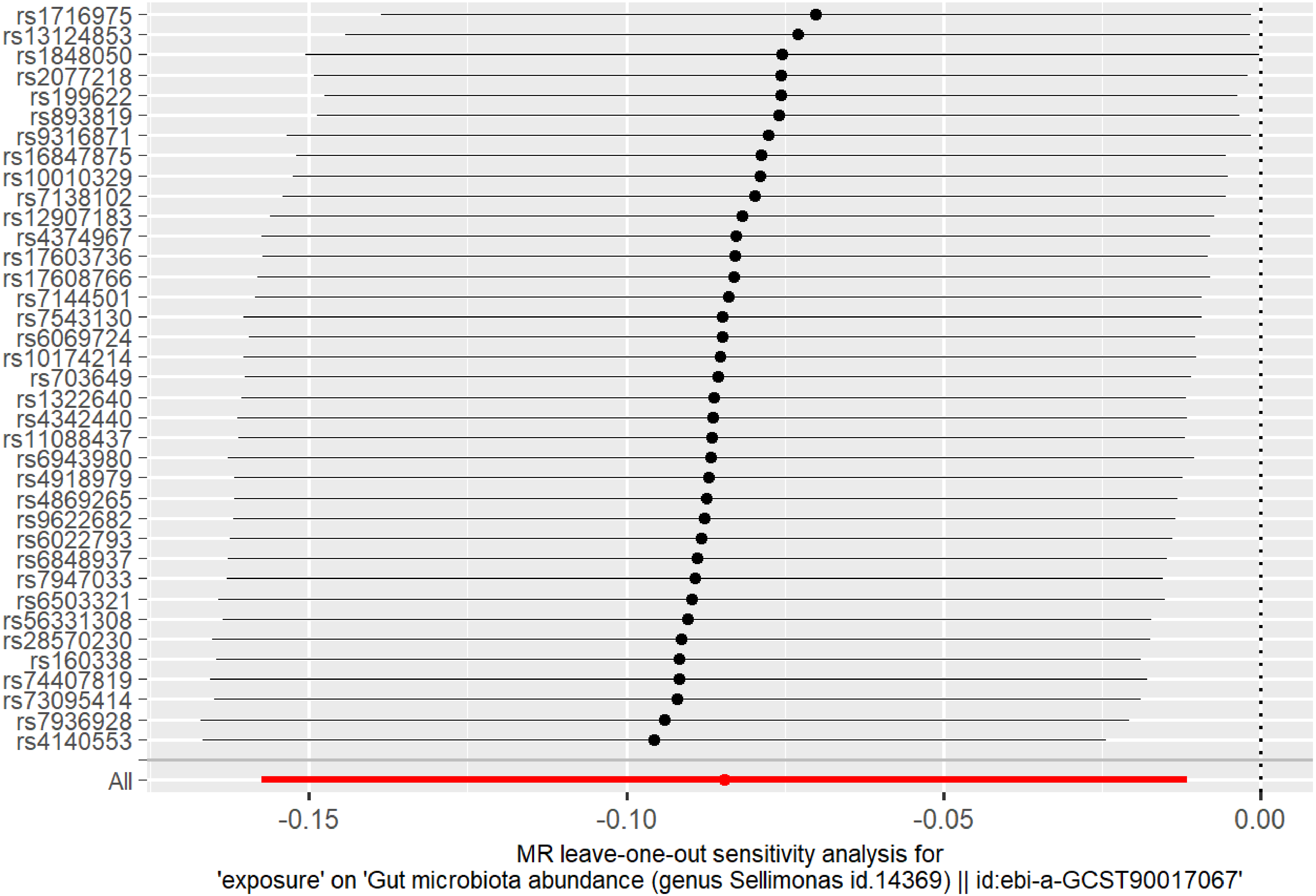
rs1716975
rs13124853
rs1848050
rs2077218
rs199622
rs893819
rs9316871
rs16847875
rs10010329
rs7138102
rs12907183
rs4374967
rs17603736
rs17608766
rs7144501
rs7543130
rs6069724
rs10174214
rs703649
rs1322640
rs4342440
rs11088437
rs6943980
rs4918979
rs4869265
rs9622682
rs6022793
rs6848937
rs7947033
rs6503321
rs56331308
rs28570230
rs160338
rs74407819
rs73095414
rs7936928
rs4140553
All
-0.15
-0.10
-0.05
0.00
MR leave-one-out sensitivity analysis for
'exposure' on 'Gut microbiota abundance (genus Sellimonas id.14369) || id:ebi-a-GCST90017067'

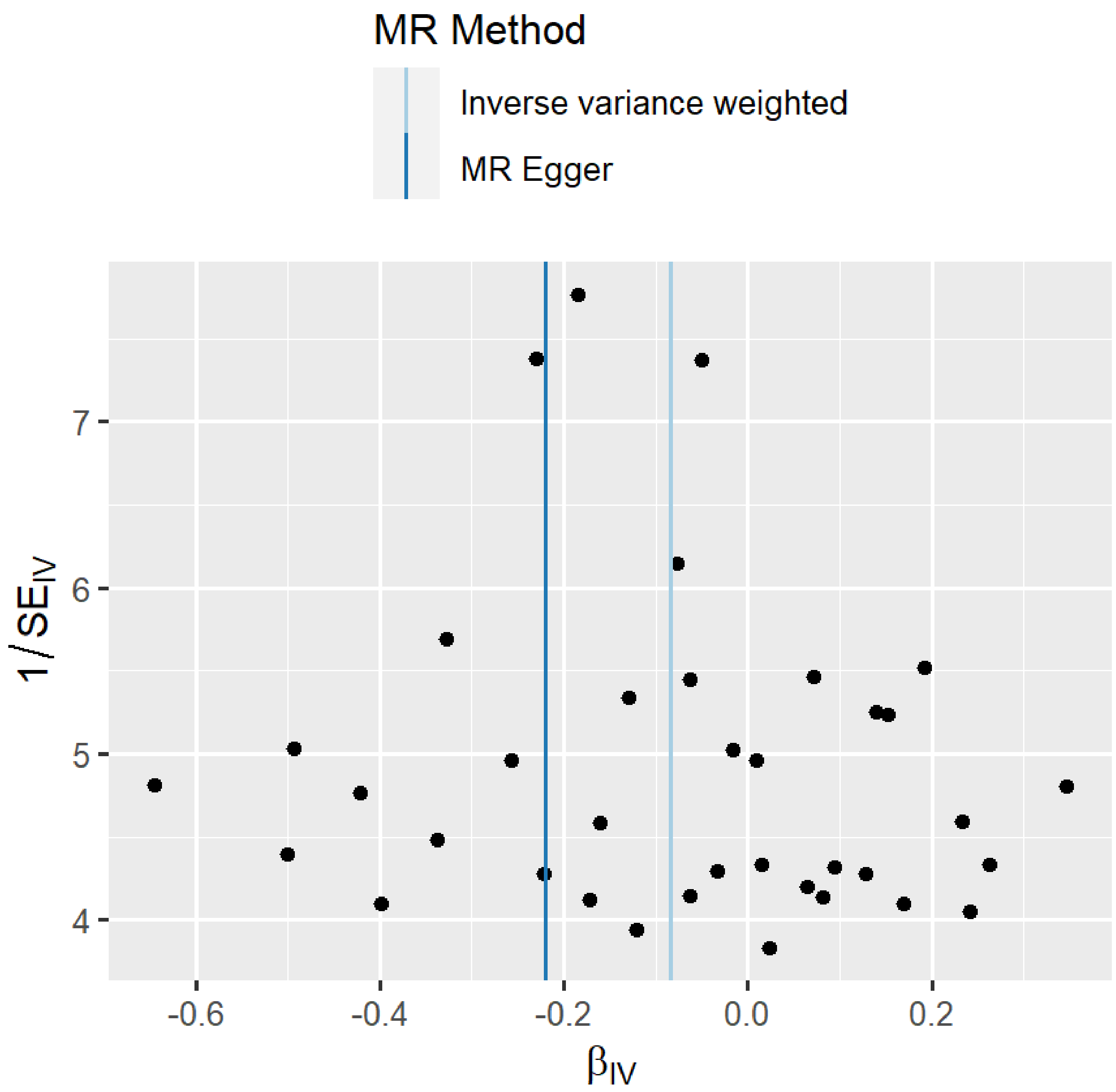
MR Method
Inverse variance weighted
MR Egger
7
6
5
4
1/SE_IV
-0.6
-0.4
-0.2
0.0
0.2
β_IV

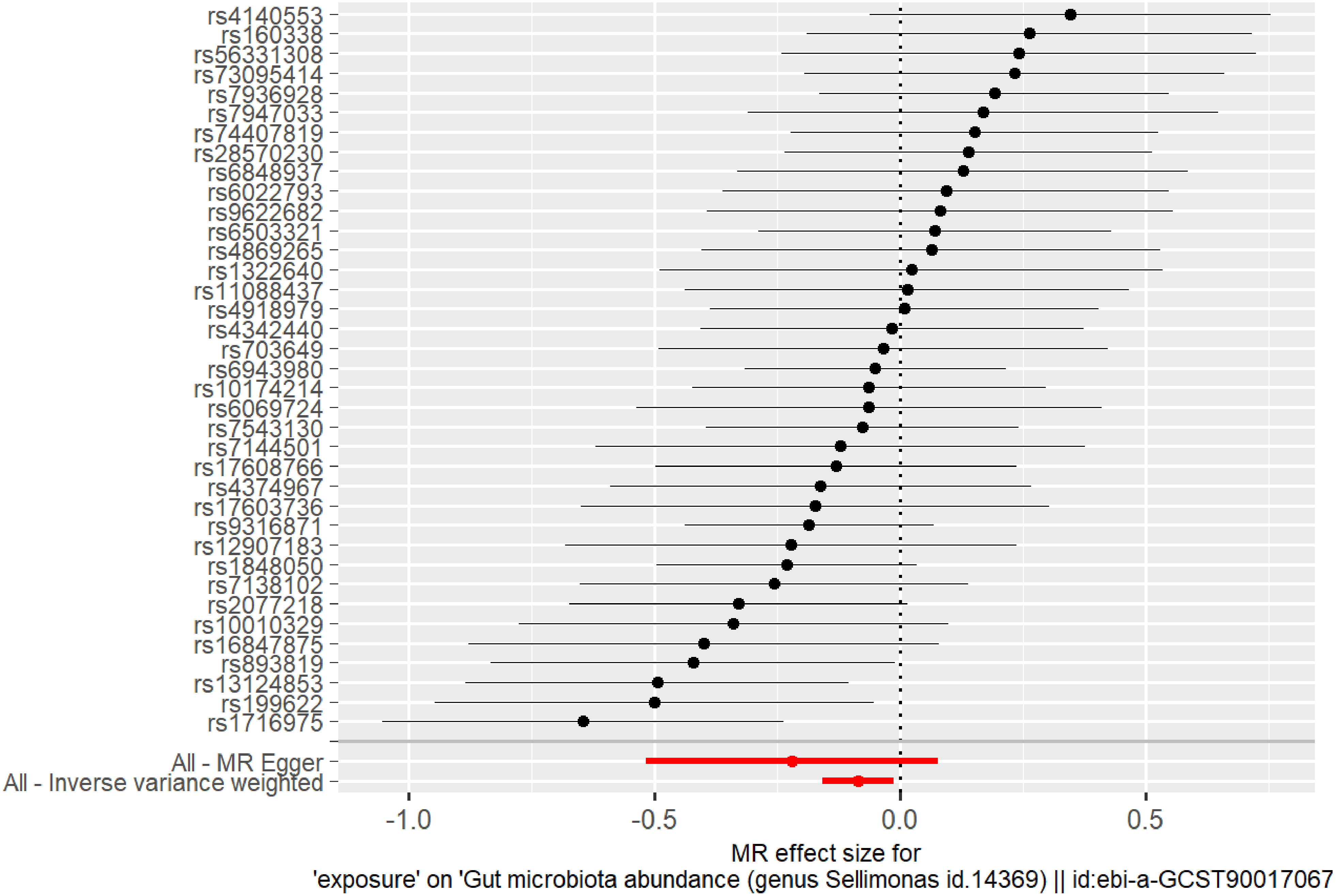
rs4140553
rs160338
rs56331308
rs73095414
rs7936928
rs7947033
rs74407819
rs28570230
rs6848937
rs6022793
rs9622682
rs6503321
rs4869265
rs1322640
rs11088437
rs4918979
rs4342440
rs703649
rs6943980
rs10174214
rs6069724
rs7543130
rs7144501
rs17608766
rs4374967
rs17603736
rs9316871
rs12907183
rs1848050
rs7138102
rs2077218
rs10010329
rs16847875
rs893819
rs13124853
rs199622
rs1716975
All - MR Egger
All - Inverse variance weighted
-1.0
-0.5
0.0
0.5
MR effect size for
'exposure' on 'Gut microbiota abundance (genus Sellimonas id.14369) || id:ebi-a-GCST90017067

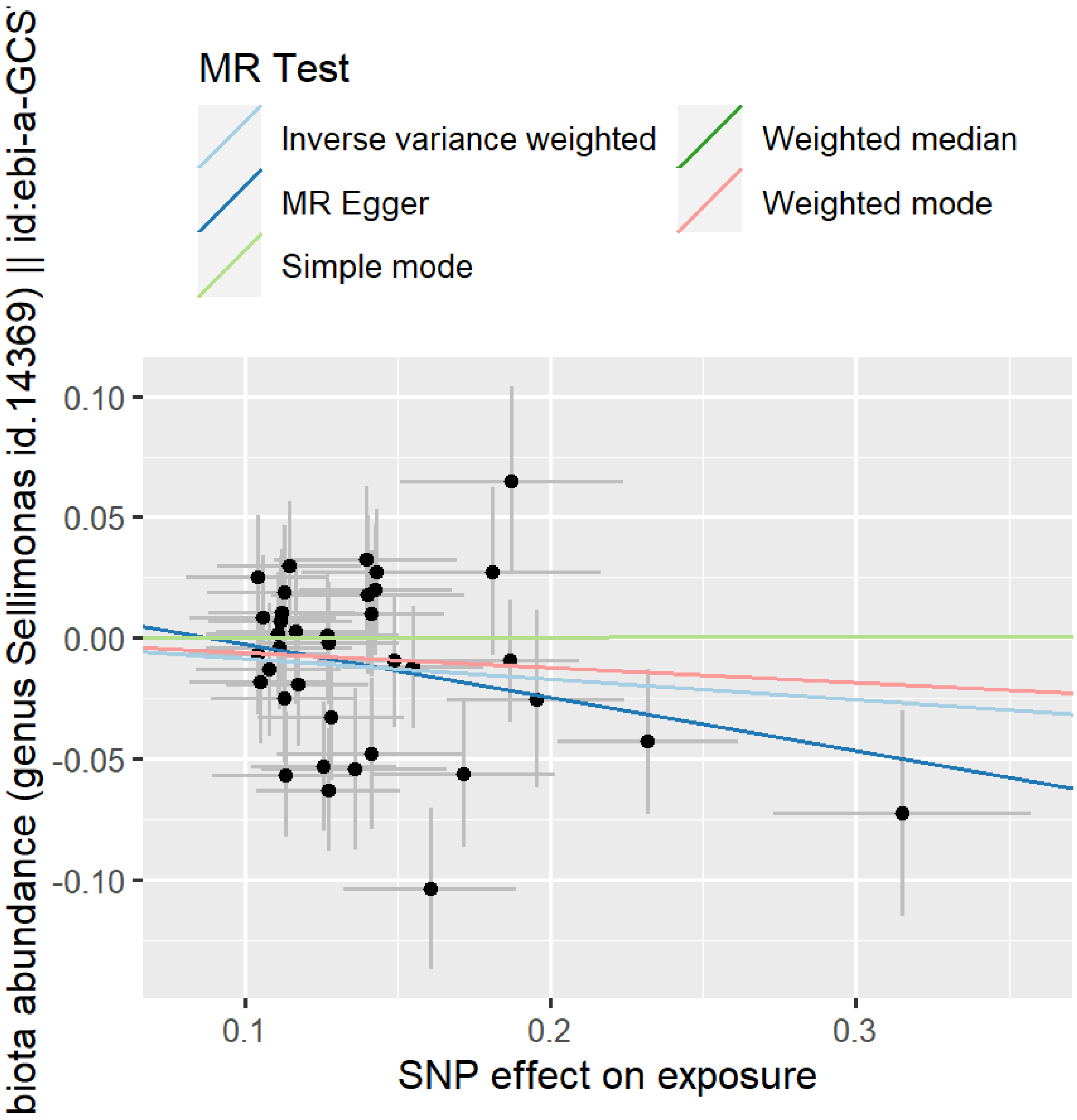
MR Test
Inverse variance weighted
MR Egger
Simple mode
Weighted median
Weighted mode
biota abundance (genus Sellimonas id.14369) || id:ebi-a-GCS
0.10
0.05
0.00
-0.05
-0.10
0.1
0.2
0.3
SNP effect on exposure

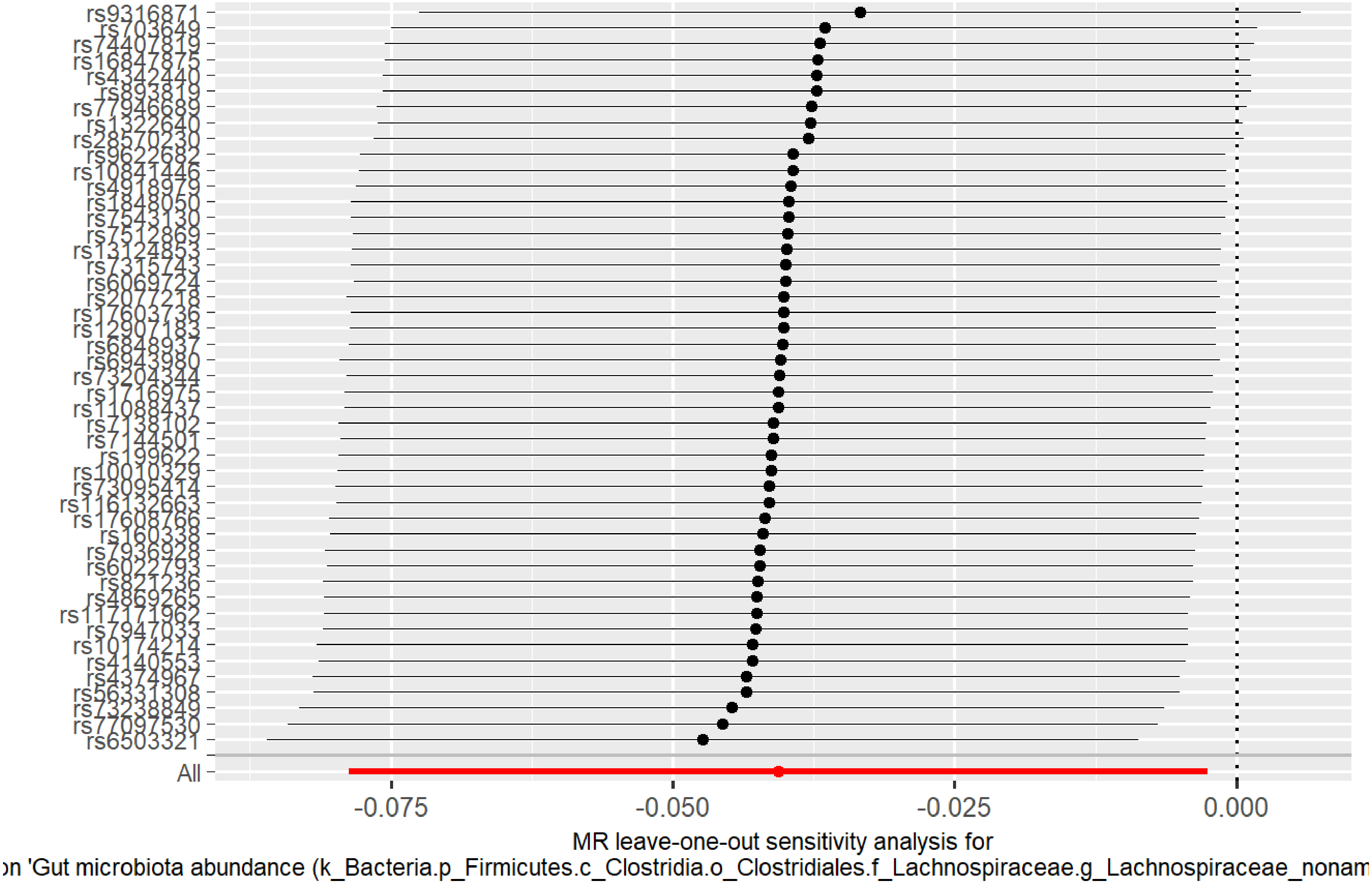
rs9316871
rs703649
rs74407819
rs16847875
rs4342440
rs893819
rs77946689
rs1322640
rs28570230
rs9622682
rs10841446
rs4918979
rs1848050
rs7543130
rs7512869
rs13124853
rs7315743
rs6069724
rs2077218
rs17603736
rs12907183
rs6848937
rs6943980
rs73204344
rs1716975
rs11088437
rs7138102
rs7144501
rs199622
rs10010329
rs73095414
rs116132663
rs17608766
rs160338
rs7936928
rs6022793
rs821236
rs4869265
rs117171962
rs79470333
rs10174214
rs4140553
rs4374967
rs56331308
rs73238849
rs77097530
rs6503321
All
-0.075
-0.050
-0.025
0.000
MR leave-one-out sensitivity analysis for
on 'Gut microbiota abundance (k_Bacteria.p_Firmicutes.c_Clostridia.o_Clostridiales.f_Lachnospiraceae.g_Lachnospiraceae_nonam

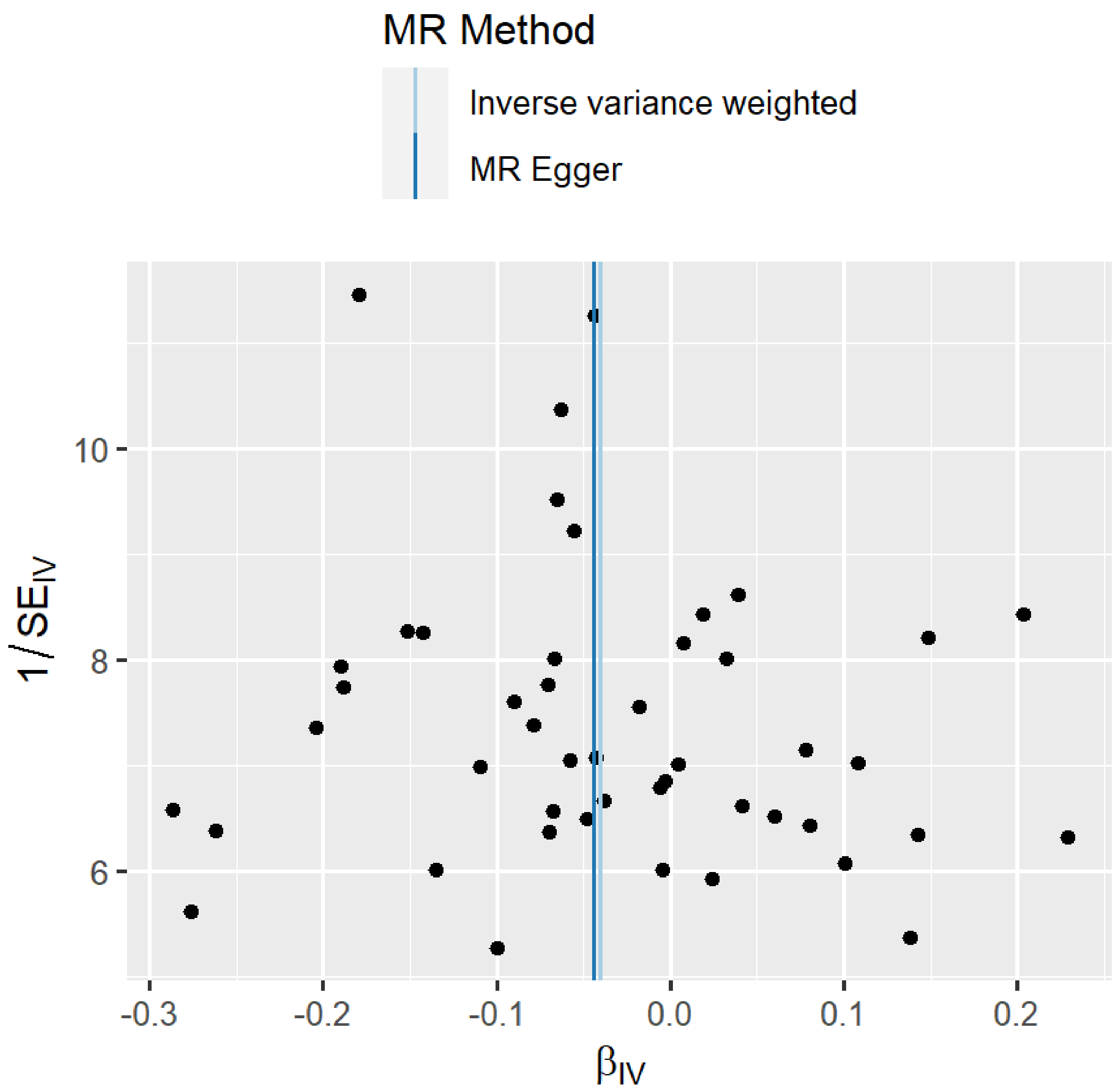

MR Method
Inverse variance weighted
MR Egger
10
8
6
$1/SE_{IV}$
-0.3
-0.2
-0.1
0.0
0.1
0.2
β_{IV}

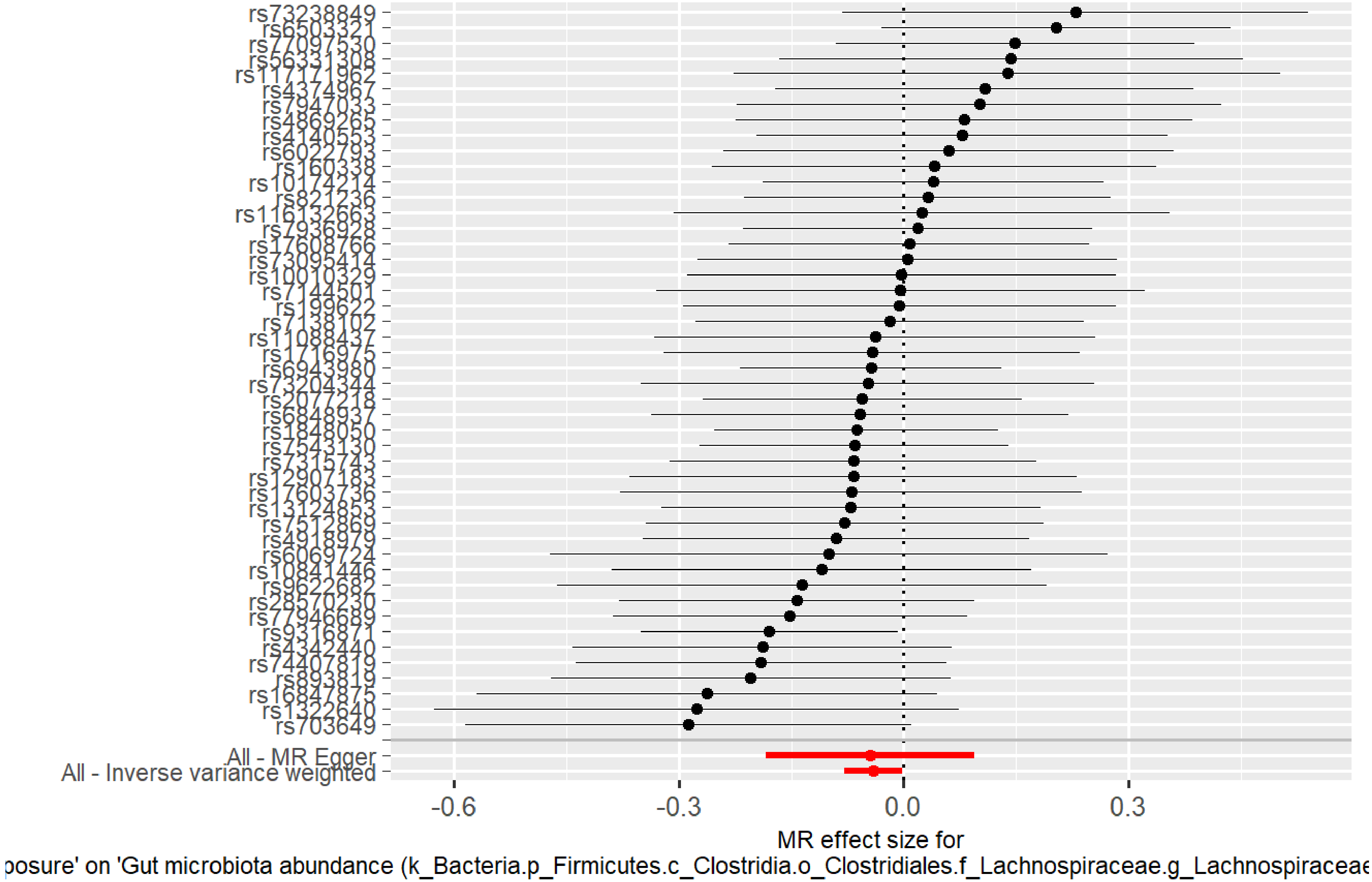
rs73238849
rs6503321
rs77097530
rs56331308
rs117171962
rs4374967
rs7947033
rs4869265
rs4140553
rs6022793
rs160338
rs10174214
rs821236
rs11613266
rs7936928
rs17608766
rs73095414
rs10010329
rs7144501
rs199622
rs7138102
rs11088437
rs1716975
rs6943980
rs73204344
rs2077218
rs6848937
rs1848050
rs7543130
rs7315743
rs12907183
rs17603736
rs13124853
rs7512869
rs4918979
rs6069724
rs10841446
rs9622682
rs28570230
rs77946689
rs9316871
rs4342440
rs74407819
rs893819
rs16847875
rs1322640
rs703649
All - MR Egger
All - Inverse variance weighted
-0.6
-0.3
0.0
0.3
MR effect size for
posure' on 'Gut microbiota abundance (k_Bacteria.p_Firmicutes.c_Clostridia.o_Clostridiales.f_Lachnospiraceae.g_Lachnospiraceae

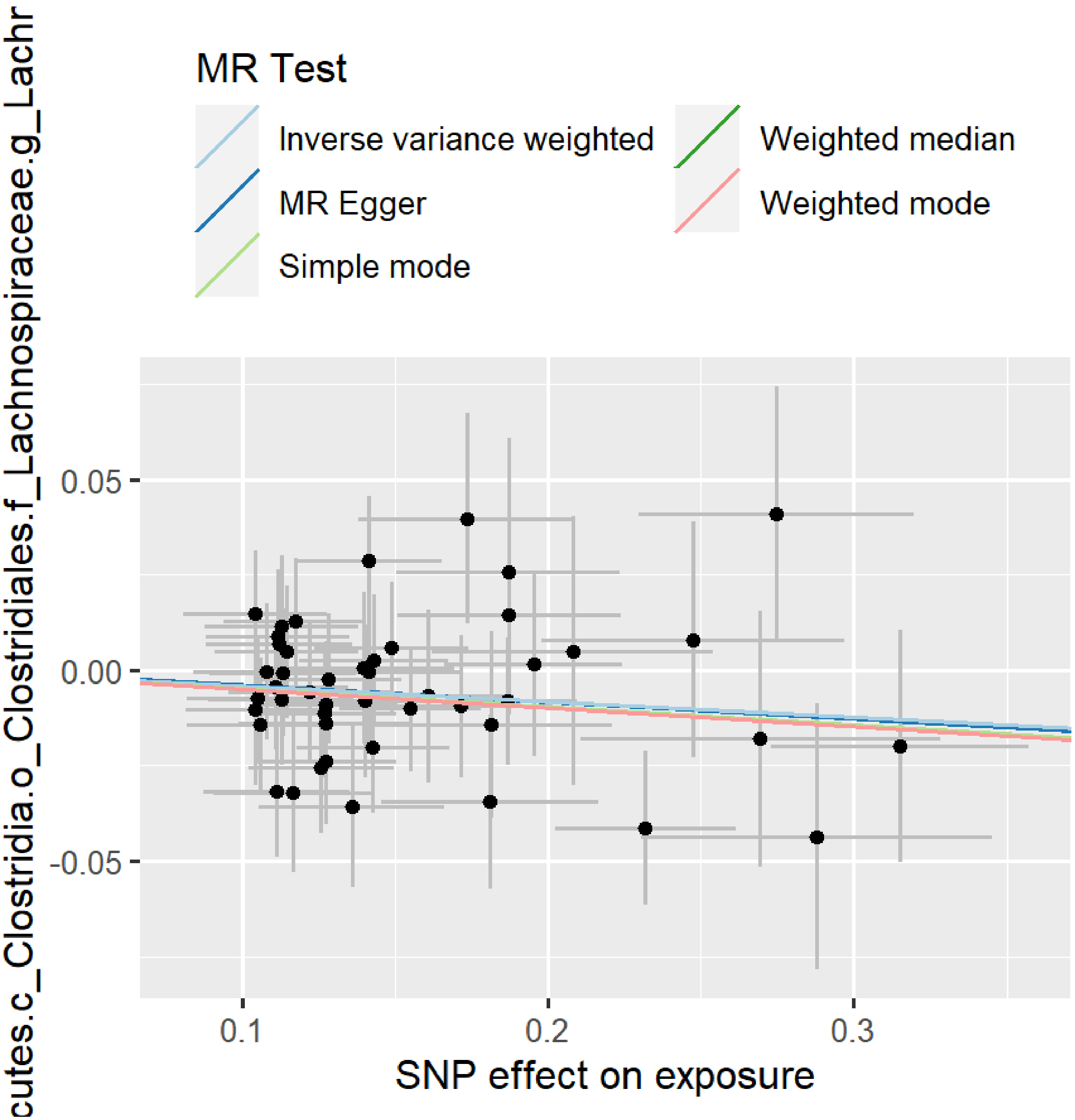

MR Test
Inverse variance weighted
MR Egger
Simple mode
Weighted median
Weighted mode
cutes.c_Clostridia.o_Clostridiales.f_Lachnospiraceae.g_Lachr
0.05
0.00
-0.05
0.1
0.2
0.3
SNP effect on exposure

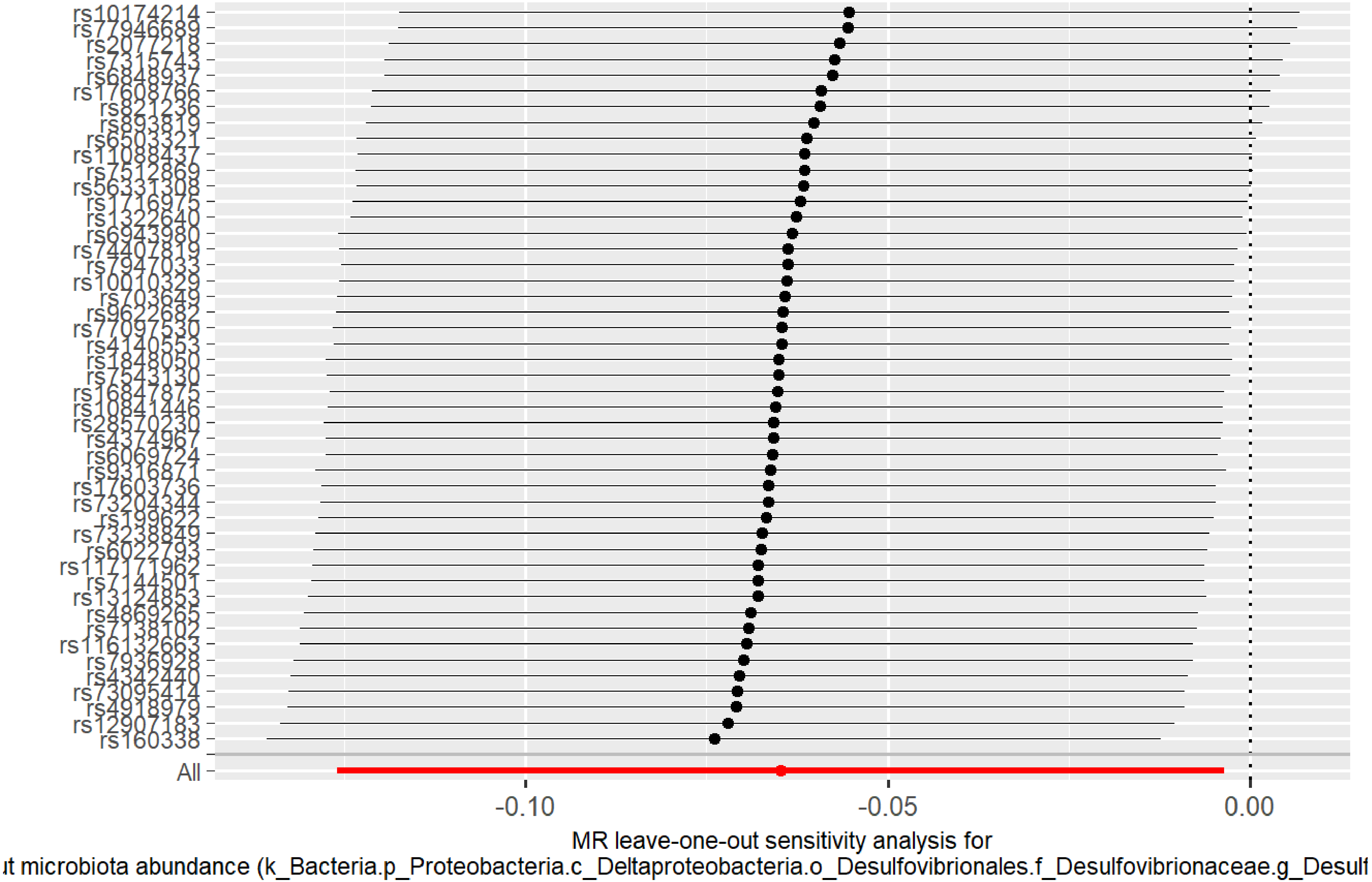
rs10174214
rs77946689
rs2077218
rs7315743
rs6848937
rs17608766
rs821236
rs893819
rs6503321
rs11088437
rs7512869
rs56331308
rs1716975
rs1322640
rs6943980
rs74407819
rs7947033
rs10010329
rs703649
rs9622682
rs77097530
rs4140553
rs1848050
rs7543130
rs16847875
rs10841446
rs28570230
rs4374967
rs6069724
rs9316871
rs17603736
rs73204344
rs199622
rs73238849
rs6022793
rs117171962
rs7144501
rs13124853
rs4869265
rs7138102
rs116132663
rs7936928
rs4342440
rs73095414
rs4918979
rs12907183
rs160338
All
-0.10
-0.05
0.00
MR leave-one-out sensitivity analysis for
ıt microbiota abundance (k_Bacteria.p_Proteobacteria.c_Deltaproteobacteria.o_Desulfovibrionales.f_Desulfovibrionaceae.g_Desulf

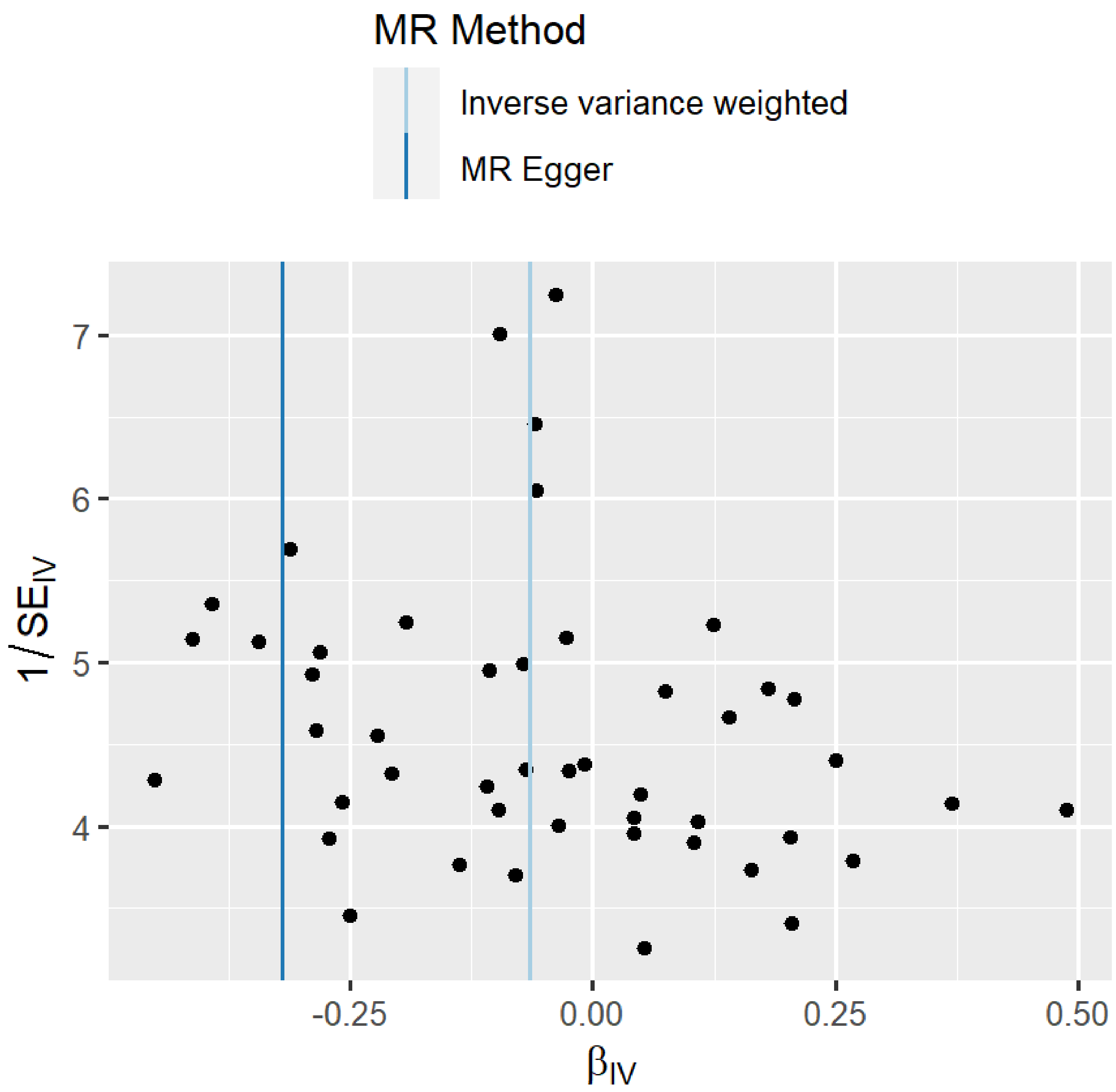

MR Method
Inverse variance weighted
MR Egger
7
6
5
4
1/SE_IV
-0.25
0.00
0.25
0.50
β_IV

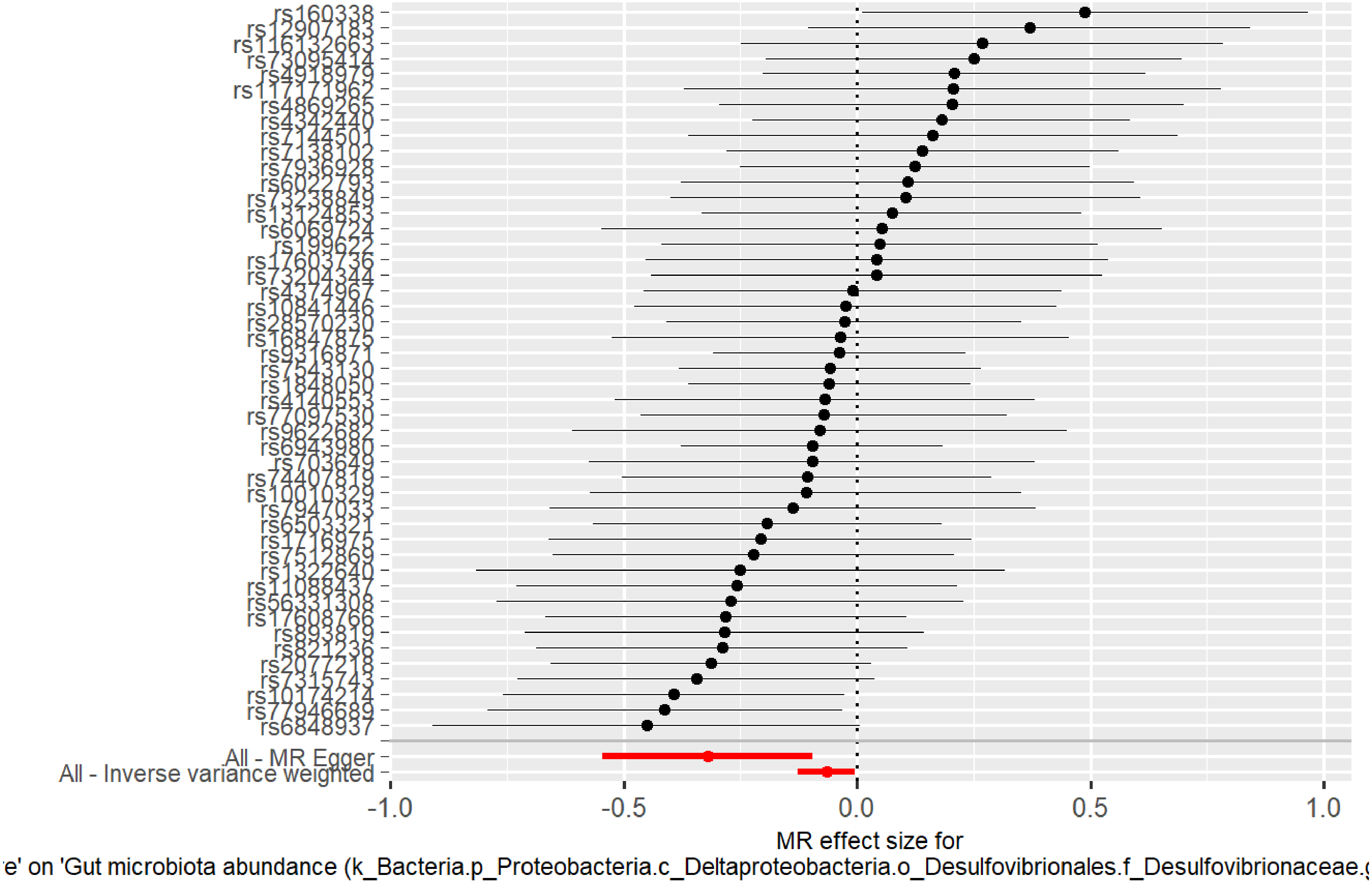
rs160338
rs12907183
rs116132663
rs73095414
rs4918979
rs117171962
rs4869265
rs4342440
rs7144501
rs7138102
rs7936928
rs6022793
rs73238849
rs1312485
rs6069724
rs199622
rs17603736
rs73204344
rs4374967
rs10841446
rs28570230
rs16847875
rs9316871
rs7543130
rs1848050
rs4140553
rs77097530
rs9622682
rs6943980
rs703649
rs74407819
rs10010329
rs7947033
rs6503321
rs1716975
rs7512869
rs1322640
rs11088437
rs56331308
rs17608766
rs893819
rs821236
rs2077218
rs7315743
rs10174214
rs77946689
rs6848937
All - MR Egger
All - Inverse variance weighted
-1.0
-0.5
0.0
0.5
1.0
MR effect size for
e' on 'Gut microbiota abundance (k_Bacteria.p_Proteobacteria.c_Deltaproteobacteria.o_Desulfovibrionales.f_Desulfovibrionaceae.

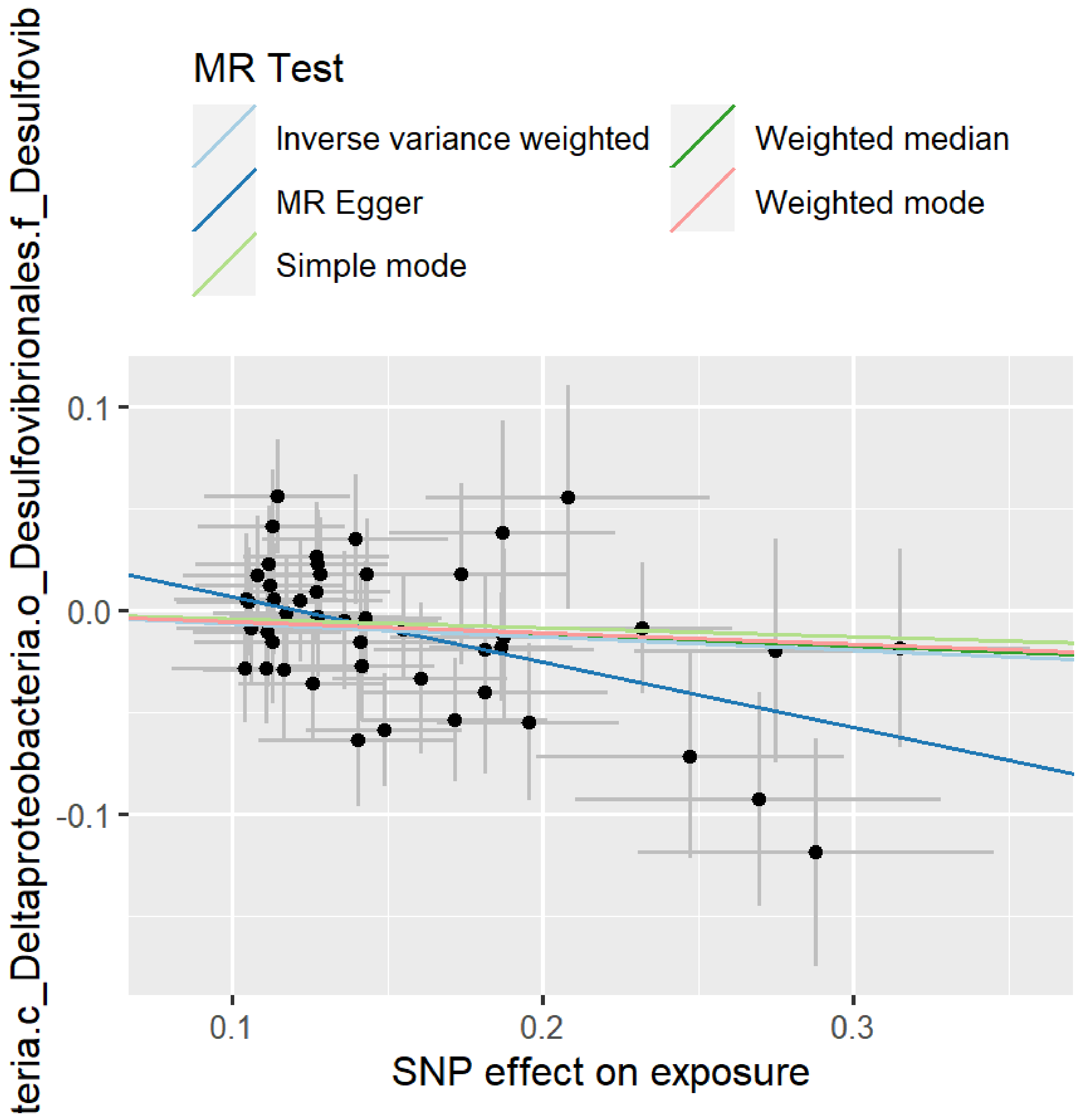
MR Test
Inverse variance weighted
MR Egger
Simple mode
Weighted median
Weighted mode
0.1
0.0
-0.1
0.1
0.2
0.3
SNP effect on exposure
teria.c_Deltaproteobacteria.o_Desulfovibrionales.f_Desulfovib

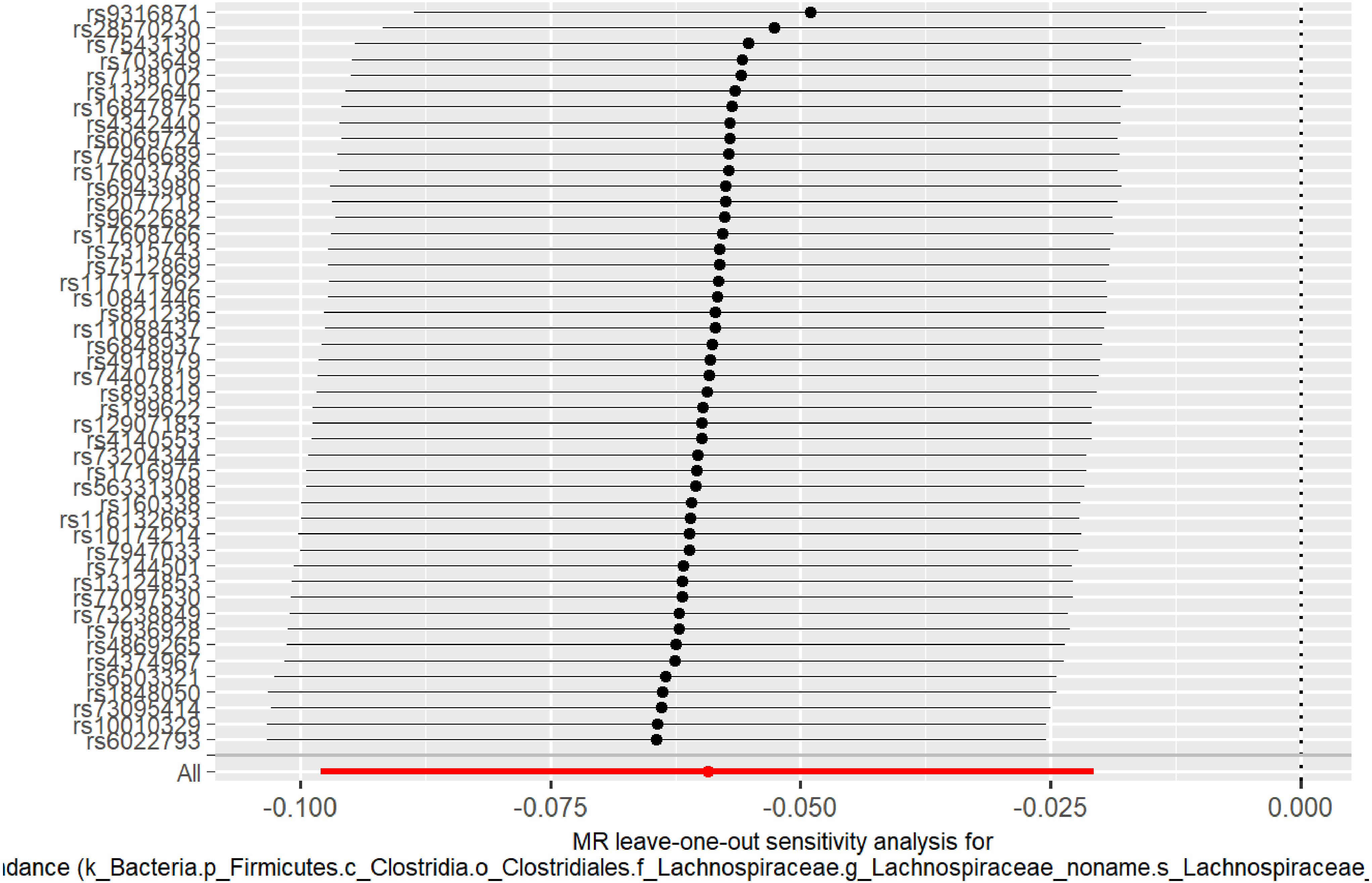
rs9316871
rs28570230
rs7543130
rs703649
rs7138102
rs1322640
rs16847875
rs4342440
rs6069724
rs77946689
rs17603736
rs6943980
rs2077218
rs9622682
rs17608766
rs7315743
rs7512869
rs117171962
rs10841446
rs821236
rs11088437
rs6848937
rs4918979
rs74407819
rs893819
rs199622
rs12907183
rs4140553
rs73204344
rs1716975
rs56331308
rs160338
rs116132663
rs10174214
rs7947033
rs7144501
rs13124853
rs77097530
rs73238849
rs7936928
rs4869265
rs4374967
rs6503321
rs1848050
rs73095414
rs10010329
rs6022793
All
-0.100
-0.075
-0.050
-0.025
0.000
MR leave-one-out sensitivity analysis for
ıdance (k_Bacteria.p_Firmicutes.c_Clostridia.o_Clostridiales.f_Lachnospiraceae.g_Lachnospiraceae_noname.s_Lachnospiraceae_

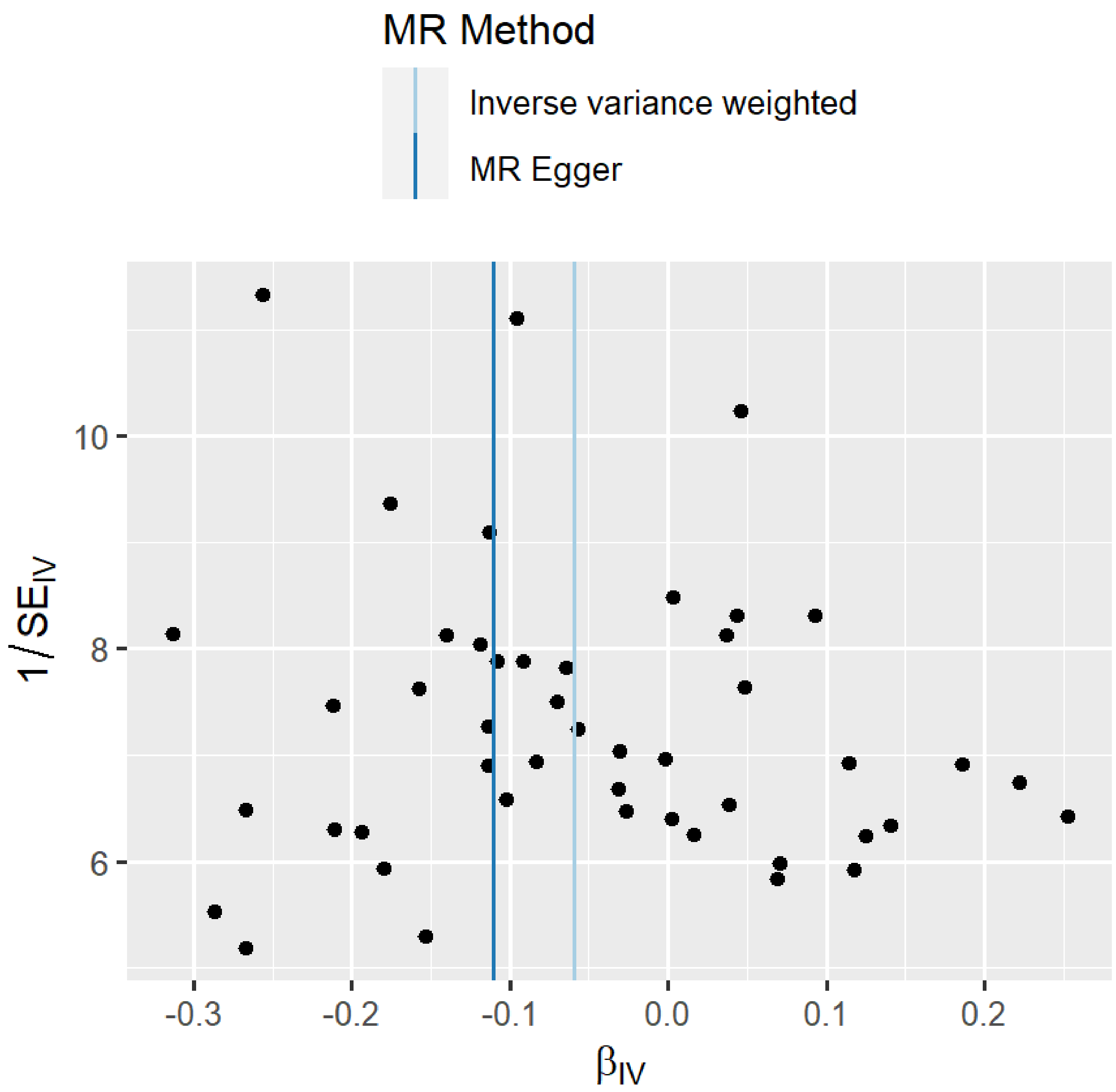
MR Method
Inverse variance weighted
MR Egger
10
8
6
-0.3
-0.2
-0.1
0.0
0.1
0.2
$1/SE_{IV}$
β_{IV}

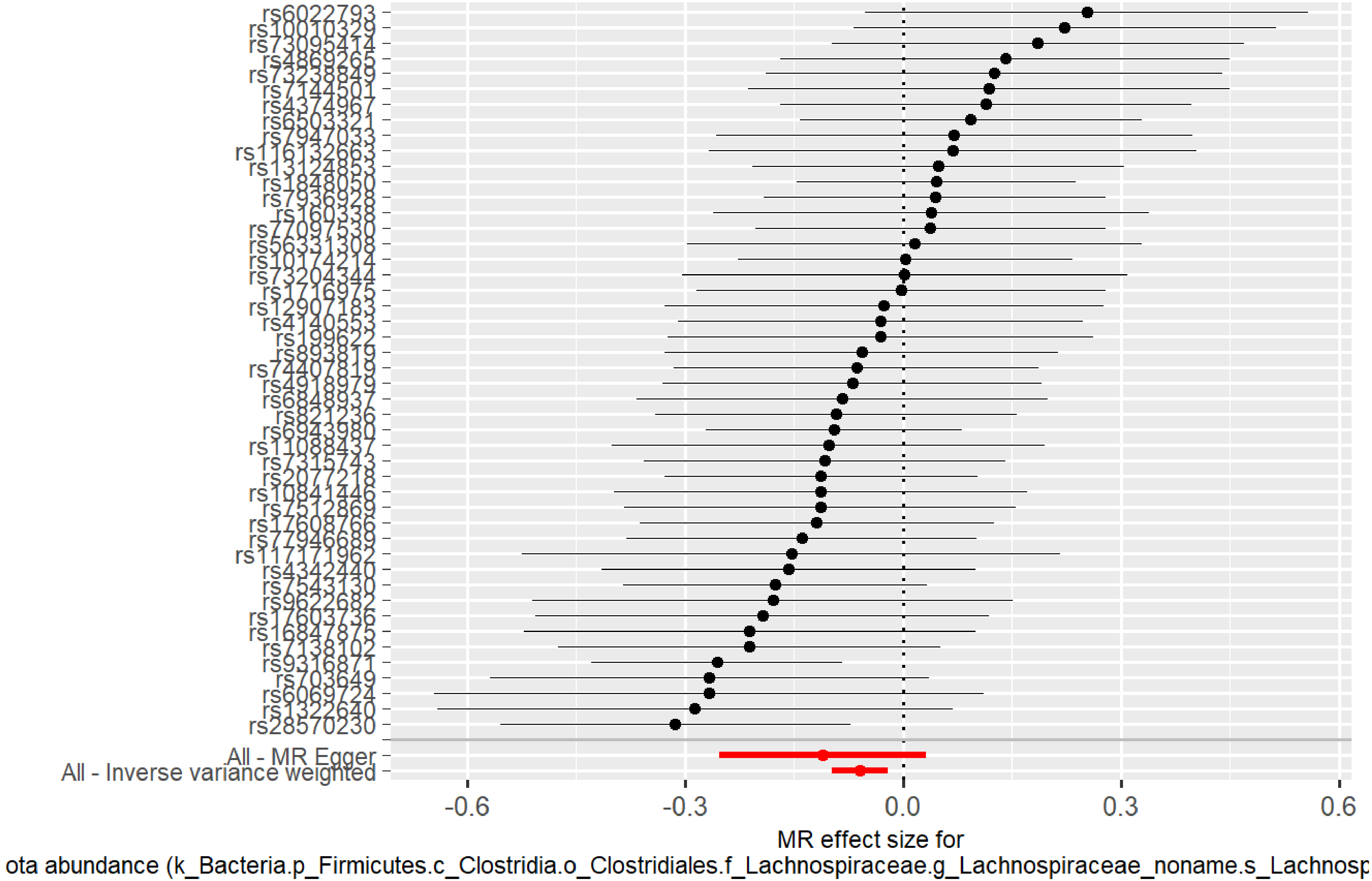
rs6022793
rs10010329
rs73095414
rs4869265
rs73238849
rs7144501
rs4374967
rs6503321
rs7947033
rs116132663
rs13124853
rs1848050
rs7936928
rs160338
rs770975530
rs56331308
rs10174214
rs73204344
rs1716975
rs12907183
rs4140553
rs199622
rs893819
rs74407819
rs4918979
rs6848937
rs821236
rs6943980
rs11088437
rs7315743
rs2077218
rs10841446
rs7512869
rs17608766
rs77946689
rs117171962
rs4342440
rs7543130
rs9622682
rs17603736
rs16847875
rs7138102
rs9316871
rs703649
rs6069724
rs1322640
rs28570230
All - MR Egger
All - Inverse variance weighted
-0.6
-0.3
0.0
0.3
0.6
MR effect size for
ota abundance (k_Bacteria.p_Firmicutes.c_Clostridia.o_Clostridiales.f_Lachnospiraceae.g_Lachnospiraceae_noname.s_Lachnosp

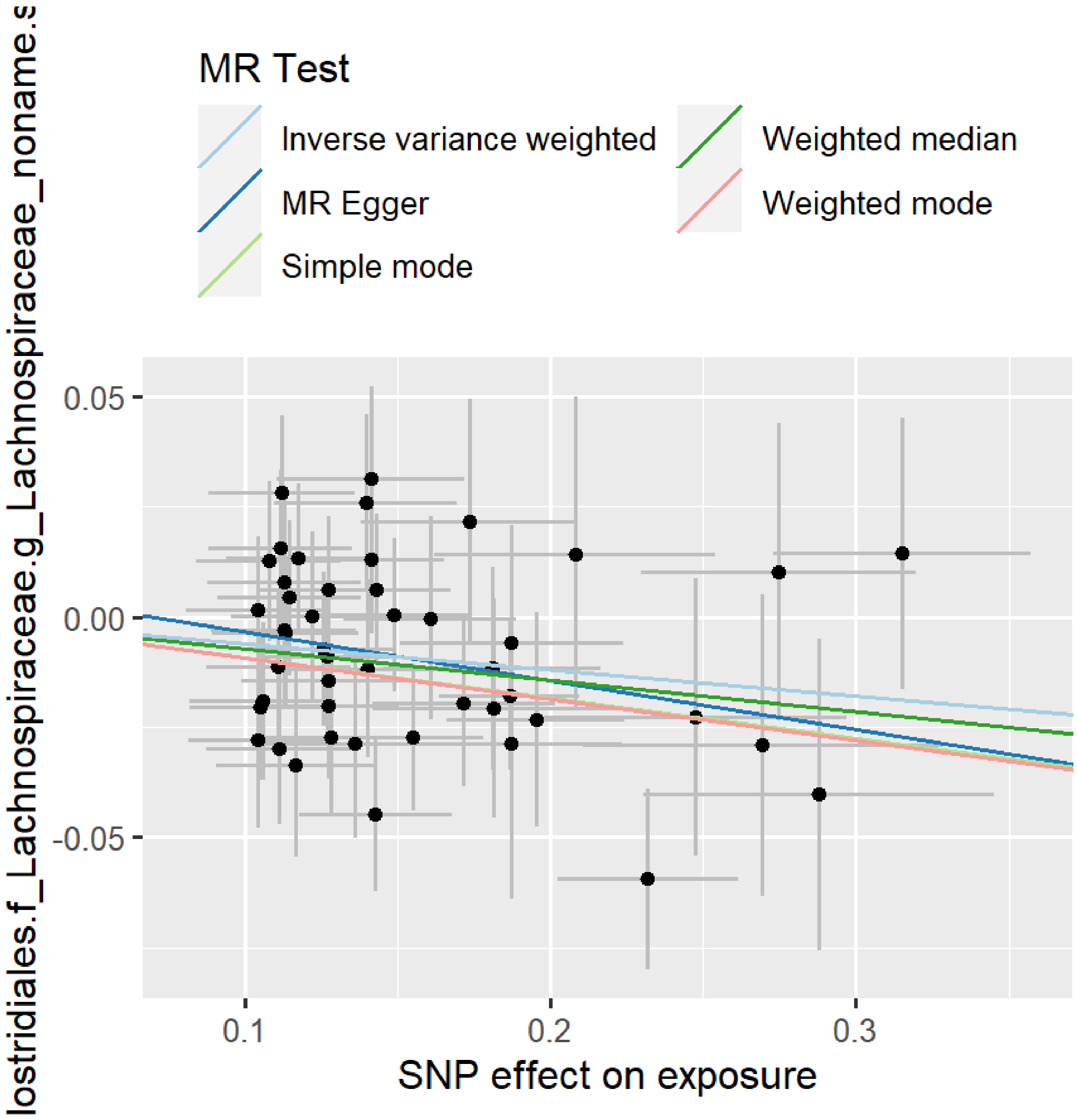

MR Test
Inverse variance weighted
MR Egger
Simple mode
Weighted median
Weighted mode
0.05
0.00
-0.05
0.1
0.2
0.3
SNP effect on exposure
lostridiales.f__Lachnospiraceae.g__Lachnospiraceae_noname.s

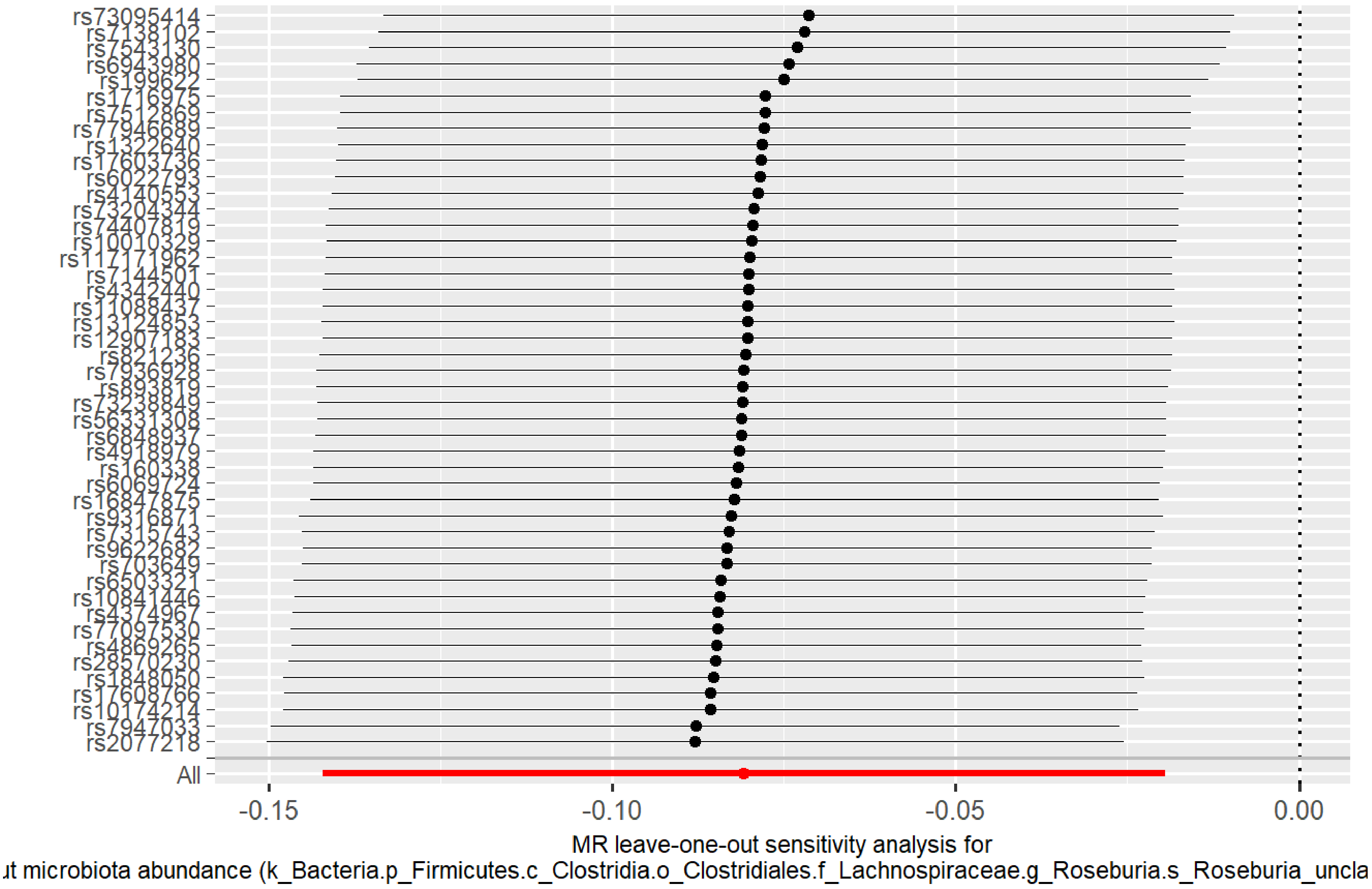
rs73095414
rs7138102
rs7543130
rs6943980
rs199622
rs1716975
rs7512869
rs77946689
rs1322640
rs17603736
rs6022793
rs4140553
rs73204344
rs74407819
rs10010329
rs117171962
rs7144501
rs4342440
rs11088437
rs13124853
rs12907183
rs821236
rs7936928
rs893819
rs73238849
rs56331308
rs6848937
rs4918979
rs160338
rs6069724
rs16847875
rs9316871
rs7315743
rs9622682
rs703649
rs6503321
rs10841446
rs4374967
rs77097530
rs4869265
rs28570230
rs1848050
rs17608766
rs10174214
rs7947033
rs2077218
All
-0.15
-0.10
-0.05
0.00
MR leave-one-out sensitivity analysis for
ıt microbiota abundance (k_Bacteria.p_Firmicutes.c_Clostridia.o_Clostridiales.f_Lachnospiraceae.g_Roseburia.s_Roseburia_uncla

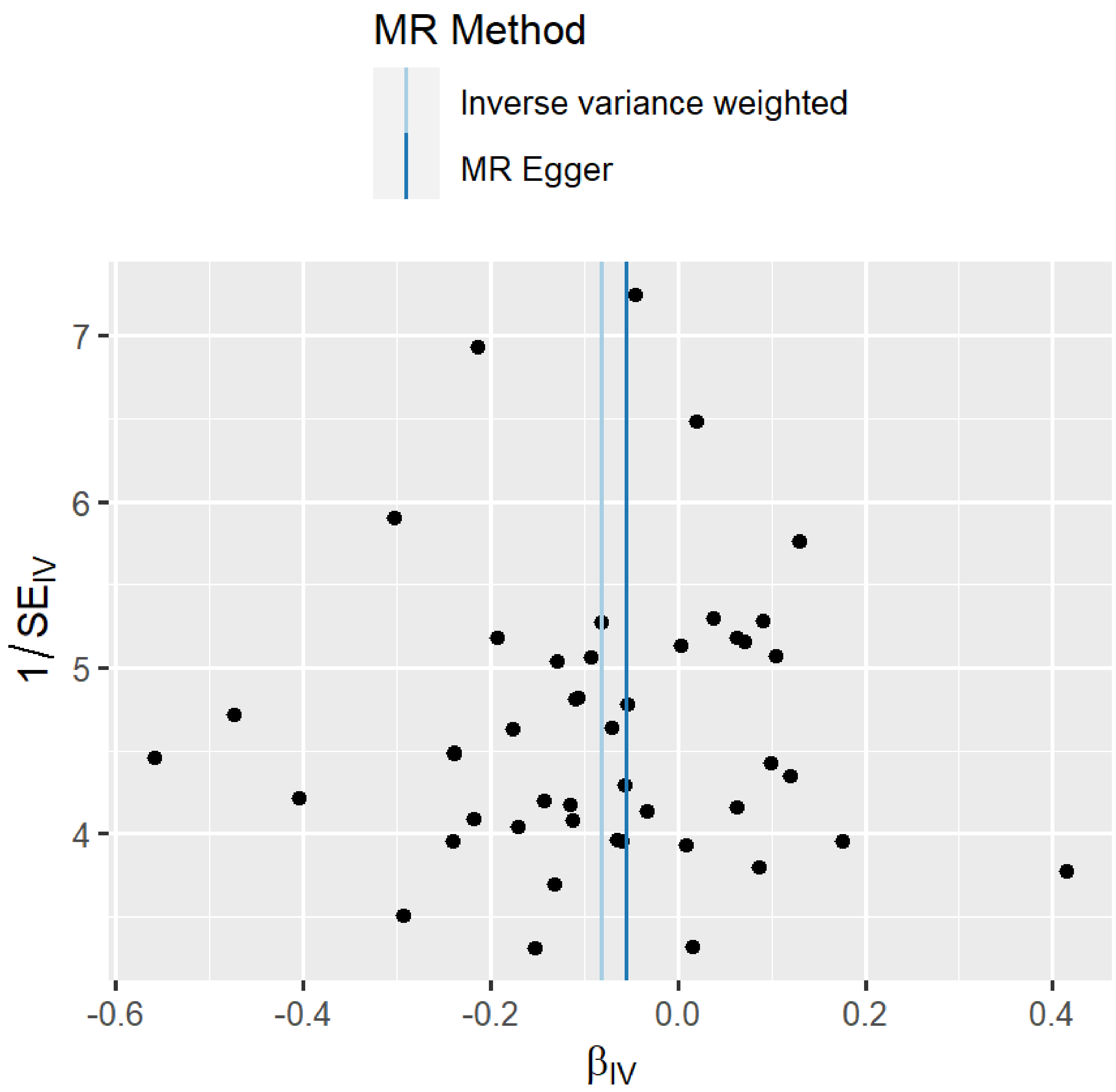
MR Method
Inverse variance weighted
MR Egger
7
6
5
4
-0.6
-0.4
-0.2
0.0
0.2
0.4
$1/SE_{IV}$
β_{IV}

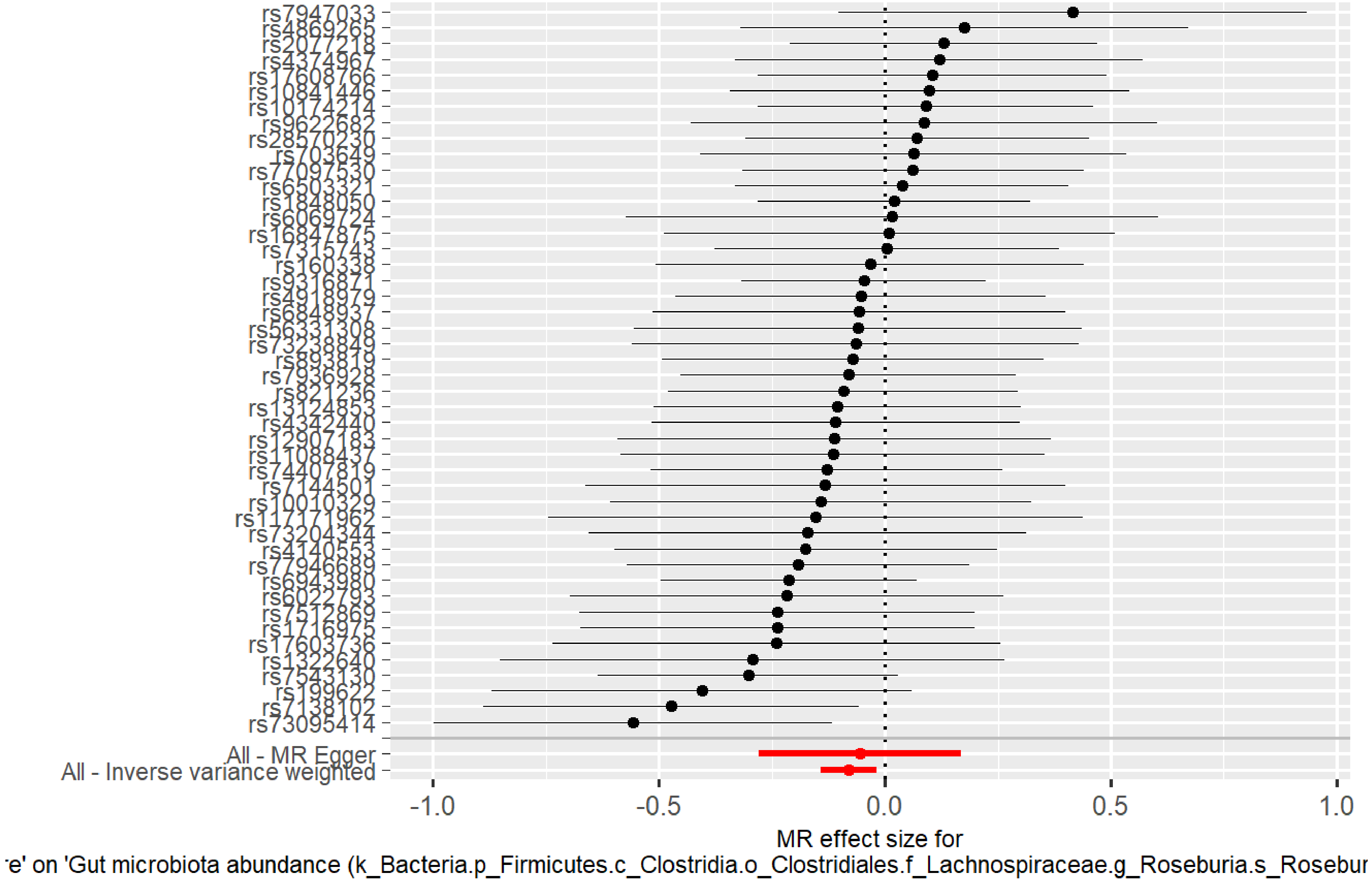
rs7947033
rs4869265
rs2077218
rs4374967
rs17608766
rs10841446
rs10174214
rs9622682
rs28570230
rs703649
rs77097530
rs6503321
rs1848050
rs6069724
rs16847875
rs7315743
rs160338
rs9316871
rs4918979
rs6848937
rs56331308
rs73238849
rs893819
rs7936928
rs821236
rs13124853
rs4342440
rs12907183
rs11088437
rs74407819
rs7144501
rs10010329
rs117171962
rs73204344
rs4140553
rs77946689
rs6943980
rs6022793
rs7512869
rs1716975
rs17603736
rs1322640
rs7543130
rs199622
rs7138102
rs73095414
All - MR Egger
All - Inverse variance weighted
-1.0
-0.5
0.0
0.5
1.0
MR effect size for
'e' on 'Gut microbiota abundance (k_Bacteria.p_Firmicutes.c_Clostridia.o_Clostridiales.f_Lachnospiraceae.g_Roseburia.s_Rosebur

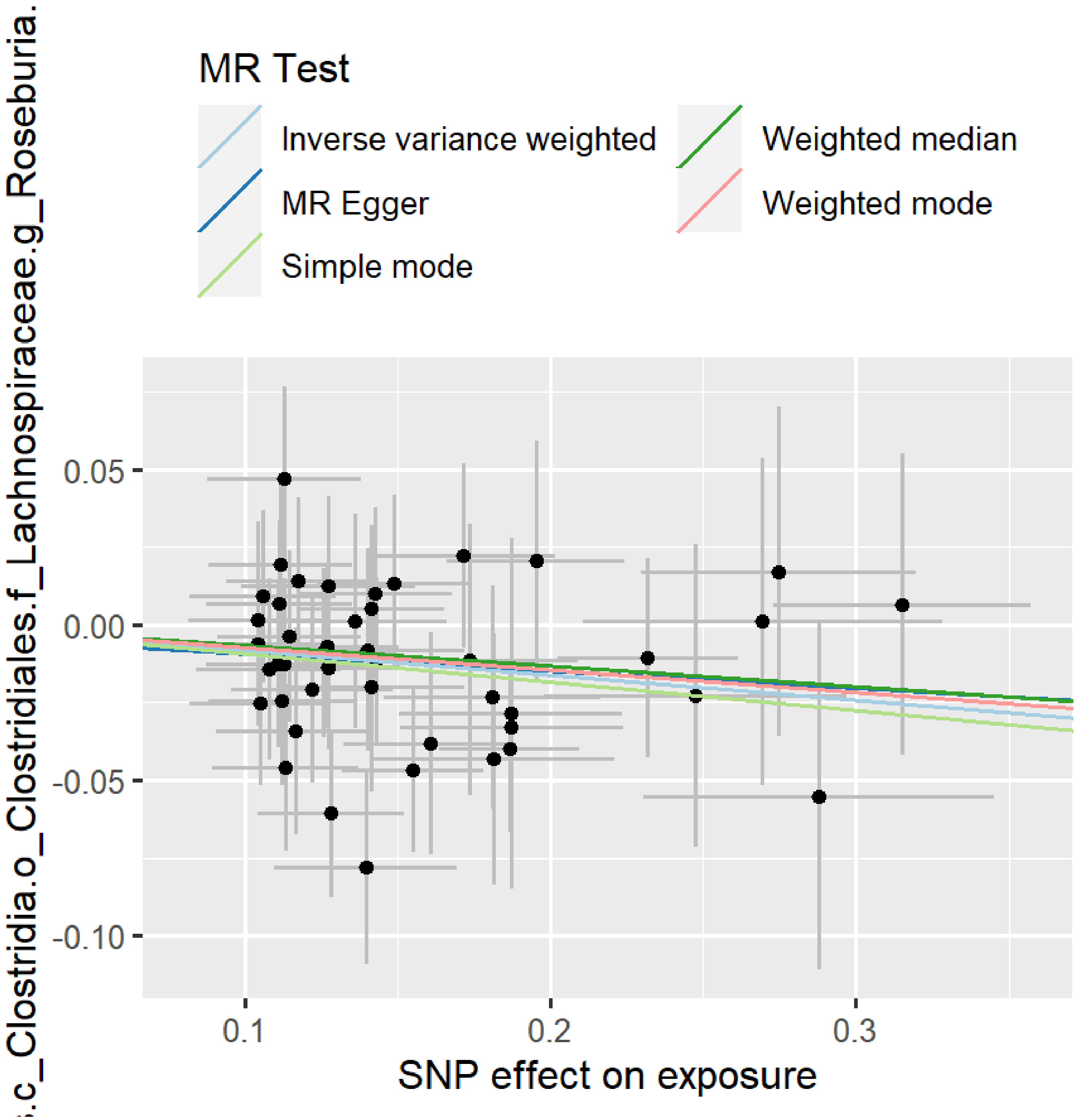
MR Test
Inverse variance weighted
MR Egger
Simple mode
Weighted median
Weighted mode
.c_Clostridia.o_Clostridiales.f_Lachnospiraceae.g_Roseburia.
0.05
0.00
-0.05
-0.10
0.1
0.2
0.3
SNP effect on exposure

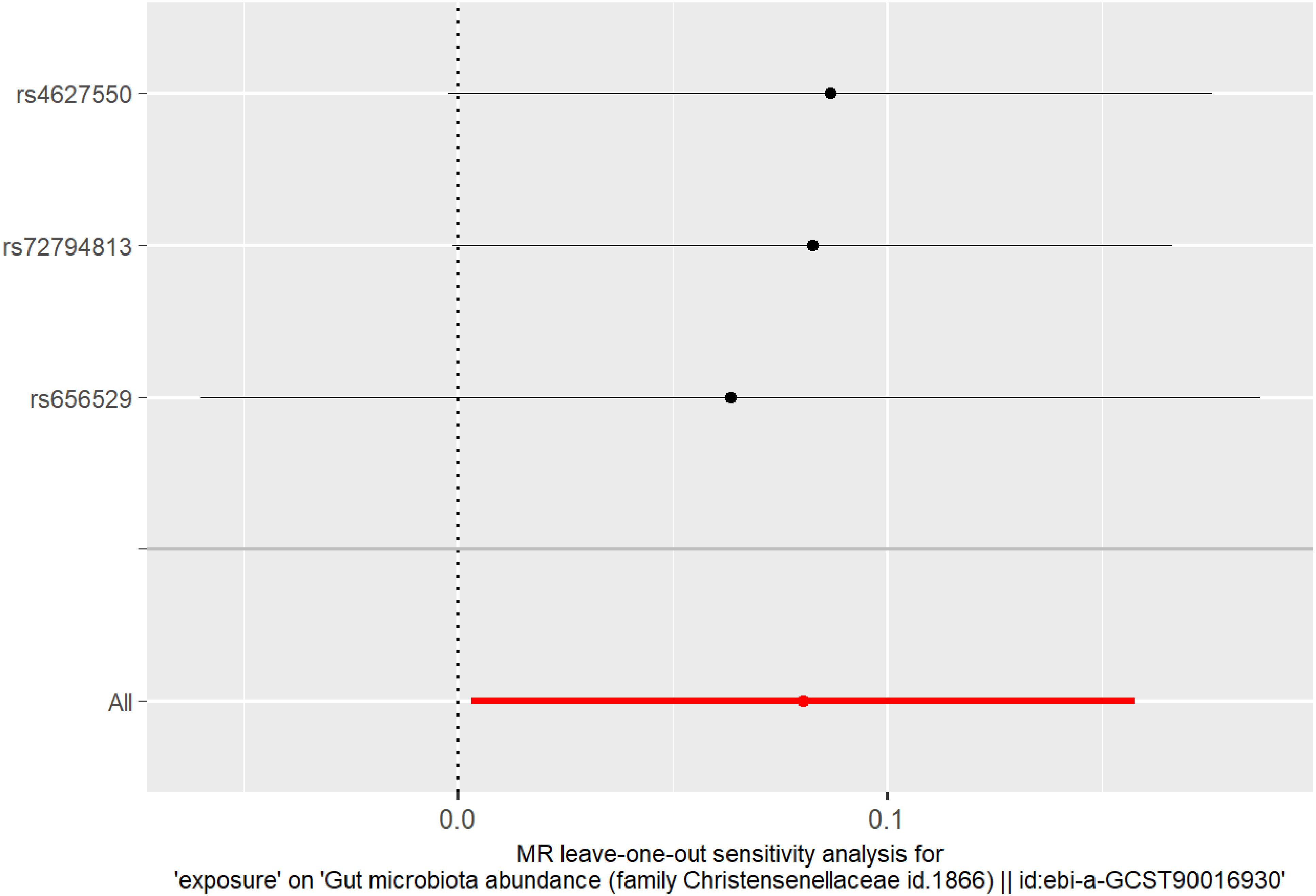
rs4627550
rs72794813
rs656529
All
0.0
0.1
MR leave-one-out sensitivity analysis for
'exposure' on 'Gut microbiota abundance (family Christensenellaceae id.1866) || id:ebi-a-GCST90016930'

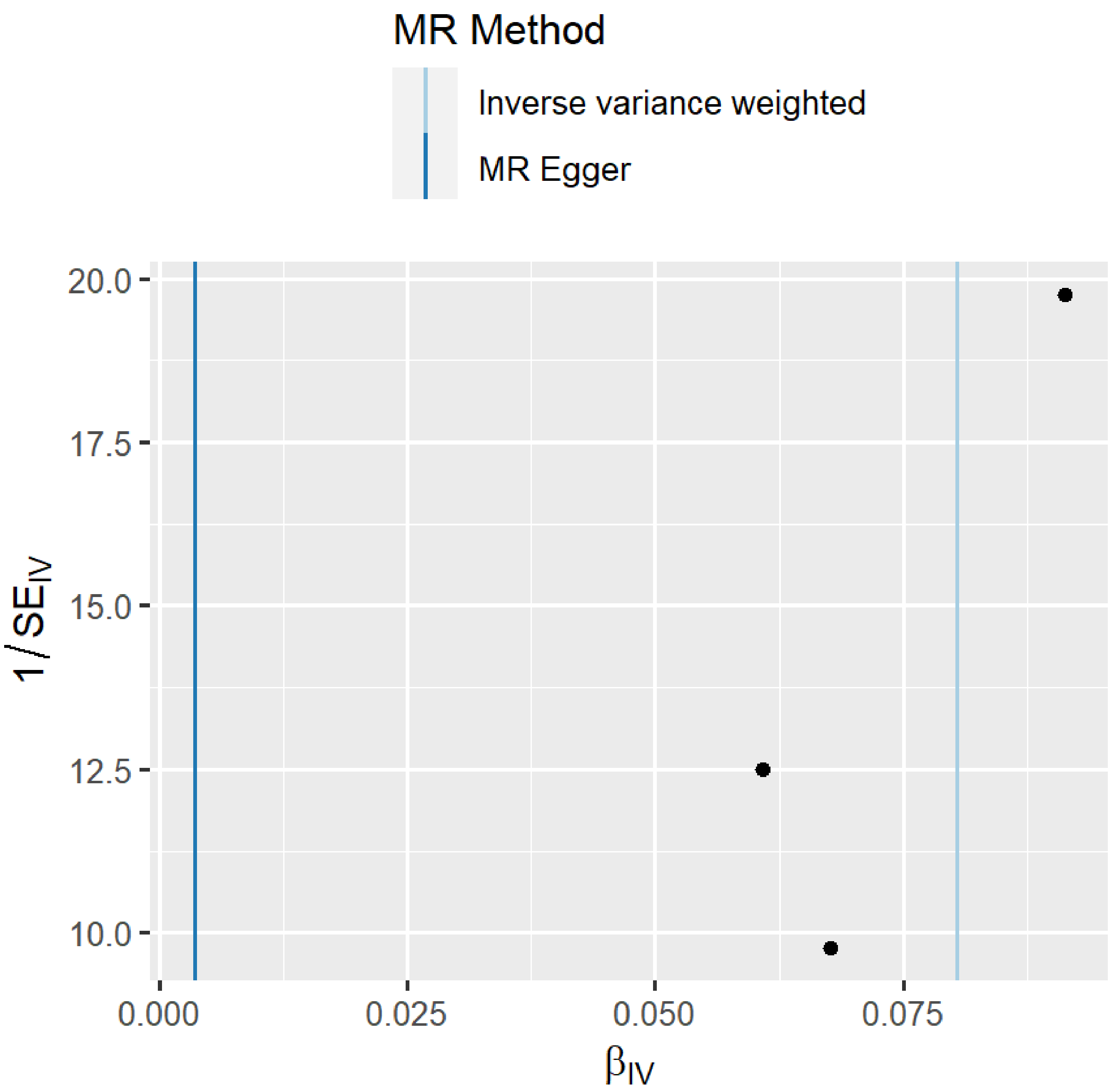
MR Method
Inverse variance weighted
MR Egger
20.0
17.5
15.0
12.5
10.0
1/SE_IV
0.000
0.025
0.050
0.075
β_IV

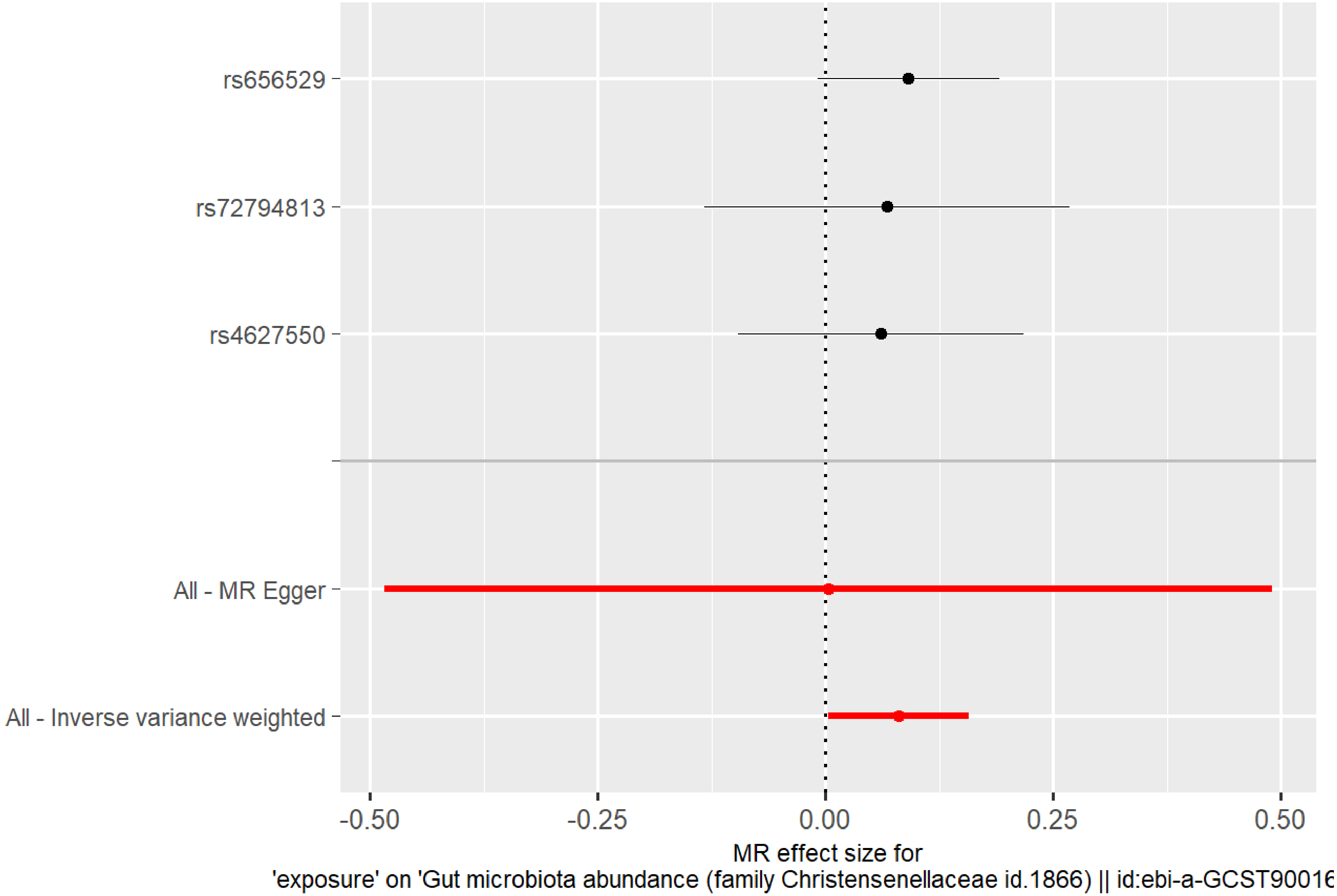
rs656529
rs72794813
rs4627550
All - MR Egger
All - Inverse variance weighted
-0.50
-0.25
0.00
0.25
0.50
MR effect size for
'exposure' on 'Gut microbiota abundance (family Christensenellaceae id.1866) || id:ebi-a-GCST90016

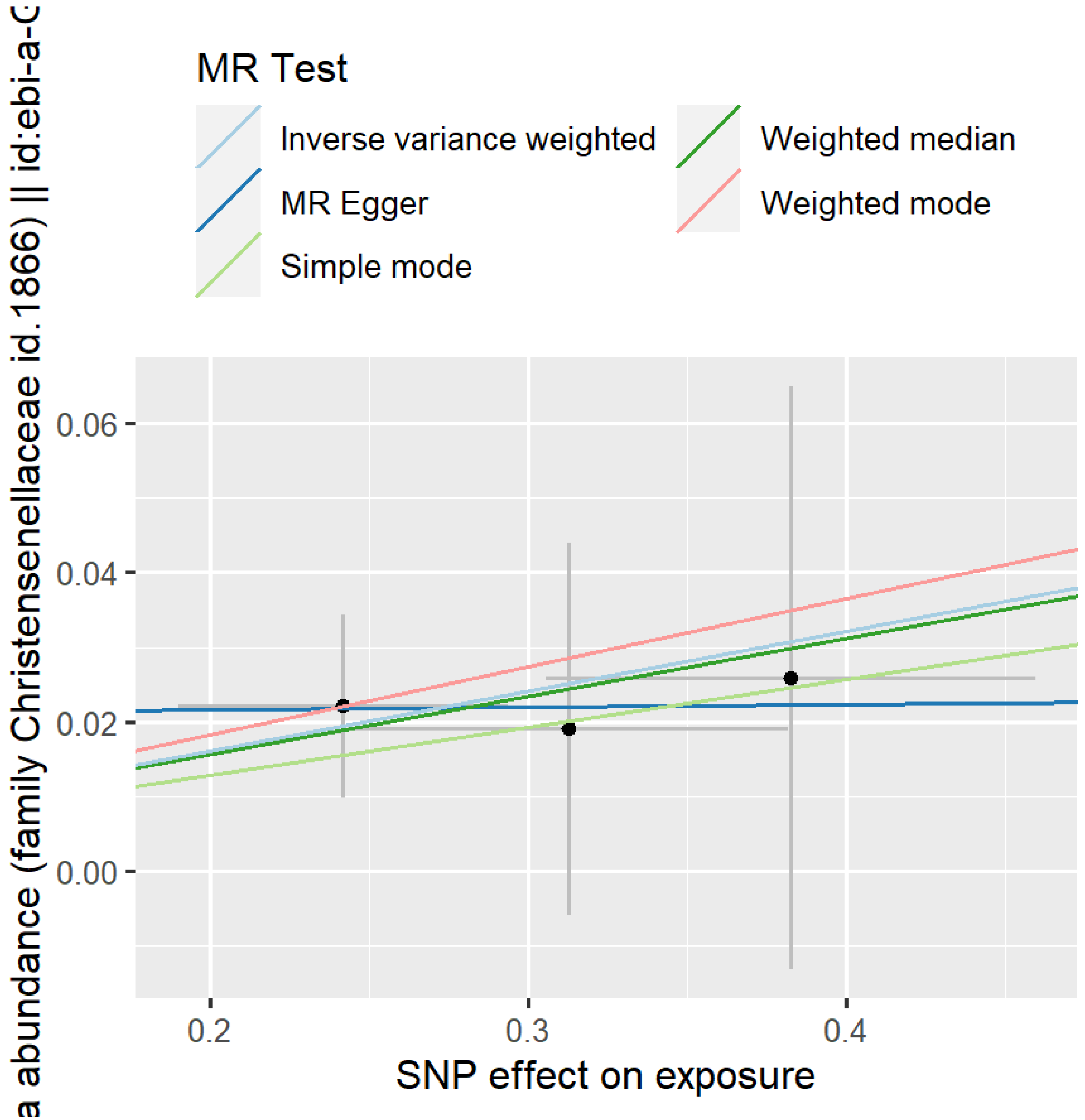
MR Test
Inverse variance weighted
MR Egger
Simple mode
Weighted median
Weighted mode
a abundance (family Christensenellaceae id.1866) || id:ebi-a-G
0.06
0.04
0.02
0.00
0.2
0.3
0.4
SNP effect on exposure

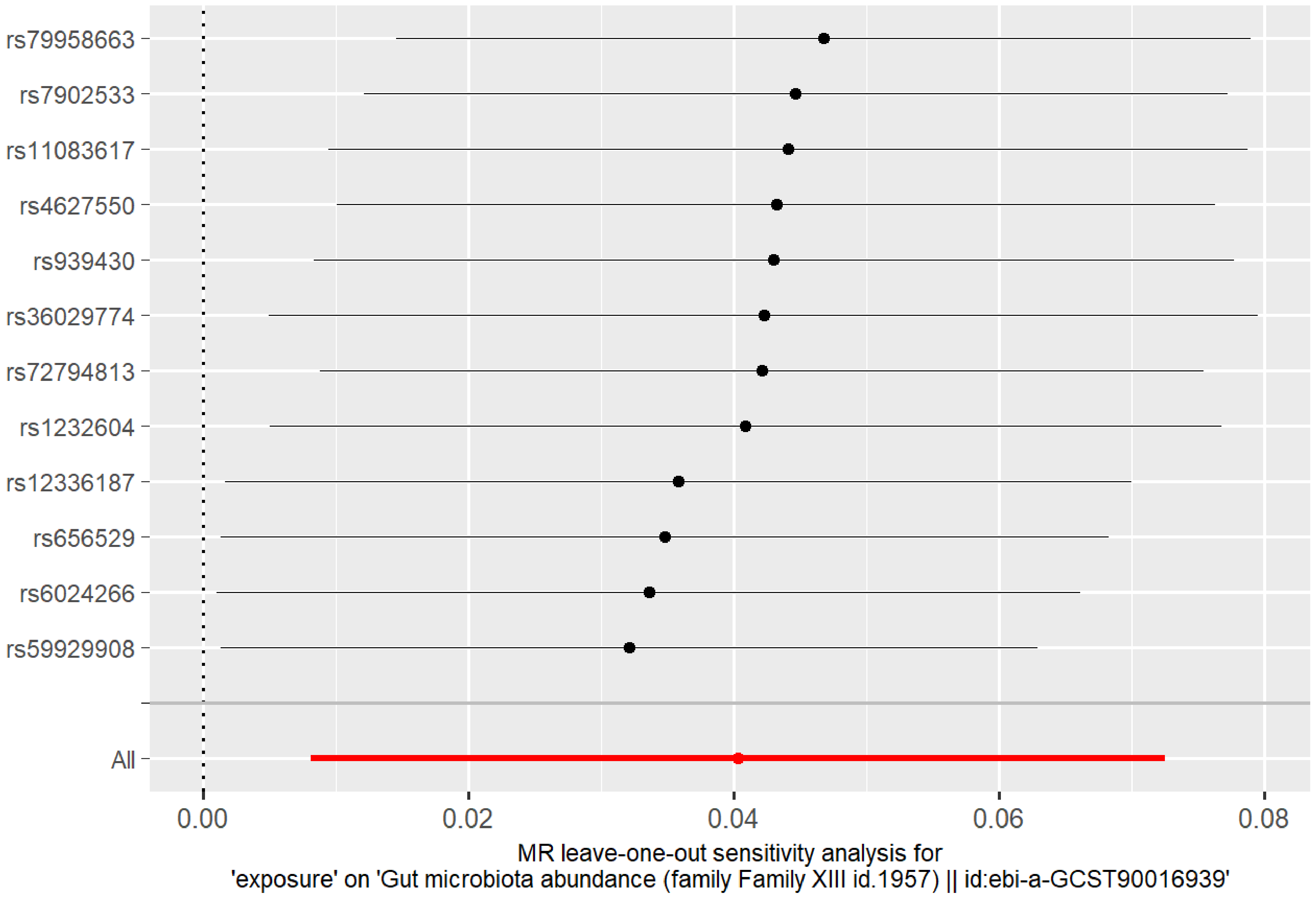
rs79958663
rs7902533
rs11083617
rs4627550
rs939430
rs36029774
rs72794813
rs1232604
rs12336187
rs656529
rs6024266
rs59929908
All
0.00
0.02
0.04
0.06
0.08
MR leave-one-out sensitivity analysis for
'exposure' on 'Gut microbiota abundance (family Family XIII id.1957) || id:ebi-a-GCST90016939'

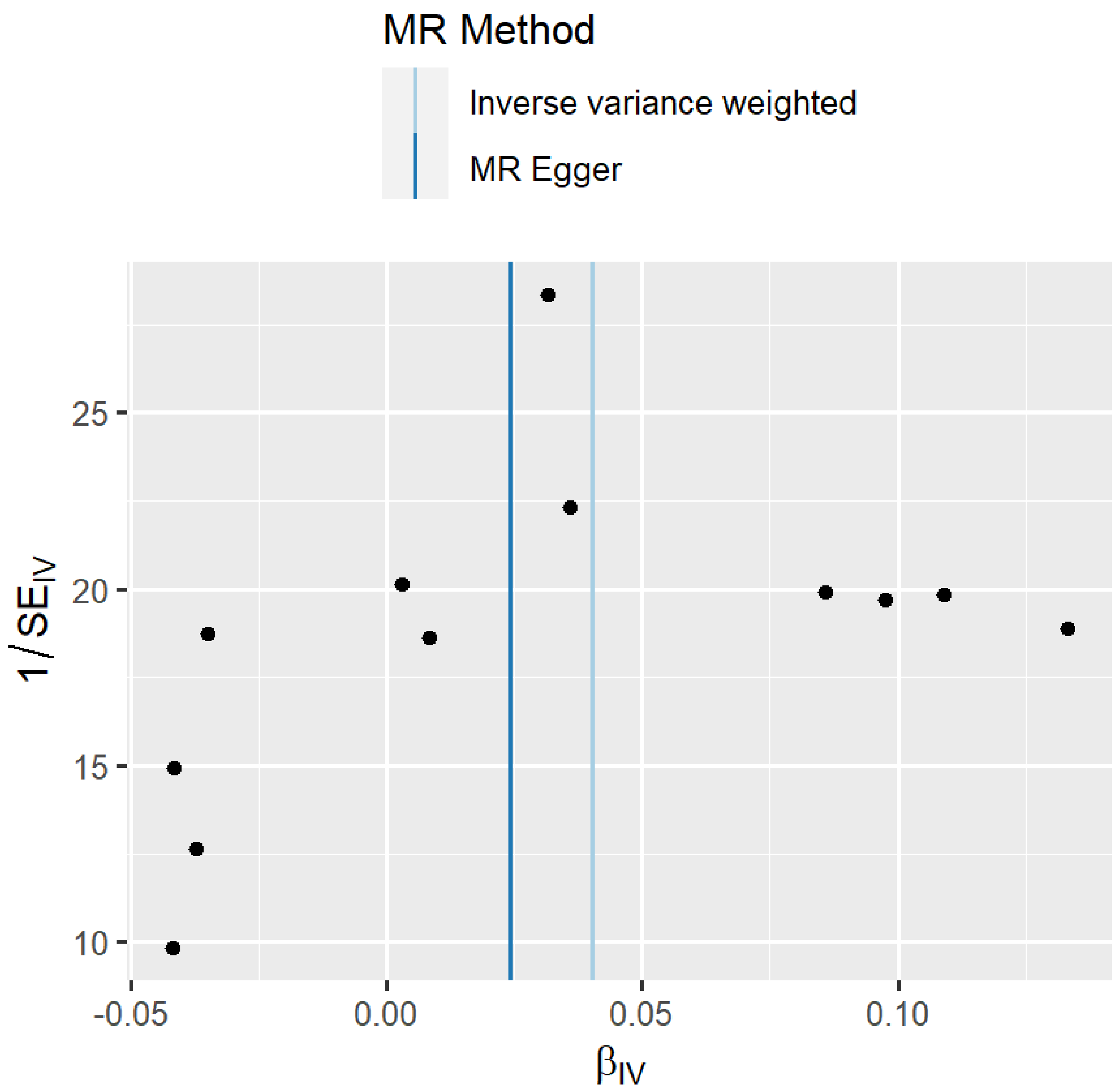
MR Method
Inverse variance weighted
MR Egger
25
20
15
10
-0.05
0.00
0.05
0.10
$1/SE_{IV}$
β_{IV}

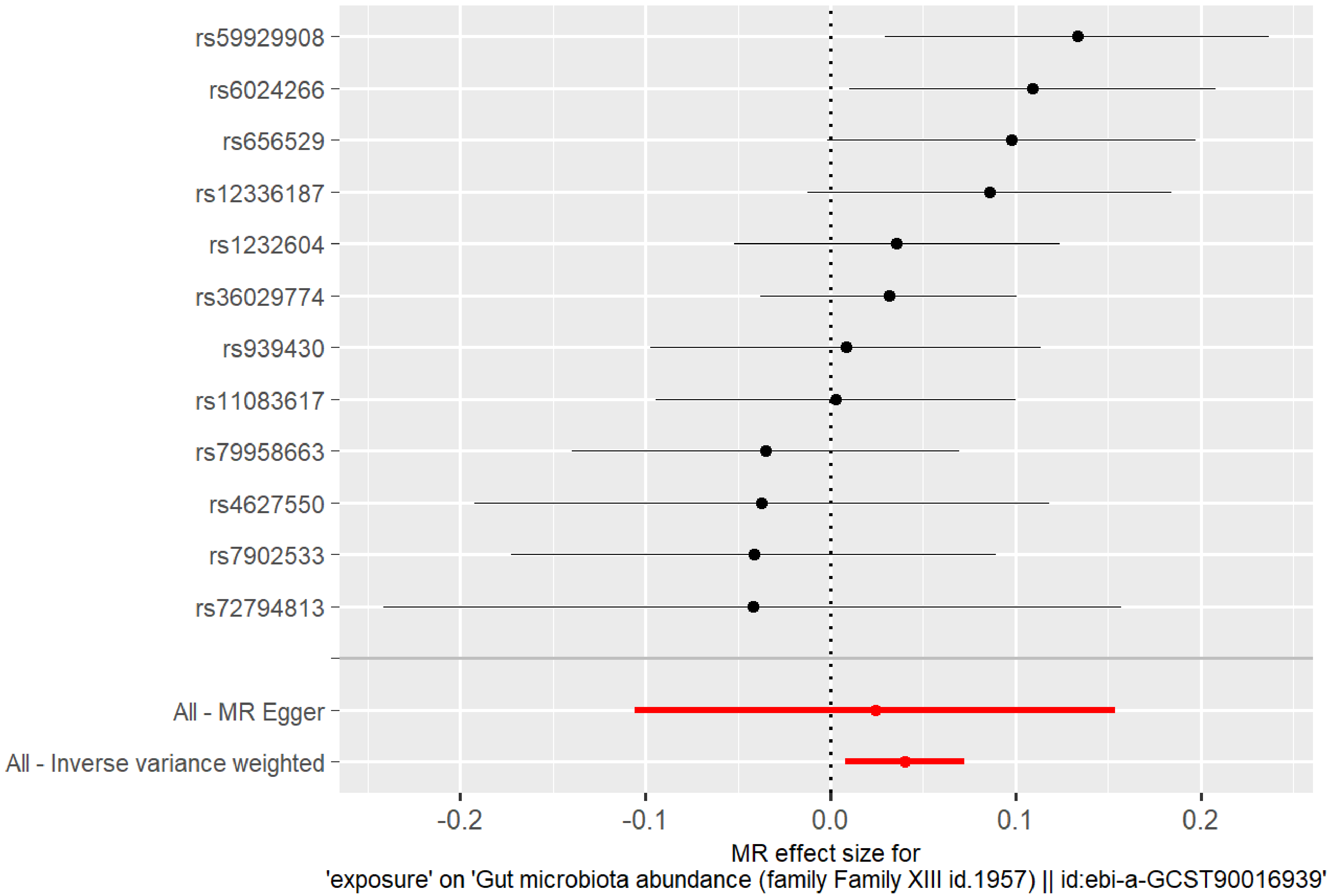
rs59929908
rs6024266
rs656529
rs12336187
rs1232604
rs36029774
rs939430
rs11083617
rs79958663
rs4627550
rs7902533
rs72794813
All - MR Egger
All - Inverse variance weighted
-0.2
-0.1
0.0
0.1
0.2
MR effect size for
'exposure' on 'Gut microbiota abundance (family Family XIII id.1957) || id:ebi-a-GCST90016939'

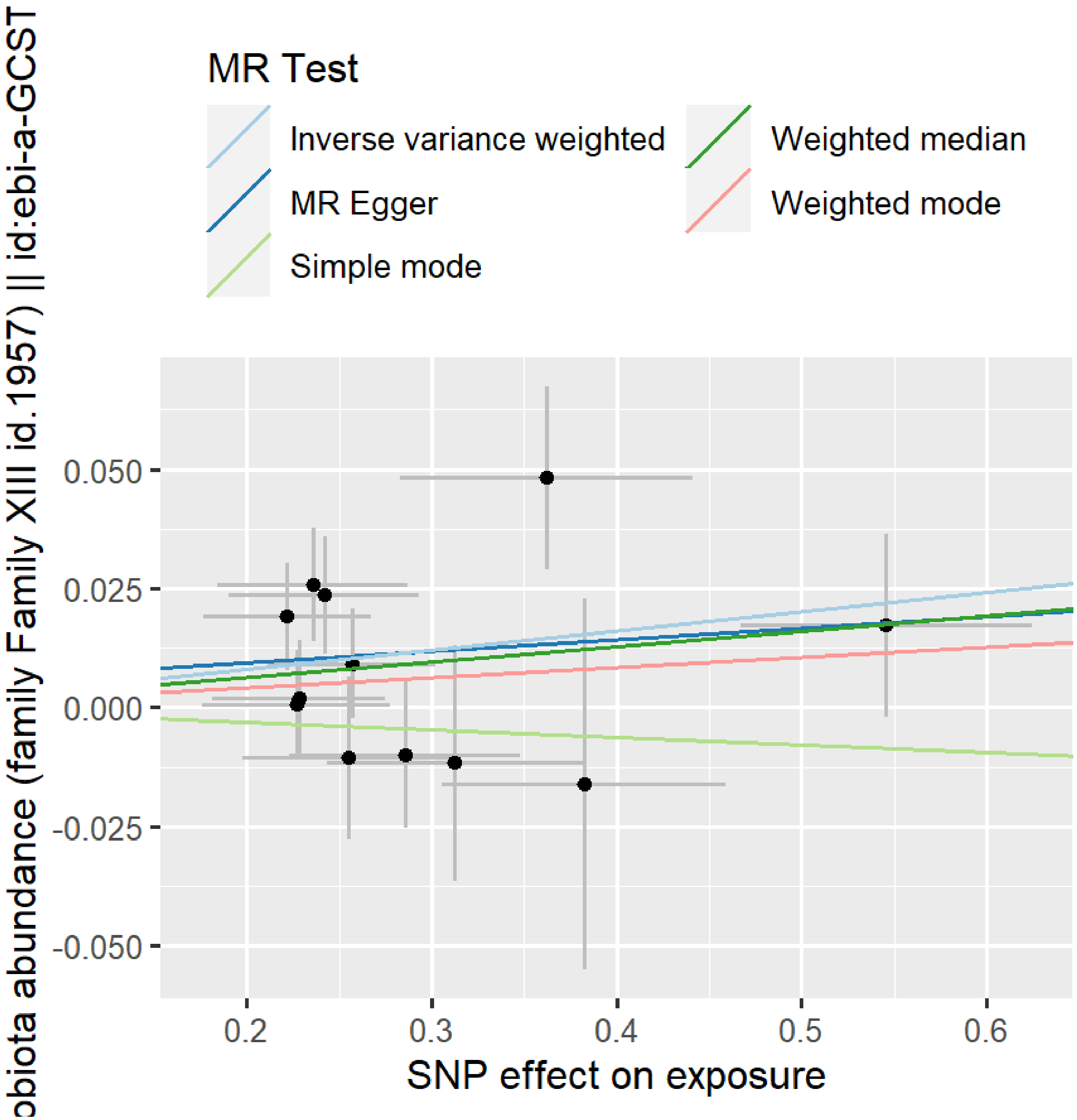
MR Test
Inverse variance weighted
MR Egger
Simple mode
Weighted median
Weighted mode
obiota abundance (family Family XIII id.1957) || id:ebi-a-GCST
0.050
0.025
0.000
-0.025
-0.050
0.2
0.3
0.4
0.5
0.6
SNP effect on exposure

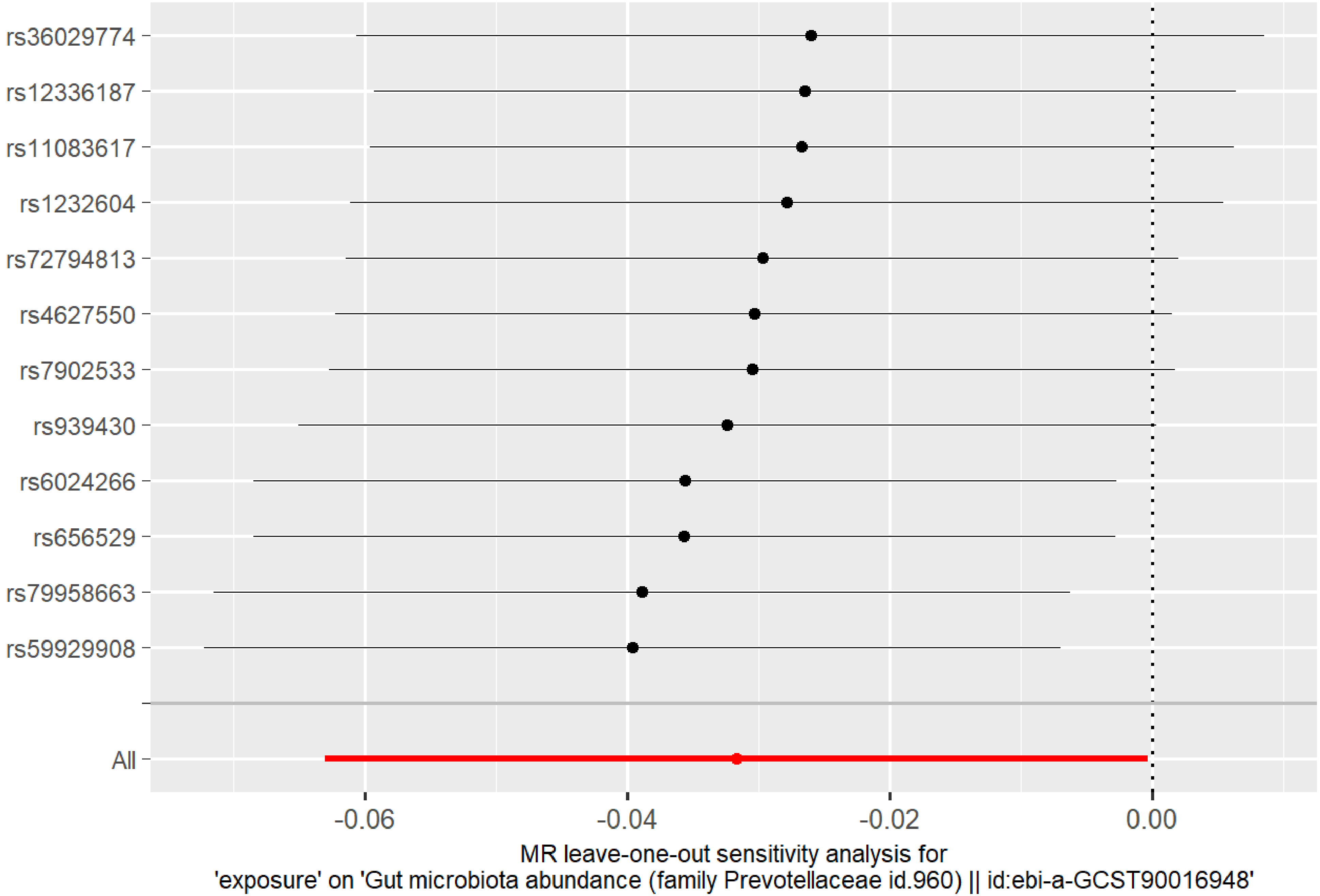
rs36029774
rs12336187
rs11083617
rs1232604
rs72794813
rs4627550
rs7902533
rs939430
rs6024266
rs656529
rs79958663
rs59929908
All
-0.06
-0.04
-0.02
0.00
MR leave-one-out sensitivity analysis for
'exposure' on 'Gut microbiota abundance (family Prevotellaceae id.960) || id:ebi-a-GCST90016948'

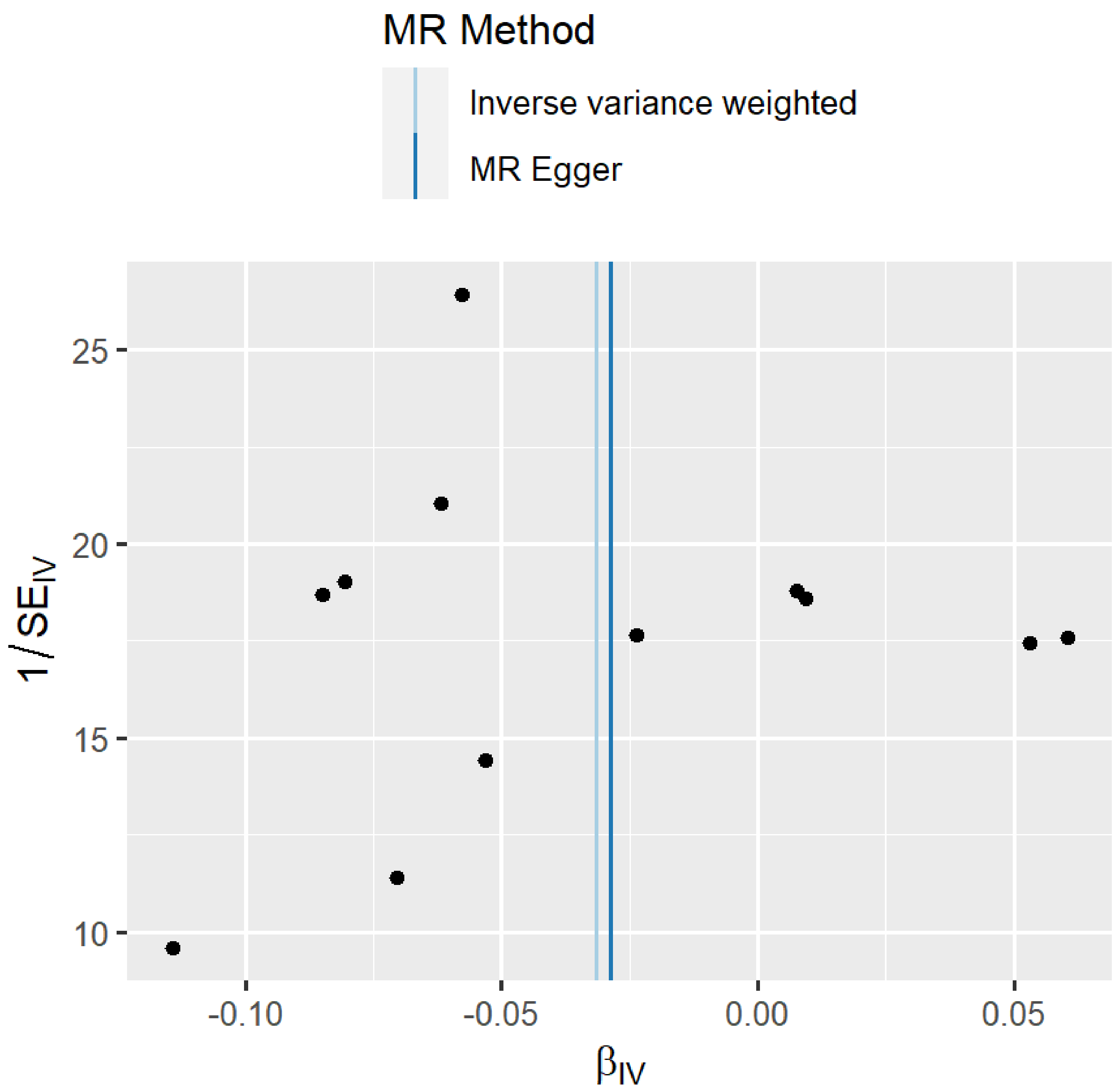
MR Method
Inverse variance weighted
MR Egger
25
20
15
10
1/SE_IV
-0.10
-0.05
0.00
0.05
β_IV

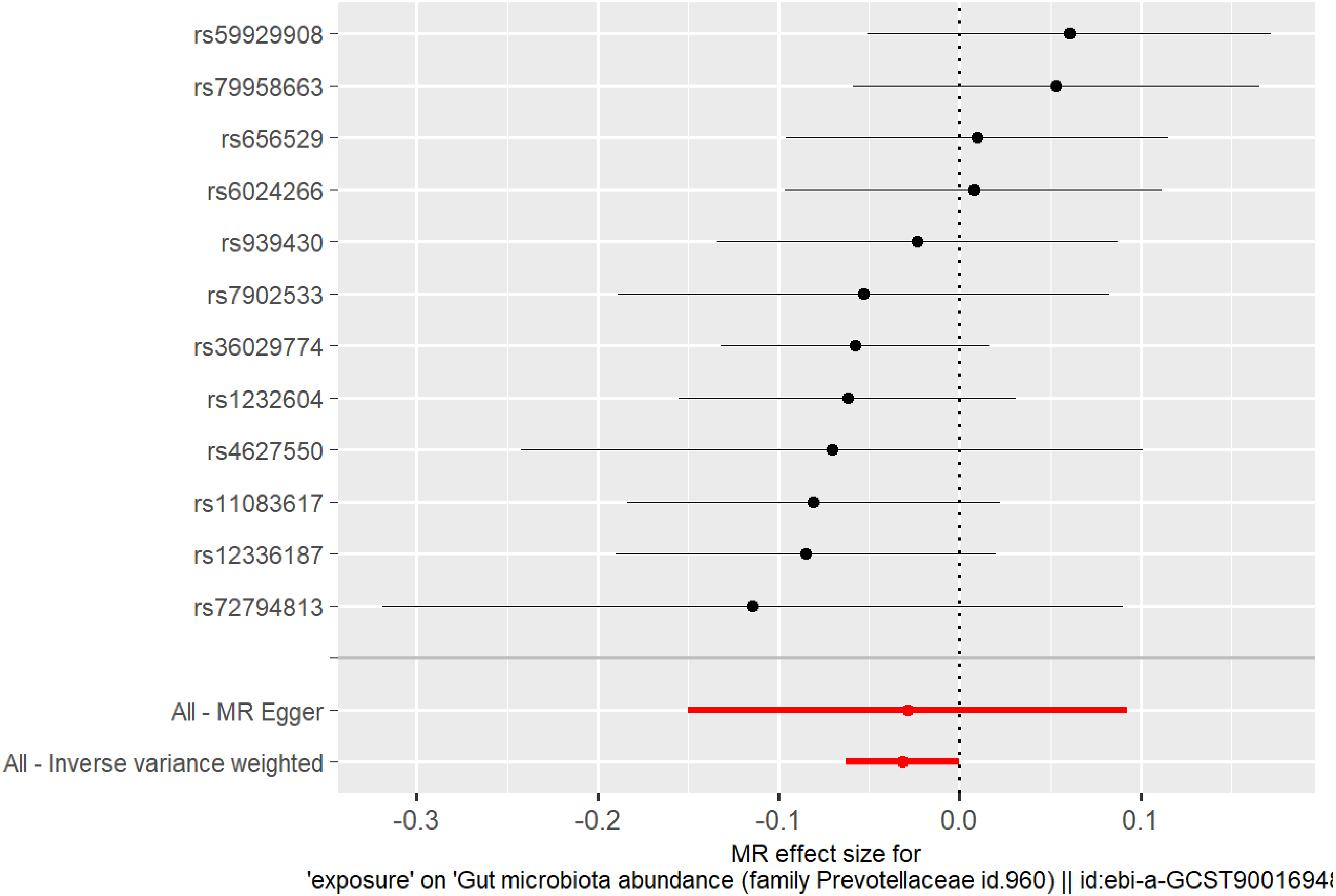

rs59929908
rs79958663
rs656529
rs6024266
rs939430
rs7902533
rs36029774
rs1232604
rs4627550
rs11083617
rs12336187
rs72794813
All - MR Egger
All - Inverse variance weighted
-0.3
-0.2
-0.1
0.0
0.1
MR effect size for
'exposure' on 'Gut microbiota abundance (family Prevotellaceae id.960) || id:ebi-a-GCST9001694

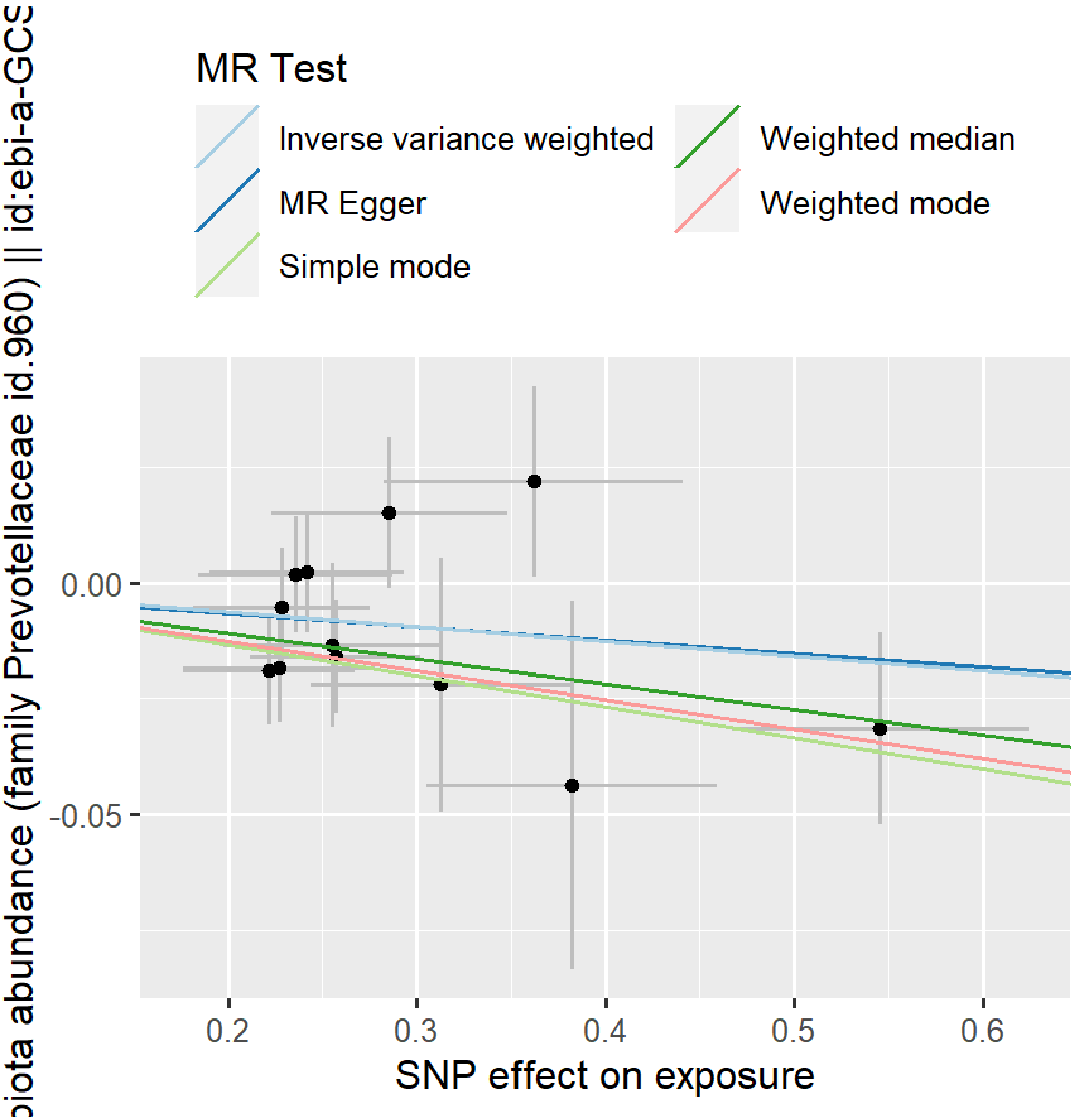

MR Test
Inverse variance weighted
MR Egger
Simple mode
Weighted median
Weighted mode
0.00
-0.05
0.2
0.3
0.4
0.5
0.6
SNP effect on exposure
biota abundance (family Prevotellaceae id.960) || id:ebi-a-GCS

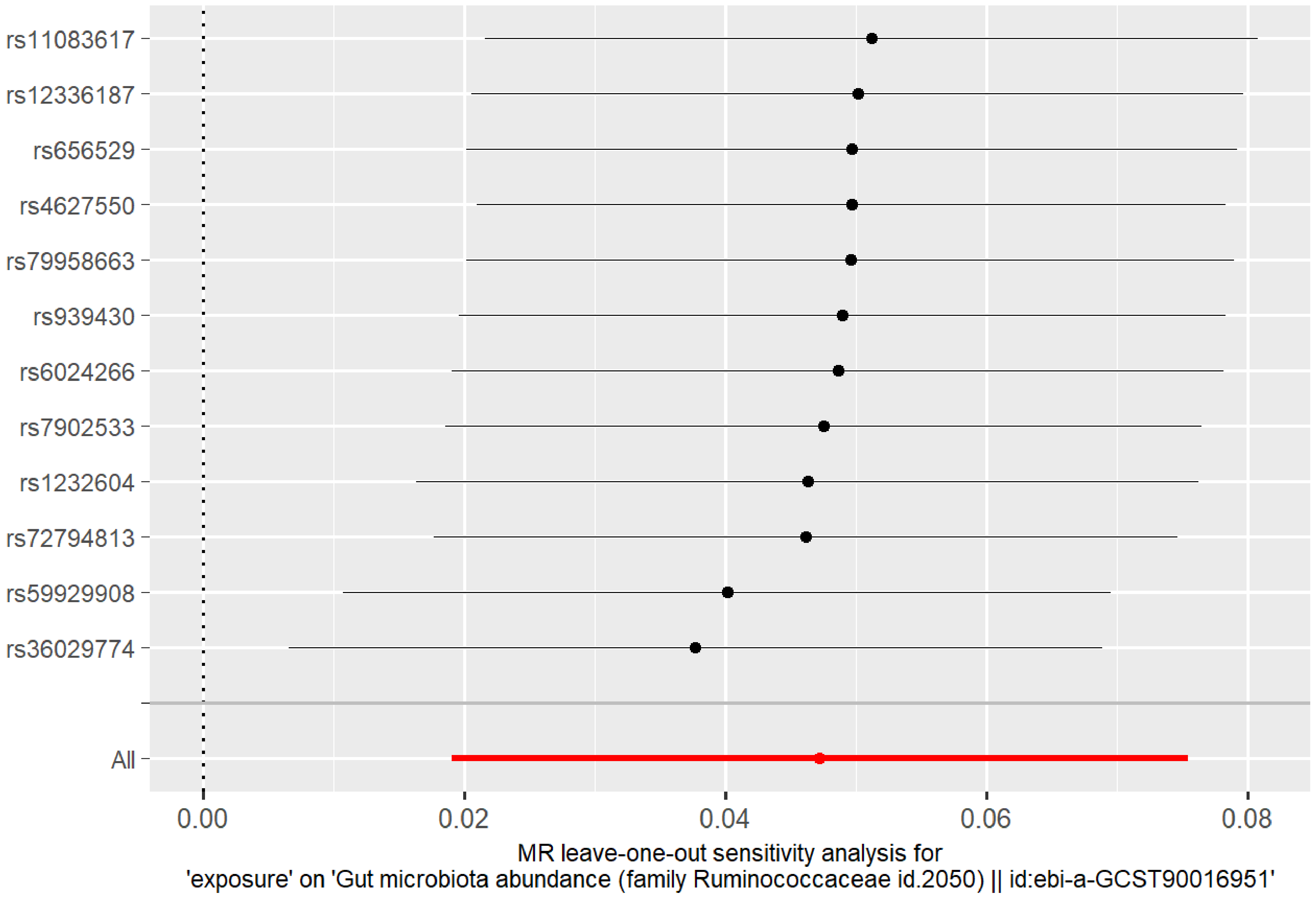
rs11083617
rs12336187
rs656529
rs4627550
rs79958663
rs939430
rs6024266
rs7902533
rs1232604
rs72794813
rs59929908
rs36029774
All
0.00
0.02
0.04
0.06
0.08
MR leave-one-out sensitivity analysis for
'exposure' on 'Gut microbiota abundance (family Ruminococcaceae id.2050) || id:ebi-a-GCST90016951'

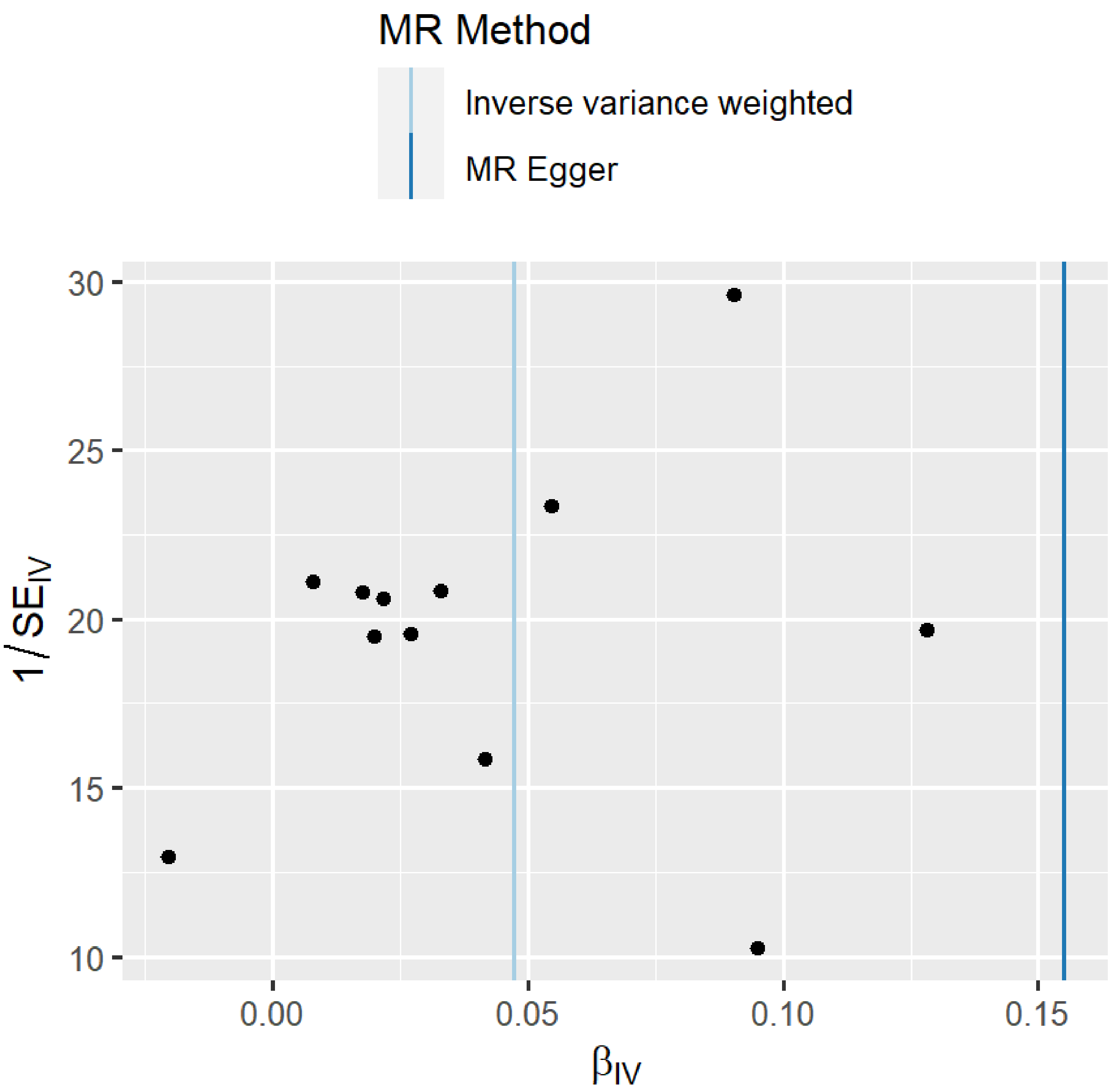
MR Method
Inverse variance weighted
MR Egger
30
25
20
15
10
1/SE_IV
0.00
0.05
0.10
0.15
β_IV

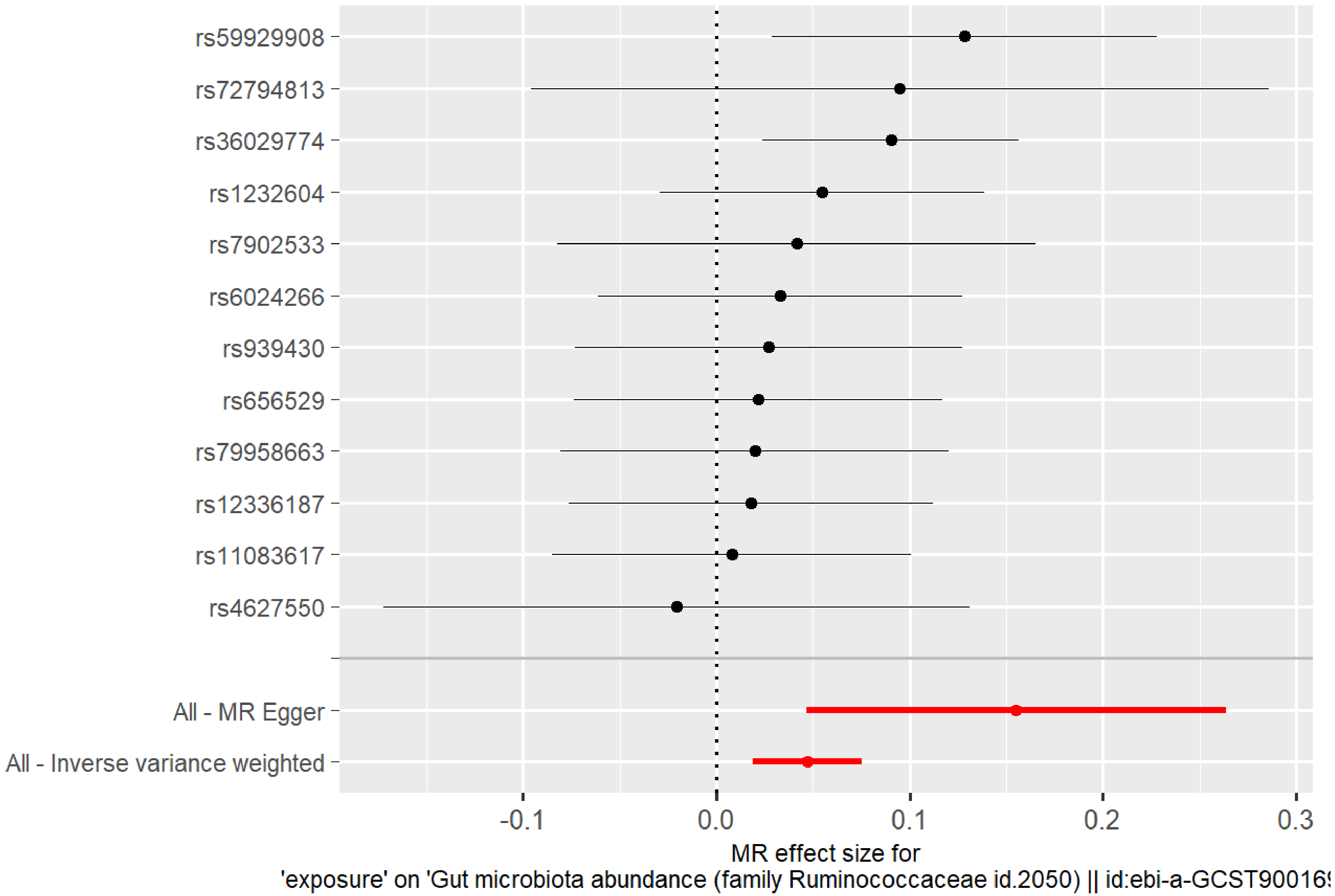
rs59929908
rs72794813
rs36029774
rs1232604
rs7902533
rs6024266
rs939430
rs656529
rs79958663
rs12336187
rs11083617
rs4627550
All - MR Egger
All - Inverse variance weighted
-0.1
0.0
0.1
0.2
0.3
MR effect size for
'exposure' on 'Gut microbiota abundance (family Ruminococcaceae id.2050) || id:ebi-a-GCST90016

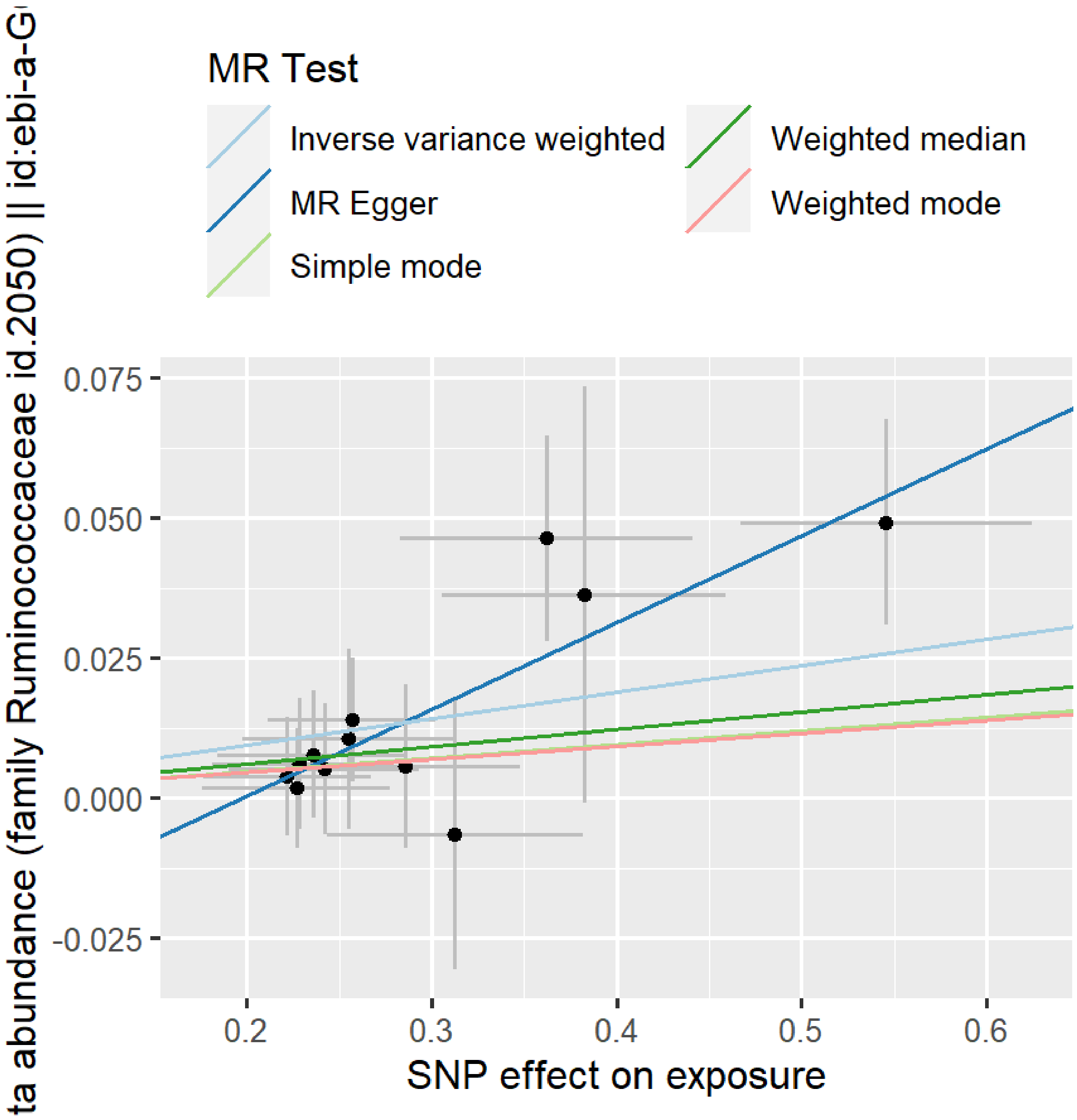

MR Test
Inverse variance weighted
MR Egger
Simple mode
Weighted median
Weighted mode
0.075
0.050
0.025
0.000
-0.025
0.2
0.3
0.4
0.5
0.6
SNP effect on exposure
ta abundance (family Ruminococcaceae id.2050) || id:ebi-a-G

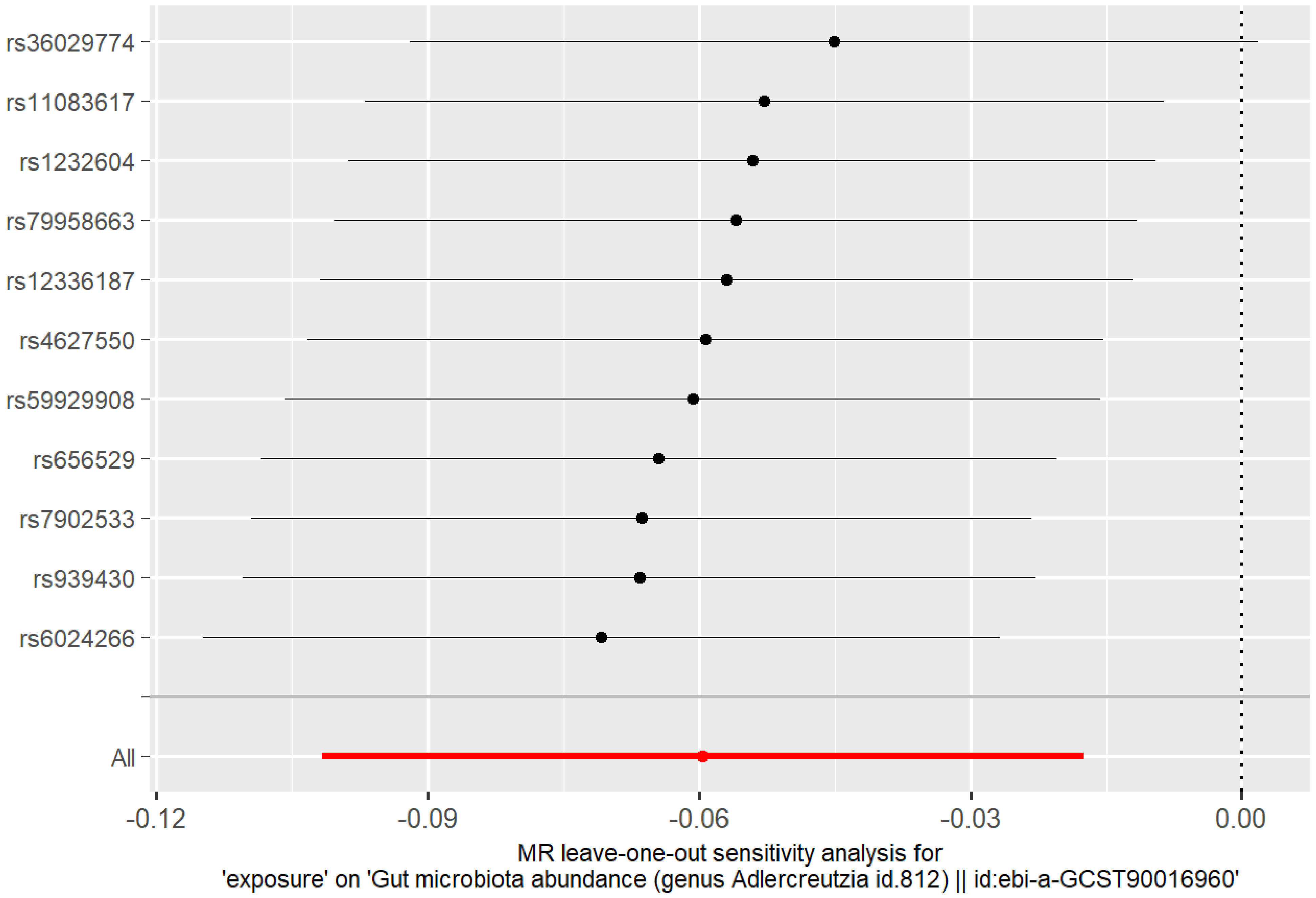
rs36029774
rs11083617
rs1232604
rs79958663
rs12336187
rs4627550
rs59929908
rs656529
rs7902533
rs939430
rs6024266
All
-0.12
-0.09
-0.06
-0.03
0.00
MR leave-one-out sensitivity analysis for
'exposure' on 'Gut microbiota abundance (genus Adlercreutzia id.812) || id:ebi-a-GCST90016960'

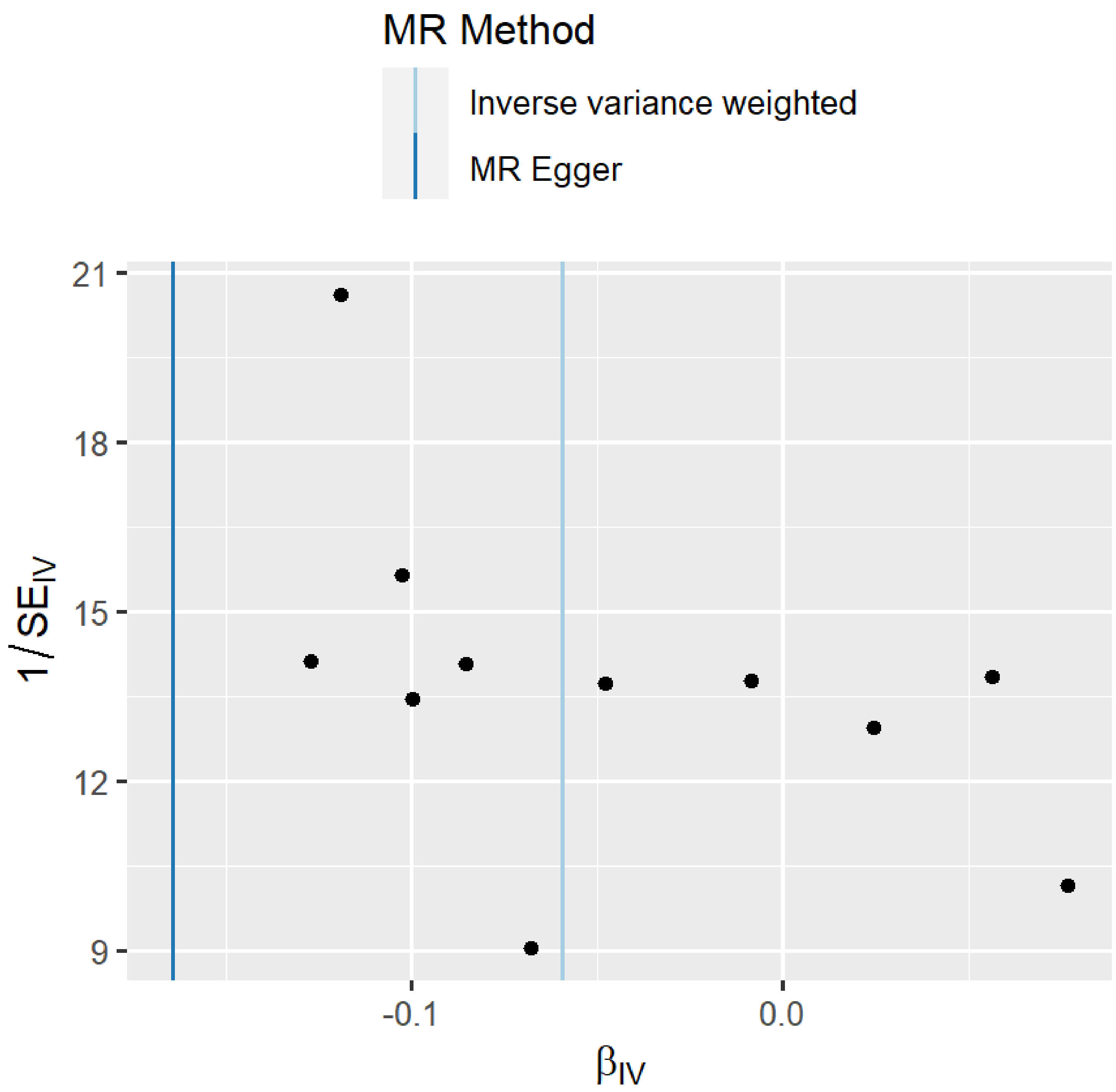
MR Method
Inverse variance weighted
MR Egger
21
18
15
12
9
-0.1
0.0
β_{IV}
$1/SE_{IV}$

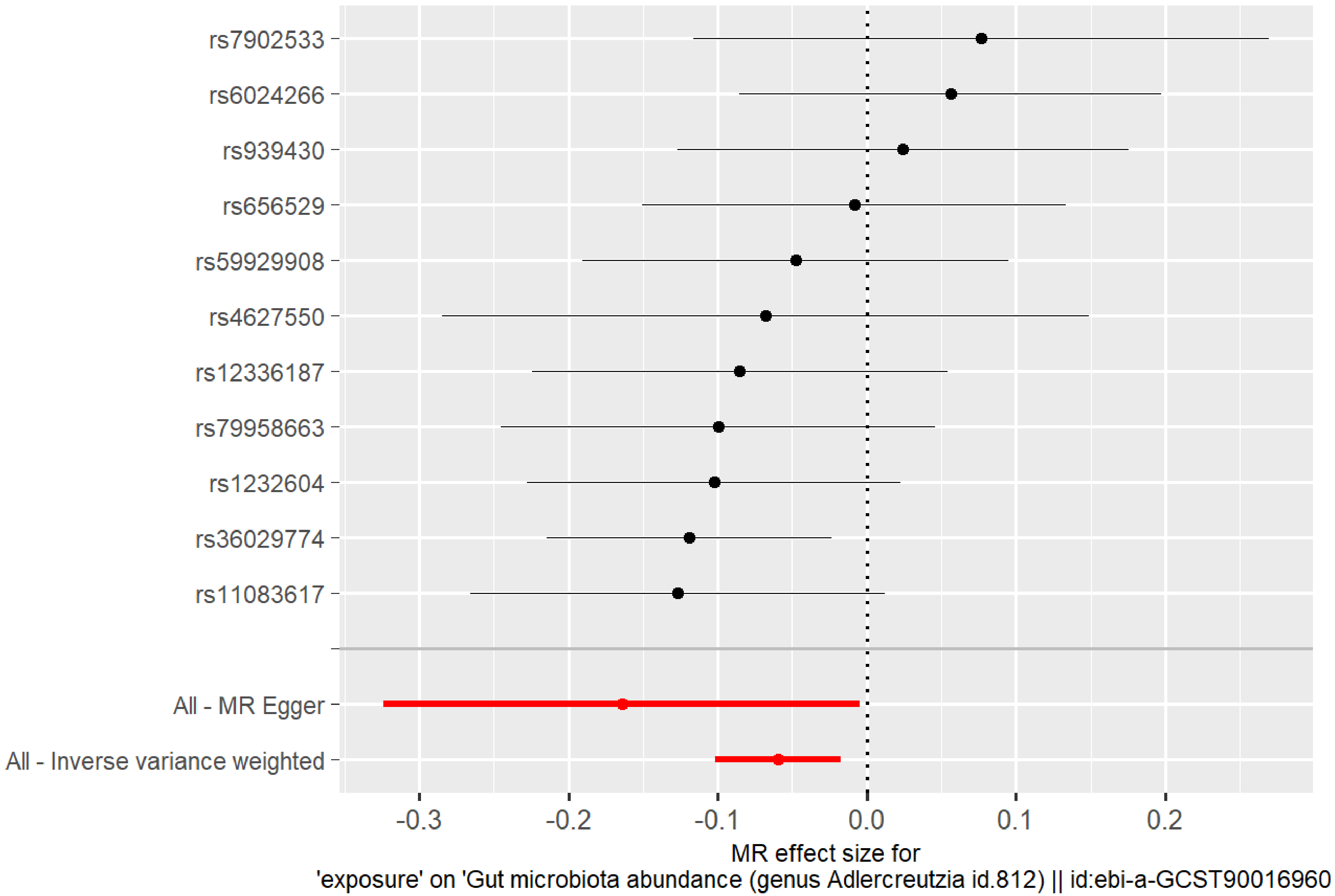
rs7902533
rs6024266
rs939430
rs656529
rs59929908
rs4627550
rs12336187
rs79958663
rs1232604
rs36029774
rs11083617
All - MR Egger
All - Inverse variance weighted
-0.3
-0.2
-0.1
0.0
0.1
0.2
MR effect size for
'exposure' on 'Gut microbiota abundance (genus Adlercreutzia id.812) || id:ebi-a-GCST90016960

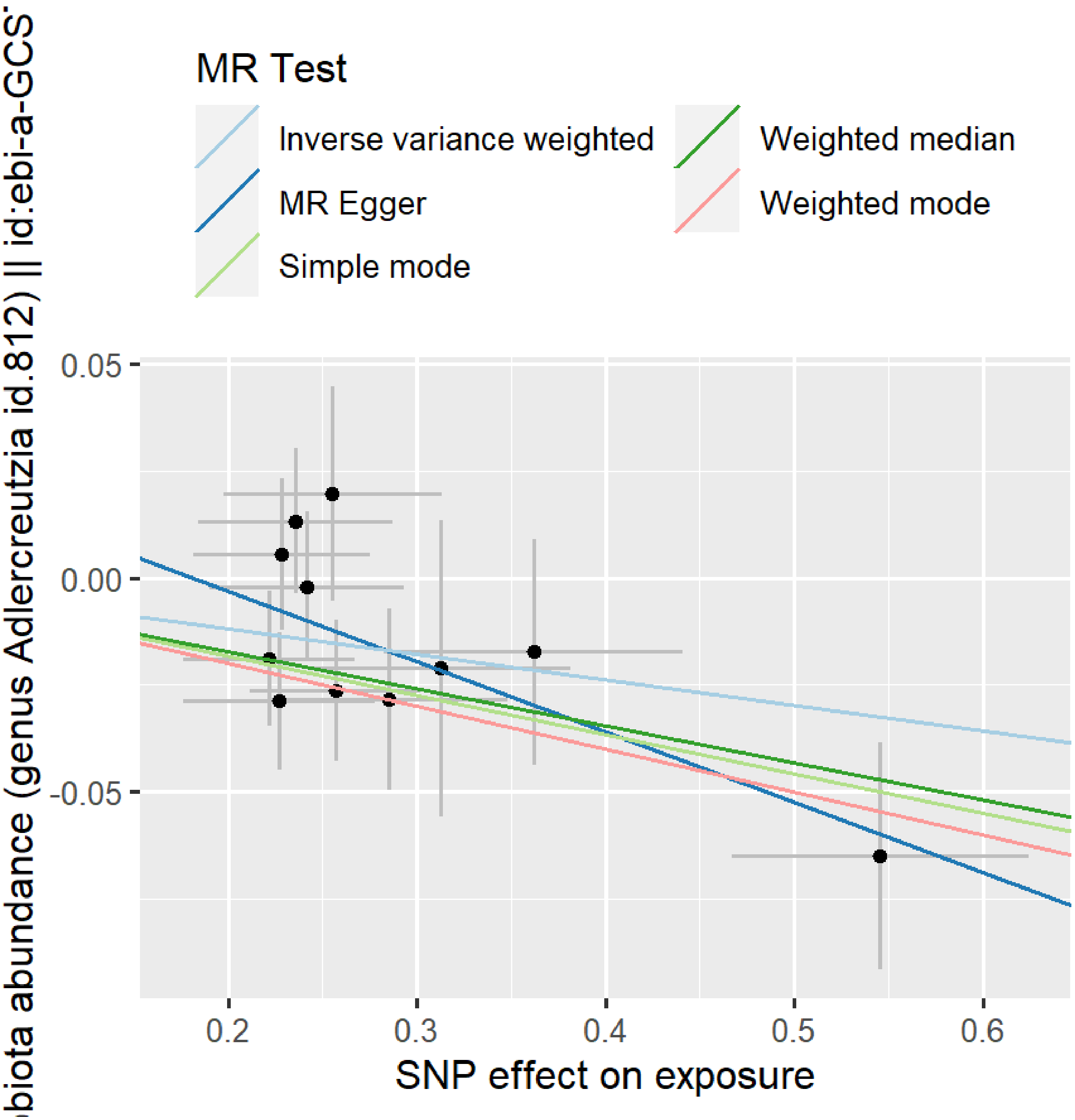
MR Test
Inverse variance weighted
MR Egger
Simple mode
Weighted median
Weighted mode
0.05
0.00
-0.05
0.2
0.3
0.4
0.5
0.6
SNP effect on exposure
biota abundance (genus Adlercreutzia id.812) || id:ebi-a-GCS

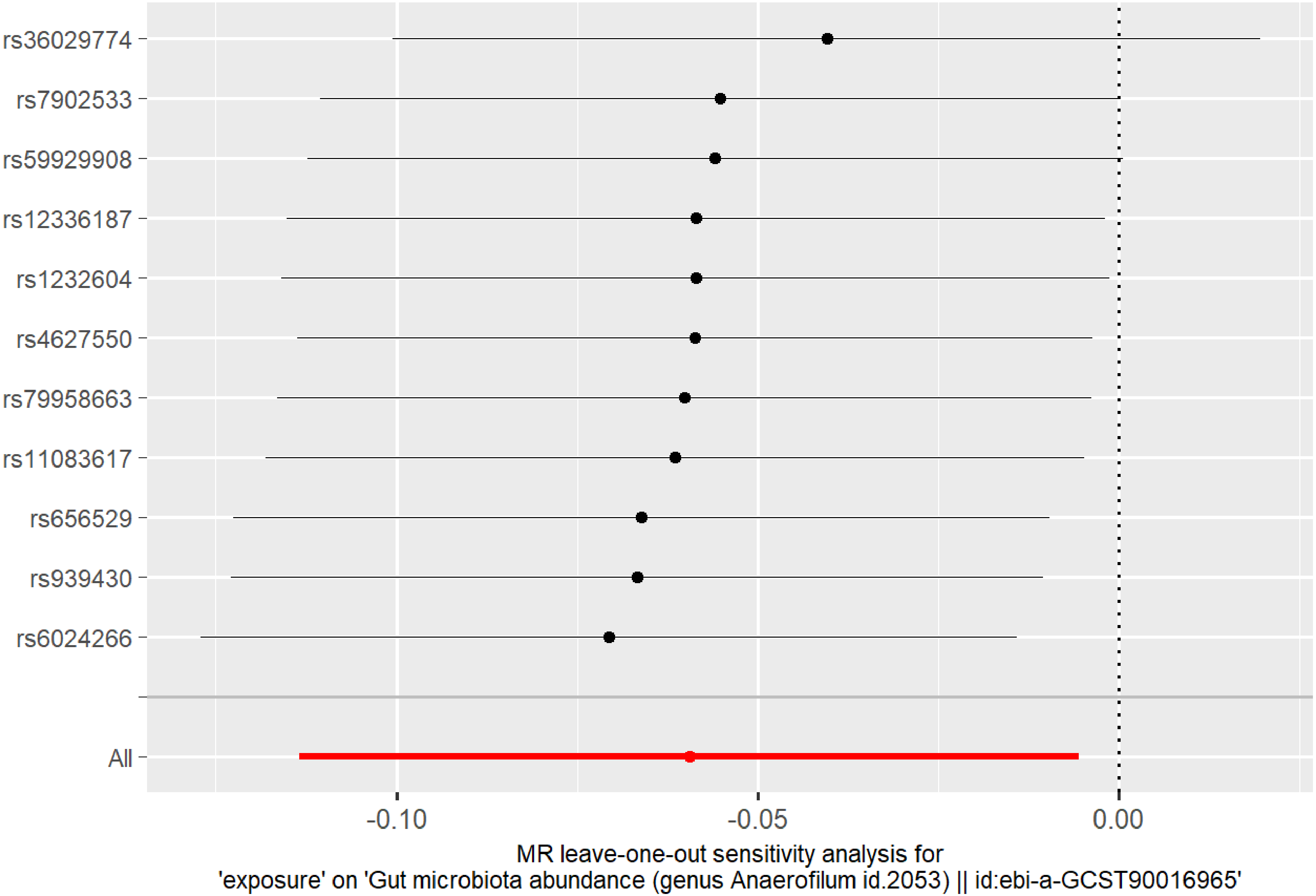
rs36029774
rs7902533
rs59929908
rs12336187
rs1232604
rs4627550
rs79958663
rs11083617
rs656529
rs939430
rs6024266
All
-0.10
-0.05
0.00
MR leave-one-out sensitivity analysis for
'exposure' on 'Gut microbiota abundance (genus Anaerofilum id.2053) || id:ebi-a-GCST90016965'

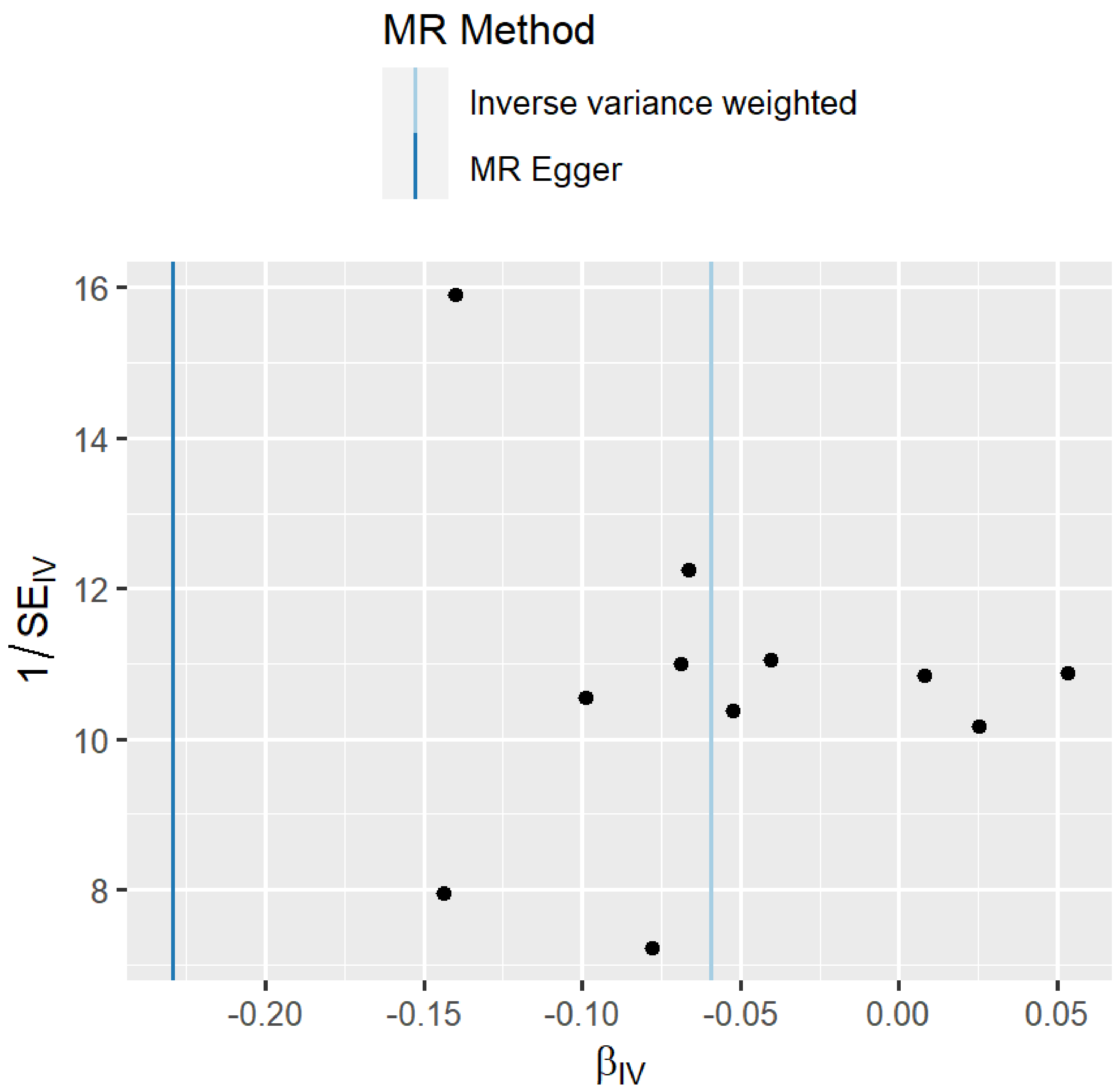

MR Method
Inverse variance weighted
MR Egger
16
14
12
10
8
-0.20
-0.15
-0.10
-0.05
0.00
0.05
β_{IV}
$1/SE_{IV}$

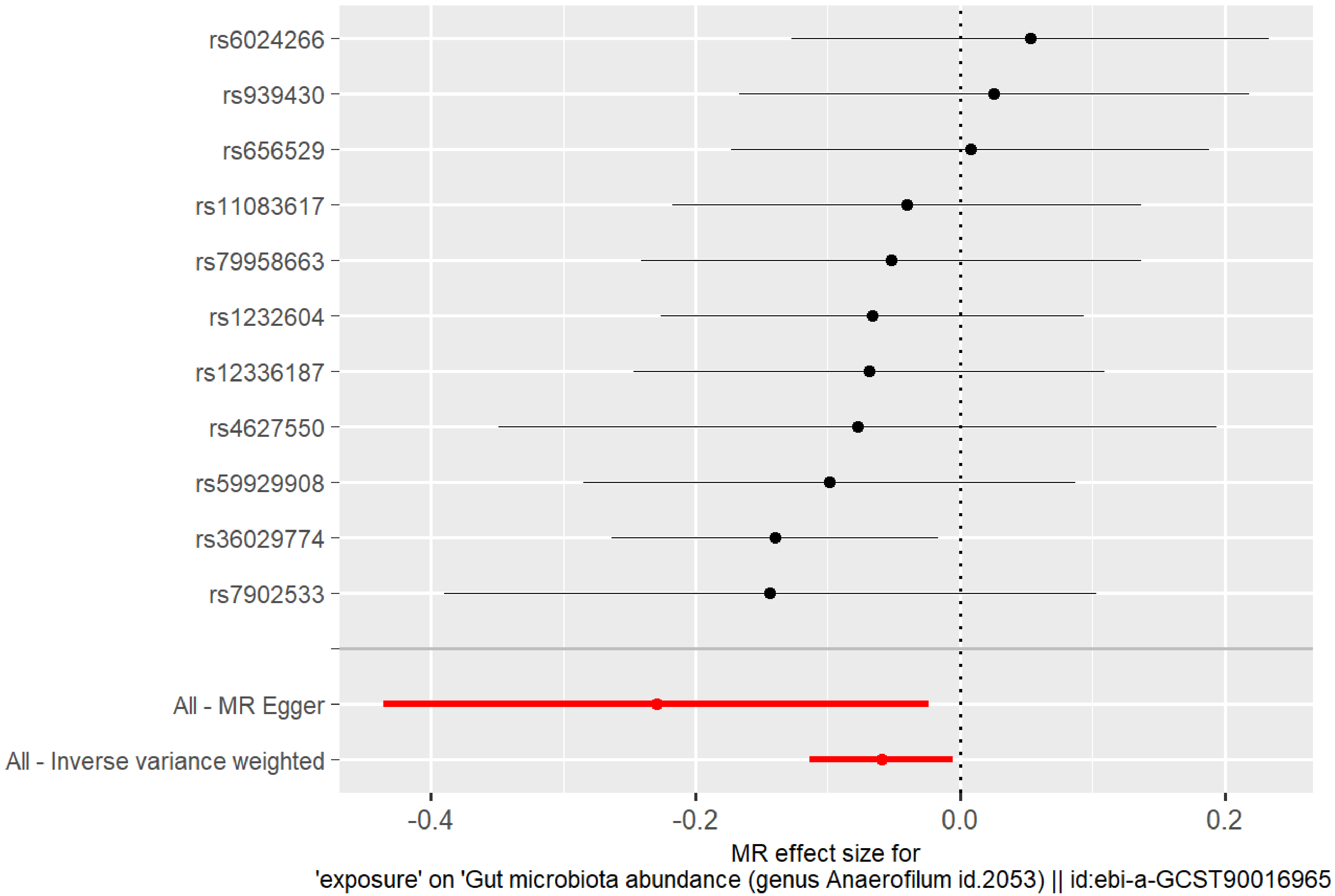
rs6024266
rs939430
rs656529
rs11083617
rs79958663
rs1232604
rs12336187
rs4627550
rs59929908
rs36029774
rs7902533
All - MR Egger
All - Inverse variance weighted
-0.4
-0.2
0.0
0.2
MR effect size for
'exposure' on 'Gut microbiota abundance (genus Anaerofilum id.2053) || id:ebi-a-GCST90016965

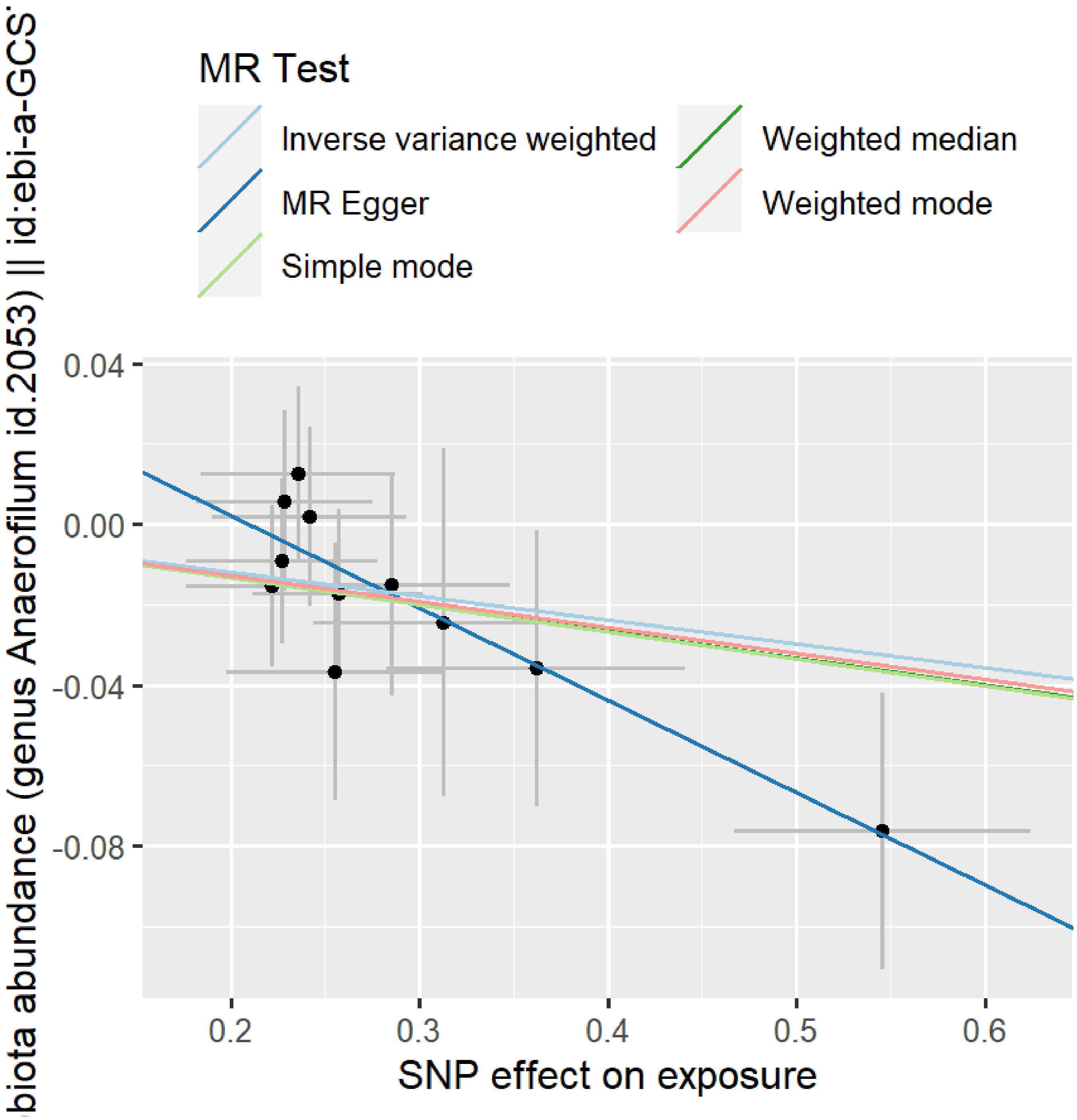
MR Test
Inverse variance weighted
MR Egger
Simple mode
Weighted median
Weighted mode
0.04
0.00
-0.04
-0.08
0.2
0.3
0.4
0.5
0.6
SNP effect on exposure
biota abundance (genus Anaerofilum id.2053) || id:ebi-a-GCS

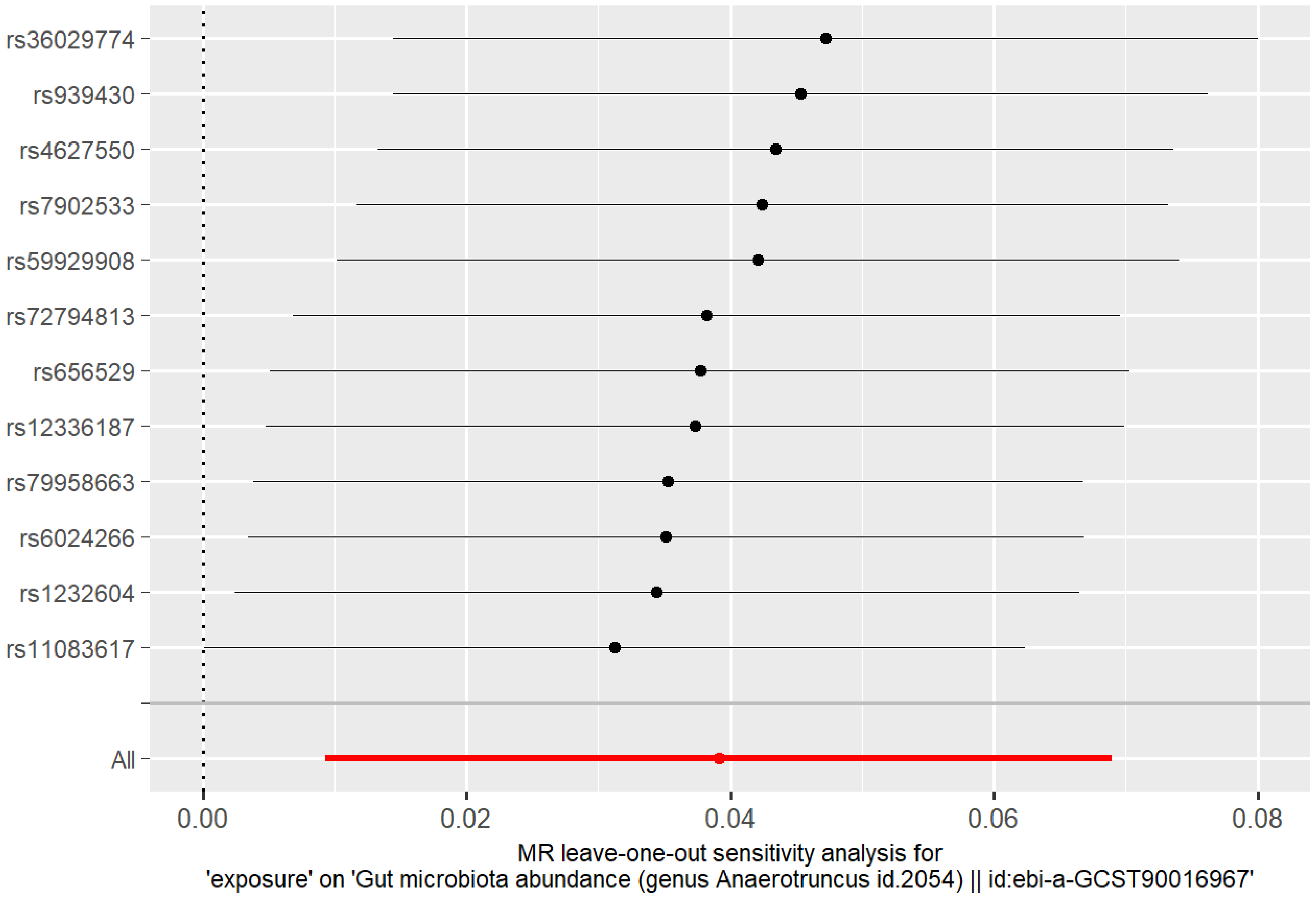
rs36029774
rs939430
rs4627550
rs7902533
rs59929908
rs72794813
rs656529
rs12336187
rs79958663
rs6024266
rs1232604
rs11083617
All
0.00
0.02
0.04
0.06
0.08
MR leave-one-out sensitivity analysis for
'exposure' on 'Gut microbiota abundance (genus Anaerotruncus id.2054) || id:ebi-a-GCST90016967'

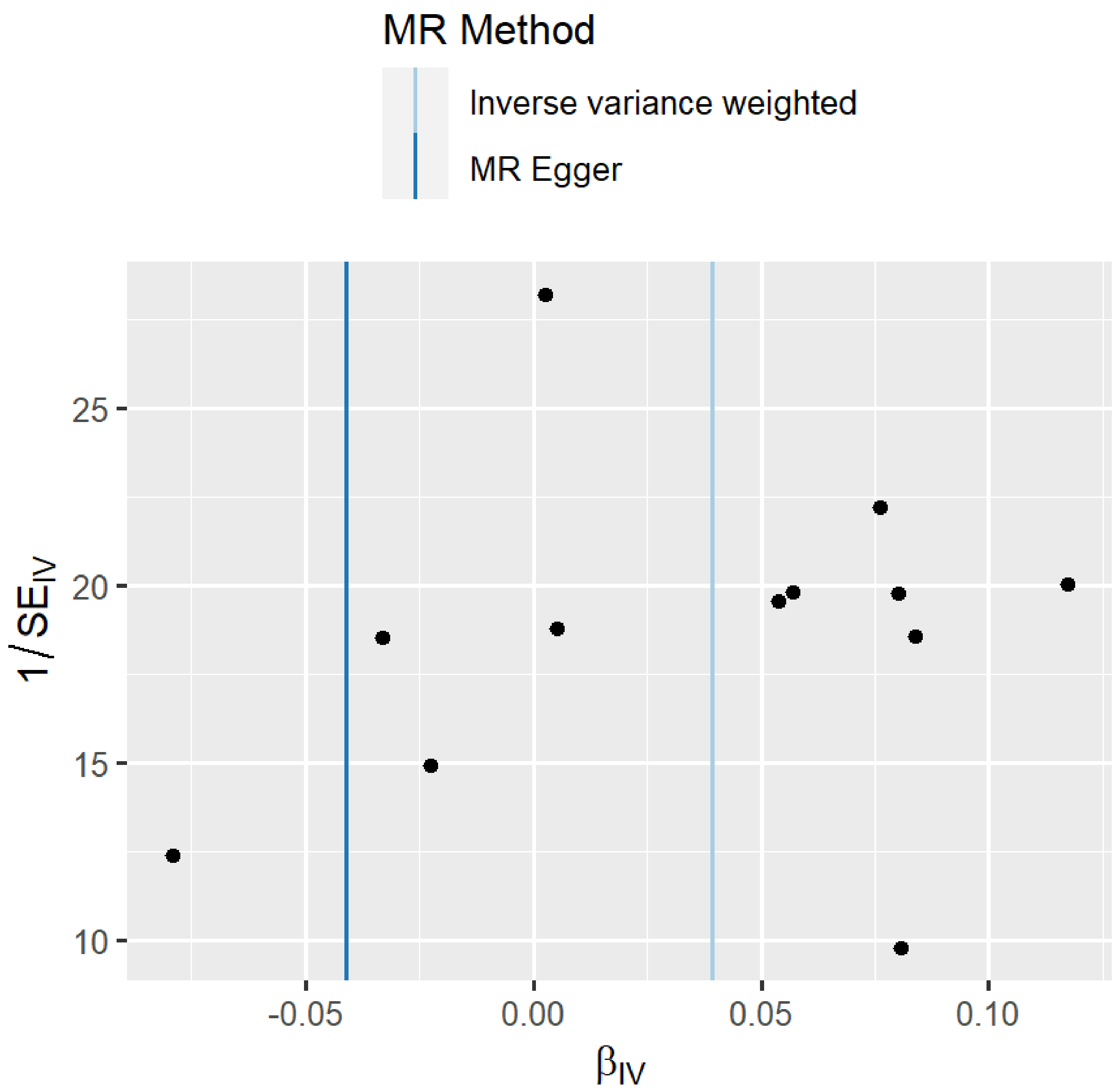
MR Method
Inverse variance weighted
MR Egger
25
20
15
10
-0.05
0.00
0.05
0.10
$1/SE_{IV}$
β_{IV}

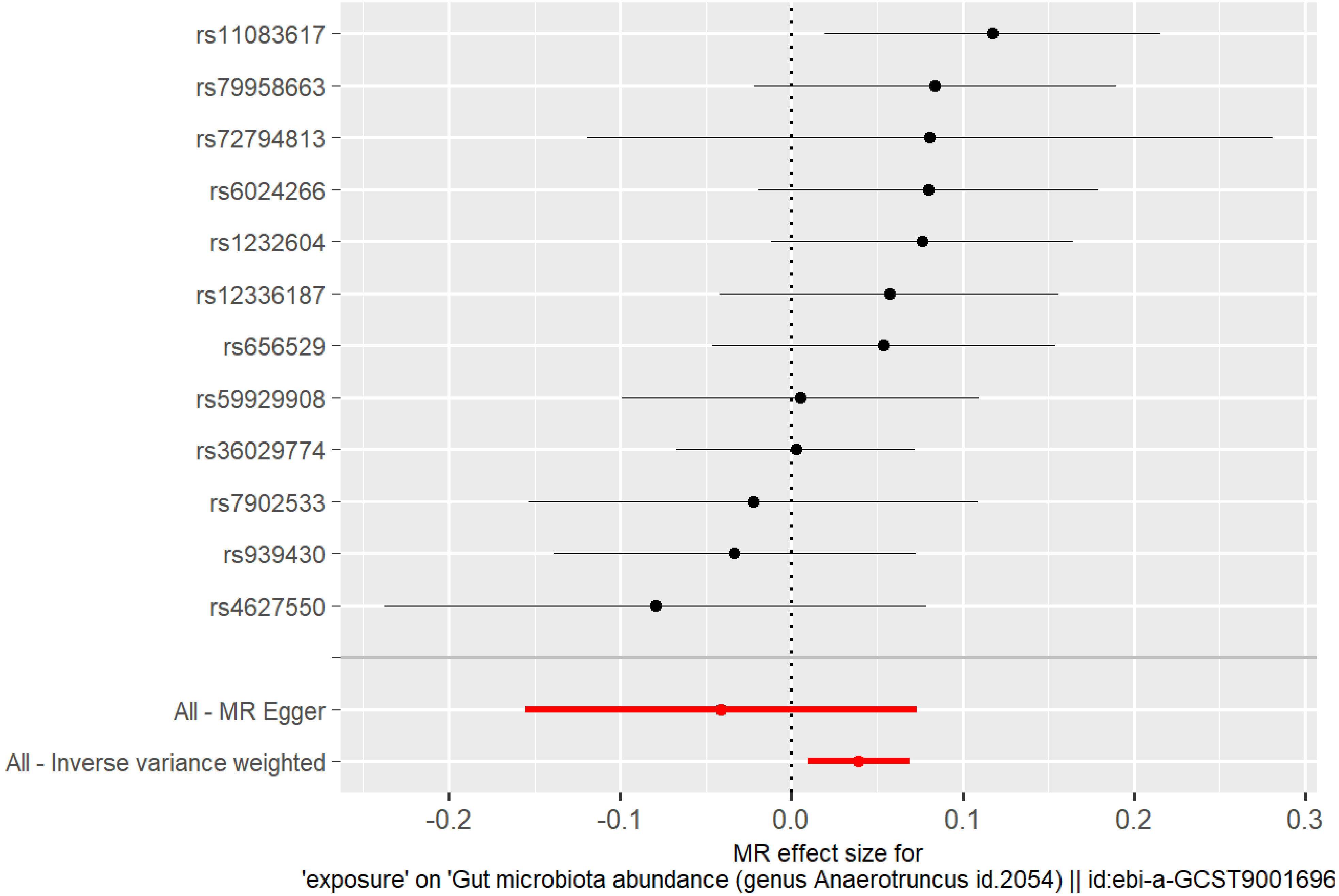
rs11083617
rs79958663
rs72794813
rs6024266
rs1232604
rs12336187
rs656529
rs59929908
rs36029774
rs7902533
rs939430
rs4627550
All - MR Egger
All - Inverse variance weighted
-0.2
-0.1
0.0
0.1
0.2
0.3
MR effect size for
'exposure' on 'Gut microbiota abundance (genus Anaerotruncus id.2054) || id:ebi-a-GCST9001696

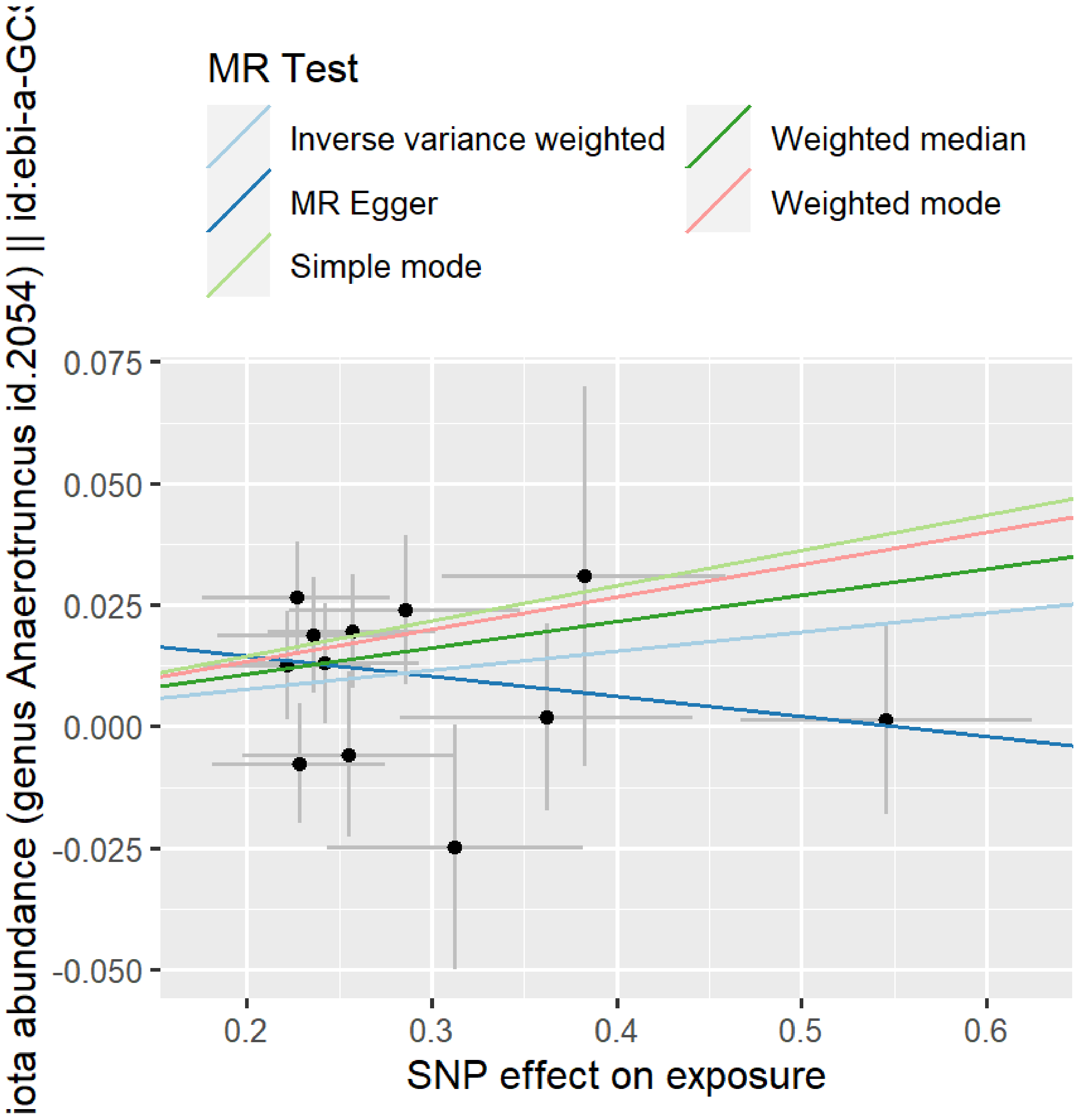
MR Test
Inverse variance weighted
MR Egger
Simple mode
Weighted median
Weighted mode
iota abundance (genus Anaerotruncus id.2054) || id:ebi-a-GC
0.075
0.050
0.025
0.000
-0.025
-0.050
0.2
0.3
0.4
0.5
0.6
SNP effect on exposure

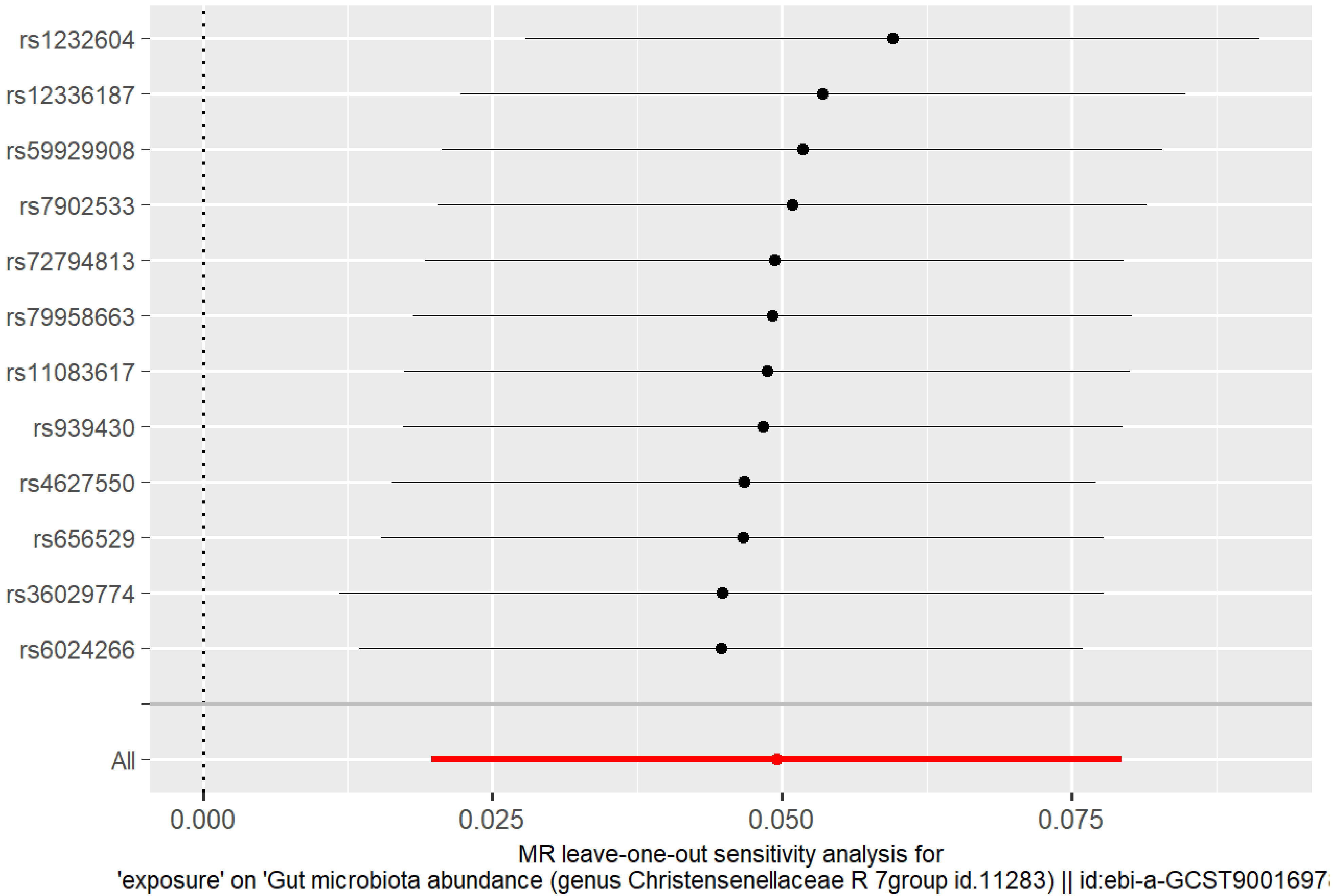
rs1232604
rs12336187
rs59929908
rs7902533
rs72794813
rs79958663
rs11083617
rs939430
rs4627550
rs656529
rs36029774
rs6024266
All
0.000
0.025
0.050
0.075
MR leave-one-out sensitivity analysis for
'exposure' on 'Gut microbiota abundance (genus Christensenellaceae R 7group id.11283) || id:ebi-a-GCST900169

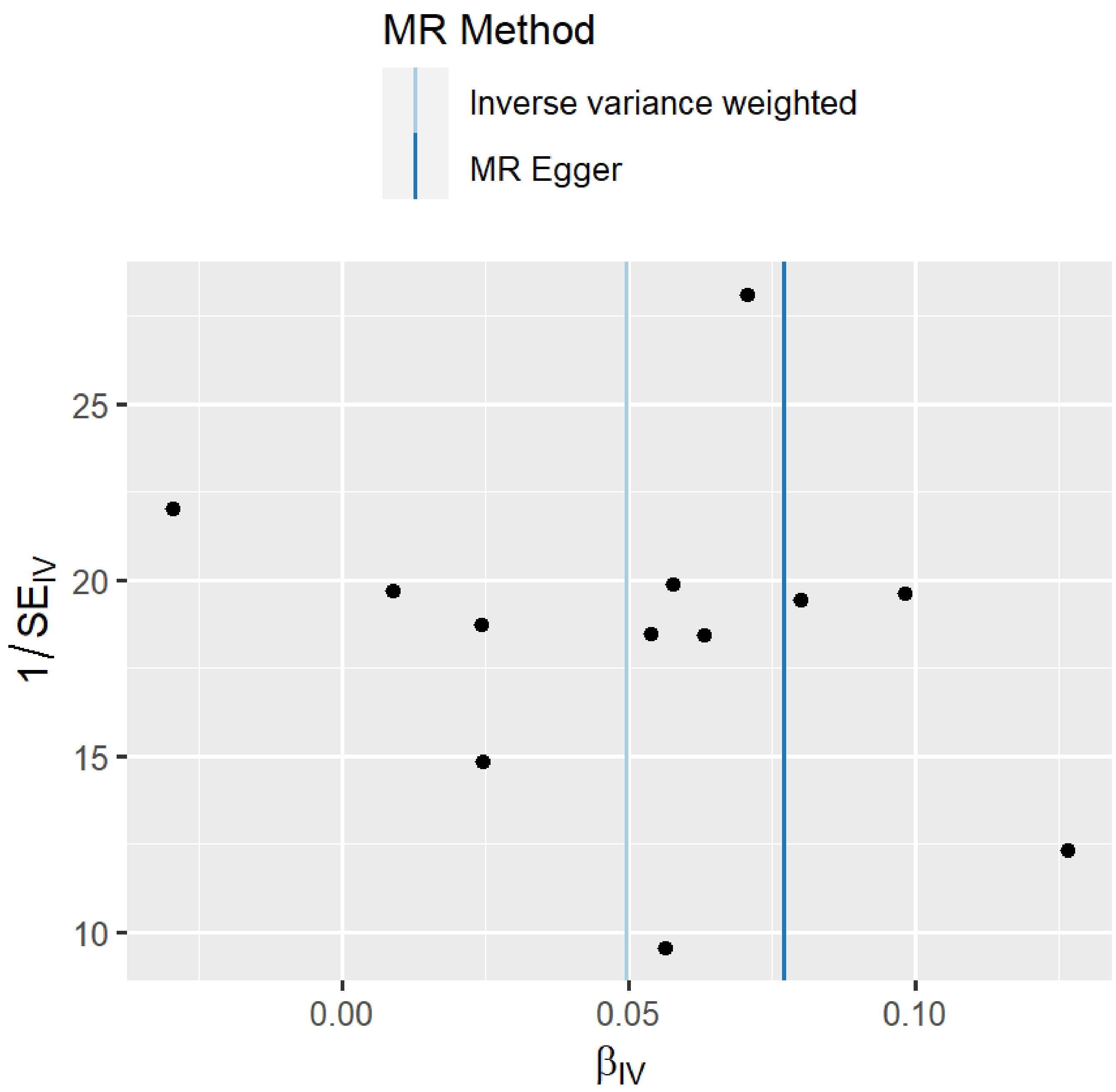
MR Method
Inverse variance weighted
MR Egger
25
20
15
10
1/SE_IV
0.00
0.05
0.10
β_IV

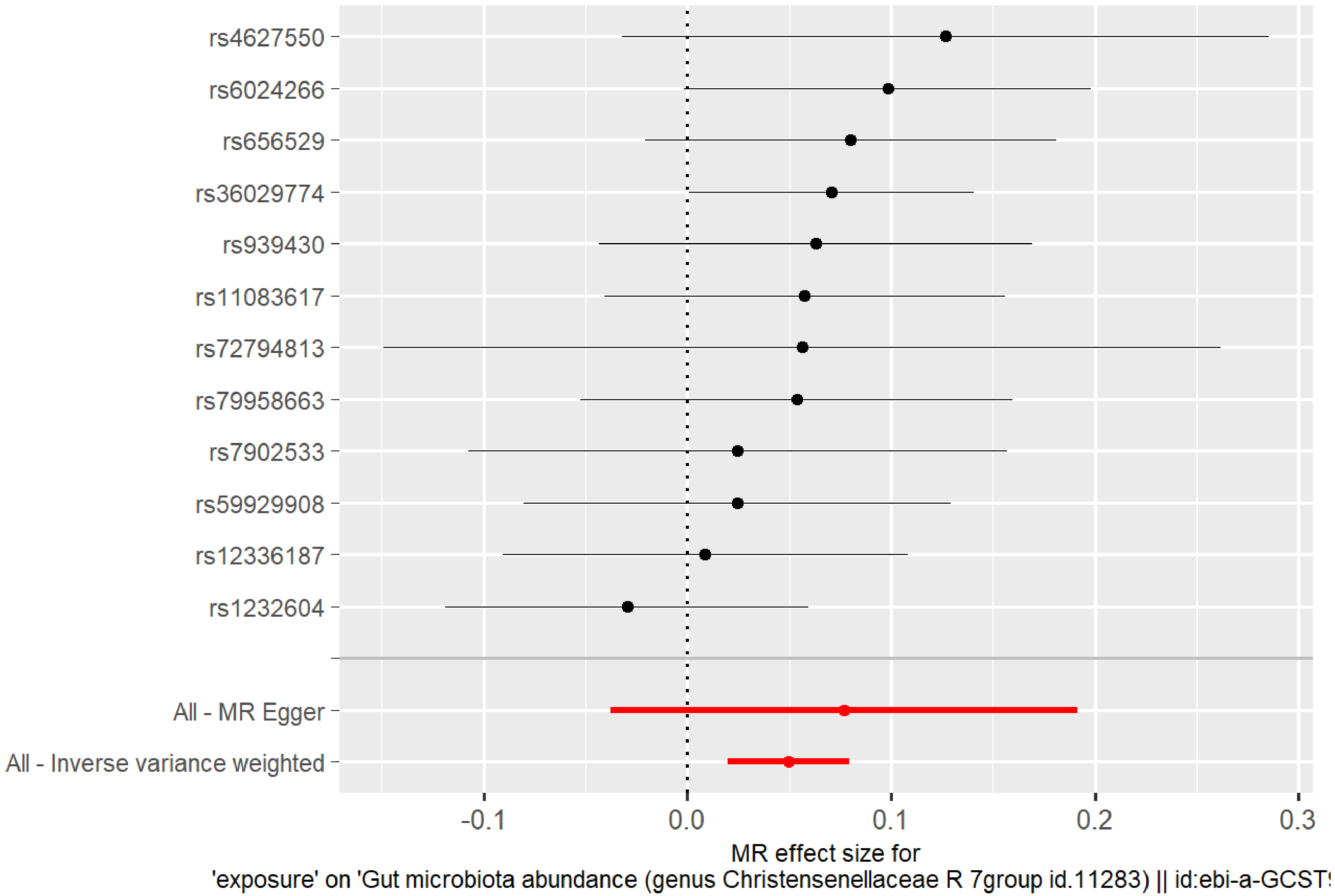
rs4627550
rs6024266
rs656529
rs36029774
rs939430
rs11083617
rs72794813
rs79958663
rs7902533
rs59929908
rs12336187
rs1232604
All - MR Egger
All - Inverse variance weighted
-0.1
0.0
0.1
0.2
0.3
MR effect size for
'exposure' on 'Gut microbiota abundance (genus Christensenellaceae R 7group id.11283) || id:ebi-a-GCST

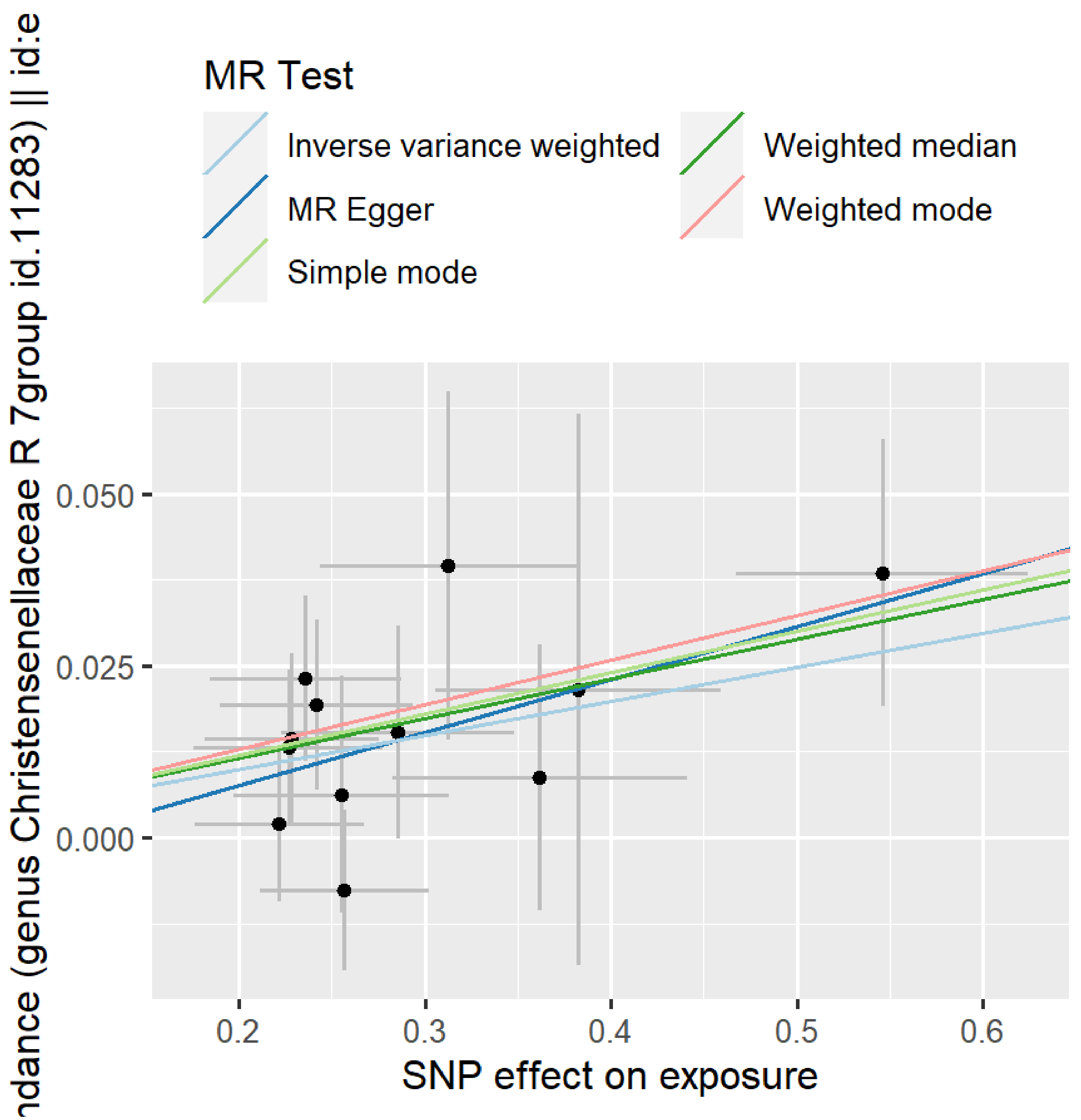
MR Test
Inverse variance weighted
MR Egger
Simple mode
Weighted median
Weighted mode
ndance (genus Christensenellaceae R 7group id.11283) || id:e
0.050
0.025
0.000
0.2
0.3
0.4
0.5
0.6
SNP effect on exposure

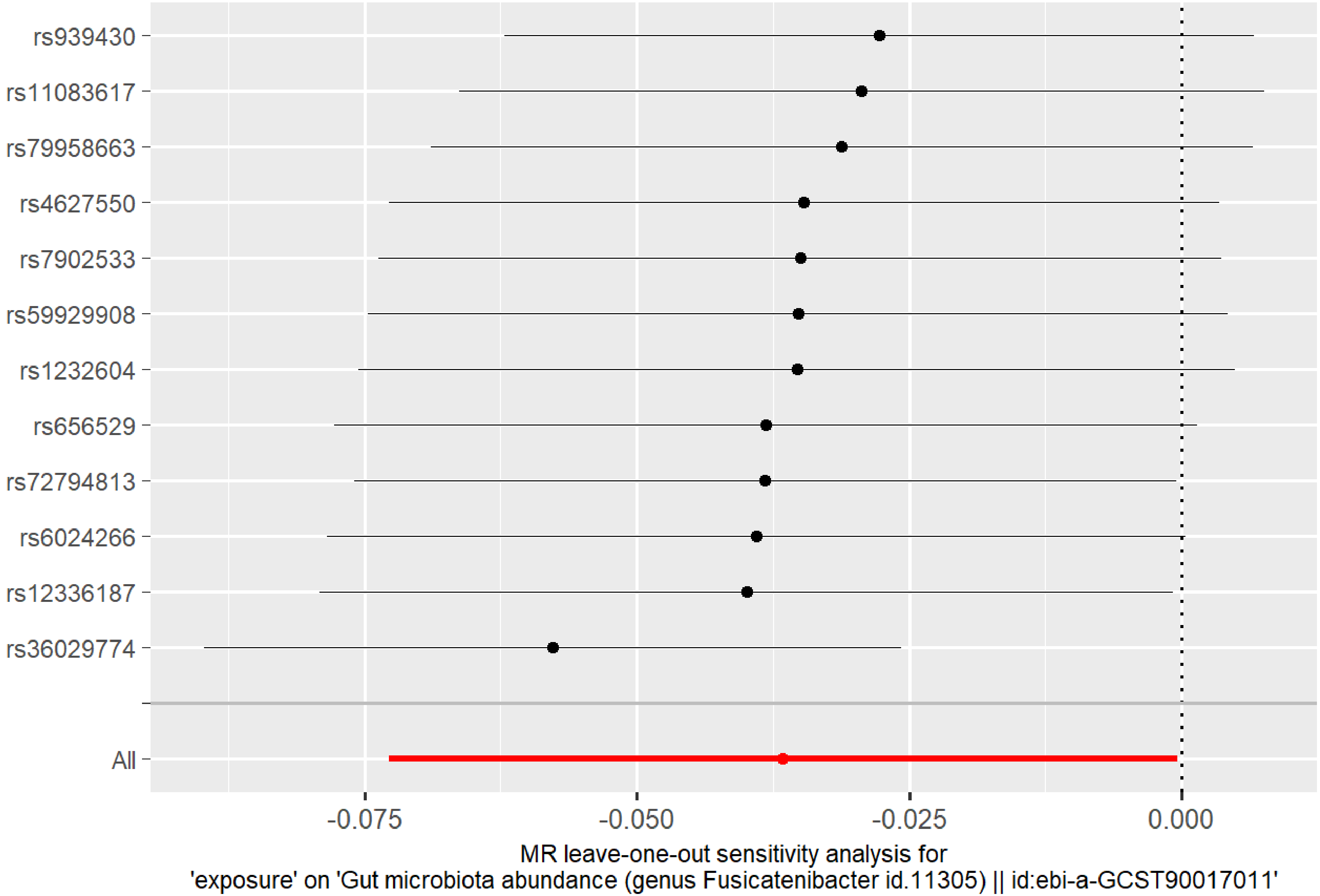
rs939430
rs11083617
rs79958663
rs4627550
rs7902533
rs59929908
rs1232604
rs656529
rs72794813
rs6024266
rs12336187
rs36029774
All
-0.075
-0.050
-0.025
0.000
MR leave-one-out sensitivity analysis for
'exposure' on 'Gut microbiota abundance (genus Fusicatenibacter id.11305) || id:ebi-a-GCST90017011'

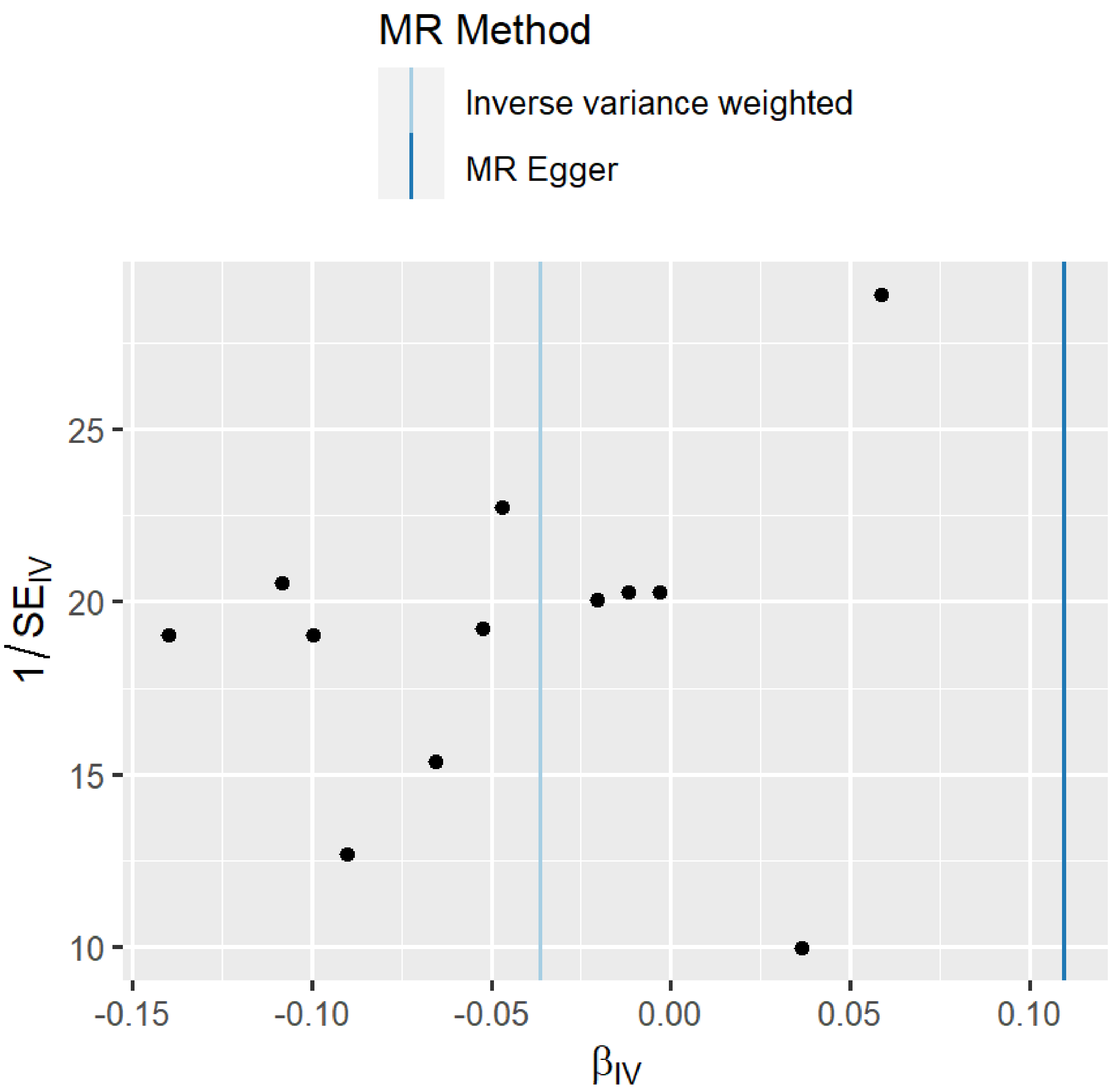

MR Method
Inverse variance weighted
MR Egger
25
20
15
10
-0.15
-0.10
-0.05
0.00
0.05
0.10
1/SE_IV
β_IV

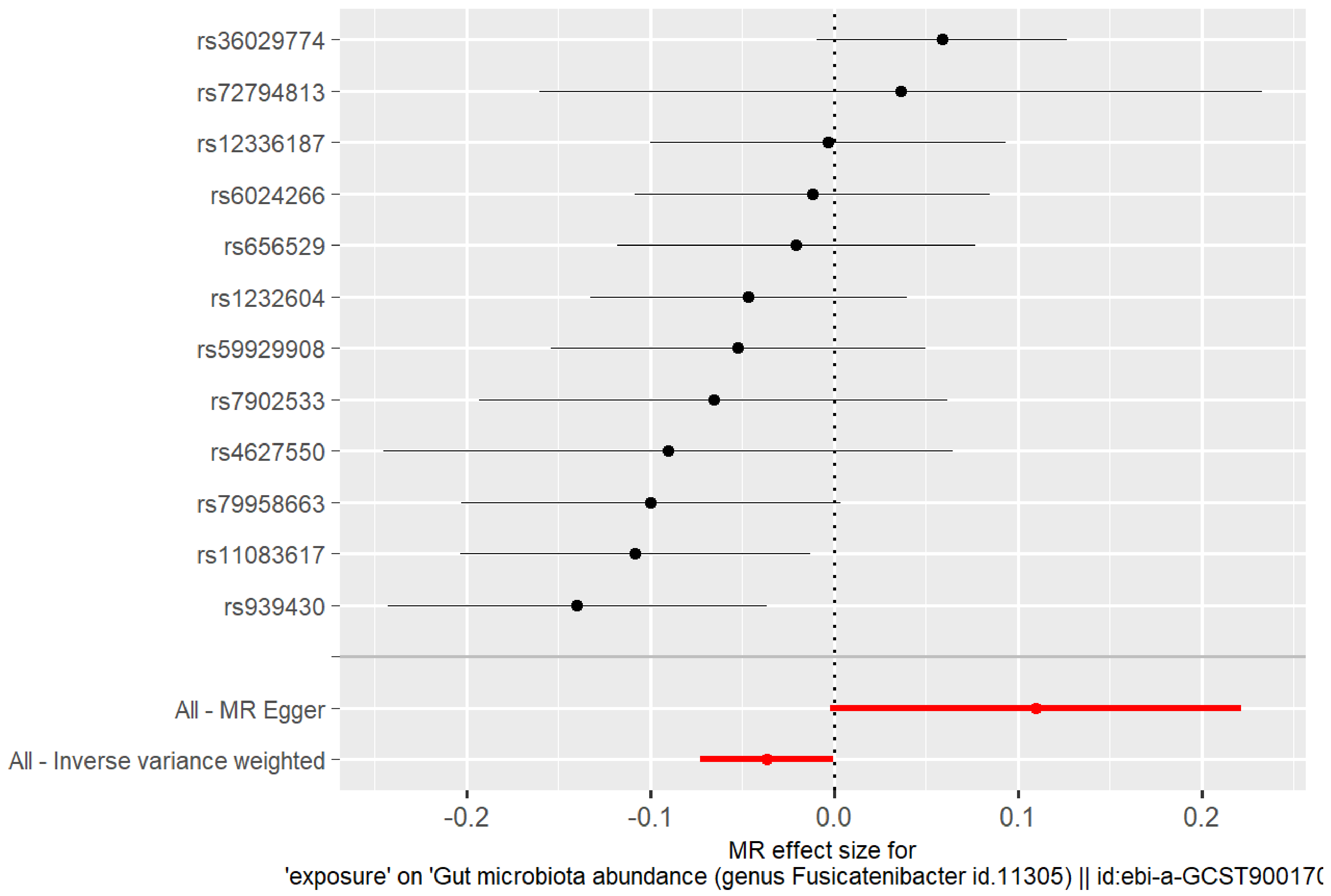
rs36029774
rs72794813
rs12336187
rs6024266
rs656529
rs1232604
rs59929908
rs7902533
rs4627550
rs79958663
rs11083617
rs939430
All - MR Egger
All - Inverse variance weighted
-0.2
-0.1
0.0
0.1
0.2
MR effect size for
'exposure' on 'Gut microbiota abundance (genus Fusicatenibacter id.11305) || id:ebi-a-GCST90017

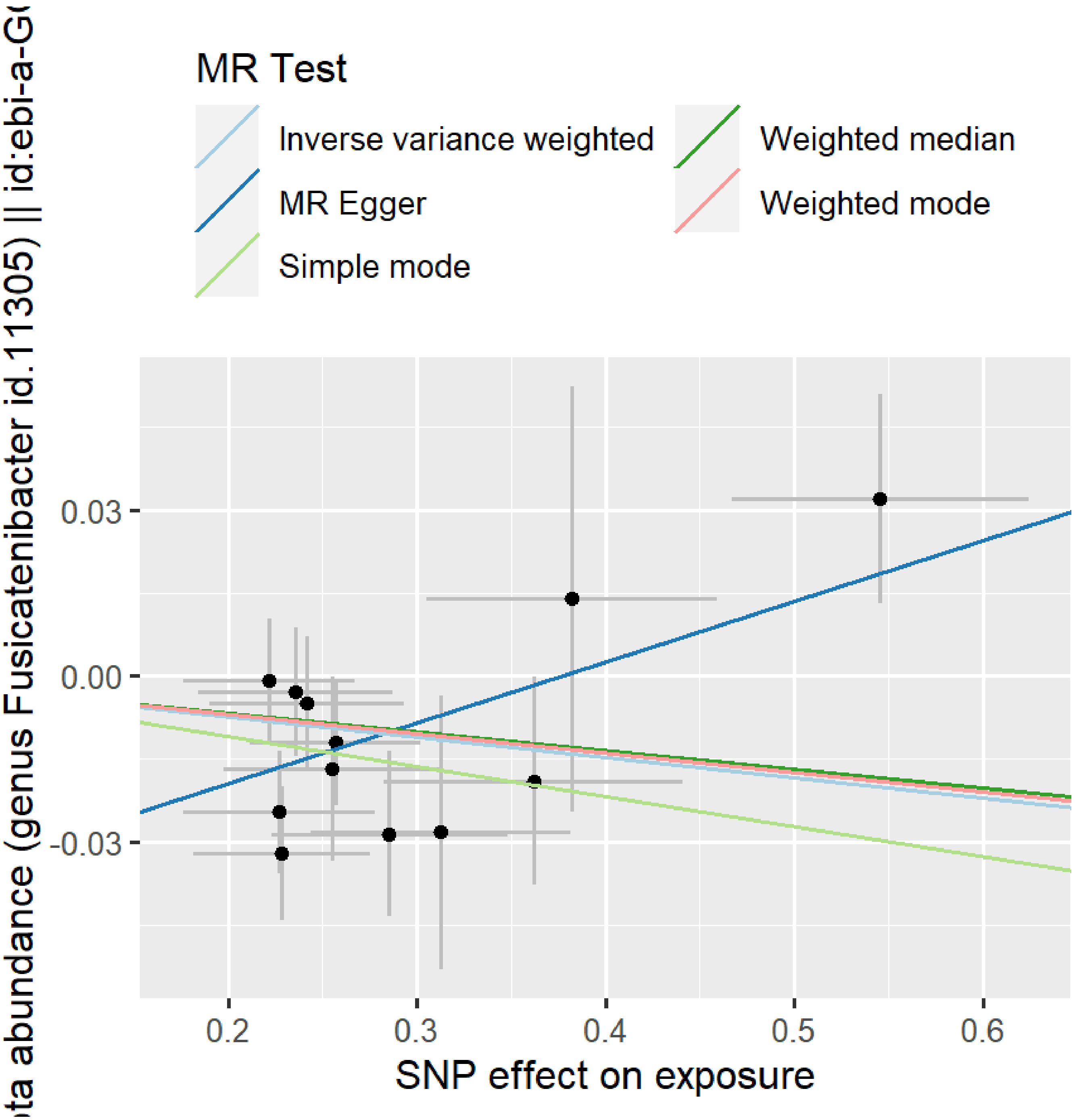

MR Test
Inverse variance weighted
MR Egger
Simple mode
Weighted median
Weighted mode
0.03
0.00
-0.03
0.2
0.3
0.4
0.5
0.6
SNP effect on exposure
ta abundance (genus Fusicatenibacter id.11305) || id:ebi-a-G

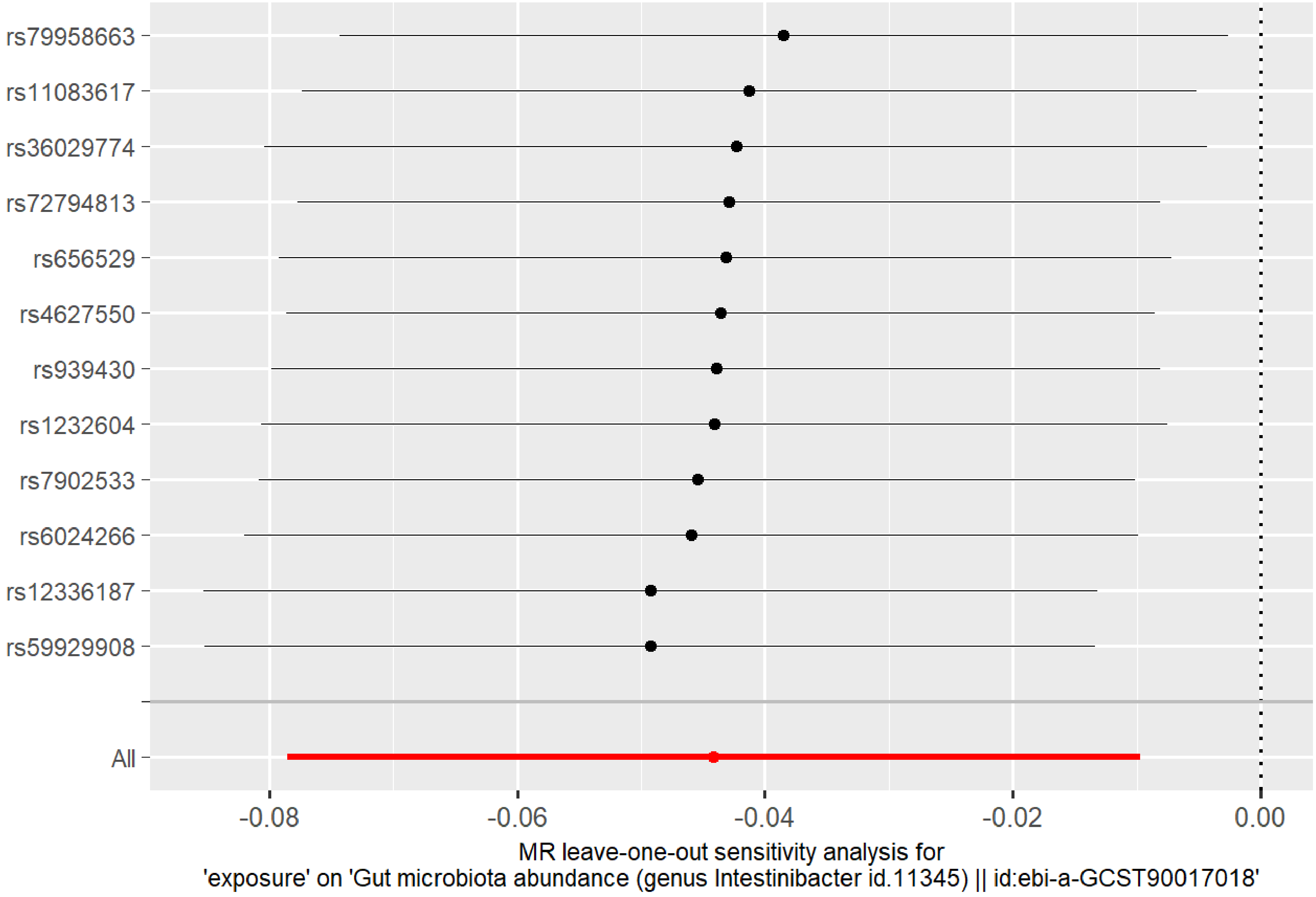
rs79958663
rs11083617
rs36029774
rs72794813
rs656529
rs4627550
rs939430
rs1232604
rs7902533
rs6024266
rs12336187
rs59929908
All
-0.08
-0.06
-0.04
-0.02
0.00
MR leave-one-out sensitivity analysis for
'exposure' on 'Gut microbiota abundance (genus Intestinibacter id.11345) || id:ebi-a-GCST90017018'

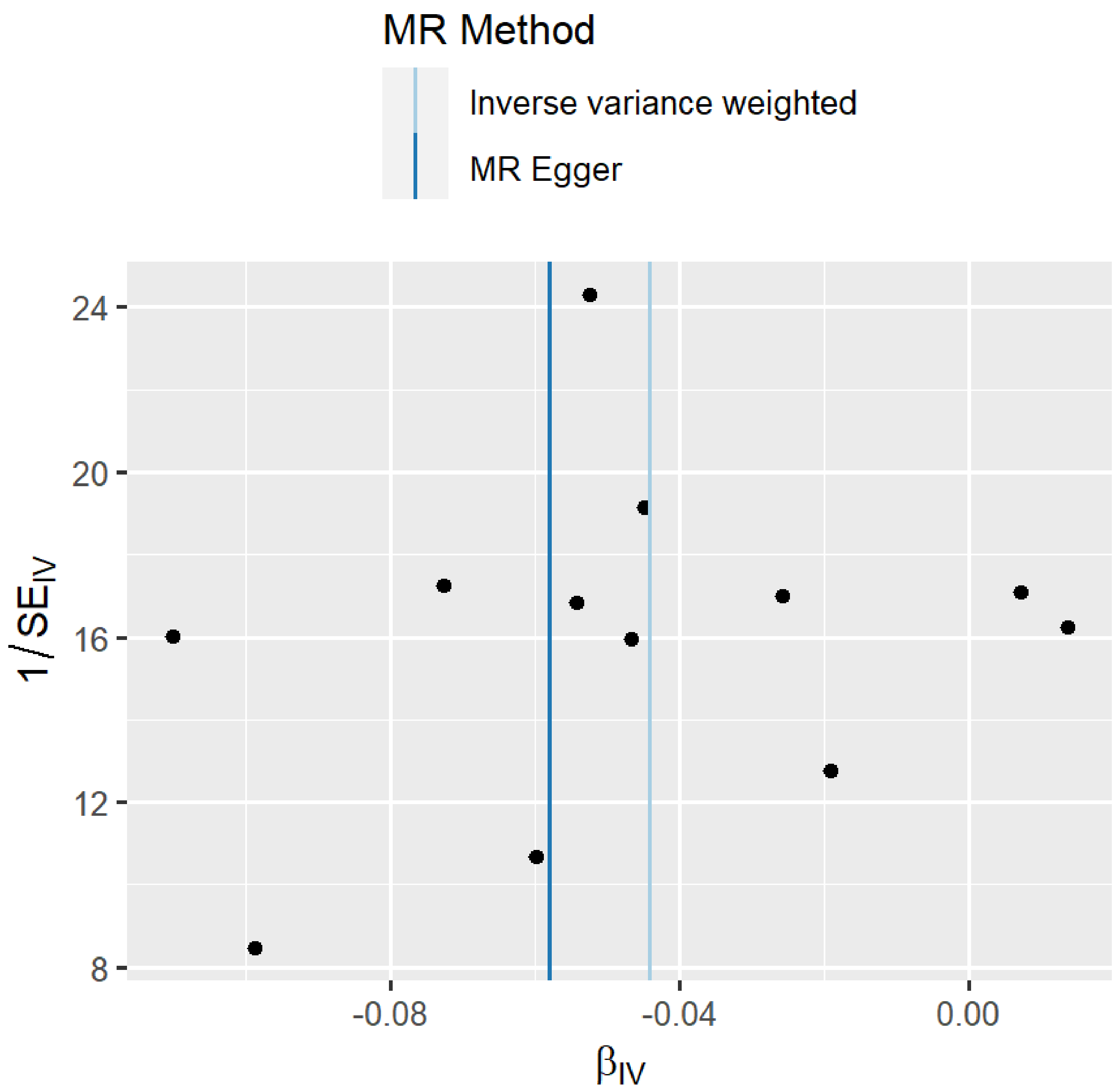
MR Method
Inverse variance weighted
MR Egger
24
20
16
12
8
1/SE_IV
-0.08
-0.04
0.00
β_IV

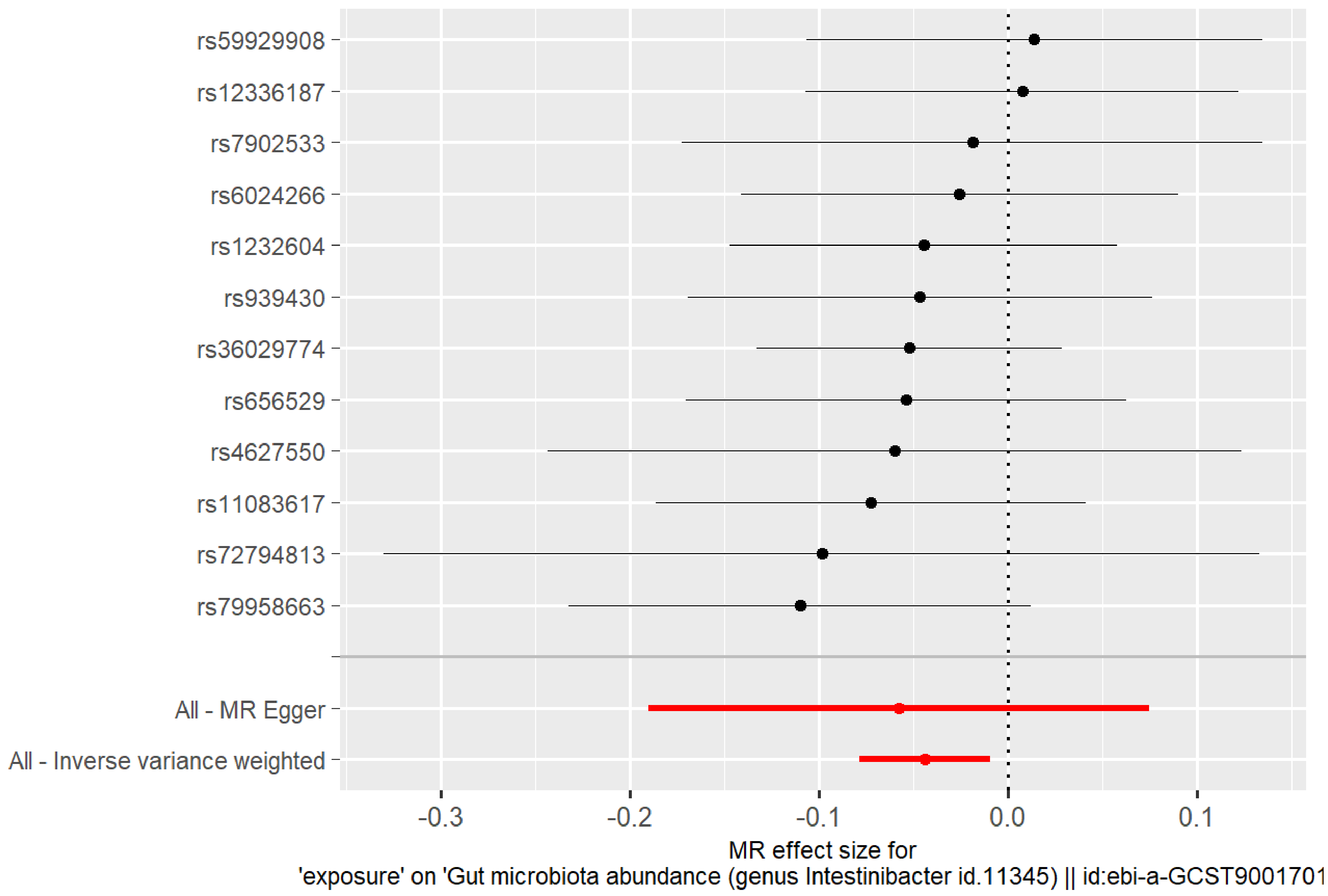
rs59929908
rs12336187
rs7902533
rs6024266
rs1232604
rs939430
rs36029774
rs656529
rs4627550
rs11083617
rs72794813
rs79958663
All - MR Egger
All - Inverse variance weighted
-0.3
-0.2
-0.1
0.0
0.1
MR effect size for
'exposure' on 'Gut microbiota abundance (genus Intestinibacter id.11345) || id:ebi-a-GCST9001701

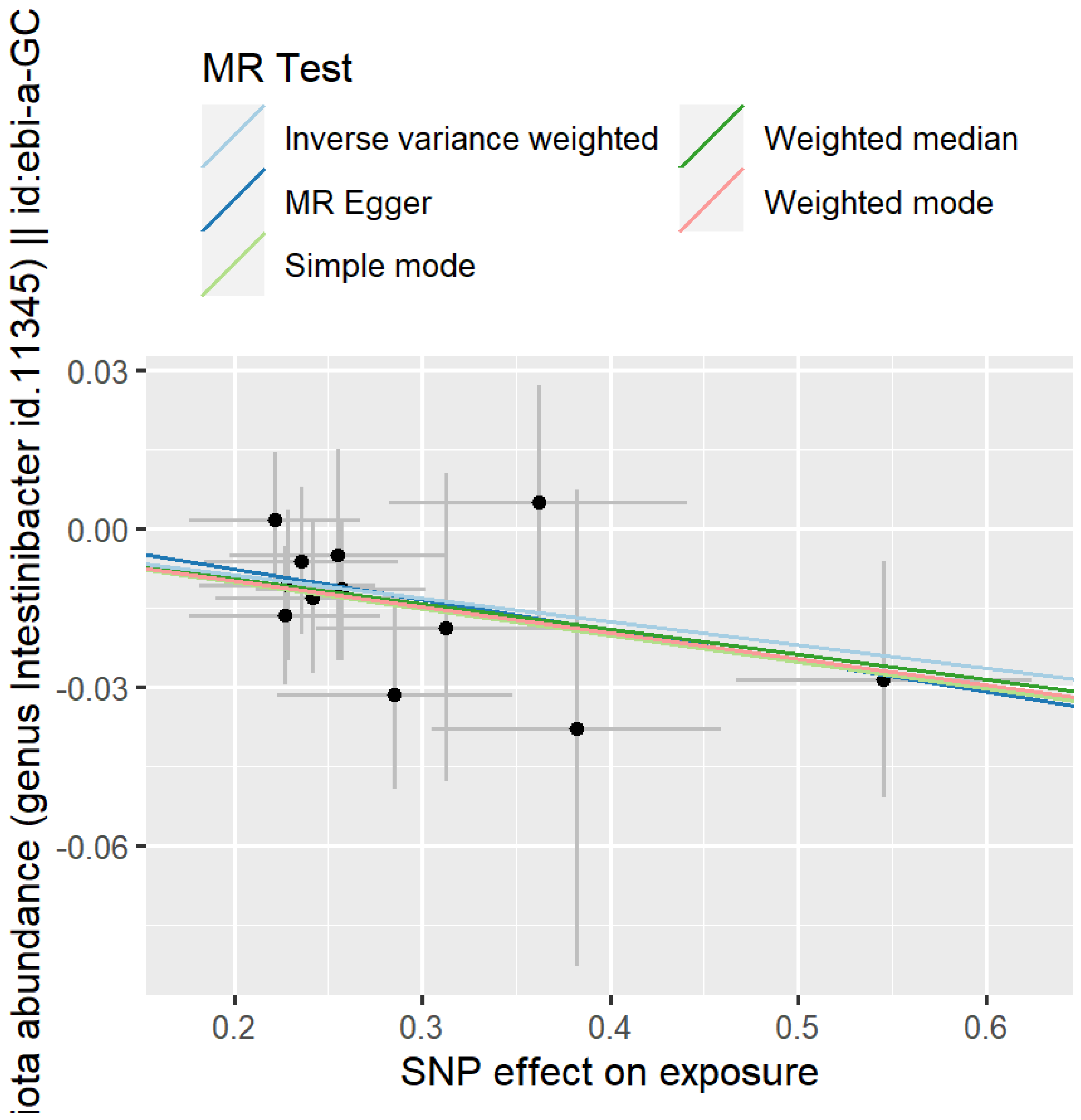
MR Test
Inverse variance weighted
MR Egger
Simple mode
Weighted median
Weighted mode
iota abundance (genus Intestinibacter id.11345) || id:ebi-a-GC
0.03
0.00
-0.03
-0.06
0.2
0.3
0.4
0.5
0.6
SNP effect on exposure

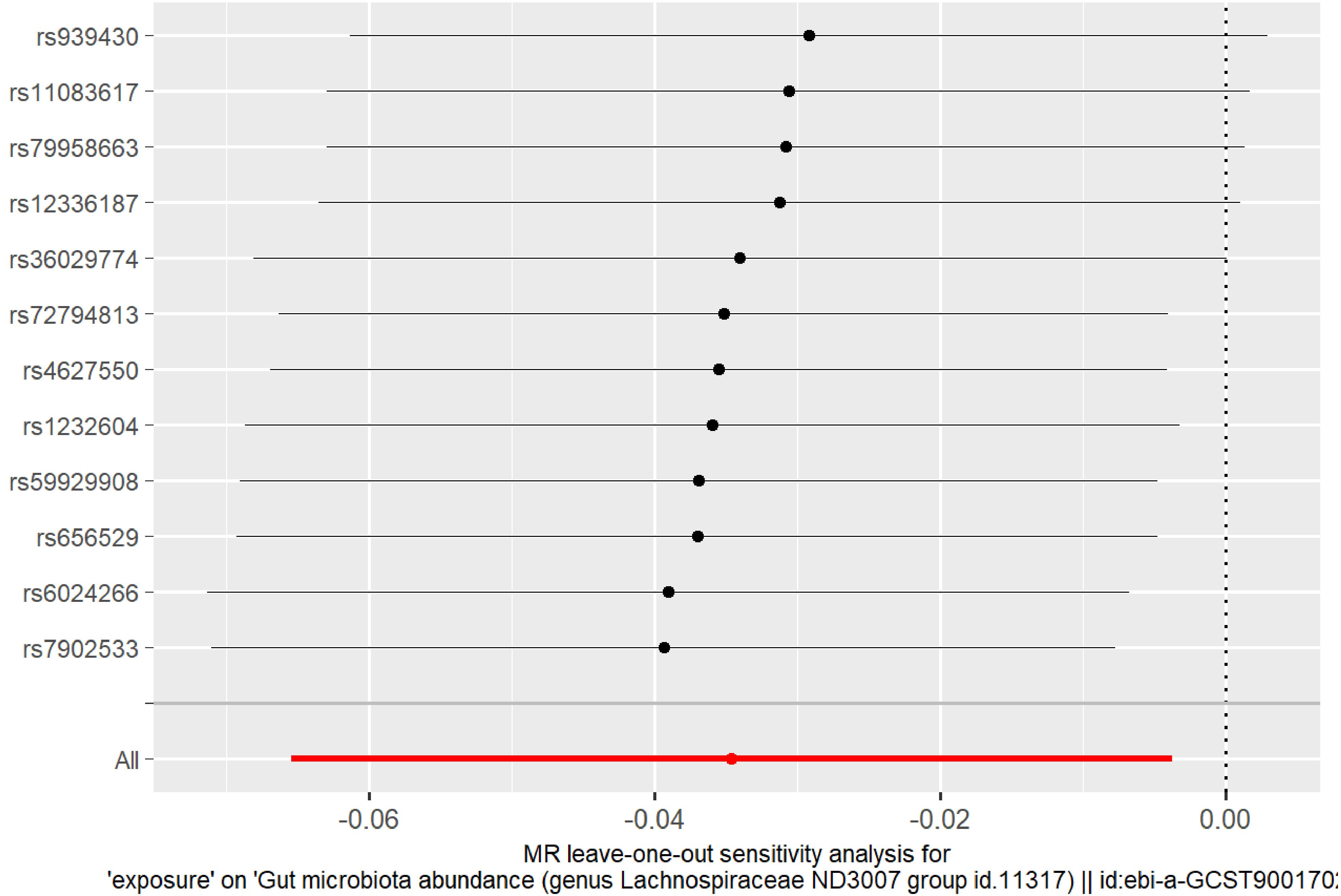
rs939430
rs11083617
rs79958663
rs12336187
rs36029774
rs72794813
rs4627550
rs1232604
rs59929908
rs656529
rs6024266
rs7902533
All
-0.06
-0.04
-0.02
0.00
MR leave-one-out sensitivity analysis for
'exposure' on 'Gut microbiota abundance (genus Lachnospiraceae ND3007 group id.11317) || id:ebi-a-GCST900170

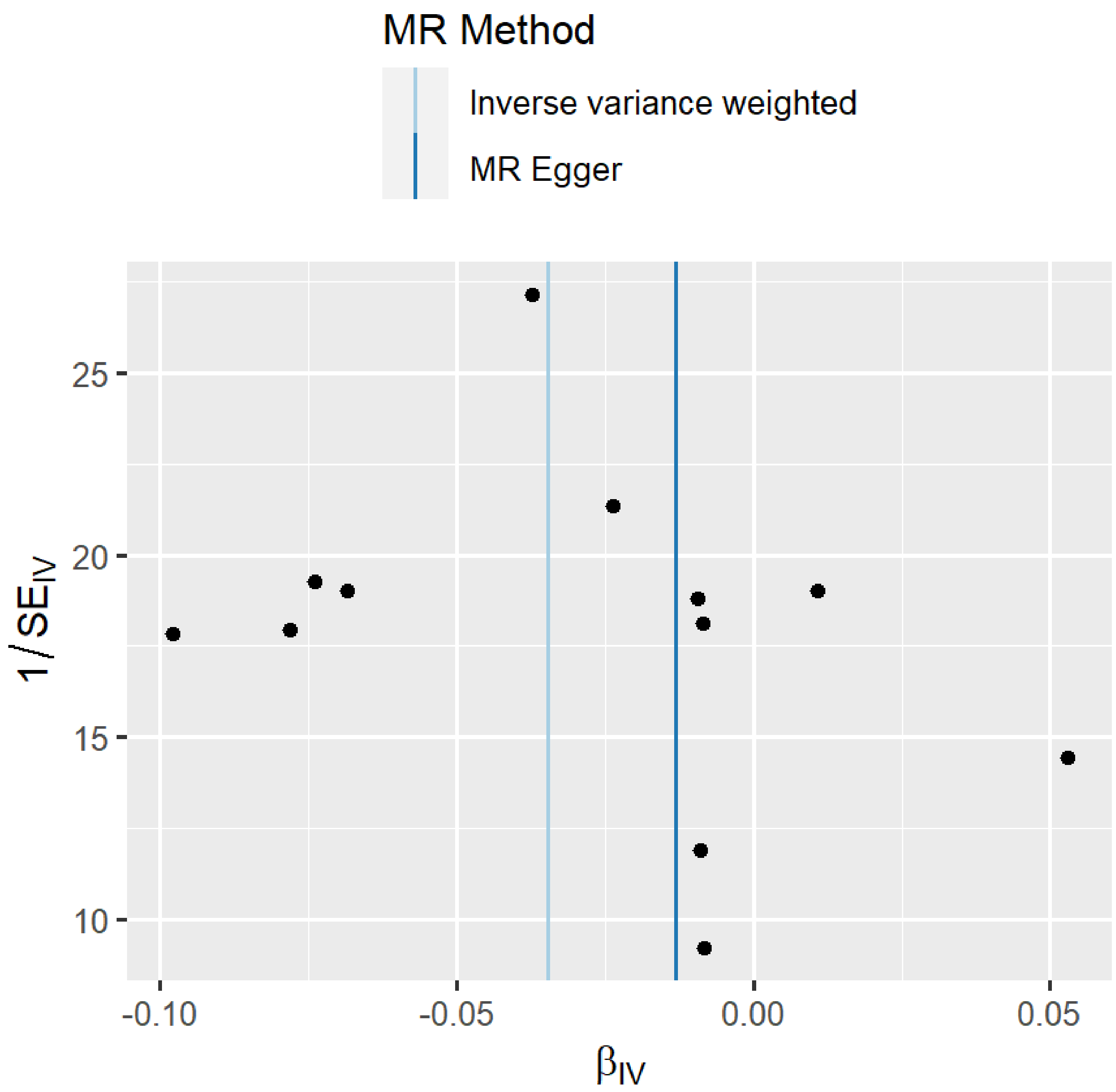
MR Method
Inverse variance weighted
MR Egger
25
20
15
10
$1/SE_{IV}$
-0.10
-0.05
0.00
0.05
β_{IV}

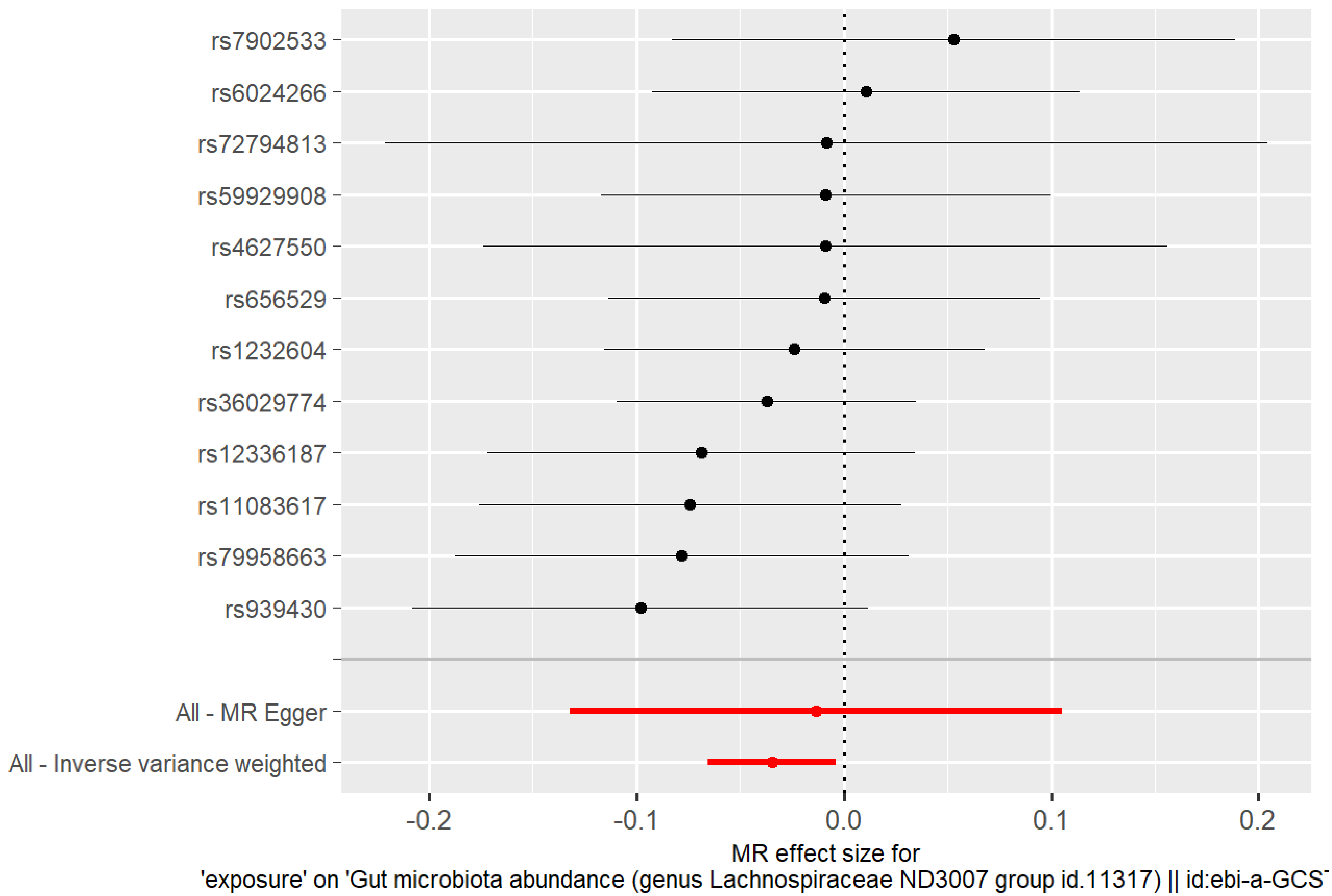
rs7902533
rs6024266
rs72794813
rs59929908
rs4627550
rs656529
rs1232604
rs36029774
rs12336187
rs11083617
rs79958663
rs939430
All - MR Egger
All - Inverse variance weighted
-0.2
-0.1
0.0
0.1
0.2
MR effect size for
'exposure' on 'Gut microbiota abundance (genus Lachnospiraceae ND3007 group id.11317) || id:ebi-a-GCS

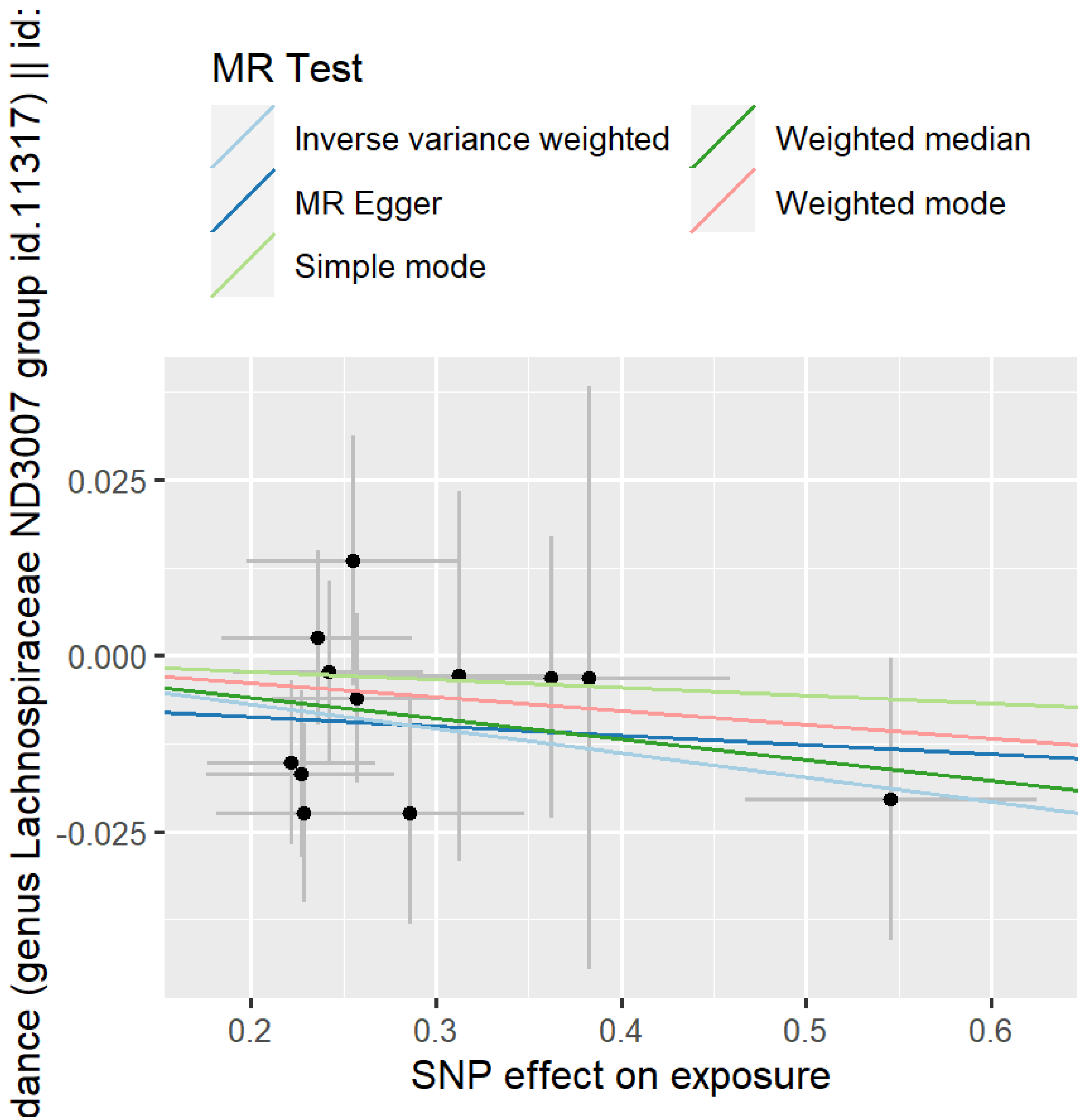
MR Test
Inverse variance weighted
MR Egger
Simple mode
Weighted median
Weighted mode
dance (genus Lachnospiraceae ND3007 group id.11317) || id:
0.025
0.000
-0.025
0.2
0.3
0.4
0.5
0.6
SNP effect on exposure

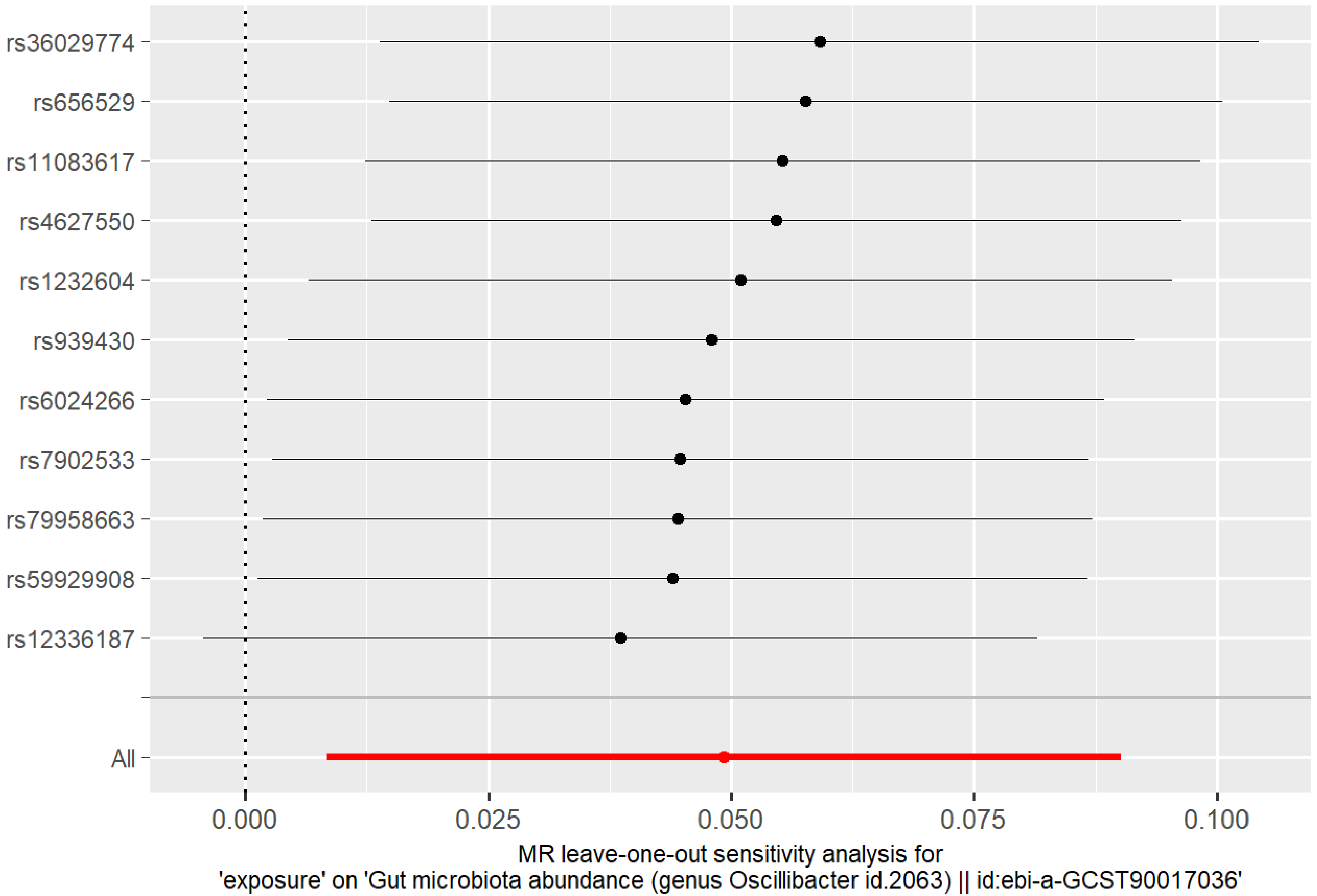
rs36029774
rs656529
rs11083617
rs4627550
rs1232604
rs939430
rs6024266
rs7902533
rs79958663
rs59929908
rs12336187
All
0.000
0.025
0.050
0.075
0.100
MR leave-one-out sensitivity analysis for
'exposure' on 'Gut microbiota abundance (genus Oscillibacter id.2063) || id:ebi-a-GCST90017036'

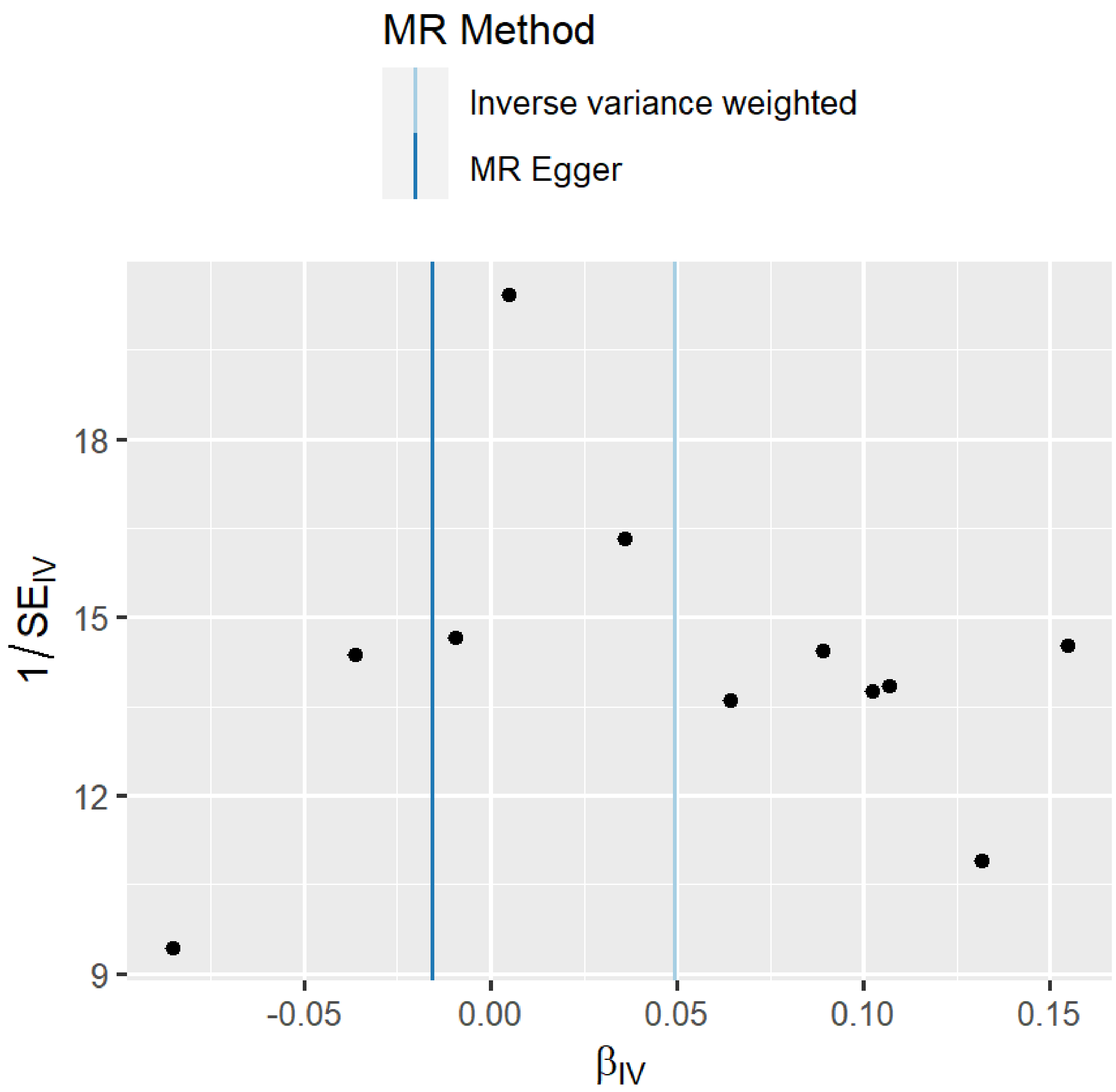
MR Method
Inverse variance weighted
MR Egger
18
15
12
9
1/SE$_{IV}$
-0.05
0.00
0.05
0.10
0.15
β$_{IV}$

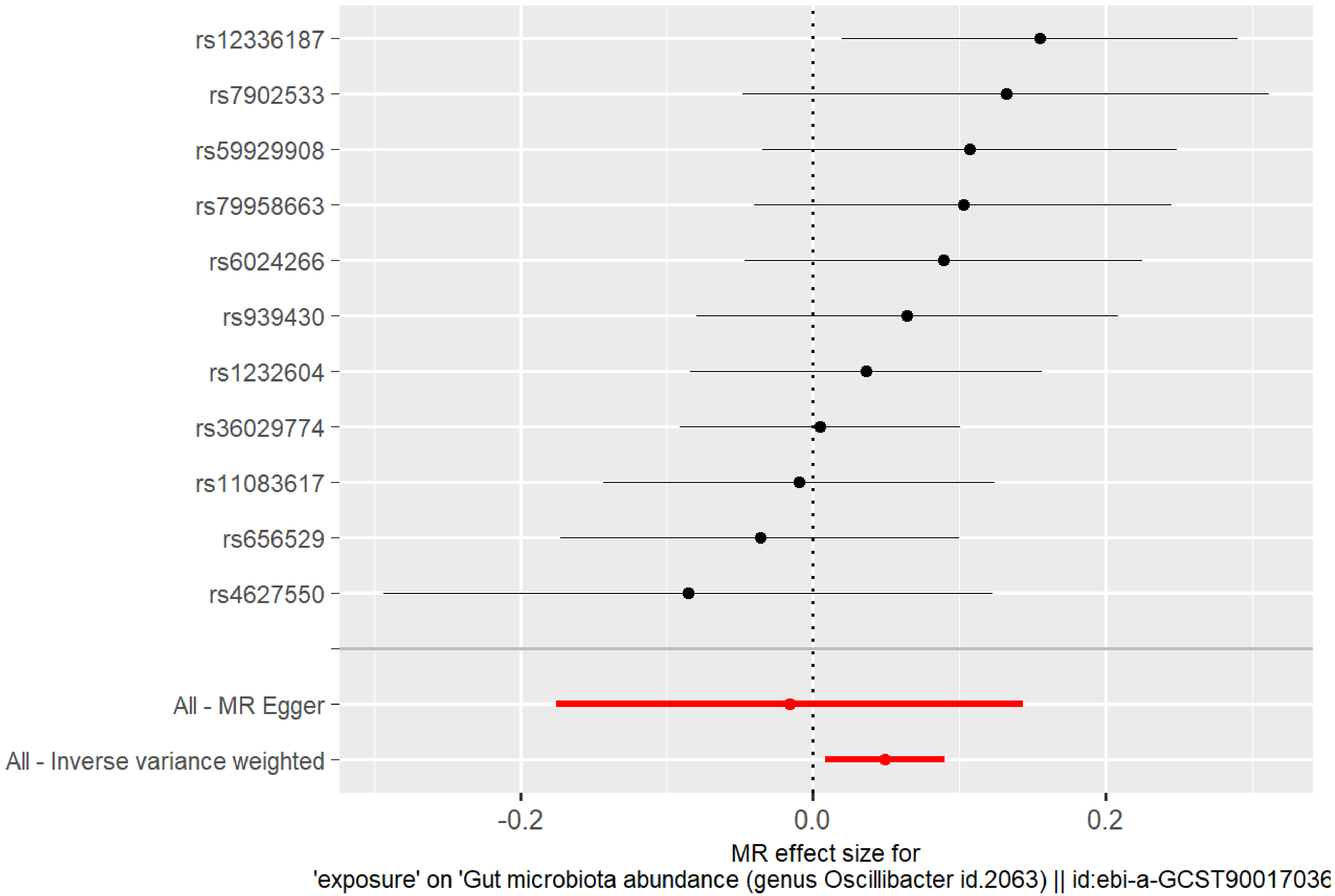
rs12336187
rs7902533
rs59929908
rs79958663
rs6024266
rs939430
rs1232604
rs36029774
rs11083617
rs656529
rs4627550
All - MR Egger
All - Inverse variance weighted
-0.2
0.0
0.2
MR effect size for
'exposure' on 'Gut microbiota abundance (genus Oscillibacter id.2063) || id:ebi-a-GCST90017036

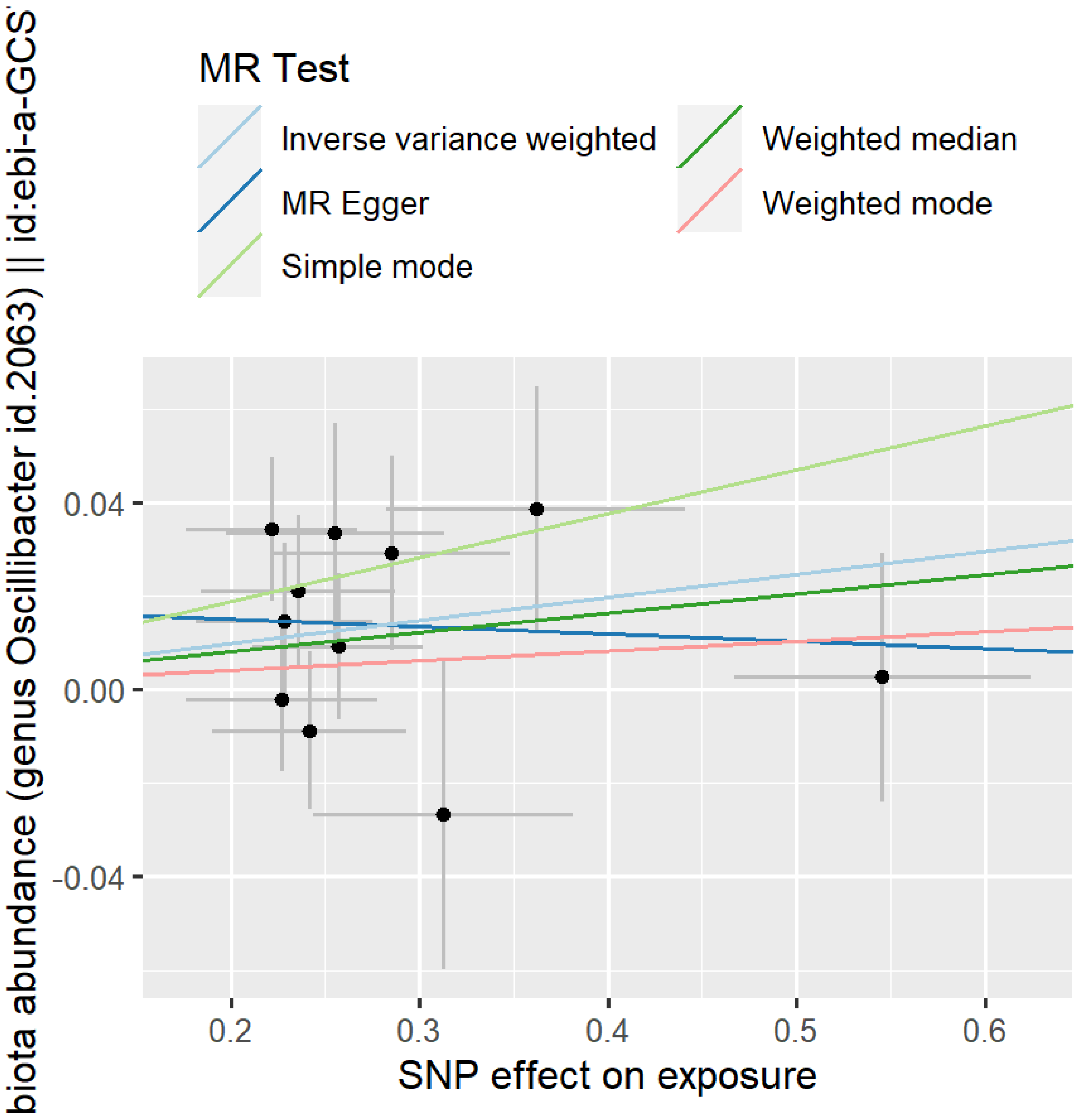

MR Test
Inverse variance weighted
MR Egger
Simple mode
Weighted median
Weighted mode
biota abundance (genus Oscillibacter id.2063) || id:ebi-a-GCS
0.04
0.00
-0.04
0.2
0.3
0.4
0.5
0.6
SNP effect on exposure

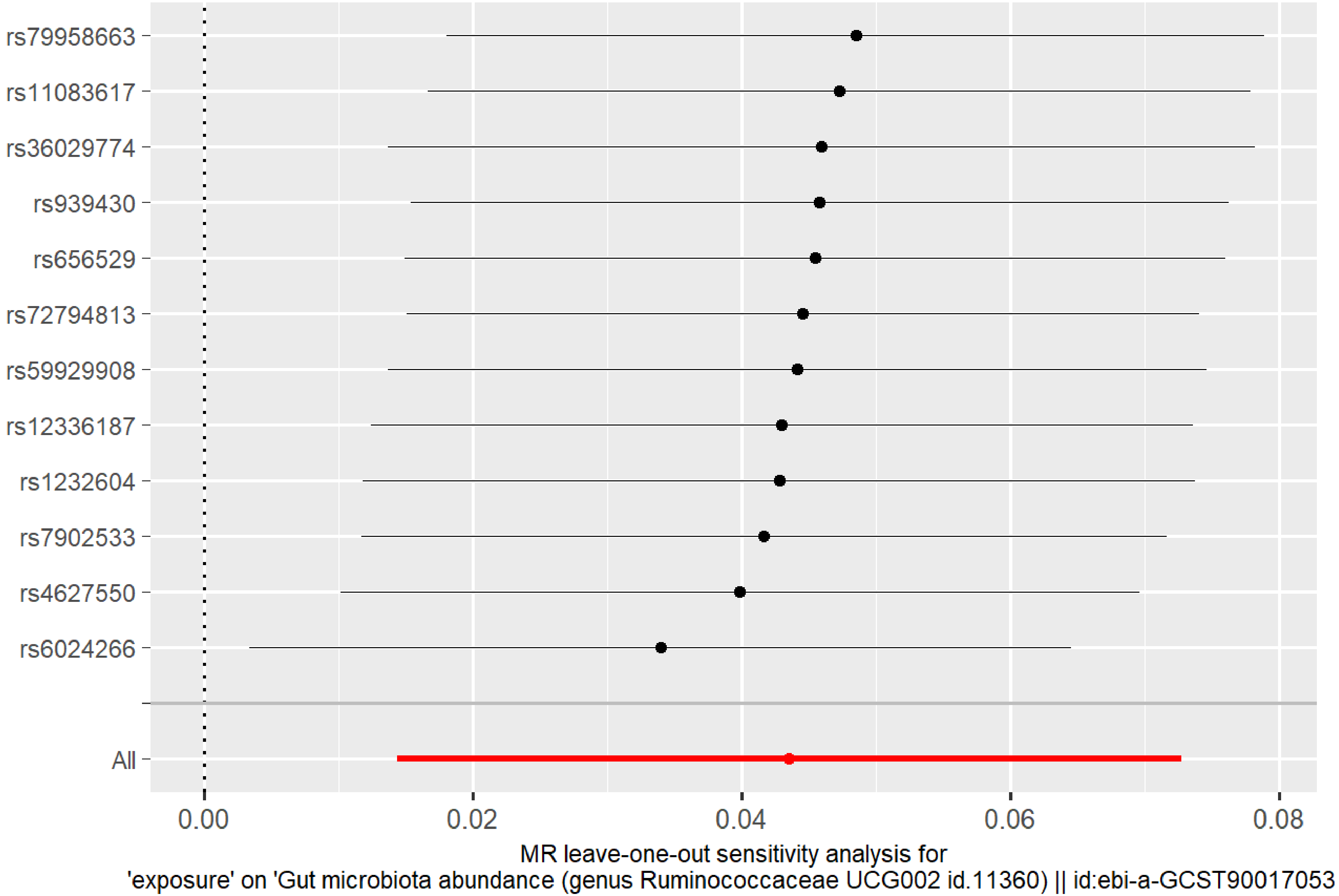
rs79958663
rs11083617
rs36029774
rs939430
rs656529
rs72794813
rs59929908
rs12336187
rs1232604
rs7902533
rs4627550
rs6024266
All
0.00
0.02
0.04
0.06
0.08
MR leave-one-out sensitivity analysis for
'exposure' on 'Gut microbiota abundance (genus Ruminococcaceae UCG002 id.11360) || id:ebi-a-GCST90017053

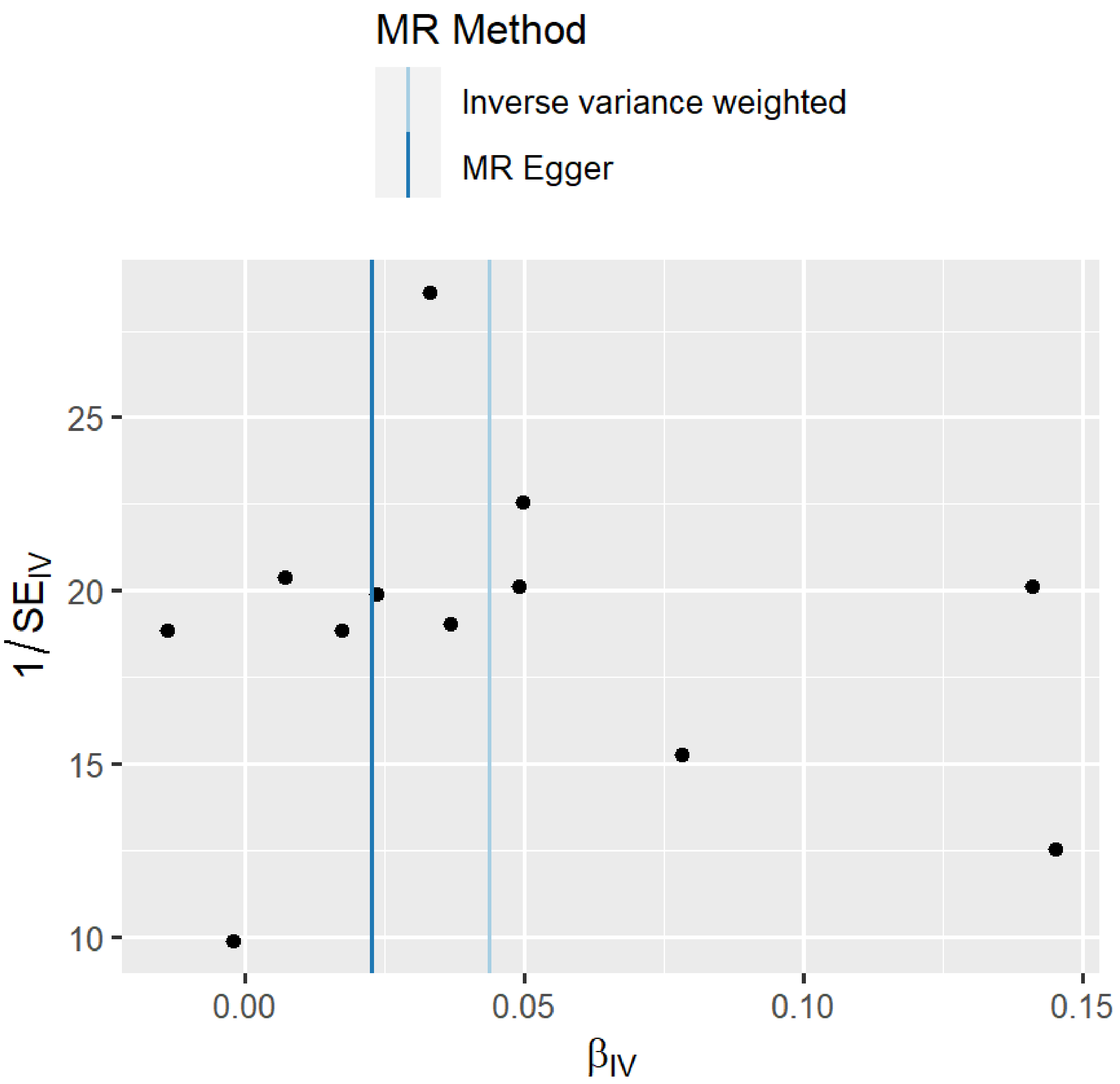
MR Method
Inverse variance weighted
MR Egger
25
20
15
10
1/SE$_{IV}$
0.00
0.05
0.10
0.15
β$_{IV}$

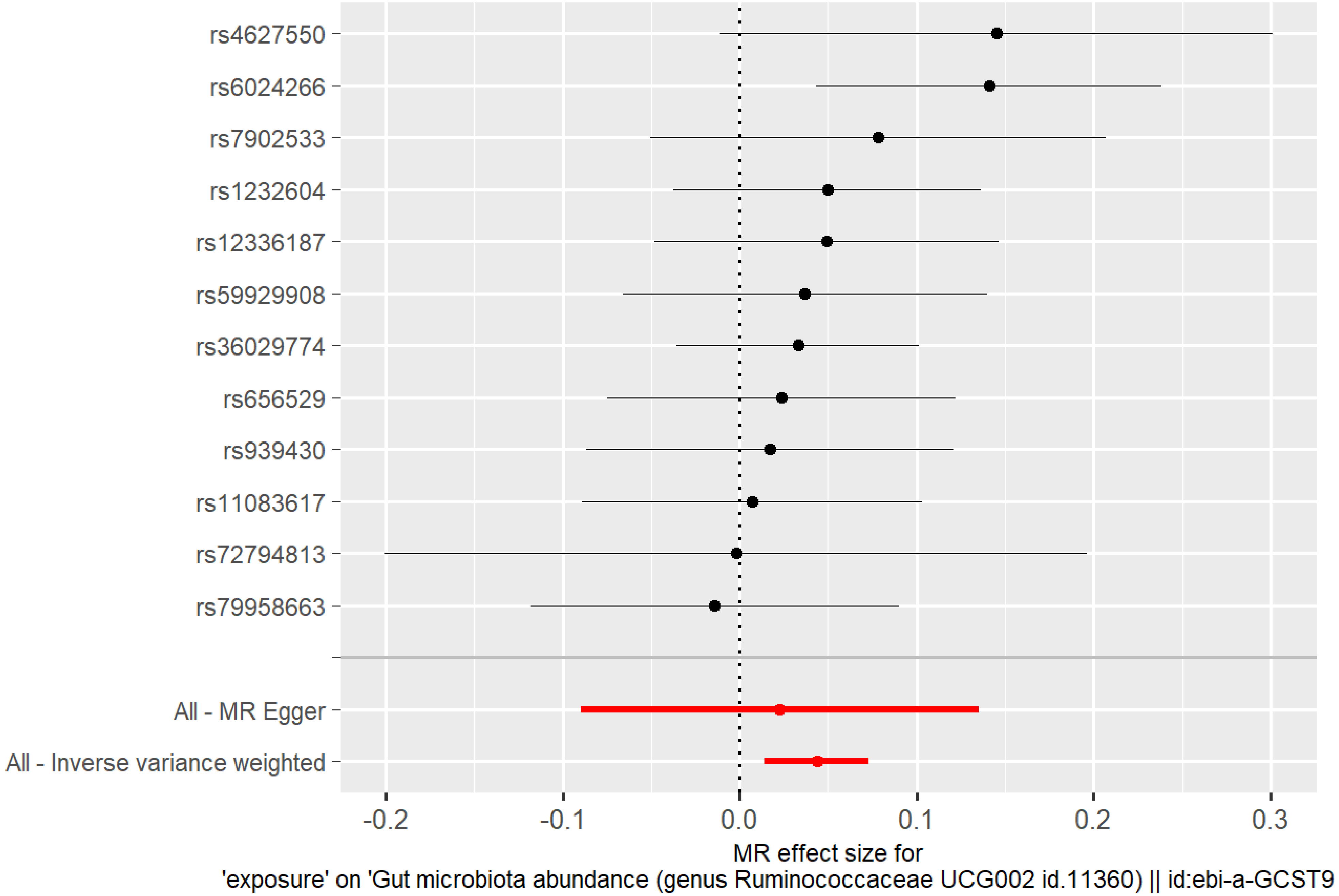
rs4627550
rs6024266
rs7902533
rs1232604
rs12336187
rs59929908
rs36029774
rs656529
rs939430
rs11083617
rs72794813
rs79958663
All - MR Egger
All - Inverse variance weighted
-0.2
-0.1
0.0
0.1
0.2
0.3
MR effect size for
'exposure' on 'Gut microbiota abundance (genus Ruminococcaceae UCG002 id.11360) || id:ebi-a-GCST9

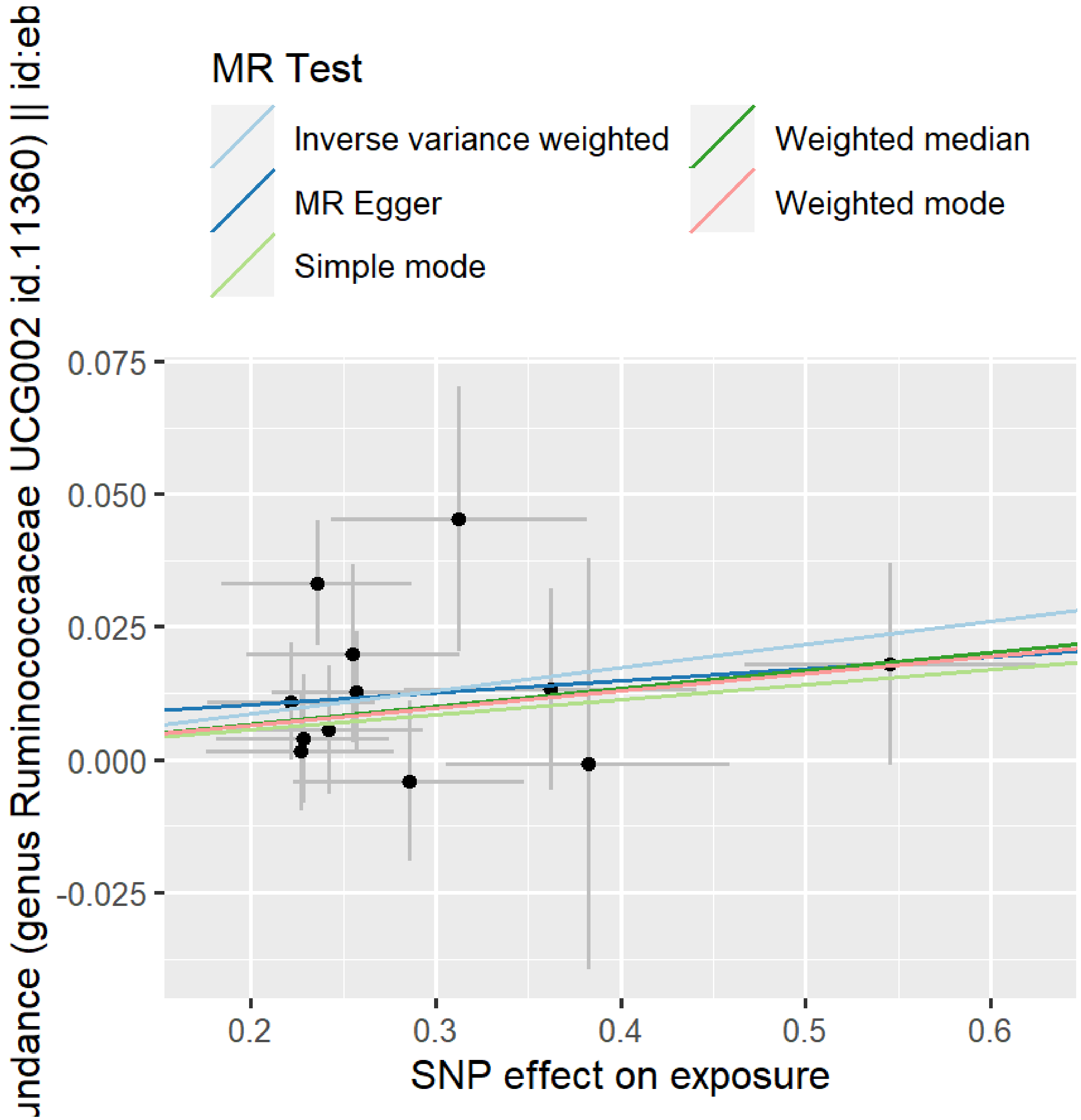
MR Test
Inverse variance weighted
MR Egger
Simple mode
Weighted median
Weighted mode
0.075
0.050
0.025
0.000
-0.025
0.2
0.3
0.4
0.5
0.6
SNP effect on exposure
undance (genus Ruminococcaceae UCG002 id.11360) || id:eb

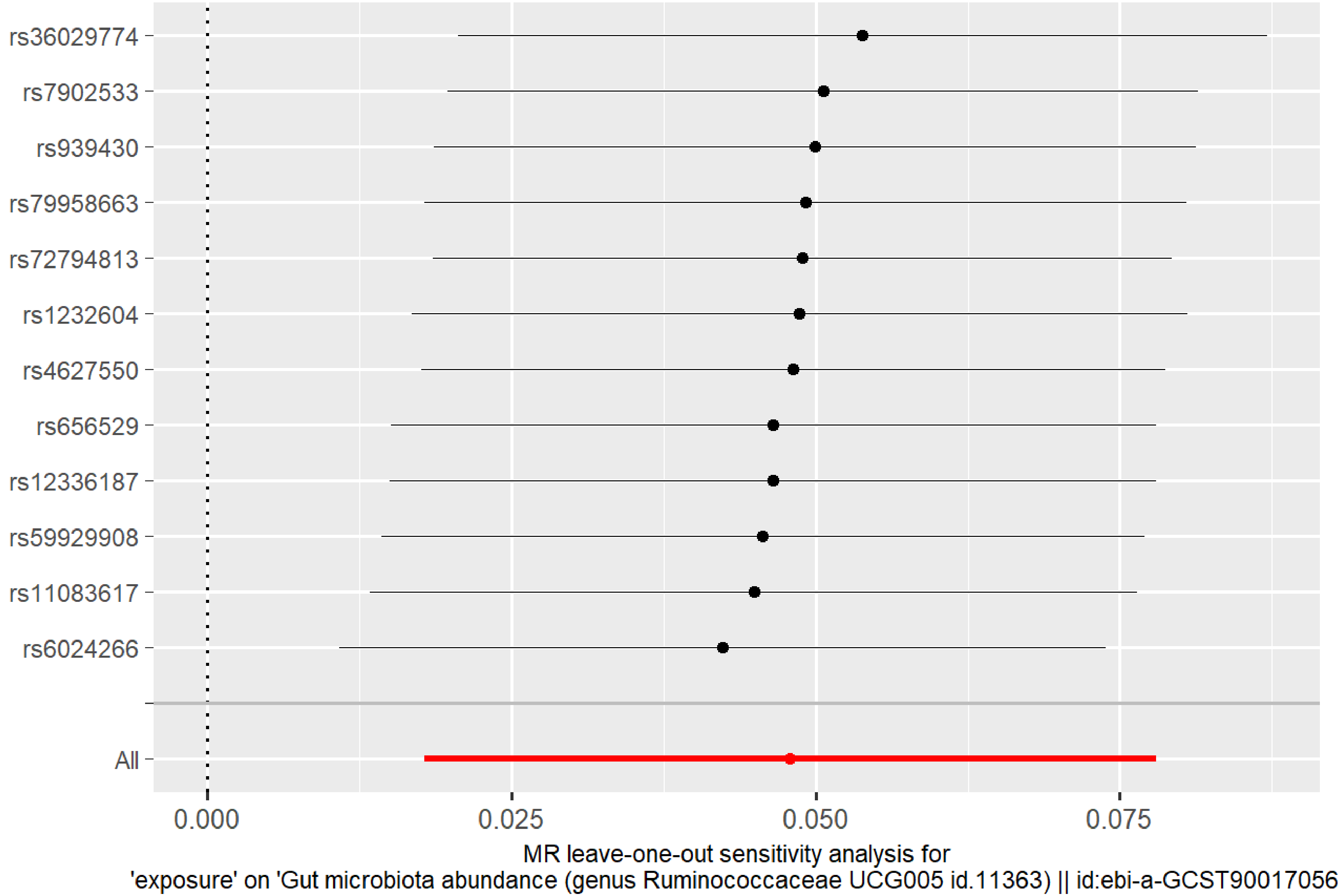
rs36029774
rs7902533
rs939430
rs79958663
rs72794813
rs1232604
rs4627550
rs656529
rs12336187
rs59929908
rs11083617
rs6024266
All
0.000
0.025
0.050
0.075
MR leave-one-out sensitivity analysis for
'exposure' on 'Gut microbiota abundance (genus Ruminococcaceae UCG005 id.11363) || id:ebi-a-GCST90017056

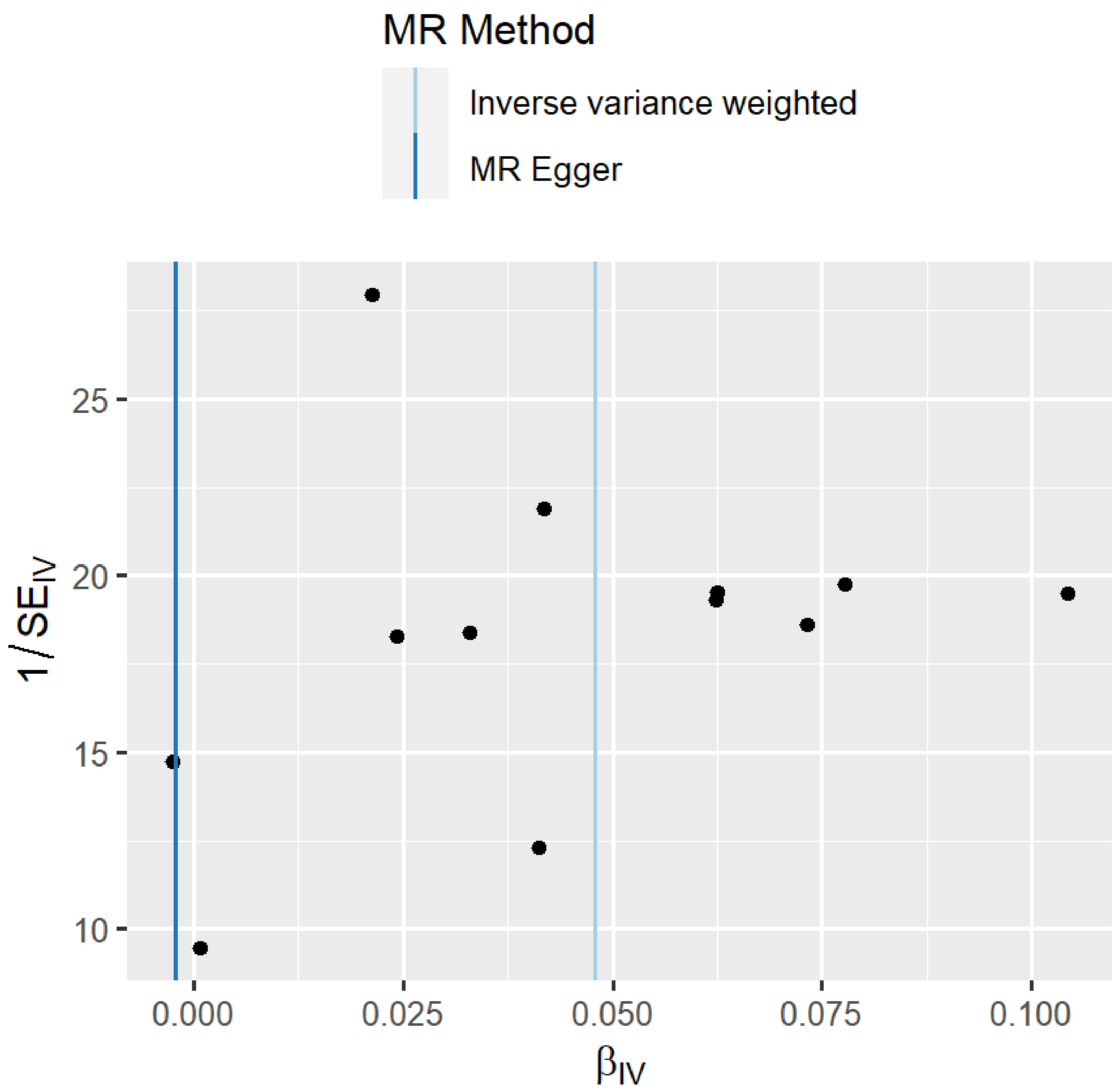
MR Method
Inverse variance weighted
MR Egger
25
20
15
10
0.000
0.025
0.050
0.075
0.100
β_{IV}
$1/SE_{IV}$

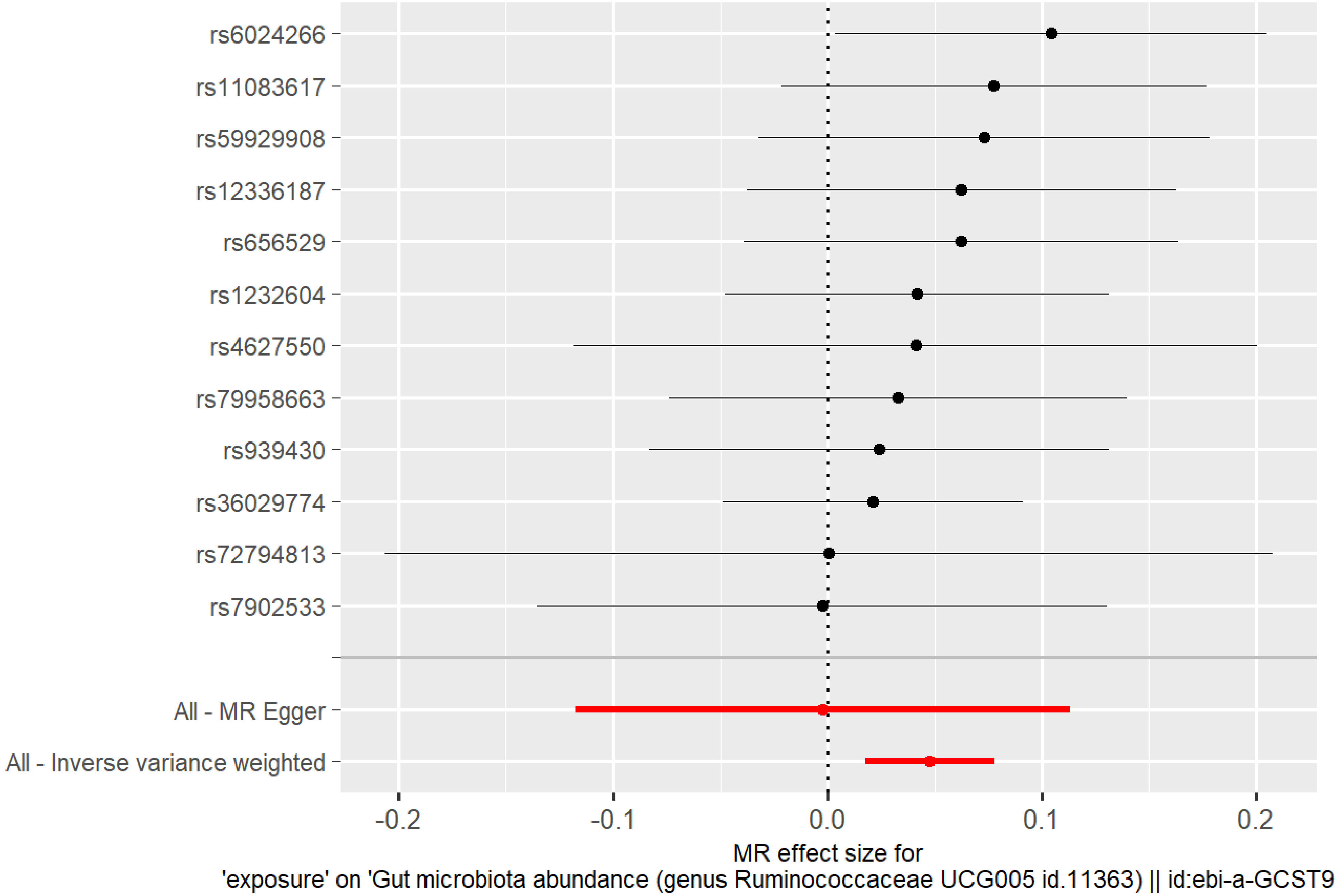
rs6024266
rs11083617
rs59929908
rs12336187
rs656529
rs1232604
rs4627550
rs79958663
rs939430
rs36029774
rs72794813
rs7902533
All - MR Egger
All - Inverse variance weighted
-0.2
-0.1
0.0
0.1
0.2
MR effect size for
'exposure' on 'Gut microbiota abundance (genus Ruminococcaceae UCG005 id.11363) || id:ebi-a-GCST9

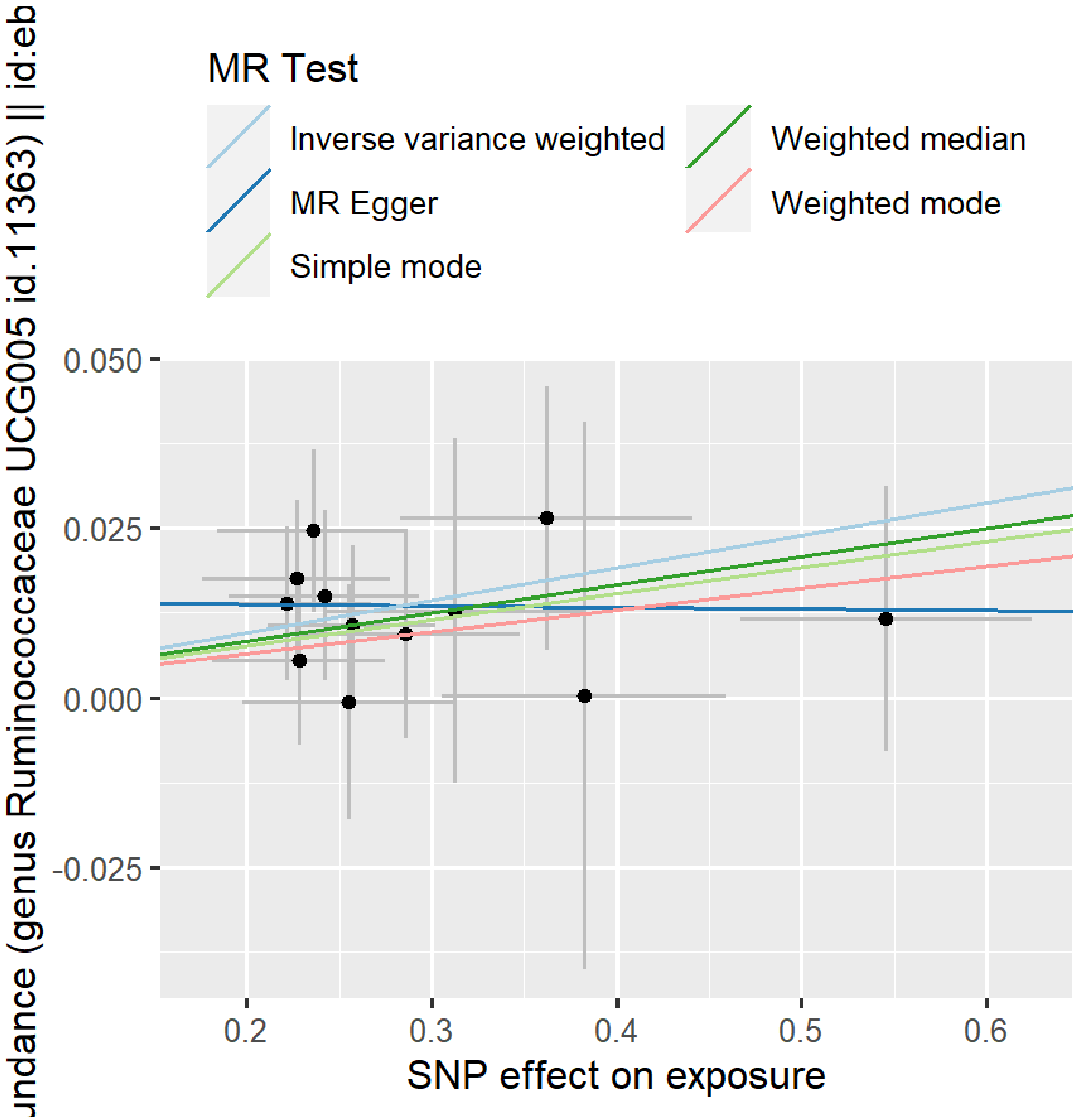
MR Test
Inverse variance weighted
MR Egger
Simple mode
Weighted median
Weighted mode
0.050
0.025
0.000
-0.025
0.2
0.3
0.4
0.5
0.6
SNP effect on exposure
undance (genus Ruminococcaceae UCG005 id.11363) || id:eb

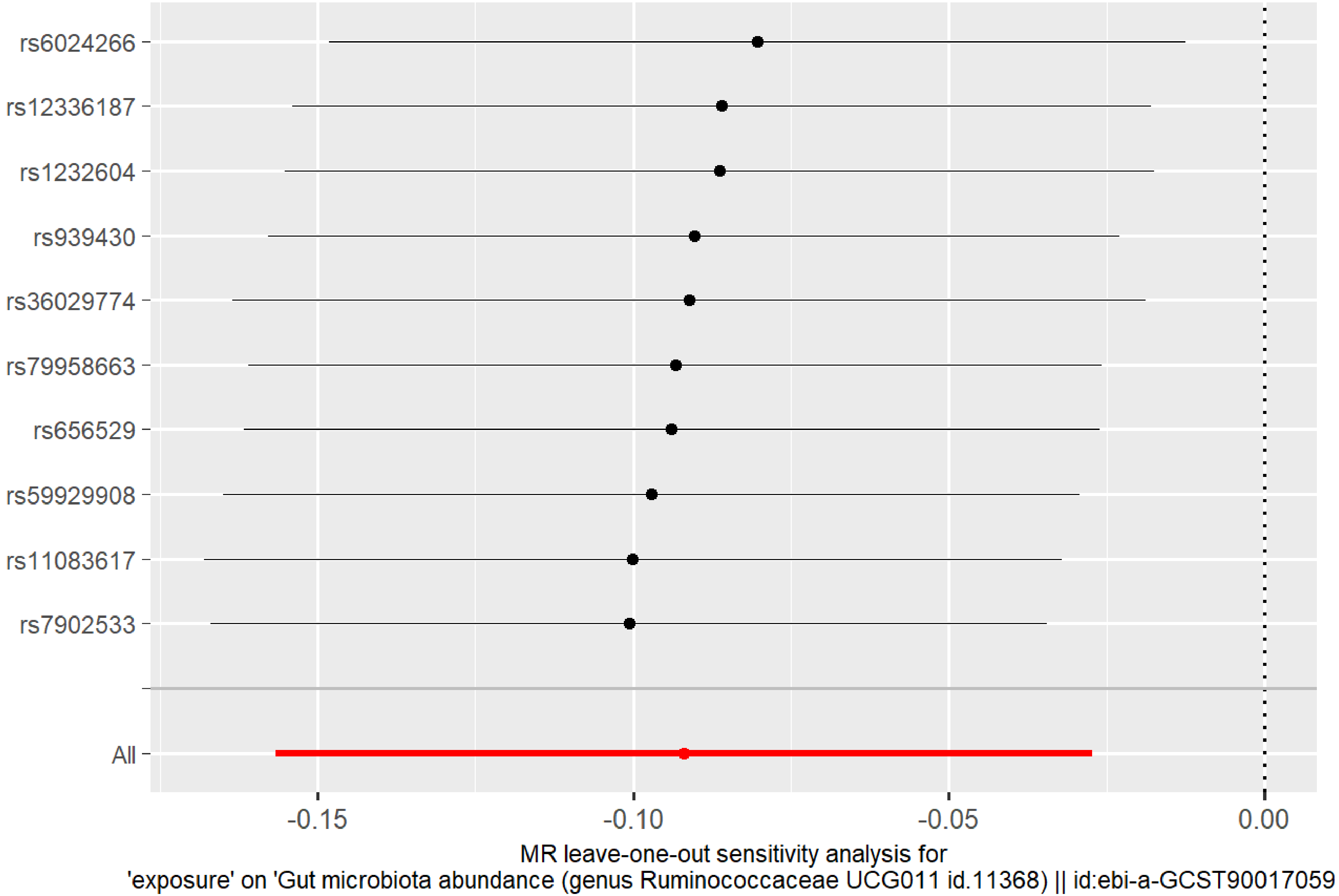

rs6024266
rs12336187
rs1232604
rs939430
rs36029774
rs79958663
rs656529
rs59929908
rs11083617
rs7902533
All
-0.15
-0.10
-0.05
0.00
MR leave-one-out sensitivity analysis for
'exposure' on 'Gut microbiota abundance (genus Ruminococcaceae UCG011 id.11368) || id:ebi-a-GCST90017059

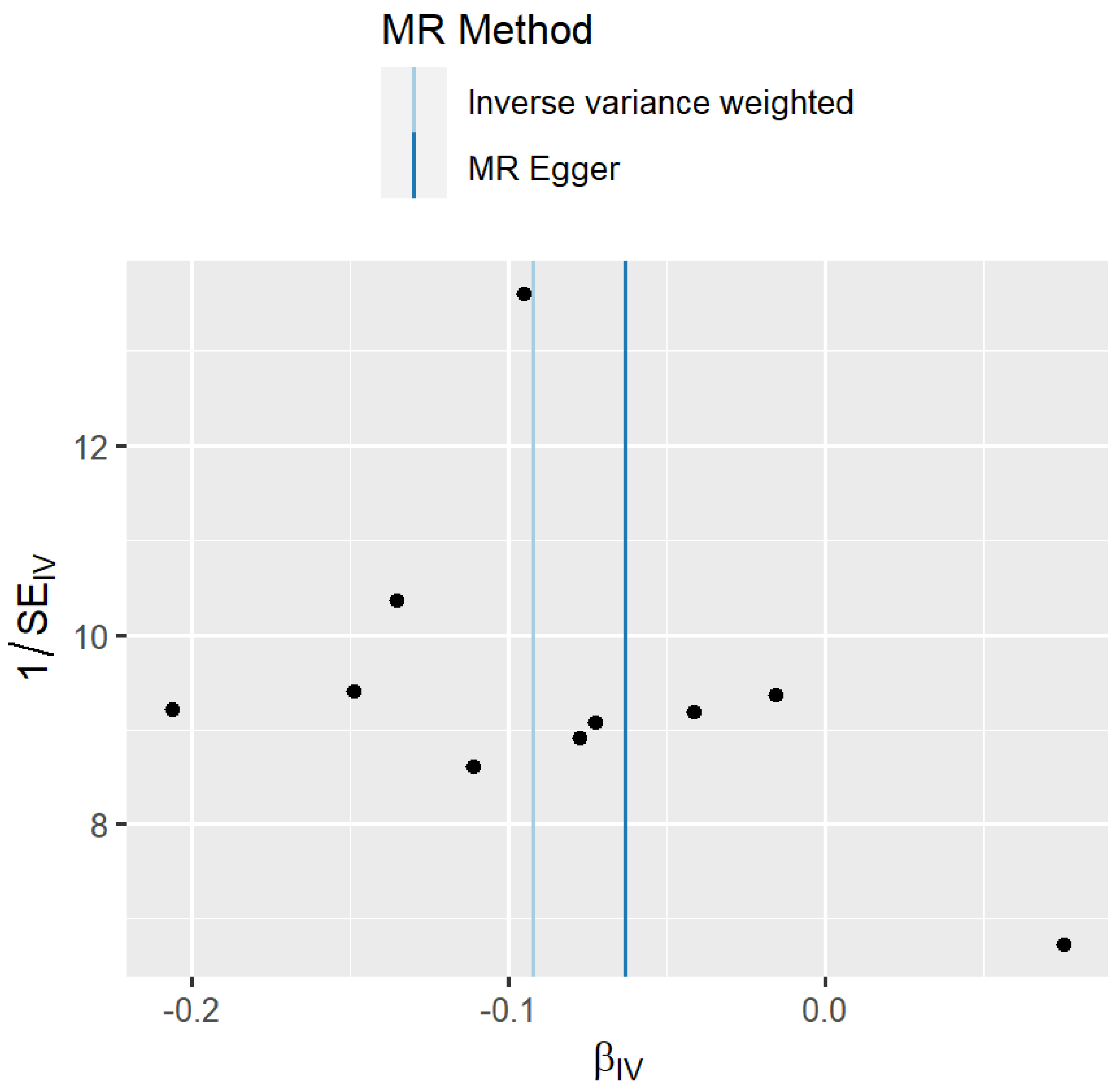
MR Method
Inverse variance weighted
MR Egger
12
10
8
1/SE_IV
-0.2
-0.1
0.0
β_IV

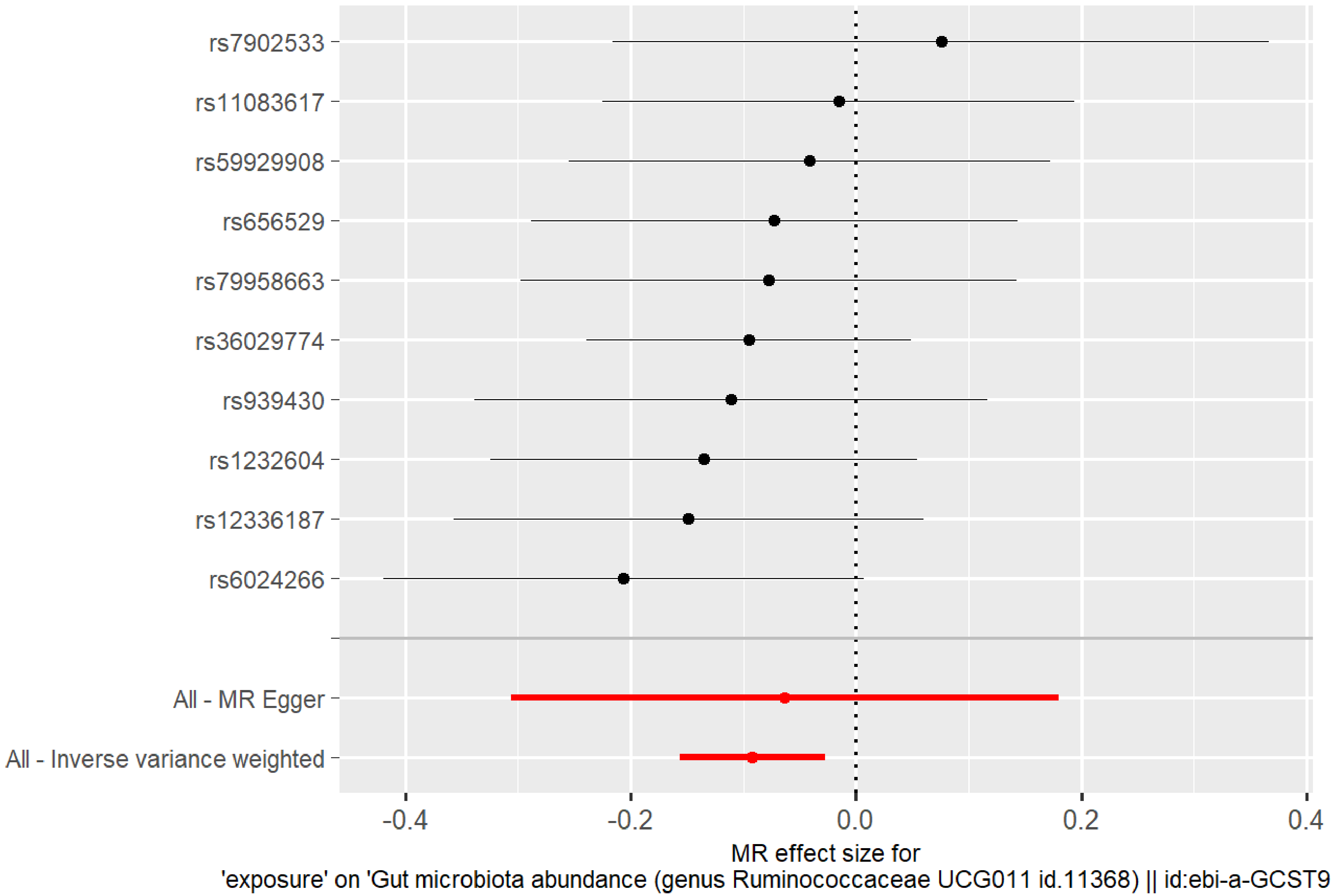
rs7902533
rs11083617
rs59929908
rs656529
rs79958663
rs36029774
rs939430
rs1232604
rs12336187
rs6024266
All - MR Egger
All - Inverse variance weighted
-0.4
-0.2
0.0
0.2
0.4
MR effect size for
'exposure' on 'Gut microbiota abundance (genus Ruminococcaceae UCG011 id.11368) || id:ebi-a-GCST9

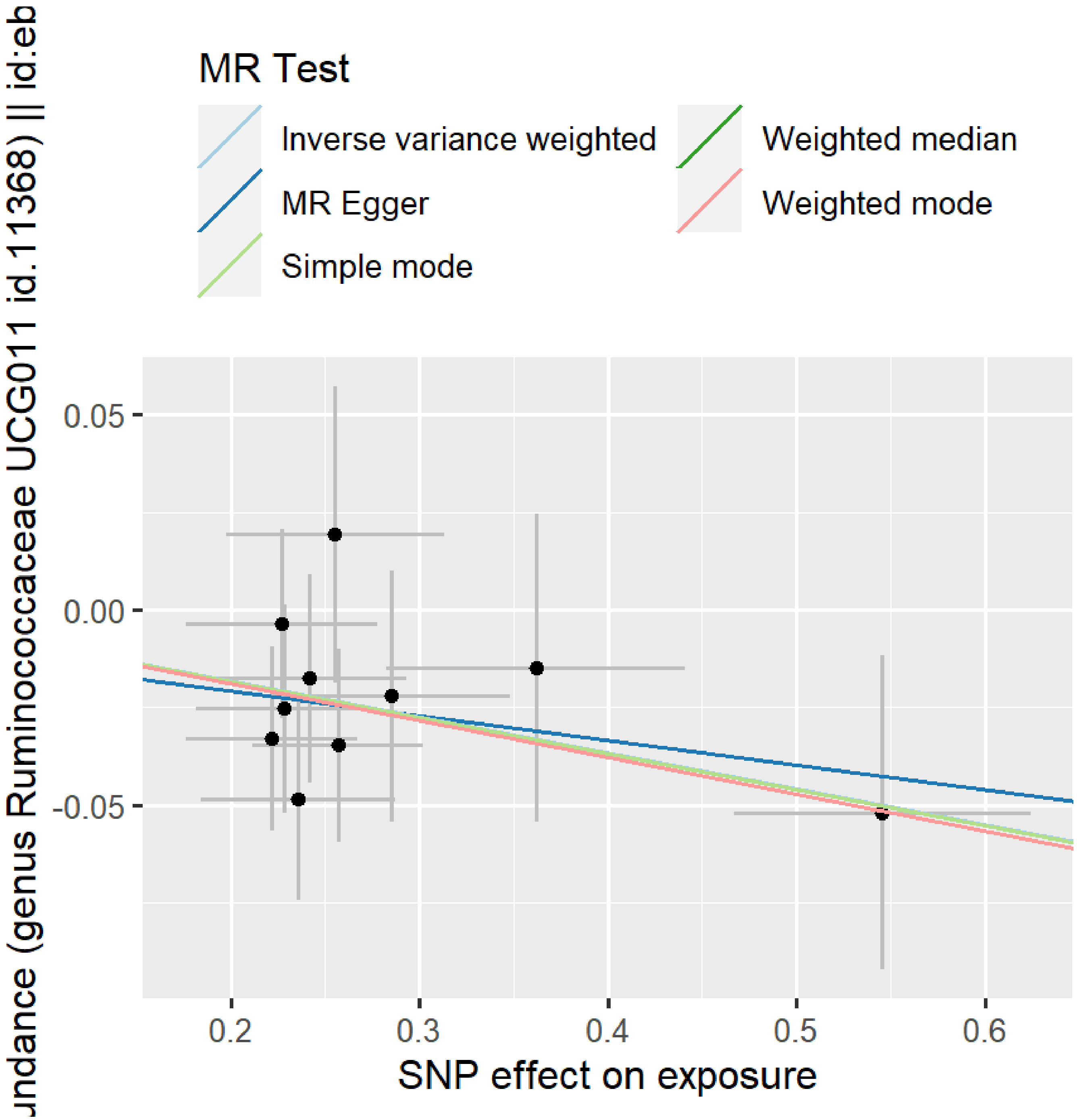
MR Test
Inverse variance weighted
MR Egger
Simple mode
Weighted median
Weighted mode
0.05
0.00
-0.05
0.2
0.3
0.4
0.5
0.6
SNP effect on exposure
undance (genus Ruminococcaceae UCG011 id.11368) || id:eb

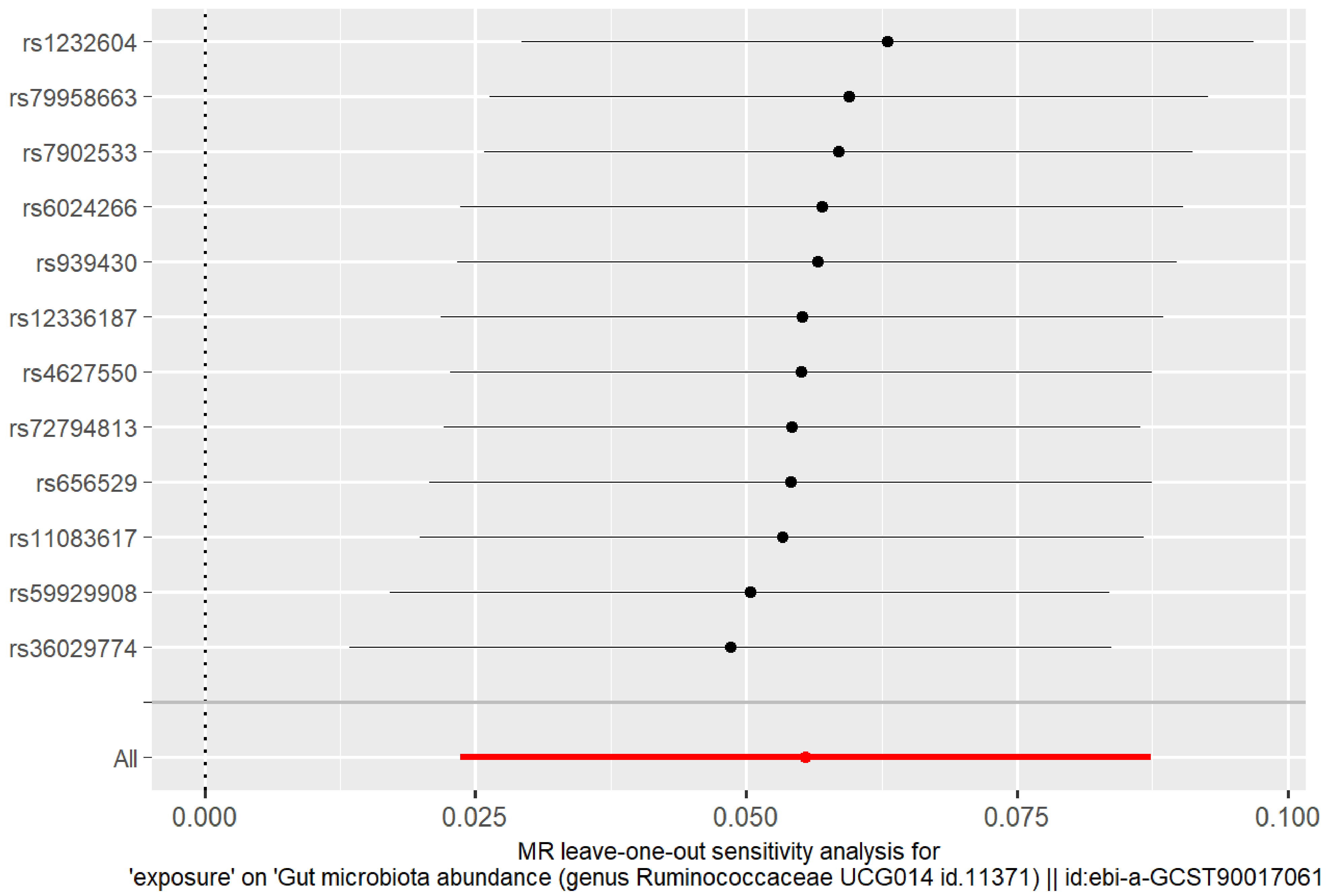
rs1232604
rs79958663
rs7902533
rs6024266
rs939430
rs12336187
rs4627550
rs72794813
rs656529
rs11083617
rs59929908
rs36029774
All
0.000
0.025
0.050
0.075
0.100
MR leave-one-out sensitivity analysis for
'exposure' on 'Gut microbiota abundance (genus Ruminococcaceae UCG014 id.11371) || id:ebi-a-GCST90017061

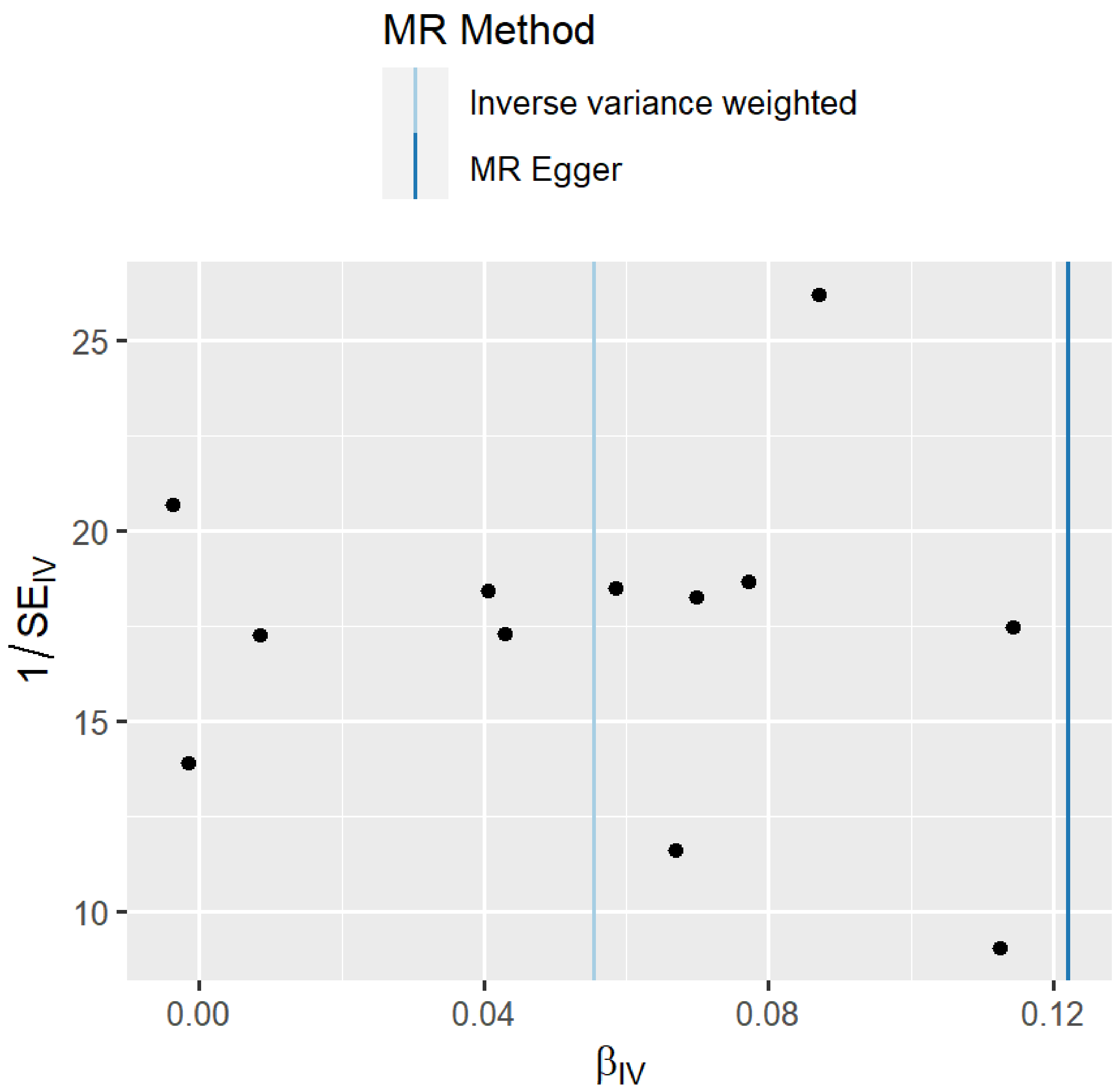
MR Method
Inverse variance weighted
MR Egger
25
20
15
10
1/SE$_{IV}$
0.00
0.04
0.08
0.12
β$_{IV}$

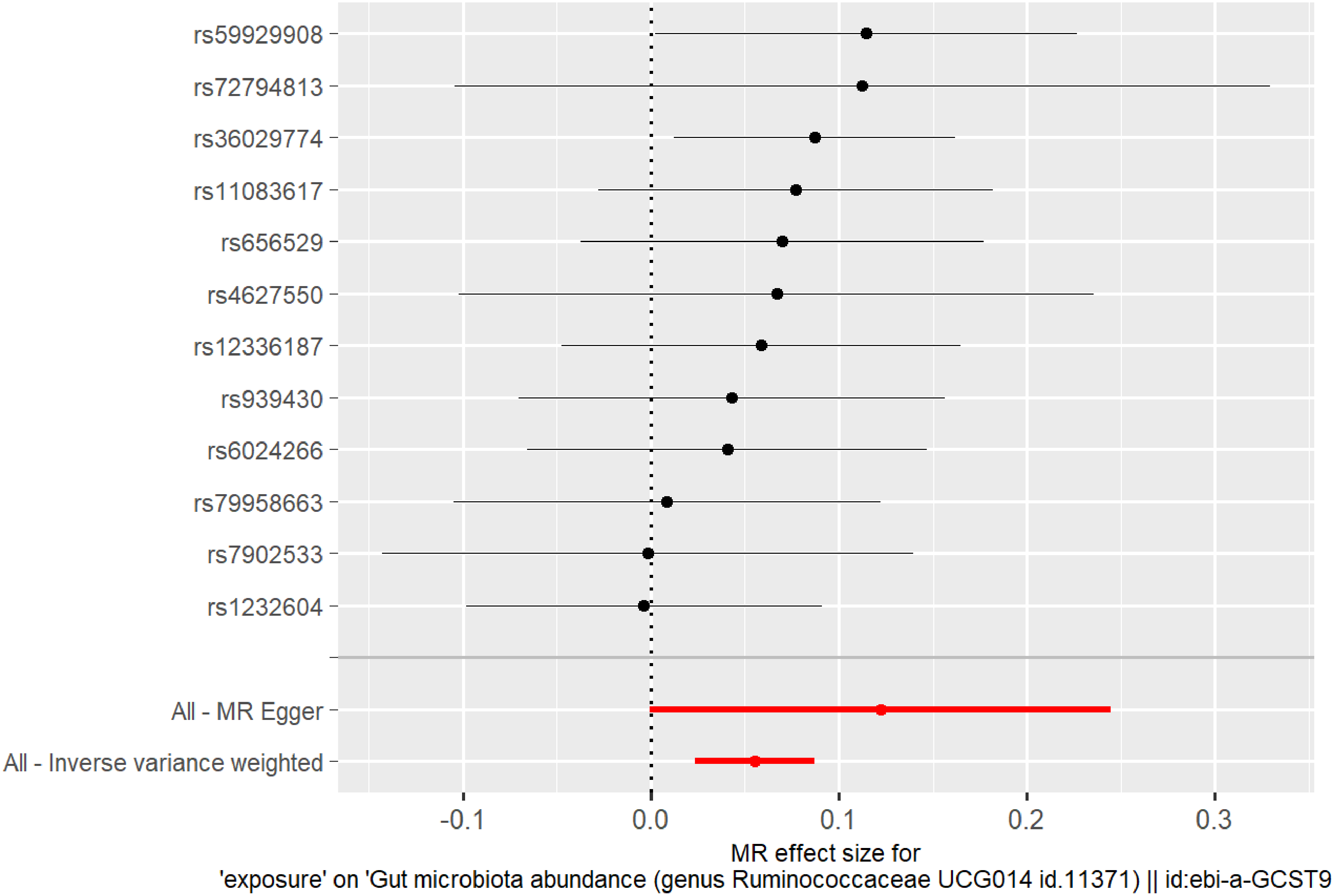
rs59929908
rs72794813
rs36029774
rs11083617
rs656529
rs4627550
rs12336187
rs939430
rs6024266
rs79958663
rs7902533
rs1232604
All - MR Egger
All - Inverse variance weighted
-0.1
0.0
0.1
0.2
0.3
MR effect size for
'exposure' on 'Gut microbiota abundance (genus Ruminococcaceae UCG014 id.11371) || id:ebi-a-GCST9

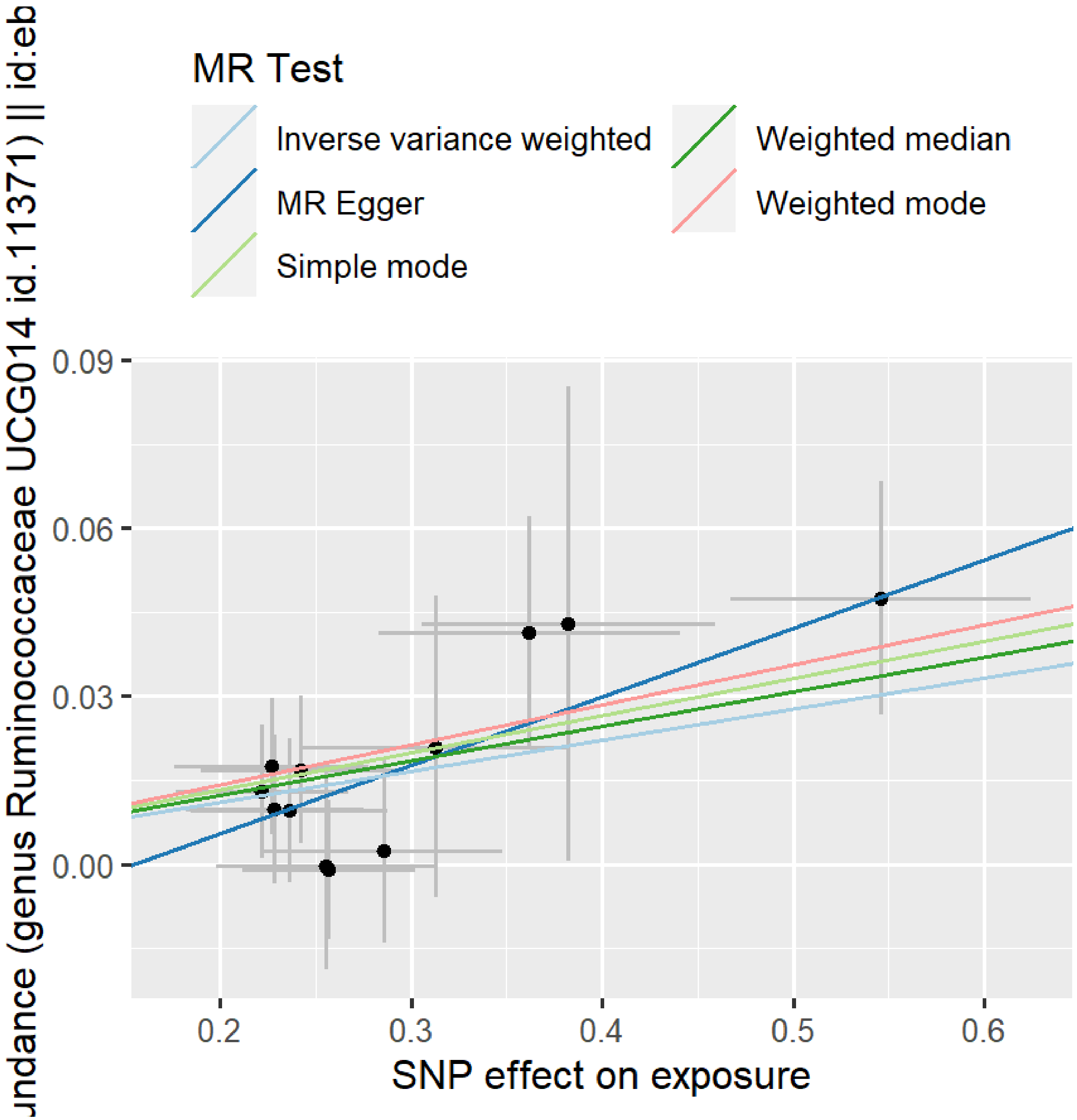

MR Test
Inverse variance weighted
MR Egger
Simple mode
Weighted median
Weighted mode
0.09
0.06
0.03
0.00
0.2
0.3
0.4
0.5
0.6
SNP effect on exposure
undance (genus Ruminococcaceae UCG014 id.11371) || id:eb

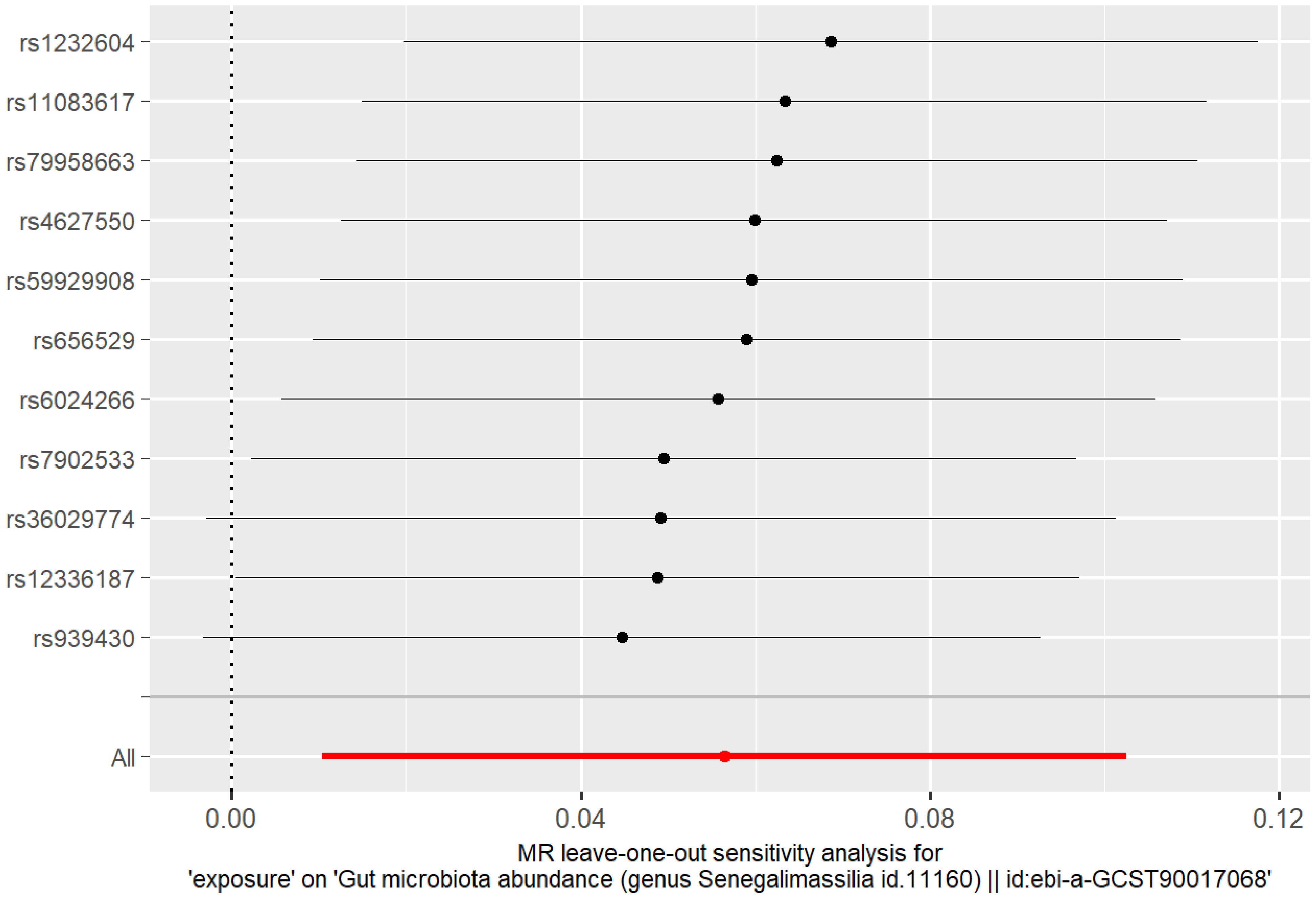
rs1232604
rs11083617
rs79958663
rs4627550
rs59929908
rs656529
rs6024266
rs7902533
rs36029774
rs12336187
rs939430
All
0.00
0.04
0.08
0.12
MR leave-one-out sensitivity analysis for
'exposure' on 'Gut microbiota abundance (genus Senegalimassilia id.11160) || id:ebi-a-GCST90017068'

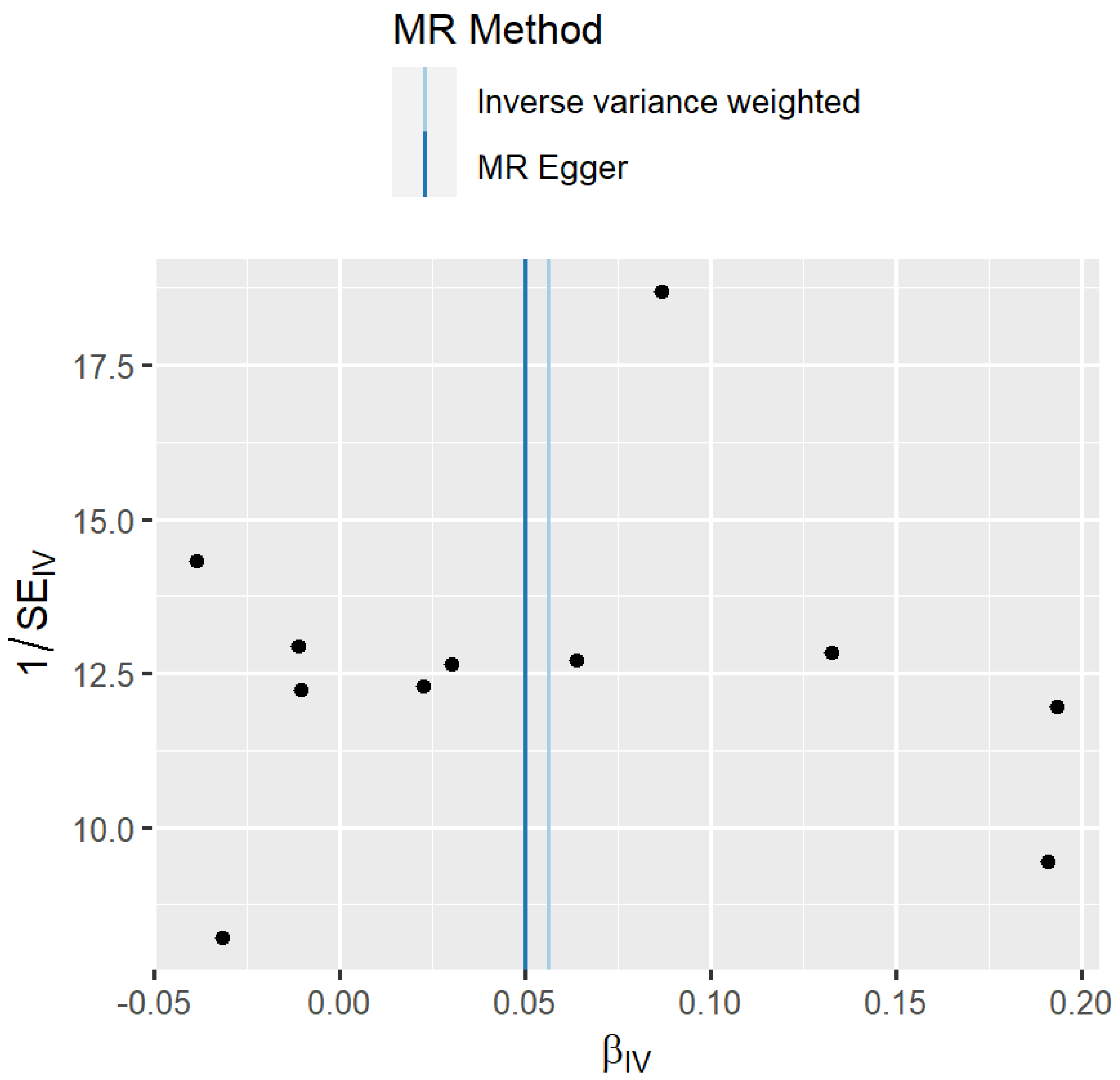
MR Method
Inverse variance weighted
MR Egger
17.5
15.0
12.5
10.0
1/SE_IV
-0.05
0.00
0.05
0.10
0.15
0.20
β_IV

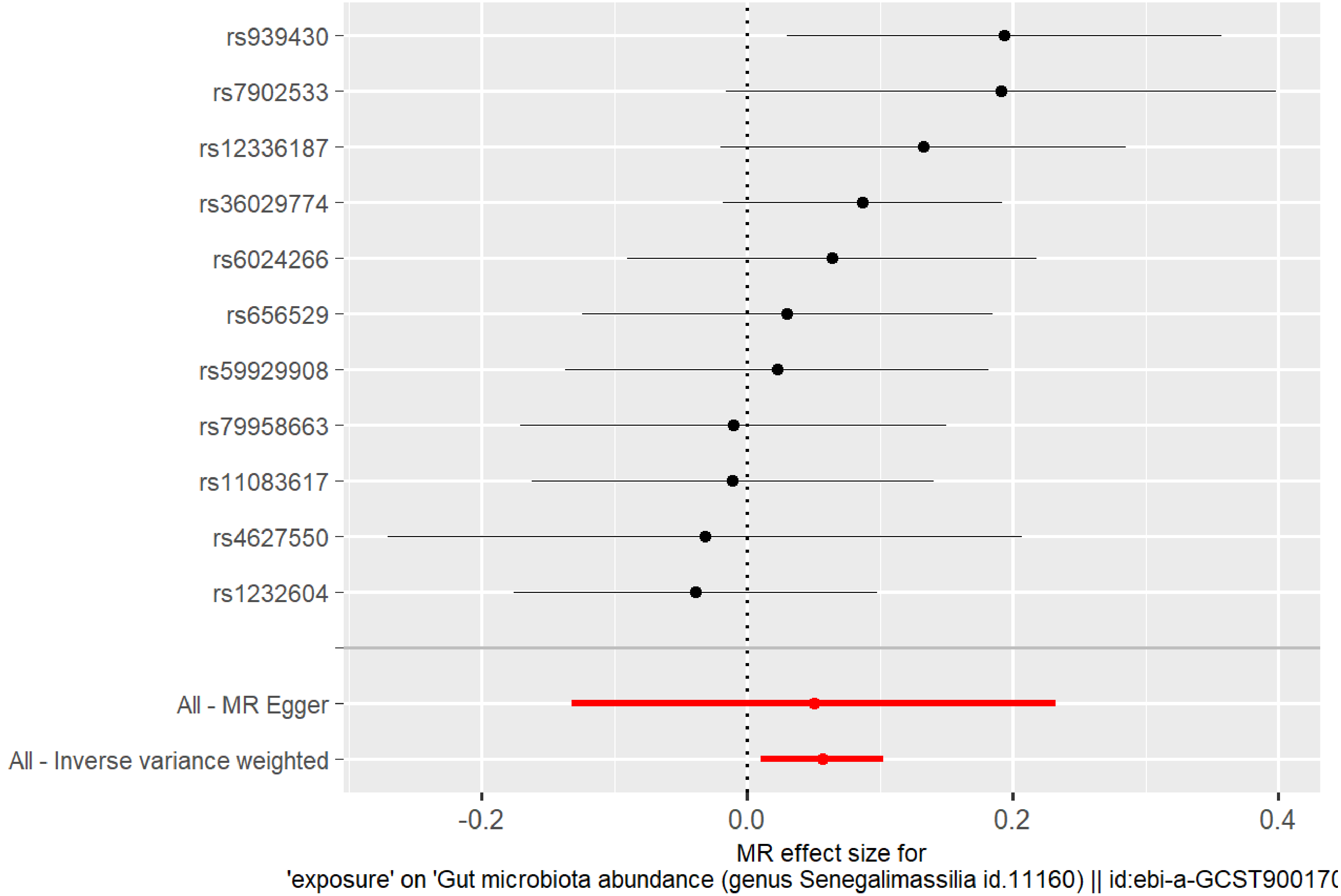
rs939430
rs7902533
rs12336187
rs36029774
rs6024266
rs656529
rs59929908
rs79958663
rs11083617
rs4627550
rs1232604
All - MR Egger
All - Inverse variance weighted
-0.2
0.0
0.2
0.4
MR effect size for
'exposure' on 'Gut microbiota abundance (genus Senegalimassilia id.11160) || id:ebi-a-GCST90017

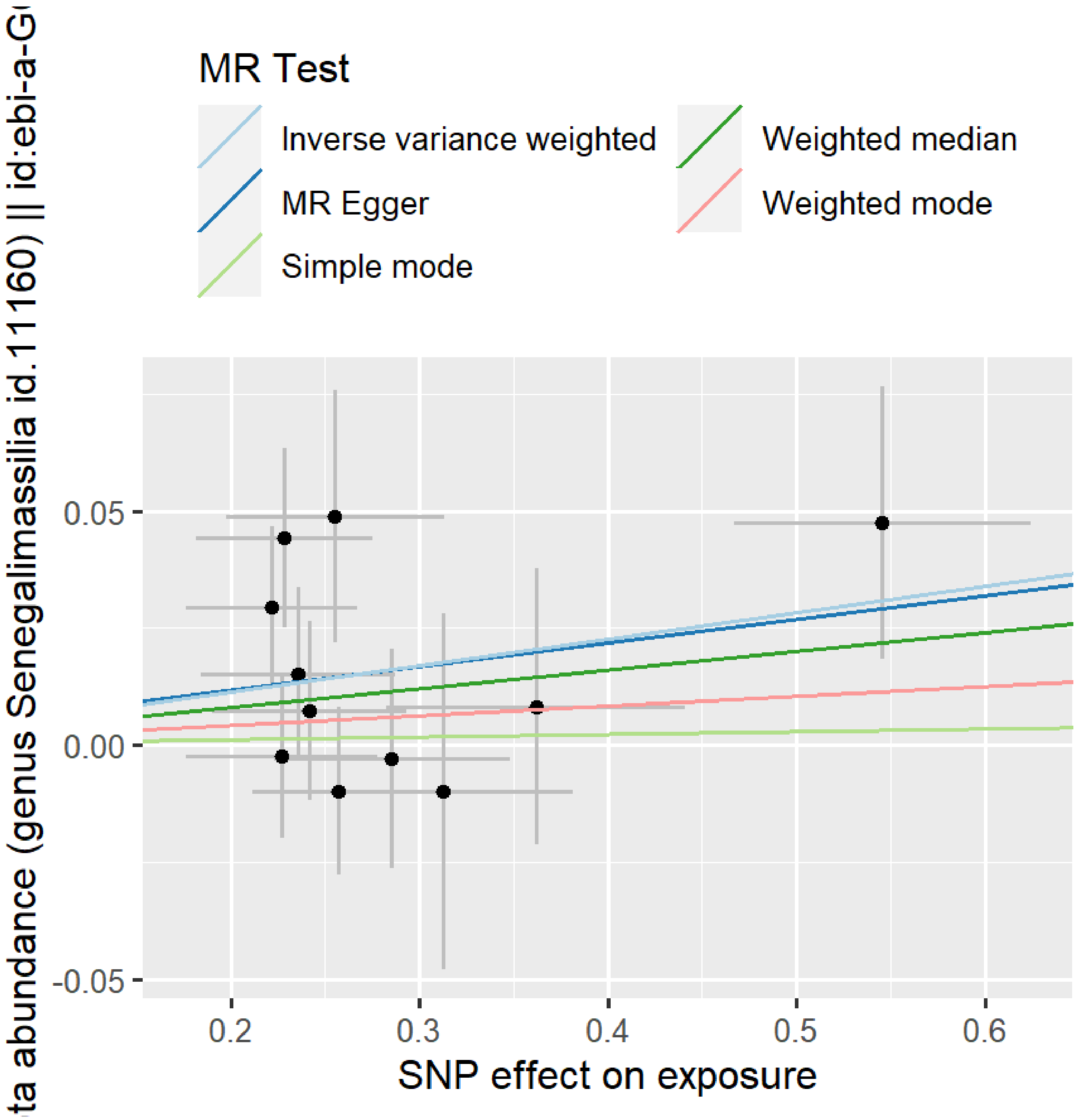
MR Test
Inverse variance weighted
MR Egger
Simple mode
Weighted median
Weighted mode
0.05
0.00
-0.05
0.2
0.3
0.4
0.5
0.6
SNP effect on exposure
ta abundance (genus Senegalimassilia id.11160) || id:ebi-a-G

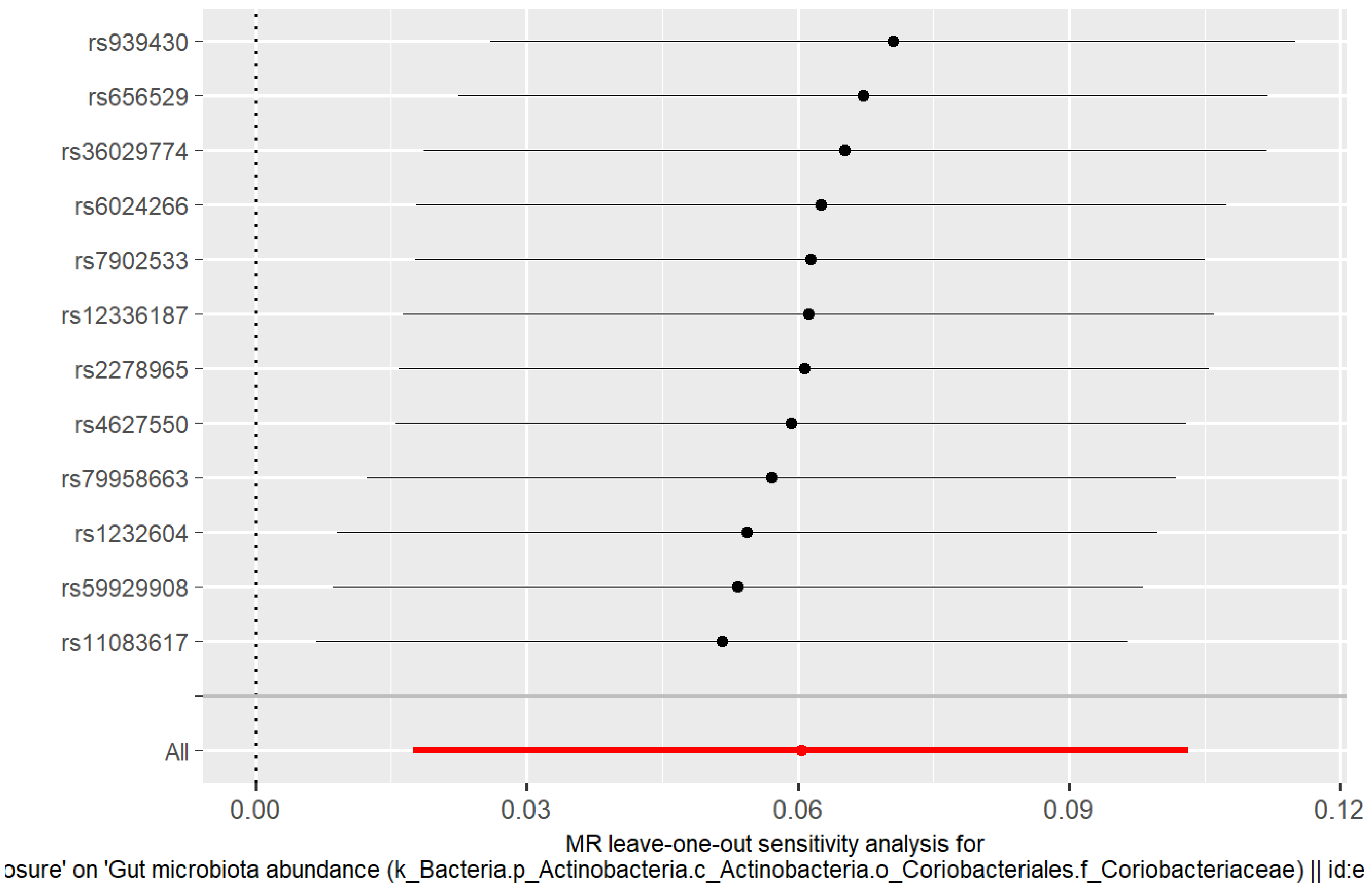
rs939430
rs656529
rs36029774
rs6024266
rs7902533
rs12336187
rs2278965
rs4627550
rs79958663
rs1232604
rs59929908
rs11083617
All
0.00
0.03
0.06
0.09
0.12
MR leave-one-out sensitivity analysis for
osure' on 'Gut microbiota abundance (k_Bacteria.p_Actinobacteria.c_Actinobacteria.o_Coriobacteriales.f_Coriobacteriaceae) || id:e

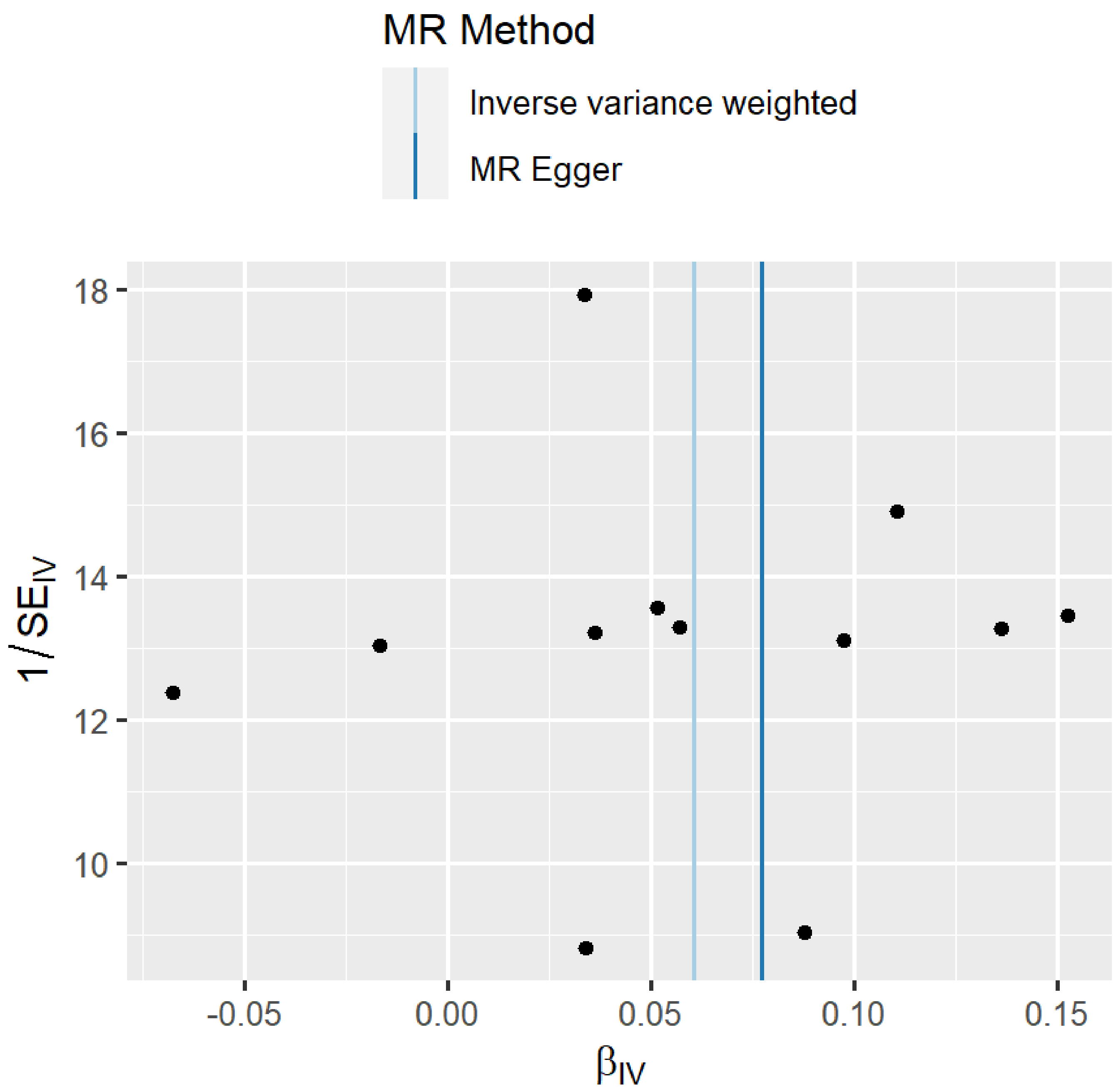
MR Method
Inverse variance weighted
MR Egger
18
16
14
12
10
1/SE_IV
-0.05
0.00
0.05
0.10
0.15
β_IV

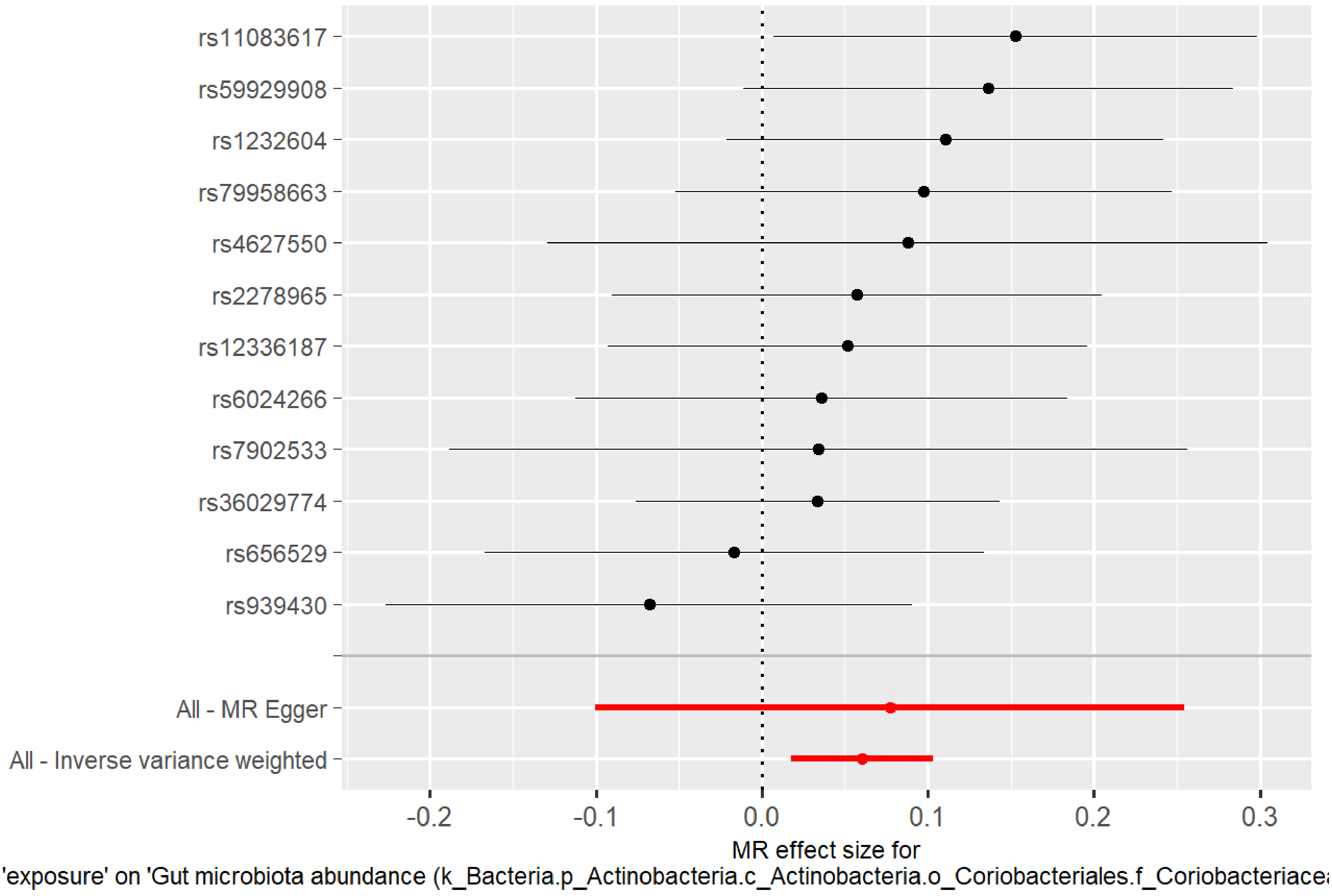
rs11083617
rs59929908
rs1232604
rs79958663
rs4627550
rs2278965
rs12336187
rs6024266
rs7902533
rs36029774
rs656529
rs939430
All - MR Egger
All - Inverse variance weighted
-0.2
-0.1
0.0
0.1
0.2
0.3
MR effect size for
'exposure' on 'Gut microbiota abundance (k_Bacteria.p_Actinobacteria.c_Actinobacteria.o_Coriobacteriales.f_Coriobacteriace

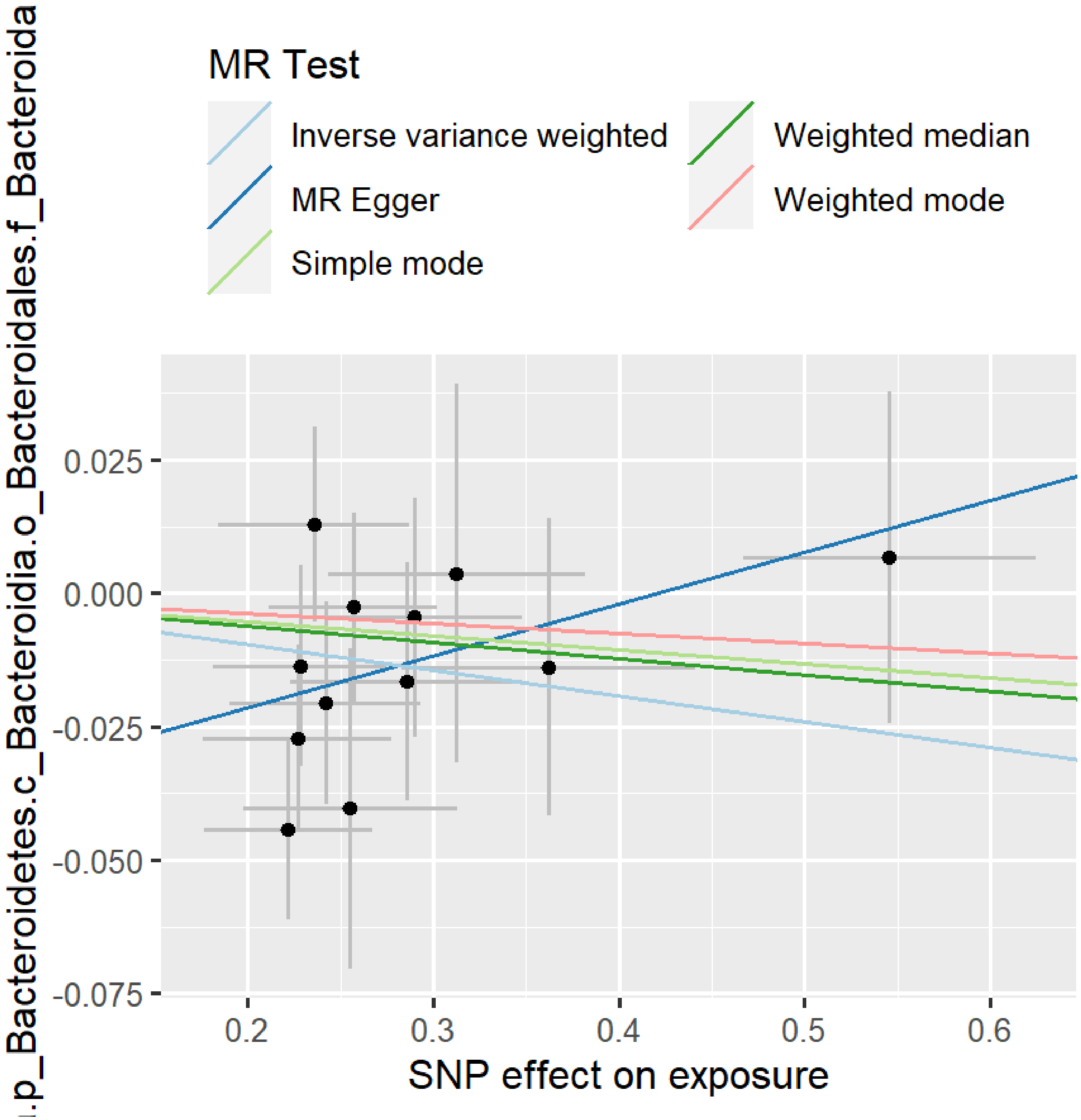
MR Test
Inverse variance weighted
MR Egger
Simple mode
Weighted median
Weighted mode
0.025
0.000
-0.025
-0.050
-0.075
0.2
0.3
0.4
0.5
0.6
SNP effect on exposure
.p_Bacteroidetes.c_Bacteroidia.o_Bacteroidales.f_Bacteroida

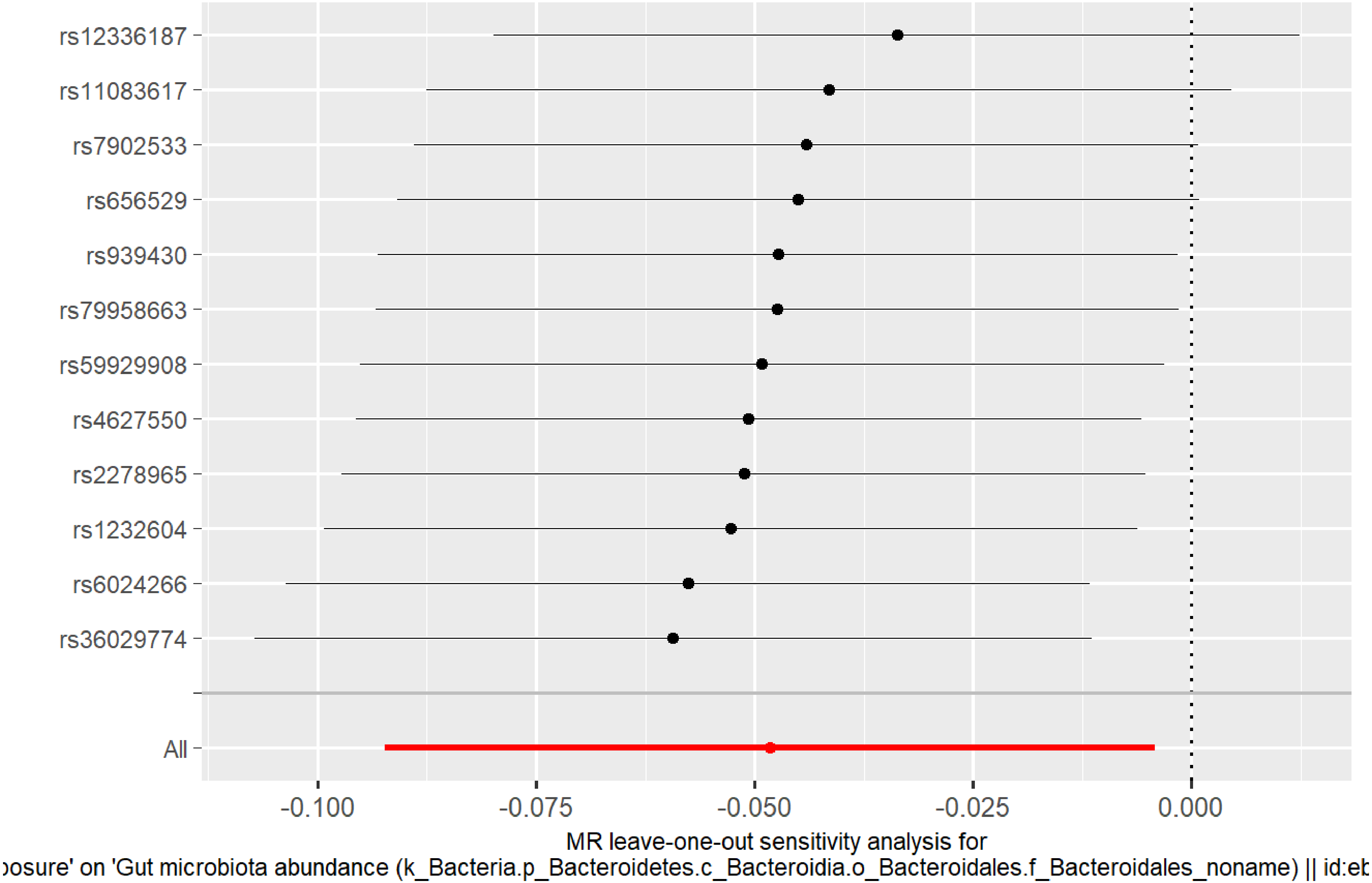
rs12336187
rs11083617
rs7902533
rs656529
rs939430
rs79958663
rs59929908
rs4627550
rs2278965
rs1232604
rs6024266
rs36029774
All
-0.100
-0.075
-0.050
-0.025
0.000
MR leave-one-out sensitivity analysis for
osure' on 'Gut microbiota abundance (k_Bacteria.p_Bacteroidetes.c_Bacteroidia.o_Bacteroidales.f_Bacteroidales_noname) || id:eb

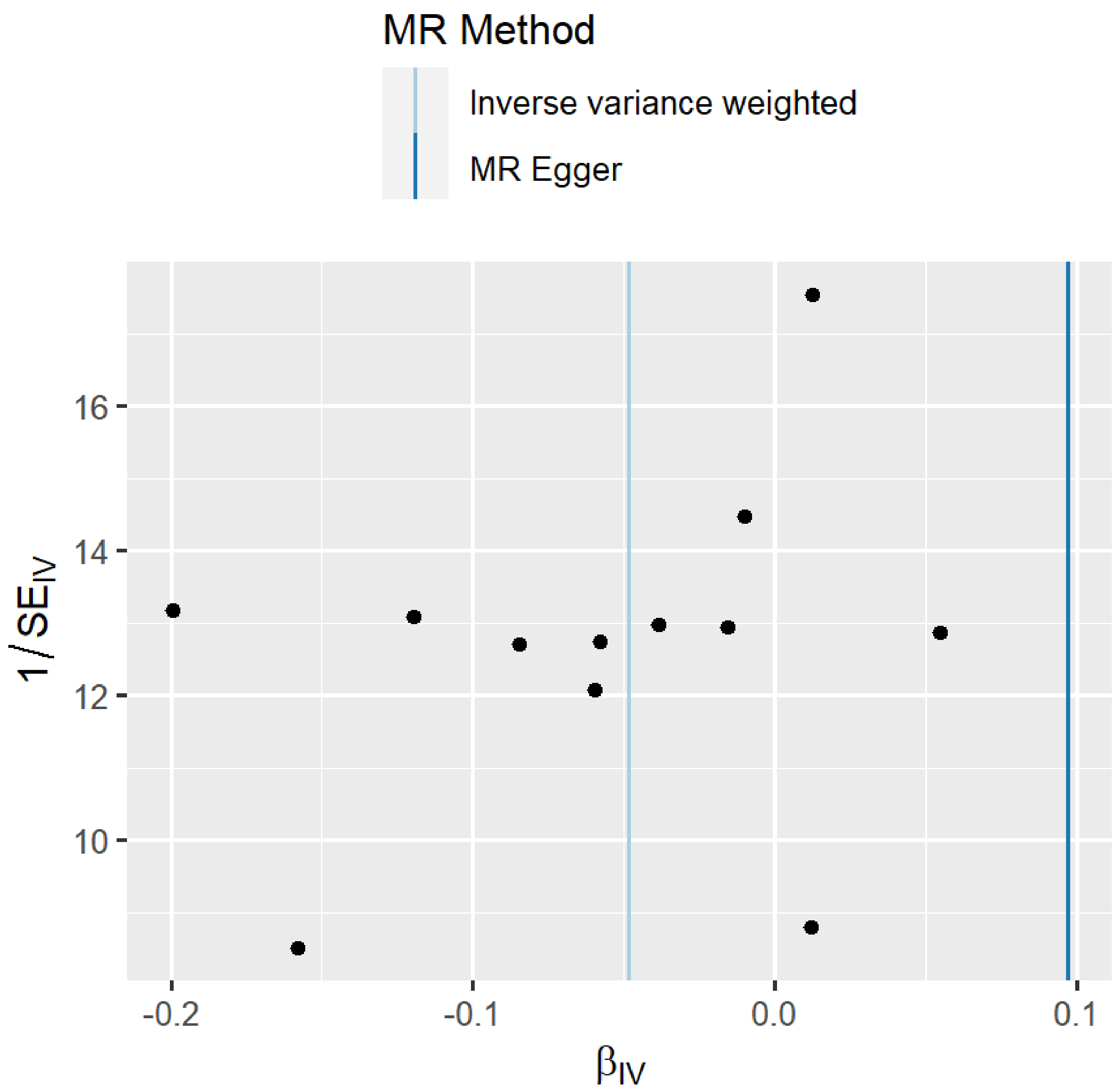

MR Method
Inverse variance weighted
MR Egger
16
14
12
10
1/SE_IV
-0.2
-0.1
0.0
0.1
β_IV

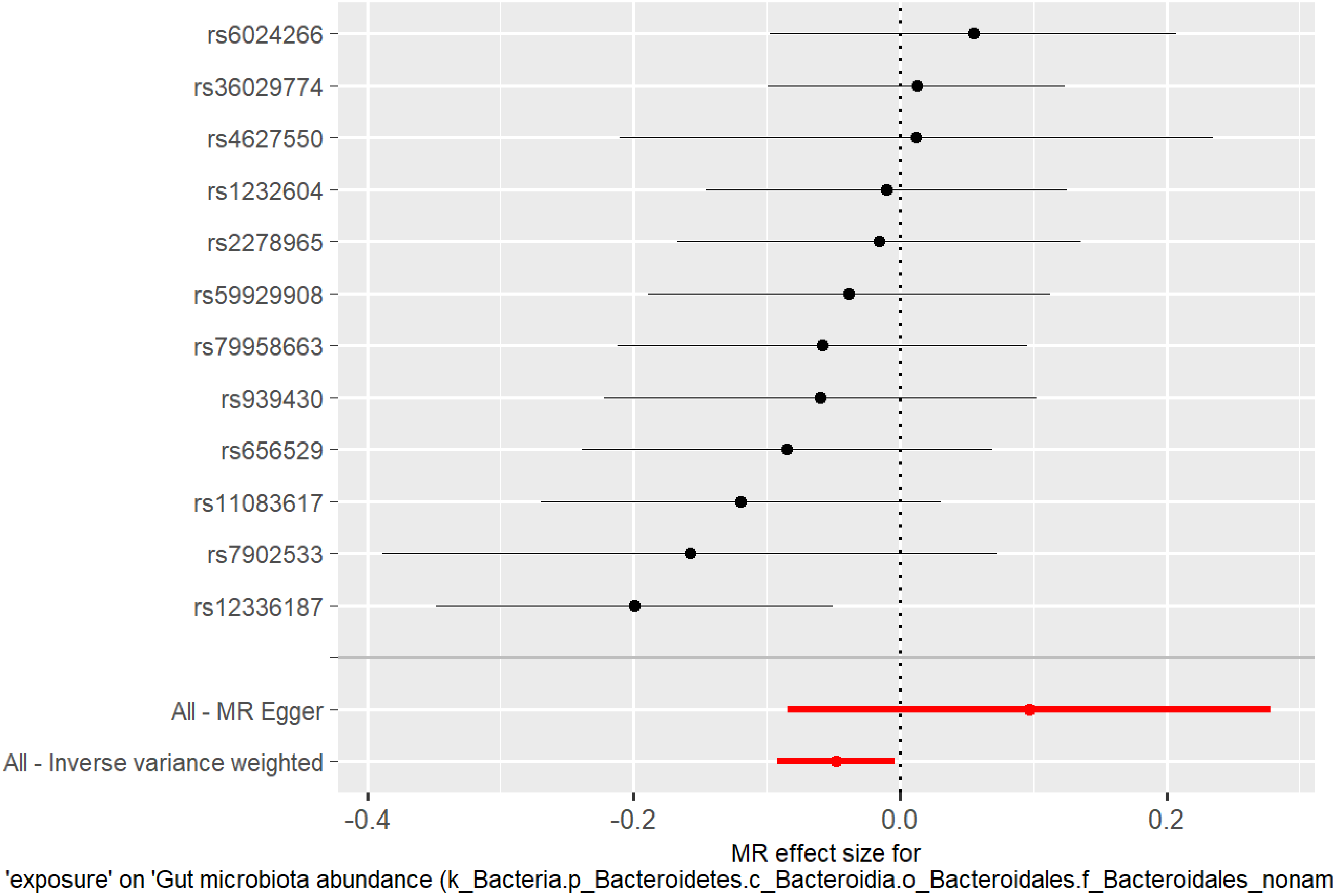
rs6024266
rs36029774
rs4627550
rs1232604
rs2278965
rs59929908
rs79958663
rs939430
rs656529
rs11083617
rs7902533
rs12336187
All - MR Egger
All - Inverse variance weighted
-0.4
-0.2
0.0
0.2
MR effect size for
'exposure' on 'Gut microbiota abundance (k_Bacteria.p_Bacteroidetes.c_Bacteroidia.o_Bacteroidales.f_Bacteroidales_nonam

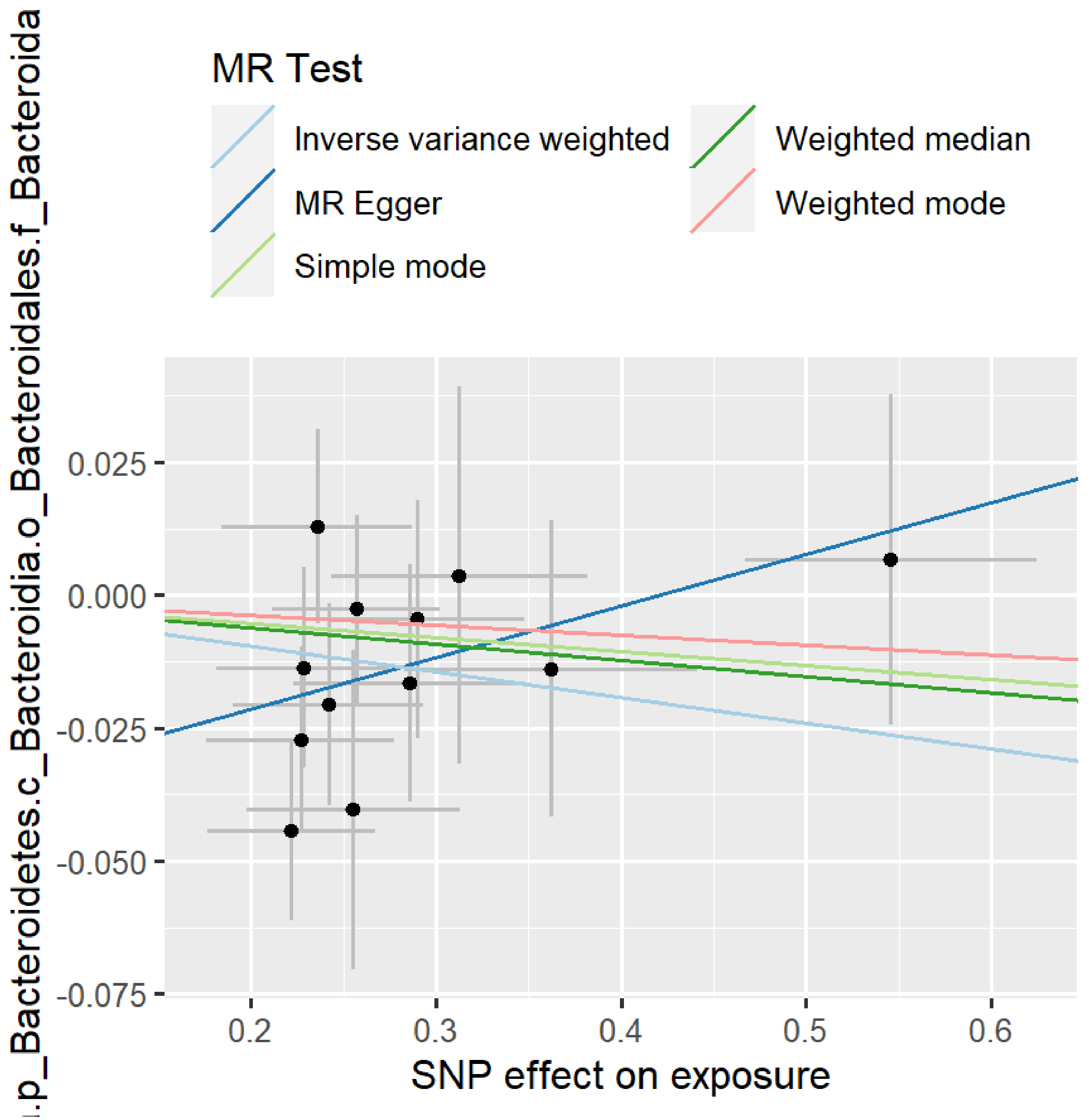
MR Test
Inverse variance weighted
MR Egger
Simple mode
Weighted median
Weighted mode
0.025
0.000
-0.025
-0.050
-0.075
0.2
0.3
0.4
0.5
0.6
SNP effect on exposure
.p_Bacteroidetes.c_Bacteroidia.o_Bacteroidales.f_Bacteroida

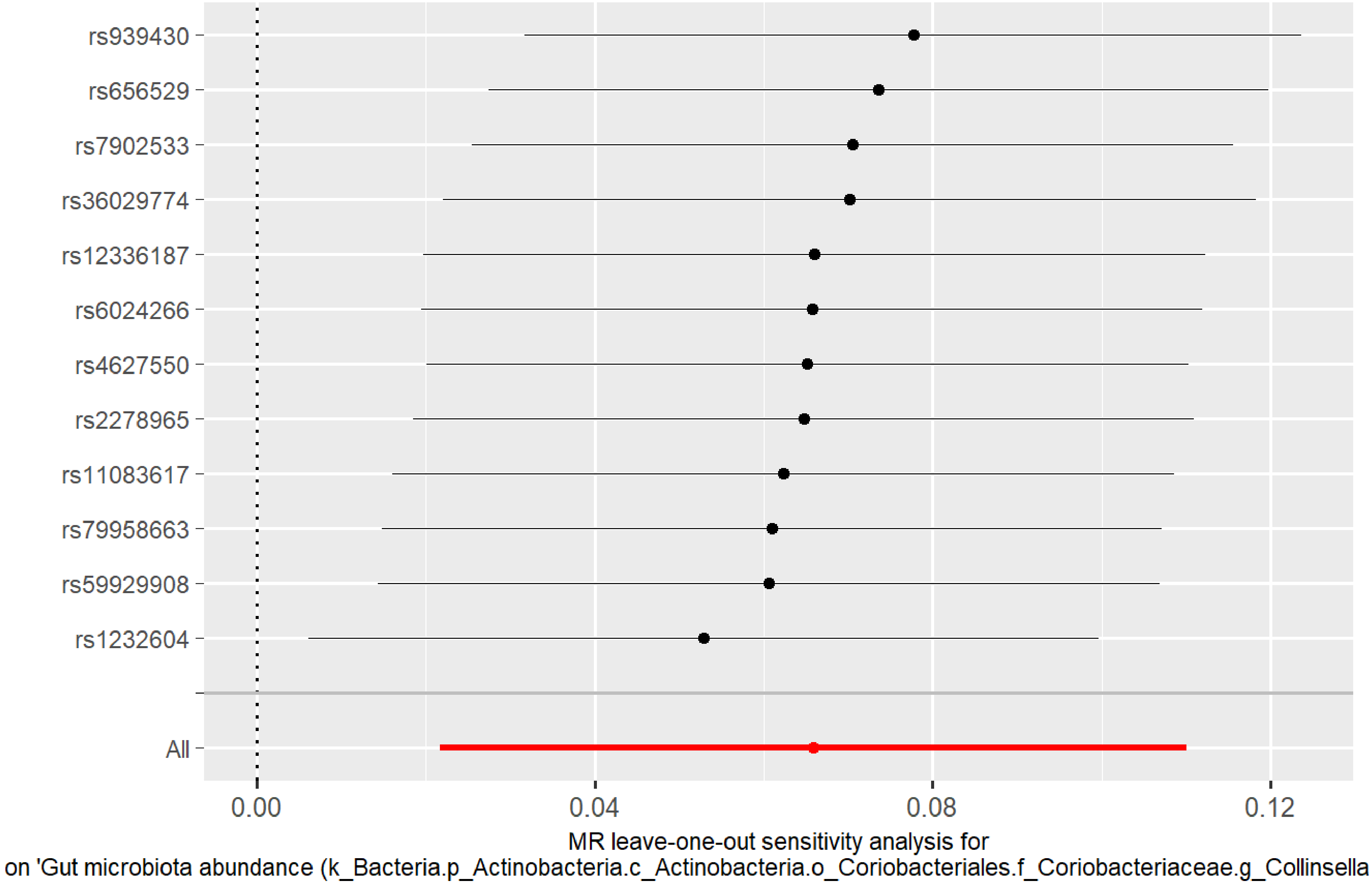
rs939430
rs656529
rs7902533
rs36029774
rs12336187
rs6024266
rs4627550
rs2278965
rs11083617
rs79958663
rs59929908
rs1232604
All
0.00
0.04
0.08
0.12
MR leave-one-out sensitivity analysis for
on 'Gut microbiota abundance (k_Bacteria.p_Actinobacteria.c_Actinobacteria.o_Coriobacteriales.f_Coriobacteriaceae.g_Collinsella

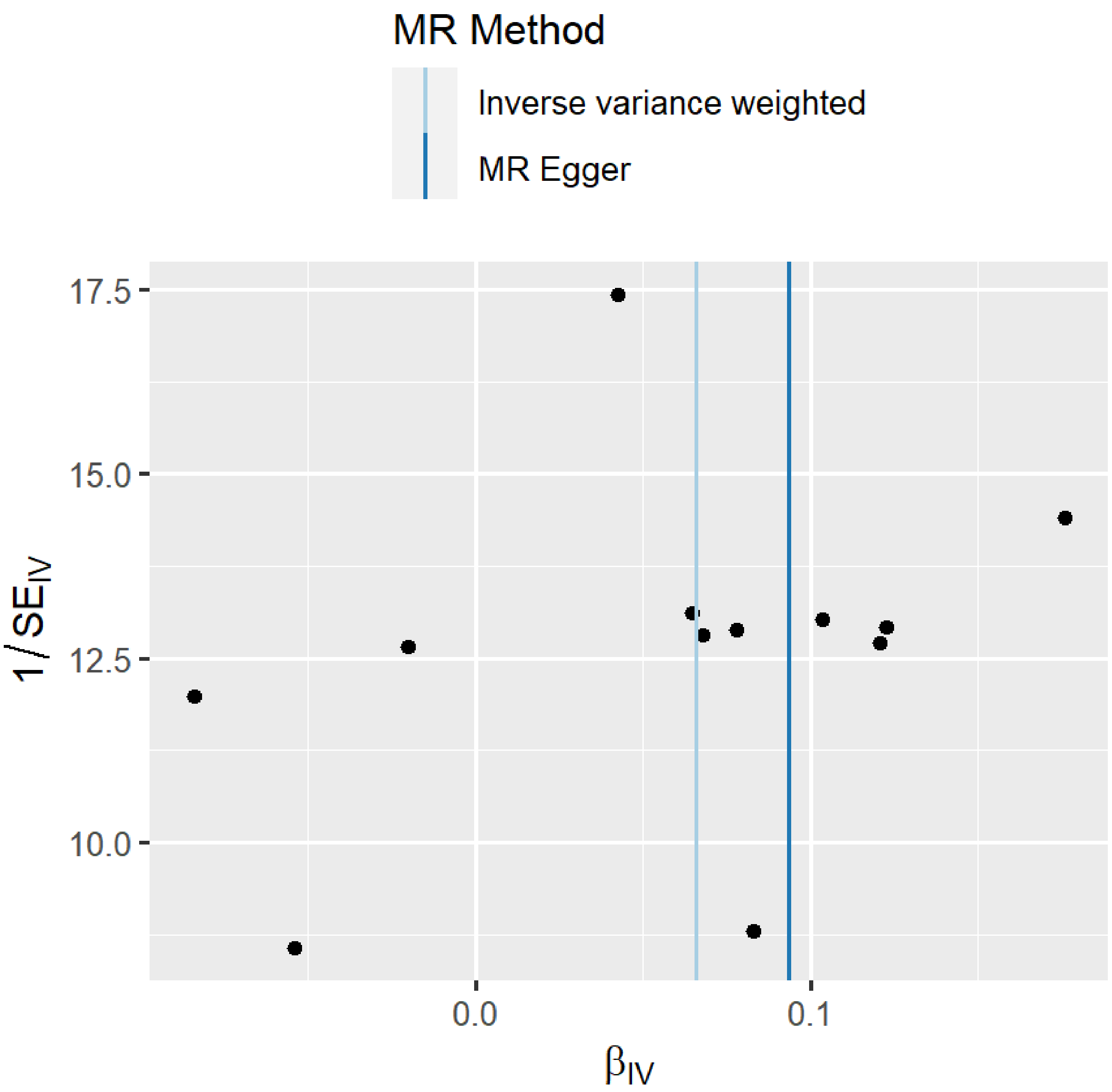
MR Method
Inverse variance weighted
MR Egger
17.5
15.0
12.5
10.0
0.0
0.1
1/SE_IV
β_IV

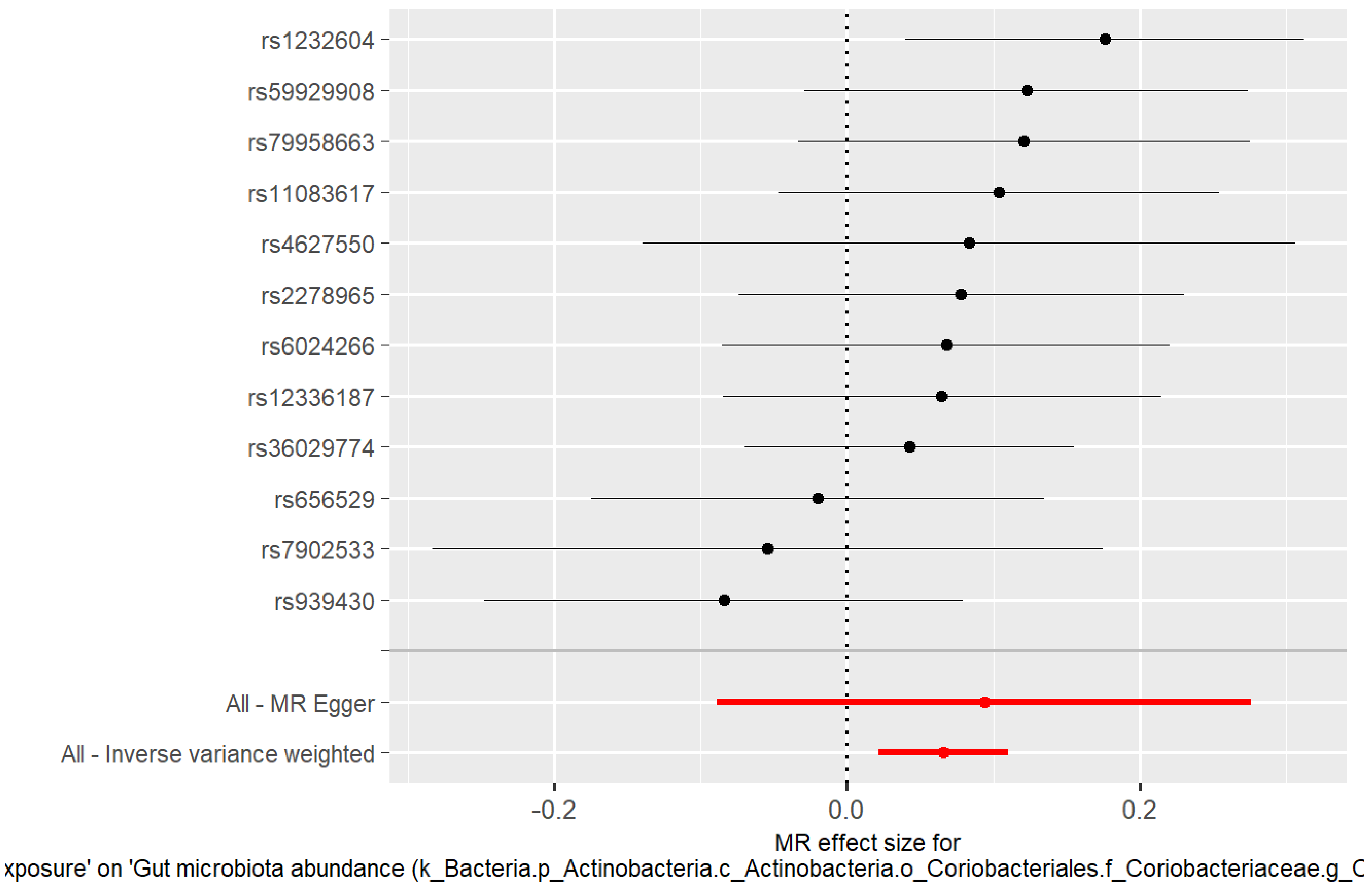
rs1232604
rs59929908
rs79958663
rs11083617
rs4627550
rs2278965
rs6024266
rs12336187
rs36029774
rs656529
rs7902533
rs939430
All - MR Egger
All - Inverse variance weighted
-0.2
0.0
0.2
MR effect size for
xposure' on 'Gut microbiota abundance (k_Bacteria.p_Actinobacteria.c_Actinobacteria.o_Coriobacteriales.f_Coriobacteriaceae.g_C

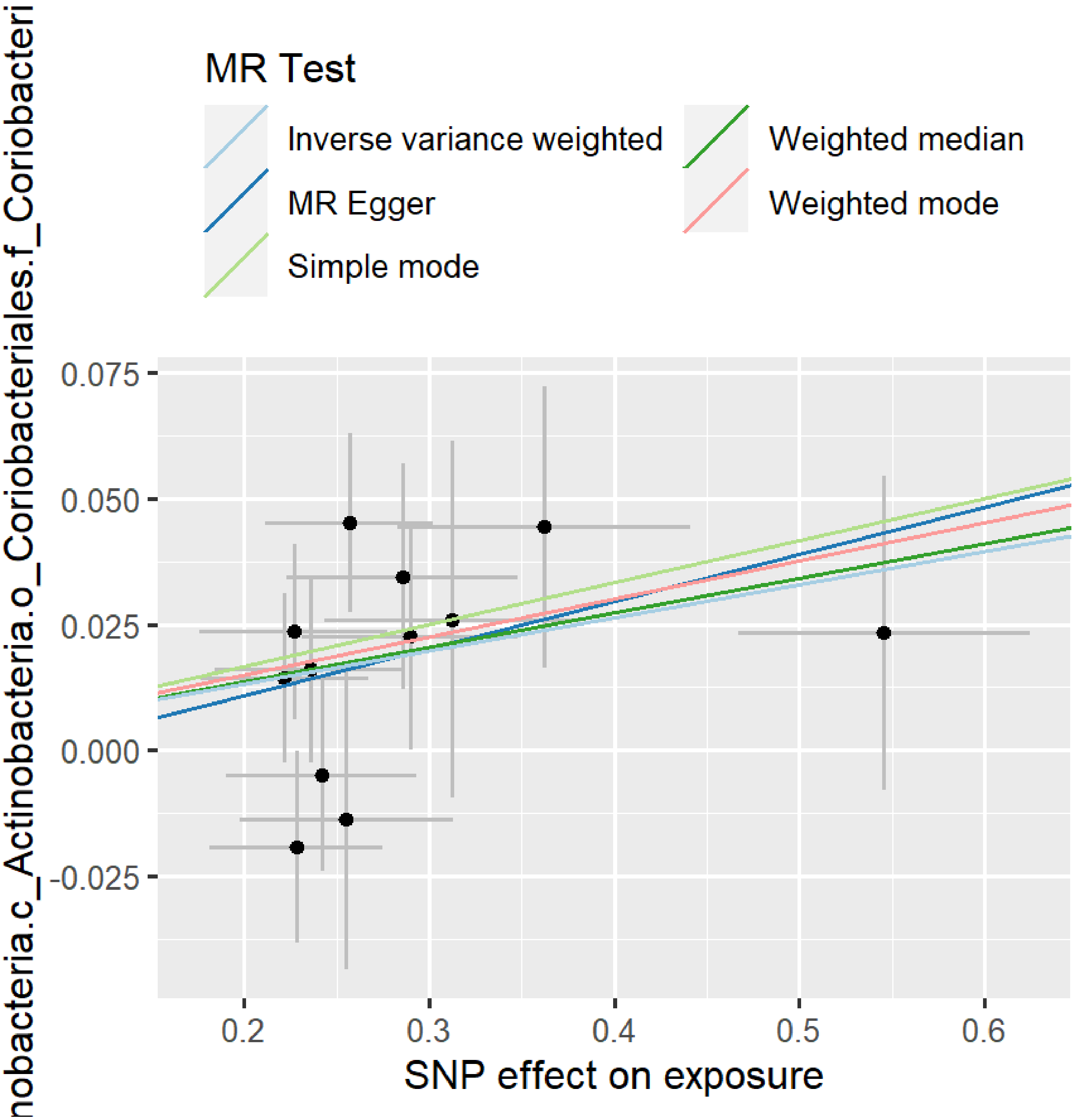

MR Test
Inverse variance weighted
MR Egger
Simple mode
Weighted median
Weighted mode
nobacteria.c_Actinobacteria.o_Coriobacteriales.f_Coriobacteri
0.075
0.050
0.025
0.000
-0.025
0.2
0.3
0.4
0.5
0.6
SNP effect on exposure

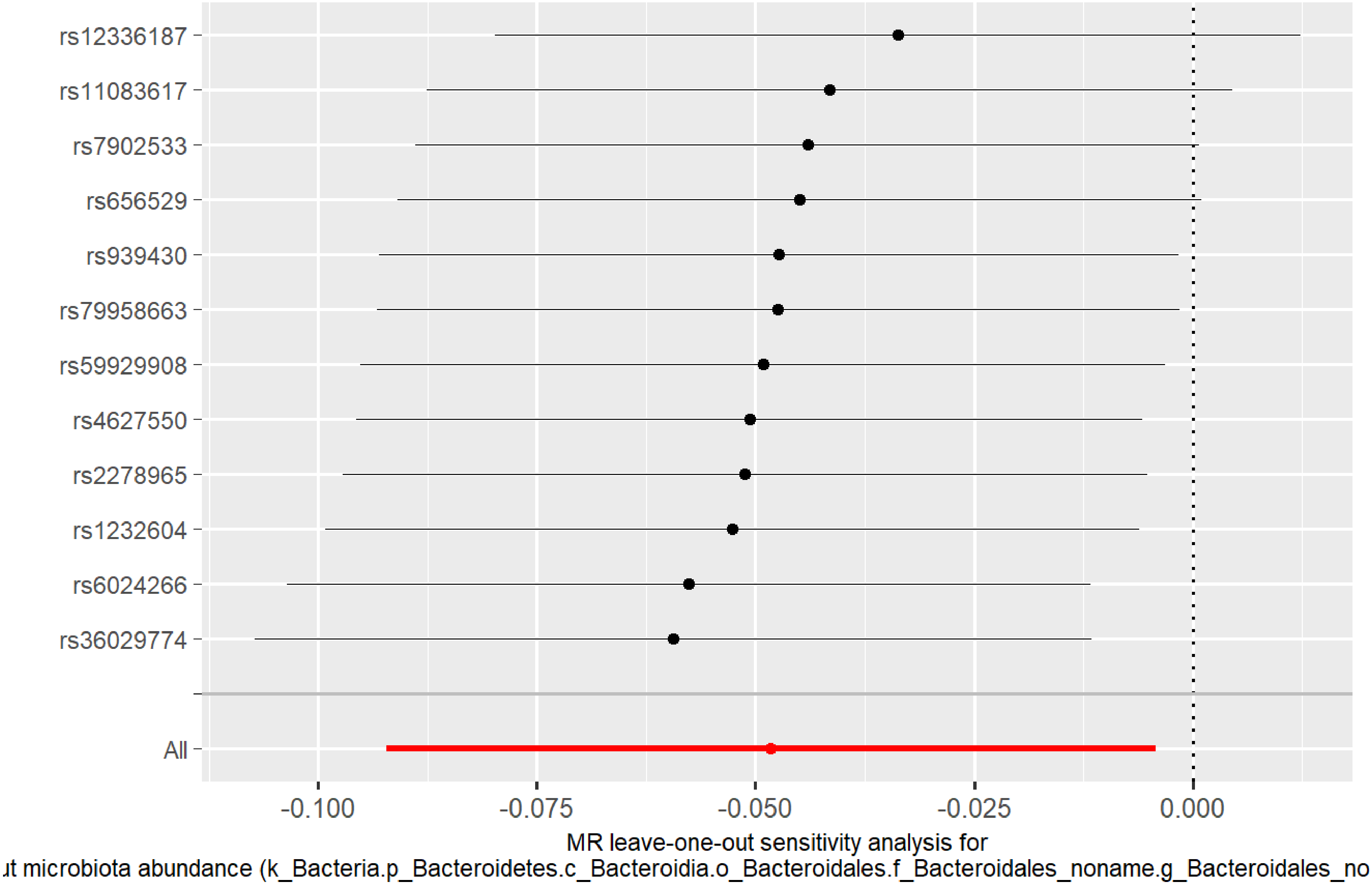
rs12336187
rs11083617
rs7902533
rs656529
rs939430
rs79958663
rs59929908
rs4627550
rs2278965
rs1232604
rs6024266
rs36029774
All
-0.100
-0.075
-0.050
-0.025
0.000
MR leave-one-out sensitivity analysis for
ıt microbiota abundance (k_Bacteria.p_Bacteroidetes.c_Bacteroidia.o_Bacteroidales.f_Bacteroidales_noname.g_Bacteroidales_no

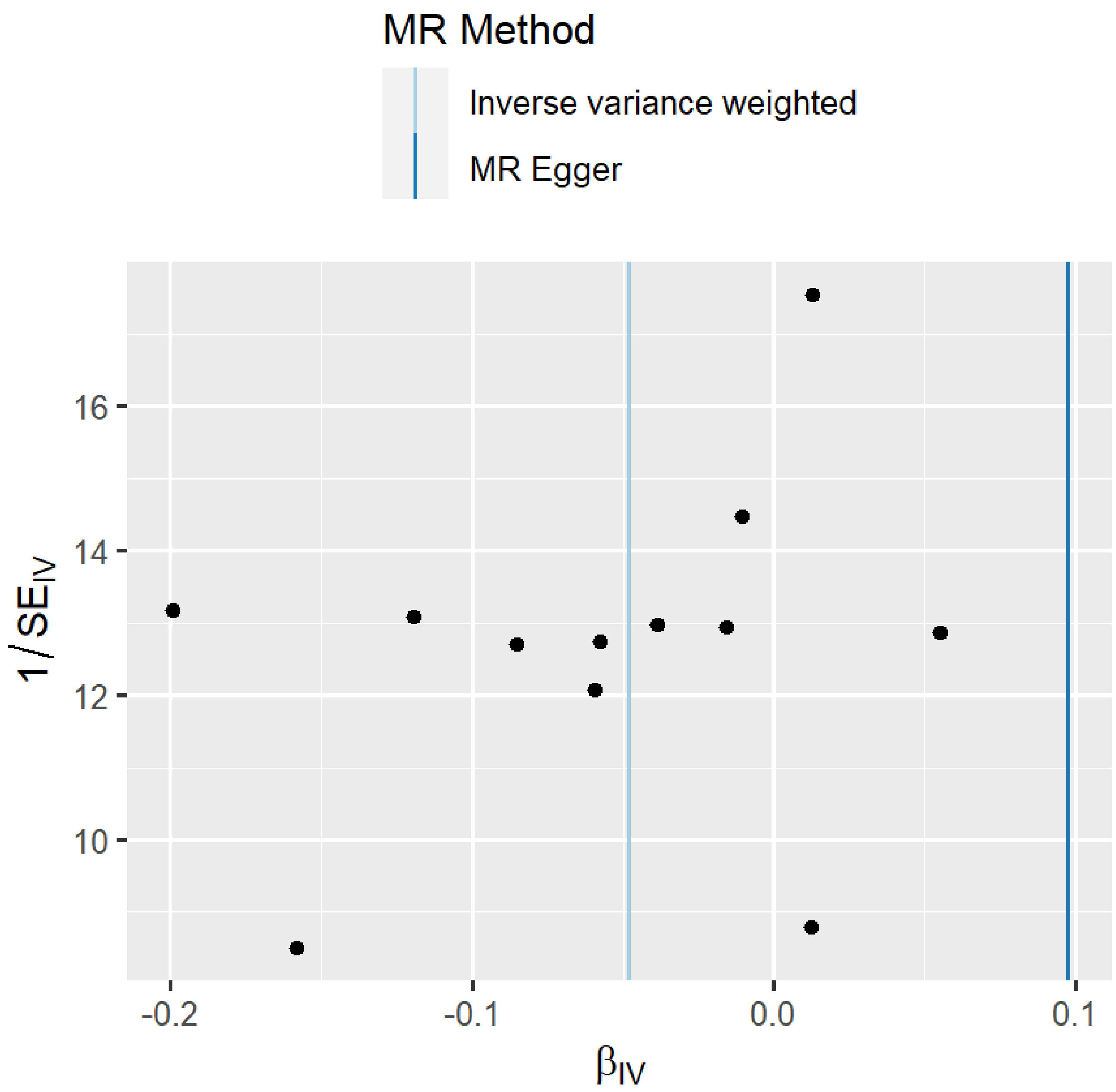
MR Method
Inverse variance weighted
MR Egger
16
14
12
10
1/SEIV
-0.2
-0.1
0.0
0.1
βIV

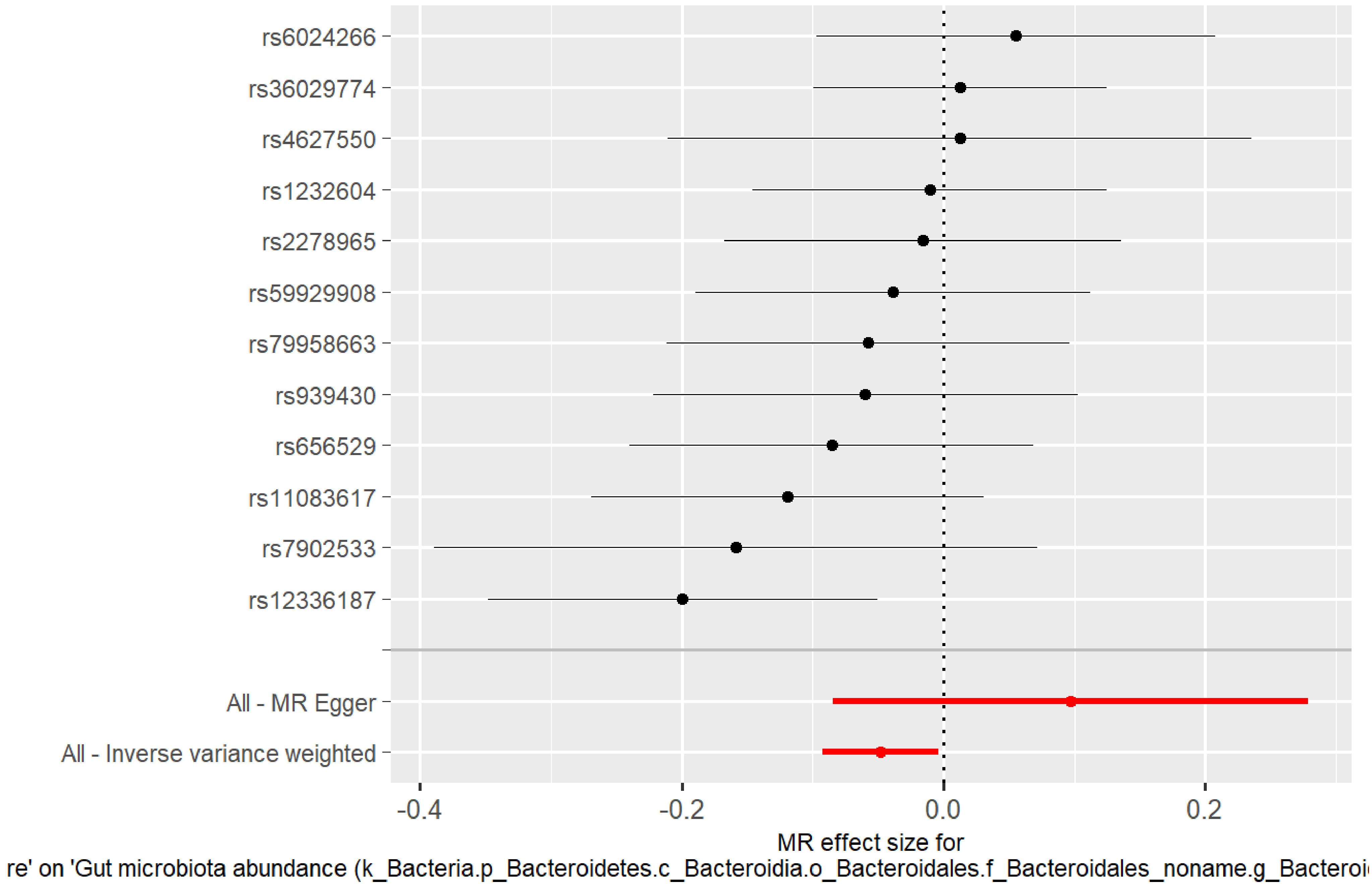
rs6024266
rs36029774
rs4627550
rs1232604
rs2278965
rs59929908
rs79958663
rs939430
rs656529
rs11083617
rs7902533
rs12336187
All - MR Egger
All - Inverse variance weighted
-0.4
-0.2
0.0
0.2
MR effect size for
re' on 'Gut microbiota abundance (k_Bacteria.p_Bacteroidetes.c_Bacteroidia.o_Bacteroidales.f_Bacteroidales_noname.g_Bacteroi

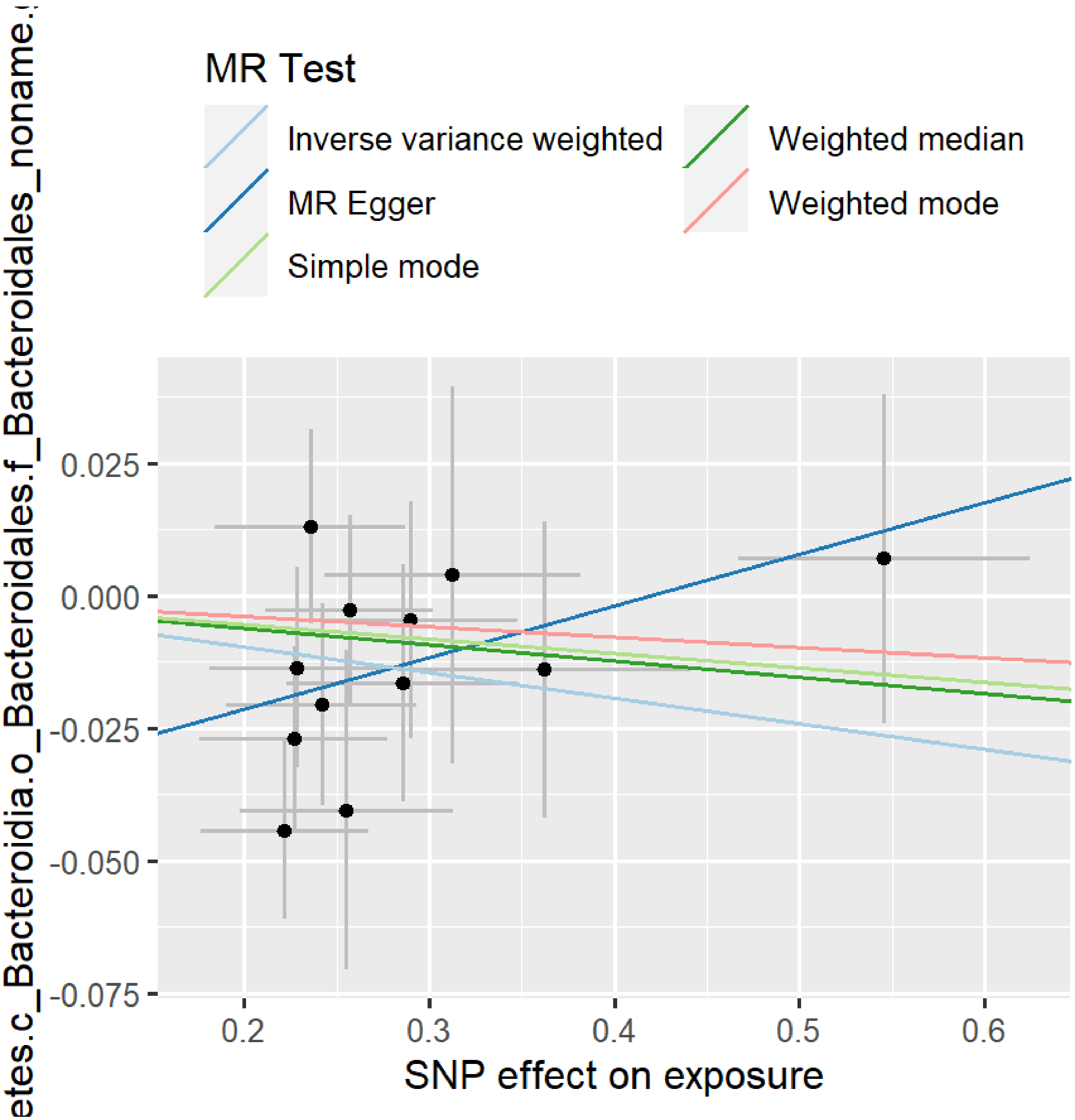
MR Test
Inverse variance weighted
MR Egger
Simple mode
Weighted median
Weighted mode
0.025
0.000
-0.025
-0.050
-0.075
0.2
0.3
0.4
0.5
0.6
SNP effect on exposure
etes.c_Bacteroidia.o_Bacteroidales.f_Bacteroidales_noname.

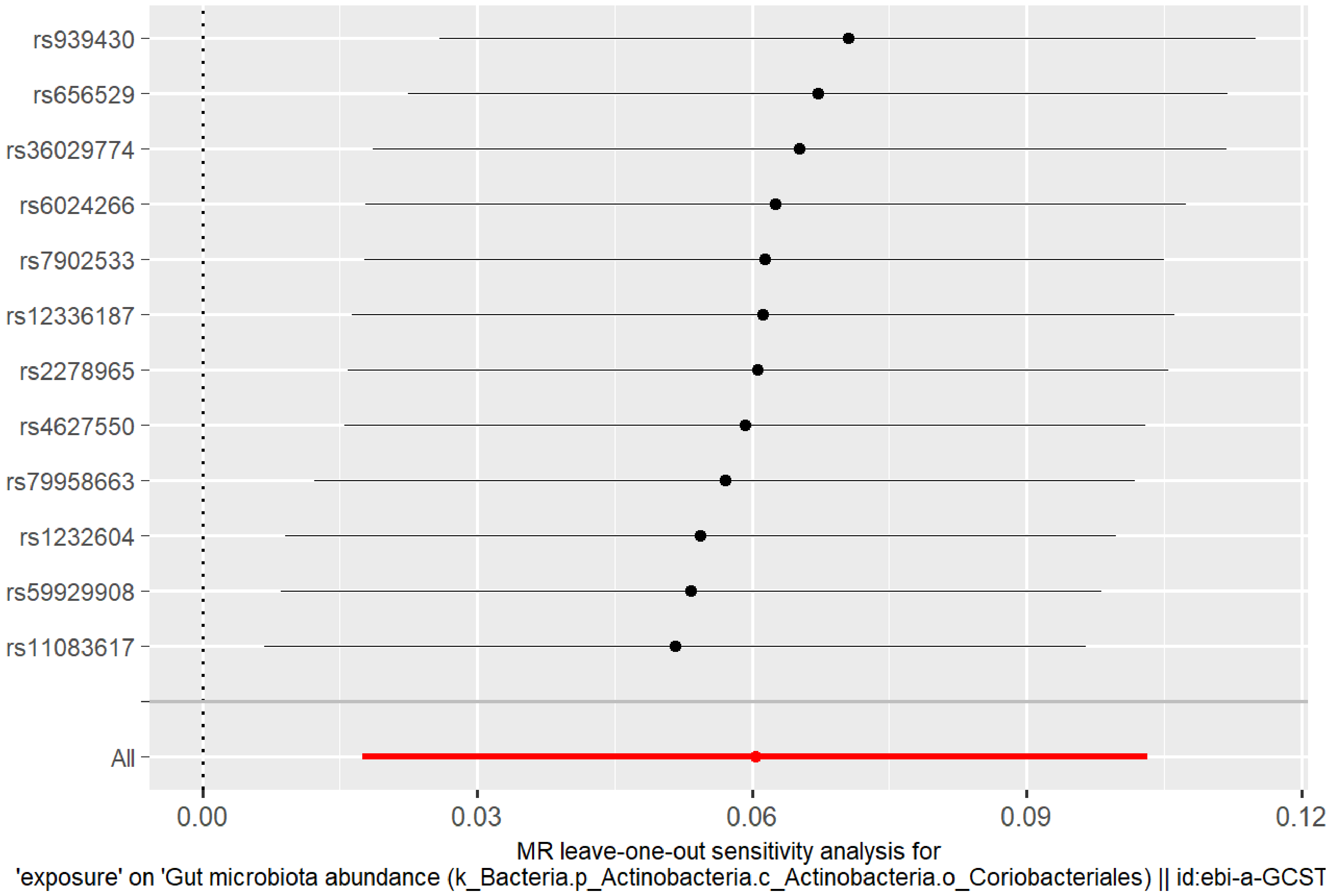

rs939430
rs656529
rs36029774
rs6024266
rs7902533
rs12336187
rs2278965
rs4627550
rs79958663
rs1232604
rs59929908
rs11083617
All
0.00
0.03
0.06
0.09
0.12
MR leave-one-out sensitivity analysis for
'exposure' on 'Gut microbiota abundance (k_Bacteria.p_Actinobacteria.c_Actinobacteria.o_Coriobacteriales) || id:ebi-a-GCST

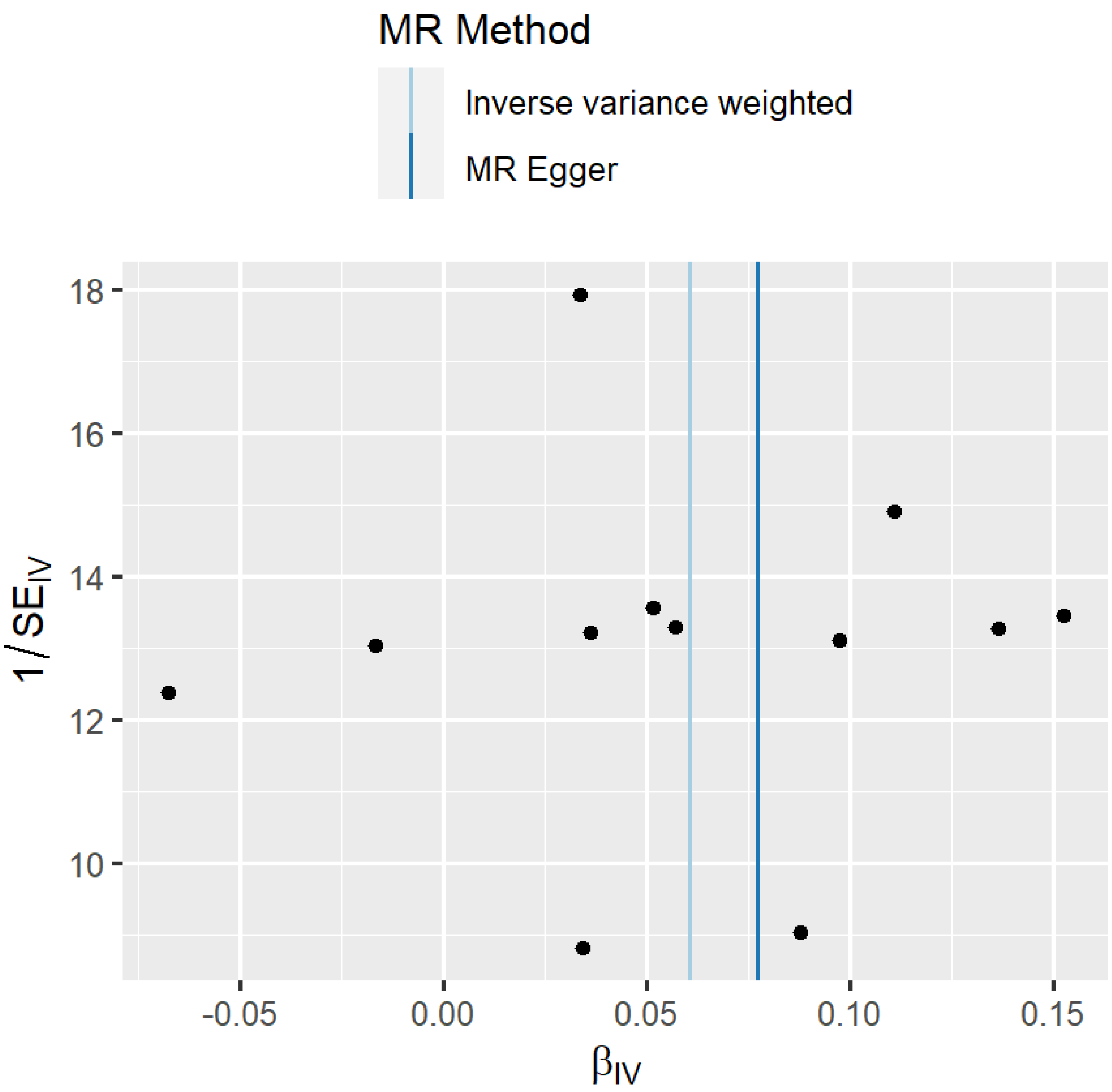
MR Method
Inverse variance weighted
MR Egger
18
16
14
12
10
1/SE_IV
-0.05
0.00
0.05
0.10
0.15
β_IV

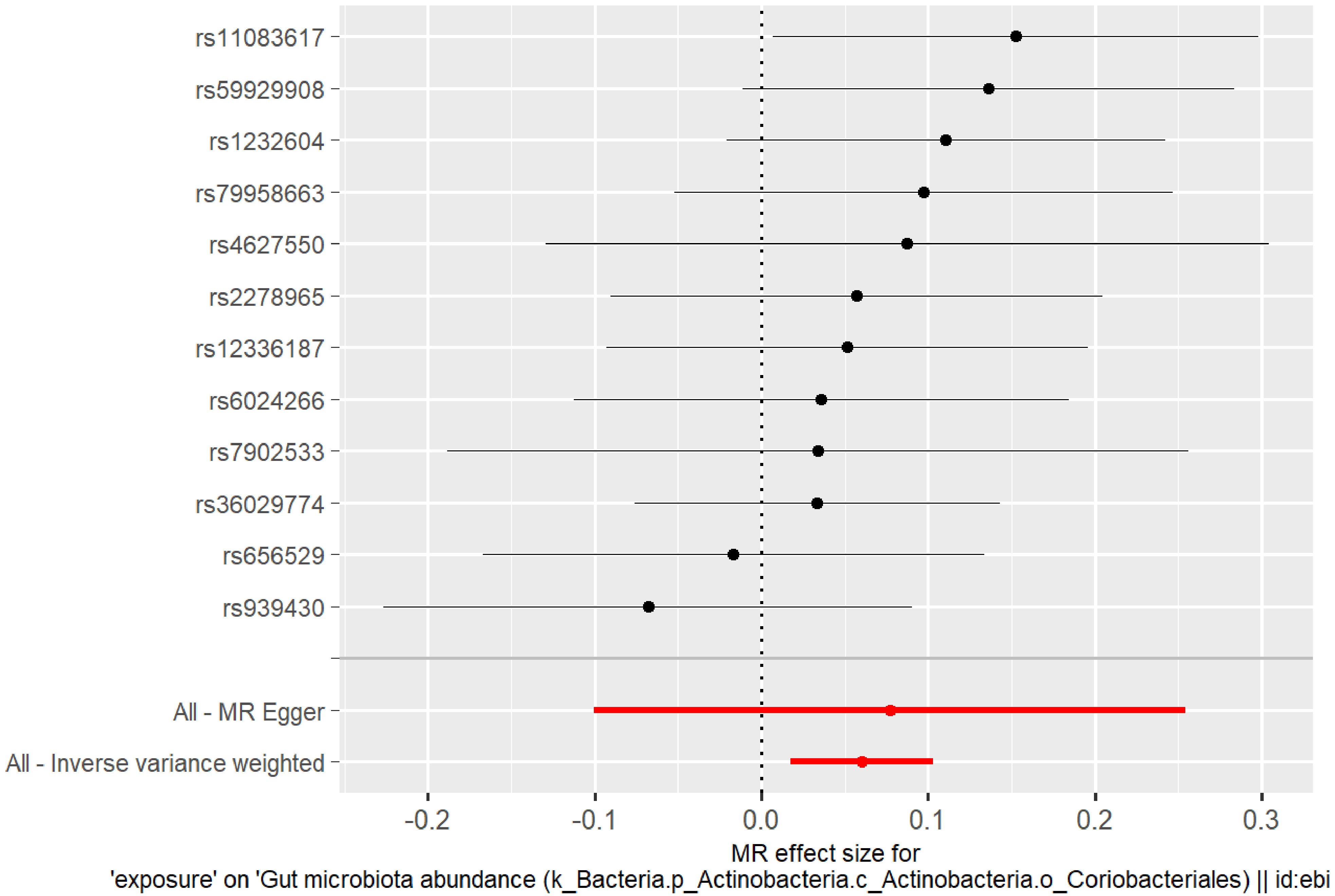
rs11083617
rs59929908
rs1232604
rs79958663
rs4627550
rs2278965
rs12336187
rs6024266
rs7902533
rs36029774
rs656529
rs939430
All - MR Egger
All - Inverse variance weighted
-0.2
-0.1
0.0
0.1
0.2
0.3
MR effect size for
'exposure' on 'Gut microbiota abundance (k_Bacteria.p_Actinobacteria.c_Actinobacteria.o_Coriobacteriales) || id:ebi

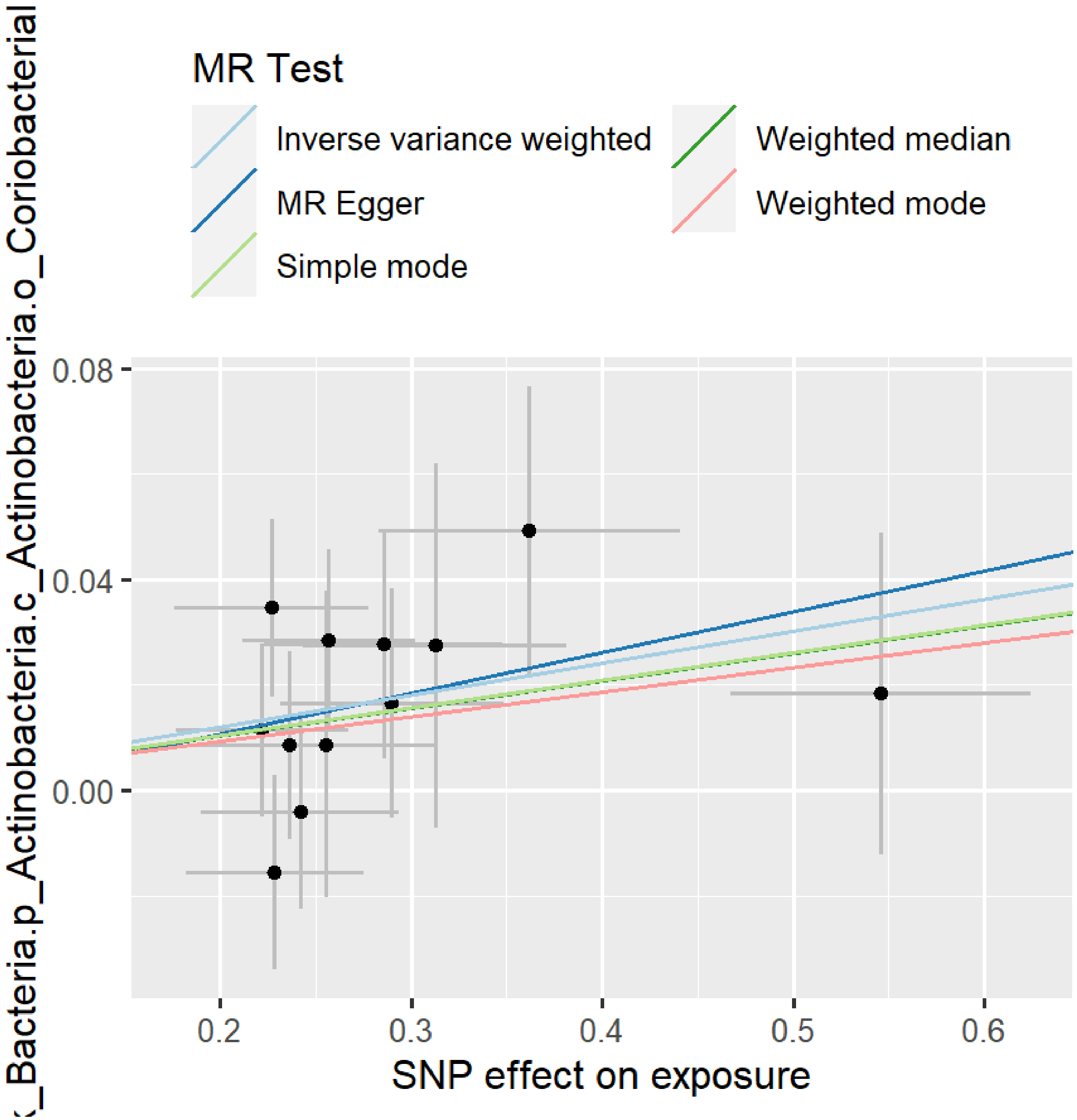
MR Test
Inverse variance weighted
MR Egger
Simple mode
Weighted median
Weighted mode
0.08
0.04
0.00
0.2
0.3
0.4
0.5
0.6
SNP effect on exposure
k_Bacteria.p_Actinobacteria.c_Actinobacteria.o_Coriobacterial

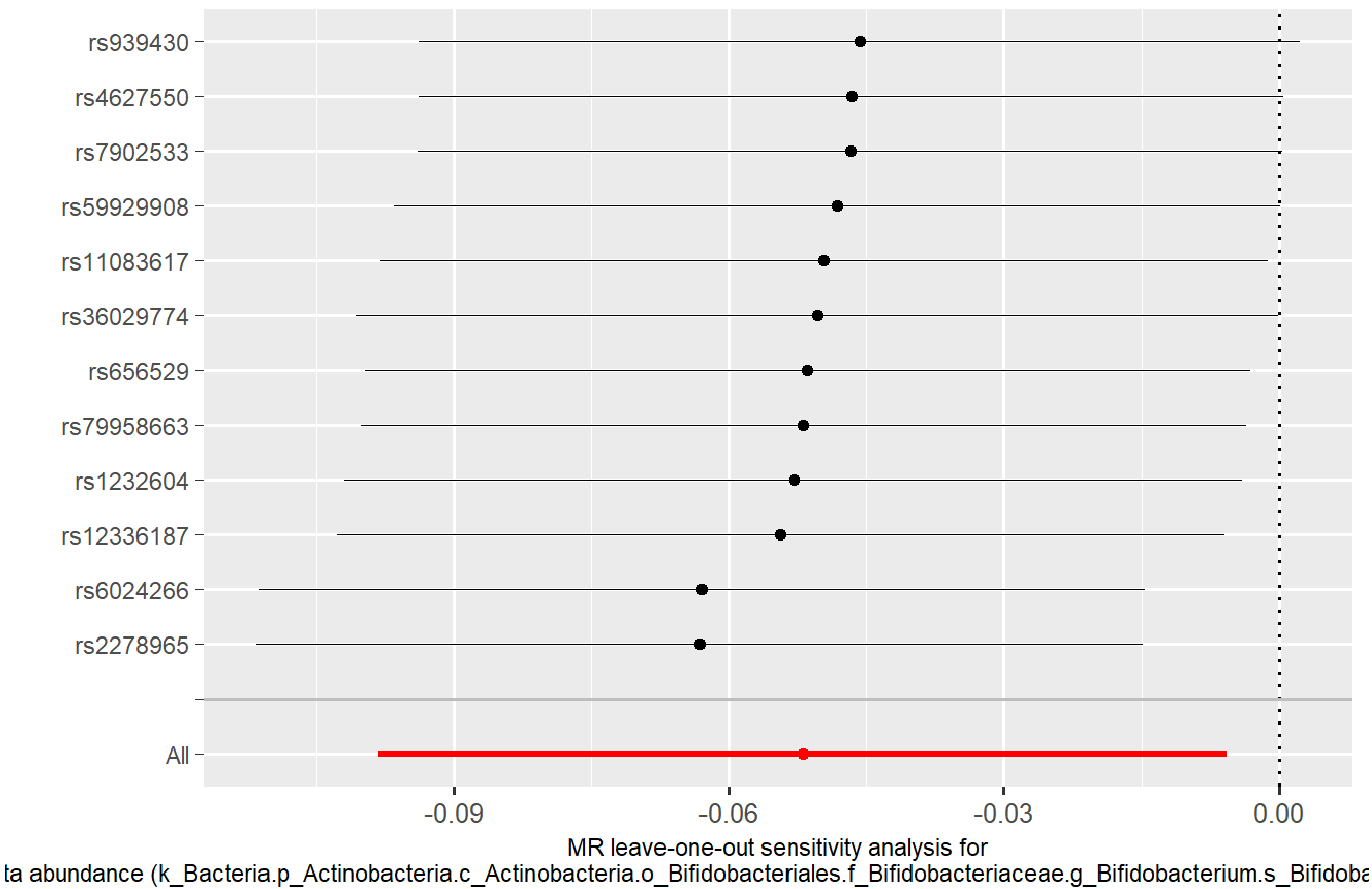
rs939430
rs4627550
rs7902533
rs59929908
rs11083617
rs36029774
rs656529
rs79958663
rs1232604
rs12336187
rs6024266
rs2278965
All
-0.09
-0.06
-0.03
0.00
MR leave-one-out sensitivity analysis for
ta abundance (k_Bacteria.p_Actinobacteria.c_Actinobacteria.o_Bifidobacteriales.f_Bifidobacteriaceae.g_Bifidobacterium.s_Bifidoba

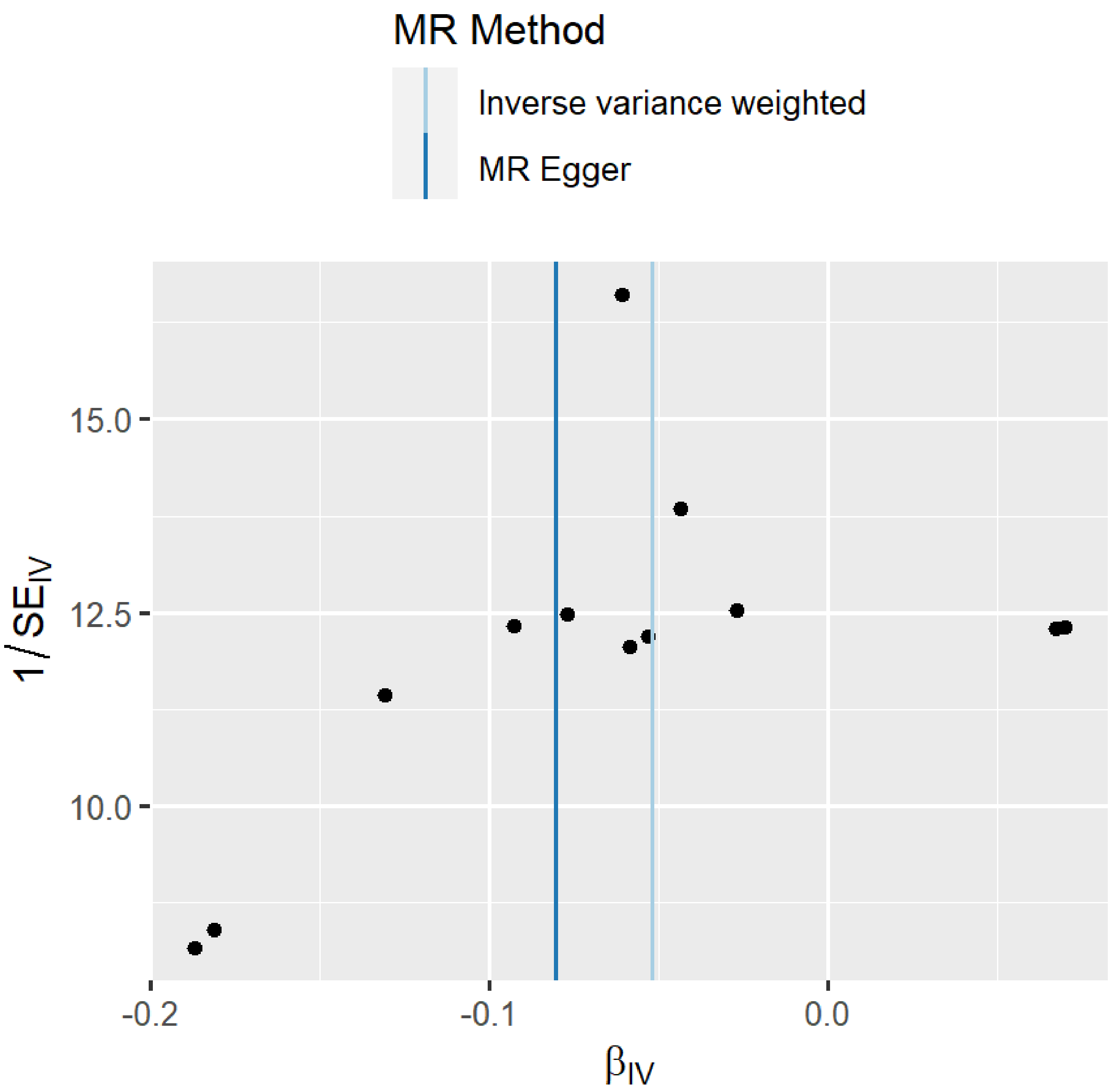
MR Method
Inverse variance weighted
MR Egger
15.0
12.5
10.0
$1/SE_{IV}$
-0.2
-0.1
0.0
β_{IV}

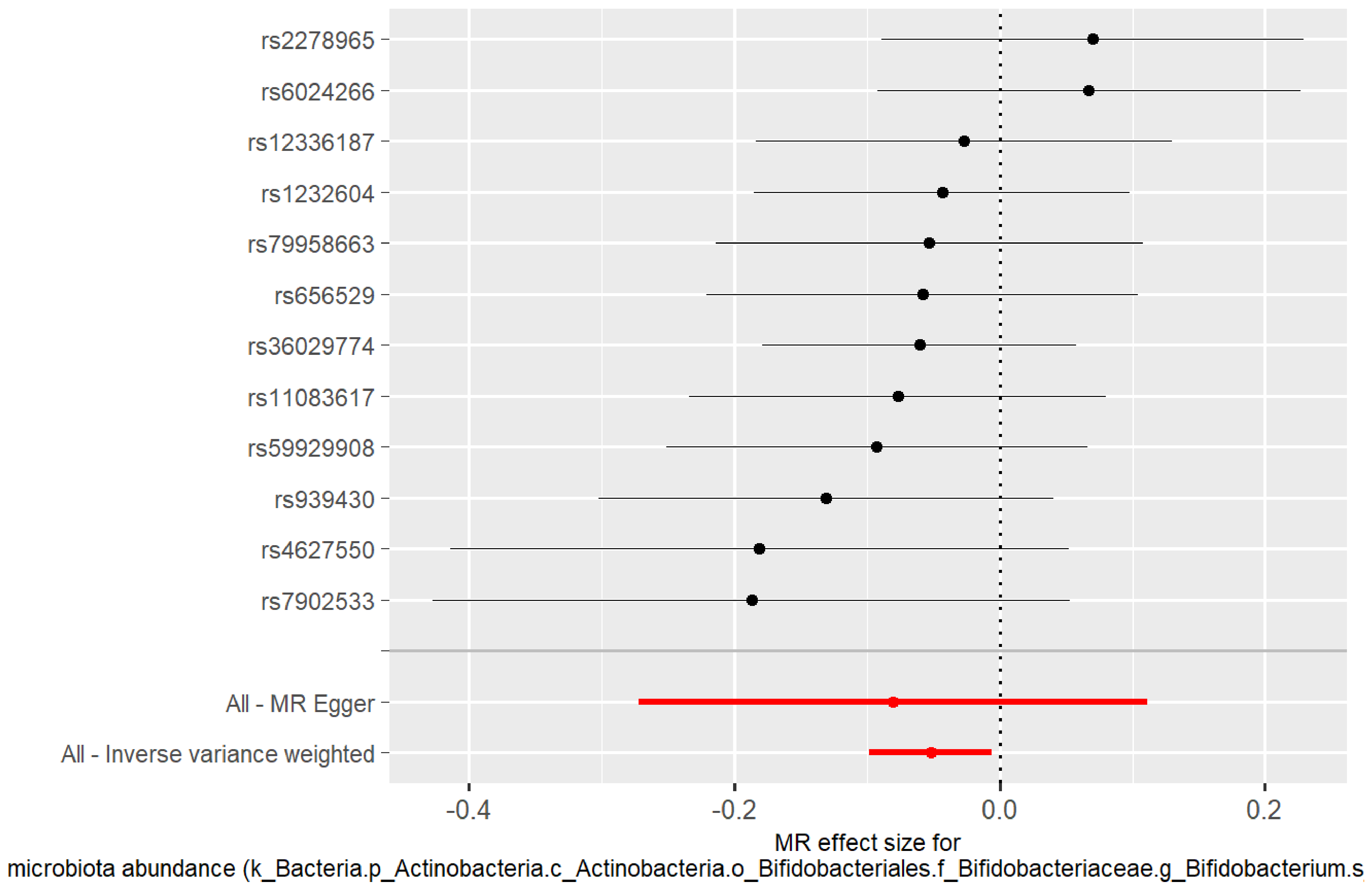
rs2278965
rs6024266
rs12336187
rs1232604
rs79958663
rs656529
rs36029774
rs11083617
rs59929908
rs939430
rs4627550
rs7902533
All - MR Egger
All - Inverse variance weighted
-0.4
-0.2
0.0
0.2
MR effect size for
microbiota abundance (k_Bacteria.p_Actinobacteria.c_Actinobacteria.o_Bifidobacteriales.f_Bifidobacteriaceae.g_Bifidobacterium.s

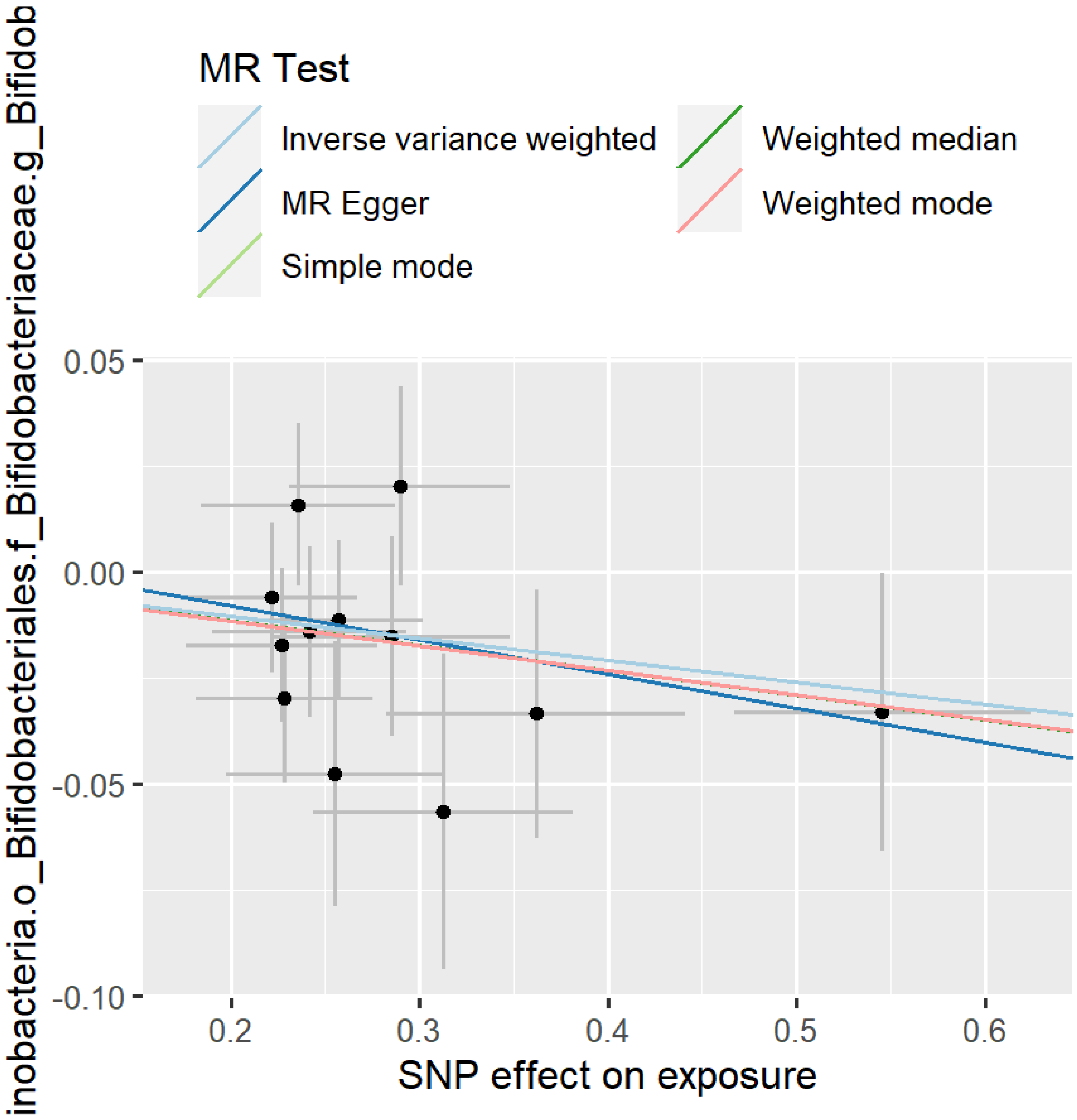
MR Test
Inverse variance weighted
MR Egger
Simple mode
Weighted median
Weighted mode
inobacteria.o_Bifidobacteriales.f_Bifidobacteriaceae.g_Bifidob
0.05
0.00
-0.05
-0.10
0.2
0.3
0.4
0.5
0.6
SNP effect on exposure

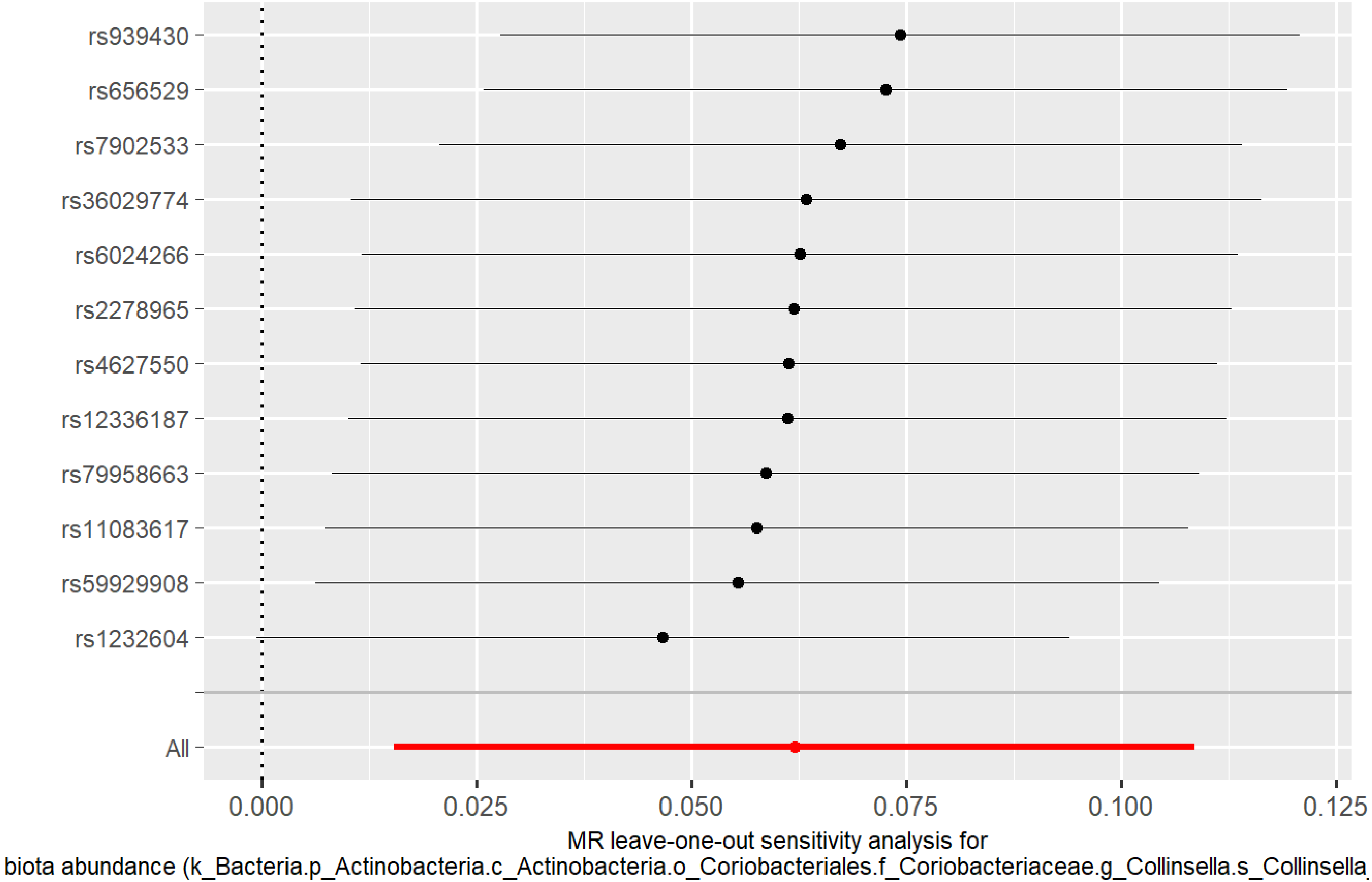
rs939430
rs656529
rs7902533
rs36029774
rs6024266
rs2278965
rs4627550
rs12336187
rs79958663
rs11083617
rs59929908
rs1232604
All
0.000
0.025
0.050
0.075
0.100
0.125
MR leave-one-out sensitivity analysis for
biota abundance (k_Bacteria.p_Actinobacteria.c_Actinobacteria.o_Coriobacteriales.f_Coriobacteriaceae.g_Collinsella.s_Collinsella_

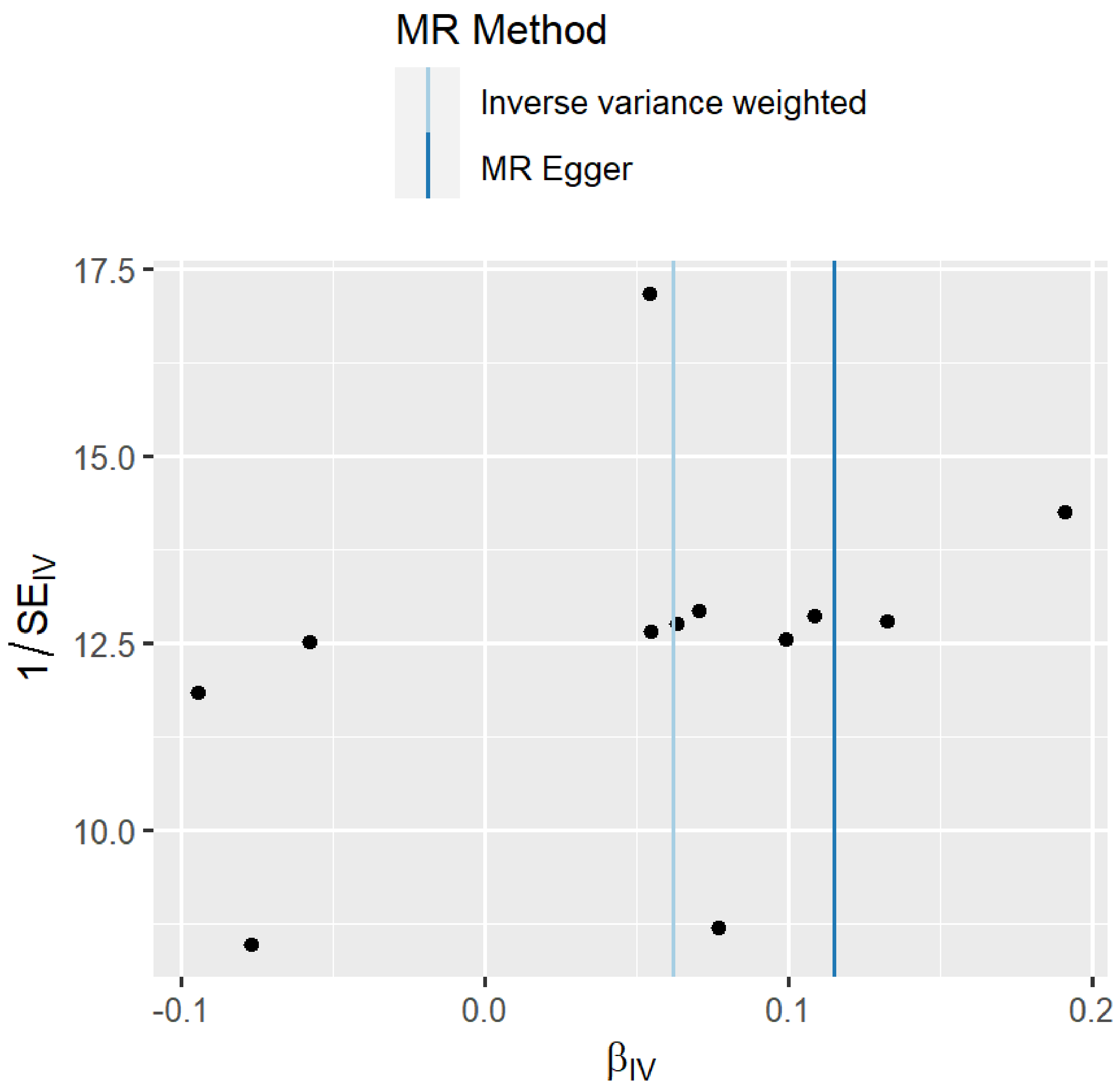
MR Method
Inverse variance weighted
MR Egger
17.5
15.0
12.5
10.0
-0.1
0.0
0.1
0.2
β_{IV}
$1/SE_{IV}$

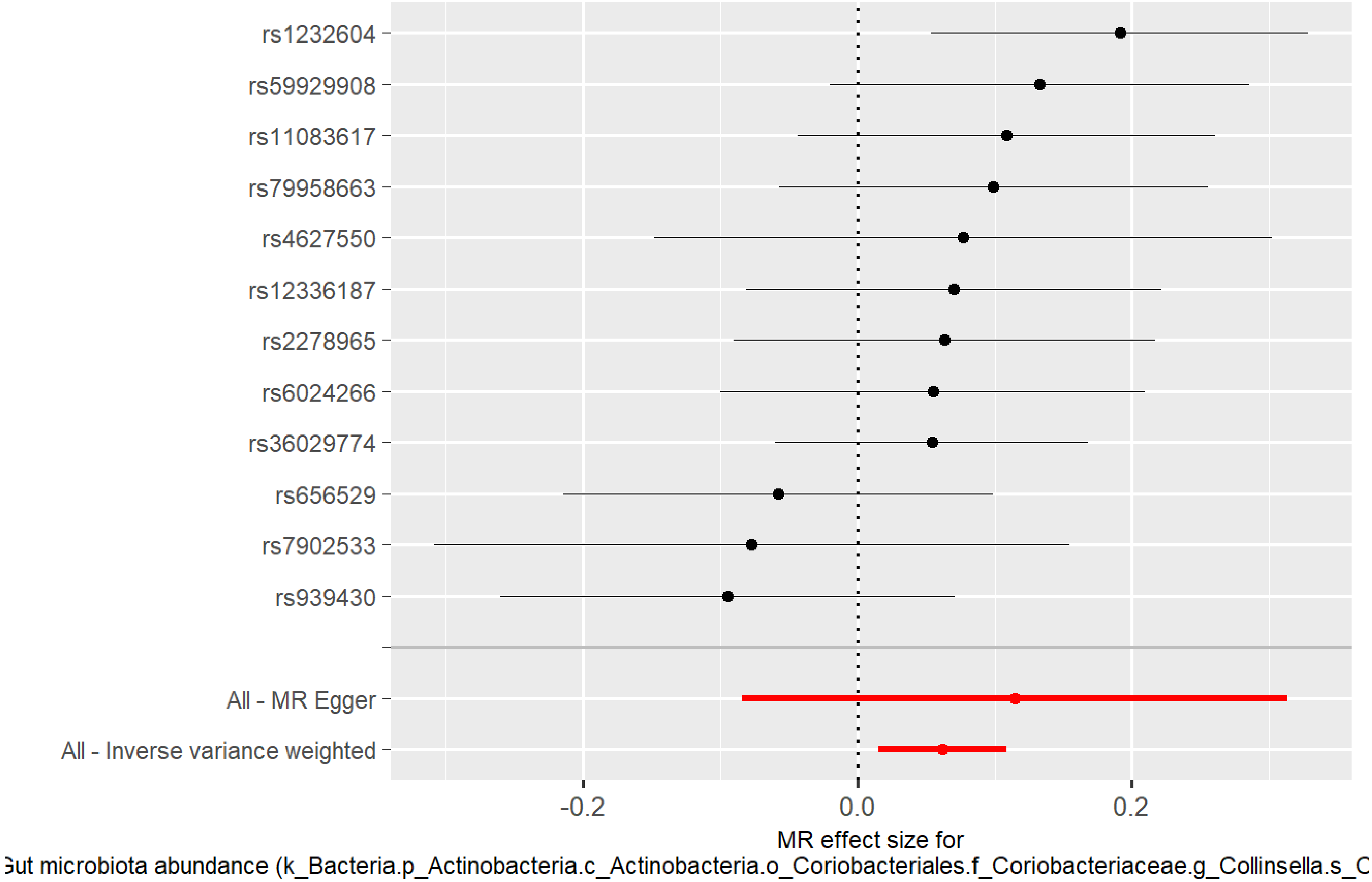
rs1232604
rs59929908
rs11083617
rs79958663
rs4627550
rs12336187
rs2278965
rs6024266
rs36029774
rs656529
rs7902533
rs939430
All - MR Egger
All - Inverse variance weighted
-0.2
0.0
0.2
MR effect size for
Gut microbiota abundance (k_Bacteria.p_Actinobacteria.c_Actinobacteria.o_Coriobacteriales.f_Coriobacteriaceae.g_Collinsella.s_C

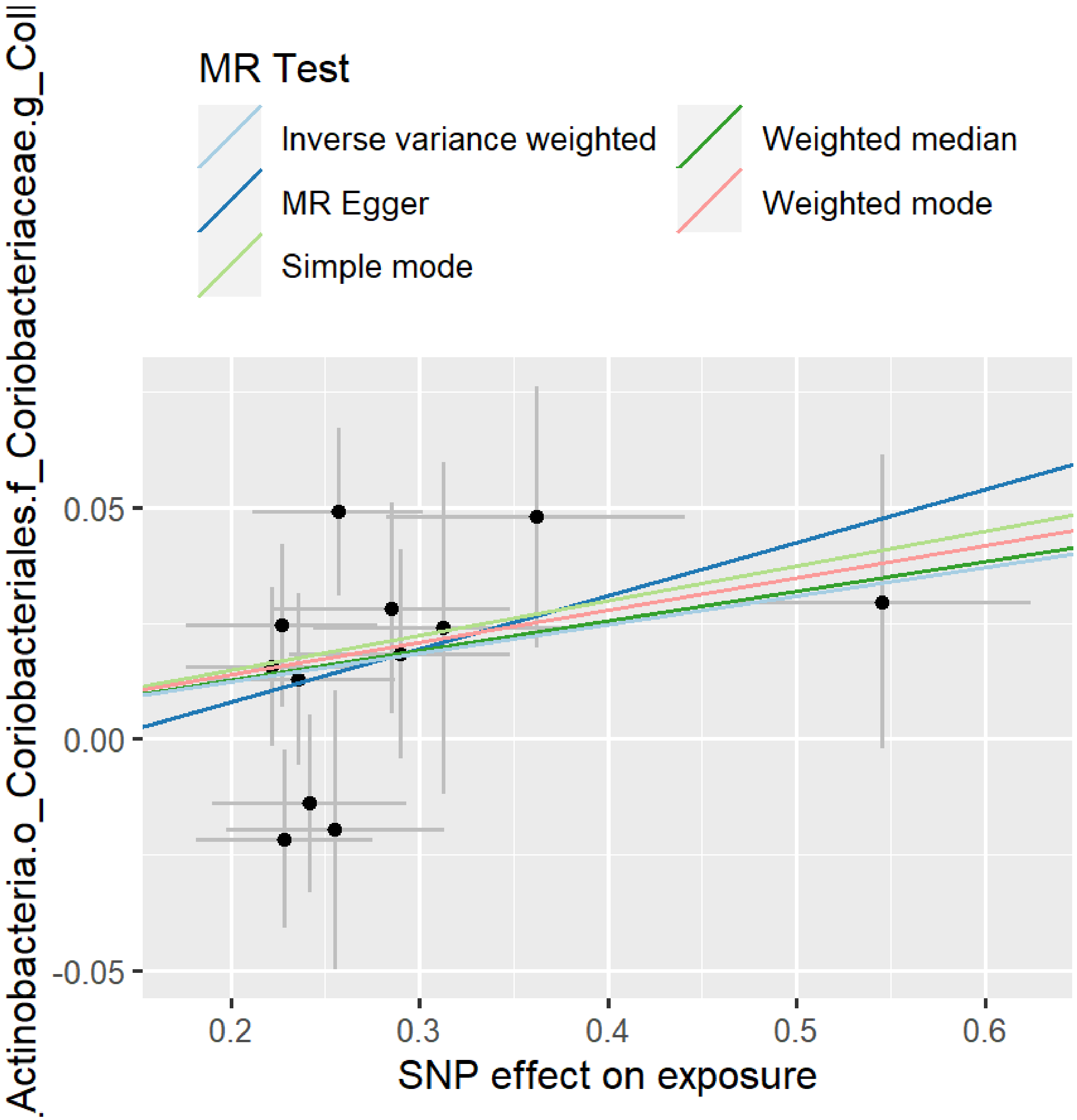
MR Test
Inverse variance weighted
MR Egger
Simple mode
Weighted median
Weighted mode
0.05
0.00
-0.05
0.2
0.3
0.4
0.5
0.6
SNP effect on exposure
Actinobacteria.o_Coriobacteriales.f_Coriobacteriaceae.g_Coll

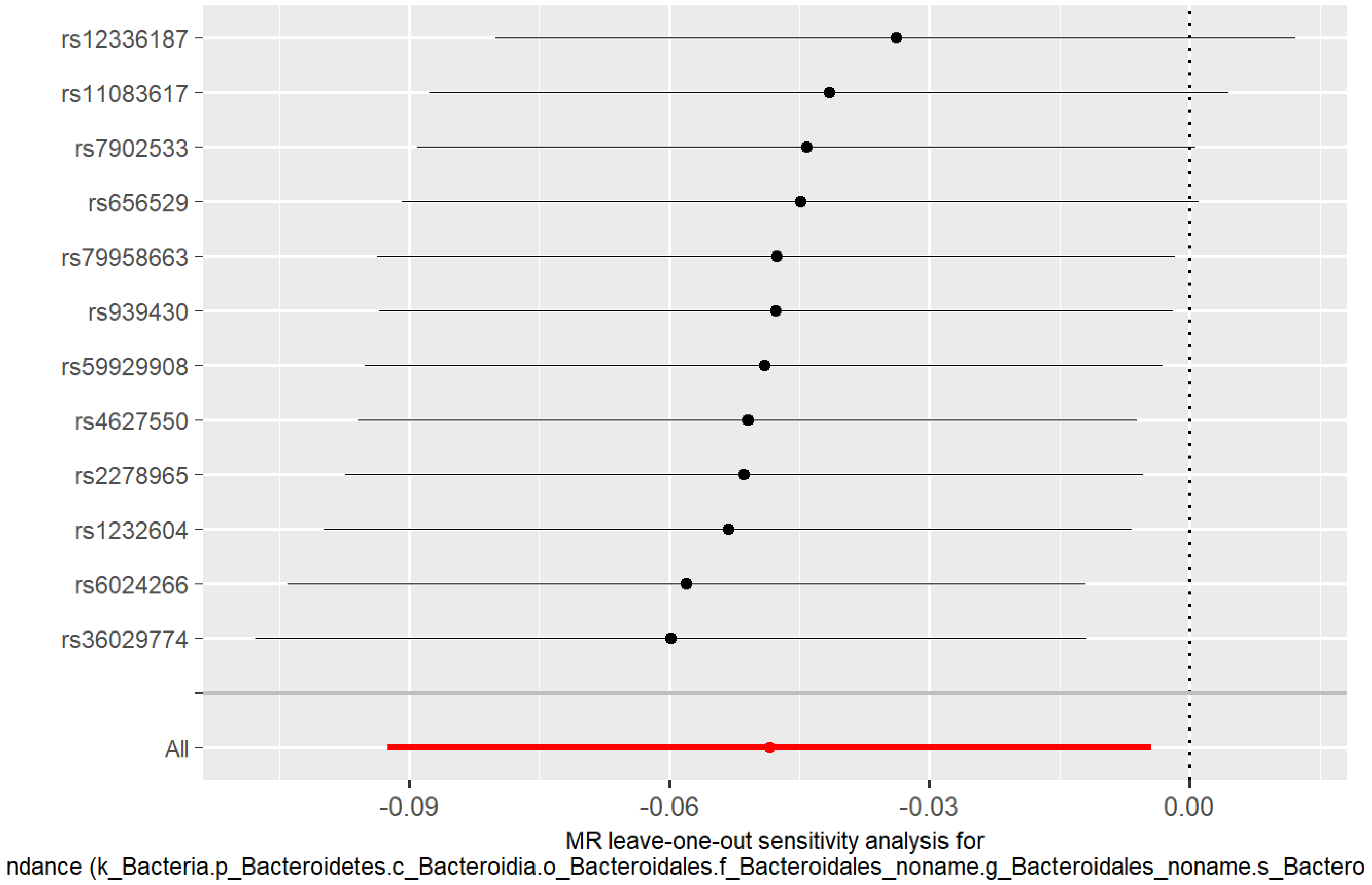
rs12336187
rs11083617
rs7902533
rs656529
rs79958663
rs939430
rs59929908
rs4627550
rs2278965
rs1232604
rs6024266
rs36029774
All
-0.09
-0.06
-0.03
0.00
MR leave-one-out sensitivity analysis for
ndance (k_Bacteria.p_Bacteroidetes.c_Bacteroidia.o_Bacteroidales.f_Bacteroidales_noname.g_Bacteroidales_noname.s_Bactero

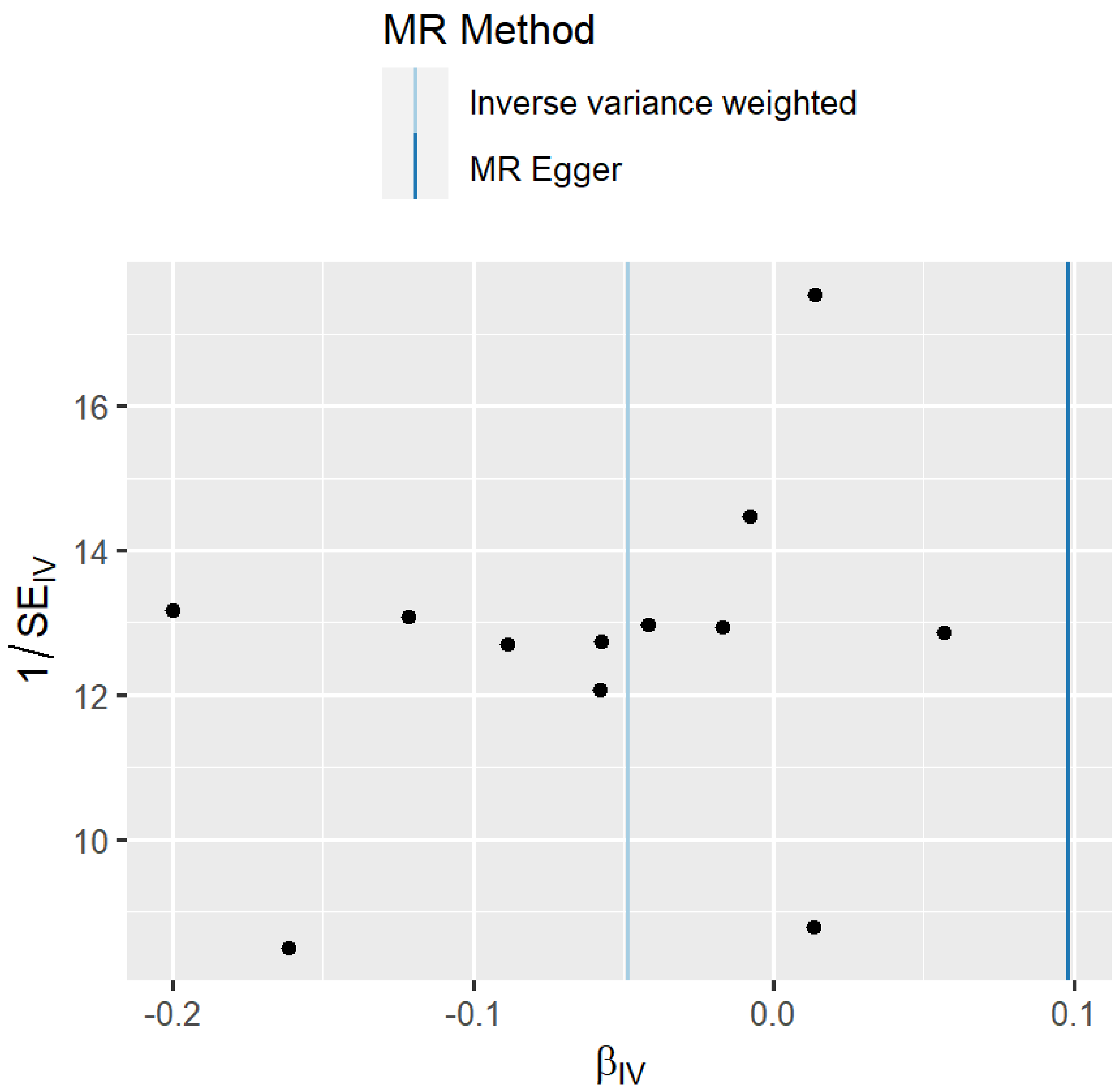
MR Method
Inverse variance weighted
MR Egger
16
14
12
10
1/SE_IV
-0.2
-0.1
0.0
0.1
β_IV

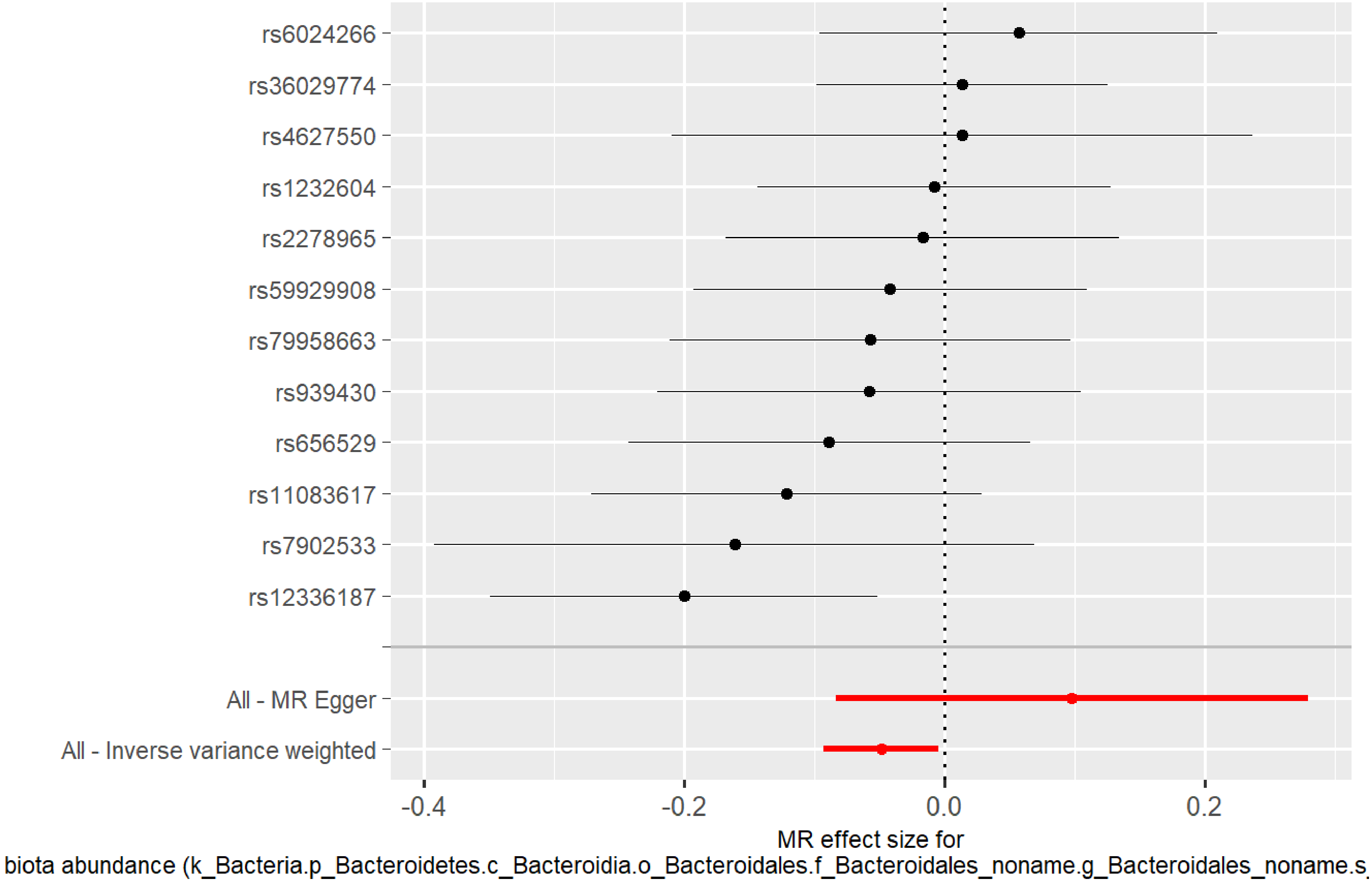

rs6024266
rs36029774
rs4627550
rs1232604
rs2278965
rs59929908
rs79958663
rs939430
rs656529
rs11083617
rs7902533
rs12336187
All - MR Egger
All - Inverse variance weighted
-0.4
-0.2
0.0
0.2
MR effect size for
biota abundance (k_Bacteria.p_Bacteroidetes.c_Bacteroidia.o_Bacteroidales.f_Bacteroidales_noname.g_Bacteroidales_noname.s_

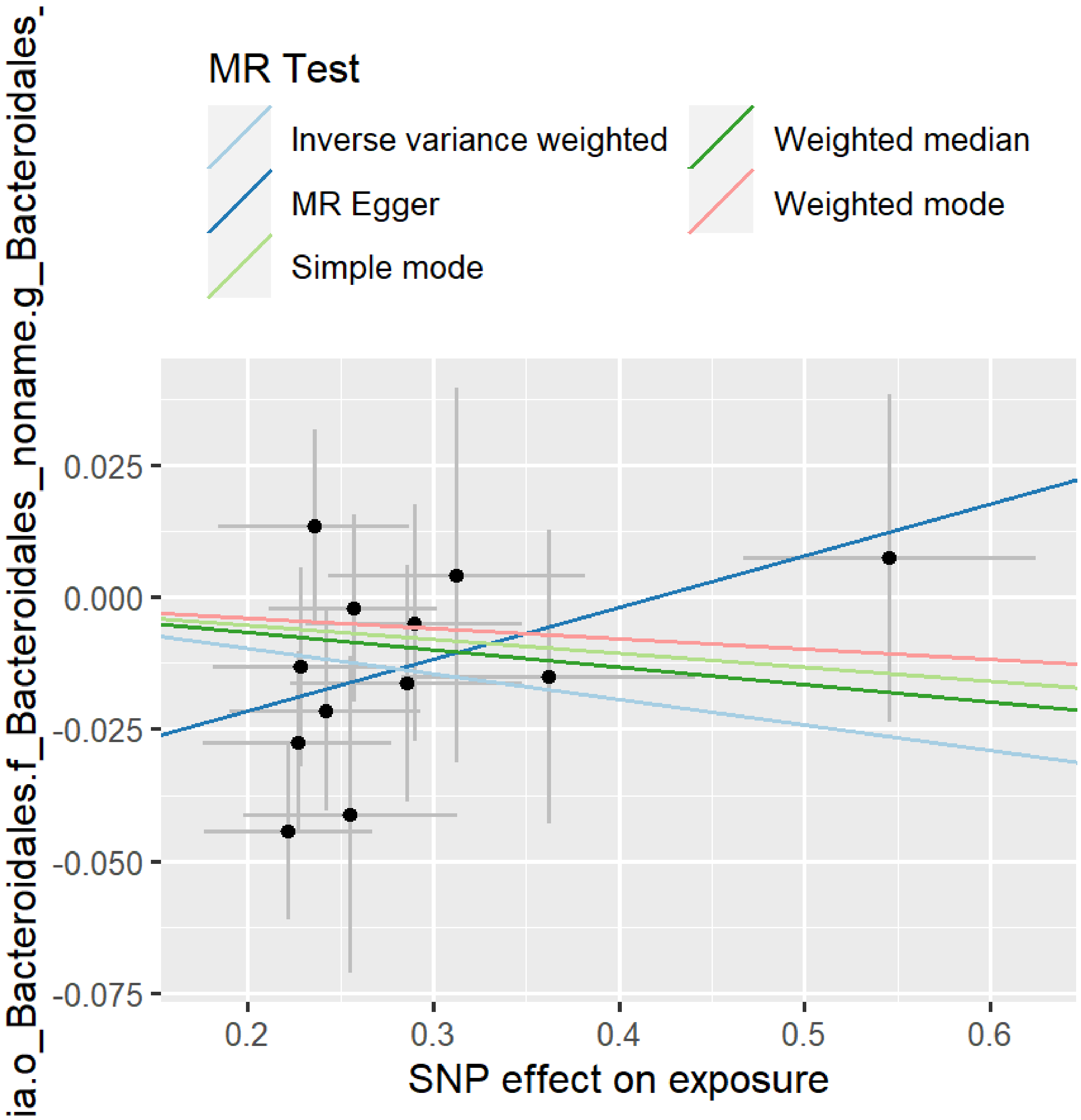
MR Test
Inverse variance weighted
MR Egger
Simple mode
Weighted median
Weighted mode
0.025
0.000
-0.025
-0.050
-0.075
0.2
0.3
0.4
0.5
0.6
SNP effect on exposure
ia.o_Bacteroidales.f_Bacteroidales_noname.g_Bacteroidales_

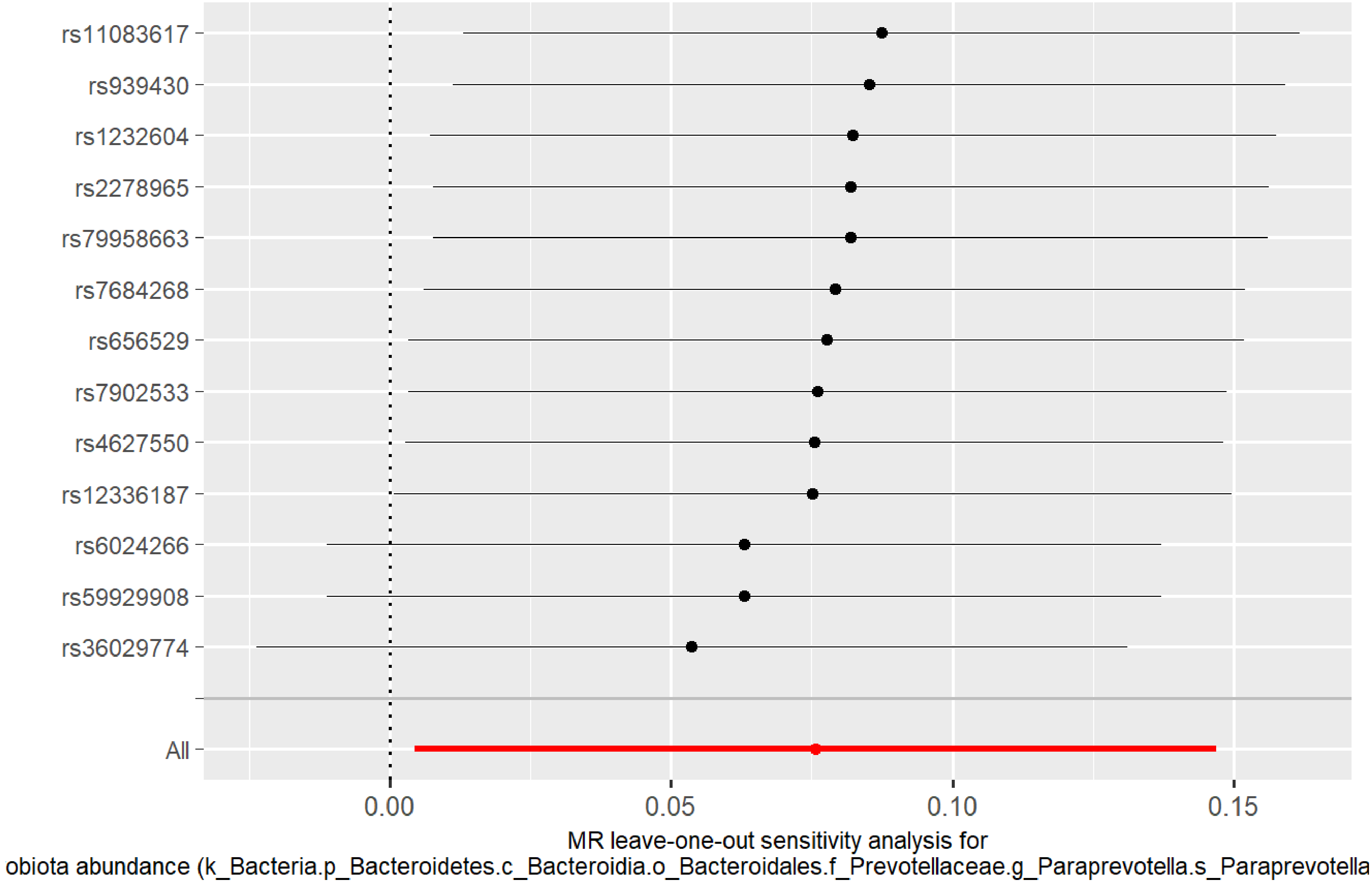
rs11083617
rs939430
rs1232604
rs2278965
rs79958663
rs7684268
rs656529
rs7902533
rs4627550
rs12336187
rs6024266
rs59929908
rs36029774
All
0.00
0.05
0.10
0.15
MR leave-one-out sensitivity analysis for
obiota abundance (k_Bacteria.p_Bacteroidetes.c_Bacteroidia.o_Bacteroidales.f_Prevotellaceae.g_Paraprevotella.s_Paraprevotella

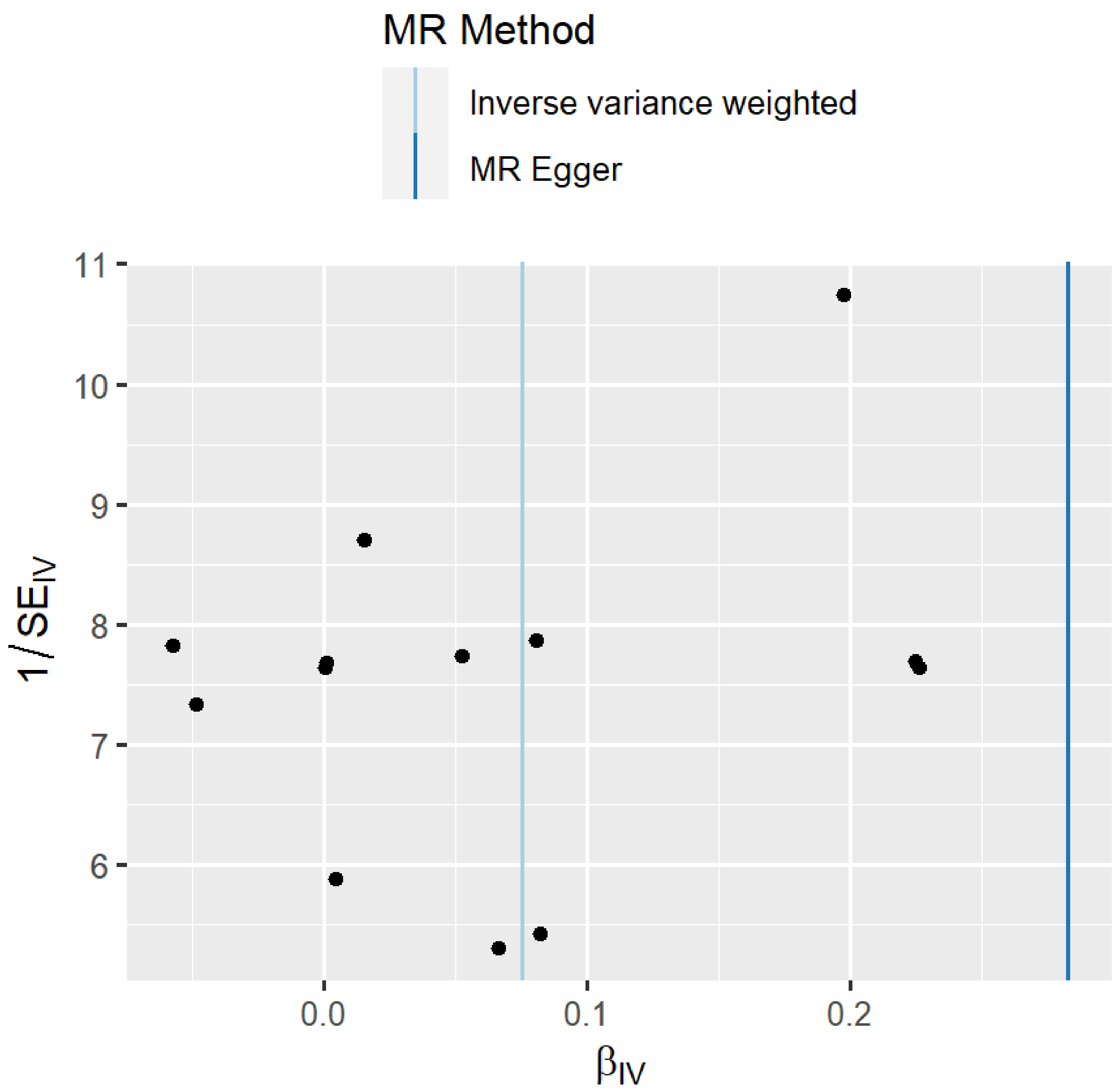
MR Method
Inverse variance weighted
MR Egger
11
10
9
8
7
6
1/SE_IV
0.0
0.1
0.2
β_IV

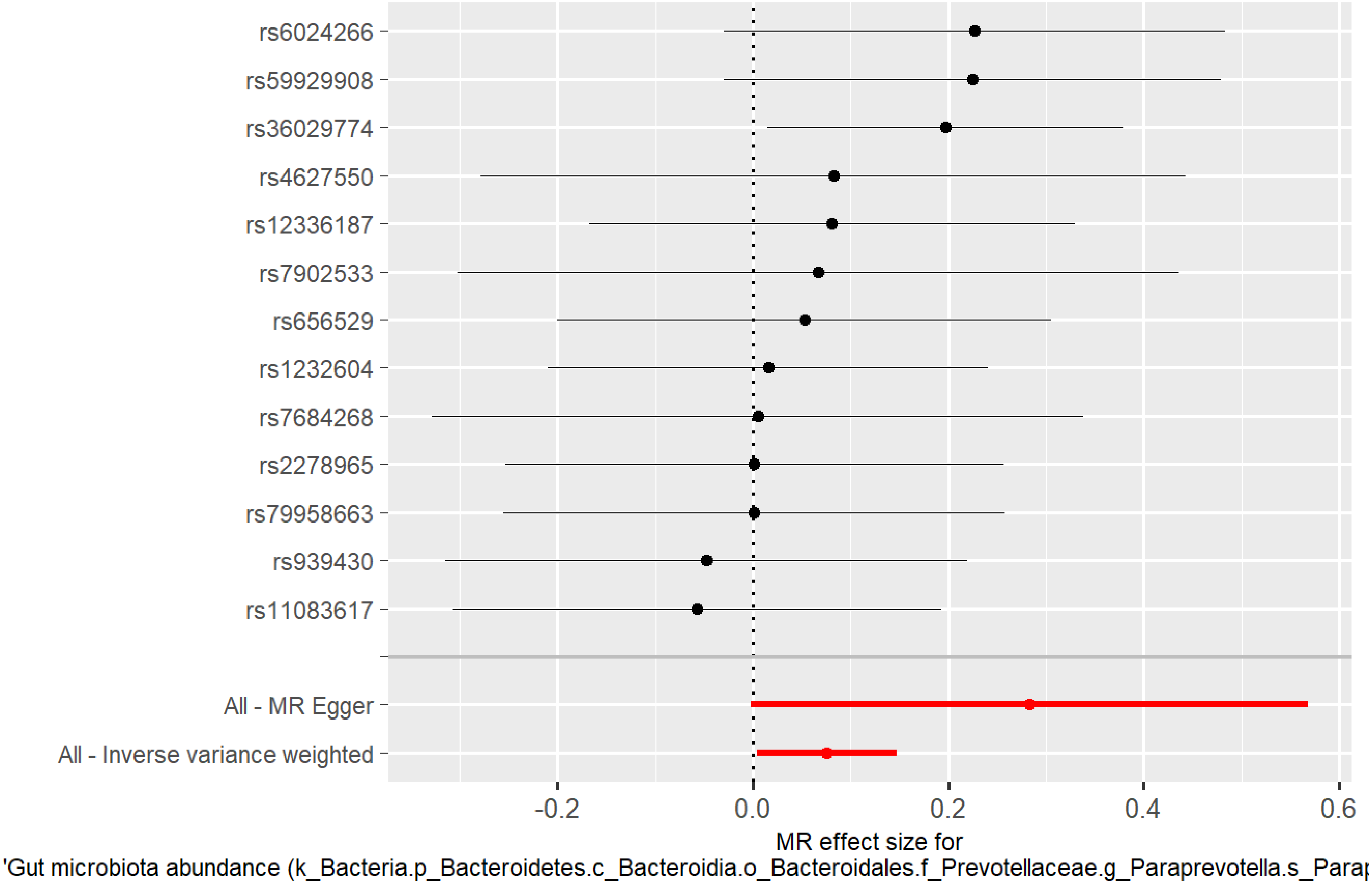
rs6024266
rs59929908
rs36029774
rs4627550
rs12336187
rs7902533
rs656529
rs1232604
rs7684268
rs2278965
rs79958663
rs939430
rs11083617
All - MR Egger
All - Inverse variance weighted
-0.2
0.0
0.2
0.4
0.6
MR effect size for
'Gut microbiota abundance (k_Bacteria.p_Bacteroidetes.c_Bacteroidia.o_Bacteroidales.f_Prevotellaceae.g_Paraprevotella.s_Parap

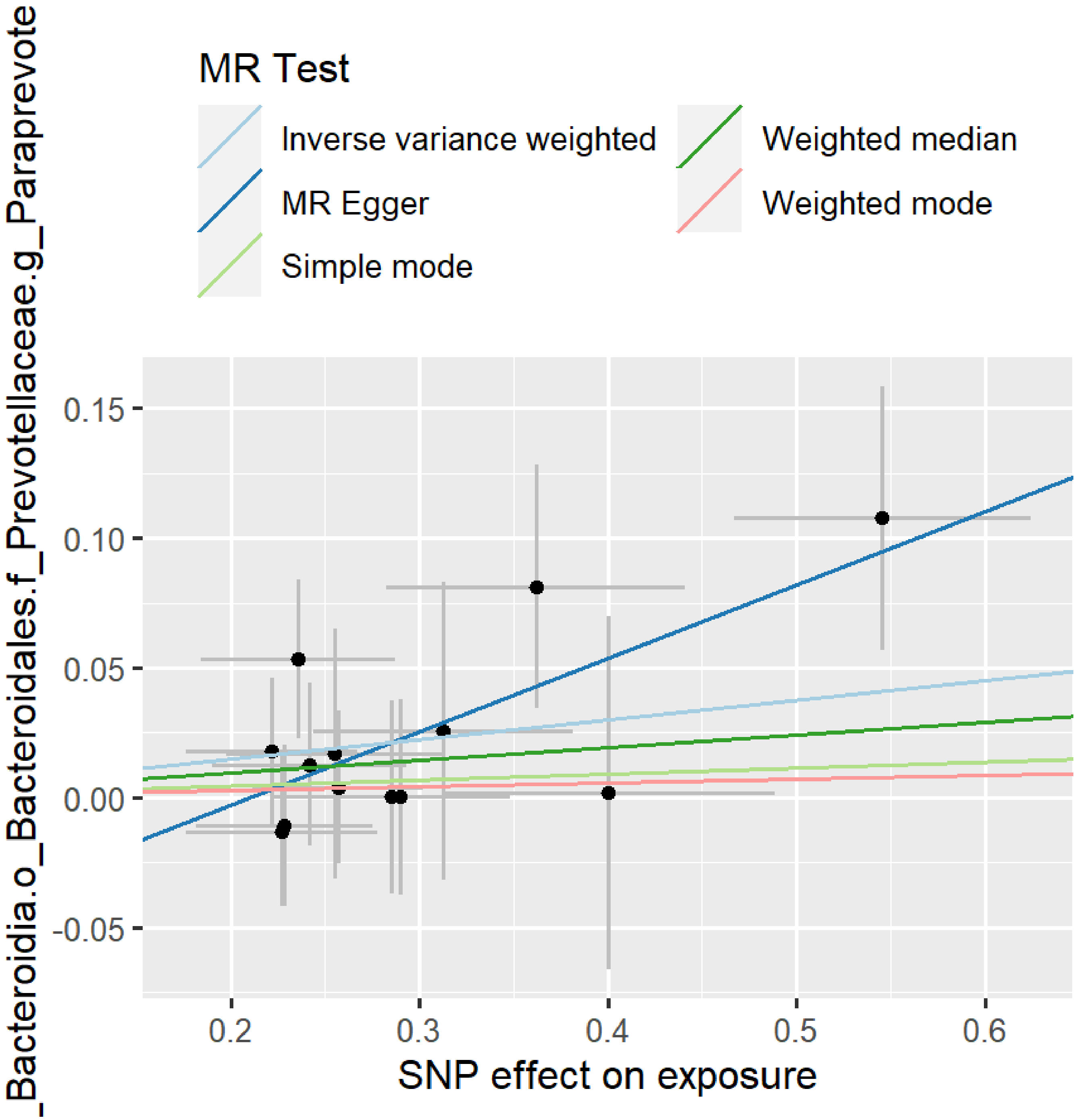
MR Test
Inverse variance weighted
MR Egger
Simple mode
Weighted median
Weighted mode
_Bacteroidia.o_Bacteroidales.f_Prevotellaceae.g_Paraprevote
0.15
0.10
0.05
0.00
-0.05
0.2
0.3
0.4
0.5
0.6
SNP effect on exposure

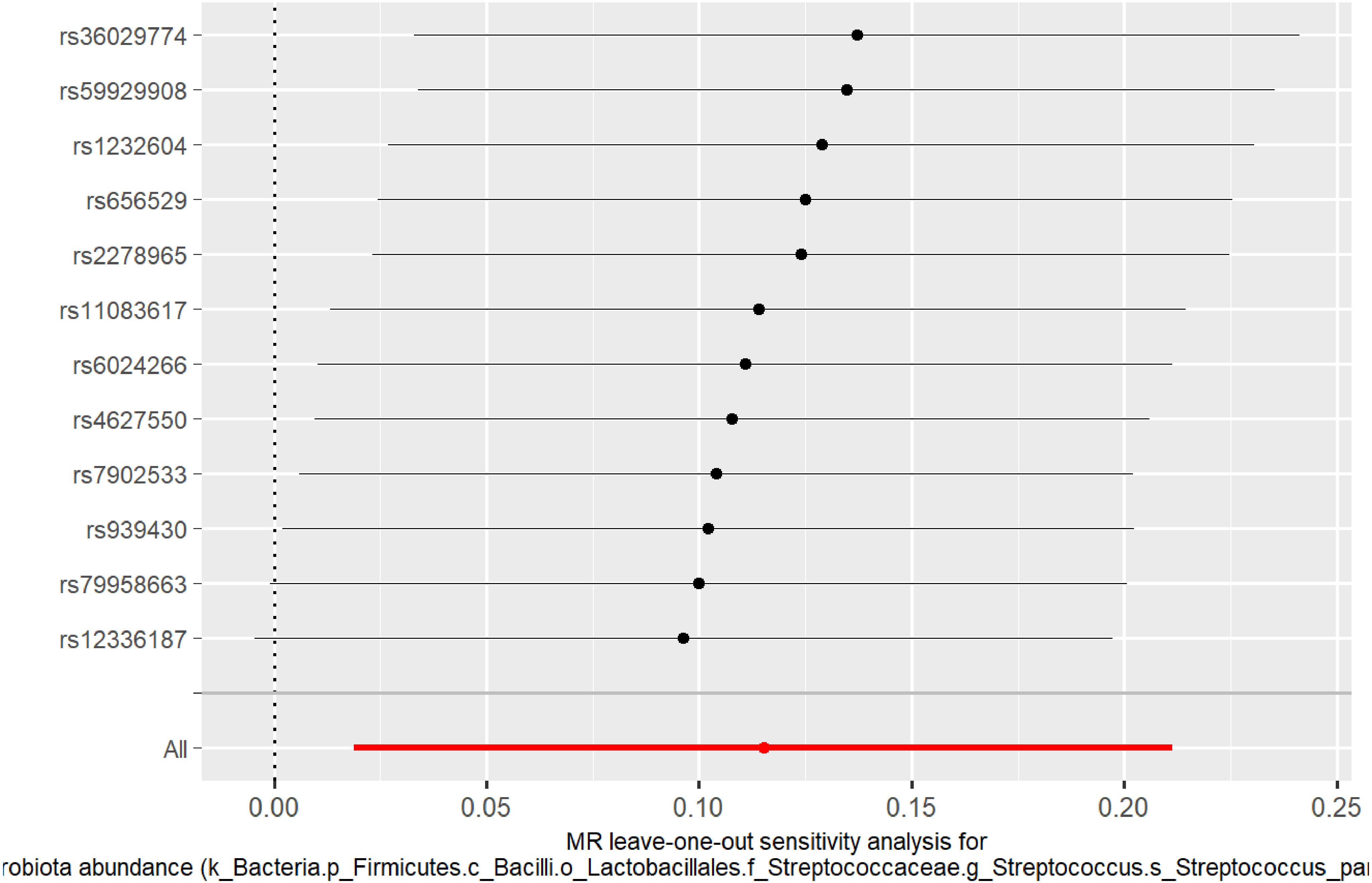
rs36029774
rs59929908
rs1232604
rs656529
rs2278965
rs11083617
rs6024266
rs4627550
rs7902533
rs939430
rs79958663
rs12336187
All
0.00
0.05
0.10
0.15
0.20
0.25
MR leave-one-out sensitivity analysis for
robiota abundance (k_Bacteria.p_Firmicutes.c_Bacilli.o_Lactobacillales.f_Streptococcaceae.g_Streptococcus.s_Streptococcus_pa

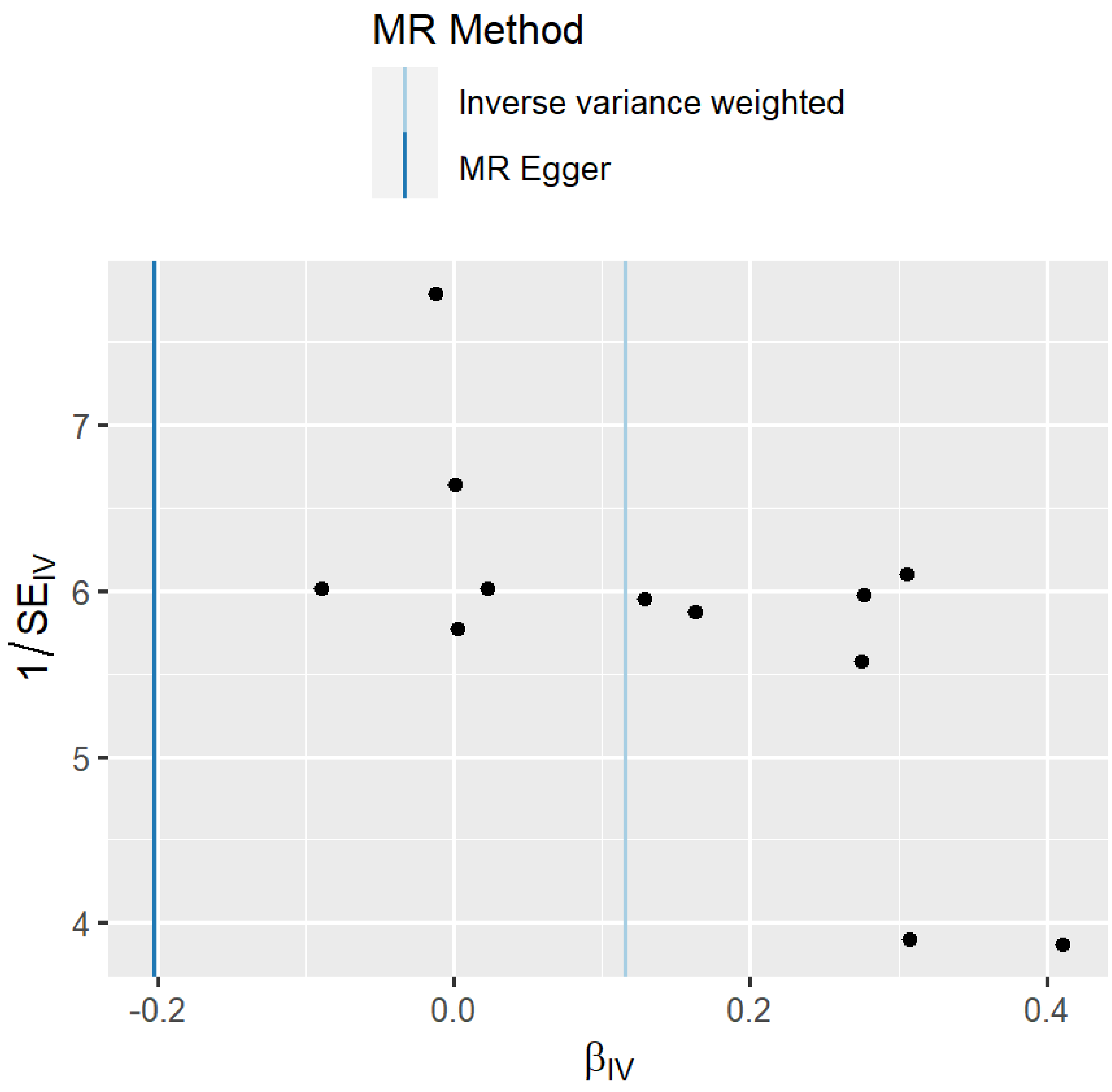
MR Method
Inverse variance weighted
MR Egger
7
6
5
4
1/SE_IV
-0.2
0.0
0.2
0.4
β_IV

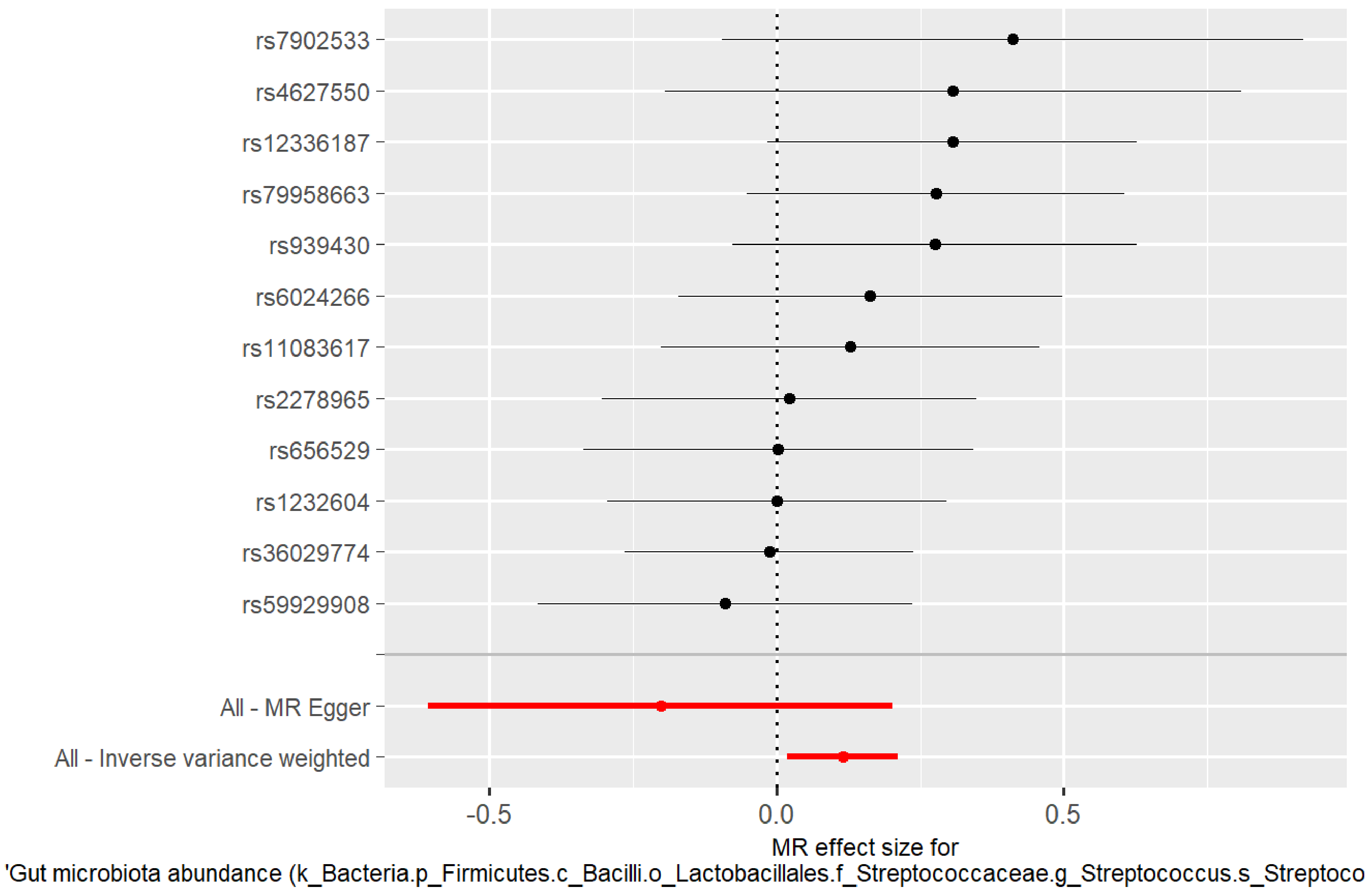
rs7902533
rs4627550
rs12336187
rs79958663
rs939430
rs6024266
rs11083617
rs2278965
rs656529
rs1232604
rs36029774
rs59929908
All - MR Egger
All - Inverse variance weighted
-0.5
0.0
0.5
MR effect size for
'Gut microbiota abundance (k_Bacteria.p_Firmicutes.c_Bacilli.o_Lactobacillales.f_Streptococcaceae.g_Streptococcus.s_Streptoco

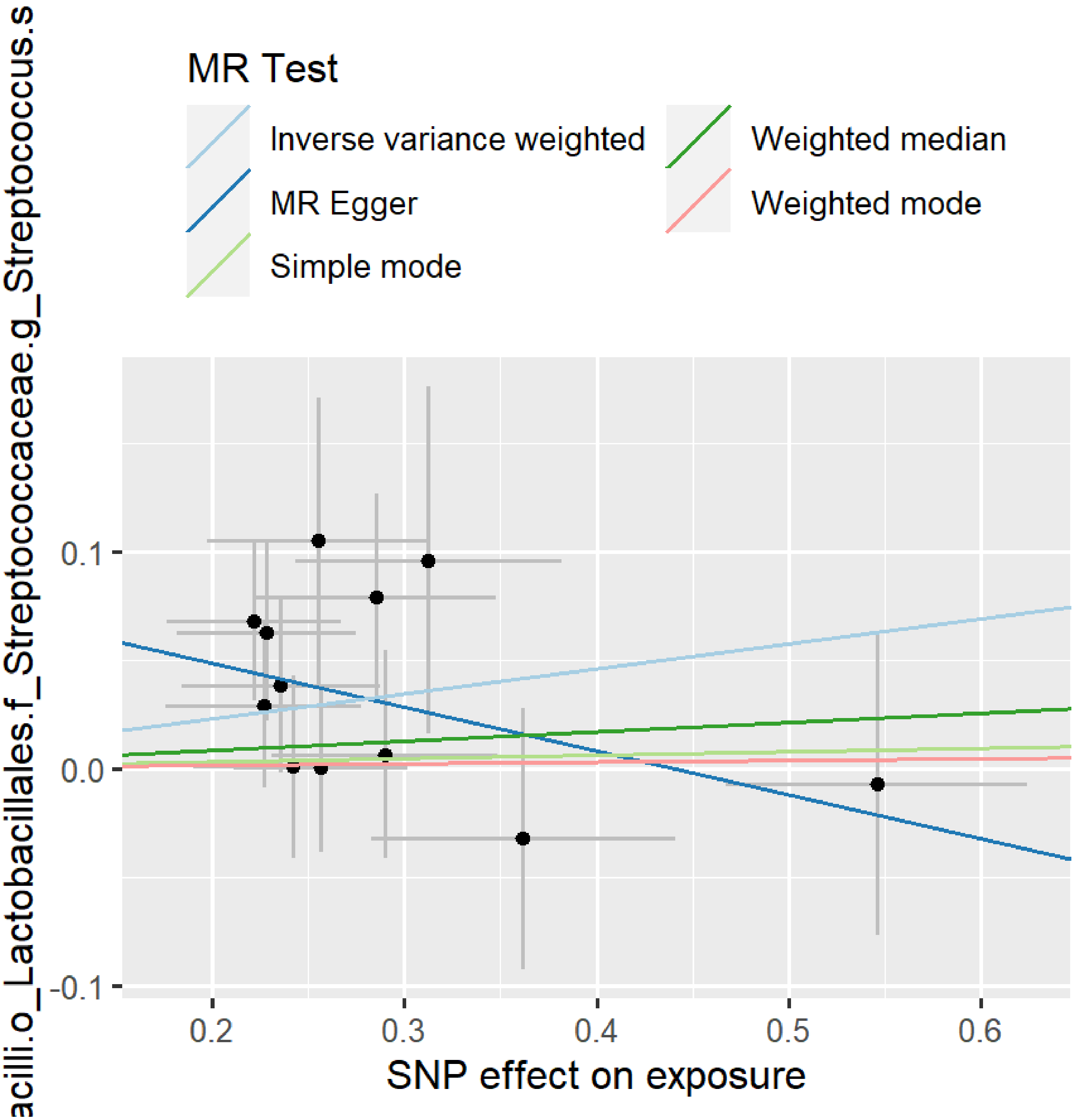
MR Test
Inverse variance weighted
MR Egger
Simple mode
Weighted median
Weighted mode
acilli.o_Lactobacillales.f_Streptococcaceae.g_Streptococcus.s
0.1
0.0
-0.1
0.2
0.3
0.4
0.5
0.6
SNP effect on exposure
