# Supplementary figures and images for: Investigating the association between gut microbiome and aortic aneurysm diseases: a bidirectional two-sample Mendelian randomization analysis
Source: Front Cell Infect Microbiol. 2024 Jul 30;14:1406845. doi: 10.3389/fcimb.2024.1406845 (PMC11319299; doi:10.3389/fcimb.2024.1406845)

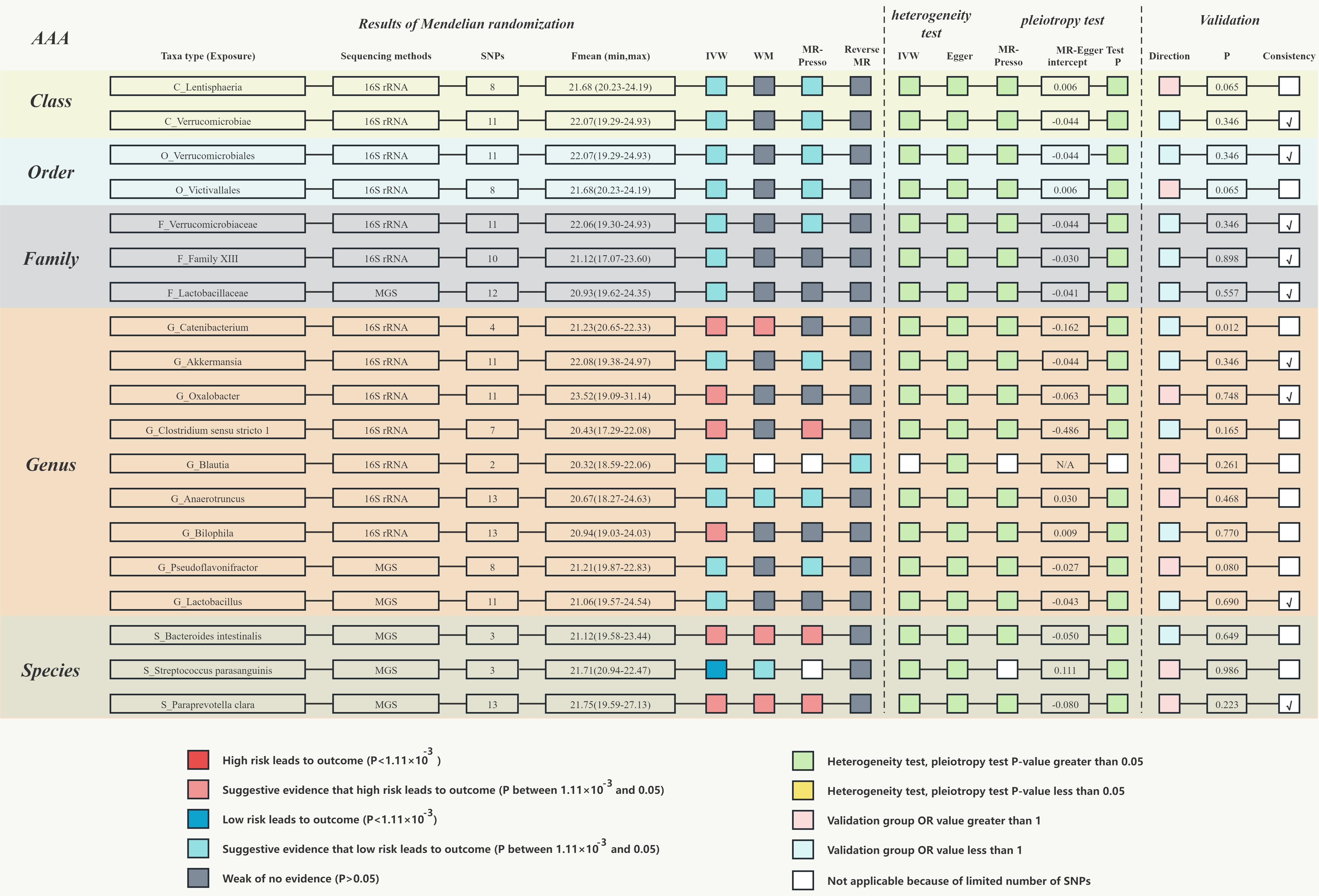

Supplement: Supplementary Figure 1 — An overview of the Mendelian randomization study results on Gut Microbiota in relation to Abdominal Aortic Aneurysm. The analysis methods include inverse variance weighted, weighted median, and MR-PRESSO analysis. 16S rRNA = 16S ribosomal RNA; MGS = Metagenomic Species; SNP = single nucleotide polymorphism; IVW = inverse variance weighted; WM = weighted median; P = probability value [file Image_1.jpeg]

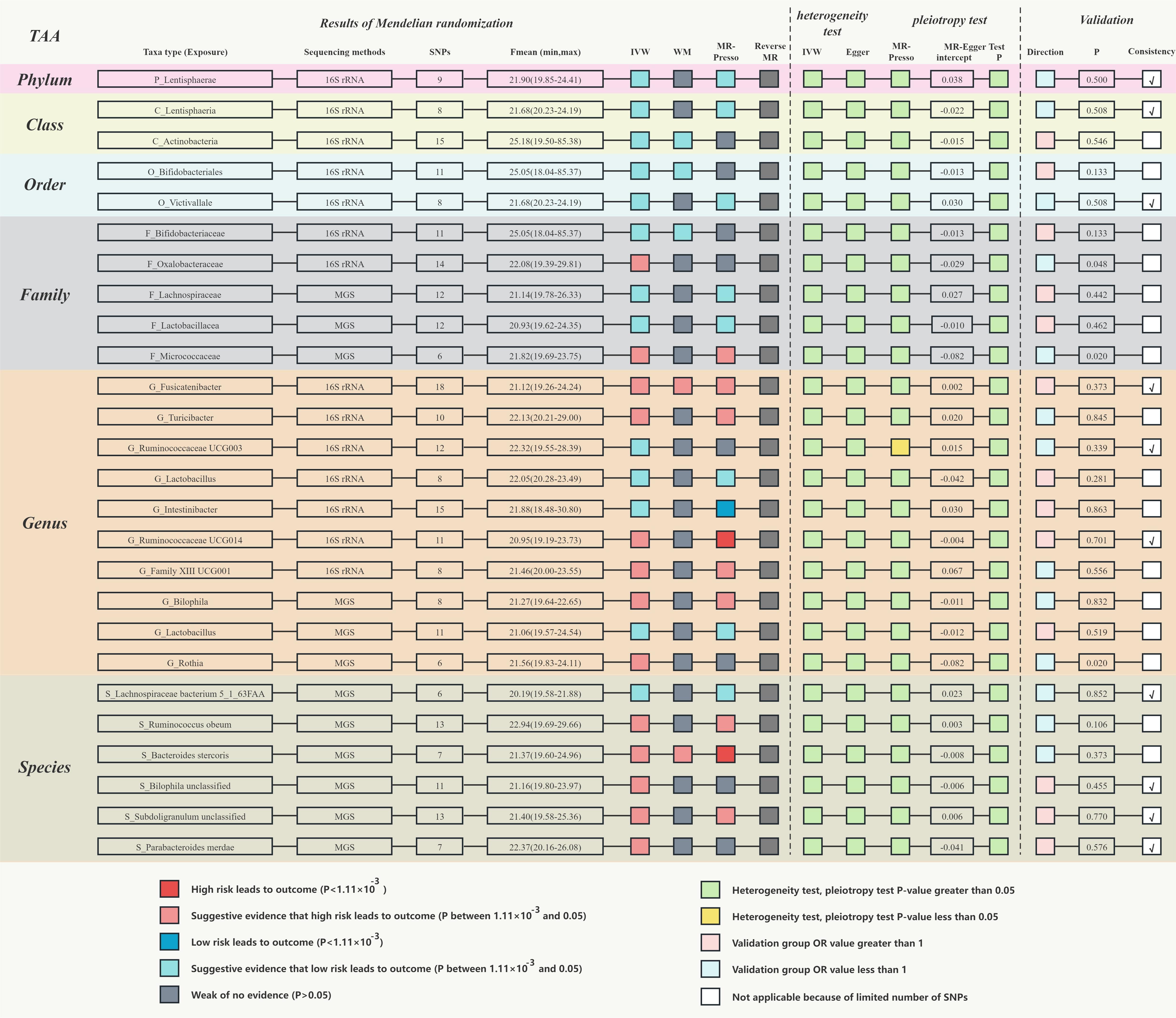

Supplement: Supplementary Figure 2 — An overview of the Mendelian randomization study results on Gut Microbiota in relation to Thoracic Aortic Aneurysm. The analysis methods include inverse variance weighted, weighted median, and MR-PRESSO analysis. 16S rRNA = 16S ribosomal RNA; MGS = Metagenomic Species; SNP = single nucleotide polymorphism; IVW = inverse variance weighted; WM = weighted median; P = probability value. [file Image_2.jpeg]

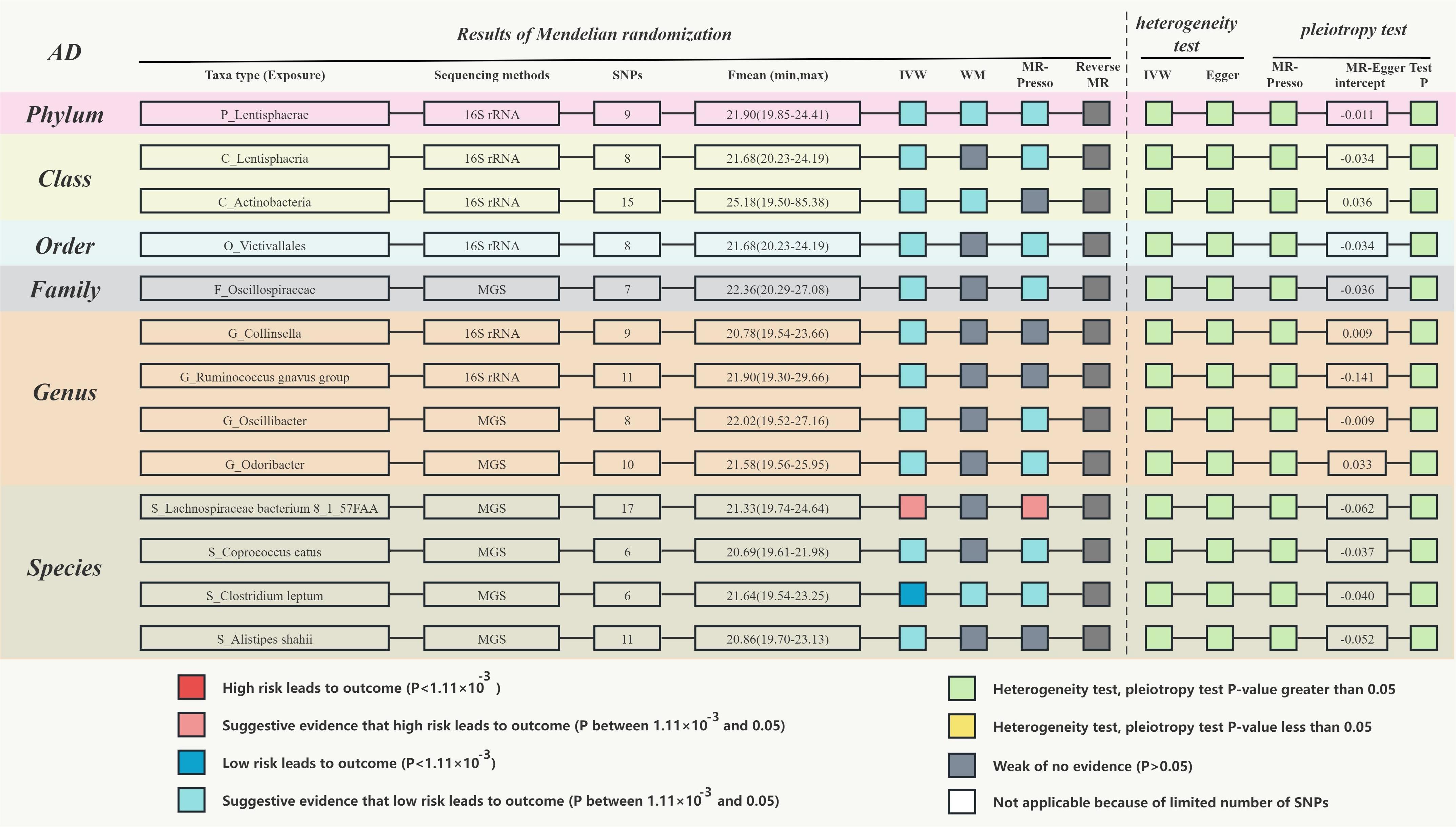

Supplement: Supplementary Figure 3 — An overview of the Mendelian randomization study results on Gut Microbiota in relation to Aortic Dissection. The analysis methods include inverse variance weighted, weighted median, and MR-PRESSO analysis. 16S rRNA = 16S ribosomal RNA; MGS = Metagenomic Species; SNP = single nucleotide polymorphism; IVW = inverse variance weighted; WM = weighted median; P = probability value. [file Image_3.jpeg]
